# Genome wide host gene expression analysis in mice experimentally infected with *Pasteurella multocida*

**DOI:** 10.1371/journal.pone.0179420

**Published:** 2017-07-13

**Authors:** G. Bhuvana Priya, Viswas Konasagara Nagaleekar, A. Arun Prince Milton, M. Saminathan, Amod Kumar, Amit Ranjan Sahoo, Sajad Ahmad Wani, Amit Kumar, S. K. Gupta, Aditya P. Sahoo, A. K. Tiwari, R. K. Agarwal, Ravi Kumar Gandham

**Affiliations:** 1 Division of Bacteriology & Mycology, ICAR-Indian Veterinary Research Institute, Izatnagar, Bareilly, Uttar Pradesh, India; 2 Division of Veterinary Public Health, ICAR-Indian Veterinary Research Institute, Izatnagar, Bareilly, Uttar Pradesh, India; 3 Division of Pathology, ICAR-Indian Veterinary Research Institute, Izatnagar, Bareilly, Uttar Pradesh, India; 4 Division of Animal Genetics, ICAR-Indian Veterinary Research Institute, Izatnagar, Bareilly, Uttar Pradesh, India; 5 Division of Veterinary Biotechnology, ICAR-Indian Veterinary Research Institute, Izatnagar, Bareilly, Uttar Pradesh, India; 6 Division of Livestock and Fishery Management, ICAR Research Complex for Eastern Region (ICAR-RCER), Patna, Bihar, India; 7 Division of Biological Standardization, ICAR-Indian Veterinary Research Institute, Izatnagar, Bareilly, Uttar Pradesh, India; J Craig Venter Institute, UNITED STATES

## Abstract

*Pasteurella multocida* causes acute septicemic and respiratory diseases, including haemorrhagic septicaemia, in cattle and buffalo with case fatality of 100%. In the present study, mice were infected with *P*. *multocida* (1.6 × 10^3^ cfu, intraperitoneal) to evaluate host gene expression profile at early and late stages of infection using high throughput microarray transcriptome analyses. Several differentially expressed genes (DEGs) at both the time points were identified in *P*.*multocida* infected spleen, liver and lungs. Functional annotation of these DEGs showed enrichment of key pathways such as TLR, NF-κB, MAPK, TNF, JAK-STAT and NOD like receptor signaling pathways. Several DEGs overlapped across different KEGG pathways indicating a crosstalk between them. The predicted protein—protein interaction among these DEGs suggested, that the recognition of *P*. *multocida* LPS or outer membrane components by TLR4 and CD14, results in intracellular signaling via MyD88, IRAKs and/or TRAF6 leading to activation of NFκB and MAPK pathways and associated cytokines.

## Introduction

Haemorrhagic septicaemia (HS) is an acute contagious disease of ungulates caused by *Pasteurella multocida* B:2 or E:2 serotype. HS is characterized by acute onset, respiratory complications, laryngeal edema and sudden death. *P*. *multocida* is a Gram-negative bacteria commonly found as a commensal or opportunistic pathogen of the upper respiratory tract [1]. HS is the most important disease in cattle and buffaloes with proven endemicity in India [2]. The pathogenesis of acute and fatal infection by *P*. *multocida* involves a complex interaction between host factors (e.g. species, age and immune status) and bacterial virulence factors (e.g., adhesions, capsule, LPS and molecules involved in nutrient acquisition).

Lipopolysaccharide (LPS) on gram negative bacteria is recognized by LPS-binding protein (LBP) forming LPS-LBP complex, which binds to opsonic receptor CD14 and triggers signalling through toll-like receptor 4 (TLR4)-MD-2 complex [3]. Binding of intracellular TLR domain, TIR (Toll/IL-1 receptor homology domain) to IRAK (IL-1 receptor-associated kinase), facilitates intracellular signalling, which in turn is mediated by two adapter proteins TIRAP (TIR domain containing adapter protein or Mal) and MyD88 (myeloid differentiation protein 88) [4]. In addition, MyD88-independent pathway is triggered by TIRAP/Mal signals through RNA-dependent protein kinase (PKR) and interferon regulatory factor—3 (IRF—3). The signals from the receptor complex activates IKK complex and NF-κB activity [3,5,6]. Activation of TLR-4 results in various events related to immune function, excessive production of proinflammatory cytokines viz. TNF-α, IL-1β, IL-6 and IL-8, and apoptosis [6]. Interaction of TNF-α with TNF-αR1 induces the recruitment of TRADD, TRAF2, RIP, FADD, cFLIP and caspase 8 [7]. Excessive production of proinflammatory cytokines results in endotoxaemia contributing to disease progression, clinical signs and death during acute diseases [8]. The most toxic cytokine is TNF-α followed by IL-1β, which induce vascular permeability, haemorrhage and severe pulmonary edema and also stimulate the macrophages to secrete other cytokines [4]. Further, it has been reported that host may also be able to respond to LPS by intracellular receptor called NOD protein. Expression of NOD1 and NOD2 proteins in the cytosol in response to Gram-negative LPS is evident but the mechanism is still not known [9–11].

As *P*. *multocida* is known for causing acute and fatal disease in various animals and birds, the effective prophylaxis through vaccination is the need of the hour. However, an effective vaccine to counter the disease doesn’t exist. In order to develop an effective *P*. *multocida* vaccine that is capable of regulating excessive inflammatory cytokine production, better understanding of cytokine profiles is essential. It is essential to investigate the expression profiles of host factors such as proinflammatory cytokines and other relevant molecules during *P*. *multocida* infection. In the present study, we analysed global gene expression profiles in spleen, liver and lungs of mice infected with *P*.*multocida* B:2 at early and late stages of infection. Using high throughput microarray transcriptome analyses many differentially expressed genes (DEGs) were identified and functional annotation of these DEGs showed enrichment of key pathways such as TLR, NF-κB, MAPK, TNF, JAK-STAT and NOD like receptor pathways under *P*.*multocida* infection.

## Materials and methods

### Mice

Swiss albino female mice (n = 50) of 6–8 weeks age were procured from Laboratory Animal Reservoir House, IVRI, Izatnagar. The animal experiments were performed following the protocol approved by Committee for the Purpose of Control and Supervision of Experiments on Animals (CPCSEA), India and Indian Veterinary Research Institute Animal Ethics Committee (IVRI—IAEC) (F.No. 1-53/2012-13-J.D(Res),dated 10.09.2013). Adequate veterinary care and husbandry practices were followed to minimize pain and distress to the animals.

### Characterization of the bacterial culture

The lyophilized culture of *P*. *multocida* serotype B:2 (strain P52) maintained in the Division of Biological Standardization, IVRI, Izatnagar was revived in 5 ml of BHI broth and incubated overnight at 37°C. Blood agar plates were streaked with broth cultures to isolate single colonies. From a single colony, an overnight primary culture (5 ml), followed by bulk subculture (100 or 200 ml) using BHI broth was done to obtain bacterial cells for infection. The culture was aliquoted and stored at -80°C as 10% glycerol stocks. Cultural, biochemical and morphological characterization was done as per standard methods [12]. Molecular identification of *P*. *multocida* serotype B:2 was carried out by using *P*.*multocida* B:2 species specific primers [13] and cap primers [14], to amplify 460 bp and 760 bp products, respectively (S1 Fig).

### Pathogenecity testing of *P*. *multocida* in mice

From the bulk culture, 10-fold serial dilutions were prepared in sterile phosphate buffer saline (PBS, pH 7.4) and 100 μl of bacterial suspension from selected dilutions (10^−3,^ 10^−4,^ 10^−5,^ 10^−6^) was inoculated intraperitoneally into four groups of mice (n = 6/group) and observed for mortality. The mice infected with 10^−5^ dilution exhibited some clinical signs as early as 6–8 hours post-infection (hpi) and ~50% mortality at 18–24 hpi. It was consistently observed in three independently repeated infections. The actual cfu at 10^−5^ dilution was found to be 1.6 x10^4^ cfu/ml, as confirmed by plating on 5% sheep blood agar.

### Experimental design of mouse challenge for transcriptome analysis

Three groups of mice (n = 6/group) were used for transcriptome analysis. Each mouse in Group 1 and Group 2 were infected intraperitoneally with 1.6 ×10^3^ cfu of *P*. *multocida* (100 μl of 1.6x10^4^ cfu/ml). Infected mice—Group 1 and Group 2 were euthanized humanely at 6 hpi (‘early’ stage of infection) and 18 hpi (‘Late’ stage of infection), respectively. Euthanasia was performed by sodium pentobarbital overdose followed by cervical dislocation. Group 3 control mice injected with 0.1 ml of PBS served as uninfected control.

### Necropsy, tissue collection and bacterial culture

Following euthanasia, necropsy was performed for blood and tissue collections. Immediately after opening the thoracic cavity, 100 μl of heart blood was collected aseptically using sterile needle directly from cardiac chamber and 20 μl was streaked onto blood agar plate for bacterial isolation. Tissues—lung, liver and spleen were collected aseptically from Group 1(6 hpi) and 2(18 hpi). Representative tissues were collected in RNase free 1.5 ml centrifuge tube containing 0.5 ml RNAlater and stored at -80°C for RNA isolation (transcriptome analysis) or tissue homogenate preparation (for cytokine analysis). Tissue samples were also collected in 10% neutral buffered formalin for histology and immunohistochemistry. Similarly, tissues were collected from Group 3 control mice at 18 hpi.

### Histology and immunohistochemistry

The formalin-fixed tissue samples were cut into pieces of 2 to 3 mm thickness and washed thoroughly with water and dehydrated in ascending grades of alcohol and cleared in xylene. The dehydrated tissues were embedded in paraffin blocks. Sections of 4μm thickness were cut and stained with haematoxylin and eosin (H&E) as per standard method. Immunohistochemistry was performed for demonstration of bacterial antigen using the procedure described by Praveena *et al*. [15]. Briefly, deparaffinised tissue sections were kept in microwave in 10 mM tri-sodium citrate buffer (pH 6.0) for 15 min (3 cycles of 5 min each) to unmask the antigenic sites. Endogenous peroxidase was blocked using 3% hydrogen peroxide (H_2_O_2_) in methanol for 30 min and then incubated with 5% normal rabbit serum for 1 h at room temperature (RT) for blocking of non-specific antigen binding. The sections were incubated with anti- *Pasteurlla multocida* P52 antibody raised in bovine for overnight in humidified chamber at 4°C. Sections were incubated with rabbit anti-bovine IgG peroxidise conjugate for 1 h at 37°C, followed by incubation with ExtrAvidin peroxidase for 30 min at RT. The positive immunoreaction was identified by adding 3, 3’-Diaminobenzidine (DAB) and counterstained with Mayer’s haematoxylin. Slides were mounted using DPX mountant.

### RNA isolation

RNA isolation from the tissues (spleen, liver and lung—15 mg approx) was performed using QIAGEN RNeasy^®^ Microarray Tissue Mini Kit as per manufacturer’s protocol. The purity and concentration of total RNA extracted was checked using Nanodrop spectrophotometer. The quality check of the isolated RNA was performed in Agilent 2100 Bioanalyzer as per manufacturer’s protocol using the Agilent RNA 6000 Nano Kit. The extracted RNA was found to be of good quality, having RNA integrity number (RIN) greater than 7.

### Host gene expression by one-color microarray based exon analysis

**Fig 1:**
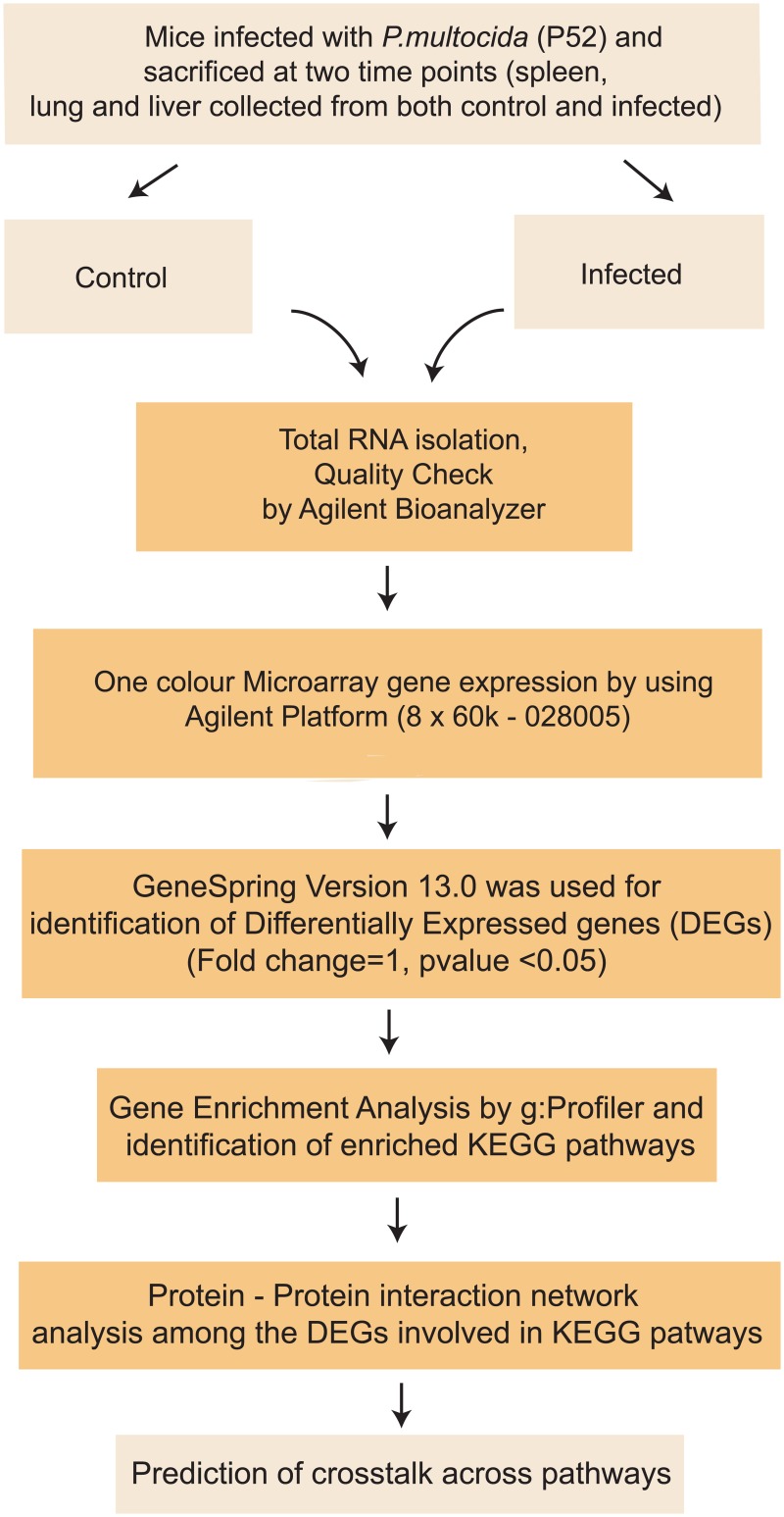
Workflow of the experiment.

Host gene expression by One-color microarray based exon analysis was performed using the mouse microarray slide (8x60K: Agilent—028005). Total RNA (100 ng) was labelled with Low Input Quick Amp WT Labelling Kit as per manufacturer’s instructions. RNA samples from three mice in each group were pooled into two samples (biological replicates) and microarray was performed in duplicates (technical replicates). The quality check of the labelled cRNA was performed using NanoDrop and the yield and specific activity were estimated. Hybridization and scanning were done as per the manufacturer’s protocol. After generating the microarray scan images, the signal intensities were extracted using Feature Extraction software version 10.7.3. The data generated after feature extraction were analyzed using Agilent software GeneSpring version 13.0 to identify the differentially expressed genes (moderate t-test; P ≤ 0.05) in mice. The data has been submitted in GEO data base with accession no GSE83502. The workflow is given in Fig 1.

### Functional annotation of differentially expressed host genes

Functional annotation of genes is commonly performed using online bioinformatics tool g:Profiler [16,17]. g:GOSt in g:Profiler performs functional profiling of gene lists using biological evidence. The tool performs statistical enrichment analysis to find Gene Ontology terms in categories such as biological processes, molecular function, and cellular component. Here, to functionally annotate the enriched processes among the differentially expressed genes (DEGs) of all the *P*. *multocida* infected tissues at both the time points, the DEGs were put in the g:Profiler against *Mus musculus* sp. Significant pathways enriched in KEGG were considered for further analysis.

### Protein-Protein interaction network of differentially expressed genes involved in key important KEGG pathways

BioGRID (Biological General Repository for Interaction Datasets) is an online interaction repository with data compiled through comprehensive curation efforts. It provides a comprehensive resource of protein–protein and genetic interactions for all major model organism species [18]. All interaction data are freely provided through search index and available via download in a wide variety of standardized formats. In this repository, protein-protein interactions in mouse are chosen. Customized Perl scripts were used to find out interactions involving the DEGs. Protein—protein interaction network of DEGs with fold change > ± 2.0 (log_2_Fold change > 1.0) resulted in a hairball. Therefore, the protein-protein interaction networks were constructed among the DEGs involved in the significantly enriched KEGG pathways for each tissue and time point and visualized in Cytoscape 3.0.2 [19]. Protein interaction network (interactome) analysis provides an effective way to understand the interrelationships between genes [20]. Venn diagram was drawn to look into the common genes that are involved in important signaling pathways such as TLR4 signaling, TNF signaling, NFκB signaling, MAPK signaling, JAK-STAT signaling, NOD signaling pathways across tissues and time points.

### Real time PCR

A total of eleven genes of interest resulting from microarray data were selected for further validation. These genes were selected on the basis of prediction for crosstalk across signaling pathways as described above. Quantitative Real time PCR (qRT-PCR) was carried out on the same biological material that was used in the microarray experiment. cDNA was synthesized using Revert Aid First Strand cDNA synthesis kit according to the manufacturer’s instructions and qRT-PCR was performed using Applied Biosystems 7500 Fast system using 2X SYBR Green Master mix (USB, Sigma). GAPDH was used as a housekeeping gene for normalization of target gene(s) of interest. The primer sequences for the genes used for validation are given in Table 1. For all genes tenfold serial dilutions were run in the study to estimate the efficiency of PCR, and the percentage efficiency ranged between 95 and 100%. All the samples were run in triplicates. The relative expression of each sample was calculated using the 2^−ΔΔCT^ method with control group as calibrator and the log_2_fold change was plotted [21].

**Table 1:** Primer sequences used for real time PCR.

Gene	Sequence of the primer	Product length (bp)
NFκB,	F—5’ CACCTGTTCCAAAGAGCACC 3’	154 bp
	R—5’ GGTTCAGGAGCTGCTGAAAC 3’	
IL6	F—5’ GCCAGAGTCCTTCAGAGAGA 3’	187 bp
	R—5’ GGTCTTGGTCCTTAGCCACT 3’	
IRF3	F—5’ ACTCCCAGGAAAACCTACCG 3’	136 bp
	R—5’ GATTTTCTTGGGGTGCAGGG 3’	
IL1β	F—5’ CCCAACTGGTACATCAGCAC 3’	180 bp
	R—5’ TCTGCTCATTCACGAAAAGG 3’	
TNF	F—5’ CCACCACGCTCTTCTGTCTAC 3’	103 bp
	R—5’ AGGGTCTGGGCCATAGAACT 3’	
RIP2	F—5’ ATGCCACCTGAGAACTATGAGCCA 3’	133 bp
	R—5’ GCAAAGGATTGGTGACCTCTTC 3’	
CASP9	F—5’ AGCAGAGAGTAGTGAAGCTG 3’	169 bp
	R—5’ ACACAGACATCATGAGCTCC 3’	
IRAK4	F—5’ CCTGGATGTCCTGGAACTTG 3’	81 bp
	R—5’ CAACACGCAGTAGGCAGAGA 3’	
RIP1	F—5’ CATGGTGTGTGCCCTTGTAC 3’	150 bp
	R—5’ CTCCCACACTCCACGTACTT 3’	
NOD2	F—5’ GCTGCTATGTGTTCTCAGCC 3’	150 bp
	R—5’ GACAGCCAAGTAGAAAGCGG 3’	

### Cytokine profile analysis

Using commercially available cytokine ELISA kits, the protein concentrations of pro inflammatory cytokines, IL6 and TNF-α (BosterBio, USA), were determined in spleen and lung tissue samples of both infected and uninfected control mice euthanized at each time points. The assays were carried out following manufacturer’s instructions. Briefly, 100 μl of lyophilized recombinant Mouse cytokine standard and test samples (including infected and control groups) were added to the duplicate wells pre-coated with anti-mouse TNF-α or IL-6 antibodies, followed by 100 μl of biotinylated anti-Mouse IL6/TNF-α antibody and incubated for 2 h. Following three washes, 100 μl of Avidin-Biotin-Peroxidase Complex was added to each well and incubated for 30 min. Following color development with 90 μl of TMB substrate, the reaction was stopped and absorbance was measured at 450 nm. From the standard curves derived individually for TNF-α or IL-6 by plotting absorbance against the known concentrations of the recombinant cytokines, the level of these cytokines in tissue samples were estimated and the significant difference was calculated using Tukey’s HSD in JMP 12.0.

## Results

### Experimental *P*. *multocida* infection in mice

*P*. *multocida*-infected mice were dull and depressed at early stage, while they were anorectic with ruffled hair coat and reluctant to move at late stage. Grossly, lungs showed congestion and haemorrhages at early stage of infection whereas congestion and consolidation were observed at late stage of infection. Liver showed congestion and slight enlargement at early stage whereas marked enlargement of liver was observed at late stage of infection. Spleen showed congestion at early stage of infection. Characteristic small, greyish, dew drop colonies of *P*. *multocida* were isolated on the blood agar plate streaked with the heart blood of infected mice (S1 Fig). Morphological, biochemical and molecular characterization confirmed the *P*. *multocida* infection. Microscopically, lungs showed congestion, marked vasodilation, haemorrhages, seroproteinaceous exudate with infiltration of polymorphonuclear (PMN) cells especially neutrophils in terminal bronchioles and mild interstitial thickening at early stage of infection (S2 Fig). At late stage, lungs showed marked interstitial thickening, infiltration of mononuclear cells, marked hyperplasia and desquamation of bronchiolar epithelium (S2 Fig). Liver showed engorged blood vessels, intracytoplasmic vacuolation in hepatocytes, and neutrophilic infiltration in portal area and capsule at early stage of infection (S2 Fig). At late stage, liver showed congestion, mononuclear cell infiltration and proliferation of kupffer cells (S2 Fig). Spleen showed depletion of red and white pulp area due to lymphocytolysis, seroproteinaceous exudate and neutrophilic infiltration at early stage of infection (S2 Fig). At late stage, spleen showed cystic spaces due to necrosis of lymphocytes in white pulp areas (S2 Fig). The positive immunoreaction was observed as brown color in the interalveolar space and cytoplasm of inflammatory cells in lungs; sinusoidal spaces in liver and cytoplasm of inflammatory cells in spleen (S3 Fig).

### Host gene expression by one-color microarray based exon analysis

On analysis, 9862 and 7640; 10260 and 15971; and 17189 and 15929, differentially expressed genes (DEGs) (S1 File) were identified in spleen, liver and lung at early and late stages of infection, respectively, in mice. These significantly (*P* ≤ 0.05) differentially expressed genes with ≥ ±2 fold (log_2_Fold change > 1.0) change were used for functional annotation using g:profiler and significant Gene Ontology (GO) terms for the differentially expressed genes were retrieved.

### Functional annotation and protein-protein interaction of differentially expressed host genes at early stage of infection

#### Functional annotation

The differentially expressed genes (DEGs) of spleen, lung and liver tissues at the early stage of infection were distributed among 2232, 2826 and 1841 categories, respectively, belonging to three branches of ontology *viz*. biological process, molecular function and cellular component.

In spleen, among biological processes, significant enrichment was found for the cellular, metabolic and positive regulation of metabolic processes besides the immune system processes (S4 Fig). In lung, significant enrichment was found for the G-protein coupled receptor signaling pathway, cell surface receptor signaling pathway and signal transduction apart from the other processes (S5 Fig). In liver, significant enrichment was found for the G-protein coupled receptor signaling pathway, cellular macromolecule metabolic process besides other biological processes (S6 Fig)

In spleen, catalytic activity, nucleoside phosphate binding and protein binding were observed to be enriched in the molecular function domain. Signaling receptor activity and transmembrane signaling receptor activity were observed to be enriched in the molecular function domain of lung and liver. In addition, signal transducer activity and molecular transducer activity were found to be enriched in liver and lung, respectively. Among the differentially expressed genes, a total of 1784, 1335 and 1264 genes exhibiting a significant enrichment (p< 0.05) were found to be associated to the immune system processes in spleen, lung and liver, respectively.

Among the documented canonical pathways found in KEGG, a total of 1037, 1636 and 1686 genes were mapped to 62, 133 and 47 statistically significant categories (p<0.05) in spleen, lung and liver, respectively (S4, S5 & S6 Figs) In spleen, Toll-like receptor, TNF, NF-Kappa B, JAK-STAT and NOD like receptor signaling pathways were annotated with 43, 53, 49, 62 and 33 genes, respectively. In lung, metabolic, MAPK signaling, natural killer cell mediated cytotoxicity, Rap1 signaling and T cell receptor signaling pathways were annotated with 350, 99, 57, 78 and 46 genes, respectively. In liver, NF-kappa B, NOD-like receptor, Toll-like receptor and RIG-I-like receptor signaling pathways were annotated with 44, 28, 42 and 33 genes, respectively.

Of many DEGs, 1475, 1712 and 1631 number of genes could also be mapped to 108, 229 and 225 significant (p<0.05) categories in the Reactome database in spleen, lung and liver, respectively. Among these categories, cytokine signaling, immune system and DNA replication were found to be over-represented with 133, 369 and 67 genes, respectively in spleen (S4 Fig). Extracellular matrix organization, signaling by GPCR downstream signaling, signaling by GPCR and metabolism were found to be over-represented with 497, 512 and 447 genes, respectively, in lung (S5 Fig). GPCR downstream signaling, signaling by GPCR and signal transduction were found to be over-represented with 623, 638 and 778 genes, respectively, in liver (S6 Fig).

#### Protein-protein interaction

**Fig 2:**
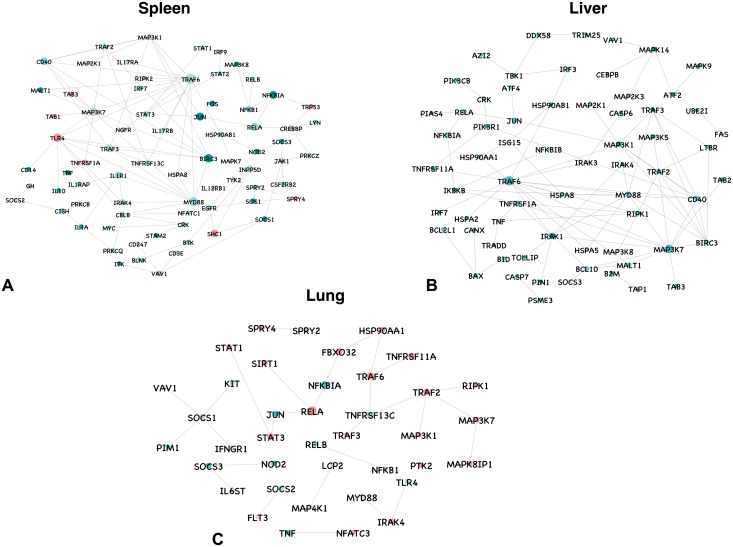
Protein–protein interaction network for differentially expressed genes involved in KEGG pathways in spleen, liver and lung at the early stage of infection. All the upregulated genes are shown in green, and downregulated genes in red, with the gradient showing the extent of expression. The diameter of the node represents the connectivity/degree of the node among the DE genes.

The protein—protein interaction network among the DEGs resulted in hair ball. To construct a representative protein-protein interaction network, genes involved in KEGG pathways were considered. In spleen, network of 9862 differentially expressed genes at the early stage of infection resulted in 2254 nodes and 4187 edges. A total of 275 genes involved in KEGG pathways (S2 File) represented a network consisting of 85 nodes and 145 edges involving interactions among them (Fig 2a). In liver, network of 10260 differentially expressed genes resulted in 2047 nodes and 4067 edges. A total of 177 genes involved in KEGG pathways represented a network consisting of 68 nodes and 125 edges involving interactions among them (Fig 2b). In lung, network of 17189 differentially expressed genes resulted in 990 nodes and 1404 edges. A total of 326 genes involved in KEGG pathways represented a network consisting of 39 nodes and 42 edges involving interactions among them (Fig 2c).

In spleen, TNF receptor-associated factor 6 (TRAF6), E3 ubiquitin protein ligase that mediates signal transduction from members of the TNF receptor superfamily, the most highly connected gene (degree = 60) was upregulated. MyD88 and IRAK4, key players in NF-kappa B signaling and Toll-like receptor signaling pathways were upregulated with a degree of 40 and 18, respectively. BIRC3, ubiquitin ligase that regulates NF-κB activation was upregulated with 37 interactions. RELA, also known as p65, which forms a heterodimeric complex with NF-κB was upregulated with a degree of 34. JUN, STAT2, STAT3, CREBB, SOCS1, SOCS2 and JAK1 involved in JAK-STAT pathway were all upregulated and connected in the network. LYN necessary for TLR4 dependent NF-κB and MAPK activation was upregulated with 6 interactions. IL-1, IL-6 and TNF, the proinflammatory cytokines were also found to be upregulated with an average connectivity of 3.6. IL-10, an anti-inflammatory cytokine involved in cytokine-cytokine receptor interaction, T cell receptor and JAK-STAT signaling pathways were upregulated. The transcription factor expression for inflammatory gene was induced by upregulation of NF-κB family genes. Chemokines (CXCL1, CXCL2, CXCL9, CXCL10 and CXCL13) involved in cytokine-cytokine receptor interactions, TNF, NOD like and Toll like receptor signaling pathways were upregulated besides NOD2. TAB1 and TAB3, which are mediators of transforming growth factor beta (TGF-β), MAPK and NFKB were downregulated with connectivity 2 and 4, respectively.

In lungs, RELA, the most highly connected gene (degree = 11) was upregulated. TRAF6, STAT1, JUN, RIPK1, TLR4, MYD88 and IRAK4 involved in Toll-like receptor signaling were upregulated and represented in the network. NFKB1, TRAF3, NFKBIA, SOCS3, MAP3K7, TNF, NOD2, JUN, RIPK1 and TRAF2 involved in TNF signaling pathway were upregulated. MAP4K1, NFATC3, MAPK8IP1, RELB, MAP3K1 and JUN involved in MAPK signaling pathway were upregulated. KIT and HSP90AA1 involved in PI3K-Akt signaling pathway were upregulated with an average connectivity of 4. Cytokines such as IL-1, TNF, IL-6, IL-10 and IL-7 were upregulated. Chemokines were also upregulated, though they are not represented in the network.

In liver, TRAF6 and BIRC3 were upregulated with 78 and 21 interactions, respectively. TBK1, IRAK1, MAPK14, PIK3R1, MAP2K1, IKBKB, TRAF3, NFKBIA, MAP3K7, PIK3CB, MAP3K8, IRF3, IRF7, CD40, TOLLIP, JUN, RIPK1, MYD88, IRAK4 and TAB2 genes that are involved in Toll-like receptor signaling were upregulated and connected in the network. Chemokines such as CXCL1, CXCL2, CXCL3, CXCL9 and CXCL10 were upregulated. The proinflammatory cytokines IL-1B, IL-6 and TNF were also found to be upregulated. BAX and BCL2L1, key genes in the intrinsic pathway of apoptosis are upregulated with connectivity of 3 and 1, respectively. TRADD, the activator of NF-κB signaling was upregulated and connected to TNF.

### Functional annotation and protein-protein interaction of differentially expressed host genes at late stage of infection

#### Functional annotation

The DEGs of spleen, lung and liver tissues at the late stage of infection were distributed among more than 1343, 2948 and 2720 categories, respectively, which belongs to three branches of ontology namely biological process, molecular function and cellular component.

In spleen, among biological processes, significant enrichment was found for the cellular, metabolic and positive regulation of metabolic processes besides the immune system processes (S7 Fig). In liver, significant enrichment was found for the G-protein coupled receptor signaling pathway, cell surface receptor signaling pathway, gene expression and signaling besides other biological processes (S8 Fig).In lung, significant enrichment was found for the positive regulation of biological processes and cellular development process besides the immune system processes (S9 Fig)

In spleen, receptor binding, macromolecular complex binding and catalytic activity were observed to be enriched in the molecular function domain (S7 Fig). In liver, signal transducer activity, signaling receptor activity and transmembrane signaling receptor activity and nucleic acid binding were observed to be enriched in the molecular function domain (S8 Fig).In lung, protein binding, catalytic activity and transmembrane signaling receptor activity were observed to be enriched in the molecular function domain (S9 Fig). Among the differentially expressed genes, a total of 1686, 1917 and 2077 genes exhibiting a significant enrichment (p<0.05) were found to be associated to the immune system processes in spleen, lung and liver, respectively.

Among the documented canonical pathways found in KEGG, a total of 754, 1836 and 2099 genes were mapped to 28, 132 and 68 statistically significant categories (p<0.05) in spleen, lung and liver, respectively (S7, S8 & S9 Figs). In spleen, among the top ten pathways, TNF, PI3K-Akt, FoxO and NOD like receptor signaling pathways and complement and coagulation cascades were annotated with 43, 53, 49, 62 and 33 genes, respectively. In lung, cytokine-cytokine receptor interaction, TNF, MAPK and Rap1 signaling pathway were annotated with 114, 56, 104 and 83 genes, respectively. In liver, proteasome, metabolic pathways and ubiquitin mediated proteolysis were annotated with 39, 376 and 62 genes, respectively.

Out of the totally differentially expressed genes, 721, 1666 and 1694 number of genes could also be mapped to 25, 214 and 253 significant (p<0.05) categories in the Reactome database in spleen, lung and liver, respectively. Among these categories, extracellular matrix organization, signaling by Rho GTPases and platelet activation, signaling and aggregation were found to be over-represented with 145, 73 and 112 genes, respectively, in spleen (S7 Fig). GPCR downstream signaling, signaling by GPCR, gene expression, Dectin-1 mediated noncanonical NF-κB signaling, cross-presentation of soluble exogenous antigens, regulation of activated PAK-2p34 by PMD and apoptosis were found to be over-represented with 490, 512, 430, 50, 41, 45 and 45 genes, respectively, in lung (S9 Fig). Regulation by apoptosis, regulation of activated PAK-2p34 by PMD and immune system were found to be over-represented with 41, 40 and 360 genes, respectively, in liver (S8 Fig).

#### Protein-protein interaction

**Fig 3:**
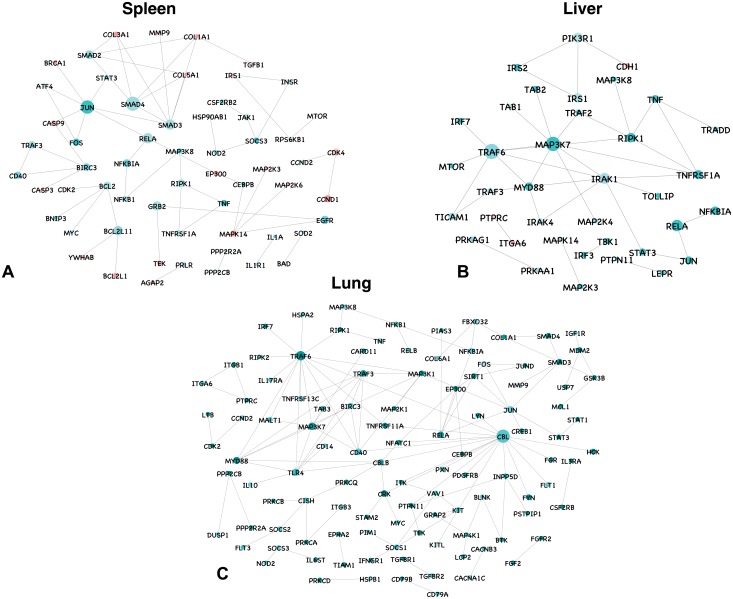
Protein–protein interaction network for differentially expressed genes involved in KEGG pathways in spleen, liver and lung at late stage of infection. All the upregulated genes are shown in green, and downregulated genes in red, with the gradient showing the extent of expression. The diameter of the node represents the connectivity/degree of the node among the DE genes.

In spleen, network of 7640 differentially expressed genes at the late stage of infection resulted in 1499 nodes and 2414 edges. A total of 346 genes involved in KEGG pathways (S2 File) represented a network consisting of 68 nodes and 91 edges involving interactions among them (Fig 3a). In liver, network of 15971 differentially expressed genes resulted in 4008 nodes and 9485 edges. A total of 150 genes involved in KEGG pathways (S2 File) represented a network consisting of 37 nodes and 61 edges involving interactions among them (Fig 3b). In lung, network of 15929 differentially expressed genes resulted in 3720 nodes and 8217 edges. A total of 501 genes involved in KEGG pathways (S2 File) represented a network consisting of 117 nodes and 188 edges involving interactions among them (Fig 3c).

In spleen, SMAD4, coactivator and mediator of signal transduction by TGF-beta (transforming growth factor) and component of the multimeric SMAD3/SMAD4/JUN/FOS complex was the most highly connected gene (degree = 30) and was upregulated. BIRC3, NFKB, FOS, RIPK1, SOCS3 and JUN involved in TNF signaling pathway were all upregulated and connected in the network. HSP90AB1, RELA and NOD2 involved in NOD-like receptor signaling pathway were upregulated with a connectivity of 4, 20 and 6, respectively. The proinflammatory cytokines and chemokines were upregulated. TGFβ an anti–inflammatory cytokine involved in tissue repair was downregulated. CASP3 (degree = 5) and CASP9 (degree = 3), which play a role on TNF signaling and PI3K-Akt signaling pathways, respectively, were downregulated.

In lung, genes involved in JAK-STAT pathway were all upregulated and connected in the network. TRAF3, TRAF6 NFKBIA, NFKB1, SOCS3, MAP3K7, MAPK10, BIRC3, FOS, RELA, TAB3, MAP3K8, JUN, RIPK and NOD2 were all upregulated. The proinflammatory cytokines TNF, IL-1 and IL-6 were upregulated. IL-18 and TGFβ (among the DE genes) were downregulated. Chemokines were also upregulated. TGFBR1, TGFBR2, DUSP1, DUSP2, PRKCA, CRK, HSPA2, MAP2K1, MAP3K8, MAP3K1, CACNA1C, PRKX and CD1 genes involved in MAPK signaling pathway were all upregulated. CCND2, IGF1R, FBXO32, CDK2, SMAD4, SIRT1, STAT3, MDM2, SMAD3 and EP300 genes involved in FoxO signaling pathway were also upregulated. IL-10, an anti-inflammatory cytokine was found upregulated.

In liver, TRAF6, was the most highly connected gene (degree = 38) and was upregulated. TBK1, TAB1, TAB2, IRAK1, IRAK4, TRAF3, MAPK14, PIK3R1, NFKBIA, MAP3K7, MAP2K3, RELA, MAP2K4, MAP3K8, TICAM1, TOLLIP, JUN, RIPK1 and MYD88 that are involved in Toll-like receptor signaling were upregulated and connected in the network. IL-1B, IL-6 and TNF, the proinflammatory cytokines were also found to be upregulated. LBP that binds to the lipid A moiety of bacterial lipopolysaccharides (LPS), a glycolipid present in the outer membrane of all Gram-negative bacteria, was found to be downregulated.

**Fig 4:**
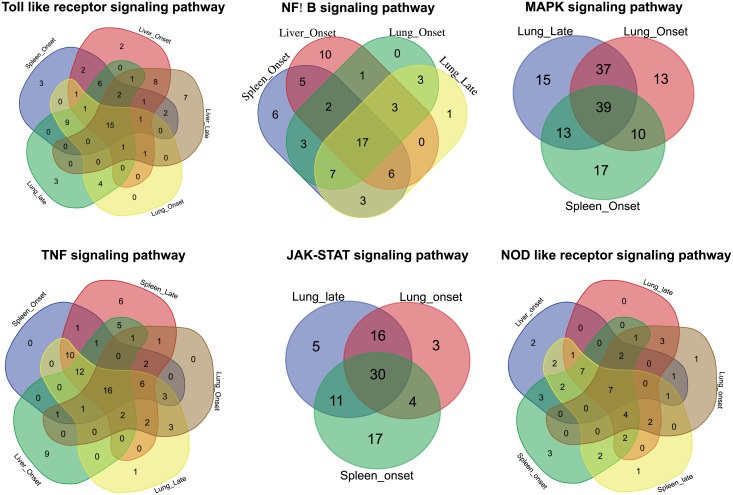
Venn diagram showing the genes involved across different tissues and time points in the enriched KEGG pathways.

Comparison of common genes involved in KEGG pathways at early and late stages of infection across different tissues also showed enrichment of Toll—like receptor, NF-κB, MAPK, TNF, JAK-STAT and NOD like receptor signaling pathways (Fig 4). Among the pathways enriched in the DEGs, it was observed that across all the tissues and time points 15, 17, 39, 16, 30 and 7 genes were commonly involved in Toll—like receptor, NF-κB, MAPK, TNF, JAK-STAT and NOD like receptor signaling pathways, respectively (Fig 4).

### Real time validation and cytokine profile analysis

**Fig 5:**
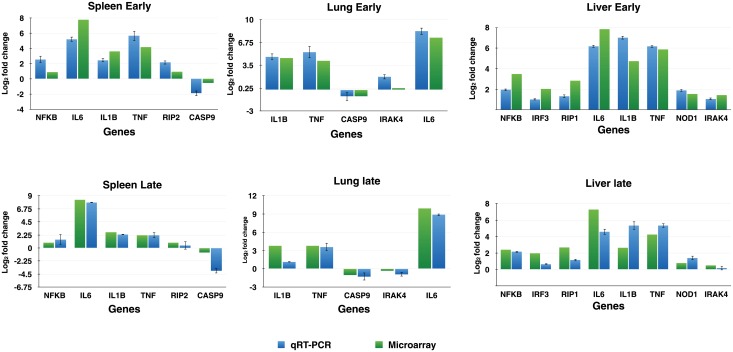
Quantitative real-time PCR to validate the RNA-seq experiment. Log_2_ fold change is plotted with the standard error of difference.

**Fig 6:**
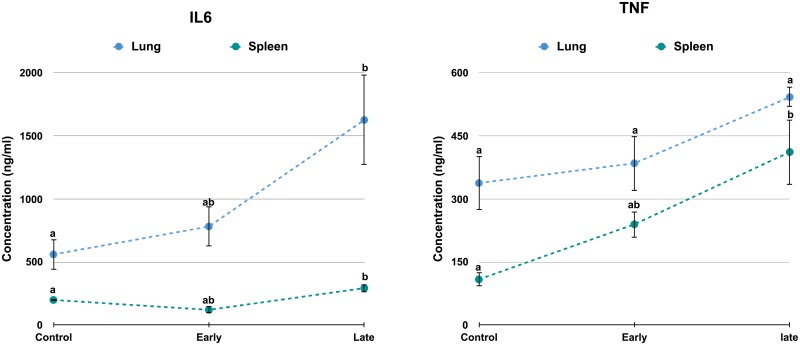
Level of TNF and IL-6 in spleen and lung by ELISA. Concentration (ng/ml) of TNF and IL6 was plotted against the control, early infection and late infection in spleen and lung. Significant difference (P ≤ 0.05) was calculated using Tukey’s HSD in JMP 12.0 (n = 3). Levels not connected by same letter are significantly different.

A total of eleven differentially expressed genes (NFκB, IL6, IRF3, IL1β, TNF, RIP2, CASP9, IRAK4, IL6, RIP1 and NOD) having an important role in the cross talk across different signaling pathways were validated for their expression using real time RT-PCR in spleen, liver and lung at both early and late stages of infection (Fig 5). The expression of these differentially expressed genes was in concordance with microarray analysis. The level of cytokines—TNF and IL-6 was found to increase with time in both the tissues (Fig 6).

## Discussion

The present study was done to profile the expression pattern of host genes in mice infected with *P*. *multocida* B:2. The current understanding on *P*. *multocida* interaction with the host suggests that bacteria establishes a complex interaction in host tissues and utilizes available niche effectively to grow rapidly and cause diseases in favourable circumstance [22]. However, the role of host factors that mediate acute pathogenesis and pathology are poorly understood. To understand the possible role of various host factors in *P*.*multocida* pathogenesis, here we sought to examine the expression of genes related to immune system and cellular development in mouse model of pasteurellosis.

In the present study, mice infected with *P*. *multocida* exhibited acute disease and histopathological lesions were consistent with previous reports [15,23,24,25]. *P*. *multocida* B:2 caused acute septicaemia, vascular damage and tissue injury possibly through overreaction of the host immune system [8]. Enrichment of genes annotated to proteosome and ubiquitin mediated proteolysis in KEGG pathway in lungs and liver (functional annotation figure) suggested the possible tissue injury. We found that depletion of red and white pulp areas in spleen is due to lymphocytolysis or apoptosis induced by bacteria or bacterial products as an immune evasion strategy to overcome cellular immune response of the host [25–27]. Apoptosis of lymphocytes associated with immune dysfunction and mortality had been reported in septicemic mice [26,27].

In this study, analysis of genes clearly signified the engagement of host’s innate immune system at early stage of *P*. *multocida* infection by upregulation of Toll-like receptor (TLR) signaling molecules, along with a wide range of inflammatory cytokines importantly TNF, IL-1, IL-6 and chemokines such as CXCL1, CXCL2, CXCL8 and IL-8 [28,29]. The infection also activated signal transduction pathways including MAPK, NF-κB, JAK-STAT, NOD and TNF signaling. Along with this, upregulation of SOCS genes and differential expression of genes involved in apoptotic pathways such as CASP1, CASP4, CASP8, BCL3, BCL6, BCL9, BAX, BIRC2 and BIRC3 indicates signalling to check the uncontrolled immune response during progression of infection [30]. TLR-induced signaling is capable of activating NF-κB through MyD88-dependent and MyD88-independent pathways, which further activates additional signaling components, including MAPKs and IRFs [6]. The expression of, TLR4 in lung early, lung late and spleen early; MyD88 and TRAF6 in all conditions and tissues; IRAK4 in liver late, liver early, lung early and spleen early; TOLLIP in liver late, liver early, spleen early; and LBP in liver late suggests the triggering of MyD88 dependent TLR signaling pathway. The expression of TRAF3 in all conditions and tissues; RIPK1 in liver late, liver early, lung early and lung late and other downstream MAPKs and IKKs also suggested triggering of MyD88 independent TLR signaling pathway. These findings were observant with the results in other gram negative bacterial infections [31–33]. The Src-family tyrosine kinases (SFK) *viz*. Hck and Lyn play a role in the differential regulation of pro—and anti-inflammatory effects downstream of TLR stimulation [34], including direct involvement in MyD88-dependent NFκB activation [35]. The expression of, LYN, NFKBIA, RELA, RELB and IL-1B in all conditions and tissues; RIPK1 in liver early, lung late and lung early; TAB3 and BIRC3 in liver early, lung late and spleen early; NFKB1 in lung late, lung early and spleen early; TAB2 in liver early and lung early and IKBKB in liver early suggested induction of NFκB signaling pathway. Further, TLR4 signaling triggers the mitogen-activated protein kinase (MAPK) pathway, a key inflammatory signal transduction pathway along with nuclear factor-κB (NF-κB) pathway [32]. RIP1 is polyubiquitinated to form a complex with TRAF6 and these two molecules appear to cooperate in facilitating TAK1 activation resulting in IKK mediated activation of NFκB and activation of the MAPK pathway. The expression of MAPK family genes, dual specificity phosphatases (DUSP) family members, TNF, IL-1, IL-6, JUN and NF-κB family genes were upregulated at early stage of infection in all the tissues indicated triggering of MAPK pathway. These findings especially genes such as MAP3K8, JUN, IL1 and JUN were in line with the results in *E*. *coli* infection [32].

The recognition of *P*. *multocida* LPS could likely result in activation of TLR, NFκB and MAPK signaling pathways for consequent release of inflammatory cytokines IL-1β, TNF, IL-6, TGF-β and IL-10. Dysregulation of TGF-β in lungs at late stage of infection and IL-10 whose expression level was lower than pro-inflammatory cytokines (though mentioned upregulated) in spleen and liver throughout the infection indicated its failure to suppress the inflammation at early stage of infection in acute infection [36]. The induction of the cytokines IL-1β and TNF by PRRs further serves to amplify the inflammatory response as they both promote NF-κB and MAPK activation. Binding of IL-1β to IL-1R triggers MyD88-dependent signaling [37], whereas TNF mediates most of its pro-inflammatory effects by binding to TNF receptor I (TNF-RI) [38]. The expression of MAP3K8, TNFRSF1B, TNF and IL1B in all conditions and tissues were observed. Further differential expression of TNFRSF1A, TAB1-3, RIPK1, TRADD, TRAF2, IKBKB, VCAM, MAPK and NFKB1 family genes indicates TNF signaling leading to activation of NF-kB and MAPK signalling. Expression of these genes was previously reported [7, 39]. TNF is also a potent inducer of apoptosis through activation of caspases [40]. The expression of CASP7, BAX, CASP6, TRADD, TNF, BCL2L1 and CASP3 approves the activation of caspases to bring about apoptosis. Expression of cell adhesion molecules such as ICAM and VCAM in all the tissues indicated the elevated leukocyte migration to the infection site that further enhances the inflammatory cascade [25].

In mammals the JAK/STAT pathway is the principal signaling mechanism for a wide array of cytokines and growth factors. The binding of the extracellular ligand (IL6) leads to pathway activation via changes to the receptors that permit the intracellular JAKs associated with them to phosphorylate one another. The activated JAKs further phosphorylate the STATs, which enter the nucleus and regulate transcription of cytokines, TFs, SOCS 1–3, etc. [41,42]. The expression of cytokines such as IL6, IL10, IL2 receptor, STAT1-3, TYK2 and SOCS family of genes reiterates the activation of JAK-STAT pathway.

NOD1 and NOD2 are sensors of different bacterial peptidoglycan components. NOD2 senses bacterial molecules derived from the synthesis and degradation of peptidoglycan [11]. Whereas NOD1 recognizes diaminopimelic acid produced primarily by Gram-negative bacteria [9,10], NOD2 is also activated by muramyl dipeptide (MDP), a component of both gram-positive and—negative bacteria [10]. Both NOD1 and NOD2 interact with the CARD-containing kinase RIP2 to activate MAPK and NF-κB signaling [43]. Upregulated expression of NOD1 and NOD2 (except liver) in all the tissues indicated its ability to sense the bacterial molecules resulting in the expression of proinflammatory genes and activation of caspases [11]. The upregulation of proinflammatory cytokines and apoptotic genes contributing to pathogenesis in *P*. *multocida* infection in mice were in conformity with Praveena *et al*. [15]. The expression of NFKBIA, NLRC4, IL1B, RELA, IL6 and TNF in liver early, lung late, lung early, spleen late and spleen early; MAP3K7 and TRAF6 in liver early, lung late, lung early and spleen early; TAB3 and BIRC3 in liver early, lung late, spleen late and spleen early; RIPK2, NFKB1 and NOD2 in lung late, lung early, spleen late and spleen early; TAB2 in liver early, lung early and spleen late; NFKBIB in liver early, spleen late and spleen early; MAPK12 in lung late, lung early, spleen late; MAPK11 in lung late, lung early and spleen early; MAPK14 in liver early and spleen late; MAPK13 and MAPK10 in lung late, lung early; TAB1 in spleen late and spleen early; MAPK9 and IKBKB in liver early; and MAPK3 in spleen late suggests the involvement of NOD like receptor pathway.

**Fig 7:**
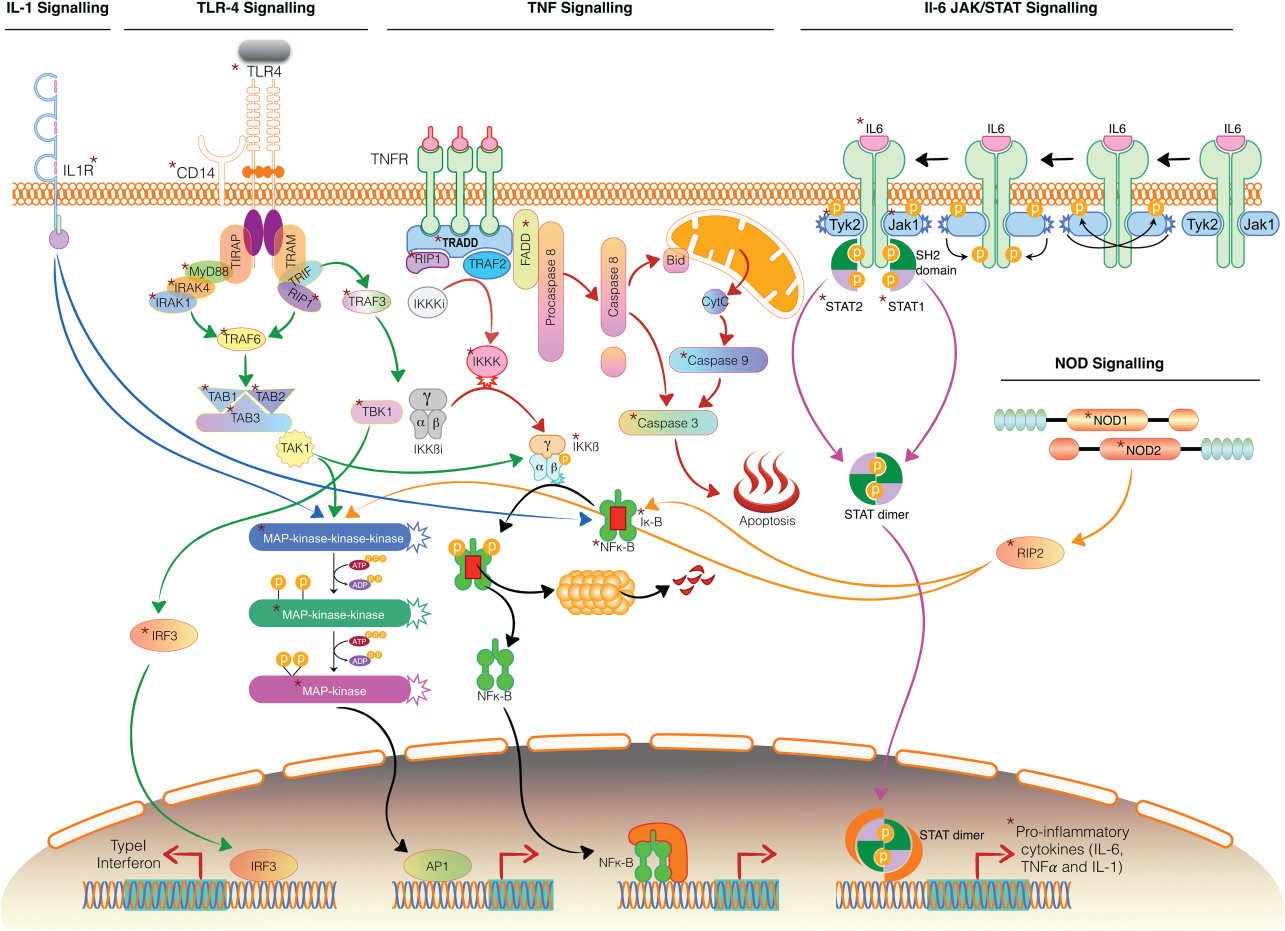
Predicted cross talk across different signaling pathways on *Pasteurella multocida* infection. The asterisk indicates the genes that are dysregulated in our study. The pathways followed by the blue, green, red, pink and orange arrows indicate IL-1,TLR4,TNF,JAK-STAT and NOD signaling pathways. Some of the genes involved have been validated by Real—Time PCR.

With the available leads in the present study and comprehensive knowledge available from the literature we predicted the possible cross talk across different signaling pathways on *P*. *multocida* infection (Fig 7). *P*. *multocida* through its outer membrane component LPS is recognized through a series of interactions with TLR4, CD14 and LBP. Upon LPS recognition, TLR4 recruits its downstream adaptors MyD88, through interactions with the TIR (Toll-interleukin-1 receptor) domains. MyD88 recruits and activates IRAK—4, which further activates IRAK -1. IRAK -1 associates with TRAF6, which interacts with TAB2 and TAB3 to stimulate two distinct pathways involving IKK complex and MAPK pathway through TAK1. The IKK complex phosphorylates IκB and activates NFκB. MAPK pathway involves MAPK kinases that were upregulated in our study. NFκB signaling and MAPK signaling pathways would consequently result in the release of inflammatory cytokines viz. IL1β, TNF and IL-6. The secreted IL-1 binds to its receptor IL-1R to activate NFκB signaling and MAPK signaling pathways. IL-6 through JAK-STAT pathway would also result in expression of several immune response molecules, which include proinflammatory cytokines, SOCS proteins, etc. TNF mediates its pro-inflammatory effects by binding to TNF receptor I (TNF-RI). Its cytoplasmic DD recruits adaptor protein TRADD, kinase RIP1 and several intracytoplasmic adaptor proteins leading to activation of NF-kB and MAPK pathways. The MyD88 independent pathway triggers interferon response through TRIF, TRAF3, TBK, IKK and IRF3. It also activates NFκB signaling and MAPK signaling pathways through TRIF and RIP1.

Our transcriptional analyses are consistent with known pattern of gene expression for acute infections. Our results could facilitate future studies to establish the molecular pathogenesis of pasteurellosis as to establish vaccine candidate that protects animals from infection by minimizing tissue damage.

## Supporting information

S1 Fig Confirmation of infection. (A). Molecular identification of *Pasteurella multocida* serotype B:2 by PM-PCR and capsular PCR assay (B). Isolation of characteristic colonies in Blood agar plate.
(EPS)

S2 Fig Histopathology of *P*.*multocida* infected lung at early and late stages of infection. (A). Normal alveoli lined by simple squamous epithelium. H&E x200 (B). Seroproteinaceous exudate with neutrophilic infiltration in the alveolar spaces due to increased vascular permeability and thickening of inter-alveolar septa. H&E x200 (C). Marked thickening of inter-alveolar septa with neutrophils and mononuclear cells. H&E x200 (D). Central vein with hepatic cords and normal sinusoidal space. H&E x200 (E). Engorged portal vein and hepatic artery in portal triad and hydropic degeneration of hepatocytes. H&E x200 (F). Proliferation and hypertrophy of Kupffer cells with degeneration of hepatocytes and dilated central vein. H&E x200 (G). White pulp areas infiltrated with lymphocytes and normal sinuses in red pulp areas. H&E x200 (H). Engorged portal vein and hepatic artery in portal triad and hydropic degeneration of hepatocytes. H&E x200 (I). Spleen showed cystic spaces due to necrosis of lymphocytes in white pulp areas. H&E x200. H&E x200.
(TIF)

S3 Fig Immunohistochemistry of *P*.*multocida* infected spleen at early and late stages of infection. (A). Negative control: Spleen showing no positive immunoreaction for the bacterial antigens. Anti-Pasteurlla multocida P52 primary antibody not added in tissue section. IP-DAB-MH x 200 (B). P. multocida-infected spleen (late): Cytoplasm of infiltrated inflammatory cells, lymphocytes and macrophages showing positive immunoreaction to P. multocida antigen. IP-DAB-MH x 200.
(EPS)

S4 Fig Functional analysis of differentially expressed genes in spleen collected at the early stage of infection. Functional annotation was carried out using g:profiler. Gene ontology (GO) terms were retrieved using g:profiler. The top 10 significantly (P≤0.05) enriched GO terms in biological process, molecular function and cellular component branches are shown. Also, significantly (p<0.05) enriched categories among the canonical pathways found in KEGG and Reactome databases are shown.
(EPS)

S5 Fig Functional analysis of differentially expressed genes in spleen collected at late stage of infection. Functional annotation was carried out using g:profiler. Gene ontology (GO) terms were retrieved using g:profiler. The top 10 significantly (p≤0.05) enriched GO terms in biological process, molecular function and cellular component branches are shown. Also, significantly (p<0.05) enriched categories among the canonical pathways found in KEGG and Reactome databases are shown.
(EPS)

S6 Fig Functional analysis of differentially expressed genes in liver collected at the early stage of infection. Functional annotation was carried out using g:profiler. Gene ontology (GO) terms were retrieved using g:profiler. The top 10 significantly (p≤0.05) enriched GO terms in biological process, molecular function and cellular component branches are shown. Also, significantly (p<0.05) enriched categories among the canonical pathways found in KEGG and Reactome databases are shown.
(EPS)

S7 Fig Functional analysis of differentially expressed genes in liver collected at late stage of infection. Functional annotation was carried out using g:profiler. Gene ontology (GO) terms were retrieved using g:profiler. The top 10 significantly (p≤0.05) enriched GO terms in biological process, molecular function and cellular component branches are shown. Also, significantly (p<0.05) enriched categories among the canonical pathways found in KEGG and Reactome databases are shown.
(EPS)

S8 Fig Functional analysis of differentially expressed genes in lung collected at the early stage of infection. Functional annotation was carried out using g:profiler. Gene ontology (GO) terms were retrieved using g:profiler. The top 10 significantly (p≤0.05) enriched GO terms in biological process, molecular function and cellular component branches are shown. Also, significantly (p<0.05) enriched categories among the canonical pathways found in KEGG and Reactome databases are shown.
(EPS)

S9 Fig Functional analysis of differentially expressed genes in lung collected at late stage of infection. Functional annotation was carried out using g:profiler. Gene ontology (GO) terms were retrieved using g:profiler. The top 10 significantly (p≤0.05) enriched GO terms in biological process, molecular function and cellular component branches are shown. Also, significantly (p<0.05) enriched categories among the canonical pathways found in KEGG and Reactome databases are shown.
(EPS)

S1 File The list of differentially expressed genes along with their log_2_Fold change in spleen, liver and lung at early and late stages of infection. (PDF)

S2 File The list of differentially expressed genes involved in KEGG pathways along with their log_2_Fold change in spleen, liver and lung at early and late stages of infection. (PDF)
