# Supplementary material for: Genome wide host gene expression analysis in mice experimentally infected with Pasteurella multocida
Source: PLoS One. 2017 Jul 13;12(7):e0179420. doi: 10.1371/journal.pone.0179420 (PMC5509158; doi:10.1371/journal.pone.0179420)
Supplement: S2 File — (PDF) [file pone.0179420.s011.pdf]

Spleen_onset

Genes	Log2 Fold Change
ACVR1B	0.96345997
BCL2A1C	2.4746747
BCL2A1D	2.2620535
BCL3	2.5083609
BIRC3	1.7918692
BLNK	1.0292406
BMP7	1.1134183
BTK	0.85606337
CACNA1S	0.60127115
CACNB3	0.9284253
CARD6	0.6339812
CASP1	0.335742
CBLB	0.43734598
CCL1	1.0456791
CCL12	3.931994
CCL17	3.5015655
CCL2	5.819554
CCL3	6.103713
CCL4	5.4779468
CCL5	0.49696016
CCL7	4.6872835
CCND2	0.78052616
CCR7	1.4655972
CCR8	0.92099
CD14	3.7933016
CD19	0.5951886
CD247	0.33286548
CD3D	0.5978079
CD3E	0.6034322
CD40	1.3697929
CD70	1.3753386
CD72	0.57064486
CD86	0.50169086
CEBPB	1.6610742
CISH	0.90105677
CR2	1.3522501
CREB3	0.4756117
CREB3L2	0.34491014
CREBBP	0.38971472
CRK	1.0127664
CRLF2	0.32533884
CSF1	0.7434416
CSF2	3.7690778

CSF2RB	1.158124
CSF3	6.319416
CX3CL1	3.4323378
CXCL1	7.3250604
CXCL12	0.5335994
CXCL13	2.3379927
CXCL16	1.0202079
CXCL2	5.6254663
CXCL3	3.0072234
CXCL5	4.432497
CXCL9	1.2320042
CXCR1	0.7033982
CXCR2	1.1469035
CXCR5	0.93828917
DAPP1	0.4347887
DDX58	1.5684328
DUSP1	1.813592
DUSP2	2.0593934
DUSP4	0.5186863
DUSP5	1.5932579
DUSP6	0.2666397
DUSP7	0.3711319
EDN1	2.933407
EGFR	1.3677685
ELK4	0.41202116
ELMO1	1.2934918
ERC1	0.19318318
FAS	2.230434
FCGR2B	0.56067276
FGF17	1.9941056
FGF21	2.7406847
FGF23	0.5094714
FGF3	0.7985103
FGFR2	0.33660364
FLNA	0.18988705
FLNB	1.6300769
FOS	2.1918736
GADD45B	2.3357725
GADD45G	1.1872578
GH	0.38226533
GM1987	0.37803888
GM5431	1.1910133
GNAI1	0.8681202
GNAI3	0.27142525
GNB3	1.3350627

GNG4	2.3555913
GNGT1	0.8952031
HCK	1.2153425
HSP90AB1	0.4672227
HSP90B1	0.34009457
HSPA1A	3.166572
HSPA8	0.27772903
HSPB1	0.982038
ICAM1	1.8613701
IFNA11	1.322186
IFNA2	1.3797648
IFNA5	0.34543228
IFNAB	0.711653
IFNAR1	0.40117884
IFNB1	4.2508535
IFNG	2.2231216
IFNK	0.7872491
IFNLR1	1.0511222
IKBKE	1.7415485
IL10	3.0187025
IL12RB1	0.43586683
IL12RB2	0.5335758
IL15RA	0.35707092
IL17A	4.235691
IL17RA	0.43265915
IL17RB	0.7269478
IL18	2.1502886
IL1A	3.4575286
IL1B	3.6146836
IL1R1	0.7844062
IL1R2	3.8958745
IL1RAP	0.866173
IL2	1.6852891
IL21	1.0160687
IL22	2.760804
IL23A	2.2281535
IL2RA	1.5691333
IL2RB	0.40103436
IL2RG	0.66580486
IL3	0.560683
IL4RA	1.0030823
IL6	7.7868
IL7R	1.537682
INHBB	3.5881553
INHBE	1.0299551

INPP5D	0.3787489
IRAK4	0.5152557
IRF7	0.86910915
IRF9	0.5210638
ITK	1.6315999
JAK1	0.3892331
JUN	1.9825015
LAT	0.65722466
LIF	3.2432673
LTA	0.7635517
LY96	0.63618517
LYN	1.2226291
MALT1	1.9365711
MAP2K1	0.575624
MAP3K1	0.24604177
MAP3K2	0.3439293
MAP3K6	0.73636675
MAP3K7	0.3022437
MAP3K8	2.2708101
MAP4K4	0.7238984
MAPK11	0.5933237
MAPK7	0.17284966
MAPK8IP3	0.31309128
MAPKAPK2	0.74232197
MEFV	2.3576398
MLKL	0.2870283
MMP14	1.2270918
MMP3	4.6143565
MOS	0.5846329
MRAS	0.8819952
MYC	0.6991663
MYD88	0.7676439
NAIP1	0.41405582
NAIP5	0.7294464
NAIP6	0.45552874
NCF1	0.72144413
NCK1	0.34001875
NFATC1	0.5206857
NFKB1	0.9200554
NFKBIA	2.0004086
NFKBIB	0.6005111
NFKBIE	0.6565237
NGF	0.8468089
NLRC4	0.9657941
NLRP1B	0.8176739

NOD2	1.6788135
NR4A1	1.2403183
OSM	0.85199237
PDCD1	1.9777141
PDGFA	0.43289042
PDGFB	0.28980398
PIK3AP1	1.0657673
PIK3CD	0.36889696
PIK3R3	0.25750852
PIK3R5	1.3595438
PLCG1	0.34619188
PLCG2	0.22274733
PPP3CC	0.7646642
PPP3R1	0.6312361
PRKCB	0.33858776
PRKCDBP	-0.63376
PRKCD	0.32718897
PRKCQ	0.71636057
PRKCZ	0.55051756
PRLR	0.43642306
PTGS2	4.569454
RAC3	0.50417805
RAP1B	0.2570758
RAPGEF2	1.5914574
RASA2	0.7406697
RASGRP1	0.48708677
RELA	0.70981216
RELB	1.4534655
RIPK2	0.97716165
RRAS2	0.622684
SAMD4B	0.6204052
SELE	3.420521
SHC1	-0.6657839
SOCS1	2.3679256
SOCS2	0.20260286
SOCS3	3.2041326
SOS1	1.6331842
SPRED1	0.96666336
SPRY2	1.2943351
SPRY4	-0.5819664
STAM2	2.5545406
STAT1	1.0214672
STAT2	1.2864275
STAT3	1.005301
STAT6	0.3228054

STK3	-0.5531182
STMN1	-1.0562091
SUGT1	0.32975435
TAB1	-0.9211865
TAB3	-0.8282819
TAOK2	-0.382194
TBK1	0.35324144
TEC	-0.286778
TGFB1	-0.6595635
TGFB2	-1.1781433
TGFB3	-0.4442935
TGFBR2	0.34888315
TIAM1	-0.9851229
TLR2	2.0033488
TLR4	-0.3496447
TLR6	1.0655465
TNF	4.206231
TNFRSF12A	2.5400085
TNFRSF13C	0.46550798
TNFRSF17	-0.5914836
TNFRSF18	1.2053876
TNFRSF1A	-0.2380071
TNFRSF1B	0.32127953
TNFRSF25	1.1673217
TNFRSF4	1.586935
TNFRSF9	0.23494434
TNFSF10	1.0401459
TNFSF11	0.4711504
TNFSF12	-0.3223906
TNFSF13B	-0.4890971
TNFSF14	0.30798817
TNFSF15	3.2862554
TNFSF8	1.4789319
TNFSF9	2.413536
TOLLIP	-0.5118837
TRAF2	0.491055
TRAF3	0.47019863
TRAF6	0.3756194
TRIM25	0.37845087
TRIP6	-0.3255639
TRP53	-0.6132464
TYK2	0.29271603
VAV1	0.8113737
VAV3	-0.6824346
VCAM1	-0.7154884

VEGFA	-0.8008142
VEGFB	-1.1410198
VEGFC	-1.2073183
WAS	-0.6120701
XCL1	0.30977297
XCR1	-2.2377143
ZAK	-0.4376452

Spleen_late

Gene	Log2 Fold change
ACKR3	0.7997894
ACVR1B	1.3582897
AGAP2	-0.5733433
AMH	1.2005613
ANGPT2	2.6205568
ATF4	0.79725313
ATM	0.6132984
BAD	-0.4769177
BCL2	0.7351556
BCL2L11	1.0366507
BCL3	1.9547296
BCL6	0.5312166
BIRC3	1.9350433
BMP7	1.6369331
BMPR2	0.9772258
BNIP3	2.2024431
BRCA1	-1.7640951
C1QA	0.7156954
C1QB	0.51039505
C1S2	1.2778034
C2	1.2307281
C3	0.7633176
C4B	0.41171455
C6	-1.434247
C8G	-0.8840294
CASP1	0.58240604
CASP3	-0.5348868
CASP7	0.5146618
CASP9	-0.7539864
CAT	-1.1805663
CCL11	3.6174178
CCL12	4.6193776
CCL17	4.744754
CCL2	5.3522506
CCL24	-3.1804965
CCL3	4.9520144
CCL4	4.180287
CCL5	1.5208344
CCL6	-0.9950681
CCL7	4.4948425
CCNB1	-2.176187
CCNB2	-2.14392
CCND1	-0.9427037

CCND2	1.0789857
CCNE1	-2.7541738
CCR3	-3.5069168
CCR5	0.91781616
CCR7	3.0074916
CCR8	1.3972266
CD40	1.6711459
CD40LG	1.0035646
CD46	0.7283685
CD70	3.776894
CDK2	-0.9675107
CDK4	-0.3636069
CDKN2B	2.2646983
CDKN2D	-1.0221882
CEBPB	2.1257248
CFB	2.6256056
CFD	-0.9784207
CFH	0.6654563
CNTF	-0.5630128
COL1A1	-1.8550992
COL1A2	-1.3854785
COL27A1	-1.4342406
COL3A1	-1.1381044
COL4A1	1.8997579
COL4A2	1.5939484
COL5A1	-1.4760485
COL5A2	-0.510073
COL6A1	-1.5712943
COL6A2	-2.066215
COL6A3	-0.7026472
CR2	-1.3124251
CREB3	0.82386875
CREB3L2	0.6433735
CREB3L3	0.50530124
CRLF2	0.72201633
CRTC2	0.8520441
CSF1	0.64739084
CSF2	3.3582351
CSF2RB2	1.4450054
CSF3	7.7814903
CSNK1E	0.4096694
CTF1	-0.3297546
CX3CL1	3.5762076
CX3CR1	-3.1579487
CXCL1	7.2812123

CXCL12	1.516212
CXCL13	3.2444267
CXCL14	2.764743
CXCL16	0.9963064
CXCL2	7.023438
CXCL3	7.1417766
CXCL5	4.157993
CXCL9	2.7529125
CXCR1	-0.8159263
CXCR3	-0.7436781
CXCR5	1.0957842
DAF2	0.9524827
DDIT4	1.1502337
EDN1	4.6177106
EFNA1	0.8041768
EFNA5	0.98385835
EGF	1.469341
EIF4EBP1	0.54106426
EP300	-0.7488952
EPHA2	0.64326954
EPOR	-3.3061824
ERBB2IP	0.40000343
F10	1.4702606
F12	1.2138221
F2	1.9110074
F3	0.98152924
F5	-1.7792315
F7	0.9847896
F8	-1.5031998
FAS	1.6610918
FGA	3.5082033
FGB	5.4701514
FGF1	-1.6518917
FGF21	1.6749434
FGF7	1.4780598
FGFR1	0.3340187
FGFR3	-0.9445927
FGFR4	-2.4488847
FGG	6.757786
FIGF	-2.1768396
FLT3	0.9881029
FLT4	0.77403903
FOS	3.824273
FOXO4	-1.2283776
GADD45A	-1.1292367

GADD45B	2.3206186
GADD45G	1.6221538
GM1987	-0.6113858
GM5431	0.8757341
GNB2	-1.0201678
GNB4	0.3984499
GNG11	-1.232204
GNG5	0.55924225
GNGT1	0.91595125
GRB2	0.57078505
GYS1	0.52698135
HC	1.4255233
HOMER2	-0.6966879
HRAS	-1.0997643
HSP90AB1	0.480062
ICAM1	1.8541679
IFNAB	0.87327576
IFNB1	3.2991629
IFNG	4.009959
IGF1	-1.54954
IL10	4.818664
IL11	1.1510792
IL12RB1	1.1471801
IL12RB2	0.814132
IL15	0.4632604
IL17A	5.5665903
IL17RB	0.8611431
IL18	2.2746024
IL1A	3.7713513
IL1B	2.7510228
IL1R1	1.0408502
IL1R2	4.3295417
IL2	1.5557206
IL22	4.4433956
IL23A	2.4010181
IL23R	2.523496
IL2RA	3.4102883
IL2RB	0.6914215
IL2RG	0.9468956
IL4	-0.8587384
IL5RA	-0.9998815
IL6	8.340477
IL7	-1.1558645
IL9R	-1.6111493
INHBB	4.4229445

INS1	3.61729
INSR	0.9182136
IRS1	-0.9625104
ITGA1	0.9072714
ITGA2B	-0.6590681
ITGA5	1.221149
ITGA6	-0.7418227
ITGA7	1.5052176
ITGA8	-3.182877
ITGA9	1.0920768
ITGAV	1.1375875
ITGB4	1.1001859
ITGB5	-1.6573215
ITGB7	-1.2005386
JAK1	0.49408388
JUN	2.1858373
KLK1B4	5.8837113
KNG1	4.781083
KNG2	3.458492
KRAS	-0.6433635
LAMA1	0.8757317
LAMA2	-1.6241465
LAMA4	1.1140542
LAMA5	1.9131861
LAMB1	0.50429535
LAMC1	0.8236742
LAMC2	2.28344
LEPR	0.97858477
LEPRE1	0.47561908
LIF	3.0903137
LPAR1	1.4254062
LPAR2	-1.0407782
LTA	-0.4755735
MAP2K1	0.71687937
MAP2K3	-1.277205
MAP2K6	-2.004179
MAP3K14	0.4784503
MAP3K8	2.7019377
MAPK12	0.9342098
MAPK14	-0.6682735
MAPK3	-0.8137946
MASP1	-0.6732757
MASP2	1.121773
MEFV	2.107893
MLKL	1.1826153

MMP14	1.9454579
MMP3	7.3080344
MMP9	-1.0223398
MPL	-0.7391472
MTOR	0.41482115
MYB	-0.912231
MYC	1.5316672
NAIP1	0.7932625
NAIP2	-0.33394
NAIP6	0.6825099
NFKB1	0.8645086
NFKBIA	1.9806142
NFKBIB	0.8955245
NGF	0.83318853
NLRC4	0.77970314
NOD2	1.1433349
NOS3	1.4926176
NR4A1	2.020092
OSM	0.7072575
PCK1	-2.3010292
PCK2	-1.0711257
PDGFA	1.0516696
PDGFD	-2.2874832
PF4	-0.6455636
PGAM5	0.63162804
PHLPP1	1.3056169
PHLPP2	-0.8087597
PIK3AP1	1.3694353
PIK3R2	-1.1634607
PIK3R3	-1.3433583
PIK3R5	1.3505158
PKN3	-0.5365982
PLAT	1.8833776
PLG	1.7543261
PLK1	-2.0595121
PLK2	2.4335504
PLK3	1.0950465
PLK4	-1.8608878
PPBP	-1.4106808
PPP2CB	0.44356966
PPP2R1B	-0.7991984
PPP2R2A	0.68584967
PPP2R2B	-1.7352362
PPP2R5B	-1.7358146
PPP2R5D	-0.3759503

PPP2R5E	-0.6330669
PRKAA1	-0.5082994
PRKAA2	-1.8102326
PRKAB1	-0.9287872
PRKAB2	-0.4140329
PRKAG1	-1.1251101
PRLR	1.3273633
PSTPIP1	-0.4295383
PTEN	0.42120886
PTGS2	5.0223465
RAG1	-2.954071
RAG2	-2.7891526
RELA	0.7493038
RELN	-1.3470926
RELT	-0.6496959
RIPK1	0.58239555
RIPK2	0.8808031
RPS6KA5	-0.8014002
RPS6KB1	0.59629965
S1PR4	-0.7595668
SELE	3.2909698
SERPINA1A	0.44704485
SERPINA1C	0.42771196
SERPINA1D	0.41743422
SERPINA5	0.87769556
SERPINC1	0.7523091
SERPINE1	5.208542
SERPING1	1.2945924
SGK2	2.6469991
SKP2	-1.069937
SLC2A4	-3.126202
SMAD2	0.9944539
SMAD3	0.7170725
SMAD4	0.86426497
SOCS3	3.7506132
SOD2	1.9517536
STAT3	1.5922232
STK11	-0.7740669
TAB1	-1.0941672
TAB2	-0.3283291
TAB3	-0.564723
TCL1B3	-0.570977
TEK	-1.4109261
TGFB1	-0.7102079
TGFB2	-0.7353251

TGFBR2	0.43873405
THBS1	0.9789958
THBS3	-0.3728352
THBS4	1.5162158
THEM4	0.8214879
TLR2	1.3897991
TNC	1.3498442
TNF	2.2247972
TNFRSF11B	1.0473626
TNFRSF12A	2.2571573
TNFRSF17	-2.119503
TNFRSF18	1.4665303
TNFRSF1A	0.5760875
TNFRSF1B	1.0171871
TNFRSF25	1.398449
TNFRSF4	2.2342062
TNFRSF8	0.5900984
TNFRSF9	0.8789964
TNFSF13B	-0.4282246
TNFSF15	2.2809033
TNFSF8	0.99408126
TNFSF9	2.201325
TNN	2.6868262
TNXB	-2.413766
TRAF3	0.8093934
USP7	-0.5949631
VCAM1	-0.6094694
VEGFB	-0.5722804
VTN	5.4852066
VWF	-1.4709516
XCL1	-1.535325
XCR1	-2.6582115
YWHAB	-0.408134

Lung_onset

**Genes**	**log2 Fold change**
C1QA	-2.3300638
C1QB	-2.2579298
C1QBP	-1.5704875
C1QC	-2.653265
BLOC1S2	-1.3259759
C1S2	-1.7225461
C2	-2.9133568
C3	-2.1411357
C4B	-3.180214
C6	-1.1189003
C8A	-10.873709
C8B	-10.797932
C8G	-10.054869
C9	-8.941826
CD59B	-5.008132
CFB	-2.8997488
CFH	-2.9597259
CFI	-10.457387
CPB2	-10.627819
CR2	-1.0024698
F10	-3.154902
F11	-8.651178
F12	-14.362644
F13B	-12.013451
F2	-12.908922
F5	-6.29021
F7	-4.0972776
F8	-4.597064
F9	-6.346343
FAS	1.6066656
FBXO32	0.72601604
FGA	-10.777
FGB	-10.708057
FGF10	3.5441253
FGF11	1.8084731
FGF12	1.1424072
FGF17	1.1147311
FGF18	2.1681595
FGF7	4.182008
FGFR1	2.2481775
FGFR2	0.9428587
FGG	-9.45623
FIGF	4.3744617

FLNA	2.0658784
FLNB	1.79989
FLNC	3.781347
FLT1	2.5088482
FLT3	1.0449159
FOS	4.4170623
FOXO6	2.123439
FYN	1.2702165
GADD45A	1.9841809
GADD45B	3.4639516
GADD45GIP1	-2.3421206
GADD45G	1.5053544
GM13304	2.4578342
GM1987	2.910431
GM5431	0.36816263
GNB4	1.4175034
GNB5	1.3854225
GNG11	0.4208293
GNG8	1.0619752
GRAP2	1.6940999
GSK3B	0.77440786
GYS1	1.2221203
HOMER3	0.69000983
HSP90AA1	0.49090576
HSPA1A	1.4282413
HSPA1L	0.578073
HSPA2	1.7292991
HSPB1	5.710805
ICAM1	3.5390153
IFNA14	0.7726519
IFNA5	0.5634656
IFNAB	0.79102993
IFNAR1	0.6276746
IFNG	2.7526507
IFNK	1.1500814
IFNLR1	4.212093
IGF1R	3.2744799
IKBKE	0.6028137
IL10	0.74110174
IL10RB	1.4384971
IL11	1.8177376
IL12RB1	0.302567
IL12RB2	0.8175061
IL17A	4.3824067
IL17RA	1.2611256

IL18R1	4.4500294
IL18RAP	4.1401324
IL1B	4.5395837
IL1R2	8.076264
IL20RB	1.5086188
IL21R	1.0383027
IL22RA2	3.5503724
IL23A	1.813232
IL2RA	4.114599
IL2RB	1.3071642
IL2RG	1.8949919
IL3RA	0.67282295
IL4RA	2.6294155
IL6	7.458927
IL7	0.7407806
INHBB	4.967063
INPP5D	1.2535157
IRAK4	0.2841568
IRS2	1.1174812
ITGA1	1.6040163
ITGA11	2.1542244
ITGA2	1.3487165
ITGA3	2.3282404
ITGA4	1.3670893
ITGA6	1.8815241
ITGA7	0.4074726
ITGA8	3.734334
ITGAV	0.38120747
ITGB1BP1	-0.2188277
ITGB1	0.8095498
ITGB3	2.7306557
ITGB4	2.2377954
ITK	2.0560484
JUN	3.946128
KIT	2.1481447
KLF2	1.9909196
LAMA2	2.66321
LAMA4	2.652299
LAMA5	3.2739367
LAMB1	1.8184557
LAMB2	2.1300597
LAMC1	1.6540756
LAMC2	6.78693
LAMC3	1.844254
LAT	1.1295719

LCP2	1.6838231
LPAR1	4.0328197
LPAR2	0.20749211
LPAR3	4.1419363
LTA	0.83822846
LTB	2.2415051
LYN	1.4088578
MALT1	2.5987668
MAP3K1	0.57268906
MAP3K3	1.0121245
MAP3K6	4.716194
MAP3K7	0.419446
MAP3K8	2.4953523
MAP4K1	1.2381611
MAP4K3	0.24417543
MAP4K4	1.2343116
MAPK10	2.2444887
MAPK11	1.7025175
MAPK12	0.99682283
MAPK13	3.3856153
MAPK7	1.02703
MAPK8IP1	0.61370754
MAPK8IP3	0.61066055
MAPKAPK2	1.0024791
MAPKAPK3	1.8800907
MAPT	4.257713
MAX	0.619256
MCL1	0.9923358
MEF2C	0.9999995
MEFV	3.3240447
MMP3	5.170152
MMP9	5.527232
MPL	1.104034
MYB	2.716485
MYC	1.8706264
MYD88	1.5814781
NAIP6	0.49379778
NCK1	0.52710867
NCK2	0.86701965
NFATC1	1.5677791
NFATC2	0.8250365
NFATC3	0.40435958
NFKB1	1.745542
NFKBIA	3.0278368
NFKBIE	2.292398

NLK	0.37855434
NLRC4	2.236875
NLRP1A	0.43206072
NOD2	4.366301
NR4A1	3.3831625
NRAS	1.3413324
NTF5	0.87815
NTRK2	0.9203265
OSM	3.7199185
PAK2	0.6441016
PCK2	0.4853809
PDCD1	1.7823114
PDGFA	1.8793483
PDGFB	3.2462788
PDGFD	1.2010536
PDGFRB	0.74780893
PF4	2.363049
PHLPP2	0.6950011
PIAS1	0.508173
PIK3AP1	1.2819929
PIK3CD	0.7616577
PIK3R3	1.6068354
PIK3R5	2.3862696
PIM1	3.0797358
PKN2	0.58896446
PKN3	0.526752
PLA2G4A	0.91808605
PLA2G4F	1.3484056
PLAU	2.0492165
PLCG2	1.2643476
PLK2	0.94822836
PPBP	2.395474
PPP2CB	0.7925134
PPP2R2A	0.87358665
PPP2R2B	0.96282077
PPP2R2C	1.4879966
PPP2R5B	0.71786594
PPP3CA	0.71466446
PPP3CB	0.18939066
PPP3CC	0.9277544
PPP3R1	0.47904205
PRKAA1	0.7285137
PRKAB2	1.0894008
PRKCA	1.277164
PRKCB	0.91753864

PRKCQ	1.2901917
PRKX	1.8347335
PRMT10	-0.87988
PRMT1	0.16884708
PSTPIP1	1.2061639
PTGS2	6.2398024
PTK2	0.57645607
PTPN11	0.5402045
PTPN6	0.86703825
PTPRC	2.0226564
PXN	1.3634577
PYCARD	1.3426914
RAC2	1.3986683
RAC3	1.7256742
RAP1A	0.5527749
RAP1B	1.5412989
RAPGEF2	0.25174904
RASA1	0.36730003
RASA2	2.259202
RASGRF1	0.6192608
RASGRP1	1.2764997
RASGRP4	2.1191669
RELA	0.9156637
RELB	2.6883588
RELT	1.4378161
RIPK1	0.45142078
RIPK2	2.995835
RPS6KA2	2.1252697
RPS6KA3	2.0062404
RPS6KA4	0.18949413
RPS6KA5	0.5891967
RPS6KA6	0.3327148
RRAS2	0.42335367
S1PR1	2.113883
S1PR4	2.4199169
SAMD4B	1.0625854
SELE	5.229238
SETD7	1.1421766
SGK1	1.0630302
SH2D2A	1.3051696
SIRT1	0.8584819
SMAD3	0.7149377
SMAD4	0.5806265
SOCS1	1.656045
SOCS2	2.016519

SOCS3	3.4395528
SOCS4	0.82553387
SOCS5	1.4294734
SOCS7	0.9182911
SOS2	0.60000134
SPHK1	7.3345294
SPRED1	1.5664315
SPRY2	1.8779514
SPRY4	0.5404625
SRF	0.52607775
STAM	0.46688318
STAT1	0.62836695
STAT3	0.8424425
STK3	0.7081084
STK4	1.0910583
TAB2	0.34160948
TBK1	1.1977406
TEK	0.8382416
TGFB1	0.4125886
TGFB2	4.083831
TGFB3	2.2638679
TGFBR1	0.7626753
TGFBR2	1.3231001
THBS1	6.1650934
THBS3	4.5941486
THBS4	1.3332331
TLR2	2.857677
TLR4	1.987256
TLR6	1.3309684
TNF	4.154597
TNFRSF11A	0.8791108
TNFRSF12A	1.0839624
TNFRSF13C	2.3890948
TNFRSF18	1.8865471
TNFRSF1B	1.2936821
TNFRSF21	1.1518412
TNFRSF25	1.9870076
TNFRSF4	1.2138343
TNFSF10	2.2230396
TNFSF13B	3.1026287
TNFSF14	2.7104964
TNFSF15	3.6604176
TNFSF8	0.5036111
TNFSF9	4.6423254
TNN	4.314877

TNXB	0.7817874
TRAF2	0.50108004
TRAF3	0.8752098
TRAF6	0.8738661
TSLP	4.0877614
VAV1	1.5279889
VAV3	0.76574135
VCAM1	2.0893917
VEGFA	1.8181062
VWF	3.0755148
XCL1	1.8451285
YWHAH	0.7903938
ZAK	2.565467

Lung_late

Gene	Log2 Fold Change
ZAK	2.0452528
WAS	0.74765587
VWF	1.6854692
VEGFA	1.2638874
VCAN	2.3611517
VCAM1	0.64147043
VAV3	0.82796955
VAV1	1.6077347
USP7	0.26909256
TSLP	2.9447913
TRAF6	0.5436778
TRAF3	0.5675473
TNN	2.4856093
TNFSF9	4.410564
TNFSF8	0.7684989
TNFSF15	2.6855485
TNFSF14	1.8618245
TNFSF13B	2.3018198
TNFSF11	1.3256779
TNFRSF4	1.4969172
TNFRSF25	0.9008632
TNFRSF21	1.1721253
TNFRSF1B	1.5765085
TNFRSF18	2.324698
TNFRSF17	0.28229785
TNFRSF13C	2.9831812
TNFRSF12A	1.8393402
TNFRSF11A	0.9960127
TNFRSF10B	2.8815746
TNF	3.7834496
TNC	1.4975402
TLR6	1.0308485
TLR4	1.5636492
TLR2	1.6774573
TIGIT	3.6209788
TIAM1	1.2450492
THBS4	3.7257855
THBS3	2.6157742
THBS1	6.55743
TGFBR2	1.6033506
TGFBR1	0.98875713
TGFB3	1.7257748
TGFB2	2.7732306

TEK	0.15780067
TBK1	1.2851996
TAB3	0.44628
STK4	0.9083972
STK3	0.58242655
STAT3	1.1934619
STAT1	0.37354946
STAM2	1.0407138
STAM	0.6151786
SRF	0.5676975
SPRY2	2.1336806
SPRED1	1.4605398
SPP1	0.15917444
SPN	1.527298
SPHK1	8.527199
SOS2	0.4980402
SOCS7	0.657146
SOCS5	0.782423
SOCS4	0.41125202
SOCS3	4.0248637
SOCS2	2.1204147
SOCS1	1.6230025
SMAD4	0.58476496
SMAD3	1.1446466
SIRT1	0.48348427
SHC4	0.5642314
SH2D2A	0.5522232
SGK1	1.2314005
SETD7	0.637825
SELPLG	1.3456335
SELP	6.1561007
SELL	2.9601846
SELE	6.237152
SDC4	0.3266015
SAMD4B	0.8918271
S1PR4	1.9378417
S1PR1	2.0484815
RPS6KB1	0.62437916
RPS6KA4	0.37386465
RPS6KA3	1.7599187
RPS6KA2	2.210785
ROCK2	1.7911196
ROCK1	0.77979517
RIPK2	2.5903394
RIPK1	0.6664424

RHOA	0.26532316
RELT	1.0461106
RELB	2.1321206
RELA	0.74139357
RASGRP4	0.83943987
RASA2	2.1330843
RAP1B	1.1863432
RAP1A	0.5900388
RAC3	1.7309184
RAC2	1.1675053
PYCARD	1.8572445
PXN	1.6619797
PVR	3.8597398
PTPRC	1.2305136
PTPN11	0.5375447
PTGS2	6.7818155
PSTPIP1	1.361382
PRMT1	0.5083232
PRKX	1.2815495
PRKCQ	0.7634604
PRKCD	1.2187867
PRKCB	0.74161196
PRKCA	0.4711795
PRKAB2	0.2856145
PREX1	0.2893648
PPP3CC	0.86762524
PPP2R5B	0.4674859
PPP2R2A	0.81175995
PPP2CB	0.6208973
PPBP	0.46439886
PLK2	0.9400983
PLD2	0.39085293
PLCG2	1.0147638
PLCB3	0.46345425
PLCB2	0.69713664
PLAU	2.147454
PLA2G4F	1.554033
PLA2G4D	2.237095
PLA2G4A	0.64863205
PKN2	0.5005865
PIM1	2.7136235
PIK3R5	2.1143775
PIK3R3	1.2640414
PIK3CD	0.48172235
PIK3AP1	1.1570759

PIAS3	0.37434602
PIAS1	0.24144077
PHLPP2	0.48183107
PGF	1.2085481
PF4	2.13722
PECAM1	1.8081269
PDGFRB	0.36302376
PDGFB	1.9077516
PDGFA	1.9476962
PDCD1LG2	1.3529508
PDCD1	2.3510494
PCK2	0.5455899
PAK2	0.61969376
OSMR	3.8426318
OSM	4.328584
NTRK2	2.9319053
NTNG2	4.066696
NTF5	0.36728215
NRCAM	5.0544844
NRAS	1.1408324
NR4A1	4.7932887
NOS3	1.4072819
NOD2	3.350358
NLRC4	1.1881046
NLK	0.14549065
NLGN3	0.5977442
NFKBIE	1.3137097
NFKBIA	2.9988866
NFKB1	1.6841941
NFATC1	1.2170839
NFASC	2.6087308
NEO1	0.51161957
NCK2	0.7011013
NCF1	1.8025889
NAIP6	0.5756221
NAIP1	0.24072242
MYD88	1.457026
MYC	2.0321965
MYB	2.397955
MPZL1	1.042315
MMP9	4.664946
MMP3	7.348541
MMP2	3.345994
MMP14	0.4488573
MLKL	0.69699097

MEFV	2.315154
MEF2C	1.0044878
MDM2	0.17502022
MCL1	0.93355083
MBL2	-6.813462
MBL1	-4.603528
MASP2	-5.6172276
MASP1	-5.5993795
MAPT	2.2219925
MAPKAPK3	2.081365
MAPK7	1.1563973
MAPK13	4.022091
MAPK12	0.49197865
MAPK11	0.7786231
MAPK10	2.7744236
MAP4K4	1.939044
MAP4K1	1.508429
MAP3K8	2.512198
MAP3K7CL	-2.8347774
MAP3K7	0.28575993
MAP3K6	5.0145845
MAP3K3	0.88606405
MAP3K14	0.8995886
MAP3K1	0.66468716
MAP2K1	0.50737953
MALT1	2.6805763
MAG	0.8002918
MADCAM1	2.069503
LYN	0.65457535
CLTB	0.71789885
LTB	0.5080843
LRRC4B	2.7860303
LRRC4	0.26604223
LPAR3	3.6523204
LPAR1	3.6387627
LIF	1.1804101
LCP2	0.49703765
LAMC3	0.75221515
LAMC2	7.762702
LAMC1	1.3958383
LAMB2	1.4057393
LAMB1	1.2940497
LAMA5	2.9092116
LAMA4	2.3137937
LAMA2	0.6962223

L1CAM	1.1483574
KRAS	0.45829868
KNG2	-4.413828
KNG1	-3.3913126
KLKB1	-8.030532
KLF2	1.6643157
KITL	5.24218
KIT	1.4887748
JUND	0.58947563
JUNB	2.8101377
JUN	3.9256206
JAM3	1.382802
JAM2	0.5825348
ITPR3	1.8189054
ITK	1.4300728
ITGB8	1.323009
ITGB4	2.5462008
ITGB3	3.0878954
ITGB2L	3.9498715
ITGB2	0.9821358
ITGB1	0.5748148
ITGAV	0.6529298
ITGAM	1.5239978
ITGA7	1.3154354
ITGA6	2.045701
ITGA4	1.3683
ITGA3	2.4683657
ITGA2B	1.1161757
ITGA2	0.6890826
ITGA11	1.4552181
ITGA1	0.9506445
IRS2	1.7258911
IRF7	0.1748352
INPP5D	1.6885242
INHBB	6.695317
IL7R	5.643655
IL6ST	0.54155016
IL6	9.970649
IL4RA	2.5053735
IL3RA	0.44489384
IL2RG	1.2892513
IL2RB	1.0919466
IL2RA	4.819016
IL23R	0.9036491
IL23A	2.3482237

IL22RA2	2.5328484
IL22	0.78087616
IL21R	0.97877145
IL20RB	1.5779648
IL1R2	7.9986944
IL1B	3.7900834
IL1A	1.5567865
IL18RAP	3.772426
IL18R1	3.4209142
IL17RA	2.0093846
IL17A	4.654379
IL12RB2	0.6264956
IL11	5.247109
IL10RB	1.1920409
IL10RA	0.26600552
IL10	3.2818475
IKBKE	0.77524424
IGF1R	2.8095655
IFNLR1	3.0288448
IFNGR1	0.77543116
IFNG	4.575471
IFNAB	0.97302294
IFNA5	0.87249565
IFNA14	0.5930052
IFNA1	0.16362357
ICOSL	2.3906593
ICAM2	1.327426
ICAM1	3.3403993
HSPB1	6.246183
HSPA2	1.5885916
HOMER3	0.8972025
HCK	1.4818735
HC	-1.5593138
HBEGF	4.1092362
H2-T24	2.0622087
GYS1	1.7983017
GSK3B	0.46723032
GRK5	2.5780983
GRK4	0.77311635
GRAP2	0.79058003
GNB5	1.4316125
GNB4	0.92256165
GNAQ	0.7829528
GNAI1	1.9498744
GM1987	1.9489446

GM13304	1.7035847
GLYCAM1	4.344466
GLG1	0.61619425
GADD45G	1.7044864
GADD45B	3.2173328
GADD45A	0.70815134
FYN	1.2369471
FOXO6	1.7624094
FOS	5.324674
FLT3	1.539484
FLT1	2.2755814
FLNC	4.7369657
FLNB	0.73902273
FLNA	1.6838555
FIGF	0.7428055
FGR	0.92225504
FGG	-1.2742682
FGFR2	0.42812538
FGFR1	2.98215
FGF7	4.857344
FGF6	0.5836663
FGF2	0.1971159
FGF18	0.68637276
FGF11	0.7123091
FGF10	3.1837902
FGB	-1.5195751
FGA	-1.4732561
FBXO32	2.2857213
FASL	0.7055867
FAS	0.19836473
F9	-4.541
F8	-5.1338806
F7	-3.2881508
F5	-5.6914816
F2R	2.1768146
F2	-4.4143066
F13B	-7.733657
F12	-5.388242
F11	-5.1155505
F10	-3.0428324
ESAM	2.456534
ERBB2IP	0.5935888
EPHA2	0.57496643
EP300	0.40212202
ELK4	1.2293129

EFNA4	1.2684247
EDN1	4.9202104
DUSP8	3.2829816
DUSP7	1.2820086
DUSP5	2.79953
DUSP4	3.2240245
DUSP2	3.9149075
DUSP16	1.2796755
DUSP1	3.857088
DOCK2	1.1569562
DDIT4	2.108601
DDIT3	1.18191
DAPP1	1.9813213
CXCR5	2.146194
CXCR4	2.9280877
CXCR2	2.995656
CXCR1	3.6259289
CXCL5	9.573448
CXCL3	6.8541846
CXCL2	8.005163
CXCL16	1.6186166
CXCL15	8.608767
CXCL14	7.74806
CXCL13	1.6614156
CXCL10	2.8854737
CXCL1	4.8407164
CX3CR1	0.9450669
CX3CL1	4.4861307
CTSK	4.3999357
CSNK1E	1.5740066
CSF3	8.046267
CSF2RB2	0.95553493
CSF2RB	1.2450933
CSF2RA	1.3021107
CSF2	5.5412235
CSF1	1.0551028
CRLF2	0.6269026
CRK	0.74908686
CREB3L2	0.6686063
CREB3L1	2.7341866
CREB3	0.23549366
CREB1	0.953758
CR2	-0.8204019
CPB2	-2.886948
COMP	1.6020968

COL6A2	1.3582196
COL6A1	2.4246302
COL5A2	0.65350795
COL4A6	0.99782467
COL4A4	1.5852437
COL4A3	3.5774088
COL4A2	3.1843548
COL4A1	3.485456
COL1A2	0.92870426
COL1A1	0.8451123
CLDN8	0.6912329
CLDN5	4.118408
CLDN4	0.88523173
CLDN22	3.7501366
CLDN18	1.3436415
CLDN10	5.7931757
CISH	1.7294979
CHRM2	3.0229192
CHAD	4.531857
CFI	-5.2741585
CFH	-2.351603
CFB	-1.7198343
CEBPB	0.39219904
CDKN2D	1.6419034
CDKN2B	5.3669114
CDK2	0.14747453
CDH5	2.4326844
CDH15	2.198609
CDH1	1.7390733
CDC25B	1.4985504
CD8B1	1.2814999
CD80	0.8098655
CD79B	1.3902636
CD79A	1.8077974
CD72	0.28123522
CD70	1.0856862
CD6	1.4332328
CD59B	-4.3090315
CD40	2.668353
CD3G	0.23918581
CD3D	0.7945304
CD34	3.710321
CD274	2.644576
CD2	1.8432856
CD19	2.063467

CD14	3.3234658
CCR8	1.2829981
CCR7	5.3706584
CCR1L1	0.67022014
CCR10	0.8565016
CCNG2	0.35839343
CCND2	2.3981524
CCL8	1.6231565
CCL7	4.9070687
CCL6	1.2765617
CCL5	1.4234304
CCL4	5.2205024
CCL3	6.53883
CCL2	4.757485
CCL17	8.273239
CCL12	3.6947422
CCL11	5.5829077
CBLB	1.9600844
CBL	2.7272897
CASP1	0.5162144
CARD11	1.2655902
CAMK2D	1.714241
CAMK2B	0.19525313
CALML3	4.139661
CALM4	4.4943476
CALM1	0.3385439
CACNB3	1.6161375
CACNB1	0.45416355
CACNA2D4	1.4144635
CACNA2D2	0.56573534
CACNA2D1	3.4146247
CACNA1S	1.910387
CACNA1D	2.0253427
CACNA1C	3.2129717
C9	-7.750138
C8G	-7.08279
C8B	-4.738978
C8A	-5.108748
C6	-1.2727351
C4BP	-4.9429207
C4B	-2.8549414
C3	-1.4945536
C2	-2.3774672
C1S2	-1.1009212
C1QC	-2.555378

C1QB	-2.2561574
C1QA	-2.404663
BTK	1.0940089
BRAF	0.95715284
BMPR2	3.249743
BLNK	2.9537716
BIRC3	3.071001
BCL6	1.4016328

Liver_onset

**Genes**	**Log2 Fold Change**
APAF1	2.1144776
ARF6	1.8438458
ARPC1A	0.9504733
ARPC1B	1.9371085
ARPC3	1.2726064
ARPC4	1.4588189
ARPC5	0.98877144
ASAP3	1.3654456
ATF2	1.1681762
ATF4	2.428388
ATG12	1.2063689
AZI2	1.879756
B2M	1.5585384
BAD	0.8827567
BAX	2.3578677
BCL10	2.5660462
BCL2A1C	3.2369423
BCL2A1D	2.7446132
BCL2L1	2.414969
BCL2L13	0.9952736
BCL3	4.8442125
BID	2.9501457
BIRC3	2.724102
CALR	2.4570813
CANX	1.7151651
CASP1	1.507772
CASP6	1.276854
CASP7	1.927382
CCL2	6.036657
CCL3	5.719592
CCL4	4.430876
CCL5	1.3589654
CD14	6.529488
CD40	2.4682698
CD74	1.5408154
CD86	1.2704403
CCDC42	-2.2314446
CDC42	1.3695054
CEBPB	3.3846107
CFL1	1.4677305
CFL2	1.5052915
CREB5	1.9641981
CRK	1.9467292

CSF1	2.8987136
CSF2RB	2.5957346
CSNK2A2	1.3440485
CTSB	2.2591586
CTSS	0.83475494
CXCL1	5.365581
CXCL2	8.263842
CXCL3	3.6419559
CXCL9	4.479806
DDX3X	1.835401
DDX58	1.7575588
DFFB	1.1254168
DHX58	1.6179304
DNM1L	1.2193375
DNM2	1.6740384
ERBB2IP	1.1416659
FAS	3.616722
FCGR2B	2.481648
GADD45B	2.7043138
H2-AA	1.5925322
H2-AB1	1.0541954
H2-D1	1.4035072
H2-EB1	0.90928745
H2-K1	1.8576622
H2-M2	1.654685
H2-Q2	2.0738263
H2-Q7	1.9920216
H2-Q8	1.8150468
H2-T23	1.8448248
HCK	1.7694583
HSP90AA1	1.747529
HSP90AB1	1.5677366
HSPA1A	3.709042
HSPA2	1.3318233
HSPA4	2.062583
HSPA5	2.98735
HSPA8	2.1307163
ICAM1	2.3301744
IFIH1	1.9949088
IFNAR1	2.0860157
IFNAR2	2.5267382
IFNG	2.190075
IKBKB	1.7780247
IKBKE	4.272011
IL1B	4.750852

IL6	7.8417587
IRAK1	0.77092505
IRAK2	1.3855486
IRAK3	4.8203144
IRAK4	1.4310675
IRF3	2.042357
IRF7	1.5674634
ISG15	3.6198869
JUN	1.8008885
LTB	1.9147286
LYN	2.408308
MALT1	2.6235409
MAP2K1	2.3460078
MAP2K3	1.9981256
MAP3K1	1.5065665
MAP3K5	1.4613299
MAP3K7	1.6863675
MAP3K8	3.7303
MAPK14	1.3038864
MAPK9	0.9723015
MARCKS	1.6384606
MEFV	4.6734195
MMP9	2.8503413
MYD88	3.268651
NAIP2	1.3266621
NAIP5	2.4703164
NCF1	2.6594706
NFKBIA	3.3306222
NFKBIB	3.4757843
NFYA	1.7913761
NFYB	2.2752237
NLRC4	1.9987414
OTUD5	1.7813206
PARP1	1.3216214
PGAM5	1.6612492
PIAS4	1.1725798
PIK3CB	0.740613
PIK3R1	0.9566884
PIK3R5	2.5419044
PIN1	1.5322361
PIP5K1A	6.4570208
PLA2G4A	1.3903532
PLD1	1.0436683
PPP3CB	0.8888669
PPP3R1	1.1859093

PRKACB	1.1820965
PRKCB	1.3298774
PRKCD	1.6032434
PSME1	1.9823742
PSME3	1.9212136
PTGS2	3.1314769
PTPRC	1.4612212
RAC2	1.9911137
RELA	2.522079
RELB	2.0631094
RFXAP	1.4446907
RIPK1	2.824726
RNF125	3.695417
RPS6KB1	1.4120283
SIKE1	0.9595876
SOCS3	4.8660665
SPP1	1.5827289
SUGT1	1.5401044
TAB2	1.2883873
TAB3	1.3730497
TANK	2.3361912
TAP1	2.2094197
TAP2	2.521069
TAPBP	2.0654993
TBK1	2.9431362
TLR2	4.3165045
TLR3	1.1375089
TLR6	2.3953714
TMEM173	2.2276697
TNF	5.8694487
TNFRSF1A	2.1380758
TNFRSF1B	1.6970997
TNFSF14	1.6440063
TOLLIP	1.3194857
TRADD	1.3656964
TRAF2	2.5938106
TRAF3	1.5550995
TRAF6	1.6272502
TRIM25	1.6859136
TRIP6	1.8839488
UBE2I	0.9876461
VASP	2.3511057
VAV1	1.7400427
VCAM1	3.0385032

Liver_late

**Genes**	**Log2Fold Change**
C1QA	2.0641377
C1QB	1.9021208
C1QC	1.609268
C1S2	2.8937666
C2	4.0796986
C3	3.7652035
C4B	4.2947817
C8A	4.7938757
C8B	5.4072266
C8G	5.0531435
C9	4.912554
CADM1	-1.0697734
CADM3	-1.4279401
CD2	-1.9864731
CD34	-2.798125
CD40LG	-3.7962584
CD6	-0.8451831
CD8A	-1.6508737
CD8B1	-1.087054
CDH1	-2.5712314
CDH5	-0.630861
CFB	5.9501534
CFH	4.549362
CFI	9.88666
CLDN13	-4.071478
CLDN15	-3.1823359
CLDN16	-4.2633886
CLDN17	-3.3157594
CLDN18	-5.17022
CLDN20	-1.3730004
CLDN4	-2.84872
CLDN5	-5.718268
CLDN6	-3.348473
CLDN8	-2.7479033
CLDN9	-3.4963312
CNTN2	-1.9606357
CPB2	10.885954
CR1L	1.6542985
DAF2	1.7736428
ESAM	-0.7831013
F10	6.367607
F11	8.4922495
F12	12.398761

F13B	9.064068
F2	10.92795
F5	4.538271
F7	3.4095504
F9	3.6609108
FGA	9.173074
FGB	9.663299
FGG	10.698914
GLYCAM1	-4.8599806
H2-AA	-0.6046169
H2-AB1	-0.9148462
H2-DMA	-7.1505904
H2-DMB1	-2.1257384
H2-DMB2	-1.9394491
H2-EB1	-1.5659902
H2-M1	-2.947433
H2-M9	-1.637775
H2-OA	-1.0264516
H2-OB	-2.4911797
H2-T18	-2.9834757
H2-T24	-0.7686567
HC	1.7227013
ICAM2	-1.9542243
IL1B	2.6184552
IL6	7.301508
IRAK1	0.78391576
IRAK4	0.5209422
IRF3	1.9987185
IRF7	2.2491696
IRS1	0.5869281
IRS2	3.9137294
ITGA4	-0.5322883
ITGA6	-1.2064121
ITGA8	-8.241262
ITGB2L	-2.6846042
ITGB7	-1.7952635
ITGB8	-3.4270377
JAM3	-0.4149952
JUN	1.7830541
L1CAM	-2.659653
LBP	4.678337
LRRC4	-3.4226708
LY96	2.6905177
MADCAM1	-1.4309137
MAP2K1	2.6594136

MAP2K2	2.5691159
MAP2K3	1.6721599
MAP2K4	1.4800174
MAP3K7	1.9393547
MAP3K8	3.5246217
MAPK14	0.7399318
MAPK9	0.9235642
MTOR	2.4041243
MYD88	2.5263698
NFKBIA	2.5546033
NFKBIB	3.7119367
NFKBIE	1.468838
NLGN1	-2.3180912
NPY	3.8314302
NRCAM	-5.355447
NRXN2	-1.7576151
NTNG1	-4.9911966
NTNG2	-0.6750443
PCK1	6.8706536
PDCD1	-0.7215135
PECAM1	-1.3196194
PIK3CB	1.1492751
PIK3R1	0.5093167
PIK3R2	1.1095321
PIK3R5	1.5761268
PPARGC1A	4.914756
PRKAA1	1.036577
PRKAA2	2.2955096
PRKAB2	0.8194208
PRKAG1	1.3975909
PRKAG2	0.9413302
PTPN11	1.483566
PTPRC	-0.5122116
PTPRM	-3.2053742
PVRL3	-1.6524374
RELA	2.9477847
RIPK1	2.6817415
RXRA	2.3270087
RXRB	2.6801684
RXRG	0.8702009
SLC2A1	3.5512712
SOCS3	4.5939703
SPP1	1.431256
STAT3	3.1285164
STK11	1.4644239

TAB1	0.37772393
TAB2	1.8461864
TBK1	3.2366788
THBD	-2.5943391
TICAM1	1.1511948
TLR1	1.7871199
TLR2	3.1554701
TLR3	1.4207106
TLR6	2.1066854
TLR9	2.0769737
TNF	4.244586
TNFRSF1B	1.2645538
TOLLIP	1.6692741
TRADD	1.9492948
TRAF2	2.5141637
TRAF3	1.0559762
TRAF6	1.5576911
