# Supplementary material for: Genome wide host gene expression analysis in mice experimentally infected with Pasteurella multocida
Source: PLoS One. 2017 Jul 13;12(7):e0179420. doi: 10.1371/journal.pone.0179420 (PMC5509158; doi:10.1371/journal.pone.0179420)
Supplement: S1 File — (PDF) [file pone.0179420.s010.pdf]

Mice_onset_spleen_DE_genes_fc

Gene	log2 Fold Change
AAAS	-1.1081614
AAED1	-0.6094956
AAGAB	-0.3715901
AAMDC	-0.7769148
AARS	-0.4350281
AARSD1	-0.4622111
AASS	-3.2934823
AATF	-0.4051771
AATK	-1.5614734
ABAT	-1.26049
ABCA2	-0.4896455
ABCA3	-0.3024902
ABCA7	-0.5232725
ABCA8A	-2.4815114
ABCA8B	-2.5012186
ABCA9	-3.6531928
ABCB10	-1.1183777
ABCB4	-1.3778749
ABCB6	-1.8042278
ABCB7	-0.8217959
ABCB8	-0.4144793
ABCC3	-2.5026407
ABCC9	-0.7629452
ABCD1	-1.0336969
ABCD2	-2.3271794
ABCD4	-0.1774926
ABCF2	-0.2728663
ABCG1	-0.6242147
ABCG2	-1.2413669
ABCG3	-1.4159253
ABCG4	-1.3622556
ABHD1	-0.4988551
ABHD10	-0.4758031
ABHD11	-0.4339414
ABHD12	-0.8446856
ABHD14B	-0.7966716
ABHD16A	-0.7772727
ABHD17A	-0.6799817
ABHD4	-0.9494858
ABHD5	-0.398056
ABHD6	-0.3457077
ABHD8	-0.3427458
ABI3BP	-0.2848938

ABL1	-0.7585282
ABTB1	-0.6223116
ACAA2	-0.7274861
ACACA	-0.5378742
ACACB	-1.4677246
ACAD10	-0.2141581
ACAD12	-0.4031947
ACADL	-0.3438821
ACADM	-0.748848
ACADS	-1.1947529
ACADVL	-0.4525008
ACAP3	-0.8508253
ACAT1	-0.9403515
ACAT3	-0.5055242
ACBD4	-0.8625708
ACBD6	-0.8991246
ACCS	-0.3137159
ACD	-0.3716693
ACE	-1.4714704
ACER3	-0.3864188
ACHE	-1.2374642
ACKR1	-1.894939
ACKR3	-0.4320736
ACKR4	-1.4766409
ACLY	-0.2151856
ACMSD	-2.0395768
ACNAT2	-0.3830395
ACO1	-0.456001
ACO2	-0.3699088
ACOT1	-1.1463907
ACOT11	-0.8038998
ACOT13	-0.6315985
ACOT2	-0.3774633
ACOT6	-0.6699174
ACOT8	-0.2986774
ACP1	-0.8378701
ACSF3	-0.4667978
ACSL1	-0.7436118
ACSL5	-0.4621592
ACSL6	-0.5373285
ACSS1	-0.7383094
ACSS2	-1.2841713
ACTL6A	-0.9709673
ACTR10	-0.3050308
ACTR1A	-0.4805341

ACTR8	-0.6182208
ACVR2B	-0.8964365
ACVRL1	-0.4276085
ACY1	-0.2933691
ACY3	-2.5495112
ACYP2	-0.2893617
ADAM10	-0.9183335
ADAM22	-1.9708323
ADAM33	-0.2529903
ADAMDEC1	-1.1601601
ADAMTS15	-0.3488071
ADAMTS16	-1.8044138
ADAMTS3	-0.1867909
ADAMTSL2	-0.6154304
ADAMTSL5	-0.9237418
ADAP2	-0.9125447
ADCY1	-2.6824827
ADCY2	-0.7473187
ADCY3	-0.5848532
ADCY5	-0.7965212
ADCY6	-0.6925588
ADCY7	-0.3579245
ADCY9	-1.4262109
ADD1	-0.7230635
ADD2	-1.913733
ADD3	-0.7009502
ADIG	-0.9150147
ADIPOQ	-3.6276762
ADIPOR1	-0.7520094
ADK	-0.5053282
ADNP2	-0.2591524
ADORA3	-0.4536221
ADPRHL2	-0.2525034
ADRA1D	-1.6357365
ADRB1	-1.5901811
ADRB2	-0.2826214
ADRB3	-1.3249574
ADSS	-0.6705833
ADSSL1	-0.8151374
AEBP1	-0.2626185
AF251705	-1.2305107
AFAP1	-0.6011396
AFAP1L1	-0.9895561
AFAP1L2	-1.418262
AFG3L1	-0.3929634

AFG3L2	-0.5162673
AFMID	-0.8878346
AGAP1	-0.5651822
AGBL1	-2.1525364
AGFG2	-1.3432646
AGL	-1.0940442
AGMO	-1.3120759
AGO1	-0.4140744
AGO2	-1.130835
AGO4	-0.4607551
AGPAT2	-0.2953219
AGPAT4	-0.354527
AGPS	-0.2413077
AGTPBP1	-0.3210487
AGTR1A	-0.6857758
AGTRAP	-0.3514924
AHCTF1	-0.564899
AHCY	-0.3403974
AHCYL1	-0.2942476
AHDC1	-0.6076565
AHNAK	-0.370575
AHRR	-1.5959096
AHSP	-1.135745
AIF1	-0.3614397
AIFM1	-0.282701
AIFM2	-1.2656503
AIG1	-0.9814196
AIMP1	-0.2125034
AIP	-0.4838681
AK1	-2.3612928
AK3	-1.1138129
AK6	-0.272285
AK8	-1.1778402
AKAP11	-0.4242802
AKAP8L	-0.4439011
AKIP1	-1.5009398
AKNAD1	-0.9928169
AKR1A1	-0.4689627
AKR1B10	-0.7719789
AKR1B7	-2.8820393
AKR1B8	-0.4830198
AKR1C13	-0.2603102
AKR7A5	-0.4945459
AKT1	-0.5149775
AKT1S1	-0.4202509

ALAD	-1.6866851
ALAS2	-1.103662
ALCAM	-0.3536706
ALDH16A1	-0.2377877
ALDH18A1	-1.0835614
ALDH1A1	-0.828629
ALDH1A7	-0.9344695
ALDH1L1	-0.8375068
ALDH2	-0.6488233
ALDH3B1	-0.6064968
ALDH3B2	-2.1675475
ALDH4A1	-0.6205897
ALDH6A1	-0.4584971
ALDH9A1	-0.4289813
ALDOC	-0.4811478
ALG13	-0.5022059
ALG2	-0.4446225
ALG5	-0.2762585
ALG6	-1.086601
ALG9	-0.5803804
ALKBH7	-0.5651383
ALKBH8	-0.8201089
ALMS1	-0.5750089
ALOX12	-1.1308966
ALOX12B	-0.2712562
ALOX12E	-3.0897586
ALOX15	-3.6403115
ALOX5	-1.2491975
ALOX5AP	-0.5409083
ALOXE3	-2.9196155
ALS2CR12	-0.8403153
ALYREF	-0.7282052
ALYREF2	-0.5690789
AMD2	-0.5768085
AMDHD2	-0.4061775
AMER1	-0.2431741
AMMECR1	-0.5925269
AMOT	-1.86271
AMPD3	-1.435956
AMZ1	-0.9002705
ANAPC1	-0.2081046
ANAPC10	-0.8786244
ANAPC15	-0.569232
ANAPC4	-0.2767582
ANAPC5	-0.7775784

ANGEL2	-0.2808962
ANGPT1	-0.9904516
ANGPTL2	-2.0759478
ANGPTL6	-0.8285132
ANGPTL7	-1.1321316
ANK	-1.3317056
ANK1	-1.3060684
ANK3	-0.6904235
ANKLE1	-1.3078747
ANKMY2	-0.7234576
ANKRD13A	-0.812583
ANKRD13B	-0.371192
ANKRD2	-2.358388
ANKRD24	-1.558269
ANKRD26	-0.5122492
ANKRD29	-0.4700086
ANKRD32	-0.7100315
ANKRD35	-0.8470357
ANKRD39	-0.2416587
ANKRD40	-0.2692373
ANKRD50	-1.1190543
ANKRD54	-0.9159765
ANKRD61	-0.2865098
ANKRD9	-1.216249
ANKS1	-0.3315601
ANLN	-0.793911
ANO1	-1.9532299
ANO10	-0.4288096
ANO6	-0.2004509
ANP32A	-0.4884181
ANP32B	-0.3923764
ANP32E	-0.3702188
ANTXR1	-0.9197235
ANXA8	-0.6550348
ANXA9	-0.2816327
AOX1	-2.1087322
AP1AR	-0.6661587
AP1M1	-0.306304
AP1S1	-0.4100051
AP2A1	-1.3934164
AP2A2	-1.4049568
AP2B1	-0.654346
AP2M1	-0.2617545
AP2S1	-0.4184389
AP3D1	-0.1792998

AP3S1	-0.2749567
AP3S2	-0.3073659
AP4B1	-0.6682305
AP4M1	-0.986908
AP5B1	-0.2051134
APAF1	-0.2847004
APBA1	-2.3831398
APC2	-0.8119464
APCDD1	-1.7675619
APEH	-0.8806725
API5	-0.5179071
APLF	-0.8377147
APLNR	-2.4473612
APLP2	-0.5174551
APMAP	-0.4898863
APOA1BP	-0.5447044
APOBR	-0.4477086
APOC1	-0.3880048
APOC4	-1.4455671
APOE	-0.2471652
APOF	-2.0774646
APOL10A	-1.6321363
APOL10B	-0.7781177
APOL11B	-1.2274323
APOL8	-1.8691325
APOO	-0.6337915
APOOL	-0.4787669
APPL2	-1.5748801
APRT	-0.3047271
AQP1	-1.5611434
AQP11	-1.1747606
AQP2	-0.9345198
AQP9	-1.5404077
ARAP3	-0.2753544
ARF2	-0.473156
ARF5	-0.9124551
ARF6	-0.6400642
ARFGAP3	-0.3915458
ARGLU1	-0.2935934
ARHGAP10	-1.623204
ARHGAP12	-0.9662595
ARHGAP15	-0.2955508
ARHGAP18	-1.73283
ARHGAP19	-0.6093874
ARHGAP20	-1.0522473

ARHGAP21	-0.5143876
ARHGAP23	-0.9385719
ARHGAP33	-2.0145848
ARHGAP39	-0.8987794
ARHGAP44	-1.2672029
ARHGAP5	-0.4931536
ARHGAP6	-0.7979732
ARHGAP9	-0.3975468
ARHGEF1	-0.2433715
ARHGEF10L	-0.3081527
ARHGEF12	-0.6800962
ARHGEF17	-0.7993956
ARHGEF2	-0.1935911
ARHGEF25	-0.7352428
ARHGEF39	-0.9339542
ARHGEF4	-0.9461815
ARHGEF40	-1.2967134
ARHGEF9	-1.8468416
ARID4A	-0.5321965
ARL11	-3.21238
ARL2	-0.3658423
ARL2BP	-0.3962064
ARL4A	-1.1896315
ARL4D	-1.0081918
ARL6	-0.4362187
ARL6IP4	-0.3863802
ARL6IP5	-0.4338293
ARMC1	-0.5021739
ARMC10	-0.3769984
ARMC2	-1.5569332
ARMC3	-0.2451773
ARMC5	-0.311152
ARMC6	-0.5483198
ARMC7	-0.5056877
ARMC9	-1.2387071
ARMCX1	-0.8836446
ARMCX5	-0.3919096
ARNT	-0.2201877
ARNT2	-1.5268283
ARPC1A	-0.7073216
ARPC4	-0.4957585
ARRB1	-1.3179111
ARRB2	-0.7031331
ARRDC2	-0.9036112
ARSB	-0.7602854

ARSG	-0.550868
ARSI	-1.2343285
ARSK	-0.7710412
ART3	-0.9749987
ART4	-1.1280556
ART5	-0.7042174
ARTN	-0.9627492
AS3MT	-0.6114125
ASAH1	-1.0456109
ASAP1	-0.6626859
ASB1	-1.1382866
ASB16	-1.6822917
ASB17	-2.0408084
ASB2	-0.7195158
ASB6	-0.6223397
ASCC1	-0.6557384
ASF1A	-0.5514083
ASF1B	-1.0834951
ASGR2	-1.1318142
ASH2L	-0.3008962
ASL	-0.5217385
ASNS	-0.2633176
ASNSD1	-0.3283567
ASPA	-2.6142569
ASPH	-1.0630894
ASPM	-1.1457558
ASRGL1	-0.7385128
ATAD2	-0.4710236
ATAD5	-0.9117003
ATE1	-0.2386251
ATF1	-0.6529594
ATF2	-0.2892952
ATF6B	-0.3526697
ATG10	-0.4896462
ATG101	-0.2490206
ATG12	-0.1934166
ATG14	-0.2710862
ATG2A	-0.770463
ATG4B	-1.1003895
ATG4C	-1.0032613
ATG4D	-0.7006693
ATG7	-0.6147528
ATG9A	-0.3494921
ATL1	-0.8186243
ATL2	-0.4782047

ATOH8	-1.3760941
ATP11B	-0.6503768
ATP13A2	-0.8835845
ATP1A1	-0.536571
ATP1A2	-1.9479728
ATP1A3	-1.5698354
ATP1B2	-2.0785599
ATP2A3	-0.6314697
ATP2B4	-1.127881
ATP5B	-0.2932582
ATP5C1	-0.4121561
ATP5C1-PS	-0.4537711
ATP5D	-0.739717
ATP5E	-0.3513866
ATP5F1	-0.4229431
ATP5G2	-0.2143564
ATP5H	-0.3028603
ATP5J	-0.3722701
ATP5J2	-0.4344559
ATP5K	-0.5426803
ATP5L	-0.3290534
ATP5O	-0.570024
ATP5S	-1.0155463
ATP6	-0.2932868
ATP6AP1	-0.2779985
ATP6AP2	-0.2765546
ATP6V0A1	-1.0254803
ATP6V0B	-0.6032267
ATP6V0C	-0.4320879
ATP6V0D1	-0.4554339
ATP6V0D2	-1.5550904
ATP6V0E2	-0.6400986
ATP6V1E1	-0.5227752
ATP7B	-1.3978078
ATP8A2	-0.4818656
ATPAF2	-0.4269557
ATPIF1	-0.892725
ATRAID	-0.6806927
ATRIP	-0.3048935
ATRX	-0.4166498
ATXN1	-0.4042013
ATXN7L3	-0.181562
ATXN7L3B	-0.2165437
AUH	-0.440412
AUP1	-0.4866505

AURKA	-0.8131867
AUTS2	-0.4778378
AWAT1	-0.3062232
AWAT2	-0.5459259
AXIN1	-0.2778893
AXL	-0.915123
B3GALNT2	-0.4428372
B3GALT1	-0.1946011
B3GAT3	-0.4913688
B3GLCT	-0.4508891
B3GNT1	-0.6768947
B3GNT8	-1.651824
B3GNT9	-1.0296144
B4GALNT2	-0.4042077
B4GALT1	-0.5339975
B4GALT2	-0.8134465
B4GALT4	-1.4300063
B4GALT6	-0.7181349
B630019K06I	-0.8519642
B9D1	-0.7304428
B9D2	-0.1874332
BACE1	-1.0034175
BAD	-0.7057958
BAG1	-0.7677956
BAG2	-1.3382573
BAG4	-0.5069206
BAG6	-0.5864768
BAHD1	-0.3768687
BAI1	-1.1068649
BAI2	-2.1029873
BAK1	-0.7195692
BANF1	-0.8292995
BAP1	-0.6371164
BARD1	-0.7721858
BAZ1B	-0.229743
BAZ2A	-0.8434014
BBIP1	-0.416589
BBS1	-1.0508342
BBS10	-0.3633485
BBS4	-0.356688
BBS5	-0.3663497
BBS7	-1.1093931
BBS9	-0.4862785
BCAM	-1.2268009
BCAP29	-0.575099

BCAR1	-0.4463153
BCAS2	-0.5855489
BCAS3	-0.6269133
BCAT2	-0.6985965
BCCIP	-0.2429895
BCKDHA	-1.1835871
BCKDK	-1.2758007
BCL11A	-0.3077025
BCL2L1	-0.6241565
BCL2L12	-0.5859399
BCL2L13	-0.3204336
BCL2L2	-0.7630665
BCL7A	-0.3006854
BCL7B	-0.2588463
BCL7C	-0.6025224
BCL9L	-0.7750812
BCO2	-0.8364234
BCORL1	-0.6476615
BDH1	-0.2362089
BDH2	-0.3025158
BECN1	-0.7116451
BEND5	-0.1810722
BEND7	-0.6908364
BET1	-0.4846263
BEX4	-0.9968405
BGN	-0.4232025
BICC1	-0.410224
BICD1	-1.0914147
BIK	-1.3528528
BIN2	-0.5253601
BIN3	-0.4089379
BIRC5	-0.9618778
BIVM	-0.6548729
BLCAP	-0.5062337
BLM	-1.3135214
BLOC1S3	-0.5766203
BLOC1S5	-1.0033555
BLVRA	-0.9767003
BLVRB	-1.2776384
BMF	-0.6535883
BMP2	-0.9741015
BMP4	-1.4660149
BMP8B	-1.0392172
BMPR2	-0.4436703
BMX	-1.0603056

BMYC	-0.4159651
BNC1	-1.4884205
BNIP1	-0.5737977
BNIP3L	-0.8704991
BOLA1	-0.2859459
BOLA3	-0.8345661
BORA	-1.0282738
BPGM	-0.4118862
BPHL	-0.7501702
BPIFA6	-0.5730598
BRAP	-0.2529426
BRCA1	-1.1001823
BRCA2	-1.1441307
BRD3	-0.5726714
BRD7	-0.1847115
BRD9	-0.2234101
BRE	-0.3176847
BRF1	-0.5879312
BRF2	-0.5000889
BRINP2	-0.7391353
BRIP1	-0.6740806
BRK1	-0.5278931
BRMS1	-0.383894
BRMS1L	-0.2502942
BRPF3	-0.8233557
BSCL2	-0.7612505
BSDC1	-0.9186053
BSG	-1.1615944
BSPH1	-0.3238599
BTAF1	-0.6159682
BTBD2	-0.9425545
BTBD6	-0.5812535
BTD	-0.8770633
BTF3	-0.254046
BTG2	-0.7994762
BTNL10	-0.6356196
BTNL2	-0.714356
BTNL9	-0.6544013
BTRC	-1.1070211
BUB1	-0.8573804
BUB1B	-1.2982125
BUB3	-0.5232024
BVES	-1.2848761
BYSL	-0.3398509
C1GALT1	-0.4501662

C1QA	-0.6348519
C1QB	-0.4179535
C1QC	-0.861268
C1QTNF2	-1.768409
C1QTNF4	-1.4835019
C1QTNF5	-1.774513
C1QTNF6	-0.9593585
C1QTNF7	-1.5321617
C1RL	-0.3760259
C1S1	-0.3424616
C2CD2L	-0.5765224
C2CD3	-0.5148277
C2CD4C	-0.7063441
C2CD4D	-0.3827984
C3AR1	-1.8699608
C5AR2	-1.2629962
C6	-1.3031902
C8G	-0.7330873
CAAP1	-0.6759892
CABIN1	-0.9144011
CABLES2	-0.3988862
CABYR	-0.5547359
CACHD1	-0.8637748
CACNA1G	-1.5593786
CACNG7	-0.2595236
CACTIN	-0.6014481
CADM1	-0.7703366
CADM3	-0.8171952
CADM4	-0.5547323
CADPS2	-0.4537725
CAGE1	-0.5655103
CALCOCO1	-0.867213
CALM2	-0.5856361
CALM3	-0.9322233
CALM4	-1.0024478
CALML4	-2.10292
CALR3	-0.5937843
CAMK1	-0.8922842
CAMK1D	-0.8138809
CAMK1G	-1.7908874
CAMK2G	-0.3001909
CAMKMT	-0.4295993
CAMP	-0.8864594
CAMSAP2	-1.042028
CAMSAP3	-0.3779371

CAP2	-1.8056417
CAPN1	-0.6911006
CAPN10	-0.3813834
CAPN3	-0.7754543
CAPN5	-0.8138633
CAPN9	-0.9536369
CAPNS1	-0.4553461
CAPRIN2	-0.333765
CAPZB	-0.4749694
CAR1	-1.7250752
CAR2	-1.3727126
CAR3	-4.161031
CAR4	-0.7420273
CAR7	-0.858911
CAR9	-0.6870406
CARD10	-1.0356302
CARD9	-1.9641693
CARF	-0.9003954
CARHSP1	-1.0782261
CARKD	-0.2956252
CARM1	-0.7408805
CASC5	-1.0162976
CASKIN2	-1.2255149
CASP12	-0.9197407
CASP14	-0.6286507
CASP2	-0.3306775
CASP3	-0.5603166
CASP6	-0.8670349
CASP7	-0.3554444
CASP8AP2	-0.5033097
CASP9	-0.5463433
CASQ2	-1.4818094
CAST	-0.6384435
CAT	-0.9344244
CAV1	-2.6803029
CAV2	-2.9423776
CBFA2T3	-0.8147435
CBR1	-0.6172643
CBR2	-3.6499052
CBR3	-1.9289618
CBR4	-0.278554
CBX2	-0.8434174
CBX3	-0.4710484
CBX5	-0.5902572
CBX6	-1.1299

CBY1	-1.2068124
CC2D2A	-1.2047503
CCAR2	-0.6633267
CCBL1	-0.3809404
CCDC103	-1.336252
CCDC104	-0.6980162
CCDC106	-0.7117145
CCDC12	-0.382864
CCDC124	-0.3759866
CCDC125	-0.6313155
CCDC127	-0.3420501
CCDC132	-0.2898512
CCDC136	-1.3512459
CCDC138	-0.2313938
CCDC144B	-0.2481377
CCDC150	-0.5744455
CCDC151	-0.3948658
CCDC157	-0.6654685
CCDC159	-0.6097534
CCDC167	-0.7165637
CCDC18	-0.8678491
CCDC181	-0.4296541
CCDC19	-1.4373708
CCDC22	-0.2598414
CCDC23	-0.2986231
CCDC28B	-1.0061736
CCDC34	-1.0456054
CCDC39	-0.7230053
CCDC51	-0.355803
CCDC57	-0.4032764
CCDC6	-0.3414416
CCDC61	-0.5435286
CCDC67	-1.363466
CCDC68	-0.6735289
CCDC71	-0.3119416
CCDC74A	-1.713212
CCDC77	-0.5055084
CCDC8	-1.1515367
CCDC80	-1.9333076
CCDC88C	-0.1793118
CCDC89	-0.6225128
CCDC97	-0.3496647
CCHCR1	-1.059413
CCIN	-0.2511854
CCL24	-1.7360933

CCL6	-2.754838
CCL8	-1.6837754
CCL9	-0.6578264
CCNA2	-1.510808
CCNB1	-1.4172521
CCNB1IP1	-1.3885128
CCNB2	-1.2311406
CCND3	-0.964263
CCNDBP1	-0.5959668
CCNE1	-2.164557
CCNF	-0.9550395
CCNG1	-0.4415159
CCNI	-0.1933327
CCNO	-1.0895019
CCP110	-1.1492286
CCPG1	-0.7973542
CCR2	-1.5582216
CCR3	-3.74362
CCR5	-0.1719422
CCRN4L	-0.507968
CCS	-0.5504694
CCSAP	-0.4092808
CCT6A	-0.1909123
CCT7	-0.220892
CCZ1	-0.4737001
CD101	-0.8184285
CD109	-1.36007
CD151	-0.7765188
CD160	-0.9022725
CD163	-2.9596457
CD177	-0.5443554
CD180	-0.9568222
CD1D1	-0.3086872
CD1D2	-0.4803686
CD200R1	-1.7813354
CD200R4	-2.201659
CD207	-0.6364324
CD209A	-1.659312
CD209B	-0.7767608
CD209F	-2.3310633
CD226	-0.3062038
CD248	-2.6208367
CD24A	-1.7096124
CD2AP	-0.6494012
CD300C	-0.9164679

CD300E	-1.2943163
CD300LB	-3.6542838
CD300LD	-1.7888706
CD300LG	-0.7064266
CD300LH	-0.972584
CD36	-0.5161963
CD37	-0.2270284
CD47	-0.4434228
CD59A	-2.2647057
CD59B	-1.9471898
CD5L	-0.6580434
CD68	-0.8042135
CD7	-1.1877966
CD79B	-0.2341781
CD80	-0.3765621
CD82	-0.4107199
CD84	-0.7591467
CD8B1	-0.3145351
CD9	-0.4981809
CD93	-0.3880115
CD99L2	-1.7290835
CDADC1	-0.4115152
CDAN1	-0.5683649
CDC20	-1.1169362
CDC25A	-0.6082506
CDC25B	-1.640564
CDC25C	-1.0735569
CDC26	-0.5451369
CDC27	-0.4131146
CDC34	-0.3438311
CDC42BPA	-0.8694897
CDC42EP1	-1.025537
CDC42EP3	-0.8050203
CDC42EP4	-0.7952128
CDC42EP5	-1.2107348
CDC45	-0.3978434
CDC6	-1.1970496
CDC73	-0.2974725
CDCA2	-1.1137595
CDCA3	-0.9869852
CDCA4	-1.0781622
CDCA5	-0.7029481
CDCA7	-0.5744128
CDCA7L	-0.4356425
CDCA8	-1.0884681

CDH1	-0.3236475
CDH11	-0.8857856
CDH18	-1.221142
CDHR3	-0.5055134
CDK1	-1.1913991
CDK13	-0.2964749
CDK14	-0.4388681
CDK16	-0.9143515
CDK19	-0.3589726
CDK2	-0.8172603
CDK20	-0.7867024
CDK2AP1	-0.6071448
CDK4	-0.4745464
CDK5RAP3	-0.2739916
CDK6	-1.1422563
CDK8	-0.3461766
CDK9	-0.2066197
CDKN1C	-1.6223617
CDKN2A	-1.162818
CDKN2AIPNL	-0.3073278
CDKN2C	-1.7573695
CDKN2D	-0.4010539
CDKN3	-1.1123548
CDO1	-0.7019994
CDON	-0.485621
CDPF1	-0.3681002
CDR2	-1.3468642
CDR2L	-0.3872075
CDS1	-1.3848753
CDS2	-0.3114719
CDT1	-0.7555709
CDYL	-0.852067
CEACAM10	-0.6797862
CEACAM2	-1.0015907
CEACAM20	-0.3142397
CEBPA	-0.7146339
CEBPE	-2.3130727
CEBPZOS	-0.4978781
CECR2	-0.2508867
CELF4	-0.9270618
CENPA	-1.2836766
CENPC1	-0.5192833
CENPE	-0.431252
CENPF	-0.9884424
CENPH	-2.0518293

CENPI	-0.6127009
CENPK	-1.2139106
CENPL	-1.1590528
CENPM	-1.081234
CENPN	-0.980825
CENPO	-0.5928025
CENPP	-1.2519779
CENPQ	-0.3269348
CENPT	-0.9316015
CENPU	-1.2082846
CENPV	-0.7020273
CENPW	-1.1206985
CEP104	-0.5787239
CEP120	-0.5835853
CEP128	-1.0520184
CEP131	-1.0569181
CEP152	-0.641005
CEP164	-0.7231808
CEP19	-0.28442
CEP192	-0.4995685
CEP250	-0.9387875
CEP290	-0.9005377
CEP41	-0.204901
CEP44	-0.8699272
CEP55	-0.331192
CEP57L1	-0.5722604
CEP70	-0.8178625
CEP72	-1.6539319
CEP76	-1.1861134
CEP89	-0.807199
CEP97	-0.2692506
CERCAM	-1.4610171
CERS2	-0.616426
CES1D	-3.179136
CES2E	-1.1565769
CES2G	-1.6524382
CETN3	-0.6176858
CETN4	-0.2420158
CFD	-2.3709626
CFH	-0.7047529
CFP	-0.4297948
CGN	-0.9938698
CGRRF1	-0.8504906
CHAC2	-1.2412581
CHAF1A	-1.1688557

CHAF1B	-1.3928509
CHCHD10	-0.5200329
CHCHD6	-0.5166531
CHCHD7	-0.4144764
CHD1L	-0.5716672
CHD3OS	-0.4770412
CHDH	-0.7209888
CHEK2	-0.9151006
CHFR	-0.4281416
CHIL3	-1.8385458
CHIL4	-2.1375403
CHIT1	-2.5449748
CHKB	-0.3921456
CHMP1A	-0.3057718
CHMP1B	-0.3406177
CHMP2A	-0.3898625
CHMP4B	-1.1582961
CHMP6	-0.4190412
CHN1	-0.6251495
CHN2	-0.702915
CHP2	-2.458742
CHPF2	-0.3283477
CHPT1	-1.0099132
CHRAC1	-0.3870044
CHRDL1	-0.2996194
CHRNB1	-0.5951991
CHRNB2	-0.9975781
CHST1	-0.7946229
CHST12	-1.5658793
CHST13	-1.356669
CHST14	-0.2894554
CHST2	-1.0541131
CHSY3	-1.3007364
CHTF18	-1.3238921
CHTF8	-0.9365635
CHURC1	-0.6929359
CIAO1	-0.6081519
CIAPIN1	-0.6180506
CIB1	-0.6318779
CIB2	-0.3305311
CIDEA	-2.1356182
CIDEC	-3.8507576
CIITA	-2.7675788
CILP	-0.5299265
CINP	-0.4149499

CIRBP	-1.3802762
CISD1	-0.5355802
CISD2	-0.477901
CIT	-1.6001368
CITED4	-1.6130996
CKAP2	-1.1436505
CKAP2L	-0.677805
CKAP5	-0.4956532
CKB	-1.8483772
CKS1B	-1.006876
CKS1BRT	-1.0391316
CKS2	-0.6390457
CLCN2	-1.5781136
CLCN3	-0.8177433
CLCN7	-0.4110208
CLDN10	-0.2424731
CLDN13	-1.6591158
CLDN15	-1.4750693
CLDN20	-1.1704156
CLDN5	-1.2747731
CLEC10A	-0.7440944
CLEC11A	-1.2787216
CLEC14A	-2.832456
CLEC1A	-1.5593348
CLEC1B	-1.0482311
CLEC4A3	-0.8444238
CLEC4A4	-0.5598254
CLEC4B2	-1.2973397
CLEC4G	-0.8918204
CLEC7A	-1.2035675
CLEC9A	-1.027461
CLHC1	-0.7679915
CLIC3	-0.6911559
CLIC5	-1.7865834
CLIP3	-1.6443608
CLK3	-0.5333018
CLMN	-0.8899393
CLN6	-1.480813
CLN8	-1.0356293
CLP1	-0.6628141
CLPB	-0.4317203
CLPP	-0.3292718
CLPTM1	-0.4730482
CLPX	-0.7861977
CLSPN	-1.565419

CLSTN1	-0.3440568
CLTC	-0.6182399
CLYBL	-1.4707494
CMAS	-0.6486707
CMBL	-2.6438756
CMC1	-0.6462898
CMC2	-0.7753091
CML5	-2.0290751
CMPK1	-0.2030172
CMSS1	-0.5039988
CMTM3	-0.2710342
CMTM4	-1.888732
CMTM5	-0.5849388
CMTM7	-0.9002962
CMTM8	-1.7025783
CNGB1	-0.3944819
CNNM1	-0.7798858
CNNM2	-0.9480968
CNOT10	-0.5550013
CNOT3	-0.7538574
CNOT7	-0.5857682
CNP	-0.4084835
CNPPD1	-0.3325863
CNPY2	-0.3288937
CNPY4	-0.643611
CNR1	-1.067682
CNTF	-0.3415747
CNTFR	-1.769594
CNTLN	-1.0837321
CNTN2	-0.2499764
CNTROB	-0.6876559
COA3	-0.4128556
COA5	-0.3367386
COA6	-0.2571316
COASY	-0.2592487
COCH	-0.2786117
COG1	-0.2634082
COG2	-0.4457979
COG4	-0.2104178
COL14A1	-1.2360377
COL15A1	-1.7165723
COL16A1	-0.8005471
COL1A1	-1.3684726
COL1A2	-1.0962982
COL26A1	-2.1841052

COL27A1	-0.8780053
COL3A1	-1.9443545
COL5A1	-1.3350801
COL5A2	-1.566397
COL5A3	-1.1423731
COL6A1	-1.3137646
COL6A2	-1.4176712
COL6A3	-0.577693
COL8A2	-2.056383
COLEC11	-2.3203325
COMMD1	-0.817039
COMMD10	-0.7299197
COMMD2	-0.2787581
COMMD3	-0.6437511
COMMD5	-0.5629363
COMMD8	-0.4582958
COMMD9	-0.4842658
COMP	-0.3631825
COMT	-0.9453673
COPE	-0.2864356
COPG1	-0.6704271
COPG2	-0.8626323
COPS2	-0.318562
COPS3	-0.5607619
COPS5	-0.3997932
COPS6	-0.3689981
COPZ1	-0.5905871
COPZ2	-1.0360444
COQ10A	-0.4109974
COQ4	-0.4123035
COQ5	-0.5496063
COQ7	-0.7543578
COQ9	-0.7273259
CORO1C	-0.5216823
CORO2A	-0.2650337
CORO2B	-0.9165788
COX11	-0.501791
COX20	-0.2516832
COX6A1	-0.2533488
COX6A2	-1.0025568
COX6B2	-2.1628442
COX7B	-0.2724934
COX7C	-0.2571526
COX8A	-0.4271436
COX8B	-2.0112965

CPA3	-0.708236
CPA6	-0.8717513
CPE	-0.9517551
CPEB4	-0.9924798
CPED1	-1.0152156
CPM	-0.5519071
CPNE2	-1.2227674
CPOX	-1.0871115
CPPED1	-0.3937316
CPQ	-0.5367651
CPSF1	-0.4457202
CPSF3	-0.2513347
CPSF3L	-0.2614484
CPSF4	-0.6080713
CPT1A	-0.313087
CPT1B	-0.3514361
CPT1C	-1.464375
CPT2	-0.4713721
CPXM1	-0.2512693
CPXM2	-0.329494
CPZ	-1.6297183
CRABP2	-0.2414546
CRADD	-0.3025322
CRAMP1L	-0.2744279
CRAT	-1.1589143
CRB2	-1.5383253
CRCP	-0.2936106
CREB5	-0.2494063
CREG1	-0.9234858
CRELD1	-0.424818
CRHR1	-1.3674569
CRIP1	-0.3819909
CRIP2	-1.8219523
CRISP3	-1.4680657
CRISPLD1	-4.486553
CRKL	-0.2036185
CRLF1	-1.1401155
CRMP1	-1.1803651
CROCC	-0.3611932
CROT	-0.6317716
CRTAP	-0.303277
CRTC1	-0.5506854
CRTC3	-0.2327399
CRXOS	-0.5838075
CRYAB	-0.7429128

CRYBA4	-0.6840785
CRYBB1	-1.2868047
CRYBG3	-0.4080932
CRYL1	-1.2234197
CRYM	-0.7997844
CRYZ	-0.8806863
CRYZL1	-0.4174657
CS	-0.4380722
CSDC2	-3.1962707
CSE1L	-0.3597937
CSF1R	-0.7668219
CSGALNACT1	-1.497817
CSGALNACT2	-1.0769424
CSL	-0.6006446
CSNK1E	-0.2063351
CSNK1G2	-0.634757
CSNK2B	-0.3547936
CSPG4	-1.4604712
CSPG5	-1.5321212
CSPP1	-0.2345552
CSRP2	-0.4136114
CSRP3	-2.2619786
CST6	-1.8787818
CSTF1	-0.2078843
CSTF2	-0.2227116
CTBP1	-0.6265378
CTC1	-0.2742457
CTDP1	-0.2680206
CTDSP2	-0.749727
CTDSPL	-0.5887833
CTIF	-0.6299405
CTNNBIP1	-1.1815252
CTNND2	-1.7289164
CTNS	-0.8194604
CTR9	-0.5880041
CTSD	-0.6613331
CTSE	-1.1850309
CTSF	-1.8113666
CTSG	-0.5925131
CTSH	-1.0122728
CTSK	-0.9577673
CTTN	-0.299602
CTTNBP2	-1.0638576
CTXN1	-0.7942758
CUEDC1	-1.369987

CUEDC2	-0.8231473
CUL1	-0.7181978
CUL3	-0.7797976
CUL4A	-0.9168267
CUL5	-0.3486829
CUL7	-0.2890286
CUL9	-0.3516493
CUTA	-0.7884412
CUTC	-0.6207171
CUX1	-0.8045511
CX3CR1	-1.816994
CXADR	-1.9098547
CXX1B	-0.9329925
CXX1C	-1.0300298
CXXC1	-0.2295618
CXXC5	-1.223443
CYB5A	-0.6582508
CYB5D2	-0.611733
CYB5R1	-1.0211663
CYB5R3	-0.6740699
CYC1	-0.4192948
CYHR1	-0.9992447
CYP19A1	-0.6028795
CYP21A1	-2.052679
CYP26C1	-0.8297606
CYP27A1	-2.222223
CYP2B10	-0.5423884
CYP2D11	-0.8776717
CYP2E1	-2.0354881
CYP2J8	-1.1513767
CYP2J9	-2.1010954
CYP2S1	-1.5717075
CYP4B1	-0.9747391
CYP4B1-PS2	-0.9520278
CYP4F14	-1.556798
CYP4F18	-1.0179334
CYP51	-0.6665809
CYSLTR1	-0.9859538
CYTH3	-0.3763504
CYTL1	-1.663476
DAAM1	-0.7927296
DAAM2	-2.5626605
DAB2	-0.7814751
DAB2IP	-1.2019658
DACT1	-0.4437971

DACT2	-0.2743366
DAG1	-1.0565953
DAGLB	-1.080657
DAK	-0.8567836
DAND5	-0.6710007
DAP	-0.977293
DAP3	-0.4300799
DAPK1	-0.3981142
DAPK2	-1.1342735
DARS2	-0.8142614
DAZAP1	-0.3549228
DBF4	-1.093987
DBNDD2	-0.5964384
DBP	-1.0816875
DBR1	-0.25874
DCAF12	-0.8145781
DCAF15	-0.2047005
DCAF4	-0.7909622
DCAF6	-0.8657384
DCAF7	-0.610877
DCAF8	-0.5742364
DCAKD	-0.3478584
DCHS1	-1.1524062
DCK	-0.8520575
DCLK2	-0.8089628
DCLRE1A	-1.3888941
DCLRE1B	-0.5007002
DCN	-0.5864978
DCPS	-0.3808036
DCSTAMP	-2.592072
DCT	-9.8521
DCTN2	-0.8250713
DCTN6	-0.6600266
DCTPP1	-0.3266335
DCUN1D1	-0.5763235
DCXR	-1.3719258
DDAH2	-1.2389565
DDC	-0.9535852
DDHD2	-0.3497734
DDI2	-0.4048948
DDIT4L	-2.9941072
DDOST	-0.5444293
DDR2	-0.685288
DDRGK1	-0.3381324
DDX1	-0.4937

DDX11	-0.3757372
DDX20	-0.5442414
DDX23	-0.649003
DDX26B	-0.4600906
DDX28	-0.555275
DDX39	-0.3668623
DDX4	-0.8840251
DDX41	-0.2593398
DDX47	-0.3577867
DDX50	-0.2272143
DDX59	-0.4186986
DEAF1	-0.4855766
DEB1	-0.4008522
DECR1	-0.6418867
DECR2	-0.3627009
DEDD2	-0.5012069
DEF8	-0.9382377
DEFB11	-0.3875356
DEFB45	-0.3142538
DEFB9	-0.6292248
DEGS2	-2.318759
DEK	-1.0644183
DENND2A	-1.1799183
DENND2C	-2.201532
DENND4C	-0.4462502
DENND6A	-0.3678007
DENND6B	-0.8190415
DEPDC1A	-1.2199216
DEPDC1B	-0.8011251
DEPTOR	-0.62588
DERA	-1.1937304
DESI1	-0.4234138
DFNA5	-1.3015347
DFNB59	-1.7341108
DGCR14	-0.3433509
DGCR6	-0.679523
DGCR8	-0.4830542
DGKG	-1.0353482
DGKI	-0.6150887
DGKZ	-0.5619545
DHCR24	-1.3104334
DHCR7	-0.6650207
DHFR	-0.8510816
DHH	-0.8072772
DHRS1	-0.834425

DHRS11	-1.8083992
DHRS13	-1.0755725
DHRS3	-0.5015011
DHRS7	-0.5630059
DHRSX	-0.3127165
DHTKD1	-1.3530672
DHX15	-0.6018248
DHX16	-0.2568226
DHX37	-0.7211211
DIABLO	-0.4582415
DIAP3	-0.5164981
DIDO1	-0.2924891
DIO1	-0.2700524
DIO2	-1.1736019
DIP2A	-0.5552635
DIP2B	-0.3010554
DIRAS2	-0.9370532
DIS3	-0.3216214
DIS3L	-0.3728194
DIS3L2	-0.4210987
DKK3	-2.7082806
DKKL1	-1.5262165
DLD	-0.3299618
DLG2	-2.0965838
DLG4	-1.14657
DLG5	-0.8234386
DLGAP5	-1.7211871
DLK2	-0.3136106
DLST	-0.5718451
DMAP1	-0.3762388
DMPK	-1.9086847
DMRTB1	-1.3069766
DMTN	-0.7335944
DMWD	-0.3473868
DNA2	-1.2065015
DNAAF2	-0.9260001
DNAAF3	-0.8268313
DNAJA4	-0.4607368
DNAJB1	-0.3595004
DNAJB12	-0.3483148
DNAJB13	-1.0031064
DNAJB14	-0.1979837
DNAJB2	-1.2733984
DNAJB3	-1.1078043
DNAJB4	-0.9672051

DNAJB7	-1.2670052
DNAJC10	-0.2341175
DNAJC11	-0.4763832
DNAJC16	-0.5108209
DNAJC17	-0.9535651
DNAJC24	-0.3792453
DNAJC25	-0.6088326
DNAJC30	-0.3060308
DNAJC4	-0.6708002
DNAJC6	-1.6734197
DNAJC8	-0.6033511
DNAJC9	-0.5284433
DNAL4	-0.3768687
DNASE1L1	-1.0673718
DND1	-0.4805691
DNLZ	-0.4605928
DNM1	-1.379091
DNM2	-0.3625741
DNMT1	-1.1227479
DNMT3B	-0.3846993
DNPEP	-0.2493439
DNPH1	-0.7745285
DNTT	-2.7313058
DNTTIP1	-0.3723722
DOCK6	-0.8272395
DOK1	-0.3373742
DOK2	-0.315424
DOK3	-0.3573813
DOK4	-1.818517
DOK7	-1.5910316
DOLK	-0.2891812
DOLPP1	-0.4285874
DONSON	-0.2933784
DOPEY2	-0.3331494
DOS	-0.6424007
DOT1L	-0.2701469
DPAGT1	-0.4518795
DPCD	-0.5391555
DPEP1	-0.2271116
DPEP2	-1.048707
DPF2	-0.2672768
DPF3	-1.2083535
DPH1	-0.6870356
DPH3	-0.3658381
DPM2	-0.3419905

DPM3	-0.3935142
DPP3	-0.3755083
DPP8	-0.3325682
DPT	-1.4268777
DPY19L1	-1.1648865
DPY30	-0.7820196
DPYD	-1.1768272
DPYSL4	-1.2503603
DR1	-0.3037515
DRC1	-1.6332302
DRG1	-0.2816706
DROSHA	-0.6548944
DSCC1	-1.3298233
DSCR3	-0.4247112
DSE	-0.6026535
DSN1	-0.4224997
DSTYK	-1.5691175
DTD2	-0.9622543
DTL	-0.934175
DTWD1	-0.4600654
DTX3	-1.0920181
DTYMK	-0.5426498
DUS1L	-0.2373514
DUSP14	-0.8600826
DUSP19	-0.708508
DUSP22	-0.5048621
DUSP26	-0.7720399
DUSP28	-0.7374716
DUSP8	-1.420207
DUT	-0.9926696
DXO	-0.2926006
DYM	-0.9289742
DYNC1I1	-0.302865
DYNC1LI1	-0.3588281
DYNC1LI2	-0.2193031
DYNC2LI1	-1.2153313
DYNLL2	-0.8691654
DYNLRB1	-0.1779418
DYNLT1C	-0.395782
DYNLT1F	-0.7282281
DYRK1A	-0.3795123
DYRK1B	-0.9257071
DYRK3	-0.6602931
DYSF	-1.6624069
DZANK1	-1.9794719

E2F1	-1.6121473
E2F2	-1.2120895
E2F3	-0.5107131
E2F4	-1.615663
E2F6	-0.4143143
E2F7	-0.9750457
E2F8	-1.266274
EAR1	-2.2626438
EAR10	-1.9798622
EAR12	-2.3303223
EAR2	-2.156355
EAR6	-2.6409607
EAR7	-2.8691459
EBPL	-0.2904325
ECD	-0.5371203
ECH1	-0.8254762
ECHS1	-0.9159355
ECI1	-0.5166431
ECI3	-0.7114649
ECT2	-0.7110572
EDF1	-0.2357249
EDN3	-0.6339738
EEF1D	-0.364852
EEF2	-0.3500862
EEF2K	-1.374625
EEFSEC	-0.8162746
EFCAB2	-0.217932
EFCAB4A	-1.8404934
EFCAB5	-0.9730654
EFEMP1	-1.0081818
EFEMP2	-0.628274
EFHC1	-0.6383054
EFHD1	-2.0099645
EFNA1	-1.6802711
EFNB1	-0.5705919
EFNB2	-0.7051466
EFTUD2	-0.5477729
EGFL7	-0.5912414
EHBP1L1	-1.0073986
EHD2	-0.6630688
EHD4	-0.2368102
EHHADH	-0.9774032
EI24	-0.87447
EID1	-0.4383278
EID2B	-0.6908033

EIF1-PS	-0.2377715
EIF2A	-0.5564222
EIF2AK1	-0.8920536
EIF2B1	-0.7605863
EIF2B4	-0.3981881
EIF2B5	-0.1968036
EIF2D	-0.7848959
EIF2S3X	-0.4082294
EIF3A	-0.2442713
EIF3B	-0.2453585
EIF3F	-0.2534146
EIF3I	-0.398766
EIF3J1	-0.3336844
EIF3K	-0.2315526
EIF3M	-0.2857904
EIF4B	-0.2608018
EIF4E2	-0.2520261
EIF4E3	-0.2335897
EIF4EBP1	-0.5439553
EIF4EBP2	-0.372838
EIF4EBP3	-0.1898403
EIF4ENIF1	-0.3910675
EIF4H	-0.4720292
EIF5	-0.2811184
EIF5A	-0.5064797
EIF5B	-0.3751366
ELANE	-1.1534777
ELF2	-0.3477125
ELK3	-0.3081594
ELL	-0.3000436
ELL2	-0.5009537
ELMSAN1	-0.2902241
ELN	-2.1672735
ELOF1	-0.3191633
ELP3	-0.357543
ELP4	-0.3280556
ELP6	-0.3425927
EMC1	-0.4424467
EMC10	-0.6001012
EMC3	-0.6113601
EMC8	-0.3199444
EMC9	-0.8584642
EMD	-0.3916936
EME1	-0.8458624
EMG1	-0.2576342

EMILIN2	-0.9560118
EML3	-0.5572443
EMP1	-0.7710376
EMP2	-0.437089
EMP3	-0.688077
ENDOD1	-1.0331192
ENDOG	-0.5309906
ENG	-0.773705
ENGASE	-0.7163544
ENHO	-1.8480616
ENKD1	-0.8855038
ENO1	-0.2490253
ENO1B	-0.2978997
ENOX1	-0.7778223
ENOX2	-0.4742575
ENPEP	-1.3902931
ENPP3	-1.4411733
ENPP5	-1.20435
ENSA	-0.5921464
ENTPD1	-0.9427245
ENTPD6	-0.529902
ENY2	-0.6037536
EOGT	-0.3189819
EP300	-0.8042154
EPB4.1	-0.9564056
EPB4.1L3	-0.618845
EPB4.2	-1.3418417
EPDR1	-1.0820832
EPG5	-0.3945684
EPHA1	-1.3619885
EPHA2	-0.7233875
EPHA3	-1.9412601
EPHA8	-0.9670668
EPHB1	-2.356316
EPHB4	-0.3435106
EPHB6	-0.8125763
EPHX2	-0.4772122
EPHX3	-0.800159
EPN1	-0.6360326
EPOR	-1.4383144
EPS15	-0.20678
EPT1	-0.3247294
EPX	-2.8919573
ERAL1	-0.4561343
ERBB2	-1.2134109

ERCC6L	-1.0988102
ERDR1	-0.5430288
ERF	-0.8572664
ERG	-1.8272161
ERGIC2	-0.394825
ERGIC3	-0.6988292
ERI1	-0.9245009
ERI2	-0.5981262
ERI3	-1.0808775
ERICH2	-0.937223
ERICH5	-0.2820015
ERLIN1	-0.2755694
ERLIN2	-0.7514424
ERMAP	-1.5562615
ERMP1	-0.6811471
ERP29	-0.4753614
ESCO2	-0.9057198
ESD	-0.4663577
ESPL1	-1.2665133
ESR1	-0.9417543
ESYT1	-0.4421306
ETAA1	-0.7894902
ETD	-1.2850246
ETFA	-0.5972576
ETFB	-0.5024738
ETFDH	-0.2627716
ETHE1	-1.5794497
ETL4	-0.8532522
ETV1	-2.4570415
ETV5	-0.8126659
EVC	-1.2883751
EVC2	-0.277055
EVI2A	-0.1891842
EVI5	-0.8041
EVI5L	-0.4588676
EVPL	-1.4622033
EWSR1	-0.4930897
EXO1	-0.8120885
EXOC3L	-1.5513864
EXOC3L2	-0.7654078
EXOC4	-0.3921318
EXOC6	-0.797616
EXOC7	-0.7137141
EXOSC3	-0.2155652
EXOSC4	-0.4404388

EXOSC5	-0.278935
EXOSC7	-0.6748123
EXOSC8	-0.6815453
EXOSC9	-0.2440257
EXT1	-0.4471178
EXTL2	-0.8430924
EZH2	-0.6929421
F2RL2	-1.6260588
F5	-1.0536251
F8	-1.2412279
FABP4	-1.1615262
FADD	-0.4136558
FADS1	-1.2242398
FADS2	-1.9403627
FADS3	-1.4428253
FAF1	-0.614624
FAHD1	-1.5350251
FAHD2A	-0.6154699
FAM101B	-0.6441226
FAM103A1	-0.3809204
FAM109B	-1.3408198
FAM110A	-0.4187083
FAM110B	-0.6682057
FAM111A	-0.2182064
FAM114A2	-0.2930956
FAM115A	-0.6723721
FAM117A	-1.2021332
FAM120A	-0.6622286
FAM122A	-0.7973132
FAM122B	-0.637672
FAM124A	-1.6394145
FAM126A	-1.1306581
FAM129C	-1.505528
FAM131A	-0.2171931
FAM132A	-1.9053059
FAM132B	-2.8446069
FAM134A	-1.1840963
FAM134C	-0.6577568
FAM135A	-0.4534888
FAM13A	-2.9052956
FAM151A	-2.2650392
FAM151B	-0.9122305
FAM161A	-0.5237122
FAM167B	-1.0040817
FAM168A	-0.4381857

FAM170B	-0.7872717
FAM171A1	-2.6953623
FAM172A	-0.2806001
FAM173A	-0.4585905
FAM175B	-0.368218
FAM178A	-0.5407105
FAM181B	-0.3140459
FAM192A	-0.6684637
FAM195A	-1.0332274
FAM198B	-1.5325916
FAM19A1	-0.7339594
FAM20A	-2.283456
FAM20B	-0.2554946
FAM210B	-1.2749152
FAM212A	-0.9246559
FAM212B	-1.4311388
FAM213A	-0.2988954
FAM213B	-0.3815961
FAM214B	-1.1968813
FAM216A	-0.4825144
FAM219A	-0.4143987
FAM220A	-0.5551376
FAM227A	-0.466938
FAM32A	-0.2932186
FAM3A	-0.6775367
FAM43A	-0.5281496
FAM45A	-0.2355514
FAM46C	-0.3466249
FAM50A	-0.43999
FAM53B	-0.8351994
FAM57B	-1.0890739
FAM63A	-0.4715462
FAM63B	-0.7461357
FAM64A	-1.1397591
FAM65A	-0.6500111
FAM69A	-1.0478868
FAM69B	-1.5950568
FAM73B	-0.9190536
FAM76A	-0.370584
FAM78A	-0.8012395
FAM83F	-0.4398952
FAM83G	-0.3070169
FAM84A	-1.0645351
FAM89B	-0.7692456
FAM92A	-0.5627332

FAM96A	-0.6879654
FAM98C	-0.2902069
FANCB	-0.4073834
FANCC	-0.2728729
FANCD2	-1.4077837
FANCE	-0.2328863
FANCG	-0.5537014
FANCI	-0.5412135
FANCL	-0.7561879
FANCM	-0.7370114
FARS2	-0.8181996
FASTK	-0.4332857
FAT1	-0.4656031
FAT4	-2.6050715
FBLN1	-1.8348708
FBLN2	-0.2670507
FBLN5	-1.260015
FBLN7	-0.803256
FBN1	-0.3831785
FBN2	-1.1843348
FBP1	-2.1484716
FBRSL1	-0.2972665
FBXL14	-0.4480615
FBXL15	-0.7180877
FBXL16	-1.1914709
FBXL17	-0.3075161
FBXL4	-0.3590479
FBXL6	-0.5687847
FBXL8	-0.503396
FBXO10	-1.5799775
FBXO18	-0.1935244
FBXO25	-0.4567318
FBXO28	-0.3382018
FBXO3	-1.2552795
FBXO30	-0.5774226
FBXO34	-0.8921647
FBXO36	-1.1025596
FBXO42	-0.2708321
FBXO44	-0.450114
FBXO45	-0.3597653
FBXO5	-1.2049041
FBXO7	-0.5432077
FBXO8	-0.72576
FBXO9	-0.7000704
FBXW8	-0.221364

FBXW9	-0.3954666
FCER1A	-1.2322683
FCER2A	-0.9978643
FCGR1	-1.941721
FCGR3	-0.4979868
FCGRT	-2.1500254
FCHO1	-1.0514503
FCHO2	-0.3360653
FCHSD1	-0.7151971
FCNA	-0.8629065
FCNAOS	-0.4402146
FCNB	-1.7253747
FDFT1	-0.4158611
FDPS	-1.1486173
FDXR	-0.2746534
FECH	-0.8471813
FEN1	-0.7637711
FERMT3	-0.7363386
FERT2	-0.6421413
FES	-1.0041695
FEZ2	-0.2779059
FFAR4	-1.4397962
FGD1	-1.6720827
FGD2	-0.6024895
FGF1	-1.1460209
FGF18	-1.9193006
FGFR1	-0.6174417
FGFR1OP2	-0.227376
FGFR3	-1.0169399
FGFR4	-3.4915926
FGFRL1	-0.8252392
FGL2	-0.6068478
FHDC1	-1.0648713
FHL5	-0.7286687
FIBIN	-1.3449869
FIBP	-0.6143942
FIG4	-0.2707658
FIGF	-1.36548
FIGNL1	-1.1162295
FIP1L1	-0.3928385
FIS1	-0.6986804
FIZ1	-0.6206846
FKBP10	-0.3841167
FKBP14	-0.5880668
FKBP1B	-1.7763329

FKBP7	-1.1559973
FKBP8	-0.9105134
FKBP9	-0.9000638
FLAD1	-0.2282095
FLOT2	-0.4099364
FLRT1	-1.0961373
FLRT2	-1.4875994
FLT3L	-0.416956
FLYWCH1	-1.1517878
FLYWCH2	-0.9466226
FMN1	-0.2649617
FMO1	-1.1629739
FMO2	-4.694352
FMO5	-1.0331593
FMOD	-0.7229283
FN1	-0.5009151
FN3K	-1.1952472
FN3KRP	-0.5952511
FNDC1	-1.5399914
FNDC4	-0.4298129
FNDC5	-1.3861387
FNIP2	-1.1151261
FNTA	-0.2737131
FNTB	-1.6608264
FOLR1	-1.6513147
FOLR2	-2.6903522
FOPNL	-0.6687484
FOXD2	-0.8580065
FOXD2OS	-0.9547379
FOXH1	-0.6715906
FOXK2	-0.6224523
FOXM1	-1.1067119
FOXN3	-0.4389162
FOXO3	-1.6111143
FOXO4	-0.6639066
FOXRED2	-0.3893399
FPGS	-0.8527422
FRAS1	-3.103071
FRAT1	-0.633728
FRAT2	-0.612082
FREM1	-1.4319401
FREM3	-1.0509212
FRG1	-0.2834997
FRK	-0.8659895
FRMD3	-0.6740065

FRMD4A	-0.3626142
FRMD5	-1.1411211
FRRS1	-0.9597454
FRY	-1.1830983
FRYL	-0.2763252
FSCN3	-0.5718622
FSIP1	-0.2438345
FSTL3	-0.4620814
FTO	-0.5816093
FTSJ1	-0.6856618
FTSJ2	-0.5399439
FTSJ3	-0.3396897
FUCA1	-0.6597858
FUCA2	-0.6769042
FUNDC2	-0.4918504
FUOM	-0.6063254
FUT7	-1.3328962
FXN	-1.3885012
FXYD1	-1.0340366
FXYD2	-0.4040251
FXYD3	-0.4603145
FXYD5	-0.5873365
FXYD6	-1.902812
FYCO1	-0.4826188
FZD2	-2.7940683
FZR1	-1.2786317
G2E3	-0.274509
G6PC3	-0.330029
G6PDX	-0.4992843
GAB1	-1.4658966
GABARAPL1	-0.473156
GABPB2	-0.1974754
GABRA3	-2.854961
GADD45A	-0.9129329
GADD45GIP1	-0.5805244
GAL3ST2	-0.6512528
GALC	-1.6733899
GALE	-0.3789587
GALNS	-0.3701563
GALNT1	-0.4439969
GALNT10	-0.7731304
GALNT15	-1.2794983
GALNT16	-1.2037656
GALNT2	-0.6062493
GAMT	-0.9602947

GAPT	-1.2684648
GAPVD1	-0.6097603
GAREM	-1.9722846
GARNL3	-0.8553927
GAS2	-1.2987187
GAS2L1	-0.4396868
GAS2L3	-0.8980303
GAS6	-1.176549
GATA1	-1.1608901
GATA5	-0.3492434
GATA6	-1.914212
GATAD1	-0.3990402
GATAD2A	-0.401856
GATAD2B	-0.622397
GATC	-0.9622057
GATM	-1.0023108
GBA2	-0.3873844
GBAS	-0.4334388
GBGT1	-0.5575261
GBP11	-0.7018828
GBP2B	-2.1630816
GBP8	-1.0811
GBX2	-0.3106501
GCC2	-0.3080668
GCDH	-0.6686077
GCFC2	-0.4384439
GCH1	-0.4304929
GCHFR	-1.3352139
GCLC	-0.9193454
GCLM	-1.3248081
GCM2	-0.7249188
GCNT1	-0.1739132
GDA	-0.646215
GDE1	-0.5605469
GDF1	-0.3838966
GDF10	-2.8517447
GDF11	-0.7671325
GDF3	-1.6236422
GDF6	-3.576693
GDNF	-1.1032445
GDPD1	-1.6352434
GDPD2	-0.4401488
GDPD3	-0.7269945
GDPD4	-6.572735
GDPD5	-0.6507583

GEMIN2	-0.8203638
GEN1	-0.7370222
GET4	-0.5261831
GFAP	-1.5734162
GFI1B	-1.6920142
GFRA2	-0.5497394
GGA1	-0.8699932
GGACT	-0.5765204
GGCX	-0.2426703
GGN	-0.42293
GGNBP2	-0.4907789
GHDC	-0.4385934
GHR	-0.6582534
GID4	-0.5319481
GID8	-0.6302772
GIGYF1	-0.2290411
GIN1	-0.9138861
GINS1	-1.0490918
GINS2	-1.0932226
GINS3	-0.3534889
GINS4	-1.1417379
GIPC1	-0.7108226
GIPC2	-1.5039518
GJA3	-0.3962355
GJB3	-2.1334646
GLB1	-0.8409982
GLB1L	-0.8198969
GLB1L2	-1.7752769
GLE1	-0.4837589
GLIPR1	-0.3970242
GLIS2	-1.852768
GLIS3	-0.7529626
GLMN	-0.3236551
GLO1	-1.0473976
GLOD4	-0.2472844
GLOD5	-1.1138344
GLRX	-0.5120978
GLRX5	-1.6270514
GLT25D1	-0.480741
GLT8D1	-0.4110665
GLTP	-0.5697107
GLTPD1	-0.8565362
GLTSCR1L	-0.3790789
GLUL	-0.9126315
GLYR1	-0.301961

GM10046	-0.426455
GM10101	-0.1810598
GM10134	-1.9797626
GM10142	-0.3207023
GM10257	-1.0127587
GM10272	-0.2991588
GM10318	-0.6541066
GM10336	-0.2025602
GM10371	-1.1058061
GM10419	-1.1995704
GM10420	-0.3606334
GM10509	-0.6667333
GM10614	-0.6091576
GM10638	-0.6162374
GM10639	-4.3660603
GM10651	-0.4311473
GM10687	-1.5553954
GM10731	-0.5598841
GM10778	-0.2120459
GM10790	-2.1853144
GM10824	-2.2230034
GM10825	-0.744406
GM10827	-0.5254204
GM11223	-0.874629
GM11545	-1.9078376
GM11559	-0.5536623
GM11627	-1.5295143
GM11710	-1.1280243
GM11783	-0.5466549
GM12052	-1.7411911
GM12060	-0.3572121
GM12247	-0.8294497
GM12271	-0.8920679
GM12618	-0.375598
GM12620	-1.2418284
GM12657	-0.6799407
GM12666	-0.2671695
GM13051	-1.3277597
GM13111	-0.5932341
GM13157	-0.2057314
GM13247	-0.8416817
GM13251	-0.9433391
GM13272	-0.5731907
GM13275	-0.5494075
GM13372	-1.1126804

GM13710	-1.933459
GM13770	-1.1601038
GM14025	-0.3164251
GM14325	-0.349659
GM14432	-0.7217932
GM14484	-0.8233974
GM14625	-0.5399525
GM15023	-0.2824774
GM15032	-0.3866205
GM15411	-1.679745
GM156	-0.8693919
GM15612	-0.8588681
GM15645	-0.6278577
GM15800	-0.5531397
GM16039	-0.5857728
GM1631	-1.656796
GM16381	-0.356142
GM16515	-0.8340082
GM166	-2.22007
GM1673	-1.0449739
GM1715	-0.3817434
GM17501	-0.9483998
GM17748	-0.5320654
GM17758	-0.3241539
GM17764	-0.4598827
GM1943	-0.2157505
GM19924	-0.8481815
GM20056	-0.498466
GM20746	-0.9493842
GM20878	-1.1615338
GM2099	-0.8451843
GM2115	-1.6864486
GM2138	-0.3636854
GM21559	-0.5293846
GM2436	-0.2934213
GM2582	-0.987812
GM2696	-0.2760472
GM2A	-0.7277198
GM3099	-0.7478294
GM3219	-0.3645954
GM3258	-0.3425779
GM3325	-1.4939849
GM3364	-2.3207781
GM3448	-0.8100295
GM3776	-4.1709604

GM3853	-2.4542038
GM3871	-0.7070298
GM4120	-0.3761215
GM4285	-0.9278624
GM4349	-0.5270391
GM4402	-0.6596699
GM4470	-0.3134813
GM4566	-1.2334862
GM4673	-1.3764875
GM4814	-1.1086278
GM4906	-0.4677937
GM4943	-0.6977301
GM4980	-1.931963
GM5068	-0.9204521
GM5069	-0.4535971
GM5086	-1.577812
GM5093	-0.2814174
GM5099	-0.8131902
GM5132	-1.1882172
GM5141	-0.6080296
GM5177	-2.0433176
GM5296	-0.4327147
GM5396	-0.2773447
GM5463	-0.5692041
GM5526	-0.3036537
GM5589	-0.5132999
GM5593	-1.7385125
GM5617	-0.5831137
GM5643	-0.4311132
GM5665	-0.8587129
GM568	-1.4160435
GM5767	-1.2104616
GM5779	-0.6458559
GM5785	-0.2424889
GM5797	-0.3291824
GM5801	-0.6548786
GM5820	-3.0303042
GM5848	-0.3369846
GM5879	-0.2550154
GM6115	-0.269412
GM6116	-0.6387029
GM6117	-0.7034884
GM6195	-0.6979141
GM6444	-0.2824569
GM6548	-0.2790752

GM6557	-0.3977594
GM6625	-0.700068
GM6899	-1.070904
GM6921	-0.2793889
GM6934	-1.0194879
GM6936	-1.1298842
GM6981	-0.3731232
GM6987	-0.4721112
GM7102	-0.316179
GM7555	-0.3581505
GM7628	-1.0789895
GM7664	-0.518889
GM7931	-0.4878211
GM7936	-0.3059926
GM7968	-0.3110635
GM8013	-0.6243742
GM8094	-0.3370781
GM8212	-0.3381877
GM8220	-0.4242494
GM826	-1.2712657
GM8587	-0.403826
GM867	-1.2491846
GM8675	-0.5179343
GM8709	-0.4561582
GM8738	-0.1865926
GM8801	-0.3885531
GM9049	-0.4276114
GM9054	-1.103198
GM9079	-0.4486113
GM9234	-0.2376919
GM9465	-0.5547345
GM9525	-0.6437192
GM9733	-1.2209864
GM9744	-1.3380628
GM9855	-0.4863045
GM9895	-1.0326395
GM9920	-0.3167458
GM996	-0.9619978
GM9963	-0.2243254
GM9992	-1.8472786
GMEB1	-0.3417697
GMEB2	-0.3378477
GML	-0.8141296
GMNN	-1.1633244
GMPPA	-0.336803

GMPR	-1.7344508
GMPS	-0.5270877
GNA11	-0.7799406
GNA12	-0.8777342
GNA14	-0.9336879
GNAI2	-0.4998708
GNAS	-0.3885489
GNAZ	-0.5101347
GNE	-0.2817874
GNG10	-0.6939459
GNG11	-1.0340538
GNG3	-0.3503737
GNG8	-1.8946202
GNPDA1	-0.7162786
GNPTG	-0.236958
GOLIM4	-0.5893874
GOLM1	-1.1519494
GOLPH3L	-0.2705164
GOLT1B	-0.2951436
GORASP2	-0.484365
GOSR1	-0.2569976
GOT2	-0.5493608
GP1BA	-0.5854788
GP1BB	-0.9226053
GP5	-0.995079
GP6	-0.2730274
GP9	-1.1873903
GPAA1	-0.6061478
GPAM	-0.7803965
GPC1	-0.8532014
GPC2	-1.2736533
GPC3	-0.658777
GPCPD1	-0.499989
GPD1	-4.046987
GPHB5	-0.4200678
GPIHBP1	-0.40343
GPM6A	-1.8273683
GPN3	-0.4912911
GPNMB	-1.2000191
GPR107	-0.2106094
GPR124	-0.2432914
GPR125	-1.3254836
GPR133	-2.5381355
GPR153	-2.004667
GPR155	-1.1717434

GPR156	-0.6697853
GPR157	-0.7004638
GPR160	-0.892704
GPR162	-0.5899038
GPR182	-0.6118608
GPR19	-1.1417758
GPR34	-0.6272616
GPR56	-0.7575531
GPR88	-2.1910686
GPR89	-0.6376717
GPR97	-0.6926827
GPRC5C	-1.4997897
GPS2	-0.9705286
GPSM2	-1.411809
GPSM3	-0.7139258
GPT	-0.4285612
GPT2	-0.4033876
GPX1	-1.0329776
GPX4	-1.1942978
GPX8	-0.6643457
GRAP2	-0.4275017
GRASP	-0.3977323
GRB10	-0.7367728
GREB1	-0.5476284
GREM2	-0.7925832
GRHPR	-0.4747858
GRIA3	-1.7416615
GRIK5	-1.1713195
GRIN2C	-1.1521435
GRINA	-0.8705201
GRK6	-0.2046385
GRN	-0.7270641
GRPEL1	-0.2904334
GRPEL2	-0.1924644
GRRP1	-0.3006225
GRSF1	-0.3685455
GRTP1	-1.3667293
GSE1	-1.4181719
GSG1	-1.7501228
GSG2	-0.3908653
GSK3A	-0.381134
GSK3B	-0.9857988
GSKIP	-0.4731894
GSN	-0.8313341
GSR	-0.3385172

GSS	-1.1830502
GSTA2	-2.4116778
GSTA3	-1.2155685
GSTA4	-1.004369
GSTK1	-0.7917604
GSTM1	-1.0520797
GSTM2	-0.5170417
GSTM3	-1.1124253
GSTM4	-0.9086869
GSTM5	-1.8274288
GSTO1	-0.47189
GSTP1	-1.0227394
GSTP2	-0.5818868
GSTT1	-0.9697318
GSTT2	-0.2989512
GSTZ1	-0.69625
GSX1	-1.4940915
GTF2A2	-0.5339346
GTF2E2	-0.5790505
GTF2H1	-0.3166823
GTF2H2	-0.3168082
GTF2H3	-0.3778052
GTF2H4	-0.3166871
GTF2IRD1	-1.2635212
GTF3A	-0.621295
GTF3C5	-0.5174379
GTL3	-0.6656213
GTPBP1	-1.0548773
GTPBP2	-0.6028771
GTPBP8	-0.2646766
GUCA1A	-0.6625121
GUCA1B	-0.4556251
GUCD1	-0.5019627
GUCY1A2	-0.8930948
GUCY1A3	-2.011519
GUCY1B3	-0.8919005
GUF1	-0.4003201
GUK1	-0.69069
GUSB	-0.2679315
GXYLT2	-0.2693977
GYPA	-1.184907
GYPC	-0.6306624
GYS1	-0.4791594
H1F0	-2.3579326
H1FX	-1.6032033

H2AFV	-0.5841432
H2AFX	-0.7542744
H2AFY	-0.5319467
H2AFZ	-0.2348537
H2-D1	-0.3921695
H2-DMA	-0.1933947
H2-M9	-1.1118684
H2-OA	-0.2971439
H2-T10	-0.3038878
H3F3A	-0.7626872
HABP4	-0.3270154
HACE1	-0.6560636
HACL1	-0.9417381
HADH	-1.2190785
HADHA	-0.5332599
HADHB	-0.4102736
HAGH	-1.4222412
HAGHL	-1.154851
HAL	-1.0006793
HARS	-0.9717121
HAT1	-0.7257772
HAUS1	-0.7865615
HAUS3	-0.1894655
HAUS4	-0.5643988
HAUS5	-0.8454247
HAUS6	-0.6713405
HAUS8	-0.9705935
HAVCR2	-1.1021883
HBB-Y	-2.6844153
HBP1	-0.3181691
HBQ1A	-1.3402472
HBQ1B	-1.3961687
HCFC1	-0.8121667
HCFC1R1	-1.0624614
HDAC10	-0.1908207
HDAC11	-1.1168964
HDAC3	-0.7404552
HDAC4	-0.285624
HDAC5	-0.7682953
HDAC7	-0.549408
HDDC3	-0.8845472
HDGF	-0.86339
HDGFL1	-1.0573697
HDGFRP2	-0.3258987
HEATR2	-0.4135389

HEATR5A	-1.0515685
HEATR5B	-0.2945123
HEBP1	-0.8427205
HEBP2	-3.3298488
HECTD1	-0.5512271
HECTD3	-0.5791612
HELB	-0.5174217
HELLS	-0.435298
HELQ	-0.7268977
HELZ	-0.347846
HEMGN	-0.2232328
HEMK1	-0.2858987
HEPH	-0.6668856
HERC1	-0.2576962
HERPUD2	-0.3654885
HES1	-0.4683602
HES6	-0.3378282
HEXA	-0.9866643
HEXB	-1.1014104
HEXDC	-0.8731353
HEXIM1	-0.5290899
HEY1	-1.40219
HEY2	-1.3242266
HEYL	-2.2505991
HFE	-1.6563029
HGF	-0.7105775
HHEX	-0.8513064
HIBCH	-0.8124328
HID1	-0.4880738
HIF3A	-0.2224104
HIGD1A	-0.2491231
HIGD2A	-0.7842012
HINT2	-0.850131
HIP1	-1.2852936
HIPK1	-0.6972556
HIPK2	-0.9015122
HIRA	-0.554935
HIRIP3	-0.6151547
HIST1H1A	-0.8768287
HIST1H1B	-0.3041592
HIST1H1C	-1.4628935
HIST1H1E	-1.4320984
HIST1H2AA	-0.3545847
HIST1H2AB	-0.422328
HIST1H2BC	-0.6164966

HIST1H2BP	-0.2133727
HIST1H3A	-0.5722578
HIST1H3C	-0.5094671
HIST1H3D	-0.4547057
HIST1H3G	-0.4525971
HIST1H3I	-0.4257546
HIST1H4H	-0.9516439
HIST1H4I	-0.2619753
HIST2H2BE	-0.5943806
HIST2H3B	-0.5595036
HJURP	-0.7037482
HK3	-1.5173955
HLCS	-1.2036159
HLF	-3.188155
HLX	-0.3968043
HMBOX1	-0.3267424
HMBS	-1.454534
HMCN1	-1.3154058
HMG20B	-0.5767055
HMGA2-PS1	-0.8342514
HMGB1	-0.5234442
HMGB1-RS17	-0.7022696
HMGB2	-0.4561896
HMGB3	-1.5205493
HMGCL	-0.5680485
HMGCS2	-1.2042248
HMGN2	-0.7493243
HMGXB4	-0.220314
HMMR	-0.6769481
HMOX2	-0.2790012
HN1	-0.5404773
HN1L	-0.4937351
HNMT	-1.1191108
HNRNPA0	-0.2193127
HNRNPA1	-0.3278513
HNRNPAB	-0.2922344
HNRNPD	-0.6453071
HNRNPH2	-0.4536939
HNRNPK	-0.3596168
HNRNPLL	-0.6127167
HNRNPU	-0.3601599
HNRNPUL2	-0.3113284
HOGA1	-1.7796674
HOMER2	-0.5636401
HOMER3	-0.6888762

HOPX	-0.359467
HOXB5OS	-1.4867449
HOXB6	-1.5111227
HOXB8	-2.2020056
HOXB9	-1.6103373
HOXC5	-1.6443367
HOXD4	-1.9234531
HP1BP3	-0.3917856
HPCA	-0.513797
HPCAL1	-0.7611194
HPGD	-2.4408185
HPN	-0.6969891
HR	-1.5128798
HRAS	-1.2859845
HRCT1	-0.5103469
HRSP12	-0.8076701
HS1BP3	-0.3738458
HS2ST1	-0.5302477
HS3ST3A1	-0.9457195
HS6ST1	-0.9002056
HSD11B1	-0.4143777
HSD17B10	-0.4153714
HSD17B11	-0.7275367
HSD17B12	-0.2951961
HSD17B4	-0.5330935
HSD3B7	-0.3201857
HSDL2	-0.7749844
HSF4	-0.3950443
HSPA12B	-1.6507266
HSPA14	-0.2667856
HSPA2	-0.3410091
HSPA4L	-0.2953897
HSPB2	-2.2218285
HSPB6	-1.3000994
HSPBP1	-0.7027507
HSPG2	-0.8784919
HTATIP2	-1.0220838
HTRA2	-0.7288098
HTRA3	-0.8988257
HUS1	-0.2028251
HYAL1	-0.4951425
IARS2	-0.8454571
IBA57	-1.3371887
ICA1	-1.1508932
ICA1L	-0.7237527

ICAM4	-1.933403
ICAM5	-0.6306357
ICK	-1.3265922
ICT1	-0.384696
ID1	-0.6955609
ID4	-2.4015749
IDH1	-1.292795
IDH2	-0.7521677
IDH3A	-0.372344
IDH3G	-0.4071159
IDI1	-0.4938612
IFFO1	-1.2409954
IFI30	-0.3743649
IFITM10	-1.4694388
IFRD2	-0.7877002
IFT172	-0.5938091
IFT20	-0.800777
IFT22	-0.4181194
IFT27	-0.4040551
IFT46	-0.4085369
IFT52	-0.2808337
IFT80	-0.4609685
IFT81	-0.3050292
IGF1	-1.0015049
IGF1R	-0.3622327
IGF2R	-0.5935707
IGFBP2	-0.8487005
IGFBP3	-0.6096137
IGFBP4	-0.6759982
IGFBP5	-2.280824
IGFBP6	-1.851152
IGH-VJ558	-1.1735191
IGIP	-0.3596015
IGKV4-53	-0.6670165
IGKV6-20	-0.7490788
IGLL1	-3.2143223
IGLON5	-1.5650935
IGSF10	-1.3301935
IGSF3	-1.7754569
IGSF9B	-0.4752328
IKBIP	-0.6959453
IKZF5	-0.3775954
IL17RD	-0.9200108
IL17RE	-0.7885618
IL18BP	-0.4771953

IL1RL1	-0.5702868
IL1RL2	-1.1148882
IL23R	-0.9306266
IL31RA	-1.3516014
IL34	-0.6644115
IL3RA	-0.8009958
IL4	-1.2360113
IL5RA	-0.4251478
IL7	-2.8816533
ILF2	-0.2589159
ILK	-0.829051
ILKAP	-0.5271182
ILVBL	-0.4818511
IMMT	-0.4227228
IMPA2	-0.6635609
IMPDH2	-0.3645229
INCENP	-0.6140471
INF2	-0.7192965
INMT	-1.1689544
INO80C	-0.7866983
INPP5E	-0.2330012
INPP5J	-1.9677434
INPP5K	-0.4789057
INSIG1	-0.5036197
INSL5	-0.2980084
INSL6	-0.1969051
INTS1	-0.6033921
INTS10	-0.6654911
INTS4	-0.312716
INTS7	-0.5137377
INTS9	-0.4028478
INTU	-0.9185939
IP6K1	-0.4786177
IP6K2	-0.413466
IPMK	-0.3769662
IPO9	-0.4766369
IPP	-0.5653462
IQCD	-0.691062
IQCE	-0.2517881
IQCF5	-0.6813722
IQCG	-0.4139652
IQGAP2	-0.5459192
IQGAP3	-1.3725874
IQSEC1	-0.396687
IRAK1BP1	-0.5599213

IREB2	-0.2712092
IRF2BP1	-0.666492
IRF2BPL	-0.2792988
IRS1	-0.267535
IRS3	-0.7818685
IRX4	-0.5154343
ISCA1	-1.4249673
ISCA2	-1.0418735
ISCU	-0.3350029
ISG20	-0.3966632
ISG20L2	-0.2315526
ISLR	-1.0153558
ISLR2	-1.5156531
ISM1	-1.3472757
ISPD	-1.595428
ISYNA1	-0.2619805
ITCH	-0.5140467
ITFG3	-0.9348674
ITGA11	-1.2161653
ITGA2B	-0.5104861
ITGA3	-0.9147582
ITGA4	-0.4875097
ITGA6	-0.6547632
ITGA8	-0.9559765
ITGAD	-0.5591846
ITGAX	-0.6154542
ITGB1	-0.3859711
ITGB1BP2	-0.877682
ITGB5	-1.881392
ITGB7	-0.6198931
ITIH5	-0.8150308
ITM2A	-0.3018494
ITM2B	-0.6734715
ITSN1	-0.2439537
IVD	-0.8623123
IYD	-0.3088613
JADE1	-0.1902542
JADE3	-1.3009009
JAG1	-0.3219805
JARID2	-0.6699691
JMJD7	-0.3227034
JMJD8	-0.2153747
JMY	-0.4370098
JOSD1	-0.8385873
JOSD2	-1.8214111

JPX	-0.7696619
JUND	-0.4499569
JUP	-1.7593701
K230015D01	-0.8272247
KANK1	-0.6495948
KANK2	-1.4354134
KANK3	-0.7109904
KANSL2	-0.5618901
KARS	-0.2757726
KAT2B	-0.5762019
KAT6B	-0.3011398
KAT7	-0.2043781
KATNAL1	-0.3984356
KATNB1	-0.4069734
KAZALD1	-2.8796854
KAZN	-0.4755602
KBTBD12	-1.5860353
KBTBD3	-1.914444
KCNA7	-0.5150456
KCNAB3	-0.8022838
KCNB1	-0.8488071
KCNC1	-1.1015666
KCNC4	-0.6551149
KCND3	-0.7330985
KCNG2	-0.889647
KCNH2	-0.4778619
KCNIP3	-0.6530771
KCNJ1	-1.8178551
KCNJ10	-1.1873894
KCNJ16	-5.7732553
KCNK12	-2.2088904
KCNK13	-1.1240919
KCNK4	-0.4905493
KCNK5	-0.3123846
KCNMB4OS2	-5.87776
KCNN4	-1.4855299
KCNS3	-0.915278
KCP	-0.867841
KCTD12B	-2.1295002
KCTD15	-0.4390874
KCTD17	-0.8896108
KCTD19	-1.3067126
KCTD2	-0.3406043
KCTD5	-0.2191129
KCTD6	-0.5150547

KDELC1	-0.8696742
KDELC2	-0.4537377
KDELR1	-0.8280273
KDELR2	-0.2588172
KDELR3	-0.9599361
KDM1A	-0.2637048
KDM3A	-0.1750679
KDM6A	-0.761148
KDM7A	-0.5449996
KDR	-0.465929
KEAP1	-0.2593808
KEL	-1.3362613
KHDRBS3	-0.9446373
KHK	-1.3068395
KIF11	-1.3276634
KIF13A	-0.622119
KIF14	-0.4474781
KIF15	-1.4438813
KIF17	-0.3410914
KIF18A	-1.1797161
KIF18B	-1.2402177
KIF19A	-0.5201564
KIF20A	-0.9589648
KIF20B	-0.4780879
KIF22	-1.2457361
KIF23	-1.2244735
KIF24	-1.0155737
KIF26A	-1.7272453
KIF2A	-0.7393799
KIF2C	-1.3002965
KIF3A	-0.4142585
KIF4	-1.1763449
KIF5A	-1.4428556
KIF5C	-0.3205135
KIF7	-0.9079492
KIF9	-0.6136217
KIFAP3	-0.5828786
KIFC1	-2.5702724
KIFC3	-1.2551548
KIN	-0.2078915
KIT	-0.6253629
KITL	-0.5502849
KLC1	-0.342093
KLC4	-0.5486937
KLF1	-1.9294882

KLF10	-0.9469037
KLF11	-0.7874341
KLF12	-0.2578092
KLF13	-0.4310851
KLF16	-1.4985461
KLF2	-0.2879491
KLF3	-0.4560294
KLF4	-0.7575612
KLF5	-0.7848315
KLHDC10	-0.701262
KLHDC2	-0.894969
KLHDC3	-0.6761336
KLHDC8B	-0.9888356
KLHDC9	-1.3791878
KLHL12	-1.049326
KLHL13	-1.3545444
KLHL14	-0.8246729
KLHL22	-0.2790685
KLHL23	-2.4298725
KLHL25	-0.5224981
KLHL30	-1.0284
KLHL32	-0.481415
KLHL36	-0.392159
KLHL7	-1.4986448
KLK1B27	-0.4054821
KLK1B9	-1.1041977
KLKB1	-1.1195586
KLRA10	-0.2614892
KLRA15	-0.8906541
KLRA19	-0.6716361
KLRA22	-0.3205276
KLRA4	-0.7908447
KLRA9	-0.9230237
KLRB1C	-1.2101002
KLRB1F	-1.1882541
KLRC3	-0.8332927
KLRD1	-0.2781439
KLRK1	-0.9010096
KMT2B	-0.3246226
KMT2E	-0.361053
KNSTRN	-0.7060542
KNTC1	-1.1298003
KPNA1	-0.3788695
KPNA2	-0.4873147
KPNB1	-0.4094148

KRAS	-0.9211745
KRT14	-0.5735288
KRT15	-0.9871526
KRT19	-1.898624
KRT20	-0.7315199
KRT7	-0.7703512
KRT79	-0.6287823
KRTAP20-2	-0.356786
KRTAP31-1	-1.3204582
KRTAP5-4	-3.5360527
KRTAP8-1	-1.2817848
KRTAP9-1	-0.4911673
KTI12	-0.199769
KXD1	-0.4774814
L2HGDH	-0.7337501
L3HYPDH	-2.383986
L3MBTL2	-0.6861134
LACE1	-0.5319507
LAG3	-0.55742
LAGE3	-0.8573942
LAIR1	-0.8816509
LAMA2	-0.8759089
LAMB2	-0.4182868
LAMP1	-0.2904296
LAMTOR1	-0.6725206
LAMTOR4	-0.7842107
LARP1B	-0.5006623
LARP7	-0.4210038
LARS	-0.261992
LARS2	-0.4043894
LASP1	-0.3415203
LAT2	-0.4238257
LBH	-0.2584219
LBP	-0.2922401
LBR	-0.4979119
LBX2	-1.4175291
LCAT	-0.8486452
LCLAT1	-0.8550131
LCMT1	-0.9388819
LCT	-1.1496696
LDB1	-0.4930005
LDB2	-2.6812296
LDHB	-1.3093932
LDHC	-1.8428597
LDLRAP1	-1.6317024

LENG8	-0.6291628
LEPREL1	-0.3449605
LEPREL2	-1.084157
LEPREL4	-0.3864014
LEPROT	-0.8162718
LFNG	-0.8724601
LGALS1	-0.7103119
LGALS2	-1.202992
LGALS3	-0.2019587
LGALS7	-2.0005636
LGALSL	-0.5680461
LGR5	-1.5312526
LGR6	-0.7145882
LHFPL2	-0.8654826
LHPP	-0.6396284
LHX2	-0.5588915
LIAS	-0.6262035
LIFR	-0.7628317
LIG1	-1.1090426
LILRA5	-0.3229077
LIMCH1	-1.687048
LIMK1	-0.6425052
LIMS1	-0.3419905
LIMS2	-1.0683482
LIN28A	-0.7461762
LIN37	-0.5689297
LIN52	-0.8559294
LIN54	-0.3484311
LIN9	-0.3520603
LIPA	-1.3874292
LIPN	-3.1985955
LMBR1	-1.1176679
LMCD1	-1.1725295
LMF2	-0.5835776
LMNA	-1.8633504
LMNB1	-1.3395615
LMO1	-1.0830688
LMO2	-0.8789711
LMO7	-0.4998877
LMTK2	-0.8032596
LMTK3	-0.5841131
LNP	-0.6880522
LOC1000450	-3.522418
LOC1000488	-4.3503284
LOC1010557	-0.4379532

LOC1010558[illegible]	-0.755914
LOC1010560[illegible]	-0.4861269
LOC1026322[illegible]	-0.2084093
LOC1026335[illegible]	-0.2054124
LOC1026336[illegible]	-1.0458243
LOC1026362[illegible]	-0.5379982
LOC1026381[illegible]	-0.3548985
LOC1026385[illegible]	-1.8321667
LOC1026386[illegible]	-0.4729347
LOC1026402[illegible]	-0.2207007
LOC1026407[illegible]	-0.509356
LOC1026407[illegible]	-1.1131554
LOC1026413[illegible]	-1.1870937
LOC1026413[illegible]	-0.3252125
LOC1026414[illegible]	-1.1102028
LOC1026415[illegible]	-0.8518715
LOC1026416[illegible]	-0.4472616
LOC1026420[illegible]	-0.8121676
LOC1026424[illegible]	-0.2098775
LOC1026425[illegible]	-0.7872083
LOC1026427[illegible]	-0.2508688
LOC1026428[illegible]	-0.3016934
LOC1026430[illegible]	-2.2873783
LOC1026430[illegible]	-0.2010298
LOC1026431[illegible]	-0.5853434
LOC1026432[illegible]	-0.1792016
LOH12CR1	-0.7743385
LOXL1	-2.4057026
LOXL2	-0.9487789
LOXL3	-1.308002
LPCAT1	-1.4824314
LPCAT3	-1.2439566
LPHN1	-0.8578503
LPIN1	-0.8176818
LPIN2	-0.603601
LPIN3	-0.2578731
LPL	-0.4974117
LRBA	-0.3937006
LRCH4	-0.3965282
LRFN3	-0.5275788
LRIG1	-0.3841202
LRIG3	-0.5019357
LRMP	-0.4801974
LRP1	-1.1949086
LRP2	-0.8704832

LRPAP1	-0.3343644
LRR1	-1.402955
LRRC1	-0.6497798
LRRC14	-0.4637604
LRRC19	-0.2693152
LRRC20	-1.5634995
LRRC29	-1.793736
LRRC30	-3.6838617
LRRC32	-0.5132833
LRRC36	-1.2516961
LRRC39	-0.5762558
LRRC40	-0.59165
LRRC42	-0.7353923
LRRC4B	-1.8042526
LRRC4C	-1.1149158
LRRC56	-0.7470732
LRRC8B	-1.2547078
LRRCC1	-0.4819965
LRRFIP2	-0.2406199
LRRN1	-3.5677097
LRRN2	-2.5604496
LRRN3	-1.167917
LRRN4	-1.5649261
LRRN4CL	-0.7788982
LRWD1	-0.7106118
LSM1	-0.3814688
LSM11	-0.4818914
LSM14A	-0.3274097
LSM14B	-1.071382
LSM2	-0.5303679
LSM3	-0.4820242
LSM4	-0.2402606
LSM5	-0.419786
LSM7	-0.3495069
LSMEM1	-1.5165911
LSP1	-0.1858506
LSS	-0.3074255
LST1	-0.4053197
LTA4H	-0.840456
LTB	-0.5118856
LTB4R1	-1.0185275
LTBP1	-0.7662499
LTBP2	-0.5017619
LTBP3	-0.7995
LTBP4	-0.5123477

LTC4S	-1.2624092
LTF	-1.0357409
LUM	-1.6036613
LURAP1L	-1.119165
LY6E	-0.4369149
LY6F	-0.6036916
LY6G	-0.2797871
LY6G6C	-0.4005399
LY6G6D	-1.037785
LY6G6E	-0.959482
LY6G6F	-1.0530324
LY6H	-1.5876367
LY6I	-0.8818283
LYL1	-0.7727909
LYNX1	-0.7131968
LYRM4	-0.7592559
LYRM5	-0.4832544
LYSMD3	-0.3950901
LYST	-0.2521598
LZTFL1	-0.4651504
LZTS2	-0.6438971
M1AP	-0.9744864
MACROD1	-0.7358122
MACROD2	-0.4739158
MAD1L1	-1.0755672
MAD2L1BP	-0.4169726
MAF	-1.4690204
MAF1	-0.7626
MAFA	-0.2451277
MAFB	-2.1269815
MAFG	-0.2895107
MAFK	-0.3757405
MAGEB16	-0.5667672
MAGED1	-0.3450265
MAGED2	-0.8988195
MAGEF1	-0.2163405
MAGEH1	-1.212234
MAGEL2	-0.6406543
MAL	-2.7023754
MAN1C1	-0.9714947
MAN2B1	-0.9294834
MAN2B2	-1.1016455
MANBA	-0.8221879
MANBAL	-0.2820706
MANSC1	-1.4097197

MAOA	-0.9753232
MAOB	-1.1141055
MAP1A	-1.27159
MAP1LC3A	-1.5251064
MAP1LC3B	-0.9332662
MAP1S	-0.3213911
MAP2	-0.7551704
MAP2K2	-0.633666
MAP2K3	-1.0637956
MAP2K4	-0.6554613
MAP2K6	-1.5748096
MAP3K11	-0.3880601
MAP3K15	-0.5309084
MAP3K3	-0.4503884
MAP3K5	-0.2808466
MAP4K2	-0.3821321
MAP4K3	-0.6754541
MAP4K5	-0.6262679
MAP6	-0.3946676
MAPK13	-0.4072166
MAPK14	-0.5361805
MAPK3	-1.3628364
MAPK8	-0.6690953
MAPK8IP1	-1.4565132
MAPK9	-0.6269999
MAPRE3	-0.8114224
MAPT	-1.8722913
MARS	-0.541985
MARVELD1	-0.4864907
MAST2	-0.7499156
MAST4	-0.417088
MASTL	-0.7560389
MAT2B	-0.4614725
MATN2	-0.399503
MATR3	-0.5835109
MAU2	-0.6212587
MAVS	-0.2930441
MAZ	-0.3106279
MBD2	-0.2480183
MBD3	-0.4016881
MBLAC1	-0.9767451
MBLAC2	-0.54232
MBNL1	-0.3569269
MBNL3	-0.8841076
MBOAT2	-1.7166061

MBP	-0.2820482
MC1R	-2.6382651
MCAM	-0.2511525
MCC	-1.2242808
MCCC2	-1.0368824
MCEE	-0.6651104
MCF2L	-0.7148373
MCM10	-0.8079534
MCM2	-1.37606
MCM3	-0.9328084
MCM3AP	-0.8813517
MCM4	-0.9845953
MCM5	-1.3972297
MCM6	-0.7659869
MCM7	-1.3431988
MCM8	-0.5641673
MCOLN3	-0.5441184
MCPH1	-0.6851959
MCPT1	-2.0975196
MCRS1	-0.7160602
MCTP1	-1.5777278
MCU	-0.8825953
MDFI	-1.3243406
MDH2	-0.2984891
MDM1	-0.7930424
ME1	-0.3459191
ME2	-0.8270865
MEAF6	-0.2780304
MED12	-0.4262996
MED18	-0.5312676
MED19	-0.6449056
MED20	-0.3707995
MED22	-0.7061329
MED24	-0.5132451
MED25	-0.9101639
MED26	-0.5097108
MED30	-0.364953
MED4	-0.4224172
MED6	-0.3710785
MED7	-0.3459024
MED9	-0.4581862
MED9OS	-1.3617642
MEG3	-1.5739508
MEGF6	-1.1328943
MEGF8	-0.6975884

MEGF9	-1.0085423
MEIOB	-0.5087879
MEIS1	-0.6269174
MEIS2	-1.1441245
MEMO1	-0.7247295
MEN1	-0.4250679
MERTK	-1.157702
MEST	-1.1620672
METAP1D	-0.453752
METAP2	-0.7524014
METRN	-0.6607571
METTL13	-0.3233728
METTL15	-0.3775244
METTL21A	-0.4783282
METTL22	-0.4892225
METTL24	-2.798071
METTL5	-0.3155108
METTL8	-0.2711453
METTL9	-0.3708744
MEX3B	-0.6002266
MFAP2	-1.1761038
MFAP3L	-1.9136124
MFAP4	-0.3481164
MFAP5	-1.5973384
MFF	-0.5518928
MFN1	-0.9067731
MFN2	-0.3680687
MFSD10	-0.8605652
MFSD11	-0.6081462
MFSD12	-1.070025
MFSD3	-0.5384355
MFSD7C	-1.9492419
MGAT2	-0.3854809
MGAT3	-3.835588
MGAT4B	-1.5063348
MGLL	-1.7812414
MGMT	-0.2765069
MGP	-2.505509
MGST1	-1.1685452
MGST2	-0.7063823
MGST3	-1.6293612
MIB2	-0.1881361
MICAL2	-0.3325825
MICAL3	-1.1150551
MICALL2	-1.3578796

MICU1	-0.4913344
MICU2	-0.4276686
MID1	-0.6479957
MID1IP1	-0.4738345
MID2	-1.2724428
MIER3	-0.8718309
MIIP	-0.7948775
MILL2	-1.3692327
MINOS1	-0.296895
MINPP1	-1.2023559
MIOX	-0.7602036
MIS12	-0.6644054
MIS18A	-0.7271638
MIS18BP1	-0.6784473
MKI67	-0.862699
MKLN1	-0.5342689
MKNK2	-0.3819223
MKRN1	-1.0105882
MLANA	-5.3769894
MLH1	-0.4040961
MLLT10	-0.5016422
MLLT3	-0.6204104
MLPH	-1.8809052
MLST8	-0.6542282
MMGT1	-0.3555894
MMP11	-1.9496074
MMP12	-1.2804506
MMP15	-2.8417647
MMP17	-0.7731137
MMP2	-1.552372
MMP23	-0.2107763
MMRN1	-1.0207844
MN1	-2.1236799
MNAT1	-0.4596391
MND1	-1.2555294
MNS1	-1.5013137
MOAP1	-0.5145087
MOB2	-0.8460851
MOCOS	-0.490809
MOCS2	-0.4367771
MOGAT2	-2.1330497
MOK	-0.9371574
MORF4L1	-0.4718018
MORF4L2	-0.3671994
MORN1	-0.9194381

MORN2	-0.1923249
MORN4	-0.7743964
MOSPD1	-0.8781228
MOSPD3	-0.9376907
MPC2	-0.633894
MPDU1	-0.2203302
MPDZ	-1.1110601
MPEG1	-0.5982046
MPG	-0.6003957
MPHOSPH6	-2.1842232
MPHOSPH9	-0.787396
MPI	-0.4633584
MPL	-0.6315613
MPLKIP	-0.4387622
MPO	-0.8229957
MPP1	-1.0320935
MPP2	-1.4168744
MPPE1	-0.2453692
MPPED2	-0.929816
MPRIP	-0.5598803
MPST	-1.1379433
MPV17	-0.2587247
MPV17L2	-1.4081063
MPZL1	-0.4385757
MR1	-1.5443707
MRAP	-2.3300903
MRAP2	-2.6468787
MRC1	-2.5503783
MRC2	-1.2198918
MRE11A	-0.4608803
MRGPRE	-0.2538085
MRI1	-0.7115564
MRM1	-0.3472672
MRPL10	-0.1944704
MRPL11	-0.3691502
MRPL12	-0.5013809
MRPL13	-0.3593774
MRPL14	-0.4453635
MRPL18	-0.3885317
MRPL19	-0.3113384
MRPL2	-0.5101242
MRPL20	-0.394639
MRPL22	-0.3805862
MRPL27	-0.5226722
MRPL28	-0.9940167

MRPL33	-0.3112063
MRPL34	-0.354888
MRPL37	-0.6151476
MRPL40	-0.3240976
MRPL41	-0.369154
MRPL42	-0.4214206
MRPL46	-0.2112446
MRPL47	-0.2066307
MRPL48	-0.4642487
MRPL49	-0.5114822
MRPL51	-0.4062519
MRPL53	-0.4933372
MRPL54	-0.3001747
MRPL55	-0.4011498
MRPL57	-0.363174
MRPL9	-0.2991524
MRPS11	-1.0095687
MRPS12	-0.202899
MRPS15	-0.3267846
MRPS17	-0.2289867
MRPS18A	-0.1881824
MRPS24	-0.4839788
MRPS26	-0.402348
MRPS27	-0.3173723
MRPS28	-0.4041939
MRPS31	-0.2590675
MRPS34	-0.2408423
MRPS35	-0.6021724
MRPS36	-0.316895
MRPS5	-0.3063574
MRVI1	-1.0795922
MS4A3	-0.2828593
MS4A8A	-0.7873416
MSANTD3	-0.6574936
MSC	-1.4184532
MSH2	-0.3367257
MSH3	-0.2190137
MSH6	-0.5670967
MSLN	-0.8142085
MSMB	-1.591404
MSMO1	-0.2941275
MSMP	-0.3148162
MSRA	-0.2364636
MSRB2	-0.5865116
MSRB3	-1.000279

MST1	-0.7619302
MST1R	-0.9775548
MSTO1	-0.1866798
MSX1	-1.5246999
MTA1	-0.6541209
MTA2	-0.2733636
MTA3	-0.4653564
MTAP7D3	-0.5175254
MTCH1	-0.2870622
MTCH2	-0.4167161
MTERFD1	-0.2804761
MTF1	-0.3584948
MTF2	-0.2871504
MTFP1	-0.8917899
MTFR1	-0.9788418
MTHFD1	-0.6813111
MTHFD1L	-0.4098177
MTHFD2	-0.3929405
MTHFR	-0.505064
MTL5	-1.731926
MTMR10	-0.5442486
MTMR2	-0.4185581
MTMR4	-0.3117147
MTMR9	-0.6922264
MTO1	-0.4716711
MTR	-0.3702755
MTRF1	-0.3564889
MTSS1L	-2.5865705
MTUS1	-0.4141405
MTUS2	-0.7797017
MTX1	-0.3529215
MTX2	-0.4551497
MUC6	-0.6128781
MUG1	-1.0918024
MUG2	-1.0332298
MUM1	-0.7644019
MUP1	-3.0010715
MUP17	-2.7033257
MUP19	-3.6626303
MUP21	-0.3830004
MUP4	-1.4366035
MUS81	-0.3865829
MUT	-0.4680133
MVB12B	-1.03159
MVK	-0.5436006

MXD3	-1.6002607
MXD4	-0.5671887
MXRA7	-1.5458357
MXRA8	-1.754519
MYADM	-0.342865
MYADML2	-1.218334
MYB	-0.9587207
MYBL2	-1.1908851
MYCT1	-1.4320269
MYEF2	-0.375073
MYG1	-0.6788287
MYH1	-2.038703
MYH10	-1.4971712
MYH2	-1.6311603
MYH8	-1.7083135
MYL12B	-0.4328442
MYL6	-0.3687382
MYL6B	-0.975549
MYL9	-0.5564256
MYLIP	-0.2914834
MYLK3	-0.8353479
MYLPF	-0.6885183
MYO18A	-0.8260012
MYO1D	-0.3945818
MYO1F	-0.6826849
MYO5B	-0.8115575
MYO7A	-0.8805757
MYO9A	-1.0307314
MYOM1	-1.396368
MYOM3	-2.8665824
MZB1	-0.3333459
MZT1	-1.2799029
MZT2	-1.0907149
NAA10	-0.7266312
NAA15	-0.5115161
NAA38	-0.5369716
NAAA	-0.6331005
NAALADL1	-1.142252
NABP2	-0.2701798
NACAD	-2.8335214
NACC1	-0.3477278
NACC2	-0.741128
NADK2	-1.0575151
NAGA	-1.1367764
NAGLU	-0.5157642

NAIP2	-0.4816279
NANOS1	-0.4364009
NANP	-1.1226163
NANS	-0.3272882
NAP1L4	-0.3230062
NAPA	-0.5200996
NAPRT1	-1.2368054
NARF	-0.9454346
NARS	-0.324163
NASP	-0.550395
NAT2	-0.3676596
NAT6	-1.4986157
NAT8L	-2.6644976
NAV1	-0.5744906
NAV3	-0.4644764
NBEAL2	-0.7269135
NBR1	-0.4415455
NCAM1	-1.4589248
NCAPD2	-1.047679
NCAPD3	-0.9208794
NCAPG	-0.8455923
NCAPG2	-1.4256201
NCAPH	-0.8357167
NCAPH2	-0.7878623
NCBP1	-0.1786122
NCK2	-0.2281451
NCKAP1	-0.3858452
NCKIPSD	-0.2678845
NCLN	-0.3615365
NCOA3	-0.2099605
NCOA4	-0.6117029
NCR1	-0.6612725
ND1	-0.2109857
ND4L	-0.3566551
NDC80	-0.7316356
NDE1	-0.5108895
NDN	-0.610805
NDRG2	-0.8671694
NDST1	-0.3925338
NDST2	-0.9182034
NDST3	-0.8995922
NDUFA10	-0.385438
NDUFA11	-0.2908702
NDUFA2	-0.4918399
NDUFA4	-0.7163291

NDUFA4L2	-0.958524
NDUFA5	-0.263227
NDUFA6	-0.3237763
NDUFA7	-0.4278135
NDUFA8	-0.214191
NDUFA9	-0.3897042
NDUFAF3	-0.65486
NDUFAF7	-0.4375854
NDUFB10	-0.550168
NDUFB11	-0.1947198
NDUFB4	-0.2759643
NDUFB7	-0.6290369
NDUFB8	-0.4970012
NDUFB9	-0.6664681
NDUFS1	-0.4936471
NDUFS4	-0.3933172
NDUFS6	-0.4045076
NDUFS7	-0.4003015
NDUFS8	-0.4478974
NDUFV1	-0.6873655
NDUFV2	-0.437366
NDUFV3	-0.3993435
NECAP2	-0.8952475
NEDD1	-0.3391066
NEDD4	-0.9829121
NEDD8	-0.2326222
NEDD9	-0.3902187
NEIL2	-1.0228887
NEIL3	-1.5131388
NEK1	-0.8859742
NEK2	-0.7963657
NEK7	-0.3102689
NEK8	-0.992106
NELFA	-0.5245237
NELFCD	-0.58288
NELFE	-0.6142449
NENF	-1.2611065
NES	-0.594914
NET1	-0.6598766
NEU3	-0.3956275
NEXN	-1.019074
NFAM1	-1.1243424
NFASC	-0.9859648
NFATC2IP	-0.3677955
NFATC4	-0.65832

NFE2	-1.2973042
NFIA	-1.8604007
NFIB	-0.8150129
NFIC	-1.5289116
NFIX	-1.0460792
NFKBIL1	-0.2311349
NFU1	-0.3061161
NFX1	-0.2179384
NFYB	-0.4432044
NFYC	-0.4219599
NGFRAP1	-1.1840863
NGP	-0.8877988
NHEJ1	-0.7023907
NHLRC2	-0.4341788
NHLRC4	-2.099299
NHP2L1	-0.1861634
NHS	-0.5668206
NICN1	-1.011384
NID2	-1.15972
NINJ2	-1.7774444
NINL	-1.243304
NIPA1	-1.0520325
NIPAL3	-0.2976508
NIT2	-0.2371049
NKAIN4	-2.0447657
NKD1	-1.5821614
NKG7	-0.7416782
NKIRAS2	-0.2396188
NKX2-3	-1.7144296
NKX2-5	-0.8641992
NLGN2	-0.3817101
NLRP12	-0.7112317
NLRP5	-1.0695393
NLRX1	-0.4045315
NME3	-1.0269284
NME4	-1.3797617
NME6	-0.2988658
NME7	-1.2781732
NME9	-2.2544944
NMNAT3	-0.8887081
NMRAL1	-0.8140664
NMT1	-0.2074814
NNMT	-1.1801035
NOC4L	-0.3116245
NOL3	-1.1997182

NOL9	-0.4815059
NOS1	-0.9468684
NOS1AP	-0.9017179
NOSTRIN	-0.7394183
NOTCH3	-0.5105686
NOTCH4	-0.8584542
NPAS4	-0.8614583
NPC1	-0.5458183
NPC2	-0.3770986
NPCD	-1.2652633
NPHP1	-1.442157
NPL	-0.3363819
NPLOC4	-0.4415798
NPM3	-0.5559208
NPNT	-0.5048339
NPPA	-0.5484951
NPR1	-2.1732802
NPR2	-0.4801831
NPRL3	-0.9663668
NPTN	-0.5231872
NPY	-3.1448357
NQO1	-2.1468263
NQO2	-0.906786
NR1D1	-1.7955186
NR1D2	-0.47521
NR2C2	-0.2725663
NR2C2AP	-0.5052605
NR2F1	-1.7077618
NR2F2	-0.7746706
NR2F6	-0.5530577
NR5A1	-0.4250197
NRADD	-0.6360459
NRARP	-1.3373988
NRBF2	-0.2845678
NRBP1	-0.5044909
NRBP2	-1.1711285
NRDE2	-0.6972466
NREP	-3.2677007
NRF1	-0.8001118
NRG1	-0.3977044
NRG2	-0.7765191
NRGN	-1.3404312
NRIP1	-0.3771796
NRIP3	-2.3388398
NRL	-1.1437535

NRM	-1.0176339
NRP1	-0.5620837
NRXN1	-0.4993331
NRXN2	-0.9793925
NSDHL	-0.7171464
NSL1	-0.990768
NSMCE1	-0.7125344
NSMCE2	-0.441545
NSMCE4A	-0.3571143
NT5C2	-1.0815964
NT5C3	-0.4845104
NT5DC1	-0.3345027
NT5DC2	-1.5082524
NTAN1	-0.3303833
NTM	-1.259295
NTMT1	-0.2779622
NTN3	-1.2633362
NTN4	-2.1191175
NTPCR	-0.5789571
NTS	-0.6614914
NUAK1	-1.487601
NUBP2	-0.4726725
NUCB1	-0.8354235
NUCB2	-0.6199131
NUCKS1	-0.4851394
NUDCD1	-0.5483398
NUDCD2	-1.0859838
NUDT1	-0.8163962
NUDT12	-1.1724114
NUDT14	-0.7187581
NUDT16	-0.3867044
NUDT16L1	-0.4249044
NUDT19	-0.6163573
NUDT2	-0.4595008
NUDT21	-0.5458813
NUDT22	-0.9885955
NUDT4	-0.6555934
NUDT5	-0.3358154
NUDT7	-0.4942689
NUDT8	-0.4096689
NUDT9	-0.62149
NUF2	-1.0899849
NUP107	-0.4734211
NUP133	-0.4898858
NUP155	-1.0041199

NUP160	-0.4107881
NUP188	-0.3604701
NUP35	-0.5055361
NUP43	-0.4816475
NUP50	-0.7969537
NUP54	-0.1729493
NUP85	-0.5392022
NUPL1	-0.391223
NUSAP1	-0.9785633
NUTF2	-0.4673972
NWD2	-1.1258323
NXN	-0.1826549
NXNL2	-1.2527442
NXPE2	-1.521491
NXPE4	-1.4540558
NXPE5	-1.69558
NXPH3	-1.4730489
NYAP1	-0.7990434
OAF	-0.8837633
OARD1	-0.7899799
OAT	-0.593565
OAZ1	-0.5327044
OCA2	-3.1264694
OCIAD2	-1.2253487
OCRL	-0.3400021
ODC1	-0.4570403
ODF2	-0.7677631
ODF3B	-1.9619033
OGDH	-0.3506913
OGFOD1	-0.4194403
OGFOD2	-0.5377545
OGFOD3	-0.8564773
OGT	-0.2226763
OIP5	-0.8731823
OIT3	-2.0079117
OLFM1	-0.4564221
OLFM2	-1.3921218
OLFML1	-3.2348373
OLFML2B	-1.9340477
OLFML3	-1.6542552
OLFR1030	-0.2851346
OLFR1054	-0.5583127
OLFR1077-PS	-0.6045692
OLFR1121	-1.0852859
OLFR1168	-1.0795381

OLFR1186	-1.3252051
OLFR12	-0.3023515
OLFR1275	-0.6740818
OLFR1340	-1.6480267
OLFR1352	-1.0993361
OLFR1463	-2.678186
OLFR1465	-1.4676094
OLFR1466	-2.1381423
OLFR1490	-1.1738329
OLFR161	-0.2822776
OLFR297	-0.7622516
OLFR339	-1.0793719
OLFR466	-0.428488
OLFR469	-1.3475077
OLFR541	-0.6145947
OLFR611	-1.2378664
OLFR649	-1.1650624
OLFR67	-0.4101403
OLFR695	-0.4424365
OLFR728	-6.072307
OLFR821	-0.6990252
OLFR916	-0.5321865
OMP	-0.7265043
OOSP1	-0.8913775
OOSP2	-1.052398
OPA3	-0.2974868
OPHN1	-1.1366732
OPLAH	-1.1095271
OPTN	-0.5909596
ORC1	-1.7432737
ORC2	-0.9640298
ORC5	-1.119523
ORC6	-1.0222654
ORMDL1	-0.2091394
ORMDL3	-0.6798506
OSBP	-0.6135855
OSBPL1A	-0.7683373
OSBPL2	-0.4967308
OSBPL8	-0.5080037
OSCP1	-1.1310041
OSGEP	-0.5935831
OSGEPL1	-1.0051289
OSGIN1	-0.9731755
OSR1	-1.6469944
OST4	-0.8269782

OSTM1	-0.4595842
OTOF	-0.3855424
OTOP1	-0.8308666
OTUB1	-0.5854502
OTUD5	-0.2628613
OVCH2	-0.1858916
OVOL2	-0.3417697
OXCT1	-0.6089845
OXCT2A	-0.9544466
OXLD1	-0.7682047
OXNAD1	-0.6096554
OXSM	-0.2960756
P2RX1	-1.3407593
P2RX7	-0.6576674
P2RY1	-0.7102547
P2RY12	-0.9298587
P2RY6	-1.2131166
P4HB	-0.3346338
PA2G4	-0.7253175
PABPC1	-0.4289966
PABPC4	-1.1120448
PABPC4L	-1.8918552
PABPC5	-0.3369422
PABPN1	-0.3084803
PABPN1L	-0.5227206
PACRG	-1.6637683
PACS1	-1.0417352
PACSIN3	-0.9712248
PADI4	-0.8297503
PAFAH1B3	-0.7401862
PAFAH2	-1.0972633
PAGR1A	-0.3651094
PAICS	-0.8843317
PAIP2	-0.2949977
PAK1	-1.9538255
PALB2	-1.1301544
PALD1	-0.471292
PALM	-1.2771511
PAN2	-0.3935037
PAOX	-1.1242323
PAPOLA	-0.5727015
PAPPA2	-0.3986993
PAPSS1	-0.482697
PAQR4	-1.8707113
PAQR6	-1.6086395

PAQR7	-0.6412621
PAQR9	-1.7382731
PARD6A	-0.7873068
PARK2	-0.4727304
PARK7	-0.384305
PARL	-0.2006292
PARP1	-0.5244403
PARP16	-0.5616517
PARP2	-0.3740115
PARPBP	-1.1995866
PARVB	-1.0805607
PARVG	-0.6117373
PASK	-1.1352844
PATZ1	-0.474154
PAX3	-2.4164448
PAX8	-0.9836628
PBDC1	-0.2805319
PBK	-1.4648948
PBRM1	-0.6697555
PBX3	-0.6195588
PCBD2	-0.3359981
PCBP4	-0.8018947
PCCA	-0.8129907
PCCB	-0.7534134
PCDH10	-0.6154838
PCDH12	-1.8642092
PCDH17	-1.4317939
PCDH8	-1.5060198
PCDHAC1	-0.6922429
PCDHB16	-0.3991973
PCDHB20	-0.9116614
PCDHB6	-0.5639203
PCGF1	-0.9244442
PCGF2	-0.7080944
PCIF1	-0.428124
PCK1	-2.3654268
PCK2	-0.9115422
PCM1	-0.4345136
PCMT1	-0.5034566
PCMTD2	-0.3688631
PCNA	-0.7707419
PCNT	-0.7165628
PCNX	-0.4260509
PCOLCE	-1.3293157
PCP4L1	-0.7395711

PCSK4	-0.2263825
PCSK5	-0.8123901
PCX	-1.2371488
PCYOX1	-0.3639979
PCYOX1L	-1.0892081
PCYT1A	-0.6457386
PCYT1B	-0.7024272
PCYT2	-0.2765942
PDAP1	-0.5250325
PDCD6	-0.2874069
PDCL	-0.495564
PDDC1	-0.6340294
PDE2A	-0.4703436
PDE6D	-0.541965
PDE8B	-1.6124821
PDGFC	-1.3545635
PDGFD	-1.5661788
PDGFRB	-0.9406414
PDGFRL	-1.2302806
PDHA1	-0.2869825
PDHB	-0.3430872
PDILT	-1.4080691
PDK2	-1.3783991
PDK3	-0.4711824
PDLIM3	-1.4519536
PDLIM7	-0.2226238
PDP2	-0.5488577
PDPK1	-0.3251095
PDS5A	-0.2959113
PDSS1	-0.4748464
PDSS2	-0.3199689
PDXDC1	-0.2038703
PDXP	-0.5323725
PDZD11	-0.499857
PDZK1IP1	-0.7254505
PEAK1	-0.3259158
PEAR1	-0.3417859
PECR	-0.6648736
PEF1	-0.2634597
PEG12	-1.0378911
PEG3	-1.5422626
PELI2	-1.2822258
PELO	-0.2618036
PEMT	-0.3227458
PENK	-0.3661237

PEO1	-0.2796068
PER3	-1.0596507
PERM1	-0.9547818
PERP	-1.4256227
PET112	-0.2709909
PEX11G	-0.4227371
PEX19	-0.8136063
PEX2	-0.6311417
PEX5	-0.7371936
PEX7	-0.6381016
PF4	-0.6307335
PFDN1	-0.4128451
PFKFB1	-1.0129585
PFKFB2	-0.968256
PFKL	-0.4956932
PFKM	-1.9322968
PFN1	-0.2931428
PFN4	-1.2968397
PGAM2	-0.9725206
PGAP2	-0.1791558
PGAP3	-0.8596196
PGBD5	-1.8399563
PGD	-0.4876614
PGGT1B	-0.5852089
PGLS	-0.7654781
PGM5	-0.4194934
PGP	-0.8885589
PGPEP1	-0.3813679
PGRMC1	-0.4593949
PGRMC2	-0.743938
PHACTR1	-1.3297629
PHACTR2	-0.9599562
PHACTR4	-0.5676704
PHB	-0.358994
PHB2	-0.8023639
PHF10	-0.8202367
PHF12	-0.5900865
PHF14	-0.5274048
PHF19	-0.3731732
PHF2	-0.4731426
PHF23	-0.2694817
PHF5A	-1.0459232
PHKA2	-0.8074789
PHKB	-0.3726485
PHKG2	-0.4057279

PHLDA3	-1.5436764
PHLDB2	-1.2988591
PHLPP2	-0.8193317
PHOSPHO1	-1.3809941
PHPT1	-0.5216808
PHYHIP	-2.6706376
PHYKPL	-0.4856811
PI16	-1.475081
PI4K2B	-1.3037949
PI4KB	-0.5456429
PIANP	-1.4042554
PIAS1	-0.3706117
PIAS4	-0.8110247
PIDD1	-1.013475
PIGA	-0.528656
PIGH	-0.5213714
PIGK	-0.4069848
PIGP	-0.5361676
PIGQ	-1.1531234
PIGT	-1.0929432
PIGYL	-0.8556018
PIGZ	-1.5482643
PIH1D1	-0.2294245
PIK3C3	-0.5688267
PIK3R1	-0.5913725
PIK3R2	-1.4350576
PILRB1	-0.4529591
PIN1	-0.3387575
PIN1RT1	-0.3032913
PINK1	-0.9554677
PIP	-0.6344664
PIP4K2A	-0.723805
PIP4K2C	-0.5400085
PIR	-1.4183836
PITHD1	-0.7602429
PITPNB	-0.4047222
PITPNM2	-0.4672098
PITPNM3	-3.3781004
PITRM1	-0.2305708
PJA2	-0.2871933
PKHD1L1	-1.2361434
PKIA	-1.350724
PKLR	-1.8134296
PKMYT1	-0.7484489
PKN2	-0.4105263

PKN3	-0.8576419
PKNOX2	-1.9119928
PLA2G12A	-1.1132283
PLA2G15	-1.611104
PLA2G16	-0.2066031
PLA2G2E	-2.6578188
PLA2G4C	-0.4426994
PLA2G6	-0.5482459
PLAC9A	-0.5987492
PLAU	-0.7962608
PLCB1	-0.8289897
PLCB3	-0.3484125
PLCD1	-0.2542648
PLCE1	-1.0888982
PLCL2	-0.7148795
PLD3	-0.6080918
PLD4	-1.2235856
PLEK2	-2.017899
PLEKHA6	-2.6444829
PLEKHA8	-0.3531852
PLEKHF1	-0.1948166
PLEKHF2	-0.5842738
PLEKHG1	-0.6472323
PLEKHG5	-1.2051766
PLEKHH3	-0.5741615
PLEKHJ1	-0.7455878
PLIN1	-1.0880997
PLIN3	-1.0498095
PLIN4	-1.2378526
PLK1	-1.1361966
PLK4	-1.1652753
PLS1	-0.532794
PLSCR3	-1.3154902
PLSCR4	-2.1678576
PLTP	-0.9186516
PLVAP	-0.6646147
PLXDC1	-2.244755
PLXDC2	-1.2187414
PLXNA4	-1.4605017
PLXNB1	-1.6667147
PLXNB2	-0.5744729
PLXNB3	-2.525587
PMEL	-5.740234
PMF1	-0.4420719
PMM1	-0.7873616

PMM2	-0.6300321
PMP22	-0.6334496
PMS1	-0.6385653
PMS2	-0.7342134
PMVK	-0.705894
PNCK	-0.7882316
PNKP	-0.4087958
PNMAL2	-2.3667417
PNPLA6	-0.5159078
PNPLA7	-0.7587738
PNPO	-1.1942096
PNRC1	-0.3986731
PNRC2	-0.1825538
POC1A	-0.7482305
PODN	-2.6725712
PODXL	-1.8024061
PODXL2	-2.0023234
POLA1	-0.4558082
POLA2	-0.9846492
POLD1	-0.8215952
POLD2	-0.6096258
POLD3	-0.2569666
POLE	-0.8196335
POLE2	-1.0789614
POLE3	-0.7114601
POLK	-0.2709646
POLQ	-0.9802761
POLR1C	-0.3695669
POLR2A	-0.5471268
POLR2B	-0.3444977
POLR2C	-0.2906017
POLR2D	-0.6028533
POLR2E	-0.3616123
POLR2F	-0.4795332
POLR2J	-0.379724
POLR2L	-0.2684197
POLR3B	-0.2820149
POLR3C	-0.2549214
POLR3GL	-0.2916598
POLR3H	-0.4513192
POLR3K	-0.4432812
POMK	-0.6735292
POMT1	-0.9995029
POMT2	-0.7704582
PON2	-0.2467547

POPDC2	-1.580934
POPDC3	-2.4841743
POSTN	-0.6648428
POT1A	-0.2756114
POU3F4	-0.804697
PPA2	-0.6842189
PPAP2A	-0.5049019
PPAP2C	-0.2087812
PPAPDC3	-1.0935304
PPARG	-1.49403
PPAT	-0.6214929
PPBP	-0.6701722
PPFIA2	-1.3751159
PPFIA4	-2.2133443
PPFIBP2	-0.3760505
PPIB	-0.5157476
PPIG	-0.3011074
PPIH	-0.463759
PPIL1	-0.8157935
PPIL2	-0.4287458
PPIL6	-0.7783315
PPM1A	-0.4473047
PPM1D	-0.62045
PPM1E	-1.4140663
PPM1G	-0.829536
PPM1L	-0.9197278
PPME1	-0.5560174
PPNR	-0.7043977
PPOX	-1.5291619
PPP1CA	-0.402463
PPP1CB	-0.9441104
PPP1CC	-0.5342531
PPP1R13L	-0.710577
PPP1R14A	-1.2679877
PPP1R14C	-0.8219872
PPP1R15A	-0.5511446
PPP1R16A	-0.4668994
PPP1R2	-0.6291251
PPP1R32	-0.2993321
PPP1R35	-0.9242449
PPP1R37	-0.844079
PPP1R3A	-0.8772347
PPP1R3B	-0.4257584
PPP1R3C	-3.7189813
PPP1R7	-0.5226188

PPP1R9A	-1.4671566
PPP1R9B	-0.4938388
PPP2CA	-0.1990027
PPP2R1B	-0.6113427
PPP2R3C	-0.337688
PPP2R4	-1.1564641
PPP2R5B	-1.0190969
PPP2R5C	-0.4741263
PPP2R5D	-0.2772059
PPP2R5E	-0.5458071
PPP4R1L-PS	-0.5675533
PPP6R1	-0.5813718
PPP6R2	-0.4071269
PPT1	-0.3136849
PQBP1	-0.2209778
PQLC1	-1.1021304
PQLC2	-0.6230793
PQLC3	-0.9667845
PRADC1	-1.36761
PRC1	-1.1077385
PRDM10	-0.5874271
PRDM16	-2.1541743
PRDM4	-0.3132749
PRDX2	-0.876667
PRDX3	-1.0873461
PRDX4	-0.7864313
PRELID1	-0.20649
PREPL	-0.356564
PRG2	-3.022488
PRG3	-2.4624238
PRG4	-1.2433124
PRIM1	-0.780911
PRIM2	-0.8972025
PRIMPOL	-0.8926439
PRKAA1	-0.5129743
PRKAA2	-1.8367367
PRKAB1	-0.7451572
PRKAG1	-1.3644896
PRKAR1B	-1.5368927
PRKAR2A	-0.6047211
PRKAR2B	-1.2271304
PRKCDBP	-0.63376
PRKDC	-0.3471293
PRKG1	-0.608027
PRKRA	-0.8778095

PRKRIP1	-0.2731433
PRL4A1	-4.5679026
PRM1	-0.3375895
PRMT5	-0.3726015
PRN	-0.1798074
PROCA1	-0.7786808
PROKR1	-1.3366923
PROKR2	-1.2685087
PROM1	-0.9738274
PROS1	-0.5927506
PROSER2	-0.534204
PROX1	-1.6889386
PROX2	-0.7686522
PRPF19	-0.5130439
PRPF3	-0.2106881
PRR11	-0.9623237
PRR12	-0.8896449
PRR15	-1.0187793
PRR24	-0.1917276
PRR5L	-0.8212142
PRR7	-1.6741405
PRRC2A	-0.5383039
PRRC2B	-0.2166572
PRRG2	-0.4738188
PRRX1	-1.0186667
PRRX2	-1.5335233
PRSS33	-0.6352623
PRSS34	-1.2879634
PRSS50	-2.2632723
PRSS57	-1.459266
PRTN3	-1.2007046
PRUNE	-0.8150621
PRX	-0.8994217
PSAP	-1.2549734
PSD	-0.9215593
PSIP1	-0.2716069
PSKH1	-0.7035148
PSMA5	-0.3083425
PSMB1	-0.380086
PSMB4	-0.2003546
PSMB7	-0.8967128
PSMC1	-0.1791267
PSMC3	-0.405952
PSMC3IP	-0.9196487
PSMC4	-0.3339748

PSMC5	-0.3420391
PSMC6	-0.2095628
PSMD11	-0.8418655
PSMD13	-0.7106304
PSMD14	-0.3999972
PSMD5	-0.80055
PSMD6	-0.2078376
PSMD8	-0.3658881
PSMD9	-1.3702207
PSME3	-0.4199362
PSME4	-0.3974738
PSMF1	-0.8979411
PSMG4	-0.342588
PSPC1	-0.7149005
PSRC1	-1.001164
PSTPIP1	-0.6348419
PTBP1	-0.532773
PTBP3	-0.8284354
PTCHD3	-0.4108884
PTDSS2	-1.2094488
PTER	-0.9374564
PTGDS	-4.5855875
PTGER1	-1.4271054
PTGES2	-0.4052115
PTGIS	-0.5363345
PTGR1	-1.5568781
PTGR2	-0.3446178
PTGS1	-1.5058475
PTH1R	-2.4096327
PTMA	-0.285408
PTOV1	-1.2424784
PTP4A2	-0.3505211
PTP4A3	-1.1999316
PTPLA	-0.5206122
PTPLAD2	-2.0014098
PTPN14	-1.0501833
PTPN21	-0.8653369
PTPN7	-1.0059967
PTPN9	-0.3386102
PTPRB	-1.6822753
PTPRD	-0.3744237
PTPRE	-0.2535453
PTPRF	-1.0505629
PTPRO	-1.5293264
PTPRS	-0.3279238

PTPRV	-0.5912635
PTRF	-0.9890389
PTRHD1	-0.4368987
PTS	-1.2107637
PTTG1	-1.0046554
PTTG1IP	-0.4682336
PUF60	-0.3514996
PUM1	-0.5681629
PUM2	-0.2293854
PURG	-0.6682568
PUS10	-0.846982
PWWP2A	-0.4844964
PWWP2B	-0.4427838
PXMP2	-0.6950684
PXMP4	-0.6979828
PXYLP1	-1.6632445
PYCR1	-0.4341874
PYCR2	-0.46032
PYCRL	-0.609982
PYGB	-0.3879275
PYGL	-0.5614801
PYGM	-0.8819041
PYROXD2	-1.3609588
PYURF	-0.561389
QARS	-0.2551351
QK	-0.5377436
QPCT	-1.3898301
QRICH1	-0.2821021
QSER1	-0.3836162
R3HCC1	-0.6402841
R3HDM4	-0.8901076
R3HDML	-0.705277
RAB11A	-0.5716786
RAB11B	-0.5944495
RAB11FIP5	-0.9437146
RAB1B	-0.4653206
RAB24	-0.256135
RAB27B	-1.0397477
RAB31	-0.7564268
RAB34	-0.6174426
RAB36	-1.4027019
RAB37	-0.529913
RAB38	-0.7649326
RAB39	-0.7024546
RAB3A	-0.3135052

RAB3B	-0.8588724
RAB3D	-1.5275958
RAB3IL1	-1.4999213
RAB40B	-1.8746829
RAB42	-1.7307656
RAB4A	-1.0829825
RAB5B	-0.2831712
RAB5C	-1.8439069
RAB6B	-0.9917953
RAB8A	-0.1944218
RABAC1	-0.2620449
RABGEF1	-0.3562713
RABGGTB	-0.2360168
RABL3	-0.3602977
RABL6	-0.2749291
RAC1	-0.3354387
RACGAP1	-0.5548511
RAD1	-0.482007
RAD18	-0.7367935
RAD21	-0.6954289
RAD23A	-0.8404634
RAD50	-0.514564
RAD51	-0.9814959
RAD51AP1	-1.3206296
RAD51B	-0.9407344
RAD52	-0.3321552
RAD54B	-0.4531615
RAD54L	-1.3297334
RAET1E	-0.5818
RAF1	-0.2670679
RAG1	-2.8238451
RAG2	-2.442539
RALBP1	-0.7545705
RALGAPA2	-0.2621563
RALGPS1	-0.358315
RALY	-1.6715708
RALYL	-0.8211949
RAMP1	-1.2558634
RAMP2	-1.4212031
RANBP10	-1.1707144
RANBP2	-0.3174205
RANBP6	-0.8525655
RANBP9	-0.2377563
RANGAP1	-0.3684378
RANGRF	-0.5909319

RAP1A	-0.3432241
RAP1GAP	-0.6678143
RAPGEFL1	-2.042456
RARA	-0.6537728
RARB	-0.5883689
RARG	-0.8248377
RASA3	-0.2840691
RASAL1	-0.4023898
RASD2	-0.9604473
RASGEF1C	-1.0222385
RASGRP2	-0.6598115
RASL11A	-2.4389422
RASL11B	-0.5463064
RASL12	-0.8116558
RASSF4	-0.3042069
RASSF7	-1.1132622
RASSF9	-0.8870459
RB1	-0.9056368
RBBP4	-0.27702
RBBP5	-0.4462862
RBBP7	-0.4794121
RBBP8	-0.401051
RBBP9	-0.8426511
RBFA	-0.863327
RBFOX1	-0.6743507
RBFOX3	-0.5665269
RBKS	-0.6557722
RBL1	-0.6087556
RBL2	-0.8137531
RBM15B	-0.2364969
RBM22	-0.5795884
RBM24	-2.0495567
RBM3	-1.3635192
RBM38	-1.3758407
RBM42	-0.2401438
RBM45	-0.3615947
RBM7	-0.3119655
RBM8A	-0.4503117
RBMS2	-0.2446232
RBMX	-0.2863855
RBMX2	-0.7293043
RBP1	-0.527257
RBP4	-1.8676729
RBP7	-3.9609554
RBPMS	-0.2622361

RC3H1	-0.2115235
RCAN1	-0.8159032
RCAN2	-1.2749228
RCAN3	-1.3541455
RCBTB2	-0.6387091
RCC1	-0.3567019
RCC2	-0.242867
RCHY1	-0.6469288
RCN2	-0.294785
RCN3	-1.1615438
RCOR1	-0.4027734
RCOR2	-0.4609065
RDX	-0.7911682
REC114	-0.6617086
RECQL	-0.5102749
RECQL4	-1.2998309
RECQL5	-0.4940875
REDRUM	-1.5803447
REEP1	-0.7134526
REEP2	-1.101753
REEP4	-0.9337745
REEP5	-0.8795681
REEP6	-1.4947729
RELL1	-0.9010887
RELL2	-2.4583914
RELT	-0.6055522
REM1	-0.6720576
REN2	-1.0086262
REPS2	-1.7417812
RETN	-2.4526742
RETNLA	-5.525751
RETSAT	-1.2163453
REV1	-0.687978
REXO2	-0.3912482
RFC1	-0.8539872
RFC2	-0.716989
RFC3	-0.688509
RFC5	-0.5864091
RFK	-0.6787725
RFNG	-0.4113588
RFWD3	-0.7437182
RFX1	-0.319921
RFX2	-2.10298
RFXANK	-0.5255876
RGAG4	-0.4194493

RGCC	-1.237719
RGL2	-0.2560787
RGMB	-0.3696196
RGS10	-1.0271173
RGS12	-1.5149403
RGS14	-0.2535296
RGS18	-0.5604839
RGS2	-1.1903834
RGS3	-0.6043434
RHAG	-1.0182128
RHBDD3	-0.6077919
RHBDL1	-0.4650459
RHBDL3	-1.2578213
RHD	-1.0088234
RHNO1	-0.6369362
RHOA	-0.2541094
RHOD	-1.0471487
RHOG	-0.3921409
RHOJ	-0.1933289
RHOT2	-0.486671
RHOX2H	-2.5079315
RHPN2	-0.9466941
RIBC1	-0.3789289
RICTOR	-0.3558307
RIIAD1	-0.6434145
RILP	-0.341115
RIMBP3	-0.7871893
RIMS3	-2.3636162
RIN2	-0.9217169
RING1	-0.3951249
RINT1	-0.7055197
RIOK1	-0.3862581
RIOK3	-0.479104
RIPPLY3	-0.6045089
RIT1	-0.7494774
RMDN1	-0.5061913
RMDN3	-0.4819932
RMI1	-0.4857287
RMND5B	-0.7657251
RNASE12	-1.7047765
RNASE2A	-2.30785
RNASE4	-0.4995747
RNASE6	-0.6423383
RNASEH2A	-0.884676
RNASEH2B	-0.9266763

RNASEH2C	-0.7387424
RNASEK	-0.613596
RNASEL	-0.9993997
RND2	-0.4989169
RND3	-0.536232
RNF10	-0.9289155
RNF11	-0.9015107
RNF123	-1.366498
RNF126	-0.3153825
RNF128	-0.5598321
RNF130	-0.5508595
RNF139	-0.4214053
RNF144B	-1.9226539
RNF150	-0.791873
RNF152	-2.7087212
RNF165	-0.4593542
RNF166	-0.8064132
RNF167	-0.4556541
RNF168	-0.408998
RNF181	-0.3422766
RNF187	-0.7823124
RNF207	-0.6194718
RNF208	-1.7749882
RNF212	-1.5950322
RNF26	-0.9811401
RNF38	-0.3765545
RNF39	-0.484416
RNF4	-0.2780371
RNF40	-0.4916325
RNF43	-0.5554962
RNF5	-0.5776744
RNF7	-0.2509093
RNF8	-0.3314829
RNH1	-0.5220418
RNPEP	-1.088892
RNPS1	-0.2427878
RNU12	-0.3896499
ROBO2	-1.6638498
ROBO3	-0.5871396
ROGDI	-0.2597709
ROM1	-0.7154784
ROMO1	-0.2907367
ROPN1L	-0.9220793
RORA	-0.4244909
RP9	-0.2789784

RPA1	-0.7043099
RPA2	-1.107914
RPA3	-0.7765613
RPAIN	-0.4462938
RPAP2	-0.3461685
RPGR	-0.2795761
RPGRIP1L	-0.7606294
RPH3AL	-0.5707817
RPL21	-0.3233562
RPL23A	-0.2165737
RPL34-PS1	-0.2460303
RPP21	-0.3375063
RPP25L	-0.4185944
RPP30	-0.4459903
RPRD1B	-0.4855852
RPRM	-1.5410011
RPS2	-0.6880412
RPS27L	-0.3404727
RPS6KA1	-0.5770783
RPS6KA5	-0.6779969
RPS6KB2	-0.4807096
RPUSD1	-0.9755006
RPUSD3	-0.3403063
RPUSD4	-0.4466705
RQCD1	-1.0891929
RRAGA	-0.8872156
RRAGD	-0.6574011
RRM1	-1.256269
RRM2	-1.619338
RSF1	-0.3022475
RSPH1	-0.7817354
RSPH3B	-0.2443914
RSPH9	-1.2675288
RSPO1	-2.425671
RSRP1	-0.5862556
RSU1	-0.3432527
RTCA	-0.3634472
RTEL1	-0.3769288
RTF1	-0.3257732
RTN2	-1.2896287
RTN3	-0.9052458
RTN4R	-2.019495
RTN4RL1	-2.08471
RTP3	-0.6690781
RUFY4	-0.9659252

RUNDC3A	-0.6339989
RUNX1T1	-1.5007064
RUNX2	-0.1855607
RUVBL1	-0.2277494
RUVBL2	-0.3145919
RWDD2B	-0.6620808
RXRA	-0.7094801
RYK	-1.35887
RYR1	-0.7334771
S100A1	-0.8500128
S100A10	-0.9167323
S100A11	-0.7380767
S100A16	-1.0482235
S100B	-4.566986
S100Z	-1.0129545
S1PR4	-0.3196588
S1PR5	-2.0413187
SAAL1	-0.5720749
SAE1	-0.3593292
SALL2	-1.2138233
SAMD1	-0.4681859
SAMD11	-0.7446852
SAMD14	-2.030344
SAMD3	-0.4528558
SAMD5	-1.2098932
SAMM50	-0.7618685
SAP30BP	-0.2446904
SAPCD2	-0.9243107
SARDH	-1.1017928
SARS	-0.3651118
SARS2	-0.6770988
SASS6	-0.45099
SAT1	-0.860405
SAT2	-1.3443158
SBK1	-0.3987098
SBSN	-0.6694031
SCAF4	-0.3986273
SCAMP2	-0.5303636
SCAMP5	-1.913286
SCAND1	-0.394587
SCAP	-0.1783528
SCARB1	-0.9707441
SCARB2	-0.3427882
SCCPDH	-0.4667363
SCD2	-0.883574

SCG5	-0.9556484
SCGB1A1	-0.5864103
SCGB2B20	-0.7537398
SCLT1	-0.5432854
SCLY	-0.7783513
SCMH1	-1.1369247
SCML2	-1.1425862
SCN1B	-1.5357974
SCN2B	-0.2853427
SCNM1	-0.5039215
SCNN1A	-2.8584108
SCOC	-1.0366216
SCP2	-0.3587441
SCRN1	-2.6456532
SCRN3	-1.0904393
SCUBE1	-1.4749007
SCUBE2	-0.5162158
SDC1	-0.3164272
SDC2	-1.1537464
SDC3	-0.9539027
SDCCAG3	-0.1934581
SDHB	-0.2270298
SDHC	-0.3282743
SDHD	-0.5185499
SDK2	-1.1206534
SDPR	-1.6547728
SDSL	-0.7431269
SEC11C	-0.611948
SEC14L1	-0.5963044
SEC14L2	-2.1768408
SEC16B	-2.6852112
SEC23A	-0.5664129
SEC23IP	-0.3397818
SEC31B	-0.586138
SECTM1A	-3.003244
SEH1L	-0.3205781
SELENBP1	-1.7531004
SELM	-1.3540025
SELO	-0.5152078
SEMA3B	-0.7122662
SEMA3C	-0.1845498
SEMA3D	-0.8653743
SEMA4B	-0.3658271
SEMA4G	-1.2290771
SEMA6A	-1.1932225

SENP1	-0.4800491
SENP3	-0.2536969
SEPHS1	-0.634125
SEPP1	-1.2649026
SEPSECS	-0.5882926
SERF1	-0.4791722
SERF2	-0.3592224
SERHL	-0.8314562
SERINC3	-0.271894
SERINC5	-0.913383
SERP2	-0.5833638
SERPINA3A	-2.0114684
SERPINA3H	-1.7258503
SERPINB10	-1.294455
SERPINB2	-0.7313197
SERPINB3C	-0.5972114
SERPINB6A	-0.4184494
SERPINB6C	-0.5032554
SERPINB8	-0.246695
SERPINF1	-0.9744153
SERPINF2	-0.3842244
SERTAD2	-0.5276208
SERTAD4	-2.8860068
SETD1A	-0.6071267
SETD1B	-0.2270846
SETD5	-0.8163714
SETD6	-0.194622
SETD8	-0.5575576
SF1	-0.2215858
SF3B2	-0.5056486
SF3B4	-0.2762418
SF3B5	-0.4585686
SF3B6	-0.3144507
SFMBT1	-0.7769704
SFRP1	-1.4321539
SFRP2	-1.9596114
SFRP4	-2.344123
SFTPB	-0.825645
SFTPC	-1.3242106
SFXN1	-0.2982688
SFXN3	-0.6996908
SFXN5	-0.2929423
SGCE	-0.5519776
SGK3	-0.5003381
SGOL1	-0.8727651

SGOL2	-1.397064
SGPP1	-0.313201
SGSH	-0.2216806
SGTA	-0.2042413
SH2B1	-0.2882705
SH2D1B1	-2.3638833
SH2D3C	-0.2497292
SH2D4A	-2.898794
SH2D5	-0.7370746
SH3BGR	-0.2817996
SH3BGRL	-0.3405943
SH3BGRL3	-0.3045759
SH3BP1	-0.386879
SH3BP5L	-0.260149
SH3D19	-1.2289908
SH3D21	-0.174367
SH3GLB1	-0.3630505
SH3GLB2	-0.4749446
SH3RF2	-3.1218877
SH3TC2	-1.9169221
SH3YL1	-0.7259927
SHANK1	-0.6446099
SHANK3	-0.5826643
SHC1	-0.6657839
SHCBP1	-0.6321821
SHF	-0.8894532
SHISA2	-0.4925883
SHISA4	-0.8230093
SHISA6	-2.0465238
SHISA9	-0.83093
SHKBP1	-0.5617213
SHMT2	-0.4217544
SHOC2	-0.6368375
SHPK	-0.6245425
SIAE	-0.5502353
SIAH1A	-0.5797114
SIAH1B	-0.7183185
SIAH3	-0.279496
SIDT2	-0.3719287
SIGLEC5	-2.274864
SIL1	-0.2831154
SIRPA	-0.4718933
SIRPB1A	-0.5739174
SIRPB1B	-0.6512504
SIRT2	-0.513433

SIRT3	-0.6885037
SIRT4	-0.2711663
SIVA1	-1.0814614
SIX5	-1.0186381
SKA1	-0.7279902
SKA2	-1.6817067
SKA3	-0.6921887
SKP1A	-0.3352323
SKP2	-0.805892
SLAMF8	-0.6170473
SLBP	-1.4183087
SLC11A2	-0.6030865
SLC12A7	-0.2804599
SLC12A9	-0.426703
SLC13A2	-2.0369143
SLC13A3	-3.0349572
SLC14A1	-1.110167
SLC16A10	-0.6812549
SLC16A6	-0.3071175
SLC16A9	-1.1256695
SLC18B1	-0.9415152
SLC19A1	-0.3359141
SLC19A2	-0.8709555
SLC1A1	-0.9400499
SLC1A5	-0.3504067
SLC20A1	-0.4249973
SLC22A17	-1.1769357
SLC22A18	-1.0287788
SLC22A23	-1.3585954
SLC22A4	-1.6764264
SLC22A7	-2.1372843
SLC24A3	-0.2324395
SLC24A5	-0.6962447
SLC25A1	-0.9771524
SLC25A12	-0.4661598
SLC25A13	-0.2352791
SLC25A16	-1.0048392
SLC25A21	-1.5349169
SLC25A24	-0.4906359
SLC25A30	-0.4539123
SLC25A31	-0.8571272
SLC25A34	-0.8480332
SLC25A35	-1.4344318
SLC25A37	-1.4521337
SLC25A38	-1.1234684

SLC25A39	-0.9829106
SLC25A40	-0.4010322
SLC25A42	-0.7045751
SLC25A44	-0.3476062
SLC25A45	-0.7678433
SLC25A47	-0.7062516
SLC25A5	-0.3861804
SLC25A51	-1.2391443
SLC27A1	-1.3158162
SLC27A3	-1.032352
SLC27A4	-0.1871078
SLC29A1	-1.1791253
SLC29A3	-0.8555653
SLC29A4	-1.814347
SLC2A3	-0.7033477
SLC2A4	-1.5833135
SLC2A8	-1.1773152
SLC30A1	-0.5825753
SLC30A10	-2.2239604
SLC30A9	-0.9997988
SLC31A1	-0.3906488
SLC32A1	-0.4739468
SLC33A1	-0.2706757
SLC35A4	-0.6170802
SLC35B2	-0.7790527
SLC35B4	-0.4645257
SLC35C2	-0.4738073
SLC35D1	-0.9019983
SLC35D3	-0.2578709
SLC35F6	-0.6289067
SLC35G1	-0.5747476
SLC36A4	-0.2694893
SLC37A2	-0.647404
SLC37A4	-0.2749052
SLC38A5	-1.5647283
SLC38A6	-0.4221771
SLC38A8	-1.2123382
SLC38A9	-0.331511
SLC39A8	-0.5866838
SLC3A2	-0.4295864
SLC40A1	-4.010758
SLC41A3	-1.2413483
SLC43A1	-0.8203096
SLC43A2	-0.9423919
SLC43A3	-0.2856183

SLC44A1	-0.5847383
SLC44A5	-0.6367946
SLC45A2	-2.8657064
SLC46A1	-1.2418673
SLC46A3	-0.7166858
SLC48A1	-1.3607678
SLC4A1	-1.1962247
SLC4A3	-0.6722281
SLC4A5	-0.5480919
SLC4A9	-0.3729069
SLC51A	-2.375247
SLC5A2	-1.5379415
SLC6A12	-2.053686
SLC6A13	-1.5168085
SLC6A20A	-1.9772687
SLC6A3	-0.5174487
SLC6A4	-0.4332576
SLC6A6	-0.2495475
SLC6A8	-0.7505188
SLC6A9	-1.1457486
SLC7A4	-1.6291101
SLC7A8	-0.5175314
SLC8A2	-0.7039053
SLC9A3R1	-0.453599
SLC9A3R2	-2.342679
SLC9A9	-1.5082529
SLCO2A1	-0.3883128
SLCO2B1	-1.3458006
SLFN14	-1.2667267
SLIT3	-1.0343249
SLITRK6	-1.8403912
SLURP1	-1.7641497
SLX4	-0.493238
SMAD6	-0.4680321
SMAP1	-0.386538
SMARCA1	-1.1643224
SMARCA2	-0.619802
SMARCA5-PS	-0.4867606
SMARCAL1	-0.2783012
SMARCC2	-1.2428017
SMARCD1	-0.6534915
SMARCD2	-0.7426801
SMARCD3	-1.9799426
SMC1A	-0.4623008
SMC2	-0.8963537

SMC3	-0.4449143
SMC4	-0.7912302
SMC6	-0.4077039
SMCO4	-0.5648365
SMEK1	-0.5198135
SMG5	-0.5809226
SMG6	-0.39323
SMG7	-0.2607007
SMG9	-0.3790131
SMIM1	-2.1671152
SMIM11	-0.3417373
SMIM12	-0.3318358
SMIM14	-0.6496759
SMIM24	-0.7867818
SMIM4	-1.3703098
SMIM5	-1.570149
SMIM6	-1.1763089
SMIM8	-0.5152931
SMN1	-0.2715368
SMNDC1	-0.5100412
SMO	-1.1144414
SMOX	-1.1851239
SMPD1	-0.8140206
SMPD2	-0.5327077
SMPD3	-1.0394306
SMPD4	-0.9854698
SMPD5	-0.4009471
SMPDL3A	-0.661129
SMTNL2	-1.4567707
SNAI2	-1.161402
SNAP47	-0.5995703
SNAPC2	-0.4917402
SNAPIN	-0.2204876
SNCA	-1.455946
SNCG	-1.7149813
SND1	-0.4264603
SNED1	-2.9774005
SNF8	-0.2877464
SNHG10	-0.4536843
SNHG3	-0.7652783
SNHG5	-0.4522796
SNIP1	-0.4166665
SNN	-0.3836427
SNPH	-1.0569963
SNRNP25	-1.1009474

SNRNP40	-0.7198329
SNRNP70	-0.3279681
SNRPB	-0.2388339
SNRPB2	-0.4988761
SNRPD2	-0.3420968
SNRPD3	-0.8048101
SNRPE	-0.2071214
SNRPF	-0.315228
SNRPG	-0.2299676
SNRPN	-1.4017515
SNX15	-1.0051599
SNX17	-0.3188105
SNX21	-1.603867
SNX22	-1.6435375
SNX24	-0.7121513
SNX30	-0.2270598
SNX32	-1.0766916
SNX33	-0.773694
SOD1	-0.6697187
SORD	-0.4896307
SORT1	-1.0581806
SOWAHA	-1.3410697
SOX10	-3.5877094
SOX12	-1.1181493
SOX15	-0.6260648
SOX17	-1.8379743
SOX18	-2.4309952
SOX4	-1.5124056
SOX5IT	-0.5634489
SOX6	-1.7559474
SP1	-0.6096301
SP3OS	-0.3430638
SP4	-0.5594645
SPA17	-1.1839781
SPACA1	-0.4799895
SPAG5	-1.2906561
SPARC	-0.4201574
SPARCL1	-0.323266
SPATA24	-1.0529637
SPATA6	-0.3320165
SPC24	-1.1863694
SPC25	-0.5704393
SPDL1	-0.995995
SPECC1	-0.5837021
SPECC1L	-0.2271137

SPEER4C	-0.8340125
SPEG	-0.948838
SPG21	-0.3043418
SPHK1	-0.6330981
SPIN2C	-0.9195461
SPINK2	-2.3582902
SPINK7	-0.8399794
SPINT1	-1.0451059
SPIRE1	-1.738091
SPN	-0.6253371
SPNS2	-0.952539
SPNS3	-1.0707073
SPON2	-3.5474305
SPOP	-0.2543416
SPOPL	-0.2844787
SPPL2B	-0.9992609
SPPL3	-0.6534843
SPR	-0.8161669
SPRY4	-0.5819664
SPRYD3	-0.8231049
SPRYD4	-0.5414815
SPSB2	-0.3833075
SPSB3	-0.7198954
SPTA1	-1.2141647
SPTB	-0.6905453
SPTBN5	-0.2854478
SPTSSA	-0.2007074
SQLE	-0.4129653
SRBD1	-0.3141079
SRCRB4D	-0.2912183
SRD5A1	-1.2672768
SRD5A3	-0.3702498
SREBF1	-0.4652722
SREBF2	-0.477468
SREK1IP1	-0.5719705
SRGAP2	-0.2480736
SRGAP3	-1.2308371
SRP9	-0.4378834
SRPK3	-0.3233366
SRPX	-0.4829347
SRPX2	-0.7485142
SRR	-0.7601404
SRRD	-0.4554629
SRRM1	-0.3200154
SRSF1	-0.4434257

SRSF10	-0.3193107
SRSF2	-0.8368082
SRSF3	-0.3858089
SRSF9	-0.5564523
SRXN1	-0.8712339
SSBP2	-0.6606197
SSBP3	-1.0224519
SSBP4	-0.7711129
SSH1	-0.5662191
SSNA1	-1.2777758
SSPN	-1.3200188
SSR4	-0.2652259
SSRP1	-0.3701139
SSSCA1	-0.2408133
SSX2IP	-1.3832054
SSXB6	-0.8931925
ST18	-1.3743277
ST3GAL2	-0.5122814
ST3GAL3	-0.3296089
ST3GAL5	-1.1711078
ST3GAL6	-0.9634066
ST5	-1.6343026
ST6GAL2	-0.330595
ST6GALNAC5	-0.7907214
ST6GALNAC6	-0.4938459
ST7	-0.4242959
STAB2	-1.6944394
STAC2	-0.6239288
STAG1	-0.5536914
STAMBPL1	-0.3234363
STAR	-1.3129072
STARD10	-2.0743442
STARD3NL	-0.3395996
STARD8	-2.28456
STAU1	-0.4121189
STBD1	-0.5903029
STEAP3	-1.6255903
STIL	-1.0031834
STK11	-0.71311
STK16	-0.8234086
STK25	-0.8478456
STK3	-0.5531182
STK32C	-1.5610638
STK38L	-0.2672615
STMN1	-1.0562091

STMN2	-1.7706404
STOM	-1.8391862
STOML2	-0.386199
STON2	-0.7612445
STOX1	-1.4888673
STRA13	-0.7056212
STRADB	-0.9312453
STRAP	-0.5447426
STRBP	-0.6414175
STRN	-0.4219022
STRN4	-0.2979722
STS	-0.8550711
STT3A	-0.5215955
STUB1	-0.5421629
STX17	-0.8713241
STX2	-1.3010669
STX3	-1.3121808
STXBP2	-0.3329434
STXBP4	-0.2542782
STXBP6	-1.4000325
SUCLA2	-0.2852941
SUCLG1	-0.4105034
SUCLG2	-0.3443818
SUCNR1	-0.2883182
SUDS3	-1.077558
SUGP1	-0.3840876
SULF2	-1.0407162
SULT1A1	-0.4187889
SULT5A1	-0.7212415
SUMF1	-0.5069599
SUMF2	-0.6391842
SUMO3	-0.3800035
SUN1	-0.6072836
SUOX	-0.8959441
SUPT16	-1.4059854
SUPT7L	-1.5417168
SURF1	-0.2884836
SUV39H2	-0.3276672
SUV420H2	-0.8856597
SUZ12	-0.3797708
SVEP1	-0.9698682
SVIP	-1.8546572
SWAP70	-0.4069457
SWSAP1	-0.4531815
SYCE1	-1.9744396

SYCE2	-1.2127867
SYCP1-PS1	-3.324686
SYDE2	-0.6501217
SYNC	-1.338078
SYNE1	-1.4291222
SYNE3	-1.8728857
SYNGR1	-1.6986673
SYNGR4	-1.333503
SYNJ2	-0.370012
SYNM	-1.7279105
SYNPO2	-0.2609327
SYNRG	-0.2688551
SYS1	-0.2596726
SYT15	-1.8702707
SYT3	-0.8101831
SYT5	-0.9377196
SYTL1	-1.5148599
SYTL2	-1.0991182
SYTL4	-0.6060009
TAAR3	-10.081129
TAB1	-0.9211865
TAB3	-0.8282819
TAC2	-0.8574271
TAC4	-1.7557607
TACC1	-0.6671362
TACC2	-0.8059924
TACC3	-0.9738007
TAF10	-0.4417038
TAF11	-0.3554707
TAF12	-0.4780006
TAF15	-1.4430256
TAF1C	-0.3553834
TAF2	-0.3406844
TAF3	-0.2582703
TAF5	-0.7735825
TAF5L	-0.5463882
TAF6L	-0.8148379
TAF9	-0.8887296
TAF9B	-0.6137776
TAL1	-1.3533163
TALDO1	-0.6934643
TAMM41	-0.7840505
TANGO2	-0.8148489
TAOK2	-0.382194
TAOK3	-0.7767191

TAPT1	-1.0776186
TARSL2	-0.9844756
TAS2R109	-0.3049769
TATDN2	-1.8213778
TATDN3	-0.8909922
TBC1D10B	-0.1762753
TBC1D12	-0.7114899
TBC1D13	-0.518693
TBC1D16	-0.2478433
TBC1D17	-0.238379
TBC1D22A	-0.5227337
TBC1D24	-1.4378712
TBC1D31	-0.8157263
TBC1D5	-0.2461929
TBC1D7	-1.05826
TBCCD1	-0.6588993
TBCD	-0.6920881
TBCEL	-0.9641342
TBKBP1	-0.2377462
TBL1XR1	-0.4526167
TBRG4	-0.2980228
TBX2	-1.9497604
TBXA2R	-1.3308883
TBXAS1	-1.8658981
TCEA1	-0.4499879
TCEA3	-2.1018507
TCEAL1	-2.1839907
TCEAL3	-0.6709778
TCEAL5	-1.6952281
TCEAL6	-0.7394085
TCEAL7	-0.5815222
TCEAL8	-1.1215239
TCEB2	-0.2868762
TCEB3	-0.6803865
TCERG1	-0.5424867
TCF15	-1.7305562
TCF19	-1.0354886
TCF20	-0.3547549
TCF21	-3.1142938
TCF23	-1.6455247
TCF3	-1.178113
TCF7L1	-1.8855784
TCF7L2	-0.6431932
TCHH	-2.550925
TCN2	-0.1959906

TCP1	-0.4628439
TCP11L2	-0.9377909
TCTA	-0.6869772
TCTN1	-0.493845
TCTN2	-0.8902471
TDP1	-0.3878903
TDP2	-0.3646579
TEAD3	-0.2772646
TEC	-0.286778
TECPR2	-0.6870627
TECR	-0.9130235
TEF	-0.497879
TEK	-2.3542206
TEKT5	-0.7268195
TEN1	-0.3642321
TENC1	-0.7480476
TEP1	-0.3626285
TESC	-0.3430691
TESCL	-1.2998483
TESK2	-0.4169915
TEX14	-1.7686479
TEX15	-0.9706252
TEX40	-1.3057983
TFB1M	-0.6074753
TFCP2	-0.4967892
TFDP1	-1.1505814
TFDP2	-1.1132011
TFEB	-0.464097
TFF1	-2.2189822
TFIP11	-0.3021784
TFPI2	-1.2322176
TFPT	-0.6915598
TFR2	-1.3248348
TFRC	-1.5345249
TGFB1	-0.6595635
TGFB1I1	-0.7183948
TGFB2	-1.1781433
TGFB3	-0.4442935
TGFBI	-1.4635239
TGFBR3	-0.7246618
TGFBRAP1	-0.6913035
TGM1	-0.183959
TGM3	-0.8194361
THAP11	-0.6316037
THAP4	-0.2864242

THAP6	-0.4024494
THAP7	-0.2956853
THBD	-0.7864704
THBS2	-1.096142
THEM6	-1.3304842
THNSL2	-1.4410815
THOC2	-0.2090178
THOC6	-0.514081
THOC7	-0.5301981
THOP1	-0.3937755
THRA	-1.5435147
THRAP3	-0.3552852
THRB	-1.2456431
THRSP	-4.5313473
THSD1	-2.7705085
THSD7B	-1.1475222
THTPA	-0.5790982
THY1	-0.7955852
TIAL1	-0.2452889
TIAM1	-0.9851229
TIAM2	-1.9947782
TICRR	-0.8497734
TIE1	-2.0741858
TIFAB	-2.9765742
TIGD3	-1.8611937
TIGD5	-1.3214102
TIMD4	-0.960568
TIMELESS	-0.9293132
TIMM13	-0.5773344
TIMM17B	-0.2906232
TIMM21	-0.3507819
TIMM22	-0.2348576
TIMM44	-0.3833556
TIMM50	-0.5077601
TIMM8A1	-0.2412109
TIMM8B	-0.6400824
TIMP2	-0.9463034
TIPIN	-1.3103771
TIPRL	-0.4786739
TJP1	-0.7136219
TJP3	-0.3265243
TK1	-1.4824214
TKT	-0.4999895
TKTL1	-1.2216885
TLCD1	-1.4334688

TLCD2	-0.7662878
TLDC1	-0.351634
TLE2	-0.824543
TLE6	-0.819222
TLK1	-0.6592093
TLR11	-1.2878277
TLR12	-0.9472823
TLR13	-0.423409
TLR4	-0.3496447
TLR8	-1.5728579
TLX1	-2.7466238
TM2D2	-0.2709975
TM6SF1	-0.3586779
TM7SF3	-0.6300764
TM9SF1	-0.200171
TMBIM4	-0.3456545
TMBIM6	-0.5409184
TMC4	-0.517066
TMC6	-1.006083
TMC8	-0.5370469
TMCC2	-1.1612248
TMCC3	-0.4206152
TMCO1	-0.4260092
TMCO6	-0.4188228
TMED1	-0.4645653
TMED2	-0.526557
TMED3	-0.3056831
TMED4	-0.4887548
TMEM100	-1.0341306
TMEM101	-0.3436675
TMEM104	-0.9557467
TMEM106A	-0.2866488
TMEM106B	-0.2386704
TMEM106C	-1.0757852
TMEM107	-0.8963804
TMEM109	-0.4138489
TMEM110	-0.2013054
TMEM115	-0.4523592
TMEM120B	-1.1014252
TMEM129	-0.5283799
TMEM132A	-1.4036493
TMEM134	-0.7686629
TMEM136	-1.4017313
TMEM138	-1.1244533
TMEM141	-1.6348958

TMEM144	-1.3724704
TMEM147	-0.4257336
TMEM14A	-0.3126791
TMEM14C	-1.331522
TMEM150B	-1.3279662
TMEM154	-0.4770124
TMEM160	-0.6287775
TMEM164	-0.3186278
TMEM165	-0.2823668
TMEM170	-0.5434165
TMEM175	-0.292069
TMEM178	-0.2764144
TMEM179B	-0.4573569
TMEM18	-0.3528438
TMEM180	-0.9839387
TMEM183A	-0.8156915
TMEM184A	-0.4804974
TMEM186	-0.454545
TMEM19	-0.2801504
TMEM191C	-0.8050904
TMEM194	-0.4713569
TMEM198B	-0.6191802
TMEM200A	-2.2412481
TMEM201	-0.3357582
TMEM203	-0.7677374
TMEM204	-1.4691799
TMEM205	-0.8292651
TMEM216	-0.9023657
TMEM218	-0.9304082
TMEM219	-0.3347321
TMEM222	-0.3343024
TMEM223	-0.3472323
TMEM229A	-1.1360679
TMEM234	-0.6778154
TMEM238	-0.8361642
TMEM241	-0.2892647
TMEM242	-0.6553602
TMEM246	-1.2871046
TMEM25	-0.7255349
TMEM254A	-0.2893577
TMEM254C	-0.328176
TMEM255A	-1.7869041
TMEM256	-1.3547306
TMEM259	-0.6197572
TMEM26	-1.1723473

TMEM261	-0.3455544
TMEM29	-0.4695082
TMEM35	-2.746441
TMEM37	-2.1398087
TMEM38A	-0.9000244
TMEM39B	-0.2878537
TMEM40	-1.1608071
TMEM41A	-0.6760631
TMEM41B	-0.5058761
TMEM43	-0.6476088
TMEM44	-1.6202321
TMEM50A	-0.5862336
TMEM51	-0.8965883
TMEM53	-1.1599741
TMEM55A	-0.569356
TMEM56	-2.150244
TMEM57	-0.6915913
TMEM59	-0.2274418
TMEM60	-0.7374754
TMEM63A	-0.4542041
TMEM65	-0.3586447
TMEM8	-1.8369799
TMEM86A	-2.827821
TMEM86B	-1.3255842
TMEM88	-1.5166774
TMEM9	-1.177659
TMEM97	-0.7132225
TMEM98	-1.933114
TMLHE	-0.6877096
TMOD1	-1.7920427
TMOD3	-0.3645825
TMPO	-0.6026201
TMPRSS4	-1.1983066
TMPRSS6	-1.5485413
TMTC3	-0.4049337
TMUB2	-0.9516568
TMX1	-0.6232925
TMX2	-0.6028171
TMX4	-1.1737671
TNFAIP8L2	-0.4549661
TNFRSF14	-0.3087316
TNFRSF17	-0.5914836
TNFRSF19	-1.0893748
TNFRSF1A	-0.2380071
TNFSF12	-0.3223906

TNFSF12TNF!	-1.1770015
TNFSF13B	-0.4890971
TNFSF13OS	-4.101684
TNIK	-0.5159206
TNK1	-1.1632924
TNK2	-0.7512498
TNNI2	-0.3495393
TNNI3	-0.8292084
TNNT2	-0.8722837
TNNT3	-0.58024
TNPO2	-0.7747469
TNRC6B	-0.772418
TNS3	-0.3424692
TNXB	-2.6298966
TOB1	-1.436003
TOB2	-0.7432036
TOLLIP	-0.5118837
TOM1	-0.290617
TOM1L1	-0.6878181
TOM1L2	-0.5294738
TOMM22	-0.6695981
TOMM34	-0.2311158
TOMM40L	-0.2410169
TONSL	-1.2102089
TOP2A	-1.0298505
TOP2B	-0.2307096
TOP3B	-0.2175102
TOPBP1	-0.7450867
TOR1A	-0.3749538
TOR4A	-0.1921039
TPBG	-1.5087137
TPCN1	-1.9239221
TPD52L2	-0.4465604
TPGS2	-0.4436145
TPI1	-0.6142211
TPM1	-0.6496201
TPM2	-0.2994962
TPMT	-1.4912698
TPP1	-0.7306881
TPPP	-3.595818
TPPP3	-2.4562547
TPRA1	-0.8623486
TPRGL	-0.5275822
TPRN	-0.598937
TPX2	-0.9811268

TRABD	-0.5282993
TRAF7	-0.3717508
TRAK2	-1.3320017
TRAM1	-0.6308799
TRAP1	-0.5885196
TRAPPC1	-0.5070233
TRAPPC10	-0.6327863
TRAPPC2L	-0.3088517
TRAPPC9	-0.531332
TREM2	-3.030385
TREML1	-0.5562048
TRERF1	-0.4605541
TREX2	-2.4063182
TRF	-1.5639653
TRHR2	-0.8576338
TRIAP1	-0.6907678
TRIL	-2.3124173
TRIM10	-1.3388133
TRIM16	-0.6978829
TRIM2	-1.1682467
TRIM23	-0.865731
TRIM24	-0.4492474
TRIM28	-0.4769316
TRIM37	-0.5762034
TRIM40	-1.1019382
TRIM45	-0.5577216
TRIM58	-0.8276253
TRIM59	-0.7417703
TRIM8	-0.4311376
TRIO	-0.1921835
TRIP12	-0.3315177
TRIP13	-0.674283
TRIP4	-0.2761827
TRIP6	-0.3255639
TRMT11	-0.5842805
TRMT112	-0.2380233
TRMT12	-0.6468673
TRO	-1.1476631
TROAP	-1.3298781
TROVE2	-0.2698028
TRP53	-0.6132464
TRPC6	-0.6284232
TRPM6	-0.2516031
TRPM8	-0.4640007
TRUB2	-0.2998405

TSC1	-0.2336726
TSC22D1	-1.5752954
TSC22D4	-0.4764414
TSEN15	-0.4385905
TSEN2	-0.1773286
TSEN34	-0.3008514
TSFM	-0.4811788
TSHZ1	-0.3763261
TSHZ2	-0.9545064
TSPAN12	-1.0565085
TSPAN15	-1.3185632
TSPAN17	-1.1108012
TSPAN18	-1.7164323
TSPAN2	-1.0350671
TSPAN33	-0.973053
TSPAN4	-0.3684025
TSPAN7	-1.0172682
TSPAN8	-1.3724289
TSPO2	-1.1340156
TSSC4	-0.5839343
TSSK5	-0.593535
TSSK6	-0.3046017
TST	-1.7889204
TSTA3	-0.4071965
TSTD3	-1.1538248
TTC1	-0.363966
TTC12	-0.8293765
TTC23	-0.607898
TTC28	-0.2251186
TTC3	-0.2770772
TTC30A2	-1.1454828
TTC30B	-1.7168646
TTC34	-0.4812999
TTC39A	-1.1593854
TTC39C	-0.2515638
TTC7	-0.204514
TTC9	-0.3945727
TTK	-1.6764073
TTLL1	-1.0710914
TTLL12	-1.3684602
TTLL5	-0.8425989
TTYH2	-0.8534775
TTYH3	-0.2614965
TUBA1B	-0.6649756
TUBA1C	-0.7357702

TUBA4A	-1.1211853
TUBA8	-0.4896541
TUBB1	-1.0404463
TUBB2A	-0.270277
TUBB2A-PS2	-0.3954125
TUBB4A	-0.6789484
TUBB4B	-0.6529656
TUBB5	-0.4745498
TUBD1	-0.2866454
TUBE1	-0.3055868
TUBG1	-1.1926494
TUBGCP2	-1.0219402
TUBGCP3	-0.35602
TUFT1	-0.9021606
TUG1	-0.2980075
TULP2	-0.3109956
TUSC1	-1.2666249
TUSC2	-0.5815525
TUSC3	-0.6824627
TVP23A	-1.8711147
TWF2	-0.3862252
TWIST1	-1.703954
TWSG1	-0.6094899
TXLNA	-0.2509003
TXN2	-0.2072015
TXNDC5	-0.6223998
TXNIP	-0.5121689
TXNRD2	-1.0363455
TYMS	-0.3763242
TYMS-PS	-1.1202455
TYRO3	-0.3524559
TYROBP	-0.4591227
TYRP1	-8.287495
U2AF1	-0.4025846
U2AF1L4	-0.889132
U2AF2	-0.5402041
U2SURP	-0.4743938
UACA	-0.5824103
UAP1L1	-0.5438757
UBA2	-0.1976948
UBAC1	-1.4173031
UBAP1	-0.6116705
UBAP2	-0.3138032
UBAP2L	-0.4608545
UBE2C	-1.3001075

UBE2E3	-1.088697
UBE2H	-0.4189959
UBE2L6	-0.731781
UBE2M	-0.9294033
UBE2O	-1.1226168
UBE2R2	-0.5321441
UBE2S	-1.6330972
UBE2T	-0.9266944
UBE2V1	-0.4775167
UBE3A	-0.266551
UBE3B	-0.4779778
UBE4A	-0.4576945
UBE4B	-0.4505177
UBIAD1	-2.583016
UBL4	-0.3208127
UBL5	-0.3272071
UBL7	-0.7211771
UBQLN4	-0.5426636
UBR1	-0.221406
UBR7	-0.4692841
UBTD1	-0.2306466
UBXN1	-0.6203771
UBXN2B	-0.7975769
UBXN6	-0.335814
UBXN8	-0.5009399
UCHL5	-0.1897931
UCP2	-1.0913386
UCP3	-1.2908449
UEVLD	-1.1334884
UFSP1	-1.3787012
UGCG	-0.4483852
UGP2	-0.3047705
UGT1A6B	-0.6271448
UHRF1	-0.9830875
UHRF1BP1	-0.3643112
UHRF2	-0.1920023
ULK2	-0.3536654
UNC119	-0.7809634
UNC119B	-0.4354005
UNC13D	-0.2220988
UNC50	-0.373939
UNC5B	-1.2953966
UNC5CL	-0.2812829
UNC93B1	-0.2619534
UPB1	-0.7762172

UPF1	-1.2396622
UPF2	-0.4974194
UPK1B	-0.7755418
UPK3A	-2.3903003
UPK3B	-1.9744482
UQCC2	-0.380723
UQCR10	-0.3053207
UQCR11	-0.6657829
UQCRC1	-1.0921564
UQCRH	-0.5658989
URB2	-0.4310184
URGCP	-0.4801102
URI1	-0.7078662
URM1	-0.437767
UROD	-1.2306962
UROS	-1.0629416
USF2	-0.4943986
USHBP1	-0.994431
USMG5	-0.319036
USP1	-0.3522363
USP14	-0.3989921
USP15	-0.6354446
USP19	-0.4100809
USP20	-0.4831886
USP21	-0.679028
USP25	-0.3046808
USP32	-0.3058629
USP33	-0.457973
USP39	-0.6785231
USP45	-0.8676958
USP46	-1.3380461
USP5	-0.5919471
USP50	-0.1764031
USP51	-1.0398161
USP6NL	-0.5774539
USP7	-0.5791402
USP9X	-0.612535
UVSSA	-0.3173041
UXS1	-0.477165
VAC14	-0.4020491
VAMP2	-0.3373432
VAMP3	-0.2676461
VAMP8	-0.7088971
VANGL1	-0.9312449
VAPB	-0.7209334

VASN	-0.8719916
VAT1	-0.5838509
VAT1L	-0.9400606
VAV3	-0.6824346
VAX2	-0.6334758
VBP1	-0.7517657
VCAM1	-0.7154884
VCL	-0.8314209
VDAC1	-0.4512348
VDAC3	-0.3008003
VEGFA	-0.8008142
VEGFB	-1.1410198
VEGFC	-1.2073183
VEZT	-0.436513
VHL	-0.3457823
VILL	-0.8955183
VIM	-0.1835542
VIPAS39	-0.2242904
VIT	-2.0936303
VKORC1	-0.7761364
VKORC1L1	-0.6920829
VMAC	-1.6937513
VMN1R204	-0.8708174
VMN1R219	-1.8803155
VMN1R56	-0.4483516
VMN1R74	-3.0362504
VMN2R114	-1.125278
VOPP1	-0.7583084
VPREB1	-1.8102431
VPS11	-0.3120413
VPS13C	-0.5672655
VPS13D	-0.2144094
VPS18	-0.2302175
VPS25	-0.6447601
VPS26B	-0.581471
VPS35	-0.685564
VPS37D	-0.7052681
VPS41	-0.5999665
VPS45	-0.454679
VPS4A	-0.4178233
VPS51	-0.5179682
VPS8	-0.4217448
VRK1	-1.176712
VRK2	-0.7666125
VSIG4	-3.4849415

VSTM4	-1.9667642
VSTM5	-1.378243
VTI1A	-0.4412279
VWA1	-0.2522435
VWA3A	-4.420879
VWA9	-0.5282993
VWCE	-2.4559307
VWDE	-0.8962324
VWF	-0.9784155
WAC	-0.2732711
WAPAL	-0.2442613
WAS	-0.6120701
WASF1	-0.1738453
WBP1	-0.9243622
WBP2	-0.5288544
WBSCR16	-0.5549831
WBSCR27	-0.9325333
WDPCP	-0.9943936
WDR18	-0.3166132
WDR24	-0.5279579
WDR26	-0.924963
WDR31	-0.2401042
WDR33	-0.3163748
WDR35	-0.867636
WDR45	-0.2237382
WDR6	-1.0165682
WDR60	-0.8556628
WDR61	-0.363204
WDR62	-1.4948139
WDR72	-2.2857828
WDR73	-0.2162814
WDR76	-0.9219959
WDR81	-0.5510254
WDR83	-0.5442405
WDR83OS	-0.4236288
WDR86	-0.6316676
WDR90	-1.1372752
WDR95	-0.718262
WDSUB1	-0.6411157
WDTC1	-0.4497185
WDYHV1	-0.6954541
WEE1	-0.8420682
WFDC1	-1.1972542
WFDC10	-2.6472878
WFIKKN1	-0.3586175

WHAMM	-0.3585763
WHSC1	-0.2346067
WIBG	-0.3464768
WIPF2	-0.338347
WIPF3	-1.7256176
WIPI1	-1.4997182
WIPI2	-0.7975373
WISP2	-2.2145426
WLS	-0.6960578
WNK4	-1.3876185
WNT4	-1.0829403
WNT5A	-0.9298928
WNT5B	-0.5128563
WRAP53	-0.3965893
WRN	-1.1840601
WWC2	-0.4701777
WWP1	-0.9877815
WWP2	-0.4490957
WWTR1	-0.4434338
XAB2	-0.4914594
XCR1	-2.2377143
XIRP1	-0.6783271
XK	-1.5012622
XKR5	-1.2753565
XKRX	-0.3543892
XLR	-0.6568062
XNDC1	-0.9048088
XPC	-0.9727039
XPNPEP1	-0.5903153
XPNPEP2	-8.707479
XPO1	-0.4434991
XPO4	-0.4333534
XPO5	-0.2623105
XPO7	-0.7489386
XPR1	-0.5386715
XRCC1	-0.3708391
XRCC2	-1.6771381
XRCC3	-1.4491358
XRCC5	-0.3115807
XRCC6	-0.4656692
XYLT2	-0.314764
YARS2	-0.345006
YBX2	-0.2947776
YBX3	-0.4393354
YIF1A	-0.5369601

YIF1B	-0.5312223
YIPF2	-0.4352326
YIPF3	-0.4973049
YOD1	-1.3145943
YPEL2	-1.215364
YPEL3	-1.1900024
YPEL4	-1.0271792
YPEL5	-0.9949694
YTHDC2	-0.3022242
YWHAB	-0.4469295
ZADH2	-0.5667749
ZAK	-0.4376452
ZBED4	-0.3821416
ZBTB1	-0.2181525
ZBTB14	-0.4123936
ZBTB22	-0.4827371
ZBTB34	-0.651154
ZBTB42	-0.5892484
ZBTB44	-0.4236302
ZBTB45	-1.2274652
ZBTB49	-0.2495191
ZBTB7A	-0.3752756
ZBTB8A	-1.4816935
ZBTBD6	-0.7220731
ZC3H14	-0.5050783
ZC3H6	-0.3378205
ZC3H7B	-0.3670979
ZC3HC1	-0.4085193
ZCCHC14	-0.5994875
ZCCHC24	-0.4278517
ZCCHC3	-1.4868159
ZDHHC1	-0.898603
ZDHHC12	-0.343791
ZDHHC14	-0.8925538
ZDHHC20	-0.6269872
ZDHHC22	-0.8253903
ZDHHC24	-0.8064237
ZDHHC3	-0.4859295
ZDHHC4	-0.8575752
ZDHHC7	-0.6226702
ZER1	-0.3493042
ZFAND2B	-0.578547
ZFAND4	-1.5049243
ZFAND6	-0.8225675
ZFP101	-1.0898438

ZFP113	-0.4472435
ZFP142	-0.1756399
ZFP143	-0.4412067
ZFP180	-0.2975512
ZFP184	-0.3829095
ZFP185	-2.1425326
ZFP207	-0.3105555
ZFP212	-0.4407587
ZFP219	-0.9890287
ZFP229	-0.825681
ZFP3	-0.5065334
ZFP30	-0.9298194
ZFP316	-0.4809737
ZFP322A	-0.192349
ZFP335OS	-0.7817483
ZFP345	-0.2627716
ZFP346	-0.7243092
ZFP35	-0.4399071
ZFP354B	-0.9100971
ZFP354C	-0.4262793
ZFP358	-0.4006238
ZFP367	-0.8807845
ZFP385A	-0.6130581
ZFP385C	-1.6967814
ZFP39	-1.2585697
ZFP395	-1.2806473
ZFP41	-0.5169447
ZFP410	-0.561945
ZFP414	-0.763216
ZFP423	-0.2524242
ZFP467	-1.1502688
ZFP503	-1.5467975
ZFP518B	-0.8971882
ZFP521	-2.242608
ZFP524	-0.6108875
ZFP526	-0.5113061
ZFP534	-0.6922884
ZFP580	-1.021029
ZFP608	-0.3372912
ZFP618	-1.4066603
ZFP619	-0.2484009
ZFP623	-0.4051652
ZFP629	-0.729773
ZFP639	-0.4636574
ZFP644	-0.1930952

ZFP646	-0.5013459
ZFP652	-0.3809524
ZFP668	-0.281261
ZFP672	-0.1922822
ZFP688	-0.6863585
ZFP703	-0.4734879
ZFP704	-2.254403
ZFP710	-0.2844396
ZFP760	-0.8776147
ZFP770	-0.3581562
ZFP771	-0.4644942
ZFP787	-0.4531183
ZFP808	-1.0503354
ZFP820	-1.1114988
ZFP821	-0.4329672
ZFP846	-0.3977523
ZFP871	-0.3879347
ZFP90	-0.4269385
ZFP91	-0.4053891
ZFP93	-0.4950035
ZFP931	-0.3745318
ZFP934	-0.632648
ZFP936	-0.3610334
ZFP949	-1.0319295
ZFP955A	-0.3462653
ZFP957	-0.4123766
ZFPM1	-1.1365681
ZFPM2	-0.7631791
ZFR	-0.415005
ZFYVE1	-0.7460127
ZFYVE19	-0.3035483
ZFYVE21	-1.4642658
ZGRF1	-0.5204177
ZHX1	-0.5304365
ZKSCAN14	-0.2903166
ZKSCAN4	-2.4073813
ZKSCAN5	-0.4885593
ZMAT2	-0.322537
ZMAT5	-0.3042502
ZMPSTE24	-0.4555807
ZMYM3	-0.2791324
ZMYND19	-0.6962495
ZNF512B	-0.5276108
ZNHIT2	-0.579371
ZNHIT3	-0.5494027

ZNRD1AS	-0.4181595
ZRANB1	-0.6273794
ZRSR1	-0.7267571
ZSCAN2	-0.700233
ZSWIM3	-0.9086254
ZW10	-0.6149001
ZWILCH	-0.8967967
ZYX	-0.6396265
A1CF	0.8669009
A3GALT2	1.1490943
AAK1	0.3239212
AASDH	0.2903266
ABCA1	0.42218018
ABCA13	0.399817
ABCA4	2.04045
ABCB1A	1.3708797
ABCB1B	0.85404444
ABCC1	0.66200686
ABHD17B	0.3908224
ABI1	0.28576326
ABI3	0.23744726
ABL2	0.43784714
ABLIM1	0.70534134
ABLIM3	0.49521065
ABR	0.29538298
ABTB2	1.9015112
ACAA1A	0.2643323
ACADSB	0.33383465
ACE2	0.5566695
ACER1	0.81341076
ACER2	1.0439174
ACKR2	0.6931968
ACOT5	0.8213937
ACOT7	0.23785591
ACP2	0.46525192
ACP5	0.65187216
ACPP	0.5206299
ACSBG2	0.5566337
ACSF2	0.63836765
ACSL4	0.22867012
ACSM1	0.78390884
ACTG2	0.82288265
ACTR3	0.33517027
ACVR1B	0.96345997
ADAD1	0.49026775

ADAM17	0.51837087
ADAM18	1.002686
ADAM6B	1.1384952
ADAM8	0.668561
ADAM9	1.0619955
ADAMTS1	1.5206666
ADAMTS14	0.6165323
ADAMTS18	0.7365384
ADAMTS4	5.7458086
ADAMTS8	0.29608822
ADAMTS9	2.6031895
ADAP1	0.2869296
ADAR	0.9148288
ADARB1	0.6452575
ADAT1	0.22131634
ADCK1	0.29227924
ADCY4	0.64289045
ADCYAP1	0.9151926
ADHFE1	0.51580167
ADM	2.9909878
ADORA2A	0.81775284
ADORA2B	1.6038871
ADPRM	0.62859535
ADRA2A	0.70691514
ADRBK2	0.22569227
ADTRP	1.3243604
AEBP2	0.72563124
AEN	0.6237688
AF067061	0.7220278
AFF3	0.8430929
AFP	1.0799909
AFTPH	0.24226332
AGBL4	1.0972404
AGK	0.22969627
AGMAT	0.61059284
AGPAT6	0.26611996
AGRN	0.49115038
AGRP	0.5703745
AHSA1	0.45115805
AHSA2	0.8878503
AICDA	1.788022
AIF1L	1.0520363
AIM1	0.61839104
AIM2	0.50244904
AK2	0.18248844

AK4	1.8192434
AKAP12	1.7254028
AKAP13	0.29281187
AKAP2	2.1012087
AKAP4	0.45289373
AKAP8	0.32303238
AKAP9	0.65987444
AKNA	0.56460476
AKP3	1.2430906
AKT3	0.24532461
ALDH1A2	1.0277
ALDH7A1	0.63282204
ALDH8A1	0.59224725
ALDOB	1.7898695
ALG1	0.41820145
ALG11	0.8437705
ALG12	0.24478245
ALG8	1.5539742
ALKBH1	0.6952691
ALKBH6	0.45415735
ALLC	0.98006487
ALPK1	1.1812987
ALPK2	0.84418416
ALPK3	0.7685778
ALPL	1.1219263
ALS2CL	0.5067625
AMACR	0.21464348
AMBP	2.4550974
AMER2	1.1012571
AMICA1	0.8566408
AMMECR1L	0.25661802
AMN1	0.7920985
AMOTL1	0.81910396
AMT	1.8090026
AMY1	8.933637
AMY2A5	10.682104
ANG	1.2975545
ANG2	1.080035
ANG3	1.2640607
ANG4	1.83077
ANGEL1	0.5770407
ANGPT2	1.7490444
ANGPTL1	0.7817974
ANKLE2	0.31977367
ANKRA2	0.51231813

ANKRD12	0.35348845
ANKRD16	0.29847956
ANKRD22	1.4497633
ANKRD23	0.5338087
ANKRD33	0.7991991
ANKRD33B	1.293405
ANKRD34B	0.8244705
ANKRD37	0.27302217
ANKRD52	0.34333086
ANKRD66	1.6590672
ANTXR2	0.38049507
ANXA2	0.37916756
ANXA3	0.24383068
ANXA4	0.3661337
AOC2	0.321002
AOC3	3.119223
AP1G1	0.4368949
AP1M2	0.52628183
AP1S3	0.3126812
AP3B1	0.3074956
AP4E1	1.4080715
AP5Z1	0.66732407
APBB3	0.511075
APEX2	0.31138706
APH1B	0.24391937
APOA2	1.2984838
APOBEC1	1.1957827
APOC3	0.4038856
APOD	0.94364667
APOL7C	0.6935096
APOLD1	1.608952
APPBP2	0.21443653
APPL1	0.45333767
AQP12	2.234786
AQP3	0.6640334
AQP4	0.9668882
AQP8	1.2737021
ARAP2	1.4470863
AREG	2.9152014
ARG2	1.5405307
ARHGAP20OS	1.9350824
ARHGAP22	1.7365999
ARHGAP27	0.43564367
ARHGAP30	0.8427048
ARHGAP31	0.47335958

ARHGAP33O!	0.8171365
ARHGAP8	1.5221047
ARHGEF18	0.36161423
ARHGEF19	0.66371155
ARHGEF38	0.86949396
ARID3A	0.94613266
ARID4B	0.7621367
ARID5A	1.9361796
ARID5B	0.59373903
ARL1	0.34607935
ARL13B	0.43302917
ARL14EP	0.6131892
ARL5B	0.66397667
ARL5C	1.2303071
ARL9	0.55721736
ARMC12	1.1530497
ARMCX2	0.64796257
ARPC1B	0.6961031
ARPC2	0.1843276
ARRDC4	0.7540288
ARRDC5	0.34976768
ARSA	0.22167397
ARSJ	1.8047252
ART2A-PS	0.44674015
ARV1	0.37481785
ARXES2	0.3354144
ASAP3	1.2133412
ASB10	0.63561773
ASB13	0.94354343
ASB4	2.0821524
ASCC3	0.3526187
ASCL1	0.7848308
ASH1L	0.5184431
ASIC3	0.5613792
ASIC4	0.65601254
ASIC5	1.399009
ASPHD1	1.1587014
ASPRV1	1.2596755
ASTE1	0.745811
ASTL	1.6065438
ASTX	0.9966743
ATAD1	0.2503481
ATAD3A	0.59560347
ATF3	1.8306861
ATF5	0.58951473

ATF6	0.21973658
ATG16L2	0.8205457
ATMIN	0.24996138
ATOH7	2.346559
ATP10A	0.7423806
ATP11A	1.1828985
ATP2A1	0.49739456
ATP6V0A2	0.3628521
ATP6V1G2	0.6817355
ATP7A	0.21508455
ATP8B4	0.21496391
ATP9B	0.6464138
ATRNL1	0.47647858
ATXN1L	0.523139
ATXN7L1OS2	0.7953265
AVPI1	0.81249714
AVPR1A	3.1798959
AVPR2	5.3302655
B3GALNT1	1.2882376
B3GALT5	1.1077776
B3GALT6	0.4453025
B3GAT1	0.9086764
B3GAT2	0.27390575
B3GNT2	0.44043016
B3GNT7	1.890636
B3GNTL1	0.20042562
B4GALT5	1.3138275
B9D1OS	0.8664031
BACH1	1.2099667
BAG3	0.42576647
BAG5	0.22192717
BANK1	0.31235886
BARHL1	0.49935174
BASP1	1.1139331
BATF	1.839428
BATF2	1.8773184
BAX	2.0428948
BAZ1A	0.37345982
BAZ2B	0.23494482
BCAR3	0.38033867
BCAS1	1.8596649
BCAS3OS2	0.42742014
BCAT1	1.2940612
BCDIN3D	0.24574566
BCKDHB	0.88318586

BCL11B	0.3948927
BCL2A1C	2.4746747
BCL2A1D	2.2620535
BCL2L11	0.67236257
BCL2L14	1.2742586
BCL3	2.5083609
BCL6	0.7524929
BCL6B	0.47641706
BCL9	1.4558625
BCR	1.0523667
BDKRB1	0.98528695
BDP1	0.31192732
BEND3	0.4505849
BEND4	0.21742392
BEX1	0.9409485
BEX2	1.9715011
BFAR	0.8008642
BFSP2	0.22245121
BHLHE40	0.5996504
BHMT	0.45696402
BIRC2	0.67487335
BIRC3	1.7918692
BLNK	1.0292406
BLOC1S6	0.23131275
BMP7	1.1134183
BMP8A	1.20294
BMPER	0.83058643
BMPR1B	0.9099252
BOLL	1.8718915
BOP1	0.31506872
BPIFB5	1.4002476
BPIFB6	2.583274
BPTF	0.20314598
BRD1	0.3221345
BRI3	0.2541132
BRICD5	0.2507131
BRIX1	0.27194977
BSND	0.9713981
BST1	1.2845159
BST2	1.5240335
BTBD16	0.63553953
BTBD19	0.43654394
BTBD8	0.58777523
BTBD9	0.25152063
BTG1	0.5996914

BTG3	0.34448576
BTK	0.85606337
BTLA	0.75589705
BZW2	0.22883654
C1RA	0.27487373
C2	0.42484283
C2CD4B	1.3838375
C3	0.33897066
C5AR1	1.3471262
CABLES1	0.57879305
CABP2	3.1404312
CABP4	0.55923843
CABP5	1.580451
CACNA1S	0.60127115
CACNB3	0.9284253
CACNG6	0.31261134
CACUL1	0.5558448
CALHM1	0.33333874
CAMK2N1	0.54488564
CAMK4	1.1173449
CAMKK1	0.3281417
CAMSAP1	0.272007
CAMTA1	1.9743655
CAMTA2	0.84701395
CANT1	0.24615335
CAPNS2	0.9638338
CAR12	1.1054156
CAR13	1.804841
CARD6	0.6339812
CARNS1	0.20661879
CARTPT	1.2735717
CASP1	0.335742
CASP16	1.5396774
CASP4	1.9992933
CASP8	0.2250328
CASZ1	0.27714944
CATIP	0.4687338
CATSPER1	1.4929333
CATSPER2	0.44254708
CATSPER4	1.3029351
CBFB	0.72570276
CBLB	0.43734598
CBS	1.8688157
CBX4	0.33646822
CC2D1B	0.2968936

CC2D2B	0.9346578
CCBE1	0.3549435
CCBL2	0.8254967
CCDC102A	0.4311695
CCDC11	1.7759421
CCDC114	0.5498078
CCDC115	0.5314684
CCDC13	0.42995167
CCDC163	0.26022005
CCDC169	0.87579703
CCDC177	1.2715623
CCDC183	0.98435116
CCDC184	0.8777137
CCDC33	0.83659434
CCDC42OS	1.8158078
CCDC50	0.78244925
CCDC59	0.32857132
CCDC62	0.51717806
CCDC64	0.32822037
CCDC66	0.4140768
CCDC78	4.858893
CCDC79	0.5938363
CCDC81	2.5879674
CCDC85A	1.2504897
CCDC86	0.4967556
CCDC88B	0.6668482
CCDC9	0.2932105
CCDC93	0.30687833
CCKAR	0.7321992
CCKBR	1.0437646
CCL1	1.0456791
CCL11	3.5423784
CCL12	3.931994
CCL17	3.5015655
CCL19	0.9074836
CCL2	5.819554
CCL25	0.5363841
CCL3	6.103713
CCL4	5.4779468
CCL5	0.49696016
CCL7	4.6872835
CCND2	0.78052616
CCNG2	0.27623463
CCNL1	0.34022474
CCNY	2.7571352

CCNYL1	0.9542928
CCR6	0.23641872
CCR7	1.4655972
CCR8	0.92099
CCRL2	2.0216956
CCSER2	0.5244436
CCT3	0.28413677
CD14	3.7933016
CD163L1	1.7561352
CD164	0.32704735
CD19	0.5951886
CD2	0.22718763
CD200	1.3166409
CD244	0.892683
CD247	0.33286548
CD274	1.7445025
CD300LF	0.88565874
CD302	0.8065386
CD33	1.1362553
CD38	1.2412539
CD3D	0.5978079
CD3E	0.6034322
CD40	1.3697929
CD44	1.435143
CD5	0.34613562
CD53	0.35348272
CD55	0.5503683
CD6	0.59400225
CD63	0.5093384
CD69	3.221467
CD70	1.3753386
CD72	0.57064486
CD83	1.3268585
CD86	0.50169086
CD97	0.20762444
CDC42EP2	1.4437413
CDH15	1.056706
CDH17	1.6016593
CDH2	0.48058844
CDH22	0.69418216
CDH4	0.62566185
CDH5	0.46611023
CDH6	2.2869387
CDK18	0.86310434
CDK5	0.3131175

CDK5R1	0.59495354
CDKL3	0.8093145
CDKN1A	1.0677052
CDKN2B	1.0847149
CDV3	0.25673962
CEACAM16	1.2007523
CEACAM18	1.2359831
CEACAM9	1.0603862
CEBPB	1.6610742
CEBPD	2.164606
CEL	8.703017
CELA1	3.4513607
CELA2A	9.057886
CELA3B	9.761423
CELSR1	0.46383142
CEMIP	0.9579022
CEP112OS2	1.2455986
CEP350	0.21299458
CEP85L	1.2502832
CEPT1	0.40971804
CERS3	1.5419362
CERS4	0.5169058
CERS5	0.35156345
CERS6	1.3139958
CES1G	0.6732378
CES1H	0.9824357
CES2B	0.85224223
CES2C	0.90745425
CES5A	0.82472014
CFB	1.4551697
CFLAR	1.3148656
CGA	1.7474618
CH25H	1.797615
CHAC1	0.6021538
CHD2	0.27175283
CHD3	0.34887123
CHD7	0.69517803
CHD8	0.33729982
CHEK1	1.5443735
CHGA	1.6957061
CHIC1	1.4627683
CHIC2	0.23345089
CHIL1	1.389247
CHIL5	1.2350969
CHIL6	2.3499222

CHL1	1.9200888
CHORDC1	0.55809736
CHPF	0.69776964
CHRM3	1.5226142
CHRM4	1.5060077
CHRNA9	0.7685051
CHRNB4	1.069269
CHST10	0.55035686
CHST11	0.7948389
CHST7	0.45572948
CIPC	0.8798857
CIR1	0.36391068
CISH	0.90105677
CK137956	1.5243785
CKMT1	1.4991622
CLCA1	0.25597048
CLCA2	0.23627043
CLCF1	0.6638222
CLCN5	0.6668327
CLDN12	0.33205962
CLDN14	1.0531723
CLDN23	0.30943465
CLDN25	0.9755964
CLDN26	1.3426337
CLDN3	1.3676932
CLEC12B	0.5985615
CLEC2D	1.2028956
CLEC2I	0.22589207
CLEC4A1	0.56737137
CLEC4A2	1.2262454
CLEC4D	2.1316385
CLEC4E	3.1272879
CLEC4F	1.5708575
CLEC4N	0.7174654
CLEC5A	3.0684366
CLIC1	0.9331727
CLK2	0.3325224
CLMP	0.8365481
CLN5	0.520833
CLPS	8.613361
CLRN3	0.2509215
CLSTN2	0.30157042
CLTB	0.39460182
CLU	0.29887486
CMA2	0.23810196

CMAH	0.35950184
CMPK2	1.0976906
CMTM6	0.5547118
CMTR1	0.570909
CMTR2	0.47662735
CMYA5	1.6750364
CNBD2	0.73999286
CNGA1	1.0917315
CNIH4	0.25236607
CNKSR3	2.0609608
CNN1	0.23317051
CNN3	0.4507947
CNOT6L	0.7092066
CNR2	0.3412404
CNTNAP5A	1.5010693
CNTRL	0.4639864
COBLL1	0.39996338
COG5	0.5086911
COL10A1	1.106226
COL11A2	0.70867276
COL20A1	0.54530954
COL22A1	3.112515
COL4A1	1.0486717
COL4A2	0.6321354
COL6A4	0.7717855
COL7A1	0.7219181
COL8A1	0.9891794
COMMD7	0.39743423
COPG2OS2	0.20113087
COPS7A	0.5894451
CORT	0.57751894
COX17	0.9592581
COX3	0.1887207
CP	2.1903424
CPA1	9.8069725
CPA2	8.89892
CPB1	11.927186
CPB2	1.4729047
CPHX1	0.70938873
CPLX2	1.4523535
CPNE9	1.1315486
CR2	1.3522501
CREB3	0.4756117
CREB3L1	0.23532963
CREB3L2	0.34491014

CREBBP	0.38971472
CREBL2	1.1286345
CREBZF	0.22847748
CRELD2	0.76067257
CREM	1.7445426
CRIM1	0.61815405
CRIP3	1.2547865
CRISPLD2	1.4883213
CRK	1.0127664
CRLF2	0.32533884
CRLF3	0.78129625
CRNDE	1.4927993
CRNN	1.7955062
CRTC2	0.6992049
CRY2	0.21734762
CRYAA	0.74818707
CRYBA1	1.1005244
CRYBB3	1.7948246
CRYGD	0.7120199
CRYGN	1.1327715
CSF1	0.7434416
CSF2	3.7690778
CSF2RA	0.5287905
CSF2RB	1.158124
CSF2RB2	1.0841818
CSF3	6.319416
CSF3R	0.6080637
CSMD2	1.1372819
CSNK1D	0.34192848
CSPRS	1.0888119
CSRNP1	1.9464216
CSRNP3	0.6559808
CST7	0.45980597
CST8	0.93694615
CSTB	0.45049858
CTGF	0.23427773
CTLA4	2.308488
CTNND1	0.6035881
CTPS	0.67397213
CTPS2	0.26802063
CTRB1	9.197462
CTRC	4.663919
CTRL	6.2564983
CTSO	0.40091372
CTSS	0.21703196

CTSZ	0.46590948
CTTNBP2NL	1.0081077
CTU2	0.46300125
CUZD1	7.1731896
CWC22	1.6203623
CX3CL1	3.4323378
CXCL1	7.3250604
CXCL10	4.017641
CXCL12	0.5335994
CXCL13	2.3379927
CXCL16	1.0202079
CXCL2	5.6254663
CXCL3	3.0072234
CXCL5	4.432497
CXCL9	1.2320042
CXCR1	0.7033982
CXCR2	1.1469035
CXCR5	0.93828917
CXXC4	1.0003572
CYB561	0.77997017
CYB5B	0.31519794
CYB5RL	0.41612554
CYBB	0.4884553
CYCS	0.2683506
CYFIP1	0.5270276
CYFIP2	0.1906619
CYLC2	2.267627
CYLD	0.48851252
CYP1B1	0.7490268
CYP20A1	0.18297267
CYP26A1	0.8602345
CYP26B1	0.9285269
CYP2A22	1.3508763
CYP2D26	0.37534142
CYP2D40	1.1162877
CYP39A1	2.0506868
CYP4A12B	1.2582977
CYP4F13	0.36182976
CYP7B1	2.2117052
CYPT10	1.2691798
CYR61	0.7331028
CYTH1	0.7028928
CYTIP	0.90150833
CYYR1	1.7834916
DAB1	0.5808177

DACH1	0.5351901
DAF2	0.60760117
DANCR	0.8131232
DAPK3	0.25778008
DAPL1	0.62563133
DAPP1	0.4347887
DAW1	1.2408798
DAXX	1.3869424
DBHOS	0.3326521
DBX2	2.2693725
DCAF12L2	1.2772002
DCC	0.9231534
DCLRE1C	0.5432062
DCP2	0.6189332
DCPP3	0.51779985
DCUN1D3	1.092562
DDAH1	1.4020123
DDB2	0.303061
DDIT4	0.53293896
DDT	0.4012351
DDX18	0.3767748
DDX21	0.6092782
DDX24	0.4504881
DDX25	0.95163155
DDX31	0.40789318
DDX43	0.31290746
DDX5	0.17553425
DDX52	0.39063072
DDX54	0.18728542
DDX58	1.5684328
DDX6	0.5794449
DEF6	0.43809462
DEFA2	1.3675883
DEFA3	0.9996884
DEFA-RS10	0.8502908
DEFA-RS12	1.3242004
DEFA-RS2	1.2667251
DEFA-RS4	1.4332705
DEFA-RS7	1.4848251
DEFB33	0.82544804
DEFB35	1.3203938
DEFB36	0.76525116
DENND2D	0.72762966
DERL2	0.2385621
DGAT2	1.4088912

DGKA	0.57899237
DGKEOS	0.5834775
DGKH	1.3733625
DGKK	0.25962758
DGKQ	0.25626612
DHPS	0.23103428
DHRS9	0.81688786
DHX30	0.29147768
DHX33	0.7135472
DHX35	0.26354647
DHX58	0.7275901
DIAP2	0.21021557
DIO3OS	0.4824574
DIRAS1	1.5626166
DIXDC1	0.4390216
DKK2	0.28794646
DLC1	0.49892426
DLK1	1.4977
DLL4	0.9513383
DMBT1	10.251303
DMP1	0.8712063
DMRTC1C2	0.6949365
DMTF1	0.4951601
DMXL2	0.52855873
DNAAF1	0.73006773
DNAH10	0.8067758
DNAH6	0.57681346
DNAJA1	0.7573805
DNAJB5	0.35771322
DNAJB6	0.22159815
DNAJB8	1.4361548
DNAJB9	0.4703641
DNAJC1	0.20097637
DNAJC2	0.7933812
DNAJC21	0.4507804
DNAJC5	0.4271207
DNAJC7	1.3490863
DNASE1	0.86065006
DNASE1L3	1.0190935
DNASE2A	0.4228258
DNASE2B	2.1533074
DOC2G	0.63428307
DOCK10	0.37080336
DOCK4	0.71833324
DOCK5	0.5210371

DOCK8	0.46345615
DOCK9	0.7247877
DPEP3	0.90470743
DPH2	0.7013383
DPH5	0.39263725
DPP7	0.31270933
DPPA4	0.4961648
DPY19L2	0.81016445
DPYSL3	0.19095945
DRAM1	0.8659477
DRD1A	0.5412104
DRD2	1.1189947
DRD5	1.884409
DSC3	0.3245542
DSCAM	0.6309092
DSG1C	1.7194018
DSG2	0.8360057
DSPP	1.3182952
DST	0.6861675
DTX1	0.7947507
DTX2	0.7758236
DTX3L	0.9513917
DUOX1	0.20521164
DUOXA1	0.8612137
DUPD1	1.3860731
DUS3L	0.32794714
DUSP1	1.813592
DUSP11	0.3977909
DUSP12	0.5050149
DUSP16	2.3833256
DUSP18	0.4544046
DUSP2	2.0593934
DUSP4	0.5186863
DUSP5	1.5932579
DUSP6	0.2666397
DUSP7	0.3711319
DUX	0.7578523
DVL3	0.78233385
DYNC1H1	0.28971434
DYNLT3	0.3965454
DYRK2	0.60493517
EBF1	0.68841934
EBI3	1.1847215
EBNA1BP2	0.30365515
ECE1	0.19045973

ECE2	0.8181958
ECEL1	0.67901707
ECHDC3	0.7724271
ECSCR	0.7358885
ECSIT	0.194808
EDN1	2.933407
EDNRA	0.18869972
EEA1	0.92930365
EED	0.23906136
EFCAB14	0.28288078
EFCAB4B	0.51636004
EFCAB8	1.3079057
EFCAB9	0.32098746
EFHD2	0.5673909
EGFBP2	3.795745
EGFEM1	1.2635729
EGFR	1.3677685
EGLN2	1.0447392
EGLN3	0.6499977
EGR1	1.3259578
EGR2	1.4794626
EHD1	1.8000484
EHD3	0.32215166
EHF	1.6203127
EID2	0.44471025
EID3	1.2727227
EIF1A	0.95985746
EIF2AK2	1.036243
EIF2S3Y	1.272197
EIF4A1	0.44537258
EIF4A2	0.7632756
ELK4	0.41202116
ELL3	1.2184806
ELMO1	1.2934918
ELOVL4	0.604054
ELOVL5	0.28433895
ELOVL6	0.4502759
EMB	0.5174694
EMC4	0.30739737
EMR1	0.26265478
ENC1	1.831326
ENO2	0.4744377
ENPP2	0.40311813
ENPP4	0.53787756
ENTPD4	2.36132

EP400	0.24458599
EPAS1	0.28776932
EPB4.1L1	0.808712
EPCAM	0.8339417
EPGN	1.1370211
EPHA10	0.27488494
EPHA6	0.89000845
EPHA7	1.3903358
EPHB2	0.6562104
EPHX4	0.6567023
EPPK1	0.81818175
EPSTI1	0.7107928
EQTN	0.7597511
ERC1	0.19318318
EREG	3.6880367
ERMN	1.3517165
ERN1	0.29063106
ERO1LB	0.28909636
ERP27	0.62116003
ERP44	0.3723774
ERRFI1	3.6864028
ERV3	1.6132131
ESM1	0.48080635
ESPNL	0.4010949
ESR2	0.25822687
ESRP2	3.740818
ESRRB	0.83247924
ETNK1	0.7089286
ETNK2	0.5451572
ETS2	0.9460721
ETV3	1.1135087
ETV3L	0.6535182
ETV6	1.0037618
EVA1C	1.0970707
EXOC1	0.2693348
EXOC3L4	0.5598879
EXOC6B	0.28871775
EXOSC6	0.3405695
EYA4	0.72562337
F10	0.979661
F11R	0.8582082
F13A1	0.22808933
F2R	0.34127092
F3	0.45386267
FABP3	0.89349747

FABP5	0.5709429
FAIM	0.37551165
FAM102A	1.0212226
FAM107A	0.73513126
FAM115E	1.2321677
FAM118A	0.5516257
FAM120B	0.55293703
FAM120C	1.077312
FAM124B	0.32471085
FAM126B	0.30549097
FAM129A	0.49701977
FAM129B	1.4794307
FAM134B	0.42589235
FAM13C	1.6681089
FAM160A1	0.51061606
FAM162A	0.23531961
FAM162B	0.37344694
FAM163B	1.4180794
FAM166A	0.55529165
FAM168B	0.31508064
FAM169A	1.2432578
FAM179B	0.19957161
FAM181A	1.1314957
FAM184B	0.8383086
FAM188A	0.30756044
FAM196A	1.1141329
FAM198A	0.76926327
FAM19A3	0.7724364
FAM206A	0.484941
FAM208A	0.43776846
FAM208B	0.835135
FAM20C	0.90886116
FAM214A	0.25547218
FAM217A	1.5553796
FAM222B	0.8468232
FAM228A	0.95367646
FAM25C	1.4295862
FAM26E	0.4436891
FAM26F	1.0503263
FAM3B	0.5947497
FAM46A	0.30805683
FAM47E	0.66921663
FAM49A	0.71050787
FAM53A	0.30751562
FAM53C	0.30335855

FAM60A	0.7009406
FAM65B	0.8837681
FAM72A	0.25744343
FAM78B	2.971177
FAM84B	0.641968
FAM89A	0.85476446
FAP	1.6568384
FAS	2.230434
FASL	0.4335568
FASTKD1	0.4213605
FASTKD3	0.56495
FASTKD5	0.19405079
FBL	0.45294046
FBXL12	0.7619157
FBXL5	0.73394156
FBXL7	0.71293354
FBXO22	0.37658596
FBXO24	1.0123429
FBXO39	1.2307179
FBXO4	0.49964476
FBXO47	0.71985245
FBXW10	0.48993587
FBXW11	0.41011906
FBXW13	2.2227733
FBXW17	0.41111422
FBXW19	0.7321477
FBXW26	1.1251721
FCAMR	1.235426
FCER1G	0.53942347
FCGR2B	0.56067276
FCRL1	1.4184222
FERD3L	1.6668253
FFAR1	1.0367787
FFAR2	1.0398083
FGB	2.3891604
FGD3	0.4623294
FGD4	0.7180357
FGD6	0.35151172
FGF17	1.9941056
FGF21	2.7406847
FGF23	0.5094714
FGF3	0.7985103
FGFR1OP	0.29082108
FGFR2	0.33660364
FGG	2.6016498

FHOD1	0.30372238
FHOD3	0.56697226
FILIP1L	0.77656317
FJX1	3.2623568
FKBP5	1.5647955
FKBP6	3.581771
FLCN	0.19771194
FLNA	0.18988705
FLNB	1.6300769
FLOT1	0.23121119
FLRT3	0.37018967
FMNL2	0.91609573
FMO3	1.9489062
FMO6	1.0296474
FNBP1L	1.1582546
FNDC3B	0.8990803
FNDC7	0.4524274
FOLH1	0.75278735
FOLR4	0.6398196
FOS	2.1918736
FOSL1	1.3210795
FOSL2	1.8208604
FOXA3	0.87757707
FOXC1	0.88847446
FOXE3	1.4129279
FOXF1	1.6151237
FOXJ3	0.33088732
FOXN2	0.3993082
FOXP1	0.37805176
FOXP4	1.3159132
FPR1	2.3586054
FPR2	2.1885934
FPR3	2.4330957
FPR-RS6	1.6926866
FRMD7	1.996829
FRMPD4	0.96017313
FRZB	0.65267324
FSCN1	1.5513806
FST	0.66314125
FUBP1	0.2642932
FUK	0.27088165
FURIN	0.29225063
FUT1	0.22646928
FYB	0.17010593
FZD1	0.7300792

FZD4	0.8477268
FZD9	0.38302588
G3BP1	0.3433938
G630055G22	1.1355083
G6BOS	0.8613572
GAB3	1.0411029
GABRB1	0.45649266
GABRG2	0.80830073
GABRP	0.999835
GADD45B	2.3357725
GADD45G	1.1872578
GALK2	0.3740058
GALNT11	0.79941463
GALNT12	0.8418255
GALNT18	0.45154238
GALNT5	0.25157905
GALNT6	0.46086788
GALNT7	0.23408985
GALNTL5	0.8073683
GAR1	0.4627161
GARS	0.28445148
GAS7	0.57047415
GAST	1.106466
GATA3	0.53840923
GATA4	1.460681
GBP10	1.1818476
GBP2	0.73166275
GBP3	0.7549586
GBP4	1.3674421
GBP5	2.6127887
GBP6	1.4949718
GBP7	0.75526476
GBP9	0.7511864
GBX1	1.3666704
GC	0.5214932
GCA	0.19509673
GCAT	0.681993
GCG	3.0153584
GCK	0.62459636
GCM1	0.9196849
GCNT2	1.0324917
GCSAM	0.6164479
GCSH	0.2194395
GDAP10	2.0893412
GDF15	3.4148464

GDF7	1.1121876
GEM	2.1451592
GFI1	0.9153919
GFPT1	0.36287165
GFPT2	0.9099965
GFRA3	1.2837589
GGCT	0.62827444
GGNBP1	0.7297485
GGPS1	0.22297716
GGT1	2.9382112
GGT5	0.5160711
GGT7	0.7110281
GGTA1	0.72254467
GH	0.38226533
GIMAP1	0.72116375
GIMAP1OS	0.82115006
GIMAP3	0.97073793
GIMAP4	0.9667411
GIMAP5	1.2346096
GIMAP6	0.8436508
GIMAP8	1.2024612
GIMAP9	0.7798052
GIPC3	0.963439
GIT2	0.4900527
GJA4	0.837461
GJA8	0.66732717
GJB1	0.7388735
GJC1	0.28977776
GJC2	1.8252931
GJD3	1.648987
GK5	0.2772379
GLA	0.4651432
GLI1	1.6821253
GLRX3	0.37213182
GLTSCR1	0.7410562
GM10099	2.4110694
GM101	2.04247
GM10153	3.0376186
GM10228	0.5914211
GM10252	0.22481918
GM10278	2.8767903
GM10286	0.64591146
GM10334	8.40825
GM10369	1.0113964
GM10389	0.9365792

GM10398	1.0896053
GM10403	1.029769
GM10433	1.0287435
GM10435	0.90954447
GM10436	0.8833504
GM10445	1.1241791
GM10461	1.0375133
GM10466	0.849622
GM10471	0.88504744
GM10510	1.2787752
GM10513	1.243825
GM10532	0.51041055
GM10556	0.83285236
GM10563	1.1643972
GM10579	1.0451822
GM10584	0.35534072
GM10589	1.0830638
GM10615	0.24018693
GM10631	0.41692543
GM10634	2.3836687
GM10640	0.23978305
GM10673	3.024167
GM10674	1.5065548
GM10696	1.8242819
GM1070	0.23431301
GM10700	1.0399783
GM10703	0.88644576
GM10741	0.7026768
GM10747	1.3937781
GM10777	0.556324
GM10789	0.9315803
GM10791	1.2639995
GM10792	0.9687829
GM10796	0.68206334
GM10814	0.6143875
GM10836	1.0925014
GM10839	3.8461192
GM10845	0.40313148
GM10847	0.8086896
GM10851	2.428845
GM10867	0.91906905
GM10869	0.2683246
GM10872	0.23233986
GM1110	0.8494501
GM11110	0.8647895

GM11123	0.42485523
GM11346	0.84443617
GM11437	0.9094901
GM11544	0.8161919
GM11696	0.45777035
GM11735	1.2419448
GM11747	0.67523766
GM11974	0.9210887
GM11992	1.2749405
GM12	1.1158149
GM12185	1.0452831
GM12250	1.4219441
GM12253	1.0388224
GM12302	0.827152
GM12503	0.8800416
GM12569	0.51784205
GM12592	1.3776913
GM12693	0.30954933
GM12758	0.25646138
GM128	0.819087
GM13011	10.15542
GM1305	0.89095926
GM13088	0.7467234
GM13238	0.72536993
GM13262	0.65201664
GM13271	0.24625087
GM13298	0.37848043
GM13375	0.99071026
GM13546	0.9036095
GM13889	3.1231751
GM13931	0.8653445
GM14005	1.5429788
GM14010	1.6558573
GM14015	1.3930626
GM14085	0.7599611
GM14129	1.2269483
GM14308	0.5040097
GM14317	0.9492183
GM14321	1.6808939
GM14420	0.7853074
GM14446	1.3002577
GM14486	0.59215903
GM14496	1.3741314
GM14499	1.341789
GM14762	0.4242065

GM14851	0.8779981
GM14858	0.39030838
GM15056	2.605391
GM15232	0.3218856
GM15340	1.011545
GM15401	0.37937403
GM15413	0.9219887
GM15421	0.41396666
GM15455	0.30335808
GM15478	0.62429
GM15628	0.63735294
GM15698	0.3894279
GM15762	1.4672682
GM15772	0.57300806
GM15821	0.94837356
GM15850	1.105464
GM15998	1.146297
GM16340	0.90192556
GM16390	0.8607733
GM16486	1.737524
GM16489	1.1667433
GM16491	0.4350369
GM16498	1.0014558
GM16505	1.9649873
GM16523	0.62023735
GM16524	0.46453738
GM16863	1.3715024
GM16982	1.59898
GM17244	0.9103894
GM17739	1.0723968
GM17753	0.8194964
GM17778	0.5775919
GM17821	1.2763395
GM1965	0.8966427
GM1966	0.7181401
GM1987	0.37803888
GM2016	0.5494089
GM20555	1.1189823
GM20558	2.491267
GM2061	0.5038171
GM20611	1.382273
GM20755	0.88513136
GM20767	0.53058624
GM20865	0.2858944
GM21944	3.9974418

GM21951	0.9587743
GM2366	1.7687438
GM2382	0.7591262
GM2447	0.99769545
GM2483	1.817992
GM2511	1.9727252
GM2545	2.0697203
GM2590	0.7560172
GM2675	1.0097218
GM2701	0.68071604
GM2824	0.48578334
GM3032	0.8591256
GM3081	1.229223
GM3086	0.6620414
GM3161	1.7538424
GM318	0.92835116
GM324	0.4603436
GM3367	1.0758927
GM3383	0.29384995
GM3428	2.4484222
GM3470	1.2008972
GM3556	1.4704099
GM3596	1.5344024
GM3696	0.85165095
GM3764	0.8780091
GM3881	0.61294293
GM3893	0.6300969
GM3907	1.1120646
GM3985	0.9263482
GM4013	0.3640504
GM4107	1.7072151
GM4146	0.85382056
GM4200	1.0628517
GM4221	0.61895657
GM4279	1.4910495
GM4354	0.99846935
GM4371	1.2036872
GM4419	0.9939201
GM4425	0.28559256
GM4432	2.6420083
GM4466	1.0176563
GM4524	1.050415
GM4544	0.35317564
GM4559	0.45501542
GM4567	1.1954618

GM4610	1.6940784
GM4745	0.9535551
GM4755	1.1593091
GM4759	0.5156679
GM4787	0.7480619
GM4832	0.40292883
GM4841	1.5126753
GM4850	1.0376315
GM4861	0.6737771
GM4884	0.6649842
GM4907	0.9493499
GM4924	0.5577874
GM4925	0.5426047
GM4951	0.98736143
GM4956	0.40384078
GM4971	0.76146984
GM4975	1.0326796
GM4983	0.9604778
GM4994	1.8013799
GM5	0.4692974
GM5067	0.8896389
GM5115	0.85119605
GM5122	1.166296
GM5129	1.3073864
GM5134	1.2296023
GM5150	0.2210145
GM5174	0.9041078
GM5331	0.72646713
GM536	2.002653
GM5409	8.222702
GM5416	2.0414853
GM5431	1.1910133
GM5464	1.941602
GM5468	0.48428416
GM5483	2.5244212
GM553	0.9574151
GM5547	0.274518
GM5590	2.18567
GM5602	0.5532565
GM5608	1.4984767
GM5637	0.7974596
GM5771	9.869521
GM5833	0.6071031
GM5858	0.4142084
GM5878	1.2869172

GM590	3.5289173
GM5908	0.5558777
GM5916	1.0514755
GM5941	0.9947145
GM5953	0.6512153
GM597	1.128747
GM614	1.6636312
GM6146	1.2023511
GM6219	0.20190454
GM6252	0.61189365
GM6287	1.1682332
GM6306	0.5184252
GM6377	3.0362554
GM6402	0.79921603
GM6403	1.3055394
GM6460	0.7364123
GM6524	0.21386385
GM6537	1.0625479
GM6566	1.013726
GM6712	0.60466313
GM6713	0.5339606
GM6756	0.3771119
GM684	0.95225024
GM6904	1.5046601
GM6943	0.9256897
GM6961	0.9177866
GM7111	0.361068
GM7160	0.8265343
GM7457	0.74098015
GM7467	1.1068006
GM7480	1.1359823
GM7609	0.95476055
GM7696	0.41172743
GM7849	1.0597432
GM7894	1.2617509
GM805	1.054893
GM8075	1.9080074
GM813	0.8634238
GM8221	0.34014845
GM8273	0.47802734
GM831	0.43795395
GM8369	0.6398325
GM8493	0.75699687
GM8630	1.0590181
GM8884	0.7185743

GM8909	0.6373601
GM8995	1.1430211
GM9258	0.55227137
GM9456	0.29470706
GM9510	0.90793324
GM9519	0.417516
GM9564	1.5477557
GM9693	0.5362778
GM9706	2.325007
GM9725	0.9927454
GM9734	0.56743956
GM9752	0.8986182
GM9779	0.50168633
GM9839	0.29492426
GM9856	0.6175513
GM9866	1.1671536
GM9869	0.8474498
GM9878	2.0788531
GM9910	1.4755578
GM9916	1.0049658
GM9946	1.0918655
GM9948	0.96896863
GM9958	0.6811595
GM9961	1.0866332
GM9981	2.2634246
GM9984	2.1440613
GMCL1	0.2093153
GMDS	0.4694047
GMFG	0.38961458
GMPPB	0.20050335
GNA13	0.45335817
GNAI1	0.8681202
GNAI3	0.27142525
GNB3	1.3350627
GNG12	1.1089811
GNG4	2.3555913
GNGT1	0.8952031
GNGT2	0.30074406
GNL1	0.7583103
GNL3	0.24737692
GNMT	0.8245311
GNPNAT1	0.67672014
GNPTAB	0.44182587
GOLGA1	0.24034452
GOLGA3	0.39776325

GOLGA4	0.8006873
GON4L	0.19606781
GOPC	0.6136384
GORAB	0.26968765
GOT1	0.6107416
GP2	8.004887
GP49A	2.024219
GPANK1	0.25616455
GPATCH4	1.0597105
GPBAR1	0.435992
GPHN	0.6592717
GPM6B	0.68918514
GPN1	0.2789178
GPR110	1.8590674
GPR113	0.91751313
GPR114	0.5930095
GPR126	1.743887
GPR132	1.159051
GPR137B	1.2294612
GPR137B-PS	1.1077371
GPR151	0.90279794
GPR171	2.0642962
GPR179	1.8204007
GPR18	1.5104012
GPR183	1.2854056
GPR21	1.1629412
GPR26	0.6680093
GPR31B	1.7614882
GPR33	1.1440959
GPR39	1.1982238
GPR55	1.7454267
GPR6	1.0661881
GPR65	0.6129346
GPR68	0.7275729
GPR84	3.9447556
GPR85	4.0348873
GPRASP1	0.551291
GPRC5A	0.48263073
GPRIN2	0.56892085
GPSM1	0.31245375
GRAMD1A	1.2039905
GRAMD1B	0.30765152
GRAMD2	0.7245145
GRAMD3	1.3497
GRAP	0.374578

GRHL1	1.5680122
GRIK2	0.65978575
GRM6	1.6161463
GRM8	1.0925727
GRPR	2.2308514
GRWD1	0.36179447
GSAP	0.783175
GSDMC4	0.91602826
GSTT4	1.9211743
GT(ROSA)26S	0.33968258
GTF3C6	0.2552104
GTPBP4	0.802083
GTPBP6	0.4535761
GTSF1L	0.9479244
GUCY2D	0.3395362
GUCY2F	2.1050682
GVIN1	0.7950492
GYK	1.5535793
GYS2	1.0958965
GZF1	0.22321558
GZMA	0.49520922
GZMB	1.849741
GZMC	2.5501878
GZMK	1.5701275
H1FOO	0.35535502
H2AFY3	0.21428776
H2BFM	0.90623856
H2-DMB2	0.7889972
H2-EB2	0.6371145
H2-M11	1.2582316
H2-OB	0.30329704
H2-Q5	0.83951855
H2-T24	0.90058756
H60A	1.9802628
H60B	2.0946007
H6PD	0.2607355
HAAO	0.28569126
HABP2	2.1501496
HAMP	1.0961108
HAMP2	0.29553962
HAND1	1.4155266
HAP1	0.22153234
HAPLN3	0.47352982
HARS2	0.44486332
HAS1	2.1909738

HAS2	3.5379071
HAS2OS	1.921108
HBEGF	2.53479
HC	2.1360881
HCAR2	0.3411126
HCK	1.2153425
HCLS1	0.6952486
HCN3	1.0444121
HDAC9	0.7936392
HDC	2.231978
HDGFRP3	0.452502
HDX	1.8630612
HEATR1	0.6720681
HEATR3	0.67027044
HECTD2	0.96704817
HEG1	0.85707164
HELZ2	1.6662903
HEMT1	0.43263793
HEPHL1	0.8504071
HERC3	0.6685171
HERC4	0.2481494
HERC6	1.3402457
HERPUD1	0.38078642
HES2	0.44255805
HES3	0.86978364
HES5	1.5534897
HEXIM2	0.3086667
HHAT	0.69074106
HHIPL2	2.1147778
HIF1A	1.2982068
HIGD1B	0.39343357
HILPDA	1.6566896
HIST1H1T	0.29032135
HIST2H3C2	0.89941406
HIVEP2	0.8596144
HIVEP3	1.1008291
HK2	0.76043844
HKDC1	1.6335363
HMCES	0.4835477
HMGB4OS	0.5741875
HMGXB3	0.491724
HMOX1	2.3006387
HNF4A	0.23273182
HNRNPA2B1	0.42503643
HNRNPH3	0.39811802

HOOK1	0.22078443
HOOK2	0.5256467
HORMAD1	0.9273803
HOXA5	0.98369837
HOXA9	1.263469
HOXC8	1.2394316
HOXD3	1.1022875
HP	1.5703201
HPDL	0.40212464
HPGDS	0.9741726
HPS5	0.40331554
HPS6	0.37440348
HPX	1.1802309
HRG	0.71034384
HS3ST1	0.751951
HSD17B13	0.80437803
HSD17B2	1.5387497
HSF1	0.26717424
HSH2D	0.92406034
HSP90AB1	0.4672227
HSP90B1	0.34009457
HSPA1A	3.166572
HSPA5	0.7154889
HSPA8	0.27772903
HSPA9	0.25347948
HSPB1	0.982038
HSPB7	0.5088117
HSPD1	0.41697264
HSPE1	0.5982771
HSPH1	0.6476755
HTATSF1	0.70355463
HTR1B	1.8455637
HTR1D	1.1208084
HTR2B	1.049176
HTR3A	0.81709576
HTR6	0.890892
HTR7	2.3899918
HTRA1	1.2747364
HTT	0.5333023
HVCN1	0.33572865
HYKK	1.153265
ICAM1	1.8613701
ICOS	0.51219845
ICOSL	0.72347975
ID2	1.0023503

IDI2	1.4647284
IDO2	1.1313713
IDUA	0.29877567
IER2	0.6474471
IER3	2.8957515
IER5	0.6292839
IER5L	0.5758405
IFFO2	0.7138257
IFI202B	1.566432
IFI203	0.94172144
IFI204	1.122077
IFI27L2A	0.25493002
IFI35	0.8571696
IFI44	2.045127
IFI44L	6.042626
IFI47	1.4566936
IFIH1	1.6540141
IFIT1	2.5585814
IFIT2	1.5053778
IFIT3	1.9094334
IFITM1	1.2822189
IFITM5	2.0217152
IFITM6	0.53207827
IFNA11	1.322186
IFNA13	1.1165977
IFNA2	1.3797648
IFNA5	0.34543228
IFNAB	0.711653
IFNAR1	0.40117884
IFNB1	4.2508535
IFNG	2.2231216
IFNGR2	0.40090704
IFNK	0.7872491
IFNLR1	1.0511222
IFRD1	1.6451678
IFT88	0.6984806
IGF2	1.3397238
IGF2BP2	0.23304248
IGFLR1	0.4830289
IGHV14-2	0.70406246
IGKV4-55	1.0162156
IGSF1	0.9439666
IGSF23	2.2230773
IGSF5	0.9069109
IGSF6	0.6540475

IGSF8	0.39193726
IGTP	0.9592142
IIGP1	1.37994
IK	0.4066472
IKBKE	1.7415485
IKZF1	0.5040488
IKZF4	0.7358551
IL10	3.0187025
IL10RA	0.21032095
IL12B	4.635165
IL12RB1	0.43586683
IL12RB2	0.5335758
IL13RA2	0.97303057
IL15RA	0.35707092
IL16	0.20706892
IL17A	4.235691
IL17RA	0.43265915
IL17RB	0.7269478
IL18	2.1502886
IL18R1	0.2857585
IL19	1.0189223
IL1A	3.4575286
IL1B	3.6146836
IL1F9	2.921327
IL1R1	0.7844062
IL1R2	3.8958745
IL1RAP	0.866173
IL1RN	3.5188403
IL2	1.6852891
IL20RB	0.98528504
IL21	1.0160687
IL21R	0.87028074
IL22	2.760804
IL23A	2.2281535
IL27	1.1113472
IL27RA	1.009068
IL2RA	1.5691333
IL2RB	0.40103436
IL2RG	0.66580486
IL3	0.560683
IL33	1.2640796
IL4I1	1.5530481
IL4RA	1.0030823
IL6	7.7868
IL7R	1.537682

ILDR1	1.8686376
IMAGE:6196	0.36754417
IMP4	0.3011818
IMPACT	0.46656513
INADL	0.6224587
ING3	0.36024904
INHBA	1.2991824
INHBB	3.5881553
INHBE	1.0299551
INIP	0.29577255
INO80B	0.2934184
INPP4B	0.5405457
INPP5D	0.3787489
INS1	3.1413305
INS2	1.4089229
INSM1	0.9572475
INTS12	0.23821545
INVS	0.7622924
IPCEF1	1.1246233
IPO4	0.48568964
IQCF1	1.1394382
IQCF4	1.6602156
IQCJ	1.1356654
IQCK	1.3010633
IRAK3	1.9714689
IRAK4	0.5152557
IRF1	1.1671348
IRF4	0.3209195
IRF7	0.86910915
IRF9	0.5210638
IRG1	4.018429
IRGM1	1.0887694
IRGM2	0.829474
IRX3	2.1959276
ISG15	1.8438182
ISM2	0.9300678
ISOC1	0.455194
ISOC2B	0.48464298
ITGA1	0.49841166
ITGA10	0.87588763
ITGA5	0.86451197
ITGA7	0.5563464
ITGA9	0.7671995
ITGAL	1.4179974
ITGAV	0.77520037

ITIH4	3.8565283
ITK	1.6315999
ITLN1	0.38160706
ITLNB	1.1281545
ITPKC	0.66672087
ITPR2	0.51813316
ITPR3	0.7721591
ITPR3OS	0.9567015
ITPRIP	0.6793585
IWS1	0.44867706
JADE2	0.7079954
JAK1	0.3892331
JAK2	0.44616175
JAK3	1.0145965
JAKMIP1	0.26843357
JAM2	1.1313319
JDP2	1.0414853
JMJD6	0.272727
JUN	1.9825015
JUNB	1.3763509
KANK4	0.5307131
KANSL3	0.37791014
KATNA1	0.39375544
KATNAL2	0.9595108
KBTBD11	1.5358467
KCNA10	0.88306475
KCNC3	0.7793813
KCNE3	0.86744857
KCNE4	2.264451
KCNG1	0.38933206
KCNH7	1.571887
KCNIP1	1.0607045
KCNJ13	1.0901859
KCNJ15	0.3962624
KCNJ2	1.0262032
KCNJ6	0.26038456
KCNJ8	1.3235211
KCNK10	1.7049477
KCNK6	0.57058764
KCNMB4	0.7086184
KCNQ2	0.46711493
KCNRG	0.6091962
KCNS1	1.1960821
KCTD11	0.65966797
KCTD12	0.54933405

KDF1	0.6699171
KDM1B	0.42635632
KDM2B	0.3077345
KDM6B	1.1510439
KHDC3	0.7830982
KIF12	1.8024111
KIF1A	0.6557193
KIF1B	0.35391426
KIF21A	0.3878224
KIF21B	0.17693806
KIF27	0.90723896
KIRREL	0.43871427
KIRREL3	0.5740547
KL	1.1706867
KLC2	0.5810919
KLF17	0.43051577
KLF6	0.74262
KLF7	0.91564655
KLHDC1	0.41516662
KLHDC4	0.80127
KLHL10	0.8000798
KLHL2	0.8376818
KLHL21	0.59521675
KLHL28	0.4712715
KLHL3	0.473289
KLHL35	0.71353483
KLHL6	1.1881185
KLK1	3.45372
KLK10	0.7849927
KLK15	0.92779446
KLK1B1	1.2303994
KLK1B11	1.2102678
KLK1B22	1.08378
KLK1B24	2.176187
KLK1B26	3.6584487
KLK1B3	3.1917448
KLK1B4	3.419353
KLK1B5	3.9660907
KLK1B8	2.8670135
KLK4	0.82385993
KLK8	0.6400709
KLK9	0.57112527
KLRA2	2.1018815
KLRA5	0.44476962
KLRB1B	0.5323348

KLRC2	1.6711879
KLRE1	0.43539953
KMT2A	0.7185173
KMT2C	0.26267004
KMT2D	0.59800386
KNG1	1.1340101
KPNA4	0.5666046
KRBA1	0.290164
KRCC1	0.3377018
KRI1	0.77014875
KRR1	0.4196391
KRT13	1.2232327
KRT16	2.21109
KRT17	0.9450841
KRT26	1.5984442
KRT31	1.3082867
KRT33B	1.279192
KRT6A	1.416513
KRT72	0.7797451
KRT86	0.33108473
KRTAP10-4	0.7075739
KRTAP11-1	1.4083478
KRTAP1-4	1.8209424
KRTAP16-3	0.8723345
KRTAP26-1	1.1204188
KRTAP28-13	0.81192327
KRTAP3-1	0.98210764
KRTAP4-1	1.6271541
KRTAP5-5	0.2592559
KRTAP9-3	1.1820433
KRTCAP2	0.18838167
KRTCAP3	0.28854847
KSR1	0.51990676
KTN1	0.40526676
L1TD1	0.9334631
L3MBTL3	0.42720175
L7RN6	0.24436283
LACC1	1.434555
LACTBL1	1.0592296
LAD1	3.3554406
LAMA5	1.325377
LAMB3	0.6836319
LAMC1	0.36872816
LAMC2	2.1937318
LAMTOR2	0.9322915

LAMTOR3	0.1795783
LANCL3	0.5195744
LAPTM4B	0.4371972
LAPTM5	0.29723644
LARP1	0.34737253
LARP4	0.37879467
LAT	0.65722466
LATS2	0.4097376
LAX1	0.42555857
LCA5	1.4278808
LCE1B	0.62571526
LCE3B	0.5626602
LCK	0.37172508
LCN2	0.51512957
LCN6	0.76966214
LCP1	0.6622634
LDHA	0.5007243
LDHD	2.190104
LEF1	0.6050749
LEFTY2	0.7350457
LELP1	0.47049236
LENG1	0.49911833
LENG9	0.36804342
LETM2	0.5945661
LGALS8	0.6023183
LGI2	0.46188712
LGR4	1.1725333
LHB	0.9253354
LHX6	0.56784296
LIF	3.2432673
LIG4	0.29866457
LILRB4	1.716013
LIMA1	0.43220186
LIMD2	0.19570017
LIME1	0.2892537
LIMK2	0.9257579
LIPC	0.8500483
LIPG	3.8304813
LIPH	0.4422524
LIPO1	0.41672325
LIPT1	0.26276064
LITAF	0.5255084
LIX1L	0.7768476
LMAN2L	0.28135395
LMBR1L	0.770823

LMBRD1	0.21281528
LMBRD2	0.6092949
LMO4	0.51315594
LMX1B	0.7233019
LNPEP	0.9982953
LNX1	0.69275904
LNX2	0.25611353
LOC1000417	0.882952
LOC1008617	0.20747662
LOC1010558	1.8112738
LOC1010562	0.5811095
LOC1010562	0.72299385
LOC1010562	0.9348972
LOC1026321	0.4770651
LOC1026324	0.23065186
LOC1026332	0.6783438
LOC1026336	0.40355062
LOC1026336	0.4227214
LOC1026337	1.1667204
LOC1026337	0.86381674
LOC1026338	0.40714598
LOC1026344	0.31969404
LOC1026361	1.6847446
LOC1026367	1.715255
LOC1026367	0.6276698
LOC1026369	0.75268626
LOC1026369	1.4663358
LOC1026377	0.7800596
LOC1026380	0.8712344
LOC1026388	0.92912316
LOC1026391	0.76989746
LOC1026392	1.2107291
LOC1026395	2.2104282
LOC1026397	1.2135386
LOC1026398	1.2047503
LOC1026413	0.39923286
LOC1026415	0.19225168
LOC1026416	0.91591287
LOC1026423	1.1073108
LOC1026423	4.290121
LOC1026424	1.493187
LOC1026424	0.8411348
LOC1026427	0.26947355
LOC171588	1.8206642
LOC553089	0.97903085

LOC553096	0.50034094
LOC630751	1.8261127
LOC675947	1.856575
LONP2	0.34649086
LOX	2.4381928
LPAR1	1.2323623
LPAR3	0.82839465
LPGAT1	1.0999622
LPHN3	0.60454655
LPO	2.1694388
LPP	0.39649153
LPXN	0.93796444
LRCH1	0.4811821
LRCH3	0.5159397
LRFN1	0.41318607
LRFN5	1.4370372
LRG1	1.0355306
LRIF1	0.26105666
LRP11	0.3958571
LRP8OS1	0.65322685
LRRC16A	0.4632926
LRRC2	0.37239742
LRRC24	0.8762796
LRRC25	0.9211068
LRRC38	0.7198067
LRRC45	0.21460867
LRRC51	0.79894996
LRRC59	0.20063877
LRRC71	0.8519268
LRRC8E	0.5879705
LRRK1	0.47043324
LRRK2	0.83158445
LRRTM2	1.2898104
LRTM2	1.1034448
LSM12	0.19129753
LTA	0.7635517
LTK	0.5876062
LTV1	0.38041162
LUC7L	0.2315588
LUC7L3	0.30865717
LY6A	1.1092858
LY6C1	0.46435452
LY6D	0.17630243
LY6G5B	0.35607505
LY6K	1.2277923

LY75	0.86425567
LY86	0.610857
LY9	0.501739
LY96	0.63618517
LYN	1.2226291
LYPD3	1.0029125
LYPD5	1.2254307
LYSMD2	0.66438437
LYVE1	2.4621248
LZIC	0.50940275
MAB21L3	2.0585947
MADCAM1	1.162663
MAFF	3.5295448
MAGEA8	1.0254209
MAGEB1	0.61940646
MAGI3	0.4701965
MAGIX	0.7746868
MAGOHB	0.31102467
MAK16	0.1809926
MAL2	1.6114888
MALT1	1.9365711
MAMDC4	0.9258559
MAMSTR	1.0687871
MAN1A	0.27252436
MAN2A1	0.5810199
MAN2C1OS	0.31816196
MAP10	0.3740964
MAP2K1	0.575624
MAP3K1	0.24604177
MAP3K19	1.6582417
MAP3K2	0.3439293
MAP3K6	0.73636675
MAP3K7	0.3022437
MAP3K8	2.2708101
MAP4K4	0.7238984
MAP7D2	1.3953817
MAPK11	0.5933237
MAPK1IP1	0.56831074
MAPK6	0.636055
MAPK7	0.17284966
MAPK8IP3	0.31309128
MAPKAPK2	0.74232197
MARCKS	1.2756772
MARCKSL1	2.2557874
MARCO	2.322536

MARF1	0.50981
MASP2	1.5807343
MAT1A	4.892556
MB21D1	0.37654018
MB21D2	1.1693692
MBD1	2.3472137
MBL1	0.47759914
MBOAT1	0.2798376
MBTPS2	0.33041382
MCEMP1	1.0347939
MCIDAS	0.36704683
MCMBP	0.30184412
MCOLN1	0.42152405
MCPT9	0.9315312
MCTP2	0.3691225
MCTS2	0.43481207
MDFIC	0.8652215
MDM4	0.9951496
MDN1	0.9535103
ME3	1.4200976
MED1	0.33406448
MED10	0.2902465
MEF2B	0.57167387
MEFV	2.3576398
MESDC1	0.7029898
METTL17	0.337219
METTL21C	1.1691511
METTL23	0.5531864
METTL3	0.4613123
METTL7A1	0.4149449
METTL7A2	0.5935807
MEX3A	0.50509334
MFGE8	0.38102007
MFNG	0.3629322
MFSD1	0.28266716
MFSD2A	1.7594113
MFSD4	1.168289
MFSD6L	3.4645877
MFSD7A	1.5253088
MFSD7B	0.48340654
MGAM	0.29477692
MGARP	0.5869851
MGAT4A	0.82314825
MGEA5	0.7484431
MGRN1	0.35924673

MIA	1.7711322
MICALL1	0.5571561
MIDN	0.39285612
MIER2	0.2978363
MIF	0.27579165
MIF4GD	0.6256609
MINA	0.47831964
MIR22HG	0.19834614
MITD1	0.5947256
MKKS	0.7185278
MKS1	0.81568336
MLKL	0.2870283
MLLT11	0.64728403
MLLT6	1.177649
MLXIP	0.6848836
MLXIPL	0.5374222
MMAA	1.1405869
MMP13	3.6239824
MMP14	1.2270918
MMP1B	0.97306013
MMP24	0.9773567
MMP25	0.71756315
MMP3	4.6143565
MMP8	2.1394944
MNDA	1.710681
MNDAL	1.0038257
MNT	0.35420895
MOB4	0.35440874
MOGS	0.43193197
MON1B	0.991282
MOS	0.5846329
MOV10	0.62090063
MPP3	0.85497475
MPP7	0.6847296
MPZL2	1.555399
MPZL3	0.48293233
MRAS	0.8819952
MREG	1.3927002
MRFAP1	0.22275734
MRGPRA2A	1.2058039
MRGPRA2B	1.2093573
MRGPRA5	0.97802925
MRGPRA6	1.7533174
MRGPRA7	0.99324036
MROH1	0.57336164

MROH7	0.91573954
MRPL21	0.2660451
MRPL24	0.23978376
MRPL52	0.37224722
MRPS18B	0.2281909
MRPS6	0.50091314
MS4A1	0.26077223
MS4A4A	2.553668
MS4A4B	0.7467556
MS4A4D	1.357307
MS4A6B	1.2304692
MS4A6C	0.85674095
MS4A6D	3.1357121
MS4A7	0.5248289
MSANTD1	0.67763734
MSGN1	2.3654695
MSI1	2.2962575
MSI2	0.34937477
MSL3L2	0.34956717
MSN	0.3114748
MSR1	1.8303294
MSX2	0.81318665
MSX3	0.9544401
MT1	2.1855965
MT2	2.125935
MT3	0.39711332
MTDH	0.778399
MTERFD2	0.21609306
MTG2	0.25393248
MTHFD2L	0.7252052
MTHFS	0.683239
MTIF2	0.75264454
MTM1	0.208673
MTMR1	0.440495
MTNR1B	0.19826627
MUC20	0.6913729
MVP	0.75451994
MX1	2.38128
MX2	2.7356563
MXD1	0.6759095
MYBBP1A	0.5385847
MYBL1	0.72899246
MYBPC2	0.39729238
MYBPC3	0.51581526
MYBPH	0.40874743

MYC	0.6991663
MYD88	0.7676439
MYEOV2	0.25716734
MYH13	2.0827725
MYH7	0.63436985
MYH9	0.28156805
MYLK2	0.30330753
MYO10	0.94477034
MYO1B	0.6774423
MYO1E	0.69745064
MYO1G	0.36054897
MYRFL	1.2147083
MYSM1	0.4468751
N4BP1	1.0713274
N4BP2	0.3343091
N4BP2L2	0.36266184
NAA20	0.2572298
NAA25	0.7667265
NAA30	0.413455
NAB2	0.35381126
NABP1	0.7374668
NADK	0.910789
NAF1	0.25309515
NAIP1	0.41405582
NAIP5	0.7294464
NAIP6	0.45552874
NAMPT	1.1046515
NAPB	0.40180707
NAPEPLD	1.1692762
NAPSA	0.6653156
NAT10	0.47419024
NCBP2	0.3715024
NCF1	0.72144413
NCF4	0.3871913
NCK1	0.34001875
NCKAP1L	0.29678774
NCL	0.61129093
NCMAP	0.86045647
ND4	0.26185226
ND6	1.1036768
NDEL1	0.34527397
NDP	1.0423114
NDRG1	0.7357683
NDRG3	0.83313465
NEB	2.908647

NEBL	1.1638322
NECAP1	0.223423
NEDD4L	0.47833252
NEIL1	0.847466
NEK6	0.70343065
NEMF	0.22484636
NEPN	0.89390254
NEU1	0.592504
NEURL1A	0.4574132
NEURL3	0.663259
NEUROD2	1.4989955
NFATC1	0.5206857
NFE2L1	0.35534668
NFE2L2	0.29277706
NFIL3	1.6342301
NFKB1	0.9200554
NFKB2	1.3144174
NFKBIA	2.0004086
NFKBIB	0.6005111
NFKBID	0.89957
NFKBIE	0.6565237
NFKBIZ	2.3790174
NGDN	0.18182659
NGF	0.8468089
NGFR	0.5039158
NHLH1	0.3345611
NHLRC1	0.30637813
NINJ1	0.3218565
NIP7	0.23996115
NKAIN2	1.1932583
NKTR	0.19206476
NLN	0.22210693
NLRC3	0.30985785
NLRC4	0.9657941
NLRC5	1.258419
NLRP1B	0.8176739
NLRP1C-PS	0.2566719
NLRP2	1.0301795
NLRP3	2.4038475
NME2	0.26486588
NME5	1.016655
NMI	0.64681816
NMNAT1	0.89888763
NMRK1	0.6448231
NOBOX	0.7748203

NOD1	0.4226141
NOD2	1.6788135
NOL11	0.3995328
NOL8	0.18684816
NOM1	0.42834616
NOP2	0.3226366
NOP58	0.48072338
NOS2	1.3178439
NOS3	0.5727143
NOTCH1	1.8183808
NOTCH2	0.27620792
NOTUMOS	1.1742849
NOX1	1.6095579
NOXA1	0.5976939
NOXRED1	0.31620455
NPAS1	0.86979914
NPBWR1	0.8813765
NPDC1	0.37068605
NPEPPS	0.48055792
NPFF	0.47062492
NPHP4	0.70894027
NPVF	1.0816376
NPW	1.0046089
NR1H3	1.1594286
NR3C1	0.36478758
NR4A1	1.2403183
NR4A2	0.25953436
NR4A3	2.851803
NR5A2	1.0459614
NR6A1	0.9277737
NRD1	0.43652344
NRG3OS	0.67658424
NRP2	0.87213326
NRROS	0.515049
NRXN3	0.5542133
NSD1	0.31098032
NSF	0.60634613
NSG2	0.5290456
NSMAF	0.3187499
NSMF	0.55938864
NSUN4	0.37228727
NSUN5	0.42744637
NT5C3B	0.3074193
NT5DC3	0.6304393
NT5E	0.5028002

NTNG2	0.7144122
NTSR2	1.1790755
NUB1	0.5246935
NUDT13	0.29360008
NUDT15	0.21971774
NUDT17	1.7239652
NUDT6	0.45641613
NUFIP1	0.7802491
NUFIP2	0.8437042
NUGGC	0.93456316
NUP153	0.31984997
NUP210L	0.6109307
NUP98	0.48609018
NUPR1	2.1318922
NUS1	0.5027585
NXF1	0.35568047
NXPE3	0.61977005
NXPH4	0.43528104
OACYL	0.6231251
OAS1A	1.1575933
OAS1B	0.35501814
OAS1C	0.48803854
OAS1D	0.77216434
OAS1E	0.46877193
OAS1F	1.134522
OAS1G	1.2590001
OAS2	1.8143187
OAS3	0.310225
OASL1	2.8415728
OASL2	1.1569386
OAZ3	0.71122956
OBSCN	0.5141711
OC90	0.42026138
ODAM	1.0544641
ODF3	0.83850884
ODF3L1	2.108632
OGDHL	1.0026445
OGFR	0.62421083
OLFM4	1.5098915
OLFR1000	3.0053635
OLFR1009	0.89439416
OLFR1022	0.9896922
OLFR1028	1.1870136
OLFR1043	1.0018988
OLFR104-PS	1.514807

OLFR1057	0.68938446
OLFR1062	0.85293484
OLFR1085	0.575294
OLFR1106	1.1283379
OLFR1156	1.0121932
OLFR1180	1.187154
OLFR1193	0.8758762
OLFR120	0.6640732
OLFR1202	1.0240216
OLFR1206	1.0013509
OLFR121	1.1234918
OLFR1220	1.5441728
OLFR1221	0.868243
OLFR1225	0.88760257
OLFR1229	0.7288909
OLFR123	1.0331044
OLFR1257	0.9890833
OLFR1258	0.6471584
OLFR1260	1.0803142
OLFR1279	1.1093659
OLFR1280	1.4728861
OLFR1284	1.001385
OLFR1288	1.278846
OLFR1289	0.88792634
OLFR13	1.0394812
OLFR1307	0.26061392
OLFR1311	1.3859308
OLFR1314	1.4833694
OLFR1333	1.4499588
OLFR1336	0.6189735
OLFR1349	1.6126988
OLFR1350	0.9219923
OLFR1372-PS	0.3340819
OLFR1383	0.3504281
OLFR1387	1.2323949
OLFR1396	3.4365938
OLFR1411	0.8143983
OLFR1414	1.1151085
OLFR1433	1.0031283
OLFR1440	0.8663995
OLFR1443	0.45599222
OLFR147	1.0635853
OLFR149	0.98244643
OLFR15	3.3198981
OLFR1509	2.1431887

OLFR152	0.7193079
OLFR159	0.7078564
OLFR164	0.87036014
OLFR199	0.88384867
OLFR2	1.0628207
OLFR215	1.0991285
OLFR224	0.37286568
OLFR231	1.268609
OLFR281	0.82975125
OLFR29-PS1	1.1479988
OLFR3	1.1024137
OLFR324	0.6558249
OLFR330	1.7231565
OLFR353	0.80901194
OLFR354	1.1234407
OLFR356	1.0673342
OLFR368	1.145756
OLFR373	0.94239044
OLFR376	0.8320348
OLFR392	0.68004274
OLFR411	1.2764792
OLFR414	1.1341407
OLFR418-PS1	1.1056054
OLFR424	0.98572516
OLFR426	0.9007616
OLFR434	1.2496567
OLFR441	2.132401
OLFR456	0.39842963
OLFR458	1.0682948
OLFR47	0.9819677
OLFR477	1.5446339
OLFR48	2.2202258
OLFR491	0.92038584
OLFR523	1.2257664
OLFR536	0.71942043
OLFR543	1.9569364
OLFR549	0.3331108
OLFR550	0.8238716
OLFR56	2.6278505
OLFR566	1.696729
OLFR571	1.197577
OLFR572	1.7362394
OLFR576	1.0783582
OLFR617	1.0715561
OLFR619	0.65302205

OLFR620	0.9403653
OLFR64	1.4844265
OLFR640	1.0192475
OLFR658	2.109233
OLFR661	0.91757774
OLFR668	1.2973776
OLFR671	1.2661178
OLFR686	0.82937765
OLFR700	1.1482847
OLFR702	1.2853692
OLFR703	1.377506
OLFR738	0.7843623
OLFR74	0.5796027
OLFR796	0.89326835
OLFR801	0.9611175
OLFR910	0.8826525
OLFR913	0.51155806
OLFR915	0.58466387
OLFR933	1.256599
OLFR934	0.92443204
OLFR98	1.0939362
OLFR994	1.3097968
ONECUT1	0.7074876
OOEP	0.9590261
OPRM1	0.61963844
ORAI1	0.31300116
ORAI2	0.4982729
ORM1	0.76409817
ORM2	0.58158493
OSBPL5	0.49814463
OSBPL9	0.38407278
OSM	0.85199237
OSMR	1.2412467
OTUD3	0.9007802
OTUD4	0.63098145
OTUD6B	0.4483869
OTUD7B	0.31185412
OTULIN	0.28370237
OXCT2B	1.0042968
OXR1	0.20288324
P2RX5	1.539592
P2RY10	1.6617694
P2RY2	2.508089
P4HA1	0.62530494
P4HA2	1.3535478

PAF1	0.21849489
PAG1	1.1609554
PAH	2.755082
PAIP1	0.44162273
PAM	0.35313082
PANK3	1.5271373
PANX1	0.63379335
PAPD7	0.42523336
PAPLN	1.6673913
PAPPA	2.062869
PAPSS2	0.3597064
PAQR3	1.1747518
PAQR8	0.2604475
PARG	0.31753922
PARM1	1.031815
PARP10	1.2814307
PARP11	1.0699658
PARP12	1.2141814
PARP14	1.5927868
PARP3	1.4730477
PARP6	0.53759575
PARP8	0.33869553
PARP9	1.0154681
PARVA	0.2851014
PAX7	1.3254507
PBSN	0.5397332
PCBP3	0.22820067
PCDH15	0.19248796
PCDH19	0.76970315
PCDHGA10	0.6369827
PCED1A	0.7310834
PCGF5	0.893837
PCNXL4	0.699579
PCP2	0.25549006
PDCD1	1.9777141
PDCD1LG2	0.8378844
PDCD4	0.57773924
PDE10A	0.47617078
PDE3A	0.58358884
PDE3B	1.0196333
PDE4B	1.3376775
PDE4C	0.41143227
PDE4D	0.3735335
PDE4DIP	0.62782097
PDE6G	0.49865985

PDE8A	0.19398737
PDF	0.30784512
PDGFA	0.43289042
PDGFB	0.28980398
PDGFRA	0.44651842
PDIA2	0.9292736
PDIA3	0.59329414
PDIA4	0.20174026
PDIA5	0.2629814
PDIA6	0.4985714
PDK4	1.3761587
PDLIM5	0.5490432
PDXK	0.36639166
PDYN	0.47123694
PDZD2	0.7911072
PDZRN4	1.08653
PECAM1	0.3098302
PELI1	1.2341318
PELI3	1.3235369
PER1	0.9437423
PES1	0.9954753
PET100	1.014452
PEX11A	1.1592035
PFKFB3	1.9762721
PFKP	0.52067184
PGA5	0.3769915
PGAM1	0.38075686
PGC	0.7484243
PGLYRP2	0.82760525
PGM3	0.38862038
PGR	0.9716635
PGS1	1.243639
PHC2	0.42492867
PHF11A	1.2805324
PHF11B	1.481246
PHF11C	1.7335076
PHF11D	1.5241823
PHF21B	0.5739529
PHF8	0.35891104
PHGDH	0.21761656
PHLDA1	0.7774863
PHOX2A	4.104809
PHTF1OS	0.5160639
PHTF2	0.7572975
PHXR4	0.43484235

PI15	2.9278307
PICK1	0.2938056
PID1	1.0830474
PIEZO1	1.0531292
PIGC	0.34230042
PIGR	1.1153135
PIK3AP1	1.0657673
PIK3C2A	0.40070224
PIK3C2G	3.252628
PIK3CD	0.36889696
PIK3CG	0.60536575
PIK3IP1	1.4414835
PIK3R3	0.25750852
PIK3R4	0.27770948
PIK3R5	1.3595438
PIK3R6	0.9713063
PIKFYVE	0.43996
PILRA	0.9184704
PIM2	0.39572334
PIM3	0.88595533
PIP5K1A	1.7570362
PIP5KL1	1.8619003
PIPOX	1.0053656
PIRA7	0.24317837
PISD	0.41755104
PISD-PS1	0.6240921
PISD-PS3	0.85625744
PITPNA	0.2519698
PITX3	0.8540714
PJA1	0.8426447
PKD1L3	0.22708225
PKDCC	0.4270773
PKP2	0.6752913
PLA1A	0.7978654
PLA2G1B	5.3349805
PLA2G4B	0.5353942
PLA2R1	0.3914821
PLAA	0.26083803
PLAC8	0.3037076
PLAGL1	0.5390601
PLAUR	1.1805272
PLCB4	0.63749456
PLCD4	0.377625
PLCG1	0.34619188
PLCG2	0.22274733

PLCL1	0.90103793
PLCXD2	1.1053143
PLD5	0.5561614
PLEK	1.1373439
PLEKHA1	1.2568011
PLEKHA3	0.82330847
PLEKHA4	0.7042613
PLEKHD1OS	1.4050894
PLEKHG2	0.4213357
PLEKHG4	1.9932132
PLEKHG6	1.051156
PLEKHH1	0.23944712
PLET1	3.433107
PLK2	0.92404723
PLK3	1.0463896
PLOD2	0.7817445
PLP1	1.2480526
PLP2	0.22733879
PLSCR1	0.73749685
PLXNA3	0.27968884
PLXNC1	0.5244026
PLXND1	0.6968365
PMAIP1	1.2797437
PMEPA1	1.3766122
PMFBP1	0.8601494
PML	0.58847713
PNLIP	10.761618
PNLIPRP1	11.06337
PNLIPRP2	7.56515
PNMA2	1.5878596
PNN	0.51086473
PNO1	0.3803444
PNP2	0.36333466
PNPLA5	1.2899663
POGK	0.8304651
POGLUT1	0.4818573
POLR3E	0.35029602
POLR3F	0.2097764
POM121L12	0.22317386
POMC	1.0380216
POMGNT1	0.50723696
POP1	0.29816055
PORCN	2.306036
POT1B	1.6813388
POU2AF1	1.0133047

POU2F2	0.22243547
POU3F1	0.3692727
POU5F2	0.83288383
PPA1	0.8305998
PPARD	0.36499786
PPCS	0.1874175
PPEF2	0.6175015
PPID	0.3698635
PPM1F	0.3862157
PPM1H	0.58294106
PPM1N	0.76384735
PPP1R10	0.30194354
PPP1R12B	0.31169343
PPP1R16B	0.53539085
PPP1R18	0.3472352
PPP1R1A	1.6804903
PPP1R21	0.7094002
PPP1R3D	0.46433687
PPP1R3F	1.1801586
PPP3CC	0.7646642
PPP3R1	0.6312361
PPP4R1	0.2384181
PPP4R2	0.29078007
PPRC1	0.20979238
PPTC7	0.50877
PPY	1.6916883
PRAMEF8	0.5594144
PRDM1	1.226932
PRDM15	0.28545904
PRDM9	0.9542239
PRDX5	0.27569962
PRDX6	0.24460125
PRELID2	0.4329958
PRF1	0.41717815
PRIMA1	1.0721602
PRKCB	0.33858776
PRKCD	0.32718897
PRKCE	0.55611515
PRKCH	0.81404924
PRKCI	0.1873827
PRKCQ	0.71636057
PRKCZ	0.55051756
PRKD3	0.41633177
PRKG2	0.8888824
PRL2C1	1.9303937

PRL2C5	0.6372814
PRLH	1.779999
PRLR	0.43642306
PRM2	1.0962002
PRMT1	0.5850353
PRMT2	0.7313013
PROC	0.5350957
PROCR	2.1747088
PROK2	4.2645335
PRORSD1	0.51482487
PROSC	0.24804258
PRP2	0.44273472
PRPF31	0.36229277
PRPF38B	0.5551486
PRPS1L1	1.1237183
PRPSAP1	0.26855755
PRR15L	0.73797035
PRR16	0.94182014
PRR18	1.5412519
PRR9	0.4426496
PRSS1	9.876165
PRSS12	0.30852842
PRSS2	9.796787
PRSS22	1.5038624
PRSS23	1.6182871
PRSS23OS	1.5626884
PRSS28	0.36679983
PRSS29	0.49592495
PRSS32	1.5816374
PRSS36	0.7019565
PRSS48	0.45872688
PRSS51	0.56897783
PRSS54	0.811151
PRSS56	0.45703197
PRSS8	1.142669
PRUNE2	0.5125854
PSEN2	0.33718872
PSG16	0.7560668
PSG19	0.9795759
PSMA6	0.29688787
PSMA7	0.23813677
PSMB10	0.31059694
PSME2B	0.35141563
PSTPIP2	0.8655772
PTAFR	1.825398

PTF1A	2.0939167
PTGDR2	0.27877164
PTGS2	4.569454
PTHLH	1.7801924
PTPN1	0.86186457
PTPN13	0.44374275
PTPN2	0.5313597
PTPN22	1.3986869
PTPN23	0.7461972
PTPRH	1.9903934
PTPRK	0.32387495
PTPRM	1.0080357
PTPRU	0.29331708
PTPRZ1	0.4652865
PTRH1	0.64309883
PTX3	4.880314
PUS3	0.46812105
PUS7	0.48473978
PUS7L	0.66038656
PVALB	1.7597058
PVR	1.6749706
PVRL2	0.6657543
PWP1	0.49908495
PWP2	0.5022721
PYDC3	1.68257
PYDC4	2.6282473
PYHIN1	1.481081
PYY	0.6504977
PZP	1.0802581
QSOX1	0.48210955
QTRT1	0.28616
QTRTD1	0.39442587
RAB11FIP1	0.9761472
RAB11FIP2	0.43757558
RAB12	0.18535948
RAB15	1.5368428
RAB19	0.24502182
RAB20	2.0749683
RAB27A	0.4907751
RAB32	0.8068795
RAB33B	0.2090764
RAB3C	0.7253237
RAB3IP	0.46318388
RAB8B	0.22963667
RABEP2	0.875443

RABGAP1	0.5241926
RABIF	0.54617643
RAC3	0.50417805
RAD21L	1.2034137
RAI14	0.5507064
RALGAPA1	0.25176334
RALGDS	0.7871599
RAMP3	1.7213168
RAP1B	0.2570758
RAP1GAP2	0.74735117
RAP1GDS1	0.51752615
RAP2A	0.6498461
RAP2B	1.0386667
RAPGEF2	1.5914574
RAPGEF5	0.70726967
RAPGEF6	0.5820055
RAPH1	0.2669506
RASA2	0.7406697
RASA4	0.5160818
RASAL3	0.5621686
RASD1	2.2318583
RASGEF1A	0.57660294
RASGEF1B	1.6640015
RASGRP1	0.48708677
RASIP1	1.4814782
RASSF1	0.26624966
RASSF2	0.26836205
RASSF6	0.90020347
RAVER2	1.8449259
RBAK	0.48583126
RBM12B2	0.3045249
RBM17	0.23766088
RBM18	0.2880988
RBM19	0.2984929
RBM26	0.31414557
RBM28	0.49181223
RBM43	0.47595215
RBM46OS	0.9830313
RBM47	0.33300972
RBM5	0.26064825
RBMS1	0.76902723
RBMS3	0.45391798
RBPJ	0.28818512
RBPJL	1.376322
RCBTB1	0.32126617

RCN1	0.41011238
RCSD1	0.57792044
RDH10	0.46208668
RDH5	0.32887316
REEP3	0.22949505
REG1	12.62068
REG2	11.986425
REG3A	9.497173
REG3B	11.766886
REG3D	7.6326466
REG3G	9.979157
RELA	0.70981216
RELB	1.4534655
REPIN1	0.29902315
RERGL	1.0620048
REST	0.67314243
RET	1.0565066
REXO4	0.62813616
RFESD	1.372509
RFPL4	1.0631294
RFT1	0.24167156
RFTN1	0.7956829
RFX5	0.35907388
RGMA	2.6202168
RGP1	0.35390496
RGS1	2.580309
RGS11	2.760117
RGS13	1.3001661
RGS16	1.2148533
RGS4	1.9788551
RGS6	0.5523603
RGS9	1.015255
RHBDF1	0.6752691
RHBDF2	0.5397358
RHOB	0.6650605
RHOC	1.378025
RHOQ	0.40303946
RHOU	1.0370197
RHOV	0.9270532
RHOX2C	1.6452737
RHOX4E	1.0501759
RHOX4G	1.1961629
RHOX8	0.8178811
RHPN1	0.3219123
RIMKLB	0.8628378

RIMS2	1.4199231
RIN3	0.87229276
RIOK2	0.36854696
RIPK2	0.97716165
RIPK4	0.9972913
RLF	0.9311938
RLIM	0.5459614
RLTPR	0.4379611
RMRP	0.3890562
RN18S	0.6742177
RNASE1	8.002242
RNASE9	1.275455
RNASET2A	0.29032278
RND1	2.5639658
RNF103	0.32808447
RNF111	0.25730228
RNF112	1.1587408
RNF114	0.7524471
RNF121	0.52270555
RNF122	1.4389231
RNF125	0.5150542
RNF149	1.0227394
RNF151	1.1246984
RNF157	1.123538
RNF183	1.1767921
RNF185	0.41545248
RNF19B	1.1190305
RNF2	0.3568492
RNF213	1.7118602
RNF217	0.31403947
RNF31	0.39386272
RNF44	0.23048687
RNF6	0.2772231
RNU1B6	0.99001837
ROBO1	0.37750292
ROBO4	0.30805826
ROR2	1.2156935
RPAP3	0.28046703
RPGRIP1	0.7050414
RPL22L1	0.4492793
RPL39L	1.124917
RPL4	0.24607229
RPP38	0.33938837
RPP40	0.36857963
RPS15A-PS6	0.21790242

RPS4L	0.23440075
RPS6KC1	0.8580439
RPTOROS	0.43075824
RRAD	1.0524979
RRAS2	0.622684
RRBP1	0.26012135
RRNAD1	0.37885618
RRP12	0.5801625
RRP15	0.38360214
RRS1	0.7695837
RS1	1.400435
RS5-8S1	3.5988007
RSAD1	0.997803
RSAD2	0.81998396
RSG1	1.0878444
RSL1	0.7426009
RSLCAN18	0.35254622
RTBDN	0.5782969
RTDR1	0.65130544
RTP4	1.7671475
RUFY2	0.78381777
RUFY3	0.48268795
RUNDC1	0.35818768
RUNDC3B	2.0037508
RUNX3	0.45424914
RXFP1	0.6582446
RXFP2	1.0395896
RXFP3	0.67452574
RYBP	0.39386463
RYR3	1.6369071
S100A4	0.17922688
S100A8	0.535254
S100PBP	0.39474058
S1PR1	0.22872972
S1PR2	0.39367437
S1PR3	0.4662633
SAA1	3.8346114
SAA2	6.7606974
SAA3	4.195894
SAA4	0.2892053
SAG	1.365694
SAMD4B	0.6204052
SAMD8	0.9310303
SAMD9L	0.9072528
SAMHD1	0.35956097

SAMSN1	1.5295138
SAMT4	1.1312013
SAP18	0.26271725
SASH1	0.6464467
SATB1	1.3931184
SAV1	0.5989008
SAYSD1	1.0936413
SBDS	0.6672301
SBNO2	0.93939734
SCAF8	0.20798588
SCD3	0.46453476
SCGB1B30	0.79029584
SCGB2B2	0.9699445
SCGN	0.94696975
SCHIP1	0.36197805
SCIMP	0.61655235
SCIN	1.1254554
SCML4	1.4041767
SCN11A	1.4346752
SCN3A	0.75200176
SCN3B	1.1425986
SCN4B	0.5924897
SCN5A	0.74988604
SCO2	0.37598085
SCRG1	0.47905326
SCRT1	1.9987218
SCX	0.37482882
SCYL2	0.3562131
SDAD1	1.6334591
SDC4	3.5028167
SDCBP2	0.24615693
SDR42E1	0.61666775
SEBOX	0.8134084
SEC1	0.7001674
SEC16A	0.33043623
SEC24A	0.5232351
SEC24B	0.47856045
SEC24C	0.28155518
SEC31A	0.2004013
SEC63	0.3775487
SECISBP2	0.3556695
SECISBP2L	0.865376
SEL1L	0.23208141
SELE	3.420521
SELL	1.0155458

SELP	0.53108454
SEMA3A	1.4363332
SEMA3F	0.32876158
SEMA3G	0.58904886
SEMA4A	0.6285367
SEMA4D	0.41111565
SEMA7A	0.82956505
SEPW1	0.3614092
SERP1	0.5551424
SERPINA10	1.6342707
SERPINA1A	0.3594284
SERPINA1C	0.35271263
SERPINA1D	0.24810648
SERPINA1E	0.2571416
SERPINA3B	0.32712364
SERPINA3C	1.7441998
SERPINA3F	2.254272
SERPINA3G	1.0286345
SERPINA3I	1.553926
SERPINA3K	1.1513762
SERPINA3M	1.2042713
SERPINA3N	2.3670697
SERPINB1A	0.24986553
SERPINB6B	0.73109484
SERPINB9	0.78048897
SERPINB9E	1.0147123
SERPINE1	4.005005
SERPINE2	0.53162074
SERPING1	0.23090696
SERPINI1	1.1223245
SERPINI2	7.0431514
SERTAD3	1.5111213
SESN3	0.66267204
SESTD1	0.23010421
SET	0.32551098
SETBP1	0.31889558
SETD2	0.1952982
SETD4	0.8333359
SETDB2	0.9776988
SEZ6L2	0.5956242
SF3B1	0.47066498
SFI1	2.20676
SFMBT2	0.897521
SFPQ	0.22316885
SFTPD	0.9079404

SFXN2	0.7136011
SGCB	0.42536688
SGK1	1.2062683
SGMS1	0.34913254
SGPL1	0.2019968
SGPP2	0.8390155
SH2B2	0.91964483
SH2B3	0.22634459
SH2D1A	0.5877533
SH2D1B2	1.1900203
SH2D2A	1.0099335
SH3BP2	0.5838003
SH3BP4	0.43780613
SH3BP5	0.65163517
SH3GL3	1.2963939
SH3KBP1	0.22881126
SH3PXD2B	0.75931454
SHB	0.22855091
SHISA3	4.2471404
SHISA5	0.90438366
SHMT1	0.7229593
SIAH2	1.4793878
SIDT1	0.48968792
SIGLECE	0.33372974
SIK1	0.97158337
SIK3	0.61460114
SIKE1	0.5080414
SIMC1	0.6935458
SIPA1L1	0.9238796
SIS	1.2109256
SIT1	0.47360277
SKAP1	0.47094393
SKAP2	0.5423322
SKIL	0.9758215
SKOR1	3.0807588
SLA	0.82619
SLA2	0.3469231
SLC10A3	0.30827284
SLC10A6	3.027766
SLC12A3	1.6918383
SLC12A5	0.46065164
SLC15A2	1.7418747
SLC15A3	1.5863318
SLC15A4	0.51553726
SLC16A1	0.2903707

SLC16A3	0.9522443
SLC17A5	0.29524565
SLC17A9	0.6369448
SLC18A1	0.64471984
SLC22A13B-F	0.28273797
SLC22A15	0.2053988
SLC22A30	1.1841211
SLC23A2	0.7702966
SLC25A19	0.8827534
SLC25A20	0.69845057
SLC25A22	1.2930758
SLC25A23	0.37447906
SLC25A25	0.64568853
SLC25A3	0.62834835
SLC25A33	0.3670535
SLC25A36	0.49317884
SLC25A41	1.3998814
SLC25A46	0.39721394
SLC25A53	1.3612907
SLC26A6	0.4826963
SLC26A8	0.766057
SLC26A9	1.8833036
SLC27A2	1.2343771
SLC28A1	0.2300663
SLC28A2	0.75312424
SLC2A1	1.0148916
SLC2A12	0.5799608
SLC2A4RG-PS	0.83795977
SLC2A6	1.9430213
SLC2A7	1.2658024
SLC30A3	1.1321797
SLC30A4	0.84688044
SLC30A6	0.3872838
SLC30A7	0.2655847
SLC31A2	0.72156096
SLC35A1	0.20097542
SLC35B1	0.29824638
SLC35F1	0.91510916
SLC35F2	1.0676327
SLC35G3	0.74688625
SLC36A2	0.39295626
SLC37A3	0.30166245
SLC38A3	0.9162514
SLC39A1	0.63754654
SLC39A14	2.3160214

SLC39A2	0.31385112
SLC39A6	0.6606889
SLC39A7	0.25202417
SLC41A1	1.0820637
SLC4A4	1.4100673
SLC4A7	0.6676221
SLC4A8	0.9144907
SLC50A1	0.23382187
SLC5A1	1.2230365
SLC5A3	1.4586663
SLC5A6	0.5397978
SLC6A20B	1.1628528
SLC6A7	0.93356276
SLC7A11	1.0168555
SLC7A2	1.3314414
SLC7A6	1.0618637
SLC7A6OS	0.5737176
SLC8A3	0.72036076
SLC9A8	0.41940165
SLCO1B2	0.84140635
SLCO3A1	0.93996716
SLCO5A1	1.5482578
SLFN1	1.2942896
SLFN10-PS	0.9212761
SLFN2	1.105464
SLFN3	0.34634113
SLFN4	2.1105037
SLFN8	1.7003131
SLFN9	0.9468589
SLMO1	0.5044217
SMAD1	1.2172618
SMAD2	0.54293585
SMAD4	0.37993002
SMAD7	1.0667577
SMCHD1	0.40491152
SMCR8	0.27674103
SMEK2	0.23460436
SMG1	0.18773794
SMIM22	1.4696102
SMIM3	0.62444067
SMPDL3B	0.46589375
SMPX	1.3030794
SMR3A	0.37950015
SMUG1	0.7584176
SMURF1	0.39432716

SMURF2	0.32484102
SMYD2	0.20624876
SNAI3	0.38219452
SNAP23	0.39886093
SNAP25	0.28423405
SNAPC1	0.9053631
SNAPC3	0.38524723
SNAPC4	0.33671093
SNAPC5	0.26611662
SNHG12	0.47399855
SNHG6	0.48388004
SNHG7	0.22809172
SNORA74A	0.47373676
SNRNP35	0.6033287
SNRNP48	0.37157726
SNRPA1	0.20280981
SNTA1	1.4025116
SNTB2	0.98213434
SNX10	1.1313438
SNX16	0.39564514
SNX18	1.1177502
SNX19	0.22755766
SNX20	0.84163046
SNX29	0.89128256
SNX7	0.614207
SNX8	0.5466256
SOCS1	2.3679256
SOCS2	0.20260286
SOCS3	3.2041326
SOD2	1.8772111
SOD3	0.545506
SOGA1	0.51023054
SOGA3	1.0264168
SOHLH1	1.3877635
SOHLH2	0.594069
SON	0.2254448
SORBS1	0.7206416
SORL1	1.3198628
SOS1	1.6331842
SOSTDC1	0.8552153
SOWAHC	0.98603106
SOX5	1.4814205
SOX7	1.4943395
SOX9	1.3813317
SP100	0.5371723

SP140	0.51891756
SP5	0.90358806
SP7	1.1677413
SPAG11A	0.6755049
SPAG16	1.5042009
SPAG9	0.33635426
SPATA1	0.3540802
SPATA13	0.29392433
SPATA21	1.5821846
SPATA5	0.3364854
SPATA5L1	1.3584335
SPATS2	0.7063203
SPCS2	0.36689663
SPDEF	1.5443966
SPDYA	0.53593874
SPDYB	0.9439826
SPEER3	1.0449393
SPEER5-PS1	0.8228235
SPEER9-PS1	0.89404106
SPEF2	0.7090101
SPI1	0.5434189
SPIB	0.23071003
SPIC	1.6042042
SPIDR	0.36427498
SPIN1	0.5937724
SPINK12	0.9078772
SPINK3	8.394373
SPINK4	1.3048742
SPINK6	0.8423848
SPNS1	0.20964003
SPOCD1	0.7296922
SPON1	0.70943475
SPPL2A	0.76049757
SPRED1	0.96666336
SPRR2D	4.943298
SPRY1	0.49174142
SPRY2	1.2943351
SPSB1	0.787411
SPTAN1	0.24248743
SPTLC1	0.50631046
SPTLC2	0.23405647
SPTSSB	0.8381705
SRFBP1	0.44678307
SRGAP1	0.64755774
SRGN	0.30866385

SRP54A	0.43763542
SRPRB	0.31659317
SRY	1.74722
SS18	0.2918725
SSH2	0.8149843
SSH3	0.6818576
SSPO	0.659369
SSU72	0.34218788
SSX9	0.89581823
SSXB10	0.24096584
ST13	0.5797415
ST3GAL1	0.27092457
ST6GALNAC2	1.5119858
ST6GALNAC4	0.5764866
STAC	0.965642
STAC3	1.0220447
STAM2	2.5545406
STAMOS	1.265522
STAP1	0.21173906
STAP2	0.9935541
STARD7	0.28129578
STAT1	1.0214672
STAT2	1.2864275
STAT3	1.005301
STAT4	1.2445035
STAT6	0.3228054
STEAP1	4.591388
STEAP2	2.082585
STEAP4	2.4804802
STFA1	1.7439575
STFA2	1.7673178
STFA2L1	1.4761219
STFA3	1.7152925
STIP1	0.28384018
STK11IP	0.34606028
STK17B	0.4546485
STK19	0.49596977
STK33	0.7008946
STK39	0.40804386
STK40	0.8055997
STMN3	0.9330113
STON1	1.6817749
STRA6	2.072874
STRIP1	0.5739198
STRIP2	0.7042055

STX11	0.25074244
STX16	0.5435262
STX1B	0.46297932
STX6	0.2817192
STX7	0.23012304
STXBP3A	0.46006584
STXBP3B	0.77747107
STXBP5L	1.06351
STYX	0.27498055
SUCO	0.4158857
SUGT1	0.32975435
SULT2A7	0.8477979
SUPT20	0.49258876
SURF4	0.46232605
SURF6	0.8754711
SUSD2	0.5637469
SUSD4	0.26990175
SUV420H1	0.7031636
SWI5	0.9236808
SYCE1L	1.3817911
SYCE3	1.3007665
SYCN	9.631811
SYF2	0.5357399
SYK	1.0386329
SYNJ1	0.30799675
SYNJ2BP	0.6785202
SYNPO	1.3000395
SYPL	0.2825203
SYPL2	0.443285
SYT1	0.26258516
SYT11	1.5823212
SYT13	1.8378472
SYT8	0.99218583
SYTL3	0.8264413
SYVN1	0.24212933
TAAR7B	2.6743498
TADA2B	0.7365308
TAF7L	0.47745323
TAGAP	0.6665182
TAGLN	0.55763435
TANK	0.25576353
TAOK1	0.6679287
TAP1	0.5404415
TAP2	0.71951675
TAPBP	0.37796688

TARM1	1.7989738
TARS	0.4261427
TARS2	0.2763443
TAS2R131	2.6550784
TAS2R134	1.6610272
TAS2R137	0.9943998
TAT	0.27651596
TBC1D1	1.0423021
TBC1D2	0.30889225
TBC1D20	0.28136253
TBC1D22BOS	0.8559902
TBC1D23	0.5227332
TBC1D30	1.1229541
TBC1D9	1.5867081
TBCA	0.2393856
TBCK	0.928087
TBK1	0.35324144
TBRG1	0.44704008
TBX10	0.5083895
TBX15	0.86949444
TBX21	0.9588051
TBX22	1.0832891
TBX3	1.4263334
TCAP	1.4053705
TCF4	0.6383805
TCF7	0.88241386
TCFL5	0.855016
TCL1B2	1.140187
TCOF1	0.44317818
TCP10B	1.9812121
TCRG-V1	0.24881792
TCSTV3	0.69667196
TCTE1	0.54022527
TDG	0.70447063
TDGF1	0.6805606
TDRD7	1.2943349
TDRP	0.5093274
TEAD1	0.7447119
TEAD2	0.21081066
TEAD4	0.67739487
TECPR1	0.20403576
TEPP	0.5244491
TERC	1.9330907
TERF2	0.3322959
TES	1.1384745

TESK1	0.619771
TESPA1	1.1876063
TET2	0.5283699
TEX101	0.55795574
TEX11	1.2568433
TEX12	1.1569533
TEX13	0.8219199
TEX19.2	1.6425116
TEX36	1.2600121
TFAP2D	1.5566077
TFB2M	0.46085072
TFEC	0.36067867
TFF2	8.051569
TFG	0.2106471
TFPI	0.5640087
TGFA	0.4734435
TGFBR2	0.34888315
TGIF1	1.1214094
TGIF2	0.77251077
TGM2	1.2889247
TGOLN1	0.39060497
TGOLN2	0.40442467
TGTP1	1.280437
TGTP2	1.1508946
THA1	1.674314
THADA	0.3460176
THBS4	1.1640158
THEMIS	0.55193806
THEMIS3	0.89592123
THYN1	0.25545168
TIFA	1.4428172
TIGIT	1.583127
TIMM10	0.43017578
TIMM9	0.2859664
TIMP1	2.8717203
TIMP3	0.9975705
TIPARP	1.489234
TIRAP	0.3677044
TKTL2	1.1054447
TLE1	0.82197523
TLE3	0.5418415
TLR2	2.0033488
TLR6	1.0655465
TLR7	1.0220017
TM2D1	0.44204235

TM2D3	0.36910295
TM4SF4	0.5472486
TM6SF2	1.2164125
TMA16	2.0368323
TMBIM1	0.69890594
TMC3	1.4841695
TMC5	2.098691
TMCO4	1.0633183
TMED10	0.2202406
TMED11	0.70908165
TMED6	2.6132224
TMED7	0.4896798
TMED8	0.41018105
TMED9	0.25397015
TMEM102	0.5405295
TMEM108	0.31049156
TMEM11	0.38867807
TMEM121	0.43134165
TMEM123	0.55040455
TMEM131	0.39748716
TMEM132E	3.159606
TMEM135	0.27333784
TMEM140	0.4056549
TMEM158	0.34993076
TMEM159	0.3523841
TMEM161B	0.25725174
TMEM163	1.0001183
TMEM167	0.18294811
TMEM169	1.8621485
TMEM173	0.9183345
TMEM181A	0.21310711
TMEM181B-I	1.8327174
TMEM184B	0.9914384
TMEM184C	0.348382
TMEM185B	0.63924646
TMEM192	0.21891356
TMEM2	1.4428306
TMEM200C	1.1285625
TMEM202	0.49871135
TMEM230	0.31587362
TMEM247	0.602669
TMEM252	1.5903778
TMEM260	0.22040486
TMEM30B	0.96116114
TMEM38B	0.4305749

TMEM39A	0.7134867
TMEM42	0.26715088
TMEM50B	0.5923643
TMEM63B	0.27239037
TMEM63C	1.970104
TMEM66	0.19529295
TMEM68	0.572597
TMEM74BOS	0.8190317
TMEM87A	0.40375805
TMEM87B	0.46201468
TMEM89	0.7601538
TMEM8C	3.6244078
TMF1	0.4472227
TMIE	0.7063296
TMOD4	1.1758323
TMPRSS2	1.7750075
TMSB15B1	0.5248561
TMUB1	0.44324827
TNF	4.206231
TNFAIP1	0.87605953
TNFAIP2	0.75712395
TNFAIP3	3.3993626
TNFAIP6	2.8871274
TNFAIP8L1	0.32443666
TNFAIP8L3	1.1385016
TNFRSF10B	1.2904685
TNFRSF12A	2.5400085
TNFRSF13C	0.46550798
TNFRSF18	1.2053876
TNFRSF1B	0.32127953
TNFRSF22	0.32639933
TNFRSF23	0.37711906
TNFRSF25	1.1673217
TNFRSF4	1.586935
TNFRSF9	0.23494434
TNFSF10	1.0401459
TNFSF11	0.4711504
TNFSF14	0.30798817
TNFSF15	3.2862554
TNFSF8	1.4789319
TNFSF9	2.413536
TNIP1	1.9032893
TNIP2	1.1229725
TNIP3	3.878213
TNKS2	0.30310297

TNN	1.4656067
TNP2	1.8870356
TNPO3	0.85857296
TNRC6A	0.20483255
TNS4	0.582108
TOMM20L	0.7451129
TOMM7	0.24097824
TOMM70A	0.3337903
TOP1MT	0.35088873
TOR1AIP1	0.2655387
TOR1AIP2	0.5141082
TOR3A	1.2259092
TOX2	0.9591422
TPD52	0.8211584
TPM4	0.40240002
TPP2	0.26506758
TRA2A	0.3935256
TRAF1	2.019199
TRAF2	0.491055
TRAF3	0.47019863
TRAF3IP2	0.34356356
TRAF3IP3	0.23311234
TRAF6	0.3756194
TRAFD1	0.6526408
TRAIP	0.27420473
TRAM2	0.47190857
TRAPPC3	0.47238445
TRAPPC4	0.20845842
TRAPPC8	0.27173543
TRAT1	0.6260636
TRAV14D-3-[	1.5672114
TRAV3N-3	1.5900626
TRCG1	0.75939226
TREM1	1.6010733
TREM3	0.4642043
TREML2	0.6526952
TREX1	1.1274133
TRIB1	1.1934814
TRIM11	0.5931568
TRIM12C	1.0328708
TRIM13	0.35990882
TRIM14	0.5160761
TRIM21	1.4279552
TRIM25	0.37845087
TRIM26	0.94758797

TRIM30A	1.7075391
TRIM30B	1.1178246
TRIM30C	2.2765975
TRIM30D	1.4879975
TRIM32	0.3594694
TRIM34A	1.0141044
TRIM34B	0.736084
TRIM35	0.9267101
TRIM36	1.6847191
TRIM38	1.0126052
TRIM41	0.25213432
TRIM43B	0.90643954
TRIM44	0.5605154
TRIM47	0.637908
TRIM5	0.25739026
TRIM50	3.066069
TRIM54	1.0937827
TRIM56	0.72844124
TRIM6	0.6284199
TRIM65	0.44441342
TRIM68	1.2021847
TRIM69	1.4408586
TRIP10	0.82314205
TRMT1	0.53849125
TRMT10A	0.25695252
TRMT2A	0.2561288
TRMT6	0.33665705
TRMT61A	0.6831322
TRNP1	1.5839815
TRP53I11	0.40674257
TRP53INP2	0.6737361
TRP53RK	0.19562817
TRP63	0.51197433
TRPA1	0.89750314
TRY4	10.361959
TRY5	10.187517
TSC22D3	1.0847745
TSEN54	0.27522564
TSGA13	1.9796567
TSKU	0.4824462
TSNAXIP1	1.1369419
TSPEAR	0.83187366
TSR1	0.5412717
TSR2	0.7601304
TSR3	0.36088276

TSTD1	1.178083
TSTD2	0.20539474
TSX	1.35396
TTC13	0.2493043
TTC14	0.2608366
TTC19	0.2767477
TTC30A1	0.30497313
TTC37	0.9531028
TTC39B	0.74762034
TTI2	0.21714497
TTL	0.53932047
TTLL3	1.0171871
TTLL4	0.3294282
TTLL7	0.77294636
TTLL8	0.8526764
TTLL9	0.38758254
TTPAL	0.5739498
TTR	1.6956773
TTYH1	0.8710282
TUBA1A	0.3510356
TUBB6	1.5336962
TULP4	0.81456375
TWIST2	0.66649246
TXK	1.327766
TXLNB	0.71582294
TXLNG	0.4434085
TXN1	0.23483467
TXNDC16	0.23263264
TXNDC8	0.63780046
TXNRD1	0.8839083
TXNRD3	0.37768698
TYK2	0.29271603
TYW5	0.78272724
UBA7	0.38578796
UBD	0.544034
UBE2D3	0.22500181
UBE2E1	0.35866308
UBE2E2	0.48245525
UBE2F	0.535275
UBE2J1	0.30886936
UBE2Q1	0.2629509
UBE2Q2	0.28347397
UBE2W	0.33849764
UBFD1	0.32929134
UBN1	0.17523813

UBOX5	0.4818735
UBQLN1	0.86011267
UCHL3	1.2355652
UCKL1	0.26873922
UCP1	1.1210771
UFD1L	0.18024874
UFM1	0.24461365
UGGT1	0.40661716
UGT2A1	4.138687
UGT8A	0.6098757
UNC45A	0.4389305
UNC79	1.2352858
UPP1	2.61094
URAD	1.0207827
URAH	1.194932
USH1G	0.37141657
USP16	0.6317196
USP17LC	0.32457328
USP17LE	1.7066586
USP18	2.787107
USP42	0.9329939
USP43	0.293113
USPL1	0.3556428
UTF1	0.5911679
UTP11L	0.5935254
UTP14A	0.5541792
UTP20	0.45359755
UTP6	0.45291948
UTRN	0.43622208
VAMP5	0.22088051
VAPA	0.27008915
VASH1	1.1927447
VASP	0.4667759
VAV1	0.8113737
VAX1	1.524631
VCPIP1	0.35449362
VDR	2.1908069
VGF	3.262848
VGLL4	0.4675088
VLDLR	0.58702755
VMN1R13	2.1572523
VMN1R151	1.1544158
VMN1R167	0.82591057
VMN1R197	1.7723374
VMN1R212	1.3264561

VMN1R59	1.0552759
VMN1R62	0.68275285
VMN1R87	1.0792558
VMN2R104	1.5557499
VMN2R11	2.017654
VMN2R111	0.95167184
VMN2R115	0.86032987
VMN2R13	1.064388
VMN2R3	0.3733139
VMN2R52	1.30445
VMN2R55	1.8674293
VMN2R58	0.6821103
VMN2R62	1.0682254
VMN2R65	1.0769091
VMN2R73	1.007586
VMN2R88	0.7803569
VMN2R96	1.072691
VMN2R97	0.8824072
VMP1	1.1159692
VNN3	4.3201632
VPS16	0.19133425
VPS37A	0.6456995
VPS4B	0.2745328
VPS54	0.28662157
VSIG2	0.40814018
VSTM2B	0.8196373
VTI1B	0.73892355
VTN	4.2276893
VWA5A	0.4111085
VWA5B2	0.7853341
WARS	0.47028065
WARS2	0.5050802
WBP1L	0.18827057
WBSCR28	0.7300782
WDR1	0.93589115
WDR3	0.17410612
WDR34	0.5257964
WDR38	0.5061226
WDR43	0.33330488
WDR45B	0.6545863
WDR47	0.4804926
WDR52	0.69373703
WDR59	0.55509233
WDR78	1.5270755
WDR92	1.8885145

WFDC15A	1.3378515
WFDC17	2.1764169
WFDC18	2.8883815
WFDC5	0.90551805
WFDC6A	0.9071727
WFDC6B	1.2815738
WFS1	0.3958521
WHRN	1.5914421
WHSC1L1	0.35048628
WISP1	0.5607705
WNK3	1.1892185
WNT1	0.5014646
WNT11	0.49293876
WNT6	0.48694038
WNT9B	0.8888123
WSB1	0.21068525
WT1OS	0.59979105
WWC1	0.34693813
XAF1	0.41461945
XBP1	0.6192384
XCL1	0.30977297
XIAP	0.26028538
XKR8	1.4182844
XLR3B	0.80622005
XLR4A	0.6611085
XLR4B	0.69215107
XLR5B	0.8187103
XNTRPC	0.40538502
XRN2	0.18547344
YAE1D1	0.5478873
YAP1	0.41791344
YJEFN3	0.35608172
YRDC	0.3842969
YTHDC1	0.6016803
YTHDF2	0.24483109
YY1	0.36429453
ZAN	2.0030196
ZAP70	0.7234001
ZBED5	0.60496545
ZBED6	0.37788653
ZBP1	1.4276052
ZBTB12	1.1144972
ZBTB16	1.422267
ZBTB20	0.5689573
ZBTB21	0.7836549

ZBTB26	0.8732896
ZBTB37	0.6252749
ZBTB40	0.57568884
ZBTB43	0.3029704
ZBTB5	0.5247016
ZBTB9	0.6805639
ZC3H12A	1.9121361
ZC3H12C	0.7365694
ZC3H13	0.21978092
ZC3H3	0.24971771
ZC3H7A	0.32303333
ZC3HAV1	0.6540966
ZCCHC11	0.7710252
ZCCHC12	1.0620618
ZCCHC18	1.3469305
ZCCHC2	1.2739921
ZCCHC7	0.4431777
ZCWPW2	0.9447694
ZDBF2	0.88102365
ZDHHC18	0.7582636
ZDHHC23	0.33907962
ZFAND5	0.2290926
ZFP1	1.4217095
ZFP105	0.31442785
ZFP112	1.3740983
ZFP12	0.5113859
ZFP131	0.54218817
ZFP169	0.4919572
ZFP182	0.35578632
ZFP189	0.26617146
ZFP202	0.61974216
ZFP239	0.2590711
ZFP26	0.3358884
ZFP260	0.6997423
ZFP263	1.0970826
ZFP27	0.26538777
ZFP275	0.40344763
ZFP276	0.23610497
ZFP281	1.0765023
ZFP287	0.4222381
ZFP292	0.691463
ZFP318	0.46444368
ZFP329	0.25355244
ZFP36	1.6848254
ZFP365	1.1179717

ZFP366	2.087771
ZFP36L1	0.6536398
ZFP36L2	0.85631084
ZFP36L3	0.8617685
ZFP386	0.4621253
ZFP397	0.56007385
ZFP420	0.71415305
ZFP426	0.23604822
ZFP428	0.3117833
ZFP429	0.9864707
ZFP446	0.66986084
ZFP456	0.85452485
ZFP458	0.8504536
ZFP46	0.65932655
ZFP462	0.47106886
ZFP51	0.40861416
ZFP513	0.6150656
ZFP516	0.72469544
ZFP52	1.023344
ZFP523	0.2679472
ZFP53	2.0738049
ZFP560	0.30468678
ZFP566	0.37120247
ZFP568	0.2542138
ZFP58	0.23476577
ZFP583	0.6102762
ZFP593	0.93474245
ZFP595	0.23188639
ZFP597	0.5306432
ZFP605	0.45581818
ZFP606	0.4960382
ZFP607	0.43612528
ZFP612	0.7245004
ZFP62	0.5013609
ZFP622	0.29759026
ZFP64	0.33572388
ZFP65	0.26010585
ZFP655	0.26272058
ZFP658	0.5803385
ZFP661	0.46748543
ZFP663	0.4266534
ZFP691	0.806685
ZFP697	1.7704821
ZFP7	0.18964052
ZFP719	0.3213396

ZFP740	0.45243788
ZFP747	0.5388527
ZFP748	0.57112765
ZFP777	0.3071642
ZFP783	1.039084
ZFP784	0.39028597
ZFP790	1.2763739
ZFP809	0.49682808
ZFP811	3.5352287
ZFP82	0.53624296
ZFP831	0.21790957
ZFP839	0.99315476
ZFP84	0.468637
ZFP850	0.41566825
ZFP868	0.6180725
ZFP874A	0.3013482
ZFP882	0.20945191
ZFP92	0.3191731
ZFP933	0.3906815
ZFP935	0.33854342
ZFP937	0.54798484
ZFP94	0.63321304
ZFP943	0.38233662
ZFP946	0.42851305
ZFP955B	0.30514145
ZFP958	0.7947781
ZG16	5.9115214
ZHX2	1.33459
ZKSCAN2	0.95640755
ZKSCAN7	0.45607376
ZKSCAN8	0.36387444
ZMAT1	0.31842566
ZMIZ1	0.38500452
ZMYND11	0.45750284
ZMYND15	0.68609333
ZNFX1	0.84312344
ZNHIT6	0.54824305
ZNRF1	0.8381939
ZPBP	0.32259417
ZPBP2	0.43201494
ZPLD1	0.95263886
ZRANB2	0.39614582
ZSCAN18	1.3500628
ZSCAN22	0.41968703
ZSCAN4C	0.7210624

ZSWIM2	1.0316076
ZSWIM6	0.3272624
ZSWIM8	0.50638866
ZUFSP	1.3418746
ZXDA	1.3240619
ZXDB	0.6508689
ZXDC	0.4920361
ZYG11B	0.83986616
ZZZ3	0.25240564

Mice_late_spleen_DEgenes_fc

Gene	log2 Fold Change
AAMDC	-0.959487
AATK	-1.1795335
ABCA13	-0.8663774
ABCA3	-0.4408875
ABCA7	-0.7676163
ABCA8A	-2.3726096
ABCA9	-4.3069563
ABCB10	-2.5878873
ABCB4	-1.947712
ABCB6	-2.5275412
ABCB7	-0.8958309
ABCB9	-1.0254898
ABCC3	-1.265307
ABCG2	-1.668158
ABCG3	-2.1026359
ABCG4	-3.339304
ABHD1	-0.6020663
ABHD12	-0.3948851
ABHD14B	-1.2474966
ABHD16A	-0.7127037
ABHD17A	-0.9170342
ABHD4	-0.4889941
ABHD5	-0.3517737
ABHD8	-0.4246254
ABI3	-0.3876023
ABTB1	-1.2796688
ACAA2	-0.851471
ACAD11	-0.3437514
ACADL	-0.3299804
ACAP1	-0.8225737
ACAP3	-1.0450544
ACAT1	-0.362958
ACAT2	-0.3674634
ACAT3	-0.4123292
ACBD4	-0.8428474
ACBD6	-1.1151862
ACD	-0.7990685
ACE	-1.5344667
ACHE	-2.3286572
ACKR1	-3.014629
ACKR2	-0.711751
ACKR4	-3.063198
ACMSD	-4.1245155

ACOT11	-0.7048488
ACOT13	-0.7776275
ACOT6	-0.871203
ACOT8	-0.4750714
ACP1	-1.2716031
ACRBP	-0.5910473
ACSL1	-0.5392094
ACSL6	-1.5493672
ACSS1	-1.2413802
ACSS2	-1.2927206
ACTA2	-0.6357961
ACTL11	-1.1834106
ACTL6A	-0.7002654
ACTR3B	-0.6527035
ACVRL1	-1.4718704
ACYP1	-0.7237701
ADAM10	-0.9380894
ADAM22	-1.4904299
ADAM26A	-0.569732
ADAM26B	-2.6157777
ADAM33	-1.0842116
ADAM6B	-0.7263827
ADAMDEC1	-0.4440551
ADAMTS13	-0.9327958
ADAMTS16	-1.4392741
ADAMTSL1	-1.1385496
ADAMTSL5	-0.8226295
ADCK3	-0.7250359
ADCY1	-4.050106
ADCY5	-1.3072209
ADCY6	-0.9594426
ADCY7	-0.5918665
ADD1	-1.2843075
ADD2	-3.3903766
ADD3	-0.8025408
ADH1	-1.1320901
ADIG	-1.1747761
ADIPOQ	-3.91424
ADIPOR1	-0.5832
ADPGK	-0.6837025
ADRB1	-0.7982235
ADRBK1	-0.5366082
ADSSL1	-0.9068437
AFMID	-1.2775822
AGAP1	-0.6919642

AGAP2	-0.5733433
AGBL1	-2.6472647
AGBL3	-0.9723332
AGFG2	-1.71189
AGMAT	-1.4057283
AGMO	-2.479797
AGO1	-0.5702064
AGO2	-1.3524461
AGPAT4	-0.8288093
AGTPBP1	-0.6307247
AHCTF1	-0.4136424
AHDC1	-1.1023777
AHI1	-0.8110199
AHNAK2	-1.1605628
AHRR	-1.1052563
AHSP	-2.6498618
AIF1	-1.0855689
AIF1L	-0.5498357
AJUBA	-0.9150784
AK1	-1.1111374
AK3	-0.8718395
AK8	-2.6419537
AKAP17B	-0.8554101
AKAP5	-1.4249341
AKAP7	-0.9642477
AKAP8L	-0.7778282
AKIP1	-1.3414173
AKR1B10	-0.9975729
AKR1B7	-1.8801217
AKR1C6	-2.5816336
AKR7A5	-0.8072953
AL118220	-0.5980034
ALAD	-3.2587461
ALAS2	-1.1776681
ALDH18A1	-0.4108987
ALDH1A1	-1.5851316
ALDH1A7	-3.4934924
ALDH2	-0.5776038
ALDH3B2	-2.7959552
ALDH4A1	-0.8117223
ALDOC	-1.3074279
ALG13	-0.3150144
ALG2	-0.3766894
ALKBH7	-0.6723485
ALKBH8	-1.0469594

ALMS1	-1.1620741
ALOX12	-1.2448087
ALOX12E	-1.3992743
ALOX15	-2.2552054
ALOX5	-1.4655704
ALOX5AP	-0.6313667
ALS2CR12	-1.3175373
ALYREF	-0.7521129
AMD2	-0.635922
AMMECR1	-1.3506641
AMPD3	-1.5309591
AMZ1	-1.507544
AMZ2	-0.4351339
ANAPC13	-0.4870815
ANAPC15	-0.4019294
ANAPC5	-0.8522277
ANGPTL2	-2.1024473
ANGPTL6	-0.4810224
ANGPTL7	-3.0931919
ANK	-1.8283486
ANK1	-2.4412398
ANK3	-1.1731873
ANKEF1	-0.6674271
ANKLE1	-3.1750202
ANKMY2	-0.6058683
ANKRD13D	-0.6382031
ANKRD2	-1.5566647
ANKRD22	-0.5567668
ANKRD26	-0.5196369
ANKRD35	-1.4755766
ANKRD50	-0.4386146
ANKRD54	-1.3447027
ANKRD9	-1.6104369
ANKS1B	-0.5188181
ANLN	-1.5578742
ANO1	-1.1765273
ANO10	-0.302012
ANXA9	-0.5629473
AOC1	-1.9579473
AOX1	-0.80532
AP1B1	-0.7967153
AP2A1	-1.2589474
AP2A2	-1.0991306
AP4M1	-1.033819
AP5M1	-0.3749919

APBA1	-0.6877852
APBB1	-0.7166758
APBB1IP	-0.4336114
APC2	-0.8587596
APCDD1	-0.9737067
APEH	-1.5092063
APH1B	-0.697926
APIP	-0.506825
APLF	-1.427372
APLNR	-3.3729587
APLP2	-0.4392824
APOA1BP	-0.6054626
APOA4	-1.5919178
APOBR	-0.6729093
APOC1	-0.7810936
APOF	-0.9177113
APOL10A	-3.6933565
APOL10B	-1.1331067
APOL11B	-2.2986403
APOL7B	-0.6804676
APOL8	-2.4173145
APOO	-0.5241971
AQP1	-2.4059849
AQP11	-1.4012835
AQP2	-2.1740236
AQP9	-1.5917933
ARF5	-1.0785398
ARG1	-0.7696688
ARHGAP18	-1.552094
ARHGAP19	-1.549079
ARHGAP23	-1.307756
ARHGAP33	-2.4959319
ARHGAP39	-1.2568922
ARHGAP4	-1.3420668
ARHGAP6	-0.8613582
ARHGAP9	-0.6145301
ARHGDIB	-0.3769755
ARHGEF1	-0.6241536
ARHGEF10L	-0.4574781
ARHGEF39	-2.0073638
ARHGEF4	-0.9331684
ARHGEF40	-1.3254485
ARHGEF9	-1.1717517
ARID4A	-0.6078811
ARL11	-1.6852555

ARL4A	-1.0086207
ARL4D	-0.5971565
ARL6IP1	-0.6207876
ARMC1	-0.4189134
ARMC2	-1.1543798
ARMC4	-0.6325345
ARMC6	-0.570189
ARMC7	-0.5415525
ARMC9	-1.821157
ARPC1A	-0.4249587
ARRB1	-1.7636971
ARRDC2	-1.374682
ARSB	-1.1903028
ARSI	-1.2135613
ARSK	-1.0571094
ART2B	-1.5565264
ART3	-0.8884149
ART4	-1.9708009
ARTN	-2.7042713
ARVCF	-1.6930308
AS3MT	-0.7690818
ASAP1	-1.2689681
ASB1	-1.6693254
ASB16	-0.9992621
ASB17	-3.5936913
ASB2	-1.055028
ASB6	-0.5435877
ASCC1	-0.6632149
ASF1A	-0.9304767
ASF1B	-1.971931
ASGR2	-1.2586367
ASIC1	-1.4928801
ASNSD1	-0.3549328
ASPA	-1.2489364
ASPH	-0.8835907
ASPM	-1.811408
ATAD2	-1.5962567
ATAD5	-1.348145
ATG2A	-0.3750453
ATG4B	-1.2023563
ATG4D	-1.7593555
ATG9A	-0.3097076
ATHL1	-0.8144414
ATL1	-0.9993527
ATL2	-0.7180891

ATOH8	-1.0916345
ATP1A2	-1.9444509
ATP1A3	-1.0003247
ATP1B2	-3.8505154
ATP2A3	-1.3030357
ATP2B2	-0.7616863
ATP2B4	-1.4246869
ATP5D	-0.4083695
ATP6V0A1	-1.4855585
ATP6V0A4	-2.3057463
ATP6V0D2	-1.5575156
ATP6V0E2	-0.408247
ATP6V1D	-0.4650245
ATP8A1	-0.4270749
ATPAF2	-0.4736924
ATPIF1	-1.4236546
ATXN1	-0.4937751
AUP1	-0.4302836
AURKA	-1.7451205
B3GALNT2	-0.6719236
B3GAT3	-0.5607429
B3GNT5	-0.6388564
B3GNT8	-2.502049
B4GALNT1	-0.4977636
B4GALNT2	-0.7626896
B4GALT4	-1.0678527
B9D1	-0.4440217
BAD	-0.4769177
BAG1	-0.5360012
BAG2	-1.9198682
BAG6	-0.4756889
BAI2	-1.8095942
BANK1	-0.8344588
BARD1	-1.3167272
BAZ1B	-0.5636973
BBIP1	-0.3242464
BBS1	-0.9628537
BBS10	-0.5898237
BBS7	-0.6195242
BBS9	-1.1850753
BCAM	-1.1421509
BCAS3	-0.6507173
BCKDK	-0.8888578
BCL2L1	-0.5729384
BCL2L13	-1.04531

BCL7A	-0.7547171
BCL7B	-0.8175755
BCO2	-0.758781
BCOR	-0.4418726
BCORL1	-1.639972
BECN1	-0.6146388
BEND5	-0.7406316
BEND7	-0.5163622
BEX4	-2.3694844
BEX6	-2.451618
BFSP2	-1.2241783
BGLAP	-0.7489452
BGLAP2	-0.8112657
BIN1	-0.3144984
BIN2	-0.5339055
BIRC5	-2.0112796
BLK	-0.6560526
BLM	-1.9113641
BLOC1S1	-0.453825
BLOC1S5	-1.1655097
BLVRA	-0.989892
BLVRB	-2.0573177
BMP4	-1.1828175
BMP6	-0.6490398
BMX	-1.0520179
BMYC	-0.3377461
BNC1	-1.3554425
BNC2	-1.1690714
BNIP3L	-0.818234
BOK	-0.7572575
BOLA1	-1.2475688
BOLA3	-1.1245971
BORA	-0.9116743
BPGM	-0.4510827
BPHL	-1.1565509
BPIFA6	-0.491178
BRCA1	-1.7640951
BRCA2	-2.0170002
BRD3	-0.4716711
BRDT	-0.8983483
BRF2	-0.4306202
BRIP1	-0.7088096
BRPF3	-1.538876
BSCL2	-0.7222924
BSDC1	-0.7032266

BSG	-1.3363853
BTBD2	-1.0354137
BTBD6	-0.6761622
BTD	-0.6273212
BTG2	-0.9493375
BTNL1	-0.5974162
BTNL10	-1.708117
BTNL2	-0.797976
BTRC	-1.1329076
BUB1	-1.629427
BUB1B	-2.1401505
BUB3	-0.5960774
BVES	-1.0893896
BZRAP1	-0.867347
C1GALT1	-0.5518994
C1QTNF2	-2.4395106
C1QTNF4	-1.1880527
C1QTNF5	-1.1774447
C1QTNF7	-1.3950038
C2CD2	-0.430649
C2CD2L	-0.8409076
C2CD3	-0.7990015
C2CD5	-0.7861886
C3AR1	-0.7847474
C5AR2	-1.4544737
C6	-1.434247
C8G	-0.8840294
CAAP1	-0.3916211
CAB39L	-0.3696399
CABIN1	-0.8363895
CACHD1	-2.0102046
CACNA1G	-4.4357405
CACNA1I	-1.6834376
CACNA2D2	-0.5381486
CACNB1	-0.3208134
CACNG1	-0.6324673
CACTIN	-0.9695172
CADM1	-1.2953472
CADM4	-0.7037015
CALHM2	-0.5195227
CALM2	-0.5219808
CALM3	-0.5590105
CALML4	-1.4474881
CALR4	-1.5471649
CAMK1	-0.8468106

CAMK1D	-0.6773586
CAMK1G	-1.0537066
CAMKK2	-0.4968081
CAMP	-2.7459354
CAMSAP2	-1.1791229
CAND2	-0.3744402
CAP2	-1.5975785
CAPN1	-0.8578286
CAPN10	-0.5520587
CAPN3	-2.4247804
CAPN5	-1.101079
CAPRIN2	-1.0813613
CAPSL	-0.7171044
CAR1	-2.0301914
CAR2	-2.0830703
CAR3	-3.888627
CAR6	-0.7027354
CAR7	-0.9266586
CARD11	-0.7070675
CARD9	-1.465862
CARHSP1	-1.418055
CARM1	-0.4680657
CARS2	-0.6454516
CASC5	-1.880286
CASP14	-0.8735638
CASP3	-0.5348868
CASP6	-0.7494168
CASP9	-0.7539864
CASQ2	-2.2483711
CASS4	-0.659615
CAT	-1.1805663
CAV1	-1.822046
CAV2	-1.0291464
CBFA2T3	-1.5682392
CBR1	-0.8220067
CBR2	-2.120467
CBX2	-0.8249652
CBX3	-0.7080054
CBX5	-1.3150563
CBX6	-1.1101966
CBY1	-1.6389828
CCDC103	-2.3983572
CCDC112	-0.3274484
CCDC113	-0.7544808
CCDC125	-0.5188592

CCDC136	-0.9009614
CCDC144B	-0.3949642
CCDC150	-1.5381284
CCDC157	-0.6447148
CCDC167	-1.0982897
CCDC18	-1.7032158
CCDC181	-0.4510102
CCDC185	-0.5250664
CCDC24	-1.5457487
CCDC28B	-0.6869941
CCDC34	-1.781831
CCDC42B	-1.5147574
CCDC51	-0.6423292
CCDC57	-0.4530656
CCDC61	-1.1360321
CCDC67	-0.631496
CCDC68	-1.0235615
CCDC69	-0.5785992
CCDC77	-1.1202278
CCDC80	-3.4598186
CCDC88A	-0.5974293
CCDC88C	-0.3955803
CCDC92	-1.1101599
CCDC97	-0.3708854
CCHCR1	-1.678637
CCL24	-3.1804965
CCL26	-0.7031794
CCL27A	-0.4242122
CCL6	-0.9950681
CCNA2	-2.4742827
CCNB1	-2.176187
CCNB2	-2.14392
CCND1	-0.9427037
CCND3	-1.5996122
CCNDBP1	-0.6558695
CCNE1	-2.7541738
CCNF	-2.065538
CCP110	-1.7053437
CCPG1	-0.3400326
CCR2	-2.0625615
CCR3	-3.5069168
CCR6	-0.8312182
CCRN4L	-0.5129795
CCS	-0.6884046
CCSAP	-0.5801241

CD101	-1.0391014
CD160	-2.4390664
CD163	-2.694939
CD180	-1.2222908
CD200R4	-3.4605894
CD207	-3.0534375
CD209A	-3.9071379
CD209B	-1.534641
CD209D	-0.5878866
CD209F	-2.4194372
CD209G	-0.9156172
CD226	-1.0107713
CD248	-1.8558855
CD24A	-1.9117718
CD300C	-1.3457396
CD300LB	-2.620972
CD300LD	-1.7587588
CD300LH	-0.718744
CD37	-0.8944516
CD3G	-0.4186215
CD47	-0.5530958
CD52	-0.6983523
CD59A	-1.0823383
CD5L	-0.5516295
CD7	-1.8102965
CD74	-0.4607554
CD79A	-1.0810156
CD79B	-1.109056
CD82	-0.7064581
CD84	-0.4092126
CD99L2	-1.7168188
CDADC1	-0.5905109
CDAN1	-1.4109707
CDC123	-0.5244012
CDC14A	-0.6817534
CDC20	-1.4904046
CDC25B	-2.2821827
CDC25C	-1.7124643
CDC27	-0.6197453
CDC42BPA	-0.8014739
CDC42EP3	-1.394949
CDC42SE1	-0.3259292
CDC45	-1.2626891
CDC6	-2.805429
CDC7	-0.4133117

CDCA2	-2.2405798
CDCA3	-1.4232545
CDCA4	-0.9850912
CDCA5	-1.3245373
CDCA8	-1.734972
CDH18	-0.9338045
CDK1	-1.252805
CDK15	-1.0215299
CDK16	-0.4672175
CDK19	-0.7840543
CDK2	-0.9675107
CDK4	-0.3636069
CDK5RAP2	-1.4241009
CDKL1	-0.9916158
CDKN1C	-0.6652508
CDKN2AIPNL	-0.3897238
CDKN2C	-3.3517969
CDKN2D	-1.0221882
CDKN3	-2.830885
CDNF	-0.5529914
CDPF1	-0.6809158
CDR2	-1.9423146
CDT1	-0.8410969
CDYL	-0.4084797
CEACAM10	-0.873945
CEACAM2	-0.9034343
CEACAM20	-0.8450832
CEBPE	-1.7820606
CECR2	-1.7909546
CECR5	-1.1018941
CENPA	-1.9456196
CENPC1	-0.880424
CENPE	-1.5027623
CENPF	-1.9182026
CENPH	-2.07906
CENPI	-1.492379
CENPJ	-0.5964661
CENPK	-1.9133902
CENPL	-1.627305
CENPM	-0.5980597
CENPN	-1.5352554
CENPO	-0.5748839
CENPP	-2.0608168
CENPQ	-0.5535612
CENPT	-1.0363593

CENPU	-0.9588988
CENPV	-0.7116466
CENPW	-1.000504
CEP104	-0.7914901
CEP112OS2	-0.6145973
CEP128	-1.3404732
CEP131	-1.4946003
CEP152	-0.9644589
CEP164	-0.8124242
CEP192	-0.5099111
CEP250	-1.0935493
CEP290	-1.5397582
CEP41	-0.8717439
CEP44	-0.8516154
CEP57	-1.3059812
CEP57L1	-0.8090017
CEP70	-1.6921115
CEP72	-1.8537025
CEP76	-2.4255025
CEP78	-0.7128866
CEP85	-0.7345221
CEP89	-1.4003382
CERCAM	-2.488683
CERKL	-0.9053857
CES1D	-2.404948
CES2G	-2.7909498
CFD	-0.9784207
CGN	-2.158708
CGRRF1	-0.5189867
CHAC2	-1.293951
CHAF1A	-1.4475245
CHAF1B	-1.8389754
CHAT	-0.6475167
CHCHD10	-0.7962265
CHD1	-0.4490359
CHD8	-0.5942178
CHEK2	-1.6254606
CHIL3	-0.6862402
CHIL4	-1.4049714
CHIT1	-4.5058765
CHN2	-0.9063842
CHP2	-1.9655905
CHRDL1	-0.6486917
CHRM4	-1.511317
CHST1	-1.7633886

CHST12	-0.6170566
CHST3	-1.630722
CHST8	-1.1814289
CHTF18	-1.6046405
CIAPIN1	-0.3879466
CIB1	-0.4873023
CIB2	-0.4140892
CIB3	-0.5975988
CIDEA	-1.9218483
CIDEC	-1.7109458
CIITA	-3.575666
CILP	-1.9306936
CILP2	-1.70892
CIT	-2.778648
CITED1	-0.8226306
CITED4	-3.2972722
CKAP2	-1.5688243
CKAP2L	-1.5867503
CKAP5	-0.7561903
CKB	-1.7684267
CKM	-2.19226
CKS1B	-0.6774654
CKS2	-1.4664044
CLCN2	-3.2948103
CLCN3	-0.7927866
CLDN13	-3.4947448
CLDN15	-2.144056
CLDN19	-0.4934499
CLDN20	-2.6543493
CLEC11A	-1.0565178
CLEC14A	-2.1063383
CLEC1A	-1.0436654
CLEC1B	-1.2805777
CLEC2L	-1.9574275
CLEC3B	-1.0611501
CLEC4A4	-2.0488963
CLEC4B2	-2.2062545
CLEC4G	-2.4134154
CLEC7A	-1.8510447
CLEC9A	-2.404333
CLHC1	-1.3728795
CLIC5	-1.4143581
CLK3	-0.669435
CLMN	-1.2149904
CLN6	-1.2506528

CLN8	-1.1546326
CLP1	-0.7956028
CLPB	-0.4478536
CLSPN	-2.2154348
CLSTN3	-1.2608206
CLTC	-0.4714661
CLYBL	-1.8307002
CMAH	-0.5620518
CMAS	-1.1365638
CMBL	-3.8730032
CMC2	-1.0656133
CML5	-1.7389851
CMTM5	-0.6223712
CMTM7	-0.6935945
CMTM8	-0.9103539
CNN1	-2.9569845
CNN2	-0.533102
CNNM1	-2.9759965
CNNM2	-1.9189515
CNOT10	-0.5431294
CNOT4	-0.4832521
CNP	-0.432343
CNPY4	-0.7368317
CNR1	-1.2189674
CNST	-0.682714
CNTF	-0.5630128
CNTLN	-1.2076988
CNTN1	-0.7127726
CNTN2	-0.4992032
CNTN6	-1.5273912
CNTROB	-1.4542439
COA3	-0.7074304
COA4	-0.6501489
COBL	-0.5287206
COCH	-0.9359031
COG2	-0.3392725
COG4	-0.3838396
COL14A1	-2.5773685
COL15A1	-1.8706324
COL16A1	-1.0234959
COL1A1	-1.8550992
COL1A2	-1.3854785
COL26A1	-2.5316007
COL27A1	-1.4342406
COL3A1	-1.1381044

COL5A1	-1.4760485
COL5A2	-0.510073
COL6A1	-1.5712943
COL6A2	-2.066215
COL6A3	-0.7026472
COL8A2	-3.1993606
COMT	-1.1487355
COPS2	-0.4670234
COPS3	-0.5592585
COPS4	-0.4024506
COPZ1	-0.7193098
COQ9	-0.5915959
CORO1A	-0.5954828
CORO2B	-1.5855441
COX19	-0.558332
COX20	-0.7752914
COX6B2	-3.2975254
COX6C	-0.5694728
COX7A1	-0.6597881
COX8B	-2.6865768
CPLX3	-2.0317266
CPM	-1.2290878
CPNE2	-0.9074688
CPNE4	-1.4045541
CPNE5	-1.2648015
CPOX	-3.2188292
CPQ	-0.3190193
CPSF3L	-0.407826
CPT1C	-1.3251717
CPT2	-0.7711716
CPXM1	-1.4770956
CPXM2	-0.6558569
CPZ	-1.4509826
CR2	-1.3124251
CRAT	-1.8155558
CREBZF	-0.41749
CREG1	-0.9570322
CRHR1	-1.426925
CRIP1	-1.0993376
CRIP2	-1.6713252
CRIP3	-0.8056099
CRISP3	-3.4122505
CRISPLD1	-2.6106076
CRKL	-0.6362467
CRMP1	-1.4304409

CRNKL1	-0.3120515
CROCC	-0.55528
CROT	-0.4461532
CRXOS	-1.1701262
CRYBA4	-0.7883091
CRYBB1	-1.4026549
CRYGS	-1.397821
CRYL1	-1.0470371
CSDC2	-3.640099
CSF1R	-0.3993669
CSGALNACT1	-0.8461604
CSGALNACT2	-0.7067981
CSL	-0.4010162
CSMD3	-1.5707288
CSNK1G2	-0.3535752
CSPG4	-1.5147042
CSPP1	-0.6602709
CSRP2	-0.7266913
CSRP3	-3.455533
CTBP1	-0.651773
CTC1	-0.5061526
CTCFL	-1.5173054
CTF1	-0.3297546
CTNND2	-3.005407
CTSE	-2.3320088
CTSF	-2.245235
CTSG	-1.1105022
CTSW	-0.7617369
CTTNBP2	-1.4026358
CTXN1	-0.7837367
CUEDC1	-0.8373458
CUEDC2	-0.6266584
CUL4A	-1.2221618
CUTA	-0.5166025
CUTC	-0.4398806
CUX1	-0.6750641
CX3CR1	-3.1579487
CXCR1	-0.8159263
CXCR3	-0.7436781
CXXC1	-0.467876
CXXC5	-1.4235468
CYB561A3	-1.0373259
CYB5A	-1.1230245
CYB5D2	-0.7857204
CYB5R1	-0.3947215

CYB5R3	-0.5810866
CYHR1	-0.4960361
CYP11A1	-1.6509097
CYP21A1	-0.4870517
CYP26C1	-1.3494623
CYP27A1	-1.7949593
CYP2AB1	-0.6792736
CYP2D9	-1.0338192
CYP2E1	-1.8255486
CYP2J9	-0.8118377
CYP2S1	-1.8990395
CYP2T4	-0.5441363
CYP4B1	-0.926723
CYP4B1-PS2	-1.6193752
CYP4F18	-1.4129467
CYTL1	-1.1502442
DAAM1	-1.1697221
DAAM2	-0.7202289
DACT1	-1.0275197
DAGLB	-1.112134
DAK	-0.7442956
DAND5	-1.2463462
DAP	-0.6522164
DAP3	-0.3852115
DAPK2	-1.6832848
DARS2	-1.4426675
DBF4	-1.2327967
DBNDD1	-0.7238865
DBP	-1.3108401
DCAF12	-0.5565314
DCAF12L1	-0.7511387
DCAF4	-0.5675163
DCAF6	-1.0091944
DCHS1	-0.8638694
DCK	-0.7854292
DCLK1	-0.6776633
DCLK2	-1.6732826
DCLRE1A	-1.8366508
DCSTAMP	-2.0479178
DCT	-8.654474
DCTN6	-0.6548734
DCUN1D1	-0.7589965
DDAH2	-0.9384399
DDC	-0.8845553
DDIT4L	-2.595881

DDX11	-0.5142043
DDX26B	-0.6159573
DDX4	-1.2823532
DDX47	-0.4035401
DEAF1	-0.788085
DEF8	-0.7832007
DEFA1	-0.7474861
DEFA10	-1.486547
DEFB25	-1.0300322
DEFB26	-3.6087394
DEFB29	-0.7235832
DEFB42	-1.3278887
DEK	-1.5905914
DENND1C	-0.4345169
DENND2A	-0.6612406
DENND2C	-2.4041636
DENND6B	-1.0035908
DEPDC1A	-1.5847166
DEPDC1B	-1.7726383
DET1	-0.3359132
DFNA5	-2.275434
DFNB59	-1.2097168
DGCR6	-0.3297482
DGKG	-1.8436024
DGKI	-1.2359669
DHCR24	-1.7805057
DHCR7	-0.7795241
DHFR	-0.6735392
DHRS1	-0.7665529
DHRS11	-2.429471
DHRS13	-1.052798
DHRS3	-0.3596063
DHX34	-0.3376799
DIAP3	-1.3071723
DIO2	-0.8218014
DIRAS2	-1.660917
DISC1	-0.3924177
DKK4	-1.1660964
DKKL1	-2.3918078
DLEU2	-0.8909907
DLG2	-1.8502498
DLGAP5	-2.379602
DLL1	-0.9837656
DLX6OS1	-0.7433205
DMPK	-2.5111873

DMRTB1	-0.6150446
DMTN	-0.591352
DMWD	-0.3520446
DNA2	-1.2200513
DNAAF2	-1.8843157
DNAH8	-0.7118273
DNAIC1	-1.5131009
DNAJA4	-1.3999515
DNAJB1	-0.9666166
DNAJB2	-1.8865619
DNAJB3	-2.5342116
DNAJB4	-1.1761489
DNAJC10	-0.4031215
DNAJC28	-1.1291287
DNAJC4	-0.5972552
DNAJC6	-1.6630411
DNAJC9	-0.893889
DNASE1	-1.1306314
DNASE1L1	-1.3274064
DNM1	-0.9224477
DNMT1	-1.1805844
DNPH1	-0.5042567
DNTT	-2.840774
DOCK2	-0.4320502
DOK2	-0.4332652
DOK4	-1.460253
DOLK	-0.3311029
DOLPP1	-0.6865106
DONSON	-0.4569292
DOS	-0.6828208
DOT1L	-0.3964729
DPEP2	-0.5471354
DPF3	-2.2119486
DPP8	-0.3327823
DPPA2	-1.2815757
DPT	-1.0076716
DPY19L1	-0.9592238
DPY30	-0.7360635
DSC2	-0.9319928
DSCC1	-1.2901769
DSN1	-0.814786
DTWD1	-0.4313166
DTWD2	-0.5102866
DTX3	-0.6187244
DUSP8	-1.3003118

DUT	-1.0486465
DYM	-0.568243
DYNLL1	-0.7319851
DYRK1A	-0.4672294
DYRK1B	-0.6620851
DYRK3	-1.2017899
DYSF	-1.5861142
E2F1	-1.7843838
E2F2	-1.419456
E2F4	-1.8112602
E2F7	-1.62148
E2F8	-2.2767606
EAR1	-0.7788634
EAR10	-1.0652881
EAR12	-0.9508486
EAR2	-1.0314445
EAR6	-0.8634095
EAR7	-1.314434
ECH1	-1.08077
ECHDC3	-0.9083371
ECI1	-0.595551
ECT2	-1.261908
EDARADD	-0.404856
EDC3	-0.6328037
EDRF1	-0.3174436
EEF1A2	-1.3238938
EEF1D	-0.3731618
EEF2K	-1.3473337
EFCAB12	-0.6331604
EFCAB5	-1.2490892
EFCAB9	-0.8178411
EFEMP1	-1.5108969
EFHD1	-2.1981897
EFNB1	-1.3364668
EFNB3	-2.2825418
EHBP1L1	-1.2179375
EHHADH	-0.3453856
EIF2AK1	-1.3244109
EIF2D	-0.7873673
EIF4EBP2	-0.3780584
ELF5	-0.6747809
ELK3	-0.4463158
ELMO3	-0.8061349
ELN	-3.0506957
ELOVL6	-0.5157404

ELP4	-0.3114255
EMC9	-1.9504089
EME1	-1.5116193
EMID1	-1.5698586
EMP3	-1.0526118
ENDOD1	-2.4409194
ENG	-0.4084759
ENGASE	-0.8122354
ENHO	-0.9769411
ENKD1	-0.9876027
ENPP1	-0.6432948
ENPP3	-2.2298355
ENPP5	-0.3887546
ENSA	-0.5170736
ENTPD5	-0.5690713
EOMES	-0.7262068
EP300	-0.7488952
EPB4.1	-0.7624598
EPB4.2	-2.2652988
EPDR1	-1.7797627
EPHB6	-0.9140902
EPHX1	-0.75209
EPHX2	-0.5858848
EPHX3	-1.3239641
EPM2AIP1	-0.449048
EPN1	-1.7526431
EPOR	-3.3061824
EPX	-1.9513164
ERBB2	-0.3497689
ERC1	-0.3799923
ERCC6L	-1.620533
ERI1	-0.691618
ERI2	-1.2444205
ERMAP	-2.688037
ERMP1	-0.7541304
ERP29	-0.4479094
ESCO2	-2.097
ESPL1	-1.8774109
ESRRG	-1.0013447
ESYT2	-0.4897251
ESYT3	-0.7900887
ETAA1	-0.9511054
ETHE1	-1.5220985
ETL4	-0.6304627
ETV1	-0.7220099

EVC	-1.6138957
EVI2A-EVI2B	-0.7368243
EVI5	-0.8382182
EVI5L	-0.7047987
EVPL	-1.368278
EXO1	-0.8982263
EXOC3L2	-0.7819297
EXOC6	-0.6778841
EXOC7	-0.5350413
EXOC8	-0.4219358
EXOG	-0.9480786
EXT1	-0.4646592
EYA2	-1.6694851
EZH2	-1.1195564
F2RL2	-1.2812321
F5	-1.7792315
F8	-1.5031998
FABP7	-1.516851
FADS1	-1.9034886
FADS2	-2.1583862
FADS3	-1.84799
FAF1	-0.7277565
FAHD1	-2.2151637
FAIM3	-1.8129249
FAM105A	-0.6743789
FAM109B	-2.522163
FAM110A	-0.6709895
FAM110B	-2.0237226
FAM111A	-1.2270088
FAM117A	-1.9543395
FAM118B	-0.3917494
FAM122A	-1.0098958
FAM122B	-0.5739145
FAM124A	-1.8097093
FAM126A	-1.1293745
FAM129C	-0.8748465
FAM131A	-0.6543052
FAM132A	-2.5466328
FAM132B	-2.774931
FAM13A	-0.9070306
FAM167B	-0.5708871
FAM170B	-1.5114982
FAM171A1	-1.1537995
FAM171B	-1.2241099
FAM173A	-0.6016107

FAM173B	-0.666337
FAM180A	-0.4342675
FAM186B	-0.875494
FAM193A	-0.3972173
FAM195A	-0.3858557
FAM198B	-1.233218
FAM20A	-0.6139395
FAM20B	-0.7121801
FAM210B	-1.8306055
FAM212A	-1.6885908
FAM212B	-1.7526557
FAM214B	-1.2765832
FAM216A	-0.6181393
FAM219B	-0.4714608
FAM220A	-0.6474609
FAM227A	-0.328624
FAM229A	-0.439589
FAM53B	-1.4461446
FAM57B	-0.9123545
FAM63B	-0.9597244
FAM64A	-1.5814595
FAM65A	-0.604383
FAM69A	-1.0669398
FAM71D	-0.5334849
FAM73B	-1.1176486
FAM78A	-1.3523035
FAM83D	-2.154006
FAM89B	-0.6461425
FAM92B	-2.6821125
FANCB	-0.7264223
FANCC	-0.605633
FANCD2	-1.8152056
FANCG	-0.8635931
FANCI	-1.2411535
FANCL	-0.9051404
FANCM	-0.9107928
FARS2	-0.6035409
FBLN1	-1.7578158
FBLN5	-2.745332
FBLN7	-1.1699371
FBN2	-1.471128
FBRSL1	-0.4316678
FBXL15	-0.8257742
FBXL19	-0.706769
FBXO10	-2.0151105

FBXO3	-0.759213
FBXO30	-1.1005292
FBXO34	-0.7520394
FBXO41	-0.83565
FBXO5	-2.5822153
FBXO7	-1.3078957
FBXO8	-0.4455433
FBXO9	-1.2121906
FBXW4	-0.5530167
FBXW7	-0.3946047
FCER1A	-1.1882083
FCER2A	-2.73274
FCGBP	-0.5080965
FCGRT	-1.7815752
FCHO1	-1.4908247
FCHSD1	-0.5364437
FCNB	-2.9833186
FCRLA	-0.9955888
FDPS	-1.0213647
FECH	-1.5997257
FEN1	-1.1513958
FERMT3	-0.5340986
FFAR4	-2.2130983
FGD2	-0.9151149
FGD5	-0.4328854
FGD6	-0.717222
FGF1	-1.6518917
FGFR3	-0.9445927
FGFR4	-2.4488847
FGFRL1	-1.1458883
FH1	-0.8016834
FHDC1	-1.5432057
FHL1	-2.6124005
FHL2	-1.3673422
FIBIN	-0.6282249
FIGF	-2.1768396
FIGNL1	-1.9816446
FIS1	-0.47545
FITM1	-1.7542658
FKBP1B	-0.5551329
FKBP4	-0.3452864
FKBP7	-0.9925609
FKBP8	-0.6039081
FLRT1	-1.3087633
FLRT2	-1.0515294

FLT3L	-1.1379313
FMNL1	-0.3428359
FMO2	-3.3950844
FMO5	-1.1376295
FN3K	-3.888003
FN3KRP	-2.3954883
FNDC1	-0.9444981
FNDC5	-2.8948712
FNTB	-1.4768176
FOCAD	-0.3753448
FOLR1	-2.3893747
FOPNL	-0.6514387
FOXD2OS	-1.9816666
FOXK2	-0.5387511
FOXM1	-1.6484945
FOXO4	-1.2283776
FRAS1	-4.061633
FRAT1	-0.5674996
FREM1	-1.1754069
FREM3	-1.0232501
FRMD3	-2.0965314
FRY	-1.7404828
FSBP	-0.9055252
FSD1L	-0.6885679
FTO	-0.3198881
FTSJ1	-1.0007801
FUCA1	-0.4429379
FUCA2	-0.5504992
FUNDC1	-0.4296675
FUNDC2	-0.7016673
FUT11	-0.4472516
FUT7	-2.417105
FUZ	-0.4295266
FXN	-1.5443625
FXYD1	-0.8571358
FXYD2	-0.4197907
FXYD6	-1.8202286
FZD2	-1.6271763
FZD8	-1.4398766
FZR1	-1.3998284
G2E3	-1.0621321
G6B	-1.2349234
GAB1	-1.099288
GABBR1	-0.3295066
GABRA3	-1.879806

GAD1	-1.0702038
GADD45A	-1.1292367
GADD45GIP1	-0.5026474
GAL3ST2	-0.9029553
GALE	-0.3398128
GALNT1	-0.6631155
GALNT10	-1.70999
GALNT14	-1.575594
GALNT3	-0.315661
GAMT	-1.2759051
GANC	-0.6342626
GAPT	-1.891473
GAPVD1	-0.642045
GAREM	-2.0409594
GAS2	-0.9828017
GAS2L1	-0.5011945
GAS2L3	-0.9203336
GAS6	-2.0425348
GATA1	-2.4793715
GATAD1	-0.5064478
GATC	-1.075645
GBA2	-0.965786
GBP2B	-1.0668664
GBX2	-0.9490647
GCDH	-1.6953526
GCH1	-1.0303411
GCHFR	-1.4528143
GCLC	-1.2735019
GCLM	-1.7816534
GCNT1	-0.7577546
GCSAM	-1.1436903
GDA	-0.7897272
GDAP1L1	-0.4758275
GDF1	-0.6125338
GDF10	-4.0763273
GDF3	-2.1425292
GDF6	-3.4342968
GDPD1	-1.4850159
GDPD2	-0.5559726
GDPD4	-6.5667076
GDPD5	-0.436816
GDPGP1	-0.4402316
GEMIN2	-0.8679078
GEN1	-1.8163962
GFAP	-3.1689513

GFER	-1.0759711
GFI1B	-2.609169
GGA2	-0.8999004
GID8	-1.0363564
GIN1	-1.2051833
GINS1	-0.9162684
GINS2	-1.7123508
GINS4	-1.0767283
GJA3	-1.5144081
GJB3	-1.8274179
GLB1L	-1.0006213
GLI2	-1.2833829
GLIPR1	-0.9677076
GLO1	-0.9696288
GLOD4	-0.4037218
GLOD5	-1.1728578
GLRP1	-1.2553678
GLRX5	-1.938756
GLTP	-0.6662831
GM10024	-1.3293309
GM10046	-0.4594629
GM10101	-0.9844513
GM10125	-0.3729315
GM10134	-0.8759956
GM10229	-1.1383188
GM10257	-0.9827409
GM10336	-0.5372791
GM10371	-1.8837125
GM10375	-0.8691502
GM10419	-0.6741879
GM10505	-1.5698164
GM10509	-0.7328739
GM10516	-1.2144856
GM10561	-0.7522392
GM10603	-0.8602185
GM10614	-1.116416
GM10638	-1.31552
GM10639	-4.1808176
GM10658	-2.153326
GM10687	-1.3930221
GM10731	-0.5176921
GM10745	-0.4150717
GM10759	-0.6905069
GM10790	-1.8809228
GM10824	-2.054278

GM10866	-0.6312804
GM10910	-0.8062239
GM10941	-1.550705
GM11223	-1.6960492
GM11545	-2.0896313
GM11564	-0.5301828
GM11747	-1.4881485
GM11937	-0.484977
GM12059	-1.2000654
GM12247	-0.6912713
GM12271	-1.085527
GM12620	-1.4513288
GM12657	-0.6191731
GM12666	-0.941226
GM13032	-0.7973788
GM13084	-0.898175
GM1322	-0.4464827
GM13272	-0.5846007
GM13275	-0.6912372
GM13372	-2.5731711
GM13710	-1.3450706
GM13861	-1.2760439
GM14085	-0.3171628
GM14325	-0.3876057
GM14420	-0.6450901
GM14483	-1.279547
GM14484	-1.3375151
GM14635	-1.0944564
GM15645	-0.9452601
GM15800	-1.2437239
GM16039	-0.807987
GM1631	-1.7605844
GM16500	-1.5520144
GM16505	-1.2137878
GM16515	-2.2573256
GM166	-2.806266
GM16982	-1.7334492
GM17748	-0.6964855
GM17778	-0.5223515
GM1987	-0.6113858
GM1993	-1.4380538
GM20740	-0.5644827
GM2085	-0.6223433
GM20939	-0.4235563
GM2099	-0.8082857

GM2115	-1.9781172
GM2138	-0.3595712
GM2155	-0.7690547
GM2A	-1.3029814
GM3227	-1.6967921
GM3258	-0.5057883
GM3285	-0.5389297
GM3325	-1.5281062
GM3604	-0.8428729
GM3776	-4.292698
GM3853	-2.6624308
GM3871	-0.5787292
GM4035	-0.8278155
GM4285	-0.3152683
GM4430	-1.2937522
GM4673	-0.4086242
GM4906	-2.0809574
GM4980	-0.9926243
GM5068	-0.7668591
GM5088	-0.514421
GM5132	-1.7634363
GM5141	-1.5695224
GM525	-0.823602
GM5382	-1.8871727
GM5463	-1.5000827
GM5493	-0.9505062
GM5553	-0.5914953
GM5570	-0.7623913
GM5593	-1.7441654
GM5595	-0.5155225
GM568	-2.4556468
GM5751	-0.807178
GM5767	-1.3709764
GM5782	-1.8444138
GM5820	-2.165197
GM608	-0.4034424
GM6653	-1.1422267
GM6760	-1.4010139
GM6792	-2.0969768
GM6936	-1.7356074
GM711	-1.3663881
GM7664	-0.5607491
GM7931	-0.6861167
GM7936	-0.3775554
GM7968	-0.3126962

GM8005	-0.944103
GM8013	-0.7272971
GM8094	-0.7108467
GM8212	-1.0008633
GM8220	-0.7950697
GM8221	-0.6361642
GM8256	-0.8519194
GM853	-0.5607343
GM867	-3.7185357
GM8817	-0.5047286
GM9054	-1.2883465
GM9525	-1.1267896
GM9530	-0.8008933
GM9694	-0.7025933
GM9733	-2.3090131
GM9767	-0.6113267
GM9831	-0.8296549
GM9868	-1.3984835
GM9895	-1.5819545
GM9903	-0.7219908
GM9920	-0.3676777
GM996	-0.8543172
GM9992	-2.540567
GM9999	-0.5082765
GMEB1	-0.7726893
GMIP	-0.3452625
GMNN	-1.0453434
GMPR	-1.3847208
GNA12	-0.8479166
GNA15	-0.7281609
GNAS	-0.4432364
GNAZ	-1.1788199
GNB2	-1.0201678
GNG11	-1.232204
GNPAT	-0.3574672
GNPDA1	-0.6678972
GOLIM4	-0.9085741
GOLM1	-2.2092056
GP1BA	-0.9559026
GP1BB	-1.153254
GP5	-1.3219357
GP6	-1.251596
GP9	-2.4057894
GPC1	-0.6507835
GPC2	-0.6298595

GPC3	-0.7443867
GPCPD1	-0.5908275
GPD1	-2.4000432
GPM6A	-0.9299152
GPNMB	-0.6008351
GPR125	-0.6170304
GPR128	-0.8747873
GPR133	-0.3706079
GPR153	-1.4949827
GPR155	-1.8901868
GPR162	-0.565686
GPR19	-0.8597586
GPR25	-0.905709
GPR27	-0.6795449
GPR34	-2.2153485
GPR88	-1.8119233
GPR97	-0.9971952
GPRASP2	-0.4033897
GPRC5D	-0.6719875
GPRC6A	-0.4168823
GPS2	-1.2394567
GPSM2	-2.653945
GPSM3	-0.7371869
GPT	-0.7045851
GPX1	-1.0396428
GPX2	-0.5484099
GPX4	-1.3580294
GRAMD1C	-0.3696237
GRIA3	-2.0416617
GRINA	-0.8822455
GRRP1	-0.5297878
GRTP1	-2.4229481
GSDMA	-0.3176911
GSE1	-1.728548
GSG1	-1.3338056
GSG2	-0.9981341
GSN	-1.0590706
GSS	-0.9971523
GSTA4	-0.7154899
GSTK1	-0.5599966
GSTM1	-1.0814719
GSTM3	-1.2536361
GSTM4	-0.7033572
GSTM5	-2.9887552
GSTP1	-0.8281283

GSTP2	-0.5686784
GSTT1	-1.5066862
GSTZ1	-0.4389176
GTF2B	-0.6335058
GTF2E2	-0.4756823
GTF3C5	-0.5807505
GTPBP1	-0.9846077
GTSE1	-0.7271295
GUCA1A	-1.0599144
GUCA2B	-0.6660337
GUCD1	-0.7285509
GUCY1A2	-2.1363237
GUCY1A3	-2.4017978
GUCY1B3	-1.0511413
GUF1	-0.5263717
GYPA	-2.2994642
GYPC	-0.7597528
GZMM	-1.4149754
H1FOO	-0.5356939
H2AFJ	-1.0329046
H2AFV	-1.1256042
H2AFX	-1.393137
H2-DMA	-0.5310078
H2-DMB1	-0.8945198
H2-DMB2	-0.4820566
H2-EA-PS	-1.1491466
H2-EB1	-0.8390255
H2-KE6	-0.5977225
H2-M11	-1.2501638
H2-OA	-0.9286366
H2-OB	-1.0927968
H3F3A	-0.7062488
HABP4	-0.4111621
HACE1	-1.3423266
HACL1	-1.1819229
HADH	-0.8161316
HAGH	-1.9472604
HAGHL	-0.3489351
HAL	-0.6780162
HAND2	-0.6093595
HARS	-1.5961461
HAUS1	-0.6652341
HAUS4	-0.8898091
HAUS5	-0.8790355
HAUS8	-2.0004663

HAVCR1	-0.7462883
HAVCR2	-0.7646434
HBB-B1	-0.7029543
HBB-BH1	-3.0349438
HBQ1A	-1.4541106
HBQ1B	-1.4856939
HCFC1	-0.6112022
HCFC1R1	-0.7483187
HDAC10	-0.7684021
HDAC5	-0.7446795
HDAC7	-0.4875059
HDGF	-0.6469312
HDHD1A	-0.5429184
HDHD3	-0.6039074
HEATR5A	-0.3769097
HEBP1	-1.2050886
HECTD3	-0.7553539
HELB	-0.5123887
HELQ	-1.093575
HEPH	-1.2013886
HERC1	-0.7803354
HES6	-0.4213228
HEXA	-0.5978475
HEXIM1	-0.486289
HEY1	-1.5132093
HEYL	-1.9564571
HFE	-1.2738876
HHEX	-0.854702
HIBCH	-0.3849821
HIF3A	-0.4677939
HIGD2A	-0.7249861
HINT2	-1.2907948
HIP1	-0.8601658
HIPK1	-0.6231427
HIPK2	-0.8474488
HIRIP3	-0.6474366
HIST1H1A	-2.5095272
HIST1H1B	-2.2383866
HIST1H1C	-1.0696979
HIST1H1E	-2.1350708
HIST1H2AA	-1.6809819
HIST1H2AB	-2.0871096
HIST1H2AF	-0.8092265
HIST1H2AH	-0.814136
HIST1H2AK	-0.6788459

HIST1H2AN	-0.8957171
HIST1H2AO	-0.7462478
HIST1H2BB	-1.6908538
HIST1H2BC	-0.8100143
HIST1H2BP	-0.4574921
HIST1H2BQ	-0.8765993
HIST1H3C	-1.322712
HIST1H3D	-1.0937715
HIST1H3F	-1.3342137
HIST1H3G	-1.302628
HIST1H3I	-0.9175658
HIST1H4C	-1.3806558
HIST1H4D	-1.1444674
HIST1H4H	-1.1319728
HIST1H4I	-1.0253773
HIST1H4J	-1.4457855
HIST1H4K	-1.081903
HIST2H2AA2	-0.8749032
HIST2H2AB	-0.7693741
HIST2H2AC	-0.6369157
HIST2H2BB	-1.3325114
HIST2H2BE	-1.8403947
HIST2H3B	-2.1241817
HIST2H4	-1.2142849
HIST3H2BB-P	-0.6645293
HIST4H4	-1.276052
HJURP	-1.6414919
HMBS	-2.367879
HMCES	-0.673934
HMCN1	-1.357811
HMG20B	-0.4389491
HMGA2-PS1	-1.031497
HMGB1-RS17	-0.4296193
HMGB2	-1.1927795
HMGB3	-2.2256355
HMGXB4	-0.5119433
HMMR	-1.1852522
HNMT	-0.936291
HNRNPLL	-0.5879326
HNRNPU	-0.4581871
HNRNPUL2	-0.9456091
HOMER2	-0.6966879
HOOK3	-0.7156096
HOXC5	-0.7358091
HPGD	-2.2199943

HPN	-0.6755605
HPSE2	-0.4869232
HR	-1.817111
HRAS	-1.0997643
HRASLS5	-1.069077
HRH2	-0.3738463
HRSP12	-1.4726701
HS6ST1	-1.1294839
HS6ST2	-1.8028035
HSD11B1	-0.4871717
HSDL2	-0.8138676
HSPA12A	-1.0891066
HSPA12B	-1.1133823
HSPA2	-0.3256297
HSPA4L	-1.4367394
HSPB2	-1.2391691
HSPB6	-1.2626224
HSPG2	-0.9511409
HSPH1	-0.3723693
HTATIP2	-0.6165361
HTRA2	-0.6034894
HVCN1	-0.8155661
HYAL1	-1.2287145
HYDIN	-0.5407083
HYLS1	-1.0116348
IARS2	-0.409472
IBA57	-1.4567332
ICAM4	-3.9259777
ICAM5	-0.7999706
ID1	-1.0857463
ID4	-0.9959269
IDH1	-0.8947961
IFFO1	-0.6760249
IFI27	-0.9398317
IFI30	-0.5864325
IFITM10	-1.0247304
IFNL3	-0.8655791
IFRD2	-1.6089759
IFT172	-0.766057
IFT27	-0.7663727
IFT80	-1.2243724
IGDCC3	-0.8628132
IGF1	-1.54954
IGFBP2	-2.1259012
IGFBP4	-1.4192615

IGFBP6	-1.8715124
IGH-V3660	-0.8242841
IGKV4-72	-0.825563
IGLL1	-2.8434312
IGSF10	-1.3404562
IGSF3	-1.228946
IKZF3	-0.5173945
IKZF5	-0.6695552
IL13RA2	-0.6374042
IL17RE	-0.8840325
IL1RL1	-1.4056449
IL22RA2	-0.5341899
IL31RA	-1.2548857
IL4	-0.8587384
IL5RA	-0.9998815
IL7	-1.1558645
IL9R	-1.6111493
ILK	-0.9793792
ILKAP	-0.4155712
INCENP	-1.3274426
INF2	-0.4260612
INMT	-1.4004683
INPP5J	-1.630111
INSL5	-2.4102595
INSRR	-0.5364394
INTS1	-0.5221357
INTS10	-0.9119926
INTS12	-0.4081039
INTS4	-0.6145186
INTS7	-0.4820719
INTS8	-0.9187021
IP6K1	-0.5992918
IP6K3	-2.7136836
IPP	-1.1819024
IQCB1	-0.4653025
IQGAP3	-1.6150367
IQSEC1	-0.3855867
IRF2BP1	-0.4706998
IRS1	-0.9625104
ISCA1	-1.6645026
ISCA2	-1.1500478
ISG20	-0.6934991
ISLR	-0.3894994
ISLR2	-1.1676955
ISOC2B	-0.6417663

ITGA11	-0.7054343
ITGA2B	-0.6590681
ITGA6	-0.7418227
ITGA8	-3.182877
ITGAX	-1.2709723
ITGB2L	-1.3671477
ITGB5	-1.6573215
ITGB7	-1.2005386
ITM2A	-0.592063
IVD	-0.6017327
IZUMO4	-0.8925445
JADE3	-0.8270395
JARID2	-0.4699326
JMY	-0.4821336
JOSD1	-0.7229896
JOSD2	-2.5235348
JUP	-0.9209213
K230015D01	-0.5778711
KALRN	-0.7096665
KANSL1L	-0.6716759
KAT2B	-0.6077061
KAT6A	-0.3840551
KATNAL1	-1.65551
KAZALD1	-3.590602
KAZN	-0.5027106
KBTBD12	-2.1773007
KBTBD3	-1.5790608
KBTBD7	-0.6711042
KCNA3	-1.2065852
KCNA7	-0.762641
KCNAB2	-0.3020208
KCNB1	-0.6027815
KCNC1	-0.9717109
KCND3	-1.7112007
KCNG2	-1.0883479
KCNIP3	-0.7339637
KCNJ10	-2.3954434
KCNJ16	-4.009507
KCNJ5	-0.5463915
KCNJ9	-0.7023287
KCNK12	-3.2038326
KCNK4	-0.5540497
KCNMB4OS2	-5.5065336
KCNN4	-2.3545852
KCNS3	-0.4860117

KCP	-0.7982807
KCTD7	-0.5524621
KDELC2	-0.7655604
KDM4C	-0.5328116
KDM6A	-0.892509
KDM7A	-0.8372669
KDM8	-0.5253997
KEL	-2.5861812
KHK	-1.144197
KIF11	-2.5753224
KIF12	-1.3638875
KIF13A	-0.5763354
KIF14	-1.3620713
KIF15	-1.9528213
KIF17	-0.4884133
KIF18A	-2.3503995
KIF18B	-2.1497688
KIF20A	-2.0566125
KIF20B	-1.2705755
KIF22	-1.5582147
KIF23	-1.7977378
KIF24	-1.4749196
KIF2A	-0.6747766
KIF2C	-1.5527585
KIF4	-2.2002337
KIF5A	-1.2405262
KIF7	-0.6874161
KIF9	-0.9189038
KIFC2	-0.6384966
KIFC3	-0.4522557
KIN	-0.5730186
KISS1R	-0.803499
KLF1	-3.7882924
KLF10	-1.1807761
KLF11	-0.8155294
KLF3	-0.838614
KLF5	-1.3725975
KLHDC2	-0.5644698
KLHDC3	-0.525281
KLHDC8B	-1.5556443
KLHL12	-1.2069659
KLHL13	-0.9404111
KLHL14	-2.2736187
KLHL17	-1.1363912
KLHL25	-0.7961907

KLHL30	-1.0203712
KLHL32	-0.8895538
KLHL7	-0.7411289
KLHL9	-0.3663397
KLRA12	-1.2510109
KLRA16	-0.638916
KLRA17	-1.3842733
KLRA22	-0.7294512
KLRA23	-0.4131124
KLRA4	-1.0064697
KLRA5	-1.2674327
KLRA9	-1.419626
KLRB1	-1.323278
KLRB1A	-0.9170308
KLRB1C	-1.8509524
KLRB1F	-1.4029965
KLRE1	-1.8508713
KLRG1	-0.957922
KLRI1	-1.0562263
KLRK1	-0.6929007
KMO	-0.4598107
KNSTRN	-1.7864037
KNTC1	-2.638028
KPNA1	-0.3742943
KPNA2	-0.59617
KRAS	-0.6433635
KRT10	-0.5350492
KRT20	-0.8460589
KRT79	-0.7747431
KRT86	-0.6534598
KRTAP12-1	-2.177911
KRTAP20-2	-0.753474
KRTAP31-1	-0.8791618
KRTAP8-1	-2.4007423
KRTAP9-1	-0.6398819
KRTCAP3	-0.3484473
L2HGDH	-0.467345
L3HYPDH	-1.4562769
LAGE3	-0.7060628
LAMA2	-1.6241465
LAMTOR4	-0.6661105
LARP7	-0.6121707
LAT2	-1.0730219
LBH	-0.7462053
LBR	-1.5552406

LBX2	-0.7905946
LCMT1	-0.7840958
LCT	-0.7877584
LDHB	-0.8746283
LDLRAP1	-1.0226369
LEPREL1	-0.855258
LEPREL2	-0.758631
LEPROT	-0.3495998
LETM2	-1.0970557
LFNG	-0.404624
LGALS1	-0.6700482
LGALS12	-0.5523703
LGR5	-2.1348224
LHFPL2	-1.0492094
LHPP	-0.8731732
LIG1	-1.7773614
LILRA5	-1.2532284
LIMCH1	-2.6875138
LIMS1	-0.9103098
LIN37	-0.4335365
LIN54	-0.6198092
LIN9	-0.5266881
LINGO4	-1.1069913
LIPA	-1.2746997
LIPN	-1.7808075
LMAN2L	-0.5749097
LMBR1	-1.3175578
LMF2	-0.458478
LMNA	-1.979075
LMO1	-1.044807
LMO2	-1.4722238
LMTK3	-1.0441065
LNP	-0.6372943
LOC1000450	-2.359019
LOC1000488	-3.8044708
LOC1010557	-0.394083
LOC1026333	-0.6528838
LOC1026336	-1.1659806
LOC1026376	-0.9528635
LOC1026387	-0.4100683
LOC1026407	-0.4401374
LOC1026415	-1.2036295
LOC1026418	-0.4942274
LOC1026420	-0.7932539
LOC1026423	-1.5701919

LOC1026427:	-0.6608546
LOC1026428:	-0.6140847
LOH12CR1	-0.7544112
LOXL1	-0.6333256
LOXL3	-0.9994605
LPAR2	-1.0407782
LPAR5	-0.8573978
LPCAT1	-1.5724821
LPCAT3	-1.7079582
LPHN1	-0.7197392
LPIN1	-0.6343098
LPIN2	-1.0397482
LPL	-0.4226003
LRBA	-0.3638988
LRCH3	-1.0222607
LRIG3	-0.5440114
LRMP	-1.1601839
LRP2	-0.7392929
LRR1	-2.3041792
LRRC14	-0.4327359
LRRC20	-2.0022511
LRRC29	-1.3478618
LRRC30	-4.369098
LRRC36	-2.288235
LRRC4B	-2.6373615
LRRC56	-1.7376041
LRRCC1	-0.5914998
LRRFIP2	-0.3272882
LRRN1	-1.0928469
LRRN2	-2.617648
LRRN3	-1.0816345
LRWD1	-1.1981492
LSM2	-0.6865239
LSP1	-0.372983
LTA	-0.4755735
LTA4H	-1.0150971
LTB4R1	-0.9135013
LTBP1	-0.4848712
LTBP2	-1.4487162
LTBP3	-0.7791643
LTC4S	-1.9586773
LTF	-2.8951678
LURAP1L	-1.6205308
LY6G	-0.7614017
LY6G5B	-1.2269602

LY6G6C	-1.0304
LY6G6D	-1.2537997
LY6G6E	-1.0033438
LY6G6F	-2.0444272
LYL1	-1.6408892
LYNX1	-0.5001946
LYPD6	-0.6859436
LYPD6B	-0.481384
LYSMD3	-0.4677649
M1AP	-1.5336633
MAB21L2	-0.4696491
MACF1	-0.5511346
MACROD1	-0.433147
MAD1L1	-1.4051228
MAD2L1	-0.88518
MAF1	-0.4650006
MAGEB16	-2.4896798
MAGEE1	-0.6157908
MAL	-1.9823585
MALL	-0.8417218
MAN2B1	-0.9898958
MAN2B2	-0.9556303
MANBA	-0.747726
MAP2K3	-1.277205
MAP2K6	-2.004179
MAP4K2	-0.8051853
MAP4K5	-0.8072557
MAPK14	-0.6682735
MAPK3	-0.8137946
MAPK4	-1.0357244
MAPK8IP1	-1.0305281
MAPK8IP3	-0.3126173
MAPT	-1.7037702
MARK3	-0.5897951
MARVELD1	-0.4744206
MASP1	-0.6732757
MAST1	-0.810812
MAST2	-1.1321826
MAST4	-0.3541861
MASTL	-1.3354225
MATK	-0.905931
MAVS	-0.5018783
MAZ	-1.0177727
MB21D1	-0.6575885
MBD2	-0.4642797

MBD4	-1.098475
MBLAC1	-0.75944
MBNL3	-1.3590565
MBOAT2	-3.1172636
MBP	-0.4139886
MBTPS1	-0.6512632
MCCC2	-0.5009449
MCEE	-1.004081
MCM10	-1.332901
MCM2	-1.5062609
MCM3	-0.7311564
MCM4	-1.1259341
MCM5	-1.4542685
MCM6	-0.8645592
MCM7	-1.7056909
MCM8	-0.6811872
MCOLN3	-1.4389849
MCPH1	-0.8932266
MCPT8	-1.886507
MCTP1	-1.5209558
MDC1	-1.0100455
MDM1	-0.732235
MDP1	-0.4224639
ME2	-0.6431661
MEAF6	-0.379806
MED19	-0.7680483
MED20	-0.4831295
MED22	-0.6674423
MED24	-0.3355517
MED25	-0.6772394
MED4	-0.4256253
MED7	-0.3584251
MED9OS	-2.2911694
MEF2C	-0.7570462
MEGF6	-2.7645133
MEGF9	-1.329937
MEMO1	-0.7332082
MEN1	-0.3821087
MEOX1	-0.7560704
MEPE	-0.4709408
MESP2	-1.649632
MEST	-2.5747466
METAP1D	-0.6313715
METAP2	-1.158073
METRN	-0.811285

METTL24	-2.4563808
METTL8	-1.1660326
MFAP2	-1.4513881
MFAP4	-1.375463
MFAP5	-2.0759661
MFHAS1	-0.402607
MFI2	-1.2233951
MFN1	-0.7617149
MFSD11	-0.7075048
MFSD12	-1.3685656
MFSD3	-0.9380817
MGAT1	-0.3730545
MGAT4B	-1.0674458
MGLL	-1.6799517
MGP	-3.5942726
MGST2	-0.7500043
MGST3	-4.5303426
MICAL3	-1.5599968
MICALL2	-2.3199487
MID1IP1	-0.8707447
MIER1	-0.5831599
MIER3	-1.4962349
MIIP	-0.5782523
MINPP1	-1.4253602
MIOX	-1.0105357
MIS12	-1.8807054
MIS18A	-0.6588855
MIS18BP1	-1.5492234
MKI67	-1.8305154
MKKS	-0.5301485
MKLN1	-0.594214
MKNK2	-0.8116407
MKRN1	-0.9280229
MLANA	-5.161493
MLC1	-1.3203073
MLF1	-0.6440835
MLH3	-0.5727456
MLST8	-0.8784695
MMEL1	-0.9980085
MMGT1	-0.412777
MMP11	-1.2524259
MMP12	-1.0531142
MMP17	-0.660399
MMP2	-0.9743636
MMP23	-0.9357762

MMP9	-1.0223398
MMRN1	-1.394728
MN1	-1.0054524
MND1	-0.5983167
MNS1	-2.7451754
MOB2	-0.5838199
MOGAT2	-1.792141
MORN2	-1.1883152
MOSPD1	-0.6012487
MOSPD3	-1.2411447
MPC2	-0.5300889
MPG	-0.8465633
MPHOSPH6	-1.6449876
MPL	-0.7391472
MPO	-1.3247762
MPP1	-1.0656061
MPP2	-2.1135495
MPPE1	-0.8577371
MPST	-1.5617127
MPV17L2	-0.571466
MR1	-0.7032464
MRC1	-2.551157
MRC2	-1.4857278
MRI1	-0.3910751
MRM1	-1.138453
MRPL14	-0.6766653
MRPL18	-0.6295672
MRPL28	-1.0210223
MRPL4	-0.4711309
MRPL49	-0.8431425
MRPL53	-0.6061201
MRPS11	-0.6163521
MRPS15	-0.6195426
MRPS25	-0.5572929
MRS2	-0.3114646
MRVI1	-1.6184602
MS4A1	-0.6303563
MSH2	-0.7168069
MSH4	-0.5566757
MSH5	-1.8924379
MSH6	-0.7158833
MSLN	-0.4957147
MSMP	-1.1655061
MSRB2	-1.1324124
MST1	-2.2878625

MST1R	-1.1659455
MSX1	-1.4243867
MT3	-0.6639748
MTA1	-0.3752747
MTA3	-1.0246091
MTERF1B	-0.8778772
MTF1	-0.321635
MTFP1	-1.3515606
MTFR1	-1.3769064
MTHFD1	-0.6298809
MTHFSD	-0.5646212
MTL5	-0.8577738
MTMR14	-0.3824582
MTMR4	-0.6181288
MTO1	-0.4633942
MTSS1L	-2.5769074
MTURN	-1.262295
MUC6	-1.5497818
MUG1	-0.6574819
MUM1	-1.0023656
MUP19	-3.7900162
MUP4	-1.5889268
MUP-PS12	-1.1473446
MUS81	-0.5476785
MUT	-0.5973134
MVB12B	-0.938169
MVK	-0.4227638
MXD3	-3.3435059
MXD4	-0.5664907
MXRA7	-1.6882353
MXRA8	-0.68629
MYADML2	-1.6197062
MYB	-0.912231
MYBL2	-0.9393311
MYCL	-1.2836404
MYCT1	-1.3846033
MYEF2	-0.7500291
MYH10	-2.8268147
MYH11	-0.9900429
MYH7	-0.8534646
MYH8	-2.9278328
MYL10	-0.6041801
MYL12B	-0.4146247
MYL2	-0.5081773
MYL4	-0.4313073

MYL6B	-0.9818199
MYL9	-2.2166672
MYLIP	-0.3930678
MYLK3	-1.8477724
MYLPF	-1.4838705
MYO18A	-0.9523573
MYO1D	-1.0215945
MYO1F	-0.9973373
MYO9A	-0.5993276
MYOM3	-1.8572958
MZB1	-0.3875666
MZT2	-0.684701
NAA38	-0.5074315
NAAA	-1.5121918
NAALADL1	-1.8040588
NACAD	-2.9983034
NADK2	-1.6831002
NAGA	-0.9981234
NAGS	-2.7566538
NAIP2	-0.33394
NANOS1	-1.9266958
NANP	-1.5829422
NANS	-0.5082755
NAPRT1	-2.1577954
NARF	-2.2366583
NAT2	-1.0493283
NAT6	-1.6022897
NAT8L	-1.9280622
NAV1	-1.2631848
NBEAL2	-0.7571044
NBN	-0.6601236
NBR1	-0.6459026
NCAM1	-0.4687488
NCAPD2	-1.7501822
NCAPD3	-1.383944
NCAPG	-2.5317376
NCAPG2	-1.6006923
NCAPH	-1.5132117
NCAPH2	-0.9776073
NCBP1	-0.3439102
NCCRP1	-0.635392
NCOA4	-0.7320399
NCOR1	-0.5773649
NDC1	-0.8253064
NDC80	-2.0726063

NDE1	-0.8110352
NDRG4	-0.8350511
NDST3	-1.4773726
NDUFA6	-0.418148
NDUFAF3	-1.0303268
NDUFB10	-0.6927853
NDUFB2	-0.3692536
NDUFB7	-1.0804377
NDUFB8	-0.4631991
NDUFB9	-0.516408
NDUFV1	-0.5092797
NECAB3	-0.7854393
NEDD1	-0.8427787
NEDD4	-1.0024328
NEIL2	-0.6322074
NEIL3	-2.3061428
NEK1	-1.1059208
NEK2	-1.1457591
NEK8	-0.734571
NELFE	-0.6448059
NET1	-0.6096239
NETO1	-0.4908924
NEURL1A	-0.6001484
NFAM1	-1.140811
NFATC2	-0.5658116
NFE2	-2.3351388
NFIA	-1.6368606
NFIC	-0.7992673
NFIX	-1.0734243
NFYA	-0.8838925
NFYB	-0.6553569
NGFR	-1.3547626
NGFRAP1	-1.3781352
NGP	-1.4452081
NHEJ1	-1.2752838
NHLRC2	-0.657404
NHLRC4	-2.2032297
NID2	-1.4997752
NINL	-2.0157259
NIPA1	-2.0185347
NIPAL3	-1.1635003
NIT1	-0.5077496
NKAIN4	-2.5597434
NKD1	-1.28438
NKG7	-0.8025656

NKPD1	-1.1073017
NKX2-3	-1.2646785
NKX6-1	-1.7468104
NLRP4F	-1.2746086
NLRP5	-1.1720219
NLRP6	-0.8778603
NLRX1	-0.3434985
NME3	-2.0982966
NME4	-1.0460024
NME7	-0.8378313
NME9	-3.0964603
NMNAT3	-1.7212868
NMRAL1	-1.024837
NMUR1	-0.7761469
NNMT	-0.444011
NOS1	-1.3746364
NOS1AP	-0.6885111
NOSTRIN	-0.3626564
NOTCH3	-0.8447485
NOTCH4	-0.58109
NPAS4	-3.1391897
NPCD	-0.845012
NPHP1	-1.2691967
NPRL3	-0.6969862
NQO1	-2.6646712
NQO2	-0.7561879
NRARP	-1.2648976
NREP	-2.350584
NRF1	-0.8480363
NRG2	-1.3965287
NRGN	-1.5653768
NRIP3	-2.9793584
NRL	-2.3305893
NRM	-1.3655977
NRN1L	-0.4166961
NRXN2	-0.7100647
NSDHL	-0.7591963
NSL1	-1.501267
NSMCE1	-0.8074236
NT5C3	-0.7910829
NT5DC2	-1.5410306
NTF3	-1.4375684
NTN3	-1.1673999
NTN4	-2.2290494
NTRK3	-1.0054944

NUAK1	-2.3614876
NUCB1	-0.8793974
NUDT1	-0.7319517
NUDT14	-0.5296669
NUDT21	-0.3749604
NUDT4	-0.952106
NUDT7	-0.4615834
NUDT9	-0.7768569
NUF2	-1.89185
NUGGC	-1.2584062
NUP155	-0.807189
NUP35	-0.7349076
NUP85	-0.8594446
NUSAP1	-1.697331
NXNL2	-1.3428898
NXPE2	-3.6972203
NXPE4	-3.1404924
NXPE5	-1.9325399
NYAP1	-1.4032047
NYNRIN	-0.7585464
OARD1	-0.5136647
OAS1C	-0.8817759
OAZ1	-0.5684142
OCA2	-2.911308
OCM	-0.4744678
ODC1	-0.8682642
ODF3B	-2.7787461
OGDH	-0.4109464
OGN	-3.5388138
OIP5	-1.8027158
OIT3	-0.774709
OLFM1	-0.6950057
OLFML1	-1.6295285
OLFML2B	-0.6164722
OLFML3	-1.8909645
OLFR1089	-0.9445481
OLFR1121	-1.8863723
OLFR1134	-0.4464681
OLFR12	-0.4549527
OLFR1241	-0.7169604
OLFR1262	-0.3750639
OLFR1324	-0.4875021
OLFR1382	-0.5569696
OLFR1413	-0.3943727
OLFR1419	-1.021549

OLFR1463	-2.7945056
OLFR1465	-1.5538385
OLFR1490	-0.4428427
OLFR207	-0.3154171
OLFR222	-1.0434933
OLFR331	-1.0019844
OLFR362	-0.7618215
OLFR380	-0.4814794
OLFR394	-0.8861303
OLFR456	-0.4960556
OLFR495	-0.9346798
OLFR508	-0.6164315
OLFR551	-0.6937316
OLFR631	-0.7351482
OLFR649	-0.8765082
OLFR704	-2.438939
OLFR707	-0.6788642
OLFR728	-6.477909
OLFR787	-1.8234768
OLFR913	-0.7319131
OLFR94	-0.8533807
OLFR945	-0.7045836
OMA1	-0.4153988
OMP	-0.4796596
OOSP1	-0.4301372
OOSP2	-0.9386284
OPHN1	-1.6291251
OPLAH	-0.4708474
OPTN	-0.7225115
ORC1	-2.1536307
ORC5	-1.5243299
ORC6	-1.0985293
ORMDL1	-0.651988
ORMDL3	-1.0271797
OSBP	-0.4073949
OSBPL1A	-0.5489349
OSBPL8	-0.6081619
OSGEP	-0.6515226
OSGEPL1	-1.3387318
OTOP1	-1.1986544
OTUB2	-1.3425312
OXCT2A	-1.1388729
OXCT2B	-1.9832699
OXLD1	-0.7367311
OXTR	-0.6559503

P2RX1	-1.0189571
P2RY1	-1.0974984
PABPC1L	-0.4372602
PABPC4	-1.3165808
PABPC4L	-1.7959211
PABPN1L	-1.3241546
PACS1	-0.7526784
PADI2	-1.1863432
PADI4	-2.0107167
PAFAH1B3	-0.6451554
PAICS	-0.6442475
PAIP2	-0.4647999
PAK1	-1.939218
PAK3	-0.4540682
PALB2	-1.0954871
PALM	-0.8038201
PANCT2	-0.7499316
PANK1	-1.6674309
PAPLN	-1.6409919
PAQR4	-1.9892294
PAQR6	-2.4593072
PAQR9	-2.4554913
PARD3B	-0.9803028
PARD6A	-0.7717104
PARP1	-0.75034
PARP16	-1.1955245
PARP2	-0.6762781
PARPBP	-1.6708674
PARVB	-1.8808854
PARVG	-0.4657865
PASK	-1.3095746
PAX3	-2.2446218
PBK	-2.4051542
PBX2	-0.6170554
PBX4	-1.3978515
PCBD2	-0.7675552
PCCB	-0.8544066
PCDH10	-0.8377185
PCDH11X	-1.0224156
PCDH12	-2.26691
PCDH17	-0.6857307
PCDHAC2	-1.6780207
PCDHGC5	-0.9066825
PCGF1	-0.7310729
PCK1	-2.3010292

PCK2	-1.0711257
PCMT1	-0.654726
PCNA	-0.8931198
PCNP	-0.3383498
PCNT	-0.7383475
PCOLCE	-1.3599143
PCP4	-2.7627292
PCP4L1	-1.4716587
PCSK6	-1.6243615
PCSK9	-1.3963084
PCX	-1.6071548
PCYT1A	-1.4352064
PCYT1B	-2.8103712
PDAP1	-0.6911182
PDE2A	-0.6625824
PDE4A	-0.6362419
PDE6B	-0.4727726
PDE6D	-0.3428249
PDE8B	-1.1717999
PDGFC	-0.9073002
PDGFD	-2.2874832
PDIK1L	-0.6852949
PDILT	-2.5882154
PDK2	-0.9406667
PDLIM2	-0.3929429
PDLIM3	-2.7413547
PDS5A	-0.5433102
PDXP	-0.6021648
PDZD11	-0.4625363
PDZD4	-1.2281706
PDZD7	-0.715158
PDZD9	-0.6241083
PDZK1	-0.3965852
PDZK1IP1	-2.9425483
PEG12	-0.7891803
PEG13	-0.9469562
PEG3	-1.71013
PER3	-1.8300564
PEX6	-0.4610381
PF4	-0.6455636
PFKFB2	-0.8199627
PFKM	-2.535658
PFN3	-1.0538514
PGAM2	-1.1663089
PGAP1	-0.9927635

PGAP3	-0.9147167
PGLYRP1	-0.9673352
PGM2L1	-0.9587855
PGP	-1.0836143
PGRMC2	-0.5389333
PHACTR2	-0.7227888
PHACTR4	-0.7895484
PHF10	-1.1605816
PHF19	-0.4451351
PHF2	-0.5754638
PHF5A	-0.9952087
PHKA2	-0.8349328
PHKB	-0.5887156
PHKG1	-0.3992333
PHLDA3	-0.9220724
PHLDB2	-0.7062588
PHLPP2	-0.8087597
PHOSPHO2	-0.3193772
PHYHIP	-4.005094
PI16	-1.4626698
PI4K2B	-1.2941351
PIANP	-0.8163948
PIDD1	-1.3441658
PIF1	-0.9395118
PIGH	-0.9940226
PIGP	-0.4538388
PIGQ	-2.1032028
PIGYL	-0.5538306
PIH1D1	-0.5677295
PIK3C2B	-0.5981677
PIK3R2	-1.1634607
PIK3R3	-1.3433583
PIK3R4	-0.4435463
PILRB2	-0.7212377
PINK1	-0.7300458
PIP4K2C	-0.6503706
PIP5KL1	-1.1980155
PIR	-1.9881103
PISD-PS2	-0.4249861
PITHD1	-0.6101589
PITPNM2	-0.6282811
PITPNM3	-2.5892076
PKHD1L1	-2.5528128
PKIA	-0.9284642
PKLR	-4.154049

PKMYT1	-1.2424212
PKN3	-0.5365982
PKNOX1	-0.8204186
PKNOX2	-1.0630071
PLA2G12A	-0.9521465
PLA2G15	-0.9492793
PLA2G2E	-2.664668
PLA2G4C	-1.7121933
PLCL2	-0.8897944
PLD4	-1.7074504
PLEK2	-2.852916
PLEKHA6	-2.642561
PLEKHF2	-0.8699775
PLEKHG5	-0.8346481
PLEKHJ1	-0.8362932
PLIN1	-0.9633138
PLIN3	-0.5615878
PLK1	-2.0595121
PLK4	-1.8608878
PLS1	-1.2064035
PLSCR2	-1.1316831
PLSCR4	-1.550008
PLXDC1	-2.2239177
PLXDC2	-0.6372404
PLXNA4	-1.7570076
PLXNB1	-1.0220051
PLXNB3	-2.078739
PMCH	-0.8790298
PMEL	-5.1614447
PMF1	-0.9017386
PMM1	-1.2736392
PMS2	-1.2533116
PNCK	-0.93679
PNKP	-0.5541401
PNLDC1	-0.955672
PNMA3	-0.6417174
PNP	-0.696425
PNP2	-2.4229863
PNPLA6	-0.5989351
PNPLA7	-0.6713247
PNPO	-1.1366644
POC1A	-1.3621755
PODN	-2.2697744
PODNL1	-0.8245063
PODXL	-1.5767388

PODXL2	-1.4636297
POLA2	-0.955754
POLD1	-1.2624621
POLD4	-0.7046557
POLE	-0.7156582
POLE2	-1.2582831
POLE3	-0.7651691
POLH	-1.0817149
POLI	-0.7773848
POLQ	-1.9351344
POLR2A	-0.447227
POLR3H	-0.3523302
POMK	-0.4480448
POMT1	-0.8347614
PON2	-0.6097388
POPDC2	-3.0675144
POPDC3	-1.9850099
POSTN	-1.1519725
POU2F1	-0.3209624
POU3F4	-1.2071042
PPA2	-0.5568733
PPAPDC3	-1.2482471
PPARG	-1.5010853
PPBP	-1.4106808
PPCDC	-0.4313352
PPCS	-0.5439172
PPDPF	-0.3145905
PPEF2	-1.0007374
PPFIA4	-2.7543845
PPFIBP2	-0.4218783
PPIL2	-0.6523757
PPM1D	-0.8219771
PPM1E	-1.8204503
PPM1G	-0.6421118
PPM1L	-1.217022
PPOX	-2.8956127
PPP1CA	-0.7296333
PPP1CB	-0.6771293
PPP1CC	-0.3416872
PPP1R14A	-1.3351154
PPP1R15A	-0.7763987
PPP1R1B	-0.8497617
PPP1R35	-1.3453684
PPP1R37	-0.8251743
PPP1R3C	-0.9548416

PPP1R9A	-1.7330098
PPP1R9B	-0.7966657
PPP2R1B	-0.7991984
PPP2R2B	-1.7352362
PPP2R4	-1.2409143
PPP2R5B	-1.7358146
PPP2R5D	-0.3759503
PPP2R5E	-0.6330669
PPP4C	-0.406127
PPP6R2	-0.505971
PPWD1	-0.6035729
PQLC1	-1.7314153
PQLC3	-0.5527873
PRADC1	-0.9496393
PRAM1	-0.7747512
PRC1	-2.1580925
PRCP	-0.7052054
PRDM10	-0.7616501
PRDX2	-1.9524717
PRDX3	-0.8414478
PREPL	-0.5179651
PRG2	-1.7142081
PRG3	-1.9266691
PRIM1	-1.7230282
PRIM2	-1.388658
PRKAA1	-0.5082994
PRKAA2	-1.8102326
PRKAB1	-0.9287872
PRKAB2	-0.4140329
PRKAG1	-1.1251101
PRKAR1B	-0.7046053
PRKAR2B	-2.0842352
PRKCG	-1.9001255
PRKD2	-0.3508039
PRKDC	-0.8214793
PRKG1	-1.5208626
PRKRA	-0.8655047
PRL2A1	-3.2434871
PRL4A1	-4.666154
PRN	-0.6127715
PRNP	-0.4345336
PROCA1	-0.9354489
PRODH	-0.4860687
PROKR1	-0.6067309
PROKR2	-0.9134035

PROSER2	-0.5480604
PRPH	-1.3615851
PRR11	-1.5158265
PRR12	-0.3190935
PRR5L	-1.1932528
PRRX2	-1.2327926
PRSS27	-1.2221179
PRSS29	-0.7826846
PRSS33	-0.721113
PRSS34	-2.1346827
PRSS50	-2.9330242
PRSS57	-2.0871658
PRTN3	-1.5538397
PRX	-0.5910637
PSAP	-0.8543129
PSD	-0.8708925
PSIP1	-0.719491
PSMA8	-0.5340009
PSMC3IP	-1.2314875
PSMD5	-1.1891332
PSMD9	-1.1959243
PSMF1	-1.1535902
PSORS1C2	-1.3518274
PSPC1	-0.4950519
PSRC1	-1.6772912
PSTPIP1	-0.4295383
PTDSS2	-2.4592774
PTGER1	-1.5761874
PTGR1	-1.5254683
PTGR2	-0.8882976
PTGS1	-1.0219135
PTH1R	-0.7579351
PTN	-1.1682787
PTOV1	-1.0743623
PTP4A3	-1.6858025
PTPLA	-0.9245429
PTPLAD2	-2.486733
PTPN14	-1.3860872
PTPN6	-0.4273071
PTPN7	-1.3938813
PTPRB	-0.5932322
PTPRO	-2.264373
PTPRV	-1.1802051
PTRF	-0.5906482
PTS	-0.797091

PTTG1	-1.0660825
PUM2	-0.3354187
PURB	-0.3459683
PURG	-0.69736
PVRL3	-0.688045
PWWP2A	-0.3694377
PWWP2B	-0.5248056
PXMP2	-1.1476018
PXMP4	-0.8283711
PXYLP1	-1.1746299
PYCR2	-0.6407342
PYGL	-0.528007
PYGM	-1.0343897
PYROXD2	-2.1321752
QARS	-0.3842983
QPCT	-1.2725589
R3HDM4	-1.2723093
R3HDML	-0.7511635
RAB24	-0.3848147
RAB27B	-1.3615377
RAB36	-1.9159601
RAB37	-0.73735
RAB38	-0.4214096
RAB3B	-1.653173
RAB3D	-0.3481703
RAB3IL1	-2.3121333
RAB4A	-0.7082372
RAB6B	-1.427557
RABGEF1	-0.5078745
RACGAP1	-1.2534709
RAD18	-0.5979939
RAD21	-0.5934281
RAD23A	-1.3744135
RAD50	-0.4719997
RAD51	-1.1948571
RAD51AP1	-1.6844754
RAD51B	-1.0863619
RAD52	-0.6027398
RAD54B	-0.9769633
RAD54L	-1.8720803
RAG1	-2.954071
RAG2	-2.7891526
RAI2	-0.974469
RALBP1	-0.3870463
RAMP1	-1.441761

RANBP10	-1.7170672
RANBP6	-1.2763722
RANGAP1	-0.8394542
RAP1GAP	-0.9844503
RAP2C	-1.190022
RAPGEF4	-0.8537209
RAPGEFL1	-2.981598
RARG	-0.6024408
RASD2	-0.9415593
RASGEF1C	-1.9821575
RASGRP2	-1.5233583
RASGRP4	-1.4608076
RASL11A	-1.2400935
RASSF6	-0.7419417
RASSF7	-1.4818573
RB1	-1.4092555
RBBP8	-0.6425767
RBFA	-0.4139605
RBFOX1	-2.093436
RBKS	-0.8872728
RBL1	-0.7991166
RBM12B2	-0.4209867
RBM15	-0.4097552
RBM24	-0.7623155
RBM38	-1.7674932
RBM4	-0.6811543
RBM43	-0.4330888
RBM4B	-0.3712831
RBMX2	-0.3182108
RBP7	-2.4487152
RCAN2	-0.5629494
RCAN3	-0.6164346
RCBTB2	-0.4879198
RCHY1	-0.3797216
RCN3	-0.8610377
RCOR1	-0.3686318
RCOR2	-1.0999019
RCOR3	-0.7085185
RDH10	-0.4056478
RDH18-PS	-0.698395
RDM1	-1.2323427
REC8	-0.7474408
RECQL	-0.688005
RECQL4	-1.6129813
RECQL5	-0.8534646

REDRUM	-2.973472
REEP1	-1.1589782
REEP2	-0.4950633
REEP4	-0.9792104
REEP5	-0.8545332
REEP6	-2.0178227
REG4	-0.7216051
RELL1	-1.1264591
RELL2	-2.2361636
RELN	-1.3470926
RELT	-0.6496959
REPS2	-1.9835317
RETN	-1.3905694
RETNLA	-2.8797104
RETNLG	-1.7779346
REXO2	-0.3359451
RFC1	-0.9011383
RFC2	-1.0550656
RFC3	-0.4694762
RFC5	-0.6949034
RFESD	-2.008132
RFK	-0.3796682
RFNG	-0.8232546
RFWD2	-0.3532071
RFWD3	-1.1020336
RFX1	-0.4105463
RFX2	-3.6055422
RFXANK	-0.798696
RFXAP	-0.7708013
RGCC	-1.1686978
RGL2	-0.7173438
RGL3	-0.4597492
RGS10	-2.2404823
RGS12	-2.019062
RGS14	-0.9199629
RGS18	-0.835393
RGS2	-1.0411372
RGS3	-0.6141634
RGS7	-1.683485
RGS7BP	-1.4526889
RHAG	-2.1571455
RHBDL3	-0.6411562
RHD	-3.4256363
RHNO1	-0.9259486
RHOT2	-0.4959016

RHOX2H	-2.2146542
RHOX5	-0.7745595
RIBC1	-0.408977
RILP	-0.3770056
RIMBP3	-0.9184065
RIMS1	-0.5392721
RIMS3	-1.424653
RINL	-0.3688555
RIPPLY2	-0.7831583
RMI1	-0.9999075
RMND5B	-0.5603643
RNASE12	-2.2278323
RNASE2A	-1.249917
RNASE6	-1.5369296
RNASEH2A	-1.0780134
RNASEH2B	-1.0949106
RNASEH2C	-1.3603902
RNASEK	-0.4458494
RNASEL	-1.372242
RND2	-0.8927219
RNF10	-0.8129344
RNF11	-1.1068859
RNF123	-1.9924998
RNF139	-0.6040139
RNF144A	-1.283627
RNF144B	-2.1460145
RNF150	-1.4109173
RNF152	-2.1214056
RNF166	-0.7557902
RNF167	-0.6081109
RNF168	-0.611506
RNF187	-1.0181608
RNF212	-5.223195
RNF26	-0.9484778
RNF38	-0.7816391
RNF43	-1.0209603
RNF5	-0.7761664
RNGTT	-0.5527825
RNU2-10	-0.4643474
ROBO2	-1.933681
ROGDI	-0.6309423
ROPN1L	-0.4559901
RP9	-0.5263743
RPA1	-0.8829103
RPA2	-1.1246543

RPA3	-0.9733629
RPAIN	-0.8008251
RPGRIP1L	-1.156245
RPH3AL	-0.6511946
RPIA	-0.7038164
RPL10L	-0.4775999
RPL21	-0.3872271
RPL3L	-1.2275405
RPP25L	-0.8998146
RPRD1B	-1.1024675
RPS6KA1	-0.5566006
RPS6KA5	-0.8014002
RPS6KB2	-0.3843093
RPS7	-0.4272852
RRM1	-2.1023746
RRM2	-1.9900765
RSF1	-0.4109147
RSLCAN18	-0.9224415
RSPH1	-1.2814827
RSPO1	-2.5622497
RSPRY1	-0.6613183
RTN4RL1	-1.5095189
RTTN	-0.5841343
RUNX1T1	-0.7785826
RXFP3	-2.0904524
RYK	-1.0441694
RYR1	-1.113039
S100A1	-0.7248931
S100A10	-1.1063452
S100A11	-0.6841078
S100A14	-0.8525505
S100A2	-0.5949042
S100A4	-0.8029013
S100A5	-0.7861946
S100A6	-0.4388447
S100B	-4.594967
S1PR2	-0.6019774
S1PR4	-0.7595668
S1PR5	-3.6651103
SALL2	-0.6148982
SAMD1	-0.8507915
SAMD11	-2.5329223
SAMD14	-2.1299624
SAMD3	-0.8240216
SAMD4	-2.4862707

SAMD5	-1.2613659
SAPCD1	-1.7350268
SAPCD2	-0.9773717
SARDH	-1.028038
SASS6	-0.9835217
SBK1	-1.4979806
SCAF1	-0.6524873
SCARB1	-0.3803692
SCD2	-0.9383106
SCEL	-1.1972139
SCG5	-1.0531044
SCGB2B15	-0.4248149
SCLT1	-0.6978905
SCLY	-0.9315004
SCMH1	-1.0233676
SCML2	-2.319101
SCN2B	-1.1893096
SCN7A	-0.8672009
SCNM1	-0.3452401
SCNN1A	-2.566139
SCOC	-1.1737292
SCP2	-0.3971224
SCRN1	-1.7999382
SCRN3	-2.4146786
SCUBE1	-1.9260068
SCUBE2	-0.783809
SCUBE3	-1.1139922
SDCCAG8	-0.5710373
SDHD	-0.6299262
SDK2	-1.3617821
SDPR	-2.3797297
SDSL	-1.4152865
SEC14L2	-4.9944715
SEC14L3	-1.0037134
SEC16B	-1.605161
SECTM1A	-2.0229735
SELENBP1	-2.7169728
SEMA3D	-0.9341354
SEMA4B	-0.7363944
SEMA4G	-1.0802596
SEPHS1	-0.6247013
SERBP1	-0.3152356
SERHL	-0.3401775
SERINC5	-0.8971839
SERPINA3A	-2.398395

SERPINB10	-2.1445255
SERPINB3C	-1.0774322
SERPINF1	-1.3899798
SERTAD2	-0.6596799
SETD1B	-0.6926394
SETD8	-0.7861891
SFRP1	-2.072903
SFRP2	-1.9609427
SFRP4	-2.5728414
SFXN4	-0.8314681
SGCE	-0.4618125
SGCG	-1.4211829
SGK3	-0.657712
SGOL1	-2.0514326
SGOL2	-2.0512824
SH2D1B1	-1.7829444
SH2D3C	-0.5980759
SH2D4A	-3.4723032
SH2D7	-0.4999511
SH3BGRL2	-0.4784601
SH3BGRL3	-0.5339708
SH3BP1	-0.5593243
SH3D19	-0.3466518
SH3GLB2	-0.5003119
SH3KBP1	-0.542726
SH3PXD2A	-0.7576857
SH3RF2	-2.7884579
SH3TC2	-3.1494622
SH3YL1	-1.6253474
SHANK2	-0.4503341
SHCBP1	-0.9760428
SHISA2	-1.4643848
SHISA4	-1.1154573
SHISA9	-1.2502236
SHROOM3	-0.7136686
SIGLEC5	-0.9199536
SIGLECG	-0.8826842
SIK2	-0.5593786
SIPA1	-0.8947101
SIPA1L3	-0.6953864
SIRPB1A	-1.523891
SIRPB1B	-1.4344859
SIRT3	-0.6367612
SIRT7	-0.4179478
SIVA1	-1.071001

SIX5	-0.5879471
SKA1	-1.8753333
SKA2	-2.1460564
SKA3	-2.4134493
SKAP1	-0.5630121
SKP2	-1.069937
SLBP	-1.9898453
SLC11A2	-0.9219732
SLC13A2	-2.485071
SLC13A3	-1.3632152
SLC14A1	-2.739832
SLC16A10	-1.7685068
SLC16A12	-1.4757757
SLC16A7	-0.5582738
SLC16A8	-0.7060471
SLC18B1	-0.5630803
SLC1A5	-0.8975625
SLC20A1	-0.6265245
SLC22A13B-F	-0.7281182
SLC22A20	-1.8346856
SLC22A23	-2.2350886
SLC22A4	-2.0938063
SLC24A3	-1.1543436
SLC24A5	-0.9604383
SLC25A1	-0.5525541
SLC25A21	-2.2855597
SLC25A37	-1.9666443
SLC25A38	-1.9760561
SLC25A39	-0.6793861
SLC25A42	-1.4847941
SLC25A44	-0.5590916
SLC25A51	-1.2259479
SLC26A1	-2.3831086
SLC26A10	-0.8956423
SLC29A1	-1.1457219
SLC29A4	-1.2147183
SLC2A3	-0.6556406
SLC2A4	-3.126202
SLC2A8	-1.0217123
SLC30A1	-0.6304498
SLC30A10	-4.255364
SLC30A9	-1.0116205
SLC35B2	-0.3958321
SLC37A2	-1.3933289
SLC38A5	-3.9150515

SLC38A8	-1.2275167
SLC39A8	-0.5149517
SLC3A1	-1.8977101
SLC40A1	-3.8984184
SLC41A3	-0.6288552
SLC43A1	-2.288322
SLC43A3	-0.4577351
SLC45A2	-2.08886
SLC45A4	-0.5170436
SLC46A1	-0.5685482
SLC46A3	-1.7989712
SLC48A1	-0.7030988
SLC4A1	-1.8819761
SLC51A	-2.1779876
SLC5A11	-0.9755049
SLC5A2	-2.1309226
SLC6A12	-1.3668015
SLC6A13	-1.8383965
SLC6A20A	-2.2920642
SLC6A3	-1.4310727
SLC6A4	-1.5040703
SLC6A9	-1.9224167
SLC7A10	-1.0978539
SLC7A4	-1.7356114
SLC9A3R2	-1.6563447
SLC9A9	-1.9362559
SLC9B2	-0.9895096
SLCO2B1	-1.2759838
SLFN14	-1.4765537
SLIT1	-0.5174491
SLN	-4.123721
SLX4	-0.6057096
SMAD6	-0.3753259
SMARCA2	-0.6162243
SMARCA5-PS	-0.5676456
SMARCAL1	-0.5092955
SMARCC2	-0.9503799
SMARCD2	-0.6013961
SMC1A	-0.5787811
SMC2	-1.3421993
SMC3	-1.0015755
SMC4	-1.2233105
SMCO1	-0.59447
SMG5	-0.3615909
SMIM1	-4.365842

SMIM11	-0.4824009
SMIM14	-0.4437742
SMIM24	-1.2480767
SMIM5	-3.734016
SMIM6	-2.375866
SMO	-0.7278748
SMOX	-1.3096366
SMPD1	-0.546473
SMPD3	-1.1697257
SMPD4	-0.8791995
SNAI2	-1.099792
SNAP91	-0.9144592
SNCA	-1.5203872
SNCAIP	-1.8510308
SNED1	-2.7186582
SNIP1	-0.7554531
SNN	-0.9562912
SNRNP25	-1.318655
SNRPN	-0.5022481
SNX15	-1.2541566
SNX21	-0.5695214
SNX22	-3.5695527
SNX3	-0.4516311
SNX32	-1.550739
SOBP	-0.7102337
SORCS2	-0.8293185
SORD	-1.3048782
SORT1	-0.8600707
SOWAHA	-1.7151566
SOX10	-2.8606513
SOX17	-0.5625126
SOX18	-1.3950238
SOX4	-1.1391759
SOX6	-2.9213505
SOX6OS	-1.8702505
SP3OS	-0.3966169
SPAG5	-2.0093508
SPARCL1	-0.3992524
SPATA24	-1.4971075
SPC24	-1.6243105
SPC25	-1.8267708
SPDL1	-1.258193
SPECC1	-0.7606368
SPEER4C	-0.4115272
SPHK1	-0.4100676

SPIB	-0.4974012
SPICE1	-0.6645589
SPIN2C	-2.013157
SPINK2	-1.4390185
SPIRE1	-2.2157876
SPIRE2	-1.0703225
SPNS2	-0.5982704
SPNS3	-1.5170908
SPON2	-4.501051
SPPL2B	-1.805409
SPPL3	-0.8667502
SPRR1A	-1.2615681
SPRYD4	-0.3286896
SPSB3	-0.9126139
SPTA1	-2.5968266
SPTB	-1.7654812
SQLE	-0.6776371
SRBD1	-0.4461446
SRD5A1	-1.7898452
SREBF1	-0.7425213
SREK1IP1	-0.4690018
SRGAP3	-1.4303594
SRPK3	-2.4679403
SRPX	-1.5950394
SRR	-0.8249674
SRSF1	-0.591711
SRSF10	-0.3915043
SRSF2	-0.3107147
SSBP3	-1.6606617
SSC5D	-0.3657184
SSH1	-0.504698
SSMEM1	-0.3777072
SSNA1	-1.4411225
SSPN	-1.6155214
SSX2IP	-2.342643
SSXB10	-0.4517541
SSXB9	-0.8790059
ST3GAL1	-0.4651246
ST3GAL2	-1.5970106
ST3GAL3	-0.7524929
ST3GAL5	-2.3258667
ST3GAL6	-1.4954805
ST5	-2.000883
STAB2	-1.3001533
STAC2	-0.7568488

STAP1	-0.7521858
STAR	-1.526155
STARD10	-2.7577643
STARD8	-1.024111
STBD1	-0.7207031
STEAP3	-2.7736268
STIL	-2.3243885
STIM1	-0.3220553
STK10	-0.5472307
STK11	-0.7740669
STK16	-1.2968388
STK35	-0.5936396
STMN1	-1.7223401
STOM	-2.3113627
STOML2	-0.3289022
STOX1	-0.8674808
STRA13	-0.7256093
STRADB	-1.9456973
STRBP	-0.6556692
STRM	-0.5738134
STRN	-0.8283341
STX17	-0.4724774
STX2	-1.9743519
STXBP4	-0.8165453
STYXL1	-1.0992379
SUCNR1	-1.0802221
SULF2	-0.3416519
SULT5A1	-2.130644
SUN1	-1.0884414
SUOX	-1.6278296
SURF1	-0.4350605
SUSD1	-0.8677607
SUSD3	-0.4276846
SUV420H2	-1.5112948
SVEP1	-1.5553203
SVIP	-3.3769724
SVOPL	-1.0659022
SVS5	-0.6702833
SWSAP1	-0.373265
SYBU	-0.5385876
SYCE2	-1.3438349
SYNE1	-1.672327
SYNE3	-2.056462
SYNGR1	-2.4815023
SYNGR4	-1.528373

SYNM	-1.4422832
SYNRG	-0.449038
SYT1	-0.6717236
SYT15	-1.0545733
SYTL1	-0.9801893
SYTL2	-1.0260215
SYTL4	-1.0361423
TAAR3	-10.162707
TAAR4	-2.7224984
TAAR7F	-0.4476719
TAB1	-1.0941672
TAB2	-0.3283291
TAB3	-0.564723
TAC2	-6.119728
TACC3	-1.3683324
TAF12	-0.6033449
TAF5	-0.5600576
TAF6	-0.6419821
TAF8	-0.5285773
TAF9B	-0.4526188
TAL1	-2.9173036
TALDO1	-0.7625623
TANGO2	-1.389647
TAPT1	-1.1247373
TARSL2	-2.2034683
TBC1D10B	-0.3771615
TBC1D10C	-0.7076116
TBC1D24	-0.7522557
TBC1D31	-1.1937721
TBC1D32	-0.4937446
TBCCD1	-1.4293461
TBCD	-0.3849716
TBX2	-0.4783852
TBX6	-0.5726826
TBXA2R	-1.2900686
TBXAS1	-1.6771088
TCEA3	-1.581516
TCEAL3	-0.4727445
TCEAL6	-0.5974398
TCEAL7	-1.6250379
TCEAL8	-1.1063614
TCF15	-3.860123
TCF19	-1.7656093
TCF21	-2.7493396
TCF3	-1.0965586

TCF7L1	-1.3136182
TCHP	-0.3860941
TCL1B3	-0.570977
TCP11	-1.4841738
TCP11L2	-0.5275154
TCRG-V1	-0.4902959
TCSTV3	-0.8070874
TCTEX1D1	-0.5737298
TCTN1	-0.9906888
TEC	-0.4823556
TECR	-1.1304569
TEK	-1.4109261
TEKT2	-0.7951665
TEN1	-0.3723512
TERF2IP	-0.3861969
TERT	-0.6963427
TESC	-0.9288721
TESK2	-0.5611198
TET1	-0.7493262
TEX15	-1.2939315
TEX38	-0.9307194
TEX9	-0.9062161
TFB1M	-0.9664688
TFCP2	-0.3853533
TFDP1	-0.7545972
TFDP2	-1.5356503
TFEB	-1.2805114
TFF1	-1.0080152
TFPI2	-0.7810993
TFR2	-2.6423311
TFRC	-2.3244152
TGFB1	-0.7102079
TGFB2	-0.7353251
TGFBI	-1.0404158
THAP11	-0.6127205
THAP6	-1.0815015
THBS3	-0.3728352
THEM6	-1.0002813
THNSL2	-0.8769281
THRA	-1.0973787
THRSP	-4.52496
THSD1	-0.5870378
THSD4	-0.5013661
THSD7B	-2.0275722
THTPA	-0.4269958

THY1	-1.028482
TIA1	-0.4323523
TIAM2	-1.345269
TICRR	-1.4956627
TIGD3	-2.0917315
TIMD4	-1.1996765
TIMELESS	-0.734376
TIMM17B	-0.8544555
TIMP2	-1.0784769
TIMP4	-0.7800574
TIPIN	-1.3106294
TJP3	-0.3795939
TK1	-1.9635735
TKTL1	-0.9004457
TLCD1	-1.6682277
TLCD2	-1.0031147
TLE2	-0.8239355
TLR11	-2.5383434
TLR12	-0.9953251
TLX1	-2.6635153
TM6SF1	-0.7148166
TM7SF2	-0.9501519
TM7SF3	-0.8498569
TMC6	-1.3670664
TMC7	-1.132031
TMC8	-1.1651926
TMCC2	-1.1493769
TMEM100	-1.5048885
TMEM106C	-0.5289516
TMEM107	-1.8827748
TMEM120B	-1.9851303
TMEM121	-1.5738428
TMEM129	-0.8737154
TMEM132A	-2.2968473
TMEM132C	-2.7246127
TMEM134	-0.5874882
TMEM136	-0.4683719
TMEM138	-0.9981496
TMEM141	-1.1827044
TMEM14A	-0.3925004
TMEM14C	-2.2351537
TMEM150B	-1.0458121
TMEM154	-0.9927921
TMEM174	-0.5206621
TMEM178B	-0.4409704

TMEM179B	-0.5430141
TMEM180	-1.1299694
TMEM183A	-0.5168619
TMEM184A	-0.7769318
TMEM191C	-0.9583745
TMEM194	-0.5411248
TMEM200A	-2.5058532
TMEM204	-0.8463752
TMEM216	-1.0024128
TMEM218	-0.8679709
TMEM229A	-2.11955
TMEM234	-0.485424
TMEM237	-0.8583026
TMEM246	-1.4326372
TMEM255A	-1.4397767
TMEM256	-1.0883374
TMEM29	-0.6045013
TMEM33	-0.5853238
TMEM35	-1.3905401
TMEM37	-1.0774593
TMEM40	-1.2959824
TMEM41B	-0.5866761
TMEM44	-1.9036043
TMEM54	-0.702059
TMEM55A	-0.6759229
TMEM56	-2.9727201
TMEM57	-0.4848156
TMEM65	-0.3913338
TMEM8	-1.2596722
TMEM80	-0.3961849
TMEM81	-0.383498
TMEM86A	-1.2783518
TMEM86B	-2.0177977
TMEM9	-1.534483
TMEM91	-0.4054506
TMEM98	-1.7712071
TMLHE	-0.514235
TMOD1	-1.9196672
TMPO	-0.9112902
TMPRSS13	-1.6770277
TMPRSS6	-2.1312277
TMTC3	-0.4118679
TMUB2	-0.4799261
TMX2	-0.6180506
TMX4	-1.1502686

TNFAIP8L2	-0.5984659
TNFRSF14	-0.7836328
TNFRSF17	-2.119503
TNFRSF19	-0.9592145
TNFSF12TNF!	-0.8097687
TNFSF13B	-0.4282246
TNIK	-1.1322305
TNK1	-2.0980513
TNK2	-1.1399546
TNNI2	-1.8188615
TNNT2	-2.75401
TNPO2	-0.6302028
TNRC6B	-1.0983315
TNXB	-2.413766
TOB1	-1.0741994
TOM1L1	-1.8113236
TONSL	-2.681294
TOP2A	-1.6254072
TOPBP1	-0.4579272
TOR4A	-0.3681417
TPCN1	-2.531269
TPM1	-0.5485244
TPM2	-1.0726013
TPMT	-1.6721544
TPP1	-0.6140404
TPPP	-3.6736088
TPPP3	-1.4825418
TPRA1	-0.8751063
TPRGL	-0.658793
TPX2	-1.925436
TRAK2	-1.3709083
TRAM1	-0.3756347
TRAPPC10	-0.5597
TREM2	-2.5127184
TREML1	-2.4763613
TREML4	-0.7972698
TRERF1	-0.5538387
TREX2	-1.8500831
TRF	-0.8517785
TRIAP1	-0.5100293
TRIM10	-2.6749501
TRIM15	-0.6143496
TRIM2	-0.8269899
TRIM23	-0.7720256
TRIM24	-0.6222467

TRIM37	-0.494817
TRIM43B	-1.4942076
TRIM45	-0.6900868
TRIM59	-1.4205241
TRIM7	-0.7800412
TRIP13	-0.3124576
TRIP4	-0.38304
TRMT12	-1.4992449
TRMT1L	-0.382719
TRMT44	-0.8057714
TROAP	-1.7894442
TRP53BP1	-0.3607802
TSC22D1	-1.5109162
TSC22D4	-0.5148516
TSHZ1	-0.7515049
TSPAN10	-2.4534724
TSPAN15	-0.5289161
TSPAN18	-0.9392767
TSPAN2	-1.799156
TSPAN32	-0.8514767
TSPAN33	-2.2542715
TSPAN7	-0.5637627
TSPAN8	-2.3860998
TSPO2	-1.4330969
TST	-1.7394576
TSTD3	-1.1873822
TTC26	-0.4975212
TTC34	-0.745075
TTC39A	-2.3347144
TTC7	-0.6306944
TTC9C	-0.4421592
TTK	-2.0756125
TTLL1	-0.8679385
TTLL10	-0.4478965
TTLL12	-1.8085847
TTLL5	-1.6937337
TUBA1B	-0.4459519
TUBA1C	-0.4169369
TUBA3A	-0.872793
TUBA4A	-1.2009687
TUBA8	-0.9512868
TUBB1	-1.4548006
TUBB4A	-0.86272
TUBB4B	-1.2198629
TUBD1	-0.6458316

TUBE1	-0.6273871
TUBG1	-1.231287
TUBG2	-0.598371
TUBGCP2	-0.7709694
TUBGCP3	-0.9244914
TUBGCP4	-0.8181558
TUFT1	-0.8047164
TUSC1	-1.0679455
TUSC2	-0.4618778
TUSC3	-0.4643278
TVP23A	-0.9142139
TXN2	-0.3507986
TXNDC5	-0.6911597
TXNL1	-0.8029249
TXNRD2	-1.7519541
TYMS	-0.8537488
TYMS-PS	-1.5053072
TYROBP	-0.6153026
TYRP1	-8.437507
TYW1	-0.3351073
UAP1	-0.3196616
UBAC1	-2.528728
UBASH3A	-0.7972829
UBE2C	-1.7814035
UBE2E3	-0.9494491
UBE2H	-0.4012036
UBE2L6	-0.7138934
UBE2O	-1.1307254
UBE2R2	-0.4933186
UBE2T	-1.8863976
UBE3B	-0.6117678
UBIAD1	-2.347414
UBL7	-0.4107285
UBR7	-0.564467
UBXN2B	-0.9485545
UCP2	-0.769125
UCP3	-1.0331359
UEVLD	-0.8185246
UFSP1	-1.6669474
UGCG	-0.3187776
UGT1A6B	-0.7136421
UHRF1	-1.482368
ULBP1	-1.2709765
ULK2	-0.3529866
UNC119	-1.0474124

UNC45BOS	-0.8643858
UNC5CL	-1.7823901
UNCX	-0.5120952
UPB1	-1.6478212
UPK1B	-0.3728056
UPK3A	-1.5639088
UPK3B	-1.8246441
UQCR11	-0.530787
UQCRC1	-0.5856834
UROD	-1.9310169
UROS	-2.7096224
USHBP1	-1.2819369
USMG2	-1.6522915
USP15	-0.9604788
USP17LD	-1.6536975
USP21	-0.7975473
USP25	-0.7772727
USP32	-1.1139402
USP39	-0.7527504
USP45	-0.7038174
USP46	-1.3992808
USP49	-1.9961588
USP6NL	-0.5904861
USP7	-0.5949631
USP8	-0.4888921
VAMP1	-0.9540844
VAMP8	-0.5090179
VANGL1	-1.9916453
VAT1L	-0.9367743
VAV3	-0.4829536
VBP1	-0.4080229
VCAM1	-0.6094694
VCL	-1.2985477
VEGFB	-0.5722804
VEZF1	-0.6871033
VKORC1	-0.6267805
VKORC1L1	-0.6216221
VMN1R122	-1.4700313
VMN1R132	-0.5738678
VMN1R195	-2.0832984
VMN1R197	-1.6070704
VMN1R219	-1.5680962
VMN1R234	-0.5467486
VMN1R25	-0.8646095
VMN1R41	-0.7337942

VMN1R74	-2.9172711
VMN2R10	-1.7051015
VMN2R7	-0.582206
VOPP1	-0.7242374
VPREB1	-2.096557
VPREB3	-1.4225216
VPS13A	-0.3593421
VPS13C	-0.7841926
VPS25	-0.5641866
VPS26B	-0.4948831
VPS35	-0.6320682
VRK1	-1.3804326
VRK2	-0.7176719
VSIG4	-2.4368958
VSTM4	-0.8785117
VSTM5	-0.5181606
VWA1	-1.4533968
VWA3A	-3.8856685
VWCE	-2.7832003
VWF	-1.4709516
WAC	-0.4920254
WAPAL	-0.5867872
WAS	-0.4186764
WBP1	-1.4936666
WBSCR27	-1.6133857
WBSCR28	-0.407249
WDPCP	-0.5973694
WDR26	-0.6838183
WDR35	-1.0014744
WDR6	-0.7582459
WDR60	-1.1727331
WDR62	-2.1354508
WDR65	-2.9483433
WDR70	-0.4610961
WDR72	-2.2513247
WDR76	-0.9770551
WDR81	-0.6817017
WDR83	-0.5708356
WDR86	-0.780231
WDR90	-1.7712171
WDSUB1	-0.7812901
WDTC1	-0.3424168
WEE1	-1.0078065
WFDC1	-2.072965
WFDC10	-2.553173

WHSC1	-0.6954453
WIPI1	-0.9751611
WIPI2	-0.7965689
WISP2	-3.4075687
WNK4	-1.2165132
WNT10A	-1.420605
WNT4	-0.4972496
WNT5A	-2.0131857
WRN	-1.6708739
WSCD1	-0.9240599
WWP1	-0.824692
XCL1	-1.535325
XCR1	-2.6582115
XK	-2.5756915
XKR5	-1.7941473
XKRX	-1.6937683
XPC	-0.8555665
XPNPEP1	-0.4999003
XPNPEP2	-8.723821
XPO7	-1.1922448
XPR1	-0.810216
XRCC1	-0.6673365
XRCC2	-1.9170227
XRCC3	-1.3691919
XYLT1	-1.1010678
YBEY	-1.1549752
YIPF2	-0.5625057
YIPF3	-0.4174261
YME1L1	-0.7057734
YOD1	-1.7018895
YPEL3	-1.1488047
YPEL4	-1.9163704
YWHAB	-0.408134
ZBED4	-0.5991826
ZBTB1	-0.5125554
ZBTB3	-0.3879979
ZBTB45	-1.2929187
ZBTB46	-0.4104915
ZBTB7A	-0.6377997
ZBTB8A	-1.1185722
ZBTBD6	-2.2421324
ZC3H12B	-0.6166818
ZCCHC17	-0.3523078
ZCCHC3	-2.7094536
ZCCHC4	-0.3780875

ZDHHC1	-0.3349772
ZDHHC14	-1.317378
ZDHHC22	-1.6615362
ZDHHC24	-0.6759605
ZDHHC7	-0.556912
ZFAND2B	-0.9916501
ZFAND4	-1.402848
ZFAT	-0.458921
ZFHX2OS	-1.5384488
ZFP101	-1.2820406
ZFP108	-1.5456462
ZFP114	-1.2770967
ZFP142	-0.389025
ZFP180	-1.1953354
ZFP212	-0.3718891
ZFP235	-0.6148677
ZFP251	-0.3729115
ZFP280B	-0.4466863
ZFP286	-0.4222961
ZFP316	-0.5378008
ZFP322A	-0.725934
ZFP324	-0.7078862
ZFP335	-0.4160986
ZFP346	-0.9962454
ZFP35	-0.6682029
ZFP354B	-1.6835785
ZFP367	-1.2680058
ZFP385A	-0.7070706
ZFP385C	-1.6843681
ZFP39	-1.7052426
ZFP395	-1.0596411
ZFP41	-0.5425355
ZFP410	-0.8162298
ZFP414	-0.6209869
ZFP467	-1.0218556
ZFP473	-0.5449295
ZFP521	-1.1174569
ZFP526	-1.0145118
ZFP599	-0.3495915
ZFP62	-0.3635387
ZFP664	-0.4857431
ZFP672	-0.7608104
ZFP688	-0.5290675
ZFP704	-1.2261999
ZFP710	-0.6357179

ZFP758	-0.4178026
ZFP760	-1.5455856
ZFP763	-0.5432258
ZFP786	-0.5452118
ZFP808	-2.6507978
ZFP821	-0.3988338
ZFP831	-0.6985822
ZFP91	-0.584125
ZFP93	-0.3689044
ZFP934	-1.675015
ZFP94	-0.431231
ZFP949	-1.2901859
ZFP950	-0.5406389
ZFP955A	-0.8498254
ZFPM1	-2.4027967
ZFR	-0.4246643
ZFR2	-0.7438886
ZFYVE1	-0.7755551
ZFYVE21	-0.7918496
ZGRF1	-0.9330328
ZIC1	-0.4905446
ZKSCAN14	-0.5262413
ZKSCAN3	-0.664813
ZKSCAN4	-1.3622658
ZMAT2	-0.6825581
ZMYM1	-0.6540909
ZMYND8	-0.7502828
ZNRD1AS	-0.7300906
ZPBP2	-0.4979899
ZRANB3	-1.2627873
ZRSR1	-0.4734397
ZSCAN2	-0.8111627
ZSCAN30	-0.4952853
ZSWIM3	-0.9887958
ZW10	-0.7369008
ZWILCH	-1.293725
AARD	2.9264567
AASS	0.7194123
AATF	0.5294614
ABCA1	1.5887165
ABCA14	0.8295531
ABCA16	0.98251677
ABCA4	1.7929916
ABCA5	0.8301854
ABCB1B	1.0648994

ABCC1	0.98258305
ABCC12	0.725898
ABHD11OS	0.5673828
ABHD12B	0.5779648
ABHD17B	0.7068577
ABHD17C	0.514915
ABHD3	1.0426486
ABL2	0.9304495
ABTB2	1.6409831
ACAA1A	0.4159875
ACACB	0.6384473
ACADSB	0.47782993
ACBD3	0.7425499
ACER2	2.1663282
ACKR3	0.7997894
ACOT10	0.6096859
ACOT7	0.37121296
ACOT9	0.5415015
ACOX2	1.6615093
ACOXL	0.44751954
ACP2	0.50127316
ACP5	1.1702256
ACPP	1.4882972
ACR	0.79655266
ACSL4	1.0874901
ACTR3	0.5899701
ACTRT2	1.3357868
ACVR1	0.65257883
ACVR1B	1.3582897
ACY1	0.55962944
ADAM15	0.48667717
ADAM3	1.9029357
ADAM32	1.4926698
ADAM9	2.0938568
ADAMTS1	3.169723
ADAMTS14	0.6954889
ADAMTS15	3.3355103
ADAMTS4	5.7920594
ADAMTS5	0.8267715
ADAMTS7	1.3835282
ADAMTS9	2.7924032
ADAMTSL4	0.76010466
ADAR	0.3410735
ADARB1	0.98384094
ADARB2	0.98096514

ADC	0.6313219
ADCK4	0.43607473
ADCY4	0.36306715
ADH7	1.3214028
ADHFE1	0.77522564
ADM	4.2551875
ADM2	0.74992275
ADORA1	1.4444842
ADORA2B	1.1381052
ADPRH	0.54336214
ADPRM	0.89830494
ADRA2A	0.60344887
ADSS	0.39845705
AEBP2	0.9884367
AEN	0.8793168
AFM	1.1848011
AFP	0.98936176
AGFG1	0.32905674
AGPAT9	0.49674606
AGT	0.75947857
AGTR1A	0.56865406
AGTRAP	0.5735998
AHCY	0.56021595
AHCYL2	0.32887554
AHSG	3.6212592
AIDA	0.36942768
AIM1	0.74422073
AIM1L	0.77044225
AIMP2	0.3467331
AK4	3.5241551
AK7	2.0738995
AKAP12	2.1362176
AKAP2	1.8141141
AKIRIN1	0.53135204
AKP3	0.87751985
AKR1B8	0.39211893
AKR1C12	0.36020637
AKR1C20	1.0899239
AKT3	0.5320225
ALAS1	0.57023525
ALB	2.4617934
ALDH1A2	4.194724
ALDH1B1	0.81415653
ALDH5A1	0.6446097
ALDH6A1	0.71353674

ALDOA	0.9635701
ALDOB	3.3195615
ALG1	0.72470856
ALG11	1.1745868
ALKBH3	0.53144217
ALKBH6	1.0659518
ALPK1	1.2664196
ALPL	2.2772493
ALS2CL	0.5727639
AMBP	3.3548503
AMH	1.2005613
AMIGO2	0.91863704
AMN	1.4306831
AMN1	0.5801401
AMOTL1	1.1235807
AMY1	9.385494
AMY2A5	10.997114
ANAPC16	0.33147478
ANG	4.3402843
ANG2	3.9564626
ANG3	4.7235494
ANG4	5.251401
ANGEL1	0.6513996
ANGPT2	2.6205568
ANGPTL4	0.63204145
ANKFN1	1.2709723
ANKIB1	0.89825964
ANKMY1	1.8430426
ANKRD33B	1.4446182
ANKRD66	0.87442565
ANO5	0.9485295
ANXA2	1.0368533
ANXA4	1.4586077
ANXA8	1.1158619
AOAH	1.6483393
AP1G1	0.68075943
AP4E1	1.1786575
APBA3	0.4619999
APBB3	0.52156305
APCS	2.9149477
APOB	1.5916584
APOBEC1	0.688457
APOC2	1.6622367
APOD	0.41078877
APOH	3.2312045

APOL6	1.0591233
APOL7A	0.57397676
APOLD1	2.6703272
APP	0.8950167
APPL1	0.598608
APRT	0.49402046
AQP12	3.7728152
AQP3	2.0276594
AQP4	0.44796968
AQP8	2.5255394
AQR	0.5912299
ARAP2	1.9290438
AREG	3.1912873
ARF1	0.5491624
ARF3	0.75977564
ARF4	0.65200615
ARFGEF1	0.42312622
ARFIP1	0.5281217
ARG2	2.7591858
ARHGAP22	1.1301012
ARHGAP24	1.0045803
ARHGAP26	0.5695958
ARHGAP29	0.4431827
ARHGAP30	0.6590848
ARHGAP31	0.9712825
ARHGAP8	1.6245646
ARHGDIG	1.239001
ARHGEF11	0.65872145
ARHGEF15	0.63238764
ARHGEF16	1.563714
ARHGEF28	0.62197757
ARHGEF38	1.0516274
ARHGEF5	1.1376519
ARHGEF7	0.36710787
ARID5A	2.1725593
ARID5B	1.1777406
ARL1	0.732739
ARL14EP	1.0281749
ARL14EPL	2.4782708
ARL15	0.5450444
ARL5B	0.6246228
ARMCX2	0.9537339
ARMCX3	0.8440528
ARMCX5	0.53113604
ARMCX6	1.1605301

ARNTL	0.393219
ARRDC4	1.2887993
ARSJ	2.0639658
ARV1	0.7105546
ASAH1	0.44084167
ASAP3	0.8913686
ASB10	0.70309496
ASB11	1.0361807
ASB13	1.0125322
ASB4	3.2486506
ASB5	1.6252294
ASIC2	2.223191
ASIC3	0.7702987
ASIC5	1.4386771
ASPDH	0.7288413
ASPG	0.8509884
ASPHD1	0.9439268
ASPRV1	1.8270445
ASTL	0.9680686
ATAD1	0.48928404
ATAD3A	1.1115007
ATF3	2.5969114
ATF4	0.79725313
ATF5	0.47227144
ATF6	0.98618746
ATG10	0.49633217
ATG5	0.42150927
ATG9B	0.52306175
ATM	0.6132984
ATP10A	0.6643219
ATP10D	0.36114073
ATP11A	0.71735525
ATP13A1	0.4001708
ATP1B3	1.0374618
ATP2C2	1.5306277
ATP4B	1.360502
ATP6V0C	0.3572464
ATP6V1A	0.42255783
ATP6V1F	0.72238064
ATP6V1G3	0.728189
ATP8B1	1.2061131
ATP9B	0.48543406
ATRNL1	0.9863219
ATXN10	0.7200446
ATXN7	0.6733537

AUH	0.45092964
AURKB	1.1772237
AVL9	1.0893917
AVPR1A	3.5561495
B3GALNT1	1.546463
B3GALT5	0.79420424
B3GALT6	0.5761099
B3GNT2	1.2689023
B3GNT7	2.012771
B430105A11	2.0837343
B4GALT5	1.240684
BAAT	0.75579643
BAIAP2L1	1.2196784
BANF2	0.32359695
BASP1	1.8538575
BATF	2.4558487
BATF2	1.7464948
BBC3	0.61297226
BBX	1.198627
BCAP29	0.47294092
BCAR3	0.944087
BCAT1	2.3834772
BCDIN3D	0.39302206
BCKDHB	1.888535
BCL10	0.57311296
BCL2	0.7351556
BCL2A1C	2.6534505
BCL2A1D	2.7121758
BCL2L10	1.0248251
BCL2L11	1.0366507
BCL2L14	2.079608
BCL2L15	1.6842651
BCL3	1.9547296
BCL6	0.5312166
BCL6B	1.2038977
BEND3	1.3408699
BEX1	1.4506593
BEX2	3.5160546
BHLHE40	1.8498282
BHMT2	0.89137816
BIRC3	1.9350433
BLOC1S4	0.37110567
BMI1	1.0179238
BMP7	1.6369331
BMPER	2.439581

BMPR2	0.9772258
BNIP3	2.2024431
BOC	1.118988
BOLA2	0.56046104
BOP1	0.7415924
BPI	0.99227214
BPIFB6	1.0398657
BPIFB9B	0.9336705
BPNT1	0.7024293
BRI3	0.8564544
BRIX1	0.43493748
BROX	0.30169153
BST1	1.9925318
BST2	2.0009046
BTBD17	0.73841906
BTBD19	0.73154306
BTBD3	1.8728399
BTBD9	0.5004349
BTG1	0.7351656
BTG3	0.86337614
BTLA	0.51114035
BTN1A1	0.448411
BZW1	0.32966042
BZW2	0.63445234
C1GALT1C1	0.31326342
C1QA	0.7156954
C1QB	0.51039505
C1QBP	0.62394047
C1QTNF1	0.8152995
C1RA	1.357552
C1RB	1.0834737
C1S1	1.2092171
C1S2	1.2778034
C2	1.2307281
C3	0.7633176
C4B	0.41171455
C4BP	1.7458436
C5AR1	0.54530144
CABLES1	0.76251936
CABP2	5.800209
CABYR	1.7429144
CACNA1A	0.8074529
CACNA1B	1.307684
CACNA1C	0.94790316
CACNA1D	0.9402847

CACNA1H	0.32998633
CACNA1S	1.0493393
CACNA2D1	0.86897564
CACNB2	1.0046697
CACNB3	2.1759996
CACNG2	1.3872757
CACNG8	0.75200295
CAD	0.41327286
CADPS2	0.44682693
CALCA	3.2682657
CALCOCO2	0.5393436
CALCR	4.3303757
CALCRL	0.5377121
CALM1	0.381495
CALR3	0.69754505
CALU	0.5211587
CAMK2B	1.8064687
CAMK2D	0.40527678
CAMK2N2	1.142273
CAMK4	2.0663898
CAMKK1	1.1214409
CAMTA1	1.5414016
CAMTA2	0.56477785
CAND1	0.32971048
CANX	0.6291013
CAPN2	0.5463109
CAPZA2	0.7067938
CAR12	2.4170537
CAR13	2.436976
CAR9	0.9210212
CARNS1	0.6770377
CASC1	2.6309507
CASKIN1	0.75118494
CASP1	0.58240604
CASP4	1.7760048
CASP7	0.5146618
CASZ1	0.8529875
CATSPER2	0.6227071
CBFB	0.7970872
CBLB	1.2152958
CBLC	1.3007636
CBS	2.650735
CBX4	0.44831514
CCBE1	1.4670687
CCBL2	1.9858742

CCDC11	2.580039
CCDC114	0.5574546
CCDC116	1.7179646
CCDC122	0.9040735
CCDC160	0.8826034
CCDC162	0.96893096
CCDC169	1.4557214
CCDC172	0.46672153
CCDC42OS	1.6104789
CCDC59	0.55708647
CCDC64B	1.3911855
CCDC65	0.8876014
CCDC79	0.41570592
CCDC81	5.8092537
CCDC85C	0.9450593
CCDC86	0.7299061
CCDC89	0.5702596
CCK	1.5618174
CCKAR	3.4742217
CCL11	3.6174178
CCL12	4.6193776
CCL17	4.744754
CCL19	0.9184599
CCL2	5.3522506
CCL22	2.1135173
CCL3	4.9520144
CCL4	4.180287
CCL5	1.5208344
CCL7	4.4948425
CCM2L	1.2065363
CCND2	1.0789857
CCNJL	1.8343105
CCNYL1	0.915535
CCR5	0.91781616
CCR7	3.0074916
CCR8	1.3972266
CCR9	0.3210094
CCRL2	2.5199962
CCSER2	0.43941402
CCT3	0.5991063
CD14	4.1083345
CD163L1	0.62565184
CD164	0.7201443
CD200	1.2581453
CD274	3.0218487

CD28	0.49586773
CD300LF	1.8059406
CD320	0.6665063
CD33	0.80995417
CD34	1.1506357
CD38	2.095446
CD40	1.6711459
CD40LG	1.0035646
CD44	1.0038462
CD46	0.7283685
CD55	0.9799566
CD6	0.6412158
CD63	0.9781213
CD69	3.2312326
CD70	3.776894
CD83	1.435224
CD86	1.0021572
CD93	0.77784824
CDC42BPB	0.5650773
CDC42EP2	1.0740771
CDC42SE2	0.45572424
CDH1	1.69382
CDH12	0.45103168
CDH17	1.3356073
CDH5	1.6447005
CDH6	2.984588
CDK18	1.8368111
CDK5R1	2.0885406
CDKAL1	0.34369755
CDKL3	0.79685473
CDKN1A	1.7596741
CDKN2B	2.2646983
CDO1	0.668828
CDR2L	0.9831872
CDV3	0.3900981
CEACAM12	2.2336411
CEACAM-PS1	0.8560505
CEBPB	2.1257248
CEBPD	1.6457729
CEL	9.560032
CELA1	5.2530065
CELA2A	10.826336
CELA3B	11.406601
CELF2	0.52863026
CELSR1	0.43844223

CELSR3	1.3609219
CENPB	0.67265224
CEP170B	0.53135014
CEP85L	1.2083087
CERS3	2.2715225
CERS6	1.0435565
CES1C	1.7968163
CES1G	1.2527792
CES2A	0.861228
CES2C	0.48026133
CFB	2.6256056
CFH	0.6654563
CFL2	0.5871682
CFLAR	1.7871037
CGA	1.1637156
CGREF1	0.6968701
CH25H	1.2295108
CHAC1	2.607943
CHCHD4	0.85791016
CHD4	0.44427776
CHIC1	0.6859288
CHIL1	0.53530455
CHIL5	2.2169094
CHIL6	2.8021464
CHL1	1.5701149
CHMP2B	0.33817816
CHMP4C	0.8718047
CHPF	0.8174896
CHRM3	2.1929777
CHST11	1.2347941
CHST14	0.47459555
CIART	2.2294862
CIPC	0.75671816
CIRBP	0.8955312
CIRH1A	0.6938901
CLCN1	0.7020526
CLCN7	0.4376278
CLDN1	2.9184113
CLDN10	0.907249
CLDN11	1.0183029
CLDN12	1.3128061
CLDN26	1.0756013
CLDN3	5.3126025
CLDN4	0.6790161
CLDN7	2.005997

CLEC12B	0.5427451
CLEC2F	1.1946969
CLEC2G	1.1475754
CLEC4A1	1.2998123
CLEC4D	1.8068714
CLEC4E	3.1510167
CLEC4N	0.8770766
CLIC4	0.6773634
CLMP	1.710834
CLN3	0.65311193
CLN5	0.58532953
CLPS	8.755043
CLSTN2	1.138875
CLTB	0.7390547
CLU	0.70687056
CLVS1	0.4215417
CMTM6	0.74996376
CMTR1	0.66575146
CMTR2	0.83079576
CNBD2	0.5540416
CNBP	0.41194773
CNDP2	1.1383991
CNGA4	1.2430167
CNIH2	0.42628884
CNIH3	1.4510176
CNKSR3	2.717967
CNN3	1.232193
CNOT6L	0.44270134
CNTNAP3	1.3462152
COBLL1	0.7768531
COG5	0.58583546
COL22A1	3.5265324
COL4A1	1.8997579
COL4A2	1.5939484
COL7A1	1.0611687
COPS7A	0.62704897
CORO2A	0.4356575
COX15	0.43244076
COX18	0.58103704
COX8C	1.0062835
CP	3.2763343
CPA1	10.285727
CPA2	9.823605
CPA5	0.62275815
CPB1	12.680012

CPEB2	1.192069
CPHX1	0.57673025
CPLX1	0.67799044
CPNE1	0.73433065
CPNE8	1.218884
CPS1	2.2014787
CPSF4L	1.1385884
CRB3	1.7183006
CREB3	0.82386875
CREB3L1	0.9539285
CREB3L2	0.6433735
CREB3L3	0.50530124
CREB3L4	1.5632391
CREBL2	1.0858455
CREBRF	0.65669274
CRELD2	0.54234314
CREM	2.3412395
CRISPLD2	1.8063874
CRLF2	0.72201633
CRNN	1.6462839
CRTAP	0.32921124
CRTC2	0.8520441
CRY2	0.79830647
CRYAA	1.6971607
CRYBA1	1.0591877
CRYBA2	1.6333218
CRYBB2	1.1716051
CRYBB3	2.161953
CSF1	0.64739084
CSF2	3.3582351
CSF2RB	1.3804584
CSF2RB2	1.4450054
CSF3	7.7814903
CSMD2	1.0818691
CSNK1E	0.4096694
CSPRS	0.6565933
CSRNP1	2.093122
CST10	1.3165917
CST7	1.1149683
CSTB	1.3973742
CSTF2T	0.54972696
CTGF	1.6563153
CTH	2.1309936
CTLA2A	1.7701626
CTLA2B	1.4669662

CTLA4	3.3691936
CTNND1	1.1803946
CTPS	0.85453224
CTRB1	10.186724
CTRC	5.447194
CTRL	7.10072
CTSC	0.91482973
CTSK	0.41522646
CTSL	1.237329
CTSS	0.48602057
CTSZ	1.0448008
CTTNBP2NL	1.9781923
CTXN2	0.94338703
CUBN	0.82473207
CUL9	0.43715
CUZD1	9.773542
CWC27	0.70098877
CWF19L2	0.64515543
CX3CL1	3.5762076
CXCL1	7.2812123
CXCL10	3.2095752
CXCL12	1.516212
CXCL13	3.2444267
CXCL14	2.764743
CXCL16	0.9963064
CXCL2	7.023438
CXCL3	7.1417766
CXCL5	4.157993
CXCL9	2.7529125
CXCR5	1.0957842
CYB561	1.9130921
CYB5B	0.5359216
CYB5R2	1.85516
CYBA	0.41216755
CYBB	1.2095761
CYFIP1	1.3997841
CYGB	0.71913576
CYLC2	1.5472529
CYLD	0.44461012
CYP1B1	1.9577646
CYP20A1	0.50376177
CYP26B1	2.6210928
CYP27B1	1.8931456
CYP2C67	1.7569497
CYP2D10	1.1496377

CYP2D26	1.6577127
CYP2J5	1.1252267
CYP3A11	0.42764854
CYP3A13	0.6074014
CYP3A16	1.1567461
CYP4A12B	0.64913845
CYP4F15	1.9604874
CYP7B1	2.461701
CYR61	0.90734863
CYTH1	0.61292887
CYTH4	0.6415992
CYTIP	1.999846
CYYR1	2.3409956
DAB1	0.5772412
DAB2	0.71238256
DACT2	1.4593956
DAF2	0.9524827
DANCR	1.0373325
DAPK3	0.76553345
DAPP1	0.65294075
DAXX	1.3089752
DBN1	1.5509644
DBX2	2.2434204
DCAF13	0.50918674
DCBLD1	0.7821226
DCN	1.0821705
DCPP1	1.076649
DCST1	1.3545425
DCTD	0.5019989
DCUN1D3	0.7855289
DDAH1	1.2783802
DDIT3	0.44036865
DDIT4	1.1502337
DDO	0.5198915
DDR2	0.38516998
DDT	0.6763177
DDX10	0.74027824
DDX18	0.56166935
DDX19B	0.7861767
DDX21	1.1932054
DDX24	0.57765675
DDX31	0.8508301
DDX3X	0.5035815
DDX49	0.46014166
DDX51	0.4929409

DDX56	0.41673326
DDX58	1.1034617
DEFA2	1.4317117
DEFA3	1.1124125
DEFA-PS1	0.41543078
DEFA-RS10	1.1889391
DEFA-RS12	1.642442
DEFA-RS2	1.879364
DEFA-RS4	1.9576693
DEFA-RS7	1.8543649
DEFB1	1.4551728
DEFB14	2.0575817
DEFB22	1.141861
DEFB28	0.46968222
DEFB33	0.92429066
DEFB36	1.6426618
DEFB41	1.3082762
DEGS1	0.5786276
DEGS2	0.8070648
DENND1B	0.48397684
DENND2D	0.47717762
DENND3	0.48757744
DENND5A	0.463789
DENR	0.69038725
DGAT1	0.775332
DGAT2	0.8330445
DGKE	0.4856298
DGKH	1.0191164
DGKK	2.6963034
DHODH	0.3301406
DHPS	0.45102406
DHX30	0.33826637
DHX33	0.86253643
DHX35	0.6901202
DHX40	0.7596202
DHX58	1.3566475
DIAP2	1.1668897
DIEXF	0.7426598
DIMT1	0.58772135
DIP2A	0.6872983
DIP2C	0.8370633
DIRAS1	1.6999352
DIS3	0.5315113
DIXDC1	2.2492173
DKK2	1.3382149

DLC1	0.40417862
DLG5	0.6651416
DLGAP3	1.4608204
DLK1	0.99884677
DLL4	2.5752678
DMBT1	13.069374
DMKN	1.0467386
DMRT2	1.5887904
DMRTA1	1.17151
DMXL2	0.9070358
DNAH1	0.98952746
DNAH2	2.4272394
DNAJB5	0.66640997
DNAJB9	0.5479932
DNAJC12	0.99073935
DNAJC13	0.5208068
DNAJC14	0.7436495
DNAJC22	1.8198576
DNASE1L3	0.8925824
DNHD1	0.67687416
DNM3OS	1.9911344
DNMT3L	1.2051635
DNTTIP2	0.39186668
DOCK10	0.35584688
DOCK4	0.8459258
DOCK7	1.1053615
DOCK9	1.2916584
DPH2	0.86198664
DPH5	0.62756824
DPH6	0.74495935
DPY19L3	0.67959785
DPYS	0.9881394
DPYSL3	0.5226984
DPYSL5	0.63227177
DRAM1	1.1218309
DRAM2	0.66465664
DRAP1	0.365417
DRD4	2.3959012
DSEL	0.5771885
DSG1B	1.1134715
DSG2	0.7194557
DSG4	0.9757204
DSP	1.3002715
DST	1.2459159
DTX1	0.67412424

DTX2	1.5215683
DTX3L	0.8885498
DTX4	0.47303772
DUPD1	1.331847
DUS3L	0.59936666
DUS4L	0.61509657
DUSP1	3.0350256
DUSP12	1.2522855
DUSP14	0.70446897
DUSP16	2.5741959
DUSP2	1.7044291
DUSP23	0.7081809
DUSP26	1.9837017
DUSP4	1.3224659
DUSP5	1.4924719
DUXBL1	1.3133361
DYNC1I1	1.347681
DYRK2	0.9069729
EAF2	1.0526481
EAPP	0.40925598
EBI3	1.1673627
EBNA1BP2	0.7678633
ECE2	1.1985536
ECHDC2	0.89834046
ECHS1	0.36285305
ECSCR	0.98570204
EDEM2	0.5493188
EDN1	4.6177106
EDNRA	1.1334538
EDNRB	0.8620024
EEA1	0.8741045
EED	0.45116186
EEF1E1	0.4081049
EEPD1	0.6481843
EFCAB2	0.44654274
EFCAB8	1.1832855
EFHB	0.7947626
EFHD2	0.933331
EFNA1	0.8041768
EFNA5	0.98385835
EFNB2	0.3624158
EGF	1.469341
EGFBP2	6.296693
EGFLAM	1.1611195
EGFR	2.034159

EGLN2	1.339364
EGLN3	1.7308121
EGR1	1.4225874
EGR2	0.75287104
EHD1	1.9279065
EHF	1.0008414
EHMT1	0.8045025
EID2	1.1198502
EIF1A	1.274322
EIF2AK2	0.5350065
EIF2AK4	0.6255646
EIF2S1	0.35702658
EIF2S3Y	1.462949
EIF4A2	0.5115404
EIF4EBP1	0.54106426
ELAC2	0.47771835
ELAVL4	0.48691225
ELF3	2.0315185
ELL	0.38064575
ELMO1	0.9285054
ELOVL4	3.0620239
ELOVL5	0.52794695
ELP5	0.3676777
EMB	1.1124082
EMC1	0.3599701
EMC4	0.6575036
EMC6	0.54086447
EMCN	0.37127018
EML1	1.297246
EMR1	0.45678568
ENAH	0.65090966
ENC1	2.1021223
ENDOG	0.3005023
ENO2	2.2393708
ENPP2	1.1760125
ENTPD1	0.7664082
ENTPD3	2.1269054
EPAS1	1.0114999
EPB4.1L4B	1.139575
EPB4.1L5	0.54660845
EPCAM	1.5684841
EPHA2	0.64326954
EPHA4	1.0650833
EPHA5	0.43673468
EPHA7	2.0375042

EPHB2	0.57713795
EPHB3	0.63840437
EPN2	1.067092
EPPK1	2.7178235
EPRS	1.1531067
EPSTI1	0.5546894
EQTN	1.7075677
ERAP1	0.4587102
ERAS	0.9390044
ERBB2IP	0.40000343
ERCC1	0.4766326
ERCC2	0.37275696
ERDR1	0.5083561
EREG	3.4224472
ERGIC1	1.0480046
ERICH2	1.2390783
ERLIN1	0.37034464
ERLIN2	0.41198015
ERP27	1.3188405
ERP44	0.44391203
ERRFI1	4.3135705
ESRP1	1.444505
ESRP2	4.386984
ESRRB	0.46876597
ETNK2	1.1600811
ETS2	1.3256054
ETV3	1.5154271
ETV6	1.3176885
EVA1B	0.813189
EVA1C	2.1780539
EXOC1	0.46902227
EXOC2	0.47409868
EXOC3L4	0.55542135
EXOC6B	0.7431202
EXTL1	0.32877994
EXTL2	0.5708399
EYA1	0.53562593
F10	1.4702606
F11R	1.6147571
F12	1.2138221
F2	1.9110074
F2R	0.5312872
F2RL3	0.85084605
F3	0.98152924
F7	0.9847896

FABP3	1.1780779
FABP4	0.6276221
FABP5	1.1277604
FAM107A	0.31633067
FAM107B	0.7793474
FAM114A1	1.0094199
FAM115E	1.2477546
FAM129B	2.5668097
FAM135A	0.93464684
FAM136A	0.63286877
FAM13C	1.4124618
FAM150B	2.7984595
FAM154A	0.51177216
FAM155A	3.2517617
FAM160A1	0.7706814
FAM163A	1.2765367
FAM163B	1.2378492
FAM169B	0.35366917
FAM174A	0.5674343
FAM181B	0.55984306
FAM184B	1.0505311
FAM185A	0.5318966
FAM187B	0.7684002
FAM188A	0.83316374
FAM188B	0.5576744
FAM19A3	0.8032658
FAM203A	0.5266309
FAM206A	0.6474285
FAM208B	0.7617984
FAM20C	1.2489457
FAM21	0.37636995
FAM219A	0.49067545
FAM222B	0.4343996
FAM26E	1.2845256
FAM26F	1.5655456
FAM3C	0.70602465
FAM46A	0.82935286
FAM46B	0.9433334
FAM60A	1.131691
FAM71F2	2.5933905
FAM73A	0.46722746
FAM78B	2.6617553
FAM83A	0.5830679
FAM83H	0.88766646
FAM84B	0.84183407

FARP1	0.6204705
FAS	1.6610918
FASL	0.5741706
FASTKD1	0.7121978
FASTKD2	1.3670435
FASTKD3	0.6283293
FAT1	0.36416817
FBL	0.56243324
FBLIM1	1.4009681
FBP1	1.0029783
FBP2	1.2664163
FBXL12	0.8662572
FBXL5	1.1922932
FBXL7	0.8187778
FBXO17	0.8318949
FBXO22	0.5447326
FBXO4	0.8053932
FBXO43	1.3654306
FBXO6	0.5968585
FBXW13	1.6411114
FBXW18	1.373178
FCER1G	0.86610985
FCGR2B	0.8970938
FCGR4	2.0701966
FCHSD2	0.42676306
FCRL1	1.0960054
FCRL5	1.0337644
FDX1	0.7889919
FER1L6	0.98924136
FERD3L	0.8186393
FERMT2	0.9281559
FETUB	1.4042969
FEZ2	0.48746443
FFAR2	0.76566076
FGA	3.5082033
FGB	5.4701514
FGF21	1.6749434
FGF7	1.4780598
FGFBP3	0.4020841
FGFR1	0.3340187
FGG	6.757786
FGGY	0.5597613
FGL1	2.3869548
FGL2	0.95793486
FHL3	0.4333818

FILIP1L	0.5672064
FITM2	0.37710667
FKBP14	0.56484866
FKBP5	2.0053754
FLNB	1.6500988
FLOT1	0.6653094
FLRT3	1.1276848
FLT3	0.9881029
FLT4	0.77403903
FMO1	0.9901633
FMO3	1.7805576
FMO9	0.91892815
FNBP1L	0.8940978
FNBP4	0.49830246
FNDC3B	1.5179267
FNDC7	1.3138843
FOLH1	3.3014297
FOS	3.824273
FOSB	1.4070814
FOSL1	3.1402082
FOSL2	2.3892365
FOXA2	1.9790826
FOXA3	2.1375751
FOXC1	1.757549
FOXC2	1.6965685
FOXF1	1.560463
FOXI3	1.0867698
FOXK1	0.55549955
FOXN2	0.50827265
FOXP1	0.56917953
FOXP3	1.1868682
FOXRED2	0.694191
FPR1	1.57799
FPR2	2.2657938
FPR-RS6	2.0645394
FRMD5	0.7324214
FRZB	0.7167764
FSCN1	2.8714657
FSCN2	1.0268381
FST	2.4858086
FSTL1	1.1350503
FSTL3	0.47012472
FURIN	0.4089594
FUT1	1.8287225
FUT2	2.882225

FXYD3	1.2353501
FYB	0.66055393
FYN	0.7927189
FYTTD1	1.2779558
FZD1	1.0162339
FZD3	0.88710046
FZD4	1.4966092
FZD7	0.7066221
FZD9	1.8741398
G0S2	0.86960125
GAA	0.7240486
GAB2	0.6153219
GABRP	1.5725343
GABRR2	0.82071924
GADD45B	2.3206186
GADD45G	1.6221538
GAK	0.3910179
GALK1	0.8176198
GALNT15	1.0245099
GALNT18	0.9278469
GALNT4	0.33763456
GAPDHS	0.52292347
GAREML	0.8924844
GARS	0.8832693
GAS7	1.6904373
GAS8	0.6061964
GATA2	0.4581952
GATA4	0.93443584
GATSL2	0.9025364
GBE1	0.92195606
GBP10	1.3849039
GBP2	1.6968875
GBP3	1.4084315
GBP4	2.2549949
GBP5	2.7128348
GBP6	1.6785626
GBP9	1.3822794
GC	3.07104
GCAT	1.49013
GCG	2.859325
GCK	1.5489299
GCNT2	0.6729183
GCNT7	0.87973595
GCSH	0.622921
GDAP10	2.2485042

GDAP2	0.4236927
GDAP5	1.4191911
GDF15	1.9443045
GDNF	0.96985364
GEM	2.7299523
GEMIN5	0.38575935
GFOD1	1.3047986
GFPT1	0.77098465
GFPT2	1.2778909
GFRA3	1.0426044
GFRA4	0.49658918
GGCT	0.71323776
GGH	0.9326701
GGT1	2.9201682
GGT6	1.7156162
GGTA1	0.5908785
GHITM	0.38584995
GIMAP1	0.7715044
GIMAP1OS	1.2983878
GIMAP3	1.2158384
GIMAP4	0.84839773
GIMAP5	1.548738
GIMAP6	1.0555639
GIMAP8	1.0803542
GIMAP9	0.565979
GIPC3	1.3296659
GIPR	0.93414974
GIT1	0.45282412
GJA10	1.3742836
GJA4	1.676652
GJB1	1.3997741
GJB2	3.2158442
GJB4	0.7253442
GLI1	0.78261185
GLIPR2	1.049458
GLS	0.6804104
GLS2	1.0812941
GLT1D1	0.5636623
GLT28D2	1.0889208
GLYATL3	0.7978339
GM10015	0.37429237
GM10021	1.2749743
GM10069	0.67078614
GM10099	2.0157652
GM101	1.2300122

GM10113	0.6464188
GM10190	0.9881582
GM10277	0.89741135
GM10278	0.97081375
GM10334	12.374493
GM10353	0.54945755
GM10385	1.5370984
GM10387	2.1415384
GM10388	1.8234904
GM10403	1.3077002
GM10405	0.59044456
GM10406	1.2028599
GM10421	1.3765569
GM10445	1.5232685
GM10461	0.700428
GM10510	0.4864893
GM10537	1.1826899
GM10589	1.045362
GM10631	0.5493512
GM10632	0.43350267
GM10634	1.6826997
GM10640	0.82332087
GM10693	0.56354475
GM10696	2.1671772
GM10732	1.8974133
GM10773	1.6675999
GM10781	0.94222593
GM10791	1.8538527
GM10815	1.1001604
GM10832	1.8506494
GM10836	0.78247595
GM10839	5.0635614
GM10845	0.50239277
GM10847	0.55286
GM10851	2.274777
GM10864	0.9972553
GM10867	0.7962816
GM10914	0.6469908
GM11381	1.3707416
GM11492	0.8706653
GM11517	2.0720975
GM11974	1.651628
GM11992	2.521618
GM12060	0.42793655
GM12216	0.66605425

GM12250	1.1145544
GM12253	0.8145876
GM12302	0.806371
GM12689	0.83772445
GM12693	0.69640684
GM12703	0.6370678
GM12758	0.5191057
GM12789	0.99936247
GM13011	11.459326
GM13152	0.5466163
GM13205	1.453573
GM13212	0.37098074
GM13238	0.6407976
GM13262	0.78300667
GM13298	1.718945
GM13315	1.1177068
GM13498	0.7751274
GM13546	1.8057115
GM136	0.7434664
GM13629	0.8937397
GM13889	3.482782
GM14005	1.4566288
GM14321	1.0900416
GM14347	0.5748346
GM14374	1.2626941
GM14499	1.26423
GM14548	0.5808263
GM14858	0.4220233
GM15056	4.579143
GM15340	1.5888638
GM15498	1.2789571
GM15520	1.1508477
GM15762	1.5002437
GM16223	0.76722693
GM16287	1.3534591
GM16340	0.79455614
GM16486	1.8424978
GM16498	1.5943115
GM16525	1.1900115
GM17769	0.7032857
GM19402	1.9597445
GM1966	0.55428505
GM2022	0.89792633
GM2030	0.4499681
GM20558	2.6652586

GM20754	0.80900836
GM20758	0.87854505
GM2090	1.1194444
GM2164	1.1525755
GM21944	4.6328897
GM21951	0.7999041
GM22	0.51091194
GM2511	1.4264929
GM2545	1.2841136
GM2696	0.5847268
GM2814	1.2034576
GM2837	0.8529341
GM2927	1.011095
GM3052	1.1105351
GM3099	0.8939278
GM3161	0.98006725
GM3239	1.557733
GM3252	1.0941195
GM3317	0.85753155
GM3367	3.5873058
GM3383	1.217711
GM3453	1.217948
GM3455	0.5765526
GM355	0.893909
GM3696	2.1969016
GM3740	0.9730923
GM3764	0.7415049
GM382	0.44408703
GM3881	1.3792777
GM3893	2.1006064
GM3985	1.258681
GM4107	0.6621709
GM4146	1.212935
GM4203	1.70677
GM4319	1.0148692
GM4371	1.0990696
GM4402	0.7330384
GM4419	0.33873892
GM4432	2.2633631
GM4461	0.5939162
GM4466	1.3482819
GM4559	0.5856459
GM4755	0.8199134
GM4776	1.2022326
GM4788	1.6738667

GM4791	1.0466223
GM4841	1.1466966
GM4862	1.3735671
GM4868	0.7275467
GM4907	0.8001838
GM4951	1.6298885
GM4983	1.0897613
GM4994	2.1881256
GM5067	6.386801
GM5069	0.58238935
GM5122	1.196775
GM5124	0.5677588
GM5129	1.1752799
GM5150	0.513175
GM5168	0.70771337
GM527	0.47340012
GM5409	10.729021
GM5416	1.8321238
GM5420	1.3237753
GM5431	0.8757341
GM5441	0.4701755
GM5458	1.303298
GM5476	1.2586184
GM5483	2.724154
GM5486	0.7041905
GM553	1.3043056
GM5533	0.30167818
GM5549	0.93016195
GM5602	0.8343196
GM5771	13.04335
GM5795	1.1808007
GM5796	0.80710816
GM5797	0.9823413
GM5862	0.7491152
GM590	1.9546316
GM5908	0.8332653
GM5932	0.86072755
GM597	1.5391693
GM614	1.2738585
GM6145	1.350555
GM6211	0.6227231
GM6252	0.825696
GM626	0.61516476
GM6280	0.4212048
GM6360	1.1385345

GM6370	0.9005816
GM6377	2.4788551
GM6402	0.87933016
GM6403	3.1398408
GM6460	1.0133755
GM6498	0.6367302
GM6524	0.39192486
GM6537	0.80433536
GM6579	0.79963255
GM6583	0.7662811
GM6602	1.681962
GM6658	0.8186023
GM6744	1.2587638
GM6756	0.9134064
GM6818	1.4816253
GM6846	2.072617
GM6904	1.2672575
GM6927	1.4137833
GM6943	0.97592926
GM6981	0.4471779
GM7133	1.5478683
GM7134	1.3919556
GM7157	1.0451496
GM7160	1.3965459
GM7609	0.607636
GM7694	0.86139894
GM7827	0.73307514
GM7967	0.4645934
GM8075	1.6911187
GM8096	1.0482163
GM8350	0.7180886
GM8369	0.8803563
GM8672	0.9387772
GM8709	0.46574163
GM8884	2.0351477
GM8909	1.2737536
GM8995	1.061655
GM9199	0.52417254
GM9258	0.486053
GM9693	0.96079826
GM9706	1.4999223
GM9757	0.73328686
GM9777	0.93995786
GM9802	1.0705543
GM9860	1.5735917

GM9866	1.19028
GM9873	0.8223264
GM9910	0.83937216
GM9917	0.54890394
GM9948	1.062067
GM9973	1.3890088
GMDS	0.6253214
GMPPB	0.40861416
GNA13	1.0926442
GNAI1	1.7393711
GNAI3	0.30567265
GNAL	1.5021529
GNAT2	0.49702168
GNB1	0.3838892
GNB4	0.3984499
GNG12	1.8649702
GNG5	0.55924225
GNGT1	0.91595125
GNL1	0.94753885
GNL3	0.54084873
GNMT	1.5448749
GNPNAT1	0.99935627
GNRHR	0.84485793
GNS	1.3454599
GOLGA3	0.47124767
GOLGA5	0.54935503
GOLGB1	0.5140691
GOLT1A	0.65908265
GOPC	0.7717366
GOT1	1.4673653
GP2	10.148325
GP49A	1.7968245
GPAT2	0.5530503
GPD2	0.7546153
GPHN	1.2228403
GPM6B	1.3902674
GPR110	1.516999
GPR113	1.2904408
GPR115	1.3114462
GPR116	1.5439787
GPR123	0.9901631
GPR126	1.7252259
GPR132	1.4432421
GPR137B	1.0633602
GPR137B-PS	1.0802622

GPR15	1.3367102
GPR150	0.948241
GPR158	0.73783064
GPR161	1.1121426
GPR171	1.8994584
GPR176	1.1664486
GPR179	2.4819763
GPR18	1.5681701
GPR183	1.0758638
GPR26	0.67358804
GPR31B	2.5268366
GPR33	1.1116791
GPR35	0.71314335
GPR4	0.73641753
GPR55	2.0847943
GPR63	1.1342816
GPR64	2.3982837
GPR65	1.1821575
GPR75	1.2273393
GPR84	2.4197607
GPR85	5.2755046
GPRC5B	1.4336548
GPRIN1	0.48689055
GPT2	1.6551104
GPX3	1.7755198
GRAMD1A	0.39515257
GRAMD1B	0.58582354
GRAMD3	2.079832
GRAMD4	0.5106039
GRB2	0.57078505
GRB7	1.8144662
GREB1L	1.3199167
GREM2	0.56259155
GRHL1	1.0654368
GRHL2	1.8953736
GRIN2C	1.2460747
GRK5	0.37365007
GRM6	1.4315817
GRN	0.7395339
GRPR	2.1360102
GRWD1	0.7913995
GSAP	0.43040466
GSDMA3	0.97113514
GSDMC4	2.234172
GSDMD	0.5230546

GSPT2	0.75646424
GSR	0.7476878
GSTO1	0.3628049
GSTO2	1.0121384
GSTT3	0.5189481
GTF2E1	0.5065303
GTPBP10	0.40048933
GTPBP6	0.5417571
GUCY1B2	0.70291924
GUCY2F	1.6489131
GXYLT2	0.5917144
GYK	0.87709284
GYLTL1B	0.49842358
GYS1	0.52698135
GZF1	0.53131914
GZMB	2.4133034
GZMC	2.687831
GZME	1.7682388
H13	0.5032916
H2AFY2	0.42839813
H2AFY3	0.62874174
H2-Q10	0.7278929
H2-T3	0.55262494
H60A	1.8971076
H60B	1.7700868
H6PD	0.63878155
HABP2	4.311308
HAMP	4.1350107
HAMP2	1.824158
HAS1	2.7744286
HAS2	1.5224702
HAX1	0.61836004
HBEGF	2.262313
HC	1.4255233
HCAR2	0.8779421
HCK	1.205471
HCLS1	1.152381
HCN2	0.8993573
HCN3	1.463331
HCRT	1.1011717
HDAC9	1.0563159
HDC	1.2204375
HDGFRP3	0.8621321
HEATR1	1.0508308
HECA	0.4104824

HECW1	0.5391965
HECW2	0.7292676
HEG1	1.6285226
HELZ2	1.396668
HENMT1	1.1432726
HEPACAM	0.822896
HERC3	0.8072381
HERC6	0.93270826
HERPUD1	0.576561
HES7	0.32206535
HGSNAT	0.40004587
HHAT	0.57342863
HIBADH	0.407094
HIC1	0.9717469
HID1	0.50322294
HIF1A	2.230392
HIGD1C	1.4727461
HILPDA	3.7239199
HILS1	0.92902255
HIST1H4M	0.34116936
HIVEP1	0.55306864
HIVEP2	0.9815731
HK2	2.0243134
HKDC1	3.4509513
HMGA1	0.5386925
HMGN3	0.8865504
HMGXB3	0.4745574
HMX1	1.2664044
HNF1A	2.0659552
HNF4A	1.3395717
HNRNPDL	1.2425275
HOMEZ	0.4739399
HOOK2	0.95091677
HORMAD2	0.965677
HOXA3	0.98775387
HOXA5	1.9966276
HOXA9	0.5531807
HOXB2	0.71145034
HOXB4	0.4690094
HOXB7	0.37216926
HOXC11	1.2481241
HP	1.3293743
HPDL	1.524895
HPGDS	1.8590426
HPX	5.33873

HRCT1	1.11677
HRH1	0.8564997
HS3ST1	0.97139144
HS3ST2	0.8535638
HSBP1	0.86236
HSD17B13	2.892547
HSD17B2	1.2883995
HSF2	0.77904606
HSH2D	0.50308275
HSP90AB1	0.480062
HSPA13	0.7318959
HSPA1A	3.155923
HSPA4	0.73359823
HSPA9	0.83525467
HSPB1	2.692803
HSPB7	2.1603258
HSPB8	1.7113938
HSPE1	0.8938842
HTR1B	2.4628897
HTR1F	1.1160576
HTR3A	1.0932751
HTR6	0.9020481
HTR7	3.1991062
HTRA1	1.8472009
HTRA4	4.91704
HTT	0.60341454
HYI	0.612669
IAPP	2.916943
IARS	0.4764967
ICAM1	1.8541679
ICOS	0.9107733
ID2	1.3256035
IDO2	3.3706443
IER3	4.3388844
IER5L	0.83817434
IFI202B	1.7391992
IFI204	1.5746942
IFI35	0.6814585
IFI47	1.6458483
IFIH1	1.2864256
IFIT1	1.4496231
IFIT2	1.2524767
IFIT3	1.5140676
IFITM1	1.8935485
IFITM2	1.1798205

IFITM3	0.9584813
IFITM5	1.9908531
IFITM6	0.4690795
IFITM7	0.42921305
IFLTD1	1.3727112
IFNA13	0.8074031
IFNAB	0.87327576
IFNB1	3.2991629
IFNG	4.009959
IFNGR2	0.6087408
IFRD1	1.697937
IFT122	0.42746544
IFT43	0.7465086
IFT88	0.8255844
IGBP1B	1.1421735
IGF2BP2	0.7005234
IGFBP1	6.427035
IGFBP7	0.89832354
IGH-VJ558	0.51978254
IGJ	0.5541682
IGKV4-55	1.5600166
IGSF11	1.2580156
IGSF23	2.5721772
IGSF5	2.5396228
IGSF6	1.13131
IGSF8	0.93131065
IGSF9	0.4949727
IGTP	0.6541014
IIGP1	1.5848422
IKBKAP	0.5762043
IKBKE	2.0756903
IKZF4	1.5022497
IL10	4.818664
IL11	1.1510792
IL12B	4.1975393
IL12RB1	1.1471801
IL12RB2	0.814132
IL15	0.4632604
IL15RA	1.4526
IL17A	5.5665903
IL17F	2.8095384
IL17RB	0.8611431
IL17RC	0.67410517
IL18	2.2746024
IL18BP	1.062469

IL19	2.0018456
IL1A	3.7713513
IL1B	2.7510228
IL1F6	2.4698527
IL1F9	2.6832747
IL1R1	1.0408502
IL1R2	4.3295417
IL1RN	4.766743
IL2	1.5557206
IL21R	1.1100049
IL22	4.4433956
IL23A	2.4010181
IL23R	2.523496
IL27	1.543437
IL2RA	3.4102883
IL2RB	0.6914215
IL2RG	0.9468956
IL33	1.9088702
IL4I1	1.3880053
IL4RA	0.96510315
IL6	8.340477
IL7R	1.56179
ILDR1	1.2502697
IMMP2L	0.49897647
IMP4	0.5684757
IMPDH1	1.3769374
INADL	1.2359953
INHBA	0.73502016
INHBB	4.4229445
INS1	3.61729
INS2	1.37396
INSIG2	0.33561325
INSL6	1.2054315
INSR	0.9182136
IPO4	0.9584632
IPPK	0.44065523
IQCF1	0.5159414
IQCF3	0.99037385
IQGAP1	0.35570478
IRAK3	2.3977914
IRF1	0.9047971
IRF2BP2	0.52545595
IRF4	1.0648947
IRF7	1.2473731
IRG1	4.658892

IRGM1	0.7555556
IRX3	1.7792025
ISG15	1.1900373
ISG20L2	0.43782187
ISL2	1.0827746
ISM2	1.5079432
ISY1	0.38033724
ISYNA1	0.30644083
ITGA1	0.9072714
ITGA5	1.221149
ITGA7	1.5052176
ITGA9	1.0920768
ITGAL	1.0471053
ITGAM	0.48736334
ITGAV	1.1375875
ITGB4	1.1001859
ITIH3	1.2081795
ITIH4	6.171816
ITK	1.5664563
ITLN1	0.9219165
ITLNB	0.45955443
ITPKC	1.3104424
ITPR3	0.46591663
ITPRIP	0.94517136
IWS1	0.9188287
JADE2	1.0862741
JAK1	0.49408388
JAK3	0.95639944
JAKMIP3	0.48259902
JAM2	1.1743274
JDP2	1.3760042
JUN	2.1858373
JUNB	1.6592565
KANK1	1.219728
KANSL3	0.53651094
KATNAL2	1.1275868
KBTBD11	0.71567106
KCNA4	0.7430301
KCNA6	0.4937694
KCNC3	1.1466272
KCNE3	1.3104143
KCNE4	3.2377505
KCNF1	0.56790066
KCNH7	0.8923807
KCNJ15	1.2432754

KCNJ8	1.5990858
KCNK1	3.4272122
KCNK15	0.6292093
KCNMB3	1.9655299
KCNQ1	1.8843288
KCNQ5	1.1287773
KCNT1	0.6898861
KCNT2	0.7483673
KCTD12	1.1413941
KCTD18	1.0263855
KCTD19	0.74200296
KDELR3	0.8128829
KDF1	2.6797645
KDM6B	1.413105
KDSR	0.6358609
KHNYN	0.42004395
KHSRP	0.36546707
KIF1A	2.4711006
KIF1B	1.1299787
KIF27	0.7886858
KIF2B	1.3690419
KIRREL2	0.8572097
KISS1	0.77696776
KIZ	0.47178555
KLC2	0.79502535
KLC3	0.45429873
KLF15	1.5692718
KLF6	1.2076674
KLF7	0.6705036
KLF9	1.3196507
KLHDC4	0.7861943
KLHL15	1.0725269
KLHL2	1.2863703
KLHL21	0.6948924
KLHL31	0.99307394
KLHL6	0.34236145
KLK1	5.973609
KLK12	0.7939272
KLK1B11	6.1765513
KLK1B21	1.9372005
KLK1B22	4.144648
KLK1B24	4.925167
KLK1B26	6.1779833
KLK1B27	1.2101221
KLK1B3	5.452294

KLK1B4	5.8837113
KLK1B5	6.4224553
KLK1B7-PS	0.8059833
KLK1B8	5.3756638
KLK5	0.82584095
KLK7	0.959635
KLK9	0.45755816
KLRA2	2.3170137
KLRB1-PS1	1.0793309
KLRC2	1.3468463
KLRI2	1.2147751
KNG1	4.781083
KNG2	3.458492
KPRP	1.2658608
KRI1	0.6424217
KRR1	0.4861455
KRT12	1.3847733
KRT13	0.58690953
KRT17	1.042067
KRT18	2.2646737
KRT19	0.5140214
KRT2	0.6668205
KRT23	3.5904102
KRT26	2.1894326
KRT31	0.8190458
KRT42	0.36290264
KRT72	1.0674913
KRT74	0.5597198
KRT77	0.7679632
KRT8	2.4890676
KRT85	0.77368903
KRTAP11-1	2.4606469
KRTAP1-4	0.8420868
KRTAP4-16	0.42251134
KRTAP9-3	0.97274184
KRTCAP2	0.53656054
KSR1	0.80301213
KTN1	1.3503165
L3MBTL1	0.94922113
LACC1	2.4181705
LAD1	2.6446218
LAMA1	0.8757317
LAMA4	1.1140542
LAMA5	1.9131861
LAMB1	0.50429535

LAMC1	0.8236742
LAMC2	2.28344
LAMP1	0.52737665
LAMP2	0.5991001
LANCL3	0.8183272
LAO1	0.6759794
LAP3	0.47559834
LAPTM4A	0.71428204
LAPTM4B	0.83444977
LARGE	1.0127344
LARP1	0.8020911
LARP4	1.0256252
LARP6	0.82596874
LARS	0.8702531
LAS1L	0.40211916
LATS1	0.68239546
LCE1C	0.67776823
LCE1M	1.6358817
LCN11	0.9959047
LCN2	1.7487674
LCP1	0.38221693
LDHA	0.82344437
LDHD	2.7786632
LECT2	1.0050747
LEF1	0.7771144
LEKR1	0.3857813
LENG9	0.43467808
LEPR	0.97858477
LEPRE1	0.47561908
LETM1	0.39948702
LGALS3BP	0.89455986
LGALS8	1.015872
LGI2	1.165112
LGMN	0.9211159
LGR4	2.212959
LHX1OS	0.85769844
LIF	3.0903137
LIG3	0.6296129
LILRA6	0.43505192
LILRB4	1.7657337
LIN28B	0.60985994
LIN52	0.50442123
LIN7C	0.85338736
LINS	0.52413845
LIPG	3.7116213

LIPM	1.196749
LIPO1	0.9948034
LIPT2	1.345505
LITAF	1.2494512
LIX1L	0.9294002
LLGL2	0.6718941
LMAN1	0.44909573
LMBRD2	0.71766067
LMO4	0.7540879
LMX1B	0.7502425
LNPEP	0.9778948
LNX1	1.2132838
LOC1008621	0.6000838
LOC1010558	0.48233604
LOC1010562	2.244411
LOC1026322	1.0962601
LOC1026331	1.279634
LOC1026336	0.41357517
LOC1026336	1.0977664
LOC1026337	0.554008
LOC1026356	1.0358956
LOC1026370	0.83708525
LOC1026380	0.89241743
LOC1026381	0.3974843
LOC1026386	0.38950253
LOC1026387	0.3772905
LOC1026391	0.64273643
LOC1026395	2.6041846
LOC1026416	0.8603351
LOC1026416	1.4134421
LOC1026424	1.3120575
LOC1026424	0.90707254
LOC1026426	0.94066286
LOC1026430	0.6840246
LOC1026431	0.742594
LOC630751	1.6630063
LOC675947	2.255162
LONRF1	0.40397024
LOX	5.1016345
LOXL2	0.5594034
LPAR1	1.4254062
LPCAT2	0.5014725
LPCAT2B	1.5590665
LPIN3	1.3687949
LPP	1.0955462

LRCH1	0.79586744
LRFN3	0.5026102
LRG1	1.6487327
LRIF1	0.69870186
LRIT2	0.8347013
LRP10	0.3370924
LRP11	0.38507032
LRPPRC	0.41885805
LRRC15	1.0190995
LRRC16A	0.7715843
LRRC25	1.5787821
LRRC34	1.207407
LRRC38	0.67703605
LRRC51	0.48934388
LRRC58	0.6876688
LRRC59	0.5279064
LRRC63	1.0064092
LRRC66	1.2240384
LRRC75A	0.86276674
LRRC8A	0.590498
LRRC8D	1.1224599
LRRK1	0.4356432
LRRTM2	1.8828206
LSG1	0.4665451
LSR	0.6625633
LTV1	1.0404816
LUZP1	0.37451982
LY6A	1.2123637
LY6C1	0.57131815
LY6K	2.456479
LY75	1.1577172
LY86	0.6294899
LY96	0.9905801
LYN	0.7373142
LYPD5	1.4307861
LYPLA2	0.4515338
LYRM1	1.0765569
LYRM7	1.5233624
LYSMD2	1.0540123
LYVE1	5.8772573
LZIC	0.48940468
LZTS1	0.3533361
M6PR	0.38941717
MACC1	1.1620202
MAD2L2	0.59100866

MADCAM1	1.0193014
MADD	0.5265293
MAFF	3.439371
MAG	0.78379107
MAGEA3	1.1942484
MAGEB17-PS	1.084713
MAGED1	1.4160414
MAGEH1	0.83393836
MAGI1	0.6158197
MAGOHB	0.5895314
MAK16	0.3081355
MAL2	3.9646535
MALSU1	0.6510658
MAMDC2	0.49217987
MAN2A1	0.8550639
MANEAL	1.1210492
MANF	0.548337
MANSC1	0.71786404
MAOB	1.9139633
MAP1B	0.6612222
MAP2K1	0.71687937
MAP3K14	0.4784503
MAP3K6	2.1813798
MAP3K8	2.7019377
MAP4	0.6811037
MAP4K3	0.41410208
MAP4K4	1.0481977
MAP7D2	0.5779896
MAPK12	0.9342098
MAPK6	0.7714119
MAPK7	0.48634005
MAPRE2	0.43435
MARCKS	1.1890674
MARCKSL1	1.7514362
MARCO	2.9514918
MARK1	1.6784344
MARK4	0.7177391
MARVELD3	2.1804857
MAS1	0.6366708
MASP2	1.121773
MAT1A	5.8755865
MAT2A	0.3305006
MATN1	2.3351865
MATN3	1.0256371
MB21D2	1.9150517

MBD1	2.8277564
MBNL2	0.43717575
MBTD1	0.31660128
MBTPS2	0.69594383
MCAM	0.5967879
MCFD2	0.6580992
MCMBP	0.40618563
MCTS1	0.5526552
MDFIC	1.4222264
MDGA1	1.0860238
MDN1	1.2129555
ME3	1.5294824
MEA1	0.41056204
MED11	0.54827166
MED28	0.35313797
MEF2A	1.171627
MEFV	2.107893
MEGF11	0.49297047
MEIG1	0.8434849
MEIS2	0.33135033
MELK	0.48423815
METRNL	0.6651306
METTL1	0.6354122
METTL16	0.522768
METTL22	0.38322353
MFSD2A	3.0896993
MFSD6L	3.6278522
MFSD7A	2.1429477
MFSD7B	1.3263597
MGARP	2.1865458
MGAT4A	1.1875181
MGEA5	0.39982462
MGRN1	0.8533592
MIA	1.5297534
MIA2	1.04604
MIER2	0.8621378
MIF	1.3101463
MIF4GD	0.9615283
MINA	0.7876663
MIPOL1	0.610677
MIR17HG	0.6597214
MITD1	0.37617207
MLKL	1.1826153
MLLT4	1.109206
MLLT6	1.5279212

MLXIP	1.052165
MMD2	0.9441309
MMP13	3.1294117
MMP14	1.9454579
MMP19	1.1979218
MMP3	7.3080344
MMP7	3.1789804
MMP8	0.79712296
MMS19	0.4508133
MMS22L	0.36041117
MNDA	1.3365707
MNT	0.5022445
MOB4	0.7602577
MORC3	0.5335598
MORF4L2	0.32675362
MORN4	0.9684181
MOV10	0.75995874
MPEG1	0.3914237
MPTX1	0.3480718
MPZL2	1.778965
MRAP2	0.46931744
MRAS	1.1315365
MREG	2.1904616
MRGPRA6	0.73936176
MRGPRB1	1.335731
MRGPRX2	0.80733395
MROH1	0.63706994
MRPL17	0.41567087
MRPL3	0.8161535
MRPL45	1.0287623
MRPS10	0.37033844
MRPS18B	0.6200652
MRPS30	0.36178255
MRPS6	0.9539981
MRPS9	0.33588886
MRRF	0.62558794
MRTO4	0.35677004
MS4A14	0.33374596
MS4A4A	3.7384038
MS4A4C	0.34437847
MS4A4D	0.68955135
MS4A6B	1.6896172
MS4A6C	1.467103
MS4A6D	3.6417885
MS4A8A	0.9629574

MSH3	0.33614063
MSN	0.8898454
MSR1	2.411994
MSRB1	0.54183865
MSTO1	0.41979694
MT1	2.896864
MT2	2.4240966
MT4	0.9171829
MTAG2	0.940135
MTAP	0.86078024
MTFR1L	0.5935855
MTFR2	0.61286354
MTHFD1L	0.38231993
MTHFD2L	1.4216862
MTM1	0.8814347
MTMR1	0.69081116
MTOR	0.41482115
MTPAP	0.5101633
MTRR	0.990469
MTUS1	0.65883064
MTX3	0.4943998
MUC2	0.8012655
MUC20	0.585788
MUL1	0.31750584
MUP3	0.4373641
MUP6	1.0512762
MUSK	0.36300135
MVB12A	0.52101994
MVP	1.4439192
MX1	2.1781206
MX2	2.0572948
MYBBP1A	1.1090465
MYBPC2	0.74261117
MYBPC3	0.36266613
MYC	1.5316672
MYCBP2	0.47175074
MYCS	0.79568434
MYD88	0.8962784
MYH4	0.95623803
MYH7B	1.3718553
MYO10	1.4927607
MYO1E	0.4805336
MYO1G	0.6007085
MYO5C	2.059449
MYPN	1.4092157

MYT1L	0.7959895
N4BP1	1.0978942
N4BP2L1	0.5093193
N4BP2L2	0.36394358
NAA20	0.52473736
NAA25	1.7503748
NAB1	0.89957047
NAB2	0.3371992
NADK	0.89504004
NAF1	0.9113097
NAGK	0.45608377
NAGPA	0.36580753
NAIP1	0.7932625
NAIP6	0.6825099
NAMPT	1.6581678
NANOS2	1.192914
NAP1L2	1.5032277
NAPSA	0.6056938
NAT10	0.83201504
NAT3	0.8836713
NCAN	1.3310657
NCDN	0.6953864
NCEH1	0.789968
NCF1	1.1562815
NCKAP5	1.0157571
NCMAP	0.8333006
NDEL1	0.60882664
NDFIP1	0.43745947
NDRG1	1.8180842
NDRG2	1.2049913
NDRG3	0.54468346
NDST1	0.40926313
NDUFA4L2	1.2516255
NDUFAF4	0.7257838
NEB	2.5923755
NECAB1	0.879323
NEFH	0.85609627
NEIL1	0.3219266
NEK6	1.4355221
NESPAS	1.4116127
NETO2	0.9046731
NEU1	1.071136
NEURL1B	0.5043206
NEURL2	0.5526295
NEURL3	1.1854486

NF1	0.7923074
NFATC4	0.57289696
NFE2L1	0.8059478
NFE2L3	0.7247653
NFIL3	3.1608481
NFKB1	0.8645086
NFKB2	1.1387668
NFKBIA	1.9806142
NFKBIB	0.8955245
NFKBID	0.78478384
NFKBIE	1.1872511
NFKBIZ	2.0981855
NFRKB	0.42941523
NGF	0.83318853
NHLH1	1.8011582
NID1	0.7617874
NIF3L1	0.4409485
NIFK	0.5236559
NINJ1	1.0137506
NIP7	0.62433577
NIPAL1	1.0461583
NIPAL2	3.6663823
NIPSNAP1	0.819705
NIPSNAP3B	0.76045704
NISCH	0.3692279
NKX1-2	0.49324393
NKX6-3	0.62461376
NLE1	0.6713848
NLGN2	0.47891712
NLN	0.95961666
NLRC4	0.77970314
NLRC5	1.3641629
NLRP3	1.4417703
NLRP4C	0.8224411
NLRP9A	1.1535149
NMB	2.0994306
NMBR	1.0425754
NME1	0.46204376
NME2	0.8453536
NME5	0.5176754
NME8	1.1132667
NMI	0.6365156
NMNAT2	0.9372964
NMRK1	0.47249198
NNAT	0.41305542

NOC2L	0.9870906
NOD1	0.67342234
NOD2	1.1433349
NOL10	0.54007673
NOL6	0.7062082
NOLC1	0.8110056
NOMO1	0.4338193
NOP10	0.5928383
NOP14	0.52339506
NOP16	1.1855989
NOP2	0.840209
NOP56	0.53760624
NOS2	2.9753404
NOS3	1.4926176
NOTUMOS	1.7559032
NOX1	0.83255243
NOXA1	2.3979673
NOXO1	0.49908495
NPC1	0.4157505
NPDC1	0.9192562
NPEPL1	0.65699244
NPFF	0.5325856
NPFFR1	0.9032345
NPHP3	0.968127
NPHP4	0.5729964
NPM1	0.6610527
NPW	1.6366134
NPY	1.8120508
NPY1R	0.82053447
NR1D2	0.5405469
NR1H3	0.9698963
NR1H4	2.892411
NR1H5	1.7134748
NR1I2	1.3237996
NR4A1	2.020092
NR4A3	2.8066244
NR5A2	2.1460028
NR6A1	0.9206691
NRAP	0.3675542
NRCAM	0.8976834
NRP2	1.4807243
NRROS	0.72197104
NRXN3	0.41388154
NSFL1C	0.7635865
NSMAF	0.5826063

NSUN4	1.0585656
NT5DC3	1.0859427
NT5E	0.80929804
NTMT1	0.525537
NTN1	1.4367414
NTNG2	1.4271281
NTRK2	0.47369146
NTS	2.7062173
NUAK2	0.9736023
NUCB2	0.48028374
NUDCD3	0.47864962
NUDT12OS	1.20785
NUDT17	2.3534946
NUFIP1	0.39086437
NUMBL	0.8301828
NUP205	0.88579655
NUP210L	0.6684258
NUP37	0.37992096
NUP98	0.4818282
NUPR1	4.745331
NUS1	1.0396032
NXN	0.40734816
NXPE3	0.6799078
OAS1A	0.93878937
OAS1F	0.8668437
OAS2	1.2951517
OASL1	2.1621833
OASL2	0.88057375
OCLN	2.339256
ODF4	0.82626176
OGFR	0.5596676
OIT1	0.44977045
OLA1	0.62762785
OLFR1	0.54026055
OLFR10	0.9740355
OLFR1012	0.90108156
OLFR1013	0.6738925
OLFR1031	1.5090137
OLFR1043	0.92969084
OLFR104-PS	1.4393394
OLFR1095	0.8123236
OLFR1097	0.95419407
OLFR1100	1.0394998
OLFR1110	1.8648324
OLFR1131	0.47350836

OLFR1135	1.6441629
OLFR1138	1.1808317
OLFR1141	1.205193
OLFR1152	1.0827212
OLFR1226	0.9643812
OLFR1232	1.5760512
OLFR1247	1.1881986
OLFR1248	1.0405571
OLFR1249	0.8877044
OLFR1251	1.5498369
OLFR1257	1.3411179
OLFR1265	1.007549
OLFR1284	1.2252581
OLFR129	1.705523
OLFR1311	0.8371742
OLFR1314	1.1273015
OLFR132	1.332911
OLFR1321	0.8292928
OLFR1322	1.1686382
OLFR1323	1.2040014
OLFR1328	1.6299968
OLFR1330	1.0475605
OLFR1335	0.8538475
OLFR1337	1.0374451
OLFR135	1.1421294
OLFR1364	1.1300254
OLFR1380	0.70134616
OLFR1390	1.3588662
OLFR1396	3.716811
OLFR1410	0.69514465
OLFR1417	0.42858362
OLFR1418	0.68223596
OLFR1423	1.487711
OLFR1425	0.9019549
OLFR1433	1.4207368
OLFR1444	2.752479
OLFR1454	1.2767236
OLFR146	1.2771256
OLFR1461	1.3338292
OLFR15	2.1912832
OLFR1508	1.3354294
OLFR157	1.1157014
OLFR160	1.0713818
OLFR164	1.0505607
OLFR187	0.69202566

OLFR190	0.47477388
OLFR194	0.6216028
OLFR204	0.94922924
OLFR213	0.7664046
OLFR224	1.0715036
OLFR247	0.49633026
OLFR273	1.5783207
OLFR286	0.9578457
OLFR29-PS1	1.3363962
OLFR352	1.565964
OLFR356	0.6917765
OLFR360	1.3480289
OLFR368	1.2838993
OLFR398	1.047749
OLFR402	1.055243
OLFR418-PS1	0.6507983
OLFR429	1.0658541
OLFR434	1.271251
OLFR441	2.181161
OLFR447	0.94815207
OLFR481	1.0753465
OLFR491	1.0912235
OLFR494	1.2553649
OLFR518	1.0670769
OLFR52	0.7604377
OLFR522	0.45390892
OLFR538	1.0572073
OLFR543	1.400774
OLFR545	0.830348
OLFR56	1.7713213
OLFR564	0.6461661
OLFR568	0.8009279
OLFR571	0.95455956
OLFR59	1.8781295
OLFR60	1.1124265
OLFR633	0.7148993
OLFR640	1.4232323
OLFR642	0.9598777
OLFR652	1.6449027
OLFR702	1.2678015
OLFR705	0.8903291
OLFR720	1.2672
OLFR730	0.77965903
OLFR745	0.56770897
OLFR792	0.8008084

OLFR796	0.8781359
OLFR811	0.946012
OLFR813	1.030076
OLFR823	1.1181698
OLFR829	1.0263987
OLFR855	0.890465
OLFR887	0.92704415
OLFR915	0.58795786
OLFR921	0.77685785
OLFR922	0.6586659
OLFR97	0.33324242
OLFR975	1.3488903
OLFR994	1.06038
OLIG2	0.53260136
OMG	1.0081134
ONECUT1	1.2742925
OOEP	1.3322942
OPN1MW	0.840888
OPRK1	0.89274216
ORM1	1.5589852
ORM2	1.9953856
ORM3	1.1531842
OSBPL10	0.62036943
OSBPL5	0.34673834
OSBPL7	0.3439374
OSM	0.7072575
OSMR	1.9837418
OSTF1	0.5625386
OSTN	1.3231254
OTOL1	1.4065251
OTUD3	1.1081586
OTUD6B	0.9490719
P2RX4	0.82289743
P2RY10	1.4546404
P2RY13	0.7867975
P2RY14	0.5683751
P2RY2	2.8449187
P2RY4	1.5848875
P4HA2	2.3601222
PACRGL	0.49437666
PACSIN3	1.1917028
PADI6	0.9539676
PAH	5.1054387
PALLD	0.4368
PAM	0.7198701

PAM16	0.42924595
PANX1	0.600729
PAPD4	0.50788975
PAPPA	2.177381
PAPPA2	1.2564888
PAQR3	1.5716403
PARD3	0.6948259
PARD3BOS1	0.9936068
PARD6B	0.8479886
PARD6G	0.6263828
PARL	0.57575226
PARM1	0.81046295
PARP10	1.0184336
PARP11	0.4981277
PARP12	1.347817
PARP14	1.1129761
PARP4	0.45535326
PARVA	0.6926451
PATL1	0.7894101
PAWR	0.80926466
PCBP3	0.5111339
PCDH19	1.9194727
PCDHB13	0.9542141
PCDHB14	1.4782963
PCDHB17	0.620162
PCDHB21	0.63982177
PCDHB22	1.2937047
PCDHGA10	1.0497596
PCDHGA9	0.829134
PCGF6	0.532289
PCMTD1	0.7896588
PCSK4	0.7877884
PCSK5	0.6832342
PDCD1	1.6317153
PDCD11	0.42628622
PDCD1LG2	1.7569675
PDCD5	0.32901525
PDE1B	0.37796164
PDE3A	0.5435939
PDE3B	1.5174928
PDE4B	1.7862859
PDE4D	0.5306096
PDE7B	1.3168564
PDE8A	0.5363612
PDGFA	1.0516696

PDGFRA	0.89512205
PDHA2	1.3183885
PDIA2	1.6590834
PDIA6	0.73323107
PDK1	0.62594604
PDK4	3.049591
PDLIM5	0.4989829
PDPN	1.2711465
PDPR	0.32402873
PDXK	0.87792873
PDXK-PS	0.8472376
PDYN	1.1006534
PEA15B	1.3349993
PECAM1	0.5841174
PELI1	1.0456758
PELI3	2.6926358
PER1	1.4352493
PERP	1.1226912
PET100	1.0734944
PEX11A	2.2034054
PEX11B	0.5582485
PEX5L	0.6141803
PFDN4	0.65816593
PFKFB3	2.9410677
PFKL	0.92311573
PFKP	0.9529185
PFN2	0.8498509
PGAM1	1.1794434
PGAM5	0.63162804
PGC	0.83192587
PGD	0.35080242
PGK1	0.94997644
PGLYRP3	0.4162619
PGLYRP4	1.1812241
PGM2	1.4826674
PGM3	0.8932059
PGS1	1.2117372
PHC1	0.3970766
PHF11A	1.4695477
PHF11B	1.1492333
PHF11C	1.4338703
PHF11D	1.4660583
PHF13	0.724998
PHF20	0.80240726
PHGDH	0.89933395

PHIP	0.48771763
PHLDA1	0.51537657
PHLPP1	1.3056169
PHOX2A	3.755387
PHYHIPL	1.5211003
PI15	4.4341507
PI4K2A	0.6091833
PIAS2	0.7232075
PID1	1.199461
PIGG	0.4474864
PIK3AP1	1.3694353
PIK3C2A	0.71100044
PIK3CG	0.6503625
PIK3IP1	1.8137751
PIK3R5	1.3505158
PIK3R6	0.6858926
PIM3	1.6493702
PIP5K1A	2.616199
PIRA2	1.0906692
PISD	0.49585915
PITPNA	0.4433565
PIWIL2	0.5406277
PIWIL4	1.283
PKD1L2	0.5819645
PKD2	0.65149164
PKM	1.1870952
PKP4	0.5339594
PLA1A	2.7266946
PLA2G1B	7.188988
PLA2G4A	0.7259064
PLA2G4F	1.8158119
PLA2G7	1.6405497
PLAC8	0.6741543
PLAGL1	0.7775676
PLAT	1.8833776
PLBD2	0.96661997
PLCB4	0.46684456
PLCD1	0.85683775
PLCL1	0.43397593
PLCXD2	1.4705572
PLD1	0.37069607
PLD2	0.49190426
PLEK	0.88423395
PLEKHA1	1.7086105
PLEKHB1	0.595639

PLEKHB2	0.41735888
PLEKHG4	3.1131659
PLEKHG6	1.2131798
PLEKHH1	1.3450077
PLEKHS1	3.5034165
PLET1	3.9196117
PLG	1.7543261
PLIN5	1.00154
PLK2	2.4335504
PLK3	1.0950465
PLOD1	0.62709904
PLOD3	0.6519437
PLSCR1	1.9996581
PLTP	0.49268198
PLXNA2	0.6099696
PLXND1	1.2297225
PMAIP1	1.7055926
PMEPA1	1.6915817
PML	0.33456564
PNLIP	11.910057
PNLIPRP1	12.272474
PNLIPRP2	8.864243
PNO1	0.88610125
PNPLA2	0.8660855
POGK	1.2910726
POGLUT1	0.83549595
POLG2	0.6774998
POLR1A	0.92193556
POLR1B	0.91803074
POLR1E	0.48014832
POLR2G	0.45386076
POLR3D	0.7631588
POLR3E	0.7147045
POLR3G	0.5974293
POM121L12	0.8660517
POMP	0.5680237
PON1	1.1308365
POP4	0.9753146
POR	0.9684715
POT1A	0.525023
POU2AF1	0.94159985
POU2F3	0.59968734
POU3F2	0.8663943
POU5F1	1.6910386
POU6F1	0.51508904

POU6F2	0.3698008
PPA1	1.2546949
PPAN	0.33552027
PPAP2B	1.2955084
PPAPDC2	0.3953519
PPARD	0.49919844
PPARGC1A	0.5838878
PPARGC1B	0.43278646
PPM1H	1.5675001
PPP1R14B	0.43252373
PPP1R16B	0.7685714
PPP1R21	0.7570596
PPP1R3B	0.6176605
PPP1R3F	0.85010195
PPP1R3G	3.314858
PPP2CB	0.44356966
PPP2R2A	0.68584967
PPP3CC	0.9734912
PPP4R1	0.6755543
PPP5C	0.67713785
PPY	0.5767069
PRDM1	1.7482834
PRDX6	0.87670565
PRELID2	0.83059454
PREX1	0.36130428
PRF1	0.6701851
PRH1	0.34071708
PRICKLE1	0.72429657
PRKAR1A	0.6603551
PRKCB	0.6048498
PRKCE	0.33992052
PRKCH	0.61297894
PRKCI	0.5507097
PRL2C5	1.5727446
PRLH	1.4533217
PRLR	1.3273633
PRM2	1.1919394
PRMT6	0.799515
PRMT8	1.2826977
PROCR	3.618147
PRODH2	0.44830418
PROK2	3.0057826
PROM2	1.2584155
PRPF31	0.7490783
PRPF40A	0.35123062

PRPF40B	0.81409264
PRPS1	0.5291939
PRPSAP1	0.3712268
PRR15L	2.6731608
PRR19	0.35007763
PRR9	0.5597544
PRRG1	0.3090222
PRRG3	1.4177318
PRRT4	1.9975364
PRSS1	11.970859
PRSS16	1.2457132
PRSS2	11.388641
PRSS22	1.2540193
PRSS23	1.9111834
PRSS23OS	1.4339793
PRSS51	0.7181952
PRSS8	3.1156712
PSAT1	1.6904659
PSD3	1.4230576
PSEN2	0.46287537
PSG18	3.2187438
PSG19	1.89183
PSG22	4.0331936
PSG25	1.3487909
PSG27	0.8402257
PSG28	1.1870906
PSMA3	0.64519453
PSMA5	0.48001003
PSMA6	0.7925892
PSMB10	0.46780777
PSMB11	2.3407602
PSMB2	0.47347116
PSMB6	0.31065512
PSMB8	0.61333036
PSMD12	0.39998293
PSME1	0.3698864
PSME2B	0.7848563
PSPH	0.77109194
PSTPIP2	0.5604315
PTAFR	1.1130052
PTBP2	0.56595945
PTEN	0.42120886
PTF1A	3.8468084
PTGER3	0.93990445
PTGER4	0.31369495

PTGES	0.60428286
PTGES3	0.4525051
PTGES3L	0.73459697
PTGFRN	1.0133717
PTGS2	5.0223465
PTH2	0.61101913
PTP4A1	0.79170465
PTPLB	0.617095
PTPMT1	0.90186405
PTPN1	1.374877
PTPN13	1.0728521
PTPN2	1.0661154
PTPN22	1.886601
PTPN23	0.6605358
PTPN5	0.5832877
PTPRK	0.72187686
PTPRM	1.2718668
PTPRN	1.2621777
PTRH1	1.1120114
PTTG1IP	0.3444252
PTX3	6.8272185
PUS7	1.0168381
PUS7L	0.935307
PVALB	1.9028728
PVR	2.4597635
PVRL2	0.89317083
PVT1	0.5821481
PWP1	1.064795
PWP2	0.8208413
PYDC3	2.377266
PYDC4	2.6992168
PYHIN1	0.7487378
PZP	3.6147332
QPRT	0.3811679
QSOX1	0.6240659
RAB1	0.6149349
RAB10	0.58015823
RAB11FIP1	1.119899
RAB11FIP5	0.7470927
RAB12	0.64742565
RAB15	0.9620557
RAB20	2.3292074
RAB25	2.1044796
RAB26	1.9378207
RAB2A	0.31566

RAB2B	0.6867566
RAB32	0.5086918
RAB33A	0.75088143
RAB34	0.53404903
RAB3IP	0.46789694
RAB4B	0.7325282
RAB9B	1.2422681
RABEPK	0.5690398
RABL3	0.3966298
RAD21L	1.0581472
RAD23B	0.67858887
RAET1A	2.4503706
RAET1B	1.1363282
RAET1C	1.4170601
RAET1E	1.1714883
RAI14	1.2184491
RALA	0.6323023
RALGPS1	0.52762985
RAMP3	4.0458937
RANBP3L	1.149523
RANBP9	0.4768119
RAP1GDS1	0.5720639
RAP2A	1.4160886
RAP2B	1.5408983
RAPGEF2	2.460144
RAPGEF5	1.2868166
RAPGEF6	0.5354252
RAPH1	0.5680857
RARRES1	0.6992583
RARRES2	1.4534912
RASA2	0.97396517
RASA4	1.1242571
RASAL2	0.53934073
RASD1	4.4723964
RASGEF1B	1.7002549
RASIP1	1.700501
RASSF1	0.55606556
RAVER2	2.9591846
RAX	1.6602025
RBBP6	0.32013893
RBFOX2	0.90900564
RBM17	0.512712
RBM19	0.47800684
RBM22	0.46239018
RBM25	0.33912992

RBM26	0.5663452
RBM27	0.6224828
RBM28	1.0091505
RBM34	0.81263494
RBM46	1.4727387
RBM47	0.568346
RBMS1	1.1784163
RBMS2	0.6889186
RBMX	0.41754675
RBP1	0.76360655
RBPJL	1.0739872
RCC2	0.7881565
RCL1	0.620718
RD3	0.7961197
RDH1	0.8223617
RDH13	0.62989545
RDH5	0.40542412
RDH9	0.5280585
REEP3	0.51108456
REG1	13.760693
REG2	13.868011
REG3A	12.278239
REG3B	14.605612
REG3D	10.019589
REG3G	13.4921055
RELA	0.7493038
RELB	1.2863464
REN1	0.4360857
REPIN1	0.90205
RFTN1	0.7601552
RFX5	0.50963545
RGAG4	1.227143
RGL1	0.32624102
RGS1	3.1003056
RGS17	1.3670671
RGS19	0.35181236
RGS4	2.069599
RGS5	0.69620895
RGS8	0.62395525
RGS9	1.1631451
RHBDF1	0.8426752
RHBDF2	0.75272036
RHEBL1	1.2307706
RHOB	0.96208596
RHOBTB1	0.58605623

RHOC	0.9923868
RHOQ	0.967504
RHOU	1.2330546
RHOX3F	0.31591654
RHPN2	0.6318579
RIBC2	1.0821962
RIC3	0.75656796
RIC8	0.6273198
RILPL1	0.5885167
RILPL2	1.287044
RIMS2	2.1230638
RIMS4	0.9608853
RIOK2	0.6819453
RIPK1	0.58239555
RIPK2	0.8808031
RIPK3	0.6283922
RIPK4	2.1969376
RLIM	0.6912074
RN18S	0.98662996
RN45S	0.5354018
RNASE1	10.185574
RNASE4	3.011959
RNASEH1	0.47139168
RNASET2A	0.68961287
RND1	1.7449088
RNF103	0.81297016
RNF125	1.9208436
RNF138	0.8497958
RNF149	1.7581081
RNF157	1.1542983
RNF185	1.044322
RNF186	3.151113
RNF19B	1.4364729
RNF213	1.7719874
RNF215	0.5160179
RNF217	1.4666889
RNF39	0.60066295
RNFT2	0.83881354
RNU1B6	0.8725338
ROBO4	1.1814508
ROR2	1.4831662
RPAP3	0.5156174
RPL39L	1.1892343
RPL7L1	0.58676624
RPP40	1.2160497

RPS4L	1.5216317
RPS6KB1	0.59629965
RPTOR	0.44326782
RPTOROS	0.3661735
RRAD	1.382029
RRAS2	1.3488345
RRBP1	0.9010148
RRP12	0.7748308
RRP15	0.7597933
RRP1B	1.023047
RRP8	0.4194579
RRP9	0.7927351
RRS1	1.2555704
RS5-8S1	2.6520095
RSAD1	1.3308706
RSL24D1	0.68459797
RTP3	0.9554813
RTP4	1.4306049
RUFY2	0.47437716
RUFY3	0.7586484
RUNDC1	0.30706072
RUNDC3B	1.9817231
RUNX1	0.60463095
RUNX2OS1	0.41221976
RUNX3	0.9555454
RUSC2	1.2382207
RXFP1	0.5484624
RYBP	0.43519592
S100PBP	0.538877
SAA1	5.298794
SAA2	8.709419
SAA3	5.4856324
SAG	2.1490066
SAMD4B	0.81084824
SAMD8	1.0169346
SAMHD1	0.8862777
SAMSN1	1.6139417
SAR1B	0.32892323
SASH1	1.1642704
SAT1	0.4189906
SATB1	1.8365579
SAV1	0.5878587
SBF2	1.2415264
SBNO2	1.5459514
SCAMP1	0.6627836

SCARA3	0.5789051
SCARA5	1.1783211
SCARF2	0.6098528
SCCPDH	0.42328835
SCGB1B19	1.3179705
SCGB1B2	0.8352697
SCGB1B27	0.9500091
SCGB2B26	0.8923559
SCGB2B7	0.90485907
SCGB3A1	0.5012944
SCHIP1	0.41977644
SCIMP	0.41928768
SCIN	3.4625468
SCML1	1.0974386
SCML4	0.8452697
SCN11A	1.286978
SCN1A	0.3991909
SCN3A	0.84670043
SCN5A	0.92508507
SCN8A	0.48725986
SCO1	1.1087518
SCPEP1	0.34847498
SCRT1	2.5961456
SCRT2	1.1023424
SCT	1.392693
SDC1	1.533021
SDC4	3.422792
SDCBP2	0.9150491
SDHAF1	1.3111482
SDK1	0.3867302
SDR39U1	0.75992775
SDR9C7	0.8162627
SDS	2.1619833
SEC1	1.8523443
SEC11A	0.357244
SEC13	0.4966793
SEC23B	0.39737082
SEC24A	0.8866811
SEC24D	1.1031218
SEC31A	0.6023731
SEC61A1	0.5380454
SEC63	0.83223486
SECTM1B	0.966177
SEL1L3	0.6518605
SELE	3.2909698

SELK	0.42493248
SELL	1.2624636
SELP	0.5074372
SELT	0.46750927
SEMA3A	2.1337109
SEMA3E	1.1268806
SEMA3F	1.5098381
SEMA3G	1.5767436
SEMA4A	0.45995808
SEMA4C	0.57885075
SEMA6B	0.76297903
SEMA6D	0.63842344
SEMA7A	1.9752951
SENP5	0.4510045
SEPW1	0.59838724
SERINC1	0.44816828
SERINC2	1.2811272
SERP1	1.1744175
SERPINA10	1.7233405
SERPINA1A	0.44704485
SERPINA1C	0.42771196
SERPINA1D	0.41743422
SERPINA3B	1.0290406
SERPINA3C	4.130486
SERPINA3F	2.873423
SERPINA3G	2.03688
SERPINA3H	0.7550812
SERPINA3I	2.1553273
SERPINA3K	6.0869713
SERPINA3M	2.5474672
SERPINA3N	3.9883847
SERPINA5	0.87769556
SERPINB1B	1.4844162
SERPINB1C	0.70320773
SERPINB6B	0.81572056
SERPINB6D	0.9631543
SERPINB7	1.9256096
SERPINB8	0.61342716
SERPINB9	0.98894024
SERPINB9D	0.95934224
SERPINB9E	0.90870094
SERPINC1	0.7523091
SERPINE1	5.208542
SERPING1	1.2945924
SERPINI1	0.74627304

SERPINI2	7.5899296
SESN1	0.44315672
SESTD1	0.9034114
SET	0.48585844
SETD4	0.63612604
SETDB2	0.681818
SEZ6L2	0.62358403
SFI1	1.4509997
SFMBT2	2.016742
SFR1	0.7105417
SGCB	1.2728329
SGK2	2.6469991
SGPL1	0.7245102
SGSH	0.5512557
SH2D1B2	1.0046391
SH3BP2	0.6798072
SH3BP5	0.62536955
SH3D21	0.6121373
SH3GL3	2.156971
SH3PXD2B	0.82336664
SHBG	0.45251656
SHE	0.541131
SHISA3	4.1441927
SHISA5	1.0528941
SHMT1	1.2072141
SHMT2	0.7895074
SHROOM2	1.2759628
SIAH2	2.130128
SIDT1	0.66260195
SIGLECE	0.6362896
SIGMAR1	0.34878826
SIK1	1.5646172
SIN3B	0.8205972
SIPA1L1	1.3467512
SIX3OS1	0.37659907
SKAP2	0.8073306
SKIL	1.1012516
SKINT1	0.97017026
SKINT7	0.5029192
SKOR1	2.1334264
SKOR2	0.49780393
SLA	0.6695566
SLAIN1	0.52078915
SLAMF1	1.3513851
SLAMF7	0.9527726

SLAMF9	1.6268735
SLC10A2	0.60418177
SLC10A3	0.4462533
SLC10A6	3.554876
SLC11A1	1.3129306
SLC12A3	1.3903363
SLC12A4	0.38147306
SLC12A8	1.5886798
SLC13A5	0.980268
SLC15A2	0.9235368
SLC15A3	2.3709831
SLC16A3	2.2731867
SLC16A9	0.6478362
SLC17A9	0.43161678
SLC18A3	0.8150432
SLC19A1	0.6285701
SLC1A4	0.8933053
SLC22A12	1.0199385
SLC22A13	0.68942094
SLC23A2	0.90735793
SLC25A17	0.43770027
SLC25A19	1.1655793
SLC25A22	2.3781998
SLC25A25	0.44929743
SLC25A28	0.42288065
SLC25A29	0.85202956
SLC25A32	0.6142607
SLC25A33	0.77701426
SLC25A34	0.8885188
SLC25A36	0.6368809
SLC25A4	0.6094923
SLC25A48	0.5751116
SLC25A5	0.35679674
SLC25A53	0.6459975
SLC26A4	2.2705345
SLC26A9	4.702795
SLC27A1	0.9123969
SLC27A5	0.74379325
SLC28A3	1.3395741
SLC29A2	1.435677
SLC2A1	2.090353
SLC2A10	1.1656337
SLC2A6	2.782195
SLC2A7	0.95133185
SLC30A4	0.85532856

SLC30A7	0.5757463
SLC31A2	1.6257424
SLC33A1	0.42683887
SLC34A2	1.2769227
SLC35B3	0.43054867
SLC35E3	0.5549288
SLC35E4	0.5177183
SLC35F2	1.6357656
SLC35F5	1.1372194
SLC35G2	0.8134506
SLC36A1OS	1.0136757
SLC36A2	0.45638895
SLC38A1	0.3674512
SLC38A2	0.30797005
SLC38A3	2.6567526
SLC38A4	1.7701435
SLC39A1	0.89051485
SLC39A10	0.85345125
SLC39A14	3.0932593
SLC39A4	0.5498781
SLC39A5	1.3576713
SLC39A6	0.9984851
SLC44A4	0.986856
SLC46A2	0.8613136
SLC47A2	0.538527
SLC4A4	2.5535862
SLC4A7	0.5550108
SLC50A1	0.4128394
SLC52A2	0.6308017
SLC5A3	0.5304842
SLC5A6	0.57000303
SLC5A8	1.2639942
SLC6A16	0.4355693
SLC6A19OS	1.2445407
SLC6A6	0.5859146
SLC6A7	0.9304879
SLC7A1	0.6717005
SLC7A11	1.622761
SLC7A13	1.0951302
SLC7A2	2.5216537
SLC7A3	3.561843
SLC7A5	0.44217634
SLC7A6	1.0541601
SLC7A8	0.82006407
SLC8B1	1.0369887

SLC9A5	1.1304317
SLC9A6	0.38847542
SLC9B1	0.7929797
SLCO1B2	0.922544
SLCO3A1	1.054429
SLCO4A1	0.74429417
SLFN1	1.3981452
SLFN2	1.3890643
SLFN4	1.2942853
SLFN5	0.44209385
SLFN8	0.7987828
SLITRK3	0.7221937
SLITRK4	0.8711877
SLITRK5	0.9603896
SLPI	0.45761156
SMAD1	1.7256789
SMAD2	0.9944539
SMAD3	0.7170725
SMAD4	0.86426497
SMAD5	0.45953083
SMAGP	0.6238165
SMCO3	1.0858402
SMIM22	3.717721
SMN1	0.36650085
SMOC1	1.3785474
SMOC2	0.58946276
SMPDL3B	0.8843465
SMS	0.63210106
SMYD2	0.7020917
SMYD3	0.3838868
SMYD5	1.1021118
SNAPC1	0.49052525
SNAPC4	0.44784355
SNHG1	0.75518036
SNHG12	1.1369357
SNHG3	0.60962486
SNHG7	0.74445057
SNORA74A	0.6754346
SNTB2	1.6369514
SNX10	1.6010828
SNX12	0.45786572
SNX18	1.4823179
SNX20	0.5238662
SNX6	0.6468134
SNX7	0.7517309

SOCS1	2.369255
SOCS2	1.7987466
SOCS3	3.7506132
SOD2	1.9517536
SOHLH2	0.5312941
SORBS3	0.7462976
SORCS1	1.4935014
SOSTDC1	1.4543424
SOWAHC	1.6592469
SOX7	1.6990426
SOX9	1.0603983
SP140	0.49705553
SP6	1.1658945
SPACA3	0.9640093
SPAG9	0.368248
SPATA20	0.5447478
SPATA25	0.6848521
SPATA5L1	0.4697349
SPATS2	0.90326476
SPDEF	2.363796
SPEER1-PS1	0.3062334
SPEER2	0.78205466
SPEER4A	0.47929478
SPEER4B	1.2317321
SPEER5-PS1	0.59509015
SPEF2	0.89495325
SPG20	0.7447121
SPG21	0.3434124
SPIC	0.9985318
SPIN1	0.9407983
SPINK14	0.87632847
SPINK3	10.965017
SPINK5	1.063067
SPINT1	1.0895622
SPINT2	0.53434706
SPINT4	1.3428802
SPO11	0.67228746
SPON1	2.191512
SPPL2A	1.0695133
SPRED1	1.0028687
SPRED2	0.879158
SPRR2D	6.259442
SPRR2E	1.401098
SPRR2G	3.1861439
SPRY2	0.8292749

SPRYD7	0.74718475
SPSB1	0.99877095
SPTLC2	0.40502167
SPTSSB	1.0652099
SRA1	0.48229122
SRD5A2	1.5772662
SREK1	0.48956108
SRFBP1	0.7982769
SRGAP1	1.3160167
SRGN	0.76314926
SRM	0.75351477
SRP68	0.33925962
SRP9	0.37924194
SRPRB	0.61704826
SRRM3OS	1.6378489
SRY	1.2563403
SSH2	1.1888514
SSH3	1.067162
SSR3	0.60394955
SSSCA1	0.52830553
SSTR1	0.7064471
SSU72	0.54593325
SSX9	0.89829826
SSXB7	0.84708214
ST13	0.5791054
ST14	0.5101285
ST6GALNAC4	1.2187662
ST6GALNAC5	1.3565638
ST8SIA1	0.72857976
ST8SIA2	1.1102021
STAB1	1.2723045
STAC	1.3148618
STAG3	0.5245116
STAM	0.525146
STAM2	1.0691328
STARD13	0.71599865
STARD5	0.45637465
STARD7	0.97462606
STAT2	1.0791183
STAT3	1.5922232
STAT4	1.4695821
STAT6	0.34333944
STC1	2.9026022
STEAP1	5.815458
STEAP2	3.4546752

STEAP4	1.7407432
STFA1	1.8842936
STFA2	2.270844
STFA2L1	2.1803465
STFA3	2.043644
STK17B	1.1825233
STK19	1.1249294
STK32A	1.9159834
STK39	1.6758242
STK40	0.8640642
STOML1	0.7959361
STOML3	1.0439568
STON1	1.8721786
STRA6	2.9757552
STRIP1	0.6257701
STRIP2	1.3041162
STX12	0.65057755
STX18	0.43388033
STX8	0.4894786
STXBP3A	1.0871911
STXBP3B	0.92165613
STXBP5L	0.925756
SUCO	0.62087154
SULF1	1.114336
SULT3A1	0.9414754
SUPV3L1	0.50230026
SURF4	0.8062811
SURF6	0.7868142
SUSD2	1.4056592
SUV39H2	0.3763671
SVAL1	1.3814425
SYAP1	0.45990038
SYCE1L	1.1351402
SYCN	11.438496
SYCP1-PS1	0.6226635
SYDE1	0.88934565
SYK	0.919302
SYN1	1.002953
SYN2	1.4022579
SYNCRIP	0.5463171
SYNGR2	0.6656656
SYNGR3	0.71668863
SYNPO	1.1792257
SYNPO2L	0.65539026
SYNPR	0.58958006

SYP	0.40308332
SYPL	0.5376773
SYT13	1.9684186
SYT2	1.0723736
SYT8	0.6178477
SYTL3	1.8535318
TAAR7B	2.1089823
TAC1	1.0268321
TADA2A	0.3336668
TADA2B	0.34549975
TAF1A	1.1950788
TAF1D	0.9417496
TAF4B	1.0538545
TAGAP	0.5232825
TAGLN2	0.6936984
TAL2	1.182488
TANC1	0.63597393
TAP1	0.94574356
TAP2	1.1194959
TAPBP	0.6789074
TAPBPL	0.73923874
TARS	0.5695834
TAS2R106	1.2936621
TAS2R134	1.8202925
TAS2R138	1.2387142
TAS2R143	0.9419315
TAT	1.1321483
TBC1D1	1.050951
TBC1D14	0.34186125
TBC1D15	0.4169197
TBC1D23	0.49372196
TBC1D30	1.4195232
TBC1D4	1.641192
TBC1D9	1.3600245
TBCB	0.57169294
TBK1	0.58095455
TBL3	0.4376073
TBX1	2.2526228
TBX3	1.5553284
TC2N	3.9086185
TCAM1	1.6297574
TCF4	0.64388895
TCF7	0.77854824
TCIRG1	0.37806654
TCL1B2	1.0477102

TCOF1	0.5511341
TCP10B	1.5949643
TCP10C	0.88962245
TDG	0.92742157
TDH	2.8079844
TDRD1	0.89934945
TDRD7	0.72508097
TDRD9	1.0056896
TDRP	1.0889893
TEAD1	1.5427728
TEAD3	1.1720121
TEAD4	1.5199823
TEKT3	1.2453947
TES	1.1836772
TET2	0.76671505
TEX10	0.5125952
TEX12	1.2617378
TEX26	1.684068
TEX264	0.4801507
TEX40	0.94087005
TFCP2L1	1.035891
TFF2	8.525592
TG	0.92957306
TGFA	0.7086618
TGFBR2	0.43873405
TGFBR3	0.7243352
TGIF1	2.2604098
TGM2	2.2604632
TGM4	0.9077215
TGOLN1	0.57487726
TGOLN2	0.44109154
TGTP1	1.0196567
TGTP2	1.0470119
THA1	1.7477489
THADA	0.63043237
THBS1	0.9789958
THBS4	1.5162158
THEG	0.8341849
THEM4	0.8214879
THEMIS2	0.4544158
THOC5	0.44535446
THUMPD1	0.65816116
TIGD2	0.72164106
TIGIT	1.2931657
TIMM10	0.3970499

TIMM23	0.55886173
TIMM8A1	0.5411372
TIMM9	0.75055027
TIMP1	4.2608776
TIMP3	1.4718428
TIPARP	2.443614
TIRAP	0.5554395
TLE1	0.477571
TLK2	0.36935997
TLR2	1.3897991
TLR6	1.0996609
TLR7	0.89715815
TM2D1	1.0242734
TM4SF1	0.39056396
TM6SF2	1.1199608
TM9SF3	0.346673
TM9SF4	0.36402702
TMA16	2.0654426
TMBIM1	1.1230416
TMBIM4	0.5713415
TMC1	1.2795935
TMC3	3.3170109
TMC5	4.697483
TMCC3	0.32741547
TMCO4	1.1469512
TMED1	0.4486246
TMED11	1.6272044
TMED5	1.1518636
TMED6	3.60428
TMEFF1	1.8298817
TMEM11	0.64293003
TMEM119	1.5560007
TMEM120A	0.7126632
TMEM123	0.807518
TMEM125	2.179367
TMEM128	0.57929134
TMEM131	1.0025492
TMEM132E	3.855729
TMEM139	0.7583778
TMEM140	1.1005473
TMEM150C	1.2308626
TMEM159	1.6043558
TMEM161B	0.55207705
TMEM17	0.61724496
TMEM171	2.7049851

TMEM173	0.6875181
TMEM176B	0.5776324
TMEM178	1.3619487
TMEM181A	0.74617195
TMEM184B	0.6925316
TMEM185B	0.8500514
TMEM192	0.60327387
TMEM199	0.49795866
TMEM202	1.6013334
TMEM213	0.96437883
TMEM215	0.44020653
TMEM217	0.7328696
TMEM239	0.53048134
TMEM243	0.6660409
TMEM245	0.33235502
TMEM248	0.47218847
TMEM251	0.439281
TMEM252	3.6473072
TMEM253	0.59702563
TMEM30A	0.48684072
TMEM30B	3.1504097
TMEM45A	1.3637865
TMEM5	0.5919938
TMEM62	0.79506874
TMEM63C	3.535708
TMEM68	0.39681578
TMEM70	0.5670576
TMEM87B	1.0862441
TMEM88	0.49681902
TMEM92	1.2119703
TMEM97	0.58215904
TMEVPG1	0.61439776
TMPRSS2	2.8746417
TMPRSS3	0.6216445
TMTC2	2.2053819
TNC	1.3498442
TNF	2.2247972
TNFAIP1	1.4842715
TNFAIP3	3.5346022
TNFAIP6	2.9627078
TNFRSF10B	1.6036491
TNFRSF11B	1.0473626
TNFRSF12A	2.2571573
TNFRSF18	1.4665303
TNFRSF1A	0.5760875

TNFRSF1B	1.0171871
TNFRSF23	0.48742795
TNFRSF25	1.398449
TNFRSF26	1.1265693
TNFRSF4	2.2342062
TNFRSF8	0.5900984
TNFRSF9	0.8789964
TNFSF15	2.2809033
TNFSF8	0.99408126
TNFSF9	2.201325
TNIP1	1.954802
TNIP2	1.6103606
TNIP3	4.466367
TNKS1BP1	0.8487234
TNKS2	0.46936607
TNN	2.6868262
TNNI3K	0.6935127
TNP2	2.1175797
TOMM20L	1.016763
TOMM40	0.4407997
TOMM5	0.7692337
TOP1MT	1.0662346
TOR3A	1.5138769
TOX2	0.6330507
TOX3	1.0783398
TPD52	0.6628871
TPD52L1	1.279635
TPI1	0.6641803
TPM3	0.66782
TPM4	0.3893795
TPSAB1	1.2964227
TPST2	0.5553222
TRAF1	1.8038673
TRAF3	0.8093934
TRAFD1	0.9884696
TRAK1	0.42465305
TRAPPC4	0.36607313
TRDMT1	0.7035732
TREM1	0.9971881
TREML2	0.6679802
TREX1	0.9749718
TRIB1	0.8739462
TRIB2	1.0894666
TRIB3	3.35849
TRIM12C	0.7314043

TRIM13	0.7711139
TRIM14	0.39172316
TRIM25	0.444983
TRIM26	0.7476773
TRIM29	1.4181259
TRIM30A	1.3126278
TRIM30B	0.66664124
TRIM30C	2.2132757
TRIM30D	1.0439835
TRIM31	0.9569483
TRIM35	0.91408396
TRIM36	0.7328589
TRIM42	0.49999523
TRIM47	0.88115025
TRIM50	3.1944678
TRIM72	0.9623537
TRIML2	1.0480249
TRIO	0.55609226
TRIP10	1.0315013
TRMT1	0.44081783
TRMT61B	0.50571203
TRNAU1AP	0.9018235
TRNP1	4.285512
TRNT1	0.52411604
TRP53I11	0.615891
TRP53INP1	0.6227355
TRP53INP2	1.0025172
TRPC5	0.92217994
TRPV4	0.88921213
TRY4	10.630831
TRY5	10.727789
TSC22D3	1.1594801
TSGA13	1.4173684
TSHZ3	1.1508942
TSKU	0.7853408
TSPAN1	2.2052345
TSPAN11	0.43615055
TSPAN3	0.8711829
TSPAN6	0.4786296
TSR1	0.60425806
TTC12	0.69598293
TTC18	1.1909707
TTC27	0.4278202
TTC39B	1.5162454
TTC39C	0.5811329

TTC39D	0.96767473
TTI2	0.65555096
TTL	0.43212295
TTLL2	1.8038628
TTLL8	0.7087438
TTLL9	1.0355744
TTR	1.7579603
TTYH1	1.5412273
TUBB2B	0.8482609
TUBB6	1.7016501
TULP3	0.5086663
TULP4	1.4875569
TUT1	0.55201435
TWIST2	1.4831185
TXK	1.4036832
TXNRD1	0.94198036
TXNRD3	1.4877176
TYRO3	0.5863886
TYW5	0.79343796
U90926	3.1142192
UACA	0.6560993
UBA5	0.588665
UBAC2	0.54751253
UBD	2.1790743
UBE2D3	0.6038208
UBE2E1	0.71363926
UBE2E2	0.3610015
UBE2G1	0.6834917
UBE2J1	0.560493
UBE2Q2	0.54853916
UBE2QL1	1.5183513
UBL3	1.040431
UBQLN1	1.0248709
UBR4	0.3432412
UBXN6	0.4326334
UCHL3	0.92898273
UCK2	0.36954355
UFL1	0.5648556
UFM1	0.6322694
UGDH	0.6073003
UGP2	0.3364849
UGT3A2	0.7106509
UMOD	2.5272975
UMODL1	0.5566051
UNC13B	0.9556143

UNC45A	0.67680836
UNC5C	0.56893635
UNC80	0.566062
UNC93A	1.6676438
UNG	0.52295876
UOX	0.72076106
UPK1A	0.5007951
UPK3BL	0.52772737
UPP1	3.4946098
URAH	4.4691677
UROC1	1.5111439
USP13	1.154702
USP17LE	1.1032503
USP18	1.8031125
USP22	0.80157423
USP3	0.84462976
USP30	0.6759758
USP53	1.4009161
UST	1.030062
UTF1	0.6169467
UTP11L	0.7855072
UTP15	0.5657253
UTP20	0.49699497
VAMP4	0.4874401
VAMP7	0.5798869
VAPA	0.7115483
VAT1	0.9817066
VAV1	0.8458824
VCAN	1.3548017
VCPKMT	0.61251307
VDAC2	0.77377367
VDR	3.7026114
VEPH1	1.2416937
VGF	6.6463757
VIL1	2.2449396
VIMP	0.7711973
VLDLR	1.3554454
VMN1R13	1.0258842
VMN1R18	1.0504339
VMN1R212	1.0800683
VMN1R236	1.2254503
VMN1R24	1.0247321
VMN1R54	0.9567051
VMN1R72	0.7387788
VMN2R11	0.6733811

VMN2R12	1.620297
VMN2R121	1.3509657
VMN2R13	1.2616496
VMN2R88	0.57313323
VMN2R96	0.8968265
VMP1	1.3127623
VNN1	1.1049211
VNN3	3.52235
VPS33B	0.49228525
VPS37B	0.6167941
VPS4B	0.4607644
VPS54	0.8166466
VSIG2	0.46673083
VSIG8	0.86445665
VTI1A	0.42990637
VTN	5.4852066
VWA2	1.063551
VWA3B	0.66003275
VWA5A	0.7472005
VWA5B2	0.88782334
VWA8	0.51903534
WARS	1.2256861
WASF1	0.7050872
WASH	0.5529032
WBSCR17	0.7931485
WDR1	1.1320806
WDR12	0.4055419
WDR3	0.37610722
WDR36	0.6367297
WDR37	0.47156858
WDR43	1.0092511
WDR45B	0.43328
WDR46	0.39084673
WDR52	0.37097645
WDR59	0.51519847
WDR64	1.0761154
WDR77	0.36481285
WDR78	0.35582495
WDR89	0.43945265
WDR92	2.5690227
WFDC12	1.050776
WFDC15A	1.2319648
WFDC17	3.1163697
WFDC18	3.1319094
WFIKKN2	0.9740672

WFS1	0.67546797
WIPF3	0.5825336
WNK2	2.5982687
WNK3	0.94614387
WNT8A	1.2858758
WNT9B	2.0715234
WT1	0.7737198
WWOX	0.6246116
WWTR1	0.45698762
XAF1	0.5886984
XBP1	0.99797773
XKR8	1.63662
XLR3B	0.5214877
XLR5B	1.1196456
XPA	0.5060172
XPOT	0.72723436
XRCC4	0.49456072
XRCC6BP1	0.85783863
XRN2	0.768219
XXYLT1	0.41712904
YAE1D1	0.9986057
YAP1	0.76591015
YARS	0.88887835
YDJC	0.82159424
YIPF6	0.3097167
YTHDC1	0.56255865
YWHAG	0.87340736
ZAP70	0.33581972
ZBBX	0.6151109
ZBED3	0.7759435
ZBED5	0.52731085
ZBED6	0.73523617
ZBP1	2.3588014
ZBTB10	1.1795781
ZBTB16	2.6813579
ZBTB18	0.6534548
ZBTB25	0.6499615
ZBTB26	0.67897916
ZBTB32	1.6441598
ZBTB37	0.60935235
ZBTB8B	1.4378688
ZC2HC1A	0.3652897
ZC3H12A	2.1730754
ZC3H12C	0.8585911
ZC3H12D	0.98511076

ZC3H7A	0.63232327
ZC3H8	0.6635146
ZC3HAV1	0.44222403
ZCCHC14	0.37848473
ZCCHC2	0.81520796
ZCCHC9	0.58644915
ZDHHC18	0.9266176
ZDHHC19	1.1738532
ZDHHC2	0.5694499
ZDHHC23	0.9118438
ZFHX4	0.86230636
ZFP1	0.98063564
ZFP105	0.60368013
ZFP112	1.1910517
ZFP133-PS	0.7767739
ZFP169	0.72185516
ZFP202	0.7740834
ZFP26	0.42027855
ZFP263	0.8831239
ZFP275	0.5189209
ZFP281	1.48663
ZFP287	0.3240943
ZFP296	0.35565233
ZFP319	0.70943356
ZFP334	1.2713356
ZFP345	0.48257184
ZFP36	1.8616858
ZFP365	1.0823321
ZFP366	1.0918634
ZFP36L1	0.95060444
ZFP36L2	1.0546021
ZFP37	0.6343641
ZFP386	0.6338992
ZFP420	0.5575874
ZFP428	0.6910851
ZFP462	1.2298632
ZFP513	0.92262983
ZFP516	0.6514549
ZFP52	0.7501354
ZFP532	0.29983234
ZFP536	0.7895498
ZFP566	0.6438627
ZFP57	0.57289886
ZFP575	1.3432176
ZFP612	0.78151083

ZFP64	0.49258327
ZFP648	0.4125061
ZFP653	0.4065609
ZFP691	0.7331891
ZFP692	0.38758945
ZFP697	1.9287434
ZFP738	1.6277845
ZFP783	1.067224
ZFP790	1.3532209
ZFP799	0.63856673
ZFP811	4.0753508
ZFP819	1.7347836
ZFP85OS	1.3585482
ZFP868	0.42039347
ZFP9	1.4823241
ZFYVE16	0.54144955
ZFYVE20	0.57118845
ZG16	6.806767
ZHX2	1.3087499
ZIC4	0.98608065
ZIC5	0.7853513
ZKSCAN2	1.9341905
ZMIZ2	0.40500355
ZMYND15	0.50298643
ZNFX1	0.7748232
ZNHIT6	0.8430691
ZNRF1	1.1627941
ZP3	1.37689
ZPR1	0.3521676
ZSCAN18	1.474529
ZSCAN26	0.38352728
ZSWIM4	1.0331421
ZSWIM5	0.8704777
ZSWIM7	0.33888483
ZSWIM8	0.58932066
ZUFSP	0.8280449
ZYG11B	0.8438854

Mice_onset_lung_DEgenes_fc

Gene	Log2 Fold Change
A1BG	-9.025497
A1CF	-8.129772
A26C2	-2.2345595
AAAS	-1.9095898
AACS	-2.1521583
AADAC	-13.358604
AADACL2	-2.193484
AADACL3	-2.066179
AADAT	-3.2825768
AAED1	-1.7279673
AAGAB	-1.177701
AAMDC	-1.7845306
AAMP	-1.666285
AANAT	-1.0991445
AAR2	-1.2071056
AARS	-2.0535097
AARS2	-1.506824
AARSD1	-1.5141568
AASDH	-0.8938384
AASDHPPT	-1.312398
AASS	-6.036329
AATF	-1.2167044
AB124611	-1.1651459
ABAT	-6.8610454
ABCA1	-1.5351415
ABCA14	-2.4935532
ABCA15	-1.798928
ABCA16	-2.496178
ABCA2	-2.1833105
ABCA3	-0.3679214
ABCA6	-6.989991
ABCA8A	-0.4063864
ABCA8B	-3.8745332
ABCA9	-0.54794
ABCB10	-1.0325403
ABCB11	-13.309639
ABCB1A	-1.0049424
ABCB4	-6.002287
ABCB5	-2.2847037
ABCB6	-4.2074585
ABCB7	-1.7845447
ABCB8	-2.0005746
ABCC10	-1.1799903

ABCC12	-2.0876913
ABCC2	-10.14431
ABCC3	-2.1922789
ABCC6	-11.013275
ABCC9	-1.7107382
ABCD1	-2.2327662
ABCD2	-3.3008637
ABCD3	-3.275691
ABCD4	-1.7991915
ABCF1	-0.3295598
ABCF2	-0.8208404
ABCF3	-1.0222206
ABCG2	-2.94903
ABCG3	-1.5342698
ABCG5	-11.504021
ABCG8	-7.3508415
ABHD1	-4.1353946
ABHD11	-2.0741086
ABHD12	-0.8912082
ABHD14A	-0.903573
ABHD14B	-3.6559434
ABHD15	-4.106105
ABHD16A	-1.8039665
ABHD17A	-2.54804
ABHD17C	-0.7275972
ABHD2	-1.1938219
ABHD4	-0.9474478
ABHD6	-3.5043247
ABHD8	-0.9643889
ABL1	-0.2677641
ABO	-2.226998
ABRA	-1.6979313
ABT1	-0.6824117
ABTB1	-0.6403608
ACAA1A	-2.4737864
ACAA1B	-6.1538754
ACAA2	-4.293739
ACACA	-2.3214831
ACACB	-5.0091662
ACAD10	-3.4625359
ACAD11	-4.9063115
ACAD12	-2.8174443
ACAD8	-0.9096601
ACAD9	-2.0155315
ACADL	-1.9374795

ACADM	-3.7630682
ACADS	-5.6085854
ACADSB	-0.5729194
ACADVL	-3.5079718
ACAT1	-4.0640054
ACAT2	-4.9434137
ACAT3	-5.6160793
ACBD4	-2.7411814
ACBD5	-1.8151002
ACBD6	-2.2658758
ACCS	-1.6891618
ACCSL	-2.0758815
ACD	-0.3786149
ACE3	-2.166792
ACHE	-1.4569798
ACLY	-2.8386254
ACMSD	-7.081786
ACN9	-1.7450922
ACNAT1	-6.41283
ACNAT2	-10.691735
ACO1	-3.1575873
ACO2	-1.89467
ACOT1	-0.7899079
ACOT12	-10.854857
ACOT13	-2.838098
ACOT3	-7.961916
ACOT4	-8.847908
ACOT5	-1.8399417
ACOT6	-2.3992722
ACOT8	-2.0753183
ACOX1	-4.867726
ACOX2	-6.2259827
ACP1	-1.6148272
ACP2	-1.1941986
ACP5	-2.3453403
ACP6	-2.5745778
ACPT	-2.8131578
ACRV1	-2.014029
ACSBG2	-2.326975
ACSF2	-0.5798245
ACSF3	-2.952835
ACSL1	-4.271555
ACSL3	-1.7706141
ACSL5	-2.6585016
ACSM1	-5.116926

ACSM2	-2.092701
ACSM3	-7.090167
ACSM4	-2.1598508
ACSM5	-9.500113
ACSS2	-5.092682
ACSS3	-1.6627524
ACTB	-0.252708
ACTBL2	-2.089941
ACTL10	-3.6825092
ACTL6A	-0.7716241
ACTL6B	-2.1206217
ACTL7A	-0.9860227
ACTL9	-2.3603473
ACTN4	-0.277051
ACTR10	-0.7939367
ACTR1B	-1.3601813
ACTR2	-0.2035017
ACTR5	-0.5446258
ACTR6	-1.6474323
ACTR8	-1.2942047
ACTRT1	-2.0640497
ACY1	-5.223765
ACY3	-5.5381722
ACYP1	-1.6780114
ACYP2	-2.2527487
ADAD1	-1.9246259
ADAD2	-3.7105653
ADAL	-1.714119
ADAM11	-1.4889913
ADAM12	-0.9341488
ADAM1B	-2.2309875
ADAM2	-1.7711849
ADAM20	-2.1138732
ADAM23	-3.4894679
ADAM25	-1.9822187
ADAM26A	-1.5786064
ADAM29	-2.0890667
ADAM30	-1.7087982
ADAM32	-2.5707266
ADAM34	-2.158697
ADAM39	-2.2246778
ADAM6A	-2.2445006
ADAM7	-1.9412832
ADAMDEC1	-2.6396365
ADAMTS13	-4.5482674

ADAMTS16	-1.8499622
ADAMTS20	-4.07976
ADAMTS7	-5.139226
ADAP2	-0.8056936
ADARB2	-2.8363612
ADAT2	-0.9759097
ADAT3	-0.5994096
ADCK1	-1.8698258
ADCK2	-1.4626894
ADCK3	-6.0449896
ADCK4	-1.2899342
ADCK5	-2.1597085
ADCY10	-2.1046064
ADCY6	-0.5396233
ADCY9	-1.3180575
ADCYAP1	-2.363018
ADH1	-6.986104
ADH4	-10.89953
ADH5	-3.3389883
ADH6A	-1.4829826
ADH7	-1.4132009
ADHFE1	-4.7114286
ADI1	-2.6878638
ADIPOR1	-0.8044252
ADIPOR2	-1.9004602
ADK	-4.0611844
ADM2	-1.6605582
ADO	-0.4975262
ADORA1	-2.974267
ADPRHL2	-1.817297
ADPRM	-0.7010427
ADRA1A	-1.2207994
ADRA1B	-3.3065972
ADRA1D	-0.700393
ADRA2A	-1.5152068
ADRA2B	-2.6283364
ADRM1	-1.174933
ADSL	-0.7141938
ADSS	-0.4208193
ADSSL1	-1.7421937
ADTRP	-5.283735
AEN	-0.6972551
AES	-1.5409579
AFF2	-1.5172911
AFG3L1	-2.1797385

AFG3L2	-2.3145742
AFM	-14.867827
AFMID	-6.5593014
AFP	-2.035723
AGA	-0.7057724
AGBL1	-2.2465804
AGBL3	-0.6884949
AGBL4	-0.6494138
AGFG2	-1.3061166
AGK	-0.370378
AGL	-2.0331993
AGMAT	-9.185667
AGMO	-4.0214124
AGPAT2	-4.475039
AGPAT3	-2.4561129
AGPAT6	-2.0042553
AGPAT9	-2.1275096
AGT	-9.407713
AGTR1A	-3.4868255
AGTR1B	-2.2018514
AGTR2	-2.4283214
AGXT	-14.140779
AGXT2	-10.242956
AHCTF1	-0.4574661
AHCY	-6.0869937
AHCYL1	-1.38236
AHR	-1.6380043
AHRR	-2.5301244
AHSA1	-0.3085585
AHSG	-11.353382
AIF1	-1.4380975
AIFM1	-2.7241426
AIFM2	-2.6927238
AIFM3	-2.5084043
AIG1	-1.5668883
AIM1	-0.6732388
AIM1L	-1.4789836
AIMP1	-0.685811
AIMP2	-0.5833483
AIP	-0.4166875
AIRE	-0.4196343
AK2	-2.419765
AK3	-3.2243052
AK4	-6.2751884
AKAP1	-3.1875243

AKAP17B	-0.7599621
AKAP4	-1.6317413
AKAP8L	-0.6531816
AKIP1	-1.3366027
AKR1A1	-1.5911136
AKR1B10	-0.4845815
AKR1B7	-7.0724344
AKR1C12	-7.360159
AKR1C13	-5.3682814
AKR1C14	-1.4554858
AKR1C18	-8.293886
AKR1C19	-7.3270974
AKR1C20	-11.86227
AKR1C21	-2.0782824
AKR1C6	-13.391624
AKR1CL	-2.1202557
AKR1D1	-9.199589
AKR1E1	-2.2293289
AKR7A5	-4.7883673
AKT1	-0.4571014
AKT1S1	-2.0615094
AKT2	-0.9488912
ALAD	-3.979045
ALAS1	-1.6460686
ALB	-11.30703
ALDH16A1	-2.4344382
ALDH18A1	-0.2915041
ALDH1A1	-0.6410604
ALDH1A7	-1.122325
ALDH1B1	-6.783326
ALDH1L1	-6.809845
ALDH2	-2.7970967
ALDH3A2	-2.040347
ALDH4A1	-5.4132404
ALDH5A1	-4.3133616
ALDH6A1	-3.3701577
ALDH7A1	-4.4610963
ALDH8A1	-9.735807
ALDH9A1	-3.9985595
ALDOART1	-2.0757823
ALDOB	-14.093827
ALDOC	-2.4989314
ALG1	-0.2808924
ALG13	-0.8322864
ALG14	-1.1578689

ALG2	-0.6013641
ALG3	-2.06679
ALG5	-2.404819
ALG6	-2.1802351
ALG8	-1.4443767
ALG9	-1.4975567
ALK	-2.3430316
ALKBH2	-2.0805078
ALKBH3	-1.2034826
ALKBH4	-0.5429311
ALKBH5	-0.6960287
ALKBH6	-0.3835473
ALKBH7	-2.0410953
ALLC	-2.070932
ALOX12B	-0.5467179
ALOX8	-2.1757193
ALPI	-2.0355175
ALPK2	-2.272282
ALPL	-1.174027
ALS2	-2.016651
ALS2CL	-0.6081452
ALS2CR11	-2.125108
ALX1	-2.2715356
ALX4	-1.0264318
AMACR	-5.4005837
AMBN	-2.1196272
AMBP	-9.441347
AMBRA1	-0.4969754
AMD2	-0.2883258
AMDHD1	-10.044319
AMDHD2	-1.3002057
AMELX	-2.0924494
AMER2	-1.9753039
AMFR	-1.6132526
AMH	-1.5381241
AMIGO1	-0.9192653
AMIGO2	-1.239548
AMMECR1L	-0.8995895
AMN	-2.7063618
AMOT	-2.1559439
AMPD2	-1.3388395
AMT	-4.1398044
AMTN	-2.011379
AMY1	-7.1803455
AMY2A4	-2.265697

AMY2A5	-6.7712336
AMZ2	-0.4338117
ANAPC1	-0.3651104
ANAPC13	-1.2587004
ANAPC15	-0.3126917
ANAPC16	-0.4859386
ANAPC4	-0.2636533
ANAPC5	-0.6531181
ANAPC7	-1.2596793
ANG	-5.1954813
ANG2	-5.252739
ANG3	-1.0234084
ANG4	-4.406863
ANGEL1	-1.531734
ANGEL2	-0.2059059
ANGPTL1	-0.7814949
ANGPTL3	-10.658822
ANGPTL4	-0.2022672
ANGPTL6	-5.308263
ANK	-2.3227444
ANK2	-0.1636839
ANKAR	-2.3203213
ANKDD1A	-1.9626613
ANKDD1B	-2.0509217
ANKLE1	-1.1106961
ANKMY1	-0.766393
ANKMY2	-1.03001
ANKRD13B	-0.9931054
ANKRD13C	-0.2371006
ANKRD2	-0.4706078
ANKRD24	-2.7928247
ANKRD27	-0.2520771
ANKRD28	-0.6700869
ANKRD33	-1.2152898
ANKRD34B	-2.1755211
ANKRD34C	-2.2895646
ANKRD36	-2.1929147
ANKRD39	-1.0426745
ANKRD46	-1.086782
ANKRD52	-0.4870515
ANKRD54	-0.6440446
ANKRD55	-1.5743821
ANKRD60	-0.4605522
ANKRD9	-0.8058944
ANKS1B	-1.76684

ANKS3	-0.4387491
ANKS4B	-5.813467
ANKUB1	-0.8842807
ANKZF1	-1.3681331
ANO10	-0.7559576
ANO3	-1.9316797
ANO5	-2.2382581
ANO7	-2.3183055
ANO8	-0.3921535
ANP32A	-1.5859828
ANPEP	-2.3413453
ANTXR2	-0.2938619
ANXA10	-2.2921674
ANXA13	-2.2161357
ANXA6	-0.8334456
ANXA7	-0.4603739
AOAH	-0.3371637
AOX1	-1.7402987
AOX2	-1.9572678
AOX3	-1.7629223
AOX4	-2.0132923
AP1AR	-0.4285188
AP1B1	-1.1631694
AP1G1	-0.5700193
AP1M1	-0.6659713
AP1S1	-1.1377788
AP2A1	-0.7200828
AP2A2	-0.9504905
AP2B1	-0.673686
AP2M1	-1.3119235
AP2S1	-1.2386961
AP3D1	-0.2230215
AP3M1	-2.077464
AP3S1	-0.1990705
AP3S2	-0.7283988
AP4B1	-1.2665286
AP4M1	-0.650486
AP5M1	-0.4756985
AP5S1	-2.2292628
APBA3	-1.9734373
APBB3	-1.1270089
APC	-1.1168983
APCS	-12.718416
APEH	-1.9209871
APELA	-0.915225

APEX1	-2.1285143
APH1A	-1.4146476
API5	-0.416687
APIP	-0.754477
APLP2	-0.4926748
APMAP	-1.479095
APOA1	-15.063332
APOA1BP	-1.7764707
APOA2	-6.9850574
APOA4	-10.10235
APOA5	-10.828673
APOB	-9.749285
APOBEC1	-0.6723561
APOBEC3	-0.3069058
APOC1	-4.765407
APOC2	-7.155035
APOC3	-11.485372
APOC4	-14.223697
APOE	-3.8837824
APOF	-13.646185
APOH	-13.819852
APOL10A	-1.3176134
APOL7A	-8.016025
APOL7C	-3.6246715
APOL7D	-2.1051474
APOL8	-1.5285244
APOL9A	-4.8123975
APOL9B	-4.6296744
APOM	-12.868542
APON	-11.680189
APOO	-1.7688694
APOOL	-1.6003976
APOPT1	-0.8583922
APRT	-1.2989192
AQP1	-0.6727085
AQP11	-7.549788
AQP12	-1.1802406
AQP2	-0.2657881
AQP6	-2.0328236
AQP8	-12.32449
AQP9	-7.170496
ARAF	-1.2595987
ARAP1	-1.0210962
ARCN1	-0.4359193
AREL1	-1.0630622

ARF1	-0.8303409
ARF4	-0.9492588
ARF5	-1.1881518
ARF6	-0.8437572
ARFGAP1	-0.5224743
ARFGAP2	-2.2477398
ARFGEF2	-0.9574928
ARFIP1	-0.2669618
ARFRP1	-0.2595024
ARG1	-11.39661
ARHGAP1	-0.3015027
ARHGAP19	-1.2247834
ARHGAP20OS	-2.0215058
ARHGAP21	-0.4029245
ARHGAP24	-1.1673298
ARHGAP26	-0.465055
ARHGAP35	-0.870966
ARHGAP36	-1.5653815
ARHGAP42	-1.0818594
ARHGAP5	-0.4345694
ARHGEF10	-0.982615
ARHGEF10L	-0.9869986
ARHGEF15	-1.6750484
ARHGEF16	-0.8120418
ARHGEF18	-0.4002724
ARHGEF19	-4.439184
ARHGEF37	-1.2078507
ARHGEF39	-1.0536985
ARHGEF40	-0.8035274
ARHGEF9	-1.9350796
ARID3C	-1.754497
ARIH2	-1.0093684
ARL1	-0.8724499
ARL13A	-1.5218389
ARL14EPL	-3.1990788
ARL4A	-0.7268438
ARL4AOS	-2.2884722
ARL4C	-0.3883371
ARL4D	-0.9516282
ARL5A	-1.3450704
ARL6IP4	-0.9648337
ARL6IP6	-1.3112073
ARL8B	-0.2840447
ARL9	-1.5526583
ARMC1	-1.3064756

ARMC12	-2.7631373
ARMC6	-0.8297062
ARMC8	-0.3839541
ARMC9	-2.1669428
ARMCX5	-0.5491443
ARNT	-0.3282538
ARNTL	-0.4114771
ARNTL2	-0.7736294
ARPC1A	-0.5648828
ARPC3	-0.2813611
ARPC4	-1.0779223
ARPC5L	-0.6006661
ARPP21	-1.3972666
ARSB	-0.4409761
ARSG	-2.8386207
ARSK	-0.9607878
ART2A-PS	-5.990324
ARV1	-0.6850045
ARVCF	-1.8968391
ARX	-2.1528044
AS3MT	-3.0162497
ASAH1	-0.6743107
ASAH2	-0.8855045
ASAP3	-0.3084903
ASB12	-2.053315
ASB13	-1.9752474
ASB15	-2.259184
ASB16	-2.6504774
ASB17	-2.1391938
ASB18	-1.5364733
ASB8	-0.8921208
ASCC1	-1.3178759
ASGR1	-9.002436
ASGR2	-8.747219
ASH2L	-0.1515307
ASIC2	-2.2292838
ASIC4	-0.7773817
ASL	-6.18895
ASNA1	-0.1525345
ASNSD1	-1.2731671
ASPA	-3.4045722
ASPDH	-4.6360955
ASPG	-4.940985
ASPM	-0.5497971
ASPSCR1	-2.305139

ASS1	-5.205804
ASTE1	-1.8549981
ASTN2	-0.6280673
ASXL3	-1.4860861
ASZ1	-1.9991026
ATAD1	-0.4716134
ATAD3A	-2.4464998
ATAD3AOS	-2.6337707
ATAT1	-1.0140014
ATCAY	-1.9166753
ATCAYOS	-1.521327
ATE1	-0.3912025
ATF1	-0.6130076
ATF5	-3.1948829
ATF6B	-0.6813655
ATF7IP2	-2.2117434
ATG10	-0.3731096
ATG101	-1.580625
ATG12	-0.4182811
ATG13	-0.5663557
ATG14	-0.7290454
ATG16L1	-0.9812827
ATG16L2	-0.6239281
ATG2A	-2.2245555
ATG2B	-0.7628748
ATG3	-0.53373
ATG4A	-1.7818213
ATG4B	-1.664135
ATG4C	-2.1538234
ATG4D	-1.3659458
ATG5	-2.2823882
ATG7	-1.2043619
ATG9B	-0.7983308
ATHL1	-1.553401
ATIC	-1.1150241
ATL2	-2.7117157
ATM	-0.2833917
ATOH1	-2.106205
ATOH7	-3.7507486
ATOH8	-1.3660922
ATOX1	-1.7269158
ATP10B	-1.8880534
ATP11B	-0.5809245
ATP11C	-2.8417988
ATP12A	-1.0610511

ATP13A1	-1.2259073
ATP13A2	-1.1049051
ATP13A5	-1.4584007
ATP1A1	-0.7480478
ATP1B3	-0.2803378
ATP1B4	-2.0970113
ATP2B2	-2.4133224
ATP2B3	-1.9914048
ATP4A	-2.322646
ATP4B	-1.9213839
ATP5A1	-1.8401251
ATP5B	-2.0634532
ATP5C1	-2.5999737
ATP5C1-PS	-1.9066348
ATP5D	-3.0046983
ATP5E	-1.9901028
ATP5F1	-1.9943008
ATP5G1	-2.691863
ATP5G2	-1.4842987
ATP5G3	-2.240652
ATP5H	-1.4369488
ATP5J	-1.8743229
ATP5J2	-1.9802179
ATP5K	-2.1863575
ATP5L	-1.6394272
ATP5O	-1.9573889
ATP5S	-1.3123441
ATP5SL	-2.1884775
ATP6	-2.2424197
ATP6AP1L	-1.9554148
ATP6V0A1	-0.1905952
ATP6V0B	-1.4915876
ATP6V0C	-1.0554256
ATP6V0D1	-0.9902496
ATP6V1C1	-0.2558899
ATP6V1D	-0.654582
ATP6V1E1	-1.6673999
ATP6V1E2	-0.8772154
ATP6V1F	-0.7568269
ATP6V1G3	-1.6338692
ATP6V1H	-0.6525822
ATP8	-2.4253254
ATP8B1	-1.7127657
ATP8B2	-2.2485917
ATP8B3	-0.6113305

ATP8B5	-0.9291327
ATP9A	-0.921824
ATPAF2	-1.9872794
ATPIF1	-0.8430858
ATRAID	-2.3149247
ATRIP	-1.0232162
ATRN	-0.8318467
ATXN10	-0.2929916
ATXN2	-1.0817637
ATXN3	-0.4722803
ATXN7	-1.4097395
ATXN7L3B	-1.0377421
AUH	-1.7571812
AUP1	-1.9260483
AURKA	-1.1352034
AURKAIP1	-1.3150883
AURKB	-2.2544742
AVEN	-1.2195811
AVP	-2.456004
AVPI1	-1.4048214
AVPR1A	-7.271783
AVPR1B	-1.703824
AXL	-0.8681684
AZGP1	-10.090959
B2M	-1.2868428
B3GALT1	-4.1492043
B3GALT4	-0.2094784
B3GALT5	-1.1186116
B3GALT6	-0.2794538
B3GAT1	-2.008205
B3GAT3	-1.102879
B3GNT1	-1.3978872
B3GNT8	-0.3888841
B3GNTL1	-1.3288355
B4GALNT1	-0.6821609
B4GALNT3	-1.6561284
B4GALNT4	-0.8401179
B4GALT7	-0.4996114
BAALC	-2.2325768
BAAT	-9.492987
BABAM1	-1.3339243
BACE1	-1.5894456
BAD	-1.1330762
BAG1	-0.339869
BAG4	-1.9519303

BAG5	-0.1965108
BAG6	-0.7531366
BAHCC1	-2.1145337
BAI1	-1.1638865
BAIAP2	-1.1236973
BAIAP2L1	-1.322753
BAIAP3	-1.3998849
BAMBI	-2.1184416
BANF1	-1.3409028
BANF2	-2.4299226
BANK1	-1.2890697
BANP	-1.1495991
BAP1	-1.1020699
BARHL2	-1.9078045
BARX1	-1.899591
BATF3	-1.5141182
BAZ2A	-0.39396
BBC3	-1.1407928
BBOX1	-7.794569
BBS10	-2.941379
BBS5	-1.4381833
BBS7	-0.6341021
BC025920	-0.979106
BCAP29	-0.4375515
BCAP31	-1.7265425
BCAS1OS2	-1.7092607
BCAS2	-0.702693
BCAS3	-0.5840292
BCAT2	-0.9513087
BCCIP	-1.3382058
BCDIN3D	-0.994411
BCHE	-6.1017017
BCKDHA	-4.638197
BCKDHB	-4.1714454
BCKDK	-0.8327251
BCL2L12	-1.5362949
BCL2L13	-1.2261953
BCL7A	-0.3849948
BCL7C	-0.4205074
BCL9L	-1.1151361
BCMO1	-5.2468553
BCO2	-4.7519827
BCORL1	-0.6401041
BCS1L	-1.8061237
BDH1	-7.6888514

BDH2	-4.4828773
BECN2	-2.0079327
BEND3	-0.3390074
BEND6	-0.2621663
BEST1	-2.1232858
BEST3	-1.5296371
BET1	-1.4578652
BET1L	-0.2293797
BEX6	-1.2175674
BGLAP	-3.406914
BGLAP2	-1.4640732
BGLAP3	-1.7586584
BHLHA9	-0.9952493
BHLHE23	-1.045712
BHLHE40	-0.951478
BHMT	-14.096851
BHMT2	-12.809559
BID	-0.6597877
BIN3	-0.2241554
BIRC5	-0.5180049
BIRC6	-0.3879385
BIVM	-1.6311498
BLCAP	-1.4557457
BLK	-1.916945
BLM	-0.6522675
BLMH	-0.918457
BLOC1S1	-1.2506251
BLOC1S2	-1.3259759
BLOC1S3	-0.8456652
BLOC1S4	-0.6257958
BLOC1S5	-2.7377071
BLOC1S6	-0.2778845
BLVRA	-0.754549
BLVRB	-3.4884138
BMP1	-2.4215136
BMP10	-2.258269
BMP15	-2.0257971
BMP2	-2.961372
BMP7	-0.8863125
BMP8B	-1.49259
BNIP1	-2.2410307
BNIP3	-1.8822846
BOD1	-1.2999692
BOK	-1.1157508
BOLA1	-0.5972066

BOLA2	-0.8410459
BOLA3	-2.157539
BOLL	-0.7626009
BOP1	-0.7846127
BPGM	-0.6759753
BPHL	-3.7547417
BPI	-2.07131
BPIFA2	-2.1718032
BPIFA3	-1.3151271
BPIFA6	-1.9146376
BPIFB2	-1.1509933
BPIFB3	-2.1164143
BPIFB9B	-3.962109
BPIFC	-1.1344228
BPNT1	-1.8787174
BRAP	-2.1021948
BRAT1	-0.7213798
BRCA1	-2.5511136
BRCA2	-1.2941027
BRCC3	-0.4028039
BRD3	-1.2098103
BRE	-0.7095485
BRF1	-0.2372532
BRF2	-1.0542526
BRI3	-0.5922251
BRI3BP	-1.6666465
BRICD5	-1.4550841
BRINP2	-2.185319
BRINP3	-2.355568
BRIP1	-0.798415
BRIP1OS	-2.1757061
BRK1	-0.7548385
BRMS1	-1.031579
BRS3	-1.9902008
BSCL2	-2.010933
BSDC1	-0.8768802
BSG	-1.9671955
BSN	-0.3513315
BSPH1	-0.7182457
BST2	-1.2089496
BSX	-2.1862662
BTBD16	-0.5626555
BTBD2	-1.484251
BTBD6	-1.9771657
BTBD8	-1.3887501

BTBD9	-0.462482
BTC	-1.3703423
BTD	-3.3889875
BTF3L4	-0.5105214
BTG4	-2.1417139
BTN1A1	-1.8645554
BTN2A2	-2.0758774
BTNL1	-2.0843792
BTNL2	-4.269161
BTNL4	-2.0309181
BUB1B	-0.5095618
BUD31	-0.3919563
BYSL	-0.5238595
BZW1	-0.4026923
C1GALT1C1	-0.3881555
C1QA	-2.3300638
C1QB	-2.2579298
C1QBP	-1.5704875
C1QC	-2.653265
C1QL3	-4.5013123
C1QTNF1	-1.4000764
C1QTNF9	-2.0791447
C1RA	-1.8811741
C1RB	-1.459105
C1RL	-4.007403
C1S1	-1.8921156
C1S2	-1.7225461
C2	-2.9133568
C2CD2	-2.5562074
C2CD2L	-1.8373837
C2CD4D	-2.5190868
C3	-2.1411357
C4B	-3.180214
C4BP	-12.319572
C5AR2	-1.2354064
C6	-1.1189003
C8A	-10.873709
C8B	-10.797932
C8G	-10.054869
C9	-8.941826
CAB39L	-1.8400984
CABIN1	-0.3831439
CABLES2	-1.8030486
CABP2	-1.6219854
CABP4	-0.6095519

CABP7	-2.2486877
CABS1	-2.1173599
CABYR	-0.7094395
CACFD1	-1.6464677
CACNA1A	-0.9821613
CACNA1B	-3.8505638
CACNA1E	-2.1808026
CACNA1F	-0.9996076
CACNB4	-1.5552804
CACNG1	-1.9611881
CACNG2	-1.4702523
CACNG3	-1.7075732
CACNG4	-0.8627639
CACNG5	-2.2013202
CACNG7	-3.8679912
CACNG8	-2.2921903
CADM3	-3.7046542
CAGE1	-0.7522571
CALB1	-1.5257843
CALB2	-2.5660958
CALCB	-1.721581
CALD1	-1.0636134
CALHM1	-0.9649303
CALHM2	-0.5630603
CALM2	-0.2233963
CALM5	-2.1975422
CALML4	-1.3678727
CALN1	-0.4112411
CALR	-1.1685467
CALR3	-2.7988796
CALU	-0.3031783
CAMK1	-0.6316247
CAMK1D	-3.3231554
CAMK2N1	-0.4204497
CAMK2N2	-0.5314841
CAML	-0.6758671
CAMSAP3	-0.9139824
CAND1	-0.5241842
CAND2	-1.2318401
CANX	-0.3095756
CAPN10	-3.2387648
CAPN12	-1.7172067
CAPN15	-0.8574958
CAPN5	-0.7304726
CAPN6	-2.602324

CAPRIN1	-0.9003325
CAPZA1	-0.8702989
CAPZA3	-1.6954236
CAR1	-3.4011548
CAR10	-1.8811178
CAR11	-1.1625698
CAR14	-5.2080317
CAR2	-0.6599536
CAR3	-4.9416485
CAR5A	-10.940637
CAR6	-3.2574291
CAR7	-2.9570088
CARD10	-1.4478879
CARD14	-1.3456154
CARHSP1	-0.3777118
CARKD	-0.9477577
CARLR	-2.2809687
CARM1	-1.1724396
CARNS1	-0.766722
CARS	-0.7726798
CARS2	-1.0460548
CASKIN1	-1.6536622
CASP6	-1.7149844
CASP7	-1.1235352
CASP8	-0.5433979
CASP9	-0.9044089
CASR	-2.2434695
CAT	-3.9614358
CATSPER1	-1.599226
CATSPER2	-1.8734374
CATSPER3	-1.4825068
CATSPERB	-2.1747644
CATSPERD	-2.2749856
CATSPERG2	-1.4705296
CBLC	-3.4790497
CBLN1	-1.7284567
CBLN2	-1.2687697
CBLN4	-2.1803808
CBR1	-4.1255307
CBR4	-2.8062596
CBS	-7.486018
CBX2	-3.2458367
CBX3	-0.2306929
CBX4	-0.5399809
CBX7	-0.9219999

CBX8	-3.11421
CBY1	-1.0197024
CC2D1A	-0.720798
CC2D2B	-1.6484287
CCAR2	-0.6517963
CCBL1	-3.8657537
CCBL2	-5.7512455
CCDC101	-0.2511706
CCDC104	-0.7495456
CCDC106	-1.0896065
CCDC107	-2.097056
CCDC12	-0.5706563
CCDC124	-0.7184072
CCDC125	-1.2116427
CCDC137	-0.5498366
CCDC138	-1.7894549
CCDC142	-1.057807
CCDC148	-2.0469975
CCDC152	-0.8403702
CCDC154	-0.3679352
CCDC159	-2.4564495
CCDC162	-2.174866
CCDC166	-2.2574096
CCDC167	-1.8691635
CCDC171	-0.8658042
CCDC178	-2.2356286
CCDC18	-0.4650829
CCDC183	-1.5018563
CCDC22	-0.6962938
CCDC23	-0.5463839
CCDC25	-1.2395363
CCDC27	-1.8742855
CCDC28A	-1.2270932
CCDC28B	-1.0132346
CCDC3	-2.5128152
CCDC32	-0.2967372
CCDC34	-0.3228018
CCDC36	-1.0165422
CCDC38	-2.1276915
CCDC42OS	-2.5619667
CCDC43	-0.7473674
CCDC47	-0.460186
CCDC51	-1.80158
CCDC53	-0.8989949
CCDC54	-2.0675354

CCDC57	-0.2706008
CCDC58	-2.3850389
CCDC60	-0.525012
CCDC7	-2.4270718
CCDC70	-1.5340507
CCDC71	-0.9320235
CCDC73	-1.8838279
CCDC74A	-1.9650874
CCDC79	-0.9091654
CCDC8	-1.6205246
CCDC80	-0.9441524
CCDC83	-1.9448755
CCDC84	-0.4610872
CCDC85C	-0.6232657
CCDC90B	-0.584929
CCDC91	-0.5984359
CCDC94	-0.3157733
CCDC97	-0.5371304
CCER1	-1.9868684
CCIN	-3.6565466
CCKBR	-1.593384
CCL20	-2.372761
CCL22	-1.7142234
CCL24	-5.173886
CCL25	-0.808423
CCL26	-1.7533712
CCL27A	-0.85356
CCL28	-1.0390053
CCL9	-2.2414966
CCM2	-0.8273153
CCNA2	-0.8252721
CCNB1	-0.7928746
CCNB1IP1	-1.3292758
CCNB2	-0.4594431
CCNB3	-2.2177856
CCNE1	-1.2755094
CCNE2	-2.3685284
CCNF	-1.340447
CCNH	-1.1094508
CCNI	-0.6249809
CCNL2	-0.2978368
CCNY	-0.2088738
CCR1	-2.0660386
CCR3	-2.7266738
CCS	-3.7085152

CCT2	-0.5064383
CCT3	-0.5858183
CCT5	-0.416348
CCT6A	-0.4090438
CCT6B	-0.8290119
CCT7	-0.866869
CCT8	-0.3371167
CCZ1	-0.575295
CD164	-1.1480756
CD180	-1.2656684
CD1D1	-5.9054136
CD1D2	-5.934004
CD200R1	-0.4010408
CD200R2	-1.2853544
CD200R3	-1.1213665
CD209B	-0.679261
CD22	-1.28402
CD248	-0.4465442
CD276	-1.7441049
CD2AP	-0.6793447
CD300A	-0.2639341
CD300E	-2.4335713
CD300LB	-1.5282197
CD300LD	-1.9138403
CD300LG	-1.2031593
CD300LH	-1.2962723
CD302	-2.6918287
CD320	-0.8336434
CD38	-0.7381492
CD4	-3.4767103
CD40LG	-2.1099732
CD59A	-2.9285746
CD59B	-5.008132
CD5L	-8.776566
CD68	-0.2403617
CD7	-0.6588984
CD74	-0.8336225
CD81	-1.93184
CD86	-0.5462451
CDA	-6.5378246
CDADC1	-1.1627908
CDC123	-0.8394122
CDC14B	-2.151031
CDC16	-0.821465
CDC20	-1.3479285

CDC25A	-0.5942345
CDC26	-0.8497701
CDC34	-1.5437741
CDC37	-0.4982772
CDC37L1	-0.4190431
CDCA2	-0.6395683
CDCA3	-1.758338
CDCA5	-0.7521935
CDCA7L	-0.4980967
CDCP2	-6.8134413
CDH17	-1.684382
CDH19	-1.623101
CDH2	-4.777109
CDH20	-2.1066413
CDH26	-0.5051768
CDH3	-2.1428802
CDH7	-1.8337684
CDH8	-1.1921518
CDH9	-1.7912602
CDHR2	-1.2433405
CDHR5	-8.214988
CDIP1	-2.2839859
CDIPT	-0.4910779
CDK1	-1.251827
CDK10	-1.4157014
CDK15	-1.494719
CDK16	-0.8766012
CDK18	-1.8331962
CDK20	-1.6498332
CDK2AP1	-0.7158432
CDK2AP2	-0.8490653
CDK4	-0.4649243
CDK5RAP1	-1.4727278
CDK5RAP3	-2.406858
CDK9	-0.5096693
CDKAL1	-0.3800206
CDKN2A	-2.104287
CDKN2AIP	-0.5004931
CDKN2AIPNL	-0.5550675
CDKN2C	-1.7295403
CDO1	-4.202666
CDRT4	-1.2810829
CDRT4OS1	-1.479784
CDS2	-1.6702156
CDT1	-0.7145667

CDV3	-0.7496972
CDX1	-1.9215877
CDX2	-2.170406
CDX4	-3.5663822
CEACAM11	-1.9585505
CEACAM12	-2.1602802
CEACAM13	-2.735763
CEACAM14	-1.9794958
CEACAM15	-2.0487688
CEACAM19	-2.3412404
CEACAM2	-0.9671836
CEACAM20	-0.7243049
CEACAM3	-1.1018701
CEACAM5	-1.6382306
CEBPA	-2.0900102
CEBPZOS	-1.9603524
CELA1	-1.8043404
CELA2A	-2.4375176
CELF3	-1.3518255
CELRR	-1.00917
CELSR1	-0.7894449
CEND1	-1.5101411
CENPA	-1.218482
CENPH	-0.920264
CENPK	-1.2854493
CENPL	-2.0747523
CENPM	-3.2036898
CENPN	-0.5051911
CENPO	-0.4929166
CENPP	-1.5015891
CENPQ	-0.2548871
CENPV	-2.243291
CENPW	-1.277278
CEP131	-1.2101564
CEP152	-0.505167
CEP170B	-1.7348657
CEP19	-0.1718373
CEP44	-0.9222374
CEP57L1	-1.1408112
CEP63	-0.1788664
CEP68	-1.4949555
CEP72	-2.7423806
CEP89	-0.2596026
CEP97	-0.5341082
CEPT1	-0.5152292

CER1	-2.0958607
CERKL	-2.6383953
CERS2	-2.0772963
CES1A	-2.8298576
CES1B	-12.434908
CES1C	-14.120815
CES1D	-3.3169494
CES1E	-5.3021407
CES1F	-5.7375975
CES1G	-5.692471
CES1H	-1.5136657
CES2A	-13.395618
CES2B	-5.275049
CES2C	-8.037493
CES2E	-9.484403
CES2F	-2.1798284
CES2G	-4.9301805
CES2H	-2.7276483
CES3A	-13.465191
CES3B	-10.495478
CES4A	-3.475373
CES5A	-2.1814377
CFB	-2.8997488
CFC1	-2.1146939
CFDP1	-0.3591385
CFH	-2.9597259
CFHR1	-11.5137
CFHR2	-4.117684
CFI	-10.457387
CFL2	-1.0631495
CFP	-2.1547556
CGREF1	-2.8683805
CGRRF1	-1.1654
CHAC2	-3.6073437
CHADL	-1.4138944
CHAF1A	-0.6949782
CHAF1B	-0.9708414
CHCHD1	-1.1159043
CHCHD10	-3.3328767
CHCHD2	-1.6139693
CHCHD3	-2.0915966
CHCHD4	-0.5435457
CHCHD5	-1.7175045
CHCHD6	-0.4531469
CHCHD7	-1.5820937

CHD1L	-0.775425
CHD3	-0.8127418
CHD4	-0.2795844
CHD9	-0.6671333
CHDH	-5.8826475
CHEK2	-1.1164019
CHID1	-1.7873528
CHIL6	-1.7776048
CHKA	-1.4634829
CHKB	-1.6007648
CHMP1A	-1.4851689
CHMP2A	-0.4941006
CHMP5	-0.3349237
CHMP6	-1.169765
CHMP7	-1.1791382
CHN1OS3	-2.2463672
CHN2	-3.7482498
CHODL	-2.4514318
CHP1	-1.5745482
CHP2	-3.5306363
CHPF2	-1.652945
CHPT1	-1.6020117
CHRAC1	-0.5584383
CHRD	-0.575099
CHRM5	-1.649349
CHRNA1	-1.1639938
CHRNA2	-2.5310717
CHRNA4	-2.6484642
CHRNA5	-2.28171
CHRNA9	-0.8671835
CHRNB2	-1.4373548
CHRNB3	-1.7489095
CHRND	-3.154769
CHRNE	-2.137851
CHRNG	-5.601495
CHST13	-4.3880005
CHST4	-0.3130693
CHST7	-0.250864
CHST9	-2.6668785
CHSY3	-1.6973879
CHTF18	-0.920655
CHUK	-1.9631639
CHURC1	-1.6745458
CIAO1	-1.7118864
CIAPIN1	-1.5463662

CIART	-1.1794531
CIB1	-2.3053155
CIB3	-3.1000557
CIDEB	-8.696653
CINP	-0.837327
CIPC	-0.6660929
CIRBP	-1.4717388
CIRH1A	-0.8299508
CISD1	-4.1876874
CISD2	-0.8370051
CISD3	-3.2505097
CITED4	-0.2188439
CKAP2	-1.9768779
CKB	-0.3695808
CKS1B	-0.5765243
CLCA2	-1.8930156
CLCA4	-2.071556
CLCA6	-1.9902692
CLCC1	-0.7922683
CLCN2	-5.6806746
CLCN3	-1.0472775
CLCN4-2	-1.1749754
CLCN7	-1.4501195
CLDN1	-4.0188527
CLDN11	-1.1576293
CLDN12	-2.3595843
CLDN14	-5.3874407
CLDN17	-1.8459129
CLDN2	-5.834505
CLDN3	-1.4437895
CLDND2	-1.1406958
CLEC11A	-1.881288
CLEC14A	-0.3947535
CLEC16A	-0.7527835
CLEC18A	-1.8600717
CLEC1B	-2.7866454
CLEC2E	-2.3944602
CLEC2F	-2.2189279
CLEC2G	-2.2526753
CLEC2H	-5.861473
CLEC3A	-1.9142067
CLEC4B2	-2.181985
CLEC4F	-11.494522
CLEC4G	-9.234663
CLEC7A	-0.7776103

CLHC1	-0.7245703
CLK3	-0.1842904
CLMN	-2.742022
CLN3	-0.610795
CLN5	-0.6989312
CLN6	-0.7951183
CLN8	-1.9462361
CLNKOS	-2.1218107
CLNS1A	-0.8749824
CLP1	-1.1388102
CLPB	-2.491344
CLPP	-2.1698227
CLPSL2	-0.651372
CLPTM1	-1.9563284
CLPTM1L	-0.8528051
CLPX	-3.6939282
CLRN3	-1.5760846
CLSPN	-0.2995532
CLSTN3	-2.826807
CLTA	-0.6870904
CLTC	-0.7308149
CLU	-3.878838
CLUH	-3.8732152
CLVS1	-0.6795607
CLVS2	-2.0721571
CLYBL	-4.233529
CMAH	-0.590035
CMAS	-1.0837855
CMBL	-5.7470984
CMC1	-1.8874974
CMC2	-2.1512384
CML1	-7.1367426
CML2	-7.9048853
CML5	-1.5547786
CMPK1	-0.7355022
CMSS1	-0.9972434
CMTM2B	-2.0822272
CMTM8	-2.1335
CMTR2	-0.2562232
CNBD1	-2.2760847
CNBP	-0.7829175
CNDP1	-2.084868
CNDP2	-1.4307275
CNGA4	-2.2648432
CNGB3	-2.3184843

CNIH1	-1.0473943
CNIH3	-2.7398121
CNN3	-0.3080692
CNNM1	-1.87426
CNNM2	-0.209322
CNNM3	-1.6023421
CNOT1	-0.9117312
CNOT10	-0.506515
CNOT11	-0.3611155
CNOT3	-1.109462
CNOT8	-1.0280638
CNP	-1.7639422
CNPPD1	-0.3295279
CNPY1	-2.59126
CNPY2	-1.9988861
CNPY3	-1.3296099
CNST	-0.5874863
CNTD1	-2.3487446
CNTFR	-0.8097835
CNTN2	-2.4943552
CNTN3	-1.8777449
CNTN4	-0.8199525
CNTN5	-1.7453463
CNTN6	-3.327578
CNTNAP1	-1.98452
CNTNAP3	-2.1670566
CNTNAP4	-2.2446904
CNTNAP5C	-1.6895998
CNTROB	-1.1584713
COA3	-1.4188199
COA4	-1.5652733
COA5	-1.500843
COA6	-2.6782627
COA7	-3.3669076
COASY	-3.4099183
COBLL1	-0.7520919
COCH	-2.0771704
COG2	-0.8801951
COG4	-1.4628935
COG6	-0.5279107
COG7	-0.3163056
COG8	-1.322972
COIL	-0.2833285
COL11A1	-2.1219451
COL14A1	-2.08185

COL17A1	-1.171634
COL18A1	-4.726365
COL19A1	-1.5248625
COL20A1	-0.7848125
COL22A1	-2.8849337
COL24A1	-1.1928177
COL25A1	-1.8722694
COL27A1	-1.5857923
COL28A1	-2.8148375
COL5A3	-1.5533321
COL6A5	-1.1872635
COL6A6	-1.3148243
COL9A2	-0.8921249
COL9A3	-1.5509887
COLEC10	-8.306814
COLEC11	-7.098563
COMMD1	-2.055056
COMMD10	-0.5150433
COMMD2	-1.5660753
COMMD3	-2.4563317
COMMD4	-0.9862037
COMMD5	-2.123796
COMMD6	-1.5276179
COMMD7	-0.7118058
COMMD8	-0.3887644
COMMD9	-2.278276
COMT	-3.6605268
COMTD1	-0.9263058
COPA	-0.8103409
COPB1	-0.7648454
COPB2	-1.2204204
COPE	-1.9062691
COPG1	-0.2345276
COPG2	-1.0060506
COPRS	-0.910182
COPS2	-0.2783594
COPS3	-1.5702691
COPS5	-1.6465769
COPS6	-2.481573
COPS7A	-0.8930507
COPS7B	-0.8144555
COPS8	-0.3403449
COPZ1	-1.2753406
COPZ2	-2.699716
COQ10A	-1.7556334

COQ2	-1.2465796
COQ3	-2.3084264
COQ4	-2.004696
COQ5	-1.7772741
COQ6	-1.7403574
COQ7	-1.9798012
COQ9	-3.5513487
CORIN	-2.0638764
CORO1B	-0.9949532
CORO6	-0.6930733
CORO7	-1.1372957
COX1	-1.3396134
COX10	-1.4332683
COX11	-1.5136585
COX14	-0.4798565
COX15	-1.4531538
COX16	-1.6783333
COX17	-1.9473112
COX18	-1.955029
COX19	-1.4038782
COX2	-1.1607594
COX20	-1.6748295
COX3	-1.4737287
COX4I1	-1.6993937
COX5A	-1.9538579
COX5B	-1.4379807
COX6A1	-1.8320937
COX6B1	-1.4269772
COX6C	-1.4540362
COX7A2	-1.5338311
COX7A2L	-1.3258734
COX7B	-2.1113782
COX7B2	-2.0341086
COX7C	-1.8613644
COX8A	-1.807744
COX8C	-2.15306
CP	-3.1063519
CPA2	-1.1865263
CPA4	-2.0641596
CPA6	-1.3572817
CPB2	-10.627819
CPEB2	-1.2509604
CPEB3	-1.278408
CPHX1	-3.5375638
CPLX1	-0.7683246

CPLX2	-0.6939693
CPLX4	-2.2415116
CPN1	-7.872017
CPN2	-9.986153
CPNE4	-2.8331206
CPNE6	-1.9428213
CPNE9	-2.0986571
CPOX	-3.9329238
CPPED1	-3.1916451
CPQ	-2.173286
CPS1	-12.247826
CPSF1	-1.2102633
CPSF3	-0.7313342
CPSF3L	-0.9284005
CPSF4	-1.3276496
CPSF4L	-5.3875456
CPT1A	-2.875452
CPT2	-3.5359545
CPVL	-2.155079
CPXCR1	-3.8250852
CR2	-1.0024698
CRABP2	-0.4350653
CRADD	-1.501811
CRAT	-0.5053489
CRB1	-1.2952952
CRB2	-1.2447519
CRB3	-0.7023811
CRBN	-0.4388075
CRCP	-1.8724928
CRCT1	-2.170727
CREB3L3	-10.58644
CREB5	-1.7165179
CREBBP	-0.4159064
CREG1	-2.6027694
CREG2	-0.9584847
CRELD1	-1.711896
CRH	-2.3462152
CRHBP	-1.9996004
CRHR2	-1.0730913
CRIPT	-0.3799219
CRISP1	-2.1208367
CRISP2	-1.7804191
CRISP3	-2.2149477
CRISP4	-2.2904599
CRLS1	-2.8204908

CRNKL1	-0.296387
CRNN	-1.8853128
CROCC	-0.3525114
CROT	-5.4743934
CRP	-9.011292
CRTAP	-0.315867
CRTC1	-1.0387225
CRX	-1.9182811
CRY1	-0.3254042
CRYBA1	-1.1439967
CRYBA4	-1.8313234
CRYBB3	-3.471603
CRYGA	-0.9087765
CRYGC	-1.5029421
CRYGE	-2.1293814
CRYGF	-1.1810038
CRYGN	-2.2698653
CRYGS	-2.7459211
CRYL1	-1.5044742
CRYM	-2.5655246
CRYZ	-4.9425974
CS	-0.3385854
CSAD	-1.8669214
CSDE1	-0.4469647
CSE1L	-0.4613209
CSF1R	-1.0056338
CSK	-0.8396792
CSL	-2.2677522
CSMD1	-0.1792336
CSMD2	-2.690236
CSMD2OS	-0.9530306
CSMD3	-2.099926
CSN1S1	-2.2633734
CSN1S2A	-2.276774
CSN1S2B	-2.299879
CSN2	-3.342747
CSN3	-1.2178495
CSNK1G2	-1.2774277
CSNK2A1	-0.7133136
CSNK2A2	-0.4579105
CSNK2B	-1.0548496
CSNKA2IP	-1.644371
CSPRS	-0.1483479
CSRNP2	-0.7456801
CSRP2	-0.5650606

CSRP2BP	-1.0916781
CSRP3	-1.9124513
CST11	-1.6474786
CST12	-1.8521714
CST13	-2.0859945
CST9	-2.2374961
CSTA1	-2.1128278
CSTB	-0.8319936
CSTF1	-1.1812668
CSTF2T	-1.0188212
CSTL1	-2.2903986
CTAG2	-2.1394572
CTAGE5	-2.3131132
CTC1	-0.9423232
CTCFL	-2.544104
CTCFLOS	-3.4329958
CTDNEP1	-0.2871971
CTDSP1	-0.7385173
CTDSP2	-0.5054154
CTF1	-2.2491312
CTF2	-2.0052269
CTH	-5.912226
CTIF	-2.1945224
CTNNA2	-2.5456624
CTNNA3	-0.9097357
CTNNBIP1	-1.42518
CTNS	-1.4369249
CTPS2	-0.2863531
CTR9	-0.2164927
CTRB1	-2.9345195
CTS3	-2.0518656
CTS8	-2.0696785
CTSA	-1.3553109
CTSB	-1.4460444
CTSC	-0.5052404
CTSD	-1.1822972
CTSE	-1.166728
CTSF	-2.4087415
CTSG	-1.1337342
CTSH	-1.3395047
CTSJ	-2.3540802
CTSL	-2.6188364
CTSLL3	-2.1517751
CTSQ	-2.9941025
CTSR	-2.1218147

CTSZ	-1.644568
CTTN	-0.4661236
CTU1	-0.6170211
CTU2	-0.4406185
CUL2	-0.5470672
CUL4A	-1.2010207
CUL4B	-0.2073746
CUL9	-1.0510647
CUTA	-0.3412314
CUTAL	-2.4303079
CUTC	-1.995425
CUX2	-2.8439345
CUZD1	-2.0659335
CWC15	-0.3767161
CWF19L1	-2.2433157
CWF19L2	-0.1996408
CWH43	-2.0424945
CXCL11	-0.5106473
CXCL12	-2.6808157
CXCL9	-0.3651981
CXXC1	-0.688406
CXXC5	-4.0810246
CYB561A3	-0.4514198
CYB561D1	-0.1794677
CYB561D2	-1.201767
CYB5A	-2.4311867
CYB5B	-2.6340694
CYB5D1	-0.618948
CYB5D2	-2.1101022
CYB5R2	-1.8956852
CYB5R3	-2.5648565
CYB5RL	-2.5010786
CYC1	-2.837953
CYCS	-1.0102515
CYCT	-0.9903805
CYHR1	-2.260962
CYLC1	-2.727576
CYM	-1.4189897
CYP11B1	-2.1066382
CYP11B2	-2.0631607
CYP17A1	-0.8947191
CYP1A2	-13.096146
CYP21A1	-1.6077209
CYP24A1	-2.2064404
CYP26A1	-5.373579

CYP26B1	-4.4947405
CYP26C1	-4.286748
CYP27A1	-6.180168
CYP2A12	-13.864351
CYP2A22	-8.954845
CYP2A4	-8.141668
CYP2B13	-11.916339
CYP2B19	-2.0328147
CYP2B9	-2.9999056
CYP2C29	-14.6297
CYP2C37	-12.438299
CYP2C38	-11.583357
CYP2C39	-13.402296
CYP2C44	-9.619181
CYP2C50	-15.708157
CYP2C54	-12.590944
CYP2C55	-6.6115727
CYP2C65	-2.3366332
CYP2C66	-2.1790268
CYP2C67	-13.671115
CYP2C68	-14.417795
CYP2C70	-10.807396
CYP2D10	-13.272583
CYP2D11	-7.2272787
CYP2D12	-8.577837
CYP2D13	-9.031832
CYP2D22	-6.513575
CYP2D26	-13.294938
CYP2D34	-10.783098
CYP2D37-PS	-11.700668
CYP2D40	-9.393446
CYP2D9	-10.283545
CYP2E1	-8.231131
CYP2G1	-8.932589
CYP2J13	-4.745509
CYP2J5	-6.1358566
CYP2J6	-3.3693137
CYP2J8	-6.1485386
CYP2J9	-1.8071697
CYP2R1	-4.327455
CYP2U1	-1.6665487
CYP39A1	-2.776813
CYP3A11	-12.894746
CYP3A13	-7.565067
CYP3A16	-13.927149

CYP3A25	-13.783697
CYP3A41B	-13.981497
CYP3A44	-12.273682
CYP3A57	-12.539465
CYP3A59	-13.302617
CYP46A1	-0.7357149
CYP4A10	-11.716343
CYP4A12A	-1.9337721
CYP4A12B	-1.2749617
CYP4A14	-15.535548
CYP4A30B	-2.2191906
CYP4A31	-11.535511
CYP4A32	-12.164034
CYP4F13	-2.976663
CYP4F14	-11.302063
CYP4F15	-5.701227
CYP4F16	-0.7886691
CYP4F17	-2.7111268
CYP4F37	-2.1260667
CYP4F39	-1.1157882
CYP4F40	-2.2245088
CYP4V3	-3.1737113
CYP4X1	-1.6652031
CYP51	-3.2583613
CYP7A1	-10.719387
CYP7B1	-2.174346
CYP8B1	-8.140261
CYPT1	-2.0029287
CYPT10	-1.3365748
CYPT12	-2.0319943
CYPT14	-3.9809117
CYPT15	-2.114825
CYPT2	-2.5174296
CYPT3	-2.2090378
CYPT7	-2.1343768
CYPT8	-2.164309
CYPT9	-2.269485
CYTB	-1.7511849
CYTH1	-0.5453968
DAB1	-1.1116006
DACT2	-4.647129
DAD1	-1.3975792
DAGLB	-1.825491
DAK	-7.6637897
DALRD3	-1.029974

DANCR	-0.2276497
DAND5	-0.8223302
DAP	-3.0872545
DAP3	-1.369154
DAPK1	-0.4514046
DAPK2	-0.5700946
DARS	-0.4569917
DARS2	-2.1244864
DAZAP1	-0.4978676
DAZL	-2.2125008
DBHOS	-2.3120584
DBI	-3.3969321
DBIL5	-2.3740392
DBNDD2	-0.2327738
DBNL	-0.1729341
DBP	-4.4377174
DBR1	-0.5663109
DBT	-3.231626
DBX1	-1.8873749
DBX2	-2.4020488
DCAF11	-2.626614
DCAF12	-0.402524
DCAF12L1	-2.0650232
DCAF13	-0.3704796
DCAF17	-0.5431819
DCAF4	-1.2481179
DCAF5	-1.249608
DCAF6	-0.5432348
DCAF7	-0.5775731
DCAF8	-1.3960471
DCAKD	-1.8217235
DCDC2A	-0.5577359
DCDC2C	-2.0850737
DCLK3	-6.464141
DCLRE1A	-2.1257432
DCN	-1.4068818
DCPP1	-2.096176
DCPP2	-2.1057942
DCPP3	-2.255498
DCPS	-0.766737
DCST2	-0.8936248
DCT	-7.8123455
DCTN2	-1.8416162
DCTN3	-0.5514145
DCTPP1	-2.8988013

DCUN1D1	-0.3958092
DCUN1D2	-0.6152811
DCUN1D4	-1.4016643
DCX	-2.0010734
DCXR	-3.2605958
DDAH1	-3.1866727
DDB1	-0.737679
DDB2	-0.5822911
DDC	-5.869725
DDHD2	-0.3848145
DDI1	-2.3653705
DDI2	-1.8865876
DDIT4L	-2.1380973
DDO	-2.2330463
DDOST	-0.4537621
DDRGK1	-2.0615687
DDT	-4.5243473
DDX1	-0.8672781
DDX10	-0.2380672
DDX19A	-0.7817433
DDX19B	-0.800993
DDX20	-0.249125
DDX27	-0.3227429
DDX28	-2.0167108
DDX31	-0.362524
DDX3Y	-2.2374208
DDX4	-2.0269938
DDX41	-1.5086918
DDX47	-0.484704
DDX49	-0.9159875
DDX51	-0.8126926
DDX54	-0.3905373
DDX56	-1.773747
DDX59	-0.2175465
DEAF1	-0.8562832
DEAR1	-2.903208
DEB1	-2.3730655
DECR1	-3.8786392
DECR2	-4.467275
DEF8	-2.7997203
DEFA1	-2.1178205
DEFA2	-2.7825341
DEFA21	-3.048607
DEFA23	-0.6490097
DEFA3	-2.758287

DEFA-PS12	-1.5027962
DEFA-PS13	-2.180853
DEFA-RS1	-2.113401
DEFA-RS10	-0.6208861
DEFA-RS12	-1.5376205
DEFA-RS2	-2.694463
DEFA-RS4	-2.1743264
DEFA-RS7	-1.8599887
DEFB12	-2.0921726
DEFB13	-1.8481102
DEFB19	-2.3099945
DEFB20	-2.1745062
DEFB21	-4.4147997
DEFB22	-0.7919395
DEFB26	-3.749054
DEFB28	-1.7228477
DEFB3	-1.5416882
DEFB34	-2.4996185
DEFB35	-1.5722134
DEFB36	-1.8579664
DEFB37	-0.9820814
DEFB38	-1.9108891
DEFB39	-1.9488046
DEFB4	-2.357683
DEFB40	-2.0082917
DEFB42	-0.6425018
DEFB43	-2.1442552
DEFB44-PS	-1.8798199
DEFB46	-1.8813505
DEFB48	-2.0239599
DEFB5	-2.0529208
DEFB50	-1.9432108
DEFB6	-3.758707
DEFB7	-2.0067074
DEFB8	-2.1197608
DEGS1	-0.5660276
DEGS2	-1.2998462
DENND1A	-1.005466
DENND2D	-0.5848489
DENND4A	-1.5621791
DENND5B	-0.4870338
DENND6A	-0.4297633
DENND6B	-2.040035
DENR	-0.8868136
DEPDC5	-0.343174

DEPDC7	-5.0004764
DEPTOR	-1.7972069
DERA	-3.9409971
DERL1	-1.6652412
DERL2	-1.5060539
DERL3	-2.29743
DESI1	-1.504529
DESI2	-1.4248161
DET1	-2.568142
DEXI	-1.8069029
DFFA	-0.4204562
DFFB	-0.5766587
DFNA5	-0.2889831
DFNB59	-1.8583527
DGAT1	-1.0585227
DGAT2	-4.0424185
DGAT2L6	-1.988205
DGCR2	-1.3403621
DGCR6	-2.6660972
DGKQ	-1.3197374
DGKZ	-1.4387302
DGUOK	-0.8646202
DHCR24	-5.2397847
DHCR7	-5.650057
DHDDS	-0.6398959
DHDH	-4.514212
DHFR	-3.5795362
DHODH	-1.6352448
DHRS1	-3.0564637
DHRS11	-3.7242374
DHRS13	-1.2782159
DHRS2	-2.228606
DHRS3	-1.2271595
DHRS4	-3.450334
DHRS7	-2.2409525
DHRS7B	-1.4696798
DHRSX	-1.1700478
DHTKD1	-4.9061356
DHX16	-0.5557075
DHX32	-1.3341236
DHX33	-0.3715925
DHX34	-0.2749605
DHX35	-0.6390095
DHX36	-0.9645567
DHX37	-1.3109128

DHX58	-0.449059
DIABLO	-1.1576285
DICER1	-0.3311348
DIO1	-8.078251
DIO3	-1.9062359
DIO3OS	-2.7367008
DIP2C	-0.3612738
DIRAS1	-1.6642604
DIS3L	-0.4145165
DIS3L2	-1.2432613
DISC1	-2.3210492
DISP1	-1.1182027
DIXDC1	-2.2477448
DKC1	-0.4319401
DKK1	-2.134708
DKK4	-2.6685996
DLAT	-0.5923715
DLD	-1.5559249
DLEC1	-0.7769275
DLEU2	-0.9136822
DLEU7	-2.1887944
DLG2	-2.039743
DLG3	-0.3995476
DLGAP1	-1.9724064
DLGAP2	-2.0989113
DLGAP3	-0.3941414
DLGAP5	-1.1147344
DLK2	-0.9541526
DLST	-1.6044383
DLX1	-2.0101151
DLX2	-2.3555317
DLX3	-1.0799875
DLX6	-1.4361181
DLX6AS2	-1.0208952
DLX6OS1	-1.7866044
DMAP1	-0.6840591
DMBT1	-4.1724234
DMBX1	-2.2751338
DMGDH	-13.086003
DMP1	-2.1597974
DMPK	-2.5535464
DMRTA1	-1.8114014
DMRTA2	-2.2600856
DMRTB1	-1.4578826
DMRTC1A	-2.0949607

DMRTC2	-2.2160227
DMTN	-1.5590563
DMWD	-0.4169197
DNA2	-0.7895753
DNAH1	-1.1820192
DNAH3	-2.3586547
DNAIC1	-1.554059
DNAJA1	-0.4816976
DNAJA2	-1.0872159
DNAJA3	-2.8532486
DNAJB11	-0.2302356
DNAJB12	-1.5465374
DNAJB2	-1.0187035
DNAJB7	-1.770258
DNAJB8	-1.1360748
DNAJC11	-1.665993
DNAJC12	-3.1639318
DNAJC15	-1.6680336
DNAJC16	-2.0291433
DNAJC17	-1.1984107
DNAJC19	-1.5247264
DNAJC22	-8.204077
DNAJC25	-2.6807995
DNAJC30	-1.7989874
DNAJC4	-1.4053845
DNAJC5G	-2.2302291
DNAJC6	-2.1028445
DNAJC8	-0.3415999
DNAL1	-2.2492802
DNASE1L3	-3.5721056
DNASE2B	-5.025454
DNER	-0.6926365
DNLZ	-1.8179302
DNM1L	-0.2653947
DNMBP	-1.511069
DNMT3B	-1.4493432
DNPEP	-1.2127452
DNPH1	-3.9748147
DNTT	-5.944254
DOC2G	-0.4255893
DOCK3	-1.666812
DOCK6	-0.5268641
DOHH	-1.0425062
DOK5	-2.0715075
DOLK	-1.3421459

DOLPP1	-1.2400184
DOPEY2	-0.3884153
DOS	-1.282906
DOT1L	-0.2518554
DOXL2	-1.0031879
DPAGT1	-1.3512788
DPCD	-0.6217856
DPEP3	-1.6029315
DPF1	-1.1489046
DPF3	-3.681247
DPH1	-2.2085218
DPH5	-0.1835995
DPH6	-0.2902374
DPM1	-0.9549232
DPM2	-0.7257719
DPM3	-1.8628435
DPP3	-1.8978033
DPP4	-2.2810903
DPP7	-2.2135592
DPP9	-0.4515042
DPPA1	-2.9660451
DPPA2	-1.2755063
DPPA3	-1.9142833
DPPA4	-1.0994077
DPY19L1	-1.6643176
DPY19L2	-1.8434672
DPY30	-0.7722936
DPYD	-7.047153
DPYS	-9.294609
DPYSL4	-1.9959953
DPYSL5	-1.6605277
DQX1	-2.234852
DRAM2	-0.6413922
DRAXIN	-2.9316888
DRD1A	-2.2192707
DRD3	-1.827744
DRD4	-2.5845609
DRD5	-1.5989692
DRG1	-0.7724557
DRG2	-1.8681622
DSC1	-0.2787571
DSC2	-6.831544
DSC3	-2.904684
DSCAML1	-1.1950166
DSCR3	-0.6056113

DSE	-0.578366
DSG1A	-2.143388
DSG1B	-2.5569575
DSG1C	-1.1121368
DSG2	-3.1129372
DSG3	-1.7574673
DSG4	-2.179488
DSP	-0.7615352
DTD1	-1.1081285
DTD2	-2.3129284
DTNBP1	-1.3242869
DTWD1	-1.5836964
DTWD2	-1.0282619
DUOX1	-2.0794282
DUOXA1	-1.4950762
DUOXA2	-0.8414202
DUS1L	-2.5381837
DUS2	-1.249104
DUS3L	-0.4235196
DUS4L	-0.5829849
DUSP12	-0.9776478
DUSP15	-2.9715972
DUSP19	-0.1768596
DUSP21	-2.1586826
DUSP22	-2.163383
DUSP23	-1.0648062
DUSP28	-2.6212015
DUT	-1.0112553
DUX	-1.2528722
DUXBL1	-2.221302
DVL1	-1.766315
DVL2	-1.1326962
DXBAY18	-0.7515109
DXO	-1.4176836
DYM	-1.086401
DYNAP	-2.0685334
DYNLL2	-2.462143
DYNLRB1	-1.0144677
DYRK1B	-1.4178271
DYRK2	-0.7337513
DYSF	-0.7903776
EAF1	-0.6660495
EAF2	-1.8498459
EAPP	-0.5120053
EARS2	-2.2200007

EBAG9	-1.1807938
EBF3	-3.0349207
EBP	-3.2791286
EBPL	-5.533006
ECD	-1.201365
ECE2	-1.7730069
ECEL1	-1.3687506
ECH1	-3.5694742
ECHDC1	-1.1279678
ECHDC2	-3.2496614
ECHDC3	-5.7408814
ECHS1	-3.8523636
ECI1	-3.187841
ECI2	-2.8880835
ECI3	-2.9722881
ECM1	-0.2740493
ECSIT	-1.4186664
ECT2	-1.2918973
EDA	-0.3519788
EDA2R	-1.8186831
EDAR	-0.3598802
EDDM3B	-2.2654502
EDEM1	-1.486166
EDEM2	-1.66429
EDF1	-1.6975117
EDN2	-2.235153
EDNRB	-0.6907511
EDRF1	-0.3516231
EEF1A1	-1.4887457
EEF1B2	-1.125474
EEF1D	-0.874341
EEF1G	-0.575839
EEF2	-1.8305697
EEF2K	-0.5669575
EEFSEC	-2.9008708
EEPD1	-1.55808
EFCAB2	-0.2300143
EFCAB8	-2.1236641
EFNA1	-1.4752159
EFNA3	-0.3959329
EFR3A	-0.7779303
EGF	-1.505024
EGFEM1	-1.2615466
EGFR	-2.6398091
EGLN2	-0.9177427

EGLN3	-1.6451311
EGR4	-2.0221627
EHD3	-3.1882515
EHHADH	-8.025709
EHMT1	-1.1088157
EHMT2	-0.7358718
EI24	-2.8263707
EID2B	-0.9671557
EID3	-0.5786562
EIF1	-0.2595143
EIF1A	-0.5641952
EIF1AD	-1.2899771
EIF1B	-0.2694497
EIF1-PS	-0.7656865
EIF2A	-0.7071695
EIF2AK1	-1.2556968
EIF2B1	-1.3842196
EIF2B4	-1.4719672
EIF2B5	-0.9565201
EIF2D	-1.9842472
EIF2S1	-0.4189267
EIF2S2	-0.2332749
EIF2S3X	-0.6553974
EIF3A	-0.6149693
EIF3B	-0.5329065
EIF3D	-0.4913855
EIF3F	-0.616571
EIF3G	-0.4581604
EIF3H	-0.4433503
EIF3I	-1.5448413
EIF3J1	-0.8666129
EIF3K	-0.4373808
EIF3L	-0.8502207
EIF3M	-0.4167109
EIF4A3	-0.5928578
EIF4B	-0.5113831
EIF4E	-0.4696546
EIF4E2	-1.3796844
EIF4E3	-0.2753601
EIF4EBP1	-1.4064035
EIF4EBP2	-1.5490785
EIF4EBP3	-1.1135845
EIF4G1	-1.6989155
EIF4G2	-0.6073165
EIF4G3	-0.3426304

EIF4H	-0.309701
EIF5	-0.4047389
EIF5A	-1.7817445
EIF5B	-0.8088479
EIF6	-1.4184804
ELAC1	-0.675981
ELAC2	-1.3246346
ELAVL2	-2.0791657
ELAVL3	-1.6661391
ELAVL4	-0.6591725
ELFN1	-2.095515
ELK1	-0.7283521
ELK3	-0.2912269
ELL	-0.8080702
ELL2	-0.6835303
ELL3	-4.051662
ELMO3	-1.4066596
ELMOD2	-0.952385
ELMOD3	-1.4016414
ELOF1	-0.5550938
ELOVL2	-12.488361
ELOVL3	-2.5831807
ELOVL4	-0.4830139
ELOVL5	-3.1318927
ELOVL6	-3.462903
ELP2	-0.4817066
ELP3	-1.2157626
ELP4	-1.1889904
ELP5	-0.4950361
ELP6	-1.3487492
ELTD1	-0.4177525
EMC1	-1.0049148
EMC10	-2.0565326
EMC3	-1.3664713
EMC4	-0.7991514
EMC6	-1.5970607
EMC7	-1.5147247
EMC8	-1.4713831
EMC9	-2.984745
EME1	-0.5491614
EME2	-1.6214347
EMG1	-0.8951664
EML3	-0.7895856
EML4	-0.3397722
EML5	-0.8915091

EMR1	-1.22506
EMR4	-0.5186939
EMX1	-1.8454895
EMX2	-2.0782905
EN1	-2.1703207
EN2	-1.3637099
ENAM	-2.251872
ENDOG	-3.022503
ENDOV	-0.671546
ENGASE	-1.9401631
ENHO	-3.5355139
ENO1	-2.3510137
ENO1B	-2.4282784
ENOX1	-1.1802905
ENOX2	-0.6126709
ENPEP	-1.6127238
ENPP1	-2.0800877
ENPP2	-1.6984181
ENPP3	-3.8981097
ENPP5	-0.483613
ENSA	-0.3762474
ENTHD1	-1.6731005
ENTHD2	-1.0161929
ENTPD2	-1.6103268
ENTPD5	-3.6225095
ENTPD6	-2.2729177
ENTPD7	-0.9451006
ENTPD8	-9.251875
ENY2	-1.4529753
EPB4.1	-0.2389956
EPB4.1L3	-0.8615336
EPB4.1L4B	-1.5940676
EPB4.1L5	-0.7303617
EPB4.2	-1.5817237
EPG5	-1.0167117
EPHA1	-4.7467785
EPHA5	-1.6885967
EPHA6	-2.1260593
EPHB2	-0.9962082
EPHB3	-1.357502
EPHB4	-0.5408144
EPHB6	-0.6579461
EPHX1	-3.2822013
EPHX2	-8.284121
EPHX3	-0.621923

EPM2AIP1	-0.4599333
EPN1	-1.2718849
EPOR	-1.8298416
EPPIN	-1.9579449
EPPK1	-1.3317757
EPRS	-1.6924794
EPS8L2	-1.3328447
EPT1	-2.68397
EPX	-2.6451328
EPYC	-2.2657743
ERAL1	-1.4995189
ERAP1	-1.4073577
ERBB3	-1.0346205
ERBB4	-0.7079487
ERCC2	-0.3794193
ERCC3	-0.946444
ERCC6L	-0.5973575
ERCC6L2	-0.419035
ERCC8	-0.4318175
ERDR1	-1.0089989
ERF	-1.1875296
ERGIC1	-2.3745494
ERGIC2	-0.5745845
ERGIC3	-1.4925957
ERI2	-1.3910108
ERI3	-1.8611372
ERICH4	-4.264982
ERICH5	-2.0031672
ERICH6	-0.5566678
ERLEC1	-0.453733
ERLIN1	-1.5743985
ERLIN2	-1.2333941
ERMARD	-1.2855
ERMP1	-1.5805392
ERN1	-0.2057388
ERO1LB	-2.1209579
ERP29	-0.8213735
ERP44	-0.2497068
ERRFI1	-1.538321
ESCO2	-1.5916572
ESD	-2.5160446
ESF1	-0.1857853
ESP1	-3.108157
ESP6	-1.9855659
ESPL1	-0.5150206

ESPN	-1.9268322
ESR1	-4.361679
ESRP2	-2.82437
ESRRA	-1.5243683
ESRRB	-1.5100918
ESRRG	-2.6404388
ESX1	-2.4924257
ETD	-1.8781471
ETFA	-3.2560768
ETFB	-3.2749057
ETFDH	-3.7927923
ETHE1	-3.9201665
ETNK2	-8.284945
ETNPPL	-10.218514
ETV2	-1.6782606
ETV4	-0.8082016
EVA1A	-4.5098534
EVC	-0.5047534
EVI5	-2.2236114
EVX1	-1.8395631
EXD1	-1.493362
EXOC3L	-1.4423132
EXOC3L2	-4.505806
EXOC6	-0.7960997
EXOC7	-1.4814386
EXOSC1	-0.478163
EXOSC2	-1.2130685
EXOSC4	-2.1386728
EXOSC5	-1.5009494
EXOSC7	-1.0260506
EXOSC9	-2.0057096
EXT1	-1.5630116
EXTL1	-5.084634
EXTL2	-1.7795238
EYA1	-1.5754595
F10	-3.154902
F11	-8.651178
F11R	-0.4623981
F12	-14.362644
F13B	-12.013451
F2	-12.908922
F2RL1	-1.623456
F2RL3	-0.7318947
F5	-6.29021
F7	-4.0972776

F8	-4.597064
F8A	-1.9981861
F9	-6.346343
FAAH	-4.5550566
FABP1	-7.287378
FABP2	-8.139683
FABP5	-2.3343015
FABP7	-6.2754555
FABP9	-2.168936
FADD	-1.3151398
FADDOS	-2.7897897
FADS1	-4.350212
FADS2	-5.9603634
FADS6	-4.9703455
FAF1	-0.5334883
FAF2	-1.2048278
FAH	-6.0568657
FAHD1	-4.1689835
FAHD2A	-3.0038385
FAM103A1	-0.4036908
FAM104A	-0.4351015
FAM107B	-0.77632
FAM109A	-0.3924184
FAM110B	-0.5253286
FAM114A1	-1.3059616
FAM114A2	-1.1029625
FAM115E	-1.7901096
FAM120A	-1.012156
FAM122B	-0.3645239
FAM122C	-1.7735615
FAM124A	-2.357035
FAM131B	-1.3767965
FAM131C	-4.999118
FAM134A	-3.1336646
FAM134C	-1.3433924
FAM135A	-1.0591197
FAM135B	-2.2573676
FAM136A	-2.9162612
FAM13A	-0.7003508
FAM149A	-0.7562103
FAM149B	-0.4277391
FAM150B	-1.9590168
FAM151B	-0.7829373
FAM155A	-2.0064294
FAM159B	-2.090783

FAM160B1	-1.0579004
FAM160B2	-1.6109743
FAM162A	-1.4313126
FAM163A	-2.0957687
FAM166A	-2.06879
FAM167B	-3.851933
FAM169A	-2.1454208
FAM169B	-3.6716013
FAM171A1	-0.2091341
FAM173A	-1.7667437
FAM173B	-1.5531325
FAM175A	-0.5122511
FAM180A	-1.4434462
FAM184B	-2.0442784
FAM185A	-1.9726765
FAM186B	-2.080607
FAM187A	-2.5432522
FAM187B	-1.8193488
FAM192A	-0.5114837
FAM193A	-0.6549063
FAM193B	-0.5173845
FAM195A	-4.9111767
FAM196B	-2.2039225
FAM198A	-2.7526398
FAM19A2	-5.6365023
FAM19A4	-2.3589573
FAM203A	-1.3991222
FAM204A	-0.6971779
FAM207A	-1.0820465
FAM20A	-4.851198
FAM20B	-0.330904
FAM20C	-3.1283784
FAM210A	-1.716815
FAM210B	-2.9033184
FAM213A	-4.3274693
FAM213B	-3.0903058
FAM214A	-2.5761476
FAM219A	-2.0843964
FAM221B	-2.0336118
FAM227B	-2.0646744
FAM228A	-2.54013
FAM228B	-1.7941723
FAM229B	-0.4789176
FAM25C	-7.209132
FAM26D	-3.1479013

FAM26F	-1.0111046
FAM32A	-0.7440486
FAM35A	-3.4351916
FAM3A	-0.9993467
FAM3C	-0.8212452
FAM43B	-2.010219
FAM45A	-0.8757734
FAM46A	-1.5842457
FAM46D	-2.018087
FAM47C	-1.941474
FAM47E	-1.4173946
FAM50A	-0.406116
FAM53A	-0.91751
FAM57B	-1.0730383
FAM58B	-0.9949732
FAM63A	-1.4953899
FAM64A	-0.5002945
FAM65C	-1.1758509
FAM69A	-0.5187297
FAM71A	-2.0482981
FAM71E2	-1.8207037
FAM71F1	-1.8128002
FAM72A	-0.2293286
FAM73B	-3.0439305
FAM83C	-2.2080731
FAM83D	-1.7293377
FAM83F	-3.4466689
FAM83G	-0.3715191
FAM83H	-1.3384662
FAM84B	-0.4445717
FAM86	-1.4859934
FAM89A	-2.7645974
FAM89B	-0.294075
FAM92A	-0.9313397
FAM96A	-1.552227
FAM98A	-0.1975007
FAM98C	-0.8123975
FAN1	-1.4062443
FANCA	-0.4872263
FANCB	-1.2198653
FANCC	-0.3651447
FANCD2	-0.9837203
FANCD2OS	-2.243964
FANCE	-0.7359543
FANCF	-1.7651112

FANCI	-1.1510518
FANCL	-0.7094979
FANCM	-1.0033779
FAR2	-0.6119096
FAR2OS2	-2.2331138
FARP2	-1.6337237
FARS2	-2.3774147
FARSA	-0.5332069
FARSB	-0.9494109
FASN	-2.643505
FASTK	-1.4354582
FASTKD1	-1.8224444
FASTKD2	-2.2873678
FASTKD3	-0.586802
FASTKD5	-1.0633082
FATE1	-2.2134023
FAU	-0.7273746
FAXC	-2.0923483
FBLL1	-1.8088977
FBLN7	-2.1167793
FBP1	-10.779409
FBXL15	-0.9816413
FBXL17	-1.4172239
FBXL18	-0.7265182
FBXL21	-1.9082391
FBXL4	-1.8356814
FBXL6	-1.2895088
FBXL8	-0.6736627
FBXO16	-0.8925612
FBXO17	-0.8068318
FBXO18	-0.5677524
FBXO21	-3.5126252
FBXO22	-1.4629135
FBXO25	-0.4145136
FBXO3	-1.893127
FBXO31	-1.4039416
FBXO34	-0.8912778
FBXO36	-0.6636448
FBXO43	-3.8468053
FBXO48	-2.1311655
FBXO6	-0.7836776
FBXO7	-0.5602355
FBXO8	-2.302166
FBXO9	-1.2665591
FBXW13	-1.4152975

FBXW14	-2.1070426
FBXW16	-1.8703046
FBXW19	-3.1213627
FBXW20	-0.4349625
FBXW21	-1.0023093
FBXW22	-1.7982106
FBXW24	-1.7673395
FBXW26	-4.5121665
FBXW27	-0.6249304
FBXW4	-0.417728
FBXW5	-1.3007369
FBXW9	-1.2013965
FCF1	-0.5727754
FCGR2B	-1.2999687
FCGRT	-3.4697242
FCHSD2	-0.4358683
FCNA	-6.631061
FCNAOS	-3.5474732
FCRL5	-1.3642707
FCRL6	-2.0364666
FDFT1	-3.570201
FDPS	-4.27346
FDX1	-1.549406
FDX1L	-1.4821434
FDXACB1	-1.971015
FDXR	-2.6123223
FECH	-2.5170774
FEM1A	-1.0691395
FER1L5	-2.2783875
FER1L6	-1.2082627
FERMT2	-0.4537072
FETUB	-8.022054
FEZ1	-0.9480763
FEZ2	-0.3791938
FEZF1	-2.0074549
FEZF2	-1.6047671
FGA	-10.777
FGB	-10.708057
FGD1	-1.2975736
FGD2	-1.3464022
FGD6	-0.892272
FGF1	-0.629777
FGF14	-1.871187
FGF15	-1.6576178
FGF16	-1.9897628

FGF20	-1.9402585
FGF21	-8.936005
FGF2OS	-2.2085042
FGF5	-2.360884
FGF8	-1.78037
FGFR1OP	-0.4615922
FGFR4	-2.6868744
FGFRL1	-2.0814342
FGG	-9.45623
FGGY	-5.829015
FGL1	-10.154966
FH1	-2.7120528
FHIT	-2.3486152
FHL4	-2.207873
FIBCD1	-2.1381903
FIBP	-1.6776819
FICD	-0.8106675
FIG4	-0.7947125
FIGLA	-2.1512525
FIGN	-1.4140987
FIGNL1	-0.317862
FITM1	-3.3657951
FITM2	-2.348709
FIZ1	-0.9856772
FKBP11	-1.8475494
FKBP1A	-1.2529755
FKBP2	-1.2605214
FKBP3	-0.8099566
FKBP4	-2.0563412
FKBP8	-3.2508292
FKBPL	-0.7128353
FKRP	-0.5190387
FLAD1	-3.098637
FLCN	-0.4225225
FLOT2	-0.6434817
FLRT1	-3.0039012
FLT4	-2.4413657
FLYWCH1	-0.3744555
FMN1	-0.9117003
FMN2	-0.7838976
FMO1	-2.616364
FMO3	-3.5634584
FMO4	-4.202684
FMO5	-5.816068
FMO6	-1.9749653

FMO9	-2.0116758
FMR1NB	-1.7690654
FN1	-2.1159172
FN3K	-4.5973463
FND3C2	-2.15549
FNDC3A	-0.4912596
FNDC3C1	-3.054206
FNDC4	-4.871687
FNIP2	-1.337563
FNTA	-0.4052854
FOCAD	-0.8277779
FOLR1	-3.0478265
FOLR2	-3.7357836
FOLR4	-0.4185538
FOPNL	-1.0682421
FOXA2	-1.2539849
FOXA3	-8.956099
FOXB1	-1.4791603
FOXB2	-1.9748914
FOXD1	-2.0177367
FOXD3	-1.8802011
FOXE1	-2.701207
FOXI1	-1.5618122
FOXM1	-0.8933051
FOXN1	-2.2250152
FOXO1	-0.6443481
FOXO4	-0.3436894
FOXP2	-0.7137914
FOXQ1	-1.0923886
FOXR2	-2.297419
FOXRED1	-1.7717943
FPGS	-4.9549217
FPGT	-0.8873954
FPR-RS3	-1.6063151
FPR-RS4	-1.7228599
FRA10AC1	-1.776051
FRAT1	-2.5089493
FREM2	-0.4798179
FREM3	-1.5242884
FRK	-0.5733874
FRMD3	-1.9047074
FRMD4B	-0.4544623
FRMD6	-1.2174203
FRMD8OS	-1.460072
FRMPD3	-2.1480842

FRMPD4	-1.5217655
FRRS1	-2.1631079
FRRS1L	-1.8180232
FRS3	-2.1731136
FRZB	-3.7733717
FSBP	-1.4028428
FSCB	-3.4640646
FSHB	-2.1191108
FSHR	-1.9258993
FSIP2	-1.5602417
FST	-1.263217
FSTL4	-1.6549225
FSTL5	-2.0506678
FTCD	-14.795364
FTH1	-1.1438375
FTHL17	-2.1105466
FTL1	-2.0095344
FTO	-0.7340126
FTSJ1	-0.8794794
FTSJ2	-2.4772027
FUCA1	-1.6728573
FUK	-1.0627189
FUOM	-3.5849566
FURIN	-1.1249037
FUT1	-1.3065319
FUT10	-1.1537077
FUT4-PS1	-2.27294
FUT9	-1.6850777
FUZ	-1.016726
FXN	-3.4011388
FXYD1	-3.9058723
FYCO1	-0.8715382
FYTTD1	-0.7463749
FZD10	-2.0476112
FZD7	-0.3001614
FZD8	-1.4915042
FZR1	-0.7540484
G0S2	-4.11478
G3BP2	-0.4370866
G6PC	-11.948431
GAA	-1.4559035
GABARAP	-0.5944505
GABARAPL1	-1.5795159
GABARAPL2	-0.6741486
GABBR2	-2.3090088

GABPA	-0.2652206
GABRA1	-2.9542384
GABRA3	-0.9441822
GABRA4	-2.075278
GABRA5	-2.0417328
GABRA6	-2.157616
GABRB1	-2.6386743
GABRB2	-0.9768837
GABRB3	-1.9506721
GABRD	-1.9485736
GABRG1	-2.0724416
GABRG3	-2.0276206
GABRQ	-2.0988622
GABRR1	-4.4013376
GABRR3	-2.3406622
GAD1	-1.5085187
GAD1OS	-2.2856007
GADD45GIP1	-2.3421206
GADL1	-1.4960737
GAK	-1.1065583
GAL3ST1	-0.5714512
GAL3ST2	-1.6759744
GAL3ST3	-2.0649886
GALE	-2.5026565
GALK1	-3.4981627
GALK2	-0.9837465
GALM	-5.2756534
GALNS	-2.6184735
GALNT10	-1.1336923
GALNT14	-2.7902033
GALNT2	-0.7315488
GALNT4	-0.2825079
GALNT5	-1.1252444
GALNT7	-0.4340072
GALNTL5	-4.9404497
GALNTL6	-1.9582689
GALP	-0.543062
GALR1	-2.149103
GALR2	-2.0939698
GALR3	-2.0708945
GALT	-1.2196743
GAMT	-7.9428625
GANAB	-1.7169604
GANC	-1.4915752
GAPDH	-1.977098

GAPDHS	-1.0063932
GAREM	-3.3181658
GAREML	-0.9297521
GART	-0.8687964
GAS1	-1.1357331
GAS2	-5.131676
GAS2L3	-1.4768724
GAS5	-0.652555
GAST	-1.9388368
GATA4	-5.12706
GATA5	-1.3046281
GATA5OS	-1.7309818
GATAD1	-0.8678641
GATC	-1.9101629
GATM	-1.3740053
GATSL3	-0.737767
GBA	-1.36864
GBE1	-3.0990658
GBP11	-2.9077983
GBP2B	-2.4318104
GBP8	-0.6240869
GC	-11.859707
GCAT	-5.7000246
GCC2	-0.6058188
GCDH	-5.8580456
GCFC2	-0.977078
GCG	-0.8517447
GCGR	-9.110204
GCH1	-2.0793567
GCHFR	-6.88943
GCK	-7.103181
GCKR	-12.01654
GCLC	-1.4845114
GCLM	-2.5762777
GCN1L1	-0.9672532
GCNT3	-1.4986899
GCNT4	-2.2317047
GCNT7	-2.1645632
GCSH	-3.5487351
GDAP1	-1.5722969
GDAP1L1	-1.0713673
GDAP2	-1.0689116
GDAP5	-1.6912744
GDF1	-0.4457033
GDF10	-2.978175

GDF15	-3.1687303
GDF2	-7.429308
GDF3	-0.634913
GDF6	-2.2132533
GDF7	-1.3049495
GDI2	-0.9940181
GDPGP1	-1.3974118
GEMIN2	-2.2064142
GEMIN4	-0.5983057
GEMIN5	-1.1211138
GEMIN6	-1.4518154
GEMIN7	-0.6272903
GEMIN8	-0.330014
GEN1	-0.3349242
GFER	-2.094019
GFM1	-1.9088335
GFM2	-1.8145804
GFOD1	-2.1793647
GFRA1	-4.482653
GFRA2	-0.1898465
GFRAL	-2.0035367
GFY	-1.0894959
GGA1	-1.3786287
GGA2	-1.6233997
GGACT	-3.300627
GGCT	-1.1548767
GGCX	-2.7960196
GGH	-0.4617338
GGNBP1	-3.2973955
GGNBP2	-0.585309
GGT6	-5.448353
GH	-2.3513498
GHDC	-1.7812839
GHITM	-1.5993485
GHR	-3.6781225
GHRHR	-1.6625535
GHSR	-2.0480556
GID4	-0.4359303
GID8	-0.7415476
GIGYF1	-0.2789083
GIGYF2	-0.476223
GIMAP9	-0.7988358
GIN1	-1.1125948
GINS1	-1.4862609
GINS2	-1.6428351

GINS4	-0.8453322
GIP	-1.4146421
GIPC1	-0.7750988
GJA10	-1.9550929
GJA6	-1.527792
GJB1	-8.612889
GJB2	-6.3641624
GJB5	-3.468816
GJD2	-3.054448
GJD4	-2.2028341
GJE1	-1.9286222
GKAP1	-0.7589293
GKN1	-1.5460696
GKN2	-2.1243432
GLB1	-1.5666938
GLCE	-2.274097
GLDC	-8.312485
GLDN	-7.544739
GLE1	-0.7909236
GLI2	-2.4956377
GLIPR1L2	-2.2358027
GLIS1	-0.7676411
GLMN	-1.2748013
GLO1	-3.6212997
GLOD4	-1.6710367
GLOD5	-3.0108588
GLRA1	-2.0784268
GLRA2	-2.0884414
GLRA3	-2.24363
GLRA4	-2.1242537
GLRB	-2.3709981
GLRX	-1.1423283
GLRX2	-1.0467429
GLRX3	-1.1313968
GLRX5	-2.9243546
GLS2	-9.728603
GLT1D1	-4.3798423
GLT25D1	-0.6481714
GLT28D2	-1.2507863
GLT6D1	-2.1277795
GLTPD1	-2.6419551
GLTPD2	-12.942047
GLTSCR2	-0.5686798
GLUD1	-3.0904994
GLUL	-1.4345517

GLYAT	-11.649736
GLYATL3	-2.0602956
GLYCAM1	-1.5271611
GLYCTK	-7.7033815
GLYR1	-0.9531655
GM10007	-2.1683078
GM10015	-0.5703058
GM10023	-2.2343936
GM10024	-2.0538619
GM10030	-2.5960371
GM10033	-1.3633611
GM10057	-2.3169575
GM10094	-0.6407132
GM10101	-0.5530415
GM10113	-1.6591496
GM10134	-1.0988445
GM10143	-2.1121836
GM10165	-2.0223844
GM10176	-2.3264794
GM10199	-1.1648884
GM10209	-3.643524
GM10220	-2.6112862
GM10248	-1.9191623
GM10252	-1.574058
GM10265	-2.141709
GM10266	-2.2025528
GM10267	-2.0067008
GM10272	-0.6768038
GM10276	-2.8174188
GM10277	-1.8741727
GM10280	-2.22553
GM10286	-0.3561833
GM10287	-1.2846026
GM10318	-0.7388477
GM10319	-4.8240232
GM10334	-0.2799587
GM10367	-2.9701338
GM10369	-2.4104896
GM10372	-1.4318225
GM10373	-2.1334906
GM10387	-2.3712025
GM10398	-1.7250147
GM10405	-1.7952776
GM10408	-1.603307
GM10413	-1.0644879

GM10415	-2.1124253
GM10419	-0.7813227
GM10420	-1.3703885
GM10421	-2.1789417
GM10426	-2.2768154
GM1043	-1.1461427
GM10432	-2.0615623
GM10433	-2.9917073
GM10435	-1.4631314
GM10436	-0.9789124
GM10437	-1.8439136
GM10439	-1.4310591
GM10440	-1.8480408
GM10441	-2.1795723
GM1045	-1.9361486
GM10450	-1.8669741
GM10459	-1.8578112
GM10465	-2.2171056
GM10466	-2.9173858
GM10468	-1.8655331
GM10469	-2.2467196
GM10471	-1.6640792
GM10473	-2.025542
GM10475	-1.5562105
GM10482	-1.8774514
GM10487	-2.1984377
GM10489	-2.192984
GM10493	-1.6491084
GM10494	-2.0347357
GM10501	-1.456094
GM10505	-3.469098
GM10509	-0.3116391
GM10512	-2.0680892
GM10513	-1.5783882
GM10516	-1.313668
GM10531	-2.8763745
GM10535	-2.2178493
GM10544	-2.1281903
GM10548	-1.0811205
GM10549	-2.2559977
GM10555	-2.2282228
GM10556	-4.0913134
GM10560	-1.1854918
GM10568	-2.221794
GM10578	-2.301672

GM10579	-1.9558208
GM10603	-3.7455854
GM10614	-2.014687
GM10616	-1.689729
GM10620	-2.15937
GM10623	-2.2779715
GM10624	-1.4751377
GM10627	-1.9155664
GM10632	-2.0741339
GM10635	-1.923429
GM10636	-2.067427
GM10637	-2.2182307
GM10639	-3.2862597
GM10640	-1.6071322
GM10641	-2.1610157
GM10649	-2.0439453
GM10651	-1.0350027
GM10652	-2.063487
GM10658	-6.0024014
GM10667	-2.1504655
GM10677	-2.087165
GM10680	-7.5105343
GM10682	-2.3657973
GM10683	-2.3099656
GM10687	-0.3241315
GM10688	-1.8995099
GM10690	-2.2244985
GM10696	-0.5901032
GM10701	-2.1983833
GM10703	-0.7255354
GM10706	-1.7426422
GM10714	-2.6487021
GM10723	-1.8828158
GM10724	-2.051895
GM10727	-1.7991514
GM10730	-2.2793803
GM10732	-0.8595233
GM10733	-2.2183452
GM10734	-3.4806645
GM10739	-2.1501367
GM10741	-0.7195914
GM10743	-2.2673616
GM10744	-2.6109128
GM10745	-1.9401071
GM10748	-1.6385174

GM10752	-5.2194223
GM10757	-2.060698
GM10759	-0.3993881
GM10763	-2.1702933
GM10764	-2.1871853
GM10766	-1.1504161
GM10767	-0.5365777
GM10768	-11.138817
GM10773	-1.5586255
GM10776	-2.6444676
GM10779	-2.4580078
GM10782	-1.936862
GM10785	-0.6967967
GM10787	-2.1451228
GM10790	-3.3544693
GM10811	-2.0568736
GM10814	-1.604707
GM10815	-1.3604665
GM10822	-2.295817
GM10823	-1.9487753
GM10824	-1.1058657
GM10825	-2.0447993
GM10826	-2.177523
GM10827	-4.347002
GM10829	-2.0007145
GM10830	-2.4914532
GM10831	-2.0241432
GM10832	-2.3592026
GM10835	-0.2419317
GM10836	-2.7946224
GM10845	-0.4415984
GM10846	-2.1294935
GM10848	-2.3104744
GM10854	-2.2407231
GM10857	-1.8665295
GM10860	-0.700397
GM10862	-2.1176188
GM10863	-1.2735004
GM10865	-0.5888419
GM10869	-2.5282602
GM10873	-1.9153142
GM10876	-2.0683737
GM10910	-1.5175996
GM10914	-1.4687953
GM11033	-3.9187646

GM11054	-2.1607583
GM1110	-1.5076265
GM11127	-1.4342294
GM11159	-2.1399274
GM11201	-2.2219543
GM11223	-0.3143892
GM1123	-0.3516839
GM11240	-3.2633953
GM11266	-1.6974852
GM11292	-2.1756005
GM11381	-2.5802822
GM1140	-2.8901412
GM1141	-0.9124739
GM11426	-2.0298028
GM11437	-6.623513
GM11468	-1.9091101
GM11492	-2.566285
GM11497	-1.7296722
GM11529	-2.3470526
GM11538	-2.0612373
GM11541	-3.139015
GM11544	-1.6264162
GM11545	-1.9010811
GM11546	-2.6293683
GM11554	-2.0702116
GM11563	-2.158302
GM11565	-2.0073946
GM11568	-2.1313233
GM11570	-1.6615565
GM11595	-1.0992286
GM11681	-2.1179223
GM11691	-0.9684894
GM11710	-1.6723907
GM11757	-1.1773884
GM11780	-2.2232323
GM11783	-1.6988754
GM11810	-0.4309712
GM11937	-1.0826461
GM12000	-2.0755522
GM12052	-1.7812388
GM12059	-2.1498861
GM12108	-1.8179932
GM12128	-2.1106024
GM12169	-2.0017567
GM12171	-1.7233129

GM12247	-1.9456053
GM12258	-1.7115517
GM12298	-0.792939
GM12302	-3.4233103
GM12371	-2.1223264
GM12408	-2.0342488
GM12447	-0.5656881
GM12503	-2.8343122
GM12511	-1.9519196
GM12522	-0.3108122
GM12526	-1.7892017
GM12542	-2.707386
GM12580	-1.6166451
GM12603	-2.1450262
GM12618	-0.6102057
GM12670	-2.106265
GM12693	-0.8481011
GM12703	-3.9609067
GM12758	-1.3638523
GM12794	-1.9513426
GM12816	-0.4185596
GM12830	-2.2394192
GM12863	-2.1611052
GM12886	-1.6252134
GM12887	-2.115766
GM12888	-2.2454386
GM12942	-1.4532962
GM12945	-1.8175654
GM12992	-1.8596768
GM13011	-1.5218186
GM13023	-2.2670941
GM13043	-2.0668848
GM13051	-2.4741626
GM13078	-1.9032416
GM13083	-1.8503342
GM13084	-2.1561508
GM13088	-1.9909704
GM13089	-3.4143786
GM13103	-2.0722966
GM13111	-1.7525306
GM13112	-1.5627108
GM13119	-1.9739695
GM13124	-2.265609
GM13128	-3.7398822
GM13177	-2.2873683

GM13180	-1.6468472
GM13194	-1.3358727
GM13202	-1.8879104
GM13205	-1.8190544
GM13215	-0.3237133
GM13219	-2.1215882
GM1322	-3.485881
GM13247	-0.1959157
GM13429	-2.3783684
GM13446	-2.0346396
GM13522	-3.9647021
GM13582	-2.4602628
GM136	-2.0343533
GM13629	-2.274372
GM13710	-2.3326032
GM13749	-2.102668
GM13770	-2.518978
GM13777	-0.9466944
GM13807	-3.3246918
GM13826	-0.505055
GM13939	-2.088593
GM13999	-2.1780531
GM14015	-5.154684
GM14025	-0.6370637
GM14124	-1.8405662
GM14129	-2.2223246
GM14137	-1.9988108
GM14149	-1.969007
GM14151	-3.5242298
GM14164	-2.233773
GM14288	-0.9570375
GM14325	-0.7075877
GM14326	-0.4723558
GM14340	-2.4457536
GM14347	-2.2602048
GM14374	-1.9202456
GM14431	-0.4729767
GM14440	-0.6456528
GM14443	-0.5474763
GM14483	-1.7207935
GM14484	-2.0241842
GM14486	-1.4322984
GM14496	-4.8081136
GM14499	-0.6798987
GM14634	-0.7456656

GM14661	-2.069397
GM14703	-2.113548
GM14725	-2.1022508
GM14743	-2.268476
GM14812	-2.0878048
GM14851	-2.4947004
GM15032	-0.6355076
GM15085	-2.0578575
GM15104	-1.8855495
GM15130	-1.7048492
GM15217	-0.4577382
GM15246	-2.110324
GM1527	-2.2555985
GM15328	-1.9340057
GM15386	-2.22568
GM15401	-2.09962
GM15411	-4.0817404
GM15412	-2.1614742
GM15417	-0.9179697
GM15441	-2.1274374
GM15478	-1.4043577
GM15482	-1.6653545
GM15498	-1.7446833
GM15506	-1.8169184
GM15514	-2.4016747
GM1553	-2.0385678
GM156	-1.626647
GM15628	-2.0172899
GM1564	-2.7836936
GM15688	-2.2216017
GM15706	-1.2466514
GM15762	-1.6800456
GM15850	-2.1562254
GM1587	-2.0759661
GM16039	-1.5997753
GM1604B	-2.9447591
GM16062	-0.4628942
GM16223	-2.537418
GM16294	-2.123219
GM16367	-2.118938
GM16381	-0.5675569
GM16390	-1.5349822
GM16432	-2.0377092
GM16442	-2.215508
GM16487	-1.95261

GM16489	-2.00612
GM16491	-1.0914512
GM16495	-2.1448195
GM16496	-2.2116523
GM16497	-2.9000874
GM16498	-1.4280989
GM16499	-2.2809162
GM16501	-1.987231
GM16505	-0.4192395
GM16508	-1.4818294
GM16509	-2.201549
GM16525	-2.353748
GM16527	-2.3375845
GM16532	-2.1432965
GM16686	-1.5450583
GM16853	-0.3012669
GM1698	-1.1682165
GM17739	-1.789603
GM17745	-3.8606079
GM17748	-0.3612175
GM17751	-2.117887
GM17753	-1.8254066
GM17764	-1.0351238
GM17767	-1.5837569
GM17783	-2.3206391
GM19668	-1.5429828
GM1968	-0.7380228
GM19840	-0.1756039
GM20056	-1.3759384
GM2011	-1.3952086
GM20604	-0.4687138
GM2061	-0.8612871
GM20736	-2.1829848
GM20740	-0.9693637
GM20746	-2.5567093
GM20747	-2.5637593
GM20754	-2.1193376
GM20755	-2.2747674
GM20757	-2.2008314
GM20758	-2.1438367
GM20764	-0.2523427
GM20783	-2.1674914
GM20806	-2.0726247
GM20822	-2.1102293
GM20837	-2.4694424

GM2085	-2.9103317
GM20867	-2.373186
GM20877	-2.0785208
GM20918	-2.0863576
GM20939	-0.3662458
GM21064	-2.1934938
GM2109	-2.231615
GM21276	-2.031717
GM2128	-2.2199101
GM21292	-1.5019236
GM21319	-2.2319036
GM2138	-2.4657977
GM21404	-2.560567
GM21464	-1.3094244
GM21498	-1.8061559
GM21559	-0.7907958
GM21637	-2.012425
GM2164	-2.216341
GM22	-0.6378872
GM2233	-2.306812
GM2240	-0.7937963
GM2270	-2.098588
GM2379	-1.3935285
GM2382	-0.8675771
GM2436	-0.9839387
GM2464	-2.2405689
GM2483	-2.164126
GM2491	-1.9078989
GM2516	-3.3369598
GM2545	-0.6135359
GM2560	-1.9281452
GM2582	-1.886353
GM266	-0.5599697
GM2670	-2.83767
GM2675	-1.6922011
GM2693	-2.2173703
GM2716	-1.4591408
GM2795	-1.7939658
GM2800	-1.441978
GM2814	-3.8187032
GM2933	-5.1514826
GM2984	-2.0244036
GM2990	-2.0693536
GM2A	-2.5987859
GM3014	-0.8929675

GM3045	-1.5817432
GM3052	-2.30161
GM3134	-2.5009296
GM3143	-2.0143442
GM3230	-0.5262044
GM3234	-1.9522219
GM3238	-0.7833772
GM3258	-0.5283942
GM3279	-1.5474877
GM3285	-0.9031985
GM3287	-2.112866
GM3317	-1.1332402
GM3331	-1.6285086
GM3336	-1.055906
GM3367	-1.4266994
GM3404	-1.781163
GM3448	-1.2843933
GM3453	-2.0824127
GM3458	-2.005861
GM3470	-1.4825625
GM3474	-2.2287226
GM3509	-1.8020036
GM3510	-1.7140582
GM3519	-2.0577948
GM355	-3.2126026
GM3561	-0.3021426
GM362	-1.8527977
GM364	-2.1954856
GM3646	-1.973666
GM3669	-2.129632
GM3693	-2.2257907
GM3696	-1.5439043
GM3704	-3.0373292
GM3728	-2.0611615
GM3740	-1.7837837
GM3763	-2.2663336
GM3772	-2.157771
GM3775	-2.2486665
GM3776	-3.4740117
GM3805	-2.0977287
GM382	-1.437845
GM3822	-1.8149097
GM3830	-4.468439
GM3831	-2.0233922
GM3858	-1.1963778

GM3877	-1.9804368
GM3907	-1.746758
GM3942	-1.3064144
GM3948	-2.3417046
GM3960	-2.1240435
GM3961	-2.028786
GM3970	-1.2083006
GM3985	-2.2725244
GM4013	-2.1171467
GM4022	-1.5351334
GM4035	-1.1695843
GM4081	-2.2165554
GM4098	-1.3429432
GM4117	-2.8108394
GM4130	-0.9815888
GM4131	-4.000696
GM4132	-0.1628707
GM4201	-2.3370867
GM4208	-1.2955382
GM4211	-1.6915698
GM4230	-0.8324668
GM4241	-1.7417946
GM4251	-1.960145
GM4278	-1.8062341
GM4279	-2.615914
GM4281	-2.09081
GM4285	-1.9633017
GM4297	-2.203451
GM4303	-3.1374977
GM4319	-2.2266917
GM4340	-0.962359
GM4342	-0.8044372
GM438	-0.7518828
GM4402	-3.5057373
GM4419	-1.1288602
GM4454	-2.1932223
GM4466	-1.1943958
GM4489	-2.778205
GM4491	-1.5725255
GM4524	-1.4674208
GM4532	-1.0504777
GM4567	-2.136315
GM4593	-0.4035592
GM4606	-2.0134676
GM4632	-2.0485957

GM4633	-1.4811025
GM4651	-2.1223166
GM4657	-1.9724836
GM4663	-1.8490846
GM4665	-2.2301455
GM4668	-1.7808816
GM4673	-0.5719385
GM4710	-1.1372192
GM4718	-2.2378364
GM4736	-0.8559873
GM4755	-1.3300757
GM4758	-2.088755
GM4763	-2.6745949
GM4776	-2.0547519
GM4786	-0.9076877
GM4787	-1.1754043
GM4788	-4.7787566
GM4791	-1.7095351
GM4792	-2.0629709
GM4794	-6.951085
GM4802	-0.8447418
GM4841	-2.1927762
GM4847	-2.1486928
GM4858	-2.0360985
GM4862	-3.0348
GM4868	-2.1883478
GM4872	-2.0981748
GM4876	-2.7977247
GM4881	-1.7681048
GM4889	-0.5190706
GM4894	-2.0853834
GM4906	-1.9241083
GM4922	-0.5623887
GM4937	-2.0673137
GM4951	-1.2255139
GM4952	-12.935814
GM4963	-0.5300064
GM4969	-0.4111178
GM4975	-0.5727236
GM4980	-1.5178258
GM4988	-1.9385424
GM4994	-1.6635463
GM4997	-0.5673814
GM5	-1.2776556
GM5067	-2.0909917

GM5072	-2.208889
GM5079	-1.6164875
GM5083	-1.9545536
GM5087	-2.1764212
GM5089	-2.398176
GM5091	-2.3392189
GM5093	-0.7934506
GM5094	-2.131291
GM5095	-1.3742466
GM5099	-2.0352864
GM5108	-1.9656415
GM5112	-1.7584307
GM5114	-1.8304379
GM5118	-2.1904168
GM5122	-2.0748925
GM5132	-2.27603
GM5134	-1.8495138
GM5142	-2.293767
GM5148	-1.9611118
GM5155	-2.0892036
GM5294	-0.749563
GM5296	-1.0493007
GM53	-2.2042274
GM534	-2.0573645
GM5346	-2.6015463
GM5347	-2.1175969
GM5382	-1.343688
GM5414	-2.274317
GM5420	-2.2085025
GM5427	-2.2389226
GM5429	-2.1843083
GM5432	-2.0647678
GM5441	-1.0915818
GM5445	-0.3352523
GM5447	-1.1905665
GM5453	-0.4916253
GM5458	-0.4734395
GM5460	-2.1475208
GM5468	-1.5204458
GM5475	-1.0664835
GM5477	-2.0677161
GM5478	-3.0304196
GM5493	-2.057594
GM550	-2.1887848
GM5523	-1.6412101

GM5524	-9.190998
GM5526	-0.6268616
GM5528	-0.2443047
GM553	-1.8215287
GM5531	-3.5473955
GM5533	-2.9613252
GM5538	-2.228336
GM5544	-2.0667663
GM5549	-2.5717306
GM5553	-0.9675877
GM5563	-2.0691056
GM5577	-2.1705265
GM5585	-3.8642945
GM5589	-2.1322644
GM5591	-2.0652404
GM5592	-2.141291
GM5593	-1.3210092
GM5600	-1.5576987
GM5606	-1.881392
GM561	-1.6445241
GM5615	-2.283779
GM5617	-2.8357024
GM5622	-3.5380893
GM5627	-1.8998904
GM5635	-1.618139
GM5636	-2.1060207
GM567	-2.0886605
GM5709	-2.1119564
GM5712	-2.3286712
GM5724	-2.0765197
GM5737	-1.9711866
GM5741	-1.0886493
GM5755	-2.1161819
GM5766	-0.8382359
GM5767	-2.085626
GM5771	-2.1406312
GM5779	-1.1624789
GM5782	-1.767143
GM5785	-0.4641471
GM5792	-1.812316
GM5795	-0.6465757
GM5801	-1.0261316
GM5848	-0.9904518
GM5849	-2.4900544
GM5879	-0.6653228

GM5891	-2.1518388
GM5897	-2.2037847
GM5907	-2.2864475
GM5908	-1.0317116
GM5916	-2.0250778
GM5922	-2.2294312
GM5925	-2.0286899
GM5934	-1.8950896
GM5938	-2.0837343
GM5941	-1.7791038
GM595	-3.5221338
GM5953	-1.5243163
GM597	-1.6289465
GM6040	-2.088262
GM6042	-1.7713959
GM6083	-1.7346582
GM6086	-2.1011715
GM609	-1.4922538
GM6093	-2.075549
GM6109	-0.223836
GM6117	-2.09328
GM6124	-2.0996714
GM6145	-1.9293652
GM6150	-2.361379
GM6155	-0.9246588
GM6195	-0.4542947
GM6209	-1.1254945
GM6211	-0.2882552
GM6213	-1.9676611
GM6219	-1.0697935
GM6238	-1.290276
GM6249	-2.0690722
GM6251	-1.0436163
GM626	-1.8421693
GM6260	-2.3263993
GM6268	-1.0307312
GM6288	-2.093403
GM6289	-2.1063557
GM6297	-2.7268844
GM6313	-2.127991
GM6328	-0.6608434
GM6360	-1.8167717
GM6404	-0.3586869
GM6406	-2.2170136
GM6410	-2.1179333

GM6432	-2.0275643
GM6436	-1.5635598
GM6444	-2.0189223
GM6460	-1.645786
GM6471	-2.3053641
GM648	-1.928483
GM6484	-7.966068
GM6498	-2.4576402
GM6525	-0.7335339
GM6548	-1.5642104
GM6559	-2.188727
GM6566	-1.3117313
GM6578	-0.7232814
GM6588	-2.181803
GM6590	-1.6007643
GM6598	-0.9798362
GM6600	-2.0063627
GM6607	-0.4306197
GM6614	-4.0322084
GM6634	-2.2976704
GM6639	-1.6826291
GM6653	-1.6339438
GM6658	-1.8944035
GM6687	-2.1643791
GM6710	-0.2791762
GM6744	-2.1446347
GM6787	-1.2934384
GM6818	-2.3573892
GM6867	-0.378438
GM6878	-2.100212
GM6890	-1.9975083
GM6899	-0.9267733
GM6921	-1.3910613
GM6927	-2.0614092
GM6938	-2.1847582
GM6943	-1.3156872
GM6958	-2.0838327
GM6961	-2.3211262
GM6980	-2.0733602
GM6981	-2.6319432
GM6987	-1.7717159
GM6994	-1.7650733
GM6999	-2.130631
GM7008	-1.2767107
GM7073	-1.6721685

GM7102	-0.4591703
GM7120	-2.407657
GM7133	-2.0443964
GM7134	-0.6338182
GM7135	-1.932807
GM715	-0.7992952
GM7157	-1.9890218
GM7160	-0.6130853
GM7167	-2.2270713
GM7241	-1.0244818
GM725	-1.5086775
GM7256	-2.8270154
GM7257	-2.262957
GM7271	-2.0780919
GM7276	-2.2091866
GM732	-1.5306215
GM7325	-2.2125564
GM7444	-1.6016324
GM7457	-2.3481047
GM7489	-2.057384
GM7534	-2.6652207
GM7538	-2.6675272
GM7544	-0.3673229
GM7550	-1.6194282
GM7562	-1.7763052
GM7596	-2.3154798
GM7607	-2.1373782
GM7616	-2.6158867
GM7628	-2.071921
GM7634	-0.5519576
GM766	-2.2734034
GM7664	-0.3635783
GM7714	-2.137438
GM7715	-2.1033278
GM773	-1.7468989
GM7759	-2.2429526
GM7789	-3.7289555
GM7849	-1.5164914
GM7854	-1.9417183
GM7897	-2.4737482
GM7903	-2.2657633
GM7936	-1.4070134
GM7972	-1.4862754
GM7978	-2.1798348
GM806	-1.5529857

GM813	-2.2119787
GM8140	-2.0966847
GM8149	-1.5027463
GM815	-2.2930284
GM8154	-2.0685587
GM8179	-2.0322526
GM8210	-0.5521131
GM8221	-3.7636166
GM826	-3.8072612
GM829	-2.210218
GM8298	-2.238201
GM8300	-2.1694503
GM8359	-2.1559207
GM839	-1.891494
GM8437	-0.3817782
GM8479	-1.0887725
GM8493	-0.9530499
GM8526	-0.3648377
GM8541	-2.3498197
GM8579	-2.4750543
GM8587	-0.8908341
GM8633	-1.9737651
GM8672	-1.0568521
GM8700	-1.8311691
GM8709	-2.5935264
GM8738	-3.9235215
GM8773	-2.0054896
GM8787	-2.196655
GM8801	-0.3144817
GM8817	-1.4265993
GM8883	-5.852044
GM8909	-0.8343301
GM8994	-0.9687433
GM9	-0.9353132
GM9054	-1.5616553
GM9059	-1.9335775
GM9079	-0.4908128
GM9112	-1.1837199
GM9125	-2.0910583
GM9199	-3.022732
GM9234	-1.2603598
GM9325	-2.195977
GM9385	-0.9512625
GM94	-2.214151
GM9457	-0.3917155

GM9502	-1.637918
GM9548	-2.0785806
GM9558	-1.283787
GM9564	-1.9407003
GM9567	-2.0400333
GM9725	-0.4358571
GM973	-1.7934921
GM9731	-0.6521111
GM9734	-0.6069057
GM9736	-2.1039007
GM9752	-2.3554528
GM9757	-0.3637118
GM9767	-1.8177123
GM9789	-2.1921039
GM9801	-1.9340794
GM9804	-1.2868452
GM9831	-1.4588408
GM9833	-3.383097
GM9835	-2.1898386
GM9860	-1.7646201
GM9861	-2.0987864
GM9871	-2.0429854
GM9872	-1.6865101
GM9873	-1.8371184
GM9878	-0.7779338
GM9886	-2.0884075
GM9891	-2.0761821
GM9895	-1.2058945
GM9903	-2.0426626
GM9909	-3.9055824
GM9916	-2.818585
GM9919	-2.117528
GM9921	-2.2469347
GM9926	-1.9293656
GM9928	-0.7357335
GM9934	-2.3265615
GM9946	-1.1518657
GM9951	-2.0681896
GM9958	-1.2098598
GM9961	-2.1042347
GM9963	-0.6624293
GM9968	-2.074047
GM9973	-1.7094612
GM9979	-0.7524447
GM9984	-2.1518214

GM9992	-0.6535869
GM9997	-0.7227171
GM9999	-2.9013145
GMCL1	-0.5608749
GMEB2	-0.5133853
GML	-1.2887905
GMNN	-1.6976445
GMPPA	-1.3762422
GMPPB	-0.9972177
GMPR2	-0.7229061
GMPS	-0.5922062
GNA11	-1.8534765
GNA12	-1.306171
GNAS	-0.4142647
GNAT1	-4.1709948
GNAT2	-4.4603996
GNB1L	-0.878202
GNB2L1	-0.632318
GNE	-3.7725286
GNG5	-0.4846282
GNG7	-1.115912
GNGT1	-4.596413
GNMT	-9.133684
GNPAT	-0.3703575
GNPDA1	-2.0053625
GNPNAT1	-3.1841779
GNPTG	-1.0023112
GNRHR	-2.538792
GNS	-1.4306026
GOLGA1	-0.1940298
GOLGA5	-0.5579987
GOLGA7	-0.5664468
GOLGA7B	-1.959758
GOLIM4	-1.2858248
GOLT1A	-3.771304
GOPC	-0.7087011
GORASP1	-2.1094866
GORASP2	-1.5044122
GOSR1	-0.6027403
GOSR2	-0.801013
GOT1	-4.0590453
GOT2	-4.089185
GPAA1	-1.3385658
GPAM	-2.2047296
GPAT2	-2.9192803

GPATCH2	-0.9578252
GPATCH3	-0.6489301
GPBAR1	-0.8354187
GPBP1L1	-0.7424259
GPC2	-2.0739064
GPC4	-2.9468603
GPC5	-1.498641
GPC6	-1.1548316
GPD1	-4.9010715
GPD1L	-1.9520531
GPD2	-2.0540895
GPHN	-2.0246897
GPI1	-1.3456321
GPIHBP1	-0.5590687
GPLD1	-3.6871197
GPN1	-1.0333838
GPN2	-0.9586735
GPN3	-0.8445549
GPR101	-1.457026
GPR107	-0.3067069
GPR108	-0.4393363
GPR110	-5.0135546
GPR111	-2.2141216
GPR112	-2.040004
GPR115	-1.7574165
GPR119	-2.1210122
GPR12	-2.2739632
GPR124	-0.163178
GPR125	-1.052012
GPR128	-1.9856586
GPR135	-1.7580681
GPR137	-0.823875
GPR143	-1.7860386
GPR149	-2.1612988
GPR150	-1.6077204
GPR151	-2.004087
GPR155	-1.4101913
GPR156	-0.266943
GPR157	-1.8503599
GPR158	-1.7152445
GPR162	-0.5661454
GPR165	-2.0925326
GPR17	-2.27856
GPR173	-2.510156
GPR180	-1.6032746

GPR182	-2.6074777
GPR19	-1.8030708
GPR37	-2.1096537
GPR37L1	-0.4599521
GPR39	-2.8151484
GPR50	-2.1950169
GPR52	-0.225209
GPR55	-1.9362111
GPR61	-0.8153391
GPR75	-2.1404006
GPR89	-2.718242
GPR98	-1.5529819
GPRC5C	-4.3879337
GPRC5D	-1.3060403
GPRIN1	-1.9752009
GPRIN3	-0.5592158
GPS1	-1.1846561
GPSM2	-2.0461974
GPT	-5.1735487
GPT2	-5.9736433
GPX1	-3.345767
GPX4	-1.8473868
GPX5	-1.9954431
GPX6	-1.7591248
GRAMD1C	-3.172576
GRAMD3	-1.549386
GRB14	-2.9262547
GRB2	-0.3959107
GRB7	-3.5185766
GRCC10	-1.4061155
GREB1L	-3.1200807
GREM1	-0.595408
GRHPR	-5.657858
GRIA2	-1.9781098
GRIA4	-1.9384918
GRID1	-2.5620215
GRID2	-2.0476441
GRIK1	-1.9690876
GRIK2	-1.5576746
GRIK3	-2.1488123
GRIK5	-1.1556084
GRIN2B	-1.2478633
GRIN3A	-1.5848229
GRIN3B	-3.23096
GRINA	-1.2111411

GRIP1OS1	-1.8631854
GRIP1OS2	-2.1298575
GRIP2	-0.7844462
GRK6	-0.2486315
GRM1	-1.3205259
GRM2	-1.6114564
GRM3	-2.21365
GRM7	-2.110415
GRM8	-2.4585142
GRN	-2.3870106
GRPEL1	-3.136713
GRPEL2	-1.0131164
GRPR	-2.8144498
GRSF1	-1.7201109
GRTP1	-3.4040298
GRWD1	-0.7773542
GRXCR1	-1.9384012
GRXCR2	-2.2010722
GSC	-2.0819905
GSC2	-1.7458439
GSDMCL2	-1.2437484
GSDMCL-PS	-2.1710753
GSDMD	-1.2516999
GSG1L	-1.2910724
GSKIP	-0.7865367
GSPT1	-0.672936
GSPT2	-0.2148738
GSR	-1.2310748
GSS	-3.2473683
GSTA2	-3.1897278
GSTA3	-3.4267316
GSTA4	-1.2186217
GSTK1	-3.3518496
GSTM1	-3.4999256
GSTM2	-2.1626415
GSTM3	-4.685863
GSTM4	-3.783958
GSTM5	-3.6836014
GSTM6	-5.7901697
GSTM7	-6.4325113
GSTO1	-1.6197433
GSTP1	-5.101807
GSTP2	-3.6506276
GSTT1	-3.6981602
GSTT2	-4.5341806

GSTT3	-3.8631988
GSTZ1	-5.296144
GSX2	-2.2620175
GTF2A1L	-0.8592229
GTF2A2	-1.2084761
GTF2E1	-1.1881576
GTF2E2	-0.3836417
GTF2H2	-0.7639179
GTF2H3	-1.1204243
GTF2H4	-1.9530149
GTF2H5	-0.6917219
GTF2I	-0.9590583
GTF2IRD1	-1.141799
GTF2IRD2	-0.5637183
GTF3A	-1.7230325
GTF3C1	-0.9304028
GTF3C2	-0.7080064
GTF3C3	-0.6942134
GTF3C4	-0.5938511
GTF3C6	-0.7003608
GTL3	-0.6197152
GTPBP1	-0.4368825
GTPBP10	-1.5675507
GTPBP3	-0.4640136
GTPBP6	-0.223907
GTPBP8	-1.1702862
GTSF1L	-2.0117254
GUCA1A	-2.7411907
GUCA1B	-0.6379829
GUCA2A	-1.0895984
GUCA2B	-3.2482758
GUCD1	-3.0687199
GUCY1B2	-3.6472297
GUCY2C	-4.0870457
GUCY2G	-1.9857807
GUF1	-0.8989644
GUK1	-1.3758292
GULO	-14.333725
GUSB	-0.5861011
GXYLT2	-2.0766363
GYK	-1.3395271
GYKL1	-1.538693
GYLTL1B	-1.0076852
GYPC	-0.8447809
GYS2	-11.134899

GZF1	-1.1872911
GZMD	-1.9643366
GZME	-3.2416673
GZMF	-2.4358807
GZMG	-2.2571104
GZMN	-2.0320907
H13	-1.293004
H19	-6.917817
H1F0	-2.81962
H1FNT	-1.5759251
H1FOO	-0.6991286
H1FX	-1.5763702
H2-AA	-0.2454557
H2-AB1	-1.005095
H2AFB1	-2.132865
H2AFB2	-2.3167505
H2AFV	-1.5217247
H2AFX	-0.7646837
H2AFY	-0.4906645
H2-DMA	-1.3006358
H2-DMB1	-0.4362612
H2-EA-PS	-1.0828285
H2-EB1	-1.3388247
H2-K1	-1.1566625
H2-K2	-0.5773635
H2-KE2	-0.7112999
H2-KE6	-2.566145
H2-M1	-2.1945148
H2-M10.1	-1.835038
H2-M10.2	-2.0692663
H2-M10.3	-2.182688
H2-M10.6	-1.606369
H2-M11	-1.0682452
H2-M2	-1.3070097
H2-M9	-1.9039092
H2-OA	-0.689353
H2-Q1	-4.4323506
H2-Q10	-3.8072104
H2-Q2	-1.3005748
H2-Q4	-0.2424116
H2-Q7	-1.1424327
H2-Q8	-0.8971205
H2-T10	-2.3727608
H2-T23	-1.5550623
H2-T3	-1.4076395

H2-T9	-2.628498
H6PD	-1.8828945
HAAO	-7.767494
HABP2	-11.769136
HABP4	-2.35292
HACL1	-7.8250833
HADH	-4.3632107
HADHA	-2.1455264
HADHB	-3.0272074
HAGH	-4.3305283
HAGHL	-0.761724
HAL	-6.5794106
HAMP	-7.701992
HAMP2	-13.739183
HAND1	-2.2432504
HAND2	-5.379454
HAO1	-12.035139
HAO2	-9.7947855
HAPLN1	-0.5476141
HAPLN2	-1.9148636
HAPLN4	-6.024231
HARBI1	-1.8758016
HARS	-0.8402157
HARS2	-1.2141142
HAS2OS	-0.503875
HAUS1	-0.959178
HAUS2	-1.1585579
HAUS4	-2.1599617
HAUS5	-1.4773927
HAUS7	-1.5677135
HAVCR1	-7.4535065
HAX1	-1.441761
HBA-A1	-0.4393086
HBA-A2	-1.117701
HBA-X	-1.9527168
HBB-BH2	-2.5981262
HBP1	-0.4956012
HBS1L	-1.2051454
HC	-2.0202003
HCCS	-0.1443176
HCFC1	-0.8342614
HCFC1R1	-1.4457054
HCN1	-2.2756844
HCN2	-1.6058874
HCN3	-2.0066576

HCRTR2	-2.0772932
HDAC10	-1.4570627
HDAC11	-4.7175655
HDAC3	-1.7262602
HDAC6	-1.5827117
HDAC9	-0.3654091
HDDC3	-2.4457793
HDGF	-0.9702368
HDGFL1	-2.244957
HDGFRP2	-0.7110963
HDHD1A	-0.6807232
HDHD2	-0.5476685
HDHD3	-5.603849
HDLBP	-1.8881583
HEATR9	-1.6354978
HEBP1	-3.7321444
HEBP2	-1.5935009
HECTD1	-0.6106653
HECTD3	-1.5360942
HECW1	-2.014535
HELB	-0.8191495
HELQ	-0.9638677
HELT	-2.0968192
HEMGN	-1.7440863
HEMK1	-2.8266566
HENMT1	-1.6432252
HERC3	-0.4359851
HERPUD1	-0.4368992
HES3	-2.114249
HES6	-2.7950191
HEXA	-1.2609739
HEXB	-0.9583073
HEXDC	-2.0564728
HEXIM1	-0.4317322
HFE	-2.5732527
HFE2	-6.8359537
HFM1	-2.0875072
HGD	-12.682838
HGF	-0.4191103
HGFAC	-8.96076
HGS	-0.6808162
HGSNAT	-1.9953566
HHEX	-3.4530945
HHIPL1	-0.9346342
HHIPL2	-3.4259162

HHLA1	-0.840518
HIATL1	-0.1751847
HIBADH	-2.8540196
HIBCH	-3.76787
HIC1	-0.9739208
HIGD1A	-1.5821333
HIGD1C	-5.2282543
HIGD2A	-1.9173517
HINT1	-1.5221219
HINT2	-3.6521735
HINT3	-2.1166983
HIST1H1C	-2.2481375
HIST1H2BA	-2.0090723
HIST1H2BC	-1.9392915
HIST1H2BM	-1.9897871
HIST1H3A	-0.7594879
HIST1H4I	-0.351366
HIST1H4M	-0.2360039
HIST2H2AA2	-0.2445498
HIST2H3C2	-1.2080421
HIST3H2BB-P	-3.0455964
HJURP	-1.5048985
HK1	-0.484592
HK3	-1.8280001
HLCS	-1.7720485
HLF	-4.1018105
HMBOX1	-1.6069055
HMBS	-2.8189125
HMG20B	-0.8032117
HMGB3	-1.3805246
HMGCL	-4.1555867
HMGCR	-2.5076869
HMGCS1	-2.7039213
HMGCS2	-6.7734976
HMGN1	-0.3982983
HMGN5	-0.7773719
HMOX1	-0.2314019
HMOX2	-1.8755298
HMX1	-1.9449184
HMX2	-2.8137484
HN1L	-0.9008274
HNF1A	-8.135511
HNF1B	-1.5114789
HNF4A	-11.901481
HNF4AOS	-6.0907974

HNF4G	-2.188185
HNMT	-1.7498417
HNRNPA0	-0.2831154
HNRNPA2B1	-0.3733115
HNRNPAB	-0.3094211
HNRNPDL	-0.2465529
HNRNPF	-0.2618055
HOGA1	-5.794197
HOMER1	-0.2207809
HOMER2	-2.7238395
HOMEZ	-0.5947418
HOOK1	-2.215229
HOOK2	-1.3458581
HOTTIP	-2.180602
HOXA10	-2.1004
HOXA11	-1.8918076
HOXA13	-0.5431628
HOXA2	-0.6448948
HOXB1	-2.1498199
HOXB13	-2.159321
HOXC12	-2.571198
HOXC13	-2.1214545
HOXC4	-0.638422
HOXC5	-1.9814367
HOXC6	-2.0531797
HOXC8	-2.4697444
HOXD1	-1.5639939
HOXD11	-1.4261987
HOXD12	-2.191573
HOXD13	-1.9816089
HOXD3	-2.549293
HOXD3OS1	-2.023022
HOXD9	-1.6797066
HP	-1.6084924
HP1BP3	-0.5145216
HPCA	-2.272886
HPCAL4	-2.2270136
HPD	-15.347865
HPDL	-1.5391881
HPGD	-1.8409305
HPN	-5.936186
HPRT	-0.6041126
HPS1	-0.8529422
HPS4	-0.6250892
HPSE	-0.8803659

HPX	-9.765099
HR	-0.8225179
HRAS	-1.8282275
HRASLS	-1.1999176
HRG	-12.397257
HRH4	-1.1366773
HRK	-3.4855683
HRNR	-0.5168691
HRSP12	-6.538277
HS1BP3	-1.3691478
HS2ST1	-1.5605359
HS3ST3A1	-1.082346
HS3ST3B1	-2.2488508
HS3ST4	-2.2660112
HS3ST5	-2.0117652
HS6ST1	-2.9139974
HSBP1	-0.6032605
HSBP1L1	-4.4456844
HSD11B1	-2.884162
HSD11B2	-1.1248562
HSD17B1	-1.3325622
HSD17B10	-3.3639393
HSD17B11	-1.7473497
HSD17B12	-2.4666429
HSD17B13	-10.663885
HSD17B2	-8.913033
HSD17B3	-2.4589138
HSD17B4	-2.665287
HSD17B6	-11.710663
HSD17B7	-3.6598053
HSD3B1	-6.4645786
HSD3B2	-0.7532439
HSD3B3	-13.023983
HSD3B4	-1.4472587
HSD3B5	-2.8136833
HSD3B6	-5.281543
HSD3B7	-4.3792686
HSDL2	-2.5550804
HSF1	-0.1963444
HSF2	-1.7718129
HSF2BP	-0.6429424
HSF3	-1.849426
HSF4	-1.8967252
HSF5	-1.5867383
HSFY2	-2.452839

HSP90B1	-0.4590926
HSPA13	-0.5196257
HSPA14	-0.2560825
HSPA4	-0.7721834
HSPA5	-0.3728814
HSPA8	-0.5795045
HSPA9	-2.2547069
HSPB11	-0.5279627
HSPB6	-1.1700115
HSPB8	-3.2621188
HSPB9	-0.4191077
HSPBAP1	-0.5971758
HSPBP1	-2.1382585
HSPD1	-1.943408
HSPE1	-1.4568686
HTATIP2	-4.343337
HTR1A	-2.0457742
HTR1D	-1.5506108
HTR1F	-1.763809
HTR3A	-1.9151192
HTR3B	-2.1813617
HTR4	-0.5094891
HTR5A	-2.0153043
HTR5B	-1.8353865
HTR6	-1.5487711
HTRA2	-0.6013641
HTRA4	-0.7014639
HYAL1	-1.7347498
HYAL2	-0.7563696
HYAL3	-1.931776
HYAL4	-2.2799232
HYAL5	-3.5958905
HYAL6	-0.9677627
HYI	-4.6280794
HYKK	-4.2555885
HYPK	-0.8399906
IAH1	-2.175447
IAPP	-2.0333557
IARS	-0.5409694
IARS2	-1.6677594
IBA57	-1.6793275
IBSP	-1.9225688
IBTK	-0.8900609
ICA1L	-1.1438487
ICK	-0.4154625

ICT1	-1.654438
ID2	-0.6064096
IDE	-1.244792
IDH1	-2.489819
IDH2	-1.6234846
IDH3A	-0.9221048
IDH3B	-2.3322868
IDH3G	-0.7781162
IDI1	-2.9721742
IDI2	-1.1255441
IDNK	-1.8048506
IDO2	-6.569771
IFFO1	-0.8232966
IFI27	-1.1157222
IFI27L2B	-2.2599778
IFI30	-0.489583
IFITM2	-1.1355772
IFITM3	-1.771555
IFITM7	-1.7403727
IFLTD1	-1.2484088
IFNA11	-1.1173573
IFNA2	-0.5479331
IFNA4	-2.252474
IFNAR2	-0.9445009
IFNB1	-1.8519168
IFNE	-2.1981583
IFRD2	-2.011839
IFT20	-1.1979213
IFT22	-0.4523516
IFT27	-1.1857495
IFT46	-1.3931165
IFT52	-0.9724717
IFT88OS	-2.1535635
IGBP1	-0.8525248
IGBP1B	-3.624101
IGDCC3	-0.7227795
IGDCC4	-4.9218082
IGF1	-5.70242
IGF1OS	-2.5981104
IGF2	-0.6620534
IGF2BP3	-4.75476
IGF2OS	-1.2537875
IGF2R	-0.6640916
IGFALS	-10.087584
IGFBP1	-11.841067

IGFBP2	-4.45442
IGFBP4	-5.151928
IGFL3	-1.5202837
IGHMBP2	-0.6962562
IGJ	-0.4898739
IGKV4-53	-1.3502755
IGKV4-55	-2.1047199
IGKV4-72	-2.0823102
IGKV6-15	-1.9930003
IGLL1	-2.0769856
IGLON5	-1.5984204
IGLV1	-0.5139284
IGSF11	-5.6356053
IGSF5	-2.2965364
IGSF8	-0.9597244
IGSF9	-1.6461921
IGSF9B	-0.7914441
IGTP	-1.0256486
IHH	-1.8455303
IIGP1	-0.8709989
IIGP1B	-2.2154875
IKBKB	-0.4151774
IKBKG	-0.9377165
IKZF3	-2.474184
IL11RA1	-0.7301111
IL12A	-2.1366346
IL13	-2.0099697
IL13RA2	-0.2530925
IL15RA	-1.5374227
IL17RB	-4.430485
IL17RC	-2.0074139
IL18BP	-1.8963017
IL19	-2.345912
IL1F5	-1.8293142
IL1F8	-2.0301244
IL1R1	-0.5298338
IL1RAP	-4.6200943
IL1RAPL1	-2.171574
IL1RAPL2	-2.004772
IL22	-1.5369532
IL22RA1	-2.877095
IL25	-1.0262995
IL27	-2.1806602
IL3	-1.735295
IL31	-2.01528

IL5	-1.704423
IL6RA	-0.8823052
IL9	-2.3748837
IL9R	-1.0246172
ILDR2	-1.4311237
ILKAP	-0.5082717
ILTIFB	-2.9121513
ILVBL	-2.6302624
IMMP1L	-0.8397565
IMMP2L	-5.093136
IMMT	-1.8480458
IMP3	-0.6130819
IMPA1	-1.4242401
IMPA2	-1.0444779
IMPAD1	-0.4252567
IMPDH2	-0.2455163
IMPG1	-1.5296955
IMPG2	-1.8426783
INCA1	-5.5722017
INF2	-1.056407
ING2	-0.246789
ING4	-0.7769494
INHBA	-1.767961
INHBC	-11.24538
INHBE	-7.8670225
INMT	-1.6159115
INO80C	-0.2497501
INPP1	-0.9793658
INPP5E	-0.1892681
INPP5F	-0.5825982
INPPL1	-1.1508074
INSC	-7.7560825
INSIG1	-3.231523
INSIG2	-4.028211
INSR	-1.5498343
INSRR	-0.7985468
INTS1	-1.1681304
INTS10	-0.284616
INTS3	-0.6403863
INTS4	-0.7203221
INTS5	-0.65555
INTS7	-0.3734644
INTU	-0.6552298
INVS	-0.7150426
IP6K1	-0.7359839

IPMK	-1.1347127
IPO11	-0.5440517
IPO4	-1.2722011
IPO5	-0.5015669
IPO8	-0.9449005
IPP	-1.0813417
IPPK	-0.2630582
IPW	-2.073242
IQCC	-1.1610451
IQCE	-0.7153668
IQCF4	-1.5047688
IQCF5	-2.096733
IQCF6	-2.0846107
IQCG	-0.5625134
IQCJ	-1.066289
IQGAP2	-3.7287912
IQGAP3	-1.3592331
IQSEC1	-0.2919407
IQSEC3	-4.142639
IRAK1	-0.2608414
IRAK1BP1	-1.134378
IRAK2	-1.2268372
IREB2	-0.5500975
IRF2BP1	-1.4377971
IRF3	-0.947711
IRF6	-1.9004779
IRF7	-0.5238481
IRGM1	-0.652463
IRGM2	-0.6371059
IRGQ	-0.5149989
IRS1	-2.592447
IRX3OS	-1.0395474
IRX6	-1.9906225
ISCA1	-1.251977
ISCA2	-1.6839395
ISCU	-0.7944994
ISG20L2	-0.4142075
ISL1	-2.0863335
ISL2	-2.1850524
ISM2	-1.6526854
ISOC1	-1.9269872
ISOC2A	-2.6725986
ISOC2B	-1.6655846
ISPD	-1.102845
ISX	-2.075794

ISY1	-0.7952056
ISYNA1	-0.3191514
ITCH	-0.7956009
ITFG1	-1.327065
ITFG2	-1.2345061
ITFG3	-1.8237963
ITGA5	-1.0256848
ITGA9	-0.616725
ITGB1BP1	-0.2188277
ITGB3BP	-1.0052676
ITGB5	-1.573031
ITIH1	-9.430674
ITIH2	-8.804731
ITIH3	-8.709879
ITIH4	-4.3681035
ITM2B	-1.4126005
ITM2C	-0.4162421
ITPA	-1.4007921
ITPK1	-0.9680557
ITPKA	-1.1934195
ITPR2	-1.0021167
ITPR3OS	-1.4845014
ITSN1	-1.8137765
IVD	-3.4406009
IVNS1ABP	-0.3506503
IYD	-5.9172697
IZUMO1	-1.4358373
IZUMO2	-2.8673995
IZUMO4	-1.684526
JADE1	-0.6083241
JAGN1	-2.0059185
JAKMIP2	-3.2230046
JAKMIP3	-1.792131
JAM2	-0.1911783
JKAMP	-1.0831904
JMJD4	-1.513608
JMJD7	-2.1259544
JMJD8	-2.3291578
JOSD1	-1.9660759
JOSD2	-1.9146595
JPH1	-1.7020259
JPH3	-1.2868402
JPH4	-2.0030394
JPX	-0.92629
JRK	-1.1655037

JTB	-1.2959161
JUNOS	-1.5270424
KANK1	-1.4016948
KANSL2	-0.6956015
KARS	-0.9205718
KAT2A	-1.5258155
KAT2B	-1.9359403
KAT8	-0.9744792
KBTBD12	-2.006864
KBTBD13	-1.493443
KBTBD4	-0.5867925
KCMF1	-0.3396277
KCNA2	-2.0620387
KCNA4	-1.6710191
KCNA5	-0.8473988
KCNA6	-0.3612413
KCNA7	-0.2777674
KCNAB2	-0.5230761
KCNAB3	-1.2738755
KCNB1	-1.369674
KCND3OS	-3.2791848
KCNE1	-1.5875685
KCNE1L	-4.188019
KCNF1	-2.7343342
KCNG2	-2.2499247
KCNG3	-0.6440764
KCNG4	-2.3184052
KCNH2	-0.5596635
KCNH4	-0.3177674
KCNH5	-2.1801608
KCNH6	-2.389091
KCNH8	-1.2105889
KCNIP1	-1.4444551
KCNJ1	-2.142074
KCNJ10	-3.8985057
KCNJ12	-0.6752603
KCNJ16	-3.6964624
KCNJ4	-1.8720968
KCNJ9	-2.320086
KCNK15	-1.4881256
KCNK5	-2.6344664
KCNMB4OS1	-2.20277
KCNN2	-6.7439194
KCNQ2	-1.4310372
KCNQ5	-0.2310224

KCNS2	-2.1197944
KCNT2	-2.0872355
KCNV1	-2.2435215
KCNV2	-2.2457755
KCP	-3.2472987
KCTD14	-0.800796
KCTD15	-0.2964382
KCTD16	-2.3610623
KCTD17	-0.4989786
KCTD18	-0.6192637
KCTD2	-0.8254228
KCTD20	-0.3385954
KCTD3	-0.177947
KCTD6	-0.3536892
KCTD7	-1.7753184
KDELC1	-0.5950232
KDELR1	-1.3662539
KDELR2	-1.81604
KDF1	-0.3323956
KDM1B	-1.7058802
KDM4A	-0.2389803
KDM4B	-0.2798686
KDM5D	-2.1413414
KDM8	-0.8545671
KDR	-0.298584
KDSR	-1.0835166
KEAP1	-0.8922057
KEG1	-7.9651966
KERA	-1.9493723
KHDC1A	-2.1798747
KHDC1B	-2.0524824
KHDRBS2	-2.7052402
KHDRBS3	-2.0037894
KHK	-6.3983936
KHNYN	-0.2725959
KHSRP	-1.876401
KIF12	-2.0277004
KIF13B	-1.1633463
KIF17	-0.7460196
KIF18B	-0.7126341
KIF19A	-0.3620024
KIF1B	-1.1259685
KIF1C	-0.8370147
KIF20A	-1.1370058
KIF21A	-2.1512356

KIF22	-0.9268198
KIF24	-0.7035344
KIF2C	-0.9008005
KIF3C	-2.1834264
KIFC1	-1.493078
KIFC2	-0.9445796
KIFC3	-1.5814648
KIFC5B	-0.7542191
KIR3DL1	-1.6770296
KIR3DL2	-2.0088122
KIRREL2	-2.3128688
KIRREL3	-0.6670218
KIS2	-1.6919079
KISS1	-3.4984217
KL	-1.8217778
KLB	-9.676165
KLC4	-2.5701418
KLF1	-4.6887503
KLF12	-2.1780398
KLF14	-2.1201808
KLF15	-1.6585183
KLF17	-1.5919359
KLHDC10	-1.5348392
KLHDC2	-1.623467
KLHDC3	-1.3900008
KLHDC7A	-1.8034544
KLHDC7B	-1.1665308
KLHL10	-0.5199626
KLHL12	-0.6768923
KLHL13	-1.6646357
KLHL14	-2.0918994
KLHL15	-0.2785447
KLHL17	-0.3691039
KLHL22	-0.6739979
KLHL25	-0.6419592
KLHL26	-0.4139805
KLHL3	-1.8044176
KLHL36	-1.3335109
KLHL4	-2.2178419
KLHL40	-2.7638001
KLHL8	-0.3504133
KLHL9	-0.573987
KLK12	-1.1524916
KLK15	-3.5264063
KLK1B1	-2.0536091

KLK1B11	-1.283999
KLK1B16	-2.2124472
KLK1B22	-1.0353596
KLK1B4	-0.6283007
KLK1B7-PS	-3.1738367
KLK1B9	-0.9283912
KLK5	-1.9502652
KLKB1	-8.231562
KLRA10	-1.7891619
KLRA19	-1.9627204
KLRB1-PS1	-1.5901487
KMO	-5.6546836
KNCN	-2.1404452
KNG1	-12.132267
KNG2	-13.23794
KNOP1	-0.8720603
KNSTRN	-0.4510422
KNTC1	-0.8935657
KPNA1	-0.3733902
KPNA2	-0.7158246
KPNA6	-0.3616047
KPNA7	-2.2904649
KPNB1	-0.8967905
KPRP	-1.4684567
KPTN	-1.7716918
KRBA1	-1.1564102
KREMEN2	-0.9692154
KRT1	-1.2174635
KRT10	-0.6288559
KRT12	-1.9133842
KRT18	-2.3049154
KRT20	-1.8806016
KRT25	-2.2132316
KRT28	-1.1609044
KRT33B	-2.270111
KRT34	-2.0499682
KRT35	-2.1323645
KRT36	-1.3447928
KRT40	-2.2079144
KRT71	-1.6920393
KRT72	-1.4980552
KRT73	-2.0770888
KRT74	-2.3519123
KRT76	-2.0950615
KRT8	-2.6507707

KRT84	-2.260809
KRT85	-0.5799818
KRTAP11-1	-0.7797856
KRTAP13	-2.0809264
KRTAP1-3	-2.188259
KRTAP13-1	-2.2889209
KRTAP14	-2.171632
KRTAP15	-2.9666605
KRTAP1-5	-2.1704113
KRTAP19-1	-2.329049
KRTAP19-3	-2.6819944
KRTAP19-5	-0.8833206
KRTAP19-9B	-2.1378422
KRTAP21-1	-2.9882565
KRTAP22-2	-2.1280036
KRTAP2-4	-2.1421502
KRTAP24-1	-1.9748883
KRTAP26-1	-2.085944
KRTAP28-10	-2.3349216
KRTAP3-1	-1.7426038
KRTAP31-2	-2.3129897
KRTAP3-2	-1.76051
KRTAP3-3	-2.02349
KRTAP4-2	-3.5351107
KRTAP4-6	-2.0682535
KRTAP4-7	-1.553766
KRTAP4-8	-0.8606043
KRTAP5-2	-0.1979942
KRTAP6-1	-2.2470927
KRTAP6-2	-2.0327535
KRTAP6-5	-2.1049619
KRTAP7-1	-2.2049253
KRTAP9-1	-0.3340414
KRTAP9-5	-1.7045765
KRTCAP2	-0.9884138
KRTDAP	-1.6293936
KSR2	-1.1946964
KTI12	-1.3278742
KXD1	-2.4715176
KY	-2.1239681
KYNU	-5.724908
L2HGDH	-3.951832
L3HYPDH	-4.0438294
L3MBTL1	-1.7544734
L3MBTL2	-1.6430459

L3MBTL4	-1.680121
L7RN6	-0.4133911
LACC1	-0.3213873
LACE1	-1.5861733
LACTB	-2.9486113
LACTB2	-3.5272512
LAGE3	-1.8218489
LAMA1	-1.8901186
LAMP1	-1.4957395
LAMP2	-1.3889394
LAMP5	-2.152463
LAMTOR1	-1.7248807
LAMTOR2	-1.683157
LAMTOR4	-2.094709
LAMTOR5	-0.4287977
LANCL1	-0.6885963
LAP3	-1.6493783
LARP1	-0.8188133
LARP1B	-2.5275087
LARP4	-0.7497907
LARS	-0.5954251
LARS2	-1.1063848
LAS1L	-1.0628805
LASP1	-0.4996266
LBP	-2.4277244
LCAT	-10.102018
LCE1A1	-1.9872725
LCE1A2	-0.6518292
LCE1D	-2.1956947
LCE1F	-2.1022968
LCE1G	-2.8546467
LCE1H	-2.1750908
LCE1I	-1.9031711
LCE1J	-0.4261203
LCE1K	-1.207694
LCE1L	-2.3575547
LCE3A	-2.290801
LCE3D	-1.3523972
LCE3E	-1.948838
LCE3F	-2.7288651
LCLAT1	-2.1558895
LCMT1	-1.328064
LCMT2	-1.6667109
LCN12	-0.9548788
LCN4	-2.1167612

LCN5	-1.6304319
LDB1	-0.9758143
LDHA	-1.7893863
LDHC	-0.5423307
LDHD	-4.9426327
LDLR	-2.4253745
LDLRAD2	-2.234874
LDLRAD3	-1.8032441
LDLRAP1	-2.2426138
LEAP2	-9.635433
LECT1	-2.8229008
LECT2	-13.810886
LEFTY1	-3.7228003
LEMD1	-2.2375689
LEMD2	-0.3514986
LENEP	-1.3808951
LENG9	-0.2589026
LEP	-2.0746999
LEPR	-0.7137222
LEPRE1	-1.1072693
LEPROT	-0.2683125
LETM1	-0.7555046
LETMD1	-1.5442977
LGALS3BP	-0.8750806
LGALS4	-2.4702768
LGALS6	-1.8394673
LGALS8	-1.3167338
LGALS9	-3.4818277
LGALSL	-0.4047737
LGI1	-2.1639624
LGMN	-1.2363253
LGR4	-3.5332408
LGR5	-4.500259
LGSN	-2.3101892
LHCGR	-2.2023964
LHFPL2	-0.579968
LHFPL4	-0.7099366
LHPP	-3.3332357
LHX2	-5.509113
LHX5	-2.1217542
LHX6	-0.4014106
LIAS	-1.7122059
LIF	-0.4075635
LIFR	-2.6014328
LIG3	-0.571197

LILRA5	-0.9789574
LIM2	-1.2726779
LIMD1	-0.2685266
LIMS2	-0.6894994
LIN28B	-1.4647787
LIN37	-1.0288367
LIN7A	-7.9137144
LIN7B	-1.9761615
LINGO1	-3.8083491
LINGO2	-2.1830466
LINGO4	-0.829813
LINS	-0.1570363
LIPA	-3.193347
LIPC	-8.871091
LIPI	-2.090813
LIPK	-2.2262247
LIPM	-2.270961
LIPO4	-1.7316432
LIPT1	-2.6848855
LIPT2	-1.4396286
LLGL1	-2.112399
LLGL2	-1.6973734
LLPH	-0.3425322
LMAN1	-2.4922023
LMAN2	-1.3817558
LMAN2L	-0.4385128
LMBR1L	-0.3477559
LMBRD1	-1.4744496
LMBRD2	-0.6376498
LMF1	-2.4972887
LMF2	-0.5154924
LMNA	-0.6348896
LMNB2	-5.8827715
LMO3	-1.4666281
LMOD1	-1.9655213
LMOD2	-1.8261385
LMOD3	-0.478399
LMX1A	-2.4646814
LOC1000488	-15.450045
LOC1008617	-1.3976092
LOC1008617	-0.3126407
LOC1008621	-2.2501569
LOC1008624	-2.3565886
LOC1010557	-1.7807498
LOC1010558	-0.436439

LOC1010558	-0.5499425
LOC1010558	-0.6889353
LOC1010559	-0.934969
LOC1010559	-2.204604
LOC1010560	-0.4661036
LOC1010560	-0.3246326
LOC1026318	-0.9740865
LOC1026319	-0.303
LOC1026322	-0.2898417
LOC1026322	-1.110822
LOC1026323	-1.3636124
LOC1026324	-0.5897646
LOC1026324	-2.673913
LOC1026324	-1.211041
LOC1026327	-0.8120947
LOC1026328	-1.7228417
LOC1026330	-1.8869243
LOC1026330	-2.1042285
LOC1026331	-2.206859
LOC1026334	-0.8923059
LOC1026335	-0.1617937
LOC1026336	-1.6825888
LOC1026337	-2.1523135
LOC1026338	-4.045076
LOC1026340	-0.2853961
LOC1026341	-1.606318
LOC1026341	-0.5163198
LOC1026344	-1.9296722
LOC1026345	-2.7424061
LOC1026345	-1.5035212
LOC1026347	-0.5943995
LOC1026349	-1.9771147
LOC1026353	-1.4940131
LOC1026354	-2.305323
LOC1026355	-2.116058
LOC1026356	-1.9905934
LOC1026357	-1.7433443
LOC1026357	-1.2766671
LOC1026362	-0.9736099
LOC1026366	-0.9225068
LOC1026369	-3.4274616
LOC1026370	-1.5033021
LOC1026371	-0.2054992
LOC1026372	-0.2075291
LOC1026373	-0.7519074

LOC1026376	-1.2375686
LOC1026377	-1.9328387
LOC1026377	-1.2913942
LOC1026378	-1.3351684
LOC1026379	-2.087566
LOC1026379	-1.4518256
LOC1026381	-2.559579
LOC1026382	-2.2147717
LOC1026385	-1.1106615
LOC1026386	-1.0439019
LOC1026386	-2.1108236
LOC1026386	-3.0121415
LOC1026388	-0.3474054
LOC1026388	-6.739231
LOC1026389	-0.3464534
LOC1026389	-0.893796
LOC1026396	-2.3600097
LOC1026396	-2.2734268
LOC1026398	-2.5270429
LOC1026399	-0.388587
LOC1026399	-0.2827973
LOC1026403	-0.249037
LOC1026403	-1.345639
LOC1026407	-2.186055
LOC1026410	-1.3737788
LOC1026410	-0.6598673
LOC1026412	-0.5271094
LOC1026412	-2.1229324
LOC1026412	-0.7026477
LOC1026412	-1.0792098
LOC1026413	-11.185307
LOC1026413	-2.1126237
LOC1026413	-0.9520795
LOC1026413	-0.9217806
LOC1026413	-0.5243735
LOC1026413	-0.4447889
LOC1026413	-1.6887908
LOC1026414	-0.9593301
LOC1026415	-0.3691144
LOC1026415	-4.464671
LOC1026416	-1.5768409
LOC1026416	-3.1292295
LOC1026416	-3.7907577
LOC1026416	-0.8519106
LOC1026416	-4.115067

LOC1026416	-2.0347116
LOC1026417	-2.3142147
LOC1026419	-1.2598305
LOC1026419	-0.6097636
LOC1026420	-2.547426
LOC1026420	-2.1813467
LOC1026420	-2.2038383
LOC1026421	-2.1255298
LOC1026422	-0.2391329
LOC1026422	-1.5572562
LOC1026423	-1.7102997
LOC1026423	-3.131071
LOC1026424	-2.3517382
LOC1026425	-0.7344842
LOC1026426	-1.8065858
LOC1026426	-0.9936743
LOC1026427	-0.629415
LOC1026428	-0.7374568
LOC1026428	-2.154992
LOC1026429	-1.4881153
LOC1026430	-4.9147124
LOC1026431	-1.6067224
LOC1026431	-2.173901
LOC1026432	-0.5436482
LOC171588	-2.356852
LOC547349	-1.555479
LOC630751	-2.027443
LOC675947	-0.9569728
LOH12CR1	-1.7040627
LONP1	-2.284112
LONP2	-2.940127
LONRF1	-0.2575364
LONRF2	-1.7914631
LOR	-1.387281
LOXL2	-0.8736372
LPAR5	-1.9256108
LPAR6	-1.5001984
LPCAT3	-2.0284224
LPCAT4	-2.1300375
LPGAT1	-1.2359028
LPIN1	-1.902731
LPIN3	-0.2805796
LPL	-0.6043205
LPP	-0.3618383
LRAT	-1.3251987

LRBA	-0.6746502
LRCOL1	-0.7879322
LRFN2	-2.120664
LRFN3	-4.8997445
LRFN5	-1.3849599
LRG1	-1.6368685
LRIG1	-1.2708178
LRIG2	-0.4546757
LRIG3	-1.2179193
LRIT1	-7.7080727
LRIT2	-2.6011872
LRP1	-2.3059044
LRP2BP	-2.0679634
LRP3	-3.4879024
LRP5	-1.730691
LRP8OS1	-3.1346092
LRPPRC	-2.587161
LRR1	-0.5503573
LRRC14	-1.2770014
LRRC14B	-1.5082698
LRRC15	-1.8881445
LRRC16B	-1.4664972
LRRC18	-0.724817
LRRC19	-1.6443801
LRRC20	-1.5432172
LRRC24	-6.7919035
LRRC28	-1.7639899
LRRC29	-2.2052088
LRRC3	-7.610231
LRRC30	-1.7299194
LRRC31	-0.4963007
LRRC39	-1.7470994
LRRC3B	-0.7341623
LRRC40	-0.7001336
LRRC41	-1.0899386
LRRC42	-1.6163955
LRRC47	-0.2445374
LRRC4C	-1.6972616
LRRC52	-2.078618
LRRC55	-1.5000033
LRRC57	-0.3364263
LRRC59	-0.4816966
LRRC61	-1.144072
LRRC66	-2.3640406
LRRC69	-2.1764736

LRRC7	-2.801724
LRRC72	-2.066727
LRRC74	-2.0578034
LRRC8D	-1.5220594
LRRC9	-1.740037
LRRD1	-2.1255903
LRRIQ3	-2.6290505
LRRIQ4	-1.9578731
LRRTM1	-0.6236687
LRRTM2	-1.3444538
LRRTM3	-1.6859851
LRRTM4	-1.746916
LRSAM1	-1.1978307
LRTM1	-5.778364
LRWD1	-0.1412554
LSAMP	-0.9985023
LSG1	-0.7441716
LSM1	-1.368362
LSM10	-1.306602
LSM11	-0.9868605
LSM14B	-0.7861753
LSM2	-1.4035258
LSM6	-0.4986615
LSM7	-0.845263
LSR	-3.3736691
LSS	-3.248476
LTA4H	-0.729104
LTB4R2	-0.5858805
LTBR	-1.1996083
LUC7L2	-1.5368133
LUM	-1.720571
LURAP1L	-1.0277805
LUZP2	-2.6277106
LUZP4	-2.2658694
LY6D	-3.8171883
LY6E	-0.2317829
LY6G5C	-1.9847212
LY6G6E	-0.3378916
LY6G6F	-1.0647979
LY86	-0.6346731
LY9	-1.0406146
LY96	-0.9271336
LYAR	-1.8267698
LYG1	-2.0630863
LYG2	-2.0730612

LYPD1	-2.2755086
LYPD4	-2.0709515
LYPD8	-2.0294342
LYPLA1	-2.2821083
LYPLA2	-0.8617821
LYPLAL1	-4.175816
LYRM4	-1.04707
LYRM5	-2.8312807
LYRM7	-1.6417475
LYRM9	-3.172897
LYSMD1	-0.9571123
LYSMD4	-1.1124325
LYZL1	-2.164623
LYZL4	-0.3809364
M6PR	-0.7778416
MAB21L1	-2.1420033
MAB21L2	-1.4377966
MACROD1	-3.3295588
MACROD2	-1.5354929
MAD1L1	-1.6058779
MAD2L1	-0.8462017
MAD2L2	-1.8074074
MADD	-1.8498573
MAF	-2.6052322
MAF1	-0.4093585
MAFB	-3.2470524
MAFG	-0.4309902
MAGEA1	-1.2064357
MAGEA10	-1.949619
MAGEA3	-1.9949939
MAGEA4	-1.7586436
MAGEA5	-2.0881982
MAGEA6	-1.7864516
MAGEB1	-1.4106345
MAGEB16	-1.6966622
MAGEB16-PS	-2.0143445
MAGEB17-PS	-1.8293667
MAGEB18	-2.471115
MAGEB4	-2.056077
MAGEB5	-2.331029
MAGED1	-1.8676333
MAGEH1	-0.5918584
MAGEL2	-0.4051769
MAGIX	-3.8313048
MALSU1	-1.2299385

MAN1A	-2.0632231
MAN1A2	-0.2555728
MAN2A1	-1.420804
MAN2A2	-0.8392639
MAN2B1	-1.5285697
MAN2B2	-2.2394671
MAN2C1	-0.3553963
MAN2C1OS	-1.4139695
MANBA	-1.6595035
MANEA	-2.100366
MANF	-0.5588822
MANSC1	-1.0499225
MANSC4	-1.2642379
MAOA	-0.3041272
MAOB	-4.7473106
MAP1LC3A	-1.2301087
MAP1LC3B	-0.5608592
MAP2K2	-2.1209168
MAP2K3	-1.3503532
MAP2K4	-0.8198743
MAP2K5	-1.2327747
MAP2K6	-3.8454366
MAP2K7	-1.0636177
MAP3K10	-0.4210627
MAP3K11	-0.2739825
MAP3K13	-1.222506
MAP3K4	-1.4402771
MAP3K7CL	-2.2430217
MAP7	-0.2511993
MAPK14	-1.1899123
MAPK15	-1.1423073
MAPK1IP1L	-0.8159132
MAPK3	-0.3933067
MAPK8	-0.6203415
MAPK9	-1.6260147
MAPKAP1	-1.1869159
MAPKAPK5	-1.0088487
MAPRE3	-1.649714
MARCO	-1.4840889
MARS	-1.201767
MARVELD1	-1.603692
MARVELD2	-1.2969356
MAS1	-1.8596811
MASP1	-9.222
MASP2	-7.528864

MAST1	-1.6421266
MAST3	-1.1568027
MASTL	-1.1867547
MAT1A	-7.8664236
MAT2B	-0.3816991
MATK	-1.1313052
MATN1	-2.2537618
MATN3	-1.608393
MAU2	-0.5050831
MAVS	-2.9850554
MB21D2	-1.2050626
MBD3	-1.1088343
MBD3L1	-2.064053
MBD3L2	-2.1669986
MBD6	-0.1709976
MBL1	-8.132347
MBL2	-14.109379
MBLAC1	-1.3406055
MBLAC2	-2.2745848
MBOAT4	-2.0004048
MBOAT7	-0.650559
MC1R	-2.0833204
MC2R	-1.9812393
MC3R	-2.0839734
MC4R	-2.3555956
MCAM	-0.164566
MCAT	-1.8835807
MCC	-0.2524586
MCCC1	-2.1226964
MCCC2	-3.309744
MCEE	-2.6348238
MCF2	-2.2229745
MCFD2	-2.979794
MCHR1	-2.1037602
MCM10	-2.8038292
MCM2	-0.7849608
MCM5	-0.6089244
MCM7	-0.613513
MCM8	-0.6601055
MCMDC2	-3.7076979
MCOLN1	-1.061532
MCPH1	-0.1904104
MCPT1	-2.1148763
MCPT2	-1.9873753
MCPT-PS1	-1.8335001

MCTP2	-0.2888184
MCTS1	-0.7126761
MCTS2	-1.6036611
MCU	-0.8014381
MCUR1	-1.0966024
MDGA2	-2.075048
MDH1	-3.0180054
MDH2	-1.3204346
MDP1	-0.7670207
ME1	-2.4445896
MEA1	-1.5492325
MECOM	-0.6356497
MECR	-2.620893
MED12L	-0.3786318
MED15	-0.2630315
MED16	-0.8339801
MED20	-0.5312161
MED22	-1.3458691
MED24	-0.3344045
MED25	-1.6714287
MED28	-0.4279418
MED29	-1.668385
MED6	-0.3977947
MED8	-1.2765493
MED9	-0.5895391
MEDAG	-1.1352491
MEGF10	-1.2852077
MEI1	-1.0939195
MEI4	-2.100443
MEIOB	-6.915598
MEIS1	-0.1859603
MEIS2	-0.6815176
MEIS3	-0.3440299
MELK	-0.7451067
MEMO1	-1.1699119
MEN1	-0.9972811
MEP1A	-2.0838203
MEP1B	-2.1273098
MERTK	-0.6825714
MESDC2	-0.7059593
MEST	-1.4008186
MET	-1.1305528
METAP1	-0.8988519
METAP1D	-3.0369742
METRN	-1.7485495

METTL1	-0.8756156
METTL11B	-2.6603227
METTL13	-1.9271169
METTL15	-1.4451146
METTL16	-0.4246173
METTL17	-1.2899959
METTL18	-0.7012882
METTL2	-0.4874208
METTL20	-1.2317233
METTL21A	-1.8904352
METTL22	-2.7323363
METTL4	-0.5442102
METTL5	-2.187447
METTL7A2HI	-2.3972058
METTL7A3	-0.8253727
METTL7B	-10.943281
METTL8	-1.1084468
METTL9	-0.5714879
MEX3A	-0.1767712
MFHAS1	-0.5989389
MFI2	-3.553026
MFN1	-1.287097
MFN2	-1.7577386
MFSD1	-0.8064175
MFSD11	-1.0430079
MFSD12	-1.2191238
MFSD2A	-3.6686091
MFSD2B	-0.4480171
MFSD3	-3.2179208
MFSD5	-0.5702176
MFSD7C	-1.708993
MFSD8	-0.52531
MFSD9	-2.496779
MGAM	-3.6299438
MGAT1	-0.9615626
MGAT2	-0.7969723
MGAT4B	-1.649128
MGAT4C	-1.4534009
MGAT5B	-1.4154325
MGLL	-3.3481922
MGME1	-2.534935
MGMT	-4.0656614
MGRN1	-0.5739594
MGST1	-3.1920571
MGST2	-2.2377682

MGST3	-0.9897566
MIA2	-4.1497917
MIA3	-1.5323434
MIB2	-1.0122633
MICAL3	-0.4307823
MICALL2	-1.1094065
MICU1	-0.676887
MICU2	-0.5122256
MID1	-0.4448104
MIEF1	-0.5322189
MIEF2	-1.5570724
MIEN1	-1.75524
MIER2	-1.4235358
MIF	-2.6968617
MIF4GD	-1.1593728
MIIP	-1.4169424
MILL1	-2.2604368
MILR1	-0.9233861
MINA	-0.9088574
MINOS1	-0.8482342
MINPP1	-1.6877232
MIOX	-6.275762
MIP	-3.368656
MIPEP	-1.9196362
MIRG	-1.8247027
MIXL1	-2.1070185
MKKS	-0.4584427
MKL2	-0.4261065
MKLN1	-0.6779985
MKNK2	-0.3688164
MKRN2	-0.9966383
MKRN3	-0.3014069
MKX	-2.986174
MLEC	-1.0352774
MLF2	-1.2377982
MLH1	-1.4113126
MLH3	-0.7743576
MLST8	-0.8316197
MLX	-1.3358245
MLXIPL	-4.6435103
MLYCD	-2.942225
MMAA	-1.3342052
MMAB	-3.056821
MMACHC	-2.1120162
MMADHC	-1.2402678

MMD	-2.467165
MMEL1	-2.0542328
MMGT1	-0.42731
MMP10	-1.8242576
MMP15	-0.7671814
MMP19	-1.6504664
MMP1A	-2.0486653
MMP20	-1.9882684
MMP21	-2.0613613
MMP27	-0.7227085
MMP28	-1.4800332
MMP7	-4.277832
MMS22L	-1.7171631
MN1	-1.5120091
MNAT1	-0.3514829
MND1	-1.914051
MNX1	-2.1445286
MOB2	-1.0734682
MOB3B	-1.4113629
MOBP	-2.7194433
MOCOS	-2.7649016
MOCS1	-1.4903574
MOCS2	-3.0450315
MOCS3	-0.8161311
MOGAT1	-1.1868849
MOGAT2	-3.699207
MOGS	-1.0561714
MON1A	-1.3792171
MORC2A	-0.3112454
MORC4	-1.3139322
MORF4L1	-0.4426913
MORF4L2	-0.5692034
MORN1	-0.9900999
MORN2	-1.4249437
MOS	-2.072307
MOSPD2	-1.0811949
MOSPD3	-2.2608771
MOSPD4	-0.5380464
MOV10	-1.6193023
MOV10L1	-1.6540523
MOXD2	-1.9682779
MPC1	-2.9802656
MPC2	-3.6044722
MPDU1	-2.3179054
MPDZ	-0.2692113

MPEG1	-1.1473827
MPG	-1.3180041
MPI	-1.8435464
MPLKIP	-0.7132926
MPND	-2.054412
MPO	-1.3312235
MPP1	-1.13691
MPP4	-2.8181982
MPP6	-2.95605
MPPE1	-1.5024436
MPST	-3.1805706
MPV17	-1.0759573
MPV17L	-2.5284612
MPV17L2	-1.738317
MPZL2	-3.092513
MR1	-1.2623689
MRAP	-6.5727077
MRAP2	-1.1864853
MRC1	-0.5920386
MREG	-4.957694
MRFAP1	-0.4574838
MRGPRA1	-2.2820346
MRGPRA8	-2.2138052
MRGPRA9	-2.2690969
MRGPRB1	-1.7239933
MRGPRB13	-2.1129675
MRGPRB2	-2.2439384
MRGPRB3	-2.098514
MRGPRB5	-2.2523992
MRGPRB8	-1.8291941
MRGPRD	-2.2365813
MRGPRE	-2.3910797
MRGPRH	-2.1818523
MRGPRX1	-2.120579
MRI1	-2.9080124
MROH2A	-2.8680649
MROH2B	-3.8533046
MROH4	-2.4168897
MROH5	-0.4594476
MROH7	-1.8547256
MROH8	-1.9977002
MRPL1	-1.4073706
MRPL10	-2.0205045
MRPL11	-1.8920102
MRPL12	-3.4213552

MRPL13	-1.8850069
MRPL14	-1.8640218
MRPL15	-2.693037
MRPL16	-2.3410168
MRPL17	-1.8780713
MRPL18	-1.4240384
MRPL19	-1.1154776
MRPL2	-2.2874646
MRPL21	-1.0865793
MRPL22	-1.9529891
MRPL23	-1.757091
MRPL24	-1.2279425
MRPL27	-2.200562
MRPL28	-2.2420506
MRPL30	-1.3212605
MRPL32	-1.0023417
MRPL34	-1.826386
MRPL35	-1.5619879
MRPL36	-1.6059308
MRPL37	-2.2778296
MRPL38	-2.0965972
MRPL39	-1.2394605
MRPL4	-1.1835132
MRPL40	-1.3328729
MRPL41	-1.3225188
MRPL42	-1.7761912
MRPL43	-1.2559657
MRPL44	-1.716136
MRPL45	-1.499795
MRPL46	-1.7269554
MRPL47	-1.2135692
MRPL48	-1.2586689
MRPL49	-1.7096057
MRPL50	-1.7888699
MRPL51	-1.2769628
MRPL52	-0.5873184
MRPL53	-1.0904236
MRPL54	-1.3038511
MRPL55	-2.0602403
MRPL57	-1.2175832
MRPL9	-1.5721583
MRPS10	-1.9643221
MRPS11	-2.1059504
MRPS12	-1.8684096
MRPS14	-1.1050754

MRPS15	-1.3062735
MRPS16	-1.6266785
MRPS17	-0.6126518
MRPS18A	-0.6845412
MRPS18B	-2.2478356
MRPS18C	-0.6624055
MRPS2	-2.1405993
MRPS21	-1.206244
MRPS22	-1.6537242
MRPS23	-1.1603794
MRPS24	-2.1108985
MRPS25	-0.7120395
MRPS26	-1.4365168
MRPS27	-2.8123417
MRPS28	-1.8022165
MRPS30	-1.80268
MRPS31	-2.2112608
MRPS33	-1.088337
MRPS34	-2.4964075
MRPS35	-2.1804829
MRPS36	-0.7987804
MRPS5	-2.4717116
MRPS7	-2.0907912
MRPS9	-0.9250693
MRRF	-0.7816026
MRS2	-0.4401617
MRTO4	-0.6181479
MS4A12	-2.0277505
MS4A13	-2.1615002
MS4A18	-2.018404
MS4A2	-2.1193106
MS4A4C	-1.1372619
MS4A5	-1.9960785
MSANTD4	-0.5265036
MSGN1	-2.0188954
MSH2	-0.5702176
MSH3	-1.1274414
MSH4	-0.8292208
MSH5	-1.2632363
MSL1	-0.1914177
MSMB	-2.0559957
MSMO1	-2.8079104
MSMP	-1.9767544
MSRA	-3.6058178
MSRB1	-1.986877

MSRB2	-1.5768814
MST1	-8.772419
MSTN	-1.8551819
MSTO1	-0.8905106
MT1	-0.843533
MT2	-0.4010024
MT4	-0.8123951
MTA1	-0.893899
MTA3	-1.3483596
MTAG2	-2.3747406
MTAP	-2.806807
MTCH2	-2.2593985
MTERF1A	-0.2843003
MTERFD1	-0.6050825
MTERFD2	-1.2030845
MTERFD3	-1.6624615
MTFMT	-1.2163601
MTFP1	-4.0945034
MTFR1	-2.4313183
MTFR1L	-1.227797
MTFR2	-1.8152809
MTG1	-2.5155635
MTG2	-1.6156101
MTHFD1	-3.530231
MTHFR	-0.8579698
MTHFS	-1.163456
MTHFSD	-1.3356974
MTIF3	-1.9344614
MTL5	-2.529616
MTM1	-0.405046
MTMR1	-0.8586426
MTMR10	-1.3267512
MTMR11	-2.569848
MTMR4	-1.0765324
MTMR7	-4.247406
MTMR9	-0.4851871
MTNR1A	-2.8200054
MTO1	-1.5984302
MTOR	-1.191467
MTPAP	-0.6559863
MTRF1L	-0.3005762
MTSS1	-2.645533
MTTP	-7.845195
MTUS1	-0.7678814
MTX1	-2.1031203

MTX2	-2.0138917
MTX3	-1.4387238
MUC15	-2.0910401
MUC19	-1.7607803
MUC2	-1.822813
MUC3	-2.0450687
MUC6	-1.2354112
MUCL1	-0.5518014
MUG1	-14.301244
MUG2	-14.937177
MUL1	-1.1485362
MUM1	-0.575943
MUP1	-13.916019
MUP17	-14.489769
MUP19	-15.274967
MUP2	-11.380484
MUP20	-12.831737
MUP21	-10.619654
MUP3	-12.830638
MUP4	-12.638853
MUP5	-11.752031
MUP6	-5.0218153
MUP-PS12	-10.595099
MUS81	-1.2107368
MUT	-2.226151
MVB12A	-1.2988577
MVD	-2.0415015
MVK	-2.2709365
MXD3	-1.2911067
MXI1	-0.9170594
MYCBP	-0.9610925
MYCL	-2.8605766
MYCS	-1.9999218
MYCT1	-0.9804373
MYEOV2	-0.6371498
MYF5	-2.1741168
MYF6	-1.661149
MYG1	-1.7566147
MYH1	-2.1914356
MYH13	-1.5381329
MYH15	-1.5802436
MYH2	-2.1638176
MYH4	-1.862709
MYL10	-2.2328095
MYL2	-1.7354791

MYL6	-0.363358
MYL6B	-0.758738
MYLIP	-0.711473
MYLK4	-1.9963117
MYLPF	-0.9923227
MYO15	-1.3813655
MYO16	-1.6440015
MYO19	-0.5247092
MYO1E	-0.3101654
MYO3A	-2.0755985
MYO7A	-2.0404606
MYO7B	-4.709583
MYOD1	-2.0966423
MYOG	-2.1599698
MYOZ1	-1.663522
MYOZ3	-1.4358413
MYPN	-2.295421
MYPOP	-0.9904926
MYRFL	-3.3240094
MYT1	-2.2726116
MYT1L	-1.4031274
MZB1	-0.5854559
MZT2	-2.3687181
N4BP2L1	-1.7988753
N4BP2OS	-1.653141
N6AMT1	-1.1530237
N6AMT2	-0.5699873
NAA10	-1.8648038
NAA11	-3.6650152
NAA15	-0.1814342
NAA20	-0.4833384
NAA35	-0.5545516
NAA38	-1.3699989
NAA50	-0.7270875
NAA60	-1.6941938
NAALADL1	-1.4740007
NAALADL2	-1.8648782
NAB2	-1.2376981
NABP2	-1.1487823
NACA	-0.4055047
NACC1	-0.3123369
NACC2	-0.9496727
NADK	-0.3110237
NADK2	-4.1778626
NADSYN1	-3.556797

NAGA	-1.3113031
NAGK	-0.9478068
NAGLU	-2.7381806
NAGPA	-0.9277496
NAGS	-8.250261
NAIP1	-0.3009899
NALCN	-2.3986826
NANOS3	-3.7088873
NANP	-2.7486267
NANS	-0.5123563
NAP1L2	-1.2889578
NAP1L3	-0.6517091
NAP1L5	-1.7327061
NAPA	-0.9836531
NAPG	-0.5833707
NAPRT1	-4.9224052
NARF	-2.1254776
NARFL	-1.78405
NARG2	-0.4889748
NARS	-0.5324984
NARS2	-0.7095647
NAT1	-2.54178
NAT10	-1.1917386
NAT2	-2.2770987
NAT3	-1.9783659
NAT6	-2.4579506
NAT8	-4.4979353
NAT9	-1.7311192
NAV2	-0.8353128
NBAS	-0.8795803
NBR1	-0.7837091
NCALD	-0.3881595
NCAM2	-1.8488204
NCAPD2	-0.3855739
NCAPD3	-0.2536416
NCAPG	-1.2929044
NCAPG2	-0.6541929
NCAPH2	-0.5567904
NCDN	-0.3799949
NCEH1	-0.4210134
NCKAP1	-0.4392095
NCKIPSD	-0.8137586
NCLN	-1.959548
NCMAP	-0.8170071
NCOA2	-0.738368

NCOA4	-1.4239335
NCOA5	-0.2955136
NCTC1	-1.0159063
ND1	-2.6479793
ND2	-2.1112747
ND3	-2.1108356
ND4	-1.3856893
ND4L	-2.3901644
ND5	-2.3740406
NDC1	-0.9855862
NDFIP1	-1.7238688
NDFIP2	-2.352664
NDNL2	-0.3301606
NDOR1	-0.3935919
NDRG2	-4.786659
NDRG3	-0.6936789
NDST1	-0.8496385
NDST2	-1.7337871
NDUFA1	-1.6517162
NDUFA10	-1.710401
NDUFA11	-1.1736379
NDUFA12	-1.3713017
NDUFA13	-1.9634786
NDUFA2	-1.7816525
NDUFA3	-1.693418
NDUFA4	-2.3831062
NDUFA5	-1.69558
NDUFA6	-1.9801741
NDUFA7	-1.6561289
NDUFA8	-2.0832424
NDUFA9	-2.812232
NDUFAB1	-2.149766
NDUFAF1	-1.7265997
NDUFAF2	-1.5924511
NDUFAF3	-1.1096025
NDUFAF4	-1.8652649
NDUFAF5	-1.2723844
NDUFAF6	-3.3288121
NDUFAF7	-1.2820301
NDUFB10	-2.2510233
NDUFB11	-2.0391617
NDUFB2	-1.6053557
NDUFB3	-0.9774485
NDUFB4	-1.5702863
NDUFB5	-1.8730583

NDUFB6	-2.077939
NDUFB7	-1.628211
NDUFB8	-1.8739924
NDUFB9	-2.2908564
NDUFC1	-1.5643797
NDUFC2	-1.0757122
NDUFS1	-2.062757
NDUFS2	-2.1365504
NDUFS3	-2.046297
NDUFS4	-1.9642339
NDUFS5	-1.5413194
NDUFS6	-2.3270698
NDUFS7	-1.6736913
NDUFS8	-2.2472672
NDUFV1	-2.5878668
NDUFV2	-2.0966687
NDUFV3	-2.4725895
NECAB1	-4.7202506
NECAB2	-0.9237061
NECAP1	-1.2798553
NEDD4	-0.5546966
NEFM	-1.6668615
NEGR1	-0.5161145
NEIL2	-0.7500296
NEIL3	-0.3851962
NEK10	-2.1109767
NEK2	-1.7012188
NEK3	-0.557508
NEK4	-1.0162077
NEK6	-1.5056963
NELFA	-0.5347099
NELFCD	-1.8021517
NELFE	-1.2867436
NELL1	-2.109274
NESPAS	-2.188543
NETO1	-1.8476706
NEU1	-1.8864098
NEU2	-3.0826952
NEU4	-2.0399878
NEURL1B	-1.8621812
NEURL2	-1.6197548
NEUROD1	-2.9631996
NEUROD4	-2.3332164
NEUROD6	-2.2037365
NEUROG2	-2.1088545

NFIA	-0.8511274
NFIB	-0.6633506
NFIC	-1.5448985
NFIX	-1.1183233
NFKBIB	-0.2683125
NFKBIL1	-0.5985704
NFS1	-2.0253587
NFU1	-1.6808
NFX1	-0.6488442
NFXL1	-0.6769052
NFYB	-0.7941594
NFYC	-1.052495
NGEF	-4.3940153
NGF	-2.1710212
NGFR	-3.5129864
NGRN	-0.8203845
NHEJ1	-1.1372995
NHLH2	-2.1024652
NHLRC1	-2.1106188
NHLRC3	-1.0607488
NHP2	-0.5973826
NHSL1	-0.6502979
NICN1	-2.7619793
NIF3L1	-1.5919032
NIFK	-0.6911535
NINJ1	-1.4969897
NIPA2	-0.6424036
NIPAL1	-4.6667356
NIPAL4	-2.0270433
NIPSNAP1	-7.4389677
NIPSNAP3A	-2.1393514
NIPSNAP3B	-2.2958722
NISCH	-0.2403626
NIT1	-2.6719394
NIT2	-5.136003
NKD2	-2.8109593
NKIRAS1	-1.7532864
NKIRAS2	-0.8751278
NKX1-2	-2.352561
NKX2-2	-1.7946303
NKX2-3	-0.5657876
NKX2-6	-1.6434557
NKX2-9	-1.8133473
NKX3-1	-2.2027452
NKX6-2	-3.8494706

NKX6-3	-2.3962173
NLE1	-1.0755391
NLGN1	-2.1124923
NLN	-1.6443238
NLRP12	-0.5077701
NLRP14	-2.6219263
NLRP1B	-0.6031432
NLRP2	-2.2318354
NLRP4A	-1.5536122
NLRP4B	-2.1155267
NLRP4E	-2.2205267
NLRP4G	-1.8378825
NLRP5-PS	-1.807766
NLRP6	-7.3265295
NLRP9A	-1.605684
NLRP9B	-2.5139475
NLRP9C	-1.0955856
NLRX1	-0.9904938
NMD3	-0.9588342
NME1	-1.3878374
NME2	-1.9770021
NME4	-2.6487012
NME6	-2.65984
NME7	-1.0326257
NMI	-0.2993808
NMNAT1	-1.8396611
NMNAT3	-2.6628842
NMRAL1	-2.7623487
NMRK1	-3.7239594
NMS	-1.7186456
NMT1	-0.5757427
NMU	-1.0088472
NNMT	-4.3287582
NNT	-2.7362995
NOA1	-0.3163471
NOB1	-0.4258084
NOBOX	-1.804621
NOC2L	-0.8507891
NOC4L	-0.4120464
NODAL	-1.4898448
NOL12	-0.6143022
NOL4	-1.8150904
NOL6	-0.9411321
NOL7	-0.9293132
NOMO1	-0.469574

NONO	-0.4774218
NOP10	-0.7726784
NOP16	-2.1448274
NOP2	-0.3308325
NOS1	-1.2672062
NOS1AP	-0.4997709
NOSIP	-0.7444644
NOTO	-1.6842744
NOTUM	-3.0793223
NOTUMOS	-5.8891716
NOX3	-2.1012583
NOXA1	-1.0807133
NPAS3	-2.0339277
NPAT	-1.684228
NPB	-1.8238411
NPC1	-2.8974113
NPC1L1	-1.8139617
NPEPL1	-2.4925761
NPHP3	-0.6455772
NPHS1OS	-2.1045969
NPLOC4	-1.6408839
NPM3-PS1	-1.6403348
NPRL2	-1.3302321
NPRL3	-1.8933821
NPS	-1.6661072
NPTX1	-2.292667
NPTX2	-1.6808553
NPY	-2.3117232
NPY2R	-1.9934754
NPY5R	-1.9937756
NPY6R	-2.1160858
NQO1	-3.3271618
NQO2	-4.1041617
NR0B1	-1.9431725
NR0B2	-7.242122
NR1D1	-1.8713291
NR1D2	-1.4153671
NR1H3	-2.2385755
NR1H4	-9.597657
NR1H5	-4.9192867
NR1I2	-9.599568
NR1I3	-8.329412
NR2C1	-0.5981112
NR2C2AP	-1.6221137
NR2E1	-2.1672125

NR2E3	-2.110785
NR2F2	-0.6888766
NR2F6	-3.1158442
NR3C2	-1.841466
NR5A1	-1.8028512
NR5A2	-4.2034454
NRAP	-1.0970085
NRBF2	-1.1433167
NRBP1	-0.7231569
NRBP2	-0.4545159
NRDE2	-0.9012375
NRG3	-2.3108864
NRG4	-3.1159327
NRIP2	-2.6213164
NRK	-2.2099814
NRL	-2.1291115
NRM	-0.4728077
NRN1	-5.3962154
NRP	-1.8034561
NRTN	-0.5296955
NRXN1	-1.3963857
NRXN2	-0.4810948
NSA2	-0.6442838
NSDHL	-3.219153
NSFL1C	-0.5307117
NSL1	-0.8759909
NSMAF	-0.3411012
NSMCE4A	-0.5267034
NSMF	-2.1277661
NSUN2	-0.600194
NSUN3	-1.0095022
NSUN4	-0.7037163
NSUN5	-0.9099321
NT5C	-0.7657223
NT5C1A	-0.8164108
NT5C2	-1.3686953
NT5DC1	-1.2932801
NT5DC3	-0.8420749
NT5E	-2.712194
NT5M	-2.070314
NTAN1	-0.3443751
NTF3	-0.8062594
NTHL1	-1.7484941
NTM	-2.723376
NTMT1	-0.6983891

NTN3	-3.281137
NTNG1	-1.0774727
NTPCR	-1.3517141
NTRK1	-1.5633509
NUBP1	-1.3601995
NUBP2	-1.3190856
NUBPL	-2.4921913
NUCB1	-1.3043356
NUCKS1	-0.5923977
NUDC	-0.5323358
NUDCD1	-0.5454652
NUDCD2	-2.124618
NUDT1	-3.1284838
NUDT12	-3.6233249
NUDT14	-2.0453796
NUDT16	-1.6923118
NUDT16L1	-1.0694637
NUDT18	-2.0718732
NUDT19	-3.0825028
NUDT2	-1.7485247
NUDT21	-0.3072953
NUDT22	-2.0976157
NUDT4	-1.7481966
NUDT5	-1.4591827
NUDT6	-2.6256204
NUDT7	-5.1373405
NUDT8	-3.208405
NUDT9	-0.9697871
NUF2	-0.6062493
NUFIP1	-2.1634326
NUGGC	-2.9667087
NUP155	-1.3017054
NUP210	-0.4662757
NUP37	-0.9596953
NUP43	-0.4954953
NUP50	-0.9703941
NUP62CL	-1.4743252
NUP85	-0.2104063
NUP88	-0.2535873
NUPL2	-0.8654726
NUSAP1	-0.4261227
NUTF2	-0.8399959
NUTM1	-2.0872798
NXF3	-1.9480183
NXF7	-1.6337295

NXN	-0.5308404
NXNL1	-2.4320915
NXPE2	-5.7599974
NXPH1	-1.6183677
NXPH2	-2.1339846
NXT1	-0.2394819
NXT2	-0.6744037
NYAP1	-0.8962567
NYAP2	-1.7283711
OAF	-6.2121763
OARD1	-1.454493
OAS1B	-1.2945437
OAS1E	-1.0174956
OAS1H	-2.154662
OAT	-1.8734455
OAZ1	-1.2859445
OAZ3	-2.8070624
OBOX1	-1.1529875
OBOX3	-1.7987347
OBOX5	-1.7410347
OBOX6	-2.180961
OBP2A	-2.1723933
OC90	-0.6988988
OCA2	-2.277701
OCEL1	-0.5944471
OCIAD1	-1.1433449
OCIAD2	-3.1966922
OCLN	-0.507329
OCSTAMP	-1.524044
ODAM	-1.8819904
ODF1	-3.6771724
ODF3	-1.4672041
ODF3B	-0.7403989
ODF3L1	-1.2498043
ODF4	-2.7759743
OFCC1	-2.0503347
OGDH	-0.7192226
OGDHL	-2.5391061
OGFOD1	-2.0094676
OGFOD2	-1.9577022
OGFOD3	-1.5902147
OGFR	-0.2858319
OGG1	-1.0273492
OIP5	-1.6149218
OIT3	-5.886168

OLA1	-1.0971785
OLFM3	-2.1791809
OLFML1	-2.198086
OLFML2A	-1.5578928
OLFR1	-3.0322976
OLFR10	-1.4316878
OLFR100	-2.233572
OLFR1002	-2.2384987
OLFR1006	-2.1472383
OLFR1008	-1.2495623
OLFR1009	-1.4487448
OLFR101	-2.1233742
OLFR1010	-2.2350378
OLFR1013	-2.2516088
OLFR1014	-1.9595046
OLFR1015	-2.7642925
OLFR1016	-2.1928248
OLFR1019	-1.9836841
OLFR102	-2.095987
OLFR1020	-2.1129155
OLFR1022	-2.1254141
OLFR1023	-2.2979774
OLFR1024	-5.174476
OLFR1028	-1.7399008
OLFR1029	-2.0159736
OLFR1031	-2.1803145
OLFR1033	-1.5225654
OLFR1038-PS	-2.0628364
OLFR1039	-1.8783116
OLFR1044	-2.370577
OLFR1045	-2.0052848
OLFR1047	-2.1499798
OLFR1048	-2.122126
OLFR1049	-5.1331406
OLFR1051	-2.1548853
OLFR1052	-2.2402816
OLFR1053	-2.1509485
OLFR1055	-1.4139218
OLFR1057	-3.203647
OLFR1058	-2.172089
OLFR105-PS	-2.5943422
OLFR1062	-1.9807441
OLFR1066	-2.0469232
OLFR107	-2.3650084
OLFR1076	-2.1140876

OLFR1079	-1.9776006
OLFR108	-2.0235286
OLFR1080	-2.5309508
OLFR1082	-2.309794
OLFR1087	-2.0685346
OLFR109	-2.1580973
OLFR1093	-2.22378
OLFR1094	-2.1182914
OLFR1095	-1.9744153
OLFR1097	-2.087038
OLFR1099	-2.738049
OLFR1100	-1.808162
OLFR1102	-3.1966648
OLFR1104	-2.2084458
OLFR1105	-2.2699878
OLFR1106	-1.9003499
OLFR1109	-2.1557233
OLFR111	-2.5229378
OLFR1111	-2.3030257
OLFR1112	-2.321164
OLFR1115	-0.5987573
OLFR1116-PS	-2.1628053
OLFR1118	-2.0135329
OLFR112	-1.9896655
OLFR1120	-1.8175669
OLFR1121	-2.2854311
OLFR1122	-1.9129891
OLFR1124	-1.5402379
OLFR1126	-2.221461
OLFR1128	-1.7637808
OLFR1129	-2.0754094
OLFR113	-2.111443
OLFR1130	-2.2274659
OLFR1132	-2.3088307
OLFR1133	-2.0922115
OLFR1141	-1.171726
OLFR1145	-1.8522081
OLFR1148	-2.0136323
OLFR1153	-3.8917267
OLFR1155	-2.0631323
OLFR1158	-1.6061919
OLFR116	-1.9479437
OLFR1160	-2.2928758
OLFR1161	-2.0675828
OLFR1163	-2.1538122

OLFR1164	-2.2138805
OLFR1166	-2.239621
OLFR1167	-2.1833944
OLFR117	-2.2816157
OLFR1170	-2.1103034
OLFR1173	-2.3434002
OLFR1176	-2.219259
OLFR1178	-2.3497095
OLFR1179	-3.044803
OLFR118	-2.9623735
OLFR1181	-2.661314
OLFR1182	-2.0899866
OLFR1183	-2.0736356
OLFR1186	-2.638392
OLFR1188	-2.1111593
OLFR1189	-2.490815
OLFR119	-2.3406332
OLFR1195	-2.2951055
OLFR1196	-2.2716727
OLFR1197	-1.8865128
OLFR1200	-2.281465
OLFR1201	-2.3517241
OLFR1204	-2.1963377
OLFR1205	-2.1043968
OLFR1206	-3.4386218
OLFR1208	-2.2594986
OLFR1209	-2.258708
OLFR121	-1.7656872
OLFR1211	-2.138239
OLFR1212	-2.2099643
OLFR1213	-2.1652768
OLFR1216	-2.0466473
OLFR1217	-2.1478329
OLFR1218	-2.068605
OLFR1219	-2.2197342
OLFR122	-0.2873659
OLFR1221	-2.2486897
OLFR1222	-2.0728211
OLFR1223	-2.3072567
OLFR1225	-2.0919976
OLFR1226	-1.8163528
OLFR1228	-2.2258902
OLFR123	-1.6045346
OLFR1230	-1.7317743
OLFR1231	-2.5873916

OLFR1233	-2.0929327
OLFR1239	-2.327015
OLFR124	-1.5119834
OLFR1240	-2.0011766
OLFR1242	-2.0647907
OLFR1245	-3.0766983
OLFR1246	-2.3719997
OLFR1247	-2.2473161
OLFR1248	-2.1023118
OLFR1249	-1.0591378
OLFR125	-2.0159173
OLFR1252	-0.4198916
OLFR1253	-2.188156
OLFR1254	-2.1607969
OLFR1255	-5.17395
OLFR1256	-2.0822709
OLFR1257	-1.079428
OLFR1258	-1.46609
OLFR126	-2.4031405
OLFR1260	-5.158141
OLFR1263	-2.1973205
OLFR1265	-1.6665223
OLFR127	-2.008329
OLFR1270	-2.1159115
OLFR1272	-2.0683355
OLFR1275	-1.9608355
OLFR1276	-2.1337025
OLFR1277	-2.2627826
OLFR1279	-2.6283324
OLFR128	-4.0373244
OLFR1280	-2.0199764
OLFR1281	-1.9927409
OLFR1283	-2.1991882
OLFR1284	-2.305365
OLFR1285	-2.2228012
OLFR1286	-1.6458232
OLFR1287	-2.402854
OLFR1288	-3.8592975
OLFR1290	-2.3381362
OLFR1294	-2.3027184
OLFR1295	-2.1651936
OLFR1297	-2.2586157
OLFR1298	-2.0207462
OLFR1299	-2.0687702
OLFR1300-PS	-1.9751871

OLFR1301	-2.1230614
OLFR1302	-2.173728
OLFR1303	-1.1407661
OLFR1306	-2.2190049
OLFR1307	-1.751276
OLFR1308	-3.8453877
OLFR1309	-2.0022237
OLFR1313	-2.2937899
OLFR1314	-1.6104307
OLFR1316	-2.4770823
OLFR1317	-5.30194
OLFR1320	-2.042935
OLFR1322	-1.749373
OLFR1323	-1.7059045
OLFR1325	-2.120798
OLFR1328	-2.5887113
OLFR1329	-2.233605
OLFR133	-1.5857086
OLFR1337	-2.4334102
OLFR1338	-2.1319447
OLFR134	-1.8942313
OLFR1340	-1.8068404
OLFR1346	-2.2142177
OLFR1347	-0.4635873
OLFR1349	-0.6939709
OLFR135	-1.6413393
OLFR1350	-1.630161
OLFR1351	-2.9469755
OLFR1353	-2.0741613
OLFR1354	-1.6089771
OLFR1356	-2.3145316
OLFR1357	-2.1386607
OLFR1358	-1.0458927
OLFR136	-2.347429
OLFR1360	-1.9968421
OLFR1361	-1.8756094
OLFR1362	-2.7497506
OLFR1366	-3.057937
OLFR137	-2.1549418
OLFR1370	-2.248041
OLFR1377	-2.3019428
OLFR138	-2.3592267
OLFR1385	-2.1604407
OLFR1386	-2.259426
OLFR1387	-2.079692

OLFR1389	-2.0118375
OLFR139	-2.303478
OLFR1390	-2.0832098
OLFR1391	-2.2018359
OLFR1393	-0.827745
OLFR140	-2.2576845
OLFR1406	-2.1602156
OLFR1408	-2.1200995
OLFR1412	-1.7035785
OLFR1414	-3.2638614
OLFR1415	-1.5306208
OLFR1416	-2.0530174
OLFR1418	-2.0494034
OLFR1419	-2.3033717
OLFR142	-2.2426147
OLFR1423	-0.8100989
OLFR1424	-2.0492842
OLFR1425	-1.6221704
OLFR1426	-2.212263
OLFR1427	-2.190315
OLFR143	-2.2236762
OLFR1431	-1.7617092
OLFR1436	-1.5149031
OLFR1440	-2.2335901
OLFR1441	-1.7746177
OLFR1442	-2.2219148
OLFR1444	-2.257514
OLFR1445	-1.177238
OLFR1447	-2.012273
OLFR1448	-2.2813888
OLFR1449	-1.6582987
OLFR1450	-1.8969047
OLFR1453	-1.9328008
OLFR1457	-2.1873765
OLFR1458	-2.2187126
OLFR1459	-1.0725522
OLFR146	-2.4464457
OLFR1461	-1.6830921
OLFR1462	-2.0700994
OLFR1463	-2.1286504
OLFR1465	-3.676618
OLFR1466	-3.527273
OLFR1467	-2.011735
OLFR1469	-2.1033144
OLFR147	-2.004791

OLFR1471	-1.8712742
OLFR1472	-2.1422997
OLFR1475	-1.9499617
OLFR1477	-2.07086
OLFR148	-2.1482654
OLFR1480	-2.0190492
OLFR1484	-2.0143182
OLFR1489	-1.8788161
OLFR149	-0.7447431
OLFR1491	-2.2063186
OLFR1494	-2.0541754
OLFR1495	-2.1129072
OLFR1499	-2.1938126
OLFR150	-2.0453982
OLFR1500	-0.871233
OLFR1501	-2.070373
OLFR1504	-2.1054652
OLFR1507	-1.3760393
OLFR1509	-3.788544
OLFR151	-1.7198701
OLFR1510	-2.073372
OLFR1511	-2.1988401
OLFR1512	-2.019833
OLFR152	-0.1855183
OLFR153	-2.5725782
OLFR1532-PS	-2.1573186
OLFR1535	-2.0918689
OLFR1537	-2.6806233
OLFR154	-2.216198
OLFR155	-1.9780738
OLFR157	-2.480263
OLFR159	-0.289638
OLFR160	-2.1898992
OLFR164	-2.0615668
OLFR165	-2.2048466
OLFR166	-2.2878754
OLFR167	-2.0310109
OLFR168	-2.0505884
OLFR169	-2.0516067
OLFR17	-2.7433577
OLFR171	-2.1158836
OLFR172	-2.2626102
OLFR173	-2.1531036
OLFR175-PS1	-2.1182728
OLFR176	-3.7747264

OLFR178	-1.9790373
OLFR180	-2.0058966
OLFR181	-2.2033455
OLFR183	-2.0406313
OLFR186	-2.1525748
OLFR187	-1.4030831
OLFR19	-2.290118
OLFR190	-1.8646994
OLFR191	-2.358721
OLFR193	-1.6392109
OLFR194	-2.221857
OLFR195	-4.599084
OLFR196	-2.205007
OLFR197	-2.2420733
OLFR198	-3.4427807
OLFR199	-1.6729975
OLFR2	-1.7667985
OLFR20	-2.861929
OLFR202	-2.1175508
OLFR203	-2.1789677
OLFR204	-2.5281153
OLFR205	-2.3088198
OLFR206	-2.1968627
OLFR207	-1.015481
OLFR209	-1.5785561
OLFR211	-1.5865319
OLFR212	-1.5787511
OLFR214	-2.0355062
OLFR223	-2.295159
OLFR225	-2.1081312
OLFR228	-1.950778
OLFR229	-2.3528433
OLFR22-PS1	-2.0793684
OLFR231	-2.0488527
OLFR235	-2.0713797
OLFR237-PS1	-1.5992634
OLFR24	-2.1452496
OLFR242	-3.2228723
OLFR243	-2.0409858
OLFR248	-4.2793574
OLFR25	-2.3481216
OLFR250	-3.081873
OLFR251	-2.0547676
OLFR262	-2.0037837
OLFR263	-2.3211403

OLFR266	-1.9846287
OLFR27	-2.2822661
OLFR270	-2.2771766
OLFR273	-2.0289848
OLFR275	-3.8148475
OLFR279	-0.8938017
OLFR281	-2.1941035
OLFR282	-1.8240533
OLFR284	-2.2811534
OLFR285	-1.6751194
OLFR286	-2.0849628
OLFR288	-2.0634565
OLFR290	-2.7367911
OLFR291	-2.3677888
OLFR292	-2.0592356
OLFR293	-1.9876773
OLFR294	-2.3013642
OLFR295	-2.1110232
OLFR298	-2.211945
OLFR299	-2.2327218
OLFR29-PS1	-2.4372866
OLFR3	-2.3399184
OLFR303	-2.1681113
OLFR304	-2.1956213
OLFR305	-2.046879
OLFR307	-1.3116591
OLFR309	-2.2707312
OLFR31	-2.3514953
OLFR310	-1.5941973
OLFR311	-2.3670337
OLFR314	-1.8353996
OLFR315	-2.0561938
OLFR316	-1.9371407
OLFR318	-2.3379605
OLFR319	-0.1667869
OLFR320	-1.9513097
OLFR322	-1.7485695
OLFR325	-2.3390894
OLFR329-PS	-2.0013146
OLFR33	-2.2203252
OLFR330	-2.4972584
OLFR331	-1.9925647
OLFR332	-1.3557825
OLFR340	-2.1689014
OLFR341	-1.9987042

OLFR342	-2.2927034
OLFR344	-2.1026092
OLFR346	-2.119561
OLFR347	-2.0577226
OLFR350	-2.1939409
OLFR351	-2.62137
OLFR353	-1.0304966
OLFR354	-1.908412
OLFR355	-3.7237844
OLFR360	-2.3516645
OLFR361	-2.0262103
OLFR366	-2.221736
OLFR368	-1.7846978
OLFR370	-2.0350986
OLFR372	-2.2196126
OLFR373	-1.1045055
OLFR38	-1.6633759
OLFR381	-2.1702137
OLFR384	-2.144825
OLFR385	-2.1196768
OLFR390	-2.8900719
OLFR392	-0.9034309
OLFR393	-2.363076
OLFR394	-1.5634866
OLFR395	-2.07787
OLFR397	-5.2146273
OLFR399	-2.2266166
OLFR401	-1.918659
OLFR402	-2.2556245
OLFR403	-1.8148375
OLFR406	-1.8600984
OLFR411	-2.7284603
OLFR412	-2.1821516
OLFR417	-0.754571
OLFR418-PS1	-2.0135045
OLFR419	-2.1429036
OLFR421-PS1	-2.0474274
OLFR424	-4.770979
OLFR427	-1.9112759
OLFR43	-2.2113664
OLFR434	-1.9468694
OLFR435	-2.1125078
OLFR437	-2.23385
OLFR44	-2.1815023
OLFR441	-2.2983167

OLFR444	-2.070079
OLFR447	-1.7064247
OLFR448	-2.120518
OLFR449	-1.234376
OLFR45	-2.339432
OLFR450	-2.1019163
OLFR452	-2.1552353
OLFR459	-1.9295609
OLFR460	-1.7529087
OLFR461	-2.2445307
OLFR463	-1.5763888
OLFR467	-2.237812
OLFR47	-0.8927777
OLFR472	-1.7054589
OLFR473	-2.1925285
OLFR474	-1.6388073
OLFR478	-2.1136453
OLFR479	-2.191529
OLFR48	-2.278056
OLFR480	-1.7726476
OLFR481	-1.3619127
OLFR483	-1.9836664
OLFR484	-1.6650805
OLFR485	-2.1393144
OLFR486	-2.086578
OLFR487	-2.3435073
OLFR488	-2.122083
OLFR49	-0.8634763
OLFR491	-2.2433825
OLFR492	-2.3007836
OLFR493	-2.3450053
OLFR494	-2.1883113
OLFR497	-1.9949131
OLFR498	-2.0736578
OLFR5	-1.844259
OLFR502	-1.575274
OLFR503	-2.069232
OLFR506	-2.0865736
OLFR507	-2.1836474
OLFR508	-2.2482953
OLFR509	-1.7139266
OLFR51	-2.2430472
OLFR516	-2.0844004
OLFR518	-1.4724045
OLFR519	-2.0340447

OLFR52	-1.9800377
OLFR520	-1.6366241
OLFR522	-0.5424578
OLFR525	-2.209434
OLFR527	-2.2092667
OLFR53	-1.6359403
OLFR530	-2.0232806
OLFR531	-1.9003372
OLFR532	-2.6934106
OLFR533	-1.7837639
OLFR535	-2.1120443
OLFR536	-2.5145507
OLFR538	-4.1441164
OLFR54	-2.2150922
OLFR541	-1.7216575
OLFR543	-1.0124543
OLFR545	-2.00904
OLFR547	-1.447485
OLFR549	-2.3414118
OLFR55	-2.4225712
OLFR550	-2.8590188
OLFR551	-1.0370622
OLFR552	-0.8773062
OLFR554	-2.8137977
OLFR557	-1.9746869
OLFR558	-1.9140427
OLFR559	-0.6850271
OLFR560	-2.2002254
OLFR561	-2.0725281
OLFR564	-2.662387
OLFR566	-1.1861348
OLFR568	-1.75318
OLFR57	-2.0514023
OLFR570	-2.2960973
OLFR572	-2.1849084
OLFR575	-2.1733406
OLFR577	-2.224
OLFR58	-1.8837752
OLFR583	-2.1020765
OLFR584	-2.067725
OLFR586	-1.792886
OLFR589	-1.672023
OLFR59	-2.3662677
OLFR591	-2.1358027
OLFR592	-2.5879726

OLFR594	-2.23649
OLFR596	-2.363767
OLFR598	-1.9629717
OLFR599	-1.5736046
OLFR60	-1.5477815
OLFR600	-2.323495
OLFR601	-1.7138462
OLFR605	-2.7010858
OLFR606	-2.2363708
OLFR607	-1.7323594
OLFR608	-2.1726081
OLFR610	-2.0683267
OLFR611	-1.7464614
OLFR612	-1.2733669
OLFR613	-2.1658888
OLFR615	-1.8177292
OLFR616	-1.7043052
OLFR617	-2.0631475
OLFR618	-2.0268996
OLFR619	-2.2911599
OLFR62	-2.3343375
OLFR620	-2.1555977
OLFR622	-2.0898006
OLFR623	-2.2256944
OLFR624	-2.1820686
OLFR63	-1.4707191
OLFR630	-2.4056923
OLFR631	-2.5209908
OLFR633	-1.9609747
OLFR638	-3.7214394
OLFR639	-2.0856614
OLFR640	-2.1330962
OLFR641	-2.0269246
OLFR642	-1.8270607
OLFR643	-1.6127396
OLFR644	-2.441266
OLFR645	-1.9786959
OLFR646	-2.152955
OLFR648	-2.2204814
OLFR65	-2.0964098
OLFR651	-2.1769092
OLFR653	-1.9374905
OLFR654	-3.7461863
OLFR656	-2.9174995
OLFR657	-5.3511143

OLFR658	-1.9730022
OLFR659	-1.7762375
OLFR66	-2.3475707
OLFR661	-2.1552837
OLFR663	-2.1135588
OLFR666	-1.2048523
OLFR668	-1.9832664
OLFR669	-1.938251
OLFR67	-1.9654412
OLFR671	-0.5121236
OLFR672	-1.9453843
OLFR675	-2.1423857
OLFR676	-1.7934594
OLFR677	-2.0751975
OLFR678	-1.7618015
OLFR68	-2.3524973
OLFR683	-2.0423052
OLFR685	-2.1533957
OLFR686	-1.633975
OLFR687	-2.1858232
OLFR688	-2.099507
OLFR689	-2.1857119
OLFR69	-2.115077
OLFR690	-2.0531
OLFR691	-2.3377025
OLFR692	-1.9856701
OLFR693	-3.1990643
OLFR694	-2.0006566
OLFR697	-2.204984
OLFR698	-2.0924785
OLFR699	-2.3454945
OLFR70	-2.7196727
OLFR701	-1.5814595
OLFR702	-3.7382221
OLFR703	-1.6769996
OLFR704	-2.4614995
OLFR707	-2.4222057
OLFR71	-1.6978285
OLFR711	-2.1019464
OLFR713	-0.9308903
OLFR716	-2.0674262
OLFR722	-1.7935739
OLFR724	-2.2801406
OLFR725	-2.062922
OLFR726	-2.0038688

OLFR727	-2.1892762
OLFR728	-1.3802476
OLFR729	-1.9683177
OLFR73	-0.6080871
OLFR730	-1.863934
OLFR731	-1.9577272
OLFR732	-2.1815276
OLFR733	-2.271842
OLFR734	-2.797322
OLFR736	-2.1015937
OLFR739	-2.0388908
OLFR740	-4.9652524
OLFR742	-2.185119
OLFR743	-2.0388227
OLFR744	-1.7233686
OLFR745	-1.6881566
OLFR746	-2.0272024
OLFR747	-2.2702308
OLFR748	-2.1762476
OLFR749	-2.0619264
OLFR75-PS1	-0.5553892
OLFR761	-2.1160588
OLFR764	-1.5887587
OLFR766	-1.7535987
OLFR767	-1.5262513
OLFR768	-1.7995017
OLFR769	-2.198063
OLFR77	-2.2686315
OLFR770	-2.5006003
OLFR771	-2.2424285
OLFR772	-1.6434264
OLFR773	-3.2184908
OLFR774	-2.042066
OLFR775	-2.2688537
OLFR776	-2.0754373
OLFR78	-1.7016716
OLFR781	-2.1175013
OLFR782	-2.0805097
OLFR784	-2.1342213
OLFR788	-2.170456
OLFR791	-2.1529417
OLFR792	-1.5348115
OLFR794	-2.082907
OLFR796	-3.2006557
OLFR798	-2.2181354

OLFR799	-2.0341606
OLFR8	-1.7579236
OLFR800	-1.4775982
OLFR801	-1.9086642
OLFR802	-2.2524388
OLFR803	-2.1268532
OLFR804	-1.6549196
OLFR805	-1.7694259
OLFR806	-2.8928938
OLFR807	-2.0691676
OLFR808	-2.754278
OLFR809	-2.1168566
OLFR810	-1.992877
OLFR811	-2.194556
OLFR812	-2.1004205
OLFR813	-2.2532115
OLFR815	-1.2556498
OLFR816	-2.0851257
OLFR818	-2.2378004
OLFR820	-1.5697675
OLFR821	-0.9278829
OLFR822	-2.238948
OLFR824	-1.897136
OLFR825	-3.6129034
OLFR826	-1.9269185
OLFR827	-2.043813
OLFR828	-1.5196455
OLFR832	-2.1649435
OLFR834	-1.8348334
OLFR835	-2.0837276
OLFR837	-2.0698311
OLFR843	-1.1774733
OLFR845	-4.276731
OLFR846	-3.009957
OLFR847	-1.764863
OLFR851	-2.0831304
OLFR853	-1.6389365
OLFR854	-1.7832134
OLFR855	-1.6935537
OLFR857	-2.0883186
OLFR859	-2.1281824
OLFR862	-2.1455321
OLFR867	-2.2705173
OLFR868	-1.1884899
OLFR869	-2.372991

OLFR870	-0.9548798
OLFR872	-2.0703251
OLFR873	-3.3266106
OLFR874	-2.203402
OLFR875	-1.9958866
OLFR877	-2.1690173
OLFR878	-1.9187691
OLFR881	-1.6968355
OLFR883	-2.1014736
OLFR884	-1.8051472
OLFR885	-2.0780025
OLFR888	-1.9230657
OLFR889	-3.4179716
OLFR890	-2.2137318
OLFR891	-0.5865648
OLFR893	-2.1154225
OLFR894	-1.932764
OLFR895	-2.072232
OLFR899	-2.0087285
OLFR90	-2.3376267
OLFR900	-2.1533914
OLFR902	-2.2830877
OLFR904	-2.542858
OLFR905	-1.7819705
OLFR908	-2.0400648
OLFR91	-2.143755
OLFR910	-1.8158331
OLFR912	-2.198083
OLFR917	-2.74735
OLFR918	-2.3633046
OLFR919	-1.9863913
OLFR92	-2.132793
OLFR922	-2.5399323
OLFR923	-2.3034556
OLFR924	-1.9990175
OLFR926	-2.0708256
OLFR93	-2.3414643
OLFR930	-2.0551434
OLFR933	-1.9719286
OLFR935	-2.1048295
OLFR936	-2.1127875
OLFR937	-2.284092
OLFR938	-2.2389998
OLFR943	-1.6797433
OLFR944	-2.1024756

OLFR947-PS1	-2.3423667
OLFR948	-2.0095382
OLFR95	-2.8022723
OLFR951	-1.8603821
OLFR952	-2.2363105
OLFR954	-2.1654975
OLFR955	-2.1430984
OLFR959	-2.2867289
OLFR960	-2.1668794
OLFR961	-2.317764
OLFR967	-2.0880735
OLFR968	-2.1269503
OLFR97	-0.7741001
OLFR970	-2.2138264
OLFR971	-1.5191982
OLFR972	-2.044089
OLFR975	-2.1904197
OLFR976	-1.6746557
OLFR982	-2.4241066
OLFR983	-2.0356488
OLFR987	-2.1591697
OLFR988	-2.1878037
OLFR99	-1.8600025
OLFR992	-2.1690001
OLFR993	-2.2615552
OLFR995	-2.1257675
OLFR996	-2.1267004
OLIG1	-5.247711
OMA1	-0.8158321
OMG	-1.6020057
OMP	-0.4932551
OMT2A	-2.1059237
OMT2B	-1.7470064
ONECUT1	-7.1593523
ONECUT2	-9.580016
ONECUT3	-2.0736651
OOEP	-1.461021
OOG3	-1.9376812
OOSP1	-1.9057193
OOSP3	-1.7933292
OPA1	-1.5506783
OPA3	-1.6646552
OPALIN	-2.1979787
OPCML	-1.6893854
OPLAH	-2.8522253

OPN1MW	-1.3875074
OPN1SW	-2.076517
OPN5	-1.0475421
OPRD1	-1.59816
OPTN	-1.4890013
ORAI1	-0.3855505
ORAI3	-0.4968462
ORC3	-0.8808508
ORC5	-1.3423989
ORLY	-1.6992986
ORM1	-7.238154
ORM2	-7.445548
ORM3	-5.463317
ORMDL1	-1.2004733
ORMDL2	-1.2653894
ORMDL3	-2.1405625
OS9	-1.2720747
OSBP	-0.6678929
OSBP2	-1.4009647
OSBPL1A	-3.7117095
OSBPL2	-0.7473345
OSCAR	-1.0897961
OSGEP	-1.1500459
OSGEPL1	-2.4391584
OSGIN1	-5.090073
OST4	-1.6818686
OSTC	-0.9433937
OSTM1	-0.296063
OTC	-9.973651
OTOA	-2.346598
OTOG	-0.4069729
OTOGL	-2.3364563
OTOL1	-2.015315
OTOP2	-1.8783851
OTOP3	-1.5309186
OTOR	-1.9437428
OTOS	-1.2035656
OTP	-2.40427
OTUB1	-1.7718406
OTUB2	-0.6000085
OTUD3	-0.6879215
OTUD6A	-1.6894794
OTUD6B	-0.9144974
OTUD7A	-1.7238708
OTULIN	-1.5563359

OTX1	-2.2262483
OVCA2	-0.5355363
OVOL2	-0.3710361
OVOL3	-2.0312076
OXA1L	-1.2355299
OXGR1	-2.1489496
OXLD1	-3.1837673
OXNAD1	-3.06846
OXR1	-0.600265
OXSM	-2.3201468
P2RX2	-0.3630076
P2RX3	-0.9948728
P2RX4	-0.9930902
P2RX6	-0.896302
P2RX7	-0.2372451
P2RY1	-0.5133891
P2RY12	-0.370815
P2RY4	-2.3202589
P4HB	-3.5347252
PABPC1L2B-F	-1.8242872
PABPC4L	-1.4152339
PABPC6	-2.079081
PACRGL	-1.0785108
PACSIN1	-0.5505538
PACSIN3	-3.1863995
PAFAH1B3	-0.8454194
PAFAH2	-3.3002872
PAGR1A	-0.7958674
PAH	-14.417759
PAICS	-2.128364
PAIP1	-0.2970266
PAK1	-0.2825868
PAK1IP1	-0.6638632
PAK4	-1.5988355
PALB2	-1.1416607
PALLD	-0.8931298
PALM	-0.7625284
PALM3	-1.8543689
PALMD	-1.3554797
PAM16	-1.0062995
PAN2	-0.9676418
PANK1	-6.8358016
PANX3	-2.2887003
PAOX	-3.6817188
PAPD4	-0.347805

PAPD5	-1.0460091
PAPD7	-0.4815779
PAPL	-2.083045
PAPLN	-0.3769329
PAPOLB	-1.7312405
PAPSS2	-4.426286
PAQR3	-1.560039
PAQR7	-2.4004579
PAQR9	-9.355356
PARD3	-0.3431897
PARD3BOS1	-1.9025211
PARD6A	-1.2795081
PARK2	-1.6544456
PARK7	-2.2421627
PARL	-0.2256112
PARN	-1.8109756
PARP1	-1.2902308
PARP12	-0.4655676
PARP16	-3.323489
PARP6	-0.3119235
PARPBP	-0.7160251
PARS2	-2.0680332
PARVG	-0.2285738
PASK	-0.5470851
PATE2	-2.3486826
PATE4	-1.5764413
PATL2	-0.8550303
PAX1	-1.9361615
PAX2	-2.8853118
PAX6	-2.1948156
PAX6OS1	-1.9883139
PAX7	-2.1850328
PAX8	-2.2846138
PAX9	-1.7637293
PBDC1	-0.6977487
PBK	-1.458991
PBLD1	-1.1547122
PBLD2	-8.255474
PBP2	-2.0289006
PCBD1	-6.1549454
PCBD2	-1.2343354
PCBP1	-0.2077246
PCBP2	-0.3801141
PCBP4	-2.325158
PCCA	-2.763389

PCCB	-1.2749407
PCDH1	-2.2371278
PCDH10	-1.598741
PCDH11X	-1.029285
PCDH12	-2.368043
PCDH15	-2.0671928
PCDH8	-2.0155177
PCDHA11	-2.0356007
PCDHAC1	-1.9511714
PCDHB12	-0.7563598
PCED1A	-1.303946
PCGF1	-0.9058836
PCGF6	-1.5839252
PCID2	-0.9590812
PCK1	-7.49684
PCLO	-0.5044899
PCMTD1	-1.8668756
PCNXL3	-0.6984825
PCP2	-1.6909151
PCP4L1	-2.8086414
PCSK1N	-1.149004
PCSK4	-4.0832386
PCSK6	-5.0451574
PCSK7	-1.0320239
PCSK9	-8.941774
PCTP	-5.9241886
PCX	-4.080582
PCYOX1	-2.8974314
PCYT2	-4.7363505
PDAP1	-1.0837221
PDC	-2.0768697
PDCD2	-1.3262992
PDCD2L	-0.6947079
PDCD5	-0.566988
PDCD6	-1.336865
PDCL	-0.3992991
PDCL2	-2.1620178
PDDC1	-1.7117004
PDE10A	-0.8246052
PDE11A	-0.5349333
PDE1C	-1.2111402
PDE2A	-2.2000651
PDE3B	-3.2744079
PDE4DIP	-1.2073073
PDE6A	-1.412951

PDE6C	-3.461955
PDE6G	-0.4277873
PDE6H	-0.6096122
PDE8A	-1.3780985
PDE9A	-1.5402713
PDF	-1.5086026
PDGFC	-2.3896902
PDHA1	-0.6759386
PDHA2	-2.3279595
PDHB	-1.452601
PDHX	-1.1753569
PDIA4	-1.1019769
PDIA5	-2.8721933
PDIA6	-1.2205606
PDIK1L	-0.2177706
PDILT	-7.32119
PDK1	-3.0560951
PDK2	-3.57656
PDLIM4	-0.8343067
PDP2	-2.8712401
PDPK1	-0.2235227
PDSS1	-0.8634534
PDSS2	-1.9970765
PDX1	-2.0456262
PDXDC1	-0.9029126
PDXK	-2.3137956
PDXK-PS	-2.2830317
PDXP	-3.0547953
PDYN	-1.0486917
PDZD11	-0.6625967
PDZD7	-1.9841695
PDZD9	-1.0152225
PDZK1	-8.508864
PEBP1	-1.1588569
PEBP4	-2.0057993
PECR	-7.0679946
PEF1	-0.9073539
PEG10	-1.1227767
PEG12	-0.647799
PELI2	-0.8512449
PELI3	-1.4386225
PELO	-0.9099398
PELP1	-0.6795635
PEMT	-8.298124
PEO1	-2.6962569

PEPD	-1.6240506
PER2	-0.4770947
PER3	-1.5058455
PERP	-2.8431091
PET112	-3.1953645
PET2	-2.0916827
PEX1	-2.776092
PEX10	-2.122285
PEX11A	-2.3200555
PEX11B	-1.9297667
PEX11G	-4.4299984
PEX12	-1.7957115
PEX13	-1.9633355
PEX14	-2.4894607
PEX16	-4.278851
PEX19	-3.3553066
PEX2	-2.0331779
PEX26	-2.6365952
PEX3	-2.2310996
PEX5	-2.693563
PEX6	-2.2886977
PEX7	-3.2205706
PFDN1	-0.8333683
PFKFB1	-5.5865774
PFKL	-1.8210478
PFKM	-1.681356
PFPL	-1.8206482
PGA5	-2.4341388
PGAM1	-1.8054929
PGAM5	-0.6527333
PGAP2	-0.7246733
PGC	-1.0404725
PGD	-0.8696303
PGK1	-2.3940387
PGLS	-1.9073777
PGLYRP2	-6.369748
PGLYRP3	-2.0044653
PGLYRP4	-1.9134879
PGM2	-4.4406567
PGM3	-1.2874503
PGP	-0.8089304
PGPEP1	-3.8163419
PGR	-1.5658815
PGR15L	-2.044925
PGRMC1	-3.390749

PGRMC2	-0.866663
PHACTR3	-1.2520275
PHACTR4	-0.252945
PHB	-2.386992
PHB2	-2.5881915
PHF10	-0.3324866
PHF14	-0.2493539
PHF5A	-1.2106442
PHF7	-2.278627
PHKA2	-2.0423908
PHKG2	-0.8695741
PHLDA2	-2.4806242
PHLDB3	-1.7318926
PHLPP1	-1.4392295
PHOSPHO2	-1.1305137
PHOX2A	-0.3332913
PHOX2B	-1.4325898
PHPT1	-1.2749004
PHYH	-5.3885236
PHYHD1	-0.8703661
PHYHIPL	-2.161645
PHYKPL	-2.1034214
PI4K2A	-1.506854
PI4KB	-0.4752111
PIANP	-0.1864233
PIAS2	-1.4002371
PIAS4	-1.120574
PICK1	-1.6661475
PID1	-1.8040466
PIDD1	-1.287251
PIGF	-1.3435321
PIGK	-1.5227485
PIGL	-0.6724131
PIGO	-1.7861838
PIGP	-1.0389585
PIGQ	-0.7653995
PIGS	-0.7580757
PIGT	-1.448895
PIGU	-0.985209
PIGV	-0.2715392
PIGX	-0.9729905
PIGYL	-1.8806977
PIGZ	-1.4347908
PIH1D1	-1.42767
PIH1D3	-1.6698234

PIK3C2G	-4.096368
PIK3R2	-1.762145
PIK3R4	-0.3690496
PIKFYVE	-0.4551611
PIN1	-1.0025616
PIN1RT1	-0.9062519
PIN4	-0.3470049
PINC	-2.2149699
PINK1	-2.5215092
PINLYP	-1.7891381
PIP4K2B	-0.5665298
PIP5K1B	-2.0225024
PIP5K1BOS	-2.2242548
PIP5KL1	-0.8361352
PIPOX	-11.32567
PIR	-1.8028688
PIRB	-0.43468
PIRT	-2.0784318
PISD	-0.8215957
PISD-PS1	-0.3417845
PISD-PS2	-1.200849
PITHD1	-0.8039985
PITPNB	-1.3038521
PITPNM2	-0.6878991
PITPNM2OS1	-1.3875463
PITRM1	-0.9138136
PITX1	-1.3592288
PITX3	-0.7836037
PIWIL1	-2.2320058
PJA1	-1.4099708
PKD2L1	-2.3064828
PKDCC	-2.5066147
PKDREJ	-1.710279
PKHD1	-1.0833311
PKHD1L1	-0.5219154
PKLR	-7.5352883
PKP1	-4.9455013
PKP2	-1.3881874
PKP3	-1.3367519
PKP4	-0.4050674
PLA1A	-3.041954
PLA2G12A	-1.2619553
PLA2G12B	-9.31297
PLA2G15	-1.6780663
PLA2G16	-1.0835533

PLA2G2D	-2.1535227
PLA2G4B	-0.4744625
PLA2G4D	-2.1487207
PLA2G6	-2.8163972
PLAA	-0.5984993
PLAC1	-2.162814
PLAC8L1	-2.1689956
PLAG1	-0.5164545
PLBD1	-0.6773357
PLBD2	-1.9783916
PLCL2	-0.7854052
PLCXD1	-1.437165
PLCXD2	-4.718568
PLCZ1	-1.9550619
PLD4	-1.3060489
PLD6	-2.6677108
PLEK2	-2.675691
PLEKHA5	-0.6311159
PLEKHA6	-3.798821
PLEKHA7	-1.3281019
PLEKHA8	-2.128696
PLEKHD1OS	-1.252986
PLEKHF1	-0.6337276
PLEKHG1	-2.471197
PLEKHG3	-1.8413105
PLEKHG6	-3.3524332
PLEKHH2	-0.9347391
PLEKHH3	-0.8906241
PLEKHJ1	-1.1490877
PLEKHN1	-1.1181121
PLG	-12.774703
PLIN2	-2.4498534
PLIN3	-1.0864592
PLIN5	-5.30048
PLK1	-0.7646694
PLK3	-1.0121956
PLK5	-1.348274
PLLP	-1.9020381
PLSCR2	-0.7229652
PLSCR5	-2.1481514
PLTP	-0.8516994
PLXNB1	-1.498436
PLXNB2	-1.2364206
PLXNB3	-0.6634903
PLXNC1	-1.1912422

PM20D1	-6.1883655
PM20D2	-0.6779432
PMCH	-2.2792428
PMEL	-2.0439265
PMF1	-1.3242321
PMFBP1	-2.14665
PMIS2	-1.6971917
PMM2	-2.160253
PMP2	-2.152357
PMPCA	-1.6304741
PMPCB	-2.129642
PMS1	-1.1130698
PMVK	-1.555923
PNKD	-2.4362416
PNKP	-0.2850785
PNLDC1	-2.1577957
PNLIP	-1.8885722
PNLIPRP2	-2.6784508
PNMA1	-1.5292773
PNMA3	-0.7874234
PNMA5	-1.9991047
PNMT	-2.5107067
PNO1	-0.7927771
PNOC	-1.6855478
PNP	-1.067101
PNP2	-0.3525221
PNPLA1	-3.7234871
PNPLA3	-4.2994833
PNPLA5	-5.391627
PNPLA6	-0.8334727
PNPLA7	-4.683184
PNPO	-3.5987082
PNPT1	-0.9659972
PNRC1	-0.2576871
POFUT1	-0.5526304
POGLUT1	-0.6637254
POGZ	-0.4208345
POLA1	-0.4788404
POLD1	-0.2400351
POLD2	-1.344974
POLD4	-1.7535763
POLDIP2	-2.7509818
POLE	-1.1972377
POLE3	-0.6291051
POLE4	-1.4503088

POLG	-1.107472
POLG2	-2.1641881
POLI	-0.3723435
POLL	-1.5031657
POLN	-0.5246069
POLR1A	-0.9347267
POLR1B	-1.0763352
POLR1C	-0.34268
POLR1D	-0.6216707
POLR1E	-0.2118497
POLR2B	-0.8121848
POLR2C	-0.2166734
POLR2D	-0.2878852
POLR2E	-0.9694867
POLR2F	-1.0807462
POLR2G	-0.8732247
POLR2H	-1.1429834
POLR2I	-1.4940748
POLR2J	-1.1859684
POLR2K	-0.506978
POLR2L	-1.7730002
POLR2M	-0.4226594
POLR3A	-1.4629993
POLR3B	-0.6035967
POLR3C	-1.4542408
POLR3D	-0.4333673
POLR3G	-2.4910707
POLR3H	-1.8108006
POLR3K	-0.3178353
POLRMT	-1.604023
POM121L2	-1.5484917
POMC	-0.5278995
POMGNT2	-1.215261
POMK	-1.889894
POMP	-1.0896907
POMT1	-2.401299
POMT2	-1.9694304
PON1	-4.6213217
PON3	-1.0646391
POP1	-0.3808265
POP5	-1.7324457
POP7	-0.4775772
POR	-2.0692878
POT1A	-1.5935473
POTEG	-2.5223076

POU1F1	-1.8781145
POU3F2	-2.172804
POU3F3OS	-2.011157
POU3F4	-2.0494168
POU4F2	-2.1001072
POU4F3	-2.3878288
POU6F1	-0.5114617
PP2D1	-0.2305641
PPA1	-3.1706662
PPA2	-2.3293552
PPAP2B	-1.7158852
PPAP2C	-2.223267
PPAPDC1A	-0.9660325
PPAPDC1B	-1.3625989
PPAPDC3	-0.7859585
PPARA	-4.5568657
PPARG	-0.5456281
PPARGC1A	-2.2286692
PPARGC1B	-3.1269834
PPAT	-0.937582
PPCS	-2.7790356
PPDPF	-2.1433578
PPFIA2	-0.9831452
PPFIA3	-0.8523159
PPFIA4	-0.8618407
PPFIBP2	-0.3307362
PPIA	-0.5812612
PPIB	-2.2655826
PPIE	-1.213973
PPIF	-4.2909822
PPIFOS	-2.1268969
PPIH	-0.4285698
PPIL1	-1.051996
PPIL2	-1.0621443
PPIL3	-0.7974105
PPIP5K2	-0.8060565
PPM1A	-1.2995486
PPM1B	-0.9626293
PPM1G	-0.484056
PPM1K	-1.0724587
PPM1L	-0.7174397
PPME1	-1.0850654
PPNR	-0.2627568
PPOX	-1.8369703
PPP1CA	-1.0518909

PPP1CB	-0.335022
PPP1R11	-0.9673843
PPP1R14B	-0.6696739
PPP1R14C	-0.5184903
PPP1R16A	-0.9489622
PPP1R17	-2.2421405
PPP1R1B	-1.4747887
PPP1R1C	-1.571775
PPP1R2-PS7	-2.0500014
PPP1R2-PS9	-2.1097555
PPP1R35	-1.3447747
PPP1R37	-2.0149584
PPP1R3B	-2.849772
PPP1R3C	-4.348772
PPP1R3E	-1.8205736
PPP1R3FOS	-1.2996423
PPP1R3G	-8.378307
PPP1R7	-0.8568339
PPP2R1A	-0.8694239
PPP2R2COS	-2.1434703
PPP2R2D	-0.3349266
PPP2R3A	-0.1947069
PPP2R3C	-1.1009755
PPP2R4	-2.570737
PPP2R5A	-1.1055617
PPP2R5D	-0.7104535
PPP2R5E	-0.682339
PPP4R4	-1.2563176
PPP5C	-0.7299347
PPP6C	-0.3444276
PPP6R2	-0.4929149
PPT2	-2.0505142
PPTC7	-0.2685914
PQBP1	-1.1590486
PQLC1	-2.0977745
PQLC2	-3.4769578
PRADC1	-1.6278133
PRAME	-2.630466
PRAMEF12O!	-2.1309988
PRAMEF17	-2.0316408
PRAMEL	-1.748908
PRAMEL1	-2.2825646
PRAMEL4	-3.4418838
PRAMEL5	-2.0430758
PRAMEL6	-2.1347775

PRAP1	-0.9756255
PRC1	-0.5548949
PRDM12	-2.0474825
PRDM14	-1.5643106
PRDM9	-1.1622589
PRDX1	-2.1210318
PRDX2	-1.8101506
PRDX3	-2.2835426
PRDX4	-3.196939
PRDX5	-0.4035111
PRDX6B	-3.4559686
PREB	-1.0853825
PRELID1	-1.4068737
PRELID2	-2.3695402
PREP	-0.2757559
PREPL	-1.3916011
PRG2	-2.6932683
PRG3	-2.4989202
PRG4	-5.310343
PRH1	-1.9396708
PRICKLE4	-2.4311414
PRIM1	-0.477448
PRIMA1	-2.4521596
PRKAA2	-2.7819867
PRKAB1	-0.7099752
PRKACA	-0.824291
PRKACB	-0.1475844
PRKAG1	-1.4920135
PRKAG2	-1.9199052
PRKAG2OS1	-2.1652174
PRKAG3	-0.7109463
PRKAR1A	-1.2061496
PRKAR2A	-1.9100847
PRKCSH	-1.5386977
PRKCZ	-0.4024439
PRKRA	-1.452683
PRKRIR	-1.0159354
PRL	-2.1914315
PRL2A1	-2.1753798
PRL2B1	-1.7617054
PRL2C5	-6.615308
PRL3A1	-2.0051444
PRL3C1	-2.2053962
PRL4A1	-2.4064693
PRL5A1	-2.3685336

PRL6A1	-1.9018836
PRL7A2	-1.9773376
PRL7B1	-2.201193
PRL7C1	-4.091339
PRL7D1	-2.246572
PRL8A1	-2.1465023
PRL8A2	-1.9828634
PRL8A6	-2.2044547
PRL8A9	-2.0919805
PRLR	-7.397389
PRM3	-1.8294506
PRMT10	-0.87988
PRMT3	-0.76999
PRMT5	-0.1821919
PRMT7	-0.6943841
PRN	-1.8052287
PROB1	-0.7701893
PROC	-11.029358
PROCA1	-0.351373
PROCR	-0.7870545
PRODH	-4.8691893
PRODH2	-12.035009
PROK1	-3.5968695
PROKR2	-2.0838938
PROL1	-2.1482964
PROM1	-0.858624
PROM2	-2.7782605
PRORSD1	-0.529881
PROS1	-0.5252123
PROSC	-1.4552617
PROSER1	-0.5446436
PROSER2	-2.4688902
PROX1	-5.4690313
PROZ	-6.3179073
PRP2	-1.2824903
PRPF19	-2.2261753
PRPF40B	-0.7347684
PRPF8	-0.6751413
PRPH2	-2.087297
PRPMP5	-1.3151746
PRPS1	-0.8252778
PRPS1L1	-1.7721305
PRPS1L3	-0.9799829
PRPSAP1	-1.4680176
PRR12	-1.3194883

PRR16	-3.8482828
PRR19	-1.4821148
PRR22	-0.9251297
PRR23A	-2.3535101
PRR27	-2.8943465
PRR3	-0.278677
PRR30	-1.6993577
PRR32	-2.2595062
PRR5	-1.3506413
PRRC2A	-0.8015866
PRRG2	-1.8000007
PRRT2	-0.9794726
PRRXL1	-1.9987984
PRSS1	-1.0594814
PRSS21	-2.2520323
PRSS27	-1.2630012
PRSS29	-2.851798
PRSS30	-1.4415932
PRSS32	-1.6153948
PRSS35	-2.0865426
PRSS36	-1.4493
PRSS37	-0.5248332
PRSS38	-1.9856458
PRSS39	-2.1822293
PRSS40	-1.7817097
PRSS41	-1.5380397
PRSS42	-1.4265347
PRSS43	-1.9923525
PRSS44	-2.0061872
PRSS45	-2.3048632
PRSS46	-3.493894
PRSS47	-2.1535466
PRSS51	-1.4250038
PRSS52	-2.1039305
PRSS55	-2.1948695
PRSS57	-2.2428498
PRSS58	-1.6867936
PRTG	-2.1708453
PRTN3	-2.527466
PRUNE	-1.6021283
PSAP	-1.6829638
PSD3	-0.8242011
PSEN1	-0.2504816
PSEN2	-2.0038743
PSENEN	-0.6992064

PSG17	-1.5423472
PSG18	-1.4344187
PSG19	-1.607671
PSG20	-2.5009632
PSG21	-2.2809083
PSG22	-2.129293
PSG23	-4.5353093
PSG25	-2.371885
PSG26	-2.163249
PSG28	-2.3204942
PSG29	-2.0680368
PSG-PS1	-2.1215587
PSKH1	-1.7430515
PSMA1	-0.636961
PSMA2	-0.4288397
PSMA3	-0.9029985
PSMA4	-1.0229125
PSMA5	-1.4525204
PSMA6	-0.8607369
PSMA7	-1.0830069
PSMB1	-1.6121945
PSMB10	-0.4903097
PSMB11	-1.8083651
PSMB3	-1.2817912
PSMB4	-1.3022456
PSMB5	-1.262547
PSMB6	-1.5231109
PSMB7	-2.4519238
PSMB8	-0.7981367
PSMB9	-1.000051
PSMC1	-1.1465158
PSMC2	-0.8040404
PSMC3	-1.0748687
PSMC4	-1.3604212
PSMC5	-0.5365515
PSMC6	-0.6602597
PSMD1	-0.7892127
PSMD11	-1.704854
PSMD12	-0.8047195
PSMD13	-1.6369133
PSMD2	-0.6625481
PSMD3	-1.4486265
PSMD4	-0.9592757
PSMD5	-1.2644181
PSMD6	-0.9580188

PSMD7	-0.7580509
PSMD9	-2.513556
PSME1	-1.0818195
PSME2B	-0.4746103
PSME3	-1.9823661
PSMF1	-1.3248382
PSMG1	-2.0391135
PSMG2	-0.7283878
PSMG3	-1.510098
PSMG4	-1.1131082
PSPH	-1.0993476
PSPN	-2.1236033
PSRC1	-1.4830568
PSTK	-1.9833455
PTBP1	-0.5757337
PTCD1	-1.1793461
PTCD2	-2.2760954
PTCD3	-1.3319907
PTCH1	-0.3687656
PTCH2	-1.0290852
PTCHD1	-1.9654665
PTCHD2	-0.6155562
PTDSS1	-0.9157896
PTDSS2	-0.7344503
PTER	-6.208015
PTF1A	-2.3172972
PTGDR	-1.9817731
PTGDR2	-0.2938643
PTGDS	-2.3992755
PTGES	-1.3254862
PTGES2	-1.4055071
PTGES3	-0.523943
PTGR1	-0.3627276
PTGR2	-1.8155265
PTGS1	-1.32722
PTH	-2.0356345
PTH1R	-3.657503
PTH2R	-2.3013406
PTK2B	-0.8162031
PTK6	-2.2351234
PTMS	-1.8007998
PTOV1	-1.003345
PTP4A1	-1.0942378
PTPLA	-1.2074618
PTPLAD1	-2.5545344

PTPLB	-2.278985
PTPMT1	-2.1289916
PTPN20	-1.8458104
PTPN3	-1.1506095
PTPN5	-0.776648
PTPN9	-0.5062208
PTPRD	-1.9496608
PTPRF	-0.6639671
PTPRH	-1.6332316
PTPRN	-0.8956492
PTPRN2	-0.9340429
PTPRQ	-3.2447126
PTPRR	-2.3211362
PTRH2	-1.4676175
PTRHD1	-1.6221447
PTS	-2.4124413
PTTG1	-0.3544974
PTTG1IP	-1.1430593
PTX4	-1.079068
PUF60	-0.6388249
PUS1	-1.4917817
PUS10	-1.7961364
PUSL1	-0.7707186
PVALB	-1.7724097
PVRL1	-1.6815434
PVRL2	-0.8016605
PWP2	-0.8127375
PXMP2	-6.0663753
PXMP4	-3.8011174
PXYLP1	-1.8885844
PYCRL	-3.0350888
PYDC3	-1.331182
PYGL	-1.9025254
PYGO1	-2.0979953
PYROXD2	-4.7724147
PYURF	-3.2840219
PZP	-9.952791
QARS	-0.8117142
QDPR	-4.992229
QPCT	-2.1276255
QPCTL	-1.4703269
QPRT	-7.670636
QRFPR	-1.6586266
QRSL1	-0.6701713
QSOX1	-2.1265192

QSOX2	-0.2595179
QTRT1	-0.7925181
R3HCC1	-1.3277788
R3HDM2	-0.4932199
RAB1	-0.4590826
RAB11A	-0.3358131
RAB11B	-0.802928
RAB11FIP2	-0.6020956
RAB11FIP4	-1.6079266
RAB11FIP4OS	-1.471585
RAB13	-0.4175272
RAB14	-0.6205058
RAB17	-5.981173
RAB18	-0.4051037
RAB1B	-1.271738
RAB21	-0.5466952
RAB22A	-0.5996079
RAB23	-0.6335409
RAB24	-1.3268313
RAB30	-4.6659555
RAB32	-0.3028126
RAB39	-1.9997005
RAB39B	-2.338372
RAB3A	-0.1543365
RAB3B	-2.4879994
RAB3D	-0.5846267
RAB3IL1	-1.1982927
RAB3IP	-0.3651061
RAB40B	-0.7816799
RAB40C	-1.0394983
RAB42	-2.1041536
RAB43	-1.305356
RAB4A	-3.6489882
RAB4B	-0.7892647
RAB5B	-0.4067707
RAB5C	-0.7442813
RAB7L1	-1.298696
RAB8A	-0.929318
RAB9	-1.5036831
RAB9B	-0.6928537
RABEP1	-0.4730964
RABEPK	-1.1562388
RABGGTA	-2.2349577
RABGGTB	-0.8035793
RABIF	-0.3050056

RABL3	-2.4239767
RABL6	-0.289012
RAC1	-0.2420163
RAD1	-0.6147981
RAD21L	-2.5010135
RAD23A	-0.9931877
RAD23B	-0.6237226
RAD51	-0.5524094
RAD51AP1	-0.6458564
RAD51AP2	-1.83758
RAD51B	-5.6207833
RAD51C	-1.8428559
RAD51D	-1.4932687
RAD54B	-0.3916767
RAD54L	-0.2372792
RAD54L2	-1.3403144
RAET1A	-4.752951
RAET1B	-2.845399
RAET1C	-0.7076252
RAET1E	-2.9439764
RAF1	-0.9040489
RAG1	-2.0153618
RAG2	-1.6888399
RALGPS1	-1.9248013
RALGPS2	-2.6918805
RALY	-0.6594338
RALYL	-1.7437224
RAMP1	-0.5140238
RAN	-0.3774176
RANBP1	-0.5417981
RANBP10	-1.8749433
RANBP17	-2.047823
RANBP3	-0.1821837
RANBP3L	-2.2407417
RANBP6	-0.3497484
RANGAP1	-0.3007426
RANGRF	-0.5123291
RAP2C	-1.7975597
RAPGEF4	-3.013993
RAPH1	-1.5389123
RARB	-2.231518
RARRES1	-7.164051
RARRES2	-2.664731
RARS2	-0.9584107
RASA4	-0.4062352

RASAL1	-4.003209
RASGRF2	-1.0482199
RASGRP2	-1.3504453
RASL2-9	-0.8177977
RASSF3	-0.4619718
RASSF4	-0.6370201
RASSF6	-2.5001528
RASSF7	-0.9351144
RAVER1	-2.047172
RBBP5	-0.7408314
RBBP7	-0.9667559
RBBP9	-0.7716422
RBCK1	-1.083756
RBFA	-2.6822743
RBFOX2	-0.6219592
RBFOX3	-1.3777237
RBKS	-2.6281834
RBL2	-0.9316273
RBM11	-1.9791551
RBM12B1	-0.86321
RBM15	-0.3136272
RBM19	-0.2365651
RBM3	-0.3554201
RBM34	-1.1231298
RBM4	-1.0071206
RBM42	-0.2169814
RBM44	-2.2541037
RBM45	-0.3824458
RBM47	-0.4130168
RBM4B	-0.9588871
RBMXL2	-2.333613
RBMY	-3.5760438
RBP1	-3.6097493
RBP2	-0.9058447
RBP3	-0.7173452
RBP4	-6.137878
RBPMS2	-4.924375
RCAN2	-1.3755951
RCAN3	-0.3656812
RCBTB1	-1.80368
RCBTB2	-1.8561835
RCC2	-0.2948194
RCHY1	-0.3293815
RCL1	-3.8772244
RCVRN	-1.0425594

RD3L	-2.0776901
RDH1	-0.540278
RDH10	-1.0782604
RDH11	-2.4641976
RDH13	-0.9013252
RDH14	-1.9111767
RDH16	-9.677496
RDH18-PS	-3.5792055
RDH5	-4.5043626
RDH7	-13.957432
RDH8	-2.201278
RDH9	-7.3969707
RDM1	-0.5480504
RDX	-0.4812245
REC114	-3.3270886
RECQL4	-1.574532
RECQL5	-1.4273095
REDRUM	-0.9550972
REEP2	-1.3285058
REEP3	-0.9802241
REEP4	-0.451119
REEP6	-5.2694883
REG1	-2.0797591
REG2	-1.8053567
REG3B	-2.0849054
REG3D	-1.5153499
REL	-2.0337849
RELL2	-1.1115608
RELN	-3.614859
REM2	-1.4918408
REN1	-1.982369
REN2	-4.309502
REPIN1	-2.5850239
REPS2	-0.5742269
RER1	-1.5871425
RETSAT	-5.493455
REX2	-2.404245
REXO2	-0.7164817
RFC1	-0.2457161
RFC2	-1.7252059
RFC3	-1.2164443
RFC5	-1.6813865
RFESD	-0.9825988
RFFL	-0.7661514
RFK	-0.5323224

RFNG	-0.6110234
RFPL4	-0.5436649
RFPL4B	-2.0694842
RFT1	-1.0747046
RFWD2	-0.593246
RFWD3	-0.5669265
RFX4	-2.037323
RFX6	-2.016399
RFX7	-1.7939546
RFXANK	-1.6947293
RFXAP	-0.9158187
RGL1	-0.2374477
RGL3	-2.1476665
RGN	-12.030905
RGS16	-2.5718231
RGS20	-1.4995008
RGS5	-2.9291105
RGS7	-0.9742134
RGSL1	-0.8971753
RHBDD1	-1.2869906
RHBDD2	-0.6848671
RHBDD3	-2.6678796
RHBDL1	-1.7076669
RHBDL2	-2.0803342
RHBG	-4.551999
RHO	-2.0737844
RHOBTB1	-1.9191902
RHOBTB2	-0.4482708
RHOBTB3	-1.7196021
RHOD	-3.6491358
RHOH	-1.3003902
RHOT1	-0.6156058
RHOT2	-1.7887731
RHOU	-1.5618148
RHOX10	-2.7149105
RHOX11	-2.2636302
RHOX12	-2.1788402
RHOX2C	-1.8252206
RHOX2F	-2.494295
RHOX3A	-1.2204773
RHOX3F	-0.9220905
RHOX3G	-1.7671962
RHOX3H	-0.4138179
RHOX4A	-1.7738371
RHOX4E	-1.88977

RHOX5	-3.4225497
RHOX6	-2.1673934
RHPN2	-1.9097443
RIC3	-0.9481959
RILP	-2.940086
RILPL2	-0.2376695
RIMBP2	-2.0646684
RIMKLB	-2.1421247
RIMS2	-1.3721461
RIMS3	-2.3394842
RIMS4	-5.710983
RING1	-1.397639
RINT1	-0.9349415
RIOK2	-0.4941759
RIOK3	-0.5051637
RIPPLY1	-1.9029493
RIT1	-0.3778009
RITA1	-0.820555
RLBP1	-1.4790165
RMDN1	-3.210854
RMDN2	-1.4201145
RMDN3	-1.8039856
RMI1	-0.4353395
RMI2	-2.0516167
RMND1	-2.9433293
RMND5A	-1.2355542
RMND5B	-1.1689558
RN28S1	-0.7883048
RN45S	-0.9783816
RNASE1	-2.2283852
RNASE11	-3.6725142
RNASE12	-0.6942627
RNASE2B	-1.9298713
RNASE4	-3.4073048
RNASE9	-1.1559744
RNASEH1	-0.4918814
RNASEH2A	-1.887846
RNASEH2B	-1.1229377
RNASEH2C	-1.4608893
RNASEK	-1.177351
RNASET2A	-1.1712246
RND2	-4.1960654
RNF10	-1.200829
RNF103	-2.2844195
RNF112	-0.7013824

RNF113A1	-0.4748492
RNF113A2	-0.31778
RNF115	-0.2743645
RNF121	-0.81563
RNF123	-3.0140114
RNF125	-1.8417177
RNF126	-0.9164772
RNF128	-4.855004
RNF13	-1.2332864
RNF130	-0.1865444
RNF135	-1.5727811
RNF138	-0.3257942
RNF138RT1	-4.7112956
RNF139	-1.1594577
RNF14	-0.8105593
RNF141	-0.6232171
RNF144B	-0.8893502
RNF145	-0.3874826
RNF148	-2.201003
RNF149	-1.0339432
RNF151	-1.087143
RNF152	-4.6375093
RNF165	-0.8639638
RNF167	-0.9264684
RNF169	-0.4882021
RNF17	-2.193532
RNF170	-1.8708134
RNF181	-1.3097491
RNF182	-2.000401
RNF183	-1.9379158
RNF185	-1.2177453
RNF186	-0.4945183
RNF187	-0.7898173
RNF212	-1.215303
RNF217	-1.6041601
RNF222	-3.6167996
RNF224	-2.20039
RNF26	-0.886395
RNF4	-1.0950456
RNF40	-0.3830705
RNF43	-2.7168632
RNF5	-2.3028054
RNF7	-0.4096556
RNFT1	-1.1963134
RNH1	-1.2288613

RNMTL1	-2.0586038
RNPEP	-0.39048
RNPEPL1	-0.9502602
RNPS1	-0.3597865
RNU3B1	-2.3425012
ROBO3	-2.4647932
ROGDI	-1.856875
ROM1	-0.6386366
ROMO1	-1.8176184
ROPN1	-2.0507393
ROPN1L	-4.0945764
RORA	-0.6000924
RORC	-3.2077694
ROS1	-1.1802647
RP1	-1.8502483
RP9	-1.1320205
RPA1	-0.9138856
RPA2	-0.4923987
RPA3	-0.3762569
RPAIN	-0.4793181
RPAP1	-1.3389716
RPAP2	-1.5883603
RPAP3	-0.8963132
RPE	-1.0888762
RPE65	-1.8718009
RPF1	-0.3979507
RPF2	-0.4682674
RPH3A	-0.5169354
RPL10	-0.9604917
RPL10A	-0.7758269
RPL11	-0.8270288
RPL12	-0.8654599
RPL13	-0.4284535
RPL13A	-0.6378856
RPL14	-0.5163465
RPL15	-0.5035229
RPL17	-0.4443679
RPL18A	-0.6079183
RPL19	-0.8936529
RPL22	-0.254756
RPL23	-0.5638137
RPL23A	-0.6295023
RPL24	-0.7576656
RPL26	-0.3560386
RPL27	-0.6988511

RPL27A	-0.5758057
RPL28	-1.0209899
RPL3	-0.5071392
RPL31	-0.7808113
RPL31-PS12	-0.525775
RPL32	-0.4173622
RPL34	-0.3527641
RPL34-PS1	-0.5225802
RPL35	-0.5974231
RPL35A	-0.5402203
RPL36	-0.9644027
RPL36A	-0.5789619
RPL36AL	-1.2824011
RPL37A	-0.8488202
RPL38	-0.7944274
RPL39	-0.1611147
RPL4	-1.002768
RPL41	-1.1298175
RPL5	-0.4148231
RPL5-PS2	-0.2855797
RPL6	-0.582006
RPL7	-0.3571534
RPL7A	-0.6642213
RPL7L1	-1.7905803
RPL8	-0.5690002
RPL9	-0.4098792
RPLP0	-0.812757
RPLP1	-1.2280688
RPLP2	-0.5231862
RPN1	-0.9010143
RPN2	-0.8809648
RPP21	-1.0407038
RPP25	-1.4005473
RPP25L	-1.0006862
RPP38	-0.175067
RPP40	-1.808775
RPS10	-0.7652659
RPS11	-0.7357421
RPS11-PS1	-0.89852
RPS12	-0.5953546
RPS12L2	-0.3065958
RPS12-PS16	-0.5173221
RPS13	-0.4643202
RPS14	-0.8774228
RPS15	-0.7813582

RPS15A	-0.3095241
RPS16	-0.807384
RPS17	-0.8203306
RPS18	-0.7734623
RPS19BP1	-1.3297296
RPS2	-1.8708992
RPS20	-0.4891553
RPS21	-0.5231481
RPS24	-0.2943611
RPS25	-0.8171148
RPS26	-0.4462466
RPS27	-0.3597131
RPS27A	-0.5574393
RPS27L	-2.420054
RPS28	-0.6532912
RPS29	-0.6443334
RPS3	-0.8489513
RPS3A1	-0.3621903
RPS4X	-0.4943733
RPS5	-0.8928905
RPS6	-0.4467702
RPS6KB2	-0.8873992
RPS7	-0.429338
RPS8	-0.911715
RPSA	-1.0182428
RPTOR	-0.8800507
RPUSD1	-0.8522389
RPUSD2	-2.2553153
RPUSD3	-3.8667822
RPUSD4	-1.1206741
RQCD1	-1.1535153
RRAGA	-1.6440034
RRAGC	-0.5505252
RRBP1	-1.1846051
RREB1	-1.017077
RRH	-1.8546233
RRM2	-0.800242
RRNAD1	-0.2822914
RRP15	-0.6757917
RRP36	-1.2303033
RRP8	-0.9114595
RRP9	-0.5092769
RRS1	-0.2908464
RSG1	-1.8551974
RSL1D1	-0.893137

RSLCAN18	-0.8376901
RSPH3B	-0.5239005
RSPH6A	-1.9387631
RSPO2	-2.0759492
RSPO3	-5.1182013
RSRC1	-0.371707
RSRP1	-0.4248714
RTBDN	-0.8173635
RTCA	-0.401649
RTCB	-0.3297811
RTEL1	-1.1282272
RTFDC1	-1.651082
RTKN	-0.3716259
RTN3	-0.7057896
RTN4	-1.1297121
RTN4IP1	-2.2293918
RTN4R	-2.0333314
RTN4RL1	-2.1459217
RTN4RL2	-1.4809914
RTP1	-1.9680235
RTP2	-1.9325607
RTP3	-3.4031978
RUFY1	-0.5895815
RUFY3	-1.3184748
RUNDC3A	-1.9846327
RUSC2	-1.0773444
RUVBL1	-0.5017653
RWDD2A	-1.031543
RWDD2B	-2.753951
RWDD3	-2.139207
RXFP3	-0.9561746
RXRA	-3.45376
RXRB	-0.6906824
RXRG	-2.296918
RYK	-0.6397643
S100A1	-1.7126493
S100A10	-0.6891661
S100A13	-0.8204446
S100A7A	-3.0408843
S100Z	-0.6253645
S1PR5	-1.7636681
SAA1	-2.729056
SAA2	-2.6070876
SAA4	-7.9072447
SAAL1	-1.50404

SAC3D1	-1.1230025
SACM1L	-0.8526206
SAE1	-0.4166098
SAFB	-0.2563748
SALL1	-7.405781
SALL3	-2.1782753
SALL4	-1.1091633
SAMD10	-0.3488054
SAMD11	-1.1470187
SAMD7	-2.2650795
SAMM50	-2.4887676
SAMT2	-0.7647424
SAMT3	-2.9127467
SAMT4	-2.0286984
SAP30BP	-0.4778781
SAP30L	-0.8242197
SAR1A	-0.4217334
SAR1B	-3.1139646
SARDH	-8.232267
SARS	-0.3697166
SARS2	-2.2615967
SART1	-0.3036666
SAT1	-0.4408145
SAT2	-5.0435734
SATL1	-1.6274116
SAYSD1	-0.2925947
SBF1	-0.3886218
SBK1	-2.7777796
SBK2	-1.4507048
SBP	-2.3409233
SBPL	-1.6859608
SBSPON	-0.9352171
SC5D	-5.348201
SCAF1	-2.6827555
SCAI	-0.9803584
SCAMP1	-0.7426434
SCAMP3	-1.0500712
SCAMP4	-0.9187098
SCAMP5	-3.54979
SCAND1	-0.7403674
SCAP	-2.5970726
SCARA3	-1.1356754
SCARB1	-3.1311731
SCARB2	-0.4925599
SCD1	-3.032588

SCD3	-2.8774352
SCFD1	-0.3576503
SCFD2	-0.7400231
SCGB1B19	-2.3827708
SCGB1B20	-1.8987188
SCGB1B27	-2.0931
SCGB1B7	-3.0133562
SCGB2B12	-2.2130861
SCGB2B15	-0.8146472
SCGB2B21	-2.0698202
SCGB2B23-PS	-2.1642456
SCGB2B24	-2.117795
SCGB2B26	-2.2617912
SCGB2B27	-1.3752131
SCGB2B3	-2.2981389
SCIMP	-1.0815153
SCLT1	-0.5024529
SCLY	-2.7732987
SCN1B	-3.584895
SCN2A1	-0.6805804
SCN4B	-2.2116277
SCN8A	-2.138163
SCN9A	-2.2931855
SCNM1	-0.5489502
SCNN1G	-2.079151
SCO1	-1.2219448
SCO2	-4.680125
SCP2	-3.834577
SCP2D1	-1.8968697
SCPEP1	-1.6297598
SCRN2	-4.4971323
SCRN3	-2.1382403
SCRT1	-1.264792
SCRT2	-1.6155164
SCYL1	-0.7260585
SCYL2	-0.1839705
SDC1	-2.62494
SDC2	-3.4849148
SDC3	-1.9247618
SDCCAG3	-0.623848
SDF2	-0.8266525
SDF2L1	-0.6943264
SDF4	-1.1866245
SDHA	-1.9311495
SDHAF1	-1.7150402

SDHAF2	-0.3660097
SDHB	-2.4391727
SDHC	-2.4157243
SDHD	-2.9176192
SDK2	-0.4819074
SDR16C5	-1.7078459
SDR39U1	-1.2400699
SDR42E1	-6.257245
SDR9C7	-5.8573866
SDS	-10.433391
SDSL	-4.5471873
SEBOX	-3.6862102
SEC11A	-0.5901022
SEC11C	-0.6876497
SEC13	-1.1824465
SEC14L2	-5.6585646
SEC14L4	-2.4654555
SEC16A	-1.4828715
SEC16B	-6.4133844
SEC22B	-0.3268337
SEC22C	-2.0810513
SEC23A	-2.446622
SEC24A	-2.3106718
SEC24C	-0.7241221
SEC24D	-1.6540108
SEC31A	-0.9379883
SEC31B	-1.9357481
SEC61A1	-1.392498
SEC61B	-0.9173198
SEC61G	-0.2422047
SEC62	-1.1052804
SEC63	-0.8207665
SECTM1A	-3.5828514
SECTM1B	-1.5194044
SEH1L	-0.3124075
SEL1L2	-1.9629269
SEL1L3	-2.3545408
SELENBP1	-0.2480841
SELK	-0.4307303
SELO	-2.5148911
SELT	-0.9728055
SEMA4A	-1.2590914
SEMA4B	-0.7037921
SEMA4C	-1.9404736
SEMA4G	-9.631674

SEMA5B	-2.7144728
SEMA6C	-1.3945322
SEMA6D	-1.6322494
SENP3	-0.8711152
SENP5	-0.2221737
SEPHS1	-0.8146155
SEPHS2	-4.45399
SEPP1	-2.5138845
SEPSECS	-1.9934235
SEPW1	-0.3135805
SERBP1	-0.2570262
SERF1	-1.1388135
SERF2	-1.5011272
SERGEF	-1.1599102
SERHL	-2.5722594
SERINC1	-0.8948255
SERINC2	-1.9843621
SERINC5	-0.9467115
SERP1	-0.3437281
SERPINA10	-8.862881
SERPINA11	-9.229885
SERPINA12	-1.7699893
SERPINA1A	-8.944029
SERPINA1C	-8.092239
SERPINA1D	-10.639513
SERPINA1E	-9.045399
SERPINA3A	-2.6463199
SERPINA3C	-3.3518624
SERPINA3H	-1.5488882
SERPINA3J	-4.229805
SERPINA3K	-13.409853
SERPINA3M	-6.083295
SERPINA3N	-1.1331272
SERPINA4-PS	-1.8714948
SERPINA5	-2.726481
SERPINA6	-12.617292
SERPINA7	-10.524595
SERPINB11	-2.0483725
SERPINB12	-1.9450226
SERPINB13	-2.2306573
SERPINB1C	-1.6090484
SERPINB3A	-2.2731478
SERPINB3B	-2.5686843
SERPINB3D	-2.2457347
SERPINB8	-1.6228557

SERPINB9B	-1.8375592
SERPINB9C	-1.3680615
SERPINB9D	-2.0671723
SERPINB9E	-2.1474555
SERPINB9F	-2.0766048
SERPINB9G	-2.2561781
SERPINC1	-12.439542
SERPIND1	-7.3367057
SERPINF1	-5.627309
SERPINF2	-12.38544
SERPING1	-2.182282
SERPINI2	-2.5422597
SERTM1	-1.6070881
SESN1	-0.8663368
SESN3	-0.5378146
SETD1A	-0.4084578
SETD3	-0.6032047
SETD4	-3.3556457
SETD6	-2.052857
SETDB1	-0.3597145
SETMAR	-1.2876766
SEZ6	-1.6732812
SEZ6L	-1.4671686
SF1	-0.2622905
SF3A2	-0.8731761
SF3B2	-0.9572587
SF3B5	-0.7684736
SFMBT1	-1.2029767
SFR1	-0.496366
SFT2D1	-0.7402787
SFT2D2	-0.7355108
SFT2D3	-1.0066786
SFXN1	-3.4687877
SFXN2	-3.4566855
SFXN5	-3.1255312
SGK2	-11.615125
SGK3	-0.427043
SGMS2	-1.373061
SGPL1	-1.2345686
SGPP1	-1.7726774
SGSH	-1.8287444
SGSM1	-1.3403964
SGSM2	-1.6232667
SGSM3	-1.5695372
SGTA	-1.362813

SGTB	-0.2703571
SH2B1	-1.1819444
SH2D4A	-1.3847399
SH2D6	-0.8762236
SH2D7	-1.5043681
SH3BGRL2	-0.977315
SH3BP2	-0.8266821
SH3BP5L	-0.4803429
SH3GLB1	-0.29389
SH3GLB2	-2.079381
SH3RF2	-0.6888292
SH3YL1	-1.652708
SHANK1	-0.3387387
SHANK2	-1.7965462
SHARPIN	-0.7054024
SHB	-0.2749553
SHBG	-2.6875074
SHC1	-0.5636501
SHCBP1	-0.4454641
SHCBP1L	-1.3350513
SHF	-3.7126608
SHISA2	-0.7378664
SHISA3	-0.9648018
SHISA6	-1.9054744
SHMT1	-5.7385187
SHMT2	-4.4311967
SHPK	-5.641552
SHQ1	-0.6047535
SHROOM1	-3.736471
SHROOM2	-0.672226
SIAE	-2.2738352
SIAH2	-1.5935173
SIDT2	-2.2245026
SIGIRR	-2.2113786
SIGLEC1	-1.6283467
SIGMAR1	-4.243677
SIK3	-0.5911379
SIL1	-2.4023633
SIM1	-2.0643868
SIN3B	-0.8304963
SIRPA	-0.8000307
SIRT2	-1.1181564
SIRT3	-4.235666
SIRT4	-0.6340137
SIRT5	-3.3474903

SIRT7	-0.7708721
SIS	-2.1243253
SIX2	-1.6262951
SIX3	-0.7402396
SIX5	-0.414144
SIX6	-3.0608242
SKI	-0.4185426
SKIDA1	-0.4003015
SKINT10	-1.313909
SKINT11	-1.7245541
SKINT2	-1.4484713
SKINT6	-2.3680942
SKINT7	-1.7702703
SKINT8	-2.1158102
SKINT9	-0.8257086
SKIV2L	-1.4027977
SKOR2	-1.9400826
SKP1A	-1.4833579
SKP2	-0.8273256
SLAIN1OS	-2.355531
SLC10A1	-0.3111911
SLC10A4	-1.6843772
SLC10A5	-3.84167
SLC11A1	-0.7172456
SLC12A1	-1.6920962
SLC12A5	-0.7957809
SLC12A7	-1.9349709
SLC12A8	-1.3840022
SLC12A9	-1.4773552
SLC13A1	-1.792541
SLC13A2OS	-1.6793795
SLC13A3	-4.4500146
SLC13A5	-5.0629716
SLC14A2	-2.7185032
SLC15A4	-0.5621753
SLC15A5	-2.0760822
SLC16A10	-1.9110899
SLC16A11	-0.6752071
SLC16A12	-4.3437757
SLC16A2	-3.9554234
SLC16A4	-0.7166402
SLC16A5	-8.506195
SLC16A7	-2.316067
SLC17A1	-10.36018
SLC17A2	-8.131926

SLC17A3	-5.7052755
SLC17A4	-8.36453
SLC17A7	-1.6217351
SLC17A8	-2.91898
SLC17A9	-1.8490496
SLC18A1	-1.5921915
SLC19A1	-1.836051
SLC19A2	-4.7153997
SLC1A2	-6.8591313
SLC1A4	-1.3714275
SLC1A7	-0.1746359
SLC20A2	-2.902903
SLC22A1	-8.548161
SLC22A15	-0.3481646
SLC22A16	-2.1628053
SLC22A18	-5.162029
SLC22A19	-2.217441
SLC22A2	-2.2576127
SLC22A22	-1.6292071
SLC22A23	-2.0679889
SLC22A26	-10.861592
SLC22A27	-8.87074
SLC22A28	-5.228238
SLC22A29	-7.6504717
SLC22A3	-2.8448489
SLC22A30	-6.3683023
SLC22A4	-1.6074836
SLC22A5	-0.8961263
SLC22A7	-10.950524
SLC23A1	-4.025301
SLC24A1	-1.9741824
SLC25A1	-3.9236336
SLC25A10	-3.5803802
SLC25A11	-1.281198
SLC25A12	-0.8699622
SLC25A13	-5.4867816
SLC25A15	-4.572854
SLC25A16	-2.4141955
SLC25A17	-1.1108041
SLC25A19	-0.4418469
SLC25A20	-2.3098392
SLC25A21	-7.458742
SLC25A22	-5.692093
SLC25A23	-4.5287914
SLC25A25	-2.253532

SLC25A26	-1.5550137
SLC25A28	-0.7606139
SLC25A3	-0.3792596
SLC25A30	-1.2119212
SLC25A32	-1.121078
SLC25A33	-1.8630424
SLC25A34	-1.7632332
SLC25A37	-0.2784329
SLC25A38	-1.1210546
SLC25A39	-4.2422
SLC25A40	-0.3083377
SLC25A42	-4.25071
SLC25A44	-2.6210525
SLC25A45	-1.9979191
SLC25A46	-0.791203
SLC25A47	-9.591143
SLC25A48	-4.6200457
SLC25A5	-2.0035768
SLC25A51	-0.2832112
SLC25A54	-2.0988686
SLC26A1	-8.048717
SLC26A10	-3.9775982
SLC26A11	-1.3851194
SLC26A5	-2.341643
SLC26A6	-1.5874727
SLC26A7	-2.2420328
SLC26A8	-1.650017
SLC27A1	-0.8870111
SLC27A2	-9.759994
SLC27A5	-12.944132
SLC29A1	-3.2170887
SLC29A2	-0.5533226
SLC2A2	-12.588102
SLC2A5	-8.498978
SLC2A7	-2.6425984
SLC2A8	-2.4887142
SLC2A9	-5.195262
SLC30A10	-8.410615
SLC30A3	-1.3252535
SLC30A5	-0.8460174
SLC30A6	-0.685308
SLC30A8	-3.2663953
SLC30A9	-0.9893842
SLC31A1	-2.019888
SLC33A1	-1.6827006

SLC34A3	-2.044883
SLC35A1	-1.0500917
SLC35A2	-0.147295
SLC35A3	-1.4739451
SLC35A5	-0.4567931
SLC35B1	-0.3189583
SLC35B2	-2.3972583
SLC35B3	-0.5511599
SLC35B4	-1.2920895
SLC35C1	-0.6572771
SLC35C2	-2.1450882
SLC35D1	-3.7148519
SLC35D2	-4.1154904
SLC35D3	-1.6527238
SLC35E2	-2.7862973
SLC35F1	-1.644156
SLC35F4	-2.1032746
SLC35F5	-1.4210095
SLC35F6	-1.0315847
SLC35G1	-3.0026383
SLC36A1	-0.838433
SLC36A1OS	-2.6630616
SLC37A2	-0.8417273
SLC37A3	-0.2062206
SLC37A4	-5.483773
SLC38A10	-0.7368856
SLC38A11	-2.81902
SLC38A3	-12.270025
SLC38A4	-4.0996275
SLC38A6	-0.496129
SLC38A7	-2.2601013
SLC38A8	-2.3397074
SLC39A1	-0.540174
SLC39A11	-1.7828941
SLC39A12	-1.4063585
SLC39A13	-0.3210149
SLC39A14	-1.2655792
SLC39A4	-1.9148717
SLC39A5	-4.2030773
SLC39A7	-0.263032
SLC3A1	-7.849313
SLC40A1	-5.7468877
SLC41A2	-2.3828
SLC41A3	-1.7868195
SLC43A1	-3.1925552

SLC44A1	-0.5697846
SLC44A3	-0.8441072
SLC44A5	-3.352324
SLC45A1	-2.849873
SLC45A2	-2.7454379
SLC46A1	-5.1277604
SLC46A2	-1.537857
SLC46A3	-2.8347497
SLC47A1	-6.6248817
SLC48A1	-1.0417047
SLC4A10	-2.156392
SLC4A2	-0.7823796
SLC4A4	-3.1235995
SLC4A9	-4.817404
SLC51B	-6.2275176
SLC52A2	-1.4195223
SLC5A10	-0.867204
SLC5A11	-2.2218668
SLC5A12	-1.2008634
SLC5A4A	-1.6156828
SLC5A4B	-2.2606485
SLC5A5	-1.8339553
SLC5A6	-1.9867449
SLC5A7	-1.7745192
SLC5A8	-1.4031432
SLC6A1	-2.4880166
SLC6A11	-2.1051197
SLC6A12	-8.741934
SLC6A13	-9.146566
SLC6A15	-0.6237049
SLC6A16	-1.24881
SLC6A17	-3.0797548
SLC6A18	-1.7068057
SLC6A19	-1.8263798
SLC6A20B	-2.1280239
SLC6A5	-2.1120932
SLC6A6	-0.7370982
SLC6A9	-1.1929326
SLC7A12	-2.0822532
SLC7A13	-1.7636881
SLC7A14	-2.168707
SLC7A15	-2.6417465
SLC7A2	-3.4659789
SLC7A4	-0.602181
SLC7A6OS	-0.5705457

SLC7A7	-0.7139144
SLC7A9	-2.2129538
SLC8A2	-1.001718
SLC8B1	-2.9952247
SLC9A3	-2.0675354
SLC9A3R1	-2.2243352
SLC9A6	-0.3437352
SLC9A8	-0.8089571
SLC9A9	-0.9293704
SLCO1A1	-7.6527586
SLCO1A4	-10.988667
SLCO1B2	-14.873056
SLCO1C1	-1.7190104
SLCO2B1	-3.8641057
SLCO6B1	-2.2475033
SLCO6C1	-2.1062427
SLCO6D1	-2.184393
SLFN14	-2.0220184
SLFN5	-0.77917
SLFNL1	-1.6738696
SLIRP	-1.0586929
SLITRK1	-2.1213222
SLITRK2	-1.8209796
SLITRK4	-1.2081506
SLITRK5	-3.3502886
SLMAP	-0.2090001
SLMO2	-0.7679796
SLX1B	-0.6384382
SLXL1	-2.1706738
SLY	-1.8609319
SMAD9	-0.758513
SMAGP	-1.2927594
SMAP2	-0.680491
SMARCA2	-0.6023111
SMARCAD1	-0.3524175
SMARCC1	-1.1977267
SMARCC2	-1.6625304
SMARCD2	-1.5747657
SMC1B	-5.460205
SMC2OS	-2.7573466
SMC6	-0.661736
SMCO2	-3.7447062
SMCO3	-1.906743
SMCO4	-1.6962161
SMCR8	-0.3643017

SMDT1	-1.2701945
SMEK1	-0.5730453
SMG5	-0.7074966
SMG6	-0.7679701
SMG7	-0.5851941
SMG8	-2.3178236
SMG9	-0.4892244
SMGC	-1.9877768
SMIM1	-1.1759195
SMIM11	-0.5069747
SMIM12	-1.2523723
SMIM13	-0.954174
SMIM14	-1.1442599
SMIM18	-1.4871004
SMIM19	-1.5914264
SMIM20	-1.8733974
SMIM22	-0.4952436
SMIM24	-0.2774701
SMIM4	-1.7768989
SMIM6	-1.3736451
SMIM7	-1.0914307
SMIM8	-1.6304212
SMLR1	-12.867917
SMN1	-0.2225585
SMNDC1	-1.5020709
SMO	-1.1242752
SMOC1	-7.260091
SMOC2	-1.3567166
SMOK2A	-2.296392
SMOK3A	-2.064489
SMOK3B	-2.492024
SMPD1	-0.830462
SMPD2	-2.9544468
SMPD3	-0.962245
SMPD4	-0.7729044
SMPD5	-2.60754
SMPDL3A	-0.6509023
SMR3A	-1.8780041
SMU1	-0.8329735
SMUG1	-0.7039366
SMURF1	-0.703218
SMYD1	-1.7113998
SMYD2	-2.0233164
SMYD4	-1.0776451
SMYD5	-1.659111

SNAI2	-2.3305497
SNAI3	-1.6348691
SNAP47	-3.417512
SNAP91	-2.20548
SNAPC2	-1.1994557
SNCB	-1.4687433
SND1	-0.5221496
SNF8	-1.2244387
SNHG10	-0.4072151
SNHG12	-0.5819969
SNHG3	-0.614028
SNHG6	-0.7291322
SNHG7	-0.4851813
SNHG8	-2.2182238
SNHG9	-1.5581322
SNIP1	-0.7156892
SNORA47	-2.2008884
SNPH	-1.5093551
SNRNP200	-0.4965434
SNRNP25	-1.9204774
SNRNP27	-0.3351631
SNRPA	-0.4106851
SNRPD2	-0.93887
SNRPD3	-0.2521133
SNRPN	-1.6249886
SNTB1	-1.445261
SNTG1	-3.1039708
SNTG2	-3.3833895
SNUPN	-0.6533341
SNX12	-0.236084
SNX14	-0.5731354
SNX15	-1.3806314
SNX19	-0.8402343
SNX2	-0.4840093
SNX21	-1.161047
SNX24	-1.1389887
SNX3	-0.3923907
SNX31	-2.1501334
SNX32	-1.4343233
SNX33	-1.0284028
SNX5	-0.4777494
SNX7	-0.4025974
SNX8	-1.3287597
SNX9	-0.504324
SOAT2	-4.387118

SOD1	-2.3489885
SOD2	-0.2273588
SOGA3	-1.821131
SOHLH2	-0.4122005
SORBS2	-5.1311665
SORBS2OS	-2.112611
SORCS3	-2.1140387
SORD	-5.950992
SOSTDC1	-1.0191817
SOWAHB	-3.1154747
SOWAHD	-2.8521419
SOX1	-2.350959
SOX12	-0.4055283
SOX3	-2.31667
SOX30	-1.9915388
SOX5	-0.9686456
SOX5IT	-1.734138
SOX5OS1	-1.5959554
SOX5OS3	-2.204908
SOX6	-2.8430424
SOX6OS	-2.8603003
SOX8	-1.9826488
SOX9	-0.5681496
SP110	-1.5216093
SP5	-1.9383438
SP7	-2.2112203
SP8	-0.4185157
SP9	-2.1463647
SPACA1	-1.447578
SPACA5	-2.2025714
SPACA7	-1.0951328
SPAG11B	-1.080317
SPAG4	-4.505172
SPAG7	-1.749795
SPANXN4	-2.183811
SPAST	-1.1718102
SPATA16	-1.9437253
SPATA21	-1.3786225
SPATA22	-5.2934732
SPATA24	-1.6182814
SPATA2L	-3.9933286
SPATA3	-1.4325938
SPATA31D1C	-2.6556818
SPATA31D1D	-1.0877345
SPATA32	-0.9485135

SPATA4	-3.2704065
SPATA45	-2.833475
SPATS1	-1.5407696
SPC24	-2.6397107
SPC25	-2.0561543
SPCS1	-0.6548195
SPCS2	-0.491004
SPCS3	-1.1147842
SPDL1	-0.5860355
SPDYA	-0.8031952
SPDYB	-1.8079762
SPEER3	-0.7386513
SPEER4E	-2.0685332
SPEER4F	-0.5729771
SPEER6-PS1	-2.0517178
SPEER7-PS1	-2.0039692
SPEER9-PS1	-1.646066
SPESP1	-1.5521228
SPG20	-0.6342673
SPG7	-1.1710129
SPHK2	-1.3447351
SPIC	-1.0710025
SPIN2C	-1.7252328
SPINK10	-2.3137832
SPINK11	-2.9338741
SPINK12	-2.198236
SPINK13	-2.7334034
SPINK14	-2.1706371
SPINK3	-7.664747
SPINK6	-2.1561615
SPINK7	-2.4058173
SPINK8	-2.0846741
SPINKL	-2.1108391
SPINT3	-3.0507073
SPINT4	-2.3679507
SPIRE2	-1.840828
SPNS2	-1.6040602
SPNS3	-1.9201465
SPOCD1	-1.2045405
SPOCK1	-1.606816
SPOCK3	-2.2891562
SPON2	-4.525356
SPOP	-0.1652641
SPP1	-0.8040538
SPP2	-13.750578

SPPL2B	-1.0079656
SPPL2C	-0.5130508
SPR	-3.133532
SPRED3	-2.1066353
SPRN	-1.8493693
SPRR1A	-3.5967958
SPRR1B	-2.0650444
SPRR2A1	-1.9799826
SPRR2A2	-0.5768094
SPRR2D	-2.1758723
SPRR2E	-2.3454342
SPRR2F	-2.2667487
SPRR2G	-2.0302668
SPRR2H	-1.994842
SPRR2I	-2.2276144
SPRR2J-PS	-2.1604996
SPRR2K	-2.0107057
SPRR3	-1.9632182
SPRY3	-1.9839432
SPRYD3	-0.6220255
SPRYD4	-3.620718
SPSB2	-1.1924329
SPSB3	-0.7032638
SPSB4	-4.064208
SPTBN2	-5.3778486
SPTBN5	-1.64924
SPTSSA	-1.7817898
SPZ1	-2.168092
SQLE	-3.2110653
SQRDL	-2.7044458
SQSTM1	-1.1690922
SRA1	-1.0952454
SRBD1	-0.5008245
SRCRB4D	-1.5720577
SRD5A1	-5.494211
SRD5A2	-1.5021994
SREBF1	-3.052566
SREBF2	-0.2980819
SREK1	-1.6698174
SREK1IP1	-1.3804073
SRM	-1.5371895
SRMS	-2.0182993
SRP68	-0.6574817
SRP72	-1.3407569
SRP9	-0.9483314

SRPK1	-0.2671838
SRPR	-1.2917562
SRPX2	-1.0328507
SRR	-3.1695614
SRRD	-1.5743728
SRRM3OS	-3.100326
SRRM4OS	-2.4743721
SRSF2	-0.1563735
SRSF9	-0.4762373
SRXN1	-2.1356401
SSBP1	-0.4711747
SSBP3	-1.6685319
SSBP4	-0.347908
SSFA2	-1.2841034
SSH1	-0.7147207
SSMEM1	-1.1632662
SSNA1	-1.2565331
SSPO	-1.8644888
SSR1	-0.6283855
SSR2	-1.2853379
SSR3	-0.9905138
SSR4	-1.9492645
SSRP1	-0.6830692
SSSCA1	-1.9278402
SST	-2.1804624
SSTR1	-1.6492901
SSTR2	-2.0337832
SSTR5	-1.0350232
SSTY1	-2.4708867
SSU2	-0.5039628
SSU72	-0.4306474
SSX9	-2.2621903
SSXA1	-1.4989641
SSXB1	-1.5261571
SSXB10	-1.5721846
SSXB2	-2.0432458
SSXB6	-4.0249653
SSXB8	-2.4825006
ST13	-0.6439681
ST18	-2.09269
ST3GAL3	-2.700426
ST3GAL4	-1.9073491
ST3GAL5	-3.386994
ST5	-0.8489132
ST6GAL1	-3.2674646

ST6GAL2	-1.0987267
ST6GALNAC4	-0.2384458
ST6GALNAC6	-2.9841895
ST7	-0.2749114
ST7L	-1.3312016
ST8SIA2	-1.9267757
STAB1	-1.2819338
STAB2	-7.389632
STAC3	-0.9193454
STAG3	-2.0726225
STAMBP	-0.4948502
STAMOS	-2.2799497
STAP2	-0.7917991
STARD10	-5.5636225
STARD4	-1.5069213
STARD5	-3.5930681
STARD7	-2.331409
STAT5A	-1.0453105
STAT5B	-0.9189572
STAU1	-1.0906649
STBD1	-2.3016572
STC1	-1.5385628
STEAP2	-0.4382362
STEAP3	-1.7574477
STEAP4	-0.5608926
STIM2	-0.7229827
STK11	-1.2391162
STK16	-2.6081862
STK25	-0.1693745
STK31	-2.0999024
STK32B	-2.1708682
STK38L	-1.0387068
STOM	-1.0646977
STOML2	-1.886559
STOX2	-0.6979423
STPG2	-2.0013113
STRA13	-1.7394295
STRA8	-0.2906418
STRADB	-1.75489
STRAP	-1.3177619
STRBP	-1.2197673
STRM	-2.0718524
STRN4	-0.3652492
STS	-2.714851
STT3A	-0.6449089

STT3B	-0.5869422
STUB1	-1.7027087
STX17	-0.5401502
STX18	-0.6719575
STX2	-0.9452717
STX4A	-0.3583574
STX5A	-0.5189338
STXBP2	-0.7780271
STXBP4	-0.3801763
STXBP5L	-2.058811
STXBP6	-0.794589
SUCLA2	-2.2641406
SUCLG1	-3.1170287
SUCLG2	-4.8445234
SUCNR1	-8.300907
SUDS3	-2.3190317
SUGCT	-4.904253
SUGP1	-0.206471
SULT1A1	-3.409605
SULT1B1	-9.675133
SULT1C1	-1.9785957
SULT1C2	-7.5209475
SULT1D1	-3.8701086
SULT1E1	-9.875339
SULT2A1	-14.640081
SULT2A2	-14.634623
SULT2A3	-8.349548
SULT2A4	-12.607882
SULT2A6	-13.145536
SULT2A7	-4.1124105
SULT3A1	-9.702054
SULT4A1	-2.7915978
SULT5A1	-0.7753863
SUMF1	-0.7413158
SUMF2	-2.05244
SUMO3	-1.8174009
SUN2	-0.2933106
SUN3	-3.908213
SUN5	-0.9135664
SUOX	-3.7510195
SUPT16	-0.2558231
SUPT3	-1.8138201
SUPV3L1	-1.1199574
SURF1	-1.3958106
SURF2	-1.5789065

SURF4	-0.7955222
SUSD1	-1.4640498
SUV39H2	-1.8950496
SUV420H2	-0.5416737
SV2B	-2.3452811
SVAL2	-2.2143135
SVIP	-1.0888171
SVOP	-2.7086737
SVS1	-1.8157134
SVS2	-2.2707577
SVS3A	-2.1569033
SVS3B	-2.2428703
SVS4	-2.109284
SVS6	-2.264689
SWI5	-0.8109083
SYAP1	-1.284338
SYBU	-2.4160025
SYCE2	-0.7546372
SYCE3	-4.3007593
SYCP1	-2.075706
SYCP1-PS1	-2.1358137
SYCP2L	-1.5707393
SYCP3	-1.4305894
SYMPK	-1.3341289
SYN1	-1.571888
SYNB	-0.5942488
SYNDIG1	-1.9371173
SYNDIG1L	-3.4629006
SYNE3	-0.574276
SYNE4	-1.0206609
SYNGAP1	-0.4844542
SYNGR2	-0.6408052
SYNGR3	-1.0447037
SYNGR4	-2.3834195
SYNJ2	-0.7234037
SYNJ2BP	-0.9570794
SYNPO2L	-1.8396187
SYNPR	-1.6229787
SYP	-0.6484227
SYPL	-0.1789141
SYPL2	-2.4091387
SYS1	-1.6808572
SYT1	-5.428543
SYT10	-4.9594526
SYT17	-1.2047386

SYT2	-2.0663981
SYT3	-3.4073236
SYT4	-1.986289
SYT6	-3.2606356
SYT8	-1.0963664
SYT9	-1.5209613
SYTL1	-1.3572803
SYTL4	-0.6859169
SYTL5	-2.5883243
SYVN1	-0.8480148
SZRD1	-1.2110472
SZT2	-0.4100308
TAAR1	-2.0708437
TAAR2	-2.0613756
TAAR3	-1.8502862
TAAR5	-2.2061906
TAAR7A	-2.2223165
TAAR7D	-2.1361754
TAAR7E	-2.1754477
TAAR8A	-2.1313756
TAAR8B	-2.1363912
TAB1	-0.4437051
TAC1	-2.068255
TACC3	-0.3428559
TACO1	-3.2608838
TACO1OS	-1.5870721
TACR3	-2.1901066
TADA1	-0.4563866
TADA2A	-0.8782439
TADA2B	-0.4719417
TAF10	-1.2178235
TAF11	-0.4221149
TAF13	-0.4880829
TAF15	-0.5570068
TAF2	-0.2563763
TAF6L	-1.1238017
TAF9	-1.3244076
TALDO1	-0.8925734
TAMM41	-0.6219392
TANGO2	-2.9166193
TAOK2	-0.427794
TAOK3	-0.2454023
TAP2	-0.2848148
TAPBPL	-0.4188094
TAPT1	-1.5405235

TARBP2	-0.4289379
TARS	-1.5523124
TARS2	-1.5499864
TARSL2	-2.1074092
TAS1R2	-2.174288
TAS1R3	-3.0236278
TAS2R102	-1.9621398
TAS2R103	-1.9532526
TAS2R104	-3.7390146
TAS2R105	-2.2230172
TAS2R107	-1.4830256
TAS2R108	-2.0979662
TAS2R109	-0.8939726
TAS2R110	-1.8043487
TAS2R113	-1.9090202
TAS2R114	-3.1132245
TAS2R115	-1.6066489
TAS2R116	-2.232288
TAS2R117	-1.8252065
TAS2R118	-1.6325307
TAS2R119	-2.372026
TAS2R120	-2.0836563
TAS2R121	-2.2472038
TAS2R122	-2.1059358
TAS2R123	-2.215247
TAS2R125	-2.145832
TAS2R126	-1.8971579
TAS2R129	-1.687532
TAS2R130	-1.9338107
TAS2R135	-1.8862517
TAS2R136	-1.9792945
TAS2R138	-2.6428277
TAS2R139	-1.7250166
TAS2R140	-2.0209515
TAS2R144	-2.0576735
TAT	-10.557934
TATDN1	-0.6161883
TATDN2	-2.0630798
TATDN3	-1.7661672
TAZ	-0.7283444
TBATA	-0.9813986
TBC1D13	-1.252821
TBC1D15	-0.3465395
TBC1D17	-1.1171179
TBC1D21	-1.0582421

TBC1D22A	-0.3024907
TBC1D24	-2.5881689
TBC1D2B	-0.2090669
TBC1D31	-1.6483037
TBC1D7	-2.8332198
TBC1D8B	-0.418715
TBC1D9B	-0.5896163
TBCA	-1.2360778
TBCCD1	-2.0151844
TBCD	-1.2520556
TBCEL	-1.2822518
TBL2	-1.2268221
TBL3	-1.3301153
TBP	-0.3116665
TBPL2	-1.9675708
TBR1	-2.1457238
TBRG4	-1.3391573
TBX15	-1.5376449
TBX18	-2.142458
TBX20	-0.7027609
TBX3OS1	-2.0932503
TBXA2R	-2.3289335
TBXAS1	-1.2224326
TCAIM	-1.4299722
TCAM1	-1.4931612
TCEA1	-0.7177315
TCEA2	-0.8223164
TCEA3	-2.5892692
TCEAL5	-2.8566298
TCEB1	-0.184072
TCEB2	-0.6078253
TCEB3	-0.394506
TCERG1L	-1.8083875
TCF19	-1.2635269
TCF24	-4.792281
TCF25	-0.9559441
TCF3	-0.4181461
TCF7L1	-0.8166065
TCHHL1	-2.0165927
TCIRG1	-1.83356
TCL1	-2.0801978
TCL1B1	-2.0590696
TCL1B3	-1.6867337
TCL1B4	-1.324444
TCL1B5	-1.9729786

TCN2	-1.0630946
TCP1	-0.4201694
TCRA	-1.3174419
TCRB-J	-0.5201943
TCTA	-1.268539
TCTN1	-1.2181358
TDH	-0.6786523
TDO2	-9.836445
TDP2	-1.6534071
TDPOZ1	-3.5332322
TDPOZ2	-2.1739972
TDPOZ3	-1.9529047
TDRD1	-2.0744774
TDRD6	-2.0369458
TDRP	-2.0431476
TEC	-2.2180727
TECPR2	-0.7636714
TECR	-3.219027
TECTA	-1.9778805
TECTB	-1.9384322
TEDDM1	-2.8670805
TEF	-0.3472357
TEFM	-1.6406384
TELO2	-1.1739454
TENM1	-1.6633744
TENM3	-3.2837617
TENM4	-1.9491353
TEP1	-0.4767289
TERF1	-0.7446952
TESK2	-1.9468274
TET1	-2.7099261
TEX101	-1.6612785
TEX12	-3.2962523
TEX14	-2.4537494
TEX16	-3.6783724
TEX18	-2.2392166
TEX19.1	-2.3573868
TEX2	-3.0631628
TEX21	-1.4686255
TEX22	-6.216119
TEX24	-2.0614314
TEX261	-0.8350024
TEX264	-1.9702458
TEX33	-1.9857068
TEX35	-2.018161

TEX36	-0.9799142
TEX37	-1.4438379
TFAM	-1.9533448
TFAP2B	-2.2631352
TFAP2D	-1.2701373
TFAP2E	-2.0954225
TFAP4	-2.2388933
TFB1M	-1.9795375
TFB2M	-1.5837936
TFCP2	-0.824846
TFDP1	-0.6876035
TFEB	-0.7642007
TFEC	-0.9191876
TFF3	-7.839992
TFG	-0.7068906
TFIP11	-0.9577413
TFPI2	-5.582143
TFPT	-1.0797873
TFR2	-10.785865
TFRC	-0.4691982
TGDS	-1.6556859
TGFA	-1.1702721
TGFBRAP1	-1.0211115
TGM3	-0.9771309
TGM4	-0.9620583
TGM5	-2.0965507
TGM7	-2.2768207
TGS1	-0.2209606
TGTP1	-0.9548183
TGTP2	-0.7469606
THA1	-1.3670616
THADA	-0.7759399
THAP11	-0.784965
THAP3	-0.9623652
THAP4	-1.1998715
THAP6	-0.889359
THAP7	-1.166451
THBS2	-1.8070953
THEM4	-2.0226505
THEM5	-0.4406436
THEM6	-1.491533
THEM7	-7.1512413
THEMIS3	-1.7749207
THNSL1	-0.5785217
THNSL2	-4.35254

THOC1	-0.1994619
THOC3	-1.2568917
THOC5	-0.7597561
THOC7	-1.4196758
THOP1	-1.3558893
THPO	-4.452317
THRB	-1.4990852
THRSP	-7.7316117
THSD1	-0.6677947
THSD7B	-2.1004956
THTPA	-2.3993635
THUMPD1	-0.4330678
THUMPD2	-0.8899024
THUMPD3	-0.4998007
THYN1	-1.0155468
TIAL1	-0.5702758
TICAM1	-1.0631824
TIGD2	-0.3585811
TIGD4	-0.5154102
TIGD5	-1.9270747
TIMD2	-8.706593
TIMD4	-4.0530624
TIMM10	-1.6554446
TIMM10B	-0.6468802
TIMM13	-2.2897654
TIMM17A	-0.175477
TIMM17B	-2.104773
TIMM21	-1.6169133
TIMM22	-0.8629746
TIMM23	-1.5937676
TIMM44	-1.9909167
TIMM50	-1.9905152
TIMM8A1	-3.2404137
TIMM8B	-1.6075182
TIMM9	-1.6395774
TIMMDC1	-1.7724571
TINF2	-0.9597592
TIPIN	-0.2377887
TIPRL	-0.3426166
TJP3	-1.8951292
TK1	-4.062481
TK2	-1.0107889
TKT	-1.5355086
TKTL1	-2.091543
TLCD1	-3.8990426

TLCD2	-4.5297194
TLDC1	-1.7625532
TLK1	-0.5441442
TLL2	-2.3565402
TLR1	-0.3927894
TLR12	-3.2303119
TLR3	-1.2047069
TLR5	-2.0681663
TLR9	-3.8905835
TLX2	-2.1731224
TLX3	-2.0316434
TM2D1	-0.3250289
TM2D2	-1.2290125
TM2D3	-1.2615232
TM4SF20	-2.1856813
TM4SF4	-6.4983187
TM4SF5	-1.7989802
TM6SF2	-6.0114822
TM7SF2	-6.412522
TM7SF3	-2.6875076
TM9SF1	-1.1684704
TM9SF2	-1.198442
TM9SF4	-1.0410013
TMA7	-0.615324
TMBIM4	-1.6204338
TMBIM6	-2.712687
TMBIM7	-1.3356271
TMC1	-1.5613208
TMC2	-1.4060616
TMC3	-2.6976924
TMC6	-0.5869918
TMC8	-0.5322266
TMCC1	-0.2615876
TMCO1	-1.5074315
TMCO2	-1.8803375
TMCO5	-3.9519851
TMED1	-2.1203241
TMED10	-0.2337937
TMED11	-1.4707346
TMED2	-0.7714338
TMED4	-1.6943097
TMED5	-1.0577426
TMED6	-1.8612609
TMED7	-1.1992893
TMEM101	-0.6554399

TMEM102	-1.9762042
TMEM104	-0.8200898
TMEM106A	-0.8183813
TMEM106B	-2.2285304
TMEM106C	-2.777796
TMEM11	-0.4298573
TMEM110	-0.7091379
TMEM115	-1.2312407
TMEM120A	-3.6060586
TMEM125	-0.630825
TMEM126A	-2.3492503
TMEM126B	-1.9961586
TMEM129	-1.4578896
TMEM130	-1.8861537
TMEM132E	-6.084711
TMEM134	-1.4345365
TMEM135	-2.6761484
TMEM141	-3.6765285
TMEM143	-2.2266989
TMEM144	-3.0923548
TMEM145	-2.1891732
TMEM147	-2.5894923
TMEM14A	-1.0512953
TMEM14C	-2.3606882
TMEM150A	-4.445985
TMEM150B	-1.9579394
TMEM150CC	-2.2623863
TMEM158	-0.7725306
TMEM160	-1.0031409
TMEM161A	-1.1535206
TMEM163	-1.1720071
TMEM167	-0.595398
TMEM168	-0.5103483
TMEM169	-1.3718395
TMEM170	-0.7516394
TMEM174	-0.8071387
TMEM175	-0.9965143
TMEM176A	-0.9640341
TMEM176B	-1.0677972
TMEM177	-1.3053522
TMEM178B	-0.6075342
TMEM179	-0.957011
TMEM179B	-0.8038836
TMEM18	-1.5493932
TMEM180	-1.126111

TMEM182	-1.3637595
TMEM183A	-2.6280055
TMEM184A	-0.6373115
TMEM184C	-0.3223033
TMEM186	-1.0722084
TMEM19	-3.006382
TMEM191C	-1.4462368
TMEM192	-1.1094966
TMEM194	-0.5050888
TMEM196	-1.6137302
TMEM198B	-0.579133
TMEM199	-0.8167841
TMEM200B	-1.3970573
TMEM201	-1.7461581
TMEM203	-2.4325404
TMEM205	-4.658755
TMEM207	-2.1775243
TMEM208	-1.2691875
TMEM209	-0.5757072
TMEM210	-0.8985348
TMEM214	-1.3858881
TMEM215	-0.660666
TMEM217	-1.5859754
TMEM218	-1.0083303
TMEM219	-1.8485246
TMEM220	-2.870553
TMEM222	-1.0773525
TMEM223	-1.4470143
TMEM225	-2.1016164
TMEM229A	-2.4506114
TMEM230	-0.7424469
TMEM233	-1.5912449
TMEM234	-0.9529791
TMEM235	-2.0294633
TMEM236	-2.1451004
TMEM238	-1.7178657
TMEM239	-1.5944095
TMEM241	-1.0522897
TMEM242	-1.6326823
TMEM248	-0.9548736
TMEM25	-5.0536437
TMEM253	-0.9954889
TMEM254A	-3.6725338
TMEM254C	-3.7959108
TMEM256	-2.414517

TMEM258	-0.3220754
TMEM259	-1.815352
TMEM26	-0.9028683
TMEM260	-0.6796012
TMEM261	-1.378295
TMEM263	-0.3571701
TMEM27	-2.1868546
TMEM29	-1.0009694
TMEM30A	-0.9368839
TMEM30B	-2.8384757
TMEM30C	-1.392261
TMEM33	-0.4337902
TMEM37	-2.6284628
TMEM38B	-1.9500785
TMEM39B	-0.8410082
TMEM41A	-1.8572631
TMEM41B	-0.4910393
TMEM42	-0.6822009
TMEM45B	-0.789999
TMEM5	-1.6226363
TMEM50A	-1.0532684
TMEM51	-1.272089
TMEM51OS1	-0.3345344
TMEM52	-1.344218
TMEM52B	-2.203899
TMEM53	-4.6540318
TMEM55A	-0.6588459
TMEM55B	-0.566236
TMEM56	-3.9725332
TMEM57	-1.2927694
TMEM59	-1.4198647
TMEM60	-1.1463537
TMEM62	-1.3471417
TMEM63B	-1.6748447
TMEM64	-1.5674405
TMEM66	-0.5518208
TMEM67	-0.3095272
TMEM69	-0.8436301
TMEM70	-2.1199193
TMEM72	-2.341585
TMEM74	-2.235807
TMEM74B	-1.2151589
TMEM8	-1.0589733
TMEM80	-0.1536665
TMEM82	-5.336231

TMEM86A	-3.1966476
TMEM86B	-7.306298
TMEM88	-0.35676
TMEM88B	-1.9615297
TMEM9	-2.0628576
TMEM91	-2.7132697
TMEM92	-2.0938606
TMEM97	-4.887149
TMEM98	-0.5069108
TMIE	-4.04566
TMIGD1	-2.1006522
TMLHE	-1.9924197
TMPRSS11A	-2.3057551
TMPRSS11B	-0.9973946
TMPRSS11C	-2.2149858
TMPRSS11D	-2.0020354
TMPRSS11E	-2.157946
TMPRSS11F	-1.9500186
TMPRSS11G	-2.173762
TMPRSS12	-1.5076828
TMPRSS2	-0.1888814
TMPRSS3	-1.7476907
TMPRSS5	-2.2082126
TMPRSS6	-11.628983
TMPRSS7	-3.2207143
TMSB15A	-1.8593516
TMSB15B1	-0.6371563
TMTC2	-0.7671738
TMTC4	-1.013041
TMUB1	-0.6334114
TMUB2	-1.3596725
TMX1	-1.0345898
TMX2	-2.212472
TMX4	-0.5264659
TNFAIP8L1	-3.4064093
TNFRSF11B	-3.026513
TNFRSF19	-0.3936644
TNFRSF1A	-0.475544
TNFRSF22	-1.8326552
TNFRSF8	-1.8031328
TNFRSF9	-1.6735878
TNFSF12	-0.2045097
TNFSF13OS	-1.5353954
TNFSF18	-2.3572698
TNFSF4	-2.1665342

TNK2	-0.7459879
TNK2OS	-1.6970062
TNMD	-2.1252759
TNNI1	-1.6833286
TNPO1	-0.3330722
TNPO2	-1.1702676
TNPO3	-0.2457619
TOB1	-2.468052
TOB2	-1.2285104
TOLLIP	-0.4969683
TOM1	-1.0082169
TOM1L1	-0.469801
TOMM20	-0.9371114
TOMM22	-1.0671072
TOMM34	-0.2337337
TOMM40	-1.9060907
TOMM40L	-0.4919438
TOMM5	-1.1906233
TOMM6	-1.4164352
TOMM70A	-1.6865687
TOMT	-1.5036793
TONSL	-0.6725674
TOP1MT	-3.755066
TOP3A	-0.6408186
TOP3B	-0.6073418
TOPAZ1	-1.9515109
TOPORS	-0.2957163
TOR1A	-2.1766043
TOR1AIP2	-1.3668799
TOR1B	-0.3826771
TOR2A	-1.8416796
TOX	-3.0800412
TPBPA	-2.091243
TPCN1	-2.246808
TPD52L1	-4.7650795
TPD52L2	-0.7190151
TPGS1	-0.557869
TPGS2	-0.5829773
TPH2	-2.2062423
TPI1	-3.3860784
TPK1	-1.657448
TPMT	-6.499669
TPO	-2.0447176
TPP1	-1.5275555
TPPP	-1.7400396

TPPP2	-1.942399
TPRA1	-0.9593601
TPRKB	-1.9353919
TPRN	-1.0200491
TPSAB1	-1.6787233
TPST1	-2.3120391
TPT1	-1.1533618
TPTE	-2.0780022
TRABD	-2.2274103
TRADD	-1.1289177
TRAF4	-2.6846051
TRAFD1	-0.5845275
TRAM1	-1.8521118
TRAM2	-0.3099046
TRANK1	-0.3015719
TRAP1	-3.3980713
TRAP1A	-2.0956004
TRAPPC1	-1.3143029
TRAPPC11	-0.5576687
TRAPPC12	-1.4875488
TRAPPC13	-0.867043
TRAPPC2L	-0.7042575
TRAPPC3	-1.5486693
TRAPPC3L	-2.3213944
TRAPPC5	-1.4024096
TRAPPC6A	-1.3570776
TRAV12-1	-1.6758332
TRAV14D-3-[	-0.7512662
TRAV14N-1	-2.1891382
TRAV3-4	-2.7095778
TRAV7N-5	-2.3066573
TREH	-2.0786037
TRF	-4.139343
TRGV2	-2.082215
TRH	-1.5507805
TRHDE	-2.166294
TRHR	-2.2103128
TRIAP1	-1.3156266
TRIB3	-1.2764964
TRIM14	-2.2098684
TRIM17	-0.2099974
TRIM2	-1.6521053
TRIM24	-1.1678452
TRIM25	-1.2840571
TRIM26	-0.4418149

TRIM27	-0.4032455
TRIM28	-1.1592779
TRIM31	-2.040685
TRIM38	-2.085841
TRIM40	-0.6176012
TRIM45	-0.5914917
TRIM52	-2.080303
TRIM6	-1.5262532
TRIM60	-2.1817703
TRIM66	-1.554261
TRIM67	-1.83775
TRIM68	-0.6023688
TRIM7	-1.2898803
TRIM71	-1.6583438
TRIM75	-2.0378692
TRIM9	-0.5224924
TRIML1	-2.0650918
TRIP13	-0.7298346
TRIP4	-0.2794805
TRIP6	-0.7159486
TRIT1	-1.4957438
TRMT1	-0.5174651
TRMT10A	-0.9915962
TRMT10B	-0.2425888
TRMT10C	-0.7609963
TRMT11	-1.1990914
TRMT112	-0.5181723
TRMT12	-0.9901748
TRMT2B	-1.4841595
TRMT5	-1.1567557
TRMT61A	-0.7275434
TRMT61B	-0.9359999
TRMU	-1.7549729
TRNAU1AP	-1.2108898
TRP53	-0.3362312
TRP53INP1	-0.2444949
TRP53INP2	-1.3606396
TRP53RK	-0.8139339
TRP53TG5	-2.0312839
TRP63	-0.5067587
TRPA1	-3.7886498
TRPC4	-2.2977893
TRPC4AP	-0.9685197
TRPC5	-1.6896873
TRPC5OS	-2.454427

TRPC7	-1.9836438
TRPM2	-0.8984652
TRPM3	-0.8767991
TRPM4	-0.4061935
TRPM7	-0.3051992
TRPM8	-0.7716074
TRPT1	-1.0105472
TRPV1	-3.646786
TRPV3	-2.1527488
TRPV5	-2.2577178
TRRAP	-0.5747733
TRUB1	-1.4629245
TRUB2	-1.0784192
TRY5	-1.7308102
TSC1	-0.856359
TSC22D2	-1.8847156
TSC22D4	-0.2291198
TSEN15	-0.504631
TSEN2	-1.8112569
TSEN34	-1.3710809
TSEN54	-0.8244023
TSFM	-1.5497012
TSGA8	-1.5456929
TSHR	-2.238247
TSHZ2	-0.7658567
TSKS	-0.8491778
TSKU	-1.4589117
TSN	-0.7138181
TSNAX	-0.3456311
TSPAN10	-2.1407392
TSPAN12	-3.6375637
TSPAN14	-0.2036233
TSPAN31	-2.932405
TSPAN33	-1.891788
TSPAN4	-1.9367399
TSPY-PS	-2.0147605
TSR3	-0.6862912
TSSC1	-0.4412723
TSSC4	-1.0737119
TSSK1	-1.590992
TSSK2	-2.353858
TSSK3	-1.0533361
TSSK5	-1.6341002
TSSK6	-0.1744232
TST	-4.103867

TSTA3	-2.0706544
TSTD1	-4.79099
TSTD3	-3.4897017
TTBK1	-3.3932247
TTC1	-0.6975021
TTC13	-1.1599722
TTC17	-0.4805236
TTC19	-1.2129679
TTC23	-3.6458483
TTC24	-1.616416
TTC27	-0.7549367
TTC3	-0.3381944
TTC30A2	-0.3444905
TTC30B	-0.4641595
TTC32	-1.6218748
TTC33	-0.591785
TTC36	-8.186899
TTC37	-0.5709403
TTC38	-0.6192064
TTC39A	-2.208074
TTC39C	-3.891437
TTC4	-0.9702268
TTC7	-1.2103033
TTC9B	-1.7976747
TTF1	-2.2838855
TTF2	-0.2654643
TTI1	-0.1658697
TTI2	-0.3654475
TTK	-0.6288858
TTLL12	-1.492661
TTLL13	-2.0642478
TTLL4	-0.2036252
TTLL5	-0.8420298
TTLL8	-1.8398116
TTPA	-6.529095
TTR	-12.372901
TTYH1	-0.2299264
TTYH2	-4.3676734
TUB	-0.269084
TUBA1C	-1.2533875
TUBA3A	-0.41927
TUBA4A	-1.3239207
TUBB2A	-1.7843795
TUBB2A-PS2	-0.8625832
TUBB3	-0.6643603

TUBD1	-0.7796578
TUBE1	-0.5590286
TUBG1	-2.456194
TUBGCP2	-1.0512652
TUBGCP6	-0.535378
TUFM	-2.410985
TUG1	-0.2091408
TULP2	-1.9238961
TUNAR	-2.0895898
TUSC2	-2.2876635
TUT1	-0.5257564
TVP23A	-0.9283068
TVP23B	-0.2775993
TWF1	-0.8702416
TWISTNB	-0.3299189
TXLNA	-0.1497517
TXN1	-0.4858561
TXN2	-2.4535813
TXNDC11	-0.3848062
TXNDC12	-1.3465147
TXNDC15	-1.5117893
TXNDC17	-0.866703
TXNDC2	-0.1867464
TXNDC5	-2.4578595
TXNDC9	-0.2703924
TXNL1	-0.5779314
TXNL4A	-0.9236746
TXNL4B	-0.8691745
TXNRD1	-0.7218471
TXNRD2	-2.753086
TYK2	-0.2356324
TYMP	-4.723894
TYMS-PS	-1.2418885
TYRP1	-2.189136
TYSND1	-2.694429
TYW1	-1.5284333
TYW5	-0.4523716
U2AF1L4	-0.4314432
UAP1L1	-1.226069
UBA1	-0.9540954
UBA3	-0.3777599
UBA5	-0.427084
UBA52	-1.1914201
UBA7	-0.2074852
UBAC1	-1.0983849

UBAC2	-1.3376708
UBALD1	-0.3954263
UBAP1	-0.9150848
UBAP2L	-0.3048892
UBB	-0.2949853
UBC	-0.9316354
UBE2A	-0.3598299
UBE2CBP	-2.861772
UBE2D2B	-1.7237389
UBE2D3	-0.4224095
UBE2DNL1	-2.364461
UBE2DNL2	-2.0797408
UBE2E1	-0.2297087
UBE2E2	-1.5881348
UBE2E3	-0.6887875
UBE2G1	-1.5868702
UBE2J1	-0.4325895
UBE2J2	-0.5966463
UBE2K	-0.4207301
UBE2L3	-0.7552905
UBE2L6	-1.220367
UBE2M	-1.3552299
UBE2QL1	-2.4340858
UBE2T	-0.9421539
UBE2U	-2.860798
UBE2V1	-0.9729924
UBE2W	-0.1452937
UBE2Z	-0.5952377
UBE3B	-1.1180787
UBE3C	-0.6457229
UBE4A	-1.5439157
UBE4B	-1.1070476
UBFD1	-0.2468076
UBIAD1	-1.4844441
UBL4	-1.1209173
UBL5	-0.6685123
UBL7	-0.9108586
UBLCP1	-0.4256549
UBQLN3	-1.893249
UBQLN4	-1.330374
UBR2	-0.7297654
UBR3	-1.0855029
UBR4	-0.24124
UBR7	-0.2406097
UBTD1	-0.5619721

UBTF	-1.677485
UBTFL1	-2.063945
UBXN1	-2.1742907
UBXN4	-2.0325582
UBXN6	-1.7320218
UBXN8	-1.3742499
UCHL3	-0.3441031
UCHL4	-1.4308238
UCK1	-1.8332582
UCKL1	-0.5763788
UCN	-2.2716687
UCN2	-1.656981
UCN3	-4.0662107
UEVLD	-0.8233745
UFC1	-1.8449855
UFD1L	-0.4559526
UFL1	-0.5075851
UFM1	-0.4410939
UFSP1	-1.7356482
UFSP2	-1.3337688
UGDH	-2.1553836
UGGT1	-0.4565568
UGP2	-3.3607173
UGT1A10	-6.4590373
UGT1A6A	-1.5113678
UGT1A6B	-4.4743347
UGT2A3	-11.33696
UGT2B1	-11.440434
UGT2B34	-13.162479
UGT2B35	-10.192818
UGT2B36	-12.918097
UGT2B37	-10.711073
UGT2B38	-13.963238
UGT2B5	-12.63341
UGT3A1	-8.676649
UGT3A2	-11.784775
UHRF1BP1	-1.3576927
UHRF1BP1L	-0.8716722
UHRF2	-0.4483781
ULK1	-0.7437286
ULK2	-0.8476691
ULK3	-0.8250203
UMOD	-2.0939946
UMODL1	-2.2905064
UMPS	-2.392107

UNC119	-0.7704902
UNC119B	-1.2064905
UNC13A	-1.1417427
UNC13C	-2.1118212
UNC45BOS	-1.0866711
UNC50	-0.7932839
UNC5A	-2.6782131
UNC5D	-1.4364128
UNC80	-2.2351515
UNC93A	-5.8153343
UNC93B1	-1.0858288
UNCX	-2.3221607
UNG	-1.6796169
UOX	-12.002151
UPB1	-9.420951
UPF1	-1.6458712
UPP2	-10.009712
UQCC1	-1.9456024
UQCC2	-1.4114175
UQCR10	-2.2673717
UQCR11	-2.731317
UQCRB	-1.5375466
UQCRC1	-3.2005954
UQCRC2	-2.1954222
UQCRFS1	-2.1278405
UQCRH	-2.144607
UQCRQ	-2.771696
URAD	-3.5415788
URAH	-5.5308022
URGCP	-1.1201539
URI1	-0.7548819
URM1	-0.9765592
UROC1	-13.484917
UROD	-2.789072
UROS	-1.941205
USB1	-0.5046926
USE1	-0.5079379
USF2	-1.5088859
USH1C	-1.6683123
USH1G	-1.6331072
USHBP1	-0.3493519
USMG2	-2.0767126
USMG5	-1.8283706
USO1	-0.5802698
USP10	-1.6387267

USP12	-0.5070686
USP14	-0.9202633
USP17LA	-1.5308836
USP17LB	-2.0864117
USP17LC	-2.7460923
USP17LD	-1.3651514
USP19	-0.9127469
USP2	-2.3281384
USP20	-0.1881909
USP21	-1.2529278
USP22	-0.3191259
USP26	-1.6248884
USP28	-0.76965
USP29	-2.170848
USP3	-0.4593711
USP30	-1.2888126
USP38	-1.2496705
USP4	-0.4671435
USP40	-1.2442718
USP44	-1.7008111
USP45	-0.4210091
USP46	-0.3239942
USP5	-1.4404206
USP50	-1.7124529
USP51	-1.6786952
USP9Y	-1.5972502
UTP18	-2.0744932
UTS2	-2.0179563
UTS2B	-2.2273824
UVRAG	-0.7591806
UVSSA	-0.507416
UXS1	-0.5630436
UXT	-1.338151
V1RD19	-2.3065703
VAC14	-1.8494496
VAMP1	-0.8439338
VAMP7	-1.1825724
VAMP8	-0.6357145
VAPB	-1.4455256
VARS	-1.2225204
VARS2	-0.4375873
VASH1	-0.8233476
VASH2	-2.073277
VAT1	-1.733428
VAV2	-1.35674

VAX1	-1.5520833
VAX2	-3.0007663
VCP	-1.1398673
VCPKMT	-0.9980459
VDAC1	-2.0392509
VDAC2	-0.7637272
VDAC3	-1.1323752
VEGFB	-1.6698217
VEGFC	-0.1785994
VEZT	-0.2643001
VGLL2	-1.8051105
VHL	-0.7189674
VIL1	-2.0132854
VIMP	-0.6607537
VIPAS39	-0.3096471
VIPR1	-5.050625
VKORC1	-4.442275
VKORC1L1	-0.8872027
VMA21	-1.0371656
VMAC	-2.4186044
VMN1R1	-2.2640376
VMN1R10	-2.0998707
VMN1R101	-1.6378617
VMN1R104	-2.4432576
VMN1R11	-2.0419734
VMN1R114	-1.9344964
VMN1R118	-2.3304617
VMN1R12	-2.089024
VMN1R121	-2.1532679
VMN1R122	-2.105537
VMN1R124	-2.0291455
VMN1R131	-2.3429437
VMN1R139	-1.8688757
VMN1R14	-2.1725423
VMN1R15	-1.8331676
VMN1R151	-1.9929783
VMN1R157	-2.269679
VMN1R167	-2.0621443
VMN1R169	-2.0991871
VMN1R170	-2.2221112
VMN1R172	-1.9712842
VMN1R173	-0.1870174
VMN1R174	-2.085403
VMN1R175	-2.0005634
VMN1R176	-2.2064888

VMN1R178	-1.3335364
VMN1R179	-2.118021
VMN1R18	-2.1777713
VMN1R180	-2.2355106
VMN1R181	-2.1442218
VMN1R183	-1.9701023
VMN1R185	-2.0756743
VMN1R186	-2.1955702
VMN1R188	-1.9944689
VMN1R189	-2.103116
VMN1R19	-2.0757728
VMN1R191	-2.3461525
VMN1R192	-3.9023175
VMN1R193	-2.3020606
VMN1R194	-1.7086945
VMN1R196	-2.2319527
VMN1R198	-2.092874
VMN1R199	-1.8867745
VMN1R2	-2.1654942
VMN1R20	-2.35844
VMN1R200	-2.1499755
VMN1R201	-2.0520263
VMN1R202	-2.1144938
VMN1R203	-2.2985756
VMN1R205	-2.001218
VMN1R206	-1.8691566
VMN1R207-F	-2.1624908
VMN1R208	-2.1037729
VMN1R209	-1.9383266
VMN1R21	-2.1164527
VMN1R210	-2.3640997
VMN1R211	-2.2409384
VMN1R212	-2.2981148
VMN1R213	-2.1829844
VMN1R214	-2.0529804
VMN1R215	-2.0803454
VMN1R216	-1.6653073
VMN1R217	-0.3883262
VMN1R218	-1.9951534
VMN1R220	-3.1065767
VMN1R222	-2.1021757
VMN1R223	-2.3720543
VMN1R224	-1.8051031
VMN1R226	-2.1346297
VMN1R227	-2.2286682

VMN1R228	-2.2999232
VMN1R229	-1.999409
VMN1R23	-2.2027955
VMN1R230	-2.1665907
VMN1R232	-2.0122023
VMN1R233	-2.1322722
VMN1R234	-1.7053227
VMN1R235	-1.8784411
VMN1R236	-2.232326
VMN1R237	-2.217785
VMN1R24	-2.0226555
VMN1R25	-0.9267995
VMN1R26	-2.2444897
VMN1R27	-1.968569
VMN1R29	-2.1619024
VMN1R30	-1.5096471
VMN1R31	-2.1918237
VMN1R32	-2.0645926
VMN1R33	-3.4616244
VMN1R35	-1.9578538
VMN1R36	-2.2526937
VMN1R38	-2.163139
VMN1R39	-2.0884938
VMN1R4	-4.758087
VMN1R40	-1.9697521
VMN1R42	-2.2120216
VMN1R43	-1.9990513
VMN1R44	-2.078399
VMN1R45	-2.2146811
VMN1R46	-2.101249
VMN1R47	-2.1046298
VMN1R48	-4.894624
VMN1R49	-2.1062174
VMN1R5	-2.2394705
VMN1R50	-2.0087807
VMN1R51	-1.6814163
VMN1R53	-3.9109225
VMN1R54	-1.9930251
VMN1R55	-2.2695577
VMN1R56	-3.1467044
VMN1R57	-2.2016957
VMN1R6	-2.1174307
VMN1R61	-2.0986304
VMN1R62	-1.6526256
VMN1R64	-2.2977018

VMN1R65	-1.5083537
VMN1R67	-2.1992507
VMN1R68	-2.103878
VMN1R69	-2.0962925
VMN1R7	-2.0961258
VMN1R71	-2.0988662
VMN1R72	-1.6966665
VMN1R73	-2.1999977
VMN1R74	-1.9630423
VMN1R75	-2.176549
VMN1R76	-0.7204421
VMN1R77	-2.3465874
VMN1R78	-2.1800385
VMN1R8	-2.1843548
VMN1R80	-1.9281492
VMN1R81	-2.2158651
VMN1R82	-2.1577575
VMN1R83	-2.3055656
VMN1R84	-2.357986
VMN1R85	-2.1834097
VMN1R86	-2.3016171
VMN1R89	-2.012033
VMN1R9	-2.0846722
VMN1R-PS10	-2.3699367
VMN1R-PS79	-2.6314201
VMN2R1	-4.121313
VMN2R10	-1.919497
VMN2R100	-2.1454756
VMN2R101	-1.8266737
VMN2R102	-1.938642
VMN2R103	-2.112272
VMN2R104	-1.6257505
VMN2R105	-2.0159323
VMN2R106	-2.2214096
VMN2R107	-2.2621174
VMN2R108	-2.3525624
VMN2R109	-2.0126908
VMN2R110	-2.204371
VMN2R111	-1.7077546
VMN2R112	-2.1492054
VMN2R113	-0.7150559
VMN2R114	-1.5510726
VMN2R115	-1.7202742
VMN2R116	-3.1261868
VMN2R118	-2.2251685

VMN2R12	-1.2075891
VMN2R120	-2.613603
VMN2R123	-2.1689985
VMN2R15	-2.0141845
VMN2R16	-1.8551788
VMN2R17	-2.3184485
VMN2R19	-1.8527789
VMN2R20	-1.5744402
VMN2R23	-2.0739357
VMN2R24	-2.3587308
VMN2R25	-2.110761
VMN2R26	-2.1381152
VMN2R27	-1.6050208
VMN2R28	-2.1639504
VMN2R29	-0.659483
VMN2R3	-1.2310214
VMN2R34	-2.066808
VMN2R35	-0.8787265
VMN2R37	-2.078759
VMN2R39	-2.0909207
VMN2R4	-1.0404937
VMN2R42	-2.33106
VMN2R48	-2.1424685
VMN2R49	-2.205579
VMN2R5	-1.9539685
VMN2R53	-2.1050012
VMN2R54	-1.4327984
VMN2R56	-2.3573422
VMN2R57	-2.0777605
VMN2R58	-1.874095
VMN2R59	-3.0351768
VMN2R61	-2.0812418
VMN2R65	-2.1647396
VMN2R66	-2.2264762
VMN2R67	-2.3971725
VMN2R68	-2.166345
VMN2R69	-2.1053011
VMN2R70	-2.00355
VMN2R71	-2.064157
VMN2R72	-2.3015683
VMN2R73	-2.032014
VMN2R75	-2.187666
VMN2R76	-2.046995
VMN2R77	-1.9395249
VMN2R78	-2.0255895

VMN2R81	-1.7499187
VMN2R82	-1.9469011
VMN2R83	-2.070397
VMN2R85	-0.5502026
VMN2R86	-1.9718719
VMN2R89	-1.7995052
VMN2R90	-1.9425337
VMN2R91	-2.130336
VMN2R94	-2.5148544
VMN2R96	-0.930203
VMN2R97	-0.7353323
VMN2R98	-2.0027003
VMN2R99	-1.9493995
VMN2R-PS10	-2.0908184
VMN2R-PS12	-1.5584645
VMN2R-PS54	-2.2944613
VMN2R-PS57	-2.3407593
VMO1	-6.08047
VNN1	-2.9512897
VNN3	-5.2403646
VPREB2	-1.9114654
VPS11	-1.0286283
VPS13D	-0.6098046
VPS16	-0.1911259
VPS18	-0.5452371
VPS25	-1.9740362
VPS26A	-0.5178204
VPS26B	-1.1960621
VPS28	-0.3248873
VPS29	-0.4118385
VPS33A	-0.6792216
VPS35	-0.525743
VPS37A	-0.2684603
VPS37B	-1.8535099
VPS41	-1.6588402
VPS45	-1.2778826
VPS52	-0.2918634
VPS9D1	-0.8366585
VRK2	-0.5338378
VRK3	-0.5413985
VRTN	-1.8078299
VSIG1	-3.4147341
VSIG4	-8.815109
VSTM2B	-2.3134797
VSTM2L	-0.6905251

VSTM5	-2.425157
VSX1	-1.6873162
VSX2	-1.9487805
VTA1	-0.5253792
VTCN1	-1.6009307
VTI1A	-0.1927519
VTN	-9.47217
VWA2	-2.0508358
VWA5B2	-1.7841437
VWA7	-1.3496556
VWA8	-3.3701515
VWC2	-2.0019593
VWC2L	-2.2299643
VWCE	-9.233886
VWDE	-3.5222046
WAP	-1.6624382
WASF1	-1.7500365
WASH	-0.9596019
WBP1	-1.4730754
WBP1L	-2.448358
WBP2	-0.5016947
WBP2NL	-2.1613555
WBP4	-0.3904815
WBSCR16	-2.1872463
WBSCR22	-0.6644068
WBSCR27	-2.885964
WBSCR28	-1.5777588
WDFY2	-0.4317398
WDR11	-0.9835622
WDR12	-1.2505698
WDR17	-1.3608489
WDR18	-2.6059566
WDR20	-0.5178275
WDR24	-2.0141778
WDR25	-2.0945106
WDR26	-0.427681
WDR31	-0.7962885
WDR33	-0.6626654
WDR34	-1.5271077
WDR36	-0.8209744
WDR4	-0.8289022
WDR41	-0.686892
WDR46	-0.7014847
WDR5	-1.4433155
WDR53	-0.7147908

WDR55	-1.0769773
WDR59	-0.1844192
WDR6	-0.5851259
WDR61	-1.6928654
WDR62	-0.4440498
WDR64	-1.5951304
WDR72	-3.0547738
WDR73	-1.3212762
WDR74	-1.2688112
WDR77	-0.4631691
WDR81	-1.5348539
WDR82	-0.5369916
WDR83	-1.3496332
WDR83OS	-1.4057832
WDR86	-0.3127954
WDR88	-2.059218
WDR89	-1.660542
WDR90	-1.0245733
WDR91	-1.064188
WDR93	-0.3990514
WDTC1	-1.4606261
WDYHV1	-0.2021337
WEE1	-0.4073134
WFDC1	-0.4863057
WFDC11	-2.1911395
WFDC13	-1.8511736
WFDC15A	-2.5039413
WFDC15B	-1.589474
WFDC16	-1.6476195
WFDC5	-1.9210684
WFDC8	-1.0027189
WFDC9	-2.2545288
WFIKKN1	-0.7820575
WFS1	-0.3098297
WHAMM	-1.0602369
WIBG	-1.5614192
WIPF3	-0.2806621
WIPI1	-0.810019
WIPI2	-1.3269606
WISP1	-1.066695
WNK3	-1.9636915
WNT10A	-1.218471
WNT2	-1.929167
WNT2B	-2.0054154
WNT3	-2.0748198

WNT3A	-2.0209248
WNT5A	-0.4997506
WNT5B	-2.0871263
WNT8A	-1.7770698
WNT8B	-3.4001427
WNT9B	-4.940209
WRAP53	-0.7400746
WRAP73	-1.0809879
WRNIP1	-1.6069417
WSB2	-0.4232073
WT1OS	-1.8872132
WWOX	-1.3864679
WWP1	-0.6664829
WWP2	-0.2554622
XAB2	-0.4721389
XIRP2	-1.2659855
XIST	-0.5683818
XK	-1.6527803
XKR4	-1.8239782
XKR5	-1.5058196
XKR6	-0.9997654
XKR7	-1.7973325
XKR8	-1.0019593
XKR9	-6.71898
XLR3A	-1.5202668
XLR5A	-1.9923754
XLR5B	-2.6702762
XNDC1	-1.4119284
XPA	-1.0578389
XPC	-0.6905618
XPNPEP1	-0.2163568
XPO5	-0.7060151
XPO6	-0.3327055
XPOT	-1.2747598
XPR1	-0.3215194
XRCC2	-0.9667103
XRCC3	-3.0566013
XRCC4	-0.5200682
XRCC5	-1.3504605
XRCC6	-1.6231008
XRCC6BP1	-2.7655826
XRN2	-0.2937179
XYLB	-5.6499414
XYLT2	-2.1366425
YAF2	-0.574182

YARS2	-1.9463263
YBEY	-1.152843
YBX1	-0.2717691
YEATS2	-0.3348069
YIF1A	-2.7935772
YIF1B	-1.5899086
YIPF1	-0.8896875
YIPF2	-1.9183106
YIPF3	-1.6420126
YIPF4	-0.6218104
YIPF5	-0.8830195
YKT6	-0.6372681
YLPM1	-0.9899051
YME1L1	-0.8201284
YOD1	-0.4722948
YPEL1	-0.2904096
YPEL2	-1.8760021
YPEL3	-0.8732934
YPEL5	-0.9146938
YRDC	-0.5472302
YWHAG	-0.9000545
ZADH2	-1.7462726
ZAR1L	-2.2253802
ZBED5	-1.621037
ZBTB17	-2.097686
ZBTB18	-0.8586807
ZBTB20	-0.6243739
ZBTB22	-0.3164382
ZBTB39	-0.5412283
ZBTB43	-0.2423415
ZBTB44	-1.0551977
ZBTB48	-2.0820727
ZBTB7B	-0.9698691
ZBTB7C	-1.1691971
ZBTB8A	-0.9870815
ZBTB8OS	-1.3184862
ZBTBD6	-0.5129643
ZC2HC1B	-2.9536154
ZC2HC1C	-2.0436425
ZC3H12B	-0.4925139
ZC3H14	-0.7836776
ZC3H7B	-0.6348105
ZC3H8	-1.2155328
ZC3HC1	-0.8470326
ZC4H2	-1.0491476

ZCCHC16	-2.109677
ZCCHC17	-0.5553765
ZCCHC24	-1.7776446
ZCCHC4	-0.4574907
ZCCHC5	-2.4479365
ZCCHC9	-1.5941243
ZCWPW1	-1.1927714
ZDBF2	-1.5415289
ZDHHC11	-2.483378
ZDHHC12	-1.9043341
ZDHHC14	-1.1434109
ZDHHC15	-0.4667559
ZDHHC16	-0.3037224
ZDHHC19	-2.159614
ZDHHC24	-0.2423201
ZDHHC25	-1.6266751
ZDHHC4	-1.2070563
ZDHHC5	-0.3343368
ZDHHC6	-2.037056
ZDHHC7	-0.4866099
ZDHHC9	-2.7235036
ZER1	-1.1134386
ZFAND1	-1.9123216
ZFAND2B	-1.4277554
ZFAND3	-0.4597039
ZFAND4	-2.5764458
ZFC3H1	-0.2885985
ZFHX2OS	-2.6853724
ZFHX4	-4.148108
ZFP108	-0.6348832
ZFP109	-2.0875058
ZFP113	-1.1541348
ZFP119B	-0.4895716
ZFP128	-0.3706222
ZFP13	-2.3078415
ZFP133-PS	-1.6384463
ZFP174	-1.8609262
ZFP189	-0.737782
ZFP191	-0.6739688
ZFP213	-1.0341272
ZFP219	-0.5864759
ZFP229	-0.3057399
ZFP239	-0.430361
ZFP264	-4.3309984
ZFP27	-0.7042007

ZFP273	-0.4568176
ZFP276	-0.3355064
ZFP277	-0.8638692
ZFP280B	-0.6852684
ZFP282	-1.9311895
ZFP286IT	-2.2264013
ZFP30	-0.626811
ZFP300	-2.3369513
ZFP316	-0.8915367
ZFP318	-0.8098626
ZFP334	-2.676332
ZFP335	-0.4920969
ZFP335OS	-1.1755371
ZFP345	-0.803503
ZFP35	-0.2174745
ZFP354A	-0.7636831
ZFP354B	-1.8200457
ZFP362	-0.4069452
ZFP367	-0.5425644
ZFP369	-0.3185177
ZFP36L2	-0.4233007
ZFP385A	-2.0363443
ZFP385B	-3.4190063
ZFP385C	-1.2500377
ZFP39	-0.8928077
ZFP395	-2.9378543
ZFP398	-0.7011764
ZFP408	-0.3867636
ZFP41	-1.2538953
ZFP42	-0.9099731
ZFP422	-0.5224519
ZFP428	-0.7873032
ZFP442	-0.7423773
ZFP444	-1.6212225
ZFP454	-1.8443699
ZFP467	-1.0188451
ZFP488	-0.5188355
ZFP507	-0.4479985
ZFP523	-0.3428173
ZFP524	-1.421782
ZFP541	-2.2115486
ZFP57	-0.2807157
ZFP574	-0.6378546
ZFP575	-2.052707
ZFP598	-0.9288573

ZFP60	-0.6793742
ZFP606	-0.2668741
ZFP612	-0.535677
ZFP616	-2.0541608
ZFP617	-0.7045302
ZFP618	-1.5595565
ZFP622	-0.3342876
ZFP623	-1.8805859
ZFP628	-1.1452703
ZFP629	-0.6264653
ZFP637	-0.9660606
ZFP639	-0.59869
ZFP64	-0.5537915
ZFP646	-0.4508781
ZFP648	-1.175096
ZFP655	-0.3077436
ZFP661	-1.531465
ZFP672	-0.3703218
ZFP688	-0.9467773
ZFP689	-0.2033453
ZFP691	-1.1775308
ZFP692	-0.4538765
ZFP706	-0.7077961
ZFP707	-2.857219
ZFP72	-0.8430584
ZFP735	-2.0893195
ZFP746	-0.45824
ZFP750	-3.3303027
ZFP759	-1.5707045
ZFP760	-0.3274958
ZFP768	-1.1590996
ZFP770	-0.828011
ZFP771	-1.9913363
ZFP775	-0.337358
ZFP784	-1.1756263
ZFP786	-2.3038135
ZFP787	-2.3132215
ZFP791	-2.0109606
ZFP799	-1.100539
ZFP804A	-2.0207877
ZFP810	-0.2458282
ZFP820	-1.0259871
ZFP825	-1.4899287
ZFP846	-0.9650834
ZFP85	-0.9358728

ZFP853	-1.6564243
ZFP85OS	-1.590457
ZFP872	-1.7565219
ZFP873	-0.9641297
ZFP874A	-1.7085223
ZFP879	-0.8120995
ZFP91	-0.3795171
ZFP932	-0.228456
ZFP936	-0.7976253
ZFP938	-0.5493968
ZFP939	-1.2747278
ZFP94	-0.6665576
ZFP940	-0.9361675
ZFP941	-1.954906
ZFP944	-0.6569002
ZFP946	-1.7255859
ZFP949	-0.8253455
ZFP951	-0.5715411
ZFP953	-1.0004697
ZFP954	-0.1866512
ZFP955A	-0.6247988
ZFP955B	-0.5989432
ZFP956	-0.522934
ZFP958	-0.7302382
ZFP960	-0.2733545
ZFP961	-0.7178903
ZFPL1	-0.7059526
ZFPM1	-2.5654588
ZFR	-0.2767005
ZFY2	-2.4893918
ZFYVE1	-1.6090794
ZFYVE19	-1.4311728
ZFYVE21	-1.6352658
ZFYVE26	-1.2083015
ZFYVE28	-1.2299621
ZFYVE9	-0.6036825
ZG16	-2.09198
ZGPAT	-0.9105387
ZGRF1	-0.5684228
ZHX3	-1.2820663
ZIC1	-0.7634909
ZIC2	-2.0399487
ZIC4	-1.6251571
ZIC5	-1.5355222
ZIM1	-1.2072842

ZIM2	-2.1346903
ZKSCAN1	-0.6213212
ZKSCAN14	-0.8189859
ZKSCAN16	-3.3515673
ZKSCAN17	-0.620029
ZKSCAN4	-1.5268335
ZKSCAN5	-1.2206264
ZMAT5	-0.9438615
ZMIZ2	-0.5909333
ZMPSTE24	-1.4232659
ZMYM3	-1.0997019
ZMYND19	-0.5660062
ZNF512B	-1.4204106
ZNHIT1	-1.188858
ZNHIT2	-2.168314
ZNHIT3	-1.1360092
ZNHIT6	-0.2067876
ZNRD1AS	-2.032065
ZNRF2	-0.5706325
ZNRF3	-1.003238
ZNRF4	-1.1208997
ZP3R	-3.0169487
ZPR1	-0.9305835
ZRANB1	-0.9739919
ZRANB3	-1.3229775
ZSCAN10	-1.200562
ZSCAN12	-0.7920756
ZSCAN2	-2.3272336
ZSCAN20	-0.9980896
ZSCAN21	-0.3137593
ZSCAN4A	-2.1580117
ZSCAN4C	-1.5235639
ZSCAN4F	-1.9636354
ZSCAN5B	-2.0515752
ZSWIM2	-1.6069963
ZSWIM3	-0.608892
ZSWIM5	-1.5553703
ZSWIM7	-2.400024
ZW10	-0.3017941
ZWILCH	-0.7487755
ZWINT	-0.9799414
ZXDA	-0.9153967
ZYG11A	-1.9664047
ZZEF1	-0.4702883
A3GALT2	0.45821047

A4GALT	3.270082
AAK1	1.8477445
AATK	0.41853333
AB041803	2.076611
ABCA13	2.36618
ABCA17	0.39240718
ABCA4	1.2796962
ABCA5	1.6931176
ABCA7	1.0608134
ABCB1B	2.0712476
ABCC1	2.042912
ABCC5	2.4072409
ABCC8	1.8153844
ABCG1	1.4596267
ABCG4	0.6616254
ABHD12B	0.47355032
ABHD13	0.6187084
ABHD17B	0.63050747
ABI1	0.8580222
ABI2	0.83873844
ABI3	0.3188424
ABI3BP	1.5322537
ABL2	0.6831622
ABLIM1	1.0172472
ABLIM3	0.40052032
ABRACL	0.6246147
ABTB2	0.3349905
ACAN	0.61733794
ACAP1	1.1488619
ACAP2	0.79275036
ACBD7	1.7535489
ACE	5.2113276
ACE2	0.8078289
ACER2	3.0165372
ACIN1	1.719913
ACKR1	1.0528474
ACKR2	3.4040875
ACKR3	4.0331993
ACOT10	1.4792562
ACOT11	1.1093283
ACOT7	0.3104148
ACOT9	1.6425953
ACOXL	5.0925307
ACPP	2.1589625
ACR	1.3450866

ACRBP	0.50232935
ACSBG1	1.13815
ACSL4	0.27684212
ACSS1	4.5019655
ACTA1	0.30318713
ACTA2	2.7514548
ACTC1	8.963499
ACTG1	0.4395337
ACTG2	4.5608015
ACTN1	0.99714565
ACTN2	2.2543845
ACTR3	0.81376266
ACVR1B	1.7422357
ACVRL1	2.5629287
ADAM10	0.39851332
ADAM15	3.0045104
ADAM17	0.9306116
ADAM19	3.101027
ADAM22	0.68301344
ADAM28	1.2982333
ADAM3	0.42423558
ADAM33	0.5031655
ADAM5	1.7927973
ADAM8	4.1443663
ADAM9	0.88983774
ADAMTS1	2.3033352
ADAMTS10	0.9687743
ADAMTS15	5.278873
ADAMTS17	2.9844933
ADAMTS2	1.1583629
ADAMTS4	5.3197618
ADAMTS5	1.2003381
ADAMTS8	3.0326953
ADAMTS9	4.156543
ADAMTSL1	1.0296803
ADAMTSL2	1.4085631
ADAMTSL3	1.7661297
ADAMTSL4	2.9489522
ADAMTSL5	1.4091294
ADAP1	0.57635975
ADAR	0.8997903
ADARB1	1.1143646
ADC	0.59731483
ADCY1	0.84331536
ADCY2	3.9895258

ADCY3	1.0686424
ADCY4	1.5853114
ADCY5	1.0312285
ADCY7	0.944016
ADCY8	1.1185541
ADCYAP1R1	2.3958995
ADD1	0.47626495
ADD3	0.62300014
ADIG	2.2043164
ADIPOQ	3.2103806
ADM	3.625969
ADNP2	0.8940525
ADORA2A	1.4585967
ADORA2B	6.1767416
ADORA3	3.406767
ADPRH	0.4856224
ADPRHL1	0.8496418
ADRB1	1.2499857
ADRB2	2.959762
ADRB3	0.6101651
ADRBK1	0.49403477
ADRBK2	1.0816531
AEBP1	2.6374989
AEBP2	1.3100748
AFAP1	2.057025
AFAP1L1	2.9353771
AFAP1L2	0.8775244
AFF1	1.7075229
AFF3	1.0311859
AFF4	0.2589264
AFTPH	0.35849476
AGAP1	2.1979127
AGAP2	1.2974157
AGAP3	0.49386072
AGBL2	1.8566382
AGER	8.131617
AGFG1	1.6809835
AGO2	0.62754726
AGO3	0.57003236
AGO4	1.4193387
AGPAT1	0.65887594
AGPAT4	4.6037507
AGPS	0.8184676
AGR2	3.58842
AGR3	4.9431653

AGRN	1.5843616
AGRP	0.826756
AGTPBP1	1.5470128
AGTRAP	0.2733431
AHCYL2	2.0107446
AHDC1	0.38928366
AHI1	2.3536506
AHNAK	3.3019123
AHNAK2	0.62998295
AHSA2	0.47472548
AHSP	1.5794353
AIDA	1.27703
AIF1L	2.9298756
AIM2	0.5846875
AIRN	0.9097271
AJAP1	0.9010489
AJUBA	0.77553034
AK1	2.684578
AK6	0.2324357
AK8	3.7410758
AK9	2.3105073
AKAP10	0.4518652
AKAP12	5.5317073
AKAP13	1.9215684
AKAP14	3.098131
AKAP2	4.828952
AKAP5	8.674387
AKAP7	0.88462925
AKAP8	0.7278781
AKAP9	0.55940914
AKIRIN2	0.19863176
AKNA	2.1073203
AKR1B3	1.0423598
AKR1B8	0.3255415
AKT3	2.7389817
AKTIP	1.0378466
ALCAM	1.5485868
ALDH1A2	3.9119487
ALDH1A3	1.7599692
ALDH1L2	0.57678604
ALDH3A1	3.7686906
ALDH3B1	1.5454187
ALG12	0.2766323
ALKBH8	0.2821722
ALOX12	0.5390196

ALOX12E	2.2624218
ALOX15	0.7878239
ALOX5	4.096627
ALOX5AP	1.9985294
ALOXE3	1.8879988
ALPK1	1.7843766
ALPPL2	0.29039288
ALS2CR12	1.9033444
AMER1	0.70576143
AMER3	1.291326
AMICA1	3.308827
AMMECR1	1.555893
AMN1	1.0999742
AMOTL1	3.1299627
AMOTL2	1.1271324
AMPD1	0.24612594
AMPD3	3.488619
AMPH	5.0878158
ANAPC10	0.70664763
ANGPT1	3.4676876
ANGPT2	3.087031
ANGPTL7	0.20088053
ANK1	2.0932844
ANK3	1.2063727
ANKFY1	1.2994139
ANKHD1	0.7392025
ANKLE2	0.76232314
ANKRA2	0.2848568
ANKRD1	6.0507393
ANKRD11	0.87115765
ANKRD12	2.7296166
ANKRD13A	0.61028194
ANKRD22	5.066899
ANKRD23	0.18074226
ANKRD29	3.092268
ANKRD32	0.8649392
ANKRD33B	1.9622955
ANKRD37	1.4579787
ANKRD42	0.93016696
ANKRD44	2.6103778
ANKRD50	0.24758005
ANKRD61	2.2343936
ANKRD66	2.134113
ANO1	3.2976747
ANO6	2.1794515

ANO9	1.7238677
ANP32B	0.42349768
ANP32E	1.6807408
ANTXR1	2.658926
ANTXRL	0.91483283
ANXA1	4.028433
ANXA11	0.4475279
ANXA2	2.5601406
ANXA3	3.9336472
ANXA4	0.18221807
ANXA5	0.36915302
ANXA8	3.3217564
ANXA9	0.7932873
AOC1	4.09629
AP1G2	0.47725272
AP1M2	3.5034342
AP1S2	2.4703608
AP1S3	1.0518885
AP3B1	0.51105356
AP5Z1	0.63594484
APAF1	1.487936
APBA1	2.0448616
APBA2	0.9520016
APBB1	3.8616295
APBB1IP	1.4789429
APBB2	2.4522471
APC2	0.5801902
APCDD1	1.3281319
APEX2	0.3084433
APH1B	1.0051422
APLF	0.84727573
APLN	6.4224524
APLNR	4.330235
APLP1	1.8486838
APOBEC4	0.6947632
APOBR	2.203899
APOD	8.171482
APOL10B	1.6611085
APOL6	0.85383606
APOL7B	0.36299992
APOLD1	1.7127872
APP	0.5906129
APPBP2	0.42927408
APPL1	0.27333975
APTX	0.3401351

AQP3	1.6067386
AQP4	0.51959324
AQP5	6.445188
AQP7	0.5184765
AR	2.0077362
ARAP2	0.50155115
ARAP3	2.6480398
ARC	1.7218001
AREG	3.408813
ARF3	0.24523401
ARFGAP3	1.283184
ARFIP2	0.4340663
ARG2	4.2382207
ARHGAP10	1.5726533
ARHGAP15	0.6960132
ARHGAP18	0.54586506
ARHGAP20	4.6181884
ARHGAP22	3.8140717
ARHGAP23	0.5816436
ARHGAP25	0.6084986
ARHGAP27	0.9101076
ARHGAP28	1.3053784
ARHGAP30	2.043644
ARHGAP31	3.013454
ARHGAP32	0.6129458
ARHGAP33	0.6606231
ARHGAP39	1.0731902
ARHGAP4	0.25617743
ARHGAP44	3.0450945
ARHGAP6	1.3705564
ARHGAP8	5.116238
ARHGAP9	1.6713514
ARHGDIA	0.29709864
ARHGDIB	1.5708408
ARHGDIG	5.5745425
ARHGEF1	0.21049786
ARHGEF17	2.2636018
ARHGEF2	1.8078814
ARHGEF25	2.0383162
ARHGEF26	0.33268023
ARHGEF28	5.4042907
ARHGEF3	0.9827981
ARHGEF38	2.8103092
ARHGEF4	0.8693602
ARHGEF5	0.29216623

ARHGEF6	1.2981105
ARHGEF7	0.6914072
ARID1A	0.2510848
ARID2	0.770103
ARID3A	2.786477
ARID3B	0.31796885
ARID4A	0.24804115
ARID4B	1.1651607
ARID5A	4.2179627
ARID5B	1.7317467
ARIH1	0.40162802
ARL10	1.0764084
ARL13B	0.8924236
ARL14EP	0.45829296
ARL15	0.3474574
ARL2	0.32072496
ARL2BP	2.0731578
ARL5B	0.4730153
ARL5C	2.3574471
ARL6	0.4975624
ARL6IP1	0.31456137
ARL8A	0.507741
ARMC2	3.6986713
ARMC3	4.362661
ARMC4	4.465731
ARMC7	0.20518112
ARMCX1	0.9978838
ARMCX2	1.730722
ARMCX3	0.8758178
ARMCX4	1.9537506
ARPC1B	1.2509432
ARPC2	0.27412796
ARPC5	0.23158121
ARRB1	0.6927695
ARRDC1	1.56851
ARRDC2	0.6399746
ARRDC4	1.433207
ARSI	0.6942735
ARSJ	3.479561
ART1	1.6555331
ART3	1.7443848
ARTN	4.2533765
ARXES1	0.8060758
ARXES2	5.3746696
ASAP1	0.5917816

ASAP2	0.7533088
ASB1	0.42593098
ASB14	2.451755
ASB3	0.54961705
ASB4	0.5584974
ASB5	0.9423125
ASB7	0.44335794
ASCC2	0.51782656
ASCC3	0.3316779
ASCL1	1.3466828
ASCL2	1.8313556
ASCL3	1.859242
ASF1A	0.8935175
ASH1L	1.6050482
ASNS	1.0596466
ASPH	0.3042779
ASPHD2	0.89814425
ASPN	2.798392
ASPRV1	6.0840335
ATAD2	0.5078368
ATAD2B	0.37539148
ATAD5	0.40069222
ATF2	0.17008495
ATF3	3.2039309
ATF7	2.5156589
ATF7IP	0.62192154
ATG9A	0.88075686
ATL1	2.062818
ATL3	0.26173496
ATMIN	0.16423559
ATP10A	3.8131638
ATP10D	1.2689457
ATP11A	3.204392
ATP13A3	0.43290806
ATP13A4	1.849406
ATP1A2	3.2689786
ATP1A3	0.69045806
ATP1A4	1.5044668
ATP1B1	1.7110991
ATP1B2	1.4082794
ATP2A1	0.9396901
ATP2A2	0.40188313
ATP2A3	3.4310384
ATP2B1	1.6758265
ATP2B4	1.2865047

ATP2C2	1.6204803
ATP6AP1	0.18165064
ATP6V0A4	5.9320254
ATP6V0D2	1.5955768
ATP6V0E	0.15753126
ATP6V0E2	0.8312106
ATP6V1C2	7.1318836
ATP6V1G1	0.4107728
ATP6V1G2	0.2933774
ATP7A	2.005416
ATP8A1	1.1808848
ATP8B4	3.7315755
ATP9B	1.7651658
ATRNL1	1.0659432
ATRX	0.65784407
ATXN1	1.2699153
ATXN1L	0.96529865
ATXN2L	0.3249035
ATXN7L1	1.4722342
AUTS2	1.8461914
AVL9	0.57846737
AWAT1	1.4078512
AZI2	0.25260592
AZIN1	0.17520952
B3GALNT1	0.9024577
B3GLCT	1.2783251
B3GNT2	0.33240604
B3GNT5	1.9881945
B3GNT7	1.2907422
B3GNT9	1.4650798
B4GALNT2	0.9501693
B4GALT2	1.6997693
B4GALT3	1.2977276
B4GALT4	2.0652084
B630019K06I	0.891093
B9D1	2.5441513
B9D1OS	1.2527328
BACH1	1.1830406
BACH2	0.18045688
BAG3	0.72267866
BAI2	0.5694258
BARX2	1.8660223
BASP1	1.2758999
BATF	2.8302484
BATF2	2.3519297

BAX	0.9436269
BAZ1A	1.6792812
BAZ1B	0.60034466
BAZ2B	1.5992904
BBIP1	1.060435
BBS1	1.2281475
BBS12	1.1489725
BBS2	2.1988049
BBS4	0.48833656
BBS9	1.2784433
BBX	0.26556492
BC021767	0.5733919
BCAM	2.7116256
BCAN	0.90781426
BCAR3	0.93102884
BCAS1	0.6008315
BCAS3OS2	2.8320823
BCL10	0.59694433
BCL11A	1.6235034
BCL11B	1.7861156
BCL2	1.2049413
BCL2A1C	4.2524705
BCL2A1D	4.270864
BCL2L1	1.7065582
BCL2L11	1.5066354
BCL2L14	1.8181534
BCL2L15	1.6715035
BCL3	2.3882618
BCL6	1.8230271
BCL6B	3.8947372
BCL9	2.122249
BCLAF1	0.8056407
BCR	0.9203558
BDKRB1	3.4605656
BDKRB2	4.2609515
BDNF	5.291429
BDP1	1.3782134
BEAN1	5.156312
BEGAIN	2.1295304
BEND4	0.8295777
BEND5	2.3125794
BEND7	2.2181225
BEST2	1.1366215
BEX1	1.6248131
BEX2	4.139961

BEX4	3.6855664
BFSP1	1.7322206
BGN	1.5534539
BHLHE22	0.84689
BICC1	1.9108758
BICD1	2.2858722
BICD2	1.4245391
BIN2	0.29732752
BIRC2	1.4316425
BIRC3	3.2338176
BLNK	1.6085596
BLZF1	0.6301141
BMF	2.166996
BMP2K	1.4214435
BMP3	4.408589
BMP4	2.34449
BMP5	1.6531358
BMP6	2.7131329
BMPER	1.5945511
BMPR1A	0.22958088
BMPR1B	2.599528
BMPR2	2.9891882
BMS1	1.5921936
BMX	1.9038229
BNC2	1.4868054
BNIP2	0.3560133
BNIPL	2.0913458
BOC	4.132844
BORA	1.5557487
BPIFA1	8.5567875
BPIFB1	6.838943
BPIFB5	5.045643
BPTF	0.6914606
BRAF	1.1108003
BRD1	1.9036794
BRD2	0.5700884
BRD4	0.7218518
BRD8	2.0221086
BRD9	0.8340931
BRINP1	4.145658
BRIX1	0.577549
BRMS1L	0.99211645
BRPF1	0.77937365
BRSK1	2.0951571
BRWD1	0.820426

BSPRY	2.7306247
BST1	4.2758636
BTAF1	0.29323673
BTBD10	1.4622111
BTBD11	1.7795529
BTBD19	0.47269154
BTBD3	0.85960937
BTBD7	1.3581455
BTG1	0.40807247
BTG2	2.1732283
BTG3	2.7534957
BTK	0.6759081
BTLA	2.6665854
BTNL10	0.37608624
BUB1	0.54652095
BUD13	0.21536493
BVES	3.1967056
BZRAP1	0.7875912
BZW2	1.4146895
C1GALT1	0.38126373
C1QTNF2	1.1591024
C1QTNF3	0.9884839
C1QTNF7	3.4843688
C2CD3	1.0780096
C2CD4A	1.4469001
C2CD4B	5.6445956
C5AR1	3.5124505
C7	4.65907
CAB39	0.58066607
CABP5	1.810133
CACHD1	2.480568
CACNA1C	3.4792247
CACNA1D	2.3996334
CACNA1S	0.34245157
CACNA2D1	3.2843513
CACNA2D2	0.8569257
CACNA2D4	2.4592712
CACNB1	0.726192
CACNB2	0.6134734
CACNB3	1.2755542
CAD	1.5829048
CALCA	4.4374137
CALCOCO1	0.7299075
CALCOCO2	0.15065098
CALCRL	3.488426

CALM1	0.30651903
CALML3	1.0912981
CALY	0.69413877
CAMK1G	1.5180604
CAMK2A	0.780051
CAMK2D	1.2195563
CAMK2G	0.27556562
CAMK4	1.2140481
CAMKK1	1.7008176
CAMKK2	1.3089409
CAMKV	0.506382
CAMP	3.692462
CAMSAP1	1.6875215
CAMSAP2	1.9568067
CAMTA1	2.7612495
CAMTA2	0.15094376
CAP1	1.2188134
CAP2	2.3458896
CAPG	2.711472
CAPN1	0.4425435
CAPN13	1.6527777
CAPN2	0.68096733
CAPNS2	2.3581123
CAPS2	2.4532576
CAPSL	2.9635677
CAR12	0.68600893
CAR13	3.4509938
CAR15	0.8490958
CAR8	0.21620011
CAR9	0.19053507
CARD11	2.1045094
CARF	0.37938356
CASC1	4.56916
CASC4	1.5406041
CASC5	0.97659755
CASK	0.8986473
CASKIN2	0.5068164
CASP1	0.687932
CASP12	0.61528134
CASP2	0.8178568
CASP4	3.2828646
CASP8AP2	1.0441089
CASQ2	2.943288
CAST	0.39643335
CASZ1	3.7012045

CATIP	0.4268446
CATSPER4	0.7457168
CAV1	4.139792
CAV2	3.522193
CAV3	3.9774194
CBFA2T3	1.8501415
CBFB	1.2075434
CBL	2.822873
CBLB	1.8101873
CBLL1	0.40305662
CBR2	9.845379
CBR3	2.0566225
CBWD1	0.5191574
CBX5	0.7191682
CBX6	0.91152143
CC2D2A	0.88269234
CCBE1	1.1015859
CCDC102A	1.4747424
CCDC103	1.9412072
CCDC108	1.9658263
CCDC109B	1.9621651
CCDC11	4.6112814
CCDC112	0.54194593
CCDC113	6.8228135
CCDC114	0.4330125
CCDC115	0.4575014
CCDC120	0.8489032
CCDC121	2.811455
CCDC122	0.39333487
CCDC126	0.33776712
CCDC127	0.19557333
CCDC129	2.150005
CCDC13	2.8956063
CCDC132	0.52190685
CCDC134	0.2608838
CCDC135	1.473742
CCDC136	1.5799723
CCDC141	1.2439477
CCDC146	0.53175974
CCDC147	3.4442298
CCDC15	1.7717283
CCDC150	3.0662692
CCDC153	6.7453594
CCDC155	2.5947828
CCDC160	1.620584

CCDC17	1.6881177
CCDC170	2.8759797
CCDC173	2.9459288
CCDC174	0.643806
CCDC176	1.3002577
CCDC181	1.2445912
CCDC184	3.6789029
CCDC19	5.7985053
CCDC30	1.6985502
CCDC33	2.2841995
CCDC37	2.9475448
CCDC39	3.3801644
CCDC40	1.96976
CCDC42B	1.2242639
CCDC50	0.25895214
CCDC59	0.56936216
CCDC62	0.50156784
CCDC64B	3.005199
CCDC65	2.761984
CCDC66	1.2056961
CCDC67	0.90648746
CCDC68	0.72199297
CCDC69	1.0362005
CCDC71L	1.5518336
CCDC77	0.6527753
CCDC78	4.338008
CCDC81	4.0755777
CCDC85A	2.8166873
CCDC88A	2.7062821
CCDC88B	2.2717452
CCDC88C	0.88038707
CCDC92	4.06405
CCDC96	0.51510906
CCKAR	5.901823
CCL1	1.9278648
CCL11	4.7757907
CCL12	5.357309
CCL17	7.50753
CCL19	0.25676727
CCL2	4.3223877
CCL3	6.5118375
CCL4	4.254403
CCL5	1.8540192
CCL6	0.8938656
CCL7	5.3001566

CCL8	2.0249825
CCM2L	1.6993718
CCNA1	1.6327465
CCNC	0.25360155
CCND2	2.5713105
CCNDBP1	0.25548887
CCNG2	1.0772495
CCNJ	1.8898764
CCNJL	0.7646606
CCNL1	2.0053334
CCNO	2.3028774
CCNT1	0.43335962
CCNT2	0.15162039
CCNYL1	1.2501979
CCP110	1.7182088
CCR10	1.0170023
CCR1L1	0.72019005
CCR2	1.8356643
CCR6	3.1928558
CCR7	3.0957384
CCR8	0.29897428
CCR9	1.4384942
CCRL2	4.1570024
CCSAP	0.35343027
CCSER1	0.87747145
CCSER2	0.9851165
CD101	0.24919724
CD109	1.1467533
CD14	2.7672124
CD160	2.174722
CD163	0.6731038
CD163L1	3.015897
CD164L2	1.3872864
CD177	4.3754654
CD19	1.0977633
CD2	2.3148255
CD200	5.149815
CD207	1.1578479
CD209A	3.5643103
CD209C	2.2422009
CD209D	2.1195183
CD209E	2.371802
CD209F	2.1734142
CD209G	1.4791644
CD226	1.5782971

CD244	0.6468396
CD24A	4.0723968
CD27	0.30684876
CD274	2.447782
CD28	1.3028827
CD300LF	2.5148802
CD33	5.511957
CD34	4.452408
CD36	2.0468702
CD37	0.8974142
CD3D	1.8823562
CD3E	0.57776594
CD3G	1.365037
CD40	3.3402271
CD44	1.8683233
CD46	0.6820111
CD47	0.29054928
CD48	0.34235477
CD5	1.8525667
CD52	1.5551667
CD53	2.858006
CD55	0.6502781
CD6	2.1547937
CD63	1.0858512
CD69	5.395208
CD72	0.7638693
CD79A	0.61448216
CD79B	1.0089211
CD80	0.66875505
CD83	1.0967245
CD8A	1.7570772
CD8B1	2.218277
CD9	1.4803224
CD93	4.2559366
CD96	1.7705994
CD97	0.70588064
CD99	1.3477893
CDAN1	0.43126297
CDC14A	1.8746872
CDC25B	1.4560919
CDC27	0.36820078
CDC42	0.16472387
CDC42BPA	1.6643262
CDC42BPG	1.3885875
CDC42EP1	0.48537922

CDC42EP2	1.8352551
CDC42EP3	2.5637174
CDC42EP4	1.2445555
CDC42SE1	1.1794538
CDC42SE2	1.2589664
CDC45	0.82389164
CDC5L	0.7998891
CDC6	0.35246468
CDC7	0.46254802
CDC73	0.83332443
CDCA4	0.37336922
CDCA7	0.7640996
CDCP1	1.0764823
CDH1	2.0235448
CDH11	1.3021214
CDH13	0.8604355
CDH15	1.6054685
CDH16	6.302677
CDH22	2.813042
CDH5	2.1032772
CDH6	0.94212747
CDHR3	3.47746
CDHR4	4.0132413
CDK11B	0.35786676
CDK12	0.31326246
CDK13	0.41569424
CDK14	2.9541135
CDK17	0.8694687
CDK19	0.84072495
CDK5R1	0.5458038
CDK6	1.0091405
CDKL1	1.0904031
CDKL2	1.5875578
CDKL3	1.1279619
CDKL4	2.0937207
CDKL5	1.6946542
CDKN1A	1.6168733
CDKN1B	0.8979354
CDKN1C	0.9937477
CDKN2B	4.2403083
CDKN2D	1.8694873
CDNF	0.9057114
CDON	1.2119677
CDPF1	0.4926777
CDR2	1.8979416

CDR2L	0.50350237
CDS1	3.4209986
CDYL2	1.54987
CEACAM10	4.8840823
CEACAM16	1.0508466
CEBPD	4.708289
CEBPE	0.514626
CEBPG	0.49975204
CECR2	1.151814
CEL	0.79881644
CELF2	1.593338
CELF6	0.30108523
CELSR2	0.5097184
CELSR3	3.8863769
CENPC1	0.7158561
CENPE	0.46278334
CENPJ	0.72213316
CENPT	0.37651587
CEP104	0.83442855
CEP112	1.7237995
CEP112OS2	1.2735329
CEP120	1.0943108
CEP128	0.76846194
CEP135	0.49250507
CEP162	0.7290573
CEP170	1.7849708
CEP192	1.1433043
CEP290	1.3162878
CEP350	0.46561575
CEP55	0.8077047
CEP70	1.3767924
CEP76	0.2107575
CEP83	0.9230571
CEP85L	0.59586644
CEP95	0.5248642
CERCAM	1.3018608
CERK	1.5032468
CERS3	0.45036364
CERS4	1.4492745
CERS5	2.0274334
CERS6	1.3688698
CETN2	0.5035038
CETN3	0.26926994
CETN4	2.1666236
CFD	4.7511926

CFL1	0.39669895
CFLAR	1.5618482
CFTR	1.2334023
CGGBP1	0.22273827
CGN	0.36801958
CGNL1	0.68014526
CH25H	7.3673854
CHAD	4.466387
CHAMP1	0.8380568
CHD1	1.5029421
CHD2	1.8630848
CHD3OS	1.2319863
CHD5	1.7444377
CHD6	1.1472831
CHD7	1.1306839
CHD8	0.67070055
CHEK1	1.0607874
CHGA	0.55040145
CHGB	1.3212781
CHIA1	7.36826
CHIC1	0.9104824
CHIC2	1.319097
CHIL1	3.5461116
CHIL3	4.849409
CHIL4	3.0106318
CHIL5	4.9247036
CHL1	3.4137983
CHM	0.4864273
CHML	1.9184203
CHMP4B	0.42240667
CHMP4C	1.2042272
CHN1	2.5321162
CHPF	1.6041908
CHRDL1	1.8999248
CHRM2	3.2526412
CHRM3	2.7222986
CHRNA10	0.9677193
CHRNA7	0.7842605
CHRNB1	3.3617518
CHST1	6.9697337
CHST10	1.009373
CHST11	4.113372
CHST12	1.835562
CHST14	0.22367334
CHST15	0.47753096

CHST3	2.3309884
CHST5	2.1901436
CHST8	4.011756
CHSY1	1.3771048
CHTF8	0.21334505
CHTOP	0.1514883
CIC	0.45088577
CIDEA	3.0406756
CIDEC	2.419828
CILP	0.81725717
CISH	2.111343
CITED1	1.2286313
CIZ1	0.43677425
CKAP4	2.6798334
CKAP5	0.6692934
CKLF	1.8938966
CKM	1.9398062
CKMT1	0.8181801
CKMT2	6.1313767
CKS2	2.2025595
CLASP1	0.6917572
CLASRP	0.18608236
CLCA3	7.603313
CLCN6	0.44041085
CLCNKA	3.0720196
CLDN10	5.734828
CLDN15	0.4080038
CLDN18	2.6136284
CLDN22	2.3352058
CLDN5	3.4468584
CLDN7	3.4059076
CLDN8	2.1803997
CLDN9	1.0648332
CLEC10A	1.6475296
CLEC12B	0.5468395
CLEC1A	2.2964644
CLEC2I	0.9270606
CLEC3B	2.9054465
CLEC4A1	0.9070473
CLEC4A3	0.4665823
CLEC4A4	1.1582584
CLEC4B1	1.0286822
CLEC4D	5.4438753
CLEC4E	5.2542295
CLEC4N	0.3844242

CLEC5A	3.267853
CLEC9A	0.8077369
CLIC1	1.4478159
CLIC3	6.7846413
CLIC4	3.0850172
CLIC5	5.8202147
CLIC6	6.79585
CLIP1	1.438262
CLIP2	0.74402523
CLIP3	2.4007745
CLIP4	4.5816875
CLK1	0.7677536
CLK2	0.8974428
CLMP	2.813775
CLNK	2.5761683
CLOCK	0.3832445
CLSTN1	2.736299
CLSTN2	3.6308093
CMA1	1.5187614
CMIP	0.78186226
CMKLR1	1.709326
CMPK2	1.5777326
CMTM2A	1.0967517
CMTM3	0.52202654
CMTM7	0.7258601
CMYA5	1.7551951
CNBD2	1.1589077
CNEP1R1	0.5703697
CNFN	1.3503745
CNGA1	0.647321
CNGA2	2.5340178
CNIH2	0.34114337
CNIH4	0.8520436
CNKSR1	2.2366605
CNKSR2	0.79043555
CNKSR3	0.45347834
CNN1	6.370219
CNN2	2.2128372
CNNM4	0.23568726
CNOT2	0.24014378
CNOT4	1.0856943
CNPY4	0.59321404
CNR1	3.0063825
CNRIP1	0.42505884
CNTLN	0.7211299

CNTN1	5.10454
CNTRL	1.3278513
COG3	0.60023785
COG5	1.8491259
COL13A1	1.034955
COL16A1	1.6118708
COL1A1	2.041441
COL1A2	2.1359706
COL23A1	4.1358676
COL2A1	1.2460701
COL3A1	0.650537
COL4A1	3.269289
COL4A2	2.8531885
COL4A3	4.80116
COL4A3BP	0.5035615
COL4A4	3.096871
COL4A5	3.2158031
COL4A6	2.025704
COL5A2	1.0853531
COL6A1	3.0834227
COL6A2	2.5745635
COL6A3	1.7690177
COL7A1	2.8636332
COL8A1	1.3141232
COL8A2	1.5777097
COLGALT2	1.0569255
COLQ	3.9805193
COMP	1.7382812
COPS4	0.23879528
CORO1A	2.0475593
CORO1C	0.35366678
CORO2A	1.2983322
CORO2B	1.1326585
COTL1	0.55813265
COX4I2	2.5614834
COX6A2	5.613739
COX6B2	0.98737717
COX8B	6.541196
CPA3	2.829223
CPB1	0.23786926
CPD	1.6549816
CPE	3.8534002
CPEB1	1.5087814
CPEB4	1.1282587
CPED1	0.6645942

CPM	2.0987296
CPNE1	0.4051242
CPNE5	0.7145114
CPNE7	1.2705705
CPNE8	0.67610025
CPSF2	0.55905676
CPSF6	0.9125562
CPSF7	0.6157279
CPXM1	0.53496504
CR1L	0.29334497
CRABP1	0.95401406
CRAMP1L	0.8313503
CREB1	1.7994044
CREB3L1	1.7869787
CREB3L2	0.44019222
CREB3L4	1.3369391
CREBL2	0.91943216
CREBRF	1.619669
CREBZF	0.17989779
CRELD2	0.6558409
CRIM1	2.113398
CRIP1	1.4262142
CRIP2	0.2283163
CRISPLD2	5.58203
CRK	1.102118
CRLF1	5.4520235
CRLF2	0.26608944
CRLF3	1.3502674
CRMP1	2.7626433
CRNDE	1.4729631
CRTAM	2.038684
CRTC3	0.40873766
CRYAA	1.7439919
CRYAB	2.9976182
CRYBG3	2.4034405
CSF1	2.1215186
CSF2	4.8627124
CSF2RA	1.6884499
CSF2RB	1.6081915
CSF2RB2	0.8228936
CSF3	4.525467
CSF3R	1.344574
CSGALNACT1	3.1429038
CSGALNACT2	1.4508686
CSNK1A1	0.3489833

CSNK1E	1.7808695
CSNK1G1	1.3055835
CSNK1G3	0.2557764
CSPG4	5.4399695
CSRNP1	3.8733983
CSRNP3	1.4294121
CSRP1	2.150486
CST3	0.36235523
CST7	1.8025956
CST8	2.291585
CSTAD	0.9863038
CSTF2	0.4852667
CTBP2	2.394413
CTBS	1.0019603
CTDP1	0.4933939
CTDSPL	1.5060887
CTDSPL2	0.97093916
CTGF	1.9441409
CTLA2A	3.5044088
CTLA2B	2.8419785
CTLA4	1.8738425
CTNNA1	1.6604629
CTNNAL1	0.5074401
CTNNB1	0.7717066
CTNND2	2.6482403
CTPS	3.590869
CTRL	1.1104896
CTSK	2.0983653
CTSO	0.536324
CTTNBP2NL	3.287592
CTXN1	1.4925566
CUEDC1	1.2178793
CUL3	0.51374626
CUL7	1.4372396
CUX1	0.94865084
CWC22	1.87285
CWC27	0.61738205
CX3CL1	5.623307
CX3CR1	3.0497947
CXCL1	3.601387
CXCL10	3.6804585
CXCL13	0.63078785
CXCL14	5.406471
CXCL15	9.817976
CXCL16	1.8971529

CXCL17	7.778163
CXCL2	5.5286794
CXCL3	3.4859731
CXCL5	6.834544
CXCR1	2.5639818
CXCR2	4.7423954
CXCR3	0.8639822
CXCR4	2.4876199
CXCR5	1.5206833
CXCR6	1.0892558
CXX1A	0.7424989
CXX1B	0.30564547
CXX1C	0.4250207
CYB561	3.1838264
CYB5R1	0.38997412
CYB5R4	0.19578838
CYBA	1.233594
CYBB	2.056806
CYBRD1	2.9103067
CYFIP2	2.5726328
CYGB	0.32203817
CYLD	0.5518217
CYP1B1	1.8263154
CYP27B1	0.78517413
CYP2B10	0.18052006
CYP2F2	0.3881178
CYP2S1	5.501855
CYP4B1	4.183475
CYP4B1-PS2	1.488652
CYP4F18	2.149136
CYR61	2.4109955
CYSLTR2	0.7239435
CYSTM1	0.7373352
CYTH3	3.783062
CYTH4	0.35633802
CYTIP	3.9528928
CYTL1	5.2337003
CYYR1	4.5113974
DAAM2	1.329802
DAB2	0.5153189
DAB2IP	0.703949
DACH1	5.3141627
DACT1	1.075964
DACT3	0.7238121
DAF2	1.2941184

DAG1	0.962801
DAGLA	2.144707
DAPL1	2.543966
DAPP1	1.7278671
DAW1	1.0124393
DAXX	2.1720862
DBF4	0.5773163
DBH	2.3878274
DBN1	2.124207
DBNDD1	3.3379872
DCAF12L2	2.0007987
DCBLD1	0.5912161
DCDC2B	0.5489769
DCLK1	1.6361201
DCLK2	1.648951
DCLRE1B	0.738996
DCLRE1C	2.3788395
DCP1A	0.39382792
DCP1B	0.40029192
DCP2	1.5596333
DCSTAMP	2.1949174
DCTN1	0.8807831
DCTN4	0.40970755
DCTN6	0.45250893
DCUN1D3	1.9385455
DDAH2	1.3691759
DDIT3	1.0368218
DDIT4	1.6740785
DDR1	2.8452172
DDR2	2.8019047
DDX11	0.5331712
DDX17	0.7876148
DDX21	0.31685162
DDX23	1.1010973
DDX25	2.041347
DDX26B	1.5417051
DDX3X	0.22757673
DDX43	1.8497355
DDX5	0.5849056
DDX50	0.33287048
DDX55	0.80431795
DDX58	0.3122096
DDX6	1.3298931
DEDD2	0.44586802
DEF6	1.3988285

DEK	0.4304347
DENND1B	0.8611901
DENND1C	1.3774748
DENND2A	0.7226443
DENND3	2.3063517
DENND4B	1.0304742
DENND5A	0.84696674
DEP1	1.9304438
DEPDC1B	0.58805704
DES	2.0005345
DGCR14	0.8524804
DGKA	1.3181834
DGKD	1.0993862
DGKE	1.0491135
DGKEOS	0.89901614
DGKG	1.7542806
DGKH	3.9130177
DHH	1.4241402
DHRS7C	2.899373
DHRS9	2.4625182
DHX29	0.27109385
DHX9	1.9355955
DIAP1	0.82556915
DIAP2	0.823061
DIAP3	1.4934056
DIEXF	0.5931277
DIMT1	0.41957426
DIO2	5.3935738
DIP2A	0.67908716
DIP2B	0.32727718
DIRAS2	4.2778625
DKK3	1.9020448
DKKL1	1.1333075
DLC1	0.81251216
DLG4	1.7894213
DLG5	0.98002386
DLGAP4	1.2805023
DLK1	0.8358376
DLL1	2.304329
DLL4	0.881145
DMKN	4.823004
DMRT2	1.2927451
DMRT3	1.4208086
DMTF1	1.6239743
DMXL1	0.8349447

DMXL2	1.4776983
DNAAF3	0.27677155
DNAH10	3.5668998
DNAH11	4.5504475
DNAH12	2.9711301
DNAH14	0.2590239
DNAH2	3.860838
DNAH5	4.542144
DNAH6	6.2196846
DNAH7A	2.6609898
DNAH7B	3.3509638
DNAH8	2.3584216
DNAH9	3.121596
DNAIC2	4.6194434
DNAJA4	2.4920907
DNAJB1	0.94763184
DNAJB13	2.1782405
DNAJB3	2.367565
DNAJB4	0.8482909
DNAJB5	1.1269631
DNAJB6	0.6325612
DNAJB9	0.14803648
DNAJC1	0.18701172
DNAJC14	0.24813557
DNAJC2	0.92274094
DNAJC21	0.4163704
DNAJC27	1.0526974
DNAJC5	0.37712574
DNAJC5B	1.8916113
DNAJC9	0.37152958
DNALI1	5.287115
DNASE2A	1.0080786
DND1	0.22550344
DNHD1	0.79412556
DNM1	0.6233554
DNM3	2.1742585
DNM3OS	1.0634813
DNMT3L	2.4620216
DNTTIP2	0.39194155
DOC2B	4.2499394
DOCK1	0.78739405
DOCK10	1.9160328
DOCK11	1.8777924
DOCK2	1.7125173
DOCK4	0.7388263

DOCK5	1.062757
DOCK8	0.3238616
DOCK9	2.6755314
DOK1	0.7528515
DOK3	0.9731202
DOK4	1.8888903
DOK7	3.2072673
DONSON	1.5054975
DOPEY1	1.1165445
DPCR1	0.837255
DPEP1	4.5261807
DPEP2	2.668651
DPH2	0.20200872
DPP8	0.9015608
DPT	2.8461094
DPYSL2	2.773345
DPYSL3	3.1249232
DR1	0.3186865
DRAM1	2.8887582
DRC1	2.5687175
DROSHA	0.9819279
DSCAM	0.50259805
DSEL	1.5554342
DST	0.98077106
DSTN	0.4081297
DSTYK	1.9159627
DTNA	2.5776768
DTX1	0.89718246
DTX2	1.4723096
DTX3	0.4128518
DTX3L	0.3210373
DUSP1	3.4646773
DUSP11	1.6798344
DUSP14	0.6407428
DUSP16	1.514277
DUSP18	2.4849722
DUSP2	3.9173408
DUSP26	2.6290264
DUSP4	2.5910902
DUSP5	2.1626446
DUSP7	1.2047253
DUSP8	3.74235
DVL3	1.4280496
DYDC2	2.617682
DYNC1H1	0.6354852

DYNC1I2	0.57231855
DYNC1LI1	1.7363896
DYNC1LI2	1.4821701
DYNC2H1	1.4277749
DYNC2LI1	3.4969916
DYNLL1	1.8184192
DYNLRB2	8.942678
DYNLT1A	1.0167522
DYNLT1C	0.6828737
DYNLT1F	0.5010519
DYRK1A	0.3604412
DYRK3	0.98368645
DYX1C1	1.7654531
DZANK1	3.1570268
DZIP1	0.96464825
E4F1	0.59513044
EAR1	3.843563
EAR10	3.9897094
EAR12	4.0689535
EAR2	4.084855
EAR6	1.9912109
EBF1	3.8580055
EBF2	0.56111145
EBI3	3.0398636
ECM2	4.161104
ECSCR	2.4945254
ECT2L	2.1520157
EDIL3	2.8255935
EDN1	5.1011233
EDN3	1.0081112
EDNRA	2.0790148
EEA1	0.9699216
EED	0.32592106
EEF1A2	2.0460024
EEF1E1	1.4127922
EFCAB1	2.7990296
EFCAB10	4.7056694
EFCAB12	2.0949721
EFCAB14	0.83831596
EFCAB4A	1.3989425
EFCAB5	0.9293554
EFCAB9	0.32353926
EFEMP1	1.9583249
EFEMP2	1.0477118
EFHB	3.6401963

EFHC1	3.6942854
EFHC2	0.6389606
EFHD1	0.47271657
EFHD2	0.6785426
EFNA4	1.3889017
EFNB1	1.4157043
EFNB2	2.939704
EFNB3	1.6829488
EFR3B	3.76189
EFS	0.9334979
EFTUD1	0.35833502
EFTUD2	1.2253437
EGFBP2	1.6103725
EGFL6	4.1261
EGFL7	1.222095
EGFLAM	0.66458535
EGR2	2.9739003
EHBP1L1	0.5681691
EHD1	1.5257306
EHD2	4.336135
EHD4	2.1813517
EHF	3.48313
EID1	1.3208766
EID2	3.6568482
EIF2AK2	0.8764515
EIF2AK4	0.3499322
EIF2B2	0.15446377
EIF2S3Y	0.35957122
EIF3C	0.2520628
EIF4A1	0.24480867
EIF4A2	0.4295416
EIF4ENIF1	1.3617916
EIF5A2	0.70410013
ELF1	1.0606146
ELF2	0.8431301
ELF3	1.9041643
ELF4	1.3423095
ELF5	5.8666444
ELK4	0.90787077
ELMO1	1.0957649
ELMO2	0.8048754
ELMOD1	3.5390482
ELMSAN1	1.4110994
ELN	1.6046672
ELOVL7	1.7733879

EMB	3.826858
EMCN	1.5065508
EMID1	2.8712897
EMILIN2	2.034978
EML1	2.5238566
EML2	0.17958307
EMP1	4.0415316
EMP2	4.25053
EMP3	1.5722098
ENAH	3.5891085
ENC1	1.4886117
ENDOD1	1.7048507
ENG	0.6053729
ENKD1	0.5458646
ENKUR	4.693643
ENO2	1.4274094
ENO3	0.70957756
ENO4	3.1940129
ENPP4	1.3614345
ENTPD1	0.9564991
ENTPD3	3.9665565
EOGT	1.3625116
EOMES	1.6766515
EP300	0.9907322
EP400	0.70470905
EPAS1	1.0311389
EPB4.1L1	0.23738408
EPB4.1L2	2.359836
EPC1	0.86188745
EPCAM	2.8514748
EPDR1	3.7245247
EPGN	1.4026849
EPHA2	0.5169301
EPHA3	2.5014992
EPHA4	3.3968866
EPHB1	1.218219
EPHX4	3.4232366
EPN2	1.5584702
EPN3	0.34777737
EPS15	0.37200642
EPS15L1	0.17584419
EPS8	1.6985526
EPS8L1	4.1652966
EQTN	0.42332053
ERBB2	2.6616926

ERBB2IP	0.6514487
ERCC1	1.3215799
ERCC6	0.20517659
EREG	2.3322918
ERG	1.8724065
ERI1	0.3437519
ERICH1	1.0127869
ERICH2	2.1145608
ERICH3	3.5329044
ERMAP	0.21632195
ERO1L	0.46699333
ERV3	0.46072078
ESAM	2.4167619
ESCO1	0.8653884
ESM1	3.9056726
ESR2	0.992671
ESRP1	2.8097527
ESYT1	0.57592916
ESYT2	1.3365192
ESYT3	2.9341445
ETF1	0.17185163
ETL4	1.2376728
ETNK1	0.7116537
ETOHI1	0.4706397
ETS1	1.7242022
ETS2	1.5012326
ETV1	1.5958076
ETV3	2.7789402
ETV5	2.1782932
ETV6	0.883729
EVA1B	0.5407696
EVA1C	3.2479339
EVC2	1.2866578
EVI2A	0.74271345
EVI5L	0.16838598
EVL	0.24237919
EVPL	3.0860786
EXOC1	0.25639772
EXOC2	0.58175516
EXOC3L4	0.22178745
EXOC4	0.37581635
EXOC5	0.50718164
EXOC6B	1.2980027
EXOC8	0.38388395
EXOG	0.717942

EXOSC10	0.17273903
EXPH5	1.3899021
EXT2	0.24679232
EYA2	6.493166
EYA3	0.22313166
EYA4	0.73070955
EZH1	0.6692395
EZH2	0.41282225
EZR	1.891098
F13A1	4.9075885
F2R	2.6890597
F2RL2	0.5347533
F3	3.605187
FABP12	2.5406795
FABP3	6.2916336
FABP4	2.0857759
FADS3	3.0911999
FAIM	0.18850231
FAIM2	2.4927106
FAIM3	0.98790646
FAM101A	3.5248313
FAM101B	3.1478364
FAM102B	1.0920577
FAM105A	3.8245046
FAM107A	2.272407
FAM110A	0.37032318
FAM111A	1.0801606
FAM115A	0.7065191
FAM117A	1.4564066
FAM117B	1.1812458
FAM120B	0.32218266
FAM124B	2.4573398
FAM126A	0.32125187
FAM129A	1.5416474
FAM129B	2.521637
FAM129C	1.1288972
FAM131A	3.0232615
FAM132A	1.2513738
FAM133B	1.4216132
FAM134B	0.430861
FAM13B	0.61310625
FAM13C	3.5615277
FAM150A	3.2408378
FAM154B	3.7759862
FAM159A	1.6415615

FAM160A1	0.6918855
FAM161A	1.9522455
FAM161B	2.4709692
FAM162B	6.421226
FAM166B	2.5244002
FAM167A	1.5316949
FAM168B	0.5175457
FAM170B	0.6795027
FAM171B	1.3341806
FAM174A	0.41477966
FAM174B	2.5640955
FAM178A	0.27455616
FAM178B	1.3504157
FAM179A	5.0527716
FAM179B	0.67070484
FAM183B	6.918971
FAM184A	1.1945531
FAM188A	0.34971476
FAM189A2	4.8705463
FAM189B	1.0586824
FAM196A	1.8147309
FAM198B	3.1715896
FAM19A3	1.4256673
FAM19A5	1.7275422
FAM206A	0.60642576
FAM208A	0.8427758
FAM208B	1.3259363
FAM212B	3.3771486
FAM216A	0.8676276
FAM216B	4.875821
FAM217A	0.9715166
FAM217B	1.6815012
FAM219B	0.3594265
FAM220A	0.30773497
FAM221A	1.70732
FAM222B	0.74340534
FAM26E	1.8154972
FAM3B	0.5159972
FAM43A	0.8620014
FAM46B	5.7459245
FAM46C	0.94117785
FAM49A	1.3796706
FAM49B	0.84882903
FAM53B	0.7514205
FAM57A	0.25265193

FAM60A	3.8156972
FAM63B	0.9461751
FAM65A	0.9386916
FAM65B	4.307409
FAM69B	1.890131
FAM71B	0.38943768
FAM71F2	1.8646505
FAM73A	1.1742387
FAM76A	0.9274254
FAM76B	0.8660321
FAM78B	3.7960322
FAM81A	0.3044963
FAM81B	2.1320338
FAM83E	3.477413
FAM92B	5.783351
FAM98B	0.77515554
FANK1	3.1374674
FAP	3.2040133
FAR1	2.636557
FARP1	1.560195
FAS	1.6066656
FASL	1.6959562
FAT1	0.32228613
FAT3	1.6393588
FAT4	0.26305985
FBF1	0.62683344
FBL	0.2129221
FBLIM1	2.7314725
FBLN1	2.7330575
FBLN5	1.6340613
FBN1	2.647101
FBP2	1.6542561
FBRS	0.5453687
FBRSL1	0.48134947
FBXL12	0.71658087
FBXL13	2.0158055
FBXL14	0.24175549
FBXL16	3.0996125
FBXL2	0.55031323
FBXL20	0.464962
FBXL22	0.7698264
FBXL3	1.1447215
FBXL5	1.5780416
FBXL7	0.9409442
FBXO10	0.9104066

FBXO11	0.38886738
FBXO2	0.68254066
FBXO24	0.666909
FBXO27	2.0718925
FBXO30	0.556057
FBXO32	0.72601604
FBXO33	0.6670604
FBXO39	0.90815496
FBXO42	0.49019623
FBXO46	0.62302876
FBXO5	0.70602655
FBXW10	1.2565613
FBXW11	0.4179263
FBXW17	0.68937063
FBXW7	0.78523874
FCER1A	1.1880157
FCER1G	1.5702972
FCER2A	0.65586686
FCGBP	1.6182067
FCGR4	0.29008484
FCHO1	0.7111778
FCHO2	1.4298615
FCHSD1	0.52341557
FCOR	0.80798745
FCRLA	2.4179552
FCRLB	0.87686324
FCRLS	2.764464
FEM1B	0.750062
FENDRR	2.3622317
FER1L4	1.2114081
FES	0.27495527
FEV	2.0802212
FFAR2	2.0963054
FFAR3	1.1325519
FFAR4	3.428452
FGD3	1.7203603
FGD4	1.2270436
FGD5	0.43267274
FGF10	3.5441253
FGF11	1.8084731
FGF12	1.1424072
FGF17	1.1147311
FGF18	2.1681595
FGF7	4.182008
FGFBP1	5.445271

FGFR1	2.2481775
FGFR2	0.9428587
FGL2	0.704257
FGR	1.667614
FHAD1	1.7421923
FHDC1	4.8083305
FHL1	4.510974
FHL3	0.80168366
FHL5	0.8465586
FHOD1	1.0775485
FHOD3	1.7515249
FIBIN	3.9650111
FIGF	4.3744617
FIGNL2	0.34140134
FILIP1	1.1370211
FILIP1L	1.6733541
FIP1L1	0.25247622
FIRRE	1.0667324
FIS1	1.0162582
FJX1	2.4813204
FKBP10	1.1449957
FKBP14	0.5723462
FKBP1B	1.6074076
FKBP5	0.90002584
FKBP6	2.2725172
FKBP7	0.94030285
FKBP9	0.5639229
FKTN	1.7360587
FLI1	2.0366993
FLII	0.22796297
FLNA	2.0658784
FLNB	1.79989
FLNC	3.781347
FLOT1	0.51167536
FLRT2	1.1136732
FLRT3	5.64748
FLT1	2.5088482
FLT3	1.0449159
FMNL1	1.5481734
FMNL2	3.3801737
FMNL3	0.90082693
FMO2	4.1295104
FMR1	0.78052044
FNBP1L	2.2842183
FNBP4	0.30816507

FNDC1	1.285259
FNDC3B	0.764822
FNDC5	1.485116
FNDC7	1.7899723
FOS	4.4170623
FOSB	0.74196196
FOSL1	2.0895443
FOSL2	3.3507004
FOXA1	1.2428341
FOXC1	1.6808763
FOXC2	1.9794834
FOXD2	1.197175
FOXD2OS	1.2607536
FOXF1	4.555743
FOXF2	5.00276
FOXI2	1.5851433
FOXJ1	5.083496
FOXJ3	1.3958864
FOXK1	0.33248615
FOXL1	0.77195287
FOXN2	1.020967
FOXN3	0.6036906
FOXN4	2.9731877
FOXO3	1.7065423
FOXO6	2.123439
FOXP1	1.7298594
FOXP3	2.0615284
FOXP4	0.40776348
FOXRED2	1.2204239
FOXS1	1.0537295
FPR1	2.2873406
FPR2	3.0803185
FPR3	2.4149945
FPR-RS6	1.441989
FRAS1	3.1649659
FRAT2	0.54260206
FREM1	3.3613553
FRG1	0.55286264
FRMD4A	0.8916073
FRMD5	2.607928
FRMD8	0.49154806
FRMPD1	1.5101664
FRY	2.3093982
FRYL	0.19330502
FSCN1	3.042067

FSD1L	2.2698185
FSIP1	1.0904593
FSTL1	1.8958206
FSTL3	2.932612
FTX	1.0411422
FUBP1	1.1796875
FUBP3	0.5206704
FUCA2	0.79234266
FUNDC1	0.5093961
FUNDC2	1.1648297
FUS	0.24701929
FUT11	1.1347785
FUT4	2.8637483
FUT8	1.0859213
FXR2	0.24174404
FXYD2	0.67597914
FXYD3	2.2840567
FXYD4	2.3867292
FXYD5	2.0181046
FXYD6	2.3441687
FYB	1.3031168
FYN	1.2702165
FZD1	1.0211997
FZD2	2.416571
FZD3	2.8792229
FZD4	1.9956942
FZD5	0.8701155
FZD6	0.9644551
FZD9	1.4055033
G2E3	1.956295
G3BP1	0.2554779
G6B	1.2400031
G6PD2	1.2107544
G6PDX	0.21736526
GAB1	0.87524223
GAB2	0.74061203
GABBR1	1.758955
GABPB1	1.360188
GABPB2	2.087957
GABRE	0.6903727
GABRP	4.4001827
GADD45A	1.9841809
GADD45B	3.4639516
GADD45G	1.5053544
GAL	0.48513412

GALNT1	1.5815673
GALNT12	2.4099464
GALNT15	4.941923
GALNT16	3.7993393
GALNT18	3.3452296
GALNT3	3.027717
GALNT6	1.1787205
GALNT9	0.5160787
GAN	2.216556
GAP43	4.8955483
GAPT	1.6096509
GAPVD1	0.42050266
GAR1	0.43612194
GARNL3	3.253386
GAS2L1	1.2269378
GAS7	2.3132434
GAS8	1.4583349
GATA1	1.565419
GATA2	5.3385344
GATA3	2.9162316
GATAD2B	1.3648367
GATSL2	1.5100117
GBA2	0.20690393
GBAS	0.52000856
GBF1	0.19413042
GBP10	0.65309286
GBP2	1.5305533
GBP3	0.7985816
GBP4	2.6430588
GBP5	3.2836022
GBP6	0.7486844
GBP7	1.9098268
GBP9	1.3915854
GCA	1.8925643
GCC1	0.5881772
GCNT1	1.1800711
GCNT2	2.0426092
GCSAM	1.7939129
GDA	0.80103827
GDAP10	1.5362344
GDF9	0.8706496
GDI1	0.6538186
GDNF	4.2221875
GDPD1	4.4891033
GDPD2	8.261341

GDPD3	0.21174383
GDPD5	1.288969
GEM	3.2437162
GFI1	2.2524962
GFI1B	1.4738617
GFPT1	0.6179843
GFPT2	4.462103
GFRA4	3.3963122
GGA3	0.72324514
GGN	0.8226578
GGPS1	1.9342437
GGT1	2.153729
GGT5	1.107101
GGT7	1.1768382
GGTA1	1.7699394
GHRL	1.6265006
GIF	0.8511205
GIMAP1	0.6817851
GIMAP1OS	0.5623045
GIMAP3	1.3262272
GIMAP4	2.166448
GIMAP5	3.1792293
GIMAP6	2.1549773
GIMAP7	0.49431086
GIMAP8	2.2660718
GIPC2	1.4310665
GIT1	1.4379025
GJA1	3.6774445
GJA5	2.7816086
GJB3	5.3755236
GJB4	0.6982589
GJB6	6.01404
GJC1	3.5878682
GK5	0.9201124
GLA	0.28226328
GLB1L	0.54701424
GLB1L2	3.736175
GLCCI1	1.0942535
GLG1	0.69153214
GLI1	1.8686604
GLI3	2.55321
GLIPR1	2.4096131
GLIPR2	1.074873
GLIS2	1.4184175
GLIS3	0.82787275

GLS	2.3433857
GLTSCR1	0.8120122
GLTSCR1L	0.34912777
GM10044	2.079978
GM10046	1.3571756
GM10065	2.1995108
GM10091	1.7113123
GM101	2.4553688
GM10158	0.19309139
GM10183	0.8576367
GM10204	0.5957997
GM10228	0.19128513
GM10257	0.46467304
GM10325	1.3273604
GM10336	0.5387373
GM10354	1.9748101
GM10371	1.3930326
GM10404	0.34625578
GM10406	1.1158886
GM10425	1.0096426
GM10427	0.8992543
GM10428	0.5751002
GM10461	0.38937283
GM10537	1.069283
GM10554	2.9671361
GM10572	1.4433424
GM10584	0.6440911
GM10589	0.4747424
GM10629	0.57416916
GM10634	3.6519883
GM10638	1.5784326
GM10648	1.895814
GM10673	2.7844439
GM10684	0.22078371
GM10700	1.9364955
GM10781	1.3457398
GM10791	2.849493
GM10792	2.7171922
GM10796	1.2456477
GM10839	3.8102026
GM10847	1.1249816
GM10851	4.1777544
GM10866	1.762825
GM10872	0.54137707
GM10941	0.80522895

GM11110	2.5199168
GM11123	0.8749809
GM11128	0.4135015
GM11536	1.0286708
GM11564	0.6117177
GM11627	2.8237495
GM11696	1.228581
GM11744	2.7480474
GM11747	1.4545054
GM11938	1.2751236
GM11974	0.2507038
GM11992	0.99762774
GM12185	1.075424
GM12250	1.699007
GM12253	0.95927167
GM12271	0.66768074
GM12592	1.6603725
GM12657	0.21168661
GM12666	0.8505068
GM12695	0.6698649
GM128	0.54802203
GM1305	1.1029243
GM13154	1.6148887
GM13157	0.5574899
GM13212	0.6497295
GM13271	0.6143055
GM13272	1.0844216
GM13286	0.33953786
GM13298	1.1924415
GM13304	2.4578342
GM13546	1.0715482
GM13547	1.3815522
GM13570	2.364638
GM13861	1.55514
GM13889	5.305728
GM14005	0.5215776
GM14010	2.7356632
GM14085	1.4592509
GM14420	0.5693891
GM14446	2.1066294
GM14548	0.9677396
GM14625	1.0957279
GM14635	2.0376365
GM14858	0.58178973
GM14964	2.0271876

GM15056	2.8112102
GM15455	1.4667158
GM15466	0.97307396
GM15645	0.5456128
GM15998	2.7849076
GM16340	1.0677376
GM16500	0.95063066
GM16524	1.192637
GM166	0.5991287
GM1673	1.312562
GM16894	2.7290308
GM1715	2.1709673
GM17501	0.9210646
GM17758	1.1193275
GM17778	1.0558805
GM1966	3.1511865
GM1987	2.910431
GM2030	0.756366
GM2044	0.92729855
GM20555	1.0370078
GM20556	1.8354738
GM20558	7.2889657
GM20611	1.4009678
GM20767	3.7382913
GM2347	0.2274692
GM2511	2.946607
GM2590	2.24116
GM2694	0.99507666
GM2701	0.710567
GM3065	0.86294675
GM3081	1.7063212
GM3161	2.6493735
GM3239	0.95243883
GM3252	0.72097015
GM3364	2.3952508
GM3383	0.5641539
GM3414	2.0181859
GM3527	1.0518637
GM3556	1.3939118
GM3718	0.44854116
GM3893	0.83738804
GM3916	1.5562525
GM4107	1.2947495
GM4120	0.8362174
GM4123	1.588551

GM4146	1.0022101
GM4203	0.47530484
GM4425	0.65766525
GM4430	0.4099717
GM4470	0.3884344
GM4473	0.3732767
GM4610	5.232136
GM4861	0.52986836
GM4884	0.54727244
GM4907	5.450813
GM4944	0.23595095
GM4956	6.3256917
GM4983	1.668947
GM5068	2.1621842
GM5105	0.72568107
GM5106	1.1578445
GM5115	1.1040337
GM5141	1.9872365
GM5150	0.3225026
GM5168	0.72273016
GM525	0.8172145
GM527	1.99752
GM5416	5.9004397
GM5431	0.36816263
GM5483	7.5057425
GM5486	0.33089113
GM5532	1.482266
GM5547	1.3485539
GM5590	0.3033955
GM5595	0.76245356
GM5602	1.132257
GM5608	1.0177174
GM5637	0.7294388
GM5784	0.92157793
GM5797	0.94314957
GM5833	0.60428023
GM5858	1.1749601
GM5878	1.8381186
GM6115	0.45664024
GM614	1.7374616
GM6146	1.8272033
GM6377	4.220102
GM6403	4.0266476
GM6524	0.8488226
GM6557	0.66572785

GM6567	0.76799655
GM6625	0.6047511
GM6712	0.6412103
GM6729	1.5990098
GM6756	1.8328853
GM684	1.1620123
GM7068	2.4033163
GM711	0.54141164
GM7111	1.7471571
GM7265	1.0169835
GM7285	2.0513916
GM7420	2.1948564
GM7609	0.595412
GM7644	0.9016068
GM7710	1.269547
GM7894	0.34513307
GM7931	1.4281168
GM7932	1.9805965
GM7967	1.1173661
GM7969	0.99769974
GM807	1.2040462
GM8096	1.2679005
GM8212	0.6720581
GM8220	1.7443838
GM8232	2.6285374
GM8256	0.5645461
GM8369	0.61615825
GM8378	1.4191656
GM8556	1.1589489
GM867	3.8435884
GM8675	0.75268316
GM8884	3.1478229
GM8995	3.2935328
GM9049	0.31153727
GM9099	2.5525568
GM9257	1.7744126
GM9456	1.0421097
GM9510	1.8177979
GM9525	0.6574745
GM9530	1.3638482
GM9648	0.9448867
GM9693	1.214597
GM9706	3.252325
GM9733	1.73912
GM9758	1.1093504

GM9779	0.36652803
GM9798	1.0696878
GM9855	0.5144477
GM9856	1.1442752
GM9868	0.92362714
GM9889	1.393228
GM9908	1.4758556
GM9910	1.5144742
GM9917	2.0922766
GM9920	0.8724694
GM9975	1.1907213
GMEB1	0.8466306
GMFB	0.32596397
GMFG	1.8946719
GMIP	1.2183385
GMNC	0.5735018
GMPR	2.917388
GNA13	1.1787
GNA14	1.7972198
GNAI1	2.7592998
GNAI2	0.6987486
GNAL	1.4259877
GNAO1	2.8660038
GNAQ	0.6307006
GNAZ	1.6042454
GNB1	0.5860982
GNB4	1.4175034
GNB5	1.3854225
GNG11	0.4208293
GNG12	0.61758375
GNG13	5.0920005
GNG2	0.965333
GNG8	1.0619752
GNGT2	0.73444223
GNL1	0.93857384
GNL3	0.3895545
GNL3L	1.5634954
GNPDA2	1.3849707
GNPTAB	1.0298891
GNRH1	0.39393568
GOLGA3	0.5981035
GOLM1	0.21997452
GOLPH3	0.48262644
GOLPH3L	1.1500897
GOLT1B	1.3742528

GON4L	1.223711
GP1BA	3.1375203
GP2	0.45835733
GP49A	3.642325
GP5	2.2601197
GP6	1.4478962
GP9	2.6357265
GPA33	0.77451086
GPANK1	0.7379837
GPATCH2L	0.43319464
GPATCH4	0.35419798
GPATCH8	0.6346469
GPBP1	0.26654816
GPC1	0.91588545
GPC3	1.6939836
GPCPD1	1.2338333
GPHA2	0.979414
GPKOW	0.50424194
GPM6A	2.7561736
GPM6B	4.089129
GPNMB	2.0812664
GPR113	1.94752
GPR114	1.588942
GPR116	2.8614364
GPR126	2.9555206
GPR132	2.989562
GPR133	1.746551
GPR137B	1.8126388
GPR137B-PS	2.2457275
GPR141	3.3665352
GPR146	0.7265949
GPR15	0.27411103
GPR153	1.8214884
GPR160	0.41372013
GPR171	3.3789468
GPR174	1.9298542
GPR179	3.0133421
GPR18	3.1037755
GPR183	1.5630867
GPR20	1.6848176
GPR25	1.1749926
GPR26	0.51877
GPR31B	1.8425398
GPR33	1.8674276
GPR34	1.1428905

GPR35	0.6767497
GPR4	2.5571082
GPR56	1.862689
GPR6	1.0182786
GPR65	1.1478124
GPR68	2.7891207
GPR82	2.1760042
GPR84	5.973679
GPR85	2.048193
GPR88	2.077779
GPR97	0.3268528
GPRASP1	1.9818311
GPRASP2	1.1738589
GPRC5A	3.7073345
GPRC5B	4.4277053
GPRC6A	2.5811515
GPSM1	0.81090665
GPSM3	1.0291958
GPX2	3.446416
GPX3	4.7718463
GPX8	3.135363
GRAMD1A	1.7692885
GRAMD1B	1.7077556
GRAMD2	3.9278302
GRAP	2.100041
GRAP2	1.6940999
GRASP	1.2831588
GRB10	0.54713225
GREB1	1.9241495
GREM2	0.49375677
GRHL1	1.5742927
GRHL2	4.122002
GRIA1	2.8207948
GRIA3	2.4272938
GRIN2C	2.2845829
GRIN2D	0.72613525
GRIP1	1.3658657
GRIPAP1	0.5239768
GRK4	1.195015
GRK5	2.9638624
GRM6	0.25139308
GRP	2.8118398
GRRP1	6.032208
GSDMA	0.8294809
GSE1	0.5722275

GSG1	0.84034777
GSG2	1.2429721
GSK3B	0.77440786
GSN	4.454149
GTF2B	0.3307166
GTF2F1	0.4894781
GTF2F2	0.7405014
GTF3C5	0.20602226
GTPBP2	0.50852203
GTPBP4	0.47628164
GTSF1	1.9656348
GUCY1A2	1.5524368
GUCY1A3	2.5571785
GUCY1B3	2.3181005
GULP1	3.0323596
GVIN1	2.5415025
GXYLT1	1.0553913
GYG	2.2491775
GYS1	1.2221203
GZMA	2.2368813
GZMB	2.4163685
GZMC	1.0010533
GZMK	2.8240867
GZMM	1.749042
H2AFY2	0.6116338
H2AFY3	1.9783196
H2-DMB2	0.36996984
H2-EB2	3.456159
H2-OB	0.5596552
H2-Q5	0.9778447
H2-T24	2.4553595
H3F3A	0.32333612
H3F3B	0.7368555
H60A	2.7397923
H60B	3.3558939
H60C	1.450921
HACE1	0.90512896
HAP1	0.9691024
HAS1	6.9865613
HAS2	2.3518791
HAUS3	0.4928484
HAUS6	0.51504827
HAVCR2	3.676156
HBB-B1	0.61934423
HBB-B2	0.47805166

HBEGF	2.4549608
HBQ1B	1.7270248
HCAR2	2.9529047
HCK	2.0434127
HCLS1	1.8566675
HCST	1.4156446
HDAC1	0.61588526
HDAC2	0.5984459
HDAC4	1.1451211
HDAC7	2.1242337
HDAC8	0.31025958
HDC	4.571857
HDGFRP3	2.4825268
HDX	2.8094282
HEATR1	0.35161686
HEATR3	0.457654
HECA	0.3791566
HECW2	1.4330306
HEG1	1.9298472
HELZ2	0.36459637
HEPACAM	1.2750959
HEPH	1.265012
HERC1	0.49286938
HERC2	0.6131439
HERC6	1.7282414
HES1	0.7091329
HES2	0.91026783
HEY1	3.5059814
HEY2	2.3197663
HEYL	1.6909828
HHIP	1.7860556
HID1	1.6662335
HIF1A	1.7125587
HIF1AN	0.61602306
HIF3A	4.271101
HIGD1B	4.82734
HILPDA	1.4653549
HIP1	0.8364682
HIPK1	1.2423506
HIPK3	0.59395456
HIRA	0.66862774
HIST1H1B	1.5918217
HIST1H1E	1.2182097
HIST1H2AA	0.4959886
HIST1H2AB	1.5925388

HIST1H2AG	0.4606123
HIST1H2BB	1.6175752
HIST1H2BE	4.131048
HIST1H3C	1.0257788
HIST1H3D	0.5567484
HIST1H3F	1.433013
HIST1H3G	1.5395572
HIST1H4C	1.2556515
HIST1H4D	2.472602
HIST1H4J	1.612195
HIST1H4K	2.0207667
HIST2H2BB	1.3688693
HIST2H3B	1.2443199
HIST2H4	1.8609495
HIST3H2A	0.95375776
HIST4H4	2.0022297
HIVEP1	1.6215568
HIVEP2	3.3544083
HK2	1.0940738
HKDC1	5.171977
HLTF	0.7557521
HLX	1.7883644
HMCN1	2.647907
HMG20A	0.30045176
HMGA2-PS1	1.220223
HMGB1-RS17	0.30955076
HMGB2	2.1442785
HMGCLL1	2.0696268
HMGN2	0.38378763
HMGN3	2.2196612
HMGXB4	0.8429694
HMHA1	1.337039
HMX3	0.8803036
HNRNPA1	0.36092043
HNRNPA3	0.7380738
HNRNPH1	0.84238386
HNRNPH2	0.625896
HNRNPH3	0.5082207
HNRNPL	0.54836607
HNRNPM	0.67385626
HNRNPU	0.23666954
HNRNPUL1	0.67191124
HNRNPUL2	0.5367341
HOMER3	0.69000983
HOPX	2.4111943

HOXA3	1.5705116
HOXA4	0.8351655
HOXA5	3.3695495
HOXA7	0.703974
HOXB2	1.6875052
HOXB3	1.2271655
HOXB4	1.776824
HOXB5	2.2354088
HOXB5OS	2.7618408
HOXB6	3.5538003
HOXB7	1.1116323
HPCAL1	0.5847273
HPGDS	0.49878907
HPS3	0.5009608
HPS5	0.37542677
HRASLS5	1.0777624
HRC	3.0394945
HRCT1	3.0514522
HS3ST1	1.125772
HS6ST2	2.6814778
HSCB	0.31704903
HSD17B14	1.6094217
HSDL1	0.47804403
HSH2D	3.142343
HSP90AA1	0.49090576
HSPA12B	1.3357053
HSPA1A	1.4282413
HSPA1L	0.578073
HSPA2	1.7292991
HSPA4L	2.483107
HSPB1	5.710805
HSPB2	1.9411764
HSPB7	2.5920897
HSPG2	0.2468276
HSPH1	1.2641282
HTATSF1	1.3870807
HTR2B	2.1169167
HTR7	3.7829144
HTRA1	1.7785993
HTT	1.9934878
HUS1	0.75569916
HUWE1	0.21785307
HVCN1	1.2492275
HYDIN	3.0765927
ICA1	1.496171

ICAM1	3.5390153
ICAM2	2.8440623
ICAM4	0.50949264
ICAM5	2.1623921
ICOS	0.21866894
ICOSL	3.2912521
ID1	1.487598
ID3	0.2837391
ID4	0.20543432
IDO1	2.0246933
IDS	2.2146225
IDUA	0.22605896
IER2	0.89709806
IER3	4.4366636
IER5	2.1629624
IER5L	3.0810118
IFI202B	1.4457998
IFI203	0.24345922
IFI204	0.5484276
IFI27L2A	1.1288795
IFI44	0.3425126
IFI44L	3.2213695
IFI47	0.91296864
IFIH1	0.424366
IFIT1	2.6922817
IFIT2	1.1064816
IFIT3	1.0699062
IFITM1	2.2595387
IFITM10	0.75733066
IFITM5	3.1042323
IFITM6	4.978847
IFNA14	0.7726519
IFNA5	0.5634656
IFNAB	0.79102993
IFNAR1	0.6276746
IFNG	2.7526507
IFNGR1	1.3264389
IFNK	1.1500814
IFNLR1	4.212093
IFRD1	2.0961409
IFT122	1.1968894
IFT140	0.6512635
IFT43	1.8584394
IFT57	2.409995
IFT74	0.28227425

IFT80	0.74266577
IFT81	1.7058463
IFT88	0.8981085
IGF1R	3.2744799
IGF2BP1	1.0513825
IGF2BP2	1.5400147
IGFBP3	0.3970313
IGFBP5	1.744174
IGFBP6	5.30266
IGFLR1	0.7901468
IGHV14-2	2.3652558
IGH-VJ558	0.34947824
IGSF1	1.1305602
IGSF10	0.89479303
IGSF3	1.9580207
IGSF6	1.2900438
IKBKE	0.6028137
IKZF1	2.2715855
IKZF2	1.3258734
IKZF4	3.7321901
IKZF5	1.9457588
IL10	0.74110174
IL10RA	0.48525906
IL10RB	1.4384971
IL11	1.8177376
IL12B	2.040392
IL12RB1	0.302567
IL12RB2	0.8175061
IL16	2.0020933
IL17A	4.3824067
IL17D	1.9790931
IL17F	2.2826045
IL17RA	1.2611256
IL17RD	3.2914047
IL17RE	4.3935905
IL18R1	4.4500294
IL18RAP	4.1401324
IL1B	4.5395837
IL1F6	1.5133834
IL1F9	6.888989
IL1R2	8.076264
IL1RL1	2.8940904
IL1RN	2.2756953
IL20RB	1.5086188
IL21R	1.0383027

IL22RA2	3.5503724
IL23A	1.813232
IL27RA	0.8112631
IL2RA	4.114599
IL2RB	1.3071642
IL2RG	1.8949919
IL33	3.0619025
IL34	0.38494062
IL3RA	0.67282295
IL4I1	2.485302
IL4RA	2.6294155
IL6	7.458927
IL6ST	1.2993889
IL7	0.7407806
IL7R	5.624848
ILDR1	4.06277
ILF2	0.78600216
IMAGE:6196	0.9267714
IMPDH1	2.205958
INA	0.8405571
INADL	1.41751
INCENP	0.31050777
ING1	0.73872566
ING3	1.6336737
ING5	0.5824361
INHA	1.5897858
INHBB	4.967063
INIP	0.87802553
INO80	0.8207803
INO80B	1.1058474
INO80D	2.7873902
INO80E	0.54423904
INPP4A	1.7583201
INPP4B	1.6533628
INPP5B	0.21342564
INPP5D	1.2535157
INPP5J	1.331573
INTS12	1.2160435
INTS2	0.98668003
INTS8	0.1764822
IP6K3	3.5897067
IPCEF1	1.2041988
IPO13	1.0631313
IPO7	0.4810009
IQCA	4.143651

IQCB1	1.8836155
IQCF1	1.4054105
IQCK	1.0933778
IQGAP1	2.8081322
IQSEC2	0.45500946
IQUB	1.5924737
IRAK3	2.4223738
IRAK4	0.2841568
IRF1	1.1703897
IRF2	0.51628494
IRF2BP2	0.6606736
IRF2BPL	0.42574024
IRF4	1.506511
IRF5	0.8358426
IRF8	0.38874102
IRG1	4.040857
IRS2	1.1174812
IRX1	9.520087
IRX2	4.2553544
IRX3	7.314578
IRX5	3.2739587
ISG15	2.3837004
ISG20	2.6716852
ISLR2	1.5647159
ISM1	5.8129153
ITGA1	1.6040163
ITGA10	0.655879
ITGA11	2.1542244
ITGA2	1.3487165
ITGA3	2.3282404
ITGA4	1.3670893
ITGA6	1.8815241
ITGA7	0.4074726
ITGA8	3.734334
ITGAE	2.69471
ITGAL	0.7077427
ITGAM	1.1774235
ITGAV	0.38120747
ITGAX	3.065495
ITGB1	0.8095498
ITGB1BP2	1.670507
ITGB2	1.4421992
ITGB2L	5.407725
ITGB3	2.7306557
ITGB4	2.2377954

ITGB6	3.6156008
ITGBL1	1.6071448
ITK	2.0560484
ITLN1	3.0062852
ITM2A	2.5865526
ITPKB	2.5042644
ITPKC	0.3257842
ITPR1	0.19206047
ITPR3	1.3918619
ITPRIP	3.1080108
ITPRIPL2	2.075584
ITSN2	0.968174
IWS1	0.36331654
JADE2	1.7320609
JAG1	1.9351201
JAG2	2.8565347
JAK2	1.4616842
JAK3	0.7628412
JAKMIP1	1.56843
JAM3	1.513782
JDP2	2.2791114
JMJD1C	1.1212344
JPH2	0.5077951
JUN	3.946128
JUNB	2.4162307
JUP	0.54481006
KANK2	0.4832816
KANK3	2.0495124
KANK4	1.9336028
KANSL1	1.1378367
KANSL3	0.37660933
KAT5	0.40405607
KAT6A	0.95274353
KAT6B	1.078944
KAT7	0.37737656
KATNA1	0.92611265
KATNAL1	1.2249413
KATNB1	0.47536898
KATNBL1	0.74683666
KAZALD1	0.7446537
KAZN	2.7701306
KBTBD11	3.3544207
KBTBD2	0.25462437
KBTBD8	0.6672561
KCNA1	1.4722345

KCNA3	3.0208893
KCND3	1.8425786
KCNE2	7.392354
KCNE3	2.37567
KCNE4	3.6224718
KCNH3	2.0421262
KCNIP3	1.0760417
KCNIP4	0.96552134
KCNJ13	4.887303
KCNJ15	4.2314787
KCNJ2	2.229875
KCNJ5	3.4548328
KCNJ8	0.3001361
KCNK1	4.5039344
KCNK12	1.1141665
KCNK2	1.2713318
KCNK3	2.8179498
KCNK6	0.813961
KCNMB1	2.407992
KCNMB2	1.1808734
KCNMB4	3.9702058
KCNN3	1.895021
KCNN4	0.6116309
KCNQ1	1.6632195
KCNQ1OT1	1.4958897
KCNRG	4.552938
KCNS3	5.440364
KCNT1	0.42067385
KCTD1	3.2612686
KCTD10	2.5773058
KCTD11	2.5630379
KCTD12	0.7235093
KCTD12B	0.6434946
KCTD21	0.5341182
KCTD4	1.1309521
KCTD5	1.7174487
KCTD8	1.895874
KCTD9	0.33886242
KDELC2	2.1199446
KDELR3	0.97471094
KDM1A	0.31899548
KDM3A	1.7641625
KDM3B	0.68449116
KDM4C	0.7680521
KDM5A	1.0563717

KDM5B	0.89783096
KDM6A	1.0430803
KDM6B	1.7630038
KDM7A	1.4841866
KEL	0.8766248
KHDC3	3.534676
KIDINS220	0.92126036
KIF13A	1.0508432
KIF15	0.8046808
KIF16B	0.6235571
KIF1A	4.1909933
KIF21B	0.72986984
KIF27	2.4595723
KIF2A	0.5511522
KIF3A	1.209094
KIF3B	1.1382291
KIF5A	0.64087796
KIF5B	1.3063867
KIF5C	1.239481
KIF6	2.0184553
KIF9	1.2330945
KIFAP3	1.0510526
KIN	0.3927312
KIRREL	1.8370581
KISS1R	1.7186437
KIT	2.1481447
KITL	7.216173
KIZ	0.42952394
KLC1	0.78367424
KLC2	0.5794897
KLC3	1.2968693
KLF10	0.15906191
KLF11	1.8607268
KLF13	0.6440344
KLF2	1.9909196
KLF3	1.2532549
KLF4	3.7407932
KLF5	6.414487
KLF6	2.7859955
KLF7	2.792388
KLF8	2.0176868
KLF9	1.0689106
KLHDC1	2.4452078
KLHDC9	1.9415059
KLHL1	1.5640883

KLHL11	0.60479593
KLHL18	0.4012041
KLHL2	0.73831797
KLHL24	1.6302118
KLHL28	1.4645796
KLHL29	2.7595236
KLHL30	0.3884976
KLHL32	1.4601634
KLHL35	0.5168793
KLHL38	2.1152208
KLHL5	1.173521
KLHL6	2.8274517
KLHL7	0.23306131
KLK1	1.5032029
KLK11	3.794918
KLK13	0.47262788
KLK1B24	0.6204078
KLK1B26	1.6284273
KLK1B27	2.7984846
KLK1B3	0.25290608
KLK1B5	1.733767
KLK4	1.3884919
KLK8	2.562583
KLK9	1.1403916
KLRA1	1.9025726
KLRA12	1.1910627
KLRA15	2.5718102
KLRA16	1.6039329
KLRA17	2.7114005
KLRA2	2.8712916
KLRA22	1.7732506
KLRA23	1.8522062
KLRA4	2.531815
KLRA6	0.21171474
KLRA7	2.0129075
KLRA8	2.5459232
KLRA9	1.8056302
KLRB1	1.7949798
KLRB1A	2.1730437
KLRB1B	1.2368538
KLRB1C	1.5058172
KLRB1F	2.8176663
KLRC1	2.073619
KLRC3	0.8171768
KLRD1	1.6749182

KLRE1	2.1640835
KLRG1	2.5261602
KLRI2	1.9849589
KMT2A	3.0382931
KMT2C	0.44618416
KMT2D	0.774313
KMT2E	0.86087227
KNDC1	2.0842645
KPNA4	0.8602872
KRI1	1.1838503
KRIT1	0.7342739
KRR1	0.44310522
KRT15	2.5389013
KRT16	1.5073223
KRT19	3.2394032
KRT222	0.2191596
KRT23	0.44038773
KRT42	1.929111
KRT5	0.84601855
KRT7	4.821994
KRT78	1.0165606
KRT79	4.0864267
KRT81	0.96656775
KRT86	2.8965318
KRTAP10-10	0.5858793
KRTAP16-3	1.2479541
KRTAP17-1	2.377662
KRTAP19-4	1.8054523
KRTAP28-13	0.3779292
KRTAP5-1	1.0466046
KRTCAP3	2.1215248
KSR1	1.3225851
KTN1	3.3067627
L1CAM	1.2602446
L1TD1	0.42582583
L3MBTL3	1.1052461
LAD1	0.74136376
LAG3	0.33123922
LAIR1	0.4434414
LAMA2	2.66321
LAMA3	3.646573
LAMA4	2.652299
LAMA5	3.2739367
LAMB1	1.8184557
LAMB2	2.1300597

LAMB3	2.3862257
LAMC1	1.6540756
LAMC2	6.78693
LAMC3	1.844254
LAMP3	2.684619
LANCL3	0.8536458
LAPTM5	2.0228992
LARP4B	0.17357397
LARP6	3.1175945
LARP7	0.6293502
LAT	1.1295719
LAT2	1.42572
LATS2	2.4274015
LAYN	1.3394089
LBH	0.63837194
LBR	1.6146388
LBX1	1.075304
LBX2	0.37881708
LCA5	2.0161064
LCA5L	1.0109384
LCK	1.4364438
LCN2	0.97295475
LCN6	0.65153
LCOR	1.3004026
LCORL	0.4237194
LCP1	2.2180786
LCP2	1.6838231
LCTL	0.7641144
LDB2	2.649241
LDB3	3.1550593
LDHB	2.184616
LDLRAD1	2.7398367
LDLRAD4	1.0896194
LEF1	3.198625
LEFTY2	2.3932776
LEKR1	1.8732057
LEMD3	0.6894388
LEPREL1	1.3161399
LEPREL2	0.7128582
LEPREL4	1.1748486
LETM2	2.0731044
LGALS1	0.8211632
LGALS2	1.2091036
LGALS3	1.2467928
LGI2	1.8947039

LGI3	7.089317
LGI4	1.1334748
LGR6	1.2103992
LHB	2.260093
LHFP	2.4680572
LHFPL3	1.2365413
LHX3	0.76510954
LHX4	0.36520576
LILRA6	1.6099629
LILRB4	2.547937
LIMA1	1.2682719
LIMCH1	7.4917254
LIMD2	0.2151084
LIME1	0.8169098
LIMK1	0.79894066
LIMK2	0.44411016
LIMS1	1.8279357
LIN28A	0.18994045
LIN54	0.29143715
LIN7C	0.42973948
LIPE	0.2294178
LIPF	1.9088721
LIPG	5.0392323
LIPH	5.4951315
LIPO1	1.7558031
LITAF	0.5700588
LIX1	0.77913165
LIX1L	2.694047
LMCD1	2.5973792
LMLN	1.5390129
LMNB1	1.9399896
LMO1	3.2372715
LMO2	0.44089222
LMO4	1.188314
LMO7	3.81495
LMTK2	0.6495626
LMTK3	1.5419135
LNP	0.67546296
LNPEP	2.0707517
LOC1000417[illegible]	1.0192232
LOC1008624[illegible]	0.5022292
LOC1010558[illegible]	0.59152794
LOC1010558[illegible]	0.95436764
LOC1010562[illegible]	1.0602958
LOC1010562[illegible]	1.8016336

LOC1026332	1.2045884
LOC1026336	2.5577164
LOC1026337	2.9066315
LOC1026338	0.93101215
LOC1026344	0.93028736
LOC1026367	0.24344134
LOC1026377	0.85496235
LOC1026380	1.9873495
LOC1026391	1.2289195
LOC1026395	2.3880234
LOC1026416	0.4608655
LOC1026416	1.053505
LOC1026420	0.24947596
LOC1026422	0.7948618
LOC1026423	1.9577129
LOC1026423	1.9837625
LOC1026424	0.41558123
LOC1026424	1.6474764
LOC1026425	1.2262578
LOC1026426	0.57212114
LOC1026427	0.97961235
LOC1026427	0.25350285
LOC1026427	0.47943592
LOC1026427	0.6526885
LOC1026428	0.65651274
LOC1026430	0.73525214
LONRF3	1.2905045
LOX	7.2594247
LOXL1	2.5742674
LOXL3	0.6886246
LOXL4	0.641886
LPAR1	4.0328197
LPAR2	0.20749211
LPAR3	4.1419363
LPCAT1	3.2415519
LPCAT2	1.0083919
LPHN1	0.9505477
LPHN2	2.387741
LPHN3	2.6715794
LPXN	1.0354958
LRCH1	1.7269216
LRCH3	0.92743874
LRFN1	0.27606368
LRFN4	3.9244008
LRGUK	2.8223665

LRIF1	0.45239878
LRP12	0.4616537
LRP2	5.3152037
LRP4	1.3124523
LRRC1	2.4033136
LRRC10	4.0316706
LRRC10B	3.3525019
LRRC16A	2.7979698
LRRC17	1.8082769
LRRC2	1.3514566
LRRC23	5.899355
LRRC25	1.6704869
LRRC26	6.949569
LRRC27	0.28990984
LRRC32	0.5938778
LRRC34	3.5583463
LRRC36	5.7579713
LRRC38	0.93952394
LRRC4	1.0887403
LRRC43	3.5419388
LRRC49	3.1133153
LRRC4B	3.9895668
LRRC51	1.1075091
LRRC6	0.66508603
LRRC71	0.6706779
LRRC75A	1.7324023
LRRC75B	1.8823617
LRRC8A	1.4655724
LRRC8B	4.324447
LRRC8C	2.402442
LRRC8E	1.7635641
LRRCC1	0.76574564
LRRFIP1	1.1472769
LRRFIP2	0.31577682
LRRK1	1.0677242
LRRK2	4.2872334
LRRN1	0.80166435
LRRN3	0.5105958
LRRN4	1.187619
LRRN4CL	2.1026177
LRTM2	3.5538661
LSM12	0.51610184
LSM8	0.9333472
LSP1	2.5503745
LTA	0.83822846

LTB	2.2415051
LTB4R1	2.7472553
LTBP1	3.027143
LTBP2	3.2803607
LTBP3	2.7954183
LTBP4	2.11477
LTC4S	0.6676984
LTF	5.171104
LUC7L3	0.9687638
LURAP1	2.1072273
LUZP1	0.7181568
LXN	2.2967687
LY6A	2.539463
LY6C1	2.167529
LY6F	1.274909
LY6G5B	3.6130118
LY6G6C	2.4554596
LY6G6D	1.4238167
LY6I	0.95671463
LY75	1.1820896
LYL1	0.64259577
LYN	1.4088578
LYNX1	0.94067526
LYPD2	6.093366
LYPD5	1.5395637
LYPD6	0.60955286
LYPD6B	4.1433005
LYRM2	0.4036889
LYSMD2	1.9834156
LYSMD3	0.3225422
LYST	1.3442993
LYVE1	3.901462
LYZ1	2.1329737
LYZ2	2.353022
LZTFL1	0.29753876
LZTS1	0.9895005
LZTS2	0.6763096
LZTS3	1.3374062
M1AP	2.5373287
MAATS1	2.5404565
MAB21L3	0.63679695
MACC1	2.8332124
MACF1	2.033049
MAFA	0.62844086
MAFF	3.7616496

MAFK	1.1117139
MAGEA7-PS	0.9341521
MAGEE1	0.5216856
MAGEF1	0.82812357
MAGI1	0.41967154
MAGI2	0.99939513
MAGI3	2.0290456
MAGOHB	1.946548
MAK	1.0737445
MAK16	0.5614743
MAL	2.096684
MAL2	1.3619714
MALAT1	2.5928946
MALL	4.1645722
MALT1	2.5987668
MAMDC2	2.5390744
MAML1	0.73662853
MAML2	0.8341017
MAML3	2.032464
MAMSTR	2.6922798
MAN1C1	0.3571372
MAP10	2.2134805
MAP1A	1.1538303
MAP1B	3.113004
MAP1S	0.3840027
MAP2	2.7213407
MAP2K3OS	0.38001013
MAP3K1	0.57268906
MAP3K14	0.91422033
MAP3K15	0.8342874
MAP3K19	0.81885076
MAP3K3	1.0121245
MAP3K6	4.716194
MAP3K7	0.419446
MAP3K8	2.4953523
MAP3K9	2.779031
MAP4	1.8304853
MAP4K1	1.2381611
MAP4K3	0.24417543
MAP4K4	1.2343116
MAP4K5	0.31758404
MAP6	3.2177086
MAP6D1	0.29948902
MAP7D1	0.41919088
MAPK10	2.2444887

MAPK11	1.7025175
MAPK12	0.99682283
MAPK13	3.3856153
MAPK1IP1	0.6694603
MAPK6	0.39683628
MAPK7	1.02703
MAPK8IP1	0.61370754
MAPK8IP3	0.61066055
MAPKAPK2	1.0024791
MAPKAPK3	1.8800907
MAPKBP1	2.098767
MAPRE1	0.65672016
MAPRE2	1.4895239
MAPT	4.257713
MARCKS	2.2192392
MARCKSL1	2.5605035
MARF1	0.8599596
MARK1	1.1806178
MARK2	0.4046836
MARK3	0.67331266
MARK4	0.1662221
MARVELD3	2.03364
MAST2	0.572649
MAST4	2.4531026
MAT2A	0.60808086
MATN2	0.24612379
MATN4	4.2192864
MAX	0.619256
MB	6.212615
MB21D1	1.6475687
MBD1	1.0030723
MBD2	1.2128844
MBD4	0.3273058
MBD5	1.0293176
MBIP	1.5671463
MBNL1	0.4528165
MBNL2	1.1221452
MBNL3	2.6589284
MBOAT1	3.3578353
MBOAT2	1.112323
MBP	3.553761
MC5R	1.3569338
MCEMP1	4.8723845
MCF2L	3.4554229
MCL1	0.9923358

MCM4	0.57891226
MCM6	0.33178234
MCMBP	0.3601899
MCOLN2	1.1309543
MCOLN3	1.704102
MCPT4	4.011248
MCPT8	2.1984394
MCRS1	0.38274765
MCTP1	3.2960267
MDC1	0.5341592
MDFI	1.1161866
MDFIC	0.6010442
MDGA1	0.74203086
MDH1B	4.600059
MDM1	0.2159872
MDM4	2.0487895
MDN1	1.0689347
ME2	0.5339074
ME3	4.561827
MECP2	1.0845866
MED10	0.1575079
MED11	0.20028543
MED12	0.5579195
MED13	0.57798266
MED13L	2.868866
MED21	0.47574043
MED30	0.4917488
MED31	1.1120868
MED9OS	0.67578125
MEF2A	0.33007002
MEF2C	0.9999995
MEFV	3.3240447
MEG3	0.5976467
MEGF6	2.3248234
MEGF8	0.5839677
MEIG1	5.536485
MEOX1	4.429046
MEOX2	5.216289
MESDC1	1.7799995
MESP1	3.2586763
MESP2	0.40103316
METAP2	0.3211298
METRNL	0.4096241
METTL14	0.8232496
METTL21C	2.2874389

METTL24	3.8927982
METTL3	0.3557868
METTL6	0.38480902
MEX3B	1.0933692
MEX3C	0.82912636
MEX3D	0.5561385
MFAP1A	1.1575766
MFAP1B	1.4496918
MFAP2	2.9402008
MFAP3	0.33114195
MFAP3L	1.8329525
MFAP4	5.418289
MFAP5	6.6129875
MFGE8	1.3061953
MFNG	1.9178228
MFSD10	0.4268422
MFSD4	2.2122965
MFSD6	1.2514825
MFSD6L	3.6267014
MFSD7A	0.9974606
MFSD7B	1.0154023
MGA	1.2288322
MGAT3	3.8834736
MGAT4A	2.36334
MGAT5	0.37751245
MGEA5	1.9340959
MGL2	0.64706373
MGP	4.0965242
MIA	1.6569343
MIB1	0.3264351
MICAL1	2.2208242
MICAL2	1.1381721
MICALL1	0.4095583
MICU3	1.6276295
MID2	1.4670482
MIDN	0.77208185
MIER1	1.5050011
MILL2	2.2687569
MIOS	0.29043245
MIPOL1	0.8322365
MIR17HG	0.70702696
MIR22HG	0.43516684
MIS12	0.6359596
MIS18A	0.2678113
MIS18BP1	1.4172776

MISP	0.76609516
MITD1	0.57669926
MITF	0.79691124
MKI67	0.62298155
MKL1	1.4827318
MLANA	4.7466044
MLC1	7.9516664
MLF1	4.19892
MLIP	0.8714392
MLLT10	0.9423876
MLLT11	0.9480057
MLLT3	0.46940327
MLLT4	4.0910788
MLLT6	0.936646
MLPH	2.267969
MLXIP	1.6390061
MME	1.441711
MMGT2	0.67077255
MMP11	1.0625114
MMP12	0.5128579
MMP13	3.862911
MMP2	2.6573677
MMP23	0.6312432
MMP25	6.2627716
MMP3	5.170152
MMP8	6.775631
MMP9	5.527232
MMRN1	2.6175027
MMRN2	0.21124887
MNDA	2.293973
MNDAL	0.6969485
MNS1	4.725691
MNT	0.4954772
MOB1A	0.4760542
MOB1B	1.4131253
MOB3A	2.1897817
MOB3C	1.1476908
MOB4	0.83654356
MOK	2.8883715
MON1B	1.1362209
MON2	0.5593467
MORC1	1.1619575
MORC3	0.77084494
MORN3	1.1796458
MORN4	1.5184875

MORN5	4.872312
MOXD1	0.85724926
MPHOSPH10	0.5373931
MPHOSPH6	0.8840542
MPL	1.104034
MPP2	0.44794536
MPP3	0.38558364
MPP5	2.2873273
MPP7	2.0174394
MPPED2	0.98527074
MPRIP	0.7594032
MPZL1	1.2060118
MRC2	1.710154
MRGPRA2A	6.8340583
MRGPRA2B	6.7873273
MRGPRA3	1.7187591
MRGPRA5	2.1333573
MRGPRA6	6.0505686
MRGPRG	1.7311296
MROH1	0.6749208
MRPL20	0.7495947
MRPL3	0.3301649
MRPS6	1.0548043
MRVI1	0.33057094
MS4A1	2.22155
MS4A14	0.60033417
MS4A15	1.3114023
MS4A3	0.69318914
MS4A4A	5.279042
MS4A4B	2.6748447
MS4A4D	3.5706758
MS4A6B	3.8277013
MS4A6C	2.6697826
MS4A6D	2.9157062
MS4A7	1.1348748
MS4A8A	0.81924057
MSANTD1	1.4509664
MSANTD2	0.54508924
MSANTD3	1.5671015
MSI2	0.8875756
MSL2	1.4025679
MSL3L2	0.48988342
MSLN	2.9539828
MSN	2.2723393
MSRB3	1.049725

MSS51	0.9091015
MST1R	1.9292536
MSX1	2.1865675
MTA2	1.075274
MTAP7D3	1.6684155
MTBP	0.74357843
MTCL1	6.072977
MTDH	0.79285765
MTF1	0.34565783
MTF2	0.5814109
MTHFD1L	0.39752483
MTHFD2	2.6832519
MTIF2	1.5704899
MTMR12	1.2962341
MTMR14	0.19896555
MTMR3	1.023232
MTMR6	0.42999363
MTRR	0.32134795
MTSS1L	1.1876822
MTURN	2.0785894
MTUS2	0.9688325
MUC1	5.9063244
MUC13	1.7728405
MUC20	4.773932
MUC4	5.4192185
MUC5B	6.6204786
MUM1L1	3.6645303
MURC	0.5039387
MUSTN1	0.9640398
MVB12B	1.8976569
MVP	0.3667059
MX1	1.0187135
MX2	0.3543954
MXD1	4.2613864
MXD4	0.7998233
MXRA7	2.3298087
MXRA8	1.25494
MYADM	2.0795531
MYADML2	0.6420975
MYB	2.716485
MYBL2	0.37793112
MYBPC2	2.0559368
MYBPC3	3.9982586
MYBPH	0.9487817
MYBPHL	4.535016

MYC	1.8706264
MYCBP2	0.37085485
MYCBPAP	3.3432198
MYD88	1.5814781
MYEF2	1.8596954
MYH10	1.4429655
MYH11	3.4439893
MYH14	3.0934062
MYH3	0.8091724
MYH6	6.603222
MYH7	6.626493
MYH7B	1.8774543
MYH8	2.4255126
MYH9	1.371088
MYL1	0.7652254
MYL12A	1.2234845
MYL12B	0.41429043
MYL3	5.061219
MYL4	4.945979
MYL7	5.9221773
MYL9	2.735569
MYLK	1.0662827
MYLK2	1.1788244
MYLK3	0.7708194
MYO10	1.4957747
MYO18B	0.25478268
MYO1C	1.6892319
MYO1D	3.3153
MYO1F	1.357863
MYO1G	1.6615582
MYO1H	2.1075833
MYO5A	2.1968656
MYO5C	4.8006907
MYO9A	0.7268801
MYO9B	0.28106976
MYOC	0.38020682
MYOCD	2.4745328
MYOF	0.45005202
MYOM1	4.1140256
MYOM2	4.6841183
MYOM3	1.9221368
MYOZ2	5.6300063
MYRF	1.3911622
MYRIP	5.066405
MYSM1	0.47054386

MYZAP	3.2955089
N28178	1.8288777
N4BP1	2.2603755
N4BP2L2	1.4463882
N4BP3	2.0540838
NAA16	0.60913944
NAAA	1.9489369
NAALAD2	1.3238997
NAB1	0.32179022
NABP1	0.6086278
NACAD	0.71965504
NAF1	0.72881365
NAIP6	0.49379778
NAMPT	0.4328165
NANOS1	0.58263373
NAP1L1	0.68363523
NAP1L4	0.33208418
NAPB	0.4063053
NAPEPLD	1.3823404
NAPSA	4.4303517
NAT14	1.3175461
NAT8L	1.2796443
NAV1	1.375474
NBEA	2.924899
NBEAL1	1.1463008
NBEAL2	0.19729853
NBL1	4.4386435
NBN	1.4091806
NCAM1	0.5213573
NCAPH	0.14866924
NCBP2	0.6694808
NCCRP1	1.9504313
NCF1	1.8797379
NCF2	1.8744836
NCF4	2.4267182
NCK1	0.52710867
NCK2	0.86701965
NCKAP1L	0.8567767
NCKAP5	5.91718
NCOA6	0.7403512
NCOA7	2.7408335
NCOR2	0.36575365
NCR1	0.76925564
NCS1	1.2634296
NCSTN	0.22100306

ND6	1.8475738
NDC80	0.5674689
NDE1	0.6055155
NDEL1	0.47868347
NDN	0.92995787
NDNF	3.5046206
NDRG1	0.73220444
NDRG4	3.444247
NDUFA4L2	2.0285134
NEAT1	1.7034721
NEBL	3.463136
NECAP2	0.47714758
NEDD9	2.5792341
NEFH	0.35992932
NEK11	2.9022324
NEK5	4.3731847
NEK7	0.6067238
NEMF	0.6576333
NEO1	1.4486961
NES	3.788031
NEURL1A	2.9019675
NEURL3	2.2737594
NEURL4	0.33067703
NEUROG1	1.1240847
NEXN	2.3224788
NFAM1	0.46659946
NFAT5	2.5704718
NFATC1	1.5677791
NFATC2	0.8250365
NFATC2IP	0.50045156
NFATC3	0.40435958
NFATC4	2.0946054
NFE2	2.0824814
NFE2L1	0.35284805
NFE2L2	1.042223
NFE2L3	0.7022495
NFKB1	1.745542
NFKB2	1.7788348
NFKBIA	3.0278368
NFKBID	2.8423886
NFKBIE	2.292398
NFKBIZ	2.5130258
NFYA	0.97250223
NGFRAP1	1.5341415
NGP	4.4172816

NHLRC2	0.87213564
NHLRC4	2.74702
NHSL2	2.451182
NID1	0.65919685
NIN	1.8216214
NINJ2	0.5041647
NINL	1.4916933
NIPA1	1.3923793
NIPAL2	3.9086475
NIPAL3	1.4232512
NIPBL	1.089118
NKAIN4	2.3331418
NKAP	0.24252272
NKD1	2.953897
NKG7	1.4145546
NKTR	1.2679839
NKX2-1	7.1993346
NKX2-5	1.3668497
NLGN2	1.7063727
NLGN3	0.7203717
NLK	0.37855434
NLRC3	0.7614889
NLRC4	2.236875
NLRC5	1.281138
NLRP1A	0.43206072
NLRP3	4.5880475
NLRP5	0.6938734
NMB	1.4088168
NME5	4.4060802
NME9	0.8943298
NMRK2	0.19220066
NMT2	0.6350701
NMUR2	4.180635
NNAT	3.7572732
NOC3L	0.8470211
NOD1	2.0135584
NOD2	4.366301
NOG	1.4598637
NOL10	0.71688914
NOL3	0.8594327
NOL8	0.43010807
NOP14	0.7518091
NOP56	0.39398003
NOP58	0.4222803
NOP9	0.9024265

NOS2	3.8704305
NOSTRIN	0.5956392
NOTCH1	1.5786929
NOTCH2	0.93442154
NOTCH3	2.7347002
NOTCH4	2.2235045
NOV	3.1622088
NOVA2	0.8180399
NOX1	0.7613549
NOX4	5.518519
NOXO1	0.28065538
NOXRED1	1.3210318
NPAS4	1.4608498
NPC2	1.3446288
NPDC1	1.6960683
NPEPPS	1.2419643
NPHP1	1.7875302
NPHP4	2.1166608
NPHS2	2.776853
NPM1	0.3010702
NPM2	1.4957957
NPNT	5.289719
NPPA	4.8660655
NPPB	0.28775215
NPPC	1.643311
NPR2	0.8899765
NPR3	1.94383
NPW	7.6772146
NPY1R	2.6269662
NR2C2	1.4245944
NR2F1	0.5382633
NR3C1	0.22054148
NR4A1	3.3831625
NR4A2	2.5885057
NRADD	0.726841
NRARP	2.7685735
NRAS	1.3413324
NRCAM	6.2047634
NRD1	1.6473479
NREP	0.41046572
NRF1	0.4682455
NRG1	0.28251314
NRG2	0.27367568
NRGN	1.3554978
NRIP1	1.4608974

NRIP3	1.8340261
NRN1L	1.0872507
NRP1	0.20027208
NRP2	1.1049829
NRROS	0.83049107
NRSN1	0.49213123
NSD1	0.44792414
NSF	1.6081333
NSG1	3.4750638
NSG2	1.6380424
NT5C3B	0.37042093
NTF5	0.87815
NTN1	1.5975051
NTN4	1.0407491
NTNG2	3.9659529
NTRK2	0.9203265
NTRK3	1.0411913
NTSR2	2.3376143
NUAK1	0.7345817
NUAK2	1.3525648
NUB1	0.705564
NUCB2	1.9441814
NUDT10	1.9590752
NUDT11	2.247596
NUFIP2	0.4143381
NUMA1	0.8158417
NUMBL	1.9386125
NUP107	0.3959775
NUP153	1.1630969
NUP160	0.6064725
NUP188	0.5763273
NUP205	0.17753887
NUP210L	0.75951743
NUP35	0.18098688
NUP62	0.27733183
NUP98	2.1039376
NUPR1	4.371491
NUPR1L	0.8407085
NVL	0.6673789
NWD2	2.735931
NXF1	0.65061855
NXF2	1.8888109
NXNL2	1.9584682
NXPE3	2.2154837
NXPE4	0.46760488

NXPE5	0.75815606
NXPH3	3.9516988
NYX	2.7276657
OAS1A	0.47075605
OAS1F	0.838984
OAS2	1.7315388
OAS3	1.5998931
OASL1	0.39308643
OASL2	2.3535419
OAZ2	1.4297857
OBFC1	1.2155724
OBSL1	3.716555
OCRL	0.33086967
ODC1	0.30350924
ODF2	0.2975316
ODF2L	0.64601135
OFD1	0.3799076
OGFRL1	0.6036954
OGN	6.7019854
OGT	0.39947462
OLFM1	1.9592853
OLFM4	4.464148
OLFML2B	2.0384512
OLFML3	1.6700912
OLFR1043	0.32521844
OLFR1131	0.5011687
OLFR1134	1.010102
OLFR1180	0.9797354
OLFR1372-PS	1.992856
OLFR1383	0.32044172
OLFR1384	1.9949269
OLFR1396	1.5284421
OLFR1443	0.32926202
OLFR1505	0.9547272
OLFR213	0.7855549
OLFR224	1.0992804
OLFR259	0.77982736
OLFR414	0.77784896
OLFR482	1.9098959
OLFR524	1.1190786
OLFR544	1.4722881
OLFR56	1.6258638
OLFR576	1.6298895
OLFR635	1.0967946
OLFR96	1.4517386

OLR1	4.008704
OMD	3.712666
OPHN1	0.8095417
OPN3	1.1382506
OPRM1	0.20243359
OPTC	1.7568636
ORAI2	2.2417154
ORC2	0.24292254
ORC4	2.3324523
OSBPL10	2.3703241
OSBPL3	1.9138184
OSBPL5	2.319488
OSBPL6	3.6581297
OSBPL7	0.412333
OSBPL9	2.5010624
OSCP1	3.1113818
OSGIN2	1.7476091
OSM	3.7199185
OSMR	3.7808084
OSR1	4.820969
OSTF1	0.57233
OTOP1	1.0814853
OTUD1	1.5152619
OTUD4	0.24643946
OTUD5	0.7176328
OTUD7B	1.2716079
OVGP1	2.408969
OVOL1	1.0008016
OXCT1	1.150547
OXSR1	0.39330292
P2RX1	1.3059397
P2RX5	4.023002
P2RY10	2.6263905
P2RY13	0.17251873
P2RY14	0.31927872
P2RY2	1.2356791
P4HA1	2.229239
P4HA2	2.9367838
P4HTM	1.5086179
PABPC1	0.34599304
PABPC1L	3.6499577
PACRG	3.3890166
PACS1	1.3818741
PACS2	0.6914878
PACSIN2	0.47724438

PADI2	1.5424223
PADI3	0.61487675
PADI4	2.2162018
PAFAH1B1	0.2695551
PAFAH1B2	0.717278
PAG1	3.4056895
PAK2	0.6441016
PAK3	0.51898193
PAK6	0.8823774
PALD1	0.9185648
PALM2	0.49673247
PAM	2.28621
PAMR1	0.4636116
PAN3	0.92498064
PANCT2	1.430857
PANK2	0.31250525
PANK3	1.0829425
PANX1	0.3776121
PAPOLG	1.0501223
PAPPA	3.236894
PAPPA2	2.2459362
PAQR5	1.9985301
PAQR6	2.3674858
PAQR8	2.9280446
PARD3B	1.3782523
PARD6B	3.5212183
PARD6G	3.1331563
PARG	0.39103127
PARM1	4.263045
PARP10	0.27660227
PARP11	0.95346165
PARP14	1.4048905
PARP2	0.42351484
PARP3	1.3073454
PARP4	1.3903108
PARP8	2.5413728
PARVB	1.9719481
PATZ1	1.198446
PAWR	1.5297818
PAXBP1	0.29430056
PAXIP1	0.95738673
PBRM1	0.6323719
PBX1	1.3151822
PBX3	1.5886168
PBXIP1	1.3926497

PCBP3	2.00597
PCDH17	3.1565552
PCDH18	2.7294722
PCDH19	3.7453022
PCDH20	0.6375294
PCDH7	0.7820611
PCDHAC2	2.0628588
PCDHB10	3.2131028
PCDHB14	2.7105277
PCDHB15	1.0717106
PCDHB16	3.406302
PCDHB17	2.3853178
PCDHB18	3.0100896
PCDHB20	3.977744
PCDHB21	3.4481802
PCDHB22	1.9643683
PCDHB3	2.284895
PCDHB4	1.7901449
PCDHB6	2.712141
PCDHB7	3.1411982
PCDHB8	0.9613383
PCDHB9	1.5503395
PCDHGA1	2.3278372
PCDHGA10	2.9005363
PCDHGA5	1.2933815
PCDHGA9	2.1564279
PCDHGB1	0.7848036
PCF11	1.8330758
PCGF3	0.95532155
PCGF5	1.4183192
PCIF1	0.84942293
PCK2	0.4853809
PCM1	0.9379349
PCNT	1.8330612
PCNXL2	1.4794924
PCNXL4	0.89044666
PCOLCE	1.5209002
PCOLCE2	3.73319
PCP4	0.82057023
PCSK2	1.1867318
PCSK5	0.73071575
PCYOX1L	1.0785043
PCYT1A	0.51557827
PDCD1	1.7823114
PDCD10	0.3882451

PDCD1LG2	0.5945561
PDCL3	1.926733
PDE1B	1.1228118
PDE3A	1.7918513
PDE4A	0.37732172
PDE4B	3.4399767
PDE4D	1.7204263
PDE5A	1.7013493
PDE6D	0.63223076
PDE7A	2.1178975
PDE7B	1.5045433
PDE8B	2.6594229
PDGFA	1.8793483
PDGFB	3.2462788
PDGFD	1.2010536
PDGFRA	4.1920557
PDGFRB	0.74780893
PDGFRL	2.9229498
PDIA2	2.310046
PDK3	1.8559213
PDK4	1.1107583
PDLIM2	3.3959522
PDLIM3	2.3993907
PDLIM5	0.60286474
PDLIM7	1.7038956
PDP1	1.3537116
PDPN	6.189088
PDS5A	0.5322161
PDS5B	0.34888077
PDZD2	3.0835142
PDZD3	1.1017413
PDZD4	3.058579
PDZD8	0.7791605
PEA15A	0.60530996
PEAK1	0.5702081
PEAR1	0.54067516
PECAM1	2.449098
PEG13	2.0271173
PEG3	1.6350636
PEG3OS	1.0815387
PELI1	0.8444543
PENK	3.2349062
PER1	1.1810756
PES1	0.9225154
PF4	2.363049

PFAS	0.62670445
PFKFB2	0.9305091
PFKFB3	4.647937
PFKFB4	2.667141
PFKP	1.6354642
PGAM2	3.2494907
PGAP1	1.1047966
PGBD5	1.374687
PGGT1B	1.0980749
PGLYRP1	7.428018
PGM1	0.8540292
PGM2L1	0.73498464
PGM5	1.5455256
PGS1	1.8786521
PHACTR1	4.085723
PHACTR2	2.1112223
PHC1	1.1160924
PHC2	0.9637618
PHC3	0.83367825
PHEX	2.6433165
PHF11A	0.8210974
PHF11B	1.3517666
PHF11D	1.4525595
PHF13	0.43110275
PHF2	0.6294098
PHF20	0.5432472
PHF20L1	1.2277989
PHF21A	0.3208065
PHF21B	1.0931275
PHF3	0.25987625
PHF6	0.7015662
PHF8	1.2437506
PHGDH	1.24646
PHGR1	0.7977381
PHIP	0.8864324
PHKA1	0.6703639
PHKG1	0.63903356
PHLDA3	0.27583075
PHLDB1	2.642806
PHLDB2	1.0475469
PHLPP2	0.6950011
PHRF1	0.36246824
PHTF1	0.811193
PHTF1OS	0.20549822
PHXR4	2.983305

PI15	2.4611444
PI16	8.346766
PI4K2B	0.91391706
PIAS1	0.508173
PIBF1	0.537555
PICALM	1.3611379
PIEZO1	1.8444242
PIEZO2	2.505785
PIFO	2.1277976
PIGA	0.45124102
PIGM	0.9370265
PIH1D2	1.3353748
PIK3AP1	1.2819929
PIK3C2A	1.3013973
PIK3CA	0.95622396
PIK3CD	0.7616577
PIK3CG	2.5315943
PIK3IP1	2.2592278
PIK3R3	1.6068354
PIK3R5	2.3862696
PIK3R6	2.3282652
PILRA	2.3315024
PILRB1	0.44961357
PILRB2	1.441792
PIM1	3.0797358
PIM2	0.7882576
PIM3	1.5977292
PINX1	0.89289284
PIP4K2A	1.6612244
PIP5K1A	0.46088886
PIP5K1C	0.61660147
PIRA11	1.0162716
PIRA2	0.76665163
PIRA6	0.9759383
PIRA7	0.97729063
PITPNA	0.3095131
PITPNM1	0.41482925
PITPNM3	3.9609694
PIWIL4	2.6267421
PJA2	0.9326596
PKD1	0.72782755
PKD1L3	1.2882128
PKD2	2.1330347
PKIA	1.8535254
PKIB	0.314106

PKM	1.5046358
PKN2	0.58896446
PKN3	0.526752
PKNOX1	0.32251787
PKNOX2	4.673096
PLA2G1B	4.036123
PLA2G2C	0.8875425
PLA2G4A	0.91808605
PLA2G4F	1.3484056
PLA2G7	1.1479201
PLA2R1	0.898088
PLAC8	1.6647692
PLAC9A	1.5055013
PLAGL1	2.1853428
PLAGL2	1.4529762
PLAT	5.358895
PLAU	2.0492165
PLAUR	4.8548865
PLB1	2.2200596
PLCB2	1.0603545
PLCB3	0.7555971
PLCB4	3.8407078
PLCD1	1.7157674
PLCD3	1.2063174
PLCG2	1.2643476
PLCH1	2.5079176
PLCH2	2.5023277
PLCL1	2.3710876
PLCXD3	1.0772865
PLD2	0.54021454
PLD5	1.0250869
PLEC	0.5728383
PLEK	3.3314872
PLEKHA1	2.2115684
PLEKHA2	1.3853307
PLEKHA3	0.4781313
PLEKHA4	1.5654621
PLEKHB1	1.1211567
PLEKHB2	1.2097001
PLEKHD1	3.629283
PLEKHF2	1.3501878
PLEKHG2	1.552958
PLEKHG4	2.0385528
PLEKHH1	2.7345796
PLEKHM2	0.6993151

PLEKHM3	0.9122524
PLEKHO1	0.7687993
PLEKHO2	1.1914482
PLET1	3.947237
PLIN1	3.2821515
PLIN4	2.1159244
PLK2	0.94822836
PLN	3.4264667
PLOD2	5.118845
PLP1	1.0820637
PLP2	4.209463
PLSCR1	1.2145939
PLSCR3	1.0031896
PLSCR4	0.8701365
PLVAP	0.8269911
PLXDC1	1.3611939
PLXDC2	3.703652
PLXNA1	0.5501957
PLXNA2	1.0773983
PLXNA4	1.5017557
PLXND1	0.6808324
PMAIP1	3.646494
PMEPA1	2.2956586
PML	0.40617752
PMP22	3.574061
PMS2	0.36508656
PNCK	2.0790796
PNISR	0.5204296
PNLIPRP1	1.7160797
PNMA2	1.5015645
PNMAL2	0.94994617
PNN	0.7329545
POC1A	1.1958561
POC1B	1.1681824
POC5	1.2923899
PODN	1.6419263
PODNL1	0.62186
PODXL	2.5576591
PODXL2	1.9690464
POF1B	2.5257053
POFUT2	0.24103928
POGK	2.5326936
POLB	0.2581606
POLD3	0.5366044
POLE2	0.2528484

POLH	2.0102959
POLK	0.41918015
POLR3E	1.321197
POLR3GL	0.48721552
PON2	0.52340126
POP4	0.3056326
POPDC2	2.560902
POPDC3	3.664267
PORCN	1.9158669
POSTN	2.8736067
POT1B	2.2452989
POU2AF1	0.94231176
POU2F1	0.31279325
POU2F2	0.8501334
POU3F1	0.3128407
POU5F2	0.61007357
PPAP2A	0.43539572
PPARD	0.689064
PPBP	2.395474
PPEF2	1.8867581
PPFIA1	0.4633212
PPFIBP1	0.41249895
PPHLN1	0.39822865
PPIC	2.888556
PPID	0.5017452
PPIG	0.7792001
PPIL4	0.3916192
PPIL6	2.0513082
PPIP5K1	1.4409628
PPL	0.9815264
PPM1D	0.61129665
PPM1E	1.5253127
PPM1F	0.6909046
PPM1H	0.64474773
PPM1J	1.9432898
PPM1M	1.1336226
PPM1N	0.91025925
PPP1R10	0.29050732
PPP1R12A	2.298273
PPP1R12B	0.9123707
PPP1R12C	0.40112638
PPP1R13B	0.64241505
PPP1R14A	1.768827
PPP1R14D	5.2655263
PPP1R15A	1.9195905

PPP1R16B	1.1879122
PPP1R18	1.9648027
PPP1R1A	0.822448
PPP1R2	1.6228967
PPP1R21	0.55310917
PPP1R26	3.8091652
PPP1R32	4.3182807
PPP1R36	0.84486985
PPP1R3A	1.7796235
PPP1R3D	3.1634622
PPP1R3F	1.4735596
PPP1R9A	2.1968741
PPP1R9B	1.7193718
PPP2CB	0.7925134
PPP2R2A	0.87358665
PPP2R2B	0.96282077
PPP2R2C	1.4879966
PPP2R5B	0.71786594
PPP3CA	0.71466446
PPP3CB	0.18939066
PPP3CC	0.9277544
PPP3R1	0.47904205
PPP4R1	0.59927464
PPP4R2	1.1681995
PPP6R3	0.5870409
PPRC1	0.3436501
PPT1	1.1672635
PPY	0.8150306
PQLC3	2.5119715
PRAF2	0.5888076
PRAM1	3.0134163
PRAMEF8	0.50713015
PRDM1	2.3694668
PRDM11	0.8763027
PRDM16	4.082652
PRDM2	1.2268438
PRDM4	0.8881955
PRDM5	3.6284547
PRDM6	3.7470105
PRDM8	2.6317441
PRELP	0.49030256
PREX1	0.48262978
PREX2	2.136293
PRF1	1.5534749
PRICKLE2	0.23962736

PRICKLE3	0.2508149
PRIM2	0.1768713
PRKAA1	0.7285137
PRKAB2	1.0894008
PRKAR1B	2.7083054
PRKAR2B	3.3863811
PRKCA	1.277164
PRKCB	0.91753864
PRKCD	1.9929748
PRKCDBP	3.871028
PRKCE	1.7003837
PRKCH	2.6892333
PRKCI	0.62621975
PRKCQ	1.2901917
PRKD1	4.313859
PRKD2	1.788888
PRKD3	0.42910242
PRKG1	4.5842724
PRKG2	2.7367797
PRKX	1.8347335
PRL2C1	3.8263526
PRM2	1.7070208
PRMT1	0.16884708
PRMT2	3.3532043
PRMT8	1.0053577
PRNP	0.17931986
PROK2	7.693137
PRPF18	0.33408403
PRPF3	0.7009959
PRPF31	0.31762886
PRPF38B	1.0612922
PRPF39	0.5905552
PRPF4	0.3029189
PRPF4B	0.55014944
PRPS2	0.46595
PRR11	0.43663454
PRR13	0.8510728
PRR15	0.9165797
PRR15L	6.025629
PRR18	4.5012083
PRR24	0.73805094
PRR5L	1.7691622
PRR7	3.565599
PRR9	1.5788505
PRRC2B	0.6069088

PRRC2C	2.0550933
PRRG1	1.5004306
PRRG3	6.6072206
PRRG4	0.7507026
PRRT1	0.8804915
PRRX1	1.494211
PRRX2	2.5782256
PRSS12	3.6842656
PRSS16	2.4946163
PRSS23	4.8718743
PRSS23OS	5.140292
PRSS34	2.957096
PRSS53	2.2925448
PRSS8	1.0526834
PRX	8.788921
PSAPL1	1.1039062
PSAT1	0.40969563
PSCA	1.1929209
PSD	1.2055554
PSD4	0.6300683
PSG16	0.716938
PSIP1	0.28275585
PSMC3IP	0.44608712
PSORS1C2	0.71396065
PSTPIP1	1.2061639
PSTPIP2	0.58362293
PTAFR	3.4636478
PTAR1	1.312592
PTBP3	1.0716581
PTGER1	1.4661734
PTGER2	0.4827478
PTGER3	1.5116742
PTGER4	2.7279282
PTGFR	1.1900725
PTGFRN	2.8422437
PTGIR	2.7682824
PTGIS	3.4263072
PTGS2	6.2398024
PTHLH	1.7333012
PTK2	0.57645607
PTK7	1.1999607
PTN	1.1693103
PTPN1	0.8138447
PTPN11	0.5402045
PTPN12	1.8772249

PTPN13	3.5737891
PTPN14	4.0528903
PTPN18	0.79622936
PTPN2	0.9052863
PTPN21	0.9295316
PTPN22	1.9923291
PTPN23	1.2410903
PTPN4	0.8299439
PTPN6	0.86703825
PTPN7	1.490644
PTPRC	2.0226564
PTPRCAP	0.9397001
PTPRE	2.3437748
PTPRG	1.8030443
PTPRK	0.8917608
PTPRM	1.0781255
PTPRO	2.0260015
PTPRS	1.3498111
PTPRU	3.068933
PTPRZ1	2.8659678
PTRF	2.5336637
PTX3	4.6716905
PUM2	0.6091533
PURA	0.26820135
PURB	0.9543309
PURG	0.22872734
PUS7L	0.20211864
PVR	3.2025685
PVRL3	0.9295912
PVRL4	1.2090182
PWWP2A	0.7063494
PWWP2B	1.2573013
PXDN	1.6234727
PXN	1.3634577
PYCARD	1.3426914
PYCR2	0.24477482
PYDC4	2.1701827
PYGM	2.364903
PYGO2	0.30900002
PYHIN1	2.6864548
PYY	1.0888717
QK	2.3446302
QSER1	0.3797059
QTRTD1	0.15965629
R74862	0.46934795

RAB10OS	0.5077014
RAB11FIP1	1.8877249
RAB11FIP5	1.564404
RAB12	0.69763327
RAB15	8.083715
RAB19	0.7276211
RAB20	0.6507535
RAB25	5.510413
RAB27A	3.93758
RAB27B	2.8734736
RAB28	0.30050325
RAB2B	0.38990688
RAB31	0.8471117
RAB33A	0.44228292
RAB33B	0.6525245
RAB34	0.1796751
RAB35	0.59938145
RAB37	0.43202543
RAB38	1.0428562
RAB3C	1.4463372
RAB44	5.034681
RAB6A	0.5017586
RAB6B	5.015742
RAB7	0.27082586
RAB8B	1.8024306
RABEP2	0.34148884
RABGAP1	1.2044878
RABGAP1L	1.6371298
RABGEF1	0.351964
RABL2	0.9423766
RAC2	1.3986683
RAC3	1.7256742
RACGAP1	0.38884306
RAD18	0.56302977
RAD21	0.3489251
RAD52	0.24236393
RADIL	3.511662
RAE1	0.22298717
RAI1	0.754375
RAI14	0.6215987
RALA	0.4776678
RALB	1.714674
RALBP1	0.7759185
RALGAPA1	2.2665281
RALGAPA2	0.8104501

RALGAPB	1.1197987
RALGDS	3.1212907
RAMP2	1.6983714
RAMP3	3.0251808
RANBP2	0.4002509
RANBP9	0.8536401
RAP1A	0.5527749
RAP1B	1.5412989
RAP1GAP	0.4596672
RAP1GAP2	0.5980146
RAP1GDS1	0.5368886
RAP2A	0.9421091
RAPGEF2	0.25174904
RAPGEF3	3.3019147
RAPGEF5	3.994835
RAPGEF6	1.4912391
RAPGEFL1	1.5846214
RARA	0.40782595
RARG	0.7367921
RARS	0.3964467
RASA1	0.36730003
RASA2	2.259202
RASA3	1.0838737
RASAL3	1.2331343
RASD1	3.1002188
RASD2	1.4568906
RASEF	4.0763326
RASGEF1A	8.817668
RASGEF1B	0.28223705
RASGEF1C	2.3803074
RASGRF1	0.6192608
RASGRP1	1.2764997
RASGRP3	1.118226
RASGRP4	2.1191669
RASIP1	4.5758495
RASL10A	1.6571286
RASL11A	2.673448
RASL12	5.654298
RASSF1	2.1357212
RASSF2	2.3389897
RASSF5	0.8511529
RASSF9	5.3295336
RAVER2	1.0841665
RB1	1.5032654
RB1CC1	0.5832615

RBAK	0.8861456
RBAKDN	0.45697093
RBBP6	0.33284712
RBBP8NL	2.679793
RBFOX1	1.9792335
RBL1	0.30606985
RBM12	0.33474207
RBM12B2	0.6510792
RBM15B	0.6147599
RBM18	0.704175
RBM24	4.5957565
RBM26	0.19499636
RBM27	1.3045025
RBM28	0.3517518
RBM38	0.4544587
RBM39	0.76227856
RBM41	1.473377
RBM43	0.32346678
RBM48	0.8409867
RBM5	0.9070673
RBMS1	1.676271
RBMS2	0.7363038
RBMS3	2.7997003
RBMX2	0.40076423
RBMXL1	0.6071787
RBP7	2.633438
RBPJ	1.6413164
RBPJL	5.026192
RBPMS	0.6417036
RC3H1	0.33118725
RC3H2	0.9743881
RCN1	1.288331
RCN2	0.59123325
RCN3	0.38546944
RCOR1	0.463943
RCSD1	1.8237047
RD3	1.6838887
RECK	2.866955
RECQL	1.2456975
REEP1	0.22692299
REEP5	1.2163625
REG3G	8.641827
RELA	0.9156637
RELB	2.6883588
RELL1	0.90778923

RELT	1.4378161
REP15	0.9839959
REPS1	0.5460911
RERE	0.8477428
RERG	0.8226299
RESP18	2.3930383
REST	1.8359437
RET	0.98024154
RETN	2.6835551
RETNLA	6.3735905
RETNLG	4.451732
REV3L	2.763111
RFTN1	1.1073055
RFTN2	2.943925
RFX2	1.5628126
RFX3	1.14523
RFX8	0.60885835
RGCC	8.427204
RGL2	0.27371788
RGMB	1.5645123
RGS1	2.4324455
RGS10	0.7248597
RGS11	3.139392
RGS12	0.5222111
RGS14	1.3015451
RGS17	3.2127638
RGS18	3.9789877
RGS19	1.4844856
RGS2	1.6964798
RGS22	1.810163
RGS3	0.7478938
RGS4	2.769014
RGS6	1.5748324
RGS9	4.5687084
RHBDF1	0.822361
RHBDF2	1.2134175
RHBDL3	4.051608
RHD	1.7701504
RHEBL1	0.69332314
RHOB	3.082768
RHOC	2.7901978
RHOF	2.1778812
RHOG	0.60360146
RHOJ	3.4088736
RHOQ	0.94138193

RHOV	1.6861305
RIBC2	0.3846414
RIC8	0.6329117
RICTOR	1.1012893
RIF1	0.6498151
RIIAD1	2.6448655
RILPL1	1.3691087
RIMKLA	0.96198726
RIN2	2.867825
RIN3	3.6291313
RINL	1.2026262
RIOK1	0.7687688
RIPK1	0.45142078
RIPK2	2.995835
RIPK3	0.8183842
RIPK4	0.5080652
RIPPLY3	0.64932775
RLF	0.6856632
RLIM	2.3361712
RMRP	1.1029024
RN18S	5.994026
RNASE10	0.72091913
RNASE2A	3.9018526
RNASE6	0.4640994
RNASEL	2.2504845
RND1	1.8884091
RND3	0.29493713
RNF111	0.36491632
RNF114	0.49373055
RNF122	0.6991093
RNF144A	2.230914
RNF146	0.7450037
RNF150	2.358594
RNF157	0.6258185
RNF19A	0.4511633
RNF19B	1.4693184
RNF20	0.61532927
RNF207	3.208083
RNF208	2.7764513
RNF213	0.7813945
RNF219	0.71993876
RNF32	1.4613957
RNF34	0.8365464
RNF38	1.5370321
RNF39	1.0906582

RNF41	0.22832203
RNF44	0.63478994
RNF6	0.24786282
RNFT2	1.0436623
RNGTT	0.5746689
RNLS	0.58798504
RNMT	0.8942399
RNPC3	0.4590621
RNU1B6	2.6546693
RNU2-10	0.7242198
ROBO1	1.4444027
ROBO2	1.4847262
ROBO4	2.3280516
ROCK1	1.1575179
ROCK2	2.554903
ROR2	3.694396
RP2H	1.0705342
RPGR	1.1168971
RPGRIP1	2.538437
RPGRIP1L	1.1139598
RPH3AL	0.8428488
RPIA	0.9474001
RPL29	0.33438778
RPL39L	0.71271324
RPL3L	1.1517112
RPRD1A	0.67224836
RPRD2	1.1636233
RPRM	4.5289054
RPRML	0.6624832
RPS15A-PS6	1.4411702
RPS19	0.52801037
RPS6KA2	2.1252697
RPS6KA3	2.0062404
RPS6KA4	0.18949413
RPS6KA5	0.5891967
RPS6KA6	0.3327148
RPS6KL1	0.92948294
RPTN	0.7708168
RPTOROS	0.70230436
RRAD	2.5686693
RRAGB	1.0447319
RRAGD	1.6462576
RRAS2	0.42335367
RRM2B	0.47084785
RRP12	0.46013832

RRP1B	1.8980103
RS1	0.66069794
RS5-8S1	2.036893
RSAD2	1.9452262
RSBN1	0.7731142
RSBN1L	0.94318485
RSF1	0.48096514
RSL1	0.8589859
RSPH1	4.190524
RSPH10B	1.1468444
RSPH3A	3.1297464
RSPH4A	0.89225197
RSPH9	3.581729
RSPO1	1.9059086
RSRC2	0.6375909
RTDR1	2.909535
RTKN2	6.9270554
RTN1	1.2774911
RTN2	0.61907864
RTP4	1.1063418
RTTN	0.88264656
RUFY2	0.46422672
RUFY4	1.3684607
RUNDC3B	1.0497591
RUNX1	4.121807
RUNX1T1	3.965445
RUNX2OS1	2.3691885
RUNX3	1.1292484
RUSC1	0.36011457
RYBP	1.35045
RYR1	0.849123
RYR2	2.245209
RYR3	0.6815572
S100A11	2.2135425
S100A14	4.1126237
S100A2	1.0858102
S100A3	0.65657973
S100A4	2.9955673
S100A6	4.4279766
S100A8	4.8721094
S100A9	2.5442257
S100B	2.2110102
S100G	3.3281837
S100PBP	0.31089115
S1PR1	2.113883

S1PR3	3.504467
S1PR4	2.4199169
SAA3	3.116706
SACS	0.67246175
SALL2	0.49350762
SAMD12	2.3403459
SAMD14	1.5067024
SAMD3	1.0090435
SAMD4	3.890999
SAMD4B	1.0625854
SAMD5	1.3446832
SAMD8	0.4388821
SAMD9L	2.1551704
SAMHD1	1.1291165
SAMSN1	4.6885266
SAP130	0.18659782
SAP25	0.36186743
SAP30	1.5244055
SAPCD1	1.4403796
SAPCD2	0.25102305
SASH1	1.0620275
SASH3	1.3089461
SASS6	1.0781488
SATB1	2.257001
SAV1	2.5499244
SBF2	0.68685675
SBK3	5.4837966
SBNO2	1.7495742
SBSN	2.3917356
SCAF8	0.34544563
SCAPER	0.7178383
SCARF2	2.2671485
SCCPDH	0.9401059
SCD2	2.2596197
SCD4	2.1085014
SCEL	4.856464
SCG3	0.8859656
SCG5	2.3021457
SCGB1A1	11.278034
SCGB1C1	5.7466073
SCGB3A1	9.760004
SCGB3A2	8.115032
SCHIP1	3.6133676
SCIN	2.4945078
SCML2	0.519912

SCML4	2.0343893
SCN10A	1.153842
SCN2B	1.3465753
SCN3A	1.9476409
SCN3B	3.287846
SCN7A	4.545292
SCNN1A	3.8388033
SCNN1B	5.333286
SCOC	0.45987988
SCRG1	5.0145116
SCRN1	4.6063757
SCT	0.60837317
SCUBE1	2.3092604
SCUBE2	6.321313
SCUBE3	1.4607091
SCX	3.1711555
SDAD1	0.98068905
SDCBP	1.4473896
SDCBP2	2.5887423
SDCCAG8	0.52273655
SDE2	0.1895504
SDK1	0.3478601
SDPR	0.9781523
SEC1	2.7899768
SEC14L1	2.0143204
SEC14L3	6.4347615
SEC24B	0.9128008
SEC61A2	0.63547015
SECISBP2	0.77293444
SECISBP2L	1.8718216
SELE	5.229238
SELL	2.823979
SELM	0.6380482
SELP	5.174917
SELPLG	1.6034193
SEMA3A	3.7858646
SEMA3B	5.3286905
SEMA3C	5.265099
SEMA3E	4.0690312
SEMA3F	2.4605832
SEMA3G	6.9583907
SEMA4D	2.747408
SEMA4F	1.8739321
SEMA5A	5.683233
SEMA6A	0.30304766

SEMA6B	0.28432512
SEMA7A	8.603933
SENP2	0.663332
SENP6	2.3958895
SENP7	1.374136
SENP8	1.112185
SEPN1	0.29181194
SERINC3	0.8998642
SERINC4	2.0785265
SERP2	1.7101767
SERPINA3B	1.3147435
SERPINA3F	2.9694915
SERPINA3G	1.0075922
SERPINA3I	2.4233274
SERPINA9	3.0089457
SERPINB10	3.7508671
SERPINB1A	3.0940685
SERPINB1B	0.896626
SERPINB2	3.6908996
SERPINB3C	1.0569899
SERPINB5	0.37563896
SERPINB6A	0.61052465
SERPINB6B	4.684094
SERPINB6C	0.5280466
SERPINB9	2.1950812
SERPINE1	7.346191
SERPINE2	3.0350637
SERPINH1	2.068562
SERPINI1	0.61506677
SERTAD1	1.1433415
SERTAD2	0.7374921
SERTAD4	0.3063755
SESTD1	2.7095318
SETBP1	3.5321012
SETD1B	0.47248077
SETD2	0.6604042
SETD5	1.7639184
SETD7	1.1421766
SETX	0.4062066
SF3B1	0.6564827
SF3B4	0.39947605
SFI1	2.1294851
SFMBT2	0.6668594
SFN	1.4701617
SFPQ	0.5394702

SFRP1	0.8909981
SFRP2	3.4973469
SFRP4	1.2860467
SFRP5	2.1014583
SFSWAP	0.9534812
SFTA2	7.6794744
SFTPA1	10.291235
SFTPB	10.449566
SFTPC	10.767405
SFTPD	7.6676183
SFXN3	2.4116082
SFXN4	0.8432245
SGCA	1.9852648
SGCB	1.4125876
SGCD	0.59060335
SGCE	1.5265884
SGCG	1.0736511
SGIP1	1.0275667
SGK1	1.0630302
SGMS1	2.574059
SGPP2	2.970663
SH2B2	1.4058237
SH2B3	0.60112
SH2D1A	2.0535975
SH2D1B1	2.515869
SH2D2A	1.3051696
SH2D3C	1.9605746
SH2D5	1.8186798
SH3BGR	4.035409
SH3BGRL3	1.3113227
SH3BP4	2.2589068
SH3D21	3.3096557
SH3GL3	3.5806847
SH3KBP1	2.7350807
SH3PXD2A	1.7180023
SH3PXD2B	3.0840063
SH3RF1	0.9242525
SH3RF3	0.9014709
SH3TC1	1.6924081
SH3TC2	0.6783757
SHANK3	3.3796449
SHC4	1.6179919
SHE	0.34222555
SHH	2.1643555
SHISA4	0.50420666

SHISA5	0.6823139
SHISA7	0.6874807
SHOC2	0.37496233
SHROOM3	1.4078419
SIAH1A	0.5807185
SIAH3	0.5064573
SIDT1	1.6576071
SIGLEC5	3.3283014
SIGLECE	0.39059305
SIGLECG	1.0042188
SIK1	2.236465
SIK2	0.30270863
SIKE1	0.9682026
SIM2	0.31435776
SIMC1	0.39068604
SIPA1	0.7578969
SIPA1L2	1.767786
SIPA1L3	1.8745718
SIRPB1A	2.350892
SIRPB1B	2.261434
SIRT1	0.8584819
SIT1	0.94146657
SIX1	3.4754803
SIX4	3.9160993
SKA1	0.43206
SKAP1	1.4283557
SKAP2	1.2855291
SKIL	1.7539577
SKIV2L2	0.716321
SKOR1	1.3932948
SLA	2.5568423
SLA2	0.20787954
SLAIN1	2.2614772
SLAMF1	3.2089188
SLAMF6	1.162853
SLAMF7	1.3540392
SLAMF9	1.2549982
SLC10A3	0.49882793
SLC10A6	4.9036107
SLC10A7	2.302988
SLC11A2	0.48357582
SLC12A2	1.0782948
SLC12A4	0.87190723
SLC12A6	1.3051624
SLC13A2	1.3090076

SLC13A4	3.8126144
SLC14A1	0.38628292
SLC15A2	3.4182367
SLC15A3	1.4797039
SLC16A13	0.8040185
SLC16A3	2.9540176
SLC16A6	0.35520267
SLC16A8	1.4285219
SLC16A9	1.946372
SLC18A2	0.40431404
SLC18B1	0.28739643
SLC19A3	1.2918141
SLC1A1	4.7322245
SLC1A3	2.8566718
SLC1A5	3.2892942
SLC22A13	1.0250802
SLC22A17	1.12813
SLC22A20	2.9489074
SLC23A2	1.4020858
SLC24A3	5.056499
SLC24A4	4.7740135
SLC25A24	1.9310565
SLC25A27	0.2149539
SLC25A35	1.8646858
SLC25A36	3.5106387
SLC25A4	1.9141269
SLC25A41	2.4141898
SLC25A43	0.97395086
SLC26A2	2.089046
SLC26A4	2.895932
SLC26A9	4.8153367
SLC27A3	1.6212811
SLC27A4	0.30905676
SLC27A6	2.137493
SLC28A2	1.2871194
SLC28A3	0.87808895
SLC29A4	1.6568952
SLC2A1	0.7181387
SLC2A10	1.5491135
SLC2A12	2.4109883
SLC2A13	1.2501864
SLC2A3	2.170292
SLC2A6	2.511568
SLC30A1	0.47689438
SLC30A4	0.52135324

SLC30A7	0.25591993
SLC31A2	0.35747433
SLC34A2	5.9132347
SLC35A4	0.22076511
SLC35E3	0.6768353
SLC35E4	2.6465893
SLC35F2	0.5999472
SLC35F3	1.3094671
SLC35G2	0.7959819
SLC35G3	0.7624111
SLC36A2	6.397305
SLC37A1	0.90890455
SLC38A1	2.602707
SLC38A2	0.5770955
SLC38A5	2.7222233
SLC38A9	0.19636583
SLC39A10	1.9282403
SLC39A3	0.8047209
SLC39A6	2.1376495
SLC39A8	2.1811838
SLC39A9	0.991848
SLC3A2	0.7178879
SLC41A1	2.2637005
SLC43A2	0.77104044
SLC43A3	5.589847
SLC44A2	1.8235126
SLC44A4	3.3945653
SLC47A2	1.6176035
SLC4A11	1.7323112
SLC4A3	1.708581
SLC4A5	3.087985
SLC4A8	1.5071914
SLC52A3	4.3685975
SLC5A1	1.7262006
SLC5A3	1.1473515
SLC5A9	1.2105019
SLC6A14	4.3443356
SLC6A2	2.9851398
SLC6A20A	1.2683372
SLC6A4	3.153694
SLC6A8	0.7728691
SLC7A1	3.6754375
SLC7A10	5.50683
SLC7A11	2.820745
SLC7A3	2.1306252

SLC7A5	0.6709299
SLC7A6	3.3314304
SLC8A1	1.0782819
SLC8A3	0.396235
SLC9A1	0.71413136
SLC9A3R2	1.070149
SLC9A4	2.3843162
SLC9A7	0.34965563
SLCO1A5	1.8753965
SLCO2A1	0.8942461
SLCO3A1	3.1290746
SLCO4A1	2.58356
SLCO4C1	4.3153877
SLFN1	3.7920814
SLFN10-PS	2.3947449
SLFN2	2.491487
SLFN3	4.720113
SLFN4	7.032175
SLFN5OS	0.5931275
SLFN8	2.6688747
SLIT2	2.9083347
SLIT3	3.6585374
SLITRK6	1.9050562
SLK	0.7932935
SLMO1	0.5312524
SLN	8.409151
SLPI	0.8210511
SLU7	0.6114373
SLURP1	4.045861
SLX	0.14913726
SLX4	0.9090066
SLX4IP	1.2619388
SMAD1	1.1026859
SMAD3	0.7149377
SMAD4	0.5806265
SMAD5	0.18517017
SMAD6	1.5518303
SMAD7	0.73829126
SMAP1	0.57699394
SMARCA1	1.6426728
SMARCA5	0.23914576
SMARCA5-PS	1.1400223
SMARCD3	0.91725874
SMARCE1	0.20830679
SMC3	0.58111954

SMC4	0.5426307
SMC5	0.42149878
SMCHD1	1.4079032
SMEK2	0.56330156
SMG1	0.7842927
SMIM15	0.59671783
SMIM3	3.4762158
SMIM5	4.666674
SMOX	2.4694905
SMPDL3B	2.4028668
SMPX	1.8465972
SMS	0.85316706
SMTN	1.1461139
SMTNL2	3.0245266
SMURF2	0.24290276
SNAI1	3.0365
SNAP23	0.77911663
SNAP25	0.28923988
SNAPC1	1.2628117
SNAPC3	1.4736881
SNAPIN	0.6045332
SNCA	0.37940216
SNCG	1.0048966
SNED1	0.76673794
SNHG1	0.72308826
SNHG11	1.1868823
SNHG18	1.0893307
SNHG5	1.0023823
SNN	1.9869962
SNORA74A	0.86627126
SNRK	0.6519532
SNRNP35	0.45508528
SNRNP48	0.18144655
SNRPC	0.16628885
SNRPD1	0.48669004
SNRPF	0.6835556
SNTB2	2.403522
SNTN	3.665868
SNX1	1.8527904
SNX10	0.7539172
SNX11	0.2727008
SNX13	0.8312998
SNX16	0.45484972
SNX17	0.49293375
SNX18	1.1359396

SNX20	2.7812905
SNX22	3.5930617
SNX25	1.7624836
SNX27	0.37457657
SNX29	1.3742037
SNX30	0.49314404
SOAT1	1.212894
SOBP	3.195225
SOCS1	1.656045
SOCS2	2.016519
SOCS3	3.4395528
SOCS4	0.82553387
SOCS5	1.4294734
SOCS7	0.9182911
SOD3	1.1646786
SOGA1	1.3390036
SON	0.73415375
SORBS3	0.6004801
SORCS2	2.5680022
SORL1	4.252915
SORT1	3.1840472
SOS2	0.60000134
SOWAHC	1.4723582
SOX10	0.83012843
SOX17	1.3893733
SOX18	1.2547894
SOX2	3.8172615
SOX2OT	0.65589666
SOX4	1.4164133
SOX7	3.7901816
SP100	1.3712807
SP140	0.8653307
SP2	2.2232747
SP3OS	0.8181925
SP4	0.89664364
SP6	1.0648999
SPA17	1.2987905
SPACA6	1.7675862
SPAG1	1.1514001
SPAG16	1.9436998
SPAG17	1.1673546
SPAG6	5.374623
SPAG9	0.4834051
SPARC	1.2402949
SPARCL1	5.5523257

SPATA1	0.74857664
SPATA13	1.1319308
SPATA17	1.0049207
SPATA18	3.111645
SPATA2	0.234303
SPATA31	0.6995306
SPATA31D1B	0.6125941
SPATA5	0.42021656
SPATA5L1	0.9305148
SPATA6	2.009529
SPATA7	0.37408686
SPATS2	0.3269539
SPATS2L	0.5370574
SPDEF	1.6280565
SPECC1	1.3890843
SPECC1L	1.8139496
SPEER4C	1.499176
SPEER5-PS1	1.4377599
SPEF1	1.7675982
SPEF2	2.1088166
SPEG	0.4597757
SPEM1	1.5827065
SPEN	0.3747964
SPHK1	7.3345294
SPI1	1.6285114
SPIB	2.4024901
SPICE1	1.0185385
SPIDR	1.1277518
SPIN4	1.5360177
SPINK2	2.324999
SPINK4	1.770184
SPINK5	2.337437
SPINT1	3.092164
SPIRE1	1.5199199
SPN	2.6427789
SPOCK2	6.4678493
SPON1	4.551349
SPOPL	0.8050003
SPPL3	0.39296865
SPRED1	1.5664315
SPRED2	1.6167502
SPRTN	0.43918943
SPRY1	2.2414947
SPRY2	1.8779514
SPRY4	0.5404625

SPRYD7	0.31255913
SPSB1	0.7869885
SPTA1	0.8843427
SPTAN1	0.8940997
SPTBN1	3.368956
SPTLC2	1.7074208
SPTLC3	1.2044702
SPTY2D1	0.37198687
SRCAP	1.041615
SRCIN1	2.9839365
SRF	0.52607775
SRFBP1	0.247262
SRGAP1	2.7759104
SRGAP3	0.46273446
SRGN	3.0422883
SRI	0.89446974
SRL	3.7552526
SRPK2	1.2687883
SRPK3	1.4237773
SRPX	4.950929
SRRM2	1.329279
SRRT	0.62632227
SRSF10	0.67602396
SRSF11	0.9128084
SRSF3	1.0289345
SRSF4	0.24276781
SRSF7	0.590538
SS18	0.34434748
SSB	1.3053427
SSBP2	0.21665859
SSH2	1.2935119
SSH3	0.5796304
SSPN	1.7931533
ST14	2.8108578
ST3GAL1	0.6521025
ST3GAL2	2.4500127
ST3GAL6	3.2517638
ST6GALNAC1	1.5341692
ST6GALNAC2	3.8219848
ST6GALNAC5	2.2075992
ST8SIA1	1.089792
ST8SIA4	1.9146438
ST8SIA6	1.9811893
STAC	0.37127233
STAG1	0.209826

STAG2	0.7252712
STAM	0.46688318
STAM2	1.0439949
STAMBPL1	0.6750097
STAP1	1.7338881
STAR	0.23302221
STARD13	0.6431508
STARD3	0.46450472
STARD3NL	0.62155867
STARD6	0.4254992
STARD8	0.58276796
STAT1	0.62836695
STAT3	0.8424425
STAT4	2.2619848
STAU2	0.27547956
STC2	0.5888736
STEAP1	3.8926423
STFA1	6.305588
STFA2	5.5144706
STFA2L1	6.0736046
STFA3	6.429322
STIL	0.66567326
STK10	1.053586
STK17B	2.1373224
STK19	0.16534996
STK24	0.4711361
STK3	0.7081084
STK32A	0.96453595
STK33	5.8739533
STK35	0.2652135
STK36	2.185643
STK39	1.2963347
STK4	1.0910583
STK40	0.27798176
STMN2	3.3237762
STMN4	1.3680801
STMND1	2.7360876
STON1	1.9879708
STRA6	2.78814
STRADA	0.62690926
STRIP2	2.5587559
STRN	1.273814
STRN3	0.63639116
STX11	3.636796
STX12	0.81426096

STX16	0.91084194
STX19	2.201543
STX3	1.2694983
STX6	1.4961057
STX7	1.0419564
STXBP1	2.2906318
STXBP3A	0.55166864
STXBP3B	1.4542279
STXBP5	0.69432306
STYX	1.2545757
STYXL1	2.788841
SUCO	1.451539
SUFU	0.7236111
SUGP2	0.3132496
SULF1	2.8177867
SULF2	0.16092539
SULT2B1	1.3995173
SUMO1	0.38395834
SUMO2	0.33246994
SUN1	0.5653391
SUPT20	0.29198575
SUSD2	1.0710418
SUSD3	0.5008149
SUSD4	5.040219
SUSD5	0.17503
SUV39H1	1.853116
SUV420H1	1.0027084
SVEP1	3.2529683
SVIL	1.4627357
SWAP70	1.3671279
SWT1	0.59916687
SYDE1	1.8119135
SYF2	1.3955865
SYK	2.0052514
SYN2	2.089811
SYN3	0.41163468
SYNC	2.182991
SYNCRIP	0.7604456
SYNE1	2.2783427
SYNE2	1.3978629
SYNGR1	2.5080562
SYNJ1	1.3173022
SYNM	3.7112541
SYNPO	2.1946392
SYNPO2	2.8434281

SYNRG	0.5071058
SYT11	2.1184201
SYT13	4.0347557
SYT15	0.68123865
SYT5	5.222079
SYTL2	2.0603137
SYTL3	1.9412942
T2	3.6358478
TAB2	0.34160948
TACC1	1.4061165
TACR2	0.3151393
TACSTD2	2.1968966
TAF1	1.7299528
TAF1C	0.30359173
TAF1D	0.66026115
TAF3	0.8108201
TAF5	0.5725572
TAF7	1.1247337
TAF7L	0.8712292
TAF8	0.88134766
TAF9B	2.3712404
TAGAP	2.1010413
TAGAP1	1.2462978
TAGLN	1.32198
TAGLN2	2.0207329
TAL1	2.0162988
TANC1	1.5543118
TANC2	1.969806
TANGO6	0.35024
TANK	1.7065587
TAOK1	1.3204751
TARDBP	0.21717358
TARM1	3.2839108
TAS2R131	1.5084417
TASP1	0.68871593
TAX1BP1	0.84463835
TAX1BP3	0.39791727
TBC1D1	0.40587473
TBC1D10B	0.91433096
TBC1D10C	1.0663757
TBC1D12	1.0343037
TBC1D16	1.4330401
TBC1D19	1.1404619
TBC1D2	2.113173
TBC1D20	1.374248

TBC1D22B	0.51779556
TBC1D23	0.8444538
TBC1D30	0.3190012
TBC1D4	0.92862654
TBC1D5	1.5746298
TBC1D8	2.2812598
TBC1D9	0.8266704
TBCK	0.78915477
TBK1	1.1977406
TBKBP1	0.7923789
TBPL1	0.7009449
TBX1	6.8795447
TBX2	2.013599
TBX21	1.846847
TBX22	1.4749739
TBX3	0.99094486
TBX4	7.7283225
TBX5	7.2882733
TBX6	0.5116956
TC2N	2.796216
TCAP	6.623627
TCEAL1	0.96842194
TCEAL3	1.401717
TCEAL6	3.684628
TCEAL7	1.8774867
TCEAL8	0.42838144
TCERG1	0.6921234
TCF12	0.22403145
TCF15	3.8338933
TCF21	2.3593345
TCF23	1.5814164
TCF4	2.3739147
TCF7	0.77908754
TCHH	3.5976286
TCHP	0.44760132
TCOF1	1.1587014
TCP10B	0.6226902
TCP10C	0.8977308
TCP11	3.1089172
TCP11L2	0.3223033
TCRG-V1	0.48603392
TCSTV3	2.367711
TCTE1	0.47061396
TCTEX1D2	0.9611621
TCTEX1D4	4.314404

TCTN2	1.2729464
TCTN3	1.4335473
TDGF1	0.8681078
TDRD3	0.80872726
TDRD5	0.6236086
TDRD7	0.32672596
TEAD1	2.3049235
TEAD2	1.2939343
TEAD3	1.1692545
TEAD4	4.1546583
TECPR1	1.2489219
TECRL	3.3458395
TEK	0.8382416
TEKT1	7.1225796
TEKT2	1.0418382
TEKT4	2.6494002
TEN1	1.3510718
TENC1	1.0329356
TERC	1.7224302
TERF2	0.5737262
TES	3.5759754
TESC	1.6465282
TESCL	1.7433822
TESK1	0.48755932
TESPA1	1.8412313
TET2	1.1763792
TET3	0.40145397
TEX11	1.1169448
TEX15	1.1365921
TEX26	3.2875571
TEX9	0.72509694
TFCP2L1	0.8971429
TFDP2	1.0157995
TFF1	1.1002433
TFF2	3.958486
TG	0.6631839
TGFB1	0.4125886
TGFB1I1	2.8725834
TGFB2	4.083831
TGFB3	2.2638679
TGFBI	0.3842411
TGFBR1	0.7626753
TGFBR2	1.3231001
TGFBR3	1.6885672
TGIF2	1.0661192

TGM2	1.3597536
THAP2	0.96812177
THBD	3.183227
THBS1	6.1650934
THBS3	4.5941486
THBS4	1.3332331
THEMIS	1.64146
THG1L	0.9859779
THOC2	0.8881273
THSD4	3.741777
THSD7A	1.309977
THY1	0.95714283
TIA1	0.76963735
TIAM1	1.4914551
TIAM2	2.592005
TICRR	0.5961714
TIE1	0.6710334
TIFA	0.4468813
TIFAB	0.9892218
TIGD3	0.5770979
TIGIT	4.183087
TIMELESS	2.0811896
TIMP1	3.9239988
TIMP2	2.7971787
TIMP3	5.1865025
TIMP4	2.9066992
TINAG	4.9603324
TINAGL1	2.1370573
TIPARP	2.5153494
TJAP1	0.4033804
TJP1	1.3591251
TJP2	1.5839572
TLE1	0.55129576
TLE3	1.4297347
TLE4	0.93212366
TLE6	0.2103653
TLK2	0.6537514
TLL1	0.936085
TLN1	0.89700556
TLR13	1.1381383
TLR2	2.857677
TLR4	1.987256
TLR6	1.3309684
TLR7	0.8350911
TLR8	1.8889089

TM4SF1	3.1538682
TM6SF1	1.3347793
TMA16	0.38185358
TMC4	3.0655327
TMC5	2.1552875
TMC7	0.6878958
TMCC2	2.9454098
TMCC3	1.3804555
TMCO3	1.116261
TMED8	1.616252
TMEFF1	3.2418761
TMEFF2	0.9023106
TMEM100	6.0009303
TMEM107	1.4015827
TMEM109	0.8487959
TMEM116	3.6499949
TMEM117	2.4096615
TMEM119	1.9957037
TMEM123	1.2420688
TMEM128	0.39649868
TMEM132A	0.83451414
TMEM132C	4.780963
TMEM132D	3.128102
TMEM136	0.66604614
TMEM139	1.0552607
TMEM140	0.87501717
TMEM154	2.6801205
TMEM159	2.919394
TMEM164	0.7859893
TMEM165	1.3047543
TMEM170B	0.7770395
TMEM171	1.114244
TMEM173	1.99653
TMEM178	2.5488095
TMEM181A	0.8977709
TMEM181B-I	3.9029617
TMEM184B	0.70857334
TMEM189	0.23938823
TMEM194B	0.71578836
TMEM198	0.9583051
TMEM2	2.8101468
TMEM200A	2.7612064
TMEM202	1.0691066
TMEM204	1.991899
TMEM212	6.5592294

TMEM213	5.1920514
TMEM229B	0.5556536
TMEM231	1.3066568
TMEM232	3.9666557
TMEM237	1.1373863
TMEM240	0.38195205
TMEM245	0.64052963
TMEM246	3.046719
TMEM252	4.1422462
TMEM255A	0.25035906
TMEM255B	0.68396926
TMEM38A	1.0797505
TMEM39A	0.8225622
TMEM40	2.612873
TMEM44	0.4653268
TMEM45A	0.33908653
TMEM47	0.36332273
TMEM50B	2.8451066
TMEM54	1.2471704
TMEM59L	2.5732894
TMEM61	1.4179821
TMEM63A	0.5037551
TMEM65	0.30730104
TMEM68	0.9303231
TMEM74BOS	0.9235828
TMEM87A	2.1364841
TMEM87B	0.5429635
TMEM89	0.42531753
TMEM95	0.40996718
TMEM9B	0.24839735
TMF1	0.8319936
TMOD1	5.094685
TMOD2	1.7122338
TMOD3	1.171731
TMOD4	0.41648293
TMPRSS13	4.6395564
TMPRSS4	1.2820382
TMSB10	1.6402373
TMSB4X	0.47602367
TMTC1	3.9093733
TMTC3	0.3207054
TMX3	1.3332357
TNF	4.154597
TNFAIP1	1.0993814
TNFAIP2	1.068944

TNFAIP3	4.4558773
TNFAIP6	1.5321624
TNFAIP8	1.8551064
TNFAIP8L3	1.0588682
TNFRSF10B	2.04219
TNFRSF11A	0.8791108
TNFRSF12A	1.0839624
TNFRSF13B	2.4932504
TNFRSF13C	2.3890948
TNFRSF14	0.19472146
TNFRSF18	1.8865471
TNFRSF1B	1.2936821
TNFRSF21	1.1518412
TNFRSF25	1.9870076
TNFRSF26	2.328202
TNFRSF4	1.2138343
TNFSF10	2.2230396
TNFSF13B	3.1026287
TNFSF14	2.7104964
TNFSF15	3.6604176
TNFSF8	0.5036111
TNFSF9	4.6423254
TNIK	1.6222076
TNIP1	3.1344032
TNIP2	1.4241738
TNIP3	2.5172367
TNK1	4.9126444
TNKS	1.0502257
TNKS1BP1	0.15113735
TNN	4.314877
TNNC1	0.9293566
TNNC2	1.2399313
TNNI2	2.3129902
TNNI3	3.7017941
TNNI3K	0.76037383
TNNT2	6.3853526
TNNT3	0.72787905
TNP2	0.7026701
TNRC18	0.3408203
TNRC6B	0.3567276
TNRC6C	0.5236125
TNS1	2.4283252
TNS3	0.7460346
TNXB	0.7817874
TOP1	0.2500658

TOP2A	0.7056675
TOP2B	0.3293004
TOPBP1	0.32283497
TOR3A	0.47759438
TOR4A	0.21342039
TOX2	2.2211022
TOX3	2.930881
TOX4	0.43641758
TPBG	2.7179816
TPD52	1.92029
TPM2	2.4467049
TPM3	1.2254968
TPM4	2.6549535
TPPP3	4.550817
TPR	1.1058807
TPSB2	0.93820524
TRA2A	2.0227232
TRABD2B	3.49826
TRAF1	4.6009507
TRAF2	0.50108004
TRAF3	0.8752098
TRAF3IP1	0.5405514
TRAF3IP2	0.97934675
TRAF3IP3	0.5061841
TRAF5	0.4096074
TRAF6	0.8738661
TRAF7	0.7204137
TRAK2	1.7001991
TRAPPC2	0.26014376
TRAPPC8	0.5165787
TRAT1	1.6643724
TRAV3N-3	1.6482549
TRAV6-3	0.75567293
TRAV9D-3	0.858042
TRCG1	1.0846834
TRDMT1	0.17084932
TRDN	0.4494996
TREM1	3.1872253
TREM2	1.9634843
TREM3	4.5605516
TREML1	2.3321967
TREML2	2.0864139
TRERF1	1.9201155
TREX1	2.1007166
TRIB1	0.95517683

TRIB2	3.3535886
TRIL	0.69431806
TRIM10	1.4577606
TRIM12A	0.6018591
TRIM12C	1.3988643
TRIM13	0.8088651
TRIM16	4.063008
TRIM21	0.8812394
TRIM29	3.3676856
TRIM3	1.6852713
TRIM30A	2.0373683
TRIM30B	4.1623464
TRIM30C	0.88706565
TRIM30D	2.3668098
TRIM33	1.0797668
TRIM34A	1.0801048
TRIM34B	1.1593795
TRIM35	1.6046567
TRIM36	1.6667936
TRIM39	0.60286474
TRIM43B	0.3726158
TRIM44	0.69955826
TRIM46	0.9923806
TRIM47	0.8116894
TRIM5	0.67601895
TRIM54	2.1698213
TRIM56	1.9326744
TRIM59	1.2265773
TRIM62	1.2918224
TRIM63	1.9941914
TRIM69	3.0926437
TRIM72	0.79001975
TRIM8	0.4846506
TRIO	0.86738014
TRIP10	1.6527162
TRIP11	0.9577851
TRIQK	0.72382736
TRMT44	0.8797991
TRNP1	3.5811481
TRO	1.122282
TROVE2	1.9711933
TRP53BP1	0.94295645
TRP53BP2	1.0526807
TRP53I11	1.362237
TRP53I13	0.27623796

TRP73	0.8924594
TRPC1	1.748606
TRPC3	0.30252934
TRPC6	4.68521
TRPM1	1.0173855
TRPM6	3.1549
TRPS1	3.091114
TRPV2	1.2859864
TRPV4	1.6566291
TRPV6	3.279622
TSC22D1	1.1755009
TSC22D3	1.0537877
TSGA10	1.9185057
TSHB	3.556679
TSHZ1	0.46165085
TSHZ3	1.9751458
TSLP	4.0877614
TSNAXIP1	2.5849864
TSPAN1	9.154874
TSPAN11	5.176068
TSPAN13	3.137405
TSPAN15	1.0520039
TSPAN17	0.64072704
TSPAN18	1.7828813
TSPAN2	4.9774375
TSPAN3	1.4006844
TSPAN32	0.93928814
TSPAN5	3.72505
TSPAN6	2.481872
TSPAN7	0.94634247
TSPAN8	6.632371
TSPO	0.3884368
TSPO2	0.6097374
TSPYL2	0.41340637
TSPYL3	1.5656252
TSPYL4	1.7996087
TSR1	0.3070612
TSR2	0.8313894
TSSK4	1.4161129
TTBK2	1.5038009
TTC12	2.6927972
TTC14	0.63288116
TTC16	1.2809095
TTC18	4.3728228
TTC21A	3.4908288

TTC25	1.3181846
TTC26	0.9521854
TTC28	1.0403533
TTC29	3.8515406
TTC34	4.1179833
TTC5	0.34629583
TTC8	0.2323196
TTC9	1.4178579
TTL	0.7255807
TTLL11	0.6486993
TTLL3	2.3683734
TTLL6	3.8124833
TTLL7	4.6337233
TTLL9	2.340083
TTN	2.420743
TTPAL	0.94708824
TUBA1A	3.9226155
TUBA1B	0.21810818
TUBA8	0.8262625
TUBAL3	0.5784235
TUBA-RS1	0.96415496
TUBB1	1.1339264
TUBB2B	3.081156
TUBB4A	0.53636837
TUBB6	1.0350103
TUBG2	1.3028185
TUBGCP3	0.71504354
TUBGCP5	1.0066595
TUFT1	0.65849686
TULP3	1.3250475
TULP4	0.96499586
TUSC3	0.3919735
TUSC5	3.0103045
TWF2	0.4632902
TWIST2	2.1706924
TXK	1.896122
TXLNB	4.3855815
TXNIP	1.4763746
TXNRD3	1.8239827
TYMS	0.8654821
TYRO3	2.0212946
TYROBP	0.7859931
TYW3	1.0610425
UACA	1.0288591
UBA2	0.34572268

UBA6	0.5305574
UBALD2	0.51521444
UBASH3A	0.38602543
UBASH3B	1.7325473
UBD	0.63221073
UBE2B	0.33326006
UBE2H	0.9398055
UBE2O	0.3642192
UBE2Q2	1.0127988
UBE2S	0.41666603
UBE3A	0.17265368
UBE4BOS1	1.2925336
UBN1	1.6578798
UBN2	0.53465176
UBP1	1.1296067
UBR1	0.27118254
UBTD2	2.2658105
UBXN10	4.1617227
UBXN11	2.2264094
UBXN2A	1.0501609
UBXN2B	0.29883814
UBXN7	0.8284116
UCHL1	1.4487491
UCK2	0.24317694
UCMA	0.29088068
UCP2	1.214561
UCP3	1.125082
UGCG	1.0527034
UGGT2	0.4547763
UGT2A1	3.808165
UGT8A	0.71158147
UIMC1	2.386856
ULBP1	0.8246021
ULK4	1.1661949
UNC13B	2.5554042
UNC13D	1.4943843
UNC45B	2.4433541
UNC5B	0.70601845
UNC5C	2.0210636
UNC5CL	0.9650426
UNC79	0.711365
UNK	0.43124628
UPF3A	0.44335985
UPF3B	0.32796526
UPK1A	0.6550002

UPK1B	2.6349816
UPK2	2.8604684
UPK3B	1.9670577
UPP1	4.2978454
URB2	1.9472208
USP11	1.4233341
USP13	1.5524483
USP16	0.6886034
USP25	0.56993484
USP31	0.9239106
USP32	0.85949135
USP33	0.17584562
USP34	1.8641596
USP36	0.48234987
USP37	1.6080527
USP42	0.49129105
USP43	1.683399
USP47	1.1002226
USP48	0.35771036
USP49	0.9913552
USP53	2.0854068
USP54	2.0894997
USP6NL	2.7465405
USP9X	0.9459748
USPL1	0.2663045
UTF1	2.9052346
UTP14A	1.9704123
UTP23	0.29088402
UTRN	2.1376715
VAMP2	0.31387138
VAMP3	0.87422085
VAMP5	1.2092242
VAPA	0.2886138
VASP	2.232502
VAT1L	0.5220885
VAV1	1.5279889
VAV3	0.76574135
VBP1	0.59302473
VCAM1	2.0893917
VCAN	2.2729168
VCL	1.7388096
VDR	1.9833951
VEGFA	1.8181062
VEPH1	3.5878756
VEZF1	1.2652435

VGF	3.5514724
VGLL3	1.314153
VGLL4	1.9530411
VIM	1.026227
VIPR2	4.343624
VIT	1.9828956
VLDLR	0.6517935
VMN1R168	0.6899779
VMN1R59	1.3101676
VMN1R87	1.807327
VMN2R11	1.7988095
VMN2R88	0.3501196
VOPP1	0.9522991
VPRBP	0.37190723
VPREB3	2.459958
VPS13A	1.1116624
VPS13B	0.23264647
VPS36	0.4123292
VPS37C	0.4210024
VPS51	0.72877645
VPS53	0.3719306
VPS54	0.61228895
VRK1	0.996953
VSIG10	0.89715576
VSIG2	4.258417
VSIG8	1.8672919
VSNL1	1.7313766
VSTM2A	0.25268006
VSTM4	2.3926547
VWA1	1.0229335
VWA3A	4.986
VWA5A	0.583302
VWF	3.0755148
WAC	0.43700886
WARS	1.8065801
WARS2	1.022752
WAS	0.93779945
WASF2	2.0184069
WASF3	0.33949614
WBP5	1.5863547
WDFY3	0.46106052
WDFY4	1.1748829
WDPCP	0.5197091
WDR1	0.99483776
WDR16	5.616757

WDR35	1.1166754
WDR37	0.44781303
WDR38	0.87470007
WDR44	1.1150658
WDR45B	0.71865606
WDR47	3.1246214
WDR52	1.6115658
WDR54	1.5742528
WDR5B	3.3996077
WDR60	0.5850072
WDR63	2.964829
WDR65	4.4120593
WDR66	3.9750838
WDR75	0.42498064
WDR78	3.52534
WDR92	1.5289135
WDR95	2.5499752
WDR96	3.284258
WDSUB1	0.72487354
WFDC10	2.4367394
WFDC17	1.368125
WFDC18	2.3359914
WFDC2	4.5282264
WFDC6A	1.5250266
WFDC6B	1.9957728
WFIKKN2	0.40281534
WHRN	4.1511965
WHSC1	0.84028864
WHSC1L1	1.0849214
WIF1	5.8204136
WIPF1	1.6158018
WIPF2	0.62664795
WISP2	0.83198786
WIZ	0.32597113
WLS	2.1195416
WNK1	1.4231825
WNT10B	2.5398145
WNT11	1.9176054
WNT16	0.98985386
WNT4	0.93807125
WNT6	2.563294
WNT7A	1.3782675
WNT9A	1.6846092
WRN	0.34397125
WSB1	1.5786757

WSCD2	1.7861841
WT1	2.648263
WTAP	0.1634016
WTIP	0.870605
WWC1	0.70802116
WWC2	1.4812317
WWTR1	1.8190026
XAF1	0.95240974
XCL1	1.8451285
XDH	1.1875677
XIRP1	0.59309864
XKRX	1.1998839
XLR3B	0.8977299
XLR4A	2.1447792
XLR4B	2.3407407
XPNPEP2	3.702274
XPO1	0.21911287
XPO4	0.4383943
XRN1	2.2260218
XRRA1	0.3663683
XXYLT1	1.3180768
XYLT1	0.5575268
YAP1	0.16205835
YBX2	0.40255666
YBX3	0.22683477
YDJC	0.8676472
YEATS4	4.115302
YIPF7	0.60659695
YTHDC1	0.6176038
YWHAH	0.7903938
YWHAZ	1.1863718
YY1	0.27055454
YY2	1.6679506
ZAK	2.565467
ZAP70	0.32262135
ZBED3	2.0112314
ZBED4	0.6542177
ZBED6	1.1515307
ZBTB10	1.6529374
ZBTB11	0.68724585
ZBTB12	0.35224438
ZBTB16	2.6423426
ZBTB2	0.7953658
ZBTB21	1.0601594
ZBTB24	0.7748885

ZBTB25	0.3715372
ZBTB26	1.8092062
ZBTB3	0.31648517
ZBTB32	2.9599538
ZBTB34	0.7417326
ZBTB37	0.8998451
ZBTB38	0.21157336
ZBTB4	0.7272029
ZBTB41	0.9709635
ZBTB46	1.4746633
ZBTB6	2.1079783
ZBTB7A	0.5175357
ZBTB8B	1.2163749
ZC2HC1A	2.3399882
ZC3H10	0.2305212
ZC3H11A	0.4365039
ZC3H12A	3.0031688
ZC3H12C	1.3727431
ZC3H12D	0.6878543
ZC3H13	1.6782985
ZC3H3	0.7113695
ZC3H4	0.28298473
ZC3H6	0.43163443
ZC3H7A	0.59270144
ZC3HAV1	0.49736786
ZCCHC10	0.256433
ZCCHC11	0.74667215
ZCCHC12	1.4571319
ZCCHC18	1.1533427
ZCCHC3	1.5725098
ZCCHC7	0.5957804
ZCCHC8	0.59700155
ZDHHC1	2.0093088
ZDHHC17	1.0458484
ZDHHC18	0.5867281
ZDHHC2	0.2929635
ZDHHC21	0.7285366
ZDHHC22	1.5352554
ZDHHC23	1.2266855
ZDHHC3	0.8378954
ZEB1	1.9909463
ZEB2	1.107501
ZEB2OS	0.90593266
ZFAND2A	0.4003048
ZFAND5	0.2335887

ZFAND6	0.505764
ZFHX3	0.30852485
ZFML	0.8010726
ZFP1	1.2553053
ZFP101	0.9161968
ZFP105	1.3975992
ZFP106	0.3649006
ZFP11	0.6686499
ZFP111	0.34726214
ZFP12	0.70146036
ZFP120	0.5590906
ZFP131	1.1366663
ZFP142	1.2512429
ZFP143	0.7868061
ZFP146	1.0384555
ZFP160	0.2465744
ZFP180	0.47305822
ZFP182	1.5519278
ZFP184	1.1164224
ZFP185	3.5444038
ZFP212	0.51887655
ZFP217	0.988595
ZFP235	0.16667295
ZFP236	1.2412071
ZFP248	1.1746681
ZFP251	1.3658128
ZFP26	1.611006
ZFP260	0.3345027
ZFP263	0.5566368
ZFP28	0.4037671
ZFP280D	1.1413522
ZFP281	0.8686204
ZFP286	0.9607146
ZFP287	2.0005429
ZFP292	0.6438279
ZFP296	0.7170892
ZFP317	1.5490162
ZFP322A	0.43078423
ZFP329	0.76267004
ZFP341	0.33162785
ZFP352	3.3537467
ZFP354C	1.2025154
ZFP36	2.0840645
ZFP365	4.414535
ZFP366	2.8781834

ZFP36L1	0.36052036
ZFP36L3	0.5418632
ZFP37	0.39791036
ZFP382	1.1785305
ZFP384	0.6171503
ZFP397	1.4578094
ZFP40	0.27645612
ZFP407	0.41711378
ZFP418	0.19027233
ZFP426	0.3074503
ZFP429	1.3824034
ZFP433	0.20397282
ZFP438	0.151093
ZFP445	1.5299706
ZFP446	0.34839463
ZFP449	0.79825926
ZFP451	0.6487403
ZFP456	2.3604567
ZFP458	0.5562544
ZFP46	4.009107
ZFP462	2.3526726
ZFP473	1.3220651
ZFP474	4.4476647
ZFP503	1.1689601
ZFP51	1.1695228
ZFP512	0.4478197
ZFP513	0.24955606
ZFP516	0.18695164
ZFP518A	0.5293181
ZFP518B	0.74073267
ZFP52	3.0857625
ZFP521	0.87292075
ZFP53	1.4788926
ZFP532	0.9916978
ZFP536	1.0975051
ZFP560	1.4605131
ZFP566	0.57013845
ZFP568	0.9399719
ZFP579	0.44793653
ZFP58	0.7783487
ZFP580	0.26668596
ZFP583	2.4099696
ZFP592	0.3091488
ZFP593	1.1098957
ZFP595	1.0511389

ZFP597	1.3969178
ZFP605	1.0090601
ZFP607	0.93396854
ZFP608	1.8584352
ZFP61	2.0552244
ZFP62	1.7486863
ZFP626	1.553318
ZFP641	1.6420646
ZFP644	0.32832384
ZFP651	0.9825597
ZFP654	0.316077
ZFP664	1.1936636
ZFP677	1.5236187
ZFP68	0.47885323
ZFP687	0.3897009
ZFP69	0.24833798
ZFP697	5.2287717
ZFP7	1.414443
ZFP703	0.8174405
ZFP704	0.71762276
ZFP708	1.0958657
ZFP709	0.5572684
ZFP711	1.8869834
ZFP712	1.4236851
ZFP715	0.5460849
ZFP719	1.6984959
ZFP74	0.75447536
ZFP740	1.1607332
ZFP747	0.8645687
ZFP748	0.18721962
ZFP758	1.5987353
ZFP764	0.65588856
ZFP78	0.32893205
ZFP781	0.95095825
ZFP783	0.8309672
ZFP790	2.6633348
ZFP800	0.21431446
ZFP809	1.1168814
ZFP81	0.28557944
ZFP819	1.1517451
ZFP82	0.77585554
ZFP821	0.6410432
ZFP830	0.64364576
ZFP839	1.2533894
ZFP84	1.0105953

ZFP850	2.106249
ZFP865	0.39179277
ZFP866	0.22863531
ZFP868	0.5536132
ZFP871	0.46313667
ZFP882	0.32068038
ZFP9	1.244751
ZFP933	0.60390186
ZFP935	0.79179764
ZFP937	0.42535257
ZFP945	0.7184341
ZFP948	0.9109535
ZFP950	0.26584268
ZFP959	0.3483119
ZFPM2	1.0799136
ZFR2	1.654006
ZFX	0.5445018
ZFYVE16	1.1904354
ZFYVE20	0.29772043
ZHX2	2.4376826
ZKSCAN6	0.55514383
ZMIZ1	1.7449102
ZMIZ1OS1	1.1361814
ZMYM2	0.58661747
ZMYM6	0.5174804
ZMYND10	2.3556042
ZMYND11	0.79998493
ZMYND12	3.315847
ZMYND15	1.2882767
ZNFX1	0.58063745
ZNRF1	1.1653929
ZPBP	0.6328945
ZPBP2	0.8774822
ZRANB2	0.44188786
ZRSR2	0.36040807
ZSCAN18	2.2817876
ZSCAN25	1.231415
ZSCAN26	0.37097788
ZSWIM4	0.35605764
ZSWIM6	1.8225579
ZSWIM8	0.3056178
ZUFSP	1.0240841
ZXDB	1.635345
ZYG11B	0.515048
ZYX	0.9059329

ZZZ3	0.22992706

Mice_onset_lung_DEgenes_fc

Gene	log2Fold change
A1BG	-8.555922
A1CF	-7.6254063
A3GALT2	-0.2799187
AAAS	-1.6664977
AACS	-2.1116657
AADAC	-7.7075458
AADAT	-2.6743624
AAED1	-1.8474431
AAGAB	-1.3348379
AAMDC	-2.5179508
AAMP	-1.468214
AANAT	-1.2355244
AAR2	-0.9762707
AARD	-3.0766273
AARS	-1.4256396
AARS2	-1.7737565
AARSD1	-1.258482
AASDH	-1.3080292
AASDHPPT	-1.3125496
AASS	-3.867856
AATF	-0.9698491
ABAT	-6.4116955
ABCA1	-0.2321267
ABCA14	-2.7692475
ABCA2	-2.934729
ABCA3	-0.9189444
ABCA6	-6.6305137
ABCA8A	-3.8970582
ABCA8B	-4.5464764
ABCA9	-2.473921
ABCB10	-1.2065191
ABCB11	-10.187672
ABCB1A	-1.4446754
ABCB4	-5.5032535
ABCB6	-3.8805103
ABCB7	-1.7380159
ABCB8	-1.611412
ABCB9	-0.4564633
ABCC10	-1.3413429
ABCC12	-2.2126508
ABCC2	-5.65816
ABCC3	-2.5171819
ABCC6	-6.879424

ABCC9	-2.155455
ABCD1	-1.8868787
ABCD2	-4.2326984
ABCD3	-3.1256924
ABCD4	-2.0953732
ABCF2	-0.444551
ABCF3	-0.9076738
ABCG2	-2.627481
ABCG3	-1.6754773
ABCG5	-6.6328917
ABCG8	-6.862006
ABHD1	-4.231255
ABHD11	-1.6371179
ABHD12	-0.6547356
ABHD14A	-1.661017
ABHD14B	-4.6504602
ABHD15	-3.727269
ABHD16A	-1.3020205
ABHD16B	-0.9186652
ABHD17A	-2.2746234
ABHD17C	-0.416182
ABHD2	-1.4694963
ABHD4	-0.6158032
ABHD6	-3.6850834
ABHD8	-0.9362321
ABI3	-0.6866827
ABL1	-0.2346706
ABLIM3	-0.2751126
ABT1	-1.1732249
ABTB1	-0.9822631
ABTB2	-0.564425
ACAA1A	-2.1706772
ACAA1B	-6.0395513
ACAA2	-4.339718
ACACA	-2.1160693
ACACB	-5.027814
ACAD10	-3.1772065
ACAD11	-4.5970674
ACAD12	-2.785326
ACAD8	-1.5686302
ACAD9	-1.5042319
ACADL	-1.594039
ACADM	-3.6717138
ACADS	-5.3146524
ACADSB	-0.9649854

ACADVL	-2.9657621
ACAT1	-3.3149633
ACAT2	-4.0307207
ACAT3	-3.8942838
ACBD4	-2.6162724
ACBD5	-2.189314
ACBD6	-2.3589697
ACCS	-1.3226843
ACCSL	-1.0861061
ACD	-0.4623785
ACKR4	-3.096647
ACLY	-2.878932
ACMSD	-6.117151
ACN9	-2.1028998
ACNAT1	-6.587571
ACNAT2	-6.3030024
ACO1	-3.242205
ACO2	-1.4263191
ACOT1	-1.0826883
ACOT12	-5.8785934
ACOT13	-2.9656878
ACOT2	-0.3844845
ACOT3	-7.4303207
ACOT4	-6.546307
ACOT5	-2.0418491
ACOT6	-2.4525168
ACOT8	-2.0821824
ACOX1	-4.019051
ACOX2	-4.4164243
ACP1	-1.5336008
ACP2	-1.5655866
ACP5	-1.1133575
ACP6	-2.7876892
ACPT	-2.871922
ACRBP	-0.4511418
ACSBG2	-1.786551
ACSF2	-0.7793484
ACSF3	-2.6069908
ACSL1	-4.3162003
ACSL3	-1.5159237
ACSL5	-2.9592724
ACSL6	-1.8572922
ACSM1	-3.92912
ACSM3	-5.950413
ACSM4	-1.5836663

ACSM5	-8.168505
ACSS2	-6.1021447
ACSS3	-3.0857046
ACTL10	-3.024538
ACTL6A	-0.8389821
ACTL7B	-1.0034552
ACTL9	-3.391691
ACTN4	-0.1521897
ACTR1B	-0.8274331
ACTR2	-0.470305
ACTR5	-0.5125165
ACTR6	-2.1017423
ACTR8	-1.2179251
ACTRT2	-1.2049823
ACTRT3	-1.6123352
ACVR1C	-1.2700176
ACVR2A	-0.6465271
ACVR2B	-0.4271488
ACY1	-4.70238
ACY3	-5.682586
ACYP2	-2.1554658
ADAD1	-1.5752504
ADAL	-1.8933201
ADAM11	-1.1304343
ADAM12	-1.2257264
ADAM18	-1.6936994
ADAM1B	-1.6158657
ADAM21	-0.5230851
ADAM23	-3.2528088
ADAM25	-1.6769786
ADAM26A	-1.4108162
ADAM3	-1.4528935
ADAM32	-2.2009633
ADAM33	-0.6087005
ADAM4	-1.4095278
ADAM6A	-1.0625644
ADAMDEC1	-2.2812152
ADAMTS12	-0.549614
ADAMTS13	-3.9604404
ADAMTS14	-1.0639181
ADAMTS16	-2.348313
ADAMTS19	-1.1028862
ADAMTS20	-3.5307355
ADAMTS7	-4.3085527
ADAMTSL1	-0.3175988

ADAP2	-0.3276
ADARB2	-1.9483664
ADAT2	-0.63167
ADCK1	-1.8586388
ADCK2	-1.681809
ADCK3	-5.682584
ADCK4	-0.3791122
ADCK5	-2.304397
ADCY10	-1.2494087
ADCY6	-0.9624
ADCY9	-1.8957057
ADCYAP1	-2.2109778
ADGB	-1.4551632
ADH1	-7.6763725
ADH4	-6.621848
ADH5	-3.3281834
ADH7	-1.0349724
ADHFE1	-3.4373112
ADI1	-3.202608
ADIPOR1	-0.7945027
ADIPOR2	-1.8959622
ADK	-3.5222712
ADNP	-0.2864304
ADO	-0.9428406
ADORA1	-1.7042208
ADPGK	-0.8235269
ADPRHL2	-1.8534865
ADPRM	-0.4571252
ADRA1A	-1.7865486
ADRA1B	-2.9773917
ADRA1D	-0.6498163
ADRA2B	-2.9141362
ADRA2C	-1.6764266
ADRB3	-1.4371495
ADRM1	-1.2248697
ADSL	-0.5392051
ADSS	-0.3009353
ADSSL1	-1.0731897
ADTRP	-4.8724384
AEN	-0.1745877
AES	-1.3096895
AFG3L1	-1.7193265
AFG3L2	-1.7627358
AFM	-6.88386
AFMID	-6.7435923

AFP	-1.5251589
AGA	-1.2172422
AGBL1	-1.6709547
AGBL3	-0.7518246
AGBL4	-0.4817548
AGFG2	-1.1921401
AGK	-0.6650796
AGL	-1.6868067
AGMAT	-7.3332553
AGMO	-4.1731505
AGO1	-0.7911201
AGPAT2	-4.343333
AGPAT3	-2.1556973
AGPAT6	-1.8741632
AGPAT9	-1.3018503
AGT	-4.9034066
AGTR1A	-4.0434175
AGTR1B	-1.636785
AGTR2	-1.9234667
AGXT	-6.8663487
AGXT2	-5.1643004
AHCTF1	-0.6932993
AHCY	-4.1074724
AHCYL1	-1.004046
AHDC1	-0.3345413
AHR	-1.2097411
AHRR	-1.8032601
AHSA1	-0.804884
AHSG	-2.5738506
AIF1	-2.0440478
AIFM1	-2.5574408
AIFM2	-2.1372273
AIFM3	-3.1802087
AIG1	-1.1524134
AIM1	-0.4038286
AIMP1	-0.454237
AIMP2	-0.3513098
AIP	-0.5249376
AIPL1	-1.2749698
AIRE	-1.0042343
AK2	-2.3368468
AK3	-3.5279622
AK4	-4.9890113
AK5	-0.8578119
AKAP1	-2.7637305

AKAP17B	-0.5784826
AKAP3	-1.0789652
AKAP4	-1.0817616
AKAP6	-1.1055973
AKAP8L	-0.9460897
AKIP1	-1.3831358
AKP3	-1.3871677
AKR1A1	-1.4821801
AKR1B10	-1.2233005
AKR1B7	-6.953574
AKR1C12	-6.1477613
AKR1C13	-4.9200063
AKR1C14	-1.6365433
AKR1C18	-7.7676725
AKR1C19	-7.056385
AKR1C20	-9.662298
AKR1C6	-12.091644
AKR1D1	-8.518875
AKR1E1	-2.5996344
AKR7A5	-4.7654104
AKT1S1	-1.9431145
AKT2	-0.7412887
ALAD	-4.215611
ALAS1	-2.058629
ALB	-3.2544422
ALDH16A1	-2.7070398
ALDH1A1	-1.4661446
ALDH1A7	-1.7483149
ALDH1B1	-5.5744348
ALDH1L1	-5.1590133
ALDH2	-2.9934263
ALDH3A2	-2.2796092
ALDH4A1	-5.39304
ALDH5A1	-3.4972596
ALDH6A1	-3.2553105
ALDH7A1	-4.671101
ALDH8A1	-6.247167
ALDH9A1	-3.8063483
ALDOB	-3.878439
ALDOC	-2.4826446
ALG14	-1.4639621
ALG2	-0.5763688
ALG3	-1.7117255
ALG5	-1.9544673
ALG6	-1.8614476

ALG8	-1.3131683
ALG9	-1.3763547
ALKBH2	-1.5599303
ALKBH3	-0.5750442
ALKBH4	-0.8052387
ALKBH5	-0.5383582
ALKBH6	-0.1510968
ALKBH7	-2.0023851
ALS2	-1.9782696
ALS2CL	-0.8111911
ALX1	-1.6536126
AMACR	-4.842181
AMBN	-1.0466664
AMBP	-3.127634
AMBRA1	-0.5828748
AMD2	-0.4411941
AMDHD1	-8.227308
AMDHD2	-0.8592157
AMELX	-1.1016257
AMER2	-4.3137627
AMFR	-1.7915487
AMH	-1.7553873
AMHR2	-1.7229891
AMIGO1	-1.2344196
AMIGO2	-1.9537172
AMMECR1L	-1.1650624
AMN	-1.9381387
AMOT	-2.357671
AMPD2	-1.2686081
AMT	-4.93569
AMY1	-4.9435935
AMY2A5	-4.5241976
AMZ1	-0.7219977
AMZ2	-0.7295771
ANAPC1	-0.3391705
ANAPC10	-0.2289262
ANAPC11	-0.4672318
ANAPC13	-1.5182757
ANAPC2	-0.2052145
ANAPC5	-0.7355781
ANAPC7	-0.8151088
ANG	-4.028846
ANG2	-4.2595587
ANG4	-3.3497949
ANGEL1	-1.7615318

ANGEL2	-0.6458902
ANGPTL1	-0.5417132
ANGPTL2	-1.9744244
ANGPTL3	-8.264864
ANGPTL4	-0.6115441
ANGPTL6	-5.340208
ANK	-2.8193266
ANKAR	-1.7061882
ANKDD1A	-1.6992846
ANKDD1B	-1.4555657
ANKEF1	-1.1765399
ANKLE1	-1.9023244
ANKMY1	-0.9048977
ANKMY2	-1.3560917
ANKRD10	-0.4172797
ANKRD13B	-0.8814373
ANKRD13C	-0.5204663
ANKRD13D	-0.3421083
ANKRD24	-3.2263098
ANKRD26	-0.5810931
ANKRD27	-0.9657803
ANKRD28	-0.2972341
ANKRD33	-1.3098333
ANKRD34B	-1.3167145
ANKRD34C	-1.6577015
ANKRD36	-2.423368
ANKRD39	-0.6153364
ANKRD40	-0.4955263
ANKRD46	-0.9512582
ANKRD52	-0.2791309
ANKRD54	-0.4376957
ANKRD55	-1.5853436
ANKRD6	-2.1838381
ANKRD63	-1.1430902
ANKRD65	-1.8661485
ANKRD7	-2.021778
ANKRD9	-0.4306898
ANKS1B	-1.5884967
ANKS3	-0.3574412
ANKS4B	-4.9229136
ANKS6	-0.3461492
ANKUB1	-0.2424274
ANKZF1	-1.4805441
ANLN	-0.6349831
ANO10	-0.5546746

ANO3	-1.5143452
ANO5	-1.6061218
ANO7	-1.6345382
ANP32A	-1.5164609
ANPEP	-2.2344007
ANTXR2	-0.6412296
ANXA10	-2.661473
ANXA13	-2.4499216
ANXA5	-0.2669668
ANXA6	-1.0089121
AOC2	-0.2359543
AOX1	-2.2279387
AOX2	-1.500634
AOX3	-1.4043355
AP1AR	-0.6650903
AP1B1	-1.0945997
AP1G1	-0.5105486
AP1M1	-0.2785811
AP1S1	-1.1010175
AP2A1	-0.5366983
AP2A2	-0.8968167
AP2B1	-1.2056603
AP2M1	-1.1230617
AP2S1	-1.0306382
AP3D1	-0.3455997
AP3M1	-1.8332615
AP3M2	-0.3272286
AP3S1	-0.313777
AP3S2	-0.4212141
AP4B1	-1.0422573
AP4M1	-0.7346263
AP4S1	-0.7397718
AP5M1	-0.8155012
AP5S1	-1.3766298
APBA3	-1.7477112
APBB3	-0.6582832
APC	-1.2554681
APCS	-4.018372
APEH	-2.0333214
APEX1	-1.6131859
APH1A	-1.379631
API5	-0.5571623
APIP	-0.9589148
APLP2	-0.8125434
APMAP	-1.4498839

APOA1	-5.9684196
APOA1BP	-2.420999
APOA2	-4.1857924
APOA4	-3.2281857
APOA5	-4.840757
APOB	-4.9471297
APOBEC1	-0.945219
APOBEC3	-0.4326119
APOC1	-3.2712903
APOC2	-4.479203
APOC3	-6.384361
APOC4	-7.508519
APOE	-2.3241482
APOF	-5.061776
APOH	-5.2754345
APOL10A	-3.221645
APOL11B	-0.4014931
APOL7A	-6.499874
APOL7C	-3.9852617
APOL7D	-1.5596051
APOL8	-0.8462291
APOL9A	-5.1111317
APOL9B	-5.0484877
APOM	-6.568464
APON	-5.4405413
APOO	-1.7334447
APOOL	-1.5512457
APOPT1	-0.7561951
APRT	-0.6572776
AQP11	-6.186006
AQP6	-1.7731271
AQP8	-8.051424
AQP9	-6.1186905
ARAF	-1.3199353
ARAP1	-0.9746938
ARCN1	-0.5901685
AREL1	-1.2191415
ARF1	-0.7547574
ARF4	-0.702806
ARF5	-1.2845054
ARF6	-0.5835009
ARFGAP1	-0.3999686
ARFGAP2	-2.101653
ARFGEF2	-0.3348069
ARFIP1	-0.2452004

ARFRP1	-0.2664533
ARG1	-3.6433387
ARHGAP17	-0.7928987
ARHGAP21	-0.4459224
ARHGAP29	-0.9584184
ARHGAP33OS	-1.0901604
ARHGAP35	-1.2724781
ARHGAP4	-0.3467746
ARHGAP42	-2.4727576
ARHGAP5	-0.4890547
ARHGEF10	-1.4396043
ARHGEF10L	-1.5183911
ARHGEF12	-0.4644008
ARHGEF15	-0.8432951
ARHGEF16	-0.4125214
ARHGEF18	-0.5938325
ARHGEF19	-4.7586126
ARHGEF37	-1.4508796
ARHGEF39	-1.6079986
ARHGEF40	-1.6615644
ARHGEF9	-2.8648717
ARID1A	-0.3603101
ARID3C	-1.8889182
ARIH2	-0.3916097
ARL1	-0.6674814
ARL13A	-1.1695089
ARL14	-2.1567593
ARL14EPL	-2.74612
ARL16	-0.6230032
ARL3	-0.2320747
ARL4AOS	-1.6302814
ARL4D	-0.7321773
ARL5A	-1.426734
ARL6IP4	-0.8048167
ARL6IP5	-0.1960287
ARL6IP6	-0.6003118
ARL8B	-0.3206658
ARL9	-1.1294098
ARMC1	-1.3142142
ARMC12	-2.1798196
ARMC6	-1.2524204
ARMC8	-0.2592144
ARMC9	-1.930237
ARNT	-0.1628237
ARNT2	-1.0990634

ARNTL	-0.4794984
ARNTL2	-0.4705365
ARPC1A	-0.6152787
ARPC4	-0.9404903
ARPC5L	-0.6545382
ARPP19	-0.4401901
ARR3	-0.9000843
ARRDC3	-0.2882328
ARRDC5	-1.1469479
ARSB	-1.4802248
ARSG	-1.8911371
ARSK	-2.0048172
ART2A-PS	-5.1734076
ART4	-1.3524423
ARV1	-0.224618
ARVCF	-2.894963
ARXES1	-1.3739603
AS3MT	-3.4024534
ASAH1	-0.559782
ASAH2	-1.659693
ASAP3	-0.6632905
ASB13	-2.188902
ASB16	-1.0812759
ASB18	-1.4608216
ASB2	-1.1251178
ASB8	-0.7759824
ASB9	-1.0910437
ASCC1	-1.5420351
ASGR1	-5.885416
ASGR2	-6.2418256
ASH2L	-0.225605
ASIC1	-1.517946
ASIC5	-5.0916147
ASL	-4.358794
ASNSD1	-0.9431806
ASPA	-3.797325
ASPDH	-4.2455077
ASPG	-3.524322
ASPM	-0.7505887
ASPSCR1	-2.1011758
ASS1	-4.4865127
ASTN2	-1.2284651
ASXL2	-0.1791549
ATAD1	-0.5498958
ATAD3A	-1.9936361

ATAD3AOS	-2.6525164
ATAT1	-1.4738615
ATCAY	-1.1494355
ATF1	-0.3211703
ATF5	-2.9614897
ATF6B	-0.554369
ATF7IP2	-1.4911232
ATG10	-0.285614
ATG101	-1.2062459
ATG12	-0.3716641
ATG13	-0.5014896
ATG14	-0.8850341
ATG16L1	-1.0685921
ATG16L2	-1.5816951
ATG2A	-1.6471753
ATG2B	-0.8774328
ATG3	-0.9447112
ATG4A	-1.5985456
ATG4B	-1.4790149
ATG4C	-1.6795025
ATG4D	-1.526434
ATG5	-1.9976993
ATG7	-0.7919197
ATG9B	-0.7302928
ATHL1	-2.0157948
ATIC	-0.782145
ATL2	-2.2274694
ATM	-0.4518299
ATOH7	-3.1034536
ATOH8	-1.4510298
ATOX1	-1.7188821
ATP11B	-0.5223007
ATP11C	-2.913218
ATP12A	-2.2976499
ATP13A1	-1.1353946
ATP13A2	-0.7355232
ATP13A5	-0.5288749
ATP1A1	-0.6629996
ATP2B2	-4.608556
ATP4A	-1.695869
ATP4B	-1.5246539
ATP5A1	-1.5414224
ATP5B	-1.8671651
ATP5C1	-2.4078193
ATP5C1-PS	-2.063024

ATP5D	-2.7721362
ATP5E	-2.1601443
ATP5F1	-1.7570071
ATP5G1	-2.3248882
ATP5G2	-1.4583807
ATP5G3	-1.9852748
ATP5H	-1.4355698
ATP5J	-1.988955
ATP5J2	-2.104322
ATP5K	-2.2065883
ATP5L	-1.4784522
ATP5O	-1.9230866
ATP5S	-1.1293559
ATP5SL	-1.8676624
ATP6	-1.8996391
ATP6AP2	-0.1324415
ATP6V0B	-1.5166726
ATP6V0C	-0.5192981
ATP6V0D1	-0.4083319
ATP6V1D	-0.8252368
ATP6V1E1	-1.2028971
ATP6V1E2	-1.1301823
ATP6V1F	-0.5461555
ATP6V1H	-0.6953812
ATP8	-1.820519
ATP8A2	-1.02227
ATP8B1	-1.5747681
ATP8B3	-1.0940068
ATP8B5	-0.4274745
ATP9A	-1.4720507
ATPAF2	-1.8754072
ATPIF1	-0.9384608
ATRAID	-2.0441241
ATRIP	-0.5949268
ATRN	-1.1159778
ATXN2	-1.3679271
ATXN3	-0.4315298
ATXN7	-0.6141014
ATXN7L3B	-1.2900214
AUH	-1.4301963
AUP1	-1.6662121
AURKAIP1	-1.1109653
AVEN	-1.1868844
AVIL	-1.1294401
AVP	-2.2802808

AVPI1	-0.6304679
AVPR1A	-4.49965
AVPR2	-0.9936578
AWAT2	-1.7276182
AXL	-0.2436595
AZGP1	-5.8032675
B2M	-1.1404343
B3GALT1	-0.8008552
B3GALT4	-0.2979131
B3GALT6	-0.409838
B3GAT2	-1.0949054
B3GAT3	-1.0690484
B3GNT1	-1.8669481
B3GNT3	-0.3001466
B3GNTL1	-1.5337121
B4GALNT1	-0.6596975
B4GALNT3	-1.232903
B4GALNT4	-0.7953799
B4GALT7	-0.798954
BAAT	-5.572482
BABAM1	-1.3624444
BACE1	-2.0300803
BACE2	-0.3838415
BAD	-0.9553475
BAG1	-0.3677893
BAG2	-0.3207822
BAG4	-1.8865271
BAG5	-0.4623256
BAG6	-0.8697124
BAHD1	-0.1928105
BAI1	-1.26596
BAI2	-0.2730379
BAI3	-1.5991509
BAIAP2	-1.1196828
BAIAP2L1	-1.3050814
BAIAP3	-2.7836924
BANF1	-1.2988582
BANF2	-1.052671
BANK1	-1.3270645
BANP	-1.8459654
BARX1	-2.8394337
BAZ2A	-0.3463893
BBC3	-1.5180125
BBOX1	-7.3196287
BBS10	-3.299917

BBS5	-1.4639199
BBS7	-0.8093944
BCAN	-0.5220065
BCAP31	-1.7276063
BCAR1	-0.4499793
BCAS1	-0.5430043
BCAS1OS2	-1.1209443
BCAS2	-0.7487865
BCAS3	-0.7173576
BCAT2	-1.7117853
BCCIP	-1.5989952
BCDIN3D	-0.8607497
BCHE	-6.8308935
BCKDHA	-3.9227743
BCKDHB	-3.4680228
BCKDK	-0.5133076
BCL2L10	-0.9948239
BCL2L12	-1.5206227
BCL2L13	-1.8975806
BCL7A	-0.8653581
BCL7C	-0.6766362
BCL9L	-2.1376343
BCMO1	-2.6928766
BCO2	-5.778481
BCOR	-0.3854036
BCORL1	-1.4659209
BCS1L	-2.2183123
BDH1	-6.99183
BDH2	-5.026293
BE949265	-2.0953557
BEND3	-0.2772203
BEND6	-0.9410651
BET1	-1.6129456
BEX6	-1.5858159
BFAR	-0.4113879
BGLAP	-1.7392871
BHLHB9	-1.5627263
BHLHE23	-1.1002283
BHMT	-8.618757
BHMT2	-5.924772
BID	-0.6983581
BIN1	-0.3580461
BIRC5	-0.7903371
BIRC6	-0.3474531
BIVM	-1.9868386

BLCAP	-1.1403198
BLM	-0.3495412
BLMH	-0.8027058
BLOC1S1	-1.6291776
BLOC1S2	-0.8617659
BLOC1S3	-0.7083931
BLOC1S4	-0.4732409
BLOC1S5	-3.0397835
BLOC1S6	-0.4874539
BLVRA	-0.6237049
BLVRB	-3.0083966
BMP1	-2.7085614
BMP10	-2.215372
BMP2	-3.589748
BMP7	-1.15958
BMP8B	-1.1960144
BMYC	-0.5817227
BNIP1	-2.025772
BNIP3	-1.4297695
BOD1	-1.1665344
BOK	-1.6608129
BOLA1	-1.7311594
BOLA2	-0.497673
BOLA3	-2.1670732
BOP1	-0.6593943
BPGM	-0.6175928
BPHL	-4.406013
BPIFA2	-1.1472659
BPIFA3	-0.7673762
BPIFB2	-1.5080628
BPIFB4	-2.3820868
BPIFB9B	-0.9637094
BPIFC	-1.1237688
BPNT1	-1.8115108
BRAP	-1.8412576
BRAT1	-0.806325
BRCA1	-2.1815772
BRCA2	-1.1539047
BRCC3	-0.7818775
BRD3	-1.393961
BRE	-0.2749243
BRF2	-1.1887074
BRI3	-0.2917399
BRI3BP	-1.3433514
BRICD5	-1.8305204

BRINP2	-1.0449796
BRIP1	-0.8777785
BRIP1OS	-2.0222504
BRK1	-0.6625991
BRMS1	-0.4973149
BRS3	-1.4926395
BRSK2	-1.614536
BSCL2	-2.0494719
BSDC1	-0.9985805
BSG	-2.136869
BSN	-1.2233572
BSPH1	-1.2731717
BSX	-2.0175934
BTBD16	-0.9440336
BTBD2	-1.7796569
BTBD6	-2.0414376
BTBD8	-1.1651945
BTBD9	-0.2875671
BTC	-1.6303971
BTD	-3.2700243
BTF3	-0.3061075
BTF3L4	-0.4750233
BTG4	-1.4309032
BTN1A1	-0.9579516
BTN2A2	-1.5718691
BTNL10	-0.3131104
BTNL9	-0.4689574
BUB1B	-0.7806511
BUD31	-0.4162736
BYSL	-0.4308138
BZRAP1	-0.3150954
BZW1	-0.1969452
C1GALT1C1	-0.4706068
C1QA	-2.404663
C1QB	-2.2561574
C1QBP	-1.2312746
C1QC	-2.555378
C1QL1	-2.6636224
C1QL2	-0.9875493
C1QL3	-3.9665852
C1QL4	-2.1652875
C1QTNF1	-0.4624596
C1QTNF2	-0.8079989
C1RA	-1.2156839
C1RB	-0.7302361

C1RL	-3.5433183
C1S1	-1.134778
C1S2	-1.1009212
C2	-2.3774672
C2CD2	-2.8591323
C2CD2L	-1.5431724
C2CD4D	-2.7110562
C3	-1.4945536
C4B	-2.8549414
C4BP	-4.9429207
C5AR2	-1.0415745
C6	-1.2727351
C8A	-5.108748
C8B	-4.738978
C8G	-7.08279
C9	-7.750138
CAB39L	-1.9798121
CABIN1	-0.3712802
CABLES1	-0.3793116
CABLES2	-1.7391944
CABP1	-1.0512154
CABP2	-3.5215533
CABP4	-1.1859162
CABP7	-1.3489478
CACFD1	-1.3476629
CACNA1A	-0.7839832
CACNA1F	-1.3453133
CACNA1G	-1.0536051
CACNA2D3	-1.6416898
CACNB4	-1.9970429
CACNG2	-1.2205484
CACNG3	-1.3841505
CACNG4	-2.463021
CACNG7	-4.198801
CACUL1	-0.5881047
CACYBP	-0.6986318
CADM1	-1.8421421
CADM3	-3.0446825
CADM4	-0.1498559
CADPS2	-0.2559452
CAGE1	-0.5325871
CALB1	-0.9407899
CALB2	-2.4901586
CALCB	-1.0696862
CALCOCO2	-0.8487856

CALD1	-1.2926054
CALHM1	-2.2075078
CALHM2	-1.3725626
CALML4	-1.9742827
CALN1	-1.2198219
CALR	-1.4052315
CALR3	-2.2683034
CALR4	-0.6080868
CAMK1	-1.1440256
CAMK1D	-3.1892633
CAMK2N1	-0.8410168
CAMK2N2	-0.1838512
CAMKMT	-0.6931427
CAML	-0.8943648
CAMSAP3	-1.1551251
CAMTA2	-0.4357758
CAND1	-0.3629036
CAND2	-1.7849262
CANX	-0.2970405
CAPN10	-2.9874105
CAPN12	-1.2415192
CAPN15	-0.7116256
CAPN5	-0.8827465
CAPN6	-1.9985287
CAPN8	-0.9964485
CAPN9	-1.2664802
CAPRIN1	-1.0470924
CAPRIN2	-0.8687098
CAPZA1	-0.5141139
CAPZA3	-1.4670403
CAR1	-4.2217875
CAR10	-1.2263238
CAR11	-1.1627853
CAR14	-5.1350937
CAR2	-0.8069387
CAR3	-5.797546
CAR5A	-10.312325
CAR6	-2.0746229
CAR7	-2.8841982
CARD10	-1.040492
CARD6	-0.6148212
CARHSP1	-0.8410935
CARKD	-1.4656072
CARM1	-0.8292017
CARNS1	-0.5337975

CARS	-0.4735985
CARS2	-1.4308896
CASD1	-0.3995166
CASKIN1	-1.0066524
CASP16	-0.9943733
CASP6	-2.1137185
CASP7	-1.1133952
CASP8	-0.6943903
CASP9	-1.056716
CAT	-4.88025
CATSPER1	-2.048196
CATSPER2	-1.8506026
CATSPERB	-1.3438754
CATSPERD	-1.821074
CATSPERG2	-1.998261
CBLC	-2.636909
CBLN1	-0.8724885
CBLN2	-1.6272054
CBLN3	-1.3529851
CBLN4	-1.7029331
CBR1	-4.0520997
CBR4	-2.1427145
CBS	-5.2166934
CBX1	-0.511198
CBX2	-3.7345815
CBX3	-0.2131949
CBX4	-0.4598618
CBX7	-1.0123882
CBX8	-2.9603398
CBY1	-1.548671
CC2D1A	-0.7547312
CC2D2B	-1.6040761
CCAR1	-0.2503762
CCBL1	-3.8598757
CCBL2	-3.9397116
CCDC101	-0.2973614
CCDC104	-0.3571248
CCDC105	-1.5971403
CCDC106	-1.3728364
CCDC107	-2.3993826
CCDC117	-0.3386788
CCDC12	-0.5684962
CCDC124	-0.8309431
CCDC125	-0.9520612
CCDC130	-0.5322676

CCDC137	-0.5376773
CCDC138	-1.102736
CCDC142	-1.015403
CCDC144B	-0.4274359
CCDC148	-1.4793234
CCDC151	-0.7419658
CCDC152	-0.9461048
CCDC154	-1.703901
CCDC157	-0.3005536
CCDC159	-2.9979682
CCDC162	-2.2174053
CCDC163	-0.2049475
CCDC166	-2.06179
CCDC167	-1.952049
CCDC171	-1.2344949
CCDC172	-1.7788899
CCDC175	-2.154872
CCDC177	-1.7310812
CCDC178	-1.6410787
CCDC183	-1.0735657
CCDC185	-2.495781
CCDC22	-0.7392235
CCDC23	-0.3834686
CCDC25	-0.3666887
CCDC27	-2.6482208
CCDC28A	-1.6072836
CCDC28B	-0.7941337
CCDC3	-2.5734081
CCDC32	-0.2254276
CCDC34	-0.7013538
CCDC36	-0.9087443
CCDC42	-0.4311087
CCDC42OS	-2.4487247
CCDC43	-1.044177
CCDC51	-1.9422936
CCDC53	-1.2149444
CCDC54	-1.5659797
CCDC58	-2.1315603
CCDC61	-0.6890888
CCDC68	-0.6112585
CCDC7	-2.0409691
CCDC70	-1.4322731
CCDC71	-0.8310871
CCDC8	-1.8933797
CCDC80	-1.6727824

CCDC83	-1.3946083
CCDC85C	-1.2232728
CCDC87	-1.2232807
CCDC90B	-0.6366944
CCDC91	-0.7438555
CCDC94	-0.5568166
CCDC97	-0.7603211
CCIN	-3.4483738
CCKBR	-1.5682385
CCL20	-1.6601014
CCL24	-3.4762614
CCL25	-1.080091
CCL27A	-1.4709496
CCL9	-2.4796805
CCM2	-0.7756472
CCNA2	-1.0257478
CCNB1	-0.7551067
CCNB1IP1	-0.6659284
CCNB2	-0.7387066
CCNB3	-1.5935638
CCND1	-0.8985996
CCND3	-0.256062
CCNE1	-0.3237619
CCNF	-1.3052135
CCNH	-1.5800257
CCNI	-0.7379708
CCNL2	-0.5701795
CCPG1OS	-0.3819323
CCR3	-2.9544892
CCR4	-0.649297
CCR5	-0.736053
CCS	-3.73483
CCT2	-0.3036175
CCT3	-0.6675401
CCT4	-0.219862
CCT5	-0.4289775
CCT6A	-0.4518876
CCT6B	-2.0702155
CCT7	-0.8584952
CCT8	-0.3879223
CCZ1	-0.2560563
CD160	-1.1529534
CD164	-0.9498119
CD180	-1.8065138
CD1D1	-5.489639

CD1D2	-5.6875157
CD200R1	-1.6134086
CD200R4	-2.0725062
CD207	-1.5896912
CD209B	-2.2934585
CD209C	-0.198976
CD22	-1.8305578
CD248	-1.7309313
CD27	-0.2464593
CD276	-1.742728
CD2AP	-0.6774759
CD300C	-1.5255132
CD300E	-2.9789872
CD300LB	-1.551337
CD300LD	-2.5910368
CD300LH	-2.2324786
CD302	-2.2162604
CD320	-0.7632842
CD3E	-0.184968
CD4	-3.4742584
CD59A	-3.5795856
CD59B	-4.3090315
CD5L	-5.8807607
CD7	-1.4984822
CD74	-1.1544437
CD81	-2.5081868
CD82	-0.701601
CD86	-0.216464
CD8A	-0.2667754
CD99L2	-0.687438
CDA	-5.89078
CDADC1	-1.3914666
CDC123	-0.8705711
CDC14B	-2.599832
CDC16	-0.7526064
CDC20	-1.3340449
CDC25A	-0.1690798
CDC26	-1.0614939
CDC34	-1.2407498
CDC37	-0.9454732
CDC37L1	-0.8966532
CDC42EP5	-0.6319165
CDCA3	-1.5235028
CDCA5	-0.5743718
CDCA8	-0.459794

CDCP2	-6.809265
CDH17	-1.547636
CDH18	-0.9365137
CDH19	-1.407835
CDH2	-4.100141
CDH20	-2.131555
CDH24	-1.3994622
CDH26	-0.7157407
CDH3	-0.8811805
CDH4	-0.8416414
CDH8	-1.1588719
CDH9	-1.0604022
CDHR2	-1.1625075
CDHR5	-4.2232065
CDIP1	-2.4106822
CDIPT	-0.5870576
CDK1	-1.0921936
CDK10	-1.6963091
CDK16	-0.4429445
CDK18	-1.6485839
CDK19OS	-1.1336591
CDK20	-1.423944
CDK2AP1	-0.6550942
CDK2AP2	-0.9485397
CDK4	-0.3677096
CDK5	-1.1875563
CDK5R2	-2.1230512
CDK5RAP1	-0.7526212
CDK5RAP3	-2.0430498
CDK7	-0.4038541
CDK8	-0.2181048
CDK9	-0.3332443
CDKAL1	-0.3093143
CDKL5	-1.0992503
CDKN1C	-0.526403
CDKN2A	-2.192596
CDKN2AIP	-1.1013448
CDKN2AIPNL	-0.7086959
CDKN2C	-2.2640407
CDKN3	-0.998189
CDO1	-2.937409
CDRT4	-1.1736472
CDS2	-1.688077
CDV3	-0.5403166
CDX1	-1.1592841

CDX4	-3.1525402
CEACAM12	-1.4525476
CEACAM13	-2.2181423
CEACAM2	-0.2063055
CEACAM20	-1.0048926
CEACAM3	-1.3995209
CEACAM5	-0.8816304
CEACAM9	-1.0804124
CEBPA	-1.8883462
CEBPZOS	-2.34861
CECR5	-1.3812535
CELA2A	-1.7671096
CELF1	-0.4642687
CELF3	-1.3231971
CELSR1	-0.2823095
CEND1	-1.6702578
CENPA	-1.3744411
CENPF	-1.4427307
CENPH	-0.590018
CENPI	-0.4709511
CENPK	-1.084446
CENPL	-2.0437107
CENPM	-1.721859
CENPN	-0.9692466
CENPP	-2.0602174
CENPQ	-0.317153
CENPV	-2.2441196
CENPW	-0.8418703
CEP131	-1.7098374
CEP152	-0.6729398
CEP164	-0.390409
CEP170B	-0.8813691
CEP19	-0.6581035
CEP41	-0.8474
CEP44	-1.0134921
CEP57L1	-1.7753086
CEP68	-1.8030863
CEP72	-3.061115
CEP76	-0.2348948
CEP78	-0.6185017
CEP89	-0.3715253
CEP97	-0.4313045
CEPT1	-0.787859
CERKL	-1.7669833
CERS2	-2.072867

CES1B	-6.885148
CES1C	-6.439987
CES1D	-4.1207337
CES1E	-5.9157376
CES1F	-7.885251
CES1G	-8.066416
CES1H	-1.163672
CES2A	-6.6003733
CES2B	-4.902066
CES2C	-6.4131393
CES2E	-6.9534492
CES2G	-4.3401766
CES2H	-2.1560574
CES3A	-9.375826
CES3B	-10.032635
CES4A	-2.9161184
CES5A	-1.4066353
CETN1	-0.3834574
CFB	-1.7198343
CFDP1	-0.4561963
CFH	-2.351603
CFHR1	-6.581663
CFHR2	-3.486517
CFI	-5.2741585
CFL2	-0.9616227
CFP	-1.5623345
CGGBP1	-0.3016367
CGREF1	-2.0882146
CGRRF1	-1.1106839
CHAC2	-3.8662016
CHADL	-2.2755833
CHAF1B	-0.5577822
CHAT	-0.7208324
CHCHD1	-1.183166
CHCHD10	-2.9440718
CHCHD2	-1.6403446
CHCHD3	-2.1065516
CHCHD4	-0.7071242
CHCHD5	-1.5780592
CHCHD6	-0.7589617
CHCHD7	-1.2356372
CHD1L	-0.424767
CHD3	-1.1313109
CHD9	-0.5262532
CHDH	-5.0213375

CHFR	-0.3779116
CHID1	-1.85865
CHKA	-1.5885577
CHKB	-1.311521
CHMP1A	-0.9513607
CHMP2A	-0.3463016
CHMP3	-0.3690462
CHMP5	-0.6023369
CHMP6	-1.3995829
CHMP7	-1.3275776
CHN1OS3	-1.5943477
CHN2	-3.4076538
CHODL	-2.0892613
CHORDC1	-1.4468431
CHP1	-1.0545464
CHP2	-3.480878
CHPF2	-0.7679272
CHPT1	-1.4161444
CHRAC1	-0.3034258
CHRD	-0.9109039
CHRDL1	-0.8109739
CHRM1	-1.4331248
CHRM5	-2.2712235
CHRNA2	-2.3636346
CHRNA4	-2.9210727
CHRNA5	-1.9414175
CHRNA6	-0.7702167
CHRNA9	-1.0735142
CHRNB2	-1.6813495
CHRNB3	-1.403089
CHRNG	-5.720277
CHST13	-4.129434
CHST2	-1.1344259
CHST7	-1.0123343
CHST9	-2.1611264
CHSY3	-1.5674813
CHTF18	-0.9802804
CHTF8	-1.5345812
CHUK	-1.5605774
CHURC1	-1.3339744
CIAO1	-1.8773537
CIAPIN1	-1.243938
CIART	-1.5642471
CIB1	-1.8766975
CIB3	-1.5303714

CIDEB	-5.8306055
CINP	-0.805974
CIPC	-0.4524751
CIRH1A	-0.5211391
CISD1	-3.7925606
CISD2	-0.6605797
CISD3	-3.0383248
CK137956	-2.1806054
CKAP2	-2.2773705
CKB	-1.300385
CKS1B	-0.2676354
CLCA1	-0.27491
CLCC1	-0.9994788
CLCN2	-5.7216597
CLCN3	-1.0609231
CLCN4-2	-1.5718379
CLCN7	-1.2201443
CLDN1	-3.6325343
CLDN12	-2.4039311
CLDN14	-2.1751513
CLDN15	-0.6234598
CLDN16	-1.643707
CLDN2	-3.098474
CLDN26	-0.5375583
CLDN3	-1.0551262
CLDND2	-1.4811687
CLEC11A	-2.7441707
CLEC12A	-3.808196
CLEC12B	-1.9372802
CLEC16A	-0.6832554
CLEC18A	-1.2522225
CLEC1B	-4.06743
CLEC2D	-0.2340927
CLEC2E	-3.6769066
CLEC2F	-1.244359
CLEC2G	-0.8175759
CLEC2H	-5.4667115
CLEC4B2	-1.6328657
CLEC4F	-9.348283
CLEC4G	-9.776196
CLEC7A	-0.9118342
CLEC9A	-0.5394869
CLHC1	-0.7299888
CLK3	-0.5171413
CLMN	-2.5739956

CLN3	-0.41188
CLN5	-0.5771184
CLN6	-0.9492116
CLN8	-1.184247
CLNS1A	-0.69873
CLP1	-1.4165573
CLPB	-2.1785336
CLPP	-1.8109379
CLPTM1	-2.336124
CLPTM1L	-0.6630378
CLPX	-3.5161676
CLRN1	-0.9296043
CLRN2	-1.638757
CLSTN3	-2.6834795
CLTA	-0.4676895
CLTC	-0.5643807
CLU	-1.9247456
CLUAP1	-0.3290482
CLUH	-3.2998862
CLVS1	-1.1653681
CLYBL	-4.042873
CMAH	-1.5243807
CMAS	-0.6935935
CMBL	-5.9034915
CMC1	-1.7194748
CMC2	-2.3883572
CML1	-6.95284
CML2	-6.1057997
CML5	-2.51935
CMPK1	-0.5341339
CMSS1	-0.7402511
CMTM4	-0.6555109
CMTM8	-3.0922694
CNBD1	-1.7518604
CNBP	-0.648314
CNDP1	-1.4408381
CNDP2	-0.6624155
CNGA1	-1.319565
CNGA3	-0.9648953
CNGA4	-1.142199
CNGB3	-1.8321297
CNIH1	-1.6284518
CNIH3	-3.0252717
CNN3	-0.4200878
CNNM1	-2.1344707

CNNM2	-0.2614212
CNNM3	-1.4960732
CNOT1	-0.9610782
CNOT10	-0.5304956
CNOT3	-1.2142007
CNOT6L	-0.2289085
CNOT7	-0.1678333
CNOT8	-1.1926379
CNP	-1.3255839
CNPY1	-1.7221675
CNPY2	-2.2558155
CNPY3	-1.2583795
CNR2	-0.2816467
CNST	-0.6247788
CNTD1	-1.6285481
CNTN2	-2.8582015
CNTN6	-3.3383522
CNTNAP1	-1.3899043
CNTNAP2	-1.6531689
CNTNAP3	-1.7041585
CNTNAP5B	-1.8747439
CNTROB	-1.7276285
COA3	-1.7343502
COA4	-1.9080672
COA5	-1.0974298
COA6	-2.913981
COA7	-2.3152406
COASY	-2.963027
COBL	-0.8194394
COBLL1	-1.0784497
COCH	-0.9283822
COG1	-0.2597928
COG2	-0.9514337
COG4	-1.7404261
COG6	-0.7097693
COG8	-1.2396502
COIL	-0.4819632
COL11A1	-1.171967
COL11A2	-0.2618089
COL14A1	-3.9313476
COL15A1	-0.6734815
COL18A1	-4.6547546
COL20A1	-1.012222
COL24A1	-0.9932127
COL25A1	-1.737289

COL27A1	-1.8141141
COL28A1	-2.1777315
COL2A1	-0.8563361
COL3A1	-0.6125345
COL5A1	-1.1983514
COL5A3	-2.4891067
COL6A3	-0.7623615
COL6A4	-1.1084769
COL6A5	-1.7287531
COL6A6	-1.3336627
COL9A1	-0.8154461
COLEC10	-7.796467
COLEC11	-9.058813
COLEC12	-1.5519145
COMMD1	-2.053761
COMMD2	-1.5799565
COMMD3	-2.3764482
COMMD4	-1.0586529
COMMD5	-1.8602829
COMMD6	-1.6160531
COMMD7	-0.5066395
COMMD8	-0.320179
COMMD9	-2.1214561
COMT	-3.6466455
COMTD1	-1.1090803
COPA	-0.6180863
COPB1	-0.7541165
COPB2	-0.7948895
COPE	-1.5421314
COPG2	-1.1101627
COPG2OS2	-1.2420747
COPRS	-0.6563568
COPS2	-0.3924031
COPS3	-1.2709718
COPS5	-1.1495261
COPS6	-2.1152945
COPS7A	-1.0291257
COPS7B	-1.5966709
COPZ1	-1.3942065
COPZ2	-3.1907642
COQ10A	-1.3796234
COQ2	-1.378408
COQ3	-1.1206198
COQ4	-2.149498
COQ5	-1.5140963

COQ6	-1.7541761
COQ7	-1.9570937
COQ9	-3.3064742
CORIN	-1.2566738
CORO1B	-1.0146918
CORO7	-0.9137135
COX1	-1.2160263
COX11	-1.3752732
COX14	-0.8018704
COX15	-1.0021753
COX16	-1.6266651
COX17	-1.4946494
COX18	-1.2587562
COX19	-1.818593
COX2	-0.9920006
COX20	-1.7564011
COX3	-1.1202073
COX4I1	-1.5482597
COX5A	-1.7663918
COX5B	-1.297554
COX6A1	-1.7094736
COX6B1	-1.4787378
COX6B2	-0.2067432
COX6C	-1.7160263
COX7A2	-1.9038534
COX7A2L	-1.3800306
COX7B	-2.0241165
COX7C	-1.7399206
COX8A	-1.4333072
CP	-1.675426
CPA1	-2.0824137
CPA2	-0.6594923
CPA6	-1.2222648
CPB2	-2.886948
CPEB2	-0.5977445
CPEB3	-1.3241534
CPED1	-0.5777204
CPHX1	-3.0083299
CPLX2	-1.6821346
CPLX4	-0.8941526
CPN1	-4.952672
CPN2	-6.7637987
CPNE3	-0.6451445
CPNE4	-2.6569593
CPNE9	-2.3762133

CPOX	-4.3727484
CPPED1	-2.932703
CPQ	-2.8076963
CPS1	-6.1775727
CPSF1	-1.3306432
CPSF3	-0.8574901
CPSF3L	-1.1557784
CPSF4	-1.3033342
CPSF4L	-4.242785
CPT1A	-2.5521364
CPT2	-3.440208
CPVL	-2.241803
CPXCR1	-3.8073533
CPZ	-0.5039394
CR2	-0.8204019
CRADD	-1.3784785
CRAT	-0.4435904
CRB3	-0.8871088
CRBN	-0.5760274
CRCP	-1.3191929
CREB3L3	-4.2809844
CREB5	-1.1339779
CREBBP	-0.8321123
CREBZF	-0.5599928
CREG1	-2.3379622
CRELD1	-2.004939
CRHBP	-1.576683
CRISP4	-1.6714582
CRLS1	-2.8904362
CRNN	-3.0926595
CROCC	-1.4528837
CROT	-5.2019815
CRP	-4.8669586
CRTAP	-0.5536246
CRTC1	-0.7430458
CRTC2	-0.2606034
CRYBA1	-1.2016687
CRYBA4	-1.9092953
CRYBB1	-1.8094962
CRYBB2	-1.6683545
CRYGA	-1.2713871
CRYGC	-0.9912899
CRYGF	-1.8418729
CRYGN	-3.3059735
CRYGS	-2.394049

CRYL1	-1.4998856
CRYM	-2.9061894
CRYZ	-4.4288583
CSAD	-2.5304356
CSDE1	-0.5732398
CSE1L	-0.6110048
CSF1R	-1.6737852
CSK	-1.4345317
CSL	-2.0327516
CSMD2	-2.4149265
CSMD3	-2.0946724
CSN1S1	-1.7628238
CSN1S2A	-1.7277563
CSN1S2B	-1.6930199
CSN2	-2.9125679
CSN3	-1.4456766
CSNK1G2	-1.1676502
CSNK2A1	-0.9616704
CSNK2A2	-0.6104879
CSNK2B	-1.0492702
CSPP1	-0.516628
CSPRS	-0.3308759
CSRNP2	-0.9777601
CSRP2	-1.3163376
CSRP2BP	-0.8663044
CSRP3	-2.874599
CST11	-1.5911603
CST9	-1.5926826
CSTF1	-0.9116778
CSTF2T	-0.881844
CSTF3	-0.3870502
CSTL1	-1.6248372
CTAGE5	-1.940691
CTC1	-1.0508604
CTCFL	-3.8375113
CTCFLOS	-1.8985143
CTDNEP1	-0.4625578
CTDSP1	-0.7251892
CTDSP2	-0.4367938
CTF1	-2.6684613
CTF2	-1.8932946
CTH	-6.336088
CTIF	-2.0794506
CTNNA2	-1.9329596
CTNNBIP1	-1.2098737

CTNS	-1.2371764
CTR9	-0.3441453
CTRB1	-2.7818635
CTS6	-1.2403166
CTS7	-1.0329804
CTSA	-1.0057526
CTSB	-0.9400768
CTSC	-0.6685591
CTSD	-0.6026707
CTSE	-2.5387154
CTSF	-2.7870731
CTSG	-1.7255859
CTSH	-1.1085153
CTSJ	-1.5758865
CTSL	-1.6545544
CTSM	-0.5782819
CTSO	-0.7087777
CTSQ	-2.3648293
CTSW	-1.1270361
CTSZ	-1.3296423
CTTN	-0.3066263
CTTNBP2	-1.0035689
CTU1	-0.8281999
CTU2	-0.4993334
CUEDC2	-0.1842217
CUL2	-0.5553594
CUL4A	-1.0779519
CUL4B	-0.3606267
CUL5	-0.2638784
CUL9	-1.1327205
CUTA	-0.4741678
CUTAL	-2.8101196
CUTC	-2.0545254
CUX2	-3.4566054
CWC15	-0.3402495
CWF19L1	-2.2490485
CWF19L2	-0.19379
CXADR	-1.6175733
CXCL11	-0.4411883
CXCL12	-3.320075
CXCR6	-0.2364526
CXXC1	-0.5683899
CXXC5	-3.919864
CYB561A3	-0.9595218
CYB561D1	-0.3132186

CYB561D2	-1.3946643
CYB5A	-2.9001784
CYB5B	-2.9247441
CYB5D1	-0.3379486
CYB5D2	-2.1676784
CYB5R3	-2.7377434
CYB5RL	-2.6956775
CYC1	-2.5470033
CYCS	-0.8484564
CYCT	-1.0182154
CYHR1	-1.9086156
CYLC1	-2.2107139
CYLC2	-2.773659
CYM	-1.0194781
CYP11A1	-1.2349062
CYP11B2	-2.2083507
CYP17A1	-0.9617806
CYP1A1	-2.6043615
CYP1A2	-9.208015
CYP20A1	-0.553395
CYP21A1	-1.8347011
CYP24A1	-1.6951194
CYP26A1	-4.8709207
CYP26B1	-2.040852
CYP26C1	-3.7195764
CYP27A1	-5.764388
CYP2A12	-6.4432926
CYP2A22	-7.7129908
CYP2A4	-5.8693795
CYP2B13	-10.1465
CYP2B19	-1.4683905
CYP2B23	-1.0999694
CYP2B9	-2.6060538
CYP2C29	-8.666621
CYP2C37	-9.003267
CYP2C38	-8.51778
CYP2C39	-8.840243
CYP2C44	-10.245741
CYP2C50	-9.413102
CYP2C54	-8.796754
CYP2C55	-6.7355294
CYP2C67	-10.34764
CYP2C68	-10.36334
CYP2C70	-6.572914
CYP2D10	-5.4644628

CYP2D11	-6.2537985
CYP2D12	-6.410355
CYP2D13	-7.112501
CYP2D22	-6.1951246
CYP2D26	-6.0651455
CYP2D34	-5.9771805
CYP2D37-PS	-7.933751
CYP2D40	-8.80895
CYP2D9	-6.4779944
CYP2E1	-6.4041286
CYP2F2	-0.838922
CYP2G1	-7.849597
CYP2J13	-4.1550617
CYP2J5	-4.2692013
CYP2J6	-3.4901521
CYP2J8	-5.578669
CYP2J9	-2.4631987
CYP2R1	-4.1604247
CYP2T4	-0.4512208
CYP2U1	-0.8772502
CYP2W1	-1.297801
CYP39A1	-2.3989596
CYP3A11	-8.156159
CYP3A13	-4.550012
CYP3A16	-7.968198
CYP3A25	-10.318481
CYP3A41B	-8.008171
CYP3A44	-11.271824
CYP3A57	-10.3561
CYP3A59	-8.669407
CYP46A1	-0.4313772
CYP4A10	-9.778972
CYP4A12A	-1.7769713
CYP4A12B	-1.1878338
CYP4A14	-10.958382
CYP4A31	-9.558351
CYP4A32	-9.414268
CYP4F13	-2.9327621
CYP4F14	-8.399601
CYP4F15	-5.9423018
CYP4F16	-1.3676319
CYP4F17	-3.0890536
CYP4F40	-1.7637768
CYP4V3	-3.4838243
CYP51	-3.5073812

CYP7A1	-10.199405
CYP7B1	-1.0791755
CYP8B1	-7.695426
CYPT10	-1.4207239
CYPT14	-3.4625857
CYPT2	-1.207643
CYPT9	-1.5360434
CYS1	-1.1301432
CYSLTR1	-0.2180095
CYSLTR2	-0.5897908
CYTB	-1.3913078
CYTH1	-0.14817
DAB1	-0.9696615
DACH2	-2.0208006
DACT2	-4.0101767
DAD1	-1.1893706
DAGLB	-1.6735435
DAK	-5.903858
DALRD3	-1.0283859
DAND5	-1.3398764
DAP	-2.8301048
DAP3	-1.3276749
DAPK1	-0.3688269
DAPK2	-1.7390409
DARS	-0.915935
DARS2	-2.395175
DBHOS	-1.1033454
DBI	-3.1820002
DBIL5	-1.8574929
DBNDD2	-0.457511
DBP	-3.7175517
DBR1	-0.6724968
DBT	-2.8727558
DCAF11	-2.4833236
DCAF12L1	-2.9186664
DCAF17	-0.9780119
DCAF4	-1.3487496
DCAF5	-0.5303359
DCAF6	-0.5072956
DCAF7	-0.3364978
DCAF8	-1.223969
DCAKD	-1.5869069
DCDC2A	-1.7168307
DCHS1	-0.712738
DCLK3	-6.610832

DCLRE1A	-2.1090472
DCPP3	-1.2690823
DCPS	-0.5378914
DCST2	-1.596091
DCT	-8.200916
DCTN2	-1.2968879
DCTN3	-0.699398
DCTN5	-0.6251807
DCTPP1	-2.707952
DCUN1D1	-0.4766092
DCUN1D2	-0.9548397
DCUN1D4	-1.703342
DCXR	-3.1169553
DDAH1	-3.4863272
DDB1	-0.9557228
DDB2	-0.8316536
DDC	-5.7532854
DDHD1	-0.5990479
DDHD2	-0.5223038
DDI1	-1.660522
DDI2	-1.6463728
DDIT4L	-2.3230476
DDO	-2.5124094
DDOST	-0.3027778
DDRGK1	-1.7670422
DDT	-4.376769
DDX1	-0.7010541
DDX18	-0.2143269
DDX19A	-0.8181915
DDX19B	-0.1550312
DDX27	-0.1559968
DDX28	-0.9778857
DDX31	-0.2729588
DDX3Y	-1.7818484
DDX41	-1.4773436
DDX42	-0.2083559
DDX47	-0.5185437
DDX49	-0.3260961
DDX51	-0.6104722
DDX54	-0.2408314
DDX56	-1.3781219
DDX60	-1.622843
DEAF1	-1.261342
DEAR1	-1.3861179
DEB1	-2.3470855

DECR1	-3.4257994
DECR2	-4.46141
DEF8	-2.4230876
DEFA1	-1.676867
DEFA10	-1.8369348
DEFA2	-2.035478
DEFA21	-2.6177454
DEFA23	-1.3154442
DEFA3	-1.7910342
DEFA-PS13	-1.6006045
DEFA-RS10	-0.2083905
DEFA-RS2	-1.4602234
DEFA-RS4	-0.892848
DEFA-RS7	-0.9057295
DEFB11	-1.7049313
DEFB15	-1.9110625
DEFB19	-1.5589824
DEFB20	-2.0207903
DEFB21	-3.802979
DEFB22	-1.0180979
DEFB25	-0.7242794
DEFB26	-4.235504
DEFB28	-2.2499108
DEFB30	-0.6399801
DEFB33	-0.6215172
DEFB34	-2.7100065
DEFB35	-1.589664
DEFB36	-2.206946
DEFB38	-1.6047251
DEFB40	-1.5734017
DEFB41	-0.9921248
DEFB42	-0.8944376
DEFB43	-1.5847931
DEFB44-PS	-1.6411612
DEFB47	-1.057919
DEFB6	-2.501064
DEFB9	-1.4091456
DEGS1	-0.7332044
DENND1A	-0.9032333
DENND2C	-0.4229715
DENND2D	-0.362042
DENND4A	-0.9872108
DENND5B	-0.5615768
DENND6A	-0.8616934
DENND6B	-1.903419

DENR	-0.5596051
DEPDC1A	-0.5842624
DEPDC1B	-0.2258868
DEPDC5	-0.3244157
DEPDC7	-4.4701395
DEPTOR	-1.881505
DERA	-3.1273513
DERL1	-1.320251
DERL2	-1.7352319
DERL3	-0.2807879
DESI1	-0.9856606
DESI2	-1.3244686
DET1	-2.6378207
DEXI	-1.8201246
DFFA	-0.5002849
DFNA5	-2.2664583
DFNB59	-1.1900978
DGAT1	-0.8741994
DGAT2	-4.277229
DGCR2	-1.0148139
DGCR6	-2.4424405
DGKB	-1.1975591
DGKQ	-1.3638878
DGKZ	-1.1957498
DGUOK	-1.2021747
DHCR24	-5.7844815
DHCR7	-5.280242
DHDDS	-0.7073822
DHDH	-4.4506664
DHFR	-3.2884007
DHODH	-1.4271927
DHRS1	-3.1649942
DHRS11	-3.5225906
DHRS13	-1.434403
DHRS2	-1.7416036
DHRS3	-1.2759223
DHRS4	-3.1976619
DHRS7	-2.0382195
DHRS7B	-2.033256
DHRSX	-0.803977
DHTKD1	-4.39072
DHX16	-1.0479193
DHX32	-0.8852005
DHX33	-0.4726157
DHX34	-0.7792826

DHX35	-0.4263625
DHX36	-0.8494878
DHX37	-1.1656563
DHX57	-0.8074508
DHX58	-0.2310143
DIABLO	-0.9347425
DICER1	-0.9871078
DIDO1	-0.4025111
DIO1	-6.8035274
DIO3	-1.7224
DIO3OS	-3.0199342
DIP2C	-0.7249227
DIRAS1	-1.8964579
DIS3L	-0.685091
DIS3L2	-1.1231785
DISC1	-2.6634545
DISP1	-1.2983642
DIXDC1	-1.9314129
DKC1	-0.4845467
DKK4	-2.9475307
DLAT	-0.5298958
DLD	-1.547174
DLEC1	-0.7443242
DLEU2	-1.6978726
DLEU7	-1.6079586
DLG3	-0.8281226
DLGAP2	-1.207576
DLGAP5	-1.6046166
DLK2	-0.8499596
DLL3	-0.3507533
DLST	-1.3503351
DLX1	-1.9668796
DLX2	-1.6600375
DLX3	-0.556973
DLX6AS2	-0.9577265
DMAP1	-0.5899701
DMBX1	-1.6847501
DMC1	-1.1731119
DMGDH	-5.593385
DMPK	-3.3960614
DMRTB1	-0.7992976
DMRTC1B	-1.014384
DMRTC2	-1.7154987
DMTN	-0.6432328
DMWD	-0.3528585

DNA2	-1.0834217
DNAAF1	-1.0117424
DNAH1	-1.1400683
DNAH3	-1.6421084
DNAIC1	-1.854166
DNAJA1	-2.1247115
DNAJA2	-1.1340141
DNAJA3	-2.658451
DNAJB12	-1.8107696
DNAJB14	-0.4004159
DNAJB2	-1.0914941
DNAJB8	-1.2816508
DNAJC11	-1.5331514
DNAJC12	-2.3212342
DNAJC15	-1.6986022
DNAJC16	-1.2297044
DNAJC19	-1.1384335
DNAJC22	-4.613846
DNAJC24	-0.2448907
DNAJC25	-2.3322477
DNAJC28	-0.622262
DNAJC30	-1.6523752
DNAJC4	-1.5674481
DNAJC5G	-1.5954621
DNAJC6	-1.4770722
DNAL1	-1.4741001
DNASE1L3	-3.6095014
DNASE2B	-4.3749323
DND1	-0.4824445
DNER	-1.7814987
DNLZ	-1.9862766
DNM1L	-0.5725699
DNMBP	-1.5261252
DNMT3A	-0.7714419
DNMT3B	-1.772716
DNPEP	-0.9776983
DNPH1	-3.5006542
DNTT	-5.7765913
DOC2A	-0.5448501
DOC2G	-0.484576
DOCK3	-1.1127114
DOCK4	-0.4652104
DOCK6	-0.8336363
DOCK8	-0.2006688
DOHH	-0.6914678

DOLK	-1.4027095
DOLPP1	-1.203104
DOPEY2	-0.7275906
DOS	-1.0767145
DOT1L	-0.2913566
DPAGT1	-1.1295037
DPCD	-0.7789388
DPEP3	-1.3008404
DPF1	-1.4760537
DPF2	-0.2251391
DPF3	-3.4738576
DPH1	-1.5731845
DPH3	-0.1502633
DPH7	-0.5018182
DPM1	-1.4465623
DPM2	-0.7703829
DPM3	-1.7947764
DPP3	-1.466394
DPP4	-3.6613803
DPP7	-2.235447
DPP9	-0.5481057
DPPA1	-2.4240208
DPPA3	-1.0603738
DPPA4	-0.6661048
DPPA5A	-1.6280615
DPY19L1	-1.0697412
DPY19L2	-1.3284678
DPY19L3	-0.5746946
DPY30	-0.9673338
DPYD	-6.079564
DPYS	-8.715271
DPYSL4	-1.827326
DPYSL5	-0.8164139
DQX1	-2.9111347
DRAM2	-0.7787175
DRAXIN	-2.9197364
DRD4	-2.6645927
DRG1	-0.7688246
DRG2	-1.6961107
DSC2	-3.2466464
DSC3	-1.9069228
DSCR3	-0.7724972
DSE	-0.8035948
DSG1B	-1.4695477
DSG1C	-0.8656583

DSG2	-2.5495608
DSP	-0.5529528
DSPP	-1.2533464
DTD1	-1.0806613
DTD2	-2.1351836
DTNBP1	-0.9714789
DTWD1	-1.342833
DTWD2	-1.0431223
DUOX2	-0.9144459
DUS1L	-1.8191485
DUS2	-1.3568668
DUS3L	-0.3293104
DUS4L	-0.8203361
DUSP12	-0.7054377
DUSP15	-2.6236465
DUSP19	-1.995969
DUSP22	-2.1590838
DUSP23	-0.5902884
DUSP28	-1.761487
DUSP3	-0.3045588
DUSP6	-0.2935066
DUSP9	-0.7945201
DUT	-0.8447635
DUX	-1.4587066
DUXBL1	-1.0487761
DUXBL2	-0.9769535
DVL1	-1.8104148
DVL2	-1.1325076
DXERTD223E	-0.3388426
DXO	-1.1070151
DYDC1	-0.9015272
DYM	-0.7220869
DYNC1I1	-0.5605171
DYNLL2	-2.6843805
DYNLRB1	-1.1272435
DYNLT3	-0.388515
DYRK1B	-1.382338
DYRK2	-0.9367275
DYRK4	-1.631757
DZIP3	-0.7875478
E2F1	-1.3958883
E2F3	-0.9819365
E2F6	-1.0907068
E2F8	-1.0579407
EAF1	-0.43782

EAF2	-0.5210905
EAPP	-0.3163996
EAR4	-2.4036036
EAR7	-0.2789137
EARS2	-2.1464357
EBAG9	-1.0947547
EBP	-3.2825365
EBPL	-5.23264
ECD	-1.2377672
ECE1	-0.2083583
ECE2	-1.4450164
ECH1	-3.5407677
ECHDC1	-1.0725834
ECHDC2	-3.3682885
ECHDC3	-6.4591494
ECHS1	-3.0576773
ECI1	-3.2999325
ECI2	-2.555214
ECI3	-2.7585583
ECM1	-0.393343
ECSIT	-1.1222119
ECT2	-1.5233035
EDA	-1.1303144
EDA2R	-1.54879
EDARADD	-1.0536268
EDC3	-0.5069621
EDC4	-0.1598992
EDDM3B	-1.581368
EDEM1	-1.8993802
EDEM2	-0.8004713
EDEM3	-0.3225503
EDF1	-1.8319302
EDNRB	-1.8620381
EDRF1	-0.3268137
EEF1A1	-1.2973361
EEF1B2	-0.928134
EEF1D	-0.8496208
EEF1G	-0.5927844
EEF2	-1.3829598
EEF2K	-0.2070012
EEFSEC	-2.3171654
EEPD1	-1.5296948
EFCAB11	-0.7273068
EFCAB3	-1.3354654
EFCAB8	-1.2856746

EFCAB9	-1.0415249
EFCC1	-0.8575921
EFNA1	-1.0443654
EFNA2	-0.9310451
EFR3A	-0.842495
EGF	-0.8481238
EGFEM1	-2.115844
EGFLAM	-2.0098097
EGFR	-1.9478359
EGLN1	-0.5510752
EGLN2	-0.2557707
EGLN3	-0.5204458
EHBP1	-0.2969379
EHD3	-3.9182177
EHHADH	-6.994393
EHMT1	-0.1852441
EHMT2	-0.6644869
EI24	-2.3649917
EID2B	-1.1249177
EIF1A	-0.3817635
EIF1AD	-0.4768639
EIF1B	-0.333539
EIF1-PS	-0.9706907
EIF2A	-0.5014958
EIF2AK1	-0.8289924
EIF2B1	-1.5778747
EIF2B4	-1.4432001
EIF2B5	-0.8126621
EIF2D	-2.1383853
EIF2S1	-0.1999769
EIF2S3X	-0.6434827
EIF2S3Y	-1.7295148
EIF3A	-0.5876546
EIF3D	-0.2418928
EIF3E	-0.1659827
EIF3F	-0.5996118
EIF3G	-0.3163934
EIF3H	-0.4779863
EIF3I	-1.3717299
EIF3J1	-0.4532466
EIF3K	-0.3539972
EIF3L	-0.6810904
EIF3M	-0.4592271
EIF4A3	-0.256393
EIF4B	-0.6426454

EIF4E	-0.4703555
EIF4E1B	-2.0025375
EIF4E2	-0.9537339
EIF4E3	-0.4354453
EIF4EBP1	-0.7562389
EIF4EBP2	-1.6378198
EIF4EBP3	-0.8729935
EIF4G1	-1.3010726
EIF4H	-0.1945829
EIF5A	-1.1672153
EIF5B	-0.443974
EIF6	-0.757782
ELAC1	-0.5207949
ELAC2	-1.1775193
ELAVL3	-2.1886318
ELFN1	-1.2898593
ELFN2	-1.0439444
ELK1	-0.5819035
ELK3	-0.3294492
ELL	-0.2761798
ELL2	-0.4864364
ELL3	-3.230442
ELMO3	-1.583971
ELMOD2	-0.5566635
ELMOD3	-1.3485098
ELOF1	-0.5426536
ELOVL2	-5.52707
ELOVL5	-3.3174505
ELOVL6	-3.9552107
ELP2	-0.3962064
ELP3	-0.7408042
ELP4	-1.0503182
ELP5	-0.5647473
ELP6	-1.0996411
ELTD1	-0.7504261
EMC1	-0.3513999
EMC10	-1.9483905
EMC2	-0.3763738
EMC3	-1.3517737
EMC4	-0.7706084
EMC6	-1.5121994
EMC7	-1.4902601
EMC8	-1.3210068
EMC9	-3.4191918
EME1	-0.5727799

EME2	-1.370975
EMG1	-0.9149752
EMILIN1	-0.4307113
EMILIN3	-1.3184597
EML3	-0.4751916
EML4	-0.3804212
EMR1	-0.8667159
EMR4	-1.913547
EMX1	-2.6074128
EMX2	-1.5090532
EMX2OS	-1.2049844
EN2	-1.2788103
ENAM	-1.757086
ENDOG	-2.589892
ENDOV	-0.4922919
ENGASE	-1.6272097
ENHO	-3.5042653
ENO1	-1.6615615
ENO1B	-1.7582293
ENOX1	-1.160366
ENOX2	-1.166894
ENPEP	-3.176599
ENPP1	-1.8475158
ENPP2	-1.8514776
ENPP3	-2.1563528
ENPP5	-1.2988825
ENSA	-0.5324187
ENTHD1	-1.734025
ENTHD2	-1.2458587
ENTPD2	-1.6224623
ENTPD4	-0.8170171
ENTPD5	-3.333005
ENTPD6	-2.4665978
ENTPD7	-0.9524224
ENTPD8	-8.564732
ENY2	-1.4222865
EPB4.1	-0.4439001
EPB4.1L3	-1.2938333
EPB4.1L4B	-2.553402
EPB4.1L5	-1.1186111
EPB4.2	-1.6849971
EPG5	-1.0076814
EPHA1	-4.0408154
EPHA10	-1.3599625
EPHA5	-0.816484

EPHA6	-1.2387385
EPHA7	-0.8188951
EPHB2	-0.6115327
EPHB3	-0.5233169
EPHB4	-1.2394176
EPHB6	-1.0929375
EPHX1	-4.1515384
EPHX2	-6.693139
EPHX3	-0.8787463
EPM2A	-0.4429414
EPM2AIP1	-0.7702794
EPN1	-1.6463223
EPOR	-3.0292907
EPRS	-0.5930872
EPS8L2	-1.3169122
EPSTI1	-0.475158
EPT1	-2.4953275
EPX	-5.165098
EPYC	-1.1147618
ERAL1	-1.533812
ERAP1	-1.4988537
ERAS	-1.3848042
ERBB3	-0.638948
ERBB4	-0.3914983
ERC1	-0.2676895
ERC2	-0.4219623
ERCC3	-0.7836585
ERCC6	-0.5406802
ERCC6L	-0.6494923
ERCC8	-0.3781347
ERF	-1.3878708
ERGIC1	-1.7476344
ERGIC2	-0.7429791
ERGIC3	-1.5219827
ERI2	-1.7626448
ERI3	-1.7418344
ERICH4	-3.7028632
ERICH5	-1.7592294
ERICH6	-0.5839138
ERLEC1	-0.5477991
ERLIN1	-1.6955805
ERLIN2	-0.8300719
ERMARD	-1.531054
ERMP1	-1.8751826
ERO1LB	-1.6712012

ERP29	-0.5852227
ERP44	-0.5091801
ERRFI1	-1.2203689
ESCO2	-1.9488406
ESD	-1.4843183
ESP1	-2.6855862
ESPL1	-0.5661116
ESPN	-2.6426256
ESPNL	-0.5736437
ESR1	-3.532317
ESRP2	-2.835205
ESRRA	-1.3260336
ESRRB	-1.7988181
ESRRG	-2.9922233
ESX1	-1.3818119
ETAA1	-0.4197629
ETD	-1.2015824
ETFA	-2.975968
ETFB	-3.168311
ETFDH	-3.3171759
ETHE1	-3.582099
ETNK2	-5.8520164
ETNPPL	-7.21267
ETOHI1	-0.5937688
ETV2	-1.871361
EVA1A	-3.796195
EVC	-2.161037
EVI5	-2.4936085
EVX1	-1.0746248
EXD1	-1.6123517
EXD2	-1.6773622
EXO5	-0.765954
EXOC3L	-1.1920736
EXOC3L2	-3.835347
EXOC6	-0.5206928
EXOC7	-1.6346135
EXOC8	-0.2249427
EXOSC1	-0.4733861
EXOSC2	-1.1829243
EXOSC3	-0.5871949
EXOSC4	-1.934835
EXOSC5	-1.2343636
EXOSC6	-0.5601387
EXOSC7	-1.385005
EXOSC9	-0.988513

EXT1	-1.6728783
EXTL1	-3.4561698
EXTL2	-0.9967146
EYA1	-0.7831578
F10	-3.0428324
F11	-5.1155505
F11R	-0.1978254
F12	-5.388242
F13B	-7.733657
F2	-4.4143066
F2RL1	-1.0930209
F5	-5.6914816
F7	-3.2881508
F8	-5.1338806
F9	-4.541
FA2H	-0.956775
FAAH	-5.144146
FABP1	-6.258186
FABP2	-5.875896
FABP5	-2.2519536
FABP7	-4.077446
FABP9	-2.6016922
FADD	-1.3218951
FADDOS	-2.1236446
FADS1	-4.54781
FADS2	-5.844115
FADS6	-4.6405973
FAF1	-0.4358912
FAF2	-1.2019529
FAH	-5.123728
FAHD1	-4.3039923
FAHD2A	-3.0142965
FAM102A	-0.569169
FAM103A1	-0.4346252
FAM104A	-0.4920859
FAM107B	-0.5999594
FAM109A	-0.5349608
FAM110B	-0.4476636
FAM114A1	-1.2429948
FAM114A2	-1.3821168
FAM115E	-1.2797604
FAM118B	-0.2132092
FAM120A	-0.9757223
FAM120B	-0.5063639
FAM122A	-0.1969008

FAM122B	-0.4382916
FAM122C	-1.5925226
FAM124A	-2.5055134
FAM126A	-0.1517134
FAM131B	-1.2426269
FAM131C	-4.7147675
FAM134A	-2.8209672
FAM134C	-1.5101881
FAM135A	-0.3257885
FAM135B	-1.6335468
FAM136A	-2.6143236
FAM13A	-1.7235479
FAM149A	-1.2554145
FAM149B	-0.9720666
FAM151B	-0.8665593
FAM154A	-1.586868
FAM160B1	-1.012301
FAM160B2	-0.7685762
FAM162A	-1.5830612
FAM163A	-1.3651085
FAM163B	-0.9512734
FAM167B	-4.7389927
FAM168A	-0.6191592
FAM169B	-3.1394167
FAM170A	-1.0998354
FAM171A2	-1.0289767
FAM171B	-2.418204
FAM173A	-2.0922556
FAM173B	-2.225809
FAM175A	-0.4609258
FAM175B	-0.3000379
FAM180A	-3.288001
FAM181A	-1.3703117
FAM185A	-1.9704773
FAM187A	-2.2344153
FAM187B	-1.1251478
FAM188B2	-0.5219681
FAM192A	-0.4481506
FAM193A	-0.8961644
FAM193B	-0.9762554
FAM195A	-4.7548003
FAM198A	-3.3187218
FAM19A2	-6.4535904
FAM203A	-0.889988
FAM204A	-0.6633797

FAM207A	-1.1114821
FAM20A	-3.7787285
FAM20B	-0.7514386
FAM20C	-2.251018
FAM210A	-2.2402556
FAM210B	-2.449935
FAM212A	-1.3407192
FAM213A	-4.3982596
FAM213B	-3.1577907
FAM214A	-2.2523718
FAM219A	-1.1303258
FAM219AOS	-0.6588705
FAM221B	-1.3668528
FAM227A	-0.9410641
FAM228A	-2.1959689
FAM228B	-1.9536524
FAM229A	-1.2153811
FAM229B	-0.9561477
FAM24A	-1.1763892
FAM25C	-1.4493237
FAM26F	-0.4446554
FAM32A	-0.6890178
FAM35A	-3.2455108
FAM3A	-1.1339993
FAM3C	-0.761507
FAM45A	-0.7232809
FAM46A	-1.324966
FAM46D	-1.5151916
FAM47E	-1.9908981
FAM53A	-0.908205
FAM57B	-1.7261751
FAM58B	-1.0967698
FAM63A	-0.9089017
FAM64A	-1.2775631
FAM65C	-1.8467336
FAM69A	-0.8434296
FAM69C	-1.1300559
FAM71A	-1.0615413
FAM71F1	-1.7790697
FAM72A	-0.9565644
FAM73B	-2.941153
FAM78A	-0.5106928
FAM83A	-1.0497749
FAM83D	-1.9987054
FAM83F	-3.1771052

FAM83G	-0.1548972
FAM83H	-0.8930483
FAM84B	-0.607806
FAM86	-1.3977885
FAM89A	-3.422134
FAM89B	-0.427248
FAM92A	-1.2115865
FAM96A	-1.3177977
FAM98C	-0.628685
FAN1	-1.4857757
FANCB	-1.5516555
FANCC	-0.8762817
FANCD2OS	-1.7734478
FANCE	-0.669013
FANCF	-1.273088
FANCI	-1.0108829
FANCL	-0.5079165
FANCM	-0.9730296
FAR2OS2	-1.5600989
FARP2	-1.8955584
FARS2	-2.188764
FARSA	-0.2801628
FARSB	-0.523355
FASN	-3.0521584
FASTK	-1.2778869
FASTKD1	-1.8428836
FASTKD2	-1.6837192
FASTKD3	-0.7308898
FASTKD5	-1.1748967
FAU	-0.6819763
FBLL1	-2.6878881
FBLN2	-0.5527549
FBLN5	-1.0263925
FBLN7	-3.3460329
FBP1	-3.1940546
FBXL12OS	-0.6042519
FBXL15	-1.1096535
FBXL17	-1.3096747
FBXL19	-0.1482282
FBXL21	-2.02851
FBXL4	-1.6311679
FBXL6	-1.3825698
FBXL8	-0.8965249
FBXO16	-0.858017
FBXO18	-0.871942

FBXO21	-3.3941884
FBXO22	-1.3649073
FBXO3	-1.7570834
FBXO31	-0.264658
FBXO34	-0.5941491
FBXO36	-0.6021366
FBXO43	-3.1999173
FBXO47	-0.9208996
FBXO48	-1.2127604
FBXO6	-0.6898885
FBXO7	-0.8752604
FBXO8	-2.1299257
FBXO9	-1.5276675
FBXW14	-1.2190995
FBXW18	-1.3561761
FBXW19	-2.6940436
FBXW2	-0.3278179
FBXW21	-1.2366271
FBXW22	-1.8081722
FBXW24	-1.687417
FBXW26	-4.3476543
FBXW27	-1.4825456
FBXW4	-0.4912462
FBXW5	-1.2251053
FBXW9	-0.6785865
FCF1	-0.4279623
FCGR2B	-1.260087
FCGRT	-3.6030898
FCHSD2	-0.5011511
FCNA	-5.4442143
FCNAOS	-3.9188557
FCNB	-0.8075862
FCRL5	-0.8690748
FCRL6	-1.5598526
FDFT1	-3.0411644
FDPS	-4.4128346
FDX1	-1.5746131
FDX1L	-1.7170663
FDXACB1	-1.7513151
FDXR	-2.6775818
FECH	-2.6461115
FEM1A	-1.3353686
FER1L5	-1.610817
FER1L6	-1.0189154
FERD3L	-3.7859101

FERMT2	-0.5153585
FERT2	-0.3487864
FETUB	-4.6407313
FEZ1	-1.3916831
FFAR1	-1.6043756
FGA	-1.4732561
FGB	-1.5195751
FGD1	-1.3019383
FGD2	-2.467608
FGD5	-0.398181
FGD6	-2.1798599
FGF1	-2.2257223
FGF14	-2.0012412
FGF16	-1.3012953
FGF20	-2.1978831
FGF21	-3.0229354
FGF22	-1.3221428
FGF23	-0.4538984
FGF5	-1.6998522
FGF8	-2.2290084
FGF9	-0.9214959
FGFBP3	-0.1662636
FGFR1OP	-0.3339143
FGFR3	-0.6489692
FGFR4	-4.6400337
FGFRL1	-2.0571132
FGG	-1.2742682
FGGY	-6.043668
FGL1	-2.5163727
FH1	-1.8336496
FHIT	-3.028653
FHL2	-0.421083
FIBP	-1.1395297
FICD	-0.4647584
FIG4	-0.8482175
FIGLA	-1.5836306
FIGNL1	-0.3962398
FITM1	-1.8646545
FITM2	-2.0852528
FIZ1	-0.8219638
FKBP11	-1.0834167
FKBP15	-0.2141233
FKBP1A	-0.9261928
FKBP2	-1.2960782
FKBP3	-1.000947

FKBP4	-2.8422608
FKBP8	-2.845377
FKBP9	-0.7835655
FKBPL	-0.5989842
FLAD1	-2.6526046
FLCN	-0.5445676
FLG	-1.0523787
FLOT2	-0.1692901
FLRT1	-3.233698
FLT3L	-1.0440121
FLT4	-1.234447
FLYWCH2	-0.196259
FMN2	-1.5184574
FMO1	-3.5498676
FMO3	-4.3943563
FMO4	-3.2858157
FMO5	-6.040477
FMO6	-1.3339462
FMO9	-1.1040506
FMOD	-0.9434404
FMR1NB	-1.9268718
FN1	-2.0573835
FN3K	-5.3502855
FNDC3A	-0.7033148
FNDC3C1	-2.069306
FNDC4	-3.208181
FNIP2	-0.4626613
FNTA	-0.5064497
FOCAD	-1.3809195
FOLR1	-3.5613658
FOLR2	-4.2402496
FOLR4	-0.3922744
FOPNL	-1.1309352
FOXA2	-1.297823
FOXA3	-4.07135
FOXB2	-2.1801586
FOXD3	-0.9489262
FOXE1	-2.132081
FOXG1	-0.4337783
FOXH1	-0.7413189
FOXI1	-1.3855
FOXI3	-0.3649712
FOXK2	-0.4858389
FOXL2	-1.2860212
FOXL2OS	-1.8233788

FOXM1	-0.9539647
FOXO1	-0.3573446
FOXO4	-0.4490502
FOXP2	-1.1204913
FOXQ1	-0.6250501
FOXR2	-1.703207
FOXRED1	-1.3285642
FPGS	-4.0051246
FPGT	-1.2594154
FPR-RS3	-0.9897323
FRA10AC1	-1.5712919
FRAT1	-2.0167007
FREM3	-2.2162228
FRMD3	-1.9656126
FRMD6	-1.0461819
FRMD8OS	-1.6476228
FRMPD3	-1.7502801
FRRS1	-1.0683033
FRRS1L	-1.9404466
FRZB	-1.5794384
FSBP	-1.7909324
FSCB	-2.8298025
FSCN2	-1.7048025
FSHR	-1.6355007
FSIP2	-1.5958276
FST	-0.4254642
FTCD	-5.7997036
FTH1	-0.9963331
FTL1	-1.715662
FTMT	-1.0952153
FTO	-1.1114745
FTSJ1	-0.7113223
FTSJ2	-2.4816086
FUCA1	-1.5571175
FUK	-1.3406434
FUOM	-2.7789235
FURIN	-1.0936723
FUT10	-1.062293
FUT4-PS1	-1.7643471
FUT7	-1.3281136
FUZ	-1.2273004
FXN	-2.8823848
FXYD1	-4.4653015
FXYD7	-2.518672
FYCO1	-1.3164363

FZD7	-0.9955332
FZD8	-1.6482596
FZR1	-0.6583705
G0S2	-2.6327467
G3BP2	-0.3615136
G6PC	-4.4774303
GAA	-1.3239446
GABARAP	-0.2898688
GABARAPL1	-1.2280927
GABPA	-0.4494953
GABRA1	-2.5649028
GABRB1	-2.8690438
GABRD	-1.7768359
GABRG3	-0.6700583
GABRR1	-4.100524
GABRR3	-1.7019258
GAD1	-0.8900561
GAD1OS	-1.4950924
GAD2	-1.714174
GADD45GIP1	-2.0062242
GADL1	-1.2875922
GAK	-0.5470867
GAL3ST1	-0.7522759
GAL3ST2	-1.467262
GAL3ST4	-1.5990748
GALE	-2.0613284
GALK1	-2.863552
GALK2	-1.5546904
GALM	-5.384798
GALNS	-2.364859
GALNT10	-1.4628348
GALNT11	-0.6272855
GALNT14	-2.9122276
GALNT2	-0.3461351
GALNT4	-0.4056034
GALNT5	-1.2073107
GALNT7	-0.3735676
GALNTL5	-4.467829
GALR3	-1.7632475
GALT	-0.9956985
GAMT	-8.554161
GANAB	-1.7550058
GANC	-1.5560789
GAPDH	-1.472117
GAPDHS	-1.1923294

GAREM	-2.2347858
GART	-0.4769282
GAS1	-1.0379918
GAS2	-5.14307
GAS2L2	-0.6592557
GAS2L3	-0.604106
GAS5	-0.181181
GAS6	-0.8595877
GAST	-0.981596
GATA4	-4.229659
GATA5	-1.0171101
GATA6	-0.2951098
GATAD1	-1.0999217
GATC	-2.0552533
GATM	-0.6657348
GATSL3	-0.9365711
GBE1	-2.6630359
GBF1	-0.7945194
GBP11	-3.1710477
GBP2B	-2.4267793
GBP8	-1.8870201
GBX1	-1.1637156
GBX2	-1.4428182
GC	-3.8623147
GCAT	-5.143148
GCC2	-0.5330596
GCDH	-5.3576355
GCG	-1.3999543
GCGR	-6.6091914
GCH1	-1.5616946
GCHFR	-6.3558917
GCK	-6.351818
GCKR	-6.960576
GCLC	-0.837904
GCLM	-2.2192836
GCM1	-0.6612358
GCM2	-0.9458129
GCN1L1	-1.065268
GCNT4	-1.6083744
GCNT7	-1.116127
GCSH	-3.1969414
GDAP1	-1.5211604
GDAP1L1	-0.5497141
GDAP2	-0.6159611
GDAP5	-1.1719007

GDF1	-0.9994443
GDF10	-5.211733
GDF11	-1.4623759
GDF15	-0.9022012
GDF2	-5.944562
GDF3	-0.8505311
GDF7	-1.3816025
GDI2	-0.7237186
GDPD4	-2.1273396
GDPGP1	-1.4731925
GEMIN2	-2.425114
GEMIN4	-0.9497838
GEMIN5	-0.8319593
GEMIN6	-1.6151559
GEMIN7	-0.658318
GEN1	-1.0900819
GFER	-2.829823
GFM1	-1.6093154
GFM2	-2.2594981
GFOD1	-1.5545833
GFOD2	-0.584794
GFRA1	-3.2744179
GFRA2	-1.6070533
GFRA3	-0.9003928
GGA1	-1.1312366
GGA2	-1.6149483
GGACT	-2.9702878
GGCT	-0.9499393
GGCX	-3.5661116
GGH	-0.6410565
GGNBP1	-3.5509143
GGNBP2	-0.503643
GGT6	-3.7537503
GH	-1.6552255
GHDC	-1.8242869
GHITM	-1.3377032
GHR	-3.8088017
GHRH	-0.3242159
GID4	-0.8136888
GID8	-0.7459965
GIGYF1	-0.3478661
GIGYF2	-0.5013967
GIMAP7	-0.6969776
GIMAP9	-1.1194062
GIN1	-1.0004323

GINS1	-0.811779
GINS3	-0.2345076
GINS4	-1.0517926
GIPC1	-0.3954845
GIT2	-0.3539205
GJA10	-1.2833071
GJA6	-2.3554566
GJA8	-1.7330523
GJB1	-6.841746
GJB2	-2.8431315
GJB5	-3.4841769
GJD2	-2.4499884
GJD3	-1.2778559
GJD4	-1.5875206
GKAP1	-1.1833034
GKN1	-1.1683097
GLB1	-1.4091511
GLCE	-2.120829
GLDC	-7.2993336
GLDN	-6.542614
GLE1	-0.5385642
GLIPR1L1	-0.982805
GLIS1	-0.8184512
GLMN	-1.0978146
GLO1	-3.510054
GLOD4	-1.581078
GLOD5	-1.9042487
GLP2R	-0.8898838
GLRA1	-1.7249682
GLRA3	-1.6298637
GLRA4	-1.5213642
GLRB	-1.6660311
GLRX	-0.9481192
GLRX2	-0.8527803
GLRX3	-1.1180587
GLRX5	-2.3326516
GLS2	-6.653305
GLT1D1	-2.1448476
GLT25D1	-0.6882014
GLT28D2	-0.9989915
GLT6D1	-1.6579177
GLT8D1	-0.268219
GLT8D2	-0.8446915
GLTPD1	-1.8989234
GLTPD2	-6.0876694

GLUD1	-3.255589
GLUL	-1.4490991
GLYAT	-6.418599
GLYATL3	-1.3994579
GLYCTK	-6.3550835
GLYR1	-0.940702
GM10007	-1.2438376
GM10015	-0.3366089
GM10023	-1.7025883
GM10030	-2.3506694
GM10034	-0.7963753
GM10057	-1.6163054
GM10094	-0.4682679
GM10101	-0.3188646
GM10113	-2.8463097
GM10134	-1.1001081
GM10176	-1.7087591
GM10190	-1.0179851
GM10209	-3.0282571
GM10220	-2.8935027
GM10229	-1.1538639
GM10252	-1.8134725
GM10266	-1.7226679
GM10272	-0.6916535
GM10276	-1.8762097
GM10277	-1.8746207
GM10286	-1.178549
GM10287	-1.7735474
GM10319	-4.305503
GM10367	-3.1503038
GM10370	-0.7406128
GM10372	-0.8191402
GM10375	-2.053298
GM10387	-1.843178
GM10388	-0.97577
GM10405	-0.8969302
GM10408	-1.0130224
GM10419	-1.0747609
GM10420	-0.8502913
GM10432	-1.0969145
GM10433	-2.7654707
GM10437	-1.4795938
GM10441	-1.5893536
GM10445	-1.7781186
GM10452	-0.3127537

GM10466	-2.3492322
GM10469	-1.7733135
GM10475	-1.1332288
GM10482	-1.1937497
GM10498	-2.133174
GM10501	-1.9185426
GM10509	-0.8876202
GM10513	-1.124646
GM10516	-1.9313927
GM10523	-0.5708022
GM10536	-2.5449529
GM10538	-1.115844
GM10548	-1.3785512
GM10549	-1.6304328
GM10555	-1.6683798
GM10556	-3.685651
GM10563	-1.3945682
GM10568	-1.5580204
GM10578	-1.7439759
GM10588	-1.4908419
GM10589	-1.1864758
GM10603	-2.8677497
GM10609	-1.1185558
GM10614	-1.5022511
GM10615	-0.5467365
GM10616	-1.5649667
GM10618	-1.165072
GM10624	-1.3098454
GM10627	-1.7358172
GM10632	-1.2654371
GM10639	-4.598691
GM10640	-1.167855
GM10651	-0.9953139
GM10653	-0.9266667
GM10658	-5.0949717
GM10680	-3.3464475
GM10682	-1.1433368
GM10683	-1.7132416
GM10687	-0.5083802
GM10696	-0.8392189
GM10697	-0.4541223
GM10703	-1.1502838
GM10706	-1.36339
GM10714	-2.3232768
GM10727	-1.6257253

GM10730	-1.686266
GM10733	-1.5936074
GM10734	-2.247904
GM10739	-1.1903737
GM10743	-1.6024935
GM10744	-3.1417716
GM10747	-1.9541862
GM10752	-4.6696243
GM10753	-1.4483676
GM10766	-0.9319294
GM10767	-0.4981933
GM10768	-7.0040865
GM10776	-2.302558
GM10777	-0.8522594
GM10778	-0.6278787
GM10779	-1.9776928
GM10782	-1.2022705
GM10785	-0.960175
GM10790	-3.4902246
GM10802	-0.6375463
GM10814	-2.388242
GM10815	-1.7552547
GM10822	-1.6091075
GM10824	-2.625926
GM10825	-1.6720455
GM10827	-4.094446
GM10830	-2.1017585
GM10836	-2.8721268
GM10845	-0.4639392
GM10854	-1.5940132
GM10863	-1.260381
GM10864	-1.0506368
GM10865	-0.855654
GM10867	-1.9339743
GM10869	-2.505111
GM10871	-0.9476225
GM10873	-1.881886
GM10910	-1.6114192
GM10914	-0.7884679
GM11033	-2.4220967
GM11054	-1.0712953
GM1110	-0.9837091
GM11127	-1.9467306
GM11128	-0.8870156
GM11159	-1.6860144

GM11223	-0.8116241
GM1123	-0.207262
GM11237	-1.5326478
GM11240	-3.0403357
GM11266	-1.6157548
GM11292	-1.591897
GM11381	-0.8986175
GM1140	-2.2042375
GM1141	-1.7527318
GM11437	-6.401659
GM11492	-2.0441206
GM11497	-1.2354484
GM11529	-1.630224
GM11541	-2.5430984
GM11544	-1.341054
GM11545	-2.2511258
GM11546	-2.56572
GM11570	-1.5442519
GM11595	-1.1800416
GM11634	-0.4357905
GM11677	-0.6843269
GM11710	-2.7774396
GM11780	-1.7426319
GM11783	-1.5433497
GM11810	-0.4549623
GM11937	-1.3257465
GM11981	-1.2843297
GM12000	-2.7327754
GM12059	-2.347122
GM12060	-0.5097508
GM12070	-1.5591516
GM12171	-1.1041243
GM12247	-1.8130627
GM12258	-0.9697199
GM12273	-0.8902838
GM12298	-0.9098051
GM12302	-2.5382028
GM12447	-0.672935
GM12474	-1.3139815
GM12503	-2.4107366
GM12508	-0.1398702
GM12511	-1.4796715
GM12522	-0.8292074
GM12542	-2.990763
GM12568	-0.5733154

GM12569	-1.1376595
GM12618	-0.3318811
GM12620	-0.979384
GM12689	-0.7144795
GM12693	-0.6873283
GM12703	-4.230812
GM12758	-1.9443662
GM12794	-1.6375444
GM12816	-0.2782211
GM12830	-1.6184247
GM12863	-1.1937749
GM12886	-1.6156683
GM12888	-1.6277206
GM12942	-1.918519
GM12992	-1.6709442
GM13011	-1.6899028
GM13023	-2.1522722
GM13032	-1.3618066
GM13051	-3.2121682
GM13084	-1.9716215
GM13089	-3.3244665
GM13111	-3.2975032
GM13112	-1.6656075
GM13119	-1.8849583
GM13124	-1.6164858
GM13125	-1.7523272
GM13128	-3.2575126
GM13177	-1.5241756
GM13180	-1.0766525
GM13194	-1.2101212
GM13202	-1.8831868
GM13205	-1.932673
GM13219	-1.3251455
GM1322	-2.8763187
GM13315	-2.2640524
GM13403	-0.875113
GM13420	-1.8449051
GM13498	-1.0380611
GM13522	-2.9595022
GM13582	-2.2636302
GM13629	-1.0419209
GM13710	-1.2524557
GM13769	-1.2705343
GM13770	-2.1032357
GM13777	-0.6033444

GM13807	-3.7105353
GM13826	-0.4489994
GM14015	-4.4548454
GM14025	-0.9537473
GM14129	-1.6325192
GM14149	-1.669308
GM14151	-3.3527822
GM14207	-0.8691156
GM14288	-1.5989761
GM14305	-0.8519046
GM14308	-0.6496065
GM14322	-0.9008617
GM14325	-1.4612889
GM14326	-0.874867
GM14340	-2.4617336
GM14347	-1.6619129
GM14374	-1.6389749
GM14391	-0.8940988
GM14393	-1.0097356
GM14410	-0.8278132
GM14431	-0.7596226
GM14432	-1.4194169
GM14440	-1.0293202
GM14443	-0.9591618
GM14484	-2.3162851
GM14486	-0.8924215
GM14496	-4.1200247
GM14499	-0.2934132
GM14703	-1.1661272
GM14725	-1.5993116
GM14851	-2.1022177
GM15023	-2.1398149
GM15032	-0.2336869
GM15104	-1.3967185
GM15232	-0.626051
GM1527	-1.4203827
GM15292	-1.1855931
GM15386	-1.7547829
GM15401	-1.815171
GM15411	-4.5745115
GM15412	-1.3609686
GM15417	-0.8994894
GM15441	-1.0242813
GM15478	-1.6644921
GM15482	-1.4395945

GM15498	-1.6542299
GM15514	-2.6149325
GM1564	-2.201036
GM15688	-1.6248393
GM15698	-0.2807341
GM15706	-0.2485285
GM15762	-2.5656285
GM15772	-0.6704712
GM15800	-0.6049228
GM15821	-0.9513314
GM16039	-1.9746192
GM1604B	-2.9694216
GM16062	-1.1128039
GM16223	-1.8007708
GM16287	-0.5230329
GM16381	-0.8763075
GM16386	-1.0510058
GM16428	-0.929425
GM16441	-0.9946613
GM16442	-1.3229153
GM16462	-0.806613
GM16489	-1.9826787
GM16491	-1.3107648
GM16497	-2.4419909
GM16498	-1.9683292
GM16499	-2.0044017
GM16525	-1.5991664
GM16527	-1.7244625
GM16532	-1.5823276
GM16853	-0.1685257
GM1698	-2.4605465
GM17359	-1.600565
GM17727	-2.6489594
GM17745	-2.914907
GM17748	-0.7716799
GM17753	-1.3822935
GM17764	-1.0100927
GM17783	-1.341054
GM19410	-2.0119817
GM1943	-1.2164695
GM1965	-1.3039088
GM19668	-1.4599872
GM1968	-0.6544592
GM1971	-1.3929343
GM20056	-1.0487185

GM2011	-1.5255256
GM2016	-0.3364525
GM2027	-1.2428095
GM20554	-1.3802576
GM20740	-1.5585389
GM20746	-2.22613
GM20747	-1.8714767
GM20754	-1.6582823
GM20755	-1.6072018
GM20764	-0.2252579
GM20837	-2.2520275
GM2085	-3.156877
GM20865	-1.1519866
GM20867	-1.6705976
GM20877	-1.9628332
GM2090	-1.4840801
GM20918	-1.3412418
GM20939	-1.6745415
GM2109	-1.6631644
GM21292	-1.1722989
GM2137	-1.3132513
GM2138	-2.2251318
GM21404	-1.981329
GM21464	-1.8111303
GM21498	-1.5299094
GM21559	-0.5768337
GM21637	-0.8593431
GM2164	-1.6000834
GM2176	-4.0730724
GM2238	-0.8877864
GM2240	-0.4966383
GM2287	-1.5056224
GM2347	-0.4982007
GM2382	-1.6139421
GM2436	-0.8595834
GM2464	-1.7421014
GM2483	-1.6582592
GM2491	-1.5857844
GM2516	-3.0357158
GM2582	-1.2331543
GM2670	-1.6413896
GM2800	-2.1352537
GM2814	-2.1616507
GM2933	-4.4431686
GM2990	-1.086302

GM2A	-2.84899
GM3032	-1.0992692
GM3045	-1.0469837
GM3052	-1.5098317
GM3134	-1.7861273
GM3143	-1.7453148
GM3146	-1.2252707
GM3219	-4.6317377
GM3227	-1.4930551
GM324	-1.3780842
GM3258	-0.8013802
GM3285	-1.1803763
GM3289	-2.6454868
GM3300	-0.6537733
GM3336	-0.9782658
GM3448	-0.6604271
GM3455	-1.1587896
GM3470	-0.9581623
GM3474	-1.6025033
GM3509	-1.2816067
GM3519	-1.5164361
GM355	-2.9305034
GM3561	-0.4333229
GM3625	-0.4708781
GM3646	-1.6867304
GM3669	-1.1127181
GM3696	-0.5755608
GM3704	-3.225829
GM3716	-1.3834293
GM3740	-1.0819798
GM3763	-1.7345364
GM3775	-1.5949731
GM3776	-4.3012958
GM3807	-0.7666764
GM3830	-4.180171
GM3853	-1.2563167
GM3858	-0.6963494
GM3896	-1.0320127
GM3907	-1.1605587
GM3942	-1.1980367
GM3948	-1.6731668
GM3960	-1.0725482
GM3970	-1.1291735
GM3985	-1.6594496
GM4013	-2.3778152

GM4035	-0.6204228
GM4081	-1.0816596
GM4098	-1.5840211
GM4117	-1.8169193
GM4130	-0.6879754
GM4131	-3.4234538
GM4201	-2.2512007
GM4230	-0.9254956
GM4241	-1.2847421
GM4279	-1.721945
GM4285	-1.3866186
GM4297	-1.7517974
GM4303	-2.5982938
GM4311	-0.3692362
GM4312	-1.3824549
GM4342	-0.6372156
GM436	-0.9845123
GM4371	-1.4877591
GM438	-0.6737037
GM44	-1.5497115
GM4402	-2.4577856
GM4419	-2.400971
GM4432	-1.218102
GM4466	-1.738888
GM4489	-3.1954668
GM4532	-0.8976405
GM4544	-1.7274487
GM4567	-2.3248749
GM4606	-1.0175383
GM4632	-1.587285
GM4633	-1.2682967
GM4657	-1.3136835
GM4665	-1.4770207
GM4668	-2.2084508
GM4710	-1.6220992
GM4719	-1.1384459
GM4736	-1.1989858
GM4745	-1.8040524
GM4758	-1.8763025
GM4763	-2.2211282
GM4786	-0.9517407
GM4787	-2.054199
GM4788	-4.0430126
GM4791	-1.3977478
GM4792	-1.2503228

GM4794	-5.236724
GM4802	-1.0094738
GM4814	-1.1335616
GM4832	-0.282187
GM4858	-1.5354533
GM4868	-1.8266559
GM4871	-0.387579
GM4876	-2.8102076
GM4881	-1.553987
GM4889	-0.3903289
GM4890	-1.8917589
GM4906	-1.590163
GM4922	-1.5446906
GM4924	-0.8906293
GM4925	-2.1611972
GM4943	-1.909369
GM4944	-0.2590747
GM4951	-1.2562904
GM4952	-9.853824
GM4963	-0.463295
GM4971	-0.9350929
GM4997	-0.4637365
GM5	-0.9395564
GM5039	-1.1048577
GM5069	-1.7896662
GM5079	-1.4979196
GM5083	-1.5715921
GM5084	-1.037457
GM5089	-2.1609066
GM5091	-1.6638365
GM5093	-0.7068977
GM5099	-1.6733878
GM5112	-1.6959071
GM5113	-0.7717717
GM5114	-1.1192324
GM5118	-1.4941607
GM5122	-2.501839
GM5124	-1.3471966
GM5126	-2.8145006
GM5127	-1.5479579
GM5132	-2.159629
GM5134	-1.763912
GM5155	-1.2185252
GM5174	-1.808794
GM525	-2.0009391

GM5296	-0.779424
GM53	-1.6600356
GM5331	-2.0850677
GM5334	-1.136276
GM5382	-2.7831411
GM5420	-1.2604544
GM5441	-0.6397126
GM5445	-0.4596014
GM5447	-1.8249335
GM5453	-0.5542722
GM5468	-1.6405456
GM5475	-1.2907474
GM5478	-2.23119
GM5481	-0.3600311
GM5523	-1.2586069
GM5524	-8.525318
GM5526	-0.5403876
GM5528	-0.4469905
GM553	-1.7398198
GM5531	-3.248255
GM5533	-2.5397766
GM5544	-1.0285244
GM5553	-1.2911386
GM5570	-0.8706832
GM5585	-3.4730408
GM5590	-0.7009144
GM5591	-1.8069773
GM5593	-1.0401673
GM5607	-0.7770901
GM561	-1.699336
GM5617	-2.4040637
GM5622	-3.111927
GM5712	-1.9891639
GM572	-1.0389242
GM5741	-1.5776813
GM5766	-0.9150543
GM5767	-1.1435885
GM5771	-2.2996469
GM5779	-0.8372378
GM5784	-1.2477269
GM5800	-1.7219081
GM5801	-0.5702877
GM5848	-1.1074286
GM5849	-1.8487167
GM5860	-0.4421687

GM5879	-0.4260187
GM5893	-1.1062636
GM5907	-1.6450405
GM5908	-0.9036017
GM5916	-1.2100067
GM5922	-1.5927198
GM5925	-0.7998476
GM5932	-2.0991013
GM595	-4.1368227
GM597	-0.8230383
GM6042	-1.0587716
GM6093	-1.1207752
GM6109	-0.5116434
GM6135	-6.591322
GM6145	-1.9628584
GM6150	-1.6953933
GM6155	-0.8127298
GM6195	-0.5691223
GM6209	-0.6977999
GM6219	-1.5084713
GM6238	-1.2888117
GM626	-0.9346294
GM6260	-1.6563301
GM6277	-1.0341306
GM6280	-1.9458618
GM6297	-2.6980865
GM6306	-0.8431432
GM6307	-1.8749354
GM6360	-1.2755425
GM6370	-1.4303029
GM6404	-0.425818
GM6406	-1.6306059
GM6416	-1.5542135
GM6436	-0.9504039
GM6444	-1.989564
GM6471	-1.7469015
GM6484	-8.037497
GM6498	-1.7759852
GM6521	-1.1604114
GM6525	-0.5414543
GM6537	-1.8479772
GM6548	-1.4300256
GM6556	-0.6160264
GM6559	-1.6662333
GM6566	-1.4035449

GM6578	-0.649066
GM6598	-1.941098
GM6607	-0.2563114
GM6614	-4.100055
GM6634	-1.7543948
GM6639	-1.352067
GM6653	-1.5947902
GM6658	-1.6503973
GM6687	-1.6845834
GM6710	-0.6513133
GM6713	-1.1554191
GM6787	-1.0418499
GM6818	-1.9220467
GM6867	-0.7396123
GM6878	-1.5966749
GM6880	-1.77071
GM6921	-1.5042825
GM6927	-1.9460435
GM694	-1.7991035
GM6943	-1.4956179
GM6961	-1.7341104
GM6980	-1.5939844
GM6981	-1.8439837
GM6987	-1.7529154
GM6994	-1.1225953
GM7008	-0.9437935
GM7104	-1.0705116
GM7120	-2.542306
GM7133	-2.25421
GM7134	-1.0207701
GM7160	-0.7875209
GM7241	-0.762888
GM7256	-2.3919935
GM7257	-1.3112183
GM7276	-1.7160683
GM732	-1.2034404
GM7325	-1.2138786
GM7457	-0.9929102
GM7467	-1.4127934
GM7534	-2.353151
GM7538	-2.3312898
GM7546	-2.1927605
GM7550	-1.1981988
GM7555	-0.5053358
GM7562	-1.6438298

GM7596	-1.3998754
GM7616	-2.7582066
GM7628	-2.47119
GM7634	-0.4863362
GM765	-1.6063163
GM766	-2.474191
GM7664	-0.7159247
GM7694	-0.2257903
GM7789	-3.184111
GM7849	-1.2599435
GM7854	-2.983485
GM7897	-2.1933222
GM7903	-1.4823349
GM7936	-1.8965497
GM7972	-2.3847039
GM7978	-1.6282802
GM805	-1.2116127
GM8094	-0.8970451
GM8149	-1.5104544
GM815	-1.7319751
GM8165	-1.5608888
GM8210	-0.3378563
GM8221	-4.588304
GM826	-3.640059
GM8479	-1.5538237
GM8493	-0.8949373
GM8505	-2.6775198
GM8526	-0.5537982
GM8541	-1.63444
GM8587	-0.8970521
GM8630	-0.4149978
GM8633	-1.1837819
GM8672	-1.6071432
GM8674	-1.650917
GM8700	-1.1228194
GM8709	-1.8212113
GM8738	-3.7377076
GM8765	-1.4724908
GM8801	-0.5945478
GM8817	-1.3404276
GM8883	-3.8068805
GM8909	-0.7024093
GM8994	-0.7790871
GM904	-1.2142344
GM9054	-1.504102

GM9079	-0.3750386
GM9112	-1.6706715
GM9182	-0.3420515
GM9199	-2.7647126
GM9234	-1.1212368
GM9325	-1.7449121
GM9376	-1.7384646
GM9385	-1.0001197
GM9457	-0.611454
GM9558	-1.0686002
GM9564	-1.171627
GM960	-1.3913531
GM973	-1.7754803
GM9731	-0.6858382
GM9734	-0.9776211
GM9752	-1.6330483
GM9753	-1.1255825
GM9801	-1.5930166
GM9804	-1.8353918
GM9833	-2.9172847
GM9860	-1.6263299
GM9861	-0.9553738
GM9869	-1.5405407
GM9871	-1.4401503
GM9872	-1.7305648
GM9876	-0.6472957
GM9878	-0.9109154
GM9883	-1.070615
GM9895	-1.6654027
GM9903	-1.0531442
GM9909	-3.8507566
GM9916	-2.6212902
GM9928	-1.0584254
GM9934	-1.6170814
GM9951	-1.2199574
GM9958	-1.6671839
GM9959	-1.6520379
GM9962	-1.7075984
GM9963	-0.9413855
GM9973	-2.058695
GM9992	-0.7141933
GMCL1	-0.9212947
GMEB2	-0.4250853
GML	-1.310391
GMNN	-1.206173

GMPPA	-0.9868221
GMPPB	-0.5002031
GMPR2	-0.9517479
GMPS	-0.3515389
GNA11	-1.9347
GNA12	-1.5865145
GNA15	-0.4865205
GNAI3	-0.2421627
GNAS	-0.8158627
GNAT1	-1.9894259
GNAT2	-3.5630262
GNB2L1	-0.5910244
GNE	-3.1346169
GNG11	-0.6786823
GNG5	-0.5788388
GNG7	-1.6523821
GNG8	-1.9818573
GNGT1	-4.682358
GNMT	-7.137781
GNPAT	-0.4068847
GNPDA1	-1.6273246
GNPNAT1	-2.1141477
GNPTG	-1.3893471
GNRHR	-2.0958936
GNS	-0.7470107
GOLGA1	-0.514298
GOLGA7	-0.5257053
GOLGA7B	-1.033864
GOLIM4	-1.8513637
GOLT1A	-2.5698872
GOPC	-0.613009
GORASP1	-2.1073036
GORASP2	-0.8465858
GOSR1	-0.8927999
GOSR2	-0.6133537
GOT1	-2.2026663
GOT1L1	-1.4417048
GOT2	-3.4518294
GPAA1	-0.7826619
GPAM	-2.9337378
GPAT2	-2.3273435
GPATCH1	-0.3540897
GPATCH2	-1.0496998
GPBP1L1	-0.5966415
GPC2	-1.3900204

GPC4	-1.9775608
GPC5	-1.2057157
GPC6	-2.1673627
GPD1	-4.6544228
GPD1L	-1.8916163
GPD2	-1.6776929
GPHN	-1.7634053
GPI1	-1.1809273
GPIHBP1	-0.5308719
GPLD1	-3.6659224
GPN2	-0.8563032
GPN3	-0.8116298
GPR1	-1.2990122
GPR101	-1.2658765
GPR107	-0.2986102
GPR108	-0.4499431
GPR110	-2.9677222
GPR111	-1.5987217
GPR12	-1.7836714
GPR125	-1.5405273
GPR128	-1.5752017
GPR135	-2.717149
GPR137	-0.8729367
GPR137C	-0.8864861
GPR142	-2.1773777
GPR143	-2.4609091
GPR150	-1.6352136
GPR155	-1.6472573
GPR157	-1.0702672
GPR162	-0.2777124
GPR17	-1.2217383
GPR173	-2.0610313
GPR182	-2.075799
GPR19	-1.3439393
GPR22	-0.9998651
GPR37L1	-1.3127964
GPR55	-1.3897433
GPR63	-1.6129673
GPR64	-0.485147
GPR75	-2.218131
GPR83	-1.626195
GPR89	-2.1954963
GPR98	-2.0003157
GPRASP2	-0.1749311
GPRC5C	-3.9511538

GPRC5D	-1.4860299
GPRIN2	-1.2439775
GPRIN3	-1.5518801
GPS1	-1.209671
GPSM2	-2.150826
GPT	-5.9803586
GPT2	-4.3514013
GPX1	-3.1915011
GPX4	-1.8793163
GPX7	-0.9355533
GRAMD1C	-2.6528616
GRAMD3	-0.8998752
GRB14	-3.356082
GRB7	-3.274588
GRCC10	-2.0500054
GREB1L	-3.7918277
GRHPR	-3.934627
GRIA1	-0.5618253
GRIA2	-1.701071
GRIA4	-0.991467
GRID1	-2.093493
GRID2	-1.6290708
GRIK1	-2.030203
GRIK2	-1.8136837
GRIK5	-1.4318838
GRIN1	-2.0906792
GRIN2A	-1.6231942
GRIN3B	-3.0428324
GRINA	-0.8322353
GRIP1OS1	-1.4999144
GRK1	-1.7148652
GRM1	-1.8196871
GRM2	-1.5633683
GRM5	-0.6052289
GRM8	-2.4173045
GRN	-1.6967788
GRPEL1	-2.6567812
GRPEL2	-1.0272775
GRSF1	-1.7435231
GRTP1	-4.1649556
GRWD1	-0.7319713
GRXCR1	-1.4944413
GRXCR2	-1.5915155
GSAP	-0.3586192
GSC	-0.8823986

GSDMA2	-1.2592044
GSDMA3	-1.1053815
GSDMCL-PS	-1.4752738
GSDMD	-0.9800639
GSG1L	-2.1220016
GSKIP	-0.7292719
GSPT1	-1.0094461
GSR	-0.4396124
GSS	-3.127996
GSTA2	-5.0098
GSTA3	-4.261742
GSTA4	-1.4057736
GSTK1	-2.7576385
GSTM1	-4.2401977
GSTM2	-2.9175243
GSTM3	-5.756903
GSTM4	-4.556192
GSTM5	-3.0325599
GSTM6	-6.4507675
GSTM7	-6.9887
GSTO1	-1.0577087
GSTP1	-4.7552867
GSTP2	-3.3567767
GSTT1	-5.040344
GSTT2	-4.376514
GSTT3	-3.8429894
GSTZ1	-4.8133574
GSX2	-1.7057042
GTF2A2	-1.0810027
GTF2E1	-1.4349298
GTF2E2	-0.1705408
GTF2H2	-0.8932324
GTF2H3	-1.2895579
GTF2H4	-1.9393845
GTF2H5	-0.6446714
GTF2I	-1.5384688
GTF2IRD1	-0.4764905
GTF2IRD2	-0.6612196
GTF3A	-1.6995649
GTF3C1	-1.2650666
GTF3C2	-0.5226517
GTF3C3	-0.6183085
GTF3C4	-0.7090311
GTF3C6	-0.8929043
GTL3	-0.7674928

GTPBP1	-0.1939158
GTPBP10	-1.262332
GTPBP3	-0.6564074
GTPBP6	-0.2967501
GTPBP8	-1.2905841
GTSF1L	-0.9293733
GUCA1A	-3.7058609
GUCA1B	-0.3840981
GUCD1	-3.1279979
GUCY1A2	-0.7393327
GUCY1B2	-1.8920784
GUCY2C	-1.047487
GUCY2E	-1.2132721
GUCY2F	-2.1916935
GUF1	-0.9701529
GUK1	-1.2701664
GULO	-8.267108
GUSB	-0.294272
GXYLT2	-1.2048144
GYK	-2.2288265
GYKL1	-0.9673727
GYLTL1B	-0.8348212
GYPC	-0.7834148
GYS2	-4.871353
GZF1	-1.436739
GZMD	-1.7609084
GZME	-2.4130487
GZMF	-2.1701856
H13	-0.8409438
H19	-4.0015235
H1F0	-1.4284127
H2-AA	-0.5831265
H2-AB1	-1.1641469
H2AFB2	-2.046076
H2AFJ	-0.3442078
H2AFV	-1.7181382
H2AFX	-1.2553158
H2AFY	-0.4821253
H2-DMA	-1.7990341
H2-DMB1	-0.9177909
H2-EA-PS	-2.000917
H2-EB1	-2.1828856
H2-K1	-1.2593145
H2-K2	-0.7038326
H2-KE2	-0.8702216

H2-KE6	-3.0386038
H2-M1	-1.6293433
H2-M10.3	-1.6869874
H2-M10.4	-2.4515646
H2-M11	-1.1992099
H2-M2	-1.0276871
H2-M3	-0.2369289
H2-M9	-2.0398145
H2-OA	-0.2246227
H2-Q1	-4.578223
H2-Q10	-3.2261667
H2-Q2	-1.279911
H2-Q4	-0.674901
H2-Q5	-0.5023804
H2-Q7	-1.2616787
H2-Q8	-1.0418382
H2-T10	-2.4702053
H2-T18	-1.1021423
H2-T23	-1.8477554
H2-T3	-1.2591112
H2-T9	-2.5990114
H60C	-1.2453678
H6PD	-1.2893419
HAAO	-6.1983304
HABP2	-6.074837
HABP4	-2.4285913
HACL1	-5.2864165
HADH	-4.4948816
HADHA	-2.011456
HADHB	-2.8440027
HAGH	-4.0818567
HAGHL	-0.2385545
HAL	-4.487222
HAMP	-3.5071373
HAMP2	-12.424212
HAND1	-1.9356027
HAND2	-4.7110195
HAO1	-8.566832
HAO2	-9.3819
HAPLN2	-1.4091249
HAPLN3	-1.0387943
HAPLN4	-4.9466276
HARBI1	-1.577944
HARS2	-1.6364927
HAS2OS	-0.6465333

HAUS1	-0.7558098
HAUS2	-0.772418
HAUS4	-1.9307313
HAUS5	-1.1275625
HAUS7	-0.699101
HAUS8	-0.8157258
HAVCR1	-6.6103506
HAX1	-1.291903
HBA-A1	-0.417347
HBA-A2	-1.2778959
HBB-BH2	-1.7760179
HBB-BT	-0.1671505
HBP1	-0.2844367
HBQ1A	-0.9046543
HBS1L	-1.1205392
HC	-1.5593138
HCAR1	-1.3991871
HCFC1	-0.6116824
HCFC1R1	-1.8609304
HCN1	-1.0295763
HCN2	-0.9987488
HCN3	-1.3059938
HDAC10	-1.884614
HDAC11	-4.7202597
HDAC3	-1.1969199
HDAC5	-0.5074105
HDAC6	-1.2548456
HDDC3	-2.472159
HDGF	-0.8207359
HDGFL1	-2.8294368
HDGFRP2	-0.6919127
HDHD2	-0.8188114
HDHD3	-5.6308274
HDLBP	-1.430069
HEATR5A	-0.2119331
HEATR5B	-0.6398032
HEATR6	-0.6562939
HEBP1	-3.9426713
HECTD1	-0.3215551
HECTD3	-1.7255301
HECW1	-1.0217886
HELB	-0.4818034
HELQ	-0.8757834
HEMGN	-0.8804135
HEMK1	-2.9759917

HEPH	-0.8660157
HERPUD1	-0.4093709
HES5	-0.6765435
HES6	-2.6489239
HESX1	-1.2724235
HEXA	-1.2723675
HEXB	-1.2763033
HEXDC	-1.8344109
HEXIM1	-0.4605613
HEXIM2	-0.6618919
HFE	-2.2825298
HFE2	-4.584532
HFM1	-1.417377
HGD	-8.876596
HGF	-0.8706484
HGFAC	-6.6154666
HGS	-0.5993166
HGSNAT	-1.6538315
HHEX	-3.3170376
HHIPL2	-2.1381881
HHLA1	-1.4552839
HIAT1	-0.4415588
HIATL1	-0.366281
HIBADH	-2.3387609
HIBCH	-3.3939238
HIGD1A	-1.166338
HIGD1C	-3.425023
HIGD2A	-2.0164642
HINT1	-1.5585895
HINT2	-3.6819558
HINT3	-2.023561
HIPK2	-0.5036101
HIST1H1C	-1.533577
HIST1H1T	-1.2258091
HIST1H2AA	-0.9218226
HIST1H2AK	-0.1395664
HIST1H2BA	-2.2313147
HIST1H2BC	-2.5721335
HIST1H2BM	-2.1692948
HIST1H4I	-0.4584861
HIST2H2AA2	-0.4096198
HIST2H2AC	-0.1878619
HIST2H3C2	-2.6586578
HIST3H2BB-P	-2.5856218
HJURP	-2.333583

HK1	-0.3993335
HK3	-1.1205993
HLCS	-1.7538812
HLF	-4.2648687
HMBOX1	-1.5937133
HMBS	-2.5308022
HMG20A	-0.21385
HMG20B	-1.046361
HMGB3	-0.5364409
HMGB4	-2.4790313
HMGCL	-3.6890278
HMGCR	-2.3928194
HMGCS1	-2.9220152
HMGCS2	-6.650187
HMGN1	-0.5950732
HMGN5	-1.1691122
HMOX2	-1.6779151
HMX1	-1.149287
HN1L	-0.9336784
HNF1A	-4.958329
HNF1B	-1.099102
HNF4A	-4.1731153
HNF4AOS	-5.6364417
HNMT	-1.8936095
HNRNPC	-0.1907497
HNRNPF	-0.2300191
HNRNPUL2	-0.169641
HOGA1	-5.207117
HOMER1	-0.5780025
HOMER2	-3.024863
HOOK1	-1.2765629
HOOK2	-1.3305001
HOOK3	-0.7506843
HORMAD1	-1.7796507
HORMAD2	-1.1518402
HOTTIP	-1.6268129
HOXA11	-1.4162498
HOXA2	-0.3858116
HOXB13	-1.4693842
HOXB9	-0.9669118
HOXC12	-2.254962
HOXC6	-1.2003043
HOXC8	-1.4765649
HOXC9	-1.4716635
HOXD11	-1.3811564

HOXD12	-1.5986474
HOXD3	-2.8168638
HP	-1.3200312
HP1BP3	-0.5167151
HPCA	-1.3106394
HPD	-8.1612
HPDL	-1.4620426
HPGD	-2.7568803
HPN	-4.691192
HPRT	-0.2988949
HPS1	-0.6462085
HPS3	-0.3072696
HPS4	-0.6865602
HPS6	-0.2968078
HPSE	-0.7808232
HPX	-1.7000961
HR	-0.3062835
HRAS	-1.6120572
HRASLS	-1.9443021
HRG	-5.525778
HRH4	-0.9804881
HRK	-2.970553
HRSP12	-6.43196
HS1BP3	-1.8724995
HS2ST1	-1.1981578
HS3ST2	-1.0181711
HS3ST3A1	-2.0959108
HS3ST3B1	-1.5944242
HS3ST4	-2.7273002
HS3ST6	-0.449666
HS6ST1	-2.4406612
HS6ST3	-1.0371761
HSBP1	-0.8542447
HSD11B1	-3.842123
HSD11B2	-1.3385127
HSD17B1	-1.6645374
HSD17B10	-3.4768553
HSD17B11	-1.8472128
HSD17B12	-2.2722888
HSD17B13	-5.018888
HSD17B2	-7.2450123
HSD17B3	-1.905494
HSD17B4	-2.772193
HSD17B6	-11.133291
HSD17B7	-2.7079623

HSD3B1	-6.070076
HSD3B2	-1.2790446
HSD3B3	-12.564512
HSD3B5	-2.3897727
HSD3B6	-5.510487
HSD3B7	-3.467557
HSDL2	-2.5889945
HSF1	-0.5719647
HSF2	-1.9906263
HSF4	-2.070848
HSFY2	-1.4030392
HSP90AA1	-0.2420502
HSP90AB1	-0.956811
HSP90B1	-0.6129346
HSPA1A	-1.9404504
HSPA4	-0.4798985
HSPA5	-0.236721
HSPA8	-0.997139
HSPA9	-1.4230227
HSPB11	-0.8443937
HSPB8	-2.7274642
HSPBP1	-1.6967392
HSPD1	-2.0858212
HSPE1	-1.6266847
HSPG2	-0.839087
HSPH1	-0.4664245
HTATIP2	-3.615335
HTR3B	-1.1860831
HTR4	-2.1321127
HTR5B	-1.6999388
HTR6	-1.3624461
HTRA2	-0.8477755
HUNK	-0.5769467
HUS1B	-0.9185028
HYAL1	-2.9117107
HYAL2	-1.5687699
HYAL4	-1.6592546
HYAL5	-3.0137463
HYI	-3.9999003
HYKK	-4.7018423
HYPK	-0.806222
IAH1	-2.1440296
IARS2	-1.3587794
IBA57	-0.9724457
IBSP	-1.6333983

ICAM4	-0.4911377
ICT1	-1.3778052
ID2	-0.9823723
ID4	-0.7300239
IDE	-1.0012307
IDH1	-2.0608177
IDH2	-1.6758113
IDH3A	-0.7861099
IDH3B	-2.109323
IDH3G	-0.4718103
IDI1	-3.3602657
IDI2	-0.865072
IDNK	-1.7407074
IDO2	-7.165178
IDUA	-0.539959
IFFO1	-0.6234479
IFI203	-0.7842479
IFI27	-1.4669785
IFI27L2B	-2.8967443
IFI30	-0.4890599
IFITM2	-1.0459309
IFITM3	-1.4180937
IFITM7	-0.9721003
IFLTD1	-1.694406
IFNA13	-1.6607263
IFNA16	-1.2639539
IFNA2	-0.6279583
IFNA4	-1.7775471
IFNAR2	-1.035244
IFNB1	-1.1921482
IFNE	-1.1663325
IFNGR2	-0.179843
IFNL3	-1.5056942
IFRD2	-2.335947
IFT20	-1.143569
IFT22	-0.3963397
IFT27	-1.3166709
IFT46	-0.9893727
IFT52	-1.1511192
IFT88OS	-1.2683554
IGBP1	-0.8592863
IGBP1B	-2.7052708
IGDCC4	-2.4244487
IGF1	-5.1407137
IGF1OS	-2.5421293

IGF2BP3	-3.9048142
IGF2OS	-1.0930586
IGF2R	-0.916883
IGFALS	-8.012356
IGFBP1	-0.7044888
IGFBP2	-6.0014124
IGFBP4	-4.3176003
IGFBPL1	-1.5429006
IGFL3	-0.9561229
IGFN1	-1.1942008
IGHMBP2	-0.3514614
IGIP	-1.1046429
IGKV4-72	-1.7674541
IGLON5	-1.6572058
IGSF11	-4.128628
IGSF5	-1.5638118
IGSF8	-0.1854711
IGSF9	-0.8558931
IGTP	-0.9380703
IHH	-2.2502944
IIGP1	-0.7037282
IKBIP	-0.6656137
IKBKAP	-0.1845751
IKBKB	-0.1970859
IKBKG	-1.1180139
IKZF3	-2.7016482
IL11RA1	-0.8354726
IL13	-1.6094317
IL15	-0.9206209
IL15RA	-0.6423073
IL17RB	-3.3848248
IL17RC	-2.160039
IL18	-1.0661893
IL18BP	-1.0400267
IL1RAP	-5.2056656
IL1RAPL2	-1.6317403
IL20	-1.4869359
IL22RA1	-1.9711428
IL27	-1.5565209
IL27RA	-0.5699296
IL31RA	-0.7320249
IL5	-0.6027372
IL6RA	-0.8410039
ILDR2	-1.1506302
ILF3	-0.316391

ILK	-0.558259
ILKAP	-0.5836849
ILTIFB	-2.323699
ILVBL	-2.0725312
IMMP1L	-1.0525265
IMMP2L	-4.6987314
IMMT	-1.4785976
IMP3	-0.4950318
IMPA1	-1.7538462
IMPA2	-0.5649047
IMPAD1	-0.5962172
IMPG1	-1.1844678
IMPG2	-2.973839
INCA1	-4.954744
INF2	-0.887619
ING4	-1.1585083
INHBA	-1.7713327
INHBC	-7.509181
INHBE	-4.2215157
INMT	-3.5256548
INPP1	-1.1495628
INPP5A	-0.3838267
INPP5E	-0.6272326
INPPL1	-1.2067037
INSC	-4.0462704
INSIG1	-3.0422215
INSIG2	-3.189404
INSL5	-0.5824933
INSM2	-1.3339322
INSR	-1.0964594
INSRR	-2.0169013
INTS1	-1.3266301
INTS10	-0.5980687
INTS3	-1.1410077
INTS4	-0.981411
INTS5	-0.2410216
INTS7	-0.1747344
INTS8	-0.4981146
INTU	-0.7015476
INVS	-1.4425821
IP6K1	-0.7021132
IPO11	-0.8054342
IPO4	-0.786983
IPO8	-1.2431116
IPP	-1.4880011

IQCC	-1.383637
IQCE	-0.7444749
IQCF5	-1.7491896
IQCG	-0.9763567
IQCJ	-1.2793033
IQGAP2	-3.7394955
IQGAP3	-1.8408196
IQSEC1	-0.5484409
IQSEC2	-0.6098456
IQSEC3	-4.3543825
IRAK1	-0.3032308
IRAK1BP1	-1.230371
IRAK2	-1.048635
IRAK4	-0.3369722
IRF2	-0.2195697
IRF2BP1	-1.3134589
IRF3	-1.150342
IRF6	-1.7789173
IRGC1	-1.2676868
IRGM1	-0.7004757
IRGM2	-0.9460697
IRGQ	-0.4016633
IRS1	-3.454605
IRS3	-0.6627166
IRS4	-0.8562045
IRX3OS	-1.0948291
ISCA1	-1.2709184
ISCA2	-1.743804
ISCU	-0.2452445
ISG20L2	-0.3789625
ISL1	-1.2930605
ISLR	-1.1530795
ISM2	-2.3692322
ISOC1	-2.1684823
ISOC2A	-2.643236
ISOC2B	-2.27336
IST1	-0.3930888
ISX	-1.3655882
ITCH	-0.7999759
ITFG1	-1.4848928
ITFG2	-0.7804437
ITFG3	-1.9679747
ITGA10	-0.3393791
ITGA5	-0.3389888
ITGA9	-0.2351685

ITGAD	-0.534178
ITGAL	-0.1694441
ITGB1BP1	-0.2840929
ITGB3BP	-1.2552516
ITGB5	-1.9173579
ITGB7	-0.4354878
ITIH1	-5.6139507
ITIH2	-4.2461224
ITIH3	-3.199945
ITIH4	-1.9533377
ITIH5	-0.5041976
ITM2B	-1.8725047
ITM2C	-0.2140975
ITPA	-1.3723755
ITPK1	-0.895237
ITPKA	-1.1239438
ITPR2	-1.3208699
ITPR3OS	-1.2226701
ITSN1	-1.0242467
IVD	-3.2430253
IVNS1ABP	-0.457613
IYD	-5.6255455
IZUMO3	-1.8136027
IZUMO4	-2.3822145
JADE1	-1.212595
JAGN1	-1.7908931
JAK1	-0.1340532
JAKMIP2	-3.2994785
JAKMIP3	-1.7433124
JAZF1	-1.1977346
JKAMP	-1.505364
JMJD4	-1.8601086
JMJD7	-1.8519907
JMJD8	-2.9352531
JOSD1	-1.6941419
JOSD2	-1.7852507
JPH4	-2.1588435
JPX	-1.0726361
JRK	-0.9599488
JTB	-1.407896
JUNOS	-0.9401298
KALRN	-0.6346119
KANK1	-0.6985669
KANSL1L	-0.5363679
KANSL2	-0.3184323

KAP	-0.6276891
KARS	-1.0076003
KAT2A	-1.5670581
KAT2B	-1.8971105
KAT8	-1.2713337
KBTBD13	-1.6512578
KBTBD4	-0.975246
KBTBD7	-0.9984729
KCNA2	-3.1526575
KCNA4	-0.5712228
KCNA6	-0.4069202
KCNB1	-1.3603702
KCNC3	-0.3735502
KCND2	-0.8639498
KCND3OS	-2.5480263
KCNE1	-1.2661886
KCNE1L	-4.120969
KCNF1	-3.0680547
KCNG2	-2.5174077
KCNG4	-1.7369916
KCNH4	-0.5860846
KCNH5	-1.6355968
KCNH6	-2.2132742
KCNH7	-1.119992
KCNH8	-1.3920147
KCNIP1	-0.2222595
KCNIP2	-0.8084672
KCNJ1	-1.3953319
KCNJ10	-3.6662858
KCNJ12	-1.7873116
KCNJ14	-1.7925239
KCNJ16	-3.3502538
KCNJ3	-0.9266591
KCNJ9	-2.3230278
KCNK10	-1.6398644
KCNK15	-1.4415433
KCNK16	-1.7927909
KCNK18	-1.067837
KCNK5	-2.092146
KCNK7	-1.1046708
KCNN1	-0.9590287
KCNN2	-6.364468
KCNQ2	-1.2740388
KCNQ3	-2.0447907
KCNQ5	-1.5421355

KCNV1	-1.7806597
KCNV2	-1.5199842
KCP	-3.4900877
KCTD14	-0.8154922
KCTD15	-0.4445367
KCTD16	-1.638689
KCTD17	-0.6055732
KCTD18	-0.2430296
KCTD2	-1.0067396
KCTD20	-0.3650093
KCTD21	-0.5540962
KCTD6	-0.3500505
KCTD7	-1.5244489
KDELC1	-0.8719974
KDELR1	-1.5232806
KDELR2	-1.2364159
KDM1B	-2.223287
KDM2A	-0.2740908
KDM2B	-0.1887727
KDM4A	-0.2000923
KDM4B	-0.2637043
KDM4D	-1.4109585
KDM5C	-0.4232788
KDM8	-0.6425235
KDR	-0.4134007
KDSR	-0.7982969
KEAP1	-0.8268304
KEG1	-7.453129
KERA	-1.4976103
KHDC1A	-1.6835351
KHDC1C	-1.4251144
KHDRBS2	-2.9155567
KHDRBS3	-1.9983006
KHK	-5.1867466
KHSRP	-1.6129298
KIF11	-0.7193623
KIF12	-2.6202083
KIF13B	-1.5468817
KIF17	-0.915287
KIF18A	-1.0104413
KIF18B	-0.9223361
KIF1B	-1.1873708
KIF1C	-0.9462519
KIF20A	-1.8548982
KIF20B	-0.6159396

KIF21A	-1.6711898
KIF22	-0.5363512
KIF24	-0.4665113
KIF26A	-0.5622208
KIF26B	-0.7977729
KIF2B	-1.1972361
KIF2C	-0.8955963
KIF3C	-2.3919353
KIF4	-0.7333956
KIF4-PS	-0.9523063
KIF7	-0.6785679
KIFC1	-1.6364386
KIFC2	-1.7539086
KIFC3	-1.8030534
KIR3DL1	-1.1147428
KIRREL2	-2.3609562
KISS1	-3.2924116
KLB	-7.8657804
KLC4	-2.2537956
KLF1	-4.3027744
KLF12	-3.2577913
KLF15	-2.096478
KLHDC10	-1.3007102
KLHDC2	-1.2984214
KLHDC3	-1.2243619
KLHDC7A	-1.7731943
KLHDC7B	-0.8277965
KLHDC8A	-1.9236417
KLHDC8B	-0.8661184
KLHL10	-1.6779873
KLHL12	-0.5197868
KLHL13	-2.3692503
KLHL17	-1.1259708
KLHL22	-0.6034021
KLHL23	-1.0593977
KLHL25	-1.19632
KLHL26	-0.3169632
KLHL3	-2.2513382
KLHL34	-2.0422773
KLHL35	-0.1413169
KLHL36	-1.2370424
KLHL4	-1.8761182
KLHL8	-0.9546065
KLHL9	-0.8800592
KLK15	-2.5238833

KLK1B1	-1.4386406
KLK1B11	-1.5795
KLK1B16	-1.7351291
KLK1B22	-2.084212
KLK1B24	-0.7097623
KLK1B3	-1.0645988
KLK1B4	-1.8433614
KLK1B7-PS	-2.1543236
KLK1B8	-1.2626545
KLK5	-1.1746399
KLK7	-0.9804936
KLKB1	-8.030532
KLRA10	-2.8120565
KLRA12	-0.2603869
KLRA5	-2.1050863
KLRA6	-1.2886422
KLRB1-PS1	-1.2651875
KLRC2	-1.4478681
KLRE1	-1.0933161
KLRK1	-1.5670354
KMO	-4.180956
KMT2B	-0.2456241
KNG1	-3.3913126
KNG2	-4.413828
KNOP1	-0.4427667
KNSTRN	-0.7852154
KNTC1	-1.1885295
KPNA1	-0.3764024
KPNA2	-0.5082631
KPNA3	-0.3807669
KPNA6	-0.5402579
KPNA7	-2.0137749
KPNB1	-1.0732675
KPTN	-2.1353498
KRBA1	-1.275444
KRCC1	-0.6995587
KRT10	-0.1822069
KRT12	-1.5870211
KRT18	-1.4591718
KRT2	-1.1594856
KRT20	-0.2176273
KRT222	-0.6125984
KRT24	-1.7582555
KRT25	-1.7597454
KRT27	-1.5634613

KRT28	-1.3033371
KRT33B	-1.6381919
KRT35	-0.9667206
KRT36	-1.8494842
KRT39	-1.5842412
KRT71	-1.0398884
KRT72	-1.2264972
KRT73	-1.87081
KRT76	-1.3632185
KRT77	-0.7122397
KRT8	-1.7189622
KRT82	-3.1801193
KRT9	-1.0193315
KRTAP12-1	-1.6447351
KRTAP13-1	-1.6034498
KRTAP1-4	-0.8432574
KRTAP15	-3.319427
KRTAP17-1	-1.171242
KRTAP19-1	-1.7342887
KRTAP19-3	-2.3326366
KRTAP19-5	-0.7375307
KRTAP21-1	-2.890701
KRTAP28-10	-1.7273791
KRTAP31-2	-1.611629
KRTAP3-3	-1.3208332
KRTAP4-2	-2.9138734
KRTAP4-8	-0.8068337
KRTAP5-2	-0.3146436
KRTAP6-1	-1.632148
KRTAP8-1	-1.0100636
KRTCAP2	-0.7386646
KSR2	-1.1634498
KTI12	-1.1060753
KXD1	-2.2088418
KY	-2.396464
KYNU	-5.079651
L2HGDH	-3.787463
L3HYPDH	-4.7275467
L3MBTL2	-1.4738019
L7RN6	-1.1903939
LACC1	-0.6508653
LACE1	-2.0076241
LACTB	-2.4363236
LACTB2	-3.1489668
LAG3	-0.220026

LAGE3	-1.9042706
LAMA1	-1.1886358
LAMP1	-1.3040934
LAMP2	-1.1521502
LAMTOR1	-1.3165865
LAMTOR2	-1.3578963
LAMTOR4	-2.0532918
LAMTOR5	-0.4043589
LANCL1	-0.6775394
LAO1	-1.6222997
LAP3	-1.9496889
LAPTM4B	-0.2678604
LARP1	-0.6451097
LARP1B	-1.9668531
LARP4	-0.2993321
LARS2	-1.3706799
LAS1L	-0.7909069
LASP1	-0.481338
LAT	-0.3137593
LBH	-0.3728423
LBP	-1.5782056
LCAT	-4.9969106
LCLAT1	-2.3339543
LCMT1	-1.5036454
LCMT2	-1.5797629
LCN11	-2.9363995
LCN12	-0.4689446
LCN5	-0.9101956
LCN8	-1.9396834
LCN9	-1.6845684
LCT	-1.2825074
LDHA	-1.5075374
LDHC	-1.0343385
LDHD	-3.5262685
LDLR	-2.6948385
LDLRAD2	-1.6120026
LDLRAD3	-1.1989646
LDLRAP1	-1.6340799
LEAP2	-8.121006
LECT1	-2.8024173
LECT2	-8.690167
LEFTY1	-2.6264625
LELP1	-0.341306
LEMD1	-1.3110275
LEMD2	-0.2681704

LENEP	-1.2342606
LENG1	-0.446795
LENG9	-0.225564
LEP	-0.7476451
LEPRE1	-0.5256202
LEPROT	-0.3156829
LEPROTL1	-0.2365856
LETM1	-0.4899836
LETMD1	-1.5463996
LGALS12	-0.7036777
LGALS2	-2.1941848
LGALS4	-2.6511106
LGALS6	-2.9372923
LGALS8	-0.7916064
LGALS9	-3.0511842
LGALSL	-0.6003819
LGI1	-2.015221
LGMN	-0.5726705
LGR4	-1.6961043
LGR5	-3.9512253
LGSN	-1.7060859
LHFPL4	-0.6537309
LHPP	-3.2061772
LHX1	-1.5000231
LHX2	-5.7668138
LHX6	-0.854929
LHX8	-0.1637332
LIAS	-1.3074913
LIFR	-3.1319828
LIG3	-0.6317134
LILRA5	-1.5091133
LIMD1	-0.553679
LIMD2	-0.5370793
LIMS2	-1.4828053
LIN37	-0.9856
LIN7A	-6.7455826
LIN7B	-2.7275267
LINGO1	-3.1389003
LINGO2	-1.5891905
LINGO3	-1.1281564
LIPA	-2.7325816
LIPC	-6.934144
LIPE	-0.5221803
LIPK	-1.6949306
LIPO4	-2.033546

LIPT1	-2.4269733
LIPT2	-1.063107
LLGL2	-1.5319633
LLPH	-0.3996005
LMAN1	-1.94837
LMAN2	-1.1807389
LMAN2L	-1.326395
LMBRD1	-1.2710218
LMBRD2	-0.8159776
LMF1	-2.442017
LMF2	-0.7504587
LMNA	-0.1870709
LMNB2	-5.0456576
LMO3	-1.3914604
LMOD2	-0.5417085
LMOD3	-0.437902
LMX1A	-2.0897436
LOC1000450	-1.6109674
LOC1000488	-13.627169
LOC1005025	-1.8366697
LOC1005030	-0.973752
LOC1005046	-0.5430565
LOC1008617	-1.1502275
LOC1010558	-0.7722378
LOC1010558	-0.9546709
LOC1010559	-1.0101962
LOC1010559	-1.7186086
LOC1010560	-0.8410959
LOC1026318	-1.6048071
LOC1026319	-0.2014561
LOC1026322	-0.4274759
LOC1026322	-1.3239846
LOC1026323	-1.046376
LOC1026324	-0.4436545
LOC1026324	-2.7672944
LOC1026324	-1.4709301
LOC1026327	-0.7513609
LOC1026328	-2.3495648
LOC1026330	-1.6420076
LOC1026330	-2.1709752
LOC1026331	-1.000509
LOC1026335	-0.2922988
LOC1026336	-1.8248634
LOC1026337	-0.9318228
LOC1026338	-3.918003

LOC1026341	-1.4098549
LOC1026345	-2.4981046
LOC1026345	-1.1627905
LOC1026346	-2.3838918
LOC1026347	-0.4495549
LOC1026350	-0.5823507
LOC1026353	-1.8597093
LOC1026354	-1.6150777
LOC1026356	-0.9225874
LOC1026356	-1.0919893
LOC1026357	-2.4156106
LOC1026362	-1.0126238
LOC1026362	-0.4648643
LOC1026366	-0.7380509
LOC1026367	-1.1129999
LOC1026368	-0.9322479
LOC1026368	-1.6569028
LOC1026369	-3.0806768
LOC1026369	-1.1479545
LOC1026370	-1.0927656
LOC1026371	-0.3034511
LOC1026372	-0.1608844
LOC1026373	-1.0868475
LOC1026374	-0.8897352
LOC1026376	-1.0146503
LOC1026377	-2.0578213
LOC1026377	-0.8121586
LOC1026378	-1.1853926
LOC1026378	-0.9635406
LOC1026379	-2.0839393
LOC1026381	-1.8521361
LOC1026385	-1.3641953
LOC1026386	-0.7027082
LOC1026386	-1.3952107
LOC1026387	-0.5937986
LOC1026387	-0.1954732
LOC1026388	-1.9426367
LOC1026388	-0.4926872
LOC1026388	-7.4611177
LOC1026389	-0.591352
LOC1026389	-1.0066907
LOC1026396	-1.2218876
LOC1026396	-1.6308577
LOC1026398	-3.9465683
LOC1026398	-2.1709573

LOC1026399	-0.5976362
LOC1026399	-0.2972345
LOC1026399	-0.9438453
LOC1026403	-1.3271785
LOC1026407	-0.6216574
LOC1026410	-1.6404285
LOC1026410	-0.2845182
LOC1026410	-0.8618937
LOC1026412	-0.3541241
LOC1026412	-1.1863983
LOC1026412	-0.8177366
LOC1026413	-8.322931
LOC1026413	-0.8927493
LOC1026413	-0.6483889
LOC1026413	-0.4723516
LOC1026413	-1.3332472
LOC1026414	-0.8183928
LOC1026415	-0.5998979
LOC1026415	-4.5588517
LOC1026416	-1.2636657
LOC1026416	-2.4149566
LOC1026416	-0.6812983
LOC1026416	-3.7308483
LOC1026416	-1.6533029
LOC1026417	-1.7290759
LOC1026418	-0.8566847
LOC1026419	-1.8066196
LOC1026419	-0.5098095
LOC1026420	-1.9050369
LOC1026420	-2.2912822
LOC1026420	-2.747625
LOC1026421	-1.761982
LOC1026422	-0.3089957
LOC1026422	-1.2949412
LOC1026423	-2.0506747
LOC1026424	-0.6023984
LOC1026424	-1.8216572
LOC1026424	-0.1974864
LOC1026425	-0.7450676
LOC1026426	-1.5548334
LOC1026426	-0.6540275
LOC1026427	-1.3614371
LOC1026428	-0.6155639
LOC1026429	-6.2057705
LOC1026429	-0.6525703

LOC1026429	-1.8958223
LOC1026430	-1.1662376
LOC1026431	-1.3433738
LOC1026431	-1.0004139
LOC1026432	-0.6028314
LOC171588	-0.9954271
LOC547349	-1.7741165
LOC630751	-0.6973023
LOC674896	-0.5089536
LOH12CR1	-1.7076018
LONP1	-1.7778697
LONP2	-3.171328
LONRF1	-0.6229477
LOXL2	-0.6615229
LOXL3	-0.2713244
LPAR5	-2.0905128
LPAR6	-1.6887317
LPCAT3	-1.5791197
LPCAT4	-1.5671942
LPGAT1	-0.8447738
LPIN1	-1.1277647
LPL	-0.2797246
LPP	-0.5308909
LRAT	-3.430598
LRBA	-0.4499259
LRCOL1	-1.3314686
LRFN3	-4.3494077
LRFN5	-1.4111705
LRG1	-0.544312
LRIG1	-0.9986296
LRIG3	-1.3743644
LRIT1	-7.380804
LRIT2	-2.696157
LRP1	-1.7352543
LRP11	-0.6712747
LRP1B	-1.853312
LRP2BP	-0.860626
LRP3	-4.071825
LRP5	-1.5402045
LRP6	-0.4780736
LRP8OS1	-1.7910812
LRPAP1	-0.5459719
LRPPRC	-2.0185595
LRR1	-0.8110642
LRRC14	-1.2571368

LRRC14B	-1.4407773
LRRC16B	-2.049194
LRRC17	-1.5146892
LRRC18	-0.583699
LRRC19	-1.4257116
LRRC20	-1.2603884
LRRC24	-7.2445927
LRRC28	-1.6564822
LRRC29	-1.6557281
LRRC3	-6.230353
LRRC30	-1.1554141
LRRC31	-0.4732451
LRRC39	-1.5023048
LRRC3B	-0.2293243
LRRC41	-1.1936908
LRRC42	-1.6400423
LRRC45	-0.4212961
LRRC4C	-1.4233952
LRRC55	-0.9341703
LRRC56	-0.1966472
LRRC57	-0.5761781
LRRC61	-1.1328907
LRRC66	-1.0126126
LRRC69	-1.5865626
LRRC7	-2.0275187
LRRC8D	-0.4916267
LRRIQ3	-2.325328
LRRIQ4	-2.0970466
LRRN3	-0.6271908
LRRTM2	-1.2748001
LRSAM1	-1.6571889
LRTM1	-6.2550893
LRWD1	-0.1810155
LSG1	-0.6281309
LSM1	-1.3929634
LSM10	-1.5422812
LSM11	-0.7813242
LSM14B	-1.4859691
LSM2	-1.4786072
LSM5	-0.4181051
LSM6	-1.1099234
LSM7	-0.6082411
LSMEM2	-1.5178218
LSR	-3.0121527
LSS	-2.5447483

LST1	-0.6721506
LTA4H	-1.1158934
LTBR	-1.2358527
LTK	-0.3590338
LTN1	-0.5173287
LUC7L	-0.4634724
LUC7L2	-1.0143123
LUM	-1.4279943
LURAP1L	-1.6775298
LUZP2	-2.2690506
LUZP4	-1.6192031
LY6D	-1.583529
LY6E	-0.3098073
LY6G	-0.9670587
LY6G5C	-1.1344111
LY6G6F	-1.9404001
LY86	-0.6390615
LY9	-1.1063969
LY96	-0.3187408
LYG1	-1.2927089
LYPD1	-1.606123
LYPD8	-1.389858
LYPLA1	-2.52208
LYPLA2	-0.7131772
LYPLAL1	-4.4010763
LYRM1	-0.5983653
LYRM4	-0.8677454
LYRM5	-2.7461739
LYRM7	-1.7103653
LYRM9	-3.0427275
LYSMD1	-0.9156046
LYSMD4	-1.2690506
LZIC	-0.2269564
M6PR	-0.6034045
MAB21L1	-0.967912
MAB21L2	-1.7949665
MACROD1	-3.0205894
MACROD2	-2.186747
MAD1L1	-1.6269412
MAD2L1	-0.7467506
MAD2L2	-1.1472087
MADD	-0.9768181
MAEL	-1.6431861
MAF	-2.1095877
MAF1	-0.2501459

MAFB	-2.0187387
MAFG	-0.3025317
MAGEA10	-1.5730305
MAGEA3	-1.8076739
MAGEA5	-0.9161136
MAGEA6	-1.6763401
MAGEA8	-1.5244806
MAGEB1	-1.990704
MAGEB16-PS	-1.1278608
MAGEB18	-3.353495
MAGEB5	-1.7067394
MAGED1	-1.6715426
MAGED2	-0.5715885
MAGEE2	-2.916872
MAGEH1	-0.4143548
MAGIX	-4.701687
MAGT1	-0.6082521
MALSU1	-0.8814025
MAMDC2	-0.3796403
MAMDC4	-1.0413728
MAN1A	-2.121561
MAN1A2	-0.4856472
MAN1B1	-0.4217024
MAN2A1	-1.2011256
MAN2A2	-0.7202392
MAN2B1	-1.6517591
MAN2B2	-2.4353228
MAN2C1	-0.3289833
MAN2C1OS	-1.495924
MANBA	-2.02205
MANEA	-1.2112627
MANF	-0.3713746
MANSC1	-0.3230848
MAOA	-0.5912833
MAOB	-5.0402117
MAP1LC3A	-0.8429713
MAP2K2	-1.7130198
MAP2K3	-1.0475259
MAP2K4	-0.5316453
MAP2K5	-1.2257428
MAP2K6	-4.39274
MAP2K7	-0.5321293
MAP3K10	-1.5328407
MAP3K11	-0.8509026
MAP3K13	-0.9896238

MAP3K2	-0.6196926
MAP3K4	-1.5386167
MAP3K5	-0.6992288
MAP3K7CL	-2.8347774
MAP4K2	-0.8729761
MAP4K3	-0.3090968
MAPK14	-1.5350285
MAPK15	-1.3190682
MAPK1IP1L	-0.6525655
MAPK3	-0.497015
MAPK4	-2.0280104
MAPK8	-0.7175446
MAPK8IP1	-0.7962394
MAPK9	-1.5541577
MAPKAP1	-1.2306318
MAPKAPK5	-0.8970194
MAPRE3	-1.773551
MARCO	-0.6250129
MARS	-0.5594506
MARVELD1	-1.4189868
MARVELD2	-0.8283191
MASP1	-5.5993795
MASP2	-5.6172276
MAST1	-2.4064844
MAST3	-1.4375191
MASTL	-1.207356
MAT1A	-3.8178391
MAT2B	-0.3392158
MATK	-0.9282661
MATN3	-1.5256627
MAU2	-0.5417409
MAVS	-3.0458999
MAZ	-0.4751043
MB21D2	-0.4905613
MBD3	-1.1061115
MBL1	-4.603528
MBL2	-6.813462
MBLAC1	-1.3976672
MBLAC2	-3.2191548
MBOAT7	-0.7269673
MC2R	-1.4316797
MC3R	-0.8193846
MC4R	-1.7070241
MCAT	-1.7433677
MCC	-1.4113097

MCCC1	-2.399086
MCCC2	-3.3642497
MCEE	-3.4561663
MCF2	-1.6331849
MCFD2	-2.248753
MCHR1	-0.805912
MCIDAS	-0.6311278
MCM10	-2.4781737
MCM2	-0.303494
MCM3AP	-0.5030124
MCM5	-0.4479227
MCM7	-0.5455313
MCMDC2	-3.393696
MCOLN1	-0.7793522
MCPT1	-1.0885088
MCPT-PS1	-1.4420371
MCTS1	-0.7825131
MCTS2	-1.6107373
MCU	-0.6790698
MCUR1	-0.9841933
MDH1	-3.0297012
MDH2	-0.8087916
MDP1	-0.8406978
ME1	-1.7500648
MEA1	-1.5585065
MECOM	-1.5621362
MECR	-2.5939696
MED11	-0.2335253
MED12L	-1.0085566
MED16	-1.1072884
MED18	-0.2461782
MED19	-0.2558208
MED20	-0.6287222
MED22	-1.2186298
MED25	-1.631733
MED28	-0.5533433
MED29	-1.7470698
MED6	-0.4440417
MED7	-0.1889195
MED8	-1.0007577
MED9	-0.3456979
MEDAG	-0.8736215
MEG3	-0.6225007
MEGF11	-0.4668365
MEGF9	-0.2173314

MEIOB	-5.863386
MEIS1	-0.3100452
MEIS3	-0.2431681
MEMO1	-0.7969894
MEN1	-1.2254639
MEP1A	-1.3221381
MEPE	-0.1897326
MERTK	-0.6585403
MESDC2	-0.6710777
MEST	-1.2708774
MET	-1.2936211
METAP1	-0.9080391
METAP1D	-3.2233737
METRN	-1.5694675
METTL1	-0.4677677
METTL10	-0.3294845
METTL11B	-2.3163087
METTL13	-1.7433476
METTL15	-1.8827956
METTL16	-0.4818311
METTL17	-2.188746
METTL18	-0.7595987
METTL2	-0.8804677
METTL20	-1.0129423
METTL21A	-1.6291237
METTL22	-2.1411514
METTL23	-1.0367932
METTL4	-0.6807804
METTL5	-2.0278955
METTL7A2	-0.9775305
METTL7A2HI	-2.6063623
METTL7A3	-0.6324973
METTL7B	-6.7695303
METTL8	-1.5754797
METTL9	-0.8394585
MFHAS1	-0.6523356
MFI2	-2.4663374
MFN1	-1.3593364
MFN2	-1.7296371
MFSD1	-0.722342
MFSD11	-1.3874164
MFSD12	-1.292234
MFSD2A	-3.0530548
MFSD2B	-0.4417796
MFSD3	-3.5270152

MFSD5	-0.4381466
MFSD7C	-1.502265
MFSD8	-1.08813
MFSD9	-3.0606966
MGAM	-3.4142694
MGAT1	-1.0201125
MGAT2	-0.9604807
MGAT4B	-1.6105552
MGAT4C	-1.6555622
MGAT5B	-1.0400395
MGL2	-0.1520777
MGLL	-4.2136245
MGME1	-3.349988
MGMT	-3.9663725
MGRN1	-0.3483853
MGST1	-2.8187284
MGST2	-2.0130758
MGST3	-1.8921032
MIA2	-3.943311
MIA3	-1.5859146
MIB2	-1.1567907
MICAL3	-0.73382
MICALL2	-0.8645825
MICU1	-0.4741073
MICU2	-0.4168434
MID1	-0.2491994
MID1IP1	-0.5893788
MIEF1	-0.6161094
MIEF2	-1.3368065
MIEN1	-2.1558495
MIER2	-0.9904413
MIF	-1.5036936
MIF4GD	-0.6714101
MIIP	-0.9825373
MILR1	-0.9534721
MINA	-0.8396821
MINK1	-0.673779
MINOS1	-0.8035097
MINPP1	-1.6411977
MIOX	-4.763569
MIP	-2.954612
MIPEP	-2.2913663
MIRG	-1.7849097
MKKS	-1.9849119
MKL2	-0.5295453

MKLN1	-0.9312997
MKNK2	-0.167367
MKRN2	-1.0776758
MLEC	-1.5393701
MLF2	-1.3130279
MLH1	-1.4552498
MLH3	-0.9669321
MLLT1	-0.251152
MLST8	-1.1559715
MLX	-1.5263634
MLXIPL	-4.3684382
MLYCD	-2.7656956
MMAA	-2.2721658
MMAB	-3.1801417
MMACHC	-2.2869964
MMADHC	-0.7905588
MMD	-2.882689
MMD2	-7.5815344
MME	-0.4990883
MMEL1	-1.9235101
MMGT1	-0.6303511
MMP10	-1.2672129
MMP15	-0.8719735
MMP19	-0.5649123
MMP1B	-1.7375205
MMP21	-1.9286842
MMP24	-0.311985
MMP27	-1.0008187
MMP28	-1.5958645
MMRN2	-0.4531608
MMS22L	-0.9401999
MN1	-2.1138737
MNAT1	-0.2582893
MND1	-2.1480365
MNX1	-1.0352128
MOB2	-0.9096036
MOB3B	-1.199738
MOB3C	-0.5798466
MOBP	-2.480257
MOCOS	-2.7097716
MOCS1	-1.0849209
MOCS2	-2.525539
MOCS3	-0.8456698
MOGAT2	-3.082892
MOGS	-1.0778389

MON1A	-1.7291455
MORC2B	-0.6749802
MORC4	-1.3735015
MORF4L1	-0.4209523
MORF4L2	-0.3328672
MORN1	-0.4137845
MORN2	-2.2994192
MOS	-1.6824617
MOSPD2	-1.2350388
MOSPD3	-2.307313
MOSPD4	-1.6143003
MOV10	-2.234673
MPC1	-2.677608
MPC2	-3.6643739
MPDU1	-1.8615665
MPDZ	-1.1450517
MPEG1	-0.5538254
MPG	-1.0897455
MPHOSPH9	-0.4978213
MPI	-1.6567245
MPLKIP	-1.219202
MPND	-2.2516913
MPO	-1.6761198
MPP1	-1.301795
MPP4	-2.6549032
MPP6	-2.2475376
MPPE1	-1.5384398
MPST	-3.4103894
MPTX1	-1.7695875
MPV17	-1.3184004
MPV17L	-3.0599399
MPV17L2	-0.9720693
MPZL2	-2.276538
MR1	-0.9814775
MRAP	-4.011363
MRC1	-0.5356312
MREG	-3.149601
MRFAP1	-0.4923296
MRGBP	-0.3125482
MRGPRA1	-1.6036663
MRGPRA9	-1.7322998
MRGPRB1	-1.6406496
MRGPRB2	-1.6193573
MRGPRB8	-1.680254
MRGPRD	-1.3521919

MRGPRE	-1.202327
MRGPRH	-1.6352048
MRGPRX1	-1.4200335
MRI1	-2.410874
MRO	-0.8828664
MROH2A	-3.2746477
MROH2B	-3.8421805
MROH4	-2.1343322
MROH5	-0.1754217
MRPL1	-1.8265083
MRPL10	-1.9183655
MRPL11	-1.6765819
MRPL12	-3.1166377
MRPL13	-1.6444955
MRPL14	-2.1872401
MRPL15	-2.6569052
MRPL16	-2.0562677
MRPL17	-1.6066117
MRPL18	-1.5077949
MRPL19	-1.3330903
MRPL2	-1.8311076
MRPL21	-1.0536675
MRPL22	-1.6478639
MRPL23	-1.7111931
MRPL24	-1.1155272
MRPL27	-1.8078547
MRPL28	-2.2380586
MRPL30	-1.3153348
MRPL32	-1.0973783
MRPL34	-2.0520253
MRPL35	-2.248416
MRPL36	-1.3553038
MRPL37	-1.7359719
MRPL38	-2.1803799
MRPL39	-1.415163
MRPL4	-1.4958396
MRPL40	-1.3510776
MRPL41	-1.111454
MRPL42	-2.0375361
MRPL43	-1.2842016
MRPL44	-1.5902886
MRPL45	-0.9810948
MRPL46	-1.5322251
MRPL47	-1.1637578
MRPL48	-1.177743

MRPL49	-1.9991059
MRPL50	-2.0023756
MRPL51	-1.0799122
MRPL52	-0.5920849
MRPL53	-1.3801308
MRPL54	-0.9075556
MRPL55	-1.5997577
MRPL57	-0.8059897
MRPL9	-1.2115312
MRPS10	-1.3525805
MRPS11	-1.9293723
MRPS12	-1.9679894
MRPS14	-1.1346402
MRPS15	-1.6783628
MRPS16	-1.7457671
MRPS17	-0.8537941
MRPS18A	-0.894495
MRPS18B	-1.5100036
MRPS18C	-0.5456076
MRPS2	-2.0230541
MRPS21	-1.1935406
MRPS22	-1.6555948
MRPS23	-0.9875212
MRPS24	-2.0337887
MRPS25	-1.0153198
MRPS26	-1.0857587
MRPS27	-2.4028745
MRPS28	-1.427979
MRPS30	-1.5007048
MRPS31	-2.285636
MRPS33	-1.1464391
MRPS34	-1.9136162
MRPS35	-1.8639379
MRPS36	-1.019495
MRPS5	-1.8549557
MRPS7	-1.7268744
MRPS9	-0.7069249
MRS2	-0.6655302
MRTO4	-0.2227345
MS4A10	-1.2679043
MS4A13	-1.4555316
MS4A4C	-0.859406
MS4A5	-1.3096704
MSANTD4	-0.6511331
MSH2	-0.6601424

MSH3	-0.8188863
MSH4	-1.620806
MSH5	-2.3625233
MSL1	-0.3175559
MSMB	-1.0627117
MSMO1	-2.411337
MSMP	-1.8193023
MSRA	-3.6452012
MSRB1	-1.8980818
MSRB2	-2.0139322
MST1	-5.2526217
MSTO1	-0.708931
MSX3	-1.754684
MT1	-0.6448641
MT3	-0.7940168
MTA1	-0.7134681
MTA3	-1.4841924
MTAG2	-1.9757686
MTAP	-2.628932
MTCH2	-2.3520584
MTCP1	-0.3800833
MTERF1A	-0.6689153
MTERF1B	-0.6868618
MTERFD1	-0.8713679
MTERFD2	-1.4507585
MTERFD3	-2.0826132
MTF1	-0.203537
MTFMT	-1.2984467
MTFP1	-4.5192795
MTFR1	-2.4637313
MTFR1L	-0.9419642
MTFR2	-0.9044685
MTG1	-2.1575155
MTG2	-1.9149327
MTHFD1	-3.9249263
MTHFR	-1.0153232
MTHFS	-2.1637259
MTHFSD	-1.5708983
MTIF3	-1.9455369
MTL5	-1.9241796
MTM1	-0.4490171
MTMR1	-0.6647201
MTMR10	-1.2308469
MTMR11	-2.642351
MTMR14	-0.4950171

MTMR2	-0.1580272
MTMR4	-1.5348811
MTMR7	-3.5742953
MTMR9	-0.1565499
MTNR1A	-2.2322497
MTNR1B	-1.907398
MTO1	-1.7741146
MTOR	-0.6743071
MTPAP	-0.6850705
MTPN	-0.2664824
MTRF1L	-0.6219745
MTSS1	-1.810009
MTSS1L	-0.277693
MTTP	-6.0962944
MTUS1	-0.4137516
MTX1	-1.901299
MTX2	-1.8714347
MTX3	-1.9094658
MUC19	-1.3486991
MUC3	-1.2596607
MUG1	-5.6442366
MUG2	-6.041017
MUL1	-0.8217301
MUM1	-0.6367264
MUP1	-12.535407
MUP17	-13.823453
MUP19	-13.628943
MUP2	-10.921619
MUP20	-8.2479725
MUP21	-8.202998
MUP3	-8.644036
MUP4	-9.984295
MUP5	-10.871084
MUP6	-5.309969
MUP-PS12	-10.971701
MUS81	-1.6070819
MUT	-2.6850429
MVD	-1.8519998
MVK	-2.5809164
MXD3	-2.089268
MXI1	-1.2573371
MYCBP	-1.2144675
MYCL	-3.0147476
MYCN	-1.7365522
MYCS	-1.390651

MYCT1	-1.5994375
MYEOV2	-0.7224064
MYF5	-1.4268699
MYG1	-1.6254287
MYH10	-0.4348273
MYH13	-1.3449962
MYH15	-2.1971354
MYL10	-1.7464769
MYL12B	-0.2922115
MYL6	-0.4968391
MYL6B	-0.88503
MYLIP	-0.4001765
MYLPF	-1.2922409
MYNN	-0.1738534
MYO15	-0.7699459
MYO16	-1.4905589
MYO18A	-0.2367659
MYO19	-0.7321453
MYO1B	-1.6063876
MYO1E	-0.548636
MYO3A	-1.3717527
MYO5B	-0.7393284
MYO6	-0.935411
MYO7A	-1.7287273
MYO7B	-3.9884555
MYPN	-2.6021311
MYPOP	-1.1638355
MYRFL	-3.3254995
MYT1	-1.6602831
MYT1L	-2.0171375
MZF1	-0.3380575
MZT2	-1.6825953
N4BP2L1	-1.7540128
N4BP2OS	-1.1814263
N6AMT1	-0.8468485
N6AMT2	-0.9968596
NAA10	-1.7590771
NAA11	-2.5778217
NAA30	-0.193049
NAA35	-0.6953821
NAA38	-1.4140587
NAA40	-0.7807474
NAA50	-0.6631393
NAA60	-1.961925
NAALADL1	-1.4027762

NAALADL2	-2.1109674
NAB2	-1.2579527
NABP2	-1.239152
NACA	-0.3260193
NACC1	-0.3899503
NACC2	-1.3242626
NADK	-0.6480694
NADK2	-4.447686
NADSYN1	-3.5153766
NAE1	-0.2649198
NAGA	-1.2947283
NAGK	-0.6974163
NAGLU	-2.2408195
NAGPA	-0.8198781
NAGS	-6.2335925
NAIF1	-1.4440367
NAIP5	-0.7675934
NAIP7	-0.5490012
NALCN	-1.7669609
NANOS3	-3.536319
NANP	-3.0498524
NANS	-0.3512712
NAP1L2	-0.9953346
NAP1L3	-1.3900003
NAP1L5	-1.5089822
NAPA	-0.3893876
NAPG	-0.5911183
NAPRT1	-4.8343406
NARF	-2.4908376
NARFL	-1.9350796
NARG2	-1.2158906
NARS2	-0.5652642
NAT1	-2.2483888
NAT10	-0.9439359
NAT2	-2.6256504
NAT3	-0.9342294
NAT6	-2.4617662
NAT8	-4.688471
NAT9	-1.4272914
NAV2	-1.410383
NBAS	-0.4622746
NBR1	-0.7121368
NCALD	-0.9714932
NCAPD2	-0.2505961
NCAPD3	-0.5666556

NCAPG	-2.5915873
NCAPH2	-0.6288714
NCEH1	-0.2743216
NCKAP1	-0.5015273
NCKIPSD	-0.6618724
NCLN	-1.4837856
NCMAP	-0.8027237
NCOA1	-0.2210493
NCOA2	-0.9266806
NCOA3	-0.3971491
NCOA4	-1.6961322
NCOA5	-0.3790698
NCTC1	-0.8281965
ND1	-2.1445274
ND2	-1.7263212
ND3	-1.6709018
ND4	-1.1991782
ND4L	-1.986103
ND5	-1.7262497
NDC1	-1.5942934
NDFIP1	-1.3467307
NDFIP2	-1.6474462
NDNL2	-0.1512103
NDOR1	-0.2840395
NDRG2	-4.299885
NDRG3	-0.8391876
NDST1	-0.4663434
NDST2	-1.6775918
NDST3	-1.6774006
NDST4	-2.0284905
NDUFA1	-1.493186
NDUFA10	-1.4657235
NDUFA11	-1.1539159
NDUFA12	-1.3949881
NDUFA13	-2.0414014
NDUFA2	-1.734127
NDUFA3	-1.7482853
NDUFA4	-2.2179527
NDUFA5	-1.7427015
NDUFA6	-2.2494588
NDUFA7	-1.7395215
NDUFA8	-1.9953008
NDUFA9	-2.4068604
NDUFAB1	-2.0445948
NDUFAF1	-1.8987842

NDUFAF2	-1.7333946
NDUFAF3	-1.2654996
NDUFAF4	-1.6085124
NDUFAF5	-1.3579216
NDUFAF6	-3.2949977
NDUFAF7	-1.3003559
NDUFB10	-2.2630954
NDUFB11	-2.056312
NDUFB2	-1.6409669
NDUFB3	-1.1944189
NDUFB4	-1.7627492
NDUFB5	-1.9086466
NDUFB6	-1.9179058
NDUFB7	-1.8714385
NDUFB8	-1.8332615
NDUFB9	-2.0913467
NDUFC1	-1.4938593
NDUFC2	-1.187758
NDUFS1	-1.755436
NDUFS2	-1.7579713
NDUFS3	-1.815361
NDUFS4	-1.746285
NDUFS5	-1.7605028
NDUFS6	-2.1865058
NDUFS7	-1.5099497
NDUFS8	-2.1904259
NDUFV1	-2.1401901
NDUFV2	-1.7764063
NDUFV3	-2.3907847
NECAB1	-3.3852756
NECAB2	-1.1799169
NECAB3	-1.1340756
NECAP1	-1.3657398
NEDD1	-0.5679994
NEDD4	-0.9697552
NEFL	-1.3907213
NEFM	-1.1927941
NEGR1	-1.2642462
NEIL2	-0.3893893
NEIL3	-0.9137189
NEK2	-0.3668566
NEK3	-0.5746074
NEK4	-0.9409971
NEK6	-0.7680154
NELFA	-0.5363951

NELFCD	-0.916121
NELFE	-1.2690158
NELL2	-2.0520413
NENF	-0.4211183
NEPN	-1.3281062
NESPAS	-1.7230878
NETO1	-1.5488856
NEU1	-1.1786432
NEU2	-2.1410358
NEU3	-0.8530073
NEURL1B	-0.9996328
NEURL2	-0.7294903
NEUROD1	-2.6538668
NEUROD4	-1.7078276
NEUROD6	-1.6063654
NFIA	-1.1322978
NFIB	-1.0245752
NFIC	-1.80761
NFIX	-1.1392136
NFKBIL1	-0.3545089
NFS1	-2.1346874
NFU1	-1.7850108
NFX1	-0.6871567
NFYB	-0.5435987
NFYC	-0.7718811
NGEF	-4.4160776
NGF	-1.5952692
NGFR	-3.6368272
NGLY1	-0.5366511
NGRN	-0.7178659
NHEJ1	-1.397852
NHLRC1	-1.8209596
NHLRC3	-0.3450401
NHSL1	-0.4685385
NICN1	-3.318366
NID2	-1.4584291
NIF3L1	-1.0326309
NIFK	-0.6063762
NIM1K	-1.0168517
NINJ1	-1.1760831
NIPA2	-0.5295057
NIPAL1	-3.3492625
NIPSNAP1	-5.8998194
NIPSNAP3B	-2.0754642
NISCH	-0.5810576

NIT1	-2.8669305
NIT2	-5.1839433
NKAIN3	-1.5266423
NKAIN4	-0.617105
NKAP	-0.1535549
NKD2	-2.8833246
NKIRAS1	-1.8697429
NKIRAS2	-0.9867401
NKX1-1	-0.5663846
NKX1-2	-2.0079896
NKX2-2	-1.4990151
NKX2-3	-1.1250618
NKX2-9	-1.0868635
NKX6-2	-3.3577926
NKX6-3	-2.2485032
NLE1	-0.8032484
NLN	-1.0691047
NLRP14	-2.2597737
NLRP1A	-0.4440045
NLRP1B	-0.815963
NLRP1C-PS	-0.5001125
NLRP2	-1.6560295
NLRP4A	-1.4587252
NLRP4E	-1.6738119
NLRP4F	-1.1123612
NLRP4G	-1.0900741
NLRP5-PS	-1.8145039
NLRP6	-5.5332174
NLRP9C	-1.2874811
NLRX1	-1.0263901
NMD3	-0.624579
NME1	-1.0599337
NME2	-1.5497551
NME3	-0.8267746
NME4	-1.9116769
NME6	-2.3145223
NME7	-0.8809905
NMI	-0.1916356
NMNAT1	-2.6785562
NMNAT3	-1.768856
NMRAL1	-2.4401755
NMRK1	-3.6628027
NMT1	-0.4259129
NNMT	-1.3815718
NNT	-3.0656147

NOA1	-0.4782295
NOB1	-0.2725797
NOBOX	-1.4864974
NOC2L	-0.5430389
NODAL	-1.3960378
NOL4	-1.363874
NOL6	-0.4334927
NOL7	-1.4380343
NOL9	-0.1901596
NONO	-0.4881358
NOP10	-1.0558176
NOP16	-1.595695
NOS1	-0.8875155
NOSIP	-0.5190969
NOTO	-1.0878253
NOTUM	-3.07366
NOTUMOS	-5.6045003
NOVA1	-0.2666085
NOX1	-1.4344332
NPAS1	-1.7848423
NPAS3	-1.913902
NPAT	-2.022714
NPB	-2.0608547
NPBWR1	-1.2484124
NPC1	-2.2858791
NPEPL1	-2.2722082
NPFFR1	-1.139534
NPLOC4	-1.1265445
NPM3-PS1	-1.0345483
NPPC	-0.9858232
NPRL2	-1.7503409
NPRL3	-1.5332627
NPS	-1.8131714
NPSR1	-0.9004321
NPTX1	-1.7318859
NPTX2	-1.2491565
NPVF	-0.8613215
NPY2R	-1.5817561
NQO1	-2.9234982
NQO2	-4.334077
NR0B1	-1.3363576
NR0B2	-7.4700985
NR1D1	-1.6397069
NR1D2	-0.9960384
NR1H3	-2.585373

NR1H4	-5.3228493
NR1H5	-3.313067
NR1I2	-6.416458
NR1I3	-7.9913836
NR2C1	-0.721982
NR2C2AP	-1.0906863
NR2F1	-0.9112399
NR2F2	-0.9167695
NR2F6	-3.304222
NR3C1	-0.3058066
NR3C2	-2.0740685
NR5A1	-1.878556
NR5A2	-3.6438327
NRBF2	-1.0673041
NRBP1	-0.40869
NRBP2	-0.4356723
NRDE2	-0.4754019
NREP	-1.3386683
NRG2	-0.6862629
NRG3	-1.7383852
NRIP2	-1.7763977
NRK	-1.607373
NRL	-1.2829375
NRM	-0.5214307
NRN1	-6.682561
NRN1L	-0.563025
NRP	-1.8279276
NRP1	-0.9444103
NRTN	-0.2465525
NRXN1	-2.1905172
NRXN2	-1.1317861
NSA2	-0.6868153
NSDHL	-3.5728707
NSFL1C	-0.2460246
NSL1	-1.1584525
NSMAF	-0.350697
NSMCE4A	-0.4233003
NSMF	-2.2635913
NSUN2	-0.2273607
NSUN3	-1.4310029
NSUN4	-0.3264008
NSUN5	-1.0606508
NT5C	-0.9959931
NT5C1A	-0.6442542
NT5C1B	-1.4956284

NT5C2	-0.5087271
NT5DC1	-0.7559567
NT5DC3	-0.8817396
NT5E	-2.5159655
NT5M	-2.0088081
NTAN1	-0.6099
NTF3	-2.0919971
NTHL1	-2.5920749
NTM	-2.8311067
NTMT1	-0.1518865
NTN3	-3.4964402
NTNG1	-1.125247
NTPCR	-1.1595511
NTRK1	-2.1474926
NUBP1	-0.728899
NUBP2	-0.9937706
NUBPL	-2.5897055
NUCB1	-1.2909479
NUDC	-0.3566299
NUDCD1	-0.4872346
NUDCD2	-1.5969934
NUDCD3	-0.1721416
NUDT1	-3.274354
NUDT12	-3.4995337
NUDT12OS	-1.0937848
NUDT13	-1.0057402
NUDT14	-2.1322236
NUDT15	-0.3544154
NUDT16	-1.509737
NUDT16L1	-1.2673464
NUDT19	-2.0998454
NUDT2	-1.860363
NUDT21	-0.3478127
NUDT22	-1.8003678
NUDT3	-0.2289391
NUDT4	-1.2890911
NUDT5	-1.0844512
NUDT6	-2.4975343
NUDT7	-2.9963436
NUDT8	-3.2162042
NUDT9	-1.1116738
NUF2	-0.7107759
NUFIP1	-2.1886227
NUGGC	-1.0066955
NUP133	-0.3919611

NUP155	-1.7952328
NUP210	-0.6578379
NUP214	-0.8242788
NUP35	-0.1532207
NUP37	-0.4643145
NUP43	-0.3976564
NUP50	-0.4748902
NUP54	-0.2696023
NUP88	-0.5356593
NUPL1	-0.1500492
NUPL2	-0.823303
NUSAP1	-0.8225741
NUTF2	-0.6752052
NWD1	-0.3816361
NXNL1	-2.2599516
NXPE2	-4.765643
NXPE4	-0.8932598
NXPH1	-1.530308
NXT1	-0.2438865
NXT2	-0.7379289
NYAP1	-1.0174108
NYAP2	-1.1175425
NYNRIN	-1.1099508
OAF	-4.9229445
OARD1	-1.2051
OAS1B	-1.0696726
OAS1E	-0.8019831
OAS1G	-1.0862322
OAS1H	-1.5633144
OAT	-1.7786374
OAZ1	-1.370007
OAZ3	-2.6549501
OBOX3	-1.8358135
OBOX5	-1.6658204
OBP2A	-1.0995922
OCA2	-1.670434
OCEL1	-0.507081
OCIAD1	-1.0478387
OCIAD2	-3.9509757
OCSTAMP	-1.6512175
ODF1	-3.1901236
ODF3	-1.3644722
ODF3B	-0.8898411
ODF4	-2.5204737
OFCC1	-1.1221743

OGDH	-0.7552252
OGDHL	-3.5726848
OGFOD1	-1.713047
OGFOD2	-1.3652349
OGFOD3	-1.343843
OGFR	-0.2047119
OGG1	-0.8680978
OGT	-0.2588139
OIP5	-1.7402129
OIT3	-5.801208
OLA1	-0.6703262
OLFM2	-1.6325514
OLFML1	-4.090928
OLFML2A	-1.5412924
OLFML3	-0.3279097
OLFR10	-1.5528054
OLFR1006	-2.4926069
OLFR1012	-0.8602901
OLFR1015	-1.50192
OLFR1019	-1.4183702
OLFR1022	-1.2374082
OLFR1023	-1.2793057
OLFR1024	-4.8848724
OLFR1026	-0.8426981
OLFR1029	-0.996562
OLFR1032	-1.4782219
OLFR1036	-1.1690636
OLFR1037	-2.2038577
OLFR1039	-1.9062214
OLFR1042	-1.6800058
OLFR1044	-1.6647482
OLFR1049	-4.541914
OLFR1051	-1.6507735
OLFR1052	-1.001859
OLFR1054	-2.3431756
OLFR1055	-1.6979065
OLFR1056	-1.2454574
OLFR1057	-2.8650508
OLFR105-PS	-2.3488407
OLFR1061	-1.0559034
OLFR1065	-1.5113552
OLFR107	-1.6549165
OLFR1077-PS	-2.6126695
OLFR108	-1.3000867
OLFR1082	-1.6818132

OLFR1089	-2.0444927
OLFR1090	-1.0665917
OLFR1093	-1.6360903
OLFR1097	-1.4193351
OLFR1099	-2.3414965
OLFR1101	-2.069991
OLFR1102	-3.203248
OLFR1105	-1.6527102
OLFR111	-2.234106
OLFR1111	-1.7140884
OLFR1112	-1.6944766
OLFR1120	-1.4342494
OLFR1121	-1.604667
OLFR1128	-1.7036748
OLFR1132	-2.1935048
OLFR1135	-1.4545882
OLFR1136	-1.0510767
OLFR1138	-1.7652111
OLFR114	-1.1943576
OLFR1140	-0.811357
OLFR1141	-1.4747653
OLFR115	-1.4564736
OLFR1151	-1.2450771
OLFR1153	-2.975804
OLFR1154	-1.7395473
OLFR1155	-1.3036597
OLFR1156	-1.2839515
OLFR1160	-1.670013
OLFR1162	-1.41505
OLFR1166	-1.595427
OLFR1168	-0.6683221
OLFR1170	-0.9947774
OLFR1176	-1.5940921
OLFR1178	-1.713449
OLFR1179	-2.291292
OLFR118	-3.5353732
OLFR1181	-2.3077931
OLFR1183	-1.548454
OLFR1186	-2.2814708
OLFR1189	-2.3689766
OLFR119	-1.6554096
OLFR1195	-1.6923773
OLFR1196	-1.651217
OLFR1199	-2.1636167
OLFR1200	-1.7580967

OLFR1201	-1.653902
OLFR1202	-1.6400363
OLFR1204	-2.02693
OLFR1206	-2.9350977
OLFR1208	-1.6456637
OLFR1209	-1.6358337
OLFR121	-1.4694145
OLFR1213	-1.5941405
OLFR122	-1.0114994
OLFR1220	-1.956727
OLFR1221	-1.6028209
OLFR1223	-1.7364838
OLFR1226	-1.704715
OLFR1228	-1.5906973
OLFR1231	-2.037006
OLFR1238	-1.332449
OLFR1239	-1.6209636
OLFR124	-1.9931571
OLFR1241	-1.4034209
OLFR1245	-2.6863477
OLFR1246	-1.6742933
OLFR1247	-1.0445352
OLFR1248	-1.4611588
OLFR1249	-0.9838054
OLFR1250	-1.2810578
OLFR1254	-1.6393931
OLFR1255	-4.8917418
OLFR1257	-1.119077
OLFR1259	-1.3568044
OLFR126	-2.0683322
OLFR1260	-3.2313142
OLFR1262	-2.1724346
OLFR1263	-1.7715983
OLFR1265	-1.5377173
OLFR1271	-0.9415543
OLFR1275	-1.6921828
OLFR1277	-1.7580135
OLFR1278	-1.7355514
OLFR1279	-1.9985836
OLFR128	-4.198153
OLFR1284	-1.6507416
OLFR1286	-1.6020823
OLFR1287	-2.22551
OLFR1288	-3.8429954
OLFR129	-1.3840694

OLFR1290	-1.6719248
OLFR1294	-2.0732217
OLFR1297	-1.764123
OLFR1305	-3.5547144
OLFR1307	-1.5443282
OLFR1308	-3.2234364
OLFR131	-1.872283
OLFR1310	-1.7465425
OLFR1313	-1.1940508
OLFR1314	-0.6897326
OLFR1316	-1.3195641
OLFR1317	-4.823657
OLFR132	-2.1564412
OLFR1320	-0.9982677
OLFR1323	-1.3977463
OLFR1328	-2.0436385
OLFR1329	-1.5922525
OLFR133	-0.9825659
OLFR1330	-2.234448
OLFR1333	-1.0887837
OLFR1336	-1.0177698
OLFR1337	-1.9770205
OLFR134	-1.75282
OLFR1342	-1.417834
OLFR1348	-1.174412
OLFR1349	-1.2502856
OLFR135	-1.2879643
OLFR1350	-1.7268841
OLFR1356	-1.7955763
OLFR1357	-1.5916843
OLFR1358	-0.6741583
OLFR136	-1.6462069
OLFR1360	-1.763005
OLFR1361	-1.0436354
OLFR1362	-2.4120011
OLFR1366	-1.7264647
OLFR1370	-2.254207
OLFR1377	-1.7521024
OLFR1378	-0.6502285
OLFR138	-1.687103
OLFR1382	-0.9457221
OLFR1386	-1.754405
OLFR1388	-1.2891936
OLFR139	-1.751833
OLFR1390	-1.7325954

OLFR1393	-1.3217821
OLFR1395	-1.410842
OLFR140	-1.7368004
OLFR1414	-3.577897
OLFR1415	-1.1818779
OLFR1418	-1.2541449
OLFR1419	-1.4453995
OLFR142	-1.3640885
OLFR1420	-2.8155787
OLFR1425	-0.6100249
OLFR1426	-1.3618298
OLFR1427	-1.5888672
OLFR143	-1.4968364
OLFR1436	-1.152345
OLFR1441	-1.9781947
OLFR1444	-1.655314
OLFR1446	-1.1019282
OLFR1448	-3.6210012
OLFR1449	-1.2748013
OLFR1450	-1.2827139
OLFR1454	-0.9427517
OLFR1458	-1.6100066
OLFR1459	-1.2153244
OLFR146	-1.55987
OLFR1461	-1.0194969
OLFR1465	-2.2062407
OLFR1466	-1.0247893
OLFR1467	-2.2791517
OLFR147	-2.397781
OLFR1475	-1.723752
OLFR149	-0.429029
OLFR1496	-1.588238
OLFR1497	-2.0478032
OLFR1499	-1.5874066
OLFR1502	-1.3906455
OLFR1507	-2.0809395
OLFR1508	-1.5497718
OLFR1509	-2.6061091
OLFR1513	-2.257583
OLFR153	-3.1196527
OLFR1537	-2.9942932
OLFR157	-2.1681812
OLFR166	-2.9897735
OLFR17	-1.8430014
OLFR172	-1.6001985

OLFR176	-3.311645
OLFR178	-2.191394
OLFR186	-1.58501
OLFR19	-1.6290538
OLFR190	-1.245614
OLFR193	-1.6053383
OLFR194	-1.9191027
OLFR196	-1.6990943
OLFR197	-1.58917
OLFR198	-3.0654914
OLFR20	-3.7593074
OLFR202	-1.6215131
OLFR204	-1.5971341
OLFR205	-1.6642992
OLFR207	-1.1659253
OLFR208	-1.5135639
OLFR211	-1.0837338
OLFR220	-1.2285347
OLFR222	-1.6193204
OLFR223	-1.5181992
OLFR229	-1.7942572
OLFR23	-0.6772943
OLFR231	-1.5921156
OLFR237-PS1	-1.1797392
OLFR24	-3.0508144
OLFR242	-2.7386773
OLFR247	-2.295951
OLFR248	-3.508527
OLFR25	-1.816309
OLFR250	-2.993288
OLFR262	-1.1692746
OLFR263	-1.637258
OLFR27	-1.7547829
OLFR270	-1.2160003
OLFR272	-1.2738974
OLFR273	-1.00594
OLFR275	-2.97026
OLFR279	-0.9052491
OLFR282	-1.8493667
OLFR290	-2.267713
OLFR291	-1.458489
OLFR294	-1.6215696
OLFR295	-1.2321196
OLFR298	-1.5902538
OLFR299	-1.5966008

OLFR3	-1.7052693
OLFR303	-1.7188501
OLFR304	-1.6121321
OLFR307	-1.5411284
OLFR308	-1.5016668
OLFR309	-1.767201
OLFR31	-1.3596406
OLFR310	-2.1506922
OLFR311	-1.6464922
OLFR312	-1.1496599
OLFR314	-1.4489093
OLFR317	-2.3038077
OLFR318	-1.6835346
OLFR323	-1.3935657
OLFR325	-1.6234422
OLFR328	-1.736109
OLFR330	-1.996671
OLFR331	-1.7316749
OLFR332	-1.6047912
OLFR338	-1.0380621
OLFR340	-2.876648
OLFR342	-1.6163583
OLFR345	-1.7345228
OLFR346	-2.0985162
OLFR351	-2.2208786
OLFR353	-1.626759
OLFR354	-1.0550082
OLFR355	-3.301918
OLFR356	-1.1485672
OLFR357	-1.4381559
OLFR360	-1.3165674
OLFR365	-1.1877303
OLFR366	-1.593487
OLFR367-PS	-3.258974
OLFR371	-1.885946
OLFR372	-0.9299507
OLFR373	-1.0815408
OLFR390	-1.4789386
OLFR393	-1.6976674
OLFR397	-4.160079
OLFR398	-1.765069
OLFR402	-1.5970204
OLFR406	-1.2980845
OLFR412	-1.6202161
OLFR418-PS1	-1.8632638

OLFR419	-1.5849936
OLFR424	-4.3701763
OLFR426	-1.241734
OLFR434	-1.0155036
OLFR437	-1.7446189
OLFR44	-1.6067667
OLFR441	-1.7474558
OLFR447	-2.4400468
OLFR449	-1.0910549
OLFR45	-1.7001214
OLFR452	-1.6042562
OLFR453	-1.6012971
OLFR455	-0.8643081
OLFR460	-1.5794697
OLFR461	-1.597635
OLFR467	-2.992213
OLFR47	-1.0478535
OLFR474	-0.9040206
OLFR477	-1.162256
OLFR479	-1.6937594
OLFR48	-0.9662612
OLFR480	-2.95464
OLFR483	-2.0384338
OLFR487	-1.6379154
OLFR49	-1.7391996
OLFR491	-1.8236742
OLFR492	-1.606859
OLFR493	-1.677186
OLFR495	-0.2087593
OLFR5	-2.298149
OLFR50	-1.5452521
OLFR507	-1.381557
OLFR508	-2.2040753
OLFR509	-1.2349956
OLFR517	-1.2108593
OLFR52	-1.283968
OLFR520	-1.1899922
OLFR523	-1.7219219
OLFR527	-1.7074332
OLFR53	-1.3111985
OLFR531	-2.2073278
OLFR532	-2.3019116
OLFR536	-2.0135808
OLFR538	-3.413057
OLFR539	-1.1785336

OLFR54	-1.6159899
OLFR541	-1.4364161
OLFR543	-1.0735745
OLFR545	-2.2160702
OLFR547	-1.1401992
OLFR549	-2.2227182
OLFR55	-1.8486824
OLFR550	-3.0363302
OLFR551	-1.1734629
OLFR553	-1.3560429
OLFR554	-2.3690891
OLFR555	-2.4867182
OLFR556	-1.6857812
OLFR557	-1.6325448
OLFR558	-1.6505094
OLFR559	-0.9818342
OLFR560	-1.3589578
OLFR561	-1.0228586
OLFR564	-2.8612878
OLFR566	-1.4630833
OLFR568	-2.755107
OLFR569	-2.8618798
OLFR57	-1.6540852
OLFR571	-2.6208496
OLFR572	-1.2191339
OLFR577	-1.6840174
OLFR578	-1.6726391
OLFR586	-1.7263451
OLFR59	-1.677674
OLFR592	-2.108725
OLFR593	-2.353029
OLFR596	-1.8875575
OLFR600	-1.6896925
OLFR605	-2.3097157
OLFR606	-1.2247918
OLFR607	-1.6342797
OLFR611	-1.456346
OLFR612	-0.9750457
OLFR616	-1.8843694
OLFR619	-1.31709
OLFR62	-1.6845918
OLFR620	-1.5974507
OLFR623	-1.6223319
OLFR624	-1.6622772
OLFR629	-0.3693297

OLFR63	-1.1491599
OLFR630	-2.0071108
OLFR632	-2.027978
OLFR638	-3.648527
OLFR64	-1.2543802
OLFR640	-2.257419
OLFR643	-1.5937147
OLFR644	-2.5912395
OLFR645	-1.5227036
OLFR646	-1.5831583
OLFR648	-1.369344
OLFR649	-0.7742939
OLFR654	-3.4440844
OLFR655	-2.0097835
OLFR656	-2.2854743
OLFR657	-5.0400386
OLFR658	-1.0898652
OLFR659	-1.4834509
OLFR66	-1.5427623
OLFR661	-1.4741993
OLFR665	-1.291553
OLFR666	-1.6685269
OLFR667	-1.8679469
OLFR668	-1.6885381
OLFR67	-1.1699901
OLFR670	-2.8068798
OLFR672	-1.3670807
OLFR675	-1.5834134
OLFR676	-1.3493738
OLFR678	-2.1555266
OLFR68	-1.6368968
OLFR681	-1.2622128
OLFR684	-1.1491013
OLFR686	-2.0247867
OLFR687	-1.6849833
OLFR691	-1.6284566
OLFR693	-2.6207237
OLFR699	-1.4793153
OLFR70	-2.2511141
OLFR701	-1.9493866
OLFR702	-3.0256214
OLFR703	-2.2455888
OLFR704	-3.3470404
OLFR705	-1.9120965
OLFR707	-2.2598162

OLFR71	-1.1755171
OLFR713	-0.8806403
OLFR714	-0.9352672
OLFR715	-1.4132495
OLFR720	-2.463616
OLFR73	-0.8260136
OLFR730	-1.4008918
OLFR731	-1.0733252
OLFR733	-1.7479024
OLFR734	-2.4532502
OLFR735	-0.9806986
OLFR740	-4.430976
OLFR741	-1.436224
OLFR744	-1.9231219
OLFR747	-0.9736359
OLFR750	-1.8932161
OLFR763	-2.252062
OLFR766	-1.3911715
OLFR768	-1.3425617
OLFR769	-1.6318831
OLFR770	-2.3729846
OLFR771	-1.7763422
OLFR773	-2.4145494
OLFR776	-1.3889337
OLFR787	-1.6598277
OLFR790	-1.3056037
OLFR792	-0.9693627
OLFR794	-1.3797963
OLFR796	-2.7581491
OLFR798	-1.4020817
OLFR8	-1.1841819
OLFR800	-1.1413884
OLFR802	-1.3251698
OLFR805	-1.6291206
OLFR806	-2.1286848
OLFR808	-3.344232
OLFR813	-1.5070727
OLFR815	-1.2249615
OLFR816	-1.7354352
OLFR818	-0.8271551
OLFR819	-1.5931168
OLFR820	-1.6835375
OLFR825	-2.6557002
OLFR827	-1.8969548
OLFR829	-1.8466358

OLFR830	-1.188108
OLFR837	-1.4944942
OLFR845	-3.6440134
OLFR846	-2.4754083
OLFR847	-1.5182972
OLFR850	-1.6046376
OLFR851	-1.3241806
OLFR854	-1.4102058
OLFR860	-1.6277254
OLFR866	-1.6430316
OLFR867	-1.7308261
OLFR868	-1.3281841
OLFR869	-1.6684396
OLFR873	-3.985961
OLFR874	-1.2130003
OLFR876	-2.4995947
OLFR883	-1.1392274
OLFR884	-1.3836222
OLFR889	-2.841182
OLFR899	-1.7730091
OLFR9	-2.0034115
OLFR90	-1.702136
OLFR900	-1.5981312
OLFR902	-0.9976244
OLFR904	-2.1405275
OLFR905	-1.6924522
OLFR914	-1.762049
OLFR915	-1.338481
OLFR916	-1.7526
OLFR917	-2.4697676
OLFR918	-2.1432648
OLFR921	-0.3260794
OLFR923	-1.6181846
OLFR93	-1.6570792
OLFR930	-2.0090852
OLFR933	-2.7428029
OLFR934	-1.4524992
OLFR937	-1.7425218
OLFR947-PS1	-1.5004153
OLFR95	-2.6938312
OLFR952	-1.7378545
OLFR957	-2.299638
OLFR958	-1.6467795
OLFR959	-1.7001781
OLFR961	-1.6132169

OLFR965	-1.0038481
OLFR97	-0.2669625
OLFR971	-1.1754496
OLFR975	-1.7826955
OLFR976	-1.4514542
OLFR979	-1.0748253
OLFR980	-2.1550581
OLFR984	-1.4271562
OLFR987	-1.5947504
OLFR995	-1.7332983
OLIG1	-0.732615
OLIG2	-0.3370128
OMA1	-0.8568313
OMT2A	-1.413373
ONECUT1	-3.1650484
ONECUT2	-5.795756
OOSP2	-1.0816252
OPA1	-1.3091817
OPA3	-1.5567112
OPLAH	-2.5997791
OPN4	-0.3412976
OPRK1	-2.3151014
OPRM1	-0.3615294
OPTN	-0.9144087
ORAI1	-0.6221824
ORAI3	-0.8288798
ORAOV1	-0.4055748
ORC3	-0.9289341
ORC5	-1.505835
ORM1	-2.1260052
ORM2	-2.105173
ORM3	-3.0513577
ORMDL1	-1.671915
ORMDL2	-1.3295827
ORMDL3	-2.2287178
OS9	-1.0323901
OSBP2	-1.3655975
OSBPL1A	-3.4608006
OSBPL2	-0.4765282
OSCAR	-1.3481963
OSGEP	-1.4345651
OSGEPL1	-2.264821
OSGIN1	-4.17948
OST4	-1.5512295
OSTC	-1.0197396

OSTN	-0.9368546
OTC	-7.282975
OTOA	-1.6416292
OTOGL	-1.6752441
OTP	-2.2683957
OTUB1	-1.1046286
OTUB2	-0.8368187
OTUD3	-0.2332234
OTUD6B	-0.7398422
OTULIN	-1.4278226
OVCA2	-0.6514731
OXA1L	-1.133935
OXLD1	-3.2884498
OXNAD1	-2.3462315
OXR1	-0.9246836
OXSM	-2.3197086
OXT	-1.4101567
P2RX4	-0.598958
P2RX6	-2.1431718
P2RY1	-1.3640225
P2RY12	-0.8094497
P2RY13	-0.5856967
P2RY4	-1.1893961
P4HB	-2.6704335
PABPC1L2B-F	-1.9392858
PABPC2	-1.7387938
PABPC4L	-1.9064634
PABPC5	-1.0344687
PABPC6	-2.7581134
PABPN1L	-0.9793921
PACRGL	-0.5969148
PACSIN3	-2.5723372
PADI2	-0.3668816
PADI3	-0.7749314
PADI6	-0.9528706
PAFAH1B3	-0.378849
PAFAH2	-3.3273797
PAGR1A	-0.9205232
PAH	-8.738743
PAICS	-1.8372374
PAIP1	-0.339603
PAIP2	-0.4041414
PAK1	-0.3569276
PAK1IP1	-0.4865322
PAK4	-0.8306956

PALB2	-1.0705149
PALLD	-1.5867186
PALM	-0.6076241
PALM3	-3.0217566
PALMD	-1.9149795
PAM16	-0.9514866
PAMR1	-0.7823052
PAN2	-0.9017744
PANK1	-6.9947624
PANK4	-0.3081927
PANX2	-1.5952408
PANX3	-1.7239826
PAOX	-2.9759588
PAPD4	-0.4585867
PAPD5	-1.2275419
PAPD7	-0.9506092
PAPLN	-0.3413727
PAPOLA	-0.2966795
PAPOLB	-1.3109598
PAPSS1	-0.4691401
PAPSS2	-4.86663
PAQR3	-1.617866
PAQR4	-0.4993405
PAQR7	-2.0285578
PAQR9	-6.6644764
PARD3	-0.2618055
PARD3BOS1	-1.6037328
PARD6A	-1.2975419
PARK2	-1.0805361
PARK7	-2.105641
PARN	-1.2524023
PARP1	-1.2513528
PARP12	-0.3851457
PARP16	-2.9217594
PARP6	-0.4814897
PARPBP	-1.3278632
PARS2	-1.8709686
PARVA	-0.5631785
PASK	-0.5461047
PATE2	-1.6312013
PATE4	-1.7006934
PATL2	-1.6557546
PAX2	-2.994431
PAX4	-1.067379
PAX6	-1.4659591

PAX8	-1.6962125
PBDC1	-0.3957343
PBK	-2.0137568
PBLD1	-0.8814006
PBLD2	-8.488889
PBSN	-1.3587334
PBX2	-0.3370976
PCBD1	-6.205677
PCBD2	-1.7010255
PCBP1	-0.4496355
PCBP4	-2.2459846
PCCA	-2.50496
PCCB	-1.6330459
PCDH1	-1.7019148
PCDH10	-0.2126114
PCDH11X	-1.1401892
PCDH12	-2.9769883
PCDH15	-1.6000292
PCDH20	-1.1270299
PCDH9	-1.704361
PCDHA12	-0.380264
PCDHA9	-2.4797962
PCDHB12	-1.5195372
PCDHB13	-0.8714185
PCDHB2	-1.6174736
PCDHB5	-1.7315934
PCED1A	-1.9064264
PCGF1	-0.4779129
PCGF6	-1.418652
PCID2	-0.9605773
PCK1	-3.4061217
PCLO	-0.7403283
PCMT1	-0.4090691
PCMTD1	-1.363462
PCMTD2	-0.5073886
PCNP	-0.4667301
PCNX	-0.4262881
PCNXL3	-0.7992702
PCOLCE	-0.6316233
PCP2	-1.5976999
PCP4L1	-3.2169738
PCSK1N	-1.2117989
PCSK4	-3.9111333
PCSK6	-4.584473
PCSK7	-1.2517695

PCSK9	-6.417041
PCTP	-5.9252
PCX	-3.1862016
PCYOX1	-2.5698028
PCYT2	-4.4954357
PDAP1	-1.0661988
PDCD2	-1.4152665
PDCD2L	-0.6307879
PDCD4	-0.6455531
PDCD5	-0.3971329
PDCD6	-1.0689149
PDCD7	-0.5003052
PDCL	-0.4695997
PDCL2	-1.6493356
PDDC1	-1.7115221
PDE10A	-0.6216991
PDE1C	-0.9700136
PDE2A	-1.7369323
PDE3B	-2.5078168
PDE4A	-0.5531373
PDE4DIP	-0.7517667
PDE6A	-0.9956646
PDE6C	-2.376446
PDE6G	-1.0667548
PDE8A	-1.3003917
PDE9A	-1.4216471
PDF	-1.9482379
PDGFC	-1.8944368
PDHA1	-0.4200468
PDHA2	-1.6362667
PDHB	-1.2518301
PDHX	-1.1275334
PDIA4	-0.2973342
PDIA5	-1.9394107
PDIA6	-1.2021475
PDIK1L	-0.5238059
PDILT	-6.8848395
PDK1	-2.4246888
PDK2	-3.1513214
PDLIM4	-0.5750046
PDP2	-3.0279682
PDPK1	-0.4467149
PDS5B	-0.1939778
PDSS1	-0.5574069
PDSS2	-1.7933159

PDX1	-1.502634
PDXDC1	-1.0624738
PDXK	-1.8997092
PDXK-PS	-1.8094504
PDXP	-3.8821309
PDYN	-1.0943513
PDZD11	-0.4984613
PDZD7	-1.133879
PDZD9	-1.1401145
PDZK1	-6.2908945
PDZRN3	-0.3773556
PDZRN4	-1.2937081
PEA15B	-1.0817034
PEBP1	-1.0284829
PECR	-6.0664573
PEF1	-0.9907842
PEG10	-1.4014709
PEG12	-0.5616491
PELI2	-1.0490546
PELI3	-2.2651675
PELO	-0.5670767
PELP1	-0.730454
PEMT	-6.919945
PEO1	-2.877186
PEPD	-2.2557015
PER2	-0.5744972
PER3	-2.1403193
PERP	-2.1551328
PET100	-1.2752798
PET112	-3.710955
PEX1	-3.0034242
PEX10	-1.3599572
PEX11A	-2.1958737
PEX11B	-1.4806862
PEX11G	-3.5563736
PEX12	-1.8309388
PEX13	-1.7860875
PEX14	-2.430528
PEX16	-3.8022127
PEX19	-2.9041977
PEX2	-1.6240206
PEX26	-3.0397472
PEX3	-1.8763943
PEX5	-2.4516134
PEX6	-2.4216418

PEX7	-2.973826
PFDN1	-0.6649361
PFDN5	-0.4619165
PFKFB1	-5.556431
PFKL	-1.0259433
PFKM	-1.4286995
PFN1	-0.1898561
PFN3	-2.2815285
PGAM1	-1.2304258
PGAM5	-0.4246721
PGAP2	-0.843142
PGBD1	-1.296541
PGC	-1.1416306
PGK1	-1.5793266
PGLS	-1.3348742
PGLYRP2	-5.2232504
PGLYRP4	-0.8508177
PGM2	-3.068275
PGM3	-0.9535861
PGP	-0.8514643
PGPEP1	-3.500543
PGPEP1L	-1.150919
PGRMC1	-3.552981
PGRMC2	-0.8954892
PHACTR3	-1.3234925
PHAX	-0.6233597
PHB	-2.4388657
PHB2	-2.1337023
PHF10	-0.6632028
PHF11C	-0.2645447
PHF14	-0.2533312
PHF21A	-0.2396941
PHF5A	-1.1886768
PHF7	-1.7836432
PHKA2	-2.0432944
PHKB	-0.6395464
PHKG2	-0.4928646
PHLDA2	-2.9745605
PHLDA3	-0.3288093
PHLDB3	-1.8258681
PHLPP1	-1.4242694
PHOSPHO2	-1.0824699
PHPT1	-1.1629753
PHTF2	-0.2449641
PHYH	-3.8214774

PHYHD1	-0.8394342
PHYHIPL	-1.1325159
PHYKPL	-1.7582016
PI4K2A	-1.0850015
PI4KB	-0.5655088
PIAS2	-0.4203072
PIAS4	-1.1245666
PICK1	-1.1598017
PID1	-1.9301243
PIDD1	-1.3475654
PIGC	-1.1363211
PIGF	-1.282495
PIGH	-0.5983138
PIGK	-1.5754266
PIGL	-1.5343165
PIGN	-0.4564276
PIGO	-1.5685554
PIGP	-1.3339472
PIGQ	-0.9813952
PIGR	-0.8781529
PIGS	-0.8726807
PIGT	-1.8408523
PIGU	-1.2196174
PIGV	-0.8713522
PIGX	-1.2026749
PIGYL	-1.8599467
PIGZ	-1.9199328
PIH1D1	-1.842113
PIK3C2G	-4.297484
PIK3R2	-1.6439347
PIK3R4	-0.633914
PIN1	-0.7308669
PIN1RT1	-0.710516
PIN4	-0.2497344
PINK1	-2.4614983
PIP	-1.0143585
PIP4K2B	-0.7595806
PIP5K1BOS	-1.6053698
PIP5KL1	-1.4349608
PIPOX	-7.940505
PIR	-2.1899014
PIRB	-0.4642096
PIRT	-1.4057691
PISD	-0.8190851
PISD-PS1	-0.4434652

PISD-PS2	-1.826952
PITHD1	-0.5294223
PITPNB	-0.7704372
PITPNM2	-0.2372003
PITPNM2OS1	-0.8967369
PITRM1	-0.7768998
PITX2	-1.0514221
PIWIL1	-1.6213908
PJA1	-1.6992784
PKD1L1	-3.222382
PKD2L1	-2.4018016
PKD2L2	-0.3733344
PKDCC	-2.863834
PKDREJ	-1.3358045
PKHD1	-0.6371405
PKHD1L1	-1.923142
PKLR	-6.2244253
PKMYT1	-0.3981271
PKP2	-1.4642525
PKP3	-1.0586257
PKP4	-0.6139131
PLA1A	-1.8877316
PLA2G10	-0.9539614
PLA2G12A	-1.0711508
PLA2G12B	-5.703437
PLA2G15	-1.9384122
PLA2G16	-0.6029997
PLA2G2D	-0.5778568
PLA2G3	-0.9464941
PLA2G4B	-0.7969637
PLA2G4C	-1.463438
PLA2G6	-2.8266115
PLA2R1	-0.3675482
PLAA	-0.7543511
PLBD1	-0.8276372
PLBD2	-1.6101356
PLCB1	-1.1410115
PLCE1	-0.6698349
PLCG1	-0.1961608
PLCL2	-1.0131269
PLCXD1	-1.2999294
PLCXD2	-3.809668
PLCZ1	-1.5142817
PLD1	-0.2279611
PLD4	-2.0843978

PLD6	-3.8661704
PLEK2	-1.9469669
PLEKHA5	-1.1824195
PLEKHA6	-3.3172889
PLEKHA7	-0.920131
PLEKHA8	-1.7399564
PLEKHD1OS	-2.3987656
PLEKHF1	-1.4165378
PLEKHG1	-2.947503
PLEKHG3	-0.8364854
PLEKHG6	-2.6350777
PLEKHH2	-1.2099731
PLEKHH3	-1.2865047
PLEKHJ1	-1.1550319
PLEKHN1	-1.4948466
PLG	-4.6598654
PLIN2	-1.6735353
PLIN3	-1.2255707
PLIN5	-3.5209484
PLK1	-0.8115521
PLK3	-0.4404006
PLK4	-0.3971317
PLK5	-2.0305474
PLLP	-3.0341687
PLOD1	-0.7258036
PLP1	-0.412425
PLS1	-0.7263191
PLS3	-0.6854863
PLSCR2	-2.7721279
PLSCR4	-0.5868459
PLTP	-1.5918164
PLXNA3	-2.4662614
PLXNB1	-1.6133299
PLXNB2	-1.3539915
PLXNB3	-1.0034175
PLXNC1	-1.6951046
PLXND1	-0.260541
PM20D1	-6.2348347
PMCH	-1.7550673
PMEL	-1.936343
PMF1	-0.9967365
PMIS2	-1.2255449
PMM2	-1.9000278
PMP2	-1.3193209
PMPCA	-1.2362285

PMPCB	-2.1880636
PMS1	-0.9113636
PMVK	-0.5808168
PNKD	-2.7249508
PNKP	-0.2861276
PNLDC1	-2.0601537
PNLIP	-1.1332798
PNLIPRP2	-3.0237944
PNMA1	-1.6133687
PNMA3	-1.1107864
PNMT	-2.1263049
PNO1	-0.833478
PNP	-1.3999944
PNP2	-0.8900981
PNPLA1	-4.146516
PNPLA3	-4.508005
PNPLA5	-5.8285313
PNPLA6	-0.9528775
PNPLA7	-4.3738565
PNPO	-3.1388364
PNPT1	-0.6611366
PNRC2	-0.278182
PODN	-0.3441091
PODNL1	-1.4821751
POFUT1	-1.0664387
POFUT2	-0.2410359
POGLUT1	-0.4066567
POGZ	-0.18472
POLA1	-0.747541
POLB	-0.2240963
POLD1	-0.5378022
POLD2	-1.1334572
POLD4	-1.658792
POLDIP2	-2.3300633
POLE3	-0.3350363
POLE4	-1.5205884
POLG	-1.3313594
POLG2	-1.7839024
POLI	-1.1088219
POLL	-1.5666127
POLN	-0.6788712
POLQ	-0.3430448
POLR1A	-0.4358077
POLR1B	-1.0227334
POLR1C	-0.2854924

POLR1D	-0.4830241
POLR2B	-0.7613721
POLR2C	-0.3017659
POLR2D	-0.7484369
POLR2E	-0.7383289
POLR2F	-0.8541188
POLR2G	-0.5715156
POLR2H	-1.1118569
POLR2I	-1.2966604
POLR2J	-0.9179115
POLR2K	-0.5678763
POLR2L	-1.5372896
POLR2M	-0.4276943
POLR3A	-1.4673171
POLR3B	-0.6396756
POLR3C	-0.9187541
POLR3F	-0.2877407
POLR3G	-2.0642126
POLR3H	-1.2333505
POLRMT	-1.6917171
POM121L2	-2.028472
POMGNT1	-0.3141141
POMGNT2	-1.3389325
POMK	-2.070836
POMP	-0.8300991
POMT1	-3.0813963
POMT2	-1.7309318
PON1	-4.819636
PON3	-0.8896227
POP1	-0.7752237
POP5	-2.246726
POP7	-0.5596929
POR	-1.0974946
POT1A	-0.5398126
POU1F1	-1.6441696
POU3F2	-1.934191
POU3F3	-1.3047152
POU3F4	-2.4411047
POU4F3	-1.3762355
POU6F1	-0.3388529
PPA1	-2.1850123
PPA2	-2.2591152
PPAP2B	-1.6115403
PPAP2C	-1.8298645
PPAPDC1B	-0.6243005

PPAPDC3	-1.3610902
PPARA	-4.4741683
PPARG	-1.009553
PPARGC1A	-0.9260759
PPARGC1B	-3.3872905
PPAT	-0.7477441
PPCDC	-1.0750191
PPCS	-2.8351264
PPDPF	-2.4806452
PPEF1	-1.1254125
PPFIA2	-1.3378894
PPFIA3	-0.3678889
PPFIA4	-1.6088152
PPFIBP2	-0.9251747
PPIB	-2.0356593
PPIE	-1.3480012
PPIF	-3.3694854
PPIFOS	-1.5359397
PPIH	-0.6928451
PPIL1	-0.8860345
PPIL2	-1.2582479
PPIL3	-0.9292135
PPIP5K2	-1.0949898
PPM1A	-0.9765501
PPM1B	-0.885313
PPM1G	-0.2788262
PPM1K	-0.9774294
PPM1L	-1.2495766
PPME1	-0.607614
PPNR	-0.5837317
PPOX	-2.2622962
PPP1CA	-1.2834272
PPP1CB	-0.6613231
PPP1R11	-1.0611329
PPP1R16A	-1.3665276
PPP1R18OS	-0.8568099
PPP1R1B	-2.25731
PPP1R2-PS9	-1.1478472
PPP1R35	-1.7205052
PPP1R37	-2.3680577
PPP1R3B	-1.9778686
PPP1R3C	-4.2126327
PPP1R3E	-1.4057627
PPP1R3FOS	-1.6665206
PPP1R3G	-0.8776088

PPP1R7	-0.468709
PPP2R1A	-0.4045358
PPP2R2B	-0.9954667
PPP2R2COS	-1.757653
PPP2R2D	-0.6014562
PPP2R3A	-0.3787062
PPP2R3C	-0.9579063
PPP2R4	-2.2956195
PPP2R5A	-1.4081998
PPP2R5D	-0.9806042
PPP2R5E	-1.1972859
PPP4C	-0.5984054
PPP4R4	-2.3607104
PPP5C	-1.0507839
PPP6C	-0.3337545
PPP6R2	-0.3731055
PPT2	-2.5389624
PPTC7	-0.1576986
PPY	-0.7164116
PQBP1	-1.229186
PQLC1	-1.8250227
PQLC2	-2.7659616
PRADC1	-1.8931298
PRAME	-2.2415946
PRAMEL	-1.174314
PRAMEL4	-3.0841694
PRAMEL7	-1.3538713
PRAP1	-0.6643429
PRB1	-1.2011752
PRC1	-0.7649298
PRDM10	-0.696564
PRDM12	-1.1889081
PRDM13	-1.0038495
PRDM14	-1.2028122
PRDM9	-1.4412079
PRDX1	-1.88308
PRDX2	-1.8528814
PRDX3	-1.9261827
PRDX4	-3.0349622
PREB	-0.8909001
PRELID1	-1.185926
PRELID2	-1.4738388
PREP	-0.4709311
PREPL	-1.5046215
PRG2	-3.5154095

PRG3	-4.1785965
PRG4	-2.4316335
PRH1	-2.2136478
PRICKLE4	-2.6633854
PRIM1	-0.932651
PRIM2	-0.3349311
PRIMA1	-2.3521438
PRKAA2	-2.9702973
PRKAB1	-0.2185116
PRKACA	-0.9616559
PRKACB	-0.4740286
PRKAG1	-1.2165976
PRKAG2	-2.4311519
PRKAG2OS1	-1.4217134
PRKAR1A	-0.719954
PRKAR2A	-1.5306249
PRKCG	-1.9015539
PRKCSH	-1.3234987
PRKCZ	-1.2138624
PRKD3	-0.3440318
PRKDC	-0.4093497
PRKRA	-2.0979605
PRKRIP1	-0.3091812
PRKRIR	-1.0882578
PRL2A1	-1.611074
PRL2B1	-1.264085
PRL2C5	-5.807377
PRL3A1	-1.5405314
PRL3C1	-1.6389132
PRL3D2	-0.9041345
PRL4A1	-2.0457404
PRL5A1	-1.6815171
PRL6A1	-1.1551638
PRL7A1	-1.0071757
PRL7B1	-1.6414881
PRL7C1	-3.3708885
PRL7D1	-1.6187191
PRLR	-6.1563864
PRM1	-0.6547458
PRM3	-1.7199306
PRMT10	-0.83355
PRMT3	-0.5768004
PRMT7	-0.5926619
PRN	-2.4985456
PROB1	-0.7999747

PROC	-6.468449
PROCA1	-1.1488383
PRODH	-5.1670127
PRODH2	-7.875861
PROK1	-3.3133948
PROM2	-2.5843956
PRORSD1	-0.7910981
PROS1	-0.7505856
PROSC	-1.3640051
PROSER1	-0.7635071
PROSER2	-2.2774892
PROX1	-3.8162684
PROZ	-4.997785
PRP2	-1.5052905
PRPF19	-2.157752
PRPF40B	-0.914216
PRPF8	-0.7855864
PRPMP5	-1.1962671
PRPS1	-0.4135757
PRPS1L3	-1.1391058
PRPS2	-0.6026094
PRPSAP1	-1.4507465
PRPSAP2	-0.684176
PRR12	-1.2742555
PRR14	-0.1677985
PRR14L	-0.293324
PRR16	-3.6492393
PRR19	-1.0259914
PRR22	-0.7238519
PRR23A	-1.7103598
PRR27	-2.5061111
PRR33	-0.9524894
PRR5	-1.0107193
PRRC1	-0.1928425
PRRC2A	-0.9568477
PRRG2	-1.9600387
PRRT2	-1.1713707
PRRT3	-0.8140135
PRRXL1	-1.0708694
PRSS1	-1.1390033
PRSS21	-1.1507726
PRSS28	-0.8417504
PRSS29	-1.5036604
PRSS35	-1.3505747
PRSS36	-2.3492773

PRSS39	-1.6031585
PRSS40	-1.5063355
PRSS41	-1.1842387
PRSS42	-1.0806272
PRSS44	-2.1116772
PRSS45	-1.2526054
PRSS46	-1.3509679
PRSS47	-1.5925283
PRSS48	-0.2612011
PRSS51	-1.789458
PRSS54	-1.6065383
PRSS57	-1.6016192
PRSS58	-1.5862846
PRTN3	-2.645086
PRUNE	-1.2976162
PSAP	-0.8227596
PSD2	-1.0343807
PSD3	-1.519484
PSEN2	-2.3047547
PSENEN	-0.7002459
PSG18	-2.2690463
PSG20	-2.002236
PSG22	-1.0588007
PSG23	-4.0079303
PSG25	-1.6619787
PSG28	-1.7670577
PSKH1	-1.630516
PSMA1	-0.4574924
PSMA2	-0.3501406
PSMA3	-0.6261535
PSMA4	-0.7301917
PSMA5	-1.137013
PSMA6	-0.6612387
PSMA7	-0.9301271
PSMB1	-1.4279723
PSMB10	-0.8233357
PSMB11	-1.6718986
PSMB3	-0.9476323
PSMB4	-1.00277
PSMB5	-1.0584435
PSMB6	-1.4228463
PSMB7	-1.9515634
PSMB8	-0.6302724
PSMB9	-1.2438011
PSMC1	-0.6762548

PSMC2	-0.5614686
PSMC3	-0.8735957
PSMC4	-0.9762278
PSMC5	-0.5607176
PSMC6	-0.7596898
PSMD1	-0.4489589
PSMD10	-0.2052593
PSMD11	-1.2968764
PSMD12	-0.4557118
PSMD13	-1.3088303
PSMD2	-0.5207925
PSMD3	-0.7177777
PSMD4	-0.8581958
PSMD5	-1.4363918
PSMD6	-0.9478078
PSMD7	-0.6681347
PSMD9	-2.0814586
PSME1	-1.0735326
PSME2B	-0.5230522
PSME3	-1.1416025
PSMF1	-0.9611177
PSMG1	-1.8610401
PSMG2	-1.1705694
PSMG3	-1.4084821
PSMG4	-1.2012272
PSPH	-1.1987371
PSRC1	-1.7715032
PSTK	-2.598824
PTCD1	-1.036706
PTCD2	-2.2721753
PTCD3	-1.3666134
PTCH1	-0.9543822
PTCH2	-0.6745958
PTCHD1	-1.6230044
PTCHD2	-1.0614674
PTDSS1	-1.0458379
PTDSS2	-0.9560556
PTEN	-0.827342
PTER	-5.5487385
PTF1A	-1.9290426
PTGDR2	-1.1413717
PTGDS	-0.5128779
PTGES	-0.7341504
PTGES2	-1.176517
PTGES3	-0.5498333

PTGR1	-0.5863123
PTGR2	-2.0569282
PTGS1	-1.4138346
PTH1R	-3.8002412
PTH2	-0.9059012
PTK2B	-0.9847741
PTK6	-1.7410209
PTMS	-2.2763205
PTOV1	-1.2937641
PTP4A1	-0.3881283
PTP4A2	-0.3733773
PTP4A3	-0.9852715
PTPLA	-1.2289677
PTPLAD1	-2.5218587
PTPLAD2	-1.0378363
PTPLB	-1.880898
PTPMT1	-1.599999
PTPN20	-1.8116517
PTPN3	-1.2721293
PTPN5	-0.4965968
PTPN9	-0.9267025
PTPRD	-3.2097547
PTPRF	-0.6278076
PTPRJ	-0.1535173
PTPRQ	-2.661809
PTPRV	-1.0503142
PTRH2	-1.4576192
PTRHD1	-1.4194069
PTS	-2.1235662
PTTG1	-0.2855091
PTTG1IP	-0.6862874
PTX4	-1.2914495
PUF60	-0.8230724
PURA	-0.45927
PURG	-0.6116519
PUS1	-1.4887872
PUS10	-1.7279582
PUSL1	-0.7800756
PVRL1	-2.4648323
PVRL2	-0.7789426
PVRL3	-0.2339993
PWP2	-0.5942478
PXMP2	-5.5167484
PXMP4	-3.7784748
PXT1	-0.6614323

PXYLP1	-2.2192936
PYCRL	-2.9891744
PYDC3	-1.0648975
PYGL	-2.3881197
PYROXD1	-0.4415009
PYROXD2	-4.936965
PYURF	-3.0169108
PZP	-1.9980564
QARS	-0.5893722
QDPR	-4.894441
QPCT	-2.7011738
QPCTL	-0.8076105
QPRT	-5.576355
QRFP	-0.7110992
QRFPR	-1.5656672
QRICH2	-1.2411077
QRSL1	-0.7529488
QSOX1	-1.541132
QTRT1	-0.6167324
R3HCC1	-0.890316
R3HDM1	-0.1596708
R3HDM2	-0.7171154
R3HDML	-0.8964043
RAB1	-0.3099566
RAB11A	-0.2117462
RAB11B	-0.3439813
RAB11FIP2	-0.7749243
RAB11FIP3	-0.3110023
RAB11FIP4	-1.554785
RAB11FIP4OS	-1.3748233
RAB13	-0.2087498
RAB14	-0.941473
RAB17	-4.8060966
RAB18	-0.6183596
RAB1B	-1.0728412
RAB21	-0.8470597
RAB22A	-0.3095655
RAB23	-0.5013163
RAB24	-1.2959809
RAB30	-2.5457919
RAB32	-0.8731365
RAB39	-1.9986105
RAB39B	-1.650058
RAB3B	-2.9510458
RAB3D	-0.2514968

RAB3IL1	-1.1342902
RAB3IP	-0.3813677
RAB40B	-1.277411
RAB40C	-1.0100064
RAB42	-2.6281748
RAB43	-1.116097
RAB4A	-3.278551
RAB4B	-0.2002473
RAB5B	-0.6393113
RAB5C	-0.5203228
RAB7L1	-1.5365977
RAB8A	-0.7986589
RAB9	-1.3229685
RABEP1	-0.7798119
RABGGTA	-2.0426378
RABGGTB	-0.4804435
RABL3	-2.0048797
RABL6	-0.3806796
RAD1	-0.3926463
RAD17	-0.3492017
RAD21L	-1.8475585
RAD23A	-0.9914963
RAD23B	-0.4138002
RAD50	-0.3798533
RAD51	-0.7849245
RAD51AP1	-0.4128594
RAD51AP2	-1.6119215
RAD51B	-5.240256
RAD51C	-1.094943
RAD51D	-1.6536708
RAD52	-0.2448988
RAD54B	-0.9953637
RAD54L2	-1.4305701
RAD9A	-0.3897095
RAET1A	-5.174647
RAET1B	-2.675582
RAET1E	-2.8525982
RAF1	-0.775476
RAG1	-1.4663696
RAG2	-1.6651769
RAI2	-0.4990284
RALGPS1	-0.9247131
RALGPS2	-1.926467
RALY	-0.9363833
RALYL	-1.3241999

RAMP1	-0.3561852
RANBP1	-0.3249908
RANBP10	-1.9533992
RANBP17	-1.2873392
RANBP3	-0.2620831
RANBP3L	-2.0977347
RANBP6	-0.7472353
RANGAP1	-0.3377709
RAPGEF1	-0.6644287
RAPGEF4	-3.0688899
RAPH1	-1.6652641
RAPSN	-0.4862504
RARB	-1.8122284
RARRES1	-6.6878157
RARRES2	-2.2034383
RARS2	-0.8036599
RASA1	-0.6943893
RASAL1	-2.675127
RASGRF2	-1.8921709
RASGRP2	-2.7746873
RASL2-9	-0.6001434
RASSF3	-0.815464
RASSF4	-1.1772795
RASSF6	-2.3576016
RASSF7	-1.0987453
RAVER1	-2.125557
RBBP5	-0.7468767
RBBP7	-0.7614017
RBBP9	-1.0053496
RBCK1	-0.9927077
RBFA	-2.1237178
RBFOX2	-0.6782675
RBFOX3	-3.2568328
RBKS	-2.4431953
RBL2	-1.3259792
RBM10	-0.397068
RBM12B1	-1.4568341
RBM20	-0.9226015
RBM34	-0.5941424
RBM4	-1.5535207
RBM44	-1.6914587
RBM45	-0.1511607
RBM46OS	-0.2404537
RBM47	-0.7444005
RBM4B	-1.1086798

RBM6	-0.2426519
RBMXL2	-1.7044117
RBMY	-3.0744874
RBP1	-4.4128623
RBP4	-4.546902
RBPMS2	-4.578865
RCAN2	-1.879056
RCBTB1	-1.644074
RCBTB2	-1.6526141
RCC1	-0.4709139
RCCD1	-0.65411
RCHY1	-0.2831531
RCL1	-2.7594452
RCN3	-0.6583352
RCVRN	-1.5033946
RDH10	-1.4770212
RDH11	-2.7452912
RDH13	-0.6121156
RDH14	-1.6291928
RDH16	-8.445192
RDH18-PS	-3.2888906
RDH19	-1.1262257
RDH5	-4.708381
RDH7	-6.2173257
RDH8	-0.9420171
RDH9	-5.558666
RDM1	-1.2275238
RDX	-0.9325156
REC114	-3.3192046
REC8	-0.5114651
RECQL4	-1.3109696
RECQL5	-1.2008283
REDRUM	-0.7626691
REEP3	-0.8454499
REEP4	-0.2091546
REEP6	-5.0494776
REG2	-1.3538799
REG3D	-1.9706988
REG4	-1.8986535
REL	-2.524313
RELL2	-0.8212962
RELN	-2.7050872
REM1	-0.3602753
REM2	-2.1132972
REN1	-0.2658141

REN2	-0.9322667
REPIN1	-2.2234225
REPS2	-2.0253496
RER1	-1.6044192
RERGL	-0.9599876
RETSAT	-4.317603
REV1	-0.5321295
REX2	-1.1856153
REXO2	-0.4869952
REXO4	-0.3763356
RFC2	-1.4433966
RFC3	-0.9873366
RFC4	-0.2949734
RFC5	-1.2290206
RFESD	-1.656404
RFFL	-0.4138331
RFK	-0.227159
RFNG	-0.8088226
RFT1	-1.1117063
RFWD2	-0.857162
RFWD3	-0.5254717
RFX7	-2.016899
RFXANK	-1.9012089
RFXAP	-1.174233
RGAG1	-1.1301067
RGL1	-0.5843353
RGL2	-0.3129582
RGL3	-2.8861713
RGN	-8.552164
RGS16	-2.615409
RGS20	-1.1492224
RGS5	-1.9407625
RGS7	-1.5841465
RGS7BP	-1.1244483
RGS8	-1.9169612
RGSL1	-1.36499
RHBDD2	-0.3122025
RHBDD3	-2.3407292
RHBDL2	-2.0344203
RHBG	-3.8909192
RHEB	-0.2157512
RHO	-1.4277873
RHOBTB1	-1.5280066
RHOBTB3	-1.1574059
RHOD	-3.2303243

RHOH	-1.017098
RHOT1	-0.6738491
RHOT2	-1.578321
RHOU	-1.1826277
RHOX10	-1.6958287
RHOX11	-1.7395179
RHOX13	-1.3966134
RHOX2C	-1.0942822
RHOX2F	-2.0856752
RHOX2H	-1.4422987
RHOX3E	-2.5676913
RHOX3F	-0.5195255
RHOX4A	-1.6522155
RHOX4G	-2.0089254
RHOX5	-3.2271264
RHOX8	-0.5713177
RHOX9	-1.0373147
RHPN2	-1.558116
RIC3	-0.3532405
RIC8B	-0.3128071
RILP	-3.4630237
RIMKLB	-1.7196906
RIMS2	-0.4232681
RIMS3	-1.7679305
RIMS4	-4.512641
RING1	-1.5623713
RINT1	-0.3101113
RIOK2	-0.5388117
RIOK3	-0.2093306
RIPPLY1	-1.0373623
RIT2	-0.8579795
RITA1	-1.1995146
RMDN1	-2.7429743
RMDN2	-1.9316316
RMDN3	-2.1806016
RMI1	-1.019979
RMI2	-1.8171866
RMND1	-3.2739868
RMND5A	-0.6961708
RMND5B	-0.7829881
RN28S1	-0.9201889
RN45S	-0.5400105
RNASE12	-1.0073886
RNASE13	-1.720356
RNASE4	-2.9849677

RNASE9	-0.8723488
RNASEH1	-0.5481892
RNASEH2A	-1.7150228
RNASEH2B	-0.7630701
RNASEH2C	-1.9340038
RNASEK	-1.3011003
RNASET2A	-0.8065305
RND2	-4.5470285
RNF10	-1.1832824
RNF103	-1.8255424
RNF11	-0.4868774
RNF112	-1.3199203
RNF113A1	-0.3682351
RNF115	-0.5743203
RNF121	-0.7450628
RNF123	-2.9578886
RNF125	-0.9958577
RNF126	-0.5091009
RNF128	-3.3814278
RNF13	-1.6385455
RNF133	-1.0385194
RNF135	-2.061913
RNF138	-0.1564207
RNF138RT1	-4.1651363
RNF139	-1.3352561
RNF14	-0.6515989
RNF141	-1.3080568
RNF144B	-0.7824402
RNF145	-0.5702591
RNF148	-2.630008
RNF149	-0.3127995
RNF151	-1.7036965
RNF152	-4.9628744
RNF165	-1.4802284
RNF167	-1.3670473
RNF169	-0.7566876
RNF17	-1.6378057
RNF170	-1.8976936
RNF181	-1.3646798
RNF183	-1.0882459
RNF185	-0.9244032
RNF186	-0.1986146
RNF187	-0.9978981
RNF212	-0.9908814
RNF214	-0.3769922

RNF217	-0.9878078
RNF222	-2.4272006
RNF24	-0.5062923
RNF25	-0.500442
RNF26	-0.4991841
RNF4	-1.1614189
RNF43	-3.180738
RNF5	-2.144637
RNF7	-0.3895946
RNF8	-0.1884084
RNFT1	-1.4637947
RNH1	-0.4557505
RNMTL1	-1.870884
RNPEP	-0.2756925
RNPEPL1	-1.0659881
RNU3B1	-1.5339336
ROBO2	-0.7488186
ROBO3	-2.1776464
ROGDI	-1.778151
ROM1	-0.3253613
ROMO1	-1.673409
ROPN1L	-4.398627
RORA	-0.4967852
RORC	-3.1863542
ROS1	-3.2845626
RP1	-1.2645752
RP1L1	-0.8442595
RP9	-1.093091
RPA1	-0.8806257
RPA3	-0.6271982
RPAIN	-0.5153589
RPAP1	-1.3705807
RPAP2	-1.3427396
RPE	-1.1857309
RPE65	-1.6345222
RPF1	-0.359911
RPF2	-0.3974319
RPH3A	-0.9949801
RPL10	-0.9416332
RPL10A	-0.6968193
RPL10L	-1.4214442
RPL11	-0.8448734
RPL12	-0.737915
RPL13	-0.3690553
RPL13A	-0.8850737

RPL14	-0.4508901
RPL15	-0.4706984
RPL17	-0.5177355
RPL18A	-0.6095071
RPL19	-0.7685952
RPL21	-0.4699407
RPL22	-0.3916168
RPL23	-0.3737602
RPL23A	-0.6879454
RPL24	-0.8317184
RPL26	-0.4067435
RPL27	-0.6191464
RPL27A	-0.614686
RPL28	-1.0715218
RPL31	-0.585546
RPL31-PS12	-0.512465
RPL32	-0.3678317
RPL34	-0.441278
RPL34-PS1	-0.5903721
RPL35	-0.5074263
RPL35A	-0.6922679
RPL36	-0.9856272
RPL36A	-0.5757995
RPL36AL	-1.1569829
RPL37A	-0.7581139
RPL38	-0.8085041
RPL39	-0.3210626
RPL4	-0.7167573
RPL41	-0.8494301
RPL5	-0.5652103
RPL5-PS2	-0.3253064
RPL6	-0.5228529
RPL7	-0.413815
RPL7A	-0.7575131
RPL7L1	-1.2607193
RPL8	-0.4912744
RPL9	-0.3895226
RPLP0	-0.6919851
RPLP1	-0.9616966
RPLP2	-0.4431295
RPN1	-0.9722314
RPN2	-0.7560325
RPP14	-0.4370089
RPP21	-1.4393954
RPP25	-1.1449347

RPP25L	-1.3790894
RPP38	-0.2204275
RPP40	-1.3943796
RPRD1A	-0.2042704
RPRD1B	-0.1703095
RPS10	-0.8075647
RPS11	-0.666245
RPS11-PS1	-0.6957736
RPS12	-0.7420025
RPS12L2	-0.2929349
RPS12-PS16	-0.4999361
RPS13	-0.4745078
RPS14	-0.7290783
RPS15	-0.8889132
RPS15A	-0.3566775
RPS16	-0.824028
RPS17	-0.7688174
RPS18	-0.571126
RPS19BP1	-1.338233
RPS2	-1.2374907
RPS20	-0.4300346
RPS21	-1.0020404
RPS24	-0.4393892
RPS25	-0.647881
RPS26	-0.205008
RPS27	-0.6097002
RPS27A	-0.4014325
RPS27L	-2.552987
RPS28	-0.5934506
RPS29	-0.7035408
RPS3	-0.7845721
RPS3A1	-0.2196007
RPS4X	-0.5135403
RPS5	-0.6917028
RPS6	-0.2569637
RPS6KB2	-0.7146864
RPS6KC1	-0.3957553
RPS8	-0.9038353
RPSA	-0.8602009
RPTOR	-0.81108
RPUSD1	-0.6453612
RPUSD2	-1.6567836
RPUSD3	-3.2269197
RPUSD4	-0.8871093
RRAGA	-1.3913164

RRAGC	-0.3304253
RRAS	-0.5016165
RRBP1	-0.6507545
RREB1	-1.1255574
RRM1	-0.3801913
RRM2	-0.5887938
RRM2B	-0.3804135
RRNAD1	-0.6392648
RRP15	-0.5243473
RRP36	-1.099299
RRP8	-0.4611416
RRS1	-0.237679
RSG1	-1.719032
RSL1D1	-0.5918732
RSLCAN18	-2.4251192
RSPH3B	-0.4872594
RSPH6A	-1.3040395
RSPO3	-3.9051979
RSPRY1	-0.1771636
RSRC1	-0.6025167
RSRP1	-1.0411873
RTEL1	-1.1425138
RTFDC1	-1.2974806
RTKN	-1.1152754
RTN3	-0.7710724
RTN4	-0.7849784
RTN4IP1	-2.105468
RTN4RL1	-3.8255188
RTN4RL2	-0.7048378
RTP2	-1.3062649
RTP3	-2.8486695
RUFY1	-0.7483325
RUFY3	-1.0758195
RUNDC3A	-1.5790551
RUNX2	-0.6085608
RUSC2	-0.8158703
RUVBL1	-0.3975043
RWDD2A	-1.120141
RWDD2B	-2.2769308
RWDD3	-1.8481953
RWDD4A	-0.2615297
RXFP2	-1.0580959
RXFP3	-1.0429945
RXRA	-2.8736892
RXRG	-2.2448654

RYK	-0.9987087
S100A1	-1.373467
S100A10	-1.4699926
S100A7A	-2.4712217
S100Z	-1.343116
S1PR5	-4.3510437
SAA1	-0.4181023
SAA4	-3.817659
SAAL1	-1.4197717
SAC3D1	-1.308382
SACM1L	-1.2260838
SAE1	-0.3209381
SAG	-1.2424805
SALL1	-5.793779
SALL4	-0.9645286
SAMD1	-0.467659
SAMD7	-1.4544032
SAMM50	-2.167316
SAMT2	-1.3162844
SAMT3	-2.4929867
SAP18	-0.4752317
SAP30BP	-0.2768889
SAP30L	-0.9878683
SAR1A	-0.3753748
SAR1B	-3.152516
SARDH	-6.9460206
SARM1	-1.2865345
SARS2	-2.0511417
SART1	-0.2566576
SAT2	-4.5657353
SAYSD1	-2.7032099
SBF1	-0.4639192
SBK1	-2.94182
SBK2	-1.4458828
SBP	-2.872519
SBPL	-2.8407145
SC5D	-5.039586
SCAF1	-2.7842379
SCAMP1	-0.5134425
SCAMP3	-1.065762
SCAMP4	-0.7370472
SCAMP5	-2.4854286
SCAND1	-0.5818048
SCAP	-2.6268468
SCARA3	-0.3930972

SCARB1	-3.1903877
SCARB2	-0.4661446
SCD1	-3.3328676
SCD3	-3.2776756
SCFD1	-0.305131
SCGB1B27	-1.5437112
SCGB1B7	-2.8453412
SCGB2B15	-0.7366073
SCGB2B21	-1.4772551
SCGB2B26	-1.1835504
SCGB2B27	-1.429676
SCGB2B3	-1.6066561
SCGB2B7	-0.976366
SCGN	-1.777451
SCIMP	-1.0944648
SCLT1	-0.6689189
SCLY	-3.158576
SCML2	-0.5894861
SCN1B	-3.0719023
SCN2A1	-1.7097192
SCN4B	-0.9132907
SCN8A	-1.2001095
SCN9A	-1.6934013
SCNM1	-0.6624551
SCO1	-1.0312266
SCO2	-4.4714627
SCP2	-3.8333468
SCP2D1	-1.6172581
SCPEP1	-1.3180709
SCRN2	-4.683589
SCRN3	-2.8302822
SCRT1	-0.7626691
SCRT2	-1.194171
SCYL1	-0.7663288
SCYL2	-0.3794804
SCYL3	-0.6917026
SDC1	-1.4275465
SDC2	-4.310366
SDC3	-1.8876367
SDCCAG3	-1.0326867
SDF2	-0.7457547
SDF2L1	-0.4225526
SDF4	-1.0087247
SDHA	-1.5167036
SDHAF1	-1.2250667

SDHAF2	-0.7533927
SDHB	-2.3928585
SDHC	-2.3313756
SDHD	-2.8755069
SDPR	-1.3014917
SDR16C5	-1.5555782
SDR16C6	-0.6361408
SDR39U1	-0.9961062
SDR42E1	-4.587285
SDR9C7	-4.5348654
SDS	-4.55709
SDSL	-0.7579336
SEBOX	-2.5163412
SEC11A	-0.4472656
SEC13	-0.695394
SEC14L2	-5.3181024
SEC14L4	-2.2830172
SEC16A	-1.0581789
SEC16B	-6.514653
SEC22B	-0.2347097
SEC22C	-1.683105
SEC23A	-2.3932295
SEC24A	-2.100246
SEC24C	-0.7728562
SEC24D	-1.1695294
SEC31A	-0.8220286
SEC31B	-1.7221682
SEC61A1	-0.8884258
SEC61B	-0.914876
SEC62	-1.2899427
SEC63	-0.5090933
SECTM1A	-1.5097136
SEL1L	-0.3315859
SEL1L2	-1.2492328
SEL1L3	-1.7774992
SELK	-0.2526913
SELO	-2.2329721
SELT	-0.964046
SEMA4A	-1.5578976
SEMA4B	-0.8107319
SEMA4C	-1.0618854
SEMA4G	-9.080381
SEMA5B	-2.9023328
SEMA6C	-1.4160879
SEMA6D	-0.8050923

SENP3	-0.7658601
SENP5	-0.3680053
SEPHS1	-1.0189898
SEPHS2	-3.5183947
SEPP1	-2.4028811
SEPSECS	-2.14534
SEPW1	-0.2267947
SERF1	-1.6636171
SERF2	-1.5851746
SERGEF	-0.3714347
SERHL	-2.525076
SERINC1	-0.8344307
SERINC2	-1.3650169
SERINC5	-1.2702069
SERPINA10	-2.6966052
SERPINA11	-4.631917
SERPINA1A	-3.7549443
SERPINA1C	-3.0987058
SERPINA1D	-5.5437226
SERPINA1E	-3.9100475
SERPINA3A	-2.1777356
SERPINA3C	-3.0786805
SERPINA3J	-4.6064944
SERPINA3K	-3.3748808
SERPINA3M	-2.8174653
SERPINA4-PS	-2.3482509
SERPINA5	-1.9850342
SERPINA6	-8.7004795
SERPINA7	-5.096842
SERPINB13	-1.593236
SERPINB1C	-1.4352298
SERPINB6E	-0.8899651
SERPINB8	-0.6782341
SERPINB9B	-1.0716155
SERPINB9G	-1.5963631
SERPINC1	-4.818849
SERPIND1	-8.455326
SERPINF1	-5.3437934
SERPINF2	-4.998319
SERPING1	-1.7471399
SERPINI2	-2.5014992
SERTAD4	-0.1704938
SESN1	-0.6110435
SESN3	-0.3629437
SETD1A	-0.2335901

SETD3	-0.6873779
SETD4	-3.1894383
SETD6	-1.9540105
SETD8	-0.3663354
SETDB1	-0.5006824
SETMAR	-1.3772645
SEZ6	-0.9260337
SEZ6L	-1.2449706
SF3B2	-0.6994658
SF3B5	-0.6724744
SF3B6	-0.5129576
SFMBT1	-1.0265694
SFT2D1	-0.8112936
SFT2D2	-1.0056038
SFT2D3	-0.6262422
SFXN1	-2.678472
SFXN2	-3.2932415
SFXN5	-3.3507342
SGCZ	-1.480381
SGK2	-9.212726
SGK3	-0.5766015
SGMS2	-1.3182969
SGOL1	-0.3644943
SGOL2	-0.6071491
SGPL1	-0.8161283
SGPP1	-1.7637553
SGSH	-0.9271741
SGSM1	-1.3583062
SGSM2	-2.108809
SGSM3	-1.297895
SGTA	-1.0934863
SGTB	-1.1446528
SH2B1	-1.7090778
SH2D1B2	-1.9089422
SH2D4A	-1.5392582
SH2D6	-0.4650242
SH2D7	-1.7897389
SH3BGRL	-0.9743419
SH3BGRL2	-0.6255579
SH3BP2	-0.778091
SH3BP5L	-0.4740014
SH3D19	-0.6207504
SH3GLB1	-0.3534269
SH3GLB2	-2.118897
SH3RF2	-2.2695565

SH3TC2	-0.6815972
SH3YL1	-1.3460512
SHANK2	-2.1501238
SHARPIN	-0.5789323
SHB	-0.3677654
SHBG	-2.2699785
SHC1	-0.3858051
SHC3	-1.5392027
SHCBP1L	-1.8836586
SHF	-3.3383145
SHISA2	-1.4737959
SHISA3	-0.8418107
SHISA6	-1.6310253
SHMT1	-5.3557158
SHMT2	-3.3074026
SHPK	-4.612007
SHPRH	-0.3056274
SHQ1	-0.4875827
SHROOM1	-3.1534953
SHROOM2	-0.5724149
SIAE	-3.0579634
SIAH2	-1.6110506
SIDT2	-1.7382116
SIGIRR	-2.2466245
SIGLEC1	-1.2066224
SIGLEC15	-1.2348948
SIGMAR1	-4.1318107
SIK3	-0.4738402
SIL1	-2.2165694
SIPA1L1	-0.3347535
SIRPA	-0.8741765
SIRT2	-0.9931169
SIRT3	-4.0557055
SIRT4	-1.1932185
SIRT5	-3.3747234
SIRT7	-0.9472532
SIS	-1.6396589
SIX5	-1.5184546
SIX6	-2.3441896
SKA2	-1.1029034
SKA3	-0.7527227
SKI	-0.4805496
SKINT1	-2.5536232
SKINT11	-1.4886944
SKINT2	-1.3501859

SKINT4	-1.6444962
SKINT6	-1.3695567
SKINT7	-1.0321813
SKIV2L	-0.9787431
SKP1A	-2.119009
SKP2	-1.4132705
SLA2	-0.1965451
SLAIN2	-0.3181095
SLAMF8	-0.6352835
SLBP	-0.6406922
SLC10A1	-0.495163
SLC10A2	-1.7119608
SLC10A5	-3.0328164
SLC12A1	-1.6431036
SLC12A5	-2.2373333
SLC12A7	-2.1770387
SLC12A8	-0.9551883
SLC12A9	-1.1853805
SLC13A1	-1.0754137
SLC13A3	-3.628553
SLC14A1	-0.9337285
SLC14A2	-2.5826137
SLC15A4	-0.9353237
SLC16A10	-2.6942744
SLC16A11	-0.6502671
SLC16A12	-5.291452
SLC16A2	-3.801877
SLC16A5	-5.9606905
SLC17A1	-4.0921016
SLC17A2	-3.8645988
SLC17A3	-5.216242
SLC17A4	-7.85545
SLC17A5	-0.6790466
SLC17A6	-1.6509299
SLC17A8	-2.996132
SLC17A9	-1.4616871
SLC18A1	-2.0277352
SLC18B1	-0.6571693
SLC19A1	-1.2767296
SLC19A2	-4.4613776
SLC1A2	-5.8534117
SLC1A4	-0.301261
SLC1A7	-0.6247635
SLC20A2	-2.2350435
SLC22A1	-7.216555

SLC22A13B-P	-1.0837495
SLC22A14	-1.5935915
SLC22A15	-0.352159
SLC22A18	-5.308019
SLC22A2	-1.609431
SLC22A23	-2.568418
SLC22A26	-5.597328
SLC22A27	-8.281913
SLC22A28	-4.8265433
SLC22A29	-6.962203
SLC22A30	-6.17296
SLC22A4	-0.9610276
SLC22A5	-1.0364184
SLC22A7	-9.838852
SLC23A1	-4.5700655
SLC24A1	-1.269695
SLC24A2	-1.0974061
SLC25A1	-3.77456
SLC25A10	-3.3044472
SLC25A11	-1.4063148
SLC25A12	-0.5760226
SLC25A13	-4.944026
SLC25A15	-2.3043847
SLC25A16	-1.9964771
SLC25A17	-1.0447373
SLC25A19	-0.3525386
SLC25A20	-1.9440193
SLC25A21	-5.670532
SLC25A22	-3.946643
SLC25A23	-4.6541853
SLC25A25	-1.7739787
SLC25A26	-1.5700374
SLC25A27	-0.3818803
SLC25A28	-0.2947269
SLC25A3	-0.3962169
SLC25A30	-1.0389385
SLC25A31	-0.7768517
SLC25A32	-1.0236034
SLC25A33	-1.4830642
SLC25A34	-0.8521161
SLC25A37	-0.5271726
SLC25A38	-1.5831094
SLC25A39	-3.187243
SLC25A40	-0.68854
SLC25A42	-3.6431794

SLC25A44	-2.4123323
SLC25A45	-2.1019316
SLC25A46	-1.3081574
SLC25A47	-3.1448736
SLC25A48	-3.2257864
SLC25A5	-1.5909476
SLC25A53	-0.4609048
SLC25A54	-1.5754569
SLC26A1	-5.0630746
SLC26A10	-6.641889
SLC26A11	-1.2283759
SLC26A5	-1.652781
SLC26A6	-1.8549778
SLC26A7	-1.6144285
SLC26A8	-1.536227
SLC27A2	-6.0610776
SLC27A4	-0.4111204
SLC27A5	-5.580925
SLC29A1	-3.8449864
SLC29A2	-0.7065303
SLC2A2	-8.05971
SLC2A4RG-PS	-0.9497471
SLC2A5	-6.0115047
SLC2A7	-2.2603326
SLC2A8	-2.4355774
SLC2A9	-4.537923
SLC30A1	-1.0010977
SLC30A10	-4.4348927
SLC30A5	-0.7827439
SLC30A6	-1.4567189
SLC30A8	-2.7180018
SLC30A9	-1.2187948
SLC31A1	-2.293604
SLC33A1	-1.3101015
SLC34A3	-1.4748874
SLC35A1	-1.3807883
SLC35A3	-1.2128534
SLC35A5	-0.5362573
SLC35B2	-2.240377
SLC35B3	-0.4427228
SLC35B4	-1.2357936
SLC35C1	-0.5643497
SLC35C2	-1.9163303
SLC35D1	-3.1921844
SLC35D2	-3.5713713

SLC35D3	-1.0837734
SLC35E1	-0.1971865
SLC35E2	-2.982149
SLC35F4	-1.1774936
SLC35F5	-1.22124
SLC35F6	-0.7462754
SLC35G1	-3.1526809
SLC36A1	-0.2319877
SLC36A1OS	-2.9257362
SLC36A3	-1.315079
SLC37A2	-0.2391515
SLC37A3	-0.7648034
SLC37A4	-4.772469
SLC38A10	-0.6839624
SLC38A11	-3.557084
SLC38A3	-6.556947
SLC38A4	-3.1973958
SLC38A6	-0.5171809
SLC38A7	-2.4135222
SLC38A8	-2.0197756
SLC38A9	-0.5319872
SLC39A1	-0.4935308
SLC39A11	-1.2318497
SLC39A12	-0.8260026
SLC39A13	-0.6319618
SLC39A14	-0.4456835
SLC39A4	-1.5020638
SLC39A5	-4.489529
SLC3A1	-5.626747
SLC40A1	-6.0607204
SLC41A2	-0.7702017
SLC41A3	-1.2450686
SLC43A1	-2.646374
SLC44A1	-0.685935
SLC44A3	-1.6706958
SLC44A5	-3.0853257
SLC45A1	-2.5767765
SLC45A2	-2.0441353
SLC45A3	-1.206007
SLC46A1	-5.488703
SLC46A3	-2.7091537
SLC47A1	-6.9882197
SLC48A1	-0.6081719
SLC4A1	-0.1522608
SLC4A1AP	-0.3958039

SLC4A4	-0.9047828
SLC4A7	-0.2537866
SLC4A9	-4.560327
SLC50A1	-0.1766763
SLC51A	-1.8665192
SLC51B	-4.7861786
SLC52A2	-1.0566182
SLC5A11	-2.7469182
SLC5A12	-2.1864734
SLC5A5	-1.6601608
SLC5A6	-1.9169612
SLC5A7	-1.0221202
SLC6A1	-2.6907003
SLC6A12	-4.3527303
SLC6A13	-6.57211
SLC6A15	-1.7339902
SLC6A16	-1.7305021
SLC6A17	-3.1260722
SLC6A19	-1.6292312
SLC6A19OS	-1.3204572
SLC6A20B	-0.9565954
SLC6A6	-0.4200716
SLC6A7	-1.2278509
SLC6A9	-0.7748075
SLC7A14	-1.3302557
SLC7A15	-2.2316103
SLC7A2	-2.3308496
SLC7A4	-1.3935072
SLC7A6OS	-1.0619249
SLC7A7	-0.7981339
SLC7A8	-0.3249846
SLC8A2	-1.1633229
SLC8B1	-1.9597816
SLC9A3R1	-2.0829706
SLC9A3R2	-0.2650628
SLC9A6	-0.4130197
SLC9A8	-0.5926747
SLC9A9	-2.1462402
SLC9B1	-1.3554862
SLC9B2	-0.2793841
SLCO1A1	-7.149026
SLCO1A4	-10.344258
SLCO1B2	-6.0776753
SLCO2B1	-4.1535997
SLCO6B1	-1.5946429

SLFN14	-1.1379457
SLFN5	-0.8621397
SLFN5OS	-1.3747416
SLIRP	-1.0538936
SLITRK4	-1.243196
SLITRK5	-3.0884354
SLMAP	-0.3865552
SLMO2	-0.4498696
SLX1B	-0.6122189
SLXL1	-1.5845726
SLY	-1.6049042
SMAD9	-2.5557764
SMAGP	-1.627985
SMAP2	-0.7220349
SMARCA2	-1.7954769
SMARCAD1	-0.5900903
SMARCAL1	-0.6251001
SMARCB1	-0.5579619
SMARCC1	-0.9515157
SMARCC2	-1.7755919
SMARCD1	-0.5517163
SMARCD2	-1.6647406
SMC1B	-4.6491394
SMC2	-0.3906455
SMC2OS	-2.153514
SMC6	-0.3995056
SMCO2	-4.1660786
SMCO3	-1.0800028
SMCO4	-1.9662862
SMCP	-1.6141391
SMCR8	-0.5305257
SMDT1	-1.194447
SMEK1	-0.7871542
SMEK3-PS	-2.1051877
SMG5	-0.5637875
SMG6	-0.6007004
SMG7	-0.3150497
SMIM1	-1.7865014
SMIM11	-0.9705849
SMIM12	-1.0591235
SMIM13	-0.8311873
SMIM14	-1.1645279
SMIM19	-1.4029856
SMIM20	-1.7773328
SMIM22	-0.6838284

SMIM23	-1.8947325
SMIM24	-0.3857613
SMIM4	-1.4580779
SMIM6	-1.4900231
SMIM7	-1.0340333
SMIM8	-1.7335067
SMIM9	-1.5024033
SMLR1	-6.485633
SMNDC1	-1.6014009
SMO	-1.487247
SMOC1	-4.7916985
SMOK2A	-1.2051139
SMOK2B	-1.0764251
SMOK3B	-2.3754947
SMOK4A	-1.138866
SMPD1	-0.7607656
SMPD2	-2.6705232
SMPD3	-1.1541388
SMPD4	-0.8590541
SMPD5	-3.4937122
SMPDL3A	-0.7710257
SMR2	-1.8163371
SMR3A	-1.9552722
SMU1	-0.538156
SMUG1	-0.5902362
SMURF1	-0.3683825
SMURF2	-0.2975116
SMYD1	-1.519325
SMYD2	-1.4208403
SMYD4	-1.1888673
SMYD5	-1.4881341
SNAI2	-3.6205301
SNAI3	-2.5812297
SNAP25	-0.5563445
SNAP29	-0.38098
SNAP47	-3.5220375
SNAP91	-1.6499391
SNAPC2	-1.0752583
SNAPC5	-0.326117
SNCAIP	-0.7851241
SNCB	-1.5787086
SNED1	-0.2243791
SNF8	-1.3862739
SNHG10	-0.7407014
SNHG8	-1.5981312

SNIP1	-0.7731981
SNORA47	-1.9816473
SNPH	-1.4068167
SNRNP200	-0.4699473
SNRNP25	-1.8719115
SNRNP27	-0.3943138
SNRNP70	-0.1518807
SNRPA	-0.3712039
SNRPC	-0.1806855
SNRPD2	-0.9737177
SNRPD3	-0.2393503
SNRPN	-2.1189008
SNTB1	-1.635643
SNTG1	-1.8630095
SNTG2	-4.465129
SNUPN	-0.9196296
SNX14	-0.9227572
SNX15	-1.2997861
SNX19	-1.0272064
SNX2	-0.5501375
SNX21	-1.354527
SNX24	-1.4363561
SNX3	-0.7419167
SNX32	-1.5349588
SNX33	-1.1859832
SNX5	-0.4112544
SNX7	-0.6494064
SNX8	-1.3116493
SOAT2	-3.4074101
SOD1	-2.5331712
SOGA3	-0.2257633
SOHLH2	-0.3166807
SORBS1	-0.3569784
SORBS2	-3.5919766
SORD	-5.9174542
SOS1	-0.4663034
SOWAHB	-2.0293188
SOWAHD	-2.6267858
SOX1	-2.5286083
SOX12	-1.1205492
SOX14	-2.4609828
SOX15	-1.2079394
SOX18	-0.6433301
SOX2OT	-1.3778038
SOX3	-1.685375

SOX5	-1.8175154
SOX5OS3	-1.5952823
SOX6	-2.6347268
SOX6OS	-3.7014399
SOX9	-0.477418
SP110	-0.9540477
SP5	-2.8774061
SPACA1	-1.5258679
SPACA5	-1.620631
SPACA7	-1.802891
SPAG11A	-2.6751297
SPAG4	-4.643856
SPAG5	-0.3294587
SPAG7	-1.0396767
SPAG8	-0.6549974
SPAM1	-1.5053015
SPAST	-1.2140107
SPATA20	-0.9898837
SPATA22	-4.816758
SPATA24	-1.405721
SPATA2L	-3.9038513
SPATA31D1C	-2.118829
SPATA32	-1.011904
SPATA33	-0.3044717
SPATA4	-2.1246161
SPATA45	-2.6372504
SPATA5L1	-0.2022936
SPATA9	-1.0140553
SPATC1L	-0.4971225
SPATS1	-2.054089
SPC24	-2.7965894
SPC25	-2.6062942
SPCS1	-0.7671356
SPCS3	-0.6979785
SPDL1	-1.0419264
SPDYA	-1.072288
SPDYB	-1.4452095
SPEER4F	-1.4128499
SPESP1	-1.4912019
SPG11	-0.2547283
SPG20	-0.2848198
SPG7	-1.1435227
SPHK2	-1.3454242
SPIC	-1.182909
SPIN2C	-2.5177822

SPINK10	-1.3541687
SPINK11	-2.4522245
SPINK13	-1.77387
SPINK3	-4.71461
SPINK8	-1.4070315
SPINT3	-2.4092524
SPINT4	-1.4681544
SPIRE2	-0.7623317
SPNS1	-0.510046
SPNS2	-1.4831872
SPNS3	-2.2808077
SPOCK1	-1.2774539
SPON2	-6.4351134
SPP2	-6.538906
SPPL2B	-0.9325423
SPR	-3.2320151
SPRR4	-1.821255
SPRYD3	-0.3328652
SPRYD4	-3.333776
SPSB2	-1.0329785
SPSB3	-0.6613059
SPSB4	-3.441763
SPT1	-1.6413841
SPTBN2	-4.3039017
SPTBN5	-1.5403571
SPTSSA	-2.0237327
SPZ1	-1.6694188
SQLE	-2.9296565
SQRDL	-3.02079
SQSTM1	-0.6548162
SRA1	-0.7939787
SRBD1	-0.9029322
SRCRB4D	-1.3364115
SRD5A1	-6.1957283
SRD5A2	-1.1912866
SRD5A3	-0.1743369
SREBF1	-3.6409116
SREBF2	-0.3388844
SREK1	-0.8777785
SREK1IP1	-1.6011472
SRGAP2	-0.2412238
SRM	-0.6750503
SRMS	-1.8502653
SRP54B	-0.3230319
SRP72	-1.4210181

SRP9	-0.4643641
SRPK1	-0.2048674
SRPR	-0.9304643
SRPX2	-1.2011824
SRR	-3.5917263
SRRD	-1.4176607
SRRM3	-1.0598016
SRRM3OS	-2.0599422
SRRM4	-2.1717176
SRRM4OS	-1.8023467
SRSF12	-0.7416625
SRSF9	-0.361608
SRXN1	-0.5478845
SS18L1	-0.8607893
SSBP1	-0.3160539
SSBP3	-1.532805
SSFA2	-1.5676026
SSMEM1	-1.0077405
SSNA1	-1.2345381
SSPO	-2.8288884
SSR1	-0.4078364
SSR2	-0.978642
SSR3	-0.5429416
SSR4	-1.4302049
SSRP1	-0.1449847
SSSCA1	-1.4571576
SSTR1	-1.1631444
SSTR3	-0.9469924
SSTR5	-0.9462161
SSTY1	-2.1223547
SSU2	-1.2379906
SSU72	-0.3675256
SSXA1	-1.2661147
SSXB1	-1.3529406
SSXB10	-1.6439202
SSXB6	-3.6154895
SSXB7	-1.0558767
SSXB8	-2.524983
ST13	-1.3255024
ST18	-1.6455307
ST3GAL3	-2.9570003
ST3GAL4	-1.2482996
ST3GAL5	-2.9430838
ST5	-0.5405107
ST6GAL1	-2.8583016

ST6GAL2	-1.3909388
ST6GALNAC6	-3.4826307
ST7L	-1.5475826
ST8SIA2	-2.8565133
ST8SIA3	-1.7225876
ST8SIA5	-0.1343279
STAB1	-0.7941456
STAB2	-4.3177156
STAC	-0.784008
STAC3	-0.9972043
STAG3	-1.3802686
STAMBP	-0.3587942
STAMOS	-1.689832
STAP2	-1.4911265
STARD10	-4.769731
STARD4	-1.7156329
STARD5	-3.0867333
STARD7	-1.9684367
STARD8	-0.5969148
STAT2	-0.1992707
STAT5A	-0.7566133
STAT5B	-1.0497155
STAT6	-0.4891453
STAU1	-1.1145258
STBD1	-3.2255478
STEAP3	-2.2886536
STIM1	-0.6403637
STIM2	-1.2054207
STIP1	-0.9516878
STK11	-0.727942
STK16	-2.9392138
STK35	-0.4009135
STK38	-0.1829658
STK38L	-0.5036974
STMN1	-0.7389813
STOM	-0.6662478
STOML2	-1.8957381
STOX2	-0.9042203
STRA13	-1.9484158
STRADB	-2.9168978
STRAP	-1.3291211
STRBP	-1.1599917
STRC	-0.4143252
STRM	-2.7818449
STRN4	-0.3837628

STS	-2.0002685
STT3A	-0.3563542
STT3B	-0.6146412
STUB1	-1.4159875
STX17	-0.5362072
STX18	-0.4757848
STX2	-1.3567307
STX4A	-0.4877796
STX5A	-0.3738785
STX8	-0.2172322
STXBP2	-0.6427431
STXBP5L	-1.5534081
STXBP6	-2.3005407
STYK1	-0.8077984
SUCLA2	-1.7132645
SUCLG1	-3.106412
SUCLG2	-4.250857
SUCNR1	-6.2458925
SUDS3	-2.5399475
SUGCT	-4.181682
SULT1A1	-3.6375551
SULT1B1	-7.9142046
SULT1C2	-6.43478
SULT1D1	-4.142336
SULT1E1	-3.4264932
SULT2A1	-11.68733
SULT2A2	-10.144642
SULT2A3	-8.565436
SULT2A4	-11.523837
SULT2A6	-9.906766
SULT2A7	-3.6536777
SULT3A1	-6.9189734
SULT4A1	-2.379742
SULT5A1	-1.5223656
SUMF1	-1.0130916
SUMF2	-1.9750295
SUMO1	-0.2496166
SUMO3	-1.6168885
SUN2	-0.3327169
SUN3	-2.492214
SUN5	-0.571666
SUOX	-3.6935096
SUPT20	-0.2299473
SUPT3	-1.8223557
SUPV3L1	-1.0998168

SURF1	-1.6118655
SURF2	-1.4215641
SURF4	-0.7112889
SUSD1	-1.2304461
SUV39H2	-0.89114
SUV420H2	-0.8078132
SV2C	-0.7849832
SVAL1	-1.2755044
SVAL2	-1.6552999
SVIP	-1.9924855
SVOP	-3.1527576
SVS2	-1.6796789
SVS3B	-1.750433
SVS5	-1.3275557
SWI5	-0.6532788
SYAP1	-1.3762274
SYBU	-2.4866345
SYCE2	-0.3772006
SYCE3	-4.668955
SYCP2	-1.3541698
SYCP3	-0.5537019
SYDE2	-0.6449385
SYMPK	-1.7106996
SYN1	-0.5903173
SYN3	-0.5568373
SYNA	-2.426396
SYNB	-0.7187474
SYNDIG1	-1.0840578
SYNDIG1L	-3.1545758
SYNE3	-1.55616
SYNE4	-0.9644432
SYNGR2	-0.5172367
SYNGR4	-2.5752478
SYNJ2BP	-1.4085517
SYNPO2L	-0.8062105
SYNPR	-1.3710659
SYP	-0.4839368
SYPL	-0.2650032
SYPL2	-1.8671937
SYS1	-1.2679386
SYT1	-4.3975315
SYT10	-4.4652624
SYT14	-0.6384358
SYT15	-0.496695
SYT17	-1.5328698

SYT2	-1.4709139
SYT3	-4.5554743
SYT6	-2.991417
SYT7	-1.0281541
SYT9	-2.541484
SYTL1	-1.3878319
SYTL4	-1.8236814
SYVN1	-0.7366333
SZRD1	-0.8685136
SZT2	-0.5608535
TAAR4	-1.2474709
TAAR7A	-1.7116349
TAAR8A	-1.8347554
TAAR9	-1.7234468
TAB1	-0.3766713
TACO1	-3.0631468
TACR2	-0.4721036
TADA1	-0.7597146
TADA3	-0.4646907
TAF10	-1.0908875
TAF11	-0.4687448
TAF12	-0.3996863
TAF13	-0.262785
TAF15	-1.4853988
TAF1A	-0.210449
TAF2	-0.4828777
TAF6	-0.814765
TAF6L	-1.0198662
TAF9	-0.7686801
TAGLN3	-1.4663506
TAL2	-0.693924
TALDO1	-0.5734601
TAMM41	-0.34728
TANGO2	-3.209816
TAOK2	-0.2867951
TAOK3	-0.4338713
TAPBP	-0.2422738
TAPBPL	-0.4093542
TAPT1	-1.3071685
TARBP2	-0.4101486
TARS	-1.405159
TARS2	-1.5694251
TARSL2	-2.5457664
TAS1R2	-1.6003654
TAS1R3	-3.1341958

TAS2R104	-2.7718177
TAS2R105	-1.697294
TAS2R106	-2.6529646
TAS2R109	-0.7169128
TAS2R113	-1.0834925
TAS2R114	-2.72626
TAS2R115	-1.344341
TAS2R116	-1.6059535
TAS2R119	-1.6604698
TAS2R121	-1.6376812
TAS2R122	-1.5997365
TAS2R126	-0.9792333
TAS2R130	-1.5029459
TAS2R136	-1.8199167
TAS2R138	-2.120306
TAS2R140	-0.941699
TAS2R143	-1.5386221
TAT	-3.1415734
TATDN1	-0.8078783
TATDN2	-1.585185
TATDN3	-1.4834771
TAZ	-0.964869
TBATA	-1.534172
TBC1D10A	-0.3050261
TBC1D13	-1.1993542
TBC1D17	-1.0644107
TBC1D21	-1.8050733
TBC1D22BOS	-2.4371793
TBC1D24	-1.7414281
TBC1D2B	-0.813704
TBC1D31	-1.8228433
TBC1D32	-0.3502896
TBC1D7	-2.56582
TBC1D8B	-0.4991903
TBC1D9B	-1.0211949
TBCA	-1.1867533
TBCCD1	-2.4668932
TBCD	-1.0096364
TBCEL	-1.0381179
TBL3	-0.9817734
TBP	-0.4782114
TBRG4	-0.8679791
TBX20	-0.3243334
TBX3OS1	-1.4855671
TBXA2R	-2.453041

TBXAS1	-1.1445799
TCAIM	-0.9811361
TCAM1	-1.2992644
TCEA1	-0.9625135
TCEA2	-0.9834352
TCEA3	-3.4032712
TCEAL5	-1.2334328
TCEB1	-0.2600999
TCEB2	-0.7456412
TCF19	-1.1693282
TCF24	-3.6741288
TCF25	-0.4980354
TCF3	-0.5860033
TCF7L1	-1.1263957
TCFL5	-1.4508419
TCIRG1	-1.1960735
TCL1	-1.1529062
TCL1B1	-1.4319911
TCL1B3	-1.4015982
TCL1B4	-1.1656275
TCL1B5	-2.1308923
TCN2	-1.408978
TCP1	-0.318378
TCRB-J	-0.4906848
TCRG-V1	-0.4088574
TCTA	-1.7816253
TCTE2	-0.8167877
TCTN1	-1.0184045
TDGF1	-0.2643316
TDO2	-6.272587
TDP1	-0.3973613
TDP2	-1.663948
TDPOZ1	-3.0914564
TDPOZ2	-1.1306174
TDPOZ4	-2.645056
TDRD1	-1.9335742
TDRD5	-1.1168032
TDRD6	-1.4781165
TDRD7	-0.2199678
TDRD9	-3.236772
TDRKH	-1.8517578
TDRP	-3.084102
TEAD2	-0.2740188
TEC	-1.7743051
TECPR2	-0.6129456

TECR	-3.4269428
TECTB	-1.1709595
TEDDM1	-1.9339752
TEF	-1.2313075
TEFM	-1.6847479
TEKT3	-1.7882011
TEKT5	-0.8464007
TELO2	-1.3207717
TENM1	-1.6527865
TENM4	-1.236496
TEP1	-0.9149704
TERF1	-0.8401208
TERF2IP	-0.4213219
TESK2	-2.1633341
TET1	-3.2148874
TEX101	-0.792141
TEX12	-3.2207646
TEX14	-2.2067127
TEX16	-3.2422795
TEX18	-1.7490733
TEX19.1	-1.6686182
TEX19.2	-0.4975777
TEX2	-2.522686
TEX21	-1.2409568
TEX22	-6.171015
TEX24	-1.1086018
TEX261	-1.1183972
TEX264	-1.9747868
TEX29	-1.3772123
TEX36	-1.0835626
TEX38	-0.7706475
TEX43	-2.1736465
TFAM	-1.8583555
TFAP2B	-1.5994511
TFAP4	-2.1754758
TFB1M	-2.355515
TFB2M	-1.47821
TFCP2	-1.1527998
TFDP1	-0.5777845
TFEB	-1.2307682
TFEC	-1.2753098
TFF3	-3.9703882
TFG	-0.6227703
TFIP11	-0.9547954
TFPI2	-4.1290903

TFPT	-0.8484602
TFR2	-4.9336843
TFRC	-0.4773459
TGDS	-2.0743492
TGFA	-1.8896153
TGFBRAP1	-1.185072
TGM3	-0.4960198
TGM4	-1.3145041
TGM6	-1.1028733
TGM7	-2.3255208
TGS1	-0.2350974
TGTP1	-0.9022698
TGTP2	-1.007843
THA1	-1.3092494
THADA	-0.4741187
THAP1	-0.7445703
THAP11	-0.8242168
THAP4	-1.1565466
THAP6	-1.1766713
THAP7	-1.0582681
THBS2	-0.9011281
THEM4	-2.3330612
THEM6	-0.9676447
THEM7	-4.2033587
THEMIS3	-1.5934954
THNSL1	-0.6710236
THNSL2	-4.6683197
THOC3	-0.9328408
THOC5	-0.4710279
THOC7	-1.4334712
THOP1	-0.7328701
THPO	-3.934678
THRA	-0.5718284
THRB	-2.095254
THRSP	-7.9050703
THSD1	-0.4103022
THTPA	-2.2963066
THUMPD1	-0.4062109
THUMPD2	-1.154922
THUMPD3	-0.5955224
THY1	-0.1742535
THYN1	-0.509984
TIAL1	-0.7223401
TICAM1	-1.2297955
TICAM2	-2.0741644

TIGD4	-1.0651517
TIGD5	-1.9848585
TIMD2	-6.6601887
TIMD4	-3.3659754
TIMM10	-1.5304322
TIMM10B	-0.5638857
TIMM13	-2.0789323
TIMM17A	-0.2083383
TIMM17B	-2.1962247
TIMM21	-1.7846227
TIMM22	-0.9147887
TIMM23	-1.2942834
TIMM44	-1.6051216
TIMM50	-1.9624505
TIMM8A1	-2.4405327
TIMM8B	-1.5594912
TIMM9	-1.4904656
TIMMDC1	-1.5057149
TINF2	-0.5775595
TJP3	-1.5906415
TK1	-3.9486876
TKT	-1.1723127
TKTL2	-1.069068
TLCD1	-3.2412505
TLCD2	-4.8506284
TLDC1	-1.8228574
TLE2	-0.8442397
TLE6	-0.8354044
TLK1	-0.6422715
TLL2	-1.7058938
TLN2	-1.7706573
TLR1	-0.5566731
TLR11	-1.110642
TLR12	-4.223994
TLR3	-1.6771963
TLR5	-2.9391375
TLR9	-3.7505994
TLX1	-2.035793
TLX3	-1.4462218
TM2D1	-0.1820426
TM2D2	-1.6537466
TM2D3	-1.0985613
TM4SF4	-3.4941816
TM4SF5	-1.3662229
TM6SF2	-5.1449933

TM7SF2	-6.150759
TM7SF3	-2.8231764
TM9SF1	-1.0784903
TM9SF2	-1.1974483
TM9SF4	-0.7820015
TMA7	-0.5605164
TMBIM4	-1.1259522
TMBIM6	-2.4776673
TMC3	-1.944027
TMC6	-0.3704987
TMC8	-0.4625063
TMCC1	-0.6470518
TMCO1	-1.7428284
TMCO2	-1.661385
TMCO5	-3.422922
TMCO5B	-1.2139342
TMCO6	-0.2259255
TMED1	-1.6961589
TMED11	-1.2013464
TMED2	-0.6730638
TMED4	-1.5814147
TMED6	-2.2668512
TMED7	-1.44837
TMEM101	-0.7063608
TMEM102	-1.847918
TMEM104	-0.4786367
TMEM106A	-1.3248987
TMEM106B	-2.0836892
TMEM106C	-2.8405387
TMEM110	-0.7975817
TMEM115	-1.1773686
TMEM120A	-2.4092093
TMEM126A	-2.2670584
TMEM126B	-2.018876
TMEM129	-1.6537004
TMEM132B	-1.5889192
TMEM132E	-2.7352035
TMEM134	-1.269517
TMEM135	-2.790718
TMEM141	-3.6383262
TMEM143	-2.59191
TMEM144	-2.8183968
TMEM145	-1.6395881
TMEM147	-2.1488419
TMEM14A	-2.4976666

TMEM14C	-2.4876914
TMEM150A	-4.750093
TMEM150B	-1.8406267
TMEM156	-0.1586337
TMEM158	-0.8183365
TMEM160	-1.2118258
TMEM161A	-1.0861964
TMEM161B	-0.1968925
TMEM167	-0.5201988
TMEM168	-1.3112042
TMEM170	-0.6298184
TMEM174	-1.2232339
TMEM175	-1.1484008
TMEM176A	-0.9575567
TMEM176B	-0.8081946
TMEM177	-1.4927709
TMEM178B	-0.8795292
TMEM179B	-0.679359
TMEM18	-1.7686162
TMEM180	-1.1259975
TMEM183A	-2.4034152
TMEM184A	-0.8081932
TMEM184C	-0.4241471
TMEM185B	-0.403461
TMEM186	-0.7621617
TMEM19	-2.9817324
TMEM191C	-1.4926116
TMEM192	-0.8014875
TMEM194	-0.4928503
TMEM198B	-0.3433485
TMEM199	-0.6398556
TMEM201	-1.925731
TMEM203	-1.9258194
TMEM205	-4.79817
TMEM206	-0.390512
TMEM208	-1.4226384
TMEM211	-1.1181142
TMEM214	-1.0994997
TMEM217	-0.9069979
TMEM218	-1.9600728
TMEM219	-1.4623199
TMEM220	-2.9365354
TMEM222	-0.8203206
TMEM223	-1.7530661
TMEM229A	-3.0607066

TMEM230	-1.3642492
TMEM234	-0.7804518
TMEM238	-1.7477992
TMEM239	-0.7665372
TMEM241	-1.20418
TMEM242	-1.2993131
TMEM248	-0.4799538
TMEM25	-5.573579
TMEM253	-1.0940413
TMEM254A	-4.156347
TMEM254C	-3.4714813
TMEM255A	-0.605248
TMEM256	-2.254356
TMEM259	-1.4689116
TMEM26	-0.4280043
TMEM260	-1.2420969
TMEM261	-1.5984111
TMEM263	-0.4253135
TMEM29	-1.2929308
TMEM30A	-0.9788156
TMEM30B	-2.476223
TMEM33	-0.9651771
TMEM35	-1.7263782
TMEM37	-1.2592306
TMEM38B	-1.7932692
TMEM39B	-0.890501
TMEM41A	-1.9320955
TMEM41B	-0.4806938
TMEM42	-0.8451877
TMEM44	-0.6287603
TMEM45A	-0.8407774
TMEM5	-1.191437
TMEM50A	-1.6201482
TMEM51	-1.2540426
TMEM51OS1	-0.9767437
TMEM52B	-1.632659
TMEM53	-4.3285775
TMEM55A	-1.1535668
TMEM55B	-0.3135262
TMEM56	-3.933988
TMEM57	-1.2776995
TMEM59	-1.5705805
TMEM60	-0.7778602
TMEM62	-0.9102631
TMEM63B	-2.350391

TMEM64	-1.3881192
TMEM65	-0.6591039
TMEM66	-0.509892
TMEM67	-0.3394141
TMEM69	-1.1032634
TMEM70	-1.8720808
TMEM72	-1.7225125
TMEM74	-1.4972069
TMEM8	-1.2486534
TMEM80	-0.6572032
TMEM81	-1.0103707
TMEM82	-3.5361118
TMEM86A	-2.2777
TMEM86B	-6.6071043
TMEM88B	-1.589891
TMEM9	-1.7843432
TMEM91	-2.9174604
TMEM97	-4.227846
TMEM98	-1.4891372
TMIE	-4.139929
TMLHE	-1.9207747
TMOD4	-0.7860551
TMPO	-0.3662624
TMPRSS11B	-0.7911847
TMPRSS11E	-1.6457675
TMPRSS11F	-1.6427114
TMPRSS2	-0.6947966
TMPRSS3	-0.7640362
TMPRSS6	-7.983371
TMPRSS7	-3.0389588
TMPRSS9	-1.2186031
TMSB15A	-1.0974131
TMSB15B1	-1.0315895
TMTC2	-0.4344783
TMTC4	-1.7135694
TMUB1	-0.5456486
TMUB2	-0.9846149
TMX1	-0.7242055
TMX2	-2.524324
TNFAIP8L1	-4.3634257
TNFAIP8L2	-0.404201
TNFRSF11B	-1.7752686
TNFRSF19	-1.8024366
TNFRSF1A	-0.296236
TNFRSF22	-1.0994356

TNFRSF8	-1.1758909
TNFRSF9	-0.7169371
TNFSF12	-0.4553609
TNFSF18	-2.2376907
TNK2	-0.4570489
TNNI1	-1.1735432
TNNT1	-0.3722367
TNPO1	-0.2244802
TNPO2	-0.4768748
TNPO3	-0.4226055
TNS3	-0.4259596
TNXB	-0.184885
TOB1	-2.1906657
TOB2	-1.0026956
TOLLIP	-0.1890941
TOM1	-0.5175519
TOM1L1	-0.9001126
TOMM20	-0.5226727
TOMM22	-0.8853765
TOMM34	-0.3529596
TOMM40	-1.406013
TOMM40L	-0.3331823
TOMM5	-0.9525809
TOMM6	-1.4236369
TOMM7	-0.3908258
TOMM70A	-1.8177114
TOMT	-0.7516065
TOP1	-0.242547
TOP1MT	-2.8469138
TOP3B	-0.8191509
TOPORS	-0.4310279
TOR1A	-1.9251885
TOR1AIP2	-0.9798284
TOR1B	-0.4942484
TOR2A	-1.5932374
TOR4A	-0.2999735
TOX	-2.0853581
TPBPB	-2.288995
TPCN1	-2.8947835
TPCN2	-1.2707484
TPD52L1	-3.2894201
TPD52L2	-0.4893913
TPH1	-0.4416952
TPH2	-1.6890173
TPI1	-2.467258

TPK1	-2.209687
TPMT	-5.591708
TPO	-1.5830536
TPP1	-1.8499885
TPPP	-3.346234
TPRA1	-0.5894876
TPRKB	-1.5544777
TPRN	-0.6076326
TPST1	-1.6649532
TPT1	-0.9138012
TPX2	-0.2944982
TRABD	-1.6904273
TRADD	-1.0304122
TRAF4	-2.370016
TRAFD1	-0.434947
TRAM1	-1.8573294
TRAM1L1	-1.1523302
TRAM2	-0.1924691
TRANK1	-0.6517777
TRAP1	-3.0333395
TRAPPC1	-1.1237803
TRAPPC11	-0.2702923
TRAPPC12	-1.611753
TRAPPC13	-0.9703012
TRAPPC2L	-0.9940672
TRAPPC3	-0.7308273
TRAPPC3L	-1.62111
TRAPPC4	-0.1723046
TRAPPC5	-1.213604
TRAPPC6A	-1.4611721
TRAV14D-3-[	-1.6768079
TRAV14N-1	-1.5938208
TRAV3-4	-2.127504
TRAV7N-5	-1.6268353
TRAV9N-2	-1.3800368
TREML4	-1.2060413
TRF	-2.6815367
TRHDE	-2.216874
TRHR2	-1.1968281
TRIAP1	-1.2293005
TRIL	-1.5606911
TRIM14	-2.8353574
TRIM2	-2.2485776
TRIM23	-0.3116574
TRIM24	-1.8306475

TRIM25	-0.8184094
TRIM26	-0.4506359
TRIM28	-1.1649313
TRIM30E-PS1	-1.6000621
TRIM31	-1.3725774
TRIM32	-0.2091866
TRIM42	-1.9374564
TRIM43C	-0.2700651
TRIM45	-1.0092931
TRIM58	-0.8368228
TRIM6	-1.0792851
TRIM60	-1.6456466
TRIM61	-1.1452541
TRIM65	-0.5260334
TRIM67	-1.1727233
TRIM68	-0.760977
TRIM7	-1.2893963
TRIM71	-1.3514731
TRIM9	-0.373538
TRIML1	-1.4790812
TRIML2	-1.5685856
TRIP13	-0.4882469
TRIP4	-0.5063396
TRIP6	-0.3971472
TRIT1	-1.5885906
TRMT1	-0.5043407
TRMT10A	-1.0434399
TRMT10B	-0.953393
TRMT10C	-0.9749198
TRMT11	-0.824903
TRMT112	-0.5150175
TRMT12	-1.5175486
TRMT1L	-0.7232971
TRMT2B	-1.9289026
TRMT5	-1.1678083
TRMT61B	-0.4223971
TRMU	-1.4404345
TRNAU1AP	-0.8819141
TROAP	-0.9245651
TRP53	-0.3875842
TRP53INP2	-1.1804132
TRP53RK	-0.7270527
TRP53TG5	-2.0169
TRP63	-0.9255858
TRPA1	-3.4446115

TRPC3	-1.3060498
TRPC4AP	-0.7651177
TRPC5OS	-1.5228348
TRPM2	-0.8528657
TRPM3	-1.3772895
TRPM4	-0.6374488
TRPM5	-0.9964542
TRPM7	-0.8388019
TRPM8	-1.1621244
TRPT1	-1.3296618
TRPV1	-3.092381
TRRAP	-1.0992184
TRUB1	-1.6335297
TRUB2	-0.5343714
TRY5	-0.8315997
TSACC	-0.9175191
TSC1	-1.0826273
TSEN15	-0.6843572
TSEN2	-1.8761191
TSEN34	-1.3607335
TSEN54	-1.1788821
TSFM	-1.3855939
TSG101	-0.285244
TSGA13	-1.2042961
TSGA8	-1.5587332
TSHB	-1.2694902
TSHR	-1.5516264
TSHZ2	-0.5193453
TSKS	-0.760828
TSKU	-0.5065846
TSN	-0.7544723
TSNAX	-0.2988577
TSPAN12	-3.719067
TSPAN31	-2.7191029
TSPAN33	-1.7968831
TSPAN4	-1.1494126
TSPAN7	-0.6259441
TSPEAR	-1.7370131
TSPYL5	-0.7234969
TSR3	-0.6078863
TSSC4	-0.6729727
TSSK1	-1.9900944
TSSK2	-1.6752961
TSSK3	-1.6877873
TSSK5	-2.3464868

TST	-4.6288686
TSTA3	-1.9614835
TSTD1	-4.9854393
TSTD2	-0.560051
TSTD3	-3.1406922
TSX	-1.5832105
TTBK1	-3.5209665
TTC1	-0.528749
TTC13	-1.2606912
TTC17	-0.7041965
TTC19	-0.7391315
TTC21B	-0.6610699
TTC23	-3.75532
TTC23L	-1.0147252
TTC27	-0.7686191
TTC30A1	-0.6140471
TTC30A2	-0.6633139
TTC30B	-0.3367572
TTC32	-1.7088718
TTC33	-0.7129407
TTC36	-6.9433994
TTC37	-1.2999239
TTC38	-0.690722
TTC39A	-1.7272696
TTC39C	-4.19636
TTC4	-0.9283333
TTC7	-1.024199
TTC8	-0.6835606
TTI1	-0.2092691
TTLL1	-0.5750928
TTLL12	-1.5722656
TTLL13	-1.9148297
TTLL5	-1.5183945
TTPA	-4.353772
TTR	-5.3454533
TTYH2	-4.2531385
TUB	-0.8980579
TUBA1C	-0.3571186
TUBA4A	-1.1648278
TUBB2A	-1.6398635
TUBB2A-PS2	-0.651156
TUBD1	-1.0380719
TUBE1	-0.8285625
TUBG1	-2.3479095
TUBGCP2	-0.5288057

TUBGCP4	-0.9191995
TUBGCP6	-0.5797868
TUFM	-2.6478462
TUG1	-0.2691436
TULP1	-0.4612792
TULP2	-1.6817358
TUSC2	-1.9805927
TUT1	-0.3719306
TVP23A	-2.4158666
TVP23B	-0.5399389
TWF1	-0.8785625
TWISTNB	-0.5104561
TWSG1	-0.9506235
TXLNG	-0.3851955
TXN2	-2.2895355
TXNDC12	-1.2529259
TXNDC15	-1.6677074
TXNDC16	-0.6804161
TXNDC17	-0.9221516
TXNDC5	-2.2659311
TXNDC9	-0.2625308
TXNL4A	-0.8033209
TXNL4B	-1.0969176
TXNRD2	-2.6442504
TYK2	-0.9068298
TYMP	-4.5362344
TYMS-PS	-1.0066233
TYSND1	-2.9241729
TYW1	-1.6042962
TYW5	-0.551352
UAP1	-0.3264575
UAP1L1	-1.3567977
UBA1	-0.6348805
UBA3	-1.1688476
UBA52	-1.2846651
UBAC1	-1.0606337
UBAC2	-1.0480781
UBAP1	-0.5036316
UBB	-0.2353125
UBC	-1.1530094
UBD	-0.7601848
UBE2A	-0.7596293
UBE2C	-0.7089548
UBE2CBP	-2.820042
UBE2D1	-0.377476

UBE2D2B	-1.3931465
UBE2D3	-0.3317118
UBE2DNL1	-1.6937118
UBE2E1	-0.3208947
UBE2E2	-1.4256268
UBE2E3	-0.7752338
UBE2G1	-1.0952506
UBE2G2	-0.4232001
UBE2J1	-0.1968184
UBE2J2	-0.231812
UBE2K	-0.1801152
UBE2L3	-0.669281
UBE2L6	-1.6207504
UBE2M	-0.7029905
UBE2N	-0.4652104
UBE2Q1	-0.1518693
UBE2QL1	-1.6596577
UBE2U	-3.4368787
UBE2V1	-0.9110727
UBE2W	-0.4234071
UBE2Z	-0.7816458
UBE3B	-1.1980443
UBE3C	-0.8338862
UBE4A	-1.838233
UBE4B	-0.9704332
UBFD1	-0.7537937
UBIAD1	-1.3636498
UBL4	-1.0202789
UBL4B	-2.5316432
UBL5	-0.8306322
UBL7	-1.0095162
UBLCP1	-0.4976983
UBOX5	-0.3090751
UBQLN3	-1.5490546
UBQLN4	-1.5713601
UBR2	-1.7580309
UBR3	-0.7554154
UBR7	-0.666254
UBTD1	-0.2123909
UBTF	-1.1007757
UBXN1	-1.7242427
UBXN6	-1.1937819
UBXN8	-0.8259115
UCHL4	-0.7152228
UCHL5	-0.1619711

UCK1	-1.7878985
UCKL1	-0.9812965
UCN	-1.690512
UCN3	-3.680481
UEVLD	-1.5140715
UFC1	-1.3933969
UFL1	-0.5448389
UFM1	-0.2799578
UFSP1	-1.2727776
UFSP2	-1.4805083
UGDH	-1.9009099
UGGT1	-0.2967382
UGP2	-3.401771
UGT1A10	-5.242904
UGT1A6A	-1.1328678
UGT1A6B	-4.4122806
UGT2A3	-7.572547
UGT2B1	-9.054056
UGT2B34	-7.81163
UGT2B35	-6.716181
UGT2B36	-8.572332
UGT2B37	-8.770136
UGT2B38	-9.048931
UGT2B5	-8.972843
UGT3A1	-6.378687
UGT3A2	-7.442036
UHMK1	-0.5020418
UHRF1BP1	-1.0682192
UHRF1BP1L	-0.4807711
UHRF2	-0.9713974
ULBP1	-0.7070899
ULK1	-0.5483818
ULK2	-1.2273064
ULK3	-0.7563682
UMODL1	-1.7183838
UMPS	-2.2518692
UNC119	-0.7130842
UNC119B	-1.3977365
UNC13A	-1.0752382
UNC45BOS	-1.2406023
UNC50	-0.9259663
UNC5A	-2.1526098
UNC5CL	-1.3900652
UNC80	-1.6920214
UNC93A	-2.8635252

UNC93B1	-0.9307189
UNG	-0.8052788
UNKL	-0.3821333
UOX	-5.184049
UPB1	-5.964579
UPF1	-1.8495188
UPK1A	-0.4848051
UPK3A	-1.574023
UPK3B	-0.6035395
UPP2	-9.130705
UQCC1	-2.0999436
UQCC2	-1.5043197
UQCR10	-2.0510054
UQCR11	-2.6377616
UQCRB	-1.997117
UQCRC1	-2.828179
UQCRC2	-1.965877
UQCRFS1	-2.3827767
UQCRH	-1.9179673
UQCRQ	-2.3231878
URAD	-2.850862
URAH	-3.5602365
URGCP	-0.8724022
URI1	-0.5957398
URM1	-0.6525445
UROC1	-9.498556
UROD	-2.5050216
UROS	-2.737069
USE1	-0.5226307
USF1	-0.2712207
USF2	-0.9689789
USH1C	-1.5262744
USH1G	-1.0690603
USH2A	-1.4901569
USHBP1	-2.3006978
USMG2	-2.173302
USMG5	-1.6877332
USO1	-0.6157713
USP10	-1.5152378
USP12	-0.4571405
USP15	-0.4490838
USP17LA	-1.3076537
USP17LC	-2.347426
USP17LD	-1.794174
USP17LE	-2.0353432

USP19	-0.486125
USP2	-2.0970716
USP21	-1.748538
USP24	-0.3460033
USP28	-0.5762529
USP30	-0.7850714
USP35	-0.7309878
USP38	-1.2605562
USP4	-0.5558868
USP40	-1.4044304
USP44	-1.3308933
USP45	-0.4325609
USP46	-0.6744537
USP5	-1.2321472
USP50	-1.5860891
USP8	-0.9102652
USP9Y	-2.1030543
USPL1	-0.2245507
UTP11L	-0.3410506
UTP14B	-2.6049032
UTP15	-0.4694922
UTP3	-0.4985924
UVRAG	-0.7125349
UXS1	-0.1752207
UXT	-0.8958335
V1RD19	-1.6340773
VAC14	-1.3017726
VAMP1	-0.9305089
VAMP7	-1.0830307
VAMP8	-0.396204
VAPB	-1.7496295
VARS	-0.7647371
VARS2	-1.3146844
VASH1	-1.6274328
VASH2	-1.4812171
VAT1	-0.7392163
VAV2	-1.3214126
VAX1	-1.6499355
VAX2	-1.7443502
VAX2OS	-1.2486501
VCP	-0.6997533
VCPIP1	-0.1470461
VCPKMT	-0.9322949
VDAC1	-1.9883261
VDAC2	-0.4317446

VDAC3	-0.9959021
VEGFB	-1.7650652
VGLL2	-1.1856704
VIMP	-0.4284782
VIPAS39	-0.4769688
VIPR1	-4.9936085
VKORC1	-4.7914476
VKORC1L1	-0.6509085
VMA21	-1.0266004
VMAC	-2.315084
VMN1R1	-1.0383024
VMN1R104	-2.0690904
VMN1R118	-1.7252147
VMN1R122	-1.3648086
VMN1R124	-1.6817596
VMN1R131	-1.671824
VMN1R132	-1.030827
VMN1R139	-1.6886086
VMN1R148	-1.2867193
VMN1R157	-1.6006827
VMN1R17	-1.7531867
VMN1R171	-1.7491872
VMN1R172	-1.6114497
VMN1R176	-1.6205344
VMN1R178	-0.8107522
VMN1R18	-1.7104108
VMN1R180	-1.6261954
VMN1R191	-1.398561
VMN1R192	-3.7833383
VMN1R193	-1.7409992
VMN1R194	-1.6114852
VMN1R20	-1.6379197
VMN1R203	-1.689857
VMN1R210	-1.2199988
VMN1R212	-1.3360701
VMN1R219	-1.5368805
VMN1R220	-3.3349798
VMN1R223	-1.6646342
VMN1R225	-1.4711068
VMN1R226	-1.2516551
VMN1R227	-0.981359
VMN1R228	-1.6415207
VMN1R230	-1.5839384
VMN1R231	-1.4387023
VMN1R235	-1.640718

VMN1R25	-0.7121491
VMN1R26	-1.7422469
VMN1R32	-1.6038995
VMN1R33	-2.8835523
VMN1R37	-1.1105182
VMN1R4	-4.4668636
VMN1R43	-1.6199121
VMN1R48	-3.5280044
VMN1R5	-1.7227914
VMN1R51	-1.8101552
VMN1R53	-3.563468
VMN1R55	-1.6247292
VMN1R56	-3.3579428
VMN1R58	-1.1503732
VMN1R64	-1.6200852
VMN1R67	-2.4204307
VMN1R72	-1.4809167
VMN1R74	-1.2847579
VMN1R75	-1.6927505
VMN1R76	-1.0455844
VMN1R77	-1.7113574
VMN1R8	-1.6307187
VMN1R83	-1.7033477
VMN1R84	-1.6325402
VMN1R86	-1.6550055
VMN1R-PS10	-1.6565742
VMN1R-PS79	-1.8627305
VMN2R1	-3.6534882
VMN2R10	-1.5276661
VMN2R100	-1.6752515
VMN2R101	-1.3041339
VMN2R102	-1.586905
VMN2R106	-1.5901494
VMN2R107	-1.6028533
VMN2R108	-1.6361265
VMN2R110	-1.1526527
VMN2R111	-1.4426374
VMN2R112	-1.6273158
VMN2R116	-1.8751035
VMN2R120	-1.8895102
VMN2R121	-0.7565613
VMN2R17	-1.6170249
VMN2R18	-1.6142473
VMN2R20	-1.4217787
VMN2R24	-1.6366656

VMN2R27	-1.2586076
VMN2R28	-1.6527419
VMN2R29	-1.4719839
VMN2R3	-0.5490038
VMN2R33	-1.6515801
VMN2R35	-1.2420945
VMN2R37	-1.6283367
VMN2R39	-1.5129104
VMN2R4	-0.9832194
VMN2R42	-1.6193838
VMN2R43	-1.0474145
VMN2R54	-2.260507
VMN2R56	-1.4349248
VMN2R58	-1.5014689
VMN2R59	-2.4354334
VMN2R62	-0.7796435
VMN2R67	-1.9135091
VMN2R77	-1.55968
VMN2R80	-2.4561236
VMN2R81	-1.2629254
VMN2R82	-2.187932
VMN2R83	-1.4926848
VMN2R85	-1.1652567
VMN2R91	-1.6587329
VMN2R94	-2.155592
VMN2R96	-0.9035294
VMN2R98	-0.8799233
VMN2R-PS54	-1.8947883
VMN2R-PS57	-1.6273808
VMO1	-5.6898165
VNN1	-1.7932482
VNN3	-1.932426
VPREB1	-2.0717251
VPS11	-0.9437108
VPS13D	-0.6646338
VPS16	-0.3141127
VPS18	-0.3156567
VPS25	-1.8729434
VPS26A	-0.4433093
VPS26B	-1.2254691
VPS28	-0.3781056
VPS29	-0.7462711
VPS33A	-0.6789722
VPS35	-0.751255
VPS37A	-0.9170013

VPS37B	-1.1420865
VPS41	-1.6224484
VPS45	-1.539526
VPS4A	-0.304421
VPS52	-0.4624462
VPS72	-0.1943255
VPS9D1	-0.8628736
VRK2	-0.8286805
VRK3	-0.638062
VRTN	-1.64908
VSIG1	-1.9514289
VSIG4	-9.938963
VSNL1	-0.5162935
VSTM2A	-0.7136922
VSTM2B	-2.7308939
VSTM2L	-1.6158257
VTA1	-0.3505368
VTN	-4.1575184
VWA5B1	-0.9786229
VWA5B2	-1.3844278
VWA8	-2.8855438
VWC2	-1.0672154
VWCE	-5.536394
VWDE	-2.6183357
WAPAL	-0.175137
WASF1	-1.6726851
WASF3	-0.5797279
WASH	-0.8821731
WASL	-0.9330149
WBP1	-1.8299217
WBP11	-0.1671877
WBP1L	-1.8293839
WBP2	-0.7136679
WBP4	-1.0867229
WBSCR16	-1.7503486
WBSCR22	-0.8743286
WBSCR27	-2.3510442
WDFY1	-0.4929586
WDFY2	-0.3206825
WDR11	-1.1438398
WDR12	-0.9112783
WDR17	-2.488937
WDR18	-2.2870803
WDR20	-0.6503215
WDR24	-1.8891649

WDR25	-1.7275059
WDR26	-0.1823783
WDR33	-0.7360444
WDR34	-1.722939
WDR36	-0.7660923
WDR4	-0.812037
WDR41	-0.4164057
WDR45	-0.2941837
WDR46	-0.6707468
WDR5	-1.2632194
WDR53	-1.0658212
WDR55	-0.980227
WDR59	-0.3434279
WDR6	-0.6969833
WDR61	-1.5260296
WDR62	-0.5105515
WDR64	-1.0848787
WDR70	-0.3926165
WDR72	-2.531302
WDR73	-1.0985222
WDR74	-0.926723
WDR77	-0.6615429
WDR81	-1.424736
WDR82	-0.9850736
WDR83	-1.0540295
WDR83OS	-1.3196669
WDR86	-1.0548041
WDR88	-1.1950793
WDR89	-0.8824329
WDR90	-1.0340002
WDR91	-0.4139757
WDR93	-1.4263208
WDTC1	-1.4078064
WEE1	-0.7870145
WEE2	-0.8815894
WFDC1	-0.8470049
WFDC11	-1.2168851
WFDC13	-1.6932604
WFDC15A	-2.0404346
WFDC8	-1.1615522
WHAMM	-0.7010679
WIPI2	-0.7578797
WISP1	-0.4571433
WNT2	-2.6439838
WNT3	-1.4810946

WNT5B	-3.3556018
WNT8A	-1.6875329
WNT9B	-4.114805
WRAP53	-0.6244226
WRAP73	-1.4397244
WRB	-0.699285
WRN	-0.5728564
WRNIP1	-1.6795254
WWOX	-1.3559139
WWP1	-1.1972866
XAB2	-0.5574484
XCL1	-0.6452403
XCR1	-2.6569176
XIAP	-0.3072863
XIST	-0.4322038
XK	-1.6627691
XKR4	-2.217393
XKR6	-1.48101
XKR7	-1.1987617
XKR8	-1.3213727
XKR9	-6.1679688
XLR	-0.5686834
XLR5B	-1.8001378
XLR5C	-1.4181046
XNTRPC	-0.2025311
XPA	-0.4811654
XPC	-0.9294038
XPO1	-0.398941
XPO5	-0.4569807
XPO6	-0.195291
XPOT	-0.8999596
XPR1	-0.4216161
XRCC1	-0.3375998
XRCC3	-2.4315898
XRCC4	-0.4422369
XRCC5	-1.0954351
XRCC6	-1.482449
XRCC6BP1	-2.6694453
XRRA1	-1.6442406
XYLB	-4.3323402
XYLT2	-1.9245458
YAF2	-0.5988793
YAP1	-0.2615218
YARS2	-1.7656589
YBEY	-2.0112267

YBX1	-0.4260426
YEATS2	-0.5265231
YES1	-0.7512052
YIF1A	-2.4144807
YIF1B	-1.4837174
YIPF1	-1.1137705
YIPF2	-1.584795
YIPF3	-1.5622077
YIPF4	-0.828927
YIPF5	-0.779429
YJEFN3	-0.3697324
YKT6	-0.2307377
YLPM1	-0.7294924
YME1L1	-1.3808489
YOD1	-0.9436002
YPEL2	-1.5431168
YPEL3	-0.7235136
YRDC	-0.1678252
YTHDF3	-0.6156802
YWHAB	-0.2835527
YWHAE	-0.1702523
YWHAG	-0.5936451
YY1	-0.5006294
ZADH2	-1.6411123
ZAN	-1.3179762
ZAP70	-0.4097562
ZAR1	-1.1082559
ZAR1L	-1.1602147
ZBED5	-1.5831366
ZBTB1	-0.1743116
ZBTB12	-0.450922
ZBTB18	-0.4836164
ZBTB20	-1.234601
ZBTB3	-0.587322
ZBTB33	-0.5297551
ZBTB39	-0.6706891
ZBTB40	-0.8718867
ZBTB44	-1.3210263
ZBTB45	-0.5505981
ZBTB48	-1.9417262
ZBTB7B	-1.0716863
ZBTB7C	-1.6666837
ZBTB8A	-0.9581428
ZBTB8OS	-1.2792206
ZBTB9	-0.2952161

ZBTBD6	-1.1090536
ZC2HC1B	-2.5369663
ZC2HC1C	-2.848624
ZC3H14	-0.7177644
ZC3H18	-1.1614261
ZC3H7B	-0.7825971
ZC3H8	-1.0333598
ZC3HAV1L	-1.6096127
ZC3HC1	-0.5644846
ZC4H2	-1.5434351
ZCCHC16	-1.0327415
ZCCHC17	-0.6551309
ZCCHC2	-0.4245577
ZCCHC24	-1.7493234
ZCCHC4	-0.6289337
ZCCHC5	-2.309787
ZCCHC9	-0.8288155
ZCWPW2	-1.1920507
ZDBF2	-1.4526093
ZDHHC11	-1.7966115
ZDHHC12	-1.304853
ZDHHC14	-0.876787
ZDHHC16	-0.3444743
ZDHHC2	-0.2088609
ZDHHC4	-0.7604704
ZDHHC5	-0.3072071
ZDHHC6	-1.8769612
ZDHHC7	-0.4879808
ZDHHC9	-2.6233358
ZER1	-1.0698528
ZFAND1	-1.6621294
ZFAND2B	-1.5180788
ZFAND3	-0.8668113
ZFAND4	-2.9939592
ZFHX2	-1.3258414
ZFHX2OS	-1.6600385
ZFHX4	-4.033253
ZFP108	-1.8372321
ZFP109	-0.8969376
ZFP112	-1.1814516
ZFP113	-1.0646293
ZFP114	-1.0776641
ZFP119A	-0.4867101
ZFP119B	-0.6814866
ZFP128	-0.8301673

ZFP13	-2.2356987
ZFP133-PS	-0.6883049
ZFP148	-0.3059568
ZFP157	-0.8681812
ZFP174	-2.585528
ZFP189	-1.2669501
ZFP191	-0.687408
ZFP2	-1.6676781
ZFP213	-1.1666107
ZFP219	-0.4941397
ZFP229	-0.7859645
ZFP235	-0.8947122
ZFP264	-3.673889
ZFP266	-0.3549366
ZFP27	-0.9051852
ZFP273	-1.019011
ZFP275	-0.5662437
ZFP276	-0.7600446
ZFP277	-0.9368167
ZFP280B	-0.856597
ZFP282	-1.6893036
ZFP286IT	-1.5986271
ZFP30	-1.1152477
ZFP300	-1.6757491
ZFP316	-0.8847961
ZFP318	-0.6854186
ZFP324	-0.8157485
ZFP335	-0.6146593
ZFP335OS	-0.4766769
ZFP345	-0.8772397
ZFP346	-0.6652319
ZFP354A	-1.7613626
ZFP354B	-2.8914113
ZFP358	-0.7852216
ZFP362	-0.3002815
ZFP367	-0.8870034
ZFP369	-0.4285743
ZFP37	-0.2515836
ZFP383	-0.4868357
ZFP385A	-2.0051668
ZFP385B	-4.40189
ZFP386	-0.131053
ZFP389	-1.971026
ZFP39	-1.400101
ZFP395	-2.6337838

ZFP398	-0.59443
ZFP408	-0.3600631
ZFP41	-0.6517205
ZFP418	-0.1714129
ZFP420	-0.670382
ZFP422	-0.4545603
ZFP426	-0.1600323
ZFP428	-0.4608598
ZFP438	-0.2130539
ZFP442	-1.0023456
ZFP444	-1.8868532
ZFP454	-3.104201
ZFP455	-0.802381
ZFP457	-0.3207536
ZFP467	-1.3625784
ZFP507	-0.5082166
ZFP523	-0.3014259
ZFP524	-1.6900334
ZFP526	-0.4930835
ZFP54	-0.9461474
ZFP551	-0.6949637
ZFP553	-0.2545404
ZFP563	-1.0930426
ZFP57	-0.6420448
ZFP574	-0.6729817
ZFP575	-1.1580672
ZFP59	-0.326062
ZFP598	-0.7628741
ZFP60	-1.4955435
ZFP606	-1.0885484
ZFP612	-0.5689926
ZFP617	-1.0709949
ZFP618	-1.3256948
ZFP619	-0.4060457
ZFP623	-1.7857754
ZFP629	-0.6420589
ZFP637	-1.4710236
ZFP639	-0.5928445
ZFP64	-0.2470121
ZFP646	-0.5354514
ZFP647	-0.6708012
ZFP648	-0.8311701
ZFP65	-0.3644385
ZFP655	-0.1950479
ZFP658	-0.5197959

ZFP661	-1.8516755
ZFP663	-0.4051414
ZFP672	-0.5074692
ZFP688	-0.8839259
ZFP689	-0.2066708
ZFP691	-1.3292351
ZFP706	-0.8212171
ZFP707	-3.0837402
ZFP708	-0.7711184
ZFP710	-0.5667186
ZFP72	-1.1437438
ZFP735	-0.9170976
ZFP746	-0.641078
ZFP748	-1.1634986
ZFP750	-2.7447195
ZFP759	-2.1427016
ZFP760	-1.6004229
ZFP763	-0.2910786
ZFP768	-1.0670905
ZFP770	-0.9690652
ZFP771	-1.7837076
ZFP772	-0.7048583
ZFP78	-1.0478973
ZFP780B	-0.9387014
ZFP784	-1.459619
ZFP786	-2.1841712
ZFP787	-1.5741959
ZFP788	-1.2468059
ZFP791	-1.3083296
ZFP799	-1.3979652
ZFP804A	-1.6760402
ZFP809	-0.2563644
ZFP820	-2.1274285
ZFP825	-1.9277375
ZFP846	-1.2246761
ZFP85	-1.2890306
ZFP87	-0.4622245
ZFP870	-0.7093236
ZFP872	-1.0244527
ZFP873	-2.177307
ZFP874B	-0.3571963
ZFP879	-1.9037771
ZFP882	-0.5644178
ZFP91	-0.6108327
ZFP930	-0.8803105

ZFP931	-1.0416126
ZFP932	-0.5807242
ZFP934	-0.4238114
ZFP936	-1.0949836
ZFP937	-0.5997019
ZFP938	-0.9545827
ZFP939	-1.6937449
ZFP94	-1.0264864
ZFP940	-0.7015328
ZFP941	-1.495451
ZFP944	-0.6356647
ZFP946	-1.6196861
ZFP949	-1.0192585
ZFP950	-0.7488005
ZFP951	-0.3559508
ZFP952	-0.401927
ZFP953	-1.0059519
ZFP954	-0.3192744
ZFP955A	-1.4059768
ZFP955B	-0.6061506
ZFP957	-0.2649598
ZFP958	-1.2883854
ZFP960	-1.0566678
ZFP961	-0.5331245
ZFP963	-0.4941857
ZFPL1	-0.6894717
ZFPM1	-1.960269
ZFR	-1.2148521
ZFY1	-1.3097651
ZFY2	-2.7168732
ZFYVE1	-1.8164353
ZFYVE19	-1.4599099
ZFYVE21	-2.2227402
ZFYVE26	-1.52812
ZFYVE28	-1.9252491
ZFYVE9	-0.759254
ZGPAT	-1.631002
ZHX3	-1.210597
ZIC1	-1.0948002
ZIC4	-1.3157275
ZIC5	-1.1813748
ZIM1	-1.3422484
ZKSCAN1	-0.6084642
ZKSCAN14	-1.1160212
ZKSCAN16	-3.0746303

ZKSCAN17	-0.5569801
ZKSCAN3	-0.3017435
ZKSCAN4	-1.8372707
ZKSCAN5	-1.0020394
ZKSCAN8	-0.6982675
ZMAT1	-1.3726618
ZMAT3	-0.4318614
ZMAT4	-1.3690608
ZMAT5	-1.2636614
ZMIZ2	-0.537849
ZMPSTE24	-1.1168337
ZMYM1	-0.6570191
ZMYM3	-0.937675
ZMYND19	-0.1799526
ZMYND8	-0.2323532
ZNF512B	-0.8476887
ZNHIT1	-1.2996345
ZNHIT2	-1.8292503
ZNHIT3	-1.0103617
ZNHIT6	-0.1870761
ZNRD1AS	-2.3594403
ZNRF2	-0.5885527
ZNRF3	-1.3738713
ZP1	-2.0712807
ZP3R	-2.3104022
ZPLD1	-1.5526125
ZPR1	-0.9173865
ZRANB1	-0.7476792
ZRANB3	-1.8049607
ZRSR1	-0.2796769
ZSCAN10	-0.7501235
ZSCAN12	-0.560328
ZSCAN2	-2.273354
ZSCAN20	-1.2400317
ZSCAN21	-0.3587513
ZSCAN22	-0.5402293
ZSCAN30	-0.9924054
ZSCAN5B	-1.5995142
ZSWIM3	-1.2042015
ZSWIM7	-1.7282052
ZW10	-0.4529448
ZWILCH	-0.9867985
ZWINT	-0.5934844
ZXDA	-1.1882601
ZZEF1	-0.5848031

A2M	7.2065787
A4GALT	2.7443328
AAK1	1.3366456
AATK	0.46369505
ABCA13	2.4918537
ABCA5	1.0903335
ABCA7	0.68332815
ABCB1B	2.0724988
ABCC1	2.1055856
ABCC4	0.77191114
ABCC5	2.4138522
ABCC8	2.4315066
ABCG1	1.1635904
ABCG4	0.77188253
ABHD10	0.46079636
ABHD12B	0.4730091
ABHD17B	0.60601425
ABI1	0.64861584
ABI3BP	0.40216637
ABL2	1.1587157
ABLIM1	1.0918784
ABR	0.35466385
ABRACL	0.5111575
ACAP2	0.7977457
ACBD7	1.9961214
ACE	3.4124956
ACE2	0.90740705
ACER2	2.6563773
ACER3	0.2334733
ACIN1	1.1716027
ACKR1	2.0599256
ACKR2	3.583117
ACKR3	5.6239386
ACOT10	1.7456484
ACOT11	1.0842681
ACOT7	0.1851492
ACOT9	1.626926
ACOXL	3.2790728
ACPP	3.5119743
ACR	1.9189188
ACSBG1	1.3422587
ACSL4	0.4519782
ACSS1	4.6325116
ACTA1	7.4759855
ACTA2	2.3175282

ACTC1	7.828638
ACTG1	0.56870604
ACTG2	3.3097267
ACTN1	1.0153422
ACTN2	3.0967836
ACTN3	4.4331603
ACTR3	0.8534827
ACVR1B	2.0386577
ADA	0.99431705
ADAM10	0.5015373
ADAM15	2.6910124
ADAM17	0.76101685
ADAM19	2.3637643
ADAM28	1.8852489
ADAM5	1.0757778
ADAM6B	1.0266879
ADAM8	4.68837
ADAM9	0.7043028
ADAMTS1	3.95584
ADAMTS10	0.38521552
ADAMTS15	5.281989
ADAMTS17	1.0086801
ADAMTS2	0.48466396
ADAMTS3	0.3919499
ADAMTS4	7.0927734
ADAMTS5	1.6260872
ADAMTS8	2.8479133
ADAMTS9	4.230517
ADAMTSL2	1.4552464
ADAMTSL4	3.0223527
ADAMTSL5	0.72309995
ADAP1	0.4906969
ADAR	0.5146613
ADARB1	1.1634073
ADC	1.0887959
ADCY2	3.5287135
ADCY4	1.4633937
ADCY7	0.394547
ADCY8	0.5544121
ADCYAP1R1	1.8081543
ADD3	1.01688
ADIG	1.9138579
ADIPOQ	1.9435654
ADM	2.9166384
ADNP2	0.5798702

ADORA2A	1.2464623
ADORA2B	5.587103
ADORA3	3.2844253
ADPRH	0.29191065
ADPRHL1	1.2195015
ADRA2A	0.6854391
ADRB1	0.24358153
ADRB2	2.944189
ADRBK2	0.6003299
AEBP1	2.6261678
AEBP2	0.935339
AFAP1	1.8625588
AFAP1L1	1.9590831
AFAP1L2	1.3049483
AFF1	1.4630108
AFF3	0.3518505
AFF4	1.1178994
AGAP1	1.679255
AGAP2	0.7376065
AGBL2	1.5770316
AGER	7.174642
AGFG1	1.3575649
AGO2	0.61030865
AGO3	0.3623898
AGO4	1.0877199
AGPAT4	4.4008875
AGPAT5	0.5970764
AGPS	0.7757335
AGR2	6.5733433
AGR3	4.6609035
AGRN	0.77945757
AGRP	1.696274
AGTPBP1	0.8940098
AGTRAP	0.5830946
AHCYL2	1.6944757
AHI1	2.2088456
AHNAK	2.7929115
AHNAK2	1.6070042
AHSP	0.4341576
AICDA	1.4669774
AIDA	1.1465149
AIF1L	2.3497734
AIRN	0.6405573
AK1	3.0148687
AK8	3.4734786

AK9	1.8419359
AKAP12	5.9166346
AKAP13	1.7116861
AKAP14	1.2667782
AKAP2	4.665906
AKAP5	6.998942
AKAP7	0.32310915
AKAP8	0.68348217
AKIRIN1	0.3199973
AKIRIN2	0.17980766
AKNA	1.5659981
AKR1B3	0.64495754
AKR1B8	1.6740813
AKT3	2.3781142
AKTIP	0.524879
ALCAM	1.0982218
ALDH18A1	0.42478848
ALDH1A2	4.5681214
ALDH1A3	3.9676023
ALDH1L2	1.0397727
ALDH3A1	2.514442
ALDH3B1	1.1117353
ALDH3B2	0.7473726
ALDOA	0.98965216
ALOX12B	1.0739849
ALOX12E	6.555781
ALOX15	1.2353365
ALOX5	3.6861782
ALOX5AP	1.53442
ALOXE3	2.728931
ALPK1	1.5371523
ALPL	0.33979607
ALS2CR12	1.1986711
ALX3	0.29588652
AMER1	0.47736216
AMER3	1.8700514
AMICA1	3.6281953
AMIGO3	0.30338073
AMMECR1	1.9314523
AMN1	0.4502678
AMOTL1	3.0520017
AMOTL2	0.95969963
AMPD1	1.4553063
AMPD3	3.6675138
AMPH	4.1467066

ANGPT1	1.25211
ANGPT2	5.5462933
ANGPTL7	1.900383
ANK1	1.2678173
ANK2	0.6071234
ANK3	0.8819506
ANKHD1	0.42517662
ANKK1	1.9777696
ANKLE2	0.54739404
ANKRD1	8.85924
ANKRD11	0.77627945
ANKRD12	2.1909251
ANKRD2	3.6364188
ANKRD22	2.32968
ANKRD23	1.2159972
ANKRD29	1.5274673
ANKRD32	0.6293347
ANKRD33B	0.8206444
ANKRD34A	0.7972598
ANKRD42	0.813395
ANKRD44	1.8554144
ANKRD45	0.79199505
ANKRD49	0.3553133
ANKRD50	0.21147251
ANKRD61	1.8591456
ANKRD66	1.0662458
ANKS1	0.3651147
ANO1	3.1286206
ANO6	2.0842676
ANO9	1.2638814
ANP32B	0.66665745
ANP32E	1.7674866
ANTXR1	2.8490233
ANTXRL	0.7593198
ANXA1	3.6835198
ANXA11	0.30202436
ANXA2	2.512857
ANXA3	3.489509
ANXA8	4.088089
AOAH	0.879622
AOC1	5.3410373
AP1M2	3.4579916
AP1S2	2.1364655
AP1S3	0.55763865
AP3B1	0.32087135

APAF1	0.9755349
APBA1	1.6339881
APBA2	0.64835334
APBB1	3.5707653
APBB1IP	1.03932
APBB2	0.95164204
APCDD1	1.1898198
APLF	0.6312213
APLN	6.345137
APLNR	1.6248503
APLP1	1.1040235
APOBEC2	1.8643334
APOBR	1.693088
APOD	9.883596
APOL10B	0.91247797
APOL6	1.3335619
APOLD1	2.3787193
APP	0.35473585
APPBP2	0.15914583
AQP1	0.31494188
AQP3	5.1139183
AQP4	0.859241
AQP5	7.0784
ARAP2	0.40344524
ARAP3	1.7382498
ARC	1.1547701
AREG	3.660187
ARF2	0.6303654
ARF3	0.19903994
ARFGAP3	1.2789273
ARG2	4.4412627
ARHGAP10	1.0613902
ARHGAP12	0.46244907
ARHGAP15	1.3509643
ARHGAP18	0.58399343
ARHGAP20	2.1653838
ARHGAP22	3.4982204
ARHGAP27	0.47386265
ARHGAP28	0.9807823
ARHGAP30	1.5547252
ARHGAP31	2.7321448
ARHGAP32	0.79277563
ARHGAP44	2.1105042
ARHGAP8	5.2059293
ARHGAP9	1.2174025

ARHGDIA	0.3900447
ARHGDIB	1.1998277
ARHGDIG	5.3739905
ARHGEF17	1.8938699
ARHGEF2	1.5699229
ARHGEF25	1.0446682
ARHGEF28	4.620964
ARHGEF3	0.5234585
ARHGEF38	1.6840785
ARHGEF4	0.67849946
ARHGEF5	0.5478587
ARHGEF6	0.62699556
ARHGEF7	0.24330854
ARID2	0.6690216
ARID3A	3.1437898
ARID4B	0.9028895
ARID5A	4.1456447
ARID5B	1.1577697
ARIH1	0.24878883
ARL10	0.8017764
ARL11	1.0008967
ARL13B	0.4171095
ARL14EP	0.87542963
ARL15	0.4314556
ARL2	0.48054075
ARL2BP	2.379047
ARL4A	0.19501066
ARL5B	0.50275517
ARL5C	1.361805
ARL6	0.41383505
ARMC10	0.4339533
ARMC2	2.8596714
ARMC3	3.7484505
ARMC4	4.490407
ARMC5	0.18734074
ARMCX2	1.2906728
ARMCX3	0.8684406
ARMCX4	1.273247
ARMCX5	0.19295597
ARPC1B	1.0394797
ARPC2	0.2698655
ARRB1	0.23127747
ARRDC1	1.5219798
ARRDC4	0.9243169
ARSJ	2.27115

ART1	4.865399
ART3	2.3398752
ART5	2.9173498
ARTN	5.4937286
ARXES2	2.6567032
ASAP1	0.20982742
ASB10	0.637691
ASB11	4.470525
ASB14	2.1052413
ASB4	2.052301
ASB5	2.5116143
ASCC2	0.3439598
ASCC3	0.1730485
ASCL1	1.0725689
ASCL2	2.0604243
ASCL3	1.7005384
ASF1A	0.53913593
ASH1L	1.3373804
ASNA1	0.2607913
ASNS	1.9509606
ASPH	0.1952982
ASPRV1	6.210242
ATAD2B	0.29108667
ATAD5	0.592437
ATF3	2.6758342
ATF4	0.7780032
ATF6	0.58386993
ATF7	2.133397
ATF7IP	0.4384346
ATG9A	0.4759202
ATL1	1.8685157
ATN1	0.2720394
ATP10A	3.4803429
ATP10B	2.0638382
ATP10D	1.346364
ATP11A	2.940723
ATP13A3	0.29248524
ATP1A2	2.837651
ATP1A3	1.4798594
ATP1B1	1.9352155
ATP1B3	0.33823824
ATP2A1	2.049892
ATP2A2	1.2082443
ATP2A3	1.7190981
ATP2B1	1.6543245

ATP2B4	1.023948
ATP2C2	2.2710445
ATP6V0A2	0.3906207
ATP6V0A4	7.138808
ATP6V0D2	1.6056919
ATP6V0E	0.26350546
ATP6V0E2	0.22987509
ATP6V1A	0.166296
ATP6V1B2	0.38823414
ATP6V1C2	7.5452003
ATP6V1G1	0.48621225
ATP7A	1.355854
ATP8A1	0.5051036
ATP8B4	3.3254173
ATP9B	1.5154617
ATRNL1	0.85672283
ATRX	0.5028515
ATXN1	0.20537567
ATXN1L	0.97922325
ATXN2L	0.22788239
ATXN7L1	1.7234182
ATXN7L2	0.34552908
ATXN7L3	0.30680466
AUTS2	1.3652663
AV356131	0.20625448
AVL9	0.6800132
AWAT1	2.489878
B3GALNT1	0.6651993
B3GLCT	1.39041
B3GNT2	0.7646189
B3GNT5	2.1088233
B3GNT7	1.6535368
B3GNT9	1.4635916
B4GALNT2	1.2489076
B4GALT1	0.24440956
B4GALT2	1.2687407
B4GALT3	1.1641154
B4GALT4	1.5272846
B630019K06I	1.2388463
B9D1	2.2985835
B9D2	0.27642965
BACH1	0.91364145
BACH2	0.47232437
BAG3	0.67496777
BARX2	2.2083693

BASP1	2.2311773
BATF	2.5877538
BATF2	1.8113384
BAX	1.2816038
BAZ1A	1.1274328
BAZ1B	0.20035172
BAZ2B	1.4615216
BBIP1	0.7031963
BBS1	1.2727149
BBS12	0.98162866
BBS2	2.0219204
BBS9	0.6940646
BBX	0.4076934
BCAM	1.9476466
BCAR3	0.8588128
BCAS3OS2	2.127878
BCAT1	1.3954945
BCL10	0.5018387
BCL11A	1.5929253
BCL11B	0.6936431
BCL2	1.0865707
BCL2A1C	3.919363
BCL2A1D	4.1602626
BCL2L1	1.4546766
BCL2L11	1.8221154
BCL2L14	2.9706578
BCL2L15	4.458628
BCL3	1.3257661
BCL6	1.4016328
BCL6B	3.338097
BCL9	2.523819
BCLAF1	0.9622946
BCR	0.635787
BDKRB1	3.7758753
BDKRB2	3.6149542
BDNF	5.336873
BDP1	1.1326938
BEAN1	5.274341
BEGAIN	2.906215
BEND4	0.7625394
BEND5	1.842613
BEND7	1.4924805
BEX1	1.5954494
BEX2	2.9850297
BEX4	2.574306

BFSP1	1.9639428
BFSP2	1.6615
BGLAP2	2.003771
BGLAP3	2.238791
BGN	0.6709571
BHLHA15	2.3194995
BHLHE22	1.8763673
BICC1	0.9781232
BICD1	2.7220924
BICD2	1.0506835
BIK	0.19964552
BIRC3	3.071001
BLNK	2.9537716
BLZF1	0.71276426
BMF	2.975359
BMP2K	1.6128349
BMP3	3.3873382
BMP4	2.1029367
BMP5	1.1418114
BMP6	1.0622997
BMPER	2.035358
BMPR1B	2.305198
BMPR2	3.249743
BMS1	1.533349
BMX	2.6290512
BNC2	1.4459596
BNIPL	1.571778
BOC	3.3505723
BORA	1.7843876
BPIFA1	10.922149
BPIFA5	1.9994073
BPIFB1	8.000013
BPIFB5	4.4994617
BPTF	0.32100058
BRAF	0.95715284
BRD1	1.7136269
BRD2	0.6956892
BRD4	0.3335948
BRD8	1.8780925
BRD9	0.20619678
BRINP1	3.0697105
BRIX1	0.6402807
BRMS1L	0.8657789
BROX	0.22849274
BRPF1	0.7928996

BRPF3	0.1758995
BRSK1	1.9667923
BRWD1	0.62532663
BRWD3	0.15591598
BSPRY	2.9231133
BST1	4.2825694
BTAF1	0.6142974
BTBD10	1.2195215
BTBD11	1.6974478
BTBD17	1.9855504
BTBD19	0.7910805
BTBD7	1.1459227
BTG1	0.70843744
BTG2	1.9948053
BTG3	3.4110565
BTK	1.0940089
BTLA	2.5660496
BVES	3.662264
BZW2	1.7309246
C1GALT1	0.36894083
C1QTNF7	2.2602978
C2CD3	0.77960587
C2CD4A	3.2541509
C2CD4B	7.4819803
C3AR1	1.1707668
C5AR1	2.8329628
C7	3.9511352
CAB39	0.41304207
CABYR	1.7443819
CACHD1	1.6687093
CACNA1C	3.2129717
CACNA1D	2.0253427
CACNA1S	1.910387
CACNA2D1	3.4146247
CACNA2D2	0.56573534
CACNA2D4	1.4144635
CACNB1	0.45416355
CACNB3	1.6161375
CAD	1.8777761
CALCA	6.058815
CALCR	2.9135382
CALCRL	1.7199621
CALM1	0.3385439
CALM4	4.4943476
CALML3	4.139661

CAMK1G	0.55746436
CAMK2B	0.19525313
CAMK2D	1.714241
CAMK4	1.8737667
CAMKK1	2.1027699
CAMKK2	0.6804576
CAMP	1.5941138
CAMSAP1	1.3944097
CAMSAP2	1.947633
CAMTA1	3.5590281
CANT1	0.24240708
CAP1	0.9168267
CAP2	2.555084
CAPG	2.7278523
CAPN1	0.48457336
CAPN13	2.968843
CAPN2	0.38370037
CAPN3	0.40033007
CAPNS2	1.9179482
CAPS2	2.261496
CAPSL	2.6487556
CAPZA2	0.3331933
CAR12	3.704955
CAR13	4.3833513
CAR15	0.32285643
CAR4	0.6754365
CAR9	0.20292592
CARD11	1.2655902
CARD14	1.5869431
CARTPT	0.71007824
CASC1	4.0699186
CASC3	0.1830287
CASC4	1.1221623
CASC5	1.1374378
CASK	0.48916292
CASKIN2	0.49213982
CASP1	0.5162144
CASP14	1.6616962
CASP2	0.9110527
CASP4	2.8428035
CASP8AP2	0.99732256
CASQ2	2.4086127
CAST	0.5585551
CASZ1	3.6035821
CAV1	2.2938156

CAV2	2.4731584
CAV3	5.5553417
CBFB	1.1386418
CBL	2.7272897
CBLB	1.9600844
CBR2	9.680853
CBR3	2.443677
CC2D1B	0.169168
CCBE1	0.6477227
CCDC102A	1.5376382
CCDC103	1.5734601
CCDC108	1.6236379
CCDC109B	1.9686408
CCDC11	4.8027945
CCDC113	6.4918284
CCDC114	0.4096527
CCDC115	0.24320412
CCDC120	1.6253538
CCDC121	2.7172954
CCDC122	0.42529607
CCDC127	0.25037003
CCDC129	0.36143517
CCDC13	2.5430412
CCDC134	0.67078114
CCDC135	1.5835619
CCDC136	1.1635847
CCDC146	0.616555
CCDC147	3.0573192
CCDC15	1.4245679
CCDC150	2.2923582
CCDC153	6.6003847
CCDC155	1.3968811
CCDC160	1.362072
CCDC17	1.1630921
CCDC170	3.3650217
CCDC173	2.3109326
CCDC174	0.6050067
CCDC176	1.2464108
CCDC181	1.3969383
CCDC184	2.4858336
CCDC19	5.78708
CCDC30	1.2102933
CCDC33	2.1596456
CCDC37	1.8390851
CCDC39	3.1718075

CCDC40	1.8455067
CCDC55	0.25490165
CCDC59	0.893518
CCDC64B	3.3616867
CCDC65	2.485494
CCDC66	0.62846947
CCDC67	1.222333
CCDC69	0.56898737
CCDC71L	0.97746706
CCDC77	0.52687883
CCDC78	3.4403012
CCDC81	3.5820189
CCDC85A	2.517708
CCDC88A	2.3118846
CCDC88B	1.5520749
CCDC88C	0.41129756
CCDC9	0.9892726
CCDC92	3.195003
CCKAR	5.0054755
CCL11	5.5829077
CCL12	3.6947422
CCL17	8.273239
CCL19	0.84732723
CCL2	4.757485
CCL3	6.53883
CCL4	5.2205024
CCL5	1.4234304
CCL6	1.2765617
CCL7	4.9070687
CCL8	1.6231565
CCM2L	1.3715711
CCNA1	2.1713903
CCND2	2.3981524
CCNG2	0.35839343
CCNJ	1.1068811
CCNJL	2.2190433
CCNL1	1.797184
CCNO	2.670817
CCNT1	0.35364914
CCNYL1	1.1431761
CCP110	1.616705
CCR10	0.8565016
CCR1L1	0.67022014
CCR6	3.0095925
CCR7	5.3706584

CCR8	1.2829981
CCR9	1.0606472
CCRL2	5.0130606
CCRN4L	0.35151386
CCSER1	0.8423929
CCSER2	0.31437254
CD101	0.5733988
CD109	0.87665606
CD14	3.3234658
CD163L1	2.08489
CD164L2	1.9889913
CD177	4.6263504
CD19	2.063467
CD2	1.8432856
CD200	5.6900983
CD209D	0.6984861
CD24A	4.82396
CD274	2.644576
CD28	1.5925064
CD300A	0.37654948
CD300LF	2.9811935
CD33	5.4307203
CD34	3.710321
CD36	0.7510772
CD37	0.88880587
CD3D	0.7945304
CD3EAP	0.5718055
CD3G	0.23918581
CD40	2.668353
CD44	1.7616911
CD46	0.6207979
CD47	0.16135263
CD5	0.42009068
CD52	0.6535735
CD53	2.5473537
CD55	0.6472006
CD6	1.4332328
CD63	1.6983638
CD68	0.5078788
CD69	4.6417212
CD70	1.0856862
CD72	0.28123522
CD79A	1.8077974
CD79B	1.3902636
CD80	0.8098655

CD83	1.1627526
CD84	0.22798824
CD8B1	1.2814999
CD9	1.390902
CD93	3.3717046
CD97	0.68383884
CDC14A	1.4707177
CDC20B	0.21168661
CDC25B	1.4985504
CDC27	0.27277517
CDC40	0.8685832
CDC42BPA	1.2503409
CDC42BPG	0.5696969
CDC42EP2	1.2762041
CDC42EP3	1.8976665
CDC42EP4	1.2096
CDC42SE1	0.9108987
CDC42SE2	1.2562613
CDC45	0.7477288
CDC5L	0.8384385
CDC73	0.46525002
CDCA4	0.64884186
CDCA7	0.9401078
CDCP1	1.9657607
CDH1	1.7390733
CDH11	0.5327196
CDH13	0.37985468
CDH15	2.198609
CDH16	4.95654
CDH22	2.07887
CDH5	2.4326844
CDH6	0.6018317
CDHR1	1.4401324
CDHR3	3.1101274
CDHR4	4.6430793
CDK11B	0.19212723
CDK12	0.5786009
CDK14	1.9092455
CDK19	0.44666433
CDK2	0.14747453
CDK5R1	0.9398625
CDKL1	0.89513874
CDKL2	1.3827043
CDKL3	1.0263546
CDKL4	1.2112346

CDKN1A	1.4450531
CDKN1B	0.665205
CDKN2B	5.3669114
CDKN2D	1.6419034
CDNF	1.0107567
CDON	0.6535089
CDPF1	0.16673756
CDR2	1.0783377
CDR2L	1.6421733
CDS1	3.6804047
CDSN	3.757984
CDYL	0.44758224
CDYL2	1.4177272
CEACAM10	5.8022757
CEACAM16	1.5498984
CEBPB	0.39219904
CEBPD	4.6307645
CEBPE	0.49688435
CECR2	0.600615
CEL	0.6866791
CELF2	1.2028112
CELF4	0.96582556
CELSR2	0.8347733
CELSR3	4.5369973
CEMIP	2.0549395
CENPB	0.4725976
CENPC1	0.17827415
CENPE	0.22523165
CENPT	0.27082014
CENPU	0.5200627
CEP104	0.79311705
CEP112	0.95375943
CEP112OS2	0.4197123
CEP120	1.2543552
CEP128	0.599025
CEP135	0.61860085
CEP162	0.53723717
CEP170	1.2673423
CEP192	0.40978575
CEP290	0.9837284
CEP55	1.230685
CEP70	0.8594413
CEP83	0.81804466
CERCAM	0.90995383
CERK	2.1451297

CERS3	2.239508
CERS4	1.1164756
CERS5	1.5931201
CERS6	0.95716524
CETN3	0.31674957
CETN4	1.8919706
CFD	4.8568244
CFL1	0.43809414
CFLAR	1.3001046
CFTR	0.4792502
CGN	0.86172104
CGNL1	0.27628803
CH25H	7.183262
CHAC1	2.5051117
CHAD	4.531857
CHAMP1	0.92563415
CHD1	0.89768934
CHD2	1.9298482
CHD3OS	0.97828746
CHD5	1.1835208
CHD6	0.8503401
CHD7	1.1162691
CHEK1	1.2329934
CHGB	1.7545896
CHIA1	8.181061
CHIC1	0.46574068
CHIC2	1.405057
CHIL1	3.880999
CHIL3	5.754044
CHIL4	5.5571346
CHIL5	2.6596828
CHIL6	1.0056129
CHIT1	2.7802808
CHL1	4.936417
CHML	1.2541208
CHMP4B	0.13383579
CHMP4C	1.1182597
CHN1	1.8133221
CHPF	1.8613477
CHRDL2	0.8936224
CHRM2	3.0229192
CHRM3	3.3297048
CHRNA1	0.531245
CHRNA10	2.1309383
CHRNA7	1.9779456

CHRNB1	3.2440066
CHRNB4	0.34744787
CHST1	5.5949564
CHST10	0.96929693
CHST11	4.990838
CHST12	1.9769082
CHST14	0.24623299
CHST3	1.8928556
CHST5	1.2257805
CHST8	3.1778116
CHSY1	1.1518989
CHTOP	0.24454975
CIDEA	4.5570297
CIDEC	2.4243078
CILP	1.452029
CIRBP	0.5397525
CISH	1.7294979
CITED1	2.424097
CITED4	0.73223114
CIZ1	0.40552473
CKAP4	1.6467898
CKAP5	0.54557705
CKLF	1.3205361
CKM	5.1026435
CKMT1	0.9910264
CKMT2	6.6989856
CKS2	1.3774397
CLASP1	0.31044292
CLASP2	0.19679451
CLASRP	0.19547749
CLCA3	6.1837773
CLCA4	4.3555346
CLCN5	0.5170121
CLCN6	0.3389485
CLCNKA	3.352077
CLCNKB	0.96071434
CLDN10	5.7931757
CLDN18	1.3436415
CLDN22	3.7501366
CLDN25	0.74938107
CLDN4	0.88523173
CLDN5	4.118408
CLDN7	4.5328107
CLDN8	0.6912329
CLEC2I	1.5643034

CLEC3B	0.23456502
CLEC4A1	1.2584391
CLEC4A4	1.5553758
CLEC4B1	0.30899668
CLEC4D	5.47831
CLEC4E	4.3590746
CLEC4N	0.16646624
CLEC5A	0.866091
CLGN	0.6017926
CLIC1	1.6322613
CLIC3	5.881249
CLIC4	2.8400292
CLIC5	4.494842
CLIC6	6.652522
CLIP1	0.99424314
CLIP2	1.2785745
CLIP3	2.4356375
CLIP4	5.933529
CLK1	0.51336575
CLK2	0.57261944
CLMP	2.8603787
CLNK	2.6157265
CLOCK	0.17567301
CLSTN1	1.8436241
CLSTN2	2.4245353
CLTB	0.71789885
CMIP	1.0427198
CMKLR1	1.4992054
CMPK2	0.6162143
CMTM2A	0.6247802
CMTM3	0.41342068
CMTM7	0.16028261
CMYA5	1.6536436
CNBD2	0.93428683
CNEP1R1	0.40834236
CNFN	6.4835863
CNGA2	2.1609383
CNIH2	0.19697833
CNIH4	0.7816324
CNKSR1	2.8489976
CNN1	4.9828286
CNN2	1.3571105
CNR1	1.6184568
CNRIP1	0.19802761
CNTLN	0.31700087

CNTN1	3.7303617
CNTRL	1.3312492
COG3	0.6412649
COG5	1.1174867
COG7	0.45792246
COL13A1	1.2725544
COL17A1	0.90927386
COL1A1	0.8451123
COL1A2	0.92870426
COL23A1	4.0986013
COL26A1	0.7831056
COL4A1	3.485456
COL4A2	3.1843548
COL4A3	3.5774088
COL4A4	1.5852437
COL4A5	1.5017471
COL4A6	0.99782467
COL5A2	0.65350795
COL6A1	2.4246302
COL6A2	1.3582196
COL7A1	2.5420294
COL8A2	0.57147074
COLQ	0.5436468
COMP	1.6020968
COQ10B	0.3890624
CORO1A	1.295774
CORO2A	1.676475
COTL1	0.54456425
COX4I2	1.2531247
COX6A2	6.1593204
COX7A1	0.5115533
COX8B	7.2794237
CPA3	2.5612206
CPD	1.91225
CPE	4.2104187
CPEB1	1.6113124
CPEB4	1.1997147
CPM	1.4794192
CPNE1	0.51383924
CPNE2	0.5458536
CPNE5	0.9371314
CPNE7	2.281767
CPNE8	1.6859794
CPSF2	0.4605217
CPSF6	0.5330181

CPSF7	0.5755248
CPT1B	0.34046578
CPXM1	0.22376108
CRABP2	0.24504185
CRAMP1L	0.61678267
CRCT1	5.215367
CREB1	0.953758
CREB3	0.23549366
CREB3L1	2.7341866
CREB3L2	0.6686063
CREB3L4	1.1809232
CREBL2	1.0230389
CREBRF	1.5788527
CRELD2	0.714643
CREM	0.8491454
CRH	0.47937036
CRHR2	1.5591264
CRIM1	2.1227512
CRIP1	0.4781642
CRISP1	1.0950298
CRISP3	2.8233502
CRISPLD1	0.8282716
CRISPLD2	6.0018287
CRK	0.74908686
CRLF1	4.996998
CRLF2	0.6269026
CRLF3	0.71938705
CRMP1	1.7798634
CRNDE	1.873357
CRTAM	1.0368414
CRY2	0.24657154
CRYAA	1.6519778
CRYAB	1.9682083
CRYBG3	2.577444
CRYZL1	0.31294584
CSDC2	0.29986238
CSF1	1.0551028
CSF2	5.5412235
CSF2RA	1.3021107
CSF2RB	1.2450933
CSF2RB2	0.95553493
CSF3	8.046267
CSF3R	0.99356747
CSGALNACT1	3.609693
CSGALNACT2	1.5204363

CSMD1	1.3604131
CSNK1A1	0.21262932
CSNK1D	0.41595507
CSNK1E	1.5740066
CSNK1G1	0.9012122
CSPG4	4.0293517
CSRNP1	3.7563806
CSRNP3	0.81416774
CSRP1	1.3497181
CST6	2.5759563
CST7	1.2837925
CST8	2.6491768
CSTAD	0.57046175
CSTF2	0.33340406
CTBP2	2.036871
CTBS	0.5999882
CTDP1	0.87746525
CTDSPL	1.0211415
CTDSPL2	0.56725574
CTGF	1.4051666
CTLA2A	4.459965
CTLA2B	3.7224922
CTLA4	2.512858
CTNNA1	1.6428509
CTNNAL1	0.19779062
CTNNB1	1.1639414
CTNND2	0.36662745
CTPS	3.6517568
CTSK	4.3999357
CTSS	0.598156
CTTNBP2NL	3.2830043
CTXN1	1.5404203
CTXN3	2.6771405
CUEDC1	1.024734
CUL3	0.43369055
CUL7	1.3379593
CUX1	0.51067114
CWC22	1.8699756
CWC25	0.17738914
CWC27	1.2528267
CWH43	0.7637129
CX3CL1	4.4861307
CX3CR1	0.9450669
CXCL1	4.8407164
CXCL10	2.8854737

CXCL13	1.6614156
CXCL14	7.74806
CXCL15	8.608767
CXCL16	1.6186166
CXCL17	9.459671
CXCL2	8.005163
CXCL3	6.8541846
CXCL5	9.573448
CXCR1	3.6259289
CXCR2	2.995656
CXCR4	2.9280877
CXCR5	2.146194
CXX1A	0.50634956
CYB561	3.0965843
CYB5R1	0.589756
CYBA	1.075324
CYBB	1.9064674
CYBRD1	1.337338
CYFIP2	2.0215716
CYLD	0.64007664
CYP19A1	1.0287013
CYP1B1	2.6143527
CYP27B1	3.1043828
CYP2S1	5.272146
CYP4B1	3.1571708
CYP4F18	2.1380315
CYPT4	1.4000764
CYR61	2.8100152
CYSTM1	0.8439679
CYTH3	3.1382012
CYTH4	0.5125756
CYTIP	4.1168647
CYTL1	3.6377263
CYYR1	3.982131
DAAM2	1.4254098
DAB2	1.2231922
DAB2IP	0.66550064
DACH1	3.8423562
DACT1	0.5672326
DACT3	0.7968936
DAF2	1.2230177
DAG1	0.61250734
DAGLA	1.84884
DAPL1	2.456394
DAPP1	1.9813213

DAW1	0.797246
DAXX	2.1629348
DAZAP2	0.29705238
DBF4	0.35379124
DBH	4.1688323
DBN1	4.367948
DBNDD1	2.783011
DCBLD1	0.2703042
DCBLD2	0.3343675
DCLK1	1.1925161
DCLK2	2.04671
DCLRE1C	1.9226453
DCP1A	0.3098774
DCP2	1.3601141
DCSTAMP	2.1316977
DCTN1	0.73771334
DCTN6	0.23736143
DCUN1D3	1.3313808
DDIT3	1.18191
DDIT4	2.108601
DDR1	2.7817817
DDR2	2.715528
DDX21	0.7093415
DDX23	0.5961275
DDX24	0.3473177
DDX26B	1.3843794
DDX39B	0.22737074
DDX3X	0.6380415
DDX43	0.6122713
DDX50	0.6143775
DDX55	0.45569372
DDX6	1.3606424
DEDD2	0.58357143
DEF6	1.3457236
DEFB14	2.0498672
DEFB3	2.8454156
DEK	0.28932142
DENND1B	0.841774
DENND1C	0.52888346
DENND3	2.7999816
DENND5A	0.51198816
DEP1	1.3594849
DES	2.219245
DGCR14	0.92470646
DGKA	1.416256

DGKD	0.5166707
DGKE	1.3860915
DGKH	3.7183967
DGKK	1.2747622
DHH	1.438005
DHRS7C	2.9363155
DHRS9	2.2282422
DHX38	0.34468746
DHX40	0.6534333
DHX9	1.9179304
DIAP1	0.90290356
DIAP2	0.60783625
DIAP3	0.3160596
DIEXF	0.36940956
DIMT1	0.42836618
DIO2	9.652472
DIP2A	0.706975
DIP2B	0.45579767
DIRAS2	4.01054
DIS3	0.7115421
DISP2	1.3613477
DKK3	1.767766
DKKL1	1.2978234
DLG5	0.9987216
DLGAP4	0.91085434
DLK1	0.86974597
DLL1	2.4160118
DMBT1	2.2664905
DMKN	5.453204
DMRT2	2.3503942
DMRT3	0.95298624
DMXL1	1.031713
DMXL2	1.138067
DNAAF3	0.4636674
DNAH10	3.0324562
DNAH11	4.142297
DNAH12	2.809088
DNAH2	4.034205
DNAH5	3.9452493
DNAH6	6.061873
DNAH7A	2.0709274
DNAH7B	3.1041949
DNAH8	1.5461068
DNAH9	2.465602
DNAIC2	4.272363

DNAJA4	1.38136
DNAJB13	1.4873986
DNAJB3	3.4263446
DNAJB4	0.25064516
DNAJB5	1.0860071
DNAJB6	0.6603284
DNAJC2	1.0225315
DNAJC21	0.5080147
DNAJC27	1.1463492
DNAJC5	0.29902315
DNAJC5B	1.7924893
DNAJC7	0.3870926
DNALI1	4.5762854
DNASE2A	0.8203993
DNHD1	0.9193196
DNM2	0.23360348
DNM3	1.0003035
DNM3OS	0.98696065
DNMT3BOS	0.5545821
DNMT3L	3.5395665
DNTTIP2	0.45906878
DOC2B	3.556512
DOCK10	1.673655
DOCK11	1.2261362
DOCK2	1.1569562
DOCK5	0.4048009
DOCK9	2.2024713
DOK1	1.0827999
DOK2	0.18289328
DOK3	1.3562422
DOK4	1.526084
DOK7	3.3916028
DONSON	1.1640496
DOPEY1	0.90028405
DPCR1	1.6703572
DPEP1	3.8499856
DPEP2	3.02142
DPH5	0.5291734
DPP10	0.6304188
DPP8	0.6772213
DPT	2.3631687
DPYSL2	1.5059271
DPYSL3	2.9750195
DRAM1	2.7588
DRC1	2.1682858

DROSHA	0.9348221
DSEL	1.3198547
DSG3	0.4623623
DST	1.0159683
DSTN	0.1764059
DSTYK	1.4879174
DTNA	1.6372299
DTX1	1.4066434
DTX2	1.9116006
DTX3	0.9507394
DUOXA1	1.3815567
DUOXA2	1.2095034
DUSP1	3.857088
DUSP11	0.79501605
DUSP13	1.2152278
DUSP14	1.014121
DUSP16	1.2796755
DUSP18	2.1271942
DUSP2	3.9149075
DUSP26	2.9294915
DUSP4	3.2240245
DUSP5	2.79953
DUSP7	1.2820086
DUSP8	3.2829816
DVL3	1.231308
DYDC2	2.4299078
DYNAP	1.5522134
DYNC1H1	0.5500159
DYNC1I2	0.58768797
DYNC1LI1	1.4214706
DYNC1LI2	1.4021964
DYNC2H1	1.3470964
DYNC2LI1	2.9674716
DYNLRB2	8.80906
DYNLT1A	0.78816867
DYNLT1C	1.0337367
DYNLT1F	0.67005396
DYRK3	1.3223963
DYSF	0.25615597
DYX1C1	1.1809108
DZANK1	2.63483
DZIP1	1.6179934
E2F2	0.65682316
E4F1	0.3242545
EAR1	4.0522494

EAR10	3.8875475
EAR12	4.2082705
EAR2	3.9892182
EAR6	2.2070785
EBF1	3.3563814
EBI3	2.363996
EBNA1BP2	0.22906494
ECM2	0.8160243
ECSCR	2.17487
ECT2L	1.9027202
EDIL3	2.524869
EDN1	4.9202104
EDNRA	1.7291737
EEA1	1.2201004
EED	0.29332256
EEF1A2	3.1962636
EEF1E1	1.1183119
EFCAB1	2.3982387
EFCAB10	4.5282474
EFCAB12	1.855454
EFCAB14	0.4938922
EFCAB4A	1.1112347
EFEMP2	0.78975105
EFHB	4.1146836
EFHC1	3.3240995
EFHD2	0.95327425
EFNA4	1.2684247
EFNB1	0.526773
EFNB2	2.3216267
EFNB3	0.27200246
EFR3B	3.887547
EFS	1.0249844
EFTUD2	1.5908709
EGFBP2	0.19810843
EGFL6	2.7183878
EGFL7	0.9178195
EGR1	0.41730022
EGR2	3.9559503
EHBP1L1	0.81090117
EHD1	1.3660254
EHD2	3.2438827
EHD4	1.9289122
EHF	4.300137
EID1	0.93145275
EID2	3.9040122

EIF2AK2	0.8426924
EIF2B2	0.319736
EIF2B3	0.29552364
EIF3C	0.5991993
EIF4A1	0.4107151
EIF4ENIF1	1.2874894
EIF5A2	0.8243532
ELAVL4	0.32562137
ELF1	0.86856365
ELF2	0.62656546
ELF3	2.5801086
ELF4	1.1586041
ELF5	5.431818
ELK4	1.2293129
ELMO2	0.7146597
ELMOD1	3.4171882
ELMSAN1	0.9634919
ELN	1.0540895
ELOVL1	0.1496191
ELOVL4	2.1038587
ELOVL7	1.6380758
EMB	4.255826
EMCN	1.0940475
EMD	0.43854332
EMID1	1.2962017
EMILIN2	2.1258817
EML1	3.585463
EMP1	4.5079474
EMP2	3.5100389
EMP3	1.3537674
ENAH	3.3857625
ENC1	2.355824
ENDOD1	2.0784945
ENDOU	0.60876846
ENG	0.43058062
ENKD1	0.5568061
ENKUR	4.1844397
ENO2	2.7049823
ENO3	1.5834808
ENO4	2.909813
ENPP4	1.0653744
ENTPD1	2.884561
ENTPD3	5.8374615
EOGT	1.3971562
EOMES	0.48299003

EP300	0.40212202
EPAS1	0.8851433
EPB4.1L2	1.6449103
EPB4.1L4A	1.139332
EPC1	0.5452509
EPC2	0.42985106
EPCAM	3.2727299
EPDR1	3.4764965
EPHA2	0.57496643
EPHA4	3.056079
EPHA8	1.8676498
EPHX4	3.7079318
EPN2	1.3484159
EPN3	0.8890171
EPS15	0.5731368
EPS8	1.9411278
EPS8L1	3.5431304
EPS8L3	1.0219197
ERBB2	2.6842203
ERBB2IP	0.5935888
ERCC1	0.87798595
ERCC5	0.251482
EREG	4.437129
ERG	2.450957
ERI1	0.66618633
ERICH1	0.625227
ERICH2	3.0290182
ERICH3	5.4724245
ERO1L	0.39776754
ESAM	2.456534
ESCO1	0.96294737
ESM1	2.0918303
ESR2	0.6966429
ESRP1	3.0489547
ESYT1	0.38986015
ESYT2	0.95545006
ESYT3	3.2532496
ETF1	0.4711547
ETL4	0.43286037
ETNK1	0.38278008
ETS1	1.5334663
ETS2	2.0293736
ETV1	0.6774001
ETV3	3.1949697
ETV5	1.128377

ETV6	0.76832294
EVA1B	0.41902733
EVA1C	3.8473196
EVC2	0.7923207
EVI2A	0.8003874
EVL	0.270689
EVPL	3.6613913
EWSR1	0.6064644
EXOC1	0.26292515
EXOC2	0.7683153
EXOC4	0.19583225
EXOC5	0.49619532
EXOC6B	1.4458432
EYA2	6.1446366
EYA4	1.2231836
EZH1	0.5554161
EZR	2.2270389
F13A1	4.8126597
F2R	2.1768146
F2RL2	0.64329004
F3	4.8698463
FABP12	1.3849623
FABP3	6.6053147
FABP4	3.049491
FADS3	3.185155
FAIM	0.22408009
FAIM3	0.8145304
FAM101B	2.6410878
FAM102B	0.8570647
FAM105A	3.18562
FAM107A	0.7507024
FAM110A	0.6450405
FAM110C	0.8641374
FAM111A	0.4170518
FAM115A	0.42880654
FAM117A	0.22704363
FAM117B	1.2929072
FAM118A	0.25170755
FAM124B	1.9233091
FAM129A	0.6888099
FAM129B	3.0456567
FAM129C	0.29804444
FAM131A	2.352652
FAM132A	0.6434126
FAM132B	1.5082331

FAM133B	1.2688603
FAM134B	0.7009492
FAM13B	0.20198488
FAM13C	3.7658625
FAM150A	1.8687518
FAM150B	0.44994545
FAM154B	4.079402
FAM160A1	0.8718219
FAM161A	1.1723218
FAM161B	1.8021436
FAM162B	3.944257
FAM166B	2.3131757
FAM167A	1.4605436
FAM168B	0.36259317
FAM174B	2.0647254
FAM178B	1.2172163
FAM179A	5.407874
FAM179B	0.6450181
FAM183B	6.3541665
FAM188A	0.40233278
FAM189A2	4.054531
FAM196A	0.6926522
FAM198B	2.7167506
FAM199X	0.9168966
FAM19A5	1.5834615
FAM206A	0.4042225
FAM208A	0.4622078
FAM208B	0.97191334
FAM209	0.43572164
FAM212B	3.6362805
FAM216A	1.1857367
FAM216B	4.7272143
FAM217B	1.4462852
FAM220A	0.3300047
FAM221A	1.5226665
FAM222B	0.23898935
FAM26E	1.609801
FAM3B	0.68479824
FAM43A	1.0931149
FAM46B	6.5000186
FAM46C	1.6921902
FAM49A	0.7110071
FAM49B	0.7711866
FAM53B	0.27738905
FAM57A	0.6321571

FAM60A	3.6731892
FAM63B	0.46034145
FAM65A	0.56261206
FAM65B	3.4340932
FAM69B	1.2823317
FAM71F2	3.354558
FAM73A	1.1068163
FAM76A	1.1062894
FAM76B	0.8162937
FAM78B	3.0854611
FAM81B	1.6880338
FAM83E	3.366245
FAM84A	0.37940073
FAM92B	5.123412
FAM98B	0.3893695
FANCA	0.4160552
FANK1	2.5716565
FAP	2.3911748
FAR1	2.3578644
FARP1	1.4838195
FAS	0.19836473
FASL	0.7055867
FAT1	0.38503885
FAT2	0.3941741
FBL	0.4968753
FBLIM1	2.4606376
FBLN1	2.0992432
FBN1	2.0831356
FBP2	3.7770627
FBXL12	0.5189953
FBXL13	1.7816765
FBXL14	0.3191433
FBXL16	2.7391646
FBXL2	0.7737596
FBXL20	0.41846132
FBXL22	0.522135
FBXL3	0.7473254
FBXL5	1.670013
FBXL7	0.27924967
FBXO10	0.47166634
FBXO11	0.16788626
FBXO15	0.34901452
FBXO2	1.6424456
FBXO27	1.3241308
FBXO28	0.48478556

FBXO30	0.26493835
FBXO32	2.2857213
FBXO33	0.7877469
FBXO4	0.7386794
FBXO42	0.7770853
FBXW10	1.5781324
FBXW11	0.23363304
FBXW17	0.9604559
FBXW7	0.64042807
FCER1G	1.4451642
FCER2A	1.3360276
FCGBP	1.5036633
FCGR1	0.32749176
FCGR4	0.9957142
FCHO1	0.7971678
FCHO2	1.0043912
FCHSD1	0.5343375
FCRLA	3.7326226
FCRLB	1.3221247
FCRLS	2.1430602
FEM1B	0.4651289
FEM1C	0.45640612
FEN1	0.61005354
FENDRR	1.9794323
FER1L4	1.8759613
FERMT3	0.43505335
FES	0.4051528
FEV	1.8895912
FFAR2	2.0124774
FFAR4	3.8323135
FGD3	1.3348274
FGD4	1.030623
FGF10	3.1837902
FGF11	0.7123091
FGF18	0.68637276
FGF2	0.1971159
FGF6	0.5836663
FGF7	4.857344
FGFBP1	4.884883
FGFR1	2.98215
FGFR2	0.42812538
FGL2	1.2271276
FGR	0.92225504
FHAD1	1.8003635
FHDC1	3.8122025

FHL1	3.4466448
FHL3	0.97366476
FHL5	0.9260528
FHOD1	0.23450994
FHOD3	2.4154606
FIBIN	4.966188
FIGF	0.7428055
FILIP1	1.5017478
FILIP1L	1.0244265
FIP1L1	0.18611765
FIRRE	0.5190768
FIS1	1.1390424
FJX1	2.3537984
FKBP10	1.6595569
FKBP14	0.3380432
FKBP1B	1.6804886
FKBP5	0.6305742
FKBP6	1.9190459
FKTN	1.1594913
FLI1	1.6038933
FLII	0.31004333
FLNA	1.6838555
FLNB	0.73902273
FLNC	4.7369657
FLOT1	0.73463774
FLRT2	1.0538774
FLRT3	4.6914196
FLT1	2.2755814
FLT3	1.539484
FMNL1	1.4265604
FMNL2	2.2069807
FMNL3	0.8271961
FMO2	3.1447654
FMR1	0.84956217
FNBP1	0.37618828
FNBP1L	1.4006634
FNBP4	0.21298313
FNDC1	1.2390842
FNDC3B	0.9556699
FNDC5	1.0496416
FNDC7	2.7539787
FOLH1	0.6252003
FOS	5.324674
FOSB	1.7573829
FOSL1	4.273935

FOSL2	3.4324708
FOXA1	1.2100458
FOXC1	2.4148896
FOXC2	2.914168
FOXF1	4.208596
FOXF2	4.0371914
FOXI2	1.3091402
FOXJ1	4.9119215
FOXJ3	1.4010568
FOXK1	0.51242447
FOXL1	1.3941748
FOXN2	0.50951433
FOXN4	2.6228385
FOXO3	0.8175416
FOXO6	1.7624094
FOXP1	1.8607106
FOXP3	1.5774806
FOXP4	0.22498178
FOXRED2	1.8302236
FPR1	1.436399
FPR2	2.8693657
FPR3	2.091085
FPR-RS6	1.5855772
FRAS1	0.5711398
FRAT2	0.77341604
FREM1	2.088735
FRG1	0.33828974
FRMD4A	0.42243958
FRMD5	2.8925042
FRMD8	0.31178188
FRMPD1	1.6842625
FRY	2.0317407
FRYL	0.79015636
FSCN1	3.3452172
FSCN3	0.8187821
FSD1L	1.7844043
FSIP1	1.4034746
FSTL1	2.6733332
FSTL3	3.5563264
FTSJ3	0.91829157
FUBP1	1.0207171
FUBP3	0.6033273
FUCA2	0.63592005
FUNDC2	0.7324467
FUS	1.1560192

FUT1	1.0685282
FUT11	0.7157669
FUT2	2.049193
FUT4	3.097041
FUT8	0.9180417
FXR1	0.13511372
FXYD2	0.43607855
FXYD3	2.959165
FXYD4	4.7741246
FXYD5	2.1538343
FXYD6	2.2559762
FYB	0.9979601
FYN	1.2369471
FZD1	1.5102377
FZD2	2.5517483
FZD3	3.6816666
FZD4	1.4300737
FZD5	0.8525212
FZD6	1.2374716
FZD9	2.863486
G2E3	1.5054853
G6B	0.69867873
G6PD2	1.5066257
GAB1	1.2292528
GAB2	1.2282534
GABBR1	0.8423016
GABPB1	0.7101774
GABPB2	1.807864
GABRE	0.84850335
GABRP	4.264512
GADD45A	0.70815134
GADD45B	3.2173328
GADD45G	1.7044864
GAL	0.8929455
GALC	0.76833534
GALNT1	1.7152743
GALNT12	1.9200239
GALNT15	5.389184
GALNT16	4.4697046
GALNT18	2.324924
GALNT3	3.1560335
GALNT6	1.8840504
GAN	2.1357827
GAP43	3.3030102
GAPT	0.68684435

GAR1	0.7691865
GARNL3	3.8303914
GARS	0.7792573
GAS2L1	0.8125739
GAS7	2.6577373
GAS8	1.4613366
GATA1	1.0543215
GATA2	4.148496
GATA3	1.6877444
GATAD2B	1.3810344
GATSL2	1.3759308
GBAS	0.68769646
GBP2	1.4681549
GBP3	0.5593929
GBP4	2.2011776
GBP5	3.0563698
GBP7	1.1632304
GBP9	0.85896206
GCA	1.1304791
GCC1	0.3427415
GCNT1	0.68549633
GCNT2	2.2761607
GCSAM	1.1484458
GDA	0.7235451
GDAP10	1.410162
GDE1	0.5913539
GDF9	0.34510326
GDI1	0.25325298
GDNF	5.0612373
GDPD1	4.4028807
GDPD2	8.95665
GDPD5	0.20479345
GEM	2.5719595
GFI1	0.6802659
GFI1B	1.3914192
GFPT1	0.9650693
GFPT2	4.6253614
GFRA4	3.6918297
GGPS1	1.5736566
GGT1	2.3913615
GGT5	0.7122326
GGT7	0.79368305
GGTA1	1.8168647
GHRL	1.1215358
GIF	1.399873

GIMAP1OS	0.810266
GIMAP3	1.1869159
GIMAP4	1.1366167
GIMAP5	2.780367
GIMAP6	1.9739528
GIMAP8	1.4008446
GIPC2	0.9223685
GIT1	1.406424
GJA1	3.8170938
GJA5	1.6149688
GJB3	5.032886
GJB4	1.8913212
GJB6	4.437649
GJC1	2.8498564
GK5	1.5156205
GKN3	0.48772955
GLB1L2	2.6043665
GLCCI1	0.75270987
GLG1	0.61619425
GLI1	0.5549488
GLI3	3.000102
GLIPR1	2.019586
GLIPR2	1.7996273
GLIS3	1.964905
GLS	2.44177
GLTP	0.16578245
GLTSCR1	0.89247155
GLYCAM1	4.344466
GM10021	0.9782336
GM10044	0.7447579
GM10046	1.5943837
GM10065	1.989311
GM10091	2.0198154
GM10099	1.1053965
GM101	1.7644861
GM10183	0.6001699
GM10325	1.8232875
GM10336	0.36040497
GM10354	3.5497396
GM10371	1.3077295
GM10406	2.304168
GM10425	1.1216469
GM10427	0.73397326
GM10537	1.4333827
GM10554	2.0753224

GM10631	0.4047923
GM10634	2.343708
GM10638	0.992275
GM10648	1.3318479
GM10673	2.1103137
GM10674	0.8451958
GM10693	0.8469653
GM10700	1.9945896
GM10731	0.83944225
GM10781	1.3453343
GM10791	2.540862
GM10792	2.11517
GM10796	0.81421447
GM10839	4.373183
GM10847	0.5103016
GM10851	3.7097595
GM10856	0.16232681
GM10866	0.59610844
GM10872	0.3140006
GM1096	1.342246
GM11110	2.0170186
GM11190	1.0467556
GM11346	1.0292401
GM11517	0.7522106
GM11536	2.0088143
GM11627	1.4065688
GM11696	0.6454592
GM11744	1.5199313
GM11938	1.188966
GM11974	0.81199694
GM12250	0.727077
GM12592	1.763377
GM12666	0.48904562
GM12695	0.44059777
GM128	0.14789891
GM1305	1.0120811
GM13154	1.240665
GM13157	0.29181337
GM13212	0.56656694
GM13272	0.8489075
GM13275	0.34058762
GM13298	1.4725332
GM13304	1.7035847
GM13375	0.8704288
GM13391	0.74050283

GM13547	1.5049701
GM13570	1.7647798
GM13889	5.903172
GM13931	0.23763514
GM14010	1.061286
GM14085	1.2173338
GM14420	0.4587667
GM14548	1.1306477
GM14625	0.97879887
GM14635	1.6015875
GM14858	0.3187468
GM14964	1.1143997
GM15056	3.4213328
GM15348	0.24075031
GM15413	0.49220037
GM15455	0.65976906
GM15466	1.0883145
GM15645	1.9194002
GM15998	2.709092
GM16340	1.0612068
GM16367	1.504431
GM16500	0.59740686
GM16523	0.4788444
GM16524	1.0371208
GM166	0.5962255
GM1673	0.7053137
GM16894	2.3547108
GM17019	1.709491
GM1715	1.6830018
GM17501	0.47507167
GM17758	0.76527643
GM17778	0.74928546
GM17821	1.1269469
GM1966	2.4449477
GM1987	1.9489446
GM2030	0.41276908
GM2042	1.2966795
GM2044	0.5631187
GM20556	1.5114381
GM20558	7.2332993
GM20611	1.5627823
GM20767	1.945384
GM21944	5.1066694
GM2447	0.96134377
GM2511	2.712713

GM2590	1.308526
GM266	1.0822561
GM2694	1.4323275
GM2696	0.697598
GM2701	0.48428202
GM3081	1.3625853
GM3086	0.7511642
GM3099	1.3066096
GM3161	2.8424125
GM3239	2.0999284
GM3252	1.5681415
GM3364	1.9406562
GM3367	0.8173702
GM3383	1.4711342
GM3414	1.890203
GM3556	0.36428332
GM3893	1.4930191
GM3916	1.5301354
GM4120	0.6795306
GM4123	0.5988195
GM4349	0.7620611
GM4425	0.2312994
GM4470	1.6876526
GM4610	6.421304
GM4759	0.9122441
GM4841	0.16787004
GM4884	0.7677398
GM4907	5.3614383
GM4956	5.2982993
GM4983	1.3197851
GM5067	2.6464686
GM5068	2.365584
GM5088	0.43731117
GM5092	0.23589182
GM5106	1.6722007
GM5107	0.66747546
GM5115	0.8250854
GM5150	0.3943329
GM5176	1.2446241
GM527	1.7275717
GM5396	1.1235733
GM5416	4.8238907
GM5483	6.2109313
GM5485	0.7739496
GM5486	0.33486795

GM5532	1.3994067
GM5547	1.0075119
GM5595	0.21737766
GM5602	1.4242373
GM5608	0.7688036
GM5640	0.85413766
GM5643	0.4072547
GM5797	1.6616151
GM5820	1.347976
GM5858	1.0165362
GM5862	0.7675128
GM5878	1.6462493
GM5936	1.19449
GM6115	1.0805593
GM6116	0.28641057
GM614	1.1248424
GM6146	1.5858383
GM6211	0.22045994
GM6252	0.34501934
GM6329	0.5533395
GM6377	3.574789
GM6403	4.724528
GM6524	0.74520206
GM6557	1.5642619
GM6579	0.18928003
GM6625	0.63919115
GM6756	2.1770558
GM684	1.5304749
GM6934	1.3263173
GM7068	1.6854177
GM7111	1.1266422
GM7285	1.6052532
GM7420	1.5657625
GM7609	0.43815374
GM7644	0.45814872
GM7710	1.4901996
GM7931	1.1899614
GM7932	1.5406487
GM7967	1.2828319
GM807	1.6935353
GM8096	1.8438053
GM8232	1.1123819
GM8439	1.0620587
GM8556	1.6566954
GM867	4.3673844

GM8675	0.6615951
GM8884	3.6249156
GM8995	2.708291
GM9049	0.39988375
GM9099	3.194146
GM9257	1.4841132
GM94	4.354566
GM9456	1.855809
GM9465	0.80954385
GM9510	1.3414443
GM9648	0.8846626
GM9693	1.4145489
GM9706	2.8027601
GM9733	0.9348006
GM9758	2.010123
GM9798	0.8091769
GM9855	0.7840061
GM9856	0.682703
GM9868	1.0951123
GM9889	3.0146878
GM9910	1.6231902
GM9917	1.69156
GM9920	0.50945234
GM9926	1.6838078
GM9948	0.94949627
GM9975	1.7194021
GMDS	0.15916967
GMEB1	0.5804491
GMFG	1.4346809
GMIP	0.5800681
GMPR	2.702506
GNA13	1.0937729
GNA14	2.4823546
GNAI1	1.9498744
GNAI2	0.33216572
GNAL	2.2870824
GNAO1	2.8566258
GNAQ	0.7829528
GNB1	0.5033541
GNB4	0.92256165
GNB5	1.4316125
GNG12	0.955791
GNG13	5.179943
GNG2	0.92865276
GNL1	1.3154545

GNL3	0.63027525
GNL3L	1.6045637
GNPDA2	1.3135619
GNPTAB	1.2764864
GOLGA2	0.2835307
GOLGA3	0.44119024
GOLGB1	0.22348118
GOLPH3	0.2709446
GOLPH3L	1.0681834
GOLT1B	1.5289583
GON4L	1.279839
GP1BA	1.6379514
GP2	5.0623064
GP49A	3.3029919
GP6	0.3757887
GP9	0.8382828
GPA33	1.0354176
GPANK1	0.4168725
GPATCH8	0.53446054
GPBP1	0.19524622
GPC1	0.84576225
GPC3	0.83946323
GPCPD1	1.5234709
GPHA2	0.87960315
GPKOW	0.58801794
GPM6A	1.0704741
GPM6B	4.1676016
GPNMB	5.233681
GPR113	2.7429876
GPR114	1.2198749
GPR116	2.938017
GPR126	1.5212646
GPR132	2.748435
GPR133	2.8527637
GPR137B	1.5470152
GPR137B-PS	1.9923553
GPR141	3.2410502
GPR146	0.41311502
GPR153	0.94478846
GPR160	0.740365
GPR171	3.55764
GPR174	1.2332067
GPR179	2.7221363
GPR18	2.798338
GPR183	1.4037104

GPR20	0.711864
GPR25	0.65853095
GPR27	0.5711789
GPR31B	1.9194934
GPR33	1.9762828
GPR35	0.9534662
GPR4	2.589366
GPR56	2.1539636
GPR6	0.8554139
GPR65	0.55839586
GPR68	1.0626674
GPR82	1.776211
GPR84	4.660898
GPR85	2.2379324
GPR87	1.6964068
GPR88	1.8028016
GPR97	0.14103508
GPRASP1	0.5717006
GPRC5A	3.490933
GPRC5B	5.7025967
GPSM1	1.0915284
GPSM3	0.62744427
GPX2	4.9070983
GPX3	4.869183
GPX8	2.556345
GRAMD1A	0.709075
GRAMD1B	1.9322519
GRAMD2	3.5425043
GRAMD4	0.515996
GRAP	1.7446504
GRAP2	0.79058003
GRASP	1.054636
GREB1	0.8856282
GREM1	3.1158626
GREM2	0.27176046
GRHL1	1.3255377
GRHL2	3.7259412
GRHL3	2.5049887
GRIN2C	4.1339617
GRK4	0.77311635
GRK5	2.5780983
GRP	1.8222568
GRRP1	4.7703667
GSDMA	3.6019561
GSDMC	0.9541931

GSDMC3	0.642534
GSDMC4	0.78330255
GSG1	1.5161142
GSG2	0.57736754
GSK3B	0.46723032
GSN	3.8144612
GSTO2	0.68048096
GTF2F1	0.7536192
GTF2F2	0.9473605
GTF2H1	0.28800774
GTPBP2	0.77547693
GTSF1	1.809166
GUCA2A	0.2573092
GUCA2B	0.552078
GUCY1A3	0.50548315
GUCY1B3	0.8506918
GULP1	1.4909699
GVIN1	1.7787628
GXYLT1	1.1704578
GYG	1.3664508
GYS1	1.7983017
GZMA	1.3248725
GZMB	1.760294
GZMC	2.4310632
GZMK	1.5430565
GZMM	0.19088292
H2AFY2	0.5472665
H2AFY3	1.6076541
H2-EB2	3.179714
H2-T24	2.0622087
H3F3B	0.9403763
H60A	1.7503984
H60B	2.4951015
HACE1	0.59673023
HAP1	1.7062721
HAS1	7.5788207
HAS2	1.9987178
HAUS6	0.44212294
HAVCR2	3.8132982
HBB-B1	0.61222124
HBB-B2	0.38966084
HBEGF	4.1092362
HBQ1B	1.2028224
HCAR2	3.396492
HCK	1.4818735

HCLS1	2.0419626
HCRTR1	0.57841516
HCST	1.0495701
HDAC1	0.25656414
HDAC4	1.2246585
HDAC7	1.9884233
HDAC9	0.5861554
HDC	4.4994674
HDDC2	0.2916808
HDGFRP3	2.429504
HDX	1.6555624
HEATR1	0.54695606
HEATR3	0.3980484
HECA	0.15353394
HECW2	0.9937115
HEG1	2.1483848
HEPACAM	1.0499084
HERC1	0.33620548
HERC3	0.2094531
HERC6	1.1726785
HES1	0.49357677
HES2	0.5818322
HEY1	3.4242048
HEY2	1.9431527
HEYL	2.1170006
HHATL	2.3465576
HHIP	0.31815863
HIC1	0.22654438
HID1	3.0263782
HIF1A	2.37042
HIF1AN	0.66578674
HIF3A	3.480293
HIGD1B	2.2654138
HILPDA	3.1254811
HINFP	0.18300414
HIP1	0.25160456
HIP1R	0.755239
HIPK1	1.5068517
HIPK3	0.39861488
HIRA	1.0491853
HIST1H1A	0.450521
HIST1H1B	1.3906336
HIST1H1E	0.91997147
HIST1H2AB	1.5264568
HIST1H2AG	0.24137402

HIST1H2BB	1.214339
HIST1H2BE	3.218068
HIST1H3A	1.3231988
HIST1H3C	0.7827859
HIST1H3D	0.94839907
HIST1H3F	1.2812169
HIST1H3G	1.9076207
HIST1H3I	0.769336
HIST1H4C	1.2913556
HIST1H4D	2.5597868
HIST1H4J	1.6424232
HIST1H4K	2.1805534
HIST1H4M	0.8454957
HIST2H2BB	0.34581184
HIST2H3B	1.0717297
HIST2H4	1.895421
HIST4H4	2.055563
HIVEP1	1.038064
HIVEP2	3.0158124
HK2	2.0814304
HKDC1	5.4514356
HLX	1.4042239
HMGA1	0.6919193
HMGA2-PS1	0.5394993
HMGB1	0.5461979
HMGB1-RS17	0.6340251
HMGB2	1.8003082
HMGB4OS	0.65778637
HMGCLL1	0.92497396
HMGN3	2.082934
HMGXB4	0.19836092
HMHA1	1.0773478
HMOX1	2.1216912
HMX3	1.3542979
HNRNPA1	0.78149366
HNRNPA3	0.88509655
HNRNPD	0.55710936
HNRNPDL	0.9435024
HNRNPH1	0.86552715
HNRNPH2	0.29447126
HNRNPL	0.9522028
HNRNPUL1	1.0133281
HOMER3	0.8972025
HOPX	2.067799
HOXA3	1.7193711

HOXA4	1.4483182
HOXA5	4.2371664
HOXA7	0.729789
HOXB2	1.2844405
HOXB3	1.0070741
HOXB4	1.3059764
HOXB5	1.2021298
HOXB5OS	1.9962277
HOXB6	3.6336572
HOXB7	0.8092468
HOXD8	1.0746939
HPCAL1	0.60887575
HPGDS	1.9357374
HRC	3.7291498
HRCT1	3.1674304
HRNR	3.4276547
HS3ST1	0.89141345
HS6ST2	1.4503355
HSD17B14	1.0515702
HSDL1	0.57266235
HSH2D	2.4420357
HSPA12B	1.5782423
HSPA2	1.5885916
HSPA4L	1.0030127
HSPB1	6.246183
HSPB2	0.88557625
HSPB6	0.3842163
HSPB7	2.8144083
HTATSF1	1.499609
HTR2B	4.4110765
HTR7	4.8206177
HTRA1	2.2732491
HTRA3	0.25559282
HTT	2.0477247
HUS1	0.52335405
HUWE1	0.21311855
HVCN1	1.3496199
HYDIN	2.8037112
HYOU1	0.34786224
ICA1	1.9701381
ICAM1	3.3403993
ICAM2	1.327426
ICAM5	1.8685026
ICOS	0.6199961
ICOSL	2.3906593

ID1	0.96322155
ID3	0.545599
IDO1	0.89231443
IDS	1.6067758
IER2	1.2036185
IER3	5.231737
IER5	1.6366782
IER5L	3.1136127
IFI202B	1.7217946
IFI204	1.1162572
IFI27L2A	0.68051624
IFI47	0.8051219
IFIH1	0.2952175
IFIT1	1.8293304
IFIT2	1.2967954
IFIT3	1.3492942
IFITM1	2.8743567
IFITM10	0.34425783
IFITM5	2.8649728
IFITM6	4.3384113
IFNA1	0.16362357
IFNA14	0.5930052
IFNA5	0.87249565
IFNAB	0.97302294
IFNG	4.575471
IFNGR1	0.77543116
IFNLR1	3.0288448
IFRD1	2.0623827
IFT122	0.8446703
IFT140	0.3758464
IFT43	1.4960871
IFT57	2.6274505
IFT80	0.43305302
IFT81	1.3757553
IFT88	0.6094675
IGF1R	2.8095655
IGF2BP2	1.040834
IGFBP3	1.4336133
IGFBP5	2.6094751
IGFBP6	5.1705093
IGFLR1	0.49654913
IGHV14-2	2.8346715
IGH-VJ558	3.290441
IGJ	3.1736054
IGKV4-53	0.19722366

IGKV6-20	4.5988116
IGLV1	1.5066428
IGSF1	1.3561685
IGSF23	0.5071213
IGSF3	1.8971281
IGSF6	0.75402546
IK	0.19546747
IKBKE	0.77524424
IKZF1	1.4038396
IKZF2	0.944361
IKZF4	3.5686002
IKZF5	1.6993012
IL10	3.2818475
IL10RA	0.26600552
IL10RB	1.1920409
IL11	5.247109
IL12B	2.0384638
IL12RB2	0.6264956
IL16	0.98986053
IL17A	4.654379
IL17D	1.1243224
IL17F	3.6789186
IL17RA	2.0093846
IL17RD	2.1577473
IL17RE	3.2344165
IL18R1	3.4209142
IL18RAP	3.772426
IL19	0.7631035
IL1A	1.5567865
IL1B	3.7900834
IL1F6	5.8234925
IL1F8	1.0825574
IL1F9	6.3062825
IL1R2	7.9986944
IL1RL1	3.3952246
IL1RL2	0.96919036
IL1RN	3.037136
IL20RB	1.5779648
IL21R	0.97877145
IL22	0.78087616
IL22RA2	2.5328484
IL23A	2.3482237
IL23R	0.9036491
IL2RA	4.819016
IL2RB	1.0919466

IL2RG	1.2892513
IL33	2.6481495
IL3RA	0.44489384
IL4I1	3.6551342
IL4RA	2.5053735
IL6	9.970649
IL6ST	0.54155016
IL7R	5.643655
ILDR1	3.8014665
ILF2	0.8809352
IMPACT	0.21614933
IMPDH1	0.8075237
INADL	1.1465921
INCENP	0.4602766
ING1	0.4990778
ING3	1.3985691
ING5	0.5163703
INHA	2.3208475
INHBB	6.695317
INIP	0.36547232
INO80	0.5103755
INO80B	1.3367572
INO80D	2.2113895
INO80E	0.6027813
INPP4A	1.3958721
INPP4B	0.55357695
INPP5B	0.16314316
INPP5D	1.6885242
INPP5J	0.8444557
INPP5K	0.40121222
INSL6	0.6388979
INTS12	0.8912802
IP6K3	2.418774
IPO13	0.6310015
IPO7	0.5094485
IQCA	4.028528
IQCB1	1.2108271
IQCF1	1.1687572
IQCK	0.9626341
IQGAP1	2.5500941
IRAK3	2.5157876
IRF1	0.71927166
IRF2BP2	0.55359983
IRF2BPL	0.31828976
IRF4	2.3242917

IRF7	0.1748352
IRG1	3.7573962
IRS2	1.7258911
IRX1	8.916662
IRX2	4.0495486
IRX3	7.1910133
IRX5	3.4755316
ISG15	2.1820116
ISG20	2.9167676
ISLR2	3.2908137
ISM1	4.9650345
ITGA1	0.9506445
ITGA11	1.4552181
ITGA2	0.6890826
ITGA2B	1.1161757
ITGA3	2.4683657
ITGA4	1.3683
ITGA6	2.045701
ITGA7	1.3154354
ITGAE	1.6503606
ITGAM	1.5239978
ITGAV	0.6529298
ITGAX	2.6492462
ITGB1	0.5748148
ITGB1BP2	3.0825844
ITGB2	0.9821358
ITGB2L	3.9498715
ITGB3	3.0878954
ITGB4	2.5462008
ITGB6	3.7605484
ITGB8	1.323009
ITGBL1	1.3514528
ITK	1.4300728
ITLN1	6.1969485
ITLNB	1.7003953
ITM2A	1.6524863
ITPKB	2.5307722
ITPKC	1.2235074
ITPR3	1.8189054
ITPRIP	4.022718
ITPRIPL2	1.7985969
ITSN2	0.7138295
IWS1	0.68726087
JADE2	1.041326
JAG1	2.0302835

JAG2	2.1347225
JAK2	1.3125324
JAK3	0.29992437
JAKMIP1	1.976969
JAM2	0.5825348
JAM3	1.382802
JDP2	2.4829412
JMJD1C	1.3619657
JMY	0.6829295
JSRP1	1.5923214
JUN	3.9256206
JUNB	2.8101377
JUND	0.58947563
JUP	1.1045372
KANK3	1.3043952
KANK4	0.7290771
KANSL1	1.075258
KANSL3	0.4235325
KAT5	0.5765314
KAT6A	0.4357605
KAT6B	0.8704667
KAT7	0.28941393
KATNA1	0.49103403
KATNAL1	0.7907777
KATNAL2	0.74543476
KATNB1	0.758132
KATNBL1	0.58290625
KAZN	3.131782
KBTBD11	3.1895852
KBTBD8	0.34937453
KCNA3	1.8920965
KCNC1	0.4117241
KCNC2	1.0879543
KCND3	0.6161244
KCNE2	6.3410373
KCNE3	2.7791643
KCNE4	5.2553954
KCNH3	1.7252421
KCNIP3	1.0856118
KCNJ13	3.315791
KCNJ15	4.3448586
KCNJ2	1.7893934
KCNJ5	1.923852
KCNJ8	2.2396054
KCNK1	5.2995777

KCNK12	1.0133762
KCNK13	1.2584004
KCNK3	1.4128969
KCNK4	0.19653058
KCNK6	0.51947594
KCNMB1	2.0924678
KCNMB2	1.2087343
KCNMB4	3.376683
KCNN3	1.8865314
KCNN4	0.3547125
KCNQ1	1.5337424
KCNRG	4.7660065
KCNS3	2.8877983
KCNT1	0.4415462
KCTD1	3.768394
KCTD10	2.689231
KCTD11	2.0020556
KCTD12	0.32600832
KCTD12B	1.3808718
KCTD19	0.5140557
KCTD5	1.7712169
KCTD9	0.59517336
KDELC2	0.22687364
KDELR3	1.4922009
KDM1A	0.3881936
KDM3A	1.8639517
KDM5A	1.4279475
KDM5B	0.6748071
KDM6A	0.860322
KDM6B	1.962575
KDM7A	1.2543669
KHDC3	2.2178082
KHDRBS1	0.26792812
KIDINS220	0.42245197
KIF1A	4.830691
KIF21B	0.74723244
KIF27	2.129265
KIF2A	0.24191785
KIF3A	1.0329933
KIF3B	1.105715
KIF5A	0.64220214
KIF5B	0.56639814
KIF5C	1.4444685
KIF6	1.3756092
KIF9	1.0993762

KIFAP3	0.47479582
KIRREL	1.9700837
KISS1R	1.0149136
KIT	1.4887748
KITL	5.24218
KIZ	0.37843037
KLC1	0.557899
KLC2	0.50530386
KLC3	2.0564795
KLF11	0.93518806
KLF13	0.62172556
KLF2	1.6643157
KLF3	1.1654062
KLF4	3.3048878
KLF5	5.934026
KLF6	2.9372482
KLF7	2.3609185
KLF8	0.98985386
KLF9	1.1081214
KLHDC1	1.523993
KLHDC4	0.6040125
KLHDC9	2.1934648
KLHL1	1.1078568
KLHL11	0.39012718
KLHL15	0.5645783
KLHL2	0.35172462
KLHL21	0.21339798
KLHL24	1.4250131
KLHL28	0.9631009
KLHL29	1.9228427
KLHL32	0.7876351
KLHL38	2.1583042
KLHL40	1.5123043
KLHL41	2.6040418
KLHL5	0.91203594
KLHL6	3.1334805
KLK10	0.8745291
KLK11	5.4602404
KLK13	1.7471004
KLK14	2.365542
KLK1B26	0.5556209
KLK1B27	1.4735949
KLK4	1.536977
KLK8	1.4593315
KLK9	4.13498

KLRA1	0.22176623
KLRA15	1.9682055
KLRA16	0.41233206
KLRA2	2.2863026
KLRA22	0.90702677
KLRA23	0.73200274
KLRA4	1.9037199
KLRA7	1.1680212
KLRA8	2.2663393
KLRA9	0.9162178
KLRB1A	1.4250989
KLRB1B	0.8441405
KLRB1C	0.340966
KLRB1F	1.45821
KLRC1	0.78076315
KLRG1	0.44470572
KLRG2	0.3690033
KLRI2	1.644774
KMT2A	2.6467566
KMT2D	0.35726166
KMT2E	1.0001869
KNDC1	2.0699918
KPNA4	0.5605316
KPRP	3.1387813
KRAS	0.45829868
KRI1	1.1773777
KRIT1	0.13612795
KRR1	0.20586395
KRT13	4.4069085
KRT14	3.8993258
KRT15	2.7126632
KRT16	2.108397
KRT17	3.8537023
KRT19	3.2584538
KRT23	0.22585344
KRT4	2.3619256
KRT42	1.8978481
KRT5	4.9773192
KRT6A	2.5060802
KRT6B	5.6755347
KRT7	5.3762717
KRT78	3.8809748
KRT79	4.196703
KRT80	0.8604169
KRT81	1.2748871

KRT83	0.82666326
KRT85	0.16398191
KRT86	1.6120293
KRTAP10-10	0.72674656
KRTAP11-1	0.69887304
KRTAP19-4	1.6921272
KRTAP20-2	0.36263275
KRTAP28-13	0.4998865
KRTAP31-1	0.9972453
KRTAP5-1	0.35860038
KRTAP5-3	0.16836262
KRTCAP3	1.9885592
KRTDAP	7.6025057
KSR1	1.2868888
KTN1	3.4596996
L1CAM	1.1483574
L3MBTL3	1.1142764
LAD1	1.5497296
LAIR1	0.26972723
LAMA2	0.6962223
LAMA3	2.2859654
LAMA4	2.3137937
LAMA5	2.9092116
LAMB1	1.2940497
LAMB2	1.4057393
LAMB3	1.9636173
LAMC1	1.3958383
LAMC2	7.762702
LAMC3	0.75221515
LAMP3	2.2633448
LANCL2	0.25499344
LAPTM4A	0.31534052
LAPTM5	1.7756042
LARGE	0.24211311
LARP6	3.2374728
LARP7	0.57476425
LAT2	1.8650618
LATS1	0.6891327
LATS2	2.1295757
LAX1	0.48196816
LAYN	1.2695525
LBR	1.0166416
LBX1	1.0504959
LCA5	1.4890311
LCA5L	0.98932123

LCE1A1	6.1379256
LCE1A2	1.3926897
LCE1B	4.6982155
LCE1C	5.020638
LCE1D	6.144812
LCE1E	5.2203717
LCE1F	4.95566
LCE1G	2.8042672
LCE1H	2.3901463
LCE1I	5.494845
LCE1J	0.8820529
LCE1K	4.6570024
LCE1L	2.6926699
LCE1M	1.0883846
LCE3A	8.005016
LCE3B	7.859097
LCE3C	7.2002835
LCE3D	5.062594
LCE3E	5.1897826
LCE3F	3.1668773
LCK	0.50845146
LCN2	2.0486598
LCN6	0.7481668
LCOR	1.310175
LCORL	0.38861585
LCP1	1.2648077
LCP2	0.49703765
LDB2	2.603901
LDB3	2.5548885
LDHB	1.5360174
LDLRAD1	2.36374
LDLRAD4	0.97670555
LDOC1L	0.14756584
LEF1	3.4992251
LEFTY2	2.2145455
LEKR1	1.5626543
LEMD3	0.5208745
LENG8	0.42968702
LEPREL4	0.9312153
LGALS1	0.5859351
LGALS3	1.304491
LGALS7	2.4259772
LGI2	2.8387938
LGI3	5.3719482
LGI4	0.6442895

LHB	1.553438
LHFP	1.5751996
LHX1OS	0.8491111
LHX4	0.51687336
LIF	1.1804101
LILRA6	1.5478063
LILRB4	2.3060231
LIMA1	0.53284025
LIMCH1	5.739079
LIME1	0.409297
LIMK1	0.35792542
LIMK2	0.4546504
LIMS1	0.98956347
LIN52	0.312891
LIN7C	0.3890872
LINGO4	1.9973431
LIPF	2.659809
LIPG	4.171123
LIPH	5.217876
LIPO1	1.2866664
LITAF	0.58360624
LIX1L	2.4901543
LMAN1L	1.1149554
LMCD1	2.2080846
LMLN	2.1022282
LMNB1	1.3526177
LMO1	3.0912786
LMO4	1.1642079
LMO7	3.117228
LMTK3	0.8547404
LNPEP	1.6058946
LNX1	0.5250881
LOC1000417	1.1862497
LOC1010558	0.7470751
LOC1010562	1.6525898
LOC1010562	1.8761578
LOC1010562	1.0892739
LOC1026326	0.52225304
LOC1026332	0.8290348
LOC1026333	0.98481154
LOC1026336	2.567357
LOC1026336	0.5269871
LOC1026337	3.1584995
LOC1026338	0.4440987
LOC1026344	0.621222

LOC1026380	2.8528073
LOC1026385	0.32975483
LOC1026391	1.7358747
LOC1026395	2.283958
LOC1026416	1.253881
LOC1026416	0.8459964
LOC1026416	0.61408734
LOC1026423	2.0049994
LOC1026423	1.5481663
LOC1026424	0.40545845
LOC1026424	1.629791
LOC1026425	0.9672663
LOC1026426	0.63806105
LOC1026427	0.9537051
LOC1026427	0.21173
LOC1026428	0.7960968
LOC1026430	0.6652131
LONRF3	0.471519
LOR	6.0487165
LOX	7.715526
LOXL1	3.469226
LOXL4	0.389791
LPAR1	3.6387627
LPAR3	3.6523204
LPCAT1	2.8417935
LPCAT2	0.47139072
LPCAT2B	0.76794624
LPXN	1.4633594
LRCH1	1.6880355
LRFN4	4.012564
LRGUK	2.7628992
LRIF1	0.29576063
LRP12	0.4620142
LRP2	4.349502
LRRC1	1.647224
LRRC10	3.5600536
LRRC10B	3.5660014
LRRC16A	2.219091
LRRC2	2.218173
LRRC23	5.394993
LRRC25	1.6213474
LRRC26	6.869556
LRRC27	0.3213365
LRRC32	1.8562598
LRRC34	2.86948

LRRC36	5.477016
LRRC38	1.1146545
LRRC4	0.26604223
LRRC43	3.0868387
LRRC49	2.822702
LRRC4B	2.7860303
LRRC51	0.76175976
LRRC71	0.57698345
LRRC73	0.7998946
LRRC75A	1.015317
LRRC75B	1.360975
LRRC8A	1.1509261
LRRC8B	3.9497533
LRRC8C	1.9765544
LRRC8E	1.710372
LRRFIP1	0.91953945
LRRFIP2	0.24200153
LRRIQ1	0.87649703
LRRK1	0.5319071
LRRK2	4.066656
LRRN4CL	3.1558988
LRTM2	4.246707
LSM12	0.2663517
LSM8	1.0044804
LSP1	1.7362571
LTB	0.5080843
LTB4R1	2.91679
LTBP1	2.4482026
LTBP2	2.119833
LTBP3	1.9996781
LTBP4	1.3876338
LTC4S	0.8623791
LTF	4.723978
LTV1	0.4611001
LUC7L3	0.9124274
LURAP1	2.1125755
LUZP1	0.6006398
LXN	1.2105169
LY6A	2.1110244
LY6C1	2.0834537
LY6F	2.231257
LY6G5B	1.7859266
LY6G6C	3.4810848
LY6G6D	0.93728375
LY6G6E	0.30941677

LY6H	1.2815042
LY6I	1.67414
LY6K	1.8761792
LY75	1.4495606
LYN	0.65457535
LYNX1	1.1728473
LYPD2	5.0305624
LYPD3	5.1299343
LYPD5	2.9711266
LYPD6B	4.8910227
LYSMD2	1.7974591
LYST	0.6372273
LYVE1	5.5131235
LYZ1	1.8930225
LYZ2	2.2324562
LZTS1	1.3699691
LZTS2	0.69394493
LZTS3	0.52826786
M1AP	2.7316005
MAATS1	2.0468206
MAB21L3	3.1795483
MACC1	2.697443
MACF1	1.258565
MADCAM1	2.069503
MAFF	4.047655
MAFK	1.7100258
MAG	0.8002918
MAGEA7-PS	0.99852514
MAGEF1	0.79290485
MAGI3	1.1380157
MAGOH	0.3086896
MAGOHB	1.866746
MAK	1.0540245
MAK16	0.59055996
MAL	2.0482235
MAL2	0.5479245
MALAT1	2.8903694
MALL	4.5910745
MALT1	2.6805763
MAML1	0.53778696
MAML3	1.977354
MAMSTR	2.8507452
MAN1C1	0.57624865
MAP10	1.4711859
MAP1A	1.2171082

MAP1B	3.223871
MAP1S	0.24115467
MAP2	0.9356251
MAP2K1	0.50737953
MAP3K1	0.66468716
MAP3K14	0.8995886
MAP3K15	0.74430466
MAP3K3	0.88606405
MAP3K6	5.0145845
MAP3K7	0.28575993
MAP3K8	2.512198
MAP3K9	2.6236064
MAP4	1.5576868
MAP4K1	1.508429
MAP4K4	1.939044
MAP6	3.6005697
MAPK10	2.7744236
MAPK11	0.7786231
MAPK12	0.49197865
MAPK13	4.022091
MAPK6	0.79683256
MAPK7	1.1563973
MAPKAPK3	2.081365
MAPKBP1	1.5773585
MAPRE1	0.896503
MAPRE2	0.655777
MAPT	2.2219925
MARCKS	1.8419809
MARCKSL1	1.4132767
MARF1	0.35340476
MARK1	2.173461
MARK2	0.7936077
MARK4	0.44892788
MARVELD3	2.1282802
MAST2	0.37914371
MAST4	1.9325833
MAT2A	0.43461275
MATN4	2.4468827
MB	8.392814
MB21D1	0.82229185
MBD1	1.3425202
MBD2	1.2131481
MBD4	0.355592
MBD5	1.0132806
MBIP	1.1547966

MBNL2	1.1285458
MBNL3	2.105342
MBOAT1	3.425228
MBOAT2	0.7775507
MBP	3.5127378
MC5R	1.3316598
MCEMP1	4.075736
MCF2L	2.959248
MCL1	0.93355083
MCM3	0.50289917
MCM6	0.5586519
MCMBP	0.26369238
MCOLN2	3.326748
MCOLN3	0.74075675
MCPT4	2.5720048
MCRS1	0.55657816
MCTP1	2.7508154
MCTP2	0.78467846
MDFI	2.451675
MDFIC	0.84595394
MDGA1	0.6247697
MDH1B	4.307129
MDM2	0.17502022
MDM4	1.9399025
MDN1	0.86545014
ME2	0.43153286
ME3	4.20266
MECP2	1.1378226
MED12	0.20059013
MED13	0.76770425
MED13L	2.7667847
MED24	0.45391512
MED26	0.2841072
MED27	0.2341857
MED31	0.7610202
MEF2A	0.8802881
MEF2B	0.42892003
MEF2C	1.0044878
MEF2D	0.5123725
MEFV	2.315154
MEGF10	0.64354444
MEGF6	1.5995858
MEGF8	0.25767994
MEIG1	5.5326366
MEOX1	3.2815523

MEOX2	4.018817
MESDC1	1.0248334
MESP1	1.7310023
METRNL	0.6723809
METTL14	0.6477659
METTL21C	2.1129172
METTL24	0.55560255
METTL25	0.16234303
METTL6	0.39806366
MEX3B	0.48949695
MEX3C	0.65162516
MEX3D	0.7195158
MFAP1A	0.96546316
MFAP1B	1.3186913
MFAP2	2.183249
MFAP3L	1.0923321
MFAP4	3.395668
MFAP5	5.4547195
MFGE8	0.8590684
MFNG	1.7624493
MFSD4	2.7105043
MFSD6	1.1851797
MFSD6L	3.836154
MFSD7A	1.1067321
MFSD7B	1.3929448
MGA	0.6605096
MGARP	2.7050164
MGAT3	3.6078897
MGAT4A	2.557509
MGAT5	0.53680134
MGEA5	1.7421827
MGP	3.4573336
MIA	1.1653643
MICAL1	2.3959613
MICAL2	0.6652386
MICALL1	0.5972986
MICU3	1.5924842
MID2	0.8768766
MIDN	0.7310438
MIER1	0.8968735
MILL2	1.752568
MIPOL1	0.93408537
MIR17HG	0.6760998
MIR22HG	1.3901424
MISP	1.774971

MITD1	0.41073465
MITF	0.6196871
MKI67	0.2657094
MKL1	1.1139493
MKRN1	0.22687292
MLANA	3.9443
MLC1	7.9406877
MLF1	3.7052374
MLIP	1.1904261
MLKL	0.69699097
MLLT10	0.9498415
MLLT11	1.0157351
MLLT4	3.8104253
MLLT6	1.148459
MLPH	2.823029
MLXIP	1.5140834
MMP11	0.69358206
MMP12	2.3194137
MMP13	3.261489
MMP14	0.4488573
MMP17	0.6082382
MMP2	3.345994
MMP23	0.2268095
MMP25	5.010382
MMP3	7.348541
MMP7	0.36163688
MMP8	5.436223
MMP9	4.664946
MMRN1	2.8394852
MNDA	1.9916654
MNS1	3.9105759
MNT	1.0036469
MOAP1	0.15405321
MOB1A	0.5384126
MOB1B	1.6145678
MOB3A	1.7767391
MOB4	0.8814697
MOK	2.915523
MON1B	0.67866945
MON2	0.7563429
MORC1	3.57994
MORC3	0.5206804
MORN3	1.236212
MORN4	1.9846401
MORN5	4.558945

MOSPD1	0.6519122
MOXD1	1.8658006
MPHOSPH10	0.76393247
MPHOSPH6	1.3367596
MPP3	0.53027105
MPP5	1.6061287
MPP7	1.6917701
MPRIP	0.59978724
MPZL1	1.042315
MRAP2	3.6147447
MRE11A	0.50280905
MRGPRA2A	4.8319683
MRGPRA2B	4.8577766
MRGPRA6	4.1009912
MROH1	1.0414948
MRPL20	0.9469242
MRPL3	0.29572153
MRPS6	0.7615714
MRVI1	0.60224557
MS4A1	2.2895923
MS4A14	0.9583247
MS4A15	0.5142708
MS4A3	1.1312108
MS4A4A	5.3973947
MS4A4B	1.386363
MS4A4D	2.7662034
MS4A6B	3.241646
MS4A6C	2.210641
MS4A6D	2.5914493
MS4A7	1.9628353
MS4A8A	1.330895
MSANTD1	1.0492775
MSANTD3	1.8364768
MSI2	0.8796859
MSL2	1.2758007
MSL3L2	0.2897215
MSLN	2.390955
MSN	2.0879922
MSR1	0.20555592
MSRB3	0.7885313
MST1R	2.2744646
MSX1	0.71328735
MSX2	0.6651659
MT4	2.5522425
MTA2	1.0888476

MTAP7D3	2.4509861
MTBP	0.91923904
MTCL1	5.222236
MTDH	0.7806449
MTF2	0.3372693
MTHFD1L	1.425056
MTHFD2	3.6204991
MTHFD2L	0.4490435
MTIF2	1.2005191
MTMR12	0.8522682
MTMR3	0.8599882
MTRR	0.73780584
MTURN	1.3066812
MTUS2	1.1027365
MUC1	5.9573727
MUC13	4.7969627
MUC20	4.6204195
MUC4	6.5283513
MUC5AC	1.3080428
MUC5B	9.865908
MUM1L1	2.5170221
MURC	1.779084
MUSTN1	1.6015491
MVB12B	1.6254301
MVP	0.8980131
MX1	1.8466363
MX2	0.5556092
MXD1	3.7566347
MXRA7	0.9523928
MXRA8	0.34806252
MYADM	1.0283365
MYADML2	0.5627792
MYB	2.397955
MYBL2	1.262867
MYBPC1	4.907195
MYBPC2	5.736462
MYBPC3	4.00275
MYBPH	1.0880415
MYBPHL	4.575226
MYC	2.0321965
MYCBP2	0.42151737
MYCBPAP	2.750482
MYD88	1.457026
MYEF2	1.5521922
MYF6	4.5680943

MYH1	8.433739
MYH11	2.810505
MYH14	2.8378043
MYH2	2.2012827
MYH3	1.5653617
MYH4	2.2491972
MYH6	7.1083665
MYH7	5.377422
MYH7B	1.5241926
MYH8	7.4964685
MYH9	1.0785208
MYL1	6.559048
MYL12A	0.6509919
MYL3	7.522196
MYL4	5.514036
MYL7	6.3049207
MYL9	1.6760693
MYLK2	4.245859
MYLK3	0.9593458
MYO10	1.3146958
MYO18B	0.8810997
MYO1C	1.4463859
MYO1D	3.0098667
MYO1F	0.6958499
MYO1G	1.5464005
MYO1H	1.9839232
MYO5A	2.8783255
MYO5C	3.5899315
MYO9A	0.30587745
MYOCD	1.8382196
MYOF	0.4126897
MYOM1	4.7461634
MYOM2	5.5947857
MYOM3	3.6329656
MYOT	3.3685703
MYOZ1	4.2565866
MYOZ2	6.009225
MYRF	0.9926641
MYRIP	4.8143373
MYSM1	0.3536477
MYZAP	1.8335238
MZB1	1.5074649
MZT1	0.7648668
N28178	1.9925418
N4BP1	1.8636923

N4BP2	0.5154009
N4BP2L2	1.0263829
N4BP3	1.7992792
NAA15	0.2572646
NAA25	0.6573782
NAAA	1.2261372
NAB1	0.30880308
NAF1	0.7544036
NAIP1	0.24072242
NAIP6	0.5756221
NAMPT	0.5924158
NAP1L1	0.6810703
NAPEPLD	0.6816225
NAPSA	4.2252593
NAT14	0.70611835
NAT8L	0.9677305
NAV1	1.1048908
NAV3	0.46875477
NBEA	2.9512339
NBEAL1	0.81401634
NBL1	4.5206165
NBN	0.9919417
NCAPG2	0.23446083
NCAPH	1.0119658
NCCRP1	2.702973
NCDN	0.2933569
NCF1	1.8025889
NCF2	1.8268604
NCF4	1.9593868
NCK2	0.7011013
NCKAP1L	0.693398
NCKAP5	4.3995414
NCL	1.1535993
NCOA6	0.4953704
NCOA7	3.067037
NCOR2	0.32226706
NCR1	0.389179
NCS1	1.6011109
NCSTN	0.49711752
ND6	1.5145569
NDE1	0.28775263
NDEL1	0.67400694
NDRG1	1.9691801
NDRG4	3.5886216
NDUFA4L2	0.35314178

NEAT1	0.9640374
NEB	3.0410833
NEBL	3.3031108
NEDD9	2.738853
NEIL1	0.35726452
NEK11	2.1589866
NEK5	4.4541306
NEK7	0.913723
NEK8	0.21934032
NEMF	0.4168191
NEO1	0.51161957
NES	3.0166912
NET1	0.29827738
NEURL1A	3.0006561
NEURL3	1.9878278
NEUROG1	0.81342006
NEXN	2.2639456
NFASC	2.6087308
NFAT5	1.9839082
NFATC1	1.2170839
NFATC2IP	0.5884495
NFATC4	3.4337916
NFE2	1.2437153
NFE2L1	0.7544856
NFE2L2	0.26925325
NFE2L3	1.1187165
NFIL3	0.84005547
NFKB1	1.6841941
NFKB2	1.5333934
NFKBIA	2.9988866
NFKBID	2.443705
NFKBIE	1.3137097
NFKBIZ	2.119977
NFRKB	0.478446
NFYA	0.4485879
NGDN	0.14067984
NGFRAP1	2.0958095
NGP	3.5478158
NHLH1	1.5580807
NHLRC4	2.6288724
NHSL2	1.9930925
NID1	0.7398224
NIN	1.3709493
NINJ2	0.71096253
NIPA1	0.29296923

NIPAL2	3.4826891
NIPAL3	0.57095957
NIPBL	1.0790925
NKAIN1	0.28472757
NKD1	2.415708
NKG7	0.16600847
NKTR	1.1526694
NKX2-1	6.8048463
NKX2-5	1.6737835
NKX6-1	0.9481337
NLGN2	1.8338976
NLGN3	0.5977442
NLK	0.14549065
NLRC3	0.5572815
NLRC4	1.1881046
NLRC5	0.7753253
NLRP10	0.6517341
NLRP3	3.6826568
NMB	0.92876005
NME5	4.3436875
NME9	0.36432457
NMT2	0.58660316
NMUR2	3.984168
NNAT	2.4915032
NOC3L	0.38489628
NOD1	1.8676729
NOD2	3.350358
NOG	1.736268
NOL10	0.76855946
NOL3	0.4756546
NOLC1	0.52898836
NOM1	0.44324398
NOP14	1.1315837
NOP56	0.53846025
NOP58	0.5773277
NOS2	3.9243927
NOS3	1.4072819
NOTCH1	1.1010866
NOTCH2	0.68213844
NOTCH3	1.1988492
NOTCH4	1.8910198
NOV	3.1269524
NOVA2	0.5955405
NOX4	4.8420086
NOXA1	0.582746

NOXO1	1.7056046
NPAS2	0.35172915
NPC2	1.4872303
NPCD	0.24578881
NPDC1	1.6671634
NPEPPS	0.97282934
NPHP1	1.5997894
NPHP4	1.9735138
NPHS2	0.78079057
NPM1	0.7611971
NPM2	1.3087807
NPNT	4.429858
NPPA	8.985999
NPPB	1.9629972
NPR2	0.56367874
NPR3	3.0338364
NPW	5.6632905
NPY	3.1355886
NPY1R	1.0822527
NR2C2	1.3276734
NR4A1	4.7932887
NR4A2	3.3974133
NR4A3	1.0576825
NRADD	0.23477507
NRARP	2.5119684
NRAS	1.1408324
NRCAM	5.0544844
NRD1	1.3782096
NRF1	0.33074427
NRG1	0.4078722
NRG3OS	0.5071044
NRG4	0.38327026
NRGN	0.716485
NRIP1	1.566999
NRIP3	3.052351
NRP2	0.8380923
NRSN1	0.7309792
NSD1	0.3479185
NSF	1.6775985
NSG1	3.2825556
NSG2	1.8491874
NSMCE2	0.30964327
NT5C3B	0.38978577
NTF5	0.36728215
NTN1	0.95731306

NTN4	0.2644043
NTN5	1.0702808
NTNG2	4.066696
NTRK2	2.9319053
NTS	1.3371501
NTSR2	1.4025414
NUAK1	0.5500963
NUAK2	1.1301794
NUB1	0.26378155
NUCB2	2.716682
NUDT10	1.0528696
NUDT11	1.3440392
NUDT17	0.6839018
NUFIP2	0.41561556
NUMA1	0.46100903
NUMBL	2.2706075
NUP107	0.47766733
NUP153	0.7967262
NUP160	0.41238594
NUP188	0.68890667
NUP205	0.47820616
NUP62	0.9013066
NUP93	0.8001466
NUP98	1.9222841
NUPR1	5.1910534
NUPR1L	0.25307393
NUS1	0.56691265
NVL	0.3981502
NWD2	2.2605114
NXF1	0.43110895
NXF2	1.5117254
NXNL2	1.5975859
NXPE3	1.3702064
NXPH3	2.2275667
NYX	2.5669312
OAS2	0.6683512
OAS3	1.407125
OASL2	2.0733194
OAZ2	1.1053252
OBFC1	1.1990733
OBSCN	0.8405483
OBSL1	3.70688
OCM	0.75189877
ODC1	1.524611
ODF2	1.254559

ODF2L	0.25798273
OFD1	0.9165993
OGN	2.7012239
OIT1	0.9580064
OLFM1	1.7187252
OLFM4	2.7395635
OLFML2B	2.8101735
OLFR103	0.22166824
OLFR1043	0.7456479
OLFR1131	0.84493876
OLFR1134	0.7207875
OLFR1180	1.4095402
OLFR1243	0.3100903
OLFR1252	0.9748235
OLFR1368	0.88958406
OLFR1372-PS	1.329473
OLFR1384	1.3091371
OLFR1396	1.618931
OLFR1402	0.74279046
OLFR1443	0.40668654
OLFR15	0.8486736
OLFR1505	1.1032739
OLFR213	1.2066576
OLFR224	1.1694331
OLFR259	0.6990063
OLFR391-PS	0.41342282
OLFR414	1.2614694
OLFR470	1.253439
OLFR482	1.457634
OLFR513	1.2576177
OLFR524	0.47057414
OLFR544	1.1209202
OLFR56	1.3995197
OLFR609	0.23475742
OLFR635	1.5473797
OLFR700	1.7433147
OLFR738	0.5931697
OLR1	3.697684
OOSP1	2.4905455
OPHN1	0.38816476
OPN3	0.9730737
ORAI2	1.9145851
ORC4	1.7638547
OSBPL10	2.2887912
OSBPL3	1.8433614

OSBPL5	2.1144552
OSBPL6	1.5824692
OSBPL7	0.5669732
OSBPL9	2.1607904
OSCP1	3.4273996
OSGIN2	1.7413902
OSM	4.328584
OSMR	3.8426318
OSR1	5.165198
OSTF1	0.9088669
OTOP2	1.8914244
OTOP3	1.9337432
OTUD1	0.8211293
OTUD4	0.33885002
OTUD5	0.447093
OTUD7B	0.8439958
OVGP1	2.0846741
OXCT1	1.3025327
OXSR1	0.27486563
P2RX5	3.7208357
P2RY10	3.188993
P2RY14	0.48071718
P2RY2	2.7182636
P2RY6	0.49605274
P4HA1	0.38252258
P4HA2	2.6429892
P4HTM	0.9437585
PA2G4	0.4787016
PABPC1	0.5078416
PABPC1L	2.708666
PABPN1	0.43200493
PACRG	3.1503847
PACS1	1.5932574
PACS2	0.43639755
PADI1	0.35820985
PADI4	0.6486697
PAFAH1B1	0.2434268
PAG1	2.6573775
PAK2	0.61969376
PAK6	1.2712414
PAK7	0.15738535
PAM	2.215785
PAN3	0.90826845
PANCT2	0.7163875
PANK2	0.29380274

PANK3	0.56144404
PANX1	0.1969347
PAPL	0.31184483
PAPOLG	0.39925694
PAPPA	5.3638053
PAPPA2	3.4078777
PAQR5	1.3552096
PAQR6	1.1372106
PAQR8	2.614602
PARD6B	4.0465126
PARD6G	2.5708346
PARL	0.22859621
PARM1	5.7541633
PARP10	0.4720168
PARP14	0.7633681
PARP2	0.16701937
PARP4	1.7532535
PARP8	2.4193048
PARVB	1.53285
PATL1	0.19205332
PATZ1	0.8451185
PAWR	1.5088892
PAX5	0.4356246
PAX9	1.0966918
PAXBP1	0.20551443
PAXIP1	0.9640782
PBRM1	0.6231103
PBX1	0.9793763
PBX3	1.5433116
PBXIP1	1.6504273
PCBP3	2.3971958
PCDH17	2.6685858
PCDH18	0.79152775
PCDH19	4.4143176
PCDHB10	2.0729592
PCDHB14	2.0323594
PCDHB16	2.27096
PCDHB17	1.5874476
PCDHB18	2.1092358
PCDHB20	2.6968315
PCDHB21	2.9470568
PCDHB22	1.9387419
PCDHB3	1.0962875
PCDHB6	2.11526
PCDHB7	2.3130841

PCDHGA1	2.1416602
PCDHGA10	2.9060748
PCDHGA9	2.049271
PCF11	1.5443804
PCGF3	1.1997933
PCGF5	1.0695682
PCIF1	1.000195
PCK2	0.5455899
PCM1	0.8563609
PCNA	0.2960806
PCNT	1.8279285
PCNXL2	1.4012189
PCNXL4	0.5197499
PCOLCE2	0.34004736
PCP4	0.7101679
PCSK2	0.9477298
PCSK5	2.0422635
PCYT1A	0.21110821
PDCD1	2.3510494
PDCD11	0.26200747
PDCD1LG2	1.3529508
PDCD6IP	0.34478998
PDCL3	1.6499853
PDE1B	1.2331839
PDE3A	2.3892004
PDE4B	4.211302
PDE4D	1.9015667
PDE5A	0.6240711
PDE7A	1.7865047
PDE7B	2.4192898
PDE8B	1.3863993
PDGFA	1.9476962
PDGFB	1.9077516
PDGFRA	3.98453
PDGFRB	0.36302376
PDGFRL	2.0067163
PDIA2	1.2683835
PDK3	2.0508876
PDK4	2.3167973
PDLIM2	2.7759843
PDLIM3	2.4378052
PDLIM5	1.0497074
PDLIM7	2.3101182
PDP1	0.81862307
PDPN	5.412135

PDPR	0.18282104
PDS5A	0.25260115
PDZD2	2.3898304
PDZD4	2.3614104
PDZD8	0.74191666
PEAK1	0.17712355
PEAR1	0.63883543
PECAM1	1.8081269
PEG13	1.4658561
PEG3	1.3237884
PEG3OS	1.0413113
PELI1	0.51265
PENK	1.7936578
PER1	1.4225421
PERM1	0.7218168
PES1	0.21989632
PF4	2.13722
PFDN2	0.17846394
PFKFB2	0.6598878
PFKFB3	4.219854
PFKFB4	2.1368241
PFKP	1.2310667
PGAM2	4.12155
PGAP3	0.25191355
PGBD5	0.81068444
PGF	1.2085481
PGGT1B	0.9262366
PGLYRP1	7.14597
PGM1	1.0457726
PGS1	2.1354008
PHACTR1	3.154193
PHACTR2	1.0972309
PHC1	1.2933276
PHC2	0.9231868
PHC3	0.51558495
PHF11A	1.3521929
PHF11B	1.331728
PHF11D	1.4113479
PHF13	0.65051174
PHF2	0.21631622
PHF20	0.75385094
PHF20L1	0.8937416
PHF21B	0.5044668
PHF23	0.31096506
PHF8	0.9601536

PHGDH	1.6988153
PHIP	0.73178196
PHKA1	1.2976608
PHKG1	0.87150526
PHLDA1	0.45865345
PHLDB1	0.8531213
PHLPP2	0.48183107
PHOSPHO1	0.46796322
PHOX2A	1.6801538
PHTF1	0.27690315
PHXR4	2.0849564
PI15	4.1737127
PI16	7.5875044
PI4KA	0.22324848
PIANP	0.16814089
PIAS1	0.24144077
PIAS3	0.37434602
PIBF1	0.5584738
PICALM	0.9397526
PIEZO1	1.5818315
PIFO	1.9607365
PIGM	0.7745075
PIH1D2	1.7493777
PIK3AP1	1.1570759
PIK3C2A	1.1445584
PIK3CD	0.48172235
PIK3CG	2.110302
PIK3IP1	3.0046477
PIK3R3	1.2640414
PIK3R5	2.1143775
PIK3R6	1.5229454
PILRA	1.2103457
PILRB2	0.31333637
PIM1	2.7136235
PIM2	0.2732389
PIM3	1.0634937
PIP4K2A	1.3427906
PIP4K2C	0.33566284
PIP5K1A	1.6230102
PIP5K1C	0.75268984
PIRA11	0.92350626
PIRA2	1.1941514
PIRA6	0.8562932
PIRA7	0.8951416
PITPNA	0.4303832

PITPNM3	3.051601
PITX1	4.4256206
PIWIL4	2.6839173
PJA2	0.66337204
PKD1L3	1.3412728
PKD2	1.8772464
PKIA	0.6222365
PKIB	0.30065417
PKM	2.3642416
PKN2	0.5005865
PKNOX2	3.2869422
PKP1	2.2277117
PLA2G1B	3.367246
PLA2G2C	0.9338832
PLA2G2E	0.7519386
PLA2G4A	0.64863205
PLA2G4D	2.237095
PLA2G4F	1.554033
PLA2G7	1.4877057
PLAC8	1.4399171
PLAC9A	0.80231524
PLAGL1	1.490181
PLAGL2	1.3266816
PLAT	6.8966217
PLAU	2.147454
PLAUR	5.3702707
PLB1	0.60851645
PLCB2	0.69713664
PLCB3	0.46345425
PLCB4	2.9854612
PLCD1	2.0267096
PLCD3	1.6022549
PLCG2	1.0147638
PLCH1	3.18655
PLCH2	1.8501904
PLCL1	1.6669934
PLCXD3	0.8761549
PLD2	0.39085293
PLD3	0.4358673
PLEK	2.644607
PLEKHA1	2.335608
PLEKHA2	0.853199
PLEKHA4	2.2543535
PLEKHB1	0.8027296
PLEKHB2	1.1509895

PLEKHD1	2.9160457
PLEKHF2	1.0495358
PLEKHG2	0.6566415
PLEKHG4	2.939907
PLEKHH1	2.498273
PLEKHM1	0.38266993
PLEKHM2	0.87693787
PLEKHM3	0.8673868
PLEKHO1	0.40539694
PLEKHO2	1.144867
PLEKHS1	3.7853932
PLET1	4.7859936
PLGRKT	0.40766096
PLIN1	2.4351087
PLIN4	3.120161
PLK2	0.9400983
PLN	2.6414845
PLOD2	3.3783255
PLOD3	0.20653296
PLP2	4.2357416
PLSCR1	1.3293071
PLSCR3	0.31855106
PLVAP	0.81197023
PLXDC2	2.9234028
PLXNA2	0.92957544
PLXNA4	1.7692485
PMAIP1	3.1769748
PMEPA1	3.2353158
PML	0.59098625
PMP22	3.1704469
PNCK	0.8117893
PNLIPRP1	0.7295785
PNN	0.44650745
PNPLA2	0.43745804
POC1A	0.59101534
POC1B	0.7461648
POC5	1.275167
PODXL	2.4243984
POF1B	2.0265331
POGK	2.4387476
POLD3	0.46538877
POLE2	0.46072507
POLH	1.9079366
POLK	0.5189979
POLM	0.46010303

POLR3D	0.1348238
POLR3E	1.2952266
POLR3GL	0.28789282
PON2	0.48397827
POP4	0.7650566
POPDC2	3.1125836
POPDC3	3.8019493
PORCN	2.827474
POSTN	0.9829836
POT1B	0.8601978
POU2AF1	2.935678
POU2F2	0.83620644
POU5F2	0.38905907
PPAP2A	0.575212
PPARD	0.43107128
PPBP	0.46439886
PPFIA1	0.23352432
PPFIBP1	0.27105093
PPIC	2.1515465
PPIG	0.54348373
PPIL4	0.1943965
PPIL6	2.4190621
PPIP5K1	1.6357026
PPL	2.0541267
PPM1D	0.7462797
PPM1F	0.35048962
PPM1H	0.87709284
PPM1J	2.670391
PPM1M	0.59776974
PPP1R12A	1.4888911
PPP1R12B	0.51875496
PPP1R13B	0.4572444
PPP1R14A	0.5757823
PPP1R14D	4.967951
PPP1R15A	2.035325
PPP1R16B	0.99667
PPP1R18	1.7404289
PPP1R2	1.5749836
PPP1R26	2.7398276
PPP1R27	1.1502602
PPP1R32	4.0082164
PPP1R3A	3.3167048
PPP1R3D	1.9001479
PPP1R8	0.55405045
PPP1R9A	1.1548538

PPP1R9B	1.3885942
PPP2CB	0.6208973
PPP2R2A	0.81175995
PPP2R5B	0.4674859
PPP3CC	0.86762524
PPP4R1	0.7082534
PPP4R2	0.94005203
PPP6R3	0.6431422
PPRC1	0.34568024
PPT1	0.6375499
PQLC3	2.2511783
PRAF2	0.48622465
PRAM1	2.147643
PRAMEF8	0.3645649
PRCC	0.17614794
PRDM1	2.5201826
PRDM16	3.4571803
PRDM2	0.96731496
PRDM4	0.7302046
PRDM5	2.9911203
PRDM6	2.9233804
PRDM8	1.844619
PREX1	0.2893648
PREX2	0.98978734
PRF1	1.3226485
PRICKLE2	0.90919924
PRKAB2	0.2856145
PRKAR1B	3.6815352
PRKAR2B	2.6249948
PRKCA	0.4711795
PRKCB	0.74161196
PRKCD	1.2187867
PRKCDBP	2.6782722
PRKCE	1.3662512
PRKCH	2.586831
PRKCI	0.29452658
PRKCQ	0.7634604
PRKD1	3.4183323
PRKD2	0.7731581
PRKG1	4.032466
PRKG2	1.7034342
PRKX	1.2815495
PRL2C1	3.290299
PRM2	1.673645
PRMT1	0.5083232

PRMT2	2.6584787
PRMT8	2.4008772
PROCR	1.8394513
PROK2	6.0423384
PROM1	0.1808939
PRPF3	0.46070862
PRPF31	0.7827983
PRPF38B	0.9727044
PRPF39	0.42165422
PRPF4	0.20053577
PRPF4B	0.4981141
PRR13	0.7519512
PRR15	1.2866926
PRR15L	5.547386
PRR18	4.82226
PRR24	0.7113652
PRR5L	0.70859766
PRR7	3.1019082
PRR9	1.3026974
PRRC2C	1.8835068
PRRG3	5.596603
PRRG4	0.7548382
PRRT1	0.6129658
PRRX1	1.4428053
PRRX2	2.910242
PRSS12	4.472967
PRSS16	2.637184
PRSS22	2.87144
PRSS23	3.7365642
PRSS23OS	4.472372
PRSS30	1.3577383
PRSS53	2.2212105
PRSS8	1.0318723
PRX	8.232361
PSAPL1	3.4114962
PSAT1	2.080205
PSCA	1.5324271
PSD	0.81374407
PSD4	0.6657481
PSG16	0.43904114
PSMC3IP	0.343163
PSMD8	0.40947008
PSME4	0.22642422
PSORS1C2	3.8909814
PSPC1	0.33204937

PSTPIP1	1.361382
PTAFR	2.7049103
PTAR1	1.3316183
PTBP3	0.88062716
PTCHD3	0.60904074
PTGER1	0.18411088
PTGER2	0.42513514
PTGER4	1.9354014
PTGES3L	0.22464085
PTGFRN	2.8454356
PTGIR	2.8060808
PTGIS	2.6751819
PTGS2	6.7818155
PTHLH	2.52777
PTK7	0.9741154
PTPN1	0.8368406
PTPN11	0.5375447
PTPN12	1.3814707
PTPN13	3.4313374
PTPN14	4.1067767
PTPN2	1.6378021
PTPN21	0.72780085
PTPN22	2.3020082
PTPN23	1.2740154
PTPN7	1.222784
PTPRA	0.2381649
PTPRB	0.52241325
PTPRC	1.2305136
PTPRE	1.988481
PTPRG	0.94168353
PTPRN2	0.5956223
PTPRU	2.8985496
PTPRZ1	4.0094485
PTRF	1.498168
PTRH1	0.9304147
PTX3	6.595067
PUM1	0.3874936
PUM2	0.44349337
PURB	0.82912874
PUS3	0.16719341
PUS7	0.2920766
PUS7L	0.23037219
PVALB	0.92695236
PVR	3.8597398
PVRL4	1.8843536

PWWP2A	0.23162103
PWWP2B	0.815238
PXDN	0.33439922
PXN	1.6619797
PYCARD	1.8572445
PYCR2	0.636919
PYDC4	1.4750533
PYGM	3.3119578
PYGO2	0.32314205
PYHIN1	2.0391068
PYY	1.2869751
QK	1.7309871
RAB10OS	0.9606085
RAB11FIP1	1.7486267
RAB11FIP5	2.2257261
RAB12	0.3641305
RAB15	7.5923624
RAB19	0.5856228
RAB20	0.9692826
RAB25	5.453719
RAB27A	4.2224336
RAB27B	2.1878881
RAB28	0.16909456
RAB2B	0.706059
RAB31	1.3707786
RAB33A	1.1032817
RAB33B	0.31411648
RAB34	0.4190731
RAB35	0.52359915
RAB37	0.46046543
RAB38	1.0343976
RAB3C	0.97966146
RAB44	4.144387
RAB6A	0.2621627
RAB6B	3.3182032
RAB8B	1.4616966
RAB9B	0.36216307
RABGAP1	0.54146767
RABGAP1L	1.0129042
RABGEF1	0.62326765
RABL2	0.589509
RAC2	1.1675053
RAC3	1.7309184
RACGAP1	0.26365948
RAD18	1.3765459

RADIL	2.638349
RAE1	0.28651285
RAI1	0.5752511
RAI14	1.0397911
RALB	1.3132071
RALBP1	0.7604823
RALGAPA1	1.6914086
RALGAPA2	0.31683636
RALGAPB	0.98612356
RALGDS	2.7891827
RAMP2	0.9354243
RAMP3	6.099728
RANBP2	0.33810902
RANBP9	0.62045383
RAP1A	0.5900388
RAP1B	1.1863432
RAP1GDS1	0.3567338
RAP2A	0.5507288
RAP2B	0.63792396
RAPGEF3	2.1039853
RAPGEF5	3.8785257
RAPGEF6	1.4166679
RAPGEFL1	0.86034393
RARA	0.16391945
RARS	0.75638914
RASA2	2.1330843
RASA3	1.2544241
RASAL3	0.69985104
RASD1	4.785783
RASD2	1.9399908
RASEF	4.161778
RASGEF1A	7.4718394
RASGEF1C	1.7449677
RASGRP3	0.5162301
RASGRP4	0.83943987
RASIP1	4.5453153
RASL10B	0.7189436
RASL11A	1.9676499
RASL11B	1.4331174
RASL12	5.592711
RASSF1	2.411499
RASSF2	2.7209725
RASSF5	0.6223402
RASSF9	4.6792836
RB1	1.279419

RB1CC1	0.3876748
RBAK	0.9772301
RBBP4	0.23604918
RBBP8NL	1.7431285
RBFOX1	2.7742004
RBM12	0.33061647
RBM15B	0.45573378
RBM18	0.7063832
RBM24	5.3384285
RBM27	1.4554706
RBM28	0.37790823
RBM3	1.0728431
RBM39	0.620172
RBM41	1.1123803
RBM48	0.7520194
RBM5	0.40725994
RBMS1	1.7251334
RBMS2	1.3736606
RBMS3	2.1914077
RBMX	0.749063
RBMX2	0.55262756
RBMXL1	0.39343643
RBP7	1.9986026
RBPJ	1.3347311
RBPJL	3.519784
RC3H2	0.4087491
RCAN1	0.9919205
RCC2	0.48283863
RCN1	0.5518365
RCOR1	0.23000765
RCSD1	0.55652285
RD3	1.9862907
RECK	1.3552356
RECQL	0.71213484
REEP5	0.6077113
REG3B	5.4499755
REG3G	11.221197
RELA	0.74139357
RELB	2.1321206
RELL1	1.4222655
RELT	1.0461106
REP15	0.60049057
REPS1	0.5877242
RERE	0.6980262
RESP18	2.7680523

REST	1.3096952
RET	1.700979
RETN	2.4018292
RETNLA	7.4446454
RETNLB	2.121073
RETNLG	2.9243197
REV3L	2.5895915
RFTN1	1.6901779
RFTN2	1.1719372
RFX2	1.385412
RFX3	0.73327255
RFX8	2.4877195
RGCC	8.2415085
RGMA	0.5545287
RGMB	1.4321108
RGS1	3.2797594
RGS11	2.286849
RGS12	0.63197994
RGS14	0.9590926
RGS17	2.518822
RGS18	2.2638779
RGS19	1.235362
RGS2	2.4729972
RGS22	1.8127158
RGS4	2.9751468
RGS9	3.5335784
RHBDF1	0.3295169
RHBDF2	1.093895
RHBDL3	2.8382912
RHEBL1	0.9568331
RHOA	0.26532316
RHOB	2.2018342
RHOC	2.6950884
RHOF	2.4531703
RHOG	0.4785881
RHOJ	3.7702818
RHOQ	1.0464225
RHOV	2.2265265
RHOX7	0.2984624
RIC8	0.8024502
RICTOR	0.9284358
RIF1	0.41497755
RIIAD1	1.8906887
RILPL1	1.41078
RILPL2	0.7079239

RIN1	1.0335565
RIN2	3.0075178
RIN3	2.1572728
RINL	0.538651
RIOK1	0.9106102
RIPK1	0.6664424
RIPK2	2.5903394
RIPK3	1.5557728
RIPK4	0.52560425
RLF	0.79906607
RLIM	2.4947114
RMRP	1.2808032
RN18S	6.273375
RNASE1	0.35770035
RNASE10	1.3236766
RNASE2A	4.1561236
RNASE2B	1.5820367
RNASEL	1.449224
RND1	1.7811403
RNF111	0.34043312
RNF114	0.26269722
RNF122	1.4147809
RNF144A	1.388782
RNF146	0.9486184
RNF150	2.2263947
RNF157	0.6504452
RNF166	0.21951246
RNF180	0.3263116
RNF19B	1.1458964
RNF207	2.4598227
RNF208	3.5107954
RNF213	0.63545036
RNF216	0.27992153
RNF220	0.35005856
RNF32	0.87329483
RNF34	0.42706442
RNF38	1.070075
RNF39	1.1260481
RNF41	0.27362156
RNF6	0.33595562
RNLS	0.6218338
RNMT	1.130486
RNU12	0.35772657
RNU1B6	2.6118875
RNU2-10	2.0872078

ROBO1	0.38479185
ROBO4	1.8936377
ROCK1	0.77979517
ROCK2	1.7911196
ROR2	2.9619105
RP2H	1.0574403
RPGR	1.0572433
RPGRIP1	2.6815171
RPGRIP1L	0.48530412
RPH3AL	0.3906846
RPIA	0.67893887
RPL29	0.41473532
RPL39L	0.50892234
RPL3L	2.9633744
RPRD2	1.2989178
RPRM	4.744242
RPRML	0.5002885
RPS15A-PS6	0.9673171
RPS19	0.26845598
RPS4L	0.1933775
RPS6KA2	2.210785
RPS6KA3	1.7599187
RPS6KA4	0.37386465
RPS6KB1	0.62437916
RPS6KL1	0.84040666
RPTN	4.2143583
RRAD	4.130578
RRAGD	1.692208
RRP12	1.3549969
RRP1B	2.0309858
RS5-8S1	2.6410527
RSAD2	1.6093502
RSBN1	0.9325514
RSBN1L	0.7224808
RSL24D1	0.3725462
RSPH1	3.513255
RSPH3A	3.2310705
RSPH9	4.080892
RSPO1	0.5650716
RTDR1	2.9106045
RTKN2	5.0564528
RTN1	0.83467627
RTN2	0.60376453
RTP4	0.71141815
RUFY2	0.41938257

RUFY4	0.46481442
RUNDC3B	1.1284356
RUNX1	4.275058
RUNX1T1	3.122151
RUNX2OS1	2.1759977
RUSC1	0.4787073
RWDD1	0.18543768
RYBP	1.185236
RYR1	2.3850732
RYR2	2.60393
S100A11	2.6551666
S100A14	5.872339
S100A4	2.2686367
S100A6	3.9606347
S100A8	4.37015
S100A9	2.8123584
S100B	2.194314
S100G	2.1364822
S100PBP	0.39318657
S1PR1	2.0484815
S1PR3	2.9833212
S1PR4	1.9378417
SAA3	3.7311149
SAMD14	1.3054147
SAMD4	1.7983382
SAMD4B	0.8918271
SAMD5	1.9396911
SAMD8	0.8739722
SAMD9L	1.6024852
SAMHD1	1.3201313
SAMSN1	4.0273476
SAP30	1.1161609
SAPCD1	0.77270126
SART3	0.2786932
SASH1	1.2553158
SASH3	0.7724967
SASS6	1.2164114
SATB1	3.045712
SAV1	2.0222344
SBDS	0.28081703
SBK3	6.316168
SBNO2	1.8893061
SBSN	3.013678
SCAF8	0.18929577
SCAPER	0.26810527

SCARA5	1.3215866
SCARF2	1.1318307
SCCPDH	1.1876736
SCD2	0.7532749
SCEL	4.096954
SCG5	2.892514
SCGB1A1	11.401237
SCGB1C1	5.2214746
SCGB3A1	11.172961
SCGB3A2	7.5824294
SCHIP1	3.1216717
SCIN	3.6459832
SCML4	1.6356575
SCN10A	0.8180063
SCN2B	0.53054667
SCN3A	0.48849154
SCN3B	3.5708356
SCN5A	1.1331754
SCN7A	2.5152051
SCNN1A	4.2661824
SCNN1B	5.208856
SCOC	0.2980771
SCRG1	4.506759
SCRN1	3.8577092
SCTR	0.63427687
SCUBE1	1.0684335
SCUBE2	2.4833
SCUBE3	0.76115537
SCX	3.0310426
SDAD1	1.0781107
SDC4	0.3266015
SDCBP	1.6814489
SDCBP2	3.5889187
SDCCAG8	0.5888226
SDK1	0.5461359
SEC1	4.9453177
SEC14L1	1.3660364
SEC14L3	4.2350984
SEC24B	0.6414037
SEC61A2	0.8141577
SECISBP2	0.55780125
SECISBP2L	2.8140898
SECTM1B	3.4813857
SELE	6.237152
SELL	2.9601846

SELP	6.1561007
SELPLG	1.3456335
SEMA3A	2.175337
SEMA3B	3.8868465
SEMA3C	3.7591352
SEMA3E	2.5082226
SEMA3F	2.1155748
SEMA3G	6.694681
SEMA4D	2.0406885
SEMA4F	0.3841691
SEMA5A	4.2541237
SEMA6A	0.32291222
SEMA6B	0.5199151
SEMA7A	8.501173
SENP2	0.5184121
SENP6	2.048287
SENP7	0.7490711
SEPN1	0.6203046
SERINC3	0.72218513
SERINC4	1.586941
SERP2	1.4477177
SERPINA3B	0.9443283
SERPINA3F	2.6782641
SERPINA3G	0.6021261
SERPINA3I	2.0233684
SERPINA3N	0.62102795
SERPINA9	3.498487
SERPINB10	1.2415214
SERPINB11	4.577564
SERPINB12	2.953943
SERPINB1A	2.2663069
SERPINB1B	1.040617
SERPINB2	4.399993
SERPINB3A	6.302219
SERPINB3B	0.47437143
SERPINB3C	8.042018
SERPINB5	4.791759
SERPINB6A	0.79302263
SERPINB6B	3.692594
SERPINB6C	0.6252942
SERPINB9	1.4936881
SERPINE1	8.162041
SERPINE2	3.2492533
SERPINH1	0.78915644
SERPINI1	0.93442655

SERTAD1	1.0837336
SERTAD2	0.23336935
SERTAD3	0.96079254
SESN2	0.7724576
SESTD1	2.330186
SETBP1	2.500367
SETD2	0.37975407
SETD5	1.3025055
SETD7	0.637825
SETX	0.27722788
SF3B4	0.5630202
SFI1	1.5782127
SFMBT2	0.87082076
SFN	2.5009265
SFPQ	0.3511448
SFRP4	2.2044277
SFRP5	2.1208456
SFSWAP	0.9511719
SFTA2	6.889769
SFTPA1	10.024581
SFTPB	9.869207
SFTPC	10.839071
SFTPD	8.144107
SFXN3	3.1219358
SGCA	2.375127
SGCB	1.4508686
SGCD	0.30009174
SGCG	1.8004944
SGIP1	0.7454684
SGK1	1.2314005
SGMS1	2.0863285
SGPP2	1.892241
SH2B2	0.8339
SH2B3	0.31229258
SH2D1A	0.7789097
SH2D1B1	2.0258038
SH2D2A	0.5522232
SH2D3C	1.634594
SH2D4B	0.6220858
SH2D5	3.1952658
SH3BGR	4.1048384
SH3BGRL3	1.0307794
SH3BP4	1.424809
SH3D21	3.5837703
SH3GL3	3.3959923

SH3KBP1	2.0464125
SH3PXD2A	2.8276339
SH3PXD2B	3.1010017
SH3RF1	0.8611865
SH3TC1	1.2109466
SHANK3	2.8286371
SHC4	0.5642314
SHH	0.6276567
SHISA4	0.45189404
SHISA5	0.7991657
SHOC2	0.52478313
SHROOM3	1.4723856
SIAH1A	0.62102675
SIAH1B	0.32364416
SIDT1	2.1024652
SIGLEC5	3.3432417
SIGLECE	0.20599508
SIGLECG	1.3054554
SIK1	2.4725518
SIK2	0.50405264
SIKE1	0.43316174
SIM2	1.328749
SIN3A	0.4905548
SIPA1L2	1.9955726
SIPA1L3	1.1280417
SIRPB1A	1.6375847
SIRPB1B	1.8466315
SIRT1	0.48348427
SIT1	0.6929271
SIX1	3.812257
SIX4	3.8651736
SKAP1	0.34837675
SKAP2	1.2936339
SKIL	1.816678
SLA	2.6894302
SLAIN1	2.2118206
SLAMF1	3.4626787
SLAMF6	1.7293882
SLAMF7	1.4159482
SLAMF9	0.77368593
SLC10A3	0.9467282
SLC10A6	5.1233892
SLC10A7	1.8615668
SLC11A2	0.38126945
SLC12A2	1.139288

SLC12A4	0.79283
SLC12A6	0.9236045
SLC13A2	2.8310113
SLC13A4	1.274514
SLC13A5	1.5316744
SLC15A2	2.849791
SLC15A3	1.8300476
SLC16A13	1.0360236
SLC16A3	3.273131
SLC16A6	0.6970296
SLC16A8	1.009906
SLC16A9	1.1598625
SLC18A3	0.5691011
SLC19A3	1.1605716
SLC1A1	5.2631145
SLC1A3	2.5574512
SLC1A5	3.3209777
SLC22A13	1.101244
SLC22A17	0.40829802
SLC23A2	1.2422075
SLC23A3	0.8361325
SLC24A3	4.080875
SLC24A4	3.5328176
SLC25A24	1.5410848
SLC25A29	0.5555577
SLC25A35	2.1317492
SLC25A36	2.9739017
SLC25A4	2.1512442
SLC25A41	1.6400621
SLC26A2	1.7399733
SLC26A4	4.92711
SLC26A9	4.010512
SLC27A3	0.72479486
SLC27A6	0.917202
SLC28A2	1.0220456
SLC28A3	2.6171193
SLC29A4	1.1093788
SLC2A1	1.2615371
SLC2A10	1.5433354
SLC2A12	2.439136
SLC2A13	0.9449115
SLC2A3	1.1511145
SLC2A6	2.243915
SLC30A7	0.33629942
SLC31A2	0.5786953

SLC34A2	5.074162
SLC35A4	0.2820487
SLC35E3	1.1211321
SLC35E4	2.9232492
SLC35F2	0.5895314
SLC35F3	0.29732227
SLC35G2	1.533145
SLC36A2	5.396637
SLC36A4	0.87836957
SLC37A1	0.7430601
SLC38A1	2.8533597
SLC38A2	0.6641574
SLC38A5	0.69936943
SLC39A10	2.1080108
SLC39A3	0.4490242
SLC39A6	2.1308684
SLC39A7	0.38683462
SLC39A8	2.3875914
SLC39A9	0.97342896
SLC3A2	1.0485878
SLC41A1	2.007537
SLC43A2	1.3611865
SLC43A3	5.209751
SLC44A2	2.0664687
SLC44A4	4.1941047
SLC45A4	0.46152925
SLC4A11	1.7023041
SLC4A3	1.4798243
SLC4A5	0.85276914
SLC4A8	1.6817107
SLC52A3	4.2693563
SLC5A1	2.7519014
SLC5A9	3.4096422
SLC6A14	4.616393
SLC6A2	0.90876126
SLC6A20A	1.695363
SLC6A4	1.565098
SLC6A8	1.0823464
SLC7A1	4.416548
SLC7A10	0.74865365
SLC7A11	4.234845
SLC7A3	3.3218658
SLC7A5	1.9175067
SLC7A6	2.7632341
SLC8A1	1.0359745

SLC9A1	0.8725939
SLC9A4	1.84953
SLCO1A5	1.3492355
SLCO2A1	0.7455578
SLCO3A1	2.4067025
SLCO4A1	3.6831775
SLCO4C1	3.8481584
SLFN1	3.7888412
SLFN10-PS	2.1505322
SLFN2	2.430634
SLFN3	3.9759479
SLFN4	5.9662714
SLFN8	1.6627555
SLIT2	1.076462
SLIT3	3.355969
SLITRK6	0.29260755
SLK	0.7156143
SLMO1	0.8434851
SLN	9.736036
SLPI	1.9433913
SLTM	0.2031231
SLU7	0.38098097
SLURP1	2.3209345
SLX4	0.5107341
SLX4IP	1.0144465
SMAD1	1.6488786
SMAD3	1.1446466
SMAD4	0.58476496
SMAD5	0.16562796
SMAD6	1.2821164
SMAD7	0.8131881
SMAP1	0.4534731
SMARCA4	0.16677761
SMARCA5-PS	1.3036947
SMARCD3	0.22364902
SMC1A	0.19465399
SMC5	0.5074229
SMCHD1	1.2029524
SMEK2	0.45271206
SMG1	0.44253254
SMIM15	0.48096466
SMIM3	3.3069315
SMIM5	3.7676885
SMOX	2.800736
SMPDL3B	2.1396217

SMPX	2.0875473
SMS	0.37567663
SMTN	0.7843962
SMTNL1	1.9818678
SMTNL2	3.3557186
SNAI1	2.6049309
SNAP23	0.33603144
SNAPC1	0.8184304
SNAPIN	0.7977123
SNCG	1.5517478
SNHG1	1.639883
SNHG11	1.6164865
SNHG12	0.26675606
SNHG18	0.30464458
SNHG3	0.31449223
SNHG5	1.7555583
SNHG7	0.26474237
SNN	1.3131847
SNORA74A	1.52847
SNRNP40	0.20462322
SNRPD1	0.6057277
SNRPF	0.35720778
SNTB2	2.3708596
SNTN	3.4306624
SNX1	1.3572073
SNX10	0.6114931
SNX11	0.4939251
SNX13	0.35047388
SNX17	0.5995393
SNX18	1.1205893
SNX20	2.3777208
SNX22	2.7733874
SNX25	1.0279722
SNX29	0.9166086
SNX30	0.47254944
SNX6	0.25310993
SOAT1	0.91009283
SOBP	3.0999622
SOCS1	1.6230025
SOCS2	2.1204147
SOCS3	4.0248637
SOCS4	0.41125202
SOCS5	0.782423
SOCS7	0.657146
SOGA1	0.27864838

SON	0.46853876
SORBS3	0.37249756
SORCS2	3.5710993
SORL1	3.03585
SORT1	2.680657
SOS2	0.4980402
SOSTDC1	3.2619832
SOWAHC	0.86135054
SOX10	1.3191242
SOX17	0.84169436
SOX2	3.8747914
SOX4	2.0121493
SOX7	2.9437785
SP100	1.046489
SP140	1.0597267
SP2	1.6127439
SP3OS	0.7601485
SP4	0.899127
SP6	1.82708
SPA17	0.91395545
SPACA6	1.8104525
SPAG1	0.77542853
SPAG16	1.5264153
SPAG17	0.8077071
SPAG6	4.4802322
SPAG9	0.31143856
SPARCL1	4.661193
SPATA1	0.5846894
SPATA13	0.28569603
SPATA17	0.6050918
SPATA18	3.0166392
SPATA31	0.4572935
SPATA31D1B	1.0614641
SPATA6	1.9942579
SPATA7	0.45659828
SPATS2	0.6110444
SPDEF	2.1549544
SPECC1	1.2768025
SPECC1L	1.5260315
SPEER1-PS1	2.5595572
SPEER2	1.1143062
SPEER4B	1.7790828
SPEER4C	3.1074924
SPEER4D	1.6430047
SPEER5-PS1	2.8066304

SPEF1	1.1571703
SPEF2	1.5869856
SPEM1	0.9781897
SPEN	0.2858014
SPERT	0.7993319
SPG21	0.44422722
SPHK1	8.527199
SPI1	1.6046071
SPIB	2.7037516
SPICE1	0.2340765
SPIDR	2.090498
SPIN4	1.6997702
SPINK2	0.84518313
SPINK5	5.9559793
SPINT1	4.2794576
SPIRE1	1.7783229
SPN	1.527298
SPOCK2	5.549729
SPON1	4.088042
SPOPL	0.82161355
SPP1	0.15917444
SPRED1	1.4605398
SPRED2	1.2894292
SPRR1A	0.36727667
SPRR1B	1.6832259
SPRR2B	2.8562236
SPRR2D	9.195426
SPRR2E	2.9325757
SPRR2F	2.389241
SPRR2G	1.4832437
SPRR2H	2.5655794
SPRR2I	6.6363096
SPRR2J-PS	3.7162876
SPRR3	3.79263
SPRTN	0.5269449
SPRY1	2.6193838
SPRY2	2.1336806
SPSB1	1.3673306
SPTAN1	0.464231
SPTB	0.8927927
SPTBN1	2.711038
SPTLC2	1.7305217
SPTY2D1	0.54379463
SRCAP	1.2171922
SRCIN1	2.4923575

SRF	0.5676975
SRFBP1	0.22255993
SRGAP1	2.5428958
SRGN	2.9641466
SRI	0.56572723
SRL	4.6244984
SRPK3	0.8469887
SRPX	3.3923419
SRRM2	1.0637956
SRRT	0.2356062
SRSF10	0.271245
SRSF11	0.7830868
SRSF3	1.0222192
SRSF5	0.36251307
SRSF7	0.39538097
SSB	1.2688689
SSBP2	0.3979864
SSBP4	0.32794
SSH2	1.2592449
SSH3	0.7246094
SSPN	1.3446302
ST14	3.7980766
ST3GAL2	1.742063
ST3GAL6	3.1338978
ST6GALNAC2	1.8393316
ST6GALNAC5	2.6547303
ST8SIA1	1.2738068
ST8SIA4	1.7025814
ST8SIA6	2.852315
STAC2	2.0682576
STAG1	0.37184763
STAG2	0.41453075
STAM	0.6151786
STAM2	1.0407138
STAP1	0.3114488
STAR	0.45800138
STARD13	0.87596893
STARD3NL	0.1442051
STAT1	0.37354946
STAT3	1.1934619
STAT4	2.1863155
STC2	1.6674292
STEAP1	5.0714064
STEAP2	0.50992775
STEAP4	0.28921223

STFA1	4.472617
STFA2	4.473102
STFA2L1	5.3194184
STFA3	5.788344
STIL	0.5656841
STK10	0.7597885
STK11IP	0.6090903
STK17B	2.5017085
STK19	0.7145839
STK24	0.38867188
STK3	0.58242655
STK33	5.171219
STK36	1.1037309
STK39	2.0592566
STK4	0.9083972
STK40	0.24390888
STMN2	1.6379623
STMN4	1.1541066
STMND1	2.3365784
STOML1	0.2575631
STON1	0.8154342
STON2	0.7954831
STRA6	3.7720716
STRIP2	2.8528605
STRN	1.043803
STRN3	0.49952316
STX11	3.0348253
STX12	1.1351619
STX16	0.67468023
STX19	1.0636597
STX3	1.5662236
STX6	1.2363615
STX7	0.7843499
STXBP1	1.6604123
STXBP3A	0.98812675
STXBP3B	1.9954877
STYX	0.80475473
STYXL1	2.6348124
SUCO	1.9836524
SUFU	0.8190932
SULF1	3.0510106
SULF2	0.3206854
SULT2B1	2.1158366
SUN1	0.38954592
SURF6	0.17556763

SUSD2	1.0706053
SUSD3	0.5445533
SUSD4	3.8516483
SUV39H1	1.9957728
SUV420H1	1.2112737
SUZ12	0.18389797
SVEP1	2.5668945
SVIL	1.0874186
SWAP70	1.3341465
SWT1	0.4253974
SYCE1	0.98659205
SYCN	0.74194884
SYDE1	1.4717216
SYF2	1.4549446
SYK	1.7485085
SYN2	1.9166427
SYNC	2.6918638
SYNCRIP	1.0238423
SYNE1	0.6926038
SYNE2	0.9123869
SYNGR1	2.6679792
SYNJ1	1.254147
SYNM	2.6694956
SYNPO	2.8100023
SYNPO2	2.6655428
SYNRG	0.5249219
SYT11	1.86731
SYT12	2.1213114
SYT13	5.5682564
SYT5	5.4889374
SYTL2	1.0978305
SYTL3	1.7008948
T2	2.3555007
TAB3	0.44628
TACC1	1.3163891
TACR1	1.318716
TACSTD2	3.44461
TAF1	1.4997368
TAF1D	1.3480635
TAF3	0.88864326
TAF5	0.384614
TAF7	0.8295579
TAF7L	0.7551613
TAF9B	2.1537216
TAGAP	1.6945009

TAGAP1	0.87314034
TAGLN	0.6978326
TAGLN2	1.853991
TAL1	1.5637898
TANC1	1.3581281
TANC2	1.9658835
TANK	0.93147373
TAOK1	0.9840317
TAP1	0.45326138
TARM1	2.7144814
TAS2R131	1.4684496
TAX1BP1	0.9267831
TBC1D1	0.59334755
TBC1D10B	0.9061904
TBC1D10C	0.32827473
TBC1D12	1.2755709
TBC1D14	0.19351578
TBC1D16	1.9049706
TBC1D19	0.8224087
TBC1D2	2.280118
TBC1D20	1.4405804
TBC1D22B	0.5414715
TBC1D23	0.6298957
TBC1D30	0.20160532
TBC1D4	1.2982807
TBC1D5	1.4699836
TBC1D8	3.4312491
TBC1D9	1.5927963
TBK1	1.2851996
TBKBP1	0.29672813
TBL1XR1	0.48241282
TBPL1	0.45797396
TBRG1	0.2075715
TBX1	6.9883494
TBX2	2.0175118
TBX22	0.27719665
TBX4	6.9987926
TBX5	6.167051
TBX6	0.29242706
TC2N	3.349247
TCAP	6.586297
TCEAL1	0.5341811
TCEAL3	0.86738944
TCEAL6	3.1751273
TCEAL7	1.5330775

TCEANC2	0.20870686
TCERG1	0.6857867
TCF15	1.1489105
TCF20	0.184834
TCF21	0.88731956
TCF23	3.0688133
TCF4	2.0809965
TCF7	0.40763903
TCHH	3.3816717
TCHP	0.49418068
TCL1B2	0.5739634
TCOF1	0.99846935
TCP10B	0.5485172
TCP10C	0.4770627
TCP11	2.8608854
TCSTV3	0.40663648
TCTE1	0.64498997
TCTEX1D2	1.2935658
TCTEX1D4	4.5990777
TCTN2	1.1263566
TCTN3	1.5842409
TDG	0.3195014
TDRD3	0.19509935
TEAD1	1.9980583
TEAD3	1.2833841
TEAD4	4.595139
TECRL	1.236969
TEK	0.15780067
TEKT1	7.102256
TEKT2	0.41491532
TEKT4	2.2111435
TEN1	1.0079961
TENC1	0.47389793
TEPP	0.83503103
TERC	1.2952597
TERF2	0.53271246
TES	3.6542392
TESCL	0.85499334
TET2	0.9536114
TET3	0.39559126
TEX26	3.327848
TEX30	0.2304802
TEX40	1.2522285
TEX9	0.3406284
TFAP2A	1.507108

TFCP2L1	0.80938673
TFDP2	0.8735242
TFF1	1.9244885
TFF2	5.2159343
TG	1.7042487
TGFB1I1	2.691111
TGFB2	2.7732306
TGFB3	1.7257748
TGFBI	0.7787914
TGFBR1	0.98875713
TGFBR2	1.6033506
TGFBR3	1.7483993
TGIF1	1.0051022
TGIF2	0.86864686
TGM2	1.6620135
TGOLN1	0.24897861
THAP2	0.43477988
THBD	3.8901706
THBS1	6.55743
THBS3	2.6157742
THBS4	3.7257855
THEM5	3.0202086
THG1L	1.2124093
THOC2	0.45070076
THOC6	0.35284615
THRAP3	0.36864662
THSD4	2.8771076
THSD7A	0.9141884
TIAM1	1.2450492
TIAM2	2.666481
TICRR	0.4751413
TIFA	1.9953904
TIGD2	0.3737383
TIGIT	3.6209788
TIMELESS	2.4420357
TIMP1	5.9118457
TIMP2	1.9408188
TIMP3	5.539886
TIMP4	2.1320868
TINAG	2.8176904
TINAGL1	2.5267363
TIPARP	3.0193152
TJP1	1.2872844
TJP2	1.8740768
TLE3	0.8318467

TLE4	0.23465538
TLK2	1.0227175
TLN1	0.5632992
TLR13	1.6094165
TLR2	1.6774573
TLR4	1.5636492
TLR6	1.0308485
TLR7	1.0072207
TLR8	2.4631145
TM4SF1	2.4085293
TM6SF1	0.86693573
TMBIM1	0.79831266
TMC4	3.376832
TMC5	5.080132
TMCC2	1.1830313
TMCC3	2.7301435
TMCO3	1.0309699
TMED8	0.96959925
TMED9	0.24640608
TMEFF1	3.159181
TMEM100	6.2316413
TMEM107	1.0733151
TMEM116	3.0163045
TMEM117	1.3670893
TMEM119	4.6363306
TMEM120B	0.30065536
TMEM123	0.8084917
TMEM128	0.2341547
TMEM132C	1.0283656
TMEM132D	2.4995286
TMEM138	0.2450347
TMEM139	1.1269045
TMEM140	0.86839485
TMEM151A	0.6172035
TMEM151B	0.48918748
TMEM154	2.0545325
TMEM159	2.7896085
TMEM164	0.49741268
TMEM165	1.0417147
TMEM167B	0.21580458
TMEM17	0.15849042
TMEM170B	0.4571519
TMEM171	1.2295399
TMEM173	1.9512148
TMEM178	1.7395549

TMEM181A	1.0759859
TMEM181B-I	4.066783
TMEM189	0.4239266
TMEM194B	0.34245753
TMEM196	1.2276685
TMEM198	1.306067
TMEM2	2.3517165
TMEM200A	1.0553236
TMEM202	0.734637
TMEM204	1.4559803
TMEM212	6.9157233
TMEM213	6.0053134
TMEM229B	0.39842653
TMEM231	1.5219424
TMEM232	3.4931495
TMEM243	0.56700945
TMEM246	2.4158525
TMEM251	0.37946033
TMEM252	5.713475
TMEM255B	0.7054353
TMEM38A	1.8420911
TMEM40	2.5783572
TMEM43	0.42057562
TMEM45B	1.2915642
TMEM50B	2.3556256
TMEM54	1.7115467
TMEM59L	2.5309477
TMEM61	1.4468842
TMEM63A	0.4317627
TMEM68	0.5134654
TMEM79	0.5312791
TMEM87A	1.8975701
TMEM87B	1.0581465
TMEM88	0.7510209
TMEM9B	0.3519435
TMF1	0.57706976
TMOD1	4.935547
TMOD3	0.5970864
TMPRSS11A	1.905236
TMPRSS11G	2.3955972
TMPRSS13	5.2050676
TMPRSS4	1.9491005
TMSB10	1.5247326
TMTC1	2.8529453
TMX3	1.2321496

TNC	1.4975402
TNF	3.7834496
TNFAIP1	0.8339672
TNFAIP2	0.9838433
TNFAIP3	4.2975836
TNFAIP6	1.9653835
TNFAIP8	1.6482368
TNFAIP8L3	1.6727545
TNFRSF10B	2.8815746
TNFRSF11A	0.9960127
TNFRSF12A	1.8393402
TNFRSF13B	2.603518
TNFRSF13C	2.9831812
TNFRSF17	0.28229785
TNFRSF18	2.324698
TNFRSF1B	1.5765085
TNFRSF21	1.1721253
TNFRSF23	0.61126184
TNFRSF25	0.9008632
TNFRSF26	3.798902
TNFRSF4	1.4969172
TNFSF11	1.3256779
TNFSF13B	2.3018198
TNFSF13OS	1.172957
TNFSF14	1.8618245
TNFSF15	2.6855485
TNFSF8	0.7684989
TNFSF9	4.410564
TNIK	0.4513924
TNIP1	2.9197683
TNIP2	1.5451984
TNIP3	2.6862535
TNK1	5.264709
TNKS	1.0745041
TNKS1BP1	0.60326576
TNKS2	0.38150167
TNN	2.4856093
TNNC1	1.243432
TNNC2	8.57741
TNNI2	6.2824864
TNNI3	3.8245807
TNNI3K	0.61338735
TNNT2	6.7358956
TNNT3	5.3878202
TNP2	0.5894511

TNS1	1.9212656
TNS4	0.5471256
TOM1L2	0.32705402
TOP2A	0.88742733
TOPBP1	0.3415413
TOR3A	0.5877404
TOX2	1.5828302
TOX3	3.5283558
TOX4	0.28495407
TPBG	3.535878
TPD52	1.6278052
TPM1	0.7925205
TPM2	2.1732626
TPM3	1.3417373
TPM4	2.6285453
TPPP3	4.135328
TPR	1.0512376
TPRG	1.8128009
TPSB2	1.8725457
TRA2A	1.129477
TRABD2B	2.939497
TRAF1	3.8939877
TRAF3	0.5675473
TRAF3IP2	1.4614625
TRAF6	0.5436778
TRAF7	0.77013063
TRAK2	1.358283
TRAPPC10	0.320014
TRAPPC8	0.26804996
TRAT1	0.6332779
TRAV3N-3	0.3318298
TRCG1	0.6834655
TRDMT1	0.20995617
TRDN	1.6207867
TREM1	2.521728
TREM2	3.3044086
TREM3	3.6383777
TREML2	1.7590084
TRERF1	1.9721887
TREX1	1.9431944
TREX2	4.42312
TRIB1	0.7276063
TRIB2	2.6539097
TRIB3	0.8895712
TRIM10	2.040784

TRIM12A	0.54737806
TRIM12C	1.455709
TRIM13	0.9298663
TRIM15	1.7815061
TRIM16	4.282848
TRIM21	0.34290123
TRIM29	3.9259887
TRIM3	1.1889443
TRIM30A	1.4289613
TRIM30B	2.8710723
TRIM30C	1.7508609
TRIM30D	1.7044616
TRIM33	0.6648226
TRIM34A	0.4408641
TRIM34B	0.7106662
TRIM35	1.6246595
TRIM36	0.6964431
TRIM39	0.557312
TRIM44	0.41874456
TRIM46	1.0347886
TRIM54	3.2715116
TRIM56	2.007823
TRIM59	0.8465643
TRIM62	1.0053642
TRIM63	5.7284164
TRIM69	1.0408626
TRIM72	1.3507886
TRIM8	0.35892344
TRIO	0.5050616
TRIP10	1.2490177
TRIP11	0.81332016
TRIQK	0.4890623
TRNP1	3.0238876
TRNT1	0.30985212
TROVE2	1.6272197
TRP53BP1	0.5005667
TRP53BP2	0.58623385
TRP53I11	2.500945
TRP53INP1	0.4498725
TRP73	0.75920606
TRPC1	0.77642846
TRPC6	3.2186859
TRPM6	3.9725657
TRPS1	2.6549711
TRPV2	1.2643514

TRPV4	2.5700846
TRPV6	3.997891
TSC22D1	1.0813346
TSC22D3	0.50190735
TSGA10	1.6260452
TSHZ1	0.43974972
TSHZ3	1.8598804
TSLP	2.9447913
TSNAXIP1	1.9723647
TSPAN1	9.685123
TSPAN11	5.59625
TSPAN13	2.1258378
TSPAN15	0.82661057
TSPAN17	0.244524
TSPAN18	0.666697
TSPAN2	3.7631402
TSPAN3	1.2837219
TSPAN32	0.42578745
TSPAN5	3.5426197
TSPAN6	1.5278912
TSPAN8	4.4403424
TSPAN9	0.29118443
TSPO	0.42440748
TSPO2	0.83029985
TSPYL2	1.0898714
TSPYL3	0.93007016
TSPYL4	1.5382061
TSR1	0.5451031
TSSK4	0.61825156
TTBK2	1.0204177
TTC12	2.75809
TTC16	1.0230112
TTC18	3.9144688
TTC21A	2.9755542
TTC22	0.88589525
TTC25	0.49845314
TTC26	0.80279064
TTC28	0.3647666
TTC29	3.583594
TTC34	4.1474686
TTC39B	1.0638547
TTC9	2.111638
TTLL11	1.0701423
TTLL2	1.0436502
TTLL3	1.6279755

TTLL6	3.4352844
TTLL7	3.159776
TTLL9	2.1883268
TTN	3.73734
TTPAL	0.9907613
TTYH1	0.6775081
TUBA1A	3.3153872
TUBA1B	0.15691423
TUBA8	0.92764425
TUBB2B	3.121043
TUBB3	0.54621744
TUBB4A	0.31021738
TUBB6	1.5189543
TUBG2	0.7347255
TUBGCP5	1.0357032
TUFT1	0.58342695
TULP3	1.4527464
TULP4	0.6976156
TUSC1	0.77125645
TUSC5	1.999974
TWF2	0.52193975
TWIST2	2.1033823
TXK	2.0250626
TXLNB	4.672156
TXNIP	1.266398
TXNRD3	1.8340254
TYRO3	1.8487878
TYROBP	0.20925522
U2AF1	0.25090075
UACA	1.0629406
UBA1Y	0.42686868
UBA2	0.22083187
UBA6	0.27590108
UBALD2	0.5466614
UBASH3B	1.4440651
UBE2H	0.8499336
UBE2O	0.26410294
UBE2Q2	1.4286227
UBE2V2	0.45525837
UBE3A	0.2564354
UBE4BOS1	0.54827046
UBL3	0.433949
UBN1	1.3286748
UBN2	0.18606234
UBP1	0.9039159

UBQLN2	0.41133547
UBTD2	1.6509852
UBXN10	4.621902
UBXN11	2.0074244
UBXN2A	0.78330374
UBXN7	0.27586293
UCHL1	1.7051866
UCK2	0.9781585
UCP1	2.4183905
UCP2	1.9773273
UCP3	1.6485
UGGT2	0.39015746
UGT2A1	2.480959
UIMC1	1.9931037
ULK4	0.9554014
UNC13B	2.5561438
UNC13D	0.8875756
UNC45B	1.366107
UNC5B	1.0212293
UNC5C	1.6900063
UNC79	0.5621705
UPF3A	0.1752305
UPF3B	0.32064438
UPK1B	2.2339323
UPK2	2.811908
UPK3BL	2.2248769
UPP1	4.816348
URB2	1.9777226
USP1	0.37528133
USP11	1.0635314
USP13	1.69982
USP16	0.6288123
USP18	0.66593456
USP31	0.6957078
USP32	0.61516905
USP33	0.26843262
USP34	1.705411
USP36	0.69665766
USP37	1.7095287
USP42	0.2934065
USP43	1.9732442
USP47	0.8174195
USP53	2.3961117
USP54	2.2246923
USP6NL	2.2457085

USP7	0.26909256
USP9X	1.0312128
UTF1	2.7843256
UTP14A	1.8231668
UTRN	1.1012611
VAMP2	0.23388863
VAMP3	1.0633917
VAMP4	0.24754906
VAMP5	0.79716396
VANGL2	0.76381636
VAPA	0.45069742
VASN	0.35794878
VASP	1.8506393
VAT1L	0.370934
VAV1	1.6077347
VAV3	0.82796955
VBP1	0.46576738
VCAM1	0.64147043
VCAN	2.3611517
VCL	0.7393837
VDR	3.0891743
VEGFA	1.2638874
VEPH1	1.2793367
VGF	4.270973
VGLL3	0.2121911
VGLL4	1.72119
VILL	0.6470089
VIM	0.58179474
VIPR2	1.8243635
VIT	0.58290863
VMN1R52	0.3970406
VMN1R59	0.8013241
VMN1R70	0.2747221
VMN1R87	1.3740427
VMN2R11	1.0384784
VOPP1	1.2252102
VPREB3	2.5900984
VPS13A	0.54295826
VPS37C	0.5931802
VPS51	1.1049056
VPS53	0.44576883
VPS54	0.44048882
VRK1	0.9237871
VSIG10	0.98881245
VSIG10L	1.0801568

VSIG2	3.6289196
VSIG8	4.097329
VSTM4	1.3347456
VWA3A	4.6560507
VWA5A	0.25628662
VWF	1.6854692
WARS	1.9059324
WARS2	0.1823821
WAS	0.74765587
WASF2	1.8320656
WBP5	1.9911385
WDFY3	0.28352642
WDFY4	0.9047117
WDHD1	0.5057075
WDPCP	0.5138378
WDR1	0.8159137
WDR16	5.0823874
WDR35	0.806025
WDR37	0.48316336
WDR38	0.56401396
WDR43	0.5219836
WDR44	1.0935872
WDR45B	0.5007596
WDR47	2.5058131
WDR48	0.39487314
WDR49	0.3679614
WDR52	1.349292
WDR54	1.3190241
WDR5B	2.9176946
WDR63	2.7230883
WDR65	3.95331
WDR66	3.9847925
WDR75	0.5080404
WDR76	0.8165381
WDR78	3.3651333
WDR92	1.9811702
WDR95	1.6950302
WDR96	2.9471269
WFDC10	1.9171624
WFDC12	4.121529
WFDC17	1.444284
WFDC18	4.578006
WFDC2	5.031354
WFDC3	0.26956606
WFDC5	0.27243733

WFDC6B	0.88360095
WFIKKN2	1.3953335
WFS1	0.3215499
WHRN	3.8222091
WHSC1	0.52833176
WHSC1L1	1.3141632
WIF1	4.130321
WIPF1	1.3039031
WISP2	1.0205846
WIZ	0.2993412
WLS	1.3750687
WNK1	1.1308789
WNK2	0.934803
WNK4	0.5881927
WNT10A	0.8919096
WNT10B	0.5206568
WNT11	2.095677
WNT16	1.5655906
WNT4	0.51129866
WNT6	0.6531849
WNT7A	1.735275
WNT7B	0.8504994
WNT9A	1.9890513
WSB1	1.2641435
WSCD2	0.6734047
WT1	2.504437
WTAP	0.3204198
WTIP	0.42737055
WWC1	0.16136456
WWC2	1.1069932
WWP2	0.21008158
WWTR1	1.2149286
XAF1	1.3701954
XBP1	0.36198902
XDH	0.9333348
XIRP1	1.6033027
XIRP2	1.1169648
XKRX	1.7130513
XLR3B	0.6864872
XLR4A	1.6997209
XLR4B	1.9374895
XPNPEP2	3.9902825
XPO4	0.78653955
XRN1	2.248176
XRN2	0.20226717

XXYLT1	0.81611514
YARS	0.43349957
YBX2	0.68529844
YBX3	0.7626405
YDJC	1.0016572
YEATS4	3.9709744
YIPF7	1.0361586
YTHDC1	0.69573116
YWHAZ	1.1666713
YY2	1.1136713
ZAK	2.0452528
ZBED3	2.2662985
ZBED4	0.19776297
ZBED6	1.4466424
ZBP1	0.4407215
ZBTB10	1.053263
ZBTB11	0.42812204
ZBTB16	2.1508675
ZBTB25	0.43397522
ZBTB32	2.647694
ZBTB34	0.7140894
ZBTB37	0.6406729
ZBTB38	0.55114317
ZBTB4	0.5569725
ZBTB41	0.6336031
ZBTB42	0.2750764
ZBTB46	0.4677055
ZBTB5	0.22079182
ZBTB6	1.7579634
ZBTB7A	0.5267453
ZC2HC1A	2.2934294
ZC3H10	0.24589062
ZC3H12A	3.1263032
ZC3H12C	1.701364
ZC3H12D	0.88859653
ZC3H13	1.455728
ZC3H3	0.20861816
ZC3H6	0.764282
ZC3H7A	0.6661229
ZC3HAV1	0.51197577
ZCCHC11	0.709929
ZCCHC7	0.39358473
ZDHHC1	1.8482943
ZDHHC13	0.27572584
ZDHHC17	0.59452105

ZDHHC18	0.62430954
ZDHHC21	0.43872547
ZDHHC22	1.0819097
ZDHHC23	0.72980356
ZDHHC8	0.17523432
ZEB1	1.3453436
ZEB2	0.8336978
ZEB2OS	0.85449624
ZFAND2A	0.582201
ZFAND6	0.5633168
ZFP1	0.9249358
ZFP105	1.0940971
ZFP106	0.67216635
ZFP11	0.40250063
ZFP12	0.50326085
ZFP131	0.56457853
ZFP142	0.74256706
ZFP143	0.86297536
ZFP146	1.0069818
ZFP169	0.47219086
ZFP182	0.929739
ZFP184	0.8534837
ZFP185	2.5833035
ZFP202	0.4948938
ZFP212	0.5321498
ZFP217	0.7606778
ZFP239	0.36626768
ZFP251	0.9829178
ZFP26	1.5125732
ZFP263	0.2562251
ZFP28	0.28803372
ZFP280D	0.750648
ZFP281	1.1588788
ZFP286	0.9780252
ZFP287	1.2734344
ZFP296	0.39063168
ZFP317	0.9371135
ZFP352	3.089064
ZFP36	1.920269
ZFP365	3.5651968
ZFP366	1.8259213
ZFP36L1	0.4963045
ZFP382	0.72921133
ZFP384	0.79071
ZFP397	0.5030279

ZFP407	0.3923831
ZFP423	0.524842
ZFP429	0.7859497
ZFP445	1.0806966
ZFP456	1.8766503
ZFP46	1.4806318
ZFP462	2.933673
ZFP474	4.4731197
ZFP503	0.89011526
ZFP51	0.78241634
ZFP512	0.4340191
ZFP513	0.35448742
ZFP52	3.4612894
ZFP521	0.93327
ZFP53	1.0028713
ZFP532	0.8120353
ZFP534	0.5323677
ZFP536	1.2631211
ZFP566	0.4870553
ZFP568	0.43829775
ZFP580	0.838294
ZFP583	1.0661528
ZFP592	0.20247364
ZFP593	1.1299605
ZFP597	0.92083406
ZFP607	0.28413105
ZFP608	1.7708673
ZFP61	1.9253793
ZFP62	1.4776325
ZFP626	1.0036983
ZFP641	2.4374561
ZFP644	0.19955444
ZFP651	0.77745247
ZFP664	0.7261281
ZFP668	0.34602928
ZFP677	1.0841572
ZFP687	0.26606798
ZFP697	4.0698385
ZFP7	1.2810206
ZFP703	1.1672277
ZFP704	0.7728708
ZFP711	1.384073
ZFP715	0.32961988
ZFP719	1.0540423
ZFP74	0.336905

ZFP740	1.348144
ZFP747	0.6256819
ZFP758	1.1891451
ZFP764	0.33605576
ZFP781	0.5689521
ZFP783	0.79073715
ZFP790	3.5059762
ZFP800	0.1828146
ZFP819	0.9960823
ZFP821	0.49571037
ZFP839	1.0146282
ZFP84	0.33334637
ZFP850	1.933141
ZFP865	0.48120975
ZFP868	0.34739304
ZFP9	0.9938257
ZFP92	0.42525077
ZFP945	0.5698061
ZFP948	1.08775
ZFP959	0.13657618
ZFPM2	0.6526842
ZFR2	0.46655512
ZFX	0.240942
ZFYVE16	0.705214
ZFYVE20	0.39239693
ZHX2	2.4287312
ZKSCAN6	1.1036725
ZMIZ1	1.9813447
ZMYM2	0.28659868
ZMYND10	1.727448
ZMYND11	0.66260576
ZMYND12	2.9460382
ZMYND15	0.3753276
ZNFX1	0.2348628
ZNRF1	1.0356731
ZRANB2	0.37111092
ZRSR2	0.2922492
ZSCAN18	2.0152798
ZSCAN25	1.0893178
ZSWIM4	0.71466017
ZSWIM6	1.4442682
ZSWIM8	0.39492702
ZUFSP	0.33014393
ZXDB	1.3857064
ZYG11B	0.24231935

Mice_onset_liver_DEgenes_fc

Gene	Log2 Fold Change
A26C2	-1.7508829
A3GALT2	-2.017697
A4GNT	-7.7581644
AADACL3	-3.0964508
AADAT	-3.7811966
ABCA12	-7.2174435
ABCA14	-4.5211353
ABCA16	-3.8805795
ABCB5	-1.5646036
ABCC12	-3.6949248
ABCG3	-4.5412216
ABHD11OS	-2.1867795
ABHD12B	-2.0941937
ABHD16B	-2.7477543
ABLIM2	-2.836097
ABT1	-3.140681
ACER1	-4.498188
ACHE	-3.8119133
ACKR4	-4.2922435
ACOT5	-3.130099
ACPT	-6.079832
ACR	-4.0470543
ACSBG2	-5.6772847
ACSL6	-3.8674092
ACSM2	-2.6844282
ACSM4	-3.5163043
ACTA1	-3.139305
ACTBL2	-1.6212232
ACTL10	-2.526831
ACTL11	-3.3588636
ACTL6B	-4.0819383
ACTL7B	-6.299217
ACTL9	-3.0782325
ACTR3B	-0.9729364
ACTRT2	-3.7191534
ACTRT3	-2.416055
ACYP2	-2.0640123
ADAD1	-1.8344796
ADAD2	-3.2383819
ADAM12	-2.9575188
ADAM18	-4.356778
ADAM1A	-1.3719141
ADAM1B	-3.3723748

ADAM2	-3.5821834
ADAM21	-2.5229669
ADAM24	-3.8433323
ADAM25	-3.010058
ADAM26A	-3.7795339
ADAM26B	-2.180071
ADAM30	-3.5007176
ADAM39	-3.790896
ADAM5	-1.9344366
ADAM6B	-1.2890937
ADAM7	-1.8018007
ADAMTS12	-3.2154708
ADAMTS16	-4.6648207
ADAMTS18	-2.5895293
ADAMTS20	-1.7056932
ADAMTS3	-2.7157152
ADAMTS6	-1.5980968
ADARB2	-3.242807
ADCY10	-3.4994392
ADD2	-2.2961354
ADH6A	-2.417916
ADM2	-2.4244828
ADPRHL1	-2.2090704
ADRA1A	-2.6498404
ADRA1D	-4.1884193
ADRA2B	-2.696878
ADRA2C	-2.4359355
ADRB3	-1.924114
AGBL4	-4.8188596
AGBL5	-2.0513935
AGTR2	-4.05326
AHNAK2	-2.7368848
AHRR	-2.1827366
AHSA2	-1.9080956
AICDA	-3.602463
AIPL1	-2.5287971
AIRE	-3.2848554
AKAP3	-5.3092484
AKAP6	-1.7557709
AKNAD1	-5.3483276
AKP3	-2.2981849
AKR1CL	-2.5569031
ALDH3B2	-2.0213265
ALDOART1	-5.1838036
ALDOART2	-1.8022788

ALK	-2.8846917
ALMS1	-4.192436
ALOX12B	-3.9776797
ALOX15	-3.2607255
ALPI	-1.2997758
ALPK2	-5.328273
ALPK3	-2.7464626
ALS2CR11	-1.9146538
ALX1	-3.099661
ALX3	-1.514035
AMBN	-5.7621765
AMELX	-2.7582216
AMH	-1.1146886
AMTN	-3.6921806
ANGEL1	-1.9441271
ANGPT4	-4.3998337
ANGPTL1	-5.245061
ANK2	-1.8860672
ANKDD1A	-2.9494395
ANKFN1	-4.7282968
ANKLE1	-2.7826967
ANKRD33	-4.8061194
ANKRD34A	-4.751786
ANKRD36	-6.4857025
ANKRD42	-2.2872682
ANKRD55	-3.7313375
ANKRD6	-4.924859
ANKRD61	-1.4563584
ANKRD63	-3.3800523
ANKRD65	-2.4451
ANKRD7	-4.16416
ANKS1B	-3.8107133
ANKUB1	-2.5476894
ANO3	-4.6560035
ANO5	-3.147168
ANO7	-5.0641212
ANO8	-1.8869224
ANXA10	-5.1538873
ANXA13	-2.647185
AP1G2	-1.6910486
AP3B2	-2.2545676
AP4E1	-1.1593411
APBA2	-2.541272
APC	-1.0080347
APOL6	-3.8623984

APOL8	-2.22733
AQP12	-2.7369282
AQP2	-1.376539
AQP6	-5.3415146
ARHGAP19	-2.4838123
ARHGAP20O!	-3.674704
ARHGAP24	-4.7649336
ARHGAP27	-3.337711
ARHGAP33O!	-4.333864
ARHGAP36	-1.4817772
ARHGEF33	-3.46566
ARHGEF37	-1.206023
ARID3C	-4.8859825
ARL10	-0.6690297
ARL13A	-3.9076629
ARL14	-3.2694337
ARL14EPL	-3.0800362
ARL2	-1.8065538
ARMC12	-6.0979967
ARMC7	-1.2245698
ARMC9	-2.3759248
ARNT2	-5.8301945
ARPP21	-4.4069185
ARRDC5	-5.1218023
ARV1	-1.207277
ASB10	-5.7739186
ASB16	-3.409896
ASB17	-1.775981
ASB18	-3.7287297
ASB3	-1.1226363
ASB5	-2.9188356
ASB9	-4.819237
ASCL4	-1.4759855
ASIC1	-5.826148
ASIC2	-3.033411
ASIC4	-2.6768837
ASPHD1	-1.8601224
ASPHD2	-2.485334
ASTE1	-2.9421396
ASTL	-3.2995336
ASTN1	-5.0313377
ASTN2	-5.4274864
ASTX	-5.561833
ASXL3	-3.3097925
ASZ1	-2.4295545

ATAD2	-1.4749348
ATCAY	-2.5552938
ATCAYOS	-2.1289034
ATF7IP2	-4.4478326
ATG9B	-4.059923
ATOH7	-3.0262628
ATOH8	-2.3694246
ATP10B	-3.0452907
ATP12A	-2.904962
ATP13A5	-3.982027
ATP2B4	-2.6220188
ATP4A	-1.3418269
ATP4B	-3.094856
ATP6AP1L	-4.0513997
ATP6V1B1	-4.68404
ATP6V1E2	-3.101808
ATP6V1G3	-3.5421407
ATP8A2	-3.6615708
ATP8B3	-4.052718
AU014756	-5.593396
AU015228	-2.2332249
AU015230	-6.303293
AU015558	-5.841559
AU015584	-2.1469874
AU015621	-2.2290459
AU015680	-1.320179
AU015723	-1.6176634
AU016102	-3.500289
AU017674	-2.0188413
AU018091	-3.1141558
AU018794	-1.6106415
AU019176	-4.630427
AU019990	-3.2057652
AU020094	-1.9203842
AU020951	-3.6777394
AU022434	-3.8407633
AU022751	-4.7884245
AU022934	-3.7049098
AU023617	-2.3416905
AU023639	-6.167323
AU023762	-4.884445
AU024180	-3.805513
AU024245	-4.1719723
AU040096	-1.025276
AU041480	-1.2677233

AU042410	-5.348673
AU042950	-6.603118
AU045094	-1.8390398
AU045717	-2.1260986
AU046084	-5.394721
AU067648	-5.1901727
AV320801	-1.5475008
AVIL	-5.0728207
AVP	-1.8315935
AVPR1B	-4.2545
AVPR2	-7.141492
AWAT2	-2.9423094
B3GALT2	-1.1617026
B3GALT5	-4.231738
B3GAT1	-1.1261725
B3GAT2	-5.107932
B3GNT3	-4.9774923
B3GNT7	-2.9926047
B3GNTL1	-1.3592992
B4GALNT3	-2.5796876
BAG2	-2.5729024
BAI1	-5.4253025
BAI3	-4.409077
BAIAP3	-6.212347
BAMBI	-2.876198
BANF2	-3.3502836
BANK1	-2.0712264
BARX1	-3.2740455
BARX2	-1.2800431
BAZ1B	-1.3305564
BBS7	-1.126689
BCAS1OS2	-3.176046
BCAS3	-1.2062879
BCAT1	-3.6515594
BCL11A	-1.5728247
BCL2L10	-2.8137183
BCORL1	-0.7605329
BECN2	-1.802563
BEND6	-1.3720582
BEST1	-3.3738904
BEST3	-2.4792452
BEX6	-5.5972033
BFSP2	-1.5627167
BGLAP	-4.0419254
BHLHA9	-1.3330941

BHLHB9	-2.3007786
BHLHE23	-4.8839693
BHLHE41	-4.7559547
BLK	-2.7868466
BMP8B	-2.5619073
BOLL	-2.4285219
BPI	-3.9833171
BPIFA2	-4.030844
BPIFA5	-2.32556
BPIFA6	-5.2289686
BPIFB2	-2.6594253
BPIFB3	-1.8491735
BPIFB4	-2.5860145
BPIFB6	-3.7310832
BPIFB9B	-3.6939025
BRICD5	-3.4325275
BRINP3	-6.5619326
BRS3	-3.8334513
BRSK2	-3.6918695
BSN	-2.989301
BSND	-7.094059
BSPH2	-3.2546866
BSX	-4.9607344
BTBD17	-1.8183069
BTBD8	-2.8176436
BTG4	-4.0853357
BTNL1	-5.1924725
BTNL4	-1.3092256
BTNL5-PS	-1.4884493
BTNL6	-2.4349809
BTNL9	-1.7562757
BZRAP1	-3.3650484
C1QL2	-4.62964
C1QL4	-6.0135293
C1QTNF4	-2.8030515
C2CD4C	-5.28119
C2CD4D	-2.7682319
CABP1	-2.9384158
CABS1	-1.5173285
CACNA1A	-1.0077746
CACNA1B	-4.131935
CACNA1E	-6.019102
CACNA1F	-1.6924078
CACNA1G	-1.390549
CACNA1H	-0.9916005

CACNA1I	-3.8124359
CACNA1S	-2.0476704
CACNA2D3	-2.3111706
CACNB2	-2.8924425
CACNB4	-3.3141844
CACNG1	-4.8669596
CACNG2	-3.5902338
CACNG3	-2.0575032
CACNG4	-4.232065
CACNG6	-2.3263326
CACNG8	-6.1158905
CADM2	-2.6507893
CALB1	-5.011591
CALB2	-6.812819
CALCB	-4.0827827
CALCOCO2	-3.616529
CALCR	-4.440233
CALHM1	-5.74175
CALM4	-4.1602473
CALM5	-2.3814256
CAMKV	-4.394308
CAPN1	-2.0615022
CAPN12	-1.8256695
CAPN5	-3.4202483
CAPN6	-1.6930592
CAPN8	-1.6533282
CAPN9	-2.7757812
CAPZA3	-3.0360448
CAR1	-1.9366577
CAR10	-2.8778317
CAR6	-6.0140724
CARF	-1.9680839
CARLR	-5.4962134
CARTPT	-3.0082207
CASC5	-2.105989
CASKIN1	-1.8707485
CASP16	-5.003069
CASP3	-2.448705
CASQ1	-3.4129405
CASR	-2.516424
CASS4	-3.859945
CATIP	-2.1150858
CATSPER3	-3.4191477
CATSPER4	-3.2078123
CATSPERB	-4.754663

CATSPERG1	-1.6928711
CATSPERG2	-1.7318006
CBLN1	-2.523894
CBLN2	-5.229104
CBLN3	-1.0636683
CCDC105	-3.952608
CCDC110	-4.064291
CCDC114	-0.6590965
CCDC116	-2.7164114
CCDC122	-2.1544328
CCDC130	-1.7274868
CCDC14	-4.390803
CCDC142	-2.3089187
CCDC148	-1.9409924
CCDC152	-5.436677
CCDC154	-1.6573956
CCDC158	-4.542261
CCDC169	-1.8126016
CCDC172	-2.8978646
CCDC177	-3.2036066
CCDC178	-1.2000799
CCDC18	-2.2356505
CCDC183	-4.044448
CCDC185	-5.232163
CCDC24	-3.165389
CCDC27	-8.398628
CCDC42	-2.2314446
CCDC42OS	-5.758211
CCDC50	-4.4911795
CCDC54	-3.2506814
CCDC60	-2.4762838
CCDC62	-3.3880954
CCDC63	-1.9270263
CCDC7	-3.2602448
CCDC70	-4.873865
CCDC73	-5.706081
CCDC79	-2.0004306
CCDC8	-4.3645897
CCDC83	-3.794846
CCDC85B	-3.2402377
CCDC87	-3.720035
CCDC89	-1.9951944
CCK	-3.3338437
CCKBR	-1.8523657
CCL20	-5.3968306

CCL22	-0.9622495
CCL26	-5.1104994
CCNB1IP1	-3.2324526
CCNB3	-3.2398362
CCNE2	-1.1812129
CCNJL	-1.2501154
CCNY	-0.8902319
CCR3	-3.5176468
CCSER1	-3.16009
CCT8L1	-3.3150783
CD101	-3.2137876
CD180	-2.5411339
CD200R2	-3.5823398
CD209G	-4.3163004
CD22	-1.4174984
CD300A	-1.672255
CD300LB	-1.2462232
CD300LH	-1.5159521
CD40LG	-4.909962
CD59B	-3.3219051
CD70	-0.9809594
CDAN1	-2.0677881
CDC20B	-4.042754
CDC23	-0.692652
CDC45	-1.6238687
CDC7	-3.2302318
CDH10	-5.486886
CDH12	-3.0741415
CDH17	-4.5898952
CDH18	-4.2845364
CDH20	-6.9300337
CDH23	-2.7860034
CDH24	-3.1923356
CDH26	-3.5107887
CDH7	-2.268508
CDH8	-3.289202
CDH9	-2.9740434
CDHR1	-2.8934247
CDK1	-1.6528392
CDK15	-2.7362716
CDK19OS	-4.9335737
CDK5R2	-5.10495
CDKN2A	-0.9768834
CDNF	-2.2312243
CDRT4	-3.1222684

CDRT4OS1	-3.838694
CDSN	-2.9483786
CDX2	-3.3991413
CDX4	-2.2653708
CEACAM12	-3.9985626
CEACAM13	-4.4734774
CEACAM14	-1.5630214
CEACAM19	-2.4277368
CEACAM20	-4.8941855
CEACAM5	-4.71708
CEACAM9	-3.4605281
CEACAM-PS1	-2.3559897
CECR6	-6.4820805
CEL	-2.3172815
CELA1	-4.185313
CELA3B	-4.016753
CELF3	-4.3716917
CELF5	-3.6897864
CELF6	-3.5674822
CELRR	-5.5233583
CEMIP	-3.7376754
CEND1	-2.5403671
CENPI	-4.170554
CENPP	-2.935065
CEP135	-1.8110356
CEP57L1	-3.1170812
CEP78	-2.581548
CER1	-4.6147313
CERS3	-4.8044415
CES1H	-2.0936267
CES2H	-4.612711
CES4A	-4.751436
CES5A	-4.571713
CETN1	-2.8665795
CFC1	-1.1757905
CFTR	-0.7845092
CGA	-2.5155766
CHAF1A	-2.1069639
CHEK2	-3.7863526
CHIT1	-3.003421
CHODL	-2.063883
CHP2	-2.2343843
CHRD	-1.5320399
CHRDL2	-2.1931267
CHRM1	-2.5978332

CHRM4	-3.291516
CHRM5	-6.6777472
CHRNA1	-1.9203551
CHRNA2	-1.4121106
CHRNA3	-3.0500004
CHRNA5	-4.4786463
CHRNA6	-3.347021
CHRNA9	-3.1677501
CHRNB2	-3.8976898
CHRNB3	-5.390534
CHRNB4	-2.022659
CHRND	-5.4706936
CHRNG	-4.5172067
CHST4	-3.69311
CHST9	-1.3517587
CIB4	-0.9509704
CIITA	-4.52442
CILP2	-4.226814
CK137956	-4.2210016
CLCA5	-7.021151
CLCA6	-2.3974607
CLCN1	-1.3051276
CLCN6	-0.7805703
CLDN11	-2.2461717
CLDN13	-4.5650377
CLDN16	-5.4603324
CLDN17	-4.177312
CLDN20	-2.9518402
CLDN23	-2.042815
CLDN24	-4.188113
CLDN26	-2.6390693
CLDN4	-2.9287074
CLDN6	-3.6809938
CLDN9	-3.002137
CLDND2	-0.7729843
CLEC11A	-0.7235599
CLEC12A	-3.8999488
CLEC18A	-3.6700985
CLEC2F	-3.9434252
CLEC2H	-5.986856
CLEC2I	-2.0373037
CLEC2L	-2.54728
CLEC4A2	-3.5491664
CLEC4A4	-1.852103
CLPS	-1.8838739

CLPSL2	-1.933455
CLRN1	-4.467123
CLRN2	-5.048008
CLRN3	-4.9694567
CLSTN3	-1.1423106
CLVS1	-2.9127173
CML5	-2.9913268
CMTM2A	-2.3895886
CMTM5	-2.038147
CNBD1	-3.2710369
CNDP1	-4.8318605
CNFN	-1.9387574
CNGA3	-3.7343514
CNGA4	-1.918895
CNGB1	-1.7794008
CNIH3	-3.4263659
CNNM1	-3.097611
CNPY1	-4.086199
CNTD1	-2.5181284
CNTN2	-2.9128225
CNTN3	-2.0673535
CNTN4	-2.7646854
CNTN5	-7.53813
CNTN6	-2.215419
CNTNAP2	-3.7768445
CNTNAP3	-5.4758997
CNTNAP5A	-5.7381115
CNTNAP5B	-5.4810534
CNTNAP5C	-2.7861934
COCH	-1.1952493
COL10A1	-3.9807904
COL11A2	-3.6839435
COL15A1	-2.327864
COL19A1	-2.2077203
COL22A1	-2.8135037
COL24A1	-3.441538
COL25A1	-5.4554777
COL26A1	-2.0484734
COL28A1	-3.1977162
COL6A6	-3.4874685
COL8A1	-2.386883
COL9A1	-5.2306433
COL9A2	-2.0323894
COPG2OS2	-4.204694
COPS7B	-0.6767805

COQ3	-2.19593
CORT	-1.7125015
COX10	-4.485511
COX8C	-2.8297021
CPA1	-3.6948
CPA2	-5.5349445
CPA4	-5.07251
CPA5	-2.902794
CPLX3	-4.037681
CPLX4	-3.9281442
CPNE4	-3.9913516
CPNE6	-2.7222593
CPNE7	-2.3362515
CPSF4L	-3.7867591
CPVL	-4.1761885
CPXCR1	-2.4298742
CPXM2	-5.412303
CRABP1	-2.0423024
CRB1	-2.2458699
CRB2	-2.448843
CRCT1	-6.0771017
CREG2	-4.5998945
CRH	-4.982259
CRHBP	-5.934413
CRHR1	-1.9103832
CRISP1	-2.3050728
CRISP2	-2.2090232
CRISP3	-3.809449
CRNN	-5.905733
CRTAC1	-2.7698205
CRX	-1.3703177
CRXOS	-3.2341099
CRYBA1	-3.4480717
CRYBA2	-4.8583527
CRYBB1	-3.394464
CRYBB2	-4.100625
CRYGB	-5.894858
CRYGC	-5.72671
CRYGD	-1.8668482
CRYGE	-2.470307
CRYGF	-3.7024393
CRYGN	-2.13141
CSDC2	-2.1513855
CSMD1	-2.3506322
CSMD2	-5.2425594

CSMD2OS	-3.53895
CSN1S2A	-2.6157804
CSN1S2B	-4.19029
CSN3	-3.9191034
CSNKA2IP	-1.9609652
CST10	-5.256765
CST11	-3.1028888
CST12	-5.035165
CST13	-2.338666
CST6	-2.5859268
CSTAD	-1.7579231
CSTL1	-4.1864095
CTCFL	-2.6708908
CTF2	-1.8370132
CTHRC1	-2.8919606
CTNNA2	-1.577733
CTNNA3	-2.66833
CTRL	-0.9415321
CTS3	-4.5734224
CTS6	-4.9927516
CTS7	-6.1256
CTS8	-1.9302351
CTSM	-1.5775535
CTSQ	-1.4090633
CTTNBP2	-2.1292062
CTXN2	-4.8507543
CTXN3	-1.3222365
CUBN	-3.1219702
CUZD1	-2.4677045
CWH43	-2.7246842
CXXC4	-2.8874865
CYB5R2	-4.1341076
CYCT	-5.5045576
CYLC2	-6.3389306
CYP11A1	-2.5175955
CYP11B2	-3.5317984
CYP19A1	-2.5254884
CYP24A1	-1.454447
CYP26B1	-4.651969
CYP26C1	-3.4344387
CYP2AB1	-3.1438034
CYP2B23	-3.7608173
CYP2C65	-3.0584462
CYP2J11	-4.2105193
CYP2J9	-1.6280842

CYP4A29	-6.328577
CYP4A30B	-2.5463834
CYP4F39	-2.4348598
CYP4F40	-4.331174
CYP4F41-PS	-4.195306
CYP4X1	-5.2163754
CYPT10	-2.0066185
CYPT12	-5.7118874
CYPT15	-2.7324495
CYPT2	-3.3263156
CYPT8	-3.1737132
CYPT9	-2.3692296
DAB1	-1.670114
DACH2	-2.8798888
DAO	-4.418554
DBHOS	-4.889377
DBIL5	-2.7817564
DBP	-2.346736
DBPHT2	-1.0115166
DBX2	-3.1958256
DCDC2A	-2.586745
DCDC2B	-2.5060341
DCDC2C	-1.6042938
DCLK1	-1.8010266
DCPP1	-5.3880687
DCST1	-2.673536
DCST2	-2.6143754
DCTD	-3.4204118
DCUN1D3	-1.8191543
DCX	-3.176572
DDI1	-1.1389709
DDIT4L	-1.54674
DDX11	-1.9609847
DEAR1	-3.9125304
DEFA1	-6.246646
DEFA10	-6.2286377
DEFA2	-5.3009114
DEFA21	-4.509836
DEFA23	-7.524692
DEFA25	-6.616301
DEFA26	-5.0200205
DEFA3	-4.1984963
DEFA-PS1	-6.228673
DEFA-PS13	-2.5534618
DEFA-RS1	-4.536931

DEFA-RS12	-2.7933235
DEFA-RS2	-1.1588836
DEFA-RS4	-1.3645506
DEFA-RS7	-1.8768275
DEFB10	-1.8107364
DEFB13	-5.899048
DEFB14	-4.906064
DEFB18	-6.281938
DEFB19	-3.8383884
DEFB2	-5.00808
DEFB20	-7.765526
DEFB21	-3.7193406
DEFB22	-5.3504972
DEFB23	-2.1373088
DEFB26	-4.5527515
DEFB28	-3.6976898
DEFB29	-3.444456
DEFB30	-3.8393946
DEFB35	-3.427053
DEFB36	-5.5332227
DEFB38	-4.2435255
DEFB39	-4.331663
DEFB4	-3.9947493
DEFB40	-4.291498
DEFB41	-5.0626793
DEFB44-PS	-1.8366172
DEFB45	-2.124277
DEFB46	-4.37479
DEFB47	-4.085261
DEFB48	-4.8289747
DEFB6	-6.1686254
DEFB7	-6.437699
DEFB9	-4.151095
DEGS2	-2.3338013
DEPDC5	-1.1000576
DFNB59	-3.6771295
DGKB	-2.922951
DGKI	-2.8175712
DGKK	-6.1034184
DHRS2	-3.7866244
DHX37	-0.981859
DIO3	-2.125058
DIO3OS	-2.3099144
DIRAS1	-3.1907153
DISP1	-0.8699331

DISP2	-4.391842
DIXDC1	-4.2392325
DKK1	-1.4382823
DKK4	-5.769397
DLEC1	-1.4103565
DLG2	-2.6669405
DLGAP2	-1.6467083
DLGAP3	-1.6813412
DLX1	-2.1133378
DLX5	-5.8606873
DLX6	-6.7720695
DLX6AS2	-1.8609223
DLX6OS1	-4.4544106
DMBT1	-3.2599566
DMC1	-3.942582
DMP1	-2.402099
DMRT1	-1.9719753
DMRT3	-5.132558
DMRTA1	-1.1247587
DMRTB1	-3.861589
DMRTC1B	-2.498415
DMRTC1C2	-3.402088
DNAH14	-2.1874495
DNAH17	-4.106693
DNAH3	-1.2301905
DNAJB7	-2.2079358
DNAJB8	-4.7765265
DNAJC5G	-1.7226493
DNAJC6	-1.5760136
DNASE1	-5.420412
DNER	-1.7928929
DNMBP	-2.041287
DNMT3AOS	-2.3234727
DNMT3BOS	-1.8055279
DOC2A	-6.164473
DOCK7	-3.3705204
DOK6	-4.35983
DOXL2	-3.3006659
DPF1	-3.6167254
DPPA2	-2.2914562
DPPA3	-4.4237
DPPA4	-2.7622175
DPYSL4	-2.1521587
DRAXIN	-6.423621
DRD1A	-1.3661315

DRD2	-4.357682
DRD3	-5.1903048
DRD4	-4.1635923
DRD5	-2.5244217
DRP2	-3.2710772
DSC1	-2.4854608
DSC3	-4.059827
DSCAM	-4.0546746
DSCAML1	-2.0487912
DSG1C	-3.192582
DSG3	-3.456806
DSG4	-4.300971
DSPP	-6.1720715
DTWD2	-1.0935915
DUOX1	-2.668474
DUPD1	-6.8225527
DUSP13	-3.8603854
DUSP15	-4.1045046
DUSP18	-8.366945
DUSP27	-3.0667264
DUX	-1.2409832
DUXBL1	-4.1200366
DUXBL2	-3.8608687
DYDC1	-3.6417847
DYNAP	-4.2602625
DYNC1I1	-2.3342378
DYRK4	-4.7767806
DYTN	-4.5203934
DZIP1L	-4.436671
EAR4	-8.288403
EBF3	-1.2609916
EBF4	-2.4453166
ECEL1	-4.7359676
EDA2R	-1.3275678
EDAR	-3.921781
EDARADD	-1.9112864
EDDM3B	-4.8012915
EDN2	-5.361759
EDN3	-3.1911175
EEF2K	-2.8362842
EFCAB11	-3.7470107
EFCAB3	-4.6708694
EFCAB4B	-1.595099
EFCAB7	-3.5085444
EFCAB8	-3.3171172

EFCAB9	-6.7866755
EFCC1	-4.548435
EFHD1	-2.9580076
EFNA2	-2.6056006
EFNA3	-2.9595454
EFNA4	-1.2391798
EGLN1	-2.4779208
EGR3	-4.3297358
EGR4	-1.3557856
EIF4E1B	-1.8398862
ELAVL3	-4.721821
ELFN2	-3.5602725
ELL3	-1.2083366
ELMO1	-3.7964077
ELOVL4	-2.3845751
EMC10	-2.100973
EMILIN3	-1.7043157
EMX1	-4.372904
EMX2	-2.3190784
EMX2OS	-3.5334647
EN2	-0.750931
ENAM	-3.8876534
ENPP6	-4.6246066
ENPP7	-2.01517
ENTHD1	-4.2766447
EPB4.2	-3.382096
EPGN	-1.7463081
EPHA10	-4.104055
EPHA6	-2.766456
EPHX3	-1.7443516
EPO	-4.5406704
EPPIN	-4.988168
EPS8L2	-3.2182102
EPSTI1	-3.0946147
EPX	-2.2255156
EQTN	-1.6066487
ERAS	-5.2096577
ERBB3	-0.6780746
ERC1	-1.149365
ERICH6	-1.4584887
ERMAP	-1.0334384
ERN2	-1.7259424
ERV3	-3.6589303
ESP1	-1.7441483
ESP6	-5.8843718

ESPN	-2.931613
ESPNL	-0.8264735
ESRRB	-1.8232665
ESRRG	-1.5931587
ETOHD2	-0.9464438
ETOHI1	-1.4741235
ETV2	-7.158449
ETV3L	-3.5415528
EVC	-3.1516542
EVI2A-EVI2B	-1.2839074
EXOC3L2	-3.149057
EXOC4	-0.8835337
EXOG	-1.2161419
EXT2	-1.1076231
EYA4	-1.3999796
F2RL2	-1.0535429
F2RL3	-2.3809783
F8A	-4.0482597
FABP6	-4.794379
FABP9	-5.910386
FADDOS	-3.76435
FAM115E	-1.3737304
FAM131B	-5.1412387
FAM131C	-4.3671536
FAM132B	-4.0977683
FAM135B	-3.027444
FAM154A	-3.9969547
FAM160A1	-2.2993848
FAM163B	-5.5107183
FAM166A	-1.3309815
FAM169A	-2.2242181
FAM170A	-4.379751
FAM171A2	-4.054721
FAM181A	-4.528
FAM184A	-1.1573172
FAM184B	-3.0311332
FAM187A	-1.9525557
FAM188B2	-5.976037
FAM196B	-2.3096325
FAM198A	-3.0709653
FAM19A4	-1.4787669
FAM19A5	-1.9088626
FAM209	-1.5497174
FAM217A	-2.2140343
FAM217B	-1.964078

FAM219AOS	-4.4408455
FAM221B	-3.5957186
FAM227A	-4.6089854
FAM227B	-4.0656834
FAM228A	-1.6626489
FAM228B	-4.06659
FAM24A	-3.5914705
FAM3B	-2.2606776
FAM46D	-2.8682714
FAM50B	-3.8745768
FAM57A	-2.742374
FAM57B	-2.0960186
FAM69C	-2.504882
FAM71A	-3.9144151
FAM71E2	-4.765522
FAM71F1	-4.3594065
FAM83B	-2.2975047
FAM83C	-4.8761635
FAN1	-1.5124838
FANCG	-3.1622508
FANCI	-4.91901
FAR2OS2	-3.3212347
FAT2	-0.8987064
FATE1	-2.5563133
FBLL1	-7.5875535
FBLN7	-3.883827
FBN2	-2.94859
FBP2	-3.0116384
FBXL2	-1.1385605
FBXL21	-1.3511553
FBXO16	-2.653238
FBXO39	-3.3382776
FBXO40	-1.890919
FBXO41	-5.7716117
FBXO43	-5.7568464
FBXO44	-4.048739
FBXO47	-5.4022994
FBXO48	-3.6716182
FBXW10	-4.655546
FBXW14	-3.3931358
FBXW15	-2.7570815
FBXW16	-1.6392217
FBXW18	-2.9930644
FBXW19	-4.5439043
FBXW20	-1.6113856

FBXW21	-2.2289326
FBXW22	-3.037213
FBXW26	-4.5296383
FBXW27	-1.4889853
FCAMR	-3.2233617
FCER2A	-1.5521777
FCNB	-0.8550563
FCRL1	-1.2732265
FCRL6	-4.0035667
FCRLB	-3.8182683
FER1L6	-3.245083
FERD3L	-6.3352942
FEV	-2.738444
FEZ1	-3.6162505
FFAR2	-2.6874776
FFAR3	-0.8371663
FGD1	-2.8791611
FGF13	-2.2413974
FGF14	-3.622183
FGF15	-2.4129305
FGF16	-1.4395387
FGF2	-2.576109
FGF20	-4.917815
FGF3	-1.4344218
FGF4	-5.9047513
FGF6	-1.0400689
FGF8	-2.7719028
FHIT	-3.4539735
FHL5	-2.4470003
FIGLA	-4.9415035
FKBP11	-4.4383717
FLG	-4.00576
FLG2	-3.0886188
FMN1	-2.7893806
FMNL1	-4.316779
FMO6	-2.3251448
FMO9	-2.1853392
FMR1NB	-4.349582
FND3C2	-4.175272
FNDC3C1	-7.1435065
FNDC9	-2.4005823
FOLR1	-2.7037127
FOLR4	-0.7939684
FOXB1	-3.6084678
FOXB2	-6.4648356

FOXD3	-1.3796592
FOXD4	-3.4436805
FOXE1	-6.1881294
FOXE3	-1.6748383
FOXG1	-2.708455
FOXH1	-3.8677664
FOXI1	-1.9097517
FOXI2	-2.457476
FOXI3	-3.0012915
FOXJ2	-3.1550705
FOXL1	-3.3099284
FOXL2OS	-3.3777628
FOXN1	-2.317119
FOXO4	-3.0437298
FOXP2	-2.2836266
FOXR1	-3.9673193
FREM2	-0.9466965
FREM3	-3.0302408
FRMD3	-2.5536623
FRMD6	-3.448892
FRMD7	-2.3038518
FRMPD3	-2.3037758
FRRS1L	-4.3402376
FRS3OS	-4.3273134
FSBP	-2.9383602
FSCN2	-2.5890617
FSCN3	-3.3838258
FSD1	-3.2220018
FSD2	-10.325954
FSHB	-5.0352783
FSHR	-4.3063
FSIP1	-2.3163457
FSIP2	-4.4635525
FTHL17	-2.4432738
FTMT	-3.2454772
FTX	-1.0633507
FUT9	-2.173056
FV1	-3.651067
FXYD2	-2.303481
FXYD7	-5.070912
FZD5	-3.0207152
G6BOS	-1.9392171
G6PC2	-5.9832325
GAB2	-3.664959
GABRA1	-1.9547961

GABRA2	-1.5220866
GABRA3	-2.1183453
GABRB1	-4.834883
GABRB2	-3.6497993
GABRD	-2.9266026
GABRG1	-7.626641
GABRG2	-3.3652341
GABRG3	-3.7827094
GABRR3	-3.994593
GAD1	-3.7204967
GAD1OS	-5.7397203
GAD2	-7.0373106
GADL1	-2.964863
GAL	-1.310132
GAL3ST2	-2.0298278
GALNT13	-3.0755773
GALNT14	-2.6657255
GALNT5	-5.459552
GALNT9	-1.7234323
GALNTL5	-2.7277074
GALP	-3.6396952
GALR3	-5.7415304
GALT	-1.0914662
GAPDHS	-1.5896101
GAREML	-4.8301306
GAS1	-1.3886452
GAS2L2	-5.494871
GAS7	-1.461515
GAST	-3.1152284
GATA5OS	-2.510133
GBGT1	-4.117504
GBX1	-6.0631247
GBX2	-2.9663565
GCAP8	-3.82347
GCG	-3.4406917
GCM2	-4.8970356
GCNT3	-7.099262
GCNT4	-3.1730795
GCNT7	-0.9776223
GDAP1	-2.010913
GDAP5	-3.7437563
GDF1	-3.2839587
GDF11	-3.2652142
GDF3	-1.2671051
GDF5	-5.826496

GDF6	-4.6176777
GDF7	-4.3783336
GDPD4	-2.533965
GEN1	-1.9626741
GFAP	-3.6667275
GFOD1	-1.4687021
GFRA3	-2.2797375
GFRAL	-4.0035634
GFY	-4.013758
GGT1	-6.3308077
GH	-3.4749074
GHRH	-1.7082069
GHRHR	-2.9786975
GIMAP1OS	-2.7393992
GINS2	-0.9410045
GIP	-6.4738493
GIPC3	-5.223678
GIPR	-5.1091824
GJA10	-1.8764403
GJA3	-4.838089
GJA6	-2.95549
GJA8	-3.1209078
GJD2	-4.8009577
GJE1	-1.9479885
GK2	-7.165703
GLB1L3	-2.8369668
GLDN	-3.208217
GLDNOS	-5.5968795
GLI1	-3.3219295
GLI2	-1.6690044
GLIPR1L1	-4.8732986
GLIS1	-1.4661787
GLP1R	-3.4629338
GLRA1	-2.4936557
GLRA2	-2.2168665
GLRA4	-6.284828
GLRB	-4.740969
GLRP1	-2.1093931
GLT6D1	-3.1048903
GLYATL3	-5.834057
GLYCAM1	-5.83482
GM10007	-1.960135
GM10023	-1.8975909
GM10024	-4.4893093
GM10069	-6.553505

GM10071	-4.018426
GM10081	-4.2136993
GM10113	-3.9171464
GM10125	-2.9776084
GM10143	-1.6251774
GM10158	-2.4553754
GM10165	-1.995347
GM10190	-1.2947185
GM10199	-4.3191814
GM10209	-2.762049
GM10220	-5.12807
GM10229	-3.385319
GM10265	-2.7811537
GM10266	-2.887699
GM10276	-3.3392348
GM10278	-5.37289
GM10286	-2.7870636
GM10287	-2.801547
GM10334	-2.019175
GM10369	-3.8875968
GM10370	-5.6997757
GM10371	-1.6370134
GM10372	-5.1682215
GM10373	-4.1295195
GM10375	-5.2374
GM10385	-2.9834828
GM10389	-2.5696414
GM10403	-5.2980614
GM10408	-3.9432724
GM10413	-3.147041
GM10415	-4.543681
GM10419	-4.0295076
GM10421	-1.9440131
GM10426	-1.5350404
GM10428	-1.6313205
GM1043	-4.949793
GM10431	-6.652645
GM10432	-4.813204
GM10435	-0.7701559
GM10437	-3.5185084
GM10439	-2.37968
GM10440	-4.5341415
GM10441	-2.9568923
GM10445	-1.8464396
GM10450	-4.015571

GM10457	-1.9792099
GM10465	-5.456974
GM10469	-4.6156588
GM10471	-3.4892795
GM10474	-3.4061894
GM10475	-3.939812
GM10482	-4.0965204
GM10487	-1.6163783
GM10489	-4.311122
GM10493	-2.4476123
GM10498	-5.3928022
GM10501	-4.041031
GM10503	-6.046542
GM10510	-2.7286549
GM10512	-3.9402497
GM10513	-4.7292037
GM10516	-4.1218095
GM10531	-5.9520016
GM10532	-2.72777
GM10535	-3.3428485
GM10536	-7.214511
GM10537	-2.9901812
GM10538	-5.3884683
GM10544	-1.9664552
GM10548	-4.8947215
GM10555	-2.3728054
GM10560	-3.6117725
GM10561	-4.669379
GM10568	-2.6701424
GM10572	-3.1300416
GM10578	-2.2033727
GM10579	-3.9220605
GM10584	-5.1034207
GM10588	-3.5993109
GM10603	-3.05697
GM10609	-2.5218766
GM10614	-3.6554463
GM10616	-2.3917964
GM10618	-1.2660906
GM10620	-1.558986
GM10623	-3.642943
GM10624	-3.2030566
GM10632	-1.2058372
GM10637	-2.9397297
GM10651	-2.2699966

GM10673	-0.8409939
GM10674	-5.1707354
GM10677	-3.279762
GM10682	-3.1209333
GM10683	-3.1115055
GM10684	-3.2964313
GM10687	-4.350784
GM10688	-4.9837503
GM10690	-3.0405173
GM10701	-3.565334
GM10703	-1.409776
GM10706	-4.322626
GM10714	-3.9139721
GM10723	-2.1636178
GM10724	-3.5978951
GM10727	-3.2406971
GM10732	-3.8830779
GM10739	-1.261558
GM10741	-5.222952
GM10744	-4.70949
GM10745	-3.0556514
GM10747	-5.7134857
GM10752	-5.152336
GM10754	-4.817847
GM10755	-2.5390978
GM10757	-3.443757
GM10759	-2.8663137
GM10764	-1.8412676
GM10773	-2.543113
GM10779	-5.0963016
GM10781	-3.5494914
GM10782	-3.1203718
GM10787	-3.9527173
GM10789	-4.2965164
GM10790	-2.3537858
GM10802	-6.324935
GM10804	-4.352523
GM10814	-3.5295649
GM10815	-4.2032146
GM10817	-4.7923207
GM10822	-2.4434144
GM10823	-2.9240227
GM10826	-3.1284409
GM10830	-3.2888696
GM10831	-3.1851156

GM10835	-3.2203295
GM10836	-5.7204847
GM10844	-4.0270076
GM10846	-2.3611715
GM10848	-1.2269735
GM10857	-4.996564
GM10860	-2.78358
GM10863	-3.2531953
GM10864	-3.7541342
GM10867	-5.597349
GM10869	-1.6808119
GM10873	-1.8197196
GM10876	-2.5540693
GM10926	-5.6006584
GM10933	-2.4133663
GM1096	-3.662611
GM1110	-1.5695963
GM11128	-2.2141142
GM11149	-2.1700537
GM11159	-2.6318946
GM11237	-2.3685722
GM11266	-3.4983368
GM11381	-4.201885
GM11468	-4.7523484
GM11492	-1.0458896
GM11497	-2.9814415
GM11529	-3.528121
GM11541	-6.252399
GM11543	-4.3305645
GM11544	-2.1305556
GM11546	-3.5437062
GM11554	-2.922224
GM11563	-2.05876
GM11568	-1.9164138
GM11570	-2.2510734
GM11595	-2.0156567
GM11634	-2.6992946
GM11677	-1.2560132
GM11681	-2.6501637
GM11691	-5.4440527
GM11710	-1.3243926
GM11735	-6.0147963
GM11747	-1.0283442
GM11757	-2.5031204
GM11780	-1.6567667

GM11938	-2.7889473
GM11961	-1.1093202
GM12	-1.4892173
GM12000	-3.3200767
GM12052	-1.4008899
GM12059	-4.0016174
GM12108	-3.0964317
GM12128	-4.8332515
GM12169	-1.4593747
GM12171	-2.2349677
GM12253	-1.7496672
GM12258	-4.388034
GM12273	-4.451142
GM12298	-1.5137358
GM12302	-2.2283287
GM12349	-2.1635628
GM12359	-1.9147077
GM12371	-3.9142807
GM12408	-4.4949408
GM12409	-2.9486613
GM12472	-4.293893
GM12474	-2.0691156
GM12511	-3.330959
GM12542	-4.9969883
GM12568	-1.972585
GM12580	-4.6451626
GM12633	-3.7780612
GM12680	-1.3472543
GM12695	-9.486035
GM12789	-4.1703835
GM12790	-5.094413
GM12794	-4.2278004
GM12830	-2.8018913
GM12863	-4.1270747
GM12866	-2.7989664
GM12886	-3.2762523
GM12888	-4.964997
GM12992	-2.941951
GM13011	-3.0043018
GM13032	-4.1276064
GM13051	-4.292367
GM13078	-1.8022599
GM13084	-2.7829251
GM13088	-3.465682
GM13112	-4.2434235

GM13119	-4.9660497
GM13124	-4.8317814
GM13125	-3.4208388
GM13128	-4.077855
GM13152	-3.4898753
GM13205	-5.171689
GM13219	-3.2669463
GM1322	-5.8876095
GM13247	-1.952807
GM13275	-2.8608902
GM13372	-3.962556
GM13375	-3.2669284
GM13391	-2.262538
GM13403	-5.266036
GM13420	-1.7750506
GM13429	-3.3533864
GM13446	-4.950182
GM13490	-3.0593226
GM13498	-1.9624314
GM13522	-1.666208
GM136	-3.3245854
GM13629	-1.4914782
GM13710	-3.0131104
GM13769	-4.0513773
GM13807	-5.452168
GM13939	-1.7984138
GM14025	-1.9979064
GM14085	-1.6221027
GM14092	-4.8594
GM14124	-1.6908267
GM14129	-4.0775213
GM14149	-3.2971365
GM14164	-4.069101
GM14243	-5.4256816
GM14317	-3.1961179
GM14340	-2.8142836
GM14461	-3.5990736
GM14483	-4.4919586
GM14484	-2.5564337
GM14486	-4.467004
GM14488	-2.7587452
GM14496	-4.106476
GM14499	-4.2966795
GM14634	-3.3450418
GM14725	-3.5252352

GM14743	-2.5516608
GM14762	-1.3213811
GM14851	-8.741274
GM14920	-4.95957
GM15023	-5.8333983
GM15085	-2.5632908
GM15104	-4.78306
GM15130	-3.2762086
GM15217	-1.5135441
GM15232	-2.058841
GM1527	-5.2920737
GM15292	-3.5421236
GM15299	-6.8341055
GM15401	-2.8530717
GM15411	-4.5224886
GM15412	-4.8444843
GM15413	-3.957052
GM15417	-1.9415472
GM15441	-3.4182029
GM15478	-4.2037354
GM15482	-2.1972752
GM15498	-9.008722
GM15506	-2.9305727
GM15514	-5.290999
GM1553	-2.4811535
GM156	-2.980731
GM15612	-4.743779
GM15628	-3.6602921
GM1564	-2.8104568
GM15762	-4.2292356
GM15821	-1.1706266
GM15850	-1.509016
GM1587	-2.2828941
GM1604B	-1.9274435
GM1631	-3.0381565
GM16367	-1.7607527
GM16390	-2.711967
GM16428	-5.520916
GM16441	-1.6954715
GM16462	-5.043065
GM16486	-2.9689405
GM16488	-4.9278173
GM16489	-3.9628043
GM16491	-2.0787203
GM16497	-3.3365543

GM16498	-4.6011662
GM16499	-3.213019
GM16505	-2.269846
GM16508	-3.253023
GM16527	-4.339819
GM16532	-5.201141
GM16686	-2.941895
GM16863	-3.4157956
GM16982	-2.5655372
GM17727	-6.0651617
GM17767	-4.266345
GM17769	-3.5165372
GM17783	-5.157304
GM19410	-5.058082
GM1965	-2.4217043
GM1968	-4.155869
GM1971	-1.9093976
GM1993	-4.4894586
GM2002	-1.4536445
GM2022	-2.555053
GM2027	-4.5391893
GM2042	-3.967278
GM20554	-1.0231619
GM20604	-3.521248
GM20611	-2.3098197
GM20740	-4.0576496
GM20747	-5.3367577
GM20754	-3.0020916
GM20758	-2.4067416
GM20783	-1.38766
GM20806	-4.6751194
GM20837	-6.649642
GM20865	-2.8241155
GM20867	-3.1210997
GM20877	-4.3303165
GM2090	-4.1967697
GM21064	-1.3443532
GM21276	-2.9966168
GM21292	-3.364309
GM2137	-3.2348123
GM21404	-1.6364667
GM21464	-5.949211
GM21498	-2.0327225
GM2155	-1.329993
GM21637	-4.2288465

GM2164	-3.352046
GM2176	-1.7602069
GM21951	-4.3948526
GM22	-0.7229285
GM2233	-3.6349182
GM2238	-2.5654066
GM2240	-5.0763893
GM2287	-3.1437726
GM2483	-3.5806708
GM2491	-5.287259
GM2545	-2.6537085
GM2560	-2.8221889
GM2582	-2.4272735
GM266	-2.74516
GM2670	-5.316619
GM2675	-3.4456036
GM2693	-2.2028325
GM2694	-1.0351171
GM2716	-3.7717192
GM2795	-1.8471215
GM2800	-4.7006836
GM2824	-0.862725
GM2837	-2.1062617
GM2927	-5.1912065
GM2990	-5.994297
GM3002	-6.4989176
GM3032	-2.3619323
GM3045	-3.0220127
GM3052	-2.6988337
GM3081	-1.962044
GM3086	-2.808846
GM3143	-3.0101802
GM318	-2.8331606
GM3227	-4.0275316
GM3230	-5.262446
GM3234	-1.4643676
GM3238	-3.1146624
GM3264	-2.6496687
GM3279	-1.9309146
GM3300	-1.1270769
GM3317	-3.1375365
GM3325	-1.9249976
GM3331	-2.4986598
GM3336	-5.9726753
GM3428	-3.7967272

GM3453	-3.2697246
GM3470	-2.5068662
GM3509	-3.7391524
GM3510	-4.3149796
GM3519	-1.4970384
GM355	-4.919246
GM3561	-2.0073104
GM3596	-4.344055
GM3598	-5.1591244
GM362	-5.0896044
GM3625	-2.1710868
GM364	-3.9493673
GM3646	-4.3178673
GM3669	-4.4468436
GM3696	-1.762136
GM3718	-2.3237538
GM3740	-4.100268
GM3763	-5.2002544
GM3772	-2.788282
GM3775	-3.5781286
GM3807	-2.357412
GM382	-5.4286785
GM3831	-3.1209774
GM3858	-2.8325906
GM3871	-1.5380261
GM3877	-2.820966
GM3881	-3.5118082
GM3896	-0.7906258
GM3907	-2.6109302
GM3916	-0.685097
GM3942	-2.8200865
GM3952	-2.074495
GM3961	-5.1811705
GM3970	-1.8934498
GM3985	-5.5060344
GM4022	-2.997722
GM4035	-2.3110096
GM4081	-3.1606429
GM4131	-5.8319464
GM4132	-2.2640805
GM4151	-6.395089
GM4200	-1.5416319
GM4201	-5.776016
GM4203	-5.7059937
GM4208	-3.220603

GM4211	-5.7689285
GM4221	-1.7803364
GM4224	-5.4408693
GM4230	-3.162068
GM4241	-2.760814
GM4251	-2.1386533
GM4278	-4.364106
GM4279	-3.6154268
GM4297	-3.2829235
GM4311	-1.9627421
GM4312	-3.0653048
GM4319	-2.434168
GM4340	-1.036411
GM4354	-2.3624032
GM436	-2.2511644
GM4371	-4.140049
GM438	-4.136548
GM4419	-4.572242
GM4430	-1.103893
GM4432	-5.5261574
GM4454	-4.5605116
GM4461	-1.78898
GM4466	-3.7293048
GM4489	-7.343181
GM4491	-2.030775
GM4532	-1.9801087
GM4566	-3.4403853
GM4567	-5.39943
GM4593	-0.9751401
GM4598	-6.7401266
GM46	-2.1247003
GM4606	-2.1385484
GM4632	-2.3741913
GM4657	-4.6751966
GM4665	-4.8558817
GM4668	-1.3428254
GM4719	-2.3561306
GM4736	-4.103694
GM4745	-6.4317083
GM4755	-6.548071
GM4758	-2.6481488
GM4763	-3.8333256
GM4776	-4.7895293
GM4791	-3.8415961
GM4792	-5.077054

GM4814	-1.0291874
GM4827	-2.2891254
GM4850	-2.3551834
GM4868	-7.0875816
GM4872	-6.420953
GM4906	-0.98805
GM4922	-3.6758633
GM4937	-2.9219174
GM4975	-4.543335
GM4981	-2.745389
GM4994	-5.5120544
GM5065	-2.4084272
GM5082	-1.4566424
GM5083	-4.061184
GM5084	-4.4737387
GM5086	-2.388517
GM5087	-1.5191171
GM5089	-1.9556704
GM5091	-2.7414079
GM5094	-3.3578172
GM5095	-3.1189516
GM5099	-3.4375467
GM5105	-1.7083774
GM5106	-1.9714289
GM5108	-4.559951
GM5111	-1.1239297
GM5118	-3.645193
GM5122	-5.183969
GM5124	-5.8686833
GM5127	-6.412524
GM5129	-3.1229453
GM5134	-6.780183
GM5144	-4.181238
GM5148	-2.9588082
GM5155	-4.529773
GM5166	-3.6177444
GM5174	-4.163549
GM5215	-1.1742916
GM5258	-2.9600098
GM5294	-4.258573
GM5296	-1.9627197
GM53	-5.883404
GM5331	-3.4702854
GM5334	-4.155544
GM534	-2.6262276

GM5346	-2.604248
GM536	-4.3070827
GM5409	-2.9482815
GM5415	-3.0595734
GM5420	-2.0159352
GM5434	-2.404111
GM5441	-1.934272
GM5447	-3.466525
GM5458	-2.8181608
GM5460	-2.3148904
GM5468	-5.198654
GM5475	-1.2023327
GM5476	-5.3070865
GM5477	-4.600315
GM5485	-4.413928
GM5493	-3.8175044
GM550	-6.4243073
GM553	-6.2738686
GM5532	-6.457628
GM5535	-2.8557763
GM5538	-2.9123337
GM5553	-1.4640393
GM5563	-6.1654644
GM5570	-3.4846795
GM5585	-2.7361202
GM5589	-4.3512344
GM5590	-2.202419
GM5591	-3.7716231
GM5592	-2.3861804
GM5600	-2.9189727
GM5606	-6.423518
GM5607	-4.0513067
GM5608	-2.0354455
GM5615	-2.8339367
GM5622	-3.7644358
GM5627	-5.4101143
GM5635	-2.7014446
GM5636	-3.7330227
GM5665	-1.8556783
GM567	-2.2318397
GM568	-4.9025464
GM5709	-3.1493328
GM572	-4.724631
GM5724	-2.960693
GM5737	-0.9118607

GM5751	-1.9033835
GM5755	-1.91379
GM5771	-3.4282405
GM5796	-2.3192203
GM5800	-4.544648
GM5820	-2.7580044
GM5849	-1.803539
GM5860	-5.472963
GM5862	-2.3804343
GM5868	-4.140953
GM5893	-3.6362605
GM590	-4.4914575
GM5901	-2.050389
GM5907	-2.073978
GM5916	-4.1551332
GM5922	-1.1259053
GM5925	-3.6266406
GM5932	-1.8202174
GM5934	-2.2634466
GM5938	-3.946476
GM595	-4.345859
GM5953	-4.420869
GM5958	-3.5542336
GM597	-2.8160777
GM6040	-2.870884
GM6042	-3.90216
GM6086	-2.4139845
GM6093	-3.4924488
GM6117	-6.99099
GM614	-1.8213613
GM6145	-3.9092312
GM6150	-2.9053006
GM6219	-4.0925207
GM6225	-2.3135805
GM6249	-1.6368957
GM6260	-3.6268196
GM6268	-3.3571959
GM6277	-3.004029
GM6287	-3.4873047
GM6288	-2.2201412
GM6289	-4.3091187
GM6307	-5.722182
GM6329	-2.7748814
GM6360	-3.6555865
GM6370	-3.578156

GM6406	-2.3505726
GM6416	-3.9296625
GM6436	-1.8651576
GM6460	-2.5649562
GM6471	-3.3923967
GM6537	-3.682403
GM6556	-3.3075402
GM6559	-2.6374598
GM6566	-4.5533266
GM6590	-4.33539
GM6600	-3.2734542
GM6602	-2.5033607
GM6614	-6.7486477
GM6639	-4.7439966
GM6653	-3.2585971
GM6713	-3.764016
GM6729	-1.3617623
GM6760	-4.9016438
GM6787	-4.894424
GM6792	-3.0420237
GM6818	-6.032345
GM6846	-3.114413
GM6890	-2.32112
GM6927	-3.9173322
GM6936	-1.9113142
GM694	-5.5865135
GM6943	-4.7352333
GM6958	-3.4707808
GM6961	-2.1186323
GM6999	-1.607106
GM7056	-3.1690068
GM7104	-3.6138144
GM7133	-3.9737132
GM7134	-4.4100223
GM7135	-2.0132651
GM715	-2.8614366
GM7157	-6.1348114
GM7167	-10.961824
GM725	-1.432785
GM7257	-5.323002
GM7271	-3.2165847
GM7276	-4.021352
GM7285	-2.5289433
GM732	-5.738241
GM7325	-2.579227

GM7444	-3.8612845
GM7457	-3.6069024
GM7467	-2.5559316
GM7480	-3.2543218
GM7489	-4.729655
GM7534	-6.8397846
GM7538	-3.8479214
GM7546	-4.6487303
GM7562	-3.7753265
GM7596	-5.2025146
GM7616	-3.4400449
GM7628	-2.9795659
GM7644	-2.583038
GM765	-2.2712262
GM766	-4.3547144
GM7714	-5.7949476
GM773	-2.789125
GM7789	-3.6324253
GM7827	-2.5221841
GM7849	-6.394404
GM7894	-4.7695694
GM7968	-1.1945896
GM7972	-4.953389
GM7978	-5.869553
GM7980	-4.7153893
GM8013	-2.811759
GM805	-3.933032
GM806	-3.8237927
GM8094	-4.2203918
GM813	-2.2860804
GM8140	-3.222237
GM8149	-4.9234753
GM815	-2.8120651
GM8165	-3.1567774
GM8212	-3.235177
GM826	-3.3191676
GM8273	-1.4945796
GM8298	-1.7119136
GM8300	-3.4960494
GM833	-1.3113885
GM8359	-2.066424
GM8439	-4.5259714
GM8479	-1.7076583
GM8505	-3.3301525
GM8579	-3.9187138

GM8630	-2.5733423
GM8633	-2.1189415
GM8672	-5.067336
GM8674	-6.3598385
GM8700	-1.7407601
GM8702	-3.7822838
GM8765	-1.49844
GM8787	-1.66994
GM8817	-4.5155096
GM884	-5.0681486
GM904	-4.759671
GM9047	-2.8473022
GM9054	-1.7718267
GM9059	-2.475089
GM9099	-2.1303782
GM9112	-3.1790934
GM9125	-2.920663
GM9258	-1.882931
GM9325	-4.428261
GM9376	-6.160387
GM94	-1.832202
GM9465	-3.1146576
GM9502	-1.2479038
GM9513	-4.609151
GM9519	-2.5545416
GM9548	-4.852371
GM9558	-2.290281
GM9564	-3.2270758
GM9573	-4.99974
GM960	-4.0344744
GM973	-3.116269
GM9753	-2.8615723
GM9758	-1.5322876
GM9767	-5.3585415
GM9776	-2.882756
GM9777	-3.931867
GM9779	-2.4350607
GM9789	-4.800221
GM9801	-3.096896
GM9804	-1.7445147
GM9831	-3.6619148
GM9839	-2.3218794
GM9860	-4.4523354
GM9866	-4.8530865
GM9868	-1.1075916

GM9869	-4.646158
GM9871	-4.9180555
GM9872	-2.9584527
GM9873	-4.801881
GM9876	-2.5654223
GM9878	-3.5015154
GM9886	-3.3134243
GM9902	-4.6177945
GM9910	-0.7951014
GM9916	-0.7842708
GM9918	-3.9227757
GM9919	-3.7128417
GM9921	-3.168201
GM9928	-5.5308485
GM9937	-6.116662
GM9946	-1.9621024
GM9951	-5.086642
GM9958	-2.5884793
GM9959	-2.8954618
GM996	-3.3809254
GM9960	-2.8361666
GM9961	-3.2460463
GM9962	-6.965482
GM9963	-1.9290078
GM9973	-4.2757115
GM9979	-2.6813498
GM9981	-2.549084
GM9984	-5.4608364
GML	-5.671089
GMNN	-2.9563673
GNA15	-2.0863395
GNAT3	-3.304484
GNAZ	-3.269711
GNB3	-1.0047245
GNG3	-2.9110386
GNG7	-0.6719136
GNRHR	-4.6265445
GON4L	-2.968306
GOT1L1	-2.234766
GP2	-3.3204756
GP6	-1.7111275
GPAT2	-1.5476191
GPBAR1	-4.8382034
GPC2	-3.6911335
GPC5	-2.6519856

GPC6	-2.0527403
GPER1	-7.6600256
GPR101	-4.4681544
GPR111	-5.6436806
GPR112	-3.0864
GPR115	-6.570714
GPR119	-1.8337781
GPR12	-2.0955372
GPR123	-5.938904
GPR128	-2.310864
GPR135	-5.6611958
GPR137C	-4.0191607
GPR139	-2.6929016
GPR142	-4.3416176
GPR143	-3.5256362
GPR149	-1.7004824
GPR150	-1.199167
GPR151	-2.6280746
GPR152	-3.069269
GPR158	-3.4835107
GPR161	-3.797951
GPR162	-2.0915487
GPR165	-1.1330812
GPR17	-3.8257024
GPR183	-2.2170777
GPR21	-4.454313
GPR27	-3.4261158
GPR35	-2.7366896
GPR37	-2.6680849
GPR37L1	-4.120364
GPR45	-2.5651524
GPR50	-2.862615
GPR55	-3.5011413
GPR61	-5.8186817
GPR63	-4.0010467
GPR75	-4.253861
GPRC5D	-1.6728163
GPRIN2	-2.641259
GPX5	-2.1682415
GPX6	-4.406903
GRB10	-1.6471591
GRHL3	-2.9232044
GRIA2	-2.6831036
GRID1	-2.5596495
GRID2	-4.0667963

GRIFIN	-3.7075944
GRIK1	-2.1860979
GRIK2	-2.6825025
GRIK3	-2.3116171
GRIK4	-3.6008923
GRIN1	-1.5364654
GRIN2A	-2.9443612
GRIN2B	-2.9967713
GRIN2D	-3.7254972
GRIN3A	-5.086169
GRIN3B	-3.0638068
GRIP1OS1	-2.7083144
GRIP2	-4.5259843
GRK1	-3.7409444
GRK4	-2.0059934
GRM1	-3.1952517
GRM4	-4.1602983
GRM5	-1.6814337
GRM7	-1.8718097
GRM8	-1.6919823
GRPR	-4.9666004
GRXCR1	-2.2321417
GRXCR2	-3.9862838
GSC	-1.6225185
GSC2	-3.1407733
GSDMA2	-3.8019953
GSDMA3	-4.4102907
GSDMC4	-5.222577
GSDMCL1	-2.7632737
GSDMCL2	-7.2210784
GSDMCL-PS	-1.7806344
GSG1L	-5.0406523
GSTT4	-0.7829077
GSX2	-4.241929
GTF2A1L	-3.3586504
GTPBP3	-1.819308
GUCY1B2	-2.6322403
GUCY2E	-3.6438942
GUCY2F	-4.401269
GUCY2G	-2.0824099
GUF1	-1.5950742
GYKL1	-5.7711344
GZMD	-2.2905974
GZME	-3.8747725
GZMG	-1.8528752

H1FNT	-1.7178168
H1FOO	-4.275447
H1FX	-4.3072414
H2AFB1	-3.0758657
H2AFB2	-4.902452
H2BFM	-1.463515
H2-DMA	-5.3222623
H2-M1	-4.1542215
H2-M10.2	-3.6921062
H2-M10.3	-3.1768317
H2-M5	-2.107426
H2-M9	-2.962242
H2-T18	-3.3959649
H2-T3	-3.0290623
HAND1	-1.7396023
HAPLN1	-1.9351606
HAPLN2	-1.8892238
HAPLN3	-2.6508756
HAS2OS	-3.065279
HAS3	-4.7130575
HBA-X	-3.033917
HBB-BH2	-4.885604
HBB-Y	-1.8756795
HBQ1A	-4.2914457
HCAR1	-1.6888347
HCN3	-5.30391
HCN4	-3.3316214
HCRT	-4.663028
HCRTR1	-1.3816075
HCRTR2	-4.3205833
HDAC6	-1.176353
HDGFL1	-7.4024715
HDHD1A	-4.9242306
HEATR9	-5.7652235
HEMT1	-2.7167137
HENMT1	-2.24131
HEPHL1	-5.547655
HES2	-2.2273307
HES3	-3.2244344
HES7	-1.0285785
HFM1	-3.205665
HHIPL1	-3.2931604
HHIPL2	-1.3344214
HHLA1	-5.8022094
HIGD1C	-3.8116503

HILS1	-5.037891
HIST1H1D	-3.0766723
HIST1H1T	-7.20014
HIST1H2BB	-1.4475064
HIST1H2BP	-4.011806
HIST1H3I	-3.2393596
HIST2H2AB	-1.4214487
HIST3H2BB-P	-2.662957
HIVEP3	-4.3380265
HMBOX1	-1.063107
HMGB4	-5.870391
HMGB4OS	-2.3352401
HMX1	-3.283226
HMX2	-5.972131
HNF4G	-3.4515846
HOMER3	-0.7885544
HORMAD1	-2.5692172
HOTTIP	-1.8836195
HOXA10	-2.7066226
HOXA11	-2.2320042
HOXA11OS	-2.3882475
HOXA13	-6.0638366
HOXA6	-4.4786587
HOXB8	-2.5228868
HOXB9	-3.881371
HOXC10	-7.0252414
HOXC11	-3.7412202
HOXC12	-3.506839
HOXC4	-3.7307985
HOXC5	-5.232769
HOXC6	-3.7535946
HOXC8	-2.6273975
HOXC9	-3.1859994
HOXD1	-4.0210266
HOXD10	-6.019288
HOXD11	-3.2266612
HOXD13	-2.6327307
HOXD3	-4.0712204
HOXD3OS1	-4.9795794
HOXD4	-2.512275
HOXD8	-3.2934718
HOXD9	-2.311613
HPCAL4	-4.838827
HPDL	-2.9073546
HPSE2	-2.9877324

HRASLS5	-2.5359747
HRH1	-2.1954112
HRH3	-3.4589694
HRK	-3.66172
HRNR	-4.0818443
HS3ST2	-3.4369917
HS3ST3A1	-1.4572501
HS3ST3B1	-2.325528
HS3ST4	-3.0312355
HS3ST5	-2.489364
HS6ST3	-1.3265235
HSD11B2	-2.5523665
HSD17B1	-2.6337192
HSD3B4	-3.3145654
HSF2BP	-2.9634662
HSF3	-3.8138175
HSF4	-1.7980208
HSFY2	-4.7743235
HSPB3	-3.3852186
HSPB9	-2.646533
HTR1A	-4.298956
HTR1F	-4.9929543
HTR2A	-6.457646
HTR3B	-4.29214
HTR4	-3.0307105
HTR5A	-1.3587122
HTR6	-3.4141958
HTRA3	-2.3491712
HYAL5	-0.8199496
HYAL6	-4.514165
IBSP	-4.1398263
ICAM5	-2.5609574
IDI2	-5.8142347
IFLTD1	-3.8340676
IFNA11	-5.422912
IFNA12	-2.653967
IFNA13	-4.1656275
IFNA14	-2.4879448
IFNA16	-4.3426256
IFNA4	-3.650927
IFNAB	-1.9335701
IFNB1	-1.15639
IFNE	-1.2513981
IFNK	-2.1920085
IFNL3	-4.122205

IFT88OS	-3.4890337
IGBP1B	-5.1332746
IGDCC3	-2.2179708
IGF2OS	-2.2384953
IGFL3	-4.773981
IGFN1	-2.8840442
IGH-V3660	-4.240699
IGKV4-53	-2.4096582
IGKV4-55	-2.1512692
IGKV4-72	-3.072475
IGKV6-20	-1.7350099
IGLL1	-2.3171613
IGLON5	-2.2092383
IGSF21	-2.625145
IGSF9B	-0.957896
IHH	-3.733116
IIGP1B	-3.7436087
IKZF3	-1.0966187
IL12RB2	-1.3263693
IL13	-4.3620777
IL17B	-6.2627215
IL17C	-1.6814907
IL1BOS	-3.628491
IL1F10	-2.249038
IL1F5	-3.6485677
IL1RAPL1	-3.4766335
IL2	-1.4715028
IL22	-4.90552
IL24	-2.674055
IL25	-5.596991
IL3	-2.8395393
IL31	-4.6347756
IL31RA	-2.8403764
IL4	-2.4555616
IL5	-3.53581
IL5RA	-3.71017
IL9	-2.1009743
IL9R	-2.5235379
INA	-1.3759797
INS1	-2.1102016
INS2	-2.5561054
INSM1	-4.271064
INSM2	-2.7593222
INSRR	-1.3789923
INTS1	-0.9240294

INTS2	-0.6707408
INTS6	-1.7501409
IPW	-3.4336562
IQCF1	-3.336814
IQCF3	-5.6819735
IQCF4	-4.94003
IQCF5	-3.419416
IQCF6	-1.662852
IQCJ	-1.5039895
IRGC1	-3.8633733
IRS3	-3.124566
IRS4	-0.8772311
IRX3OS	-2.2908084
IRX4	-5.0738087
ISL1	-4.1922617
ISL2	-1.8846829
ISLR2	-2.3204925
ISM2	-6.170227
ISOC2B	-1.46894
ISPD	-1.8495994
ISX	-1.6328301
ITGA2B	-2.451355
ITGAD	-4.679532
ITGB8	-3.8211927
ITPR3OS	-2.426885
ITPRIPL1	-2.5768535
IVL	-5.198413
IZUMO1	-7.160513
IZUMO2	-6.074259
IZUMO3	-1.2437611
JADE3	-4.0966134
JAKMIP1	-3.4638968
JAKMIP2	-5.1249685
JAKMIP3	-5.681075
JAZF1	-3.4429064
JMJD7	-1.1429915
JPH3	-3.3420453
JPH4	-5.837138
JUNOS	-5.4146223
JUP	-0.9868276
KANSL1L	-3.8854048
KAP	-5.7270174
KATNAL2	-4.631465
KBTBD12	-3.9570029
KBTBD13	-3.192463

KCNA10	-3.220358
KCNA2	-2.2868562
KCNA5	-2.9526558
KCNA6	-2.1908443
KCNAB1	-2.5407145
KCNAB2	-1.8732784
KCNAB3	-2.1991482
KCNB2	-4.2584653
KCNC1	-2.904389
KCNC3	-3.0091245
KCNC4	-2.5660846
KCND2	-3.760169
KCND3OS	-2.6273394
KCNF1	-3.329451
KCNG1	-3.7829235
KCNG2	-1.715251
KCNG3	-3.3317437
KCNG4	-2.7496881
KCNH1	-3.2291625
KCNH5	-2.1776476
KCNH6	-2.8542135
KCNH7	-3.1736505
KCNH8	-5.1854415
KCNIP1	-1.4124732
KCNIP2	-6.586464
KCNJ1	-2.8036265
KCNJ11	-3.6886978
KCNJ14	-2.0333903
KCNJ3	-2.8984659
KCNJ6	-1.4846728
KCNJ9	-5.425251
KCNK10	-3.7551594
KCNK15	-5.0808573
KCNK16	-3.4496439
KCNK18	-5.4681134
KCNK4	-1.2837577
KCNK7	-3.6127756
KCNMA1	-3.7275898
KCNMB3	-4.171177
KCNMB4OS2	-3.402532
KCNN1	-5.7257414
KCNQ2	-2.6996517
KCNQ3	-3.3689427
KCNS1	-4.0881557
KCNS2	-2.1932454

KCNT1	-3.8574462
KCNU1	-2.6143441
KCNV1	-1.7445967
KCTD16	-4.3127594
KCTD7	-7.426784
KDM2A	-1.0316973
KHDC1B	-2.7654347
KHDC1C	-6.7129025
KHDRBS2	-2.1293766
KIF13B	-3.032969
KIF14	-2.6480513
KIF17	-2.3966863
KIF24	-2.1922455
KIF26B	-1.633956
KIF2A	-2.0138884
KIF2B	-3.9998496
KIF4-PS	-3.3291388
KIF5B	-0.8725309
KIF7	-3.4362028
KIR3DL1	-4.0319304
KIR3DL2	-1.4599838
KIRREL2	-6.91535
KIS2	-2.1634328
KISS1	-4.517874
KL	-1.3072832
KLF14	-2.8784385
KLF17	-5.2435894
KLHDC7B	-2.1364818
KLHDC8A	-1.9830451
KLHL1	-8.759438
KLHL10	-3.404248
KLHL14	-2.0549984
KLHL23	-3.281408
KLHL3	-5.4943757
KLHL30	-3.9401712
KLHL31	-4.325609
KLHL34	-4.9216146
KLHL40	-5.585018
KLK10	-3.0640957
KLK12	-3.2098908
KLK14	-2.3455427
KLK15	-3.4270306
KLK1B1	-5.176775
KLK1B11	-3.6140113
KLK1B21	-1.0798092

KLK1B7-PS	-2.847387
KLK1B9	-0.916512
KLK4	-2.3577323
KLK5	-4.777045
KLK6	-4.2523923
KLK7	-2.306225
KLK9	-1.4415364
KLRA10	-4.316268
KLRA19	-1.4962394
KLRB1	-3.9195824
KLRB1-PS1	-5.3733506
KLRG2	-2.8966868
KMT2D	-0.9308987
KNCN	-2.1890552
KPNA7	-3.2359552
KPRP	-5.594097
KREMEN2	-4.6620617
KRT1	-1.9375443
KRT10	-1.353467
KRT12	-2.3439145
KRT13	-4.026352
KRT14	-1.2128761
KRT2	-4.4508467
KRT20	-5.918381
KRT24	-6.094632
KRT25	-1.618021
KRT26	-5.7479277
KRT27	-7.99304
KRT28	-3.0286326
KRT32	-2.0331569
KRT33A	-5.4470367
KRT33B	-2.7893672
KRT34	-2.9728367
KRT35	-3.9616334
KRT36	-3.774659
KRT39	-5.000224
KRT4	-6.8684893
KRT40	-3.1896243
KRT5	-4.1964445
KRT6A	-4.3294487
KRT6B	-4.574517
KRT71	-2.2534037
KRT72	-4.4676266
KRT74	-5.794335
KRT76	-3.174762

KRT77	-2.0001702
KRT82	-3.8679676
KRT84	-2.3310273
KRT9	-4.499755
KRTAP10-4	-2.6835651
KRTAP1-3	-2.5949388
KRTAP1-4	-1.4834731
KRTAP15	-5.247716
KRTAP16-1	-3.7866416
KRTAP16-3	-1.8750658
KRTAP19-5	-2.5568492
KRTAP19-9B	-2.7890432
KRTAP21-1	-3.7395506
KRTAP2-4	-3.3082924
KRTAP24-1	-5.392658
KRTAP26-1	-3.6388724
KRTAP31-2	-6.2275105
KRTAP4-1	-4.277997
KRTAP4-2	-3.2917204
KRTAP4-7	-3.787697
KRTAP4-9	-2.7691176
KRTAP5-5	-1.8858116
KRTAP6-2	-2.119956
KRTAP6-5	-3.3706799
KRTAP8-1	-4.568946
KRTAP9-3	-3.3744729
KRTDAP	-4.8046327
KSR2	-2.3546717
KY	-5.2681484
L1CAM	-2.854489
L3MBTL1	-2.8726282
L3MBTL2	-2.9680605
L3MBTL4	-3.1934323
LACTBL1	-4.156348
LAD1	-1.3517416
LALBA	-0.9794095
LAMP5	-1.9003625
LAO1	-2.5465283
LAX1	-3.6916645
LCE1A1	-2.8552997
LCE1C	-2.2057247
LCE1E	-2.3280323
LCE1G	-4.9409533
LCE1H	-4.0313835
LCE1I	-4.6022034

LCE1J	-2.618264
LCE1K	-2.5353265
LCE1L	-3.787541
LCE1M	-3.306523
LCE3C	-4.1594725
LCE3D	-1.6423578
LCE3F	-2.5858212
LCN10	-6.531321
LCN11	-4.488651
LCN3	-5.676535
LCN5	-4.2220316
LCN9	-2.7560103
LCT	-3.1195202
LCTL	-4.0608783
LDHAL6B	-1.1104186
LDHC	-3.9788878
LEFTY1	-2.0959074
LELP1	-2.0081122
LEMD1	-2.5455735
LEP	-2.4927282
LGALS7	-3.984495
LGI1	-2.7154508
LHB	-2.1102345
LHFPL1	-4.950965
LHX1	-4.6933727
LHX4	-3.7426136
LHX8	-1.7305572
LHX9	-2.5549347
LIF	-4.5766582
LIM2	-5.603474
LIMK2	-1.2264769
LIN28B	-3.4825475
LIN7B	-3.6914232
LINGO3	-4.5548525
LIPM	-1.8633459
LIPN	-3.3274133
LIPO4	-5.26951
LIX1	-4.308586
LKAAEAR1	-4.8570127
LMAN1L	-2.0297077
LMO3	-1.6406498
LMOD1	-3.0069635
LMOD2	-2.2089942
LMX1A	-1.9857285
LMX1B	-3.3972836

LOC1000481	-2.5918853
LOC1005025	-2.5978274
LOC1005030	-5.5242243
LOC1008621	-4.338274
LOC1008624	-2.3899093
LOC1010556	-4.8261967
LOC1010557	-3.634882
LOC1010558	-2.5313375
LOC1010559	-4.649659
LOC1010559	-4.4355373
LOC1010559	-3.7477102
LOC1010559	-3.177519
LOC1010560	-3.369617
LOC1010560	-3.156851
LOC1010562	-1.2049153
LOC1010562	-5.715647
LOC1026318	-5.5824604
LOC1026321	-2.1954024
LOC1026322	-3.3871145
LOC1026324	-4.336919
LOC1026328	-3.0986328
LOC1026330	-3.5647562
LOC1026331	-4.3127995
LOC1026333	-3.2348025
LOC1026337	-5.965897
LOC1026338	-4.520099
LOC1026340	-1.8399506
LOC1026341	-3.1605015
LOC1026344	-1.1449075
LOC1026345	-3.7211027
LOC1026345	-3.5422134
LOC1026346	-4.038794
LOC1026352	-5.5394697
LOC1026353	-3.5684052
LOC1026354	-2.6053162
LOC1026355	-5.356573
LOC1026356	-3.7291496
LOC1026356	-2.7266483
LOC1026360	-5.8620977
LOC1026361	-2.6188004
LOC1026362	-1.8649662
LOC1026367	-1.7463
LOC1026367	-3.1098692
LOC1026369	-4.5903854
LOC1026369	-0.8360066

LOC1026370	-4.094926
LOC1026374	-1.310698
LOC1026377	-2.89603
LOC1026377	-2.1133256
LOC1026379	-3.6578608
LOC1026379	-2.2005384
LOC1026382	-2.7942207
LOC1026386	-5.02042
LOC1026387	-4.4621716
LOC1026389	-2.6006322
LOC1026392	-1.4110506
LOC1026396	-5.312641
LOC1026396	-1.6884685
LOC1026397	-3.6424763
LOC1026398	-5.8661237
LOC1026412	-2.2423503
LOC1026412	-5.100396
LOC1026413	-1.7302017
LOC1026413	-2.8819032
LOC1026416	-2.5795414
LOC1026416	-3.160191
LOC1026417	-4.60875
LOC1026418	-2.2519717
LOC1026420	-5.1147566
LOC1026420	-3.705585
LOC1026420	-4.377096
LOC1026421	-3.8898063
LOC1026422	-4.8493342
LOC1026423	-2.7334924
LOC1026424	-4.1475916
LOC1026424	-4.2822976
LOC1026425	-1.5051396
LOC1026426	-4.9622936
LOC1026427	-0.8210113
LOC1026427	-1.4523141
LOC1026429	-1.5909655
LOC1026431	-6.0854816
LOC1026431	-4.539545
LOC171588	-3.149933
LONRF2	-1.5268996
LOR	-2.1946437
LOXHD1	-4.3951135
LPAR4	-5.0638795
LPAR5	-1.3103969
LPCAT2B	-4.690996

LRCH3	-2.9307292
LRFN1	-0.9407802
LRFN5	-4.0385947
LRP1B	-4.5561066
LRR1	-2.7685928
LRRC15	-2.192705
LRRC16B	-3.6668634
LRRC18	-2.629334
LRRC29	-3.3383658
LRRC30	-3.0084267
LRRC39	-2.8143036
LRRC4C	-2.6854224
LRRC55	-2.5158694
LRRC57	-0.8346856
LRRC63	-1.6493678
LRRC66	-2.0100894
LRRC69	-1.8266225
LRRC7	-2.4550855
LRRC74	-7.1988797
LRRC9	-3.1192105
LRRD1	-2.7883456
LRRIQ4	-6.359236
LRRN2	-3.4896548
LRRTM1	-3.4696634
LRRTM2	-4.7368183
LRRTM3	-2.3814077
LRTM1	-3.9360802
LTA	-3.227303
LUZP2	-3.2537394
LY6G5C	-2.4849052
LY6G6F	-3.2172983
LY6H	-3.0574567
LY75	-1.8872881
LYG1	-2.4854293
LYG2	-5.465376
LYPD3	-2.8647718
LYPD6	-1.8696692
LYPD8	-5.9833755
LYZL1	-4.8114805
LYZL4	-3.458561
LYZL6	-2.4113455
MAB21L3	-1.228035
MACROD2OS	-5.1884103
MAD2L1	-1.2737751
MADCAM1	-1.685276

MAEL	-5.735488
MAGEA1	-4.7758064
MAGEA10	-5.5531945
MAGEA3	-2.8955128
MAGEA4	-2.2842124
MAGEA5	-6.969707
MAGEA6	-2.633354
MAGEB1	-3.0822756
MAGEB16-PS	-3.5773487
MAGEB18	-2.5573907
MAGEB3	-3.3453026
MAGEB4	-2.0604653
MAGEE1	-1.1909904
MAGEE2	-4.2821097
MAGEL2	-3.089499
MAGI2	-2.9173193
MAMDC4	-1.4502852
MAMLD1	-4.2589808
MAN2C1OS	-0.9284124
MANEAL	-4.627206
MAP2K6	-1.8589709
MAP2K7	-2.1896813
MAP3K10	-2.9099264
MAP3K12	-1.5994437
MAP3K13	-1.2672482
MAP3K7CL	-3.922086
MAP7D2	-3.2660842
MAPK8	-9.027595
MAPK8IP2	-2.693372
MAPKBP1	-2.0098932
MAST1	-2.5717058
MATK	-1.6098495
MATN3	-2.546807
MBD3L1	-2.4634707
MBOAT4	-1.7453752
MC1R	-1.8706594
MC4R	-1.5309014
MCHR1	-4.247959
MCM8	-1.51003
MCPT1	-2.9844642
MCPT2	-3.822451
MCPT9	-7.514761
MCPT-PS1	-4.229372
MDGA2	-3.5288944
MDK	-0.9798765

MED18	-1.0545766
MEF2B	-2.733096
MEGF10	-2.856425
MEGF11	-2.5346692
MEI1	-5.1086235
MEI4	-2.9251502
MEIS3	-1.2453597
MEP1A	-1.817694
MEP1B	-2.6527255
METTL11B	-5.6761374
METTL13	-4.6400256
METTL21E	-2.7702086
METTL7A1	-1.3349617
METTL8	-2.1243274
MEX3A	-3.898879
MFSD9	-2.389096
MGAT4C	-5.0565495
MGAT5	-0.8662412
MICAL2	-1.0030017
MICALCL	-3.5468295
MICU3	-1.959852
MID2	-2.3978617
MIP	-3.2762887
MIPOL1	-1.8892789
MIRG	-3.1610887
MISP	-4.655727
MIXL1	-3.5849125
MKNK1	-1.5002728
MKRN2	-1.0156107
MMP10	-4.370723
MMP16	-2.346756
MMP1B	-4.598752
MMP21	-4.121434
MMP24	-1.3726776
MMP27	-0.8985679
MMP28	-3.304237
MMS19	-1.064462
MOG	-0.6696463
MOGAT1	-2.7006927
MORC1	-1.1351688
MORC2B	-3.4252305
MORN2	-0.9234338
MOS	-4.02666
MOV10L1	-3.1164143
MOXD2	-3.0937948

MPHOSPH9	-2.9288704
MPP3	-1.6821375
MPP4	-3.2204225
MPTX1	-1.7902944
MRGPRA1	-3.775188
MRGPRA4	-2.0637608
MRGPRA8	-3.2755122
MRGPRB1	-2.727656
MRGPRB13	-1.6694157
MRGPRB3	-3.0889492
MRGPRB4	-3.0375621
MRGPRE	-2.0135314
MRGPRH	-3.899016
MRGPRX1	-3.0410914
MRGPRX2	-2.2785842
MRO	-2.5492575
MROH2A	-3.1962862
MROH2B	-1.7499673
MROH7	-5.513009
MROH8	-1.6306641
MROH9	-2.4980278
MRPL28	-5.393664
MS4A10	-4.5368257
MS4A12	-2.1591678
MS4A13	-2.6323404
MS4A14	-2.1309433
MS4A18	-2.4723742
MS4A5	-1.9871836
MSGN1	-4.625385
MSH4	-2.3523133
MSH5	-2.8778543
MSLNL	-4.4913836
MSMB	-4.3231754
MSMP	-3.7566605
MSX2	-1.326273
MSX3	-3.6796117
MT4	-0.8495805
MTAG2	-2.25491
MTERF1B	-0.9420099
MTL5	-1.7746758
MTNR1A	-1.8037832
MTNR1B	-5.256503
MTRR	-1.1074636
MUC15	-2.4208572
MUC19	-2.9614828

MUC5AC	-1.7289014
MUC6	-3.0186346
MUCL1	-5.142537
MUP6	-4.1360283
MURC	-3.5713203
MUSK	-1.0005825
MXI1	-2.711308
MYBPC1	-3.1717448
MYBPH	-3.4566126
MYCS	-3.480309
MYCT1	-2.8041158
MYH1	-5.290783
MYH13	-4.48386
MYH15	-5.495089
MYH2	-0.8346567
MYH4	-4.861977
MYL10	-5.1563077
MYL2	-5.955183
MYL6B	-1.1940727
MYLK4	-1.5168912
MYLPF	-2.3126352
MYO15	-4.0545883
MYO16	-3.2620502
MYO18B	-1.8080754
MYO1A	-2.9918933
MYO3A	-4.290766
MYO3B	-2.0746896
MYO7A	-0.9350793
MYO7B	-2.521913
MYOC	-3.5920517
MYOD1	-3.3152626
MYOG	-2.609749
MYOM3	-1.394969
MYOT	-9.734974
MYOZ1	-3.7018337
MYOZ3	-3.3205144
MYPN	-4.9683857
MYRFL	-2.2018726
MYT1L	-3.6745455
N4BP1	-0.9758856
N4BP2OS	-2.8097086
NAA11	-3.4246676
NAALADL1	-3.2249062
NAIF1	-2.5686088
NAIP7	-3.41514

NALCN	-3.7949057
NANOS2	-3.543546
NAP1L2	-1.1048834
NAP1L3	-0.8841124
NAP1L5	-2.7773757
NAT14	-1.5481796
NAT3	-3.506845
NAV3	-1.676553
NCAM2	-3.2308843
NCAN	-3.4217603
NCKAP5L	-3.63272
NCTC1	-1.5535033
NDC80	-4.669631
NDFIP2	-3.987218
NDP	-2.484322
NDST3	-5.325759
NDST4	-3.3703463
NDUFAF5	-1.2424033
NEB	-2.836904
NEFH	-3.0944037
NEFM	-4.46476
NEK10	-3.8179011
NELL1	-2.5328946
NEPN	-4.2268515
NESPAS	-4.9021983
NETO1	-2.7337737
NETO2	-1.8036807
NEU2	-1.6696517
NEU4	-2.67321
NEUROD4	-3.0510676
NEUROG1	-1.2900763
NEUROG3	-3.318427
NGB	-3.383409
NHLH1	-0.7452915
NHLH2	-1.880444
NIM1K	-3.8822238
NINJ2	-1.2596014
NIPAL4	-0.9452825
NIPSNAP3A	-1.6906681
NKAIN1	-0.9612713
NKAIN2	-2.5632763
NKAIN3	-1.4287271
NKPD1	-5.645807
NKX1-1	-2.5803478
NKX1-2	-4.364814

NKX2-3	-4.849162
NKX2-5	-1.6884961
NKX2-6	-5.318774
NKX2-9	-3.9286366
NKX3-1	-4.340516
NKX3-2	-7.1323395
NKX6-2	-2.209157
NKX6-3	-5.678098
NLE1	-2.9468246
NLGN1	-3.6439528
NLRP10	-4.4217367
NLRP14	-2.9053657
NLRP1B	-1.8543477
NLRP2	-3.6519916
NLRP4A	-3.35178
NLRP4B	-5.426713
NLRP4C	-6.5631433
NLRP4D	-4.490678
NLRP4E	-3.971736
NLRP4F	-5.2535105
NLRP4G	-4.5464153
NLRP5	-2.859994
NLRP5-PS	-3.4257011
NLRP9A	-3.7346995
NLRP9C	-1.6477242
NMBR	-3.7638829
NME8	-4.4510574
NMS	-1.1531699
NMU	-2.9391377
NMUR1	-4.5711184
NOBOX	-3.2214293
NOL3	-3.2803323
NOL4	-1.5157394
NOP9	-1.1351099
NOS1	-3.334188
NOTO	-4.5557714
NOTUMOS	-3.110425
NPAS1	-3.9318895
NPAS3	-2.4845345
NPAS4	-12.536355
NPAT	-2.173613
NPBWR1	-3.94304
NPC1L1	-1.5183241
NPFFR1	-3.5557578
NPFFR2	-3.473548

NPHS1	-3.3786922
NPM3	-1.3204911
NPM3-PS1	-2.307826
NPPB	-3.0482402
NPS	-1.413661
NPSR1	-3.5490937
NPTX2	-1.9741998
NPVF	-3.1686592
NPY2R	-4.466177
NPY4R	-3.2645524
NPY5R	-2.2146509
NPY6R	-1.5535171
NR0B1	-2.0299823
NR0B2	-3.276401
NR2E3	-1.7622294
NR3C2	-4.6653924
NRAP	-0.8857369
NRG2	-2.3539867
NRG3	-2.3238251
NRGN	-1.9505813
NRIP2	-3.2899325
NRK	-2.055793
NRL	-6.121304
NRP	-5.3800116
NRSN1	-2.5019841
NRXN3	-2.3252625
NSG2	-3.630865
NSUN7	-2.3438942
NT5C1A	-1.8486228
NT5C1B	-2.8340335
NTF5	-2.8525765
NTHL1	-2.6017535
NTN5	-3.0658937
NTNG1	-5.7127123
NTRK1	-6.1468897
NTSR1	-5.099345
NUAK1	-2.7935145
NUDT12OS	-4.293097
NUDT15	-1.8137763
NUDT17	-3.4282055
NUDT18	-2.269577
NUP62CL	-1.5795865
NXF2	-1.3338642
NXNL1	-1.4294021
NXPH1	-3.6413426

NYAP2	-4.972131
NYNRIN	-4.04105
OAS1D	-4.517184
OAS1E	-0.9729197
OAS1G	-0.8600166
OAS1H	-4.7283745
OAZ3	-7.6977386
OBOX1	-2.6054637
OBOX3	-3.7555666
OBOX5	-5.7390866
OBOX6	-2.0419168
OBP1A	-1.392004
OBP2B	-4.2004137
OCA2	-1.9256866
OCM	-3.4994066
ODAM	-3.120577
ODF1	-4.7644486
ODF3	-3.0284061
ODF3L2	-3.2171066
ODF4	-4.580792
OFCC1	-4.53906
OGDHL	-3.8518891
OIP5	-3.4027538
OIT1	-3.0525706
OLAH	-6.8091955
OLFM2	-1.527492
OLFM3	-4.1199703
OLFML1	-1.080867
OLFML2A	-1.6039386
OLFR1	-3.388219
OLFR10	-2.694504
OLFR100	-3.6063604
OLFR1000	-4.494857
OLFR1006	-4.40602
OLFR1009	-5.42854
OLFR101	-3.0054088
OLFR1010	-4.1235085
OLFR1012	-4.3266973
OLFR1013	-4.045413
OLFR1014	-3.5056472
OLFR1015	-4.22116
OLFR1016	-3.0810297
OLFR1018	-4.271831
OLFR1019	-5.131439
OLFR102	-3.239711

OLFR1023	-3.4416153
OLFR1024	-3.6880205
OLFR1026	-1.9611864
OLFR1028	-3.7717712
OLFR1029	-2.1653764
OLFR1030	-4.8893456
OLFR1031	-3.5205982
OLFR1032	-5.0693445
OLFR1033	-6.1651273
OLFR1036	-2.1646323
OLFR1039	-4.183452
OLFR1040	-5.6667414
OLFR1042	-5.598878
OLFR1043	-2.166436
OLFR1044	-2.9140136
OLFR1045	-3.4927156
OLFR1046	-5.4412403
OLFR1047	-2.01371
OLFR1049	-3.0276318
OLFR104-PS	-4.2912025
OLFR1051	-4.7657633
OLFR1052	-5.7224593
OLFR1054	-6.180791
OLFR1055	-6.8197412
OLFR1056	-4.433797
OLFR1057	-3.8900805
OLFR1058	-1.8028591
OLFR105-PS	-5.154659
OLFR1061	-4.985028
OLFR1062	-3.9674513
OLFR1065	-4.6577244
OLFR1066	-2.149032
OLFR107	-5.532036
OLFR108	-2.2536988
OLFR1082	-3.413617
OLFR1085	-3.6815493
OLFR1086	-4.000363
OLFR1089	-4.711958
OLFR109	-1.9884672
OLFR1090	-4.1439495
OLFR1093	-2.672543
OLFR1094	-1.1633673
OLFR1097	-3.284165
OLFR1099	-2.533349
OLFR11	-4.2489347

OLFR110	-6.568904
OLFR1100	-7.355258
OLFR1102	-3.607678
OLFR1106	-2.170474
OLFR1107	-5.877541
OLFR1109	-2.5239065
OLFR111	-4.880269
OLFR1110	-8.292561
OLFR1111	-6.3034964
OLFR1112	-4.8557863
OLFR1115	-2.2287908
OLFR1118	-1.2849667
OLFR112	-1.0831952
OLFR1120	-2.8690307
OLFR1122	-2.9111047
OLFR1123	-4.971882
OLFR1124	-5.878521
OLFR1128	-3.3349044
OLFR1129	-2.397124
OLFR113	-3.3090186
OLFR1130	-1.8744397
OLFR1135	-5.4444723
OLFR1136	-6.890385
OLFR1137	-5.6305885
OLFR1138	-4.473613
OLFR114	-4.7869377
OLFR1140	-6.357556
OLFR1141	-4.7443314
OLFR1145	-5.982619
OLFR1148	-1.0956347
OLFR115	-3.0388806
OLFR1151	-2.8156736
OLFR1152	-6.328397
OLFR1153	-4.6907487
OLFR1155	-4.377325
OLFR1156	-4.5095663
OLFR1157	-5.852304
OLFR1158	-4.9482813
OLFR1160	-2.1047404
OLFR1161	-1.904665
OLFR1162	-5.1471205
OLFR1164	-3.8929408
OLFR1168	-1.2296598
OLFR117	-2.1689372
OLFR1170	-4.7185817

OLFR1173	-6.089417
OLFR1176	-1.6340752
OLFR1178	-4.5473714
OLFR1179	-6.437972
OLFR118	-4.7212095
OLFR1180	-1.7152438
OLFR1181	-4.564623
OLFR1182	-2.948347
OLFR1183	-2.0335908
OLFR1184	-3.5195427
OLFR1186	-2.5739384
OLFR1189	-5.189066
OLFR1193	-6.3420544
OLFR1195	-2.6355953
OLFR1196	-2.604395
OLFR1199	-1.7288046
OLFR12	-2.169
OLFR120	-4.9717374
OLFR1200	-5.258831
OLFR1202	-6.176873
OLFR1205	-2.9753802
OLFR1206	-5.595895
OLFR1209	-1.1555583
OLFR121	-2.0221055
OLFR1214	-4.8828163
OLFR1215	-3.4551659
OLFR1216	-2.688924
OLFR1217	-3.5138874
OLFR122	-4.353279
OLFR1220	-3.8094053
OLFR1221	-1.0000277
OLFR1222	-2.215763
OLFR1223	-1.5814202
OLFR1226	-5.949319
OLFR1230	-1.5609608
OLFR1231	-4.686142
OLFR1232	-1.8795967
OLFR1233	-2.8363943
OLFR1234	-1.9178119
OLFR1238	-5.5584793
OLFR124	-4.4204035
OLFR1241	-2.991118
OLFR1243	-2.5407844
OLFR1245	-5.244704
OLFR1248	-4.985655

OLFR1250	-2.8894672
OLFR1251	-1.6905329
OLFR1252	-1.3408635
OLFR1254	-7.3193445
OLFR1255	-1.6958041
OLFR1256	-3.9760537
OLFR1257	-4.1929793
OLFR1258	-4.3032846
OLFR1259	-4.696792
OLFR126	-4.7095213
OLFR1261	-5.5733314
OLFR1262	-5.785922
OLFR1263	-3.5675945
OLFR1265	-3.9400225
OLFR1270	-3.3793151
OLFR1271	-4.4973736
OLFR1272	-4.897155
OLFR1273-PS	-1.636167
OLFR1275	-6.3350344
OLFR1276	-2.7882023
OLFR1277	-4.087457
OLFR1278	-5.0232687
OLFR1279	-3.3756182
OLFR1280	-3.388985
OLFR1285	-3.092447
OLFR1286	-5.164554
OLFR1287	-4.7364073
OLFR1288	-5.134206
OLFR1289	-6.141038
OLFR129	-7.3803062
OLFR1290	-2.4310906
OLFR1294	-2.7643032
OLFR1295	-2.0889034
OLFR1297	-4.8283005
OLFR13	-3.7016678
OLFR130	-3.914447
OLFR1300-PS	-4.480224
OLFR1302	-6.236945
OLFR1303	-4.6127014
OLFR1306	-2.6706738
OLFR1307	-2.190363
OLFR1308	-4.8453803
OLFR131	-4.8950334
OLFR1310	-3.2433088
OLFR1311	-6.3280973

OLFR1313	-6.0218296
OLFR1314	-5.5574493
OLFR1316	-2.9294395
OLFR1318	-3.1665165
OLFR132	-7.3513885
OLFR1320	-4.2847986
OLFR1321	-4.6411743
OLFR1322	-5.3284945
OLFR1323	-4.899122
OLFR1324	-3.1842089
OLFR1325	-4.938958
OLFR1328	-5.658992
OLFR1329	-2.2291818
OLFR133	-5.4720182
OLFR1330	-5.183936
OLFR1333	-9.698632
OLFR1335	-5.3824196
OLFR1336	-5.391307
OLFR1337	-4.7327166
OLFR1338	-3.975735
OLFR1339	-3.7143974
OLFR134	-1.9185731
OLFR1340	-4.4965153
OLFR1341	-3.4651046
OLFR1342	-5.884499
OLFR1344	-4.9921155
OLFR1346	-5.3200383
OLFR1347	-1.6408527
OLFR1348	-6.187012
OLFR135	-5.5281525
OLFR1351	-5.1473465
OLFR1352	-4.7467136
OLFR1354	-3.423376
OLFR1358	-3.5854547
OLFR136	-2.7790542
OLFR1360	-7.0919456
OLFR1361	-3.6763535
OLFR1362	-5.5808134
OLFR1364	-5.917015
OLFR1366	-2.041961
OLFR1367	-3.9543881
OLFR137	-4.8711023
OLFR1370	-3.5825465
OLFR1377	-4.1920314
OLFR1378	-5.680814

OLFR138	-3.3706717
OLFR1382	-2.9789805
OLFR1383	-3.8241224
OLFR1385	-4.891505
OLFR1386	-2.1926045
OLFR1387	-2.9300153
OLFR1388	-3.4516377
OLFR1389	-1.5117521
OLFR1390	-1.2213647
OLFR1393	-2.6331105
OLFR1394	-3.1204638
OLFR1395	-1.8267434
OLFR1396	-1.5744491
OLFR1404	-2.7990744
OLFR1406	-2.0604506
OLFR1411	-4.581085
OLFR1414	-5.452466
OLFR1415	-6.331277
OLFR1416	-1.2605841
OLFR1417	-1.6363263
OLFR1418	-5.3621507
OLFR1419	-2.758438
OLFR142	-3.8004456
OLFR1420	-4.259772
OLFR1423	-6.207452
OLFR1424	-3.9188254
OLFR1425	-4.494418
OLFR1426	-1.1222522
OLFR1427	-3.0970438
OLFR1433	-5.6314554
OLFR1436	-2.9609716
OLFR1440	-1.6845984
OLFR1441	-4.2080083
OLFR1442	-3.998749
OLFR1444	-3.0962467
OLFR1445	-5.1644826
OLFR1446	-3.359533
OLFR1448	-6.3916044
OLFR1449	-3.3841634
OLFR145	-3.5675647
OLFR1450	-3.3245025
OLFR1451	-7.5804114
OLFR1454	-6.907421
OLFR1458	-2.0226865
OLFR1459	-4.5984735

OLFR146	-4.1036015
OLFR1461	-6.1232176
OLFR1462	-3.7129884
OLFR1465	-5.1275682
OLFR147	-5.595417
OLFR1472	-2.7987332
OLFR1474	-4.512265
OLFR1475	-2.4831371
OLFR148	-2.333831
OLFR1490	-7.5262055
OLFR1491	-3.7981474
OLFR1494	-2.3955889
OLFR1496	-4.7052803
OLFR1497	-3.9935167
OLFR1499	-2.2893946
OLFR150	-4.9901605
OLFR1500	-6.1281366
OLFR1501	-4.7117734
OLFR1502	-4.037641
OLFR1504	-1.9327779
OLFR1507	-3.5701027
OLFR1508	-5.0468645
OLFR1509	-3.170713
OLFR1510	-1.275635
OLFR1511	-4.2678127
OLFR1513	-5.3504186
OLFR152	-5.4011755
OLFR1532-PS	-4.8858614
OLFR1535	-3.1900718
OLFR154	-2.1675603
OLFR155	-3.9723291
OLFR156	-4.006955
OLFR157	-3.5738645
OLFR160	-6.5053635
OLFR161	-8.19437
OLFR165	-1.7500129
OLFR166	-1.8592138
OLFR167	-3.421996
OLFR17	-5.6135077
OLFR170	-2.8913028
OLFR171	-3.0484905
OLFR175-PS1	-3.0524204
OLFR176	-2.9927483
OLFR178	-4.81559
OLFR181	-3.3973343

OLFR183	-4.3327584
OLFR186	-5.4122324
OLFR187	-3.825739
OLFR19	-4.9452477
OLFR190	-4.462944
OLFR193	-2.6522527
OLFR194	-3.1017988
OLFR195	-3.590829
OLFR196	-5.4641237
OLFR197	-1.2309391
OLFR199	-4.0482707
OLFR2	-3.0899107
OLFR20	-4.615279
OLFR203	-1.2142537
OLFR204	-5.702698
OLFR205	-2.6803567
OLFR206	-2.265892
OLFR207	-3.2622328
OLFR208	-6.03832
OLFR211	-0.6857588
OLFR212	-3.8833992
OLFR213	-3.2472136
OLFR218	-3.456799
OLFR220	-1.5206542
OLFR221	-5.2895617
OLFR222	-4.9376564
OLFR223	-4.0219803
OLFR225	-3.181806
OLFR228	-2.0874543
OLFR229	-3.041034
OLFR22-PS1	-2.1197789
OLFR23	-5.3039885
OLFR231	-3.697639
OLFR235	-2.8518767
OLFR237-PS1	-5.426551
OLFR24	-4.809211
OLFR242	-2.9401853
OLFR247	-6.3025084
OLFR25	-3.123186
OLFR250	-6.487712
OLFR255	-4.774151
OLFR26	-5.003907
OLFR262	-2.7808478
OLFR263	-1.8232355
OLFR267	-3.0809524

OLFR27	-2.216139
OLFR270	-5.5234165
OLFR272	-5.2787676
OLFR275	-5.911295
OLFR279	-4.4875965
OLFR281	-6.953697
OLFR282	-5.6142683
OLFR283	-6.6456947
OLFR284	-3.7755005
OLFR285	-1.681256
OLFR286	-3.1749592
OLFR288	-3.3276842
OLFR290	-2.4469302
OLFR291	-3.2499418
OLFR293	-1.507988
OLFR294	-2.4318538
OLFR297	-5.129754
OLFR299	-3.0765529
OLFR29-PS1	-3.0823164
OLFR3	-3.0713236
OLFR30	-1.4641404
OLFR301	-3.6623118
OLFR303	-4.155739
OLFR304	-3.500463
OLFR305	-3.7804537
OLFR307	-6.3433723
OLFR308	-6.076254
OLFR309	-4.491317
OLFR310	-5.143984
OLFR312	-1.9830806
OLFR314	-4.4479146
OLFR317	-3.2774332
OLFR318	-1.8771868
OLFR32	-6.3168373
OLFR320	-2.4985194
OLFR322	-2.402016
OLFR323	-3.598803
OLFR328	-5.3319397
OLFR33	-2.4394135
OLFR330	-3.0963764
OLFR331	-3.1872408
OLFR332	-4.8621626
OLFR338	-4.49327
OLFR339	-4.293194
OLFR340	-3.3181677

OLFR341	-3.5386589
OLFR342	-2.4502463
OLFR344	-4.8836
OLFR345	-6.460149
OLFR346	-2.5345516
OLFR347	-2.4664407
OLFR348	-5.4343433
OLFR350	-1.7137794
OLFR351	-5.046442
OLFR352	-4.9128456
OLFR354	-5.6599846
OLFR355	-4.007475
OLFR356	-5.71111
OLFR357	-5.070463
OLFR358	-4.675497
OLFR360	-2.2585049
OLFR361	-2.817996
OLFR362	-2.5033748
OLFR365	-4.2321234
OLFR367-PS	-3.6901553
OLFR368	-5.981045
OLFR370	-2.8692222
OLFR372	-4.192444
OLFR374	-3.4809813
OLFR376	-2.893803
OLFR38	-6.0179596
OLFR380	-5.008568
OLFR381	-2.965786
OLFR382	-5.3784723
OLFR384	-2.077096
OLFR385	-2.9184976
OLFR389	-1.3581238
OLFR39	-5.652604
OLFR390	-5.332155
OLFR391-PS	-2.601549
OLFR393	-3.4713013
OLFR394	-2.9281514
OLFR395	-2.7791321
OLFR397	-3.6396976
OLFR398	-5.516702
OLFR399	-3.2733736
OLFR401	-2.6419902
OLFR402	-5.4381175
OLFR403	-2.4732132
OLFR406	-5.297319

OLFR411	-2.5218372
OLFR412	-2.7104936
OLFR418-PS1	-4.3484726
OLFR420	-3.6283352
OLFR426	-3.371775
OLFR427	-5.5757456
OLFR429	-4.6180725
OLFR43	-3.396314
OLFR430	-4.63131
OLFR432	-4.8728514
OLFR433	-6.310345
OLFR434	-2.9971876
OLFR437	-3.0543082
OLFR441	-5.1555614
OLFR444	-3.7785861
OLFR446	-6.9400845
OLFR447	-5.4396086
OLFR448	-2.2778826
OLFR449	-2.8562894
OLFR450	-3.247855
OLFR452	-3.0222347
OLFR455	-3.2581031
OLFR456	-2.106014
OLFR458	-4.4491234
OLFR46	-4.745468
OLFR460	-3.3512175
OLFR461	-5.5274076
OLFR462	-2.8162417
OLFR463	-2.4290822
OLFR464	-4.2436504
OLFR466	-6.250979
OLFR467	-4.739443
OLFR469	-1.8314843
OLFR47	-1.7041259
OLFR470	-2.9478436
OLFR472	-2.44961
OLFR474	-1.4643307
OLFR476	-4.360242
OLFR478	-4.339168
OLFR479	-4.6906867
OLFR480	-5.11604
OLFR481	-7.084715
OLFR484	-3.2935572
OLFR485	-3.9432857
OLFR486	-1.4412324

OLFR487	-5.723073
OLFR49	-5.1832676
OLFR490	-5.8805637
OLFR491	-4.637042
OLFR492	-3.3366466
OLFR493	-2.4858587
OLFR494	-2.7542746
OLFR495	-6.4350185
OLFR498	-3.031637
OLFR5	-4.0061255
OLFR50	-5.257789
OLFR502	-4.1281166
OLFR503	-2.938096
OLFR508	-4.139533
OLFR509	-4.509115
OLFR51	-2.1527293
OLFR510	-2.7510087
OLFR512	-4.8782187
OLFR513	-1.3966396
OLFR514	-5.6761208
OLFR516	-3.4409645
OLFR517	-3.9279423
OLFR518	-5.5794616
OLFR52	-4.634733
OLFR520	-4.9978447
OLFR524	-1.5806682
OLFR525	-3.3614135
OLFR527	-1.1116805
OLFR53	-2.7162995
OLFR533	-2.1874437
OLFR535	-2.3970783
OLFR538	-3.8311126
OLFR539	-3.0438452
OLFR543	-5.886114
OLFR544	-0.9955454
OLFR545	-5.1389556
OLFR547	-5.80689
OLFR550	-3.8043778
OLFR551	-4.0196123
OLFR552	-3.3216655
OLFR553	-1.7444563
OLFR554	-7.3473577
OLFR555	-5.3017707
OLFR556	-1.8568254
OLFR557	-3.2015018

OLFR558	-4.720619
OLFR559	-3.7909744
OLFR560	-3.6317902
OLFR561	-2.2925994
OLFR564	-3.594659
OLFR566	-2.8547606
OLFR568	-1.8962669
OLFR569	-4.818561
OLFR57	-1.5615752
OLFR570	-4.4879236
OLFR571	-4.8502026
OLFR574	-6.2155666
OLFR575	-3.0669274
OLFR577	-3.8812227
OLFR578	-4.8371854
OLFR58	-4.012331
OLFR582	-6.2893276
OLFR584	-4.7106576
OLFR586	-3.6522558
OLFR589	-1.3580527
OLFR59	-7.092127
OLFR591	-3.5161037
OLFR593	-6.055032
OLFR594	-3.1649795
OLFR596	-4.299973
OLFR598	-6.1031294
OLFR599	-3.2121787
OLFR6	-5.3397818
OLFR60	-3.9430296
OLFR601	-3.5290167
OLFR605	-2.1275196
OLFR607	-4.6090183
OLFR608	-2.1695783
OLFR61	-4.44053
OLFR610	-3.323059
OLFR612	-2.298015
OLFR615	-2.8606358
OLFR617	-4.754406
OLFR618	-4.7068534
OLFR619	-6.0299673
OLFR62	-5.1673326
OLFR620	-4.9109144
OLFR623	-4.2921033
OLFR624	-3.408659
OLFR629	-2.3879182

OLFR63	-2.7425063
OLFR630	-4.405511
OLFR631	-3.1571648
OLFR632	-8.551832
OLFR635	-1.400821
OLFR638	-3.1745162
OLFR639	-3.7355614
OLFR640	-4.8666263
OLFR645	-6.5397053
OLFR646	-4.822464
OLFR648	-5.172146
OLFR65	-3.9753437
OLFR651	-4.726565
OLFR652	-5.3110037
OLFR653	-1.3171327
OLFR655	-5.6213193
OLFR656	-2.2676635
OLFR657	-1.660749
OLFR658	-3.986141
OLFR659	-3.0604057
OLFR66	-6.248142
OLFR661	-4.280135
OLFR665	-3.52663
OLFR666	-5.4118285
OLFR667	-2.1426203
OLFR668	-4.0869603
OLFR669	-2.2683203
OLFR67	-3.6857448
OLFR670	-8.195032
OLFR671	-5.263721
OLFR676	-5.623675
OLFR678	-6.6500826
OLFR681	-2.030635
OLFR683	-3.4063954
OLFR684	-5.5296774
OLFR686	-4.0608454
OLFR687	-1.8683078
OLFR688	-3.9673731
OLFR69	-2.0921774
OLFR690	-2.6516743
OLFR691	-6.2232466
OLFR692	-5.3676176
OLFR693	-2.5699418
OLFR694	-1.9527025
OLFR695	-4.4152517

OLFR697	-4.6785593
OLFR698	-6.2600484
OLFR699	-6.1065884
OLFR70	-4.140787
OLFR700	-1.9097202
OLFR701	-5.9862475
OLFR702	-3.9953961
OLFR703	-2.2752166
OLFR705	-4.951089
OLFR706	-1.3877175
OLFR707	-4.890355
OLFR71	-6.511918
OLFR710	-4.721506
OLFR713	-3.3683987
OLFR714	-7.0438213
OLFR715	-4.663487
OLFR716	-2.4484997
OLFR720	-4.636197
OLFR722	-5.773761
OLFR723	-5.9195175
OLFR724	-3.1977901
OLFR726	-2.4944577
OLFR728	-3.3371656
OLFR729	-5.09649
OLFR73	-1.7055657
OLFR731	-1.3733878
OLFR732	-3.3443446
OLFR733	-2.4496694
OLFR734	-3.116493
OLFR738	-2.2236793
OLFR739	-1.3724847
OLFR74	-0.8977983
OLFR741	-4.0747104
OLFR743	-5.953985
OLFR744	-1.9179313
OLFR745	-2.3572166
OLFR747	-2.1541607
OLFR749	-7.193883
OLFR75-PS1	-1.5462451
OLFR76	-6.8737955
OLFR761	-4.736795
OLFR763	-3.940657
OLFR764	-3.7610073
OLFR767	-5.235125
OLFR768	-3.3189914

OLFR769	-3.6490166
OLFR77	-2.5725298
OLFR770	-3.769795
OLFR771	-2.3892264
OLFR772	-3.37384
OLFR773	-2.1143305
OLFR774	-3.477512
OLFR775	-2.2505534
OLFR776	-4.2036104
OLFR78	-2.5777674
OLFR780	-3.6129804
OLFR784	-3.4958663
OLFR786	-3.9116082
OLFR787	-1.0329773
OLFR788	-3.495594
OLFR790	-4.7355595
OLFR791	-2.315247
OLFR792	-3.2283978
OLFR794	-1.8627923
OLFR796	-1.4938316
OLFR798	-4.512343
OLFR799	-3.265247
OLFR8	-1.7255785
OLFR802	-3.6641252
OLFR803	-3.8523245
OLFR804	-3.5326252
OLFR805	-2.792113
OLFR806	-5.125165
OLFR808	-3.1122384
OLFR809	-2.5279021
OLFR810	-1.7716646
OLFR811	-2.5445826
OLFR813	-3.8359423
OLFR815	-4.2598114
OLFR816	-5.097319
OLFR819	-4.471176
OLFR820	-4.650645
OLFR821	-2.521344
OLFR823	-4.1861596
OLFR824	-5.0435524
OLFR826	-1.7824082
OLFR827	-3.5755486
OLFR828	-6.778736
OLFR829	-4.6398506
OLFR830	-7.3949804

OLFR835	-2.4186654
OLFR836	-7.3777723
OLFR837	-5.601759
OLFR843	-3.8811493
OLFR845	-4.476722
OLFR846	-2.094017
OLFR847	-4.5616136
OLFR849	-5.908963
OLFR850	-6.7813177
OLFR853	-3.0231304
OLFR854	-6.4195185
OLFR855	-4.337046
OLFR860	-2.8025613
OLFR866	-4.841757
OLFR867	-3.9884384
OLFR869	-3.226602
OLFR870	-3.6309493
OLFR871	-4.2079234
OLFR873	-5.1482306
OLFR874	-4.0301247
OLFR876	-4.1013327
OLFR877	-5.3916254
OLFR878	-1.1826613
OLFR884	-2.3876307
OLFR887	-4.092169
OLFR888	-2.698884
OLFR889	-1.1746123
OLFR890	-2.540849
OLFR891	-3.9249365
OLFR894	-1.6481953
OLFR895	-3.8177555
OLFR899	-5.0050383
OLFR9	-3.7790668
OLFR90	-3.6386878
OLFR901	-1.989156
OLFR902	-1.0785353
OLFR904	-5.6046453
OLFR905	-4.443569
OLFR906	-5.701433
OLFR907	-4.9661865
OLFR908	-4.0562954
OLFR910	-5.588648
OLFR911-PS1	-3.8211734
OLFR912	-2.3732991
OLFR913	-4.490458

OLFR914	-3.8856723
OLFR915	-3.0323405
OLFR916	-3.0606368
OLFR917	-4.5607753
OLFR918	-5.242674
OLFR919	-3.3530483
OLFR92	-2.233837
OLFR920	-1.5166945
OLFR921	-5.270172
OLFR922	-7.940523
OLFR923	-3.75012
OLFR924	-2.3684323
OLFR926	-1.6216407
OLFR930	-2.838336
OLFR933	-5.832039
OLFR934	-3.8521152
OLFR936	-1.9644687
OLFR938	-2.5532439
OLFR94	-3.7448773
OLFR943	-2.8536665
OLFR945	-5.091445
OLFR947-PS1	-2.4100811
OLFR948	-2.7357314
OLFR95	-6.0382385
OLFR951	-3.8310404
OLFR954	-3.9973395
OLFR955	-2.3695073
OLFR957	-6.726713
OLFR958	-2.9866593
OLFR959	-2.1834347
OLFR961	-5.58414
OLFR963	-5.1461267
OLFR965	-4.293868
OLFR969	-3.455248
OLFR971	-5.3976912
OLFR974	-3.8312817
OLFR975	-6.8269186
OLFR976	-4.5396495
OLFR978	-3.4946854
OLFR979	-6.879799
OLFR98	-2.0273688
OLFR980	-7.1628475
OLFR981	-4.5895977
OLFR982	-5.3119216
OLFR984	-5.5972166

OLFR985	-5.9555397
OLFR986	-6.996829
OLFR988	-2.2637587
OLFR99	-3.04988
OLFR992	-1.5556939
OLFR994	-4.0144444
OLFR995	-4.037789
OLFR996	-3.0278897
OLFR998	-5.3345675
OMP	-1.7237525
OMT2A	-11.938915
OOEP	-3.773709
OOG2	-3.1977143
OOG3	-3.0005548
OOG4	-1.7319083
OOSP1	-2.1946263
OOSP2	-1.7035751
OPCML	-2.6757503
OPN1MW	-3.2414408
OPN1SW	-1.142427
OPN4	-3.2861571
OPN5	-4.429599
OPRD1	-5.388998
OPRK1	-5.0780706
OPRL1	-3.527539
ORLY	-3.8584716
OSBP2	-2.2937
OSTN	-4.693007
OTOA	-6.2138844
OTOG	-2.9822748
OTOGL	-2.6526399
OTOL1	-3.8050475
OTOP3	-3.70848
OTOR	-3.731528
OTOS	-3.2945704
OTP	-4.4293942
OTUB1	-0.6966162
OTUD6A	-5.0297575
OTUD7B	-3.020066
OTX2	-2.5262764
OVOL1	-4.3659754
OXCT2A	-3.29638
OXCT2B	-1.4276977
OXT	-1.0713301
P2RX2	-2.0487633

P2RX6	-4.53321
P2RY4	-5.2755384
P4HA3	-4.2708197
PABPC1L2B-F	-3.2079859
PABPC4L	-1.0858026
PABPC5	-2.2533398
PABPC6	-6.0505505
PADI6	-3.6715355
PAK7	-1.5846958
PALM3	-2.1636283
PANX2	-4.1431923
PAPL	-3.4480023
PAPLN	-1.1631305
PAPOLB	-4.030156
PAPSS1	-0.7376909
PARD3B	-4.4029226
PARD3BOS1	-5.2142067
PARPBP	-3.815793
PAX2	-4.8270283
PAX3	-2.2777815
PAX4	-5.0038943
PAX5	-3.6401386
PAX6OS1	-3.3009467
PAX7	-3.2191281
PAX8	-5.2169447
PAX9	-3.1446285
PBP2	-1.569154
PBSN	-4.0004444
PBX4	-5.3766127
PCDH10	-5.779232
PCDH11X	-2.978423
PCDH15	-4.135721
PCDH8	-2.4037974
PCDH9	-3.5167246
PCDHA11	-5.072406
PCDHA12	-4.6141863
PCDHA4	-3.0310109
PCDHA8	-0.7569714
PCDHA9	-4.5182743
PCDHAC1	-5.321674
PCDHB1	-3.2649713
PCDHB12	-1.363586
PCDHB19	-4.5826263
PCDHB2	-3.1622531
PCDHGB1	-2.0144055

PCLO	-3.5561092
PCSK1N	-2.744006
PCSK2	-3.0121894
PCSK4	-1.7697039
PCYT1B	-2.7775893
PDC	-2.670245
PDCL2	-5.080019
PDE10A	-3.2340393
PDE11A	-3.587798
PDE1C	-5.8335686
PDE3A	-2.0641804
PDE5A	-2.6189656
PDE6A	-2.6591148
PDE6C	-4.9292707
PDGFD	-2.2745297
PDHA2	-5.777896
PDK2	-1.1901898
PDX1	-3.035834
PDYN	-2.8740444
PDZD7	-3.4896998
PDZD9	-4.3230743
PDZRN4	-4.3670464
PEA15B	-3.81331
PEBP4	-1.3407793
PEG10	-2.4319224
PEG12	-2.3179586
PERM1	-3.0157351
PET100	-3.0768452
PEX5L	-1.8045933
PFN3	-5.0444174
PFN4	-4.8692327
PFPL	-4.1398916
PGA5	-5.187845
PGBD1	-4.2701235
PGC	-1.909709
PGK2	-4.038516
PGLYRP3	-2.0556817
PGLYRP4	-0.8815088
PGPEP1L	-5.0330453
PGR	-1.894995
PHACTR3	-3.8840914
PHF20L1	-0.6453853
PHGR1	-3.2241213
PHLDA2	-2.4702604
PHOX2A	-4.484223

PHOX2B	-1.9452221
PHTF1OS	-2.7460697
PHYHIP	-6.0193357
PIBF1	-2.639569
PIH1D3	-0.7635047
PIK3C2B	-3.5579958
PIKFYVE	-1.9834964
PILRB1	-1.7915988
PINC	-4.4570684
PIP	-4.232048
PIP5K1BOS	-4.7615094
PIP5KL1	-2.8702087
PIRT	-2.4185479
PISD-PS2	-2.531009
PITX1	-3.3695016
PITX2	-3.316602
PITX3	-2.9770565
PIWIL2	-3.1566834
PKD1L1	-3.756829
PKD2L1	-6.9011974
PKDREJ	-3.1743474
PKHD1	-2.0587027
PKHD1L1	-2.5788631
PLA2G2A	-3.4014995
PLA2G2D	-0.7288144
PLA2G2E	-2.261996
PLA2G2F	-3.0647662
PLA2G3	-5.681224
PLA2G4C	-2.0402443
PLA2G4D	-4.638119
PLA2G4E	-3.0910673
PLA2G5	-3.9733086
PLA2R1	-2.2786496
PLAC1	-4.4127474
PLAC8L1	-2.955262
PLAG1	-4.215987
PLCB2	-1.781502
PLCZ1	-2.7049537
PLD6	-4.167138
PLDI	-6.2413664
PLEKHG1	-2.9471798
PLEKHN1	-5.281152
PLK4	-2.228648
PLLP	-2.645696
PMEL	-1.883182

PMFBP1	-1.8184559
PMIS2	-6.502198
PMP2	-1.7861431
PNLIP	-3.267707
PNLIPRP2	-3.1417632
PNMA3	-2.1499922
PNMAL1	-3.8046067
PNOC	-2.7605634
PNPLA5	-2.4112625
POLK	-0.8650034
POLR3F	-1.1789763
POLR3H	-3.183747
POM121L12	-8.132439
POM121L2	-3.9800067
POMC	-0.9699221
POTEG	-5.176588
POU1F1	-2.9225535
POU3F1	-1.7767196
POU3F2	-5.9875884
POU3F3OS	-1.624078
POU3F4	-2.8261435
POU4F3	-3.111368
POU5F1	-2.5163586
PP2D1	-2.9253938
PPAPDC1A	-4.431254
PPARGC1B	-0.869416
PPEF1	-3.3066664
PPFIA2	-2.9816651
PPIE	-0.8559592
PPP1R12B	-1.7448428
PPP1R17	-3.0601497
PPP1R27	-1.2146993
PPP1R3FOS	-2.656006
PPP2R2COS	-1.9977098
PPP2R3A	-2.9021466
PPP3R2	-3.1643798
PPP6R2	-0.9918556
PRAMEF12O!	-3.6235828
PRAMEL	-5.410907
PRAMEL1	-3.861479
PRAMEL7	-7.4139624
PRAP1	-4.305331
PRB1	-4.239977
PRDM11	-2.1022437
PRDM12	-2.7049153

PRDM14	-2.3712735
PRDM15	-2.7494488
PRDM9	-2.6921663
PRDX6B	-1.3564694
PRG2	-2.1837466
PRICKLE2	-5.2964554
PRICKLE4	-5.9944143
PRKAB1	-1.2852387
PRKAG2OS1	-4.987216
PRKAG3	-3.3217082
PRL2B1	-5.331045
PRL2C5	-3.7170858
PRL3A1	-6.088008
PRL3B1	-2.493988
PRL3C1	-4.16755
PRL3D1	-2.4337997
PRL3D2	-2.734783
PRL4A1	-4.550893
PRL6A1	-2.3950381
PRL7A1	-5.6884766
PRL7A2	-3.185488
PRL7C1	-3.6079452
PRL7D1	-1.9690659
PRL8A6	-3.426099
PRL8A8	-3.1489851
PRL8A9	-2.0006144
PRLH	-2.9236794
PRLHR	-1.358552
PRM1	-1.8275089
PRM2	-3.6953325
PRM3	-3.343301
PRMT8	-4.777812
PROCA1	-3.8112261
PROKR1	-2.5047162
PROKR2	-2.882614
PROL1	-2.901943
PROM2	-2.3654888
PROP1	-3.203834
PROSER1	-2.2871904
PRP2	-1.9757044
PRPH2	-3.465856
PRPMP5	-3.5237925
PRPS1L1	-2.851855
PRR19	-4.8017616
PRR27	-1.7780719

PRR30	-5.864298
PRR32	-2.4458091
PRR33	-2.0072546
PRR9	-1.5756595
PRRG4	-1.6293821
PRRT2	-6.067554
PRRT4	-3.3976135
PRRXL1	-3.9255946
PRSS16	-3.6612911
PRSS2	-3.3965929
PRSS22	-3.3364365
PRSS27	-2.1732302
PRSS28	-5.572902
PRSS30	-3.015337
PRSS32	-2.6495266
PRSS33	-2.7837253
PRSS35	-2.1601696
PRSS39	-2.9693198
PRSS40	-1.8406796
PRSS41	-1.9310031
PRSS42	-6.7937956
PRSS44	-5.398526
PRSS45	-3.1935973
PRSS46	-3.2059247
PRSS52	-4.578925
PRSS54	-6.876036
PRSS55	-2.002014
PRSS56	-3.591855
PRSS58	-6.1165276
PRUNE	-1.6269495
PRUNE2	-2.65522
PSD2	-3.3195083
PSG18	-4.611459
PSG19	-3.5584707
PSG20	-1.1769097
PSG21	-4.3406982
PSG22	-2.903414
PSG25	-3.9792633
PSG27	-3.4018705
PSG28	-4.5039325
PSG29	-3.0640275
PSMA8	-2.0353677
PSMB11	-5.4522114
PSMC3IP	-3.834441
PSPN	-2.4683876

PSTK	-1.0853362
PTCD1	-1.7071807
PTCH1	-2.4038134
PTCHD2	-3.6033463
PTCRA	-6.68629
PTF1A	-6.21486
PTGDR	-1.8329253
PTGDR2	-5.6870995
PTHLH	-1.2546201
PTK6	-1.367435
PTPN20	-6.096821
PTPRH	-3.2733128
PTPRM	-1.9281874
PTPRN	-5.133678
PTPRN2	-2.7777267
PTPRQ	-4.9018583
PTPRR	-2.678773
PTX4	-1.2733519
PVALB	-2.3319812
PVT1	-1.0008976
PXT1	-4.6267347
PYROXD1	-1.2457848
QRFP	-5.4019756
QRFPR	-3.8714614
QTRT1	-2.57254
R3HDML	-3.2480018
RAB11FIP2	-6.457923
RAB11FIP4OS	-5.8638496
RAB26	-1.6075387
RAB9B	-1.387095
RAD21L	-1.4718819
RAD51AP2	-1.1734273
RAD9A	-0.7703588
RAET1A	-3.3269823
RAG1	-1.3468409
RAG2	-1.2557309
RALYL	-3.0097957
RANBP17	-3.4986343
RANBP3L	-2.3446107
RAP1GAP2	-2.8155348
RASAL2	-2.2272022
RASGEF1C	-2.6976745
RASGRF2	-2.919597
RASL10B	-2.7906241
RASSF10	-1.4808495

RAVER2	-1.2369375
RAX	-7.5619965
RB1CC1	-4.009852
RBAKDN	-3.1764205
RBBP8NL	-1.2350013
RBM15B	-1.4907265
RBM20	-3.4791617
RBM38	-2.7040327
RBM46	-1.8964269
RBM46OS	-3.0951319
RBMY	-3.128168
RBP2	-4.929166
RCOR2	-4.864549
RCVRN	-2.447382
RD3L	-1.9863219
RDH18-PS	-3.8225238
RDH19	-3.8687449
RDH8	-3.8063357
RECQL4	-1.5825782
REEP2	-4.25109
REG2	-4.7167206
REG3A	-5.3763747
REG3D	-2.3217852
REL	-5.0426254
RELL2	-1.7925715
REM2	-3.7990246
RERGL	-2.3221166
REX2	-3.0760777
RFC3	-0.980252
RFESD	-1.3721883
RFPL3S	-2.504188
RFPL4	-2.9200938
RFPL4B	-3.689995
RFX4	-3.2219346
RFX8	-1.7339826
RGAG1	-6.8226757
RGMA	-2.3157554
RGR	-4.4949474
RGS13	-4.136966
RGS21	-3.2470436
RGS7	-3.8870273
RGS8	-3.8264008
RGS9BP	-2.2042599
RGSL1	-0.9683061
RHAG	-3.5221994

RHBDD1	-2.9819405
RHBDL2	-6.0489626
RHCG	-4.4426413
RHO	-2.4530551
RHOH	-3.1744297
RHOX1	-1.3611977
RHOX10	-5.6053963
RHOX11	-3.5252228
RHOX12	-5.651334
RHOX13	-4.6938496
RHOX2C	-4.0972366
RHOX2E	-3.9594889
RHOX2F	-1.5435867
RHOX2H	-4.200887
RHOX3E	-3.9885247
RHOX3F	-3.7599685
RHOX3G	-4.217631
RHOX3H	-2.8776965
RHOX4A	-7.102749
RHOX4E	-5.2757206
RHOX4G	-4.701936
RHOX6	-3.1293938
RHOX7	-3.2633932
RHPN2	-1.5474896
RIBC2	-3.3188531
RIC3	-1.5869381
RIMBP2	-2.8931935
RIMBP3	-4.2347393
RIMKLB	-1.1842325
RIMS1	-4.772647
RIMS2	-2.0101824
RIPPLY2	-4.7200212
RLBP1	-3.7497106
RLN1	-3.9173055
RLN3	-3.598702
RLTPR	-0.9826446
RNASE1	-5.1709027
RNASE12	-1.7229862
RNF112	-2.9200578
RNF122	-2.3657598
RNF151	-5.7330847
RNF165	-1.4309483
RNF180	-3.0688214
RNF183	-2.2242165
RNF212	-2.5924618

ROBO3	-1.6397061
ROPN1	-2.8680522
ROR1	-1.8878822
RORB	-2.7774644
RP1L1	-2.471543
RPE65	-5.563098
RPH3A	-3.508847
RPL10L	-3.6023521
RPP25	-6.4818773
RPRML	-2.700414
RPTN	-6.907871
RRH	-2.3576422
RSG1	-5.967146
RSPH6A	-5.078081
RSPO4	-3.862266
RSRC1	-1.6757414
RTBDN	-2.826092
RTCA	-1.0243578
RTL1	-7.064253
RUNX2	-2.927674
RWDD2A	-4.851553
RXFP2	-2.224088
RXFP3	-2.980757
RXFP4	-2.764296
S100A2	-3.2839563
S100A5	-1.594666
S100Z	-2.4051583
SAG	-1.7812204
SALL3	-2.1646855
SAMD11	-4.493487
SAMD15	-2.3124976
SAMD3	-2.8343048
SAMD7	-3.1862595
SAMT2	-3.319751
SAMT3	-1.5860224
SARM1	-3.518775
SATB2	-2.619034
SATL1	-2.865425
SBK1	-2.6045954
SBK2	-3.8917575
SBP	-2.7027993
SBPL	-4.5822325
SCARLETLTR	-6.6272516
SCG3	-2.1416078
SCGB1B19	-5.9445715

SCGB1B2	-6.373408
SCGB1B20	-4.0826564
SCGB1B24	-3.059641
SCGB1B27	-1.0944457
SCGB1B3	-2.4954567
SCGB1B30	-0.7277818
SCGB1B7	-3.6071484
SCGB2B2	-2.6480331
SCGB2B20	-1.253042
SCGB2B21	-3.1143172
SCGB2B24	-1.3249295
SCGB2B26	-4.792679
SCGB2B27	-4.8756256
SCGB2B7	-3.0299454
SCLT1	-1.0258665
SCML1	-3.291329
SCML2	-6.103098
SCN1A	-3.444766
SCN2A1	-3.2453456
SCN4A	-1.6762633
SCN4B	-3.238884
SCN9A	-1.8513603
SCP2D1	-4.403512
SCRT2	-2.0845716
SCT	-2.9323187
SDHD	-2.6123695
SDK2	-1.7042925
SDR16C6	-3.798842
SEC14L5	-3.826993
SEC22C	-3.2246306
SECTM1A	-1.7251592
SEL1L2	-1.3581672
SEMA3D	-2.2831876
SEMA5B	-3.823058
SEMA6C	-2.1595988
SERAC1	-1.3070977
SERPINA12	-1.8800845
SERPINA1F	-2.7286515
SERPINA3A	-4.7503643
SERPINA3J	-3.1994853
SERPINA5	-4.23606
SERPINB12	-3.8427145
SERPINB1C	-3.5288253
SERPINB3A	-6.0044847
SERPINB3B	-5.9898205

SERPINB3D	-5.9227734
SERPINB6D	-4.9050126
SERPINB6E	-4.5307302
SERPINB9C	-4.6941752
SERPINB9D	-1.3890688
SERPINB9E	-2.7958498
SERPINB9F	-2.9738066
SERPINI1	-1.4553809
SERPINI2	-4.34941
SERTM1	-2.1039665
SETDB2	-2.8534997
SEZ6	-2.5684226
SEZ6L2	-1.3608389
SFXN4	-4.0413713
SFXN5	-2.312157
SGCD	-4.765771
SGCZ	-3.254753
SGMS2	-1.8352537
SGTB	-4.2587705
SH2D1B2	-5.918888
SH2D4A	-1.5190918
SH2D5	-2.66292
SH2D6	-1.9503772
SH2D7	-4.0014944
SH3BP1	-1.8130674
SH3GL2	-4.318762
SHANK1	-1.3034866
SHANK2	-2.4746475
SHBG	-6.0256605
SHC3	-2.8844452
SHCBP1L	-4.361205
SHD	-3.8663661
SHISA2	-2.7919183
SHISA7	-3.231147
SHISA9	-4.593496
SHOX2	-3.5745645
SHROOM3	-5.3147364
SHROOM4	-3.8844907
SIAH3	-2.4366643
SIGLEC15	-2.7344184
SIM2	-0.9266093
SIS	-5.274451
SIT1	-1.5704412
SIX2	-3.5587227
SIX3	-4.48446

SIX3OS1	-4.0839844
SIX6	-3.1987348
SKIDA1	-5.4563684
SKINT1	-4.703971
SKINT10	-2.208657
SKINT2	-3.2296326
SKINT3	-3.299723
SKINT4	-4.921582
SKINT5	-3.739092
SKINT6	-3.9700284
SKINT8	-4.773421
SLA2	-0.6644495
SLAIN1OS	-3.3679576
SLC10A4	-5.511042
SLC12A3	-5.579573
SLC13A1	-2.1526139
SLC13A2OS	-2.004199
SLC16A1	-2.0035367
SLC16A4	-1.86431
SLC17A7	-2.3457382
SLC17A8	-0.9788702
SLC18A1	-1.1138809
SLC18A3	-3.773665
SLC1A7	-3.201942
SLC22A12	-3.007864
SLC22A19	-1.7730777
SLC22A2	-3.6987667
SLC22A22	-3.970315
SLC22A4	-2.716271
SLC24A1	-2.5444593
SLC24A2	-2.7114468
SLC25A18	-2.6056988
SLC25A31	-3.5307271
SLC25A43	-3.8692453
SLC25A53	-2.1551108
SLC25A54	-2.5075595
SLC26A3	-3.3959472
SLC26A5	-5.0026484
SLC26A7	-2.6917481
SLC26A8	-2.885457
SLC2A7	-4.9973087
SLC30A2	-2.2991724
SLC30A3	-2.3506522
SLC32A1	-2.2957475
SLC34A1	-2.0280092

SLC34A3	-2.2568033
SLC35A5	-2.5468364
SLC35D3	-2.3689435
SLC35F1	-2.3046503
SLC35F4	-1.4028866
SLC35G2	-1.982444
SLC35G3	-1.7607911
SLC36A1OS	-3.642194
SLC36A3	-1.7260542
SLC36A4	-1.0690923
SLC37A2	-2.0624714
SLC38A11	-3.7693334
SLC38A8	-3.8376496
SLC39A12	-1.9816177
SLC39A2	-1.3625786
SLC39A5	-3.669244
SLC44A5	-1.8529036
SLC45A2	-4.55389
SLC46A2	-2.7442503
SLC51A	-6.098879
SLC52A2	-1.3981509
SLC5A10	-2.5987685
SLC5A11	-4.7362394
SLC5A12	-3.9861934
SLC5A4A	-4.8608284
SLC5A4B	-1.0907452
SLC5A5	-1.8562629
SLC5A7	-2.873319
SLC5A8	-4.1784606
SLC6A1	-4.2309837
SLC6A16	-3.0720026
SLC6A17	-5.233758
SLC6A18	-4.2878613
SLC6A19OS	-4.0052476
SLC6A20B	-2.9768863
SLC6A7	-2.564607
SLC7A12	-1.1910849
SLC7A13	-3.9205759
SLC7A14	-3.3391368
SLC7A15	-4.1978445
SLC8A1	-1.5848043
SLC9A2	-2.1188471
SLC9A5	-3.5425532
SLC9A9	-3.0072877
SLC9B1	-5.9887934

SLC9B2	-3.618931
SLC9C1	-4.3169394
SLCO1C1	-2.7785978
SLCO5A1	-3.7474504
SLCO6B1	-1.4523411
SLCO6C1	-4.3180265
SLFNL1	-2.61598
SLITRK2	-1.0111439
SLITRK3	-6.2423935
SLITRK4	-5.9913626
SLITRK5	-4.9182105
SLMO1	-0.9985969
SLX1B	-0.9838579
SLXL1	-2.5496628
SLY	-3.3661573
SMC2OS	-2.0049708
SMCO1	-3.9420085
SMCO2	-1.9203777
SMCO3	-3.6236007
SMCP	-5.5788765
SMEK3-PS	-3.4017508
SMIM23	-4.133399
SMIM9	-1.8770185
SMOK2A	-3.3377883
SMOK2B	-1.5898359
SMOK3B	-1.385071
SMOK4A	-4.95979
SMPD5	-1.0141788
SMR2	-3.0881505
SMR3A	-1.7382793
SMURF1	-1.3160167
SMYD3	-1.46996
SMYD4	-1.3848703
SNAI3	-4.1089625
SNAP91	-1.6984687
SNCB	-2.485081
SNHG7OS	-1.1686373
SNHG8	-3.4784825
SNTB1	-1.0363183
SOGA3	-5.24195
SOHLH1	-3.6166682
SORCS1	-4.404067
SOSTDC1	-2.07438
SOX1	-1.278203
SOX11	-2.7883086

SOX12	-3.9743774
SOX14	-3.530554
SOX15	-3.8909209
SOX21	-1.5385547
SOX3	-4.244216
SOX30	-8.187131
SOX5IT	-2.1080825
SOX5OS1	-2.7075276
SOX6OS	-2.7562573
SOX8	-2.2212162
SP7	-2.9806635
SP9	-5.5171876
SPACA1	-3.26965
SPACA5	-1.3724265
SPACA7	-4.0318985
SPAG11A	-3.372145
SPAG11B	-3.0983498
SPAG17OS	-4.2405486
SPAG8	-4.1565127
SPAM1	-4.0030556
SPATA16	-1.7214341
SPATA19	-1.7873802
SPATA20	-4.668265
SPATA21	-2.9582105
SPATA24	-2.1136763
SPATA25	-2.6089988
SPATA2L	-2.197797
SPATA3	-1.7652063
SPATA31	-2.6891363
SPATA31D1B	-3.4097486
SPATA31D1D	-5.431061
SPATA33	-5.170229
SPATA4	-6.1672816
SPATA45	-3.805756
SPATA9	-2.5007849
SPATC1	-3.3376467
SPDL1	-1.7127926
SPDYA	-3.2993891
SPDYB	-4.451607
SPEER1-PS1	-4.442157
SPEER2	-5.6679525
SPEER3	-2.4638293
SPEER4A	-4.390025
SPEER4D	-3.9666467
SPEER4F	-2.1697576

SPEER5-PS1	-2.5620081
SPEER8-PS1	-5.3218327
SPEER9-PS1	-7.887099
SPEM1	-1.153662
SPERT	-3.2781272
SPESP1	-1.568841
SPIN2D	-2.807621
SPINK11	-2.8468227
SPINK13	-3.6968496
SPINK6	-2.2800364
SPINK7	-3.606971
SPINK8	-3.421526
SPINKL	-2.6995432
SPINT3	-2.3068082
SPINT4	-3.0522406
SPINT5	-4.442478
SPO11	-3.7349021
SPOCD1	-2.1180375
SPON2	-2.4808931
SPPL2C	-3.1110396
SPRN	-1.8368213
SPRR1B	-4.9284067
SPRR2B	-1.2808793
SPRR2F	-2.5676756
SPRR2H	-7.3196497
SPRR2I	-3.7336812
SPRR2J-PS	-3.2006567
SPRR4	-5.705799
SPRTN	-2.029925
SPRY2	-2.7773132
SPRY4	-1.6537411
SPSB4	-2.9840045
SPTBN5	-3.796852
SPTSSB	-1.1591053
SPZ1	-2.2932863
SRC	-3.5992906
SRCRB4D	-2.6026843
SRRM3	-3.2737093
SRRM3OS	-5.174608
SRRM4OS	-4.397331
SRSF12	-4.4309487
SRY	-3.989
SSMEM1	-3.025287
SST	-2.6187785
SSTR1	-4.8473487

SSTR2	-2.0064206
SSTR5	-1.6404309
SSTY1	-3.8526506
SSU2	-3.043851
SSX9	-4.308833
SSXA1	-3.8590362
SSXB1	-5.615633
SSXB10	-5.193306
SSXB7	-4.2519627
SSXB8	-3.9055912
ST18	-4.917457
ST6GAL2	-3.2471669
ST6GALNAC3	-2.471839
ST8SIA3	-2.3528707
ST8SIA5	-2.8774464
STC2	-3.8036747
STEAP3	-1.8829615
STIL	-1.42367
STIM2	-2.1063044
STK32B	-13.82603
STK32C	-4.190162
STK36	-2.1690207
STMN4	-4.068952
STOML3	-4.867666
STON2	-1.4463212
STOX2	-1.1651716
STPG2	-1.7402892
STRA8	-3.4719768
STRC	-5.148964
STRM	-5.4692626
STXBP5L	-3.6439278
STYK1	-2.347897
SUFU	-1.2276962
SULT2A7	-3.5730867
SUN5	-2.0981812
SUPT20	-1.016381
SUPT3	-0.9711878
SUSD1	-3.1064801
SUSD3	-3.050082
SUV39H1	-1.5811303
SV2A	-1.6373672
SV2B	-3.0488026
SV2C	-0.9281292
SVAL1	-4.348947
SVAL2	-2.8791428

SVAL3	-1.5340204
SVOP	-3.427142
SVOPL	-0.9411304
SVS1	-1.7494049
SVS2	-3.8901172
SVS3B	-3.4324236
SVS4	-3.75925
SVS5	-4.831647
SYCE1L	-3.9195526
SYCE3	-4.10499
SYCN	-5.555417
SYCP1-PS1	-2.1450946
SYCP2L	-2.124297
SYN1	-3.109037
SYN3	-1.3126926
SYNA	-5.15222
SYNB	-1.758704
SYNDIG1	-2.36566
SYNDIG1L	-4.956065
SYNGAP1	-3.361579
SYNGR3	-3.32824
SYNGR4	-2.2323217
SYNPR	-10.606516
SYPL2	-2.6386697
SYT10	-4.890686
SYT14	-5.3602915
SYT16	-4.4154134
SYT17	-3.904647
SYT2	-3.0474567
SYT3	-2.0629723
SYT7	-4.276276
SYT8	-5.527748
SYTL1	-2.3171124
SYTL4	-2.07582
SYTL5	-4.2066407
TAAR1	-3.0497465
TAAR5	-3.2423759
TAAR6	-1.4619842
TAAR7A	-4.301094
TAAR7B	-2.6260374
TAAR7D	-3.1277242
TAAR7F	-5.744463
TAAR8A	-5.555339
TAAR9	-2.1131394
TAC2	-2.1433954

TAC4	-2.5237203
TACO1OS	-4.5420456
TACR1	-0.6585505
TACR3	-3.780856
TAGLN3	-3.0595555
TAMM41	-1.6278818
TANC2	-2.0374973
TAS1R1	-3.6277823
TAS1R2	-4.6381903
TAS2R103	-3.792573
TAS2R104	-5.0881014
TAS2R105	-1.7791181
TAS2R106	-4.218651
TAS2R107	-1.5186851
TAS2R110	-4.693568
TAS2R114	-2.7965727
TAS2R115	-2.5478122
TAS2R118	-3.6328237
TAS2R119	-3.2297363
TAS2R120	-3.3234088
TAS2R121	-3.6972692
TAS2R122	-1.459245
TAS2R124	-6.524609
TAS2R125	-3.9434667
TAS2R126	-1.6506801
TAS2R130	-2.6702938
TAS2R134	-6.544508
TAS2R135	-2.444213
TAS2R138	-4.24039
TAS2R139	-4.0914326
TAS2R140	-4.9638553
TAS2R143	-2.9082737
TBATA	-2.0977602
TBC1D22BOS	-2.4621458
TBC1D31	-1.4486792
TBCE	-2.2489998
TBL3	-2.1534305
TBPL2	-2.429541
TBR1	-5.1731634
TBX10	-2.484687
TBX15	-4.1974974
TBX20	-3.9713798
TBX3OS1	-5.1774426
TCAM1	-3.0793362
TCEA2	-2.5054324

TCEANC	-3.2838507
TCERG1L	-2.6618774
TCFL5	-5.1389637
TCHHL1	-2.7614155
TCL1	-4.1890955
TCL1B1	-1.5191531
TCL1B3	-4.702839
TCL1B4	-2.7287998
TCL1B5	-5.6097693
TCP10C	-2.906824
TCRA	-3.0324306
TCRB-J	-2.2855804
TCSTV1	-1.9384701
TCTE1	-1.8718014
TCTE2	-6.394599
TCTEX1D1	-1.9659944
TDH	-3.3639479
TDPOZ3	-2.6679337
TDPOZ4	-2.374281
TDRD1	-4.9777064
TDRD6	-2.7649646
TDRD9	-2.896362
TECTA	-1.9458008
TEKT3	-3.6971133
TEKT5	-3.368927
TENM2	-5.9046416
TENM3	-2.7991965
TEPP	-1.255599
TET1	-1.5979631
TET3	-2.050164
TEX101	-6.907621
TEX13	-5.4187403
TEX13A	-4.6141834
TEX16	-1.5682628
TEX19.1	-3.3752258
TEX19.2	-3.5638638
TEX21	-4.900079
TEX24	-3.5027003
TEX29	-2.8497944
TEX33	-4.747443
TEX35	-2.8411229
TEX36	-5.116211
TEX37	-5.6394405
TEX38	-5.0595217
TEX40	-2.261105

TEX43	-4.3836803
TFAP2A	-2.6681814
TFAP2D	-2.7286723
TFAP2E	-1.1617308
TFAP4	-1.3728724
TG	-4.1022134
TGIF2	-2.7402077
TGIF2LX1	-2.605959
TGIF2LX2	-4.977434
TGM4	-3.3230991
TGM5	-2.5916085
TGM7	-6.3183303
TH	-0.9850004
THADA	-1.6480513
THEG	-4.504755
THEM5	-2.2398472
THEM6	-1.3192513
THEMIS	-3.0552227
THEMIS3	-3.9861858
THNSL1	-2.865372
THSD7A	-0.9874933
TIA1	-8.604101
TKTL1	-3.2992053
TKTL2	-4.8288608
TLL2	-3.0157447
TLR11	-3.2062953
TLR8	-2.624621
TLX1	-3.201826
TLX3	-4.0923853
TMBIM7	-2.6213799
TMC1	-4.3474674
TMC2	-4.3455467
TMCO2	-2.4564197
TMCO5B	-4.119493
TMED11	-4.332806
TMEM106A	-0.8072553
TMEM108	-1.9514151
TMEM130	-3.692221
TMEM132B	-3.6340666
TMEM143	-0.8982611
TMEM144	-0.7729971
TMEM150B	-2.2989395
TMEM150C	-4.6615186
TMEM151A	-1.092099
TMEM156	-1.9916048

TMEM163	-4.296758
TMEM168	-1.2075934
TMEM169	-5.5865335
TMEM179	-4.2037473
TMEM182	-5.913478
TMEM191C	-2.9852004
TMEM196	-3.6927316
TMEM198	-1.3578236
TMEM209	-1.3126674
TMEM210	-3.3004537
TMEM217	-3.8258157
TMEM218	-2.5199172
TMEM221	-4.9748025
TMEM225	-3.2724364
TMEM233	-4.137523
TMEM240	-2.0685482
TMEM247	-2.2423477
TMEM253	-3.4483173
TMEM26	-1.3671775
TMEM28	-0.9513736
TMEM30C	-1.1033056
TMEM51OS1	-2.1759858
TMEM52	-2.5939777
TMEM63C	-3.6615112
TMEM72	-4.2505608
TMEM74	-4.8388247
TMEM74B	-1.9064317
TMEM74BOS	-0.999383
TMEM95	-3.2762072
TMIGD1	-1.7022212
TMPRSS11C	-2.4592195
TMPRSS11D	-2.8142486
TMPRSS11E	-2.0869408
TMPRSS11G	-3.7689888
TMPRSS12	-4.4211807
TMPRSS5	-1.1534402
TMPRSS9	-3.4342728
TNC	-4.1507797
TNFRSF14	-0.7419527
TNFRSF8	-4.167822
TNFSF18	-3.7517452
TNK2OS	-6.9377394
TNMD	-1.1763206
TNNC2	-3.1491444
TNNI1	-5.5238056

TNNI3K	-3.4146607
TNR	-4.486164
TNS4	-1.1614542
TOPAZ1	-2.7857628
TOPORSL	-1.6327491
TPBPB	-3.4015777
TPCN2	-2.4450188
TPGS2	-1.9949524
TPH1	-2.700597
TPH2	-3.8834307
TPPP2	-3.480716
TPSG1	-4.645091
TPST1	-1.4059386
TPTE	-4.3757615
TRAF3IP1	-1.5421195
TRAIP	-3.7194953
TRANK1	-1.0761871
TRAV14N-1	-4.817217
TRAV3-4	-2.3799202
TRAV7N-5	-2.159534
TRAV9D-3	-1.003901
TRAV9N-2	-3.677767
TREX2	-3.1929352
TRGV2	-5.0786343
TRH	-1.6196017
TRIM15	-5.1929283
TRIM17	-2.3224058
TRIM30E-PS1	-0.7359676
TRIM31	-3.4738002
TRIM36	-1.2844193
TRIM42	-5.76684
TRIM45	-2.6572745
TRIM50	-4.0492163
TRIM55	-5.903928
TRIM58	-3.5645711
TRIM61	-6.2549453
TRIM66	-3.8188848
TRIM67	-3.1491926
TRIM7	-2.5030246
TRIM71	-4.5994053
TRIM9	-1.2948883
TRIML1	-4.5373425
TRIML2	-4.400958
TROAP	-2.8023453
TRP53TG5	-5.500961

TRPA1	-3.3461263
TRPC3	-2.4467843
TRPC4	-3.2966914
TRPC5	-4.2942624
TRPC5OS	-2.556087
TRPD52L3	-1.3257539
TRPM3	-1.9467561
TRPM5	-2.530611
TRPV3	-3.8881073
TRY5	-4.820259
TSC2	-4.015598
TSGA10IP	-0.8090446
TSGA13	-1.9505181
TSGA8	-1.3282061
TSHR	-3.624674
TSKS	-2.841648
TSPAN10	-3.1011875
TSPAN32	-4.612625
TSSK1	-4.552013
TSSK2	-1.5908527
TSSK3	-4.062133
TSSK5	-2.620297
TSSK6	-2.582741
TSX	-3.6587164
TTBK1	-2.4172835
TTC22	-2.8863013
TTC23L	-2.5178475
TTC39D	-5.32092
TTC8	-0.8870473
TTC9B	-3.255077
TTLL13	-4.75481
TTLL2	-1.377428
TTLL8	-2.0818107
TUBA3A	-3.4356117
TUBA3B	-4.4561963
TUBAL3	-1.0392985
TUBB3	-1.9569359
TULP2	-2.5016594
TXLNG	-0.6701098
TYMS	-0.961479
TYR	-2.63931
TYW3	-1.7467182
U90926	-2.7863386
UBAP1L	-3.1202276
UBE2DNL1	-4.7838535

UBL4B	-5.0111055
UBQLN3	-2.8831508
UBQLNL	-3.8367538
UCN	-2.676652
UCN2	-2.9353452
UCN3	-2.7563195
UCP1	-2.9498534
UFSP1	-1.9642184
UGT8A	-2.1822958
ULK4	-2.9303668
UNC13A	-3.4720116
UNC45BOS	-3.1198575
UNC5A	-3.3003895
UNC5D	-13.725019
UNC79	-2.6227424
UNC80	-2.8666427
UNCX	-2.0569894
UPK3BL	-2.9022987
UPRT	-0.71947
USH1C	-4.1170683
USH1G	-2.5141232
USH2A	-2.7733636
USMG2	-0.9737954
USP17LE	-3.8741841
USP26	-2.061039
USP27X	-2.8886583
USP29	-2.0030696
USP35	-2.817735
USP37	-2.3054094
USP44	-4.9939227
USP49	-2.1152043
USP51	-2.9586258
USP8	-0.6278999
USP9Y	-4.3144865
UTP14B	-3.7026083
UTP18	-1.6264732
UTS2	-3.6228168
UTS2B	-3.210935
UTY	-3.5074902
UVSSA	-4.3789864
V1RA8	-3.5192547
V1RD19	-2.330278
VANGL2	-1.0132167
VASH2	-4.3956623
VAX1	-4.5979853

VAX2OS	-2.3120189
VEZT	-2.0271237
VGLL1	-3.8687599
VGLL2	-6.2332544
VGLL3	-3.1977286
VIL1	-2.3582473
VIP	-5.7236814
VMN1R1	-4.4257526
VMN1R101	-3.6290407
VMN1R12	-3.3430753
VMN1R121	-1.3367324
VMN1R122	-3.4164352
VMN1R131	-3.239217
VMN1R132	-4.771061
VMN1R139	-2.7074738
VMN1R148	-4.716922
VMN1R157	-5.083062
VMN1R16	-3.448979
VMN1R167	-1.5132892
VMN1R168	-2.2045941
VMN1R17	-3.7138746
VMN1R171	-3.3590496
VMN1R172	-4.9310184
VMN1R174	-2.216755
VMN1R175	-2.7693677
VMN1R176	-1.8600326
VMN1R18	-2.4480293
VMN1R181	-3.0956473
VMN1R183	-1.8177819
VMN1R185	-4.33099
VMN1R188	-1.6376977
VMN1R189	-3.15523
VMN1R19	-2.6303859
VMN1R191	-2.717335
VMN1R194	-3.1988754
VMN1R196	-2.2583592
VMN1R198	-1.6582766
VMN1R199	-4.9107494
VMN1R2	-4.1478148
VMN1R20	-5.3083067
VMN1R200	-1.4010425
VMN1R201	-3.0279343
VMN1R202	-4.289192
VMN1R203	-2.0590205
VMN1R205	-2.4216614

VMN1R206	-3.0068643
VMN1R207-F	-3.528905
VMN1R209	-2.71053
VMN1R210	-4.131401
VMN1R211	-1.9326627
VMN1R212	-3.2162771
VMN1R214	-2.897503
VMN1R216	-2.5242686
VMN1R218	-1.6605515
VMN1R22	-3.234173
VMN1R223	-4.288166
VMN1R224	-4.000209
VMN1R225	-4.0263343
VMN1R226	-3.2207804
VMN1R227	-4.867984
VMN1R228	-2.414662
VMN1R230	-5.5199976
VMN1R231	-2.5951269
VMN1R234	-1.0052092
VMN1R235	-4.5041933
VMN1R236	-3.2909086
VMN1R24	-4.6463
VMN1R26	-2.252701
VMN1R27	-2.8942313
VMN1R28	-4.590542
VMN1R30	-1.8207545
VMN1R31	-3.3324723
VMN1R32	-3.7813933
VMN1R33	-4.6502924
VMN1R34	-6.8281927
VMN1R35	-1.671833
VMN1R39	-4.338155
VMN1R4	-2.1329153
VMN1R40	-2.030872
VMN1R41	-3.9528093
VMN1R42	-5.8230906
VMN1R45	-3.0329807
VMN1R47	-6.8755903
VMN1R51	-2.246312
VMN1R54	-3.9390361
VMN1R55	-3.0270677
VMN1R57	-2.7739732
VMN1R58	-3.000473
VMN1R59	-0.8582883
VMN1R6	-4.4321275

VMN1R62	-2.0523913
VMN1R63	-3.7641852
VMN1R64	-3.8000956
VMN1R66	-6.746436
VMN1R67	-5.8446145
VMN1R68	-1.3505466
VMN1R69	-2.7378783
VMN1R72	-3.278473
VMN1R73	-3.2846074
VMN1R74	-4.277013
VMN1R75	-6.01675
VMN1R76	-2.6860068
VMN1R8	-1.8983688
VMN1R80	-2.763537
VMN1R81	-3.6447082
VMN1R82	-2.5710723
VMN1R84	-5.755088
VMN1R85	-2.3553448
VMN1R86	-5.60122
VMN1R89	-2.8266892
VMN1R-PS79	-1.6453905
VMN2R10	-1.8434372
VMN2R100	-1.8619783
VMN2R101	-3.3168187
VMN2R102	-3.583321
VMN2R103	-3.9827516
VMN2R104	-4.494755
VMN2R106	-2.8755798
VMN2R107	-4.147086
VMN2R108	-2.3808331
VMN2R109	-2.8858757
VMN2R11	-1.4318252
VMN2R111	-3.8389733
VMN2R112	-3.0773816
VMN2R114	-4.0790615
VMN2R115	-3.5754466
VMN2R116	-2.4842184
VMN2R118	-6.758322
VMN2R12	-2.7390227
VMN2R120	-3.5418057
VMN2R121	-3.0477772
VMN2R123	-2.256913
VMN2R13	-2.1802409
VMN2R17	-2.836586
VMN2R20	-1.1661172

VMN2R27	-3.112913
VMN2R29	-1.2067659
VMN2R3	-2.8963654
VMN2R35	-3.2810595
VMN2R37	-2.548988
VMN2R39	-4.5794716
VMN2R42	-2.24144
VMN2R43	-1.7883964
VMN2R48	-4.467497
VMN2R5	-3.3208215
VMN2R53	-3.1051388
VMN2R54	-5.229656
VMN2R55	-2.917046
VMN2R57	-2.891217
VMN2R58	-4.394376
VMN2R60	-6.0621414
VMN2R62	-5.0139213
VMN2R65	-2.2615836
VMN2R67	-4.5748725
VMN2R68	-1.6600914
VMN2R7	-3.5693426
VMN2R71	-4.3546343
VMN2R72	-4.615489
VMN2R73	-4.4241266
VMN2R77	-6.6213408
VMN2R80	-5.972185
VMN2R81	-3.0639985
VMN2R82	-4.3073606
VMN2R83	-4.2942805
VMN2R85	-3.8316169
VMN2R86	-2.9309425
VMN2R89	-2.5459619
VMN2R90	-3.041712
VMN2R91	-4.522463
VMN2R96	-2.263112
VMN2R97	-1.0099461
VMN2R98	-3.793233
VMN2R99	-1.079102
VMN2R-PS1(	-3.3508534
VMN2R-PS12	-5.7780848
VMN2R-PS54	-4.0187902
VMN2R-PS57	-3.9950244
VPREB1	-2.8396635
VPREB2	-6.349286
VPS72	-2.2760448

VSIG1	-1.1930668
VSIG10L	-2.6786895
VSTM2A	-2.3129742
VSTM2B	-2.4867263
VSTM2L	-6.46608
VSTM5	-3.8842742
VSX2	-4.1824675
VTCN1	-3.2136483
VWA2	-4.3283973
VWA5B1	-4.513956
VWA5B2	-1.8585808
VWC2	-2.6131415
VWC2L	-4.0718403
VWDE	-3.654659
WAP	-3.4128385
WBP2NL	-2.9608934
WBSCR17	-2.7780035
WBSCR25	-3.5655975
WBSCR28	-2.4137979
WDHD1	-1.2884514
WDR17	-4.7665462
WDR20RT	-1.6397767
WDR27	-4.4325795
WDR49	-3.698693
WDR62	-2.7193315
WDR64	-3.9160402
WDR70	-2.4971538
WDR86	-2.2183156
WDR88	-1.383193
WDR93	-2.6724124
WEE2	-2.3392339
WFDC11	-5.116538
WFDC12	-4.5187464
WFDC15A	-7.3227525
WFDC15B	-3.2312849
WFDC16	-4.0619316
WFDC8	-3.3637204
WFIKKN2	-2.8721912
WIBG	-2.9137878
WISP3	-6.657363
WNK3	-3.0173237
WNT1	-0.9872904
WNT2	-3.2111895
WNT2B	-3.6170712
WNT3	-1.2996297

WNT8A	-2.7036633
WNT8B	-4.1932483
WNT9B	-3.015638
WSCD1	-4.350999
WT1OS	-2.7856107
XCR1	-2.1996856
XK	-3.0831857
XKR4	-3.736302
XKR5	-2.4291284
XKR7	-3.6139593
XLR5A	-3.3249748
XLR5B	-3.2194173
XLR5C	-2.9078395
XRCC6BP1	-5.0666876
XYLT1	-3.4600322
YIPF7	-2.6303785
YLPM1	-2.3401694
YPEL1	-2.05091
YPEL4	-3.7378528
ZAN	-3.4100416
ZAR1	-5.2515154
ZBTB7C	-4.7300544
ZC2HC1B	-5.344583
ZC3H8	-2.3392906
ZC3HAV1L	-3.0938945
ZC4H2	-3.0790505
ZCCHC13	-4.4118214
ZCCHC16	-4.975584
ZCCHC18	-3.149213
ZCCHC4	-0.8723304
ZCCHC5	-4.2934465
ZCWPW2	-3.0476713
ZDBF2	-1.7125268
ZDHHC11	-2.9777713
ZDHHC15	-4.35522
ZDHHC25	-6.267687
ZFHX2OS	-3.1789534
ZFP109	-1.4390852
ZFP11	-3.355174
ZFP114	-2.6923237
ZFP119A	-2.0193257
ZFP12	-2.4769506
ZFP13	-1.6208403
ZFP142	-1.441685
ZFP160	-3.489705

ZFP300	-5.3247747
ZFP334	-2.9748504
ZFP335OS	-0.9281817
ZFP345	-3.8089976
ZFP37	-3.5265281
ZFP385A	-0.772428
ZFP385B	-1.0974767
ZFP385C	-4.4143105
ZFP398	-1.1846752
ZFP42	-4.476367
ZFP420	-1.1075532
ZFP428	-2.0502794
ZFP438	-2.5723963
ZFP444	-3.5506296
ZFP454	-2.1495552
ZFP526	-2.4937625
ZFP563	-1.5031829
ZFP57	-1.245441
ZFP575	-2.3729348
ZFP605	-0.673945
ZFP609	-1.9507821
ZFP616	-4.1340556
ZFP619	-1.3065343
ZFP65	-0.652786
ZFP667	-1.8080015
ZFP69	-0.6564431
ZFP72	-1.3207982
ZFP735	-4.6772695
ZFP759	-2.1869323
ZFP775	-1.926678
ZFP781	-1.0378807
ZFP786	-6.114771
ZFP808	-6.2384768
ZFP820	-1.455569
ZFP827	-4.004676
ZFP85	-2.2598534
ZFP853	-1.4115298
ZFP85OS	-2.2617416
ZFP870	-2.1027403
ZFP874A	-3.8286786
ZFP879	-3.463534
ZFP882	-0.7438657
ZFP92	-1.4707344
ZFP933	-1.5589795
ZFP934	-2.500676

ZFP939	-4.4987464
ZFP94	-2.8277829
ZFP951	-2.7226257
ZFP957	-1.3011847
ZFY1	-2.526978
ZFY2	-4.293514
ZFYVE28	-4.7072086
ZG16	-3.8475864
ZGRF1	-3.4747798
ZIC2	-3.3998284
ZIC3	-3.7847877
ZIC4	-4.6860247
ZIC5	-4.294779
ZIM1	-2.684826
ZIM2	-1.5296988
ZKSCAN2	-4.134117
ZKSCAN4	-3.6323454
ZMAT4	-4.0787277
ZNRF4	-5.7916822
ZP1	-3.02876
ZP2	-1.8786795
ZP3	-3.4453373
ZP3R	-4.578809
ZPBP2	-2.2706604
ZPLD1	-3.3745394
ZRSR2	-1.3576281
ZSCAN10	-3.8858056
ZSCAN2	-2.2150884
ZSCAN30	-4.1065226
ZSCAN4A	-1.774503
ZSCAN4B	-2.3594007
ZSCAN4C	-3.6613264
ZSCAN5B	-3.52103
AAAS	1.250258
AAGAB	1.6448913
AAK1	1.2437992
AAR2	1.5388265
AARSD1	1.1765623
AATF	1.549612
ABCB10	1.3317461
ABCC1	2.1353612
ABCC10	1.3727727
ABCC3	3.5285363
ABCC4	1.1780343
ABCE1	2.3047943

ABCF1	1.8012266
ABCG8	0.93211484
ABHD11	1.8414669
ABHD13	1.4248827
ABHD17B	1.455709
ABHD17C	1.620534
ABHD2	2.071782
ABHD4	1.2959819
ABHD5	1.5236912
ABI1	1.6564136
ABL1	0.7843733
ABL2	0.911222
ABRA	0.77219987
ABRACL	1.6980815
ABTB1	0.9144678
ABTB2	2.8142228
ACACA	1.7134662
ACADSB	1.8232665
ACBD3	2.1448379
ACBD4	1.45333
ACD	1.5640059
ACER3	1.593914
ACIN1	1.3971095
ACKR1	6.453738
ACLY	0.85406256
ACO2	1.7384181
ACOT2	1.0917201
ACOT8	1.5348878
ACP2	1.6844859
ACRBP	1.8172398
ACSF2	1.8155699
ACTB	1.4572458
ACTG1	1.5025163
ACTR10	2.4778533
ACTR1A	1.2381463
ACTR3	1.5436492
ACTR5	0.62762165
ACTR6	1.0296516
ACTR8	1.2461076
ACVR1	1.5195208
ACVR1B	2.590454
ADAM17	1.277314
ADAM9	2.1899753
ADAMTS1	1.0805907
ADAMTS4	2.8655062

ADAP2	1.0187407
ADAR	1.7804394
ADAT2	1.7645335
ADCK2	1.0832419
ADCY1	3.607187
ADI1	1.548646
ADIPOR1	1.3667316
ADIPOR2	1.9447155
ADM	2.9323153
ADNP	0.84023
ADO	0.9877608
ADORA2A	2.4491367
ADPGK	2.261868
ADPRH	1.6000504
ADPRHL2	1.0169549
ADPRM	1.556418
ADRB2	1.8140187
ADRBK1	1.6208353
ADRBK2	1.209197
ADSS	0.8829632
ADSSL1	1.227798
AEBP2	1.5179214
AEN	2.4261208
AFF2	1.1817243
AFTPH	1.5927386
AGAP1	0.67808247
AGFG1	1.3663144
AGGF1	1.4335175
AGK	1.1113486
AGO2	1.395349
AGO3	1.1778519
AGPAT5	1.7909317
AGPAT6	2.1496663
AGPAT9	2.3100352
AHCTF1	0.67486095
AHCYL1	1.568572
AHSA1	2.2722735
AIFM2	1.0522888
AIMP1	1.6223149
AIMP2	2.2560034
AIP	1.2847738
AK2	2.2627902
AK6	2.0160503
AKAP10	1.0820088
AKAP11	1.1292353

AKAP12	3.1898398
AKAP7	0.9058871
AKAP8L	1.0303588
AKAP9	1.3505583
AKIP1	0.8915987
AKIRIN1	1.8377285
AKIRIN2	1.2878566
AKT2	1.873899
ALAS1	1.8288217
ALDH1A1	1.214911
ALDOA	1.2331386
ALG1	1.3960567
ALG10B	1.1560855
ALG13	1.6226094
ALG9	1.8289862
ALKBH1	2.109261
ALKBH4	0.88979435
ALKBH6	1.4844337
ALKBH7	0.7000456
ALKBH8	1.2260013
ALPK1	2.7262845
ALPL	1.8521595
ALS2CR12	1.9992054
ALYREF	1.9520655
AMBRA1	1.7883139
AMD2	0.8790412
AMDHD2	0.7948065
AMFR	2.3796425
AMIGO1	1.0334814
AMMECR1	1.1265562
AMMECR1L	2.12017
AMN1	1.4488931
AMPD2	2.173963
AMZ2	1.0210519
ANAPC1	1.1116571
ANAPC11	0.98392534
ANAPC15	1.0349607
ANAPC16	0.929893
ANAPC5	0.7880206
ANAPC7	1.3829207
ANGEL2	1.097691
ANGPT2	1.8184762
ANGPTL4	2.7977004
ANKFY1	1.5989141
ANKHD1	1.6466064

ANKIB1	1.516232
ANKLE2	0.6290288
ANKRA2	0.9493141
ANKRD11	1.6359963
ANKRD13C	1.7157936
ANKRD16	0.82539034
ANKRD17	1.5729661
ANKRD22	5.034899
ANKRD27	1.3440819
ANKRD28	1.4046273
ANKRD46	1.2923217
ANKRD49	1.8363986
ANKRD52	1.889893
ANKRD9	1.3653135
ANKZF1	2.0008135
ANO10	1.2705097
ANP32B	0.70823526
ANXA6	0.84204483
ANXA7	1.9786091
AOX3	0.7523246
AP1B1	1.7133203
AP1M1	1.5381889
AP1S1	1.2014332
AP2A1	1.3614969
AP2S1	1.396883
AP3B1	1.7749949
AP3D1	1.9415731
AP3S1	1.9777098
AP4B1	1.7267494
AP4S1	1.0612247
AP5B1	1.8146114
AP5M1	1.4600339
APAF1	2.1144776
APBB1IP	1.226161
APEH	1.5082808
APH1B	1.6636086
API5	1.25528
APLP2	1.0245953
APMAP	1.5941439
APOLD1	2.4407392
APOPT1	1.7415404
APPBP2	1.3981943
APPL1	1.5262637
APRT	1.4277539
AQR	1.2874918

ARAF	1.9719748
ARAP1	1.2983875
AREL1	2.094791
ARF4	2.643612
ARF5	1.0779424
ARF6	1.8438458
ARFGEF1	1.5119939
ARFIP2	2.0207071
ARG2	2.8394957
ARGLU1	1.2914205
ARHGAP17	1.2542272
ARHGAP26	1.7566476
ARHGAP42	0.9222741
ARHGDIA	1.2492528
ARHGEF12	1.0276823
ARHGEF15	1.4641285
ARID1A	1.126545
ARID1B	1.0199599
ARID4A	1.0624728
ARID5A	3.7783036
ARIH1	1.4552546
ARIH2	1.8676772
ARL1	2.3594904
ARL16	0.89378667
ARL4A	2.217959
ARL5C	1.7921724
ARL6IP4	0.8982816
ARL6IP5	1.4961772
ARL6IP6	2.018145
ARL8A	2.2929106
ARL8B	1.8364129
ARMC8	1.3553581
ARMCX5	1.0772142
ARNT	1.3138742
ARNTL	1.9061732
ARPC1A	0.9504733
ARPC1B	1.9371085
ARPC2	1.3432221
ARPC3	1.2726064
ARPC4	1.4588189
ARPC5	0.98877144
ARPC5L	0.8366046
ARPP19	1.1811554
ARRB2	1.7545576
ARRDC4	3.9845176

ARSA	1.1730523
ARSG	1.8593173
ARTN	1.2414763
AS3MT	1.8559241
ASAP3	1.3654456
ASB13	1.1834621
ASB4	2.147494
ASB6	1.4556603
ASB7	1.0578823
ASCC2	0.99363565
ASH1L	1.3166547
ASNS	2.3094916
ASNSD1	1.0657377
ASPRV1	4.2574534
ASUN	1.649929
ASXL1	1.1526484
ASXL2	0.7489438
ATAD1	1.8393812
ATAD2B	0.8435664
ATAD3A	2.3262997
ATE1	0.79451275
ATF1	1.4574051
ATF2	1.1681762
ATF3	4.1645656
ATF4	2.428388
ATF6	1.7532005
ATF7IP	1.2639074
ATG101	1.1222978
ATG12	1.2063689
ATG14	1.5014277
ATG16L1	2.1908393
ATG2B	1.3835092
ATG3	1.806428
ATG4B	1.6612608
ATG4D	1.6861696
ATG7	0.9277034
ATG9A	1.7670941
ATIC	1.1082749
ATP10D	1.7801251
ATP11A	1.8349886
ATP11B	1.5203834
ATP11C	2.0893135
ATP13A1	1.8761959
ATP13A3	0.93612385
ATP1A1	1.149662

ATP1B3	1.1621733
ATP2A2	1.5847449
ATP5G2	0.99579763
ATP5G3	2.516543
ATP6AP1	1.2064552
ATP6AP2	1.6306481
ATP6V0A1	1.4144139
ATP6V0A2	1.4545784
ATP6V0A4	3.346972
ATP6V0B	2.0083232
ATP6V0C	1.8897929
ATP6V1A	1.4274211
ATP6V1B2	1.5653911
ATP6V1C1	1.6082735
ATP6V1D	2.0947008
ATP6V1E1	1.613225
ATP6V1F	1.3522768
ATP6V1G1	1.1893225
ATP6V1H	2.169465
ATP8B1	2.7991338
ATP9A	1.7937818
ATP9B	0.94473815
ATR	1.2646623
ATXN10	1.0362124
ATXN2	1.5190511
ATXN2L	2.349214
ATXN7	2.0124903
ATXN7L1OS2	2.3892374
ATXN7L3B	1.8010192
AU017193	1.816819
AU019202	1.5483282
AU019823	1.3565912
AU040320	1.5564837
AU040972	1.4449787
AURKAIP1	1.6840205
AVEN	1.7894983
AVL9	1.7783093
AVPI1	1.1799803
AXIN1	1.0598836
AZI2	1.879756
AZIN1	1.4180493
B2M	1.5585384
B3GALNT2	1.8582177
B3GALT6	1.6160231
B3GNT2	1.2370725

B4GALT1	2.1183004
B4GALT5	1.4846735
B9D2	1.3097129
BABAM1	1.6476316
BACH1	2.2413678
BAD	0.8827567
BAG5	1.2422137
BAG6	1.42029
BAHD1	1.0564866
BAK1	1.5807009
BANF1	1.7662964
BAP1	2.526144
BATF	3.3163033
BATF2	2.8192844
BATF3	3.1917677
BAX	2.3578677
BAZ1A	1.2628074
BAZ2A	0.97736835
BBS9	0.87458897
BBX	0.8439996
BCAS2	1.5184526
BCAT2	1.1409192
BCCIP	2.6018581
BCL10	2.5660462
BCL2A1C	3.2369423
BCL2A1D	2.7446132
BCL2L1	2.414969
BCL2L13	0.9952736
BCL3	4.8442125
BCL6	1.8311315
BCL7B	1.3235826
BCLAF1	1.4608788
BCOR	1.0025959
BCR	1.3873072
BDP1	1.360796
BECN1	1.7568092
BEND3	1.5167546
BEST2	0.9566345
BFAR	2.33319
BHLHE40	2.3483891
BID	2.9501457
BIN3	1.0105443
BIRC2	4.5488963
BIRC3	2.724102
BIRC6	1.2058024

BLCAP	2.036344
BLMH	1.6236777
BLOC1S1	1.5841107
BLOC1S2	0.97629213
BLOC1S4	2.0858655
BLOC1S6	1.4883909
BLZF1	1.870543
BMP2	2.5733528
BMP2K	0.7696996
BMPER	1.2856245
BMS1	1.5489192
BNIP1	1.5411296
BNIP3	1.9726887
BNIP3L	0.714993
BOD1	1.279913
BOLA2	1.7775955
BOP1	2.1311617
BPIFC	1.7643328
BPTF	0.8099251
BRAF	1.1547842
BRAP	1.4394507
BRAT1	1.1051607
BRCC3	0.89519405
BRD1	1.0039032
BRD2	1.084063
BRD4	0.97093487
BRD7	0.8718281
BRD8	1.5188086
BRE	1.3688269
BRI3	0.98726416
BRI3BP	1.1943822
BRIX1	2.2741408
BRMS1	1.4130545
BROX	1.2333286
BRPF1	1.6469398
BRPF3	1.4055333
BRWD1	0.83552694
BST2	3.4108706
BTAF1	0.9512601
BTBD1	0.89103365
BTBD16	1.888207
BTF3	1.698863
BTF3L4	1.5025525
BTG1	1.259203
BTG2	2.6248994

BTN1A1	2.083898
BUB3	1.5154128
BUD13	1.18611
BUD31	1.9516048
BZW1	2.1098003
C1GALT1	1.492095
C1GALT1C1	1.2615261
C1QA	1.6404657
C1QB	1.6239867
C1QBP	2.2944613
C1QC	1.7620654
C1QTNF9	1.6535399
C1RA	3.1824574
C5AR1	1.83496
C6	0.9758997
CAAP1	1.8354666
CABLES2	1.2232099
CABP5	1.198906
CACTIN	1.6815071
CACUL1	2.2708154
CACYBP	2.2348032
CADPS	0.66185427
CALM1	1.1777372
CALR	2.4570813
CALU	2.022325
CAMSAP1	1.2773352
CAMSAP3	0.8281584
CAMTA2	2.3200326
CAND1	1.2683759
CANT1	1.2843983
CANX	1.7151651
CAP1	1.377779
CAPN7	1.3498673
CAPRIN1	1.0372534
CAPZA1	1.9894962
CAPZA2	1.7721186
CAPZB	1.636518
CAR13	2.5999556
CARHSP1	2.1741314
CARKD	1.2517776
CARM1	1.3635125
CARS	2.4366522
CASC3	1.3997498
CASP1	1.507772
CASP4	3.2127967

CASP6	1.276854
CASP7	1.927382
CASP8	2.3840208
CASP8AP2	0.952343
CBL	1.1728709
CBLB	1.4210019
CBLL1	0.97771835
CBWD1	1.3697464
CBX1	0.80255556
CBX4	0.9313979
CC2D1A	0.9473977
CC2D1B	1.0741515
CCAR1	1.7085948
CCBL1	3.006569
CCDC101	2.0342789
CCDC106	1.430021
CCDC115	0.78336906
CCDC117	1.795423
CCDC120	1.0173879
CCDC124	1.5259833
CCDC126	0.809597
CCDC132	1.688344
CCDC137	0.65341926
CCDC138	1.8498559
CCDC144B	1.2295775
CCDC163	1.6597433
CCDC22	1.4695077
CCDC3	0.9829478
CCDC43	1.3391101
CCDC53	1.533606
CCDC55	1.7578297
CCDC6	1.2977185
CCDC66	1.1814818
CCDC71L	2.3739228
CCDC77	0.8617058
CCDC80	1.0015264
CCDC82	1.2903271
CCDC86	2.5955691
CCDC88B	2.469676
CCDC97	1.2681761
CCL1	1.0904148
CCL19	3.0358367
CCL2	6.036657
CCL3	5.719592
CCL4	4.430876

CCL5	1.3589654
CCL7	4.879653
CCM2	1.1940084
CCNC	0.7201812
CCNDBP1	0.66643953
CCNE1	1.226943
CCNH	1.3098931
CCNI	1.0050635
CCNK	1.8325853
CCNL1	2.861638
CCNL2	2.1979432
CCNT1	1.9562607
CCNT2	1.5060725
CCNYL1	1.5333266
CCP110	1.9631982
CCPG1	0.9035473
CCPG1OS	1.0212564
CCR9	0.91664433
CCRL2	2.317933
CCRN4L	2.2377234
CCT2	2.0320244
CCT3	2.5132627
CCT4	2.0327616
CCT5	1.525362
CCT6A	2.3012352
CCT7	1.5751915
CCT8	2.3742785
CCZ1	1.3645816
CD14	6.529488
CD164	1.7647924
CD274	3.027197
CD300LG	1.2023201
CD33	3.1591215
CD38	3.020606
CD3EAP	1.5243421
CD40	2.4682698
CD44	1.0964503
CD47	2.195475
CD48	1.0124125
CD55	1.6650519
CD68	0.9526658
CD69	3.4387627
CD72	1.3115101
CD74	1.5408154
CD82	1.6175828

CD86	1.2704403
CDC123	1.9569159
CDC16	1.2942023
CDC26	0.9613061
CDC37	1.5623918
CDC37L1	1.8568087
CDC42	1.3695054
CDC42EP2	2.6403556
CDC5L	1.1946707
CDC73	1.3355598
CDCA4	1.4946113
CDIPT	0.8683357
CDK10	1.2706685
CDK11B	1.6146822
CDK12	1.1990232
CDK13	0.7020538
CDK17	2.3615098
CDK18	1.8746958
CDK2	1.0588434
CDK2AP1	1.0060987
CDK4	1.003829
CDK5R1	1.3896306
CDK7	2.1423678
CDK8	1.3545804
CDK9	1.3393927
CDKAL1	1.0686827
CDKN1A	3.1498032
CDKN1B	1.2232575
CDKN2AIP	1.4319584
CDKN2AIPNL	1.7667594
CDYL	1.1091893
CEBPB	3.3846107
CEBPD	3.6766734
CEBPG	2.697423
CEBPZ	2.4776564
CEBPZOS	0.63767576
CECR5	2.8396628
CELF1	1.9534101
CELSR3	1.9008553
CENPJ	0.68346643
CENPL	1.717278
CENPO	1.044719
CENPW	0.6470442
CEP120	0.95496917
CEP162	1.1417689

CEP170	1.2396064
CEP170B	1.9218326
CEP350	1.9076447
CEP57	1.1777935
CEP63	1.5075135
CEP95	1.8129544
CEPT1	2.0501719
CERS2	1.4997888
CERS6	2.5813293
CETN3	0.88762236
CFDP1	1.7843595
CFL1	1.4677305
CFL2	1.5052915
CFLAR	2.353026
CGGBP1	1.0631213
CH25H	3.922731
CHAMP1	1.5093791
CHCHD1	2.1385608
CHCHD2	2.2291822
CHCHD4	2.3364835
CHCHD6	1.154933
CHD1	1.5902588
CHD2	1.1314182
CHD4	0.9491868
CHD7	2.46459
CHD8	1.6696224
CHD9	0.84771585
CHERP	1.7237868
CHFR	1.3751321
CHIC2	1.9471164
CHIL1	2.4854746
CHKA	2.7545128
CHKB	1.890686
CHM	1.4970102
CHMP1A	1.0947323
CHMP2A	1.8327236
CHMP2B	1.3739667
CHMP3	1.3371468
CHMP4B	1.3788004
CHMP5	1.1569366
CHMP6	1.3035064
CHMP7	1.4676871
CHORDC1	1.9994264
CHP1	1.5517821
CHPT1	0.8510866

CHRAC1	1.7098408
CHRM3	3.508911
CHST15	1.2906256
CHTOP	1.7383046
CIAPIN1	2.1927524
CIC	0.9953108
CIDEC	1.2985597
CIRH1A	2.1754947
CISD2	1.2111135
CISH	3.536919
CKS1B	1.2619829
CLASRP	1.1501613
CLCC1	1.6134725
CLCN4-2	2.0891151
CLCN7	1.6316051
CLCNKB	0.7300303
CLDN25	1.4590006
CLEC4A1	2.0523348
CLEC4D	3.3222904
CLEC4E	5.018941
CLEC4N	1.1640897
CLHC1	1.1142633
CLIC1	1.3963499
CLINT1	1.7481122
CLIP1	1.4471059
CLK1	1.75354
CLK2	1.2911763
CLK3	1.0675154
CLK4	2.1182418
CLMN	1.7776213
CLN5	1.977531
CLNS1A	1.576468
CLOCK	1.1920867
CLPTM1	1.7510767
CLTA	1.5498052
CLTB	1.9873004
CLTC	1.4629803
CMIP	1.6214261
CMPK1	1.9490681
CMPK2	1.6954398
CMSS1	0.9639473
CMTM6	1.3799119
CMTM8	0.79346514
CMTR1	1.932096
CMYA5	1.7499981

CNBP	1.4944363
CNDP2	1.402472
CNEP1R1	1.2733293
CNIH1	2.5574832
CNIH4	1.1174846
CNKSR3	2.479186
CNN3	2.0574608
CNNM2	1.4659896
CNNM3	1.1789842
CNNM4	1.2713051
CNOT1	1.1899304
CNOT10	1.1193094
CNOT2	1.0960336
CNOT4	1.6959257
CNOT6	0.9993682
CNOT6L	2.1856136
CNOT7	1.0525994
CNOT8	0.90004873
CNPPD1	1.2490826
CNPY3	1.9483733
CNR2	0.87910366
COA3	1.3698955
COA5	1.4386406
COG1	0.6716361
COG2	1.9206738
COG4	0.91461515
COIL	1.3204198
COL17A1	1.8492827
COMMD10	1.2046242
COMMD4	1.3484511
COMMD7	1.1939473
COMTD1	1.8723245
COPS2	2.1453037
COPS4	2.239335
COPS7A	1.7300663
COPS8	1.910615
COQ10A	0.81943464
COQ10B	2.4123669
COQ2	1.6089783
COQ5	2.116664
CORIN	1.117295
CORO1B	1.155643
CORO7	1.0646281
COX11	2.0980768
COX14	1.019454

COX19	2.0451365
COX2	1.6139269
COX4I1	1.6655965
COX6C	2.096024
COX7A2L	1.0809422
CPEB2	1.2954636
CPEB4	1.7824631
CPNE3	1.0582304
CPSF1	1.8225894
CPSF2	1.7860799
CPSF3	1.6171355
CPSF3L	1.4937444
CPSF4	1.7101846
CPSF6	1.5776958
CPSF7	1.7310319
CR1L	1.5271273
CRAMP1L	1.1222677
CRBN	1.6462836
CREB5	1.9641981
CREBBP	1.3659277
CREBL2	1.103693
CREBZF	1.2134471
CRIP3	1.0844889
CRIPT	1.3733821
CRK	1.9467292
CRLF2	1.3836045
CRTC1	0.8152108
CRTC2	2.375105
CRTC3	1.3000765
CRY1	2.2494187
CRYZL1	1.1926966
CSDE1	1.2580643
CSE1L	1.9644179
CSF1	2.8987136
CSF1R	1.0478721
CSF2RA	1.9481878
CSF2RB	2.5957346
CSF2RB2	2.2263346
CSF3R	1.9103594
CSNK1A1	1.568572
CSNK1D	1.5339651
CSNK1G1	1.265027
CSNK1G2	0.79881
CSNK1G3	0.97941685
CSNK2A1	1.6031194

CSNK2A2	1.3440485
CSNK2B	1.6337824
CSPP1	1.3658845
CSPRS	0.9618249
CSRNP1	3.5877957
CSRP2	1.6299782
CSRP3	2.5284886
CSTF1	1.9973445
CSTF2	1.0247867
CSTF2T	1.8518629
CSTF3	1.9093552
CTCF	1.0455222
CTDNEP1	1.5738206
CTDP1	1.8490591
CTDSP1	1.4306388
CTLA2B	1.060802
CTNNB1	1.0555081
CTNNBL1	1.2334132
CTNND1	0.9072175
CTPS2	1.1643496
CTSA	2.4683347
CTSB	2.2591586
CTSC	1.7853441
CTSD	1.20642
CTSH	1.0346847
CTSO	1.5191371
CTSS	0.83475494
CTSZ	2.2793827
CTTN	1.5080843
CTU2	1.9067187
CUL1	1.8782301
CUL2	1.7823639
CUL3	1.4255309
CUL4A	1.9556203
CUL4B	1.6984258
CUL5	1.3983016
CWC15	1.5986247
CWC22	1.4308677
CWC25	1.4880047
CWC27	1.2242513
CXCL1	5.365581
CXCL10	4.8744035
CXCL11	1.6523962
CXCL2	8.263842
CXCL3	3.6419559

CXCL5	2.840157
CXCL9	4.479806
CXXC1	1.90379
CYB561	1.3449016
CYB561D2	1.9961042
CYB5R4	1.4758544
CYBB	2.162806
CYCS	2.3070683
CYFIP1	1.0401373
CYP20A1	0.8114331
CYP2U1	1.9722457
CYP7B1	3.2050166
CYPT4	2.6388197
CYSLTR2	1.3316619
CYTH1	1.433919
CYTH2	1.2468538
CYTH4	1.1118126
DAD1	2.1798224
DANCR	2.1229892
DAP3	2.0527067
DAPK3	1.2533295
DAXX	2.112113
DAZAP1	1.273613
DAZAP2	0.934916
DBN1	2.0407915
DBNL	1.5606089
DBR1	1.0026531
DCAF10	1.4626782
DCAF12L2	1.9218478
DCAF13	1.928433
DCAF6	0.9939711
DCAF8	1.1919675
DCC	3.539279
DCP1A	1.375854
DCP1B	1.0924234
DCTN2	1.0874448
DCTN3	1.4528637
DCTN4	0.73325634
DCTN5	1.2786217
DCUN1D1	1.1754656
DCUN1D2	1.3574953
DCUN1D5	1.7747402
DDA1	1.5314517
DDB1	1.9408827
DDHD1	1.9366417

DDI2	2.6316586
DDIT4	2.2749934
DDX1	2.3334913
DDX10	1.790463
DDX17	1.4723296
DDX18	1.7432637
DDX20	1.4195614
DDX24	1.5856414
DDX39	2.1122103
DDX39B	1.4795141
DDX3X	1.835401
DDX41	1.576747
DDX42	1.2370348
DDX46	0.97793627
DDX47	1.1867342
DDX5	1.541081
DDX50	2.0339928
DDX51	1.1663136
DDX52	1.6433339
DDX54	2.0225968
DDX58	1.7575588
DEDD	0.95782757
DEDD2	1.8477712
DEGS1	1.5746813
DENND1A	0.94304633
DENND1B	0.9465771
DENND2D	1.3609638
DENND4A	1.9410176
DENND5B	1.4424229
DENND6B	1.5880573
DERL2	2.2092485
DESI2	2.3240304
DFFB	1.1254168
DGAT1	1.5044637
DGCR14	1.0040135
DGCR2	0.7702751
DGCR8	1.0322764
DGUOK	1.218586
DHPS	1.1636486
DHRS7B	0.76231766
DHX15	2.4359674
DHX16	1.0981731
DHX29	1.0326476
DHX30	1.5043569
DHX33	1.1577411

DHX35	0.94836664
DHX36	1.2826443
DHX38	1.8988347
DHX40	1.8168654
DHX58	1.6179304
DHX8	0.7790127
DIABLO	1.978704
DIAP2	1.0181179
DICER1	0.87518406
DIDO1	0.7936888
DIEXF	1.9597063
DIMT1	2.008214
DIP2C	0.8356862
DIS3	1.8192797
DIS3L	1.4159188
DIS3L2	0.90120554
DKC1	1.8286314
DLGAP4	1.7245798
DLST	1.9984331
DMAP1	1.3845501
DMPK	1.1605363
DMTF1	2.5382593
DMTN	1.5848956
DMWD	1.3614388
DMXL1	1.6969879
DMXL2	1.82265
DNA2	0.84828925
DNAIC1	0.7996187
DNAJA1	2.5282536
DNAJA2	2.0523748
DNAJB1	1.7630978
DNAJB14	0.97231054
DNAJB2	2.4438114
DNAJB6	1.7286439
DNAJC1	1.5133176
DNAJC13	1.8956695
DNAJC15	1.9110937
DNAJC18	1.0468316
DNAJC24	1.2145333
DNAJC30	1.6196184
DNAJC5	1.5690274
DNAL4	0.80384636
DNLZ	2.1409364
DNM1L	1.2193375
DNM2	1.6740384

DNTTIP1	1.1151042
DNTTIP2	1.8880296
DOC2G	0.6971965
DOCK4	1.435049
DOCK5	1.802011
DOHH	1.1713805
DOK2	2.095448
DOK3	1.3485107
DOLK	0.8038573
DOLPP1	1.4754591
DONSON	2.364174
DOPEY2	1.3649979
DOS	1.1435051
DOT1L	1.5884714
DPCD	0.7247343
DPF2	1.6844115
DPH2	2.0214128
DPH7	0.9629619
DPM1	1.537909
DPM2	1.5484953
DPP6	1.7014809
DPP8	1.3184495
DPY30	0.91723394
DR1	1.8987103
DRAM2	1.3110552
DRG1	2.2044535
DSCR3	1.050951
DSE	1.4410048
DST	1.2245326
DSTN	1.6305676
DTNBP1	1.6163402
DTX2	1.8946018
DTX3L	2.0342607
DTYMK	1.2440681
DUS2	2.2019758
DUS3L	1.6336923
DUSP11	1.3256149
DUSP16	2.8392477
DUSP2	3.7428002
DUSP8	2.3393917
DVL1	1.8014717
DVL3	0.8513508
DXERTD223E	0.6902778
DXO	1.6411276
DYNC1H1	1.5806093

DYNC1I2	0.91001844
DYNC1LI1	1.0375638
DYNLL2	1.1699781
DYNLRB1	1.2241206
DYNLT3	0.87600803
DYRK1A	1.7041216
DYRK2	1.1188974
E2F5	1.4188452
E2F6	1.2695737
E4F1	1.1617112
EAF1	1.5870843
EAPP	1.919219
EBAG9	1.6496935
EBI3	3.342698
EBNA1BP2	1.7393198
ECD	1.5451503
ECM1	1.6016479
EDC4	1.5005951
EDRF1	1.7126944
EEA1	1.4242659
EED	1.6485338
EEF1A1	1.4689322
EEF1B2	1.6727209
EEF1D	1.5995111
EEF1E1	2.339303
EEF1G	1.4412675
EEF2	1.8640308
EFCAB14	1.0738616
EFHD2	1.8048244
EFR3A	1.21596
EFTUD2	1.2546921
EGFL8	0.9999566
EGLN2	1.343637
EGR1	3.327736
EGR2	1.7729168
EHD1	2.708778
EHD3	1.1920776
EHMT1	1.881402
EHMT2	1.2379541
EIF1	1.7533898
EIF1A	3.1069016
EIF1AX	1.2688937
EIF1B	1.335177
EIF1-PS	1.855197
EIF2A	1.740974

EIF2AK2	2.4909716
EIF2AK4	1.2013133
EIF2B1	3.2056804
EIF2B2	1.8850646
EIF2B3	1.9753065
EIF2B5	1.3408556
EIF2S1	1.7851481
EIF2S2	2.4930916
EIF3A	2.127636
EIF3B	1.8168793
EIF3C	1.7761722
EIF3D	2.2307997
EIF3E	1.3616633
EIF3F	1.3650517
EIF3G	2.1783094
EIF3H	0.912025
EIF3I	1.6439261
EIF3J1	1.6091857
EIF3K	0.9957876
EIF3L	1.371491
EIF3M	1.6722493
EIF4A1	2.2753263
EIF4A2	1.22784
EIF4A3	1.327971
EIF4B	1.5031157
EIF4E	1.4834089
EIF4E3	1.3759341
EIF4ENIF1	1.6911836
EIF4G1	2.6298285
EIF4G2	3.2314749
EIF4G3	1.1777444
EIF4H	1.5138102
EIF5	1.845974
EIF5A	2.1783466
ELAVL1	1.8871851
ELF2	1.694768
ELF3	2.7178297
ELK1	1.495595
ELL	2.7104626
ELOF1	1.2251029
ELOVL5	1.4001598
ELOVL7	1.5500517
ELP5	1.7660022
EMC1	0.85623026
EMC2	1.563334

EMC3	1.2788091
EMC4	1.4352727
EMC6	2.029089
EME2	0.90381336
EMG1	2.056386
EMILIN1	1.335269
EMILIN2	2.3659315
EML3	0.6493244
EML4	1.2666545
EML6	1.6894138
EMR4	1.8995214
ENC1	2.9674635
ENO1	1.7510386
ENOX2	0.6305113
ENPEP	0.9251189
ENPP2	0.97925854
ENPP4	2.2136526
ENSA	0.95215416
ENTHD2	1.9194727
ENY2	2.2807522
EP400	1.4594979
EPB4.1	1.2445965
EPB4.1L3	0.6970539
EPC2	1.0390496
EPHA5	1.8861337
EPHA8	1.1250756
EPHB2	1.1951714
EPHB3	1.7541714
EPM2AIP1	1.1392384
EPN1	2.4227915
EPPK1	2.9574594
EPS15	0.88341427
EPS15L1	1.3328557
EPT1	1.5912967
ERAL1	1.814107
ERAP1	2.095172
ERBB2IP	1.1416659
ERCC2	1.4616175
ERCC3	1.2612972
ERCC4	0.7048478
ERCC5	1.281836
ERCC6	1.4695673
ERCC8	1.1310122
ERDR1	1.4007702
ERGIC2	2.2451525

ERH	1.9255824
ERI1	1.1335399
ERLIN1	2.0444736
ERLIN2	1.073781
ERMN	3.3003273
ERMP1	1.0699553
ERO1L	2.1684837
ERP29	2.3204284
ERP44	1.8490844
ERRFI1	4.1874
ESCO1	1.2509608
ESF1	1.6264496
ESR2	1.4342871
ESRRA	1.2331848
ETAA1	0.89358735
ETF1	2.3958778
ETNK1	1.1556811
ETS2	2.383676
ETV3	1.463789
ETV6	3.249011
EVI5L	0.89015627
EXD1	1.7587576
EXD2	2.212647
EXOC3	0.77058315
EXOC3L4	1.4888272
EXOC5	1.3405857
EXOC6	0.6253128
EXOC6B	1.4098353
EXOC7	0.983325
EXOSC2	1.8427286
EXOSC3	2.2178068
EXOSC5	1.1760974
EXOSC6	1.5689483
EXOSC7	0.9329982
EXOSC9	1.7577767
EXTL3	1.4365592
EYA1	1.9971194
EYA3	2.653934
EZH2	1.2023921
F10	5.3397384
F11R	1.6683283
FABP4	1.7748704
FAF1	1.328341
FAF2	2.349309
FAM103A1	1.000473

FAM104A	1.5745325
FAM107A	1.7070012
FAM114A2	1.0784287
FAM118A	1.8655949
FAM118B	1.2008829
FAM120A	1.6703534
FAM120B	1.323286
FAM122A	1.7291231
FAM129C	1.0833039
FAM132A	1.2594223
FAM134C	0.83222675
FAM151B	1.0843031
FAM160A2	1.3100939
FAM160B1	1.0498071
FAM160B2	1.5460792
FAM167B	1.8205228
FAM168B	1.2954268
FAM169B	2.476645
FAM173A	1.2350965
FAM178A	2.7354217
FAM188A	1.7992144
FAM192A	1.7968268
FAM193A	1.4663968
FAM193B	1.4107509
FAM195B	0.85245514
FAM199X	2.8560383
FAM204A	1.1827359
FAM206A	1.0147729
FAM207A	1.3978176
FAM208A	1.1822686
FAM208B	2.1045823
FAM21	1.6192365
FAM210A	1.7034342
FAM214B	1.2356615
FAM32A	1.3476391
FAM49A	0.7822795
FAM50A	2.498942
FAM53A	1.2368326
FAM53C	1.7622356
FAM58B	0.785357
FAM63A	1.2961211
FAM71D	2.9986968
FAM76A	0.803452
FAM76B	1.0678096
FAM83G	0.9703784

FAM92A	1.7533367
FAM96A	1.6568289
FAM96B	2.120339
FAM98B	1.5549302
FANCE	1.0561781
FAP	2.435192
FARP1	1.2151723
FARSA	1.6233659
FARSB	2.2435274
FAS	3.616722
FASTKD1	1.6534169
FASTKD3	2.3526735
FAU	1.9461269
FBL	2.2569165
FBRS	1.4430141
FBRSL1	1.1365948
FBXL12	1.2039356
FBXL12OS	1.9737582
FBXL15	2.0154152
FBXL3	1.1903567
FBXL5	2.548029
FBXL6	0.9543915
FBXO3	0.7730417
FBXO33	1.4610887
FBXO38	1.6828718
FBXO4	1.5471315
FBXO42	1.898489
FBXO45	1.3322854
FBXO46	1.6511512
FBXO6	2.6114416
FBXO7	1.7072601
FBXW11	1.6507773
FBXW4	1.1485271
FBXW5	1.6163607
FBXW7	0.6534486
FBXW8	1.406795
FCER1A	1.7656105
FCER1G	2.3484263
FCF1	1.6398525
FCGR2B	2.481648
FCHSD2	1.7021418
FDX1	1.8589673
FDX1L	1.7141829
FEM1A	2.103004
FEM1C	1.2141876

FEN1	1.4085007
FERMT2	1.1472912
FERT2	1.480453
FEZ2	1.4992437
FGD3	1.4235768
FGD4	1.2308841
FGFR1OP	1.4042816
FGFR1OP2	1.0712814
FGFR2	1.1176772
FGFRL1	0.8809571
FGR	1.8545475
FIP1L1	1.5897632
FIS1	1.6599393
FITM2	1.6000986
FKBP1A	0.8865943
FKBP3	1.1889267
FKBP5	3.5005589
FKRP	1.4609857
FLCN	2.1179967
FLNB	2.6495967
FLOT1	2.1487107
FLOT2	1.843926
FMNL2	1.7568879
FMO3	1.5793018
FMR1	0.94490576
FNBP4	1.7120574
FNDC3A	2.5064917
FNDC3B	2.995575
FNIP1	1.7054586
FNIP2	1.4562359
FNTA	0.7846031
FOCAD	2.0449147
FOPNL	0.85032845
FOSL1	3.3653264
FOXC2	1.8997374
FOXJ3	1.0686011
FOXK2	1.6241183
FOXL2	1.5337148
FOXN2	1.7670965
FOXO1	1.2234616
FOXP4	1.7307329
FOXQ1	2.1311731
FPR1	4.144503
FPR2	3.8984199
FPR3	4.5699215

FRA10AC1	1.7372303
FRAT1	1.0454788
FRG1	0.9741039
FRMD8OS	1.4781404
FRS2	1.0810871
FRYL	1.5351267
FSCN1	2.1114826
FTH1	2.0229979
FUBP1	1.222614
FUBP3	1.485076
FUCA1	1.2248702
FUK	1.0052981
FUNDC1	1.2552209
FURIN	2.0634556
FUS	1.2795529
FXR1	1.6904664
FXR2	2.02282
FYB	1.8606043
FZD9	1.972301
FZR1	1.0916471
G3BP1	2.2348595
GAA	1.2966204
GAB3	3.6306834
GABARAP	2.1134315
GABARAPL1	1.5712523
GABPB1	1.9792862
GABPB2	0.8478341
GADD45A	1.5899138
GADD45B	2.7043138
GADD45G	4.3793187
GAK	1.3439412
GALK2	1.2980423
GALNS	1.7637043
GALNT11	1.4247041
GALNT2	0.78974533
GALNT4	0.8851361
GALNT7	1.0572033
GANAB	2.3771129
GAPDH	2.4485188
GAPVD1	1.5257616
GAR1	2.3225622
GARS	1.6388454
GAS2L1	0.9588399
GAS5	1.6173716
GAS6	1.1179333

GATA5	1.4395449
GATAD1	1.1410322
GBA	3.0942163
GBP10	3.2315884
GBP2	2.5244002
GBP2B	2.6178026
GBP3	2.912171
GBP5	3.3242822
GBP6	4.1454525
GBP7	2.993876
GBP8	1.5849423
GBP9	2.2511668
GCA	0.88983846
GCC1	1.7411432
GCH1	4.0471363
GCLC	2.2280927
GCM1	2.7314315
GCN1L1	1.3913698
GDA	1.9317174
GDAP10	3.4052563
GDAP2	2.1879683
GDE1	1.2144165
GDI1	1.0803285
GDI2	1.3836217
GEM	2.128356
GEMIN5	1.0537925
GEMIN6	1.8338675
GET4	0.9120574
GFER	3.510365
GGA1	1.209857
GGA3	1.7676735
GGCT	2.380005
GGH	0.9387207
GGNBP2	1.5124083
GHDC	1.4407315
GID8	1.286006
GIGYF1	0.8868246
GIGYF2	1.5974574
GIMAP9	2.020009
GINM1	1.5513916
GINS3	1.0949364
GIT2	1.0755277
GKAP1	1.1415644
GLA	1.169796
GLG1	1.3012938

GLIPR2	2.3265772
GLRX	3.0610142
GLRX2	1.679472
GLRX3	1.6015925
GLT8D1	1.5216737
GLTSCR2	0.7533212
GLUL	2.441484
GLYR1	1.7615438
GM10015	1.5585017
GM10065	1.5976717
GM10101	0.9125478
GM10134	0.96846056
GM10204	1.3814173
GM10405	1.3447161
GM10420	1.6315022
GM10631	0.93494797
GM10634	4.310249
GM10636	0.91830826
GM10653	1.6712885
GM10693	2.067287
GM10765	1.3351717
GM10767	0.81957054
GM10796	1.484297
GM10839	1.8763943
GM10845	1.7844143
GM10856	1.2891698
GM10872	3.489748
GM10910	0.8379035
GM10914	1.1590436
GM11127	1.7349725
GM1141	1.5272126
GM11449	0.9520135
GM11538	0.81923795
GM11559	1.2978003
GM11696	1.3493428
GM11810	0.9720826
GM11974	2.0877519
GM12070	2.402079
GM12216	0.93692446
GM12250	2.1568942
GM12447	1.5856285
GM12508	1.7278819
GM12618	1.5740156
GM12693	1.4715486
GM12758	1.26865

GM12816	1.4289412
GM12942	0.88116074
GM1305	2.009059
GM13215	1.0209594
GM13570	2.135926
GM13770	1.6186619
GM13777	1.1594582
GM13826	1.0433645
GM14010	4.270259
GM14135	0.89928913
GM14378	0.9223523
GM14432	1.5290303
GM14548	2.0092845
GM14635	1.2080464
GM15056	2.8317652
GM15348	2.052444
GM15421	1.407659
GM15645	2.7675533
GM15772	2.0176783
GM15998	1.4184008
GM16340	2.2717252
GM16381	1.3586102
GM16853	1.6878574
GM17745	1.5296154
GM17748	1.1792407
GM17757	1.8541958
GM17821	1.9038982
GM1943	1.2571502
GM19840	0.96888256
GM2016	2.9490209
GM2099	1.0341082
GM2270	0.9087181
GM2347	0.7344215
GM2382	2.07728
GM2436	1.5863876
GM2590	1.5441105
GM3285	1.5393951
GM3414	1.7029066
GM3527	1.0801651
GM4123	2.6737292
GM4130	1.0562172
GM4342	1.0483999
GM4470	0.8536668
GM4559	0.8540957
GM4583	1.3683281

GM4786	1.5936394
GM4802	1.7023115
GM4832	1.4560633
GM4841	1.3015976
GM4861	3.34787
GM4889	1.2365742
GM4951	3.208313
GM4963	1.4168429
GM4969	1.0982461
GM4971	2.3728724
GM4997	1.6141224
GM5069	2.3379307
GM5079	0.74386597
GM5136	1.2902222
GM5150	0.8274417
GM5176	1.3455477
GM5396	1.2876043
GM5445	1.2644496
GM5453	1.5546169
GM5483	3.9884377
GM5523	2.3356247
GM5528	0.9228091
GM5595	0.91332626
GM561	0.89133024
GM5637	3.2265925
GM5766	1.9188123
GM5779	0.8763027
GM5785	1.1130784
GM5848	0.9632158
GM5878	0.9671359
GM5879	1.1970282
GM5908	1.8050828
GM6034	1.5157528
GM6109	1.3434472
GM6115	1.2756472
GM6155	1.1976609
GM6211	1.789432
GM6251	1.1321917
GM626	1.7937093
GM6328	1.4520869
GM6377	4.0831184
GM6403	2.8250728
GM6404	1.1287518
GM6498	1.9572959
GM6524	2.2379193

GM6525	1.1313481
GM6548	1.7533822
GM6570	1.3014159
GM6578	1.6674514
GM6579	1.2356901
GM6607	1.3677082
GM6904	1.066916
GM6994	1.8599138
GM7111	2.416956
GM7241	1.0786483
GM7265	1.761251
GM7337	2.213358
GM7555	1.2078252
GM7634	0.86778307
GM7664	1.2136688
GM7696	0.8641927
GM7967	2.1761556
GM807	1.2365477
GM8154	0.6902211
GM8221	1.5856781
GM831	0.6650646
GM8526	0.84950924
GM8580	1.8169222
GM8709	2.6300893
GM8801	2.53997
GM8884	1.4312348
GM8909	3.0455484
GM8994	2.2886138
GM8995	2.3709316
GM9049	1.2231598
GM9079	2.132545
GM9178	2.4174066
GM9234	1.3529735
GM9385	1.6890302
GM9456	1.4949007
GM9457	1.7292914
GM9693	1.2259817
GM9706	3.4926805
GM9920	1.2771139
GMCL1	1.3432536
GMDS	0.88866806
GMEB1	1.2468414
GMEB2	1.3109322
GMIP	1.7642121
GMPR2	1.4456892

GNA13	2.012714
GNAI3	2.2862563
GNAS	1.2866106
GNB2	1.9671655
GNB2L1	1.6143618
GNG12	2.3616843
GNG5	1.7071576
GNL2	2.4232535
GNL3	3.255774
GNPAT	1.3540516
GNPDA1	0.8125529
GNPTG	1.2951441
GNS	1.3697095
GOLGA1	1.9110055
GOLGA3	2.5200744
GOLGA4	2.0541635
GOLGA7	1.411521
GOLGB1	2.3746758
GOLPH3	2.548564
GOLPH3L	1.0804706
GOLT1B	1.4896898
GOPC	1.3269529
GORAB	1.5469372
GOSR1	1.8052282
GOSR2	1.6951246
GP49A	3.274694
GPAA1	1.1720848
GPATCH11	1.4976006
GPATCH2L	1.6501384
GPBP1	1.4755454
GPBP1L1	0.8472433
GPI1	1.919826
GPKOW	1.6684704
GPN1	1.0939646
GPN3	0.9721513
GPR107	1.659585
GPR108	1.1664085
GPR137	1.6542039
GPR146	2.3501506
GPR18	2.7051969
GPR3	1.0963678
GPR31B	1.2007608
GPR65	2.0676837
GPR84	5.7860346
GPR85	2.6871245

GPRIN1	0.870738
GPS1	1.9137316
GPS2	1.2542157
GRAMD1A	2.8335152
GRB2	0.96100616
GRB7	1.6372461
GRCC10	0.74753
GRINA	2.7503843
GRIPAP1	2.7215471
GRSF1	1.7950583
GRWD1	2.3694754
GSDMD	2.7537422
GSK3A	2.219038
GSKIP	1.3677487
GSPT1	2.3302479
GSR	2.314609
GSTO1	1.2275691
GSTT3	2.8978782
GSX1	0.7460377
GT(ROSA)26S	1.3477907
GTDC1	1.2649384
GTF2A1	1.3077459
GTF2B	2.363268
GTF2E2	1.0300574
GTF2F1	2.0294604
GTF2F2	1.9421024
GTF2H1	1.7801614
GTF2H2	1.0319633
GTF2H3	1.8590674
GTF2H4	1.4342642
GTF2IRD2	1.4204645
GTF3C1	0.8938241
GTF3C2	1.3798261
GTF3C4	1.3691072
GTF3C5	1.5772214
GTF3C6	2.0154805
GTPBP1	0.7652044
GTPBP2	1.0505052
GTPBP4	1.7109075
GTPBP6	1.738195
GUSB	0.8442774
GYK	3.056273
GZF1	1.8235769
GZMB	2.910102
H13	2.3526711

H2-AA	1.5925322
H2-AB1	1.0541954
H2AFX	1.5899367
H2AFY	1.091084
H2AFZ	0.9652505
H2-D1	1.4035072
H2-EA-PS	1.559763
H2-EB1	0.90928745
H2-K1	1.8576622
H2-K2	1.9641294
H2-KE2	1.5789676
H2-M2	1.654685
H2-Q2	2.0738263
H2-Q4	2.526847
H2-Q5	2.3224359
H2-Q7	1.9920216
H2-Q8	1.8150468
H2-T10	1.7225523
H2-T23	1.8448248
H2-T9	1.1233215
H3F3B	2.5726595
H60A	2.5973034
H60B	1.9284055
HARS	2.50062
HAT1	1.3748846
HAUS2	1.0088241
HAUS3	1.5352736
HAUS8	1.6013422
HAX1	2.6646137
HBA-A2	1.283575
HBP1	1.9130583
HCAR2	2.5380425
HCCS	1.4272633
HCFC1	0.9776306
HCFC1R1	1.0726881
HCK	1.7694583
HCLS1	2.0141177
HDAC10	0.95002174
HDAC3	1.0971951
HDAC4	1.2667432
HDAC5	0.8692951
HDC	2.522862
HDGF	1.3787656
HDGFRP2	1.5044713
HEATR1	2.7021441

HEATR2	1.1390419
HEATR3	1.871573
HEATR5A	0.85088205
HEATR5B	0.732954
HECTD1	2.171967
HECTD3	1.309423
HECW1	1.8793254
HELZ	1.7430577
HELZ2	3.3516521
HEMGN	1.9333463
HERC1	1.1715894
HERC2	1.4084778
HERC4	1.5908351
HERC6	2.1918602
HERPUD1	3.2976418
HERPUD2	1.9352899
HEXA	0.9231248
HEXB	1.5222712
HGF	1.1155953
HGS	2.0537486
HIAT1	1.5751343
HIATL1	1.8333149
HIF1A	3.3767061
HIF1AN	1.480526
HIGD2A	1.7132211
HIPK3	1.6636424
HIRIP3	0.8970542
HIST1H3D	1.8104281
HIST1H4C	0.9804859
HIST2H2BB	0.8430171
HIST2H3C2	1.7211118
HIVEP1	1.7130432
HIVEP2	1.6576695
HK1	1.5154066
HK2	2.5627265
HK3	1.4955654
HLTF	1.1253934
HMCES	1.1290183
HMG20B	1.1276841
HMGA1	1.8586717
HMGB1	0.9308386
HMGN5	1.3398428
HMGXB3	1.6932654
HMMR	1.3691244
HMOX2	1.6269975

HNRNPA0	1.0816283
HNRNPA2B1	2.5864105
HNRNPA3	1.4821215
HNRNPAB	1.8145504
HNRNPC	1.8172321
HNRNPD	1.3186097
HNRNPF	1.6895113
HNRNPH1	1.4217868
HNRNPH2	1.9559045
HNRNPH3	1.4103382
HNRNPK	1.9233365
HNRNPLL	1.0891142
HNRNPM	2.003264
HNRNPR	0.87045527
HNRNPUL1	0.9687495
HNRNPUL2	1.5898013
HOMER2	0.8544285
HOOK2	1.4639626
HOOK3	1.8452153
HP	2.7476292
HP1BP3	0.9011617
HPRT	1.1583877
HPS3	1.4018373
HPS4	1.3401465
HPSE	0.8071718
HRAS	0.8324318
HSBP1	1.3975296
HSD17B11	1.4040236
HSD3B2	2.069282
HSF1	1.9964018
HSF2	1.2523499
HSF5	1.7595968
HSP90AA1	1.747529
HSP90AB1	1.5677366
HSPA14	1.452712
HSPA1A	3.709042
HSPA2	1.3318233
HSPA4	2.062583
HSPA5	2.98735
HSPA8	2.1307163
HSPB11	1.0502348
HSPBP1	1.8261929
HSPH1	3.2196064
HTATSF1	0.7969723
HTRA2	1.0402493

HTRA4	1.461381
HTT	1.6964092
HUS1	1.3030298
HUWE1	1.7011328
IARS	2.385909
ICAM1	2.3301744
ICMT	1.2084866
ICOSL	2.2200704
ID2	2.360001
ID3	1.6769371
IDH3A	1.0644155
IDH3G	1.3932056
IDNK	2.1151972
IDUA	1.3370175
IER2	1.4854927
IER3	2.3414578
IER3IP1	1.5064402
IFFO2	2.0307288
IFI202B	3.1873484
IFI203	1.2480388
IFI204	2.7621908
IFI35	1.9543638
IFI44	1.6064596
IFI44L	1.2457197
IFI47	3.5207834
IFIH1	1.9949088
IFIT1	3.2574096
IFIT2	2.0096889
IFIT3	1.4505167
IFITM1	2.4811344
IFITM2	3.1274877
IFITM3	2.6810317
IFITM5	1.6178594
IFITM6	3.605041
IFITM7	2.0854464
IFNAR1	2.0860157
IFNAR2	2.5267382
IFNG	2.190075
IFNGR2	2.046061
IFRD1	2.659504
IFRD2	2.184914
IFT46	0.74468994
IFT52	1.4338274
IFT57	1.311408
IFT80	1.1213112

IFT88	0.8862996
IGBP1	1.1268435
IGF2BP2	1.5958853
IGF2R	0.7665129
IGSF6	2.707543
IGSF8	2.2757955
IGSF9	1.856524
IGTP	2.9437995
IIGP1	3.3827
IK	2.1087165
IKBKB	1.7780247
IKBKE	4.272011
IKZF1	1.6538842
IKZF4	1.3338332
IKZF5	1.7049618
IL12RB1	1.02268
IL13RA1	2.6315513
IL17RC	1.2638149
IL18BP	3.00635
IL1B	4.750852
IL1F6	3.712277
IL1F9	4.2408366
IL1R2	6.290367
IL21	1.4870377
IL23A	1.1487255
IL27RA	1.7325716
IL2RG	2.5160537
IL4I1	2.3360796
IL6	7.8417587
IL6RA	1.0874205
IL6ST	2.1672359
ILF3	1.622477
ILK	1.3527327
ILKAP	1.3799205
ILVBL	1.493474
IMMP1L	1.4178367
IMMT	2.2153144
IMP3	1.6511784
IMP4	2.3748136
IMPA1	1.445373
IMPAD1	1.5091543
IMPDH2	1.6193781
IMPG2	1.8878474
INF2	1.0965567
ING2	0.91112494

ING3	0.9494915
ING5	1.3215127
INHBA	3.5618544
INHBB	4.62226
INO80C	2.3708823
INO80D	1.5002136
INPP1	1.3654408
INPP4A	1.1692762
INPP5B	0.95715904
INPPL1	0.89787483
INTS12	2.1066322
INTS8	2.0413485
IP6K1	1.5302038
IPO11	1.2007728
IPO5	1.34026
IPO7	1.3182163
IPO8	1.4744902
IPO9	1.19238
IQCG	0.97483826
IRAK1	0.77092505
IRAK2	1.3855486
IRAK3	4.8203144
IRAK4	1.4310675
IRF1	2.5525017
IRF2	2.037602
IRF2BP1	1.2567468
IRF2BP2	1.8041697
IRF3	2.042357
IRF7	1.5674634
IRF8	2.453969
IRF9	1.5840411
IRG1	4.952163
IRGM1	3.1432428
IRGM2	2.6652393
IRS2	3.3530865
ISCA1	1.4092193
ISCA2	1.6228938
ISCU	1.7732148
ISG15	3.6198869
ISG20	1.9992671
ISG20L2	1.4534659
IST1	2.1952786
ISY1	1.5835977
ISYNA1	1.4984136
ITCH	1.2820272

ITFG3	1.9195676
ITGA10	1.1954389
ITGA5	1.7111802
ITGAL	2.1679153
ITGAM	1.5407295
ITGB1BP1	1.6223669
ITGB3	1.4716687
ITK	1.8608255
ITM2B	1.083631
ITM2C	1.0146456
ITPK1	0.6198559
ITPR1	1.2303104
ITPR2	0.9265852
ITSN1	1.6671362
ITSN2	0.9920244
IVNS1ABP	1.2786727
IWS1	0.92789555
JAGN1	1.9276791
JAK1	2.0870843
JAK2	1.4055395
JAK3	2.3706732
JDP2	1.8417563
JKAMP	1.2831626
JMJD1C	2.4562497
JMJD8	1.214524
JPX	1.9058347
JTB	1.7718511
JUN	1.8008885
JUNB	2.694684
JUND	1.8536487
KANK1	0.97379494
KANSL3	1.4750037
KARS	2.4056382
KAT2A	1.0646248
KAT2B	1.05723
KAT5	0.82273436
KAT7	1.6781039
KAT8	2.3419423
KATNA1	2.3277998
KATNAL1	1.0770807
KATNBL1	1.3262491
KBTBD2	1.2587657
KBTBD4	1.9666276
KBTBD7	1.8291175
KCMF1	1.8758254

KCNA4	1.8068929
KCNH3	0.85011196
KCNJ8	2.332287
KCNQ1OT1	1.468575
KCTD14	1.6511965
KCTD20	2.0762138
KCTD21	1.3165071
KCTD5	1.6265726
KCTD6	1.6690335
KCTD9	1.4651985
KDM1A	0.947165
KDM2B	0.8057537
KDM3A	0.90202713
KDM4B	1.3336601
KDM4C	1.025897
KDM5A	1.1328349
KDM5B	0.9839859
KDM5C	1.7936015
KDM6A	1.8796449
KDM6B	1.9438872
KHDC3	1.1952264
KHDRBS1	1.2048464
KHDRBS3	1.611392
KHNYN	1.306541
KHSRP	1.1327581
KIF1B	0.88660526
KIN	0.7034254
KIZ	1.0950327
KLC2	0.8399081
KLF10	1.7695847
KLF15	2.3272686
KLF3	1.4868197
KLHDC10	2.091301
KLHDC2	1.6940107
KLHDC3	2.1092215
KLHDC4	1.565505
KLHL15	1.856966
KLHL18	1.230906
KLHL2	2.2157068
KLHL20	1.1581554
KLHL21	2.0349069
KLHL24	0.7110305
KLHL25	1.6424932
KLHL28	1.3530564
KLHL7	1.1854312

KLHL9	1.3230014
KLRA1	2.1455522
KLRA2	3.7388306
KMT2B	1.3850284
KMT2C	1.1629443
KPNA1	1.6457806
KPNA2	1.7111878
KPNA3	2.091259
KPNA4	0.96774054
KPNA6	0.7753172
KPNB1	1.3295522
KPTN	1.1702943
KRAS	1.0000458
KRCC1	1.9572797
KRI1	1.8887634
KRIT1	1.7288389
KRR1	1.7467074
KRT16	1.1101618
KRT17	1.2646081
KRTAP3-3	0.84054637
KRTAP4-8	1.5240536
KXD1	1.0498857
L7RN6	1.4099598
LACC1	3.6902068
LAMP2	1.9488072
LAMTOR1	1.3376231
LAMTOR3	1.1280088
LAMTOR4	1.423614
LAMTOR5	1.1563091
LANCL1	0.94757605
LAP3	2.7852287
LAPTM4A	1.2580624
LARP1	2.2678914
LARP4	2.0148282
LARP4B	1.6426477
LARP7	1.4401813
LARS2	1.8790483
LCN2	5.112878
LCP1	2.4216013
LCP2	2.116747
LDHA	2.9198866
LDLR	1.432353
LDLRAD3	2.1329904
LEFTY2	0.7522564
LEMD2	1.1229715

LENEP	1.8497062
LENG8	0.8580797
LENG9	1.5471869
LEPROT	0.77959585
LEPROTL1	1.0324788
LETM1	1.46276
LETMD1	0.7206359
LGALS3BP	1.9049282
LGALSL	1.1759806
LHFPL2	0.69008994
LILRA5	0.9108739
LILRA6	2.6668773
LILRB4	3.2495298
LIMD1	1.1265254
LIN28A	1.3795574
LIN37	1.4300594
LIN52	0.9305887
LIN7C	0.81543136
LITAF	3.7321382
LLGL2	0.8108144
LLPH	1.582355
LMAN2L	1.4161844
LMBRD1	0.88436127
LMO4	1.1206036
LMTK2	1.404974
LNX2	1.4428787
LOC1008617	2.1846828
LOC1008617	1.343462
LOC1010557	1.5115275
LOC1010558	1.3108959
LOC1010558	1.5114279
LOC1010558	0.8448105
LOC1010559	1.3929567
LOC1010560	1.3592076
LOC1026319	1.4816275
LOC1026322	1.4243197
LOC1026324	1.2672272
LOC1026327	1.8197098
LOC1026332	2.0072474
LOC1026335	1.8503528
LOC1026338	1.3337915
LOC1026341	2.9121547
LOC1026347	0.9212222
LOC1026350	1.7731729
LOC1026362	1.244894

LOC1026371	2.0536652
LOC1026377	2.0803466
LOC1026381	2.7160258
LOC1026385	1.8423076
LOC1026388	1.2881994
LOC1026399	1.452152
LOC1026399	1.4003048
LOC1026399	1.0867233
LOC1026402	1.1357508
LOC1026410	1.9458675
LOC1026410	0.89334726
LOC1026410	0.7909751
LOC1026410	1.9409521
LOC1026413	1.1621389
LOC1026413	2.0836754
LOC1026413	0.9577236
LOC1026413	2.2543755
LOC1026414	1.2208433
LOC1026415	1.0512743
LOC1026416	1.3975978
LOC1026416	2.3009963
LOC1026419	1.1910062
LOC1026422	1.1686926
LOC1026423	1.6576152
LOC1026424	1.0902658
LOC1026424	0.77796173
LOC1026424	1.5492415
LOC1026425	1.1014414
LOC1026426	0.7137165
LOC1026426	1.0116391
LOC1026427	0.9573536
LOC1026428	0.92319155
LOC1026430	1.870275
LOC1026431	2.0556073
LOC1026432	0.8944473
LOC547349	1.8343039
LOC674896	1.5450883
LOH12CR1	0.82383394
LPAR1	1.537019
LPCAT2	1.6791773
LPCAT3	0.9994745
LPIN3	1.1702456
LPL	0.7699928
LPP	1.3218284
LRBA	0.637218

LRG1	5.286798
LRIF1	2.053043
LRIG3	1.26929
LRP10	1.5085597
LRRC14	1.1830637
LRRC25	2.469903
LRRC31	1.4324698
LRRC38	1.8520138
LRRC40	2.3225904
LRRC41	2.2798247
LRRC47	1.8763599
LRRC58	1.6382728
LRRC8A	2.6270714
LRRC8C	1.8853178
LRRC8D	0.94697905
LRWD1	0.9035344
LSG1	2.138278
LSM1	1.9562454
LSM10	0.9722266
LSM11	0.9909446
LSM12	1.7665553
LSM14A	1.4986043
LSM2	1.7356577
LSM4	1.8174553
LSM5	1.430913
LSM6	1.5868845
LSM7	1.2295332
LSM8	1.0416722
LST1	1.1989121
LTA4H	0.8505106
LTB	1.9147286
LTB4R2	1.1173186
LTBR	1.7051969
LTF	3.6871939
LTN1	1.6443019
LUC7L	2.60323
LUC7L2	1.6765451
LUC7L3	1.132165
LY6E	1.3763967
LY6G	2.7969437
LYN	2.408308
LYPD5	1.1209381
LYPLA2	1.5535808
LYRM1	1.3320754
LYRM4	0.99672556

LZIC	2.1697922
LZTFL1	2.0296068
LZTS3	1.0957975
M6PR	1.6801586
MAD1L1	1.379468
MAD2L1BP	1.1446209
MAD2L2	1.7992592
MADD	1.6855288
MAEA	1.4868212
MAF	1.027101
MAFB	0.89988947
MAFF	4.0016985
MAFG	2.0739207
MAFK	2.2674894
MAGOH	1.762999
MAGOHB	2.3541617
MALT1	2.6235409
MAML1	1.6254463
MAN1A2	0.73581505
MAN1B1	1.5584173
MAN2A1	2.350655
MAN2B1	1.13134
MAN2C1	1.4695477
MANBA	0.9932027
MAP1LC3B	2.331873
MAP1S	0.92446566
MAP2K1	2.3460078
MAP2K3	1.9981256
MAP2K5	0.8383894
MAP3K1	1.5065665
MAP3K11	1.1982813
MAP3K15	1.0168526
MAP3K5	1.4613299
MAP3K7	1.6863675
MAP3K8	3.7303
MAP4K5	0.8310032
MAP6D1	1.168426
MAP7	1.8248234
MAP7D1	1.1939144
MAPK1	1.4874477
MAPK14	1.3038864
MAPK1IP1	1.5592499
MAPK1IP1L	1.4637756
MAPK6	1.3771172
MAPK7	1.0843081

MAPK8IP3	1.5448899
MAPK9	0.9723015
MAPKAPK2	2.6668997
MAPKAPK5	1.8179908
MAPRE1	1.0652752
MAPRE3	1.3674822
MARCKS	1.6384606
MARCKSL1	5.120109
MARF1	0.6998229
MARK1	1.4176455
MARK2	0.8720882
MARK3	1.1916833
MARK4	1.5678463
MARS2	1.3818638
MAST2	1.4047241
MAT2A	1.8703752
MAT2B	0.80512476
MATR3	1.7093921
MAX	1.4776206
MBD1	4.291724
MBD2	1.6637526
MBD3	1.0608058
MBD6	1.3877068
MBNL2	0.8107166
MBOAT7	2.0037537
MBTD1	1.147006
MBTPS1	1.830761
MC2R	0.89416623
MCAM	1.6451054
MCCC1OS	1.173192
MCL1	1.527627
MCM10	1.2698007
MCM3AP	1.3401518
MCM7	1.1974249
MCMBP	1.4229093
MCOLN1	1.3778787
MCTP2	3.3721595
MCTS1	1.470479
MDC1	1.1004109
MDFIC	0.80940056
MDH2	1.9232879
MDM2	1.5460672
MDM4	0.9212129
MDN1	2.2588456
MEA1	1.7876263

MEAF6	0.9397354
MED1	1.7565312
MED10	1.3463092
MED11	1.2487507
MED12	1.2346883
MED15	1.4944081
MED16	1.7520552
MED17	1.6788826
MED20	1.7807069
MED24	1.3210859
MED26	1.5971866
MED27	1.6544981
MED28	1.1360426
MED31	1.5716963
MED4	1.6141996
MED6	1.2170467
MED7	2.211484
MED8	1.9500599
MED9	1.6743689
MEDAG	1.4320726
MEF2A	1.8628626
MEFV	4.6734195
MEMO1	1.1846871
MEN1	0.7344513
MEPCE	1.1929078
MERTK	1.1326184
MESDC2	1.4425144
METAP2	1.8996558
METRNL	1.4871521
METTL14	1.3163757
METTL15	0.78915644
METTL16	1.4511008
METTL20	1.427845
METTL23	0.82795525
METTL3	1.6555438
METTL6	1.4955816
METTL9	1.8896365
MEX3C	1.0958123
MFAP3	1.398561
MFF	1.2760587
MFN1	1.9042277
MFN2	1.3187046
MFSD1	1.1953211
MFSD5	1.4899406
MFSD7B	1.7902699

MFSD8	0.9550531
MGA	1.6935892
MGAT1	1.2363253
MGAT2	2.3565617
MGAT4A	1.5707941
MGEA5	1.2678742
MGRN1	1.3744202
MIB1	1.4758596
MIB2	0.9575925
MICALL1	1.3226323
MICALL2	0.98478365
MICU1	0.9685817
MICU2	1.2135568
MIDN	1.6173711
MIEN1	2.3577132
MIER2	1.5306959
MIER3	1.56358
MIF4GD	1.2272878
MILR1	1.7297516
MINA	1.8629241
MINK1	1.3416853
MINOS1	0.73399734
MINPP1	1.6416149
MIOS	1.0695438
MIR17HG	2.2742405
MIR22HG	1.259592
MIS12	2.1965094
MITD1	1.8889441
MKI67	1.0631747
MKKS	1.8252692
MKLN1	1.7286305
MKNK2	0.9488478
MKX	1.6407337
MLEC	2.0323062
MLF2	1.6181669
MLLT1	1.1154895
MLLT4	0.97680235
MLLT6	2.2990985
MLST8	0.7252183
MLX	2.555457
MMAA	1.5320096
MMP8	4.7812777
MMP9	2.8503413
MMS22L	2.1291828
MNDA	2.6139073

MNDAL	1.4299326
MOAP1	1.457401
MOB1A	1.1432648
MOB2	0.99846363
MOB3C	1.8120358
MOB4	1.4373088
MOCS1	2.868382
MOCS3	2.3715954
MON1A	2.5909767
MON1B	1.6432238
MON2	1.3759103
MORC2A	1.2390904
MORC3	1.9214382
MORF4L1	1.6605415
MOSPD1	0.89002657
MOSPD2	1.2416697
MOV10	1.7453327
MPHOSPH6	0.90888214
MPHOSPH8	1.4217782
MPLKIP	0.74990606
MPO	1.1778038
MPST	1.5504041
MPZL2	2.2817202
MRE11A	1.1280861
MRFAP1	1.4132495
MRGBP	1.3181334
MRGPRA2A	3.6174808
MRGPRA2B	3.5557246
MRGPRA6	5.654917
MRM1	0.8904457
MRPL11	1.6780801
MRPL14	1.3306336
MRPL15	2.4536972
MRPL19	1.7268262
MRPL20	1.900949
MRPL21	1.850637
MRPL23	1.5370688
MRPL3	1.5297203
MRPL32	1.4165368
MRPL33	0.9832511
MRPL34	1.5832014
MRPL37	2.4948215
MRPL39	2.1945758
MRPL4	2.1062999
MRPL40	1.6743698

MRPL41	2.0344653
MRPL42	1.3423805
MRPL43	1.8876314
MRPL45	2.0100055
MRPL48	1.3113346
MRPL49	0.96501255
MRPL52	2.4315877
MRPL53	1.916286
MRPL9	2.0229983
MRPS10	1.6986761
MRPS15	2.5830555
MRPS16	1.7773232
MRPS17	1.492302
MRPS18C	1.6019955
MRPS25	1.1812167
MRPS26	1.3774085
MRPS33	1.2931714
MRPS36	0.8837557
MRPS6	1.990066
MRPS9	1.5589409
MRTO4	2.1703181
MS4A4A	3.0118604
MS4A4C	2.147904
MS4A6B	2.5175073
MS4A6C	2.6493912
MS4A6D	3.890904
MS4A8A	2.444302
MSANTD4	1.2943501
MSH6	1.3479342
MSL1	0.9528456
MSL2	1.6188827
MSL3	1.7828622
MSR1	4.1515083
MSRB1	1.7358527
MT1	4.9117727
MT2	5.5096626
MTA2	1.7084365
MTA3	1.3680129
MTBP	1.9123926
MTDH	1.6042724
MTERF1A	1.1377339
MTERFD1	1.6033397
MTERFD2	1.4820328
MTF1	1.5049372
MTF2	1.4137192

MTFMT	1.5055957
MTFR1L	1.1813946
MTFR2	1.8608537
MTG2	2.1268973
MTHFD2	2.4630861
MTHFS	2.195529
MTIF2	2.6952777
MTM1	0.9148226
MTMR10	0.8375015
MTMR12	1.4318805
MTMR14	2.4429073
MTMR2	1.0129294
MTMR3	1.0707712
MTMR6	1.1123567
MTPAP	1.8984313
MTPN	1.8830471
MTRF1L	1.7398429
MTX1	1.9380965
MVB12A	2.9911604
MVP	2.8314667
MX1	1.3388901
MX2	2.8702435
MXD1	3.7409773
MYC	3.3810492
MYCBP2	1.0392256
MYD88	3.268651
MYEOV2	1.3738117
MYG1	1.2864814
MYH9	1.3785095
MYO1B	1.24722
MYO1E	1.1842818
MYO9B	0.9682565
MYSM1	0.8219645
N4BP2L2	0.74545
N6AMT2	1.4957013
NAA10	1.063684
NAA15	1.5932956
NAA20	1.0528016
NAA25	2.706695
NAA30	1.9284649
NAA35	1.5419612
NAA40	1.3366971
NAA50	1.3523235
NAA60	1.0033388
NAB1	2.0772195

NAB2	1.1978927
NABP1	1.7578073
NABP2	1.5532079
NACA	1.2177134
NACC2	0.75542545
NADK	2.5656157
NAE1	1.468061
NAF1	1.1033874
NAGK	1.0896797
NAIP2	1.3266621
NAIP5	2.4703164
NAMPT	3.4090366
NAPA	1.9780536
NAPEPLD	1.2573767
NAPG	1.5043392
NARFL	1.6970344
NARS2	2.9971447
NASP	1.059155
NAT10	1.3436613
NBN	1.3132806
NCAPH2	0.9451046
NCBP1	1.2431707
NCF1	2.6594706
NCF4	2.4495468
NCKAP1	1.3900266
NCKAP1L	1.4277401
NCL	2.2128634
NCLN	1.5796537
NCOA2	1.6795774
NCOA5	0.96811724
NCOR1	0.8456788
NCOR2	1.1170931
NCSTN	0.7559831
ND6	1.0283899
NDC1	1.7780259
NDEL1	1.487628
NDFIP1	1.3748574
NDOR1	1.5188489
NDRG1	2.1614943
NDUFA12	1.9867196
NDUFA3	1.8944688
NDUFA7	1.7434974
NDUFA9	2.454327
NDUFAF3	1.0462031
NDUFAF4	1.940568

NDUFAF7	1.3673396
NDUFB3	1.3290281
NDUFB7	2.4304142
NDUFC2	1.8105125
NDUFS2	1.998188
NDUFS7	1.7945824
NECAP1	2.259428
NECAP2	1.8897753
NEDD1	1.4398403
NEDD4	1.1554723
NEDD4L	2.7683678
NEDD8	1.4494591
NEK7	1.7084422
NEK9	1.2100377
NELFA	1.2997341
NELFB	1.2259159
NELFCD	1.2852821
NEMF	1.5780439
NEURL1B	1.8743591
NEURL3	3.07448
NF1	1.2170086
NFE2L1	0.81463003
NFE2L2	2.256784
NFIL3	3.6538038
NFKB2	2.8598166
NFKBIA	3.3306222
NFKBIB	3.4757843
NFKBID	2.8193574
NFKBIE	1.949698
NFKBIL1	1.4085913
NFKBIZ	2.066978
NFRKB	1.3500068
NFU1	1.6827278
NFYA	1.7913761
NFYB	2.2752237
NFYC	1.2254457
NGDN	2.4026542
NGLY1	1.6542642
NGRN	1.6200199
NHEJ1	0.88370466
NHP2	1.821661
NHP2L1	2.6729388
NIFK	2.1647067
NINJ1	3.652666
NIPBL	0.8917222

NISCH	0.8458419
NKAP	0.9223342
NKIRAS2	0.9661474
NKRF	1.1431639
NKTR	1.9859886
NLRC4	1.9987414
NLRC5	2.1122766
NLRP3	2.5670958
NME3	1.0936809
NME4	0.7484276
NME7	1.1462126
NMI	1.8372397
NMNAT3	1.2709122
NMT1	1.7133665
NOB1	2.037003
NOC2L	2.1372147
NOC3L	1.3746552
NOC4L	2.184463
NOD1	1.5402155
NOL12	3.189238
NOL7	1.3411314
NOLC1	2.1485586
NOM1	2.171863
NOMO1	1.813756
NONO	1.0135665
NOP10	1.637125
NOP14	2.396649
NOP16	2.3627362
NOP2	3.1488447
NOP56	1.5472322
NOP58	3.027964
NOS2	5.0581536
NOSIP	1.0802293
NOXO1	0.8273082
NPEPPS	1.881536
NPL	2.160483
NPLOC4	1.9535365
NPM1	1.5397396
NR1H2	1.3627715
NR1H3	2.4689808
NR2C1	0.7145004
NR6A1	0.7967334
NRAS	1.290597
NRBF2	2.4969459
NRBP1	1.4165764

NRBP2	0.6346941
NRIP1	1.8333106
NRN1L	2.45153
NRP2	1.0963798
NSA2	0.9267664
NSF	0.74750614
NSMCE1	1.2104821
NSMCE2	0.7983732
NSMCE4A	0.99812794
NSMF	1.7622933
NSUN2	2.1509118
NSUN4	1.2989593
NT5C3	1.5886188
NTMT1	1.6491771
NUB1	1.4802113
NUBP2	1.9276443
NUCB1	1.5025682
NUCB2	0.78982973
NUCKS1	1.241476
NUDC	1.3851676
NUDT21	1.3738475
NUDT9	1.519825
NUFIP2	1.7806339
NUP107	1.2567258
NUP133	0.905478
NUP153	1.4232993
NUP160	1.8577142
NUP205	1.034226
NUP35	1.7911129
NUP43	1.5232911
NUP50	2.2398815
NUP54	2.3897743
NUP62	1.4714527
NUP85	1.6853342
NUP88	1.7464232
NUP98	1.636694
NVL	0.97798705
NXF1	1.7465105
NXT1	1.8736091
NXT2	1.1610332
OARD1	0.72476196
OAS1A	2.5054603
OAS1B	1.7440786
OAS1F	2.0146713
OAS2	2.385254

OAS3	2.4966083
OASL1	4.393441
OASL2	2.301032
OAT	2.1232634
OAZ1	1.6030684
OCEL1	1.3209329
OCIAD1	1.913763
ODC1	3.0060692
ODF2L	1.3875663
OFD1	1.1073136
OGDH	1.208981
OGFOD1	1.8044825
OGFOD3	0.9428172
OGFR	2.2819543
OGT	1.1578765
OLA1	1.2850676
OLFM4	3.9460902
OLFR1229	0.75717974
OLFR1380	0.8754091
OLFR153	1.7922938
OLFR611	1.7093604
OLR1	1.9467528
OPA3	1.5891209
ORAI2	1.2962823
ORAOV1	0.7111745
ORC3	1.5371075
ORC4	0.62187815
ORC5	1.3196754
ORMDL1	1.4705586
OSBPL11	0.986876
OSBPL3	1.2849565
OSBPL8	1.1412058
OSBPL9	1.1897097
OSER1	1.8366055
OSGEP	1.1363678
OSMR	2.7534518
OSTF1	1.1658216
OTOF	1.0590863
OTUD3	2.153195
OTUD4	1.655746
OTUD5	1.7813206
OTUD6B	1.3393357
OVCA2	1.2242427
OVOL2	1.0171351
OXA1L	1.1062083

OXSR1	1.7128005
P2RY13	3.4272776
P2RY2	3.5884762
PA2G4	1.7389607
PABPC1	1.3893976
PABPN1	1.3394685
PAF1	1.5695829
PAFAH1B1	1.47651
PAFAH1B2	2.3250856
PAGR1A	1.1087775
PAICS	1.5004368
PAIP2	1.1040645
PAIP2B	1.6191759
PAK1IP1	1.9923806
PAK2	1.3468499
PAK4	1.9525285
PAM16	1.8495126
PAN2	1.4171147
PAN3	0.84867764
PANK2	1.0940084
PANK3	1.450769
PANK4	1.6693783
PAPD4	1.926775
PAPD5	1.177701
PAPD7	1.6031227
PAPOLA	1.5525279
PARD6A	1.109173
PARG	1.4970598
PARL	1.5682349
PARP1	1.3216214
PARP10	2.3378267
PARP11	1.9248445
PARP12	2.423562
PARP14	2.9423943
PARP2	1.7510586
PARP3	1.8294158
PARP6	0.7383785
PARP9	3.1861005
PATL1	1.7287269
PAWR	3.0265098
PAXBP1	1.2973485
PBDC1	2.2887964
PBRM1	0.6857977
PCBP1	2.2504783
PCBP2	1.4317989

PCF11	1.2069011
PCGF1	0.8328531
PCGF3	1.2964303
PCGF5	1.7456203
PCID2	2.5740852
PCMT1	1.5277758
PCMTD1	0.8873563
PCNP	1.04393
PCNXL3	1.5895619
PCYT1A	1.2934585
PDAP1	2.3808374
PDCD10	2.3784175
PDCD2	1.8504019
PDCD2L	1.2851067
PDCD5	1.811346
PDCD6	1.7790709
PDCD6IP	2.224926
PDCD7	1.6357179
PDCL	1.5583129
PDCL3	1.2818675
PDE1B	0.98359585
PDE4B	3.0051584
PDE4C	1.2648001
PDF	1.702043
PDK4	3.1613145
PDLIM1	1.2047114
PDLIM7	2.1988134
PDPK1	1.4431543
PDRG1	0.8746705
PDS5A	0.9584298
PDSS1	1.4175305
PDXK	1.0348697
PDZD11	1.4902878
PDZD8	1.8269024
PEBP1	1.8531342
PEF1	1.3808146
PEG3	1.3555894
PEG3OS	1.7035389
PELI1	1.4803042
PELP1	1.7443194
PEPD	2.6067734
PER1	1.7877088
PES1	2.134264
PEX11B	1.2904487
PEX13	2.140504

PFDN1	0.986825
PFDN2	1.4409709
PFDN4	1.367135
PFDN5	0.9310074
PFKFB2	1.988172
PFKFB3	4.4080696
PFKL	1.9341993
PFN1	1.8595977
PGAM1	2.3938508
PGAM5	1.6612492
PGAP3	1.1499391
PGD	1.1615915
PGGT1B	1.6165452
PGK1	2.7710714
PGM1	1.5354013
PGRMC2	1.1947136
PGS1	1.6575384
PHACTR4	1.745925
PHF10	1.9412823
PHF11A	1.9893188
PHF11D	1.9193926
PHF12	1.5656638
PHF2	1.7355132
PHF23	1.4654245
PHF3	1.4079685
PHF5A	1.9676228
PHF8	1.5799799
PHIP	1.29473
PHKG2	1.4628015
PHLDA1	2.4995952
PHPT1	1.8012524
PHRF1	1.4648538
PHTF2	1.275579
PHYHD1	1.4646349
PI4K2A	2.279048
PI4KA	1.2024369
PI4KB	1.2772436
PIAS4	1.1725798
PICALM	2.4545379
PIF1	1.3367276
PIGC	1.3789537
PIGF	1.1617928
PIGL	2.0416346
PIGN	1.0268157
PIGR	3.0572443

PIGS	0.8163719
PIGT	1.4520683
PIGV	0.8833165
PIGX	1.1518326
PIGYL	0.8929267
PIH1D1	1.6421595
PIK3CA	1.1048331
PIK3CB	0.740613
PIK3CG	1.714433
PIK3R1	0.9566884
PIK3R4	1.3661346
PIK3R5	2.5419044
PIK3R6	1.7684999
PILRA	2.335999
PILRB2	2.2872066
PIM1	3.7223077
PIM2	1.9868863
PIM3	3.8503065
PIN1	1.5322361
PIN1RT1	1.3477821
PIN4	1.454042
PINX1	3.0575304
PIP4K2C	1.1353517
PIP5K1A	6.4570208
PIRA11	1.9406805
PIRA2	2.1869555
PIRA6	2.4306931
PIRA7	2.3654084
PIRB	1.7375622
PISD	1.6800976
PISD-PS1	1.5881538
PITPNA	1.1751318
PITPNB	1.4560966
PITPNM2OS1	1.0955524
PJA1	1.135664
PJA2	1.312891
PKN2	1.5368576
PKNOX1	2.2490802
PLA2G12A	1.952992
PLA2G4A	1.3903532
PLA2G4B	1.7760129
PLA2G7	2.3667293
PLAGL2	1.5952172
PLAUR	2.7582202
PLBD1	1.7847538

PLCD4	1.4012456
PLCL2	1.4624357
PLD1	1.0436683
PLEC	2.053187
PLEK	2.275054
PLEKHA3	2.5039477
PLEKHA4	0.8780565
PLEKHA5	1.4375765
PLEKHF1	0.75025845
PLEKHF2	1.087491
PLEKHG3	1.9311175
PLEKHG4	5.21315
PLEKHJ1	1.5399299
PLEKHM1	1.2723603
PLEKHM2	1.2809834
PLEKHS1	3.0859535
PLOD3	1.4919419
PLP1	0.8079953
PLSCR1	3.7870274
PLXNA2	2.069962
PLXNB2	1.6979375
PML	1.8913751
PMM2	1.998642
PMVK	2.7875605
PNISR	1.1220708
PNKP	1.3514571
PNMA2	2.8561206
PNN	1.6828127
PNO1	2.69314
PNP	4.085326
PNP2	1.48509
PNPLA2	1.7671561
PNPLA6	1.1692486
PNRC1	3.2596054
PNRC2	1.1243267
POC5	1.0318131
POFUT1	0.8529992
POFUT2	0.9068713
POGK	1.4586596
POGLUT1	1.5722008
POGZ	1.4516106
POLA2	0.6686225
POLB	1.954936
POLD2	1.1035092
POLDIP3	1.5525618

POLE3	1.0115767
POLG	1.4718552
POLR1A	1.8934178
POLR1C	1.9690671
POLR1D	0.86885643
POLR1E	1.915937
POLR2B	1.6167474
POLR2C	1.347466
POLR2D	1.2977376
POLR2E	0.68889475
POLR2F	1.3960013
POLR2G	0.7488823
POLR2H	1.3604546
POLR2J	1.3352122
POLR2K	1.238215
POLR2M	1.0683608
POLR3A	1.5400505
POLR3B	1.4236875
POLR3E	1.4051247
POLR3K	0.7438326
POLRMT	1.6546164
POM121	1.6235662
POMGNT1	1.3625073
POMK	0.72427464
POMP	1.5296249
POMT1	1.027633
POMT2	1.0948703
PON2	0.78757477
PON3	1.1033964
POP1	0.9713559
POP4	0.81314373
POP5	2.017014
POP7	1.1833987
POR	1.715364
POT1A	1.5405369
POT1B	1.8018236
POU2F2	1.3889275
PPAN	2.905645
PPAT	0.98106813
PPDPF	0.856771
PPFIA1	1.576561
PPFIBP1	1.1555991
PPIA	1.3994036
PPID	1.7693248
PPIG	1.2298737

PPIL1	0.7923324
PPIL3	1.0606351
PPIL4	1.541121
PPM1B	1.6007023
PPM1G	1.3326716
PPM1H	0.9390383
PPM1K	1.5138321
PPM1N	2.670817
PPME1	1.9437275
PPNR	1.3283801
PPP1CA	1.2096095
PPP1CB	1.2300134
PPP1CC	1.3776598
PPP1R11	2.2034416
PPP1R12C	1.1434221
PPP1R13L	1.6898084
PPP1R14B	1.5356603
PPP1R15A	2.2038722
PPP1R16A	1.7720642
PPP1R21	1.940836
PPP1R35	1.4765792
PPP1R37	1.1400146
PPP1R7	1.483674
PPP2CA	1.770834
PPP2CB	1.1430049
PPP2R1A	1.4311109
PPP2R2A	1.6218462
PPP2R2D	2.4170694
PPP2R3C	1.6028247
PPP2R4	0.9141011
PPP2R5A	0.83498573
PPP2R5B	1.8052363
PPP2R5D	1.1428938
PPP3CB	0.8888669
PPP3R1	1.1859093
PPP4C	1.8038244
PPP4R1	1.5633512
PPP4R2	1.4214725
PPP5C	1.2290895
PPP6C	1.5276155
PPP6R1	0.8151498
PPP6R3	1.1437459
PPRC1	2.3918774
PPT1	1.0324616
PPTC7	1.0071278

PQBP1	1.3602238
PQLC1	1.2217913
PRAF2	1.2757549
PRAM1	2.6209865
PRAMEF8	1.5225887
PRCC	0.7929754
PRDM2	1.1680975
PRDM4	1.9919105
PRDX5	3.4152174
PRICKLE3	1.3071926
PRIM1	1.278758
PRKAA1	1.5582323
PRKACB	1.1820965
PRKAG1	1.3468409
PRKAR1A	1.5974002
PRKCB	1.3298774
PRKCD	1.6032434
PRKRIP1	1.6411805
PRKRIR	0.86287117
PRMT1	1.6148047
PRMT10	1.2835169
PRMT3	2.0877824
PRMT5	1.4941192
PRMT7	1.4301028
PRNP	1.4513869
PROK2	7.619211
PRORSD1	2.2969499
PROSC	2.2826185
PROSER2	1.1782846
PRPF18	1.3339081
PRPF3	1.3007374
PRPF31	1.7045741
PRPF38A	1.6419353
PRPF38B	1.6478944
PRPF39	1.6147051
PRPF4	1.3867998
PRPF40A	2.7933483
PRPF4B	1.5104852
PRPF6	2.058539
PRPF8	1.5205646
PRPS1	1.8263721
PRPSAP1	1.3899708
PRPSAP2	1.4842529
PRR14	1.3701959
PRR14L	1.2737141

PRRC2A	1.4321404
PRRC2C	1.1673827
PRRG2	1.2053795
PSAP	0.8322296
PSAT1	0.8052554
PSEN1	2.3080034
PSEN2	2.337473
PSENEN	1.5927119
PSMA2	1.5788884
PSMA6	2.0861273
PSMA7	2.2104578
PSMB10	2.4243436
PSMB5	2.0682445
PSMB8	1.4750395
PSMB9	2.2781253
PSMC1	1.7380905
PSMC2	2.1373796
PSMC3	1.7192049
PSMC4	2.1909475
PSMC5	1.7637854
PSMC6	2.1614542
PSMD1	2.0748754
PSMD11	2.1456127
PSMD12	2.404354
PSMD13	1.6226535
PSMD14	2.0702968
PSMD2	2.2825708
PSMD3	2.179336
PSMD4	2.1556315
PSMD6	2.3376741
PSMD7	2.019435
PSMD9	1.8052807
PSME1	1.9823742
PSME2B	1.8111143
PSME3	1.9212136
PSMF1	1.4432287
PSMG3	1.8485136
PSMG4	2.0674753
PSPH	0.9554992
PSTPIP2	2.3968992
PTBP1	1.5000057
PTBP3	1.1139779
PTCD3	2.090692
PTDSS1	0.7759342
PTEN	1.302103

PTGES3	1.8293738
PTGS2	3.1314769
PTMS	0.8339224
PTOV1	0.87670135
PTP4A2	0.8565178
PTPLA	1.6248164
PTPN1	0.80876064
PTPN11	1.2163363
PTPN12	1.9538851
PTPN2	2.3847685
PTPN23	2.4773011
PTPN6	2.0389457
PTPRC	1.4612212
PTPRF	0.6939783
PTPRK	1.5021429
PTRH1	0.70736504
PTRHD1	1.3825231
PTTG1IP	1.738358
PUF60	1.4694648
PUM1	1.4075494
PUM2	1.2711372
PURA	0.797699
PURB	1.0698633
PUS1	2.163959
PUS10	1.4789162
PUS3	0.68905973
PUS7	1.7874994
PUS7L	1.1172826
PUSL1	1.3443222
PVR	2.6915727
PVRL1	1.8188524
PVRL2	1.0223651
PWP1	2.3693438
PWP2	2.2596483
PXDC1	2.7160757
PXK	1.673682
PYCR2	0.9909911
PYDC4	0.85890985
PYGL	2.1681724
PYGO1	1.3951092
PYGO2	1.8015418
PYHIN1	2.5041556
QARS	1.9977727
QRICH1	1.3047194
QRSL1	1.7732096

QTRTD1	2.7455602
R3HCC1L	1.2361941
R3HDM1	1.2174473
R3HDM2	1.5870132
RAB1	2.5282335
RAB10	1.6905432
RAB11A	1.7491121
RAB11B	0.9430785
RAB12	1.2043543
RAB14	1.1571336
RAB18	2.165524
RAB1B	0.7579503
RAB20	2.046442
RAB21	1.8462391
RAB22A	1.8225088
RAB24	1.9407358
RAB26OS	2.4057176
RAB2A	1.7196274
RAB32	3.0757952
RAB33B	1.4166155
RAB35	1.5783815
RAB37	0.7955518
RAB3C	0.8545723
RAB3GAP1	1.467555
RAB3GAP2	1.6659532
RAB3IP	1.5152082
RAB40C	1.3068337
RAB43	1.3251963
RAB4B	1.7229424
RAB5A	2.1261554
RAB6A	1.8210659
RAB7	3.1504989
RAB7L1	0.7412405
RAB8A	1.4020987
RAB8B	1.7448688
RABAC1	1.0045476
RABEP1	0.93158674
RABEP2	0.9078779
RABGEF1	1.1625562
RABGGTA	1.2624011
RABGGTB	1.7678623
RABL2	1.460566
RAC1	1.3483286
RAC2	1.9911137
RAD17	2.2249584

RAD21	1.0519543
RAD23B	1.988069
RAD51C	1.774581
RAD52	1.7901721
RAD54L2	1.1317782
RAE1	1.7804427
RAF1	1.49506
RAI1	0.81679296
RALA	1.4283638
RALGAPA1	1.1694732
RALGAPA2	1.3617463
RALGDS	2.0541244
RALY	1.3524089
RAN	1.952301
RANBP1	2.019844
RANBP10	1.3880844
RANBP2	1.8678584
RANBP3	2.8003173
RANBP9	1.7378945
RANGAP1	2.4075308
RAP1B	1.4532785
RAPGEF6	0.96091413
RARS2	1.7675881
RASA1	1.2105057
RASA4	1.6956348
RASD1	4.481538
RASGEF1B	2.917266
RASGRP1	1.874423
RASL2-9	2.348247
RASSF1	1.7998223
RASSF4	1.6455588
RBAK	0.9079838
RBBP4	0.86904526
RBBP5	1.7915373
RBBP6	1.5267944
RBBP7	1.0740166
RBCK1	1.5238438
RBFOX2	1.5649204
RBM10	2.2438383
RBM12	1.1890798
RBM12B2	0.71177626
RBM14	1.695755
RBM15	2.8570542
RBM17	2.0980916
RBM18	1.477529

RBM19	2.5122104
RBM22	1.156451
RBM25	1.5684118
RBM26	2.139051
RBM27	1.4117448
RBM28	1.1701527
RBM33	2.1116428
RBM34	1.2194681
RBM39	1.7327356
RBM3OS	2.3201091
RBM4	2.3764787
RBM42	1.6767826
RBM45	0.81448054
RBM47	2.1806974
RBM4B	2.1097112
RBM5	1.331605
RBM6	1.2795949
RBM8A	1.5184565
RBMXL1	1.7262411
RBPMS	1.2562766
RBX1	1.3547449
RC3H1	1.0653467
RC3H2	1.5127444
RCAN1	3.2534552
RCC2	0.94493103
RCHY1	1.8041363
RCOR1	0.74370575
RDH1	1.9227419
RDH10	1.943665
RDX	0.98187685
REEP3	1.8142223
RELA	2.522079
RELB	2.0631094
RELL1	2.0346556
REPS1	1.3758864
REPS2	0.96723366
RER1	2.0251784
REST	1.5611763
REXO4	1.8221722
RFC2	0.7529297
RFK	1.0897651
RFWD2	1.7243557
RFWD3	1.2732038
RFX5	1.4156659
RFXAP	1.4446907

RGL3	2.6351047
RGS1	2.6850772
RGS16	5.202181
RHBDD2	1.204534
RHBDF2	2.6326513
RHOB	1.8544388
RHOC	2.74786
RHOQ	1.3247252
RHOT1	1.4150128
RHOT2	0.81865025
RHOU	3.678513
RHOX8	3.3118129
RIC8	1.0656075
RICTOR	1.7340336
RIF1	1.0530217
RILP	1.3823133
RILPL2	1.4849148
RIOK1	1.0608344
RIOK2	2.3230152
RIOK3	1.4536972
RIPK1	2.824726
RIT1	0.7726674
RLF	1.2606511
RLIM	1.3013206
RMDN2	0.82557917
RMDN3	3.2369685
RMRP	0.7066512
RN28S1	1.5623655
RN4.5S	2.1226273
RNASE10	1.201396
RNASEH1	1.6761823
RNASEH2B	1.4281306
RNASEH2C	1.2572427
RNASEK	1.4949183
RND1	5.9354925
RND3	2.976759
RNF10	1.7166395
RNF11	1.6805024
RNF111	1.3723025
RNF114	1.6515722
RNF115	1.3210678
RNF123	2.0558848
RNF125	3.695417
RNF13	1.7870722
RNF130	1.6605043

RNF138	1.0212607
RNF139	1.4910817
RNF14	1.5524178
RNF141	0.81652594
RNF144B	0.7731087
RNF146	0.7484827
RNF149	2.583354
RNF167	1.3073225
RNF168	0.81118584
RNF169	1.8682542
RNF181	0.973259
RNF19B	2.7824984
RNF2	1.6315589
RNF20	1.4555902
RNF213	2.584289
RNF214	0.6446459
RNF216	1.5176649
RNF24	1.3654971
RNF25	0.9431491
RNF31	1.7584939
RNF4	2.081738
RNF40	2.9747105
RNF41	0.80174327
RNF44	1.7765546
RNF6	1.3414583
RNF7	1.344842
RNF8	1.3220854
RNFT1	1.5942535
RNGTT	1.3779161
RNH1	1.5507355
RNPC3	1.2570508
RNPEPL1	0.8222966
RNPS1	1.5794272
RNU12	0.7027092
RPA2	1.2768645
RPAIN	2.3830404
RPE	1.2060547
RPF1	1.7823834
RPF2	2.6951337
RPGRIP1	1.357204
RPL10	1.3251162
RPL10A	1.8687687
RPL11	1.4528131
RPL12	1.5877843
RPL13	1.2724853

RPL13A	1.4029717
RPL14	0.76209354
RPL15	1.441853
RPL17	1.2346811
RPL18	1.4367714
RPL18A	1.1021018
RPL19	1.3297563
RPL21	1.394629
RPL22	1.3554311
RPL22L1	1.544538
RPL23	1.0294542
RPL23A	1.1830406
RPL24	1.118958
RPL26	1.1576066
RPL27	1.2961373
RPL27A	1.4665966
RPL28	1.3228898
RPL29	1.3064332
RPL3	1.4043541
RPL30	0.94584656
RPL31	0.98036623
RPL31-PS12	1.1192002
RPL34	1.1982636
RPL35	1.1961622
RPL35A	1.3007879
RPL36	1.6489711
RPL36A	1.497572
RPL36AL	1.7999277
RPL37A	1.246419
RPL38	1.5430479
RPL39	1.2485423
RPL4	1.5199118
RPL41	2.0361366
RPL5	0.8263364
RPL5-PS2	1.2760673
RPL6	1.2980084
RPL7	1.4952288
RPL7A	1.7193942
RPL8	1.295227
RPL9	1.1350498
RPLP0	1.5586467
RPLP1	0.95756865
RPLP2	1.7294273
RPN1	2.8300958
RPN2	1.4249444

RPP14	1.688139
RPP21	1.158102
RPP25L	1.3172388
RPP30	1.2187922
RPRD2	1.0996988
RPS10	1.2548227
RPS11	1.2337575
RPS11-PS1	1.1537771
RPS12	1.4143066
RPS12L2	1.3598013
RPS12-PS16	1.2207284
RPS13	1.5540066
RPS14	1.1971312
RPS15	0.99609184
RPS15A	1.1360326
RPS16	1.3221035
RPS17	1.4077702
RPS18	1.4422708
RPS19	1.0258174
RPS19BP1	2.1455545
RPS2	1.7252479
RPS20	1.1164694
RPS21	1.1026454
RPS23	1.6724668
RPS24	1.1259613
RPS25	1.0883112
RPS26	1.2115259
RPS27	1.431788
RPS27A	1.3573828
RPS28	1.4284735
RPS29	1.0808115
RPS3	1.4659481
RPS3A1	1.142405
RPS4L	0.83168936
RPS4X	1.1276073
RPS5	1.3809061
RPS6	1.1066165
RPS6KB1	1.4120283
RPS6KB2	1.3131876
RPS6KL1	1.013449
RPS7	2.016603
RPS8	1.3858919
RPS9	1.208642
RPSA	1.6556935
RPTOR	1.2059865

RQCD1	1.8850799
RRAGA	2.612486
RRAGC	1.7006621
RRAS2	1.8036408
RREB1	0.97013235
RRN3	1.5220842
RRNAD1	0.7642963
RRP1	1.3971853
RRP15	2.5405416
RRP1B	0.7438655
RRP36	1.7688656
RRP7A	1.6558032
RRP8	1.1817205
RRP9	2.1015697
RRS1	2.5466037
RSAD2	3.19039
RSL1	1.3889942
RSL1D1	1.8466854
RSL24D1	1.4720826
RSPH3B	2.025866
RSPRY1	2.0201426
RSRC2	2.10107
RSRP1	1.3336625
RTCB	1.5990944
RTEL1	0.7790432
RTF1	1.1913111
RTFDC1	1.4307952
RTN3	1.1724386
RTN4	1.5636349
RTP4	2.5118499
RUFY1	1.502429
RUFY3	1.3590517
RUNDC1	1.4499326
RUSC1	0.87578773
RUSC2	1.3602943
RUVBL1	1.9134445
RXRB	2.5379462
S100A10	1.7772598
S100A8	3.3754263
S100A9	2.6162338
SAA1	7.2229996
SAA2	9.01216
SAA3	7.074836
SAC3D1	1.205894
SACM1L	2.2359548

SACS	1.076179
SAE1	0.97228813
SAFB	1.8739386
SAFB2	1.8902726
SAMD4B	1.9961557
SAMD8	1.2454457
SAMD9L	2.011198
SAMM50	1.899641
SAMSN1	3.3693862
SAP130	1.2988749
SAP18	1.6084819
SAP30	1.5461445
SAP30BP	1.5467587
SAP30L	1.1147437
SAR1A	2.1974235
SARNP	1.6137176
SARS	1.5705209
SART3	1.6655202
SASH1	1.5150776
SBDS	1.3001871
SBF1	1.8236814
SBNO1	1.7150297
SBNO2	2.5778995
SCAF1	0.79110146
SCAF4	1.3970852
SCAF8	1.0755563
SCAMP1	1.6669674
SCAMP2	1.2332993
SCAMP4	2.1299558
SCAND1	1.4673591
SCAP	1.481102
SCARB2	1.607491
SCIMP	1.1827178
SCNM1	1.48282
SCOC	0.8035157
SCPEP1	1.6124635
SCRIB	1.6276827
SCYL1	1.943429
SCYL2	2.5069633
SCYL3	1.7163973
SDAD1	2.2546144
SDC4	3.950244
SDCCAG3	1.7256408
SDE2	2.0464754
SDF2	1.494245

SDF4	1.4630699
SDHAF1	1.3588891
SDHAF2	1.7244349
SDR39U1	0.78690434
SEC1	1.3669598
SEC11A	1.9915552
SEC11C	1.2468681
SEC14L4	2.3318868
SEC23IP	1.8614321
SEC24B	1.5156946
SEC24C	1.7080398
SEC62	1.2667952
SECISBP2	1.1116419
SECISBP2L	0.8699124
SEH1L	2.2189474
SELK	2.1367173
SELP	4.6926584
SELT	1.6933227
SEMA4A	2.0517464
SEMA4B	0.6557369
SEMA4C	1.7968869
SEMA6B	1.5048556
SEMA6D	1.439743
SENP2	1.5490298
SENP3	1.563664
SENP5	1.8692007
SEPW1	0.68833256
SERBP1	2.1125455
SERF2	1.34554
SERGEF	1.0076399
SERHL	1.3536434
SERINC1	1.8887463
SERINC3	1.3654513
SERPINA3F	3.7608747
SERPINA3G	3.101521
SERPINA3I	4.3343544
SERPINA3N	7.0658545
SERPINB2	2.4895825
SERPINB8	1.7699418
SERPINE1	5.309525
SET	1.8442602
SETD1A	1.5059185
SETD1B	1.3091574
SETD2	1.3538404
SETD5	1.2396355

SETD6	1.993442
SETD8	1.0726204
SETDB1	1.2538881
SETX	1.4289594
SF1	1.8888173
SF3A1	1.625771
SF3A3	1.7042427
SF3B2	1.6066661
SF3B3	2.083228
SF3B4	1.7983537
SF3B5	1.2999802
SF3B6	2.0467763
SFI1	1.8265591
SFMBT1	1.5590477
SFPQ	1.8838434
SFR1	1.0688572
SFSWAP	1.2746243
SFT2D3	0.7424054
SGK3	0.91828585
SGMS1	2.4664183
SGPL1	1.8100181
SGSH	1.8085313
SGSM3	1.3257346
SGTA	1.3020873
SH2B1	2.9534316
SH2B2	3.6803575
SH3BGRL	1.2719054
SH3BP5L	0.7739186
SH3GL1	1.8121405
SH3GLB1	1.273612
SH3PXD2B	2.7135596
SHARPIN	1.6016002
SHB	1.3908639
SHC1	2.0735583
SHC4	1.0428691
SHFM1	1.5113049
SHKBP1	1.0457444
SHOC2	1.6997328
SHPRH	0.9283836
SHQ1	2.9526951
SHROOM2	1.0289316
SIGLECE	1.714735
SIGLECH	1.5924478
SIK1	2.0601401
SIK3	1.277597

SIKE1	0.9595876
SIN3A	1.3663769
SIPA1L1	1.0057015
SIRPA	1.8099046
SIRT1	1.2647166
SIRT2	1.1292963
SIRT7	1.5172286
SKAP2	1.2374701
SKIL	1.6556253
SKINT7	1.8669429
SKIV2L2	1.5971608
SKP1A	1.6374459
SLA	2.3391376
SLAMF7	1.9745555
SLBP	0.7886162
SLC10A1	1.9403944
SLC10A3	1.3011751
SLC10A6	3.3947759
SLC11A1	3.0038033
SLC12A4	1.3296666
SLC12A7	1.7800412
SLC15A3	3.0493526
SLC15A4	1.2063727
SLC16A3	2.62988
SLC16A6	3.1430316
SLC1A4	1.9084373
SLC20A1	2.3300467
SLC22A5	2.8187814
SLC24A5	1.1542144
SLC25A11	0.92436934
SLC25A12	1.4778843
SLC25A19	1.1791167
SLC25A25	4.2372317
SLC25A28	1.8698215
SLC25A3	2.1942153
SLC25A30	1.2530844
SLC25A33	2.6656137
SLC25A37	0.9052882
SLC25A38	1.8480544
SLC25A46	1.5973911
SLC25A5	1.9815679
SLC25A51	0.6655817
SLC26A2	1.3660209
SLC29A1	0.98349667
SLC29A2	1.6287186

SLC2A1	2.3069649
SLC2A4RG-PS	1.2351589
SLC2A6	3.4702468
SLC30A4	1.4584675
SLC30A6	2.4799333
SLC30A9	1.826549
SLC31A2	2.164506
SLC35A4	1.1187963
SLC35B2	1.0958152
SLC35B3	1.580637
SLC35C2	1.639276
SLC35E1	1.6505141
SLC35F6	1.120522
SLC38A2	3.5634303
SLC38A4	4.0965977
SLC39A1	2.5714507
SLC39A13	0.6623769
SLC39A6	0.9660425
SLC3A2	2.4644017
SLC41A1	2.1880624
SLC41A3	0.682981
SLC5A6	1.2405319
SLC6A8	1.5257182
SLC7A1	2.060163
SLC7A11	1.6014695
SLC7A6OS	2.452035
SLC7A7	1.818006
SLC7A8	1.9223967
SLC9A6	1.2859225
SLC9A8	2.1474452
SLFN1	2.886678
SLFN10-PS	0.92507505
SLFN2	2.3680825
SLFN3	2.2933495
SLFN4	4.947805
SLFN5	1.2662282
SLFN8	1.6446476
SLIRP	1.6310139
SLK	1.371799
SLMAP	0.9791951
SLMO2	1.6439047
SLPI	2.523252
SLTM	1.7504373
SLX4	1.9549775
SMAD1	1.4620388

SMAD2	0.9543805
SMAD4	1.509933
SMAGP	0.95551443
SMAP1	1.1111484
SMAP2	1.3451014
SMARCA4	1.3601351
SMARCA5	1.5498829
SMARCA5-PS	1.0361562
SMARCAD1	1.5403199
SMARCB1	1.2486353
SMARCC2	1.2102804
SMARCD2	0.9385948
SMC1A	1.4173379
SMC3	1.3159094
SMC5	1.6455779
SMCHD1	1.2353635
SMCR8	2.2800245
SMDT1	1.7874599
SMEK1	1.6228461
SMEK2	1.4484878
SMG1	1.7763624
SMG5	1.8654785
SMG7	1.3113184
SMG9	0.8166113
SMIM11	1.2943449
SMIM12	0.8935394
SMIM13	1.2696366
SMIM19	0.877553
SMIM20	1.5791335
SMIM7	1.4284525
SMN1	1.3085098
SMNDC1	2.253878
SMOX	2.147519
SMPD1	1.9047413
SMPD4	1.0576124
SMPDL3A	0.7593031
SMURF2	0.74120235
SNAP23	1.8197236
SNAP29	2.0452447
SNAPC1	2.5661016
SNAPC2	1.3134027
SNAPC3	1.0960083
SNAPC4	1.4432015
SNF8	1.6466203
SNHG1	1.7878156

SNHG12	2.3657885
SNHG6	3.1436958
SNIP1	1.7444482
SNORA74A	1.1478903
SNRNP200	1.2141461
SNRNP25	0.89411306
SNRNP27	1.2748895
SNRNP48	2.3133492
SNRNP70	1.8626862
SNRPA	2.0625257
SNRPA1	1.5836535
SNRPB	0.9122133
SNRPB2	0.7476015
SNRPC	1.9083719
SNRPD1	1.7394629
SNRPD2	1.3865056
SNRPD3	1.540606
SNRPE	1.0958962
SNRPG	1.4672661
SNTB2	2.3291721
SNTG2	3.18989
SNUPN	1.367651
SNW1	1.2206316
SNX1	1.6101675
SNX10	4.06307
SNX11	1.4139261
SNX13	1.4672823
SNX14	1.1619468
SNX15	1.3291397
SNX16	1.6156907
SNX17	1.196475
SNX18	2.3256092
SNX19	1.9254289
SNX2	0.9106474
SNX20	2.2486057
SNX27	1.1162157
SNX3	2.0222168
SNX4	1.1456625
SNX5	1.0994601
SNX7	1.863235
SNX8	1.6826601
SOCS1	3.9234796
SOCS2	1.469459
SOCS3	4.8660665
SOCS4	1.4874341

SOCS7	1.4932275
SOD2	4.583622
SON	1.5403099
SORBS1	1.7804723
SOWAHC	3.1910868
SOX9	5.6855936
SP1	1.7163239
SP110	1.9364729
SP140	1.504664
SP3	0.9990654
SPAG7	2.0600572
SPAG9	1.3276334
SPAST	1.7965121
SPATA13	2.1534886
SPATA2	0.8152733
SPATA5	2.1186304
SPATA5L1	1.8063769
SPC24	1.1532295
SPCS1	1.7142897
SPCS2	2.9787865
SPEN	1.3260612
SPG11	1.0136323
SPG21	1.3202882
SPI1	1.5942094
SPIDR	1.6173353
SPIN1	1.4104223
SPNS1	1.6298809
SPP1	1.5827289
SPPL2A	2.8897023
SPRED1	1.7323103
SPRR2A2	1.1732988
SPRYD7	2.2096663
SPSB2	1.5116439
SPSB3	1.1093769
SPTLC1	1.8756647
SPTLC2	1.4076705
SPTY2D1	1.8698602
SRD5A3	0.76903915
SREBF1	1.3231096
SREBF2	1.5090003
SREK1	2.114358
SRF	1.6681743
SRFBP1	2.369213
SRGN	2.1111155
SRI	1.4838047

SRP14	1.148068
SRP54C	3.0204296
SRPK1	2.2123928
SRRM1	0.8290272
SRSF1	1.5885682
SRSF10	1.4932876
SRSF11	1.0990376
SRSF3	1.8265867
SRSF4	1.704381
SRSF5	1.8650827
SRSF6	2.1173472
SRSF7	1.8975549
SRSF9	1.365047
SS18	1.4324074
SS18L1	1.8116331
SSB	1.5999556
SSH3	2.041943
SSNA1	1.0561743
SSRP1	0.8804965
SSU72	2.006971
ST13	2.5757952
ST3GAL1	3.2305298
ST6GALNAC4	0.9838939
ST7	2.0019312
ST7L	1.7868748
STAB1	1.3834052
STAG1	1.7020211
STAP2	4.298627
STARD3	1.2015009
STARD5	1.9216075
STAT2	2.4340076
STAT3	2.716607
STAT5A	1.4871073
STAT5B	0.7489414
STAT6	1.2399836
STAU1	1.9473481
STBD1	1.2319927
STEAP1	4.1295233
STFA1	3.0438313
STFA2L1	2.5957632
STIP1	2.931241
STK11	1.1974993
STK16	1.6127954
STK24	1.0475574
STK25	0.7271261

STK3	0.7018862
STK35	1.7971077
STK38	1.3075557
STK40	2.624597
STRA13	1.240067
STRADB	1.5034995
STRIP1	1.5391083
STRN3	1.0513835
STRN4	1.6456738
STT3B	1.3513203
STUB1	1.5478563
STX12	1.5322981
STX16	1.3902822
STX4A	1.5908427
STX8	1.075078
STXBP2	1.4222693
STXBP3A	1.6412444
STXBP3B	1.4143128
SUB1	1.275908
SUCO	2.5202165
SUDS3	1.029305
SUGP1	1.7623954
SUGT1	1.5401044
SUMF1	1.40904
SUMO1	1.5050802
SUMO2	0.86888885
SUN2	1.2615576
SUPT16	2.5505059
SUPT5	1.6237597
SUPT6	1.5591574
SUPT7L	2.8388436
SUPV3L1	2.6377034
SURF2	1.4987006
SURF6	1.6693454
SUSD2	1.9016726
SUV39H2	1.9187708
SUZ12	1.490438
SWT1	1.2343521
SYAP1	1.318471
SYF2	1.1319084
SYK	2.6736755
SYMPK	1.8363385
SYNCRIP	1.5387621
SYNGR2	1.0986257
SYNJ1	1.1733756

SYNJ2BP	1.596797
SYNPO	1.1463361
SYNPO2L	1.6810284
SYPL	0.9919362
SYS1	1.6417732
SZRD1	1.4788957
SZT2	0.86120987
TAB2	1.2883873
TAB3	1.3730497
TACC1	1.0502825
TACR2	1.4819279
TADA1	1.8461652
TADA2A	0.92578316
TADA3	1.0517821
TAF1	0.7344854
TAF10	1.8594985
TAF11	1.2956185
TAF12	1.8437848
TAF15	1.0251832
TAF1B	0.908412
TAF1C	1.3507221
TAF1D	1.0630608
TAF2	1.1131577
TAF3	0.8168473
TAF4A	1.1536336
TAF4B	1.0820282
TAF5	2.0410442
TAF5L	1.6751304
TAF6	1.5711455
TAF6L	1.1772406
TAF7L	1.4322681
TAGAP	1.0538969
TALDO1	1.1250534
TANK	2.3361912
TAP1	2.2094197
TAP2	2.521069
TAPBP	2.0654993
TAPT1	1.7437277
TARBP2	1.6003075
TARDBP	1.5646577
TARM1	3.0553846
TAX1BP1	2.3161988
TAZ	1.435771
TBC1D10B	1.6265192
TBC1D13	1.1910706

TBC1D15	2.5128255
TBC1D17	1.4684834
TBC1D20	1.5816364
TBC1D22A	0.7358761
TBC1D23	1.7480245
TBC1D25	1.2662144
TBC1D2B	1.8069077
TBC1D5	0.9603348
TBC1D8	1.371074
TBC1D9B	1.2985644
TBCB	1.2428436
TBCC	1.8275728
TBCEL	0.75669146
TBK1	2.9431362
TBPL1	1.6199088
TBRG1	2.4305487
TCAIM	1.5848248
TCEA1	2.2073345
TCEB1	2.0207596
TCEB2	1.7908106
TCEB3	1.3628774
TCERG1	1.72924
TCF25	1.5231657
TCIRG1	2.0172415
TCN2	1.4606571
TCOF1	1.1527991
TCP1	1.9711633
TCP11L2	2.1035666
TCTA	1.0764925
TDP2	1.4864697
TDRD7	1.7949166
TEAD1	1.7035189
TEAD3	1.4996893
TELO2	0.8388448
TEP1	0.9493294
TERF1	1.0281916
TESK1	1.4912591
TET2	1.3218999
TEX10	1.2568932
TEX261	1.0850277
TEX264	1.4557824
TEX30	1.6232071
TFAM	1.8393569
TFDP1	1.9296756
TFE3	2.7380223

TFEC	2.2209053
TFIP11	1.6058245
TFPT	1.6236806
TGFBRAP1	1.1215303
TGIF1	2.0610743
TGM1	2.6443205
TGM2	3.0908127
TGOLN1	2.0659776
TGS1	1.5145202
TGTP1	3.4386082
TGTP2	2.495862
THA1	2.9233532
THAP11	0.8339195
THAP4	1.1602101
THAP7	1.320334
THG1L	1.4144175
THOC1	1.2973356
THOC2	1.3164215
THOC3	1.6931806
THOC5	2.0904703
THOC6	1.132432
THOP1	1.1332562
THRAP3	0.9297066
THUMPD1	1.7044926
THUMPD3	1.842485
THYN1	2.351633
TIAL1	1.7552881
TIGD2	1.4055619
TIGD4	1.7465703
TIGD5	1.3293612
TIMM10	2.6970763
TIMM10B	1.7399931
TIMM17A	1.549017
TIMM17B	3.042398
TIMM22	0.92534876
TIMM23	2.683652
TIMM8A1	2.5467572
TIMM9	1.9326315
TIMMDC1	2.0211916
TIMP1	3.460411
TINF2	1.8287001
TIPARP	2.780961
TIPIN	1.0642214
TIPRL	1.5969348
TJAP1	2.0377502

TK2	0.9324212
TKT	1.2714286
TLE1	1.5452614
TLK1	0.96934366
TLK2	1.5921521
TLR2	4.3165045
TLR3	1.1375089
TLR6	2.3953714
TLR7	1.2636323
TM2D1	1.0167427
TM2D2	1.4920459
TM2D3	1.4241247
TM9SF1	1.8304439
TM9SF2	2.1474056
TM9SF3	1.6935692
TMA16	3.2347631
TMBIM4	2.3854356
TMBIM6	1.6908741
TMC7	1.421932
TMC8	0.70524025
TMCO1	1.3172951
TMCO4	1.9708838
TMCO6	2.5259519
TMED10	1.5315013
TMED2	2.137003
TMED3	1.8976936
TMEM106C	1.5152483
TMEM11	1.4957318
TMEM110	0.8349867
TMEM115	1.5494375
TMEM123	1.9473076
TMEM125	1.3249307
TMEM127	1.26228
TMEM128	1.6689081
TMEM129	1.706111
TMEM134	1.1307797
TMEM140	1.8086138
TMEM141	1.1574636
TMEM147	1.4583063
TMEM14C	1.411171
TMEM158	2.1682296
TMEM160	0.99359703
TMEM165	1.0604
TMEM167B	1.1194973
TMEM170B	1.541914

TMEM173	2.2276697
TMEM176B	2.0677757
TMEM179B	1.7494969
TMEM181B-I	1.1795881
TMEM184B	1.3602762
TMEM184C	1.6028676
TMEM185B	1.8880162
TMEM192	1.6704702
TMEM199	1.8729196
TMEM2	2.9171786
TMEM203	1.7048535
TMEM206	1.2847791
TMEM208	1.6751876
TMEM215	0.6454921
TMEM222	1.7306142
TMEM223	0.9883499
TMEM230	1.6811023
TMEM239	1.7890553
TMEM243	1.4718723
TMEM251	1.9803886
TMEM261	1.456419
TMEM30A	1.393373
TMEM33	2.0618324
TMEM37	0.9752922
TMEM50A	2.040121
TMEM51	2.1129575
TMEM55A	0.97722244
TMEM55B	1.4973001
TMEM57	1.0942597
TMEM60	0.88481
TMEM63B	0.9214897
TMEM64	1.0571308
TMEM66	2.0019135
TMEM67	1.2648695
TMEM68	1.7562385
TMEM8	2.0206518
TMEM80	0.96322346
TMEM9B	0.93661594
TMF1	1.4041209
TMOD4	1.5107646
TMPRSS3	2.3089504
TMSB4X	1.1857352
TMTC3	1.1343865
TMUB1	1.1859736
TMX1	1.7065315

TMX2	1.5508833
TMX4	0.918365
TNF	5.8694487
TNFAIP1	1.3605747
TNFAIP2	3.8504605
TNFAIP3	5.195488
TNFAIP8L1	3.4665499
TNFRSF11A	1.2082839
TNFRSF12A	3.0750604
TNFRSF1A	2.1380758
TNFRSF1B	1.6970997
TNFRSF21	1.342617
TNFRSF4	1.8455305
TNFRSF9	1.9615531
TNFSF14	1.6440063
TNFSF9	3.3039308
TNIP1	3.5584035
TNIP2	2.4724336
TNIP3	1.4902315
TNKS1BP1	1.2504001
TNKS2	1.0691004
TNPO1	1.5462284
TNPO3	2.4955597
TNRC6B	0.7901716
TOB2	1.6544552
TOE1	1.2203119
TOLLIP	1.3194857
TOM1	1.3973651
TOM1L1	1.1387281
TOM1L2	1.2248645
TOMM20	1.7976322
TOMM34	1.1335382
TOMM5	1.0584111
TOMM6	2.034752
TOMM7	1.4947457
TOP1	1.7973142
TOP3A	1.3044729
TOP3B	0.8813443
TOPORS	1.8956604
TOR1A	2.0542812
TOR1AIP1	1.345665
TOR1B	1.0621986
TOX	1.6915517
TOX4	1.8718648
TPD52	1.9366488

TPD52L2	1.352447
TPP1	1.5439091
TPP2	1.2883339
TPR	0.70612144
TPRN	0.8578718
TPST2	0.8653388
TPT1	1.3233261
TRA2A	2.1080737
TRADD	1.3656964
TRAF1	2.0761452
TRAF2	2.5938106
TRAF3	1.5550995
TRAF6	1.6272502
TRAFD1	1.9984851
TRAK2	1.5533118
TRAPPC10	1.3651037
TRAPPC13	1.8490715
TRAPPC2	0.7430625
TRAPPC2L	0.99523973
TRAPPC4	1.5318065
TRAPPC6A	1.0893741
TRAPPC6B	1.8389006
TRAPPC8	1.015657
TREM1	2.0194473
TREM3	2.7372956
TREML4	0.7684288
TREX1	2.7549334
TRIAP1	0.93081427
TRIB1	3.381733
TRIM11	1.7432904
TRIM12A	1.6998563
TRIM12C	1.5444522
TRIM14	1.8352976
TRIM2	1.4422307
TRIM21	1.7727466
TRIM23	1.3754668
TRIM24	2.281971
TRIM25	1.6859136
TRIM26	0.93673944
TRIM27	2.3085666
TRIM28	1.4729004
TRIM3	0.8801155
TRIM30A	2.5570407
TRIM30B	1.9247203
TRIM30D	3.0065908

TRIM33	1.0350633
TRIM34A	1.5514116
TRIM34B	1.6645079
TRIM41	1.5544677
TRIM43C	0.7427752
TRIM46	0.73321176
TRIM47	1.9420972
TRIM5	1.3364165
TRIM56	1.7971921
TRIM8	1.3192635
TRIO	1.0237579
TRIOBP	1.1668363
TRIP10	1.8372588
TRIP11	1.5002551
TRIP13	1.0062168
TRIP4	1.3231816
TRIP6	1.8839488
TRMT11	1.450273
TRMT112	1.489963
TRMT13	1.0391922
TRMT1L	1.8636427
TRMT2A	1.861671
TRMT2B	1.0090885
TRMT61A	3.5188708
TRNAU1AP	1.6241007
TRNT1	1.7708101
TRP53BP1	1.5572064
TRP63	1.0970373
TRPC4AP	1.7588911
TRRAP	0.8104441
TSC1	1.2438545
TSEN54	1.8781705
TSG101	1.5492358
TSN	1.2813287
TSNAX	1.3881402
TSPAN14	1.2022128
TSPAN4	2.317183
TSPYL1	0.89508724
TSR2	1.1792326
TSR3	1.7223496
TSTD2	1.8981867
TTBK2	1.1649451
TTC1	2.0657816
TTC14	1.004899
TTC17	1.5238934

TTC27	1.4133253
TTC30A1	1.1109672
TTC33	1.0465698
TTC38	1.6445384
TTC4	1.2102389
TTC7B	0.89201593
TTC9C	1.5966463
TTF2	0.78982306
TTI1	1.6568556
TTPAL	2.2745423
TUBA4A	1.7914824
TUBA-RS1	1.0136137
TUBB4A	0.9066925
TUBB4B	1.3622222
TUBB5	1.1380091
TUBB6	2.5082555
TUBGCP2	0.90590715
TUBGCP3	1.5469351
TUBGCP4	1.3719769
TUG1	1.1329236
TWF1	1.8884034
TXN1	2.0734143
TXNDC11	2.159667
TXNDC12	1.6393309
TXNDC17	1.3202968
TXNDC9	1.6329322
TXNL4B	1.1536255
TYMS-PS	1.1517015
TYW5	1.4066219
U2AF1	1.809895
U2SURP	0.6913624
UAP1	2.770893
UBA2	1.1470089
UBA3	1.0426786
UBA52	1.3825016
UBA6	1.6794024
UBA7	1.1401172
UBAC1	0.9533849
UBALD2	1.3237491
UBAP1	1.9356985
UBAP2	1.0808172
UBAP2L	0.7045312
UBB	1.5313787
UBC	1.7937193
UBD	3.5077682

UBE2A	1.3462362
UBE2B	1.8191938
UBE2D1	0.9421439
UBE2D2A	1.4612966
UBE2D3	2.3440995
UBE2E1	1.334765
UBE2F	2.7897396
UBE2G1	1.4485593
UBE2G2	1.6205816
UBE2H	0.705966
UBE2I	0.9876461
UBE2J2	1.7608991
UBE2K	1.5184479
UBE2L3	1.6968427
UBE2L6	2.1393943
UBE2M	1.4728146
UBE2N	1.4129109
UBE2O	0.8407712
UBE2Q1	1.2282295
UBE2Q2	1.5562496
UBE2W	1.3475575
UBE2Z	1.6689296
UBE3A	1.5471063
UBE3B	0.9919696
UBE3C	1.4691715
UBIAD1	1.5751367
UBL5	1.3621144
UBLCP1	1.4066892
UBN1	1.827796
UBP1	1.6404057
UBQLN1	2.012889
UBQLN2	1.0927792
UBR2	1.3566384
UBR4	1.9173722
UBR5	1.3888245
UBR7	1.5959401
UBTD1	1.44027
UBTF	2.1253266
UBXN1	1.8260255
UBXN2A	1.2422924
UBXN2B	1.2782266
UBXN6	1.5210567
UBXN7	0.9982791
UCHL4	1.9490566
UCHL5	1.7834849

UCK1	1.9186277
UCK2	2.5497055
UCKL1	1.5062494
UFD1L	2.2660046
UGCG	3.1903234
UGGT1	2.5052238
UHMK1	0.83321524
UHRF1BP1L	1.5477233
UHRF2	2.0741892
ULK2	1.0993581
ULK3	1.6283722
UMPS	2.1923976
UNC119B	3.2207756
UNC45A	1.6267633
UNC93B1	1.6378069
UNG	1.8495474
UNK	2.6073852
UNKL	0.87213516
UPF1	0.85154486
UPF3A	1.2244287
UPF3B	1.3837328
UQCC2	1.5014801
UQCRB	2.2559743
URB1	1.8187037
URB2	2.1839218
URGCP	1.4023223
URI1	0.915241
USE1	1.0677328
USF1	0.9820154
USP1	1.295424
USP10	2.1291866
USP12	1.3132992
USP14	2.7102113
USP15	1.5739794
USP16	2.3306856
USP17LA	1.1491256
USP18	3.0897045
USP19	1.7614222
USP21	1.0735517
USP22	0.8098812
USP24	1.3667881
USP25	1.5226612
USP3	1.2621408
USP30	1.5595784
USP31	0.9343157

USP32	1.6141133
USP33	1.2097082
USP38	2.2539334
USP39	0.89926815
USP4	1.6031637
USP42	1.3504152
USP45	0.880321
USP47	0.96481276
USP48	1.2379751
USP53	2.3649616
USP7	1.4366822
USP9X	1.3270879
USPL1	2.2779803
UTP11L	2.0457702
UTP14A	0.8842468
UTP15	1.2281199
UTP23	2.4206257
UTP3	1.8198233
UTP6	1.4069877
VAMP4	1.1759987
VAPA	1.800252
VAPB	1.4850507
VARS	2.3491054
VASH1	1.0981112
VASN	0.7568197
VASP	2.3511057
VAV1	1.7400427
VBP1	1.3029485
VCAM1	3.0385032
VCAN	1.5526147
VCPIP1	1.9382691
VCPKMT	1.626297
VDAC1	1.0752621
VDAC3	1.4938688
VEGFC	1.8104982
VILL	1.4979763
VIMP	2.3105364
VIPAS39	1.0958834
VMN1R173	1.4029808
VMN1R195	0.7614212
VMN1R197	1.3871782
VMN1R52	1.9533229
VMP1	2.6976814
VNN1	2.1158237
VPS11	1.0918717

VPS13B	1.0377679
VPS16	1.7035651
VPS18	1.6051378
VPS25	1.6423745
VPS26A	1.4308825
VPS26B	1.0060983
VPS28	1.4372911
VPS29	1.3533807
VPS33B	1.4803023
VPS35	1.6107264
VPS37B	1.9907784
VPS39	0.84667635
VPS45	1.1157441
VPS4B	1.931499
VPS52	1.4210382
VPS54	0.8992162
VRK2	1.1710944
VRK3	2.0206356
VTA1	1.8367858
VTI1B	1.7699308
VWA5A	1.3283
VWA9	1.2995038
WAC	1.2615385
WAPAL	1.8143067
WARS	1.8668184
WARS2	1.4631195
WASH	0.8905711
WASL	1.6624813
WBP11	1.9091496
WBP2	1.4249368
WBP5	0.954124
WBSCR16	2.404285
WDFY3	1.382215
WDR1	2.3326874
WDR11	1.2727559
WDR12	1.8763056
WDR26	1.9320474
WDR3	1.9681139
WDR31	2.029828
WDR33	1.3757062
WDR34	2.26154
WDR36	1.8862448
WDR4	2.2859402
WDR43	1.8031721
WDR44	0.8405571

WDR45	1.054224
WDR46	1.8997598
WDR5	1.6638517
WDR53	1.7096901
WDR55	2.239068
WDR59	1.5885715
WDR73	1.2826595
WDR75	1.4375758
WDR81	1.5658207
WDR82	1.3590012
WDR83OS	1.46275
WDR89	1.878593
WDR92	3.3338227
WDYHV1	1.484932
WFDC17	4.0040154
WFDC18	2.641819
WHAMM	2.184979
WHSC1L1	1.3949742
WIPF2	2.085936
WIZ	1.3671126
WNT5B	1.0039983
WNT6	1.8644385
WRAP73	1.5432348
WRNIP1	2.0803924
WSB1	2.2453198
WTAP	1.6903629
WWP2	1.5140352
XAB2	1.1278381
XAF1	1.9998665
XBP1	2.7844763
XCL1	2.6321077
XDH	3.2347255
XIAP	1.23732
XKR8	2.1738133
XLR3B	1.2928498
XPA	1.5998325
XPNPEP1	1.517549
XPO4	1.4942579
XPO6	1.7997603
XPOT	1.3938651
XPR1	0.96308184
XRCC4	1.0573726
XRCC6	1.52875
XRN1	0.97802687
XRN2	1.3384814

YAF2	1.0523033
YAP1	1.051157
YBX1	1.0181704
YBX3	1.3532243
YES1	1.8413064
YIF1B	1.2853727
YIPF1	1.2034631
YIPF4	1.5936275
YIPF6	1.0223336
YJEFN3	0.9936092
YKT6	1.1302676
YME1L1	2.6339808
YPEL5	0.8943758
YTHDC1	1.4557385
YTHDC2	1.7098715
YTHDF1	1.6693773
YTHDF2	1.4325447
YTHDF3	1.8364811
YWHAB	1.4469638
YWHAE	1.7234383
YWHAG	1.4727745
YWHAH	1.4924831
YWHAQ	0.9771638
YWHAZ	1.192666
YY1	1.6610146
ZADH2	1.0523052
ZBED6	1.2972455
ZBP1	2.7112694
ZBTB11	0.73485327
ZBTB12	1.4452891
ZBTB16	3.0085812
ZBTB17	0.873132
ZBTB18	0.7467041
ZBTB20	2.0489058
ZBTB24	1.4796352
ZBTB25	0.8612857
ZBTB33	1.5584302
ZBTB39	1.3594449
ZBTB40	1.9420733
ZBTB43	1.3450618
ZBTB5	0.9238992
ZBTB6	0.94647074
ZBTB7B	1.030848
ZC2HC1C	1.3173671
ZC3H11A	2.0122423

ZC3H12A	2.07308
ZC3H12C	1.3087819
ZC3H15	2.1022825
ZC3H18	1.8103628
ZC3H3	1.7404795
ZC3H4	0.7721081
ZC3H7A	2.3576717
ZC3HAV1	1.8753595
ZC3HC1	0.9693551
ZCCHC10	1.1988926
ZCCHC17	1.4442897
ZCCHC2	1.918262
ZCCHC6	1.380022
ZCCHC7	1.6157522
ZCCHC8	1.8685215
ZCCHC9	1.8840456
ZCRB1	1.6502771
ZDHHC13	1.9189548
ZDHHC16	1.5118032
ZDHHC17	1.2270851
ZDHHC2	1.3659039
ZDHHC21	1.0062613
ZDHHC24	0.6463022
ZDHHC5	1.6540508
ZDHHC7	1.2843995
ZFAND1	0.92034197
ZFAND2B	1.6400347
ZFAND3	1.2195606
ZFAND4	2.2472866
ZFAND5	1.1995792
ZFAND6	1.8077059
ZFAT	1.160018
ZFC3H1	2.1431313
ZFML	1.3008122
ZFP1	1.069664
ZFP106	1.2898998
ZFP110	1.9128032
ZFP119B	1.2924604
ZFP120	0.84099054
ZFP131	1.9523377
ZFP133-PS	1.3856487
ZFP14	1.2158632
ZFP148	0.8759594
ZFP180	2.4058747
ZFP191	0.995224

ZFP202	1.7226701
ZFP207	1.5409751
ZFP212	0.9989953
ZFP213	1.4760518
ZFP236	2.8541422
ZFP26	0.83993626
ZFP260	1.4072285
ZFP263	2.2819424
ZFP275	2.0361466
ZFP276	1.4724498
ZFP277	0.77884054
ZFP280B	1.3032832
ZFP280D	0.85981226
ZFP281	2.212069
ZFP292	1.539321
ZFP316	0.6812072
ZFP317	1.3004639
ZFP319	0.8171277
ZFP326	1.4327407
ZFP330	3.0767465
ZFP335	2.0438123
ZFP341	1.286037
ZFP35	1.206419
ZFP36	4.7424855
ZFP367	0.75439
ZFP36L1	1.9374518
ZFP384	1.7155585
ZFP397	2.0544446
ZFP407	1.1585307
ZFP408	1.3745394
ZFP410	1.8027744
ZFP426	1.3302336
ZFP429	2.3819933
ZFP433	1.9031496
ZFP445	1.1018357
ZFP446	1.4273589
ZFP451	0.9371362
ZFP457	1.2368546
ZFP458	1.9271829
ZFP488	1.849885
ZFP51	0.89598846
ZFP511	1.0850635
ZFP512	1.0525784
ZFP513	1.966124
ZFP518A	0.79452634

ZFP518B	0.9460993
ZFP524	0.8102913
ZFP53	1.0453508
ZFP553	0.6944752
ZFP566	0.9641938
ZFP574	1.0635738
ZFP58	0.9744587
ZFP592	1.409791
ZFP593	1.8179603
ZFP597	0.8732538
ZFP62	1.1194334
ZFP622	1.4235287
ZFP626	0.8550401
ZFP628	1.2519917
ZFP639	1.2186294
ZFP653	1.5870414
ZFP655	1.846734
ZFP658	1.6574783
ZFP668	1.4119258
ZFP68	0.778656
ZFP687	1.073895
ZFP691	2.3639297
ZFP706	1.0771213
ZFP708	1.0408261
ZFP709	1.2122769
ZFP719	0.8394027
ZFP747	1.1207213
ZFP748	1.2641315
ZFP758	0.73436
ZFP770	1.3088632
ZFP777	1.1566
ZFP791	2.0599947
ZFP800	2.2036812
ZFP809	2.4233017
ZFP830	1.3622022
ZFP865	0.801327
ZFP867	0.9897094
ZFP868	1.8216014
ZFP869	1.4191647
ZFP87	0.9673891
ZFP871	1.3674264
ZFP91	1.0432849
ZFP931	1.4754472
ZFP935	0.8162401
ZFP937	0.7133491

ZFP943	1.0742264
ZFP947	1.1280355
ZFP954	1.5866618
ZFP955A	1.2854781
ZFP959	1.7240252
ZFP960	0.9338093
ZFP963	1.7226181
ZFR	1.1119862
ZFYVE16	1.2126594
ZFYVE20	1.4459171
ZFYVE26	2.0862236
ZFYVE27	1.1344166
ZHX1	1.2835116
ZHX2	0.8335812
ZHX3	0.85367346
ZIK1	1.0388987
ZKSCAN17	1.1602836
ZKSCAN3	1.627665
ZKSCAN6	1.7746677
ZMAT1	1.36355
ZMAT2	1.7752657
ZMPSTE24	1.3873482
ZMYM1	0.8737583
ZMYM2	0.7626128
ZMYND15	2.043939
ZMYND19	1.8272476
ZNFX1	2.186956
ZNHIT1	1.9458413
ZNHIT3	0.72243905
ZNHIT6	1.2559352
ZNRD1	1.6157227
ZNRF1	2.9873724
ZNRF2	1.4767616
ZPR1	2.5355244
ZRANB1	1.6367064
ZRANB2	1.866879
ZSCAN21	1.283256
ZSCAN22	1.9661529
ZSWIM7	1.9825897
ZSWIM8	1.4405146
ZUFSP	2.285717
ZW10	1.9599695
ZWINT	2.5599804
ZYG11B	1.2889154
ZYX	2.2785459

ZZEF1	1.1583748
ZZZ3	1.2515526

Mice_onset_liver_DEgenes_fc

Gene	Log2 Fold Change
A4GNT	-7.1721334
AADACL3	-2.5918586
AADAT	-2.5883238
AAMDC	-1.6425028
AARD	-4.5775533
AARS2	-0.5318263
ABCA12	-6.1048613
ABCA13	-1.700203
ABCA16	-2.4572964
ABCA2	-1.2202282
ABCA3	-0.7845552
ABCA4	-2.7949917
ABCA8A	-4.442295
ABCA9	-3.19486
ABCB1A	-0.5779545
ABCB9	-0.6662781
ABCC12	-3.3919325
ABCC8	-0.4221308
ABCD1	-0.6747162
ABCG1	-0.981549
ABCG3	-3.6526644
ABHD12B	-2.655893
ABHD14A	-0.7749364
ABHD16B	-1.3454797
ABHD8	-1.5175226
ABI3BP	-0.8579376
ABLIM1	-1.1606433
ABLIM2	-3.4791985
ABLIM3	-1.9150407
ABT1	-3.0353274
ACAD8	-0.7712364
ACAN	-1.651398
ACAP1	-1.1893699
ACBD7	-4.5162096
ACE	-5.511085
ACE2	-4.521343
ACER1	-3.4636478
ACHE	-2.8503542
ACKR2	-0.8712266
ACKR3	-0.5381229
ACOT5	-3.9363332
ACOT7	-1.1045415
ACOXL	-6.1037474

ACPT	-5.0255356
ACR	-5.359834
ACSBG1	-1.5680976
ACSBG2	-5.232827
ACSL6	-2.7221541
ACSM4	-2.3473594
ACSS1	-4.1672573
ACSS2	-2.4577317
ACSS3	-2.519436
ACTA1	-4.258929
ACTA2	-3.274879
ACTC1	-8.959878
ACTG2	-3.956104
ACTL7A	-2.1444714
ACTL7B	-5.0445423
ACTL9	-2.315448
ACTN2	-4.289836
ACTRT2	-3.656799
ACVRL1	-1.981209
ACYP2	-2.150041
ADAD1	-0.8254104
ADAD2	-2.4817972
ADAM12	-2.9601274
ADAM15	-1.1641252
ADAM18	-5.0059986
ADAM19	-1.7461994
ADAM1A	-2.1704774
ADAM2	-2.5063627
ADAM21	-2.2826123
ADAM22	-3.1781778
ADAM24	-1.9484129
ADAM25	-2.269978
ADAM26A	-2.4195964
ADAM26B	-2.1253557
ADAM28	-4.1507072
ADAM30	-2.5216157
ADAM32	-0.9222832
ADAM33	-2.7365053
ADAM39	-3.9378715
ADAM4	-0.866117
ADAM6B	-1.9819431
ADAMTS10	-3.4872773
ADAMTS12	-2.7158115
ADAMTS15	-1.5187237
ADAMTS16	-3.7151728

ADAMTS17	-3.82005
ADAMTS18	-2.7695076
ADAMTS2	-1.0271919
ADAMTS3	-1.9184418
ADAMTS5	-3.624414
ADAMTS6	-0.9465599
ADAMTS8	-3.815812
ADAMTSL2	-2.5277789
ADAMTSL4	-1.1381044
ADAMTSL5	-2.3007212
ADARB1	-0.3730657
ADARB2	-1.762188
ADC	-4.109031
ADCY10	-2.6450512
ADCY2	-3.454515
ADCY3	-4.416996
ADCY4	-2.012739
ADCY5	-3.524454
ADCY6	-1.1928179
ADCY7	-0.9072526
ADCY8	-6.7777796
ADCYAP1R1	-1.8873262
ADD3	-1.9688776
ADGB	-1.1905732
ADH6A	-2.5270448
ADIG	-2.53751
ADIPOQ	-5.9006653
ADORA2B	-1.1609132
ADORA3	-1.080363
ADPRHL1	-2.9902604
ADRA1A	-1.8936701
ADRA1D	-4.2822742
ADRA2B	-2.513542
ADRA2C	-2.328401
ADRB1	-2.9855003
ADRB3	-1.9543154
AEBP1	-1.3866374
AFAP1	-1.9291096
AFAP1L1	-3.2402377
AFAP1L2	-1.4428515
AFF3	-2.0694034
AGAP1	-0.4053133
AGAP2	-0.91869
AGBL2	-3.9932044
AGBL4	-4.3466206

AGBL5	-1.2718
AGER	-10.061924
AGO1	-0.8035069
AGPAT4	-1.3973658
AGR2	-5.366805
AGR3	-5.552376
AGRN	-0.8635323
AGRP	-4.252938
AGTPBP1	-0.7590792
AGTR2	-2.907146
AHDC1	-0.8827338
AHI1	-1.5139908
AHNAK	-3.5933628
AHRR	-2.0566
AHSA2	-2.3372838
AHSP	-2.8827393
AICDA	-2.9806683
AIF1L	-3.5759156
AIFM3	-2.345757
AIM1L	-2.7996223
AIRE	-2.9279773
AJAP1	-3.0845852
AJUBA	-4.0599365
AK1	-3.5757005
AK8	-4.198618
AKAP14	-2.5529687
AKAP17B	-1.4357972
AKAP3	-3.7793388
AKAP5	-9.191523
AKNA	-0.3232935
AKR1B3	-1.0651515
AKR1C14	-2.6648357
AKR1D1	-1.6376
AKT3	-1.0091045
ALAS2	-0.6509583
ALDH1A1	-2.5113184
ALDH1A2	-3.2956219
ALDH1A7	-1.3843019
ALDH3A1	-1.8346407
ALDH3A2	-0.278157
ALDH3B1	-1.0035002
ALDOART1	-4.230694
ALDOART2	-2.3045826
ALMS1	-4.822544
ALOX12B	-3.3455446

ALOX12E	-1.5040584
ALOX15	-4.5697784
ALOX5	-2.7408578
ALOX5AP	-0.7194297
ALOXE3	-2.2014256
ALPK2	-4.1470156
ALPK3	-2.9846673
ALX3	-2.2145789
AMBN	-4.892886
AMER2	-0.8259077
AMHR2	-3.880726
AMOTL1	-0.5492547
AMOTL2	-2.1709454
AMPH	-5.9595685
AMT	-2.6192195
AMTN	-2.7584357
AMZ1	-1.4630396
ANGEL1	-1.1930969
ANGPT1	-5.6773996
ANGPT4	-3.2260067
ANGPTL1	-4.226357
ANGPTL7	-2.9425707
ANK2	-2.267811
ANK3	-2.269581
ANKEF1	-2.2529716
ANKFN1	-4.2032228
ANKLE1	-3.4781203
ANKMY2	-0.8815424
ANKRD1	-1.7032721
ANKRD12	-0.7015178
ANKRD29	-3.924153
ANKRD33	-3.7023792
ANKRD33B	-0.9203396
ANKRD34A	-5.1719227
ANKRD36	-5.426233
ANKRD42	-2.2394688
ANKRD44	-1.9369125
ANKRD45	-0.3599179
ANKRD50	-0.8800814
ANKRD53	-2.0070703
ANKRD55	-1.9756813
ANKRD6	-3.9419866
ANKRD60	-3.9496784
ANKRD61	-1.7649052
ANKRD63	-3.944637

ANKRD65	-1.7329412
ANKRD7	-2.9979212
ANKUB1	-3.188878
ANLN	-1.5051606
ANO1	-2.7367191
ANO3	-3.986185
ANO6	-1.2777884
ANO7	-3.8124778
ANO8	-2.1942134
ANO9	-3.6124682
ANTXR1	-2.317818
ANTXRL	-0.5797393
ANXA1	-1.2978699
ANXA10	-3.8598697
ANXA13	-3.163598
ANXA2	-0.6346033
ANXA3	-1.6504824
ANXA8	-2.768167
ANXA9	-1.0714519
AOC1	-2.661462
AOC3	-2.1787167
AOX3	-4.420767
AP1G2	-1.9535947
AP1M2	-3.3301668
AP1S2	-0.5353117
AP1S3	-1.1773658
AP3B2	-3.19544
APBA1	-2.632963
APBA2	-2.624031
APBB1	-3.9158473
APBB2	-0.8698764
APC	-1.0059745
APC2	-1.7950506
APCDD1	-1.6946483
APEX2	-1.234365
APLF	-2.1272159
APLN	-5.2146006
APLNR	-7.5114365
APOBEC4	-1.1225386
APOD	-4.731511
APOL10A	-3.4808807
APOL11B	-3.012017
APOL6	-3.9931412
APOL7D	-1.7946396
APTX	-1.7037487

AQP1	-0.4767878
AQP12	-1.1479847
AQP3	-1.5618782
AQP4	-1.578085
AQP5	-7.9881296
AQP6	-4.176865
ARAP3	-0.9442351
ARC	-1.2010958
ARHGAP15	-1.6982608
ARHGAP18	-1.3605418
ARHGAP20	-3.12796
ARHGAP20OS	-2.5734792
ARHGAP22	-1.4672713
ARHGAP23	-0.4960129
ARHGAP27	-3.7123928
ARHGAP28	-1.8589885
ARHGAP31	-1.2175915
ARHGAP33	-3.8877468
ARHGAP33OS	-3.6821635
ARHGAP40	-3.4107363
ARHGAP44	-2.853748
ARHGAP6	-2.437663
ARHGAP8	-2.2476232
ARHGAP9	-0.6944001
ARHGDIG	-5.22362
ARHGEF1	-0.3850195
ARHGEF17	-3.3795927
ARHGEF19	-0.8187707
ARHGEF25	-3.1294534
ARHGEF28	-5.7627378
ARHGEF33	-2.3412273
ARHGEF38	-4.373337
ARHGEF39	-3.049125
ARHGEF4	-2.0704727
ARHGEF6	-1.6997502
ARHGEF9	-2.8047512
ARID3B	-0.4659028
ARID3C	-4.5027876
ARL11	-0.4570848
ARL13A	-2.4083476
ARL14	-1.3588204
ARL14EPL	-1.9117675
ARL2	-1.6829693
ARL5C	-0.7314813
ARL9	-0.6752191

ARMC12	-4.8724766
ARMC2	-2.4682245
ARMC3	-3.8337078
ARMC4	-4.7973146
ARMC6	-0.4671781
ARMC9	-2.054132
ARMCX1	-1.5966167
ARMCX2	-0.4369581
ARMCX4	-1.0334058
ARMCX6	-0.98967
ARNT2	-5.1374054
ARPP21	-3.4075952
ARR3	-1.3910093
ARRB1	-1.4871628
ARRDC1	-1.8546402
ARRDC3	-1.0825534
ARRDC5	-4.2557573
ARSB	-1.9031701
ARSJ	-3.016074
ART1	-2.8845425
ART3	-2.2449968
ART4	-4.7504954
ART5	-2.1881166
ARV1	-0.3369701
ARVCF	-2.6983452
ARXES2	-4.3292007
ASAH2	-0.4026485
ASB1	-0.4061589
ASB10	-6.344965
ASB11	-2.8298242
ASB16	-2.6331673
ASB18	-3.4040937
ASB3	-1.3393307
ASB5	-3.1320198
ASB9	-3.3421135
ASCL3	-3.1204672
ASF1A	-0.8892839
ASF1B	-0.7905235
ASIC1	-4.367959
ASIC4	-2.1935854
ASIC5	-0.5829511
ASPHD1	-0.8039408
ASPHD2	-3.156267
ASPM	-1.0373728
ASTL	-4.5695305

ASTN2	-6.2421703
ASTX	-5.725442
ASXL3	-2.686323
ATAD2	-1.8932288
ATF7	-1.8475931
ATF7IP2	-3.2935932
ATG4A	-1.4720745
ATG9B	-3.6955957
ATMIN	-0.3400986
ATOH8	-1.6313856
ATP10A	-1.2068341
ATP13A4	-4.5609593
ATP13A5	-2.523815
ATP1A2	-4.694298
ATP1A3	-1.2864087
ATP1A4	-2.8874419
ATP1B2	-3.4239805
ATP2A1	-0.376276
ATP2A3	-3.6901934
ATP2B4	-3.529076
ATP2C1	-0.3648694
ATP2C2	-0.9862495
ATP6AP1L	-3.1298368
ATP6V0D2	-5.4191394
ATP6V0E2	-1.8323073
ATP6V1B1	-4.686256
ATP6V1C2	-6.9667997
ATP6V1E2	-2.9262204
ATP6V1G2	-1.1751385
ATP6V1G3	-2.654291
ATP7B	-1.3597782
ATP8A2	-4.1543207
ATP8B3	-2.9726288
ATRNL1	-0.5664427
ATXN1	-1.0016334
ATXN7L3	-0.4113801
AWAT2	-2.3813555
B3GALT5	-3.3677416
B3GAT2	-4.304005
B3GLCT	-0.9687789
B3GNT3	-5.0048904
B3GNT4	-1.2016809
B3GNT5	-2.2151027
B3GNT7	-3.940243
B3GNT9	-1.9561906

B3GNTL1	-1.1982374
B4GALNT4	-1.0175114
B4GALT2	-2.240263
B4GALT4	-2.668468
B9D1	-1.20452
BACH2	-1.2934968
BAG2	-2.7894871
BAI1	-5.5045094
BAI2	-2.3679295
BAI3	-3.3079078
BAIAP2	-0.4630048
BAIAP3	-5.5223284
BANF2	-2.6923234
BANK1	-1.6508944
BARD1	-1.2550218
BARX1	-2.6694572
BARX2	-1.1954515
BAZ1B	-1.7313807
BBS1	-0.478226
BC021767	-0.6342795
BCAM	-3.8687496
BCAN	-4.597036
BCAS1	-1.877173
BCAS1OS2	-1.7203975
BCAS3	-0.752512
BCAT1	-3.434213
BCAT2	-0.2530603
BCL11B	-3.7311869
BCL2	-0.7560213
BCL2L12	-0.9212942
BCL6B	-0.5948923
BCL9L	-1.9430611
BCORL1	-1.2192984
BDH1	-4.0151196
BDKRB2	-4.761613
BDNF	-3.7561316
BE949265	-2.3523433
BEND4	-2.0139685
BEND6	-1.9566729
BEND7	-0.9793859
BEST1	-2.2729716
BEX1	-2.8986166
BEX2	-1.0027969
BEX4	-1.6038611
BEX6	-4.9374523

BFSP1	-2.5923555
BFSP2	-1.9037917
BGLAP	-2.4514384
BGN	-1.548029
BHLHA9	-1.8496785
BHLHB9	-1.4029217
BHLHE22	-0.5607576
BHLHE23	-3.4383225
BHLHE41	-4.255904
BICC1	-1.0457532
BIK	-1.2935627
BIN2	-1.9122932
BIRC5	-0.7235808
BLK	-2.970181
BLNK	-1.6503694
BMF	-1.9106202
BMP10	-2.7384405
BMP3	-4.6660266
BMP4	-2.4188166
BMP5	-3.806571
BMP6	-1.9090426
BMP7	-4.1539583
BMP8B	-1.4249561
BMPR2	-1.0638506
BMX	-2.509581
BMYC	-0.6599209
BNC1	-1.8943877
BNIPL	-2.9547734
BOC	-4.2434134
BOLA1	-0.5789666
BPGM	-0.3855479
BPI	-3.2143326
BPIFA1	-6.5019846
BPIFA2	-3.3932157
BPIFA3	-3.3369908
BPIFA5	-2.365233
BPIFA6	-3.1000037
BPIFB1	-3.6400843
BPIFB4	-0.8980451
BPIFB5	-4.6745586
BPIFB6	-3.3301008
BPIFB9B	-5.2891397
BRF1	-0.671046
BRICD5	-2.7438176
BRINP1	-2.2677932

BRINP3	-5.4585495
BRS3	-2.8918483
BRSK1	-0.6051555
BRSK2	-2.9353902
BSN	-2.3327038
BSND	-5.6617002
BSPH1	-2.6316228
BSPRY	-3.8958976
BSX	-3.6705112
BTBD11	-0.4511244
BTBD2	-0.5831945
BTBD3	-1.5670779
BTBD8	-1.1991131
BTG4	-2.9316466
BTLA	-2.1045935
BTNL1	-4.0505037
BTRC	-1.3533094
BUB1	-0.806201
BUB1B	-0.6335738
BZRAP1	-4.2965865
C1D	-1.2952633
C1QL1	-2.6876411
C1QL2	-4.314613
C1QL4	-5.635515
C1QTNF2	-4.6098814
C1QTNF3	-3.3100462
C1QTNF4	-3.6503408
C1QTNF5	-1.380343
C1QTNF6	-0.8477855
C1QTNF7	-3.8879855
C2CD3	-0.4152958
C2CD4A	-2.0623121
C2CD4B	-5.4838243
C2CD4C	-4.4398637
C2CD4D	-1.6001668
C5AR2	-0.3098347
C6	-0.7394724
C7	-3.2050884
CABP2	-2.7081552
CABP4	-2.2877722
CACHD1	-3.278361
CACNA1A	-0.7867904
CACNA1B	-3.0189157
CACNA1C	-4.535331
CACNA1D	-5.455304

CACNA1E	-4.9444942
CACNA1F	-1.2574404
CACNA1H	-0.8930416
CACNA1I	-3.819404
CACNA1S	-3.0649219
CACNA2D1	-2.2716856
CACNA2D3	-2.151605
CACNA2D4	-3.0450726
CACNB1	-1.4380215
CACNB2	-2.7665489
CACNB3	-2.1349125
CACNB4	-1.9296608
CACNG1	-3.8703244
CACNG2	-1.9706392
CACNG4	-2.5839317
CACNG8	-4.817683
CADM1	-1.0697734
CADM3	-1.4279401
CALB1	-4.001606
CALB2	-5.0267496
CALCA	-2.3070555
CALCB	-2.7581544
CALCOCO1	-1.0565
CALCOCO2	-4.6915236
CALCR	-2.659916
CALCRL	-4.5897226
CALHM1	-4.792267
CALM4	-3.4795473
CALML3	-3.6814857
CALML4	-1.1468513
CALY	-0.7960417
CAMK1	-1.0518208
CAMK1G	-2.512897
CAMK2A	-1.413291
CAMKK2	-0.5580194
CAMKV	-4.6115117
CAMP	-0.3803294
CAMTA1	-2.810702
CAP2	-2.2127235
CAPN1	-2.3665764
CAPN12	-0.9709878
CAPN15	-0.4541884
CAPN2	-0.4820063
CAPN3	-1.3332014
CAPN5	-3.801419

CAPN9	-2.7103767
CAPNS2	-1.8188934
CAPS2	-3.199904
CAPSL	-4.1728477
CAR1	-2.8645246
CAR10	-2.0035853
CAR14	-0.7261238
CAR15	-1.1849816
CAR2	-1.8162086
CAR3	-0.798044
CAR5B	-1.4561677
CAR6	-4.6296673
CARD11	-2.7226787
CARD9	-2.2488403
CARF	-1.3278167
CARLR	-4.253886
CARTPT	-3.133894
CASC1	-2.4438362
CASC4	-0.4686303
CASC5	-1.7024968
CASD1	-0.2753272
CASP12	-1.783391
CASP16	-3.9830163
CASP3	-1.7718928
CASQ1	-3.1464937
CASQ2	-3.4347646
CASZ1	-0.8974705
CATIP	-1.9782951
CATSPER1	-2.028378
CATSPER4	-2.9663398
CATSPERB	-3.4444532
CATSPERG1	-1.4763288
CAV1	-5.3094597
CAV2	-5.2129755
CAV3	-4.326257
CBFA2T3	-3.248424
CBLN1	-1.5118098
CBLN2	-4.485105
CBR2	-12.63266
CBR3	-1.2772076
CBX5	-1.6772068
CBX6	-2.5936182
CBY1	-1.8525617
CC2D2A	-1.7360902
CCBE1	-1.4977648

CCDC102A	-0.7032392
CCDC103	-1.8531625
CCDC105	-3.130529
CCDC108	-3.1817832
CCDC109B	-2.2596633
CCDC11	-5.6829076
CCDC110	-2.296487
CCDC112	-2.8958764
CCDC113	-5.9360676
CCDC114	-0.6679218
CCDC116	-2.0017595
CCDC121	-4.153556
CCDC122	-1.9620183
CCDC129	-7.649254
CCDC130	-2.036723
CCDC136	-3.884502
CCDC14	-3.4824584
CCDC141	-3.1132598
CCDC142	-2.2161078
CCDC146	-1.8488448
CCDC147	-3.8156152
CCDC149	-2.2314615
CCDC15	-1.5682216
CCDC150	-2.4268963
CCDC152	-4.830906
CCDC153	-7.2733173
CCDC154	-2.3218565
CCDC155	-3.972136
CCDC158	-4.2128286
CCDC162	-1.0307395
CCDC17	-1.1409051
CCDC171	-2.2279985
CCDC172	-3.1943634
CCDC176	-1.8252239
CCDC177	-2.2281952
CCDC18	-2.224219
CCDC183	-3.097693
CCDC185	-3.954113
CCDC19	-3.6845198
CCDC24	-3.6441677
CCDC27	-6.5327826
CCDC28B	-0.3128083
CCDC30	-2.258757
CCDC33	-3.2769203
CCDC34	-3.2243698

CCDC37	-3.5351658
CCDC39	-2.87122
CCDC40	-4.9994392
CCDC42	-2.5706952
CCDC42B	-0.878464
CCDC42OS	-4.8439794
CCDC50	-4.50473
CCDC51	-1.0825808
CCDC54	-2.3769422
CCDC62	-4.035125
CCDC63	-1.4475741
CCDC64	-1.1633658
CCDC64B	-3.1356936
CCDC65	-3.1577682
CCDC67	-1.853154
CCDC7	-1.8092413
CCDC73	-4.496309
CCDC74A	-2.4302518
CCDC8	-3.6031651
CCDC83	-2.653716
CCDC85A	-5.180965
CCDC85B	-3.0401566
CCDC85C	-1.0570164
CCDC87	-3.509605
CCDC88C	-0.8212667
CCDC89	-1.5490403
CCDC92	-4.009636
CCHCR1	-0.8950675
CCKAR	-6.166521
CCL1	-1.9592493
CCL12	-0.6425829
CCL20	-3.5445726
CCL24	-1.8986509
CCL25	-1.0563738
CCL26	-3.7016811
CCL27A	-0.9983926
CCL6	-1.4597285
CCL8	-3.24246
CCM2L	-0.9581296
CCNA2	-1.1689954
CCNB1IP1	-2.3929605
CCNB2	-0.6801446
CCND1	-2.316137
CCND2	-1.424926
CCNO	-1.3024282

CCNY	-1.3275268
CCR2	-2.1920862
CCR3	-5.108329
CCR6	-3.9769824
CCSER1	-3.2821925
CCT8L1	-2.3741906
CD101	-3.6729293
CD151	-0.3084471
CD160	-2.8864563
CD163L1	-3.2744439
CD164L2	-3.8584352
CD177	-1.6957226
CD19	-1.4197474
CD2	-1.9864731
CD200R1	-2.206774
CD200R2	-2.9883318
CD200R4	-2.95722
CD207	-2.7799296
CD209A	-3.688334
CD209B	-3.7792866
CD209D	-2.043885
CD209E	-2.621885
CD209F	-3.612603
CD209G	-4.9085846
CD22	-0.9847624
CD226	-2.1850207
CD247	-2.7341294
CD248	-1.2915828
CD24A	-2.4195116
CD27	-1.6327391
CD300C	-1.202574
CD300LB	-2.6764834
CD300LD	-0.9800921
CD300LH	-1.7591302
CD34	-2.798125
CD36	-1.0875871
CD3D	-0.6888549
CD3E	-0.575721
CD3G	-1.4241326
CD40LG	-3.7962584
CD44	-0.6112926
CD46	-1.3055325
CD5	-1.4821696
CD52	-1.0786598
CD59B	-2.7176123

CD6	-0.8451831
CD7	-0.7672019
CD79A	-2.60532
CD79B	-2.802652
CD8A	-1.6508737
CD8B1	-1.087054
CD96	-1.9822556
CDAN1	-2.185313
CDC14A	-1.7180443
CDC20	-1.3147025
CDC20B	-4.0820575
CDC25B	-1.8991885
CDC27	-0.3411129
CDC42BPB	-0.7625771
CDC42BPG	-2.7907722
CDC42EP3	-3.0083914
CDC45	-2.2733617
CDC6	-1.0147314
CDCA2	-1.6420257
CDCA3	-0.5597363
CDCA5	-0.907918
CDCA7L	-1.0205903
CDCA8	-0.660733
CDCP1	-1.303489
CDH1	-2.5712314
CDH10	-4.165737
CDH12	-3.0257933
CDH13	-3.6538587
CDH15	-1.6661172
CDH16	-6.7099214
CDH17	-3.7152991
CDH18	-3.168717
CDH20	-5.599224
CDH22	-3.1531928
CDH23	-2.4140124
CDH24	-1.9148116
CDH26	-4.4228435
CDH5	-0.630861
CDH8	-2.6597126
CDH9	-2.2342787
CDHR3	-2.6448898
CDK14	-3.1319547
CDK15	-2.4787009
CDK19	-0.527591
CDK19OS	-3.228683

CDK5R2	-4.1002364
CDK6	-0.9209263
CDKL1	-2.275113
CDKL3	-2.1286612
CDKL4	-1.187305
CDKL5	-1.728087
CDKN1C	-4.171606
CDKN2A	-1.4513972
CDKN2B	-3.8520215
CDKN3	-3.3369231
CDNF	-2.463898
CDON	-2.0380056
CDR2	-2.935394
CDRT4	-1.9026608
CDRT4OS1	-3.1724973
CDS1	-2.9014573
CDSN	-2.6071036
CDX2	-2.2098374
CEACAM12	-3.2481694
CEACAM13	-3.331368
CEACAM16	-0.4048822
CEACAM20	-4.7036667
CEACAM3	-1.8796005
CEACAM5	-3.357615
CEBPE	-2.4851806
CEL	-2.9371457
CELA1	-2.3007953
CELA3B	-3.6914394
CELF2	-0.8167703
CELF3	-4.0170007
CELF6	-2.9968734
CELRR	-4.9135485
CELSR2	-1.9174259
CEMIP	-3.6583083
CEND1	-1.4744182
CENPA	-0.7913799
CENPE	-0.8556731
CENPF	-0.8378477
CENPH	-0.8917549
CENPI	-5.415908
CENPN	-0.8382192
CENPP	-3.0864954
CENPU	-2.2337964
CEP104	-1.0843091
CEP112	-3.3785033

CEP128	-1.0558777
CEP135	-1.4068744
CEP152	-0.773068
CEP250	-0.8445699
CEP41	-1.0485222
CEP57L1	-3.0657544
CEP78	-2.5965512
CER1	-3.6082773
CERCAM	-2.474366
CERS3	-5.0346317
CERS4	-1.5817118
CES1A	-3.4897337
CES2H	-3.5800586
CES4A	-3.4976099
CES5A	-3.2784061
CETN1	-2.9453812
CETN4	-0.6095254
CFD	-7.942511
CFTR	-2.8361993
CGA	-1.2737613
CGN	-1.0784991
CHAD	-3.7549105
CHADL	-1.4223433
CHAF1A	-2.4414525
CHAT	-2.5262392
CHD5	-3.619713
CHD6	-1.1030772
CHEK2	-3.4173543
CHGA	-2.1844316
CHIA1	-7.9589553
CHIL3	-2.403896
CHIL4	-3.7995758
CHIL5	-2.8456788
CHL1	-4.035235
CHMP4C	-0.8235915
CHN1	-3.8096104
CHP2	-1.884867
CHRDL1	-3.3008847
CHRM2	-2.6007571
CHRM4	-3.9400735
CHRM5	-4.9926457
CHRNA10	-3.7184262
CHRNA2	-1.2783687
CHRNA3	-2.6303189
CHRNA4	-0.7733164

CHRNA5	-3.460417
CHRNA6	-2.96802
CHRNA9	-2.9174798
CHRNB1	-2.3769863
CHRNB3	-4.5171156
CHRNB4	-3.4626303
CHRNG	-3.4723492
CHST1	-5.2019095
CHST12	-1.2519195
CHST2	-2.2104828
CHST3	-3.6325655
CHST4	-3.4132714
CHST5	-2.5337188
CHST7	-1.7111366
CHSY1	-0.3093703
CHTF18	-0.8968606
CIB2	-0.8320081
CIB3	-3.2378163
CIDEA	-2.232064
CIITA	-5.268592
CILP2	-4.5394735
CIT	-3.4694076
CITED1	-1.2818608
CK137956	-4.0653133
CKAP2	-2.2137434
CKAP2L	-1.0109661
CKAP4	-3.3967226
CKB	-2.8323624
CKMT2	-5.3460493
CLASP2	-0.7847185
CLCA3	-7.777214
CLCA5	-5.6278973
CLCN6	-0.6450112
CLCNKA	-2.147676
CLCNKB	-0.4387014
CLDN13	-4.071478
CLDN15	-3.1823359
CLDN16	-4.2633886
CLDN17	-3.3157594
CLDN18	-5.17022
CLDN20	-1.3730004
CLDN4	-2.84872
CLDN5	-5.718268
CLDN6	-3.348473
CLDN7	-1.9651883

CLDN8	-2.7479033
CLDN9	-3.4963312
CLEC10A	-1.726227
CLEC12A	-3.7707663
CLEC12B	-3.9672346
CLEC14A	-3.564147
CLEC18A	-2.6053662
CLEC1A	-5.88367
CLEC2F	-3.2315514
CLEC2H	-4.3761406
CLEC2I	-2.4337022
CLEC2L	-1.8654618
CLEC4A2	-2.581221
CLEC4A3	-0.7221754
CLEC4A4	-3.1858351
CLEC4B1	-1.5286446
CLEC5A	-4.4299297
CLEC7A	-3.9926593
CLIC3	-8.379709
CLIC5	-6.279014
CLIC6	-6.008561
CLIP3	-1.8641703
CLIP4	-4.0847263
CLMP	-5.636949
CLNK	-2.1217647
CLPS	-1.7612605
CLPSL2	-1.9058604
CLRN2	-3.9050784
CLRN3	-3.6298246
CLSTN1	-3.0923975
CLSTN2	-1.535521
CLSTN3	-0.7830334
CLVS1	-2.786617
CMKLR1	-1.5734882
CMTM2A	-3.2878387
CMTM4	-0.3262789
CNBD2	-1.7050177
CNDP1	-4.172367
CNFN	-2.2587392
CNGA2	-2.0873606
CNGA3	-2.380571
CNGB3	-0.5407431
CNIH3	-3.6375837
CNKSR1	-3.569926
CNN1	-6.4864073

CNN2	-1.621249
CNNM1	-1.2965593
CNPY1	-2.9036279
CNPY4	-1.295599
CNR1	-3.0455453
CNRIP1	-1.2696197
CNST	-0.5182686
CNTLN	-1.4764292
CNTN1	-6.103075
CNTN2	-1.9606357
CNTN5	-6.9445333
CNTN6	-1.0528755
CNTNAP3	-4.311499
CNTNAP5A	-5.0684156
CNTNAP5B	-4.260293
COL10A1	-2.9618857
COL11A2	-3.5357714
COL12A1	-2.153037
COL13A1	-1.3110569
COL15A1	-1.590359
COL16A1	-3.0526893
COL1A1	-3.4955711
COL1A2	-3.587537
COL23A1	-4.511297
COL25A1	-3.729396
COL26A1	-2.498645
COL3A1	-2.4085515
COL4A3	-6.73176
COL4A3BP	-1.3452432
COL4A4	-5.400158
COL4A5	-4.61751
COL4A6	-1.9409194
COL5A1	-1.0381761
COL5A2	-3.641494
COL5A3	-0.3622479
COL6A1	-5.541952
COL6A2	-3.496992
COL6A3	-4.2225075
COL6A6	-3.3439329
COL7A1	-2.622065
COL8A1	-2.8140295
COL8A2	-3.5163019
COL9A1	-3.2688384
COL9A2	-1.5550954
COLGALT2	-4.764329

COLQ	-5.1882553
COPG2OS2	-2.7695549
COPS7B	-1.5660169
CORO1A	-1.7371032
CORO2A	-1.358506
CORO2B	-3.0669827
CORO6	-2.0623832
CORT	-2.238481
COX10	-3.841429
COX4I2	-1.8023696
COX6A2	-2.7225404
COX6B2	-1.609731
COX7A1	-1.5297575
COX8B	-6.1608114
CPA1	-3.9070566
CPA2	-4.81476
CPA3	-3.1739223
CPA4	-3.6253755
CPA5	-2.4183109
CPE	-1.5735097
CPEB1	-0.351522
CPHX1	-1.6442116
CPLX3	-3.0955195
CPLX4	-1.9317021
CPM	-4.164617
CPNE4	-2.832051
CPNE7	-1.2210104
CPNE9	-1.6594529
CPSF4L	-1.4947687
CPT1C	-3.2476315
CPVL	-2.3679504
CPXM1	-3.489415
CPXM2	-6.1338043
CPZ	-3.663813
CRABP1	-2.3486726
CRABP2	-0.6560023
CRB2	-1.9063392
CRCT1	-5.1094255
CREG2	-4.1437216
CRH	-3.7460444
CRHBP	-4.7579837
CRHR1	-0.9909012
CRHR2	-1.5857592
CRIM1	-1.8767114
CRIP1	-3.582009

CRIP2	-2.4101212
CRISP3	-2.7072277
CRISPLD2	-0.2748711
CRLF1	-5.597558
CRMP1	-5.162241
CRNN	-6.000537
CROCC	-0.8885198
CRTAC1	-2.1268463
CRXOS	-3.4734921
CRYAA	-2.071374
CRYAB	-3.6333594
CRYBA1	-2.9234369
CRYBA2	-5.1505003
CRYBB1	-2.604739
CRYBB2	-3.394289
CRYBG3	-0.8900809
CRYGA	-0.7222083
CRYGC	-3.1111927
CRYGD	-2.1388025
CRYGF	-3.22717
CRYGN	-2.7272863
CRYL1	-0.4371977
CSDC2	-3.1694188
CSMD2	-4.44141
CSMD2OS	-2.2794285
CSN3	-3.2570117
CSPG4	-4.025898
CSRNP2	-0.5036037
CSRNP3	-0.8136971
CST10	-4.1901836
CST12	-3.967315
CST3	-0.6955245
CST6	-2.7737849
CST8	-5.692548
CSTAD	-2.6071758
CSTL1	-3.3044717
CTBP2	-0.7542107
CTCFL	-2.6359234
CTDSPL	-1.8592541
CTF2	-2.086426
CTGF	-1.7475574
CTNND2	-3.9986408
CTRL	-1.7951367
CTS6	-3.992878
CTS7	-5.175448

CTSK	-3.3706477
CTSW	-1.4591172
CTTNBP2	-2.374616
CTXN1	-4.386861
CTXN2	-4.3149834
CUBN	-1.5313213
CUEDC1	-2.1237812
CUX1	-0.4207077
CUX2	-0.4518662
CX3CL1	-2.4591627
CXCL14	-3.1455402
CXCL15	-8.508532
CXCL17	-1.6698506
CXCR1	-1.1557376
CXCR2	-2.4815626
CXCR4	-2.0431538
CXCR5	-1.3242121
CXCR6	-0.4620678
CXX1A	-1.449898
CXX1B	-2.0790517
CXX1C	-2.3447058
CXXC4	-2.3834493
CYB5R2	-2.992117
CYBRD1	-3.3835835
CYCT	-3.8788
CYFIP2	-0.6692665
CYLC2	-5.013752
CYM	-0.6474328
CYP11B2	-2.5110245
CYP19A1	-2.0357642
CYP1A1	-3.0231264
CYP21A1	-0.4427195
CYP26B1	-1.7921407
CYP26C1	-2.6783066
CYP2A22	-3.7372665
CYP2AB1	-3.110749
CYP2C66	-2.9925885
CYP2F2	-3.063812
CYP2J11	-3.9763498
CYP2J9	-2.4556644
CYP2S1	-7.2052917
CYP2T4	-0.6723421
CYP2W1	-1.5841589
CYP4A29	-5.239087
CYP4B1	-3.481219

CYP4B1-PS2	-3.2175164
CYP4F18	-2.9105287
CYP4F39	-2.9059954
CYP4F40	-3.337152
CYP4F41-PS	-3.0480824
CYP4X1	-4.121257
CYPT12	-4.08853
CYPT2	-2.475719
CYR61	-1.0303218
CYS1	-2.2946632
CYSLTR1	-3.320777
CYTH3	-1.1949265
CYTIP	-0.5189979
CYTL1	-7.144908
CYYR1	-1.5453179
DAAM1	-0.7387278
DAAM2	-2.282497
DAB2IP	-1.003165
DACH1	-3.4532735
DACH2	-1.937712
DACT1	-1.864352
DACT3	-0.4580405
DAG1	-0.4972494
DAND5	-1.2199025
DAO	-4.296154
DAPK2	-1.0645702
DAPL1	-3.9747412
DBH	-4.471038
DBHOS	-3.6512556
DBIL5	-2.7256205
DBNDD1	-2.9639742
DBP	-1.1082551
DCDC2A	-2.9996934
DCDC2B	-2.5702894
DCLK1	-2.5969777
DCLK2	-0.6503031
DCPP1	-3.934432
DCST1	-2.4710374
DCSTAMP	-2.3022602
DCT	-2.1925187
DCTD	-2.9451175
DCUN1D3	-2.6259983
DCX	-2.298738
DCXR	-0.6009085
DDAH1	-0.9018028

DDAH2	-3.1481135
DDIT4L	-2.2442958
DDR2	-2.9023108
DDX11	-2.2525163
DEAF1	-0.265543
DEAR1	-2.7046525
DEFA1	-5.543997
DEFA10	-5.6617427
DEFA21	-3.393597
DEFA23	-6.593322
DEFA25	-4.7372274
DEFA26	-3.7763834
DEFA3	-3.9146202
DEFA-PS1	-5.3058405
DEFA-PS13	-0.7176564
DEFA-RS1	-3.376767
DEFA-RS12	-2.3447108
DEFA-RS2	-1.286385
DEFA-RS4	-1.8513799
DEFA-RS7	-2.4696283
DEFB11	-1.7501578
DEFB13	-4.811466
DEFB14	-3.4896278
DEFB18	-5.509198
DEFB19	-2.5036385
DEFB2	-3.800849
DEFB20	-6.583228
DEFB21	-2.482564
DEFB22	-4.766227
DEFB23	-1.5623097
DEFB25	-1.2355678
DEFB26	-3.2157238
DEFB28	-3.530088
DEFB29	-2.4707623
DEFB30	-2.9222655
DEFB35	-2.0456333
DEFB36	-4.5654902
DEFB38	-3.024488
DEFB4	-3.0057049
DEFB40	-3.602761
DEFB41	-3.3478646
DEFB45	-1.0430889
DEFB46	-3.0976634
DEFB47	-3.2665071
DEFB48	-3.2375903

DEFB6	-4.3741026
DEFB7	-5.276052
DEFB9	-3.1225922
DENND2A	-1.9629729
DENND2C	-3.1860557
DENND3	-0.7209411
DEPDC1A	-2.0635705
DEPDC1B	-1.1428235
DEPDC5	-1.0640423
DES	-2.8939335
DFNB59	-2.6131687
DGKA	-1.320718
DGKB	-2.9696636
DGKEOS	-0.7784343
DGKG	-3.644493
DGKH	-0.2959731
DGKI	-2.024485
DGKK	-6.0507555
DHFR	-1.2950845
DHH	-1.8011119
DHRS1	-1.2745817
DHRS2	-2.6557643
DHRS3	-0.8356831
DHRS7C	-6.5395784
DHRS9	-1.384887
DHX37	-0.4897895
DIAP1	-0.9758995
DIAP3	-2.0169995
DIO3	-1.4572265
DIO3OS	-1.833565
DIP2A	-0.7396286
DIRAS2	-4.854745
DISC1	-0.592597
DISP1	-0.701669
DIXDC1	-2.5727751
DKK3	-1.6650102
DKK4	-5.279909
DKKL1	-1.9741118
DLC1	-2.240665
DLEC1	-1.392956
DLEU2	-0.3868232
DLG4	-1.8762732
DLGAP3	-1.9756951
DLK1	-2.756823
DLL1	-1.3216746

DLL4	-3.0657403
DLX5	-5.0300913
DLX6	-5.78777
DLX6AS2	-2.1571703
DLX6OS1	-3.5411203
DMC1	-3.1945295
DMD	-1.1430434
DMKN	-3.0391428
DMRT1	-1.9031949
DMRT3	-5.1991143
DMRTB1	-3.4566789
DMRTC1B	-2.4328022
DMRTC1C2	-3.394575
DNAAF1	-3.4694595
DNAAF3	-0.7852442
DNAH1	-2.6018422
DNAH10	-3.675891
DNAH12	-4.3677998
DNAH14	-1.9536841
DNAH17	-3.6862822
DNAH5	-4.355846
DNAH6	-1.9069233
DNAH7A	-2.1724925
DNAH7B	-3.8921585
DNAH8	-2.4119737
DNAH9	-2.8207185
DNAIC1	-0.5988286
DNAIC2	-3.9229784
DNAJA4	-0.6045439
DNAJB12	-0.4474351
DNAJB13	-2.0485973
DNAJB4	-0.5342662
DNAJB8	-3.5966997
DNAJC4	-0.2649052
DNAJC5B	-2.593317
DNALI1	-6.359765
DNASE1	-4.7516813
DND1	-1.866106
DNM1	-1.9385862
DNM3	-2.42999
DNMBP	-0.8567946
DNMT3A	-0.6078382
DNMT3AOS	-2.7058454
DNMT3B	-0.3485603
DNMT3BOS	-1.035147

DOC2A	-5.9479537
DOCK2	-1.2829967
DOCK3	-0.8427775
DOCK6	-0.9423101
DOCK7	-3.0608177
DOCK8	-1.5624304
DOCK9	-2.5551977
DOK1	-0.9732621
DOK4	-2.870534
DOK6	-3.0753055
DOK7	-3.8469725
DOPEY1	-0.3786798
DOXL2	-4.082863
DPEP1	-5.489747
DPF1	-2.641797
DPF3	-1.172154
DPPA3	-3.9198194
DPPA4	-1.8928471
DPT	-4.507796
DPYS	-0.6097882
DPYSL2	-3.120058
DPYSL3	-0.8871696
DPYSL4	-2.1425514
DRAM1	-1.3975537
DRAXIN	-5.469443
DRC1	-4.184264
DRD2	-3.7310872
DRD3	-3.9263115
DRP2	-3.6622539
DSC1	-1.0124221
DSC3	-2.446578
DSCAM	-3.9312024
DSEL	-0.8150744
DSG1C	-3.4257452
DSG3	-2.4342294
DSG4	-3.39206
DSPP	-5.4506783
DTL	-1.2251363
DTNA	-2.428051
DTWD2	-0.8619955
DTX4	-0.5423405
DUOX1	-2.2709324
DUPD1	-5.776309
DUSP13	-3.7874744
DUSP14	-1.6997592

DUSP15	-3.1576037
DUSP18	-9.114308
DUSP19	-1.0518999
DUSP26	-3.0031874
DUSP7	-1.4086201
DUSP9	-2.249776
DUT	-0.6447198
DUX	-1.2170575
DUXBL1	-2.9739904
DUXBL2	-2.4421852
DXBAY18	-3.818662
DYDC1	-2.9474561
DYDC2	-3.72716
DYNAP	-3.2041144
DYNC2H1	-0.4271381
DYNC2LI1	-2.018942
DYNLL1	-3.2982268
DYNLRB2	-7.8330564
DYSF	-0.33757
DYTN	-4.019317
DYX1C1	-1.0491483
DZANK1	-2.7924423
DZIP1L	-4.167182
E2F2	-2.2383602
E2F4	-1.1066837
EAR1	-4.824317
EAR10	-4.0741463
EAR12	-5.0691233
EAR2	-4.241288
EAR4	-7.255369
EAR6	-4.5396485
EAR7	-2.5031228
ECE1	-0.5679805
ECHDC1	-1.817533
ECM2	-4.6916323
EDAR	-3.3200204
EDARADD	-1.7903593
EDDM3B	-3.6377418
EDN2	-4.4033823
EDN3	-2.5219116
EEF2K	-2.4985273
EFCAB1	-2.7656953
EFCAB10	-4.4756885
EFCAB11	-3.1045964
EFCAB12	-1.4162152

EFCAB3	-3.5234528
EFCAB4A	-2.6715307
EFCAB4B	-1.6648123
EFCAB5	-3.016764
EFCAB7	-2.4108725
EFCAB9	-7.20887
EFCC1	-4.044627
EFEMP1	-2.392605
EFEMP2	-0.6310432
EFHB	-2.3847392
EFHC1	-2.9061487
EFHD1	-3.0431404
EFNA2	-3.0114202
EFNA3	-2.5388901
EFNA4	-1.2307184
EFNB1	-2.0500915
EFNB2	-3.0329113
EFNB3	-1.3139191
EFR3B	-2.3515685
EFS	-1.1041114
EGFBP2	-1.0777946
EGFEM1	-1.128387
EGFL6	-4.2843313
EGFL7	-2.9978244
EGFLAM	-2.6243021
EGLN1	-2.204207
EHD2	-4.173874
EHD4	-1.5938623
EHF	-2.504929
ELAVL3	-4.441444
ELF4	-1.9945097
ELF5	-6.5305986
ELFN2	-1.6168106
ELK3	-2.8957162
ELL3	-1.9372739
ELMO1	-5.1887426
ELMO2	-1.5036771
ELN	-6.851184
ELTD1	-2.9766495
EMB	-2.4547133
EMC10	-2.0704184
EMCN	-2.4812
EMID1	-4.737626
EML1	-2.4678063
EMP1	-1.9527953

EMP2	-3.7679908
EMP3	-1.4520199
EMX1	-3.897288
EMX2OS	-2.8216665
EN2	-0.4138056
ENAH	-2.329251
ENAM	-2.7451017
ENDOD1	-2.9455223
ENDOU	-3.902754
ENG	-0.7589486
ENKD1	-1.7416139
ENKUR	-0.7551668
ENO4	-3.8665578
ENOX1	-2.632756
ENPP2	-0.7546904
ENPP6	-3.6356573
ENTHD1	-3.0526063
ENTPD3	-2.9920645
ENTPD7	-2.0582528
EOMES	-3.2007565
EPB4.1L1	-2.1184902
EPB4.1L2	-1.8773658
EPB4.1L4A	-1.703758
EPB4.2	-2.389994
EPCAM	-3.8847806
EPDR1	-2.419247
EPG5	-0.605149
EPGN	-1.6190934
EPHA10	-3.9404478
EPHA3	-2.346559
EPHA4	-3.787807
EPHA7	-1.8323989
EPHB6	-1.6391101
EPHX3	-1.9707742
EPHX4	-2.6833153
EPO	-4.1118627
EPOR	-2.8693275
EPPIN	-3.9996328
EPS8	-1.6907544
EPS8L1	-3.2042499
EPS8L2	-3.727868
EPSTI1	-3.7910259
EPX	-3.172696
ERAS	-4.628866
ERBB2	-3.017569

ERC1	-1.1769793
ERC2	-0.5902469
ERG	-2.6425843
ERICH3	-2.5298944
ERICH6	-1.8233421
ERMAP	-1.0640597
ERN2	-2.2648277
ERV3	-4.2963057
ESAM	-0.7831013
ESM1	-0.8724473
ESP6	-3.9560585
ESPL1	-2.2620206
ESPNL	-0.9278302
ESRP1	-2.4649575
ESRRB	-1.0685937
ESRRG	-2.7556498
ESYT3	-4.1142426
ETL4	-1.3925326
ETOHI1	-2.137411
ETV1	-2.9029315
ETV2	-5.5424967
ETV3L	-3.104709
ETV5	-2.473707
EVA1C	-0.2900279
EVC	-5.033366
EVPL	-4.2498603
EXO1	-0.7298486
EXOC3L	-1.1109974
EXOC3L2	-1.3514287
EXOC4	-0.5524006
EXOG	-2.6049085
EXT2	-1.3271585
EYA2	-7.5387735
EYA4	-2.1736298
EZR	-0.6771557
F2R	-0.5938857
F2RL2	-1.4971089
F3	-2.2393382
F8A	-2.5056634
FABP12	-1.2163548
FABP3	-4.383831
FABP6	-5.408747
FABP9	-5.8825316
FADDOS	-2.62176
FADS3	-2.2458858

FADS6	-1.3929014
FAIM2	-5.1728964
FAIM3	-2.6672297
FAM101A	-4.0130377
FAM101B	-3.5533485
FAM105A	-2.3649397
FAM109B	-1.9237916
FAM110A	-1.3808868
FAM110B	-1.8328927
FAM110C	-1.0293217
FAM111A	-0.5436556
FAM115C	-1.9518518
FAM117A	-1.9432933
FAM122B	-1.3189046
FAM124B	-1.0950782
FAM126A	-0.4400413
FAM129A	-0.9504032
FAM129B	-2.0472858
FAM131A	-2.1426697
FAM131B	-3.912162
FAM131C	-2.7421432
FAM132B	-2.8158288
FAM13A	-1.9696865
FAM150A	-2.8370142
FAM151A	-2.4024384
FAM154A	-2.9635692
FAM154B	-3.7982066
FAM155A	-2.649304
FAM159A	-3.5297604
FAM160A1	-2.4211338
FAM161A	-2.4053645
FAM161B	-1.9613893
FAM162B	-6.3923817
FAM163B	-4.079216
FAM166B	-5.9802375
FAM170A	-3.179683
FAM170B	-3.6680052
FAM171A1	-1.7405686
FAM171A2	-2.915696
FAM171B	-2.6751337
FAM174B	-3.5116477
FAM178B	-0.860379
FAM179A	-4.411509
FAM180A	-1.7601409
FAM181A	-4.2301164

FAM181B	-5.35174
FAM183B	-7.7101564
FAM184A	-1.779237
FAM188B2	-4.303918
FAM189A2	-4.4859686
FAM189B	-1.3460093
FAM198A	-3.762583
FAM198B	-4.1358204
FAM19A1	-2.3598065
FAM19A5	-2.8389287
FAM212A	-2.0573401
FAM212B	-2.1473107
FAM216B	-4.583846
FAM217A	-2.377336
FAM217B	-2.473634
FAM219AOS	-4.944992
FAM219B	-0.805794
FAM221A	-2.5816598
FAM221B	-2.9740956
FAM227A	-5.2774925
FAM227B	-2.6343727
FAM228B	-2.9896305
FAM229B	-1.436404
FAM24A	-2.5522804
FAM26E	-1.4298146
FAM3B	-3.2537632
FAM46B	-3.4808977
FAM46C	-0.8850539
FAM47E	-3.0290284
FAM50B	-2.786138
FAM57A	-2.8854148
FAM57B	-1.771445
FAM60A	-2.1089296
FAM64A	-3.169325
FAM65A	-0.4663637
FAM65B	-1.3102741
FAM65C	-1.4816076
FAM69A	-0.4594235
FAM69B	-3.2868114
FAM71A	-3.1321828
FAM71B	-3.0518985
FAM71E2	-3.2694333
FAM71F1	-3.2910254
FAM81B	-3.8296733
FAM83A	-1.3032482

FAM83B	-1.102673
FAM83C	-3.6173747
FAM83E	-3.2239845
FAM84A	-1.4425724
FAM89A	-1.3067148
FAM92B	-4.712693
FAN1	-1.071967
FANCD2	-2.5622838
FANCG	-2.909738
FANCI	-4.926924
FANK1	-3.618649
FAR1	-2.6182775
FASN	-1.1404905
FAT2	-1.0520022
FAT3	-3.0811162
FAT4	-2.8927143
FBF1	-2.2917376
FBLL1	-7.3436437
FBLN1	-3.7193694
FBLN2	-2.430216
FBLN5	-5.410521
FBLN7	-4.0321403
FBN1	-1.7109728
FBN2	-3.5298543
FBP2	-2.7993422
FBXL16	-2.5736494
FBXL2	-1.2293391
FBXL7	-3.4608288
FBXO10	-1.4950025
FBXO15	-0.730571
FBXO16	-2.4585807
FBXO17	-1.1525376
FBXO2	-0.4845226
FBXO24	-2.5107427
FBXO32	-0.6075246
FBXO36	-0.9333196
FBXO39	-3.8761544
FBXO41	-4.6418643
FBXO43	-4.6904564
FBXO44	-3.0469117
FBXO47	-3.8756766
FBXO48	-2.9521728
FBXO5	-1.5506105
FBXW10	-6.1557717
FBXW13	-2.3894284

FBXW19	-3.3877769
FBXW20	-1.3788695
FBXW21	-2.046033
FBXW26	-3.7937317
FBXW27	-2.2853687
FCAMR	-2.7471154
FCER2A	-2.4188256
FCGBP	-3.3775914
FCHO1	-1.0460908
FCNB	-1.5144358
FCOR	-1.6574318
FCRL6	-2.9359617
FCRLB	-3.6005328
FCRLS	-1.2026606
FENDRR	-3.8947148
FER1L4	-3.2765799
FER1L6	-2.4418166
FERD3L	-5.993969
FEV	-2.7286649
FFAR2	-3.7976198
FFAR4	-0.6277599
FGD1	-2.6717937
FGD2	-1.181207
FGD5	-2.4085612
FGD6	-0.6717901
FGF1	-2.783285
FGF10	-4.0625424
FGF11	-3.5031118
FGF12	-2.5916967
FGF13	-2.1517038
FGF14	-2.9685395
FGF17	-1.8724804
FGF18	-4.630459
FGF2	-2.3183157
FGF20	-3.739254
FGF22	-0.9219499
FGF4	-4.9097304
FGF6	-0.9839327
FGF7	-4.5214386
FGF8	-2.121355
FGF9	-3.6037307
FGFBP1	-5.1660285
FGFBP3	-1.5332444
FGFR1	-1.2691493
FGFR3	-1.9833579

FHDC1	-3.864531
FHIT	-3.471372
FHL1	-4.516389
FHL2	-2.5976443
FHL5	-2.4241405
FHOD1	-1.2403395
FHOD3	-3.0985305
FIBIN	-5.0943747
FIGF	-5.2957835
FIGLA	-3.6546812
FIGNL1	-0.7907617
FILIP1	-0.7529786
FKBP10	-0.7670224
FKBP11	-2.7104545
FKBP15	-0.4885738
FKBP1B	-0.8507667
FKBP4	-0.9649434
FKBP7	-2.3340595
FLG	-2.8996325
FLI1	-0.7727993
FLNC	-3.1418438
FLRT2	-3.3402574
FLRT3	-6.56553
FLT1	-2.174146
FLT3	-2.3617172
FLT3L	-0.8759608
FLT4	-0.290139
FLYWCH2	-1.8872619
FMN1	-2.1845305
FMN2	-2.0501585
FMNL1	-5.9002624
FMNL3	-2.4869642
FMO2	-5.479324
FMO3	-1.5322704
FMOD	-2.788177
FN3K	-2.1441958
FND3C2	-3.0580103
FNDC1	-3.8628314
FNDC3C1	-6.1734867
FNDC5	-4.4373302
FNDC7	-2.1459727
FNDC9	-2.172525
FOLR1	-2.3533745
FOLR4	-1.5286787
FOSB	-3.542759

FOXB1	-3.146861
FOXB2	-5.4949546
FOXC1	-1.777497
FOXD2	-2.4228857
FOXD2OS	-1.9055047
FOXD4	-3.6428776
FOXE1	-4.933557
FOXF1	-3.2307088
FOXF2	-5.7530384
FOXH1	-3.470099
FOXI1	-2.4001853
FOXI2	-3.8550704
FOXI3	-3.1005034
FOXJ1	-5.621475
FOXJ2	-3.33028
FOXL1	-2.4774787
FOXL2OS	-2.6743379
FOXM1	-1.92434
FOXN4	-3.6167538
FOXO3	-1.8600547
FOXO4	-3.5732632
FOXO6	-3.8154683
FOXP2	-1.6894441
FOXP3	-1.9950829
FOXR1	-2.9256606
FOXS1	-0.613271
FRAS1	-4.215372
FRAT2	-1.149164
FREM1	-4.4859896
FREM2	-1.0356977
FREM3	-2.3299718
FRMD3	-1.9839773
FRMD4A	-1.5838888
FRMD5	-2.0125875
FRMD6	-3.1563787
FRMD7	-2.683954
FRMPD1	-3.6617167
FRRS1L	-3.3196654
FRS3OS	-3.8195977
FRY	-0.9758611
FSCN3	-2.4098296
FSD1	-2.4419024
FSD2	-9.474304
FSHB	-3.905808
FSHR	-3.4429352

FSIP1	-2.7830276
FSIP2	-3.2910964
FSTL1	-0.9185889
FSTL3	-3.9375694
FTMT	-2.4620752
FTSJ2	-0.36394
FUT10	-0.4267533
FUT4	-2.664606
FUT7	-3.0608127
FUT8	-0.4846115
FUT9	-1.5529113
FUZ	-0.3145266
FV1	-3.3958385
FXYD2	-3.632992
FXYD3	-3.674202
FXYD4	-3.5847454
FXYD6	-4.5777793
FXYD7	-5.4744287
FZD2	-4.8271914
FZD3	-1.3469651
FZD5	-3.8524525
FZD6	-0.6975083
FZD7	-1.0636313
G0S2	-1.0623562
G2E3	-1.0448651
G6B	-3.940211
G6BOS	-1.7228687
G6PC2	-4.8451595
G6PD2	-1.1283674
GAB2	-4.292278
GABBR1	-3.5762537
GABRA3	-1.2222004
GABRB1	-3.6716733
GABRB2	-2.9977736
GABRD	-2.08757
GABRG1	-6.5716276
GABRG2	-2.3091545
GABRG3	-2.607646
GABRP	-3.2923117
GABRR3	-2.7593582
GAD1	-3.0409522
GAD1OS	-4.476002
GAD2	-4.5646152
GAL	-1.0720913
GAL3ST1	-1.565392

GAL3ST2	-2.409774
GALNT12	-2.4111612
GALNT13	-2.959567
GALNT14	-3.3899581
GALNT15	-3.171592
GALNT16	-2.8177896
GALNT18	-2.89212
GALNT3	-1.18644
GALNT5	-5.306819
GALP	-3.2047508
GALR3	-5.2449646
GALT	-0.9107521
GAP43	-6.0826836
GAPDHS	-1.7841005
GAREML	-4.0515985
GARNL3	-3.8738618
GAS2L2	-5.050393
GAS7	-1.6263623
GAST	-2.642717
GATA1	-3.2225642
GATA2	-3.7089612
GATA3	-3.1058743
GATSL2	-1.6569433
GATSL3	-1.635946
GBF1	-0.8316929
GBGT1	-3.7883527
GBX1	-5.415117
GBX2	-2.8346376
GCAP8	-3.0578551
GCG	-2.5883338
GCK	-2.4853985
GCNT1	-2.9230325
GCNT3	-6.2464542
GCNT4	-2.0320632
GDAP5	-3.003724
GDF1	-4.1169815
GDF11	-2.9640307
GDF3	-0.9414921
GDF5	-5.4483347
GDF7	-4.150171
GDF9	-1.1189928
GDNF	-4.21163
GDPD1	-0.6648676
GDPD2	-4.7409887
GDPD5	-2.2072256

GEN1	-2.3282533
GFAP	-3.125214
GFI1	-1.5205305
GFI1B	-1.7421148
GFRA2	-1.0755618
GFRA3	-3.1569266
GFRAL	-2.971679
GFY	-3.7451665
GGN	-2.4278624
GGT1	-8.489059
GGT5	-0.8084003
GGTA1	-1.6718531
GH	-2.5905104
GHRH	-0.9298937
GHRL	-0.8749509
GIMAP1	-0.4810507
GIMAP1OS	-2.3232746
GIMAP4	-0.454982
GIMAP8	-0.5274465
GINS2	-0.5907393
GIP	-5.8261757
GIPC2	-2.217237
GIPC3	-4.167987
GIPR	-4.9671407
GJA3	-4.1698914
GJA5	-5.1247215
GJA8	-3.7116914
GJB3	-5.072995
GJB6	-3.7863123
GJC1	-2.2472124
GJD2	-3.5820813
GK2	-6.0386534
GLB1L2	-4.6953163
GLCCI1	-0.6077869
GLDN	-1.5631423
GLDNOS	-4.0077662
GLI1	-5.475675
GLI3	-0.5778017
GLIPR1	-1.7035778
GLIPR1L3	-1.1102836
GLIS2	-2.4591436
GLIS3	-1.9268289
GLP1R	-3.5700302
GLRA4	-6.1033545
GLRB	-3.4755023

GLT28D2	-1.038974
GLT6D1	-1.8712893
GLTP	-1.2883487
GLTSCR1	-2.2008615
GLTSCR1L	-0.4367285
GLYATL3	-4.4998646
GLYCAM1	-4.8599806
GM10021	-0.6024122
GM10024	-3.3307164
GM10030	-1.8125045
GM10033	-0.8390555
GM10034	-1.1056743
GM10044	-2.653232
GM10069	-4.8849974
GM10071	-2.8495655
GM10081	-2.2213085
GM101	-1.5591266
GM10113	-3.21487
GM10144	-0.8703485
GM10158	-2.4314535
GM10199	-4.9119263
GM10209	-2.5096269
GM10220	-4.69587
GM10229	-2.9007986
GM10252	-1.3926611
GM10276	-1.5354776
GM10278	-4.2024775
GM10286	-3.8707337
GM10287	-2.911983
GM10319	-2.313335
GM10334	-1.6513622
GM10336	-2.0550973
GM10354	-2.1029966
GM10369	-2.9041238
GM10370	-5.723751
GM10371	-2.460465
GM10372	-4.4270477
GM10373	-2.989299
GM10375	-4.3120813
GM10385	-1.6799395
GM10389	-2.2259347
GM10403	-4.1281204
GM10408	-2.4333637
GM10415	-3.6491964
GM10419	-2.892574

GM10428	-1.084218
GM1043	-2.949042
GM10431	-5.8911133
GM10432	-3.8688571
GM10436	-2.2020974
GM10437	-2.4412465
GM10440	-3.396999
GM10450	-3.6621106
GM10457	-0.9484863
GM10465	-4.2389145
GM10469	-3.4805236
GM10471	-2.8726938
GM10474	-1.5764298
GM10475	-2.796918
GM10482	-2.9060562
GM10489	-3.1217093
GM10498	-4.448678
GM10501	-4.796458
GM10503	-5.4194403
GM10510	-2.0233035
GM10512	-2.8880706
GM10513	-3.1271832
GM10516	-4.1431756
GM10531	-4.4459877
GM10532	-2.044706
GM10536	-6.980798
GM10537	-2.9868774
GM10538	-3.8969507
GM10548	-4.4576
GM10560	-2.8864594
GM10572	-3.3464253
GM10579	-3.319927
GM10584	-4.108696
GM10588	-2.6480932
GM10603	-1.6068459
GM10609	-1.2695282
GM10615	-0.5443239
GM10623	-2.4121733
GM10624	-3.0413325
GM10638	-1.7288284
GM10651	-1.4277971
GM10673	-1.066951
GM10674	-4.272662
GM10682	-1.69327
GM10683	-2.358812

GM10684	-4.0965037
GM10687	-4.2546487
GM10688	-3.8586118
GM10697	-0.6689782
GM10701	-2.4381192
GM10714	-3.5969653
GM10727	-2.4528756
GM10732	-3.3626416
GM10744	-3.5324445
GM10747	-4.600769
GM10752	-3.9667132
GM10755	-2.4432738
GM10757	-2.2151623
GM10759	-2.5111895
GM10777	-1.1106865
GM10779	-3.4117157
GM10781	-3.615168
GM10787	-2.7649822
GM10789	-2.3353288
GM10790	-2.3945794
GM10802	-4.8725085
GM10804	-3.1522455
GM10814	-3.3004143
GM10815	-3.196007
GM10817	-3.8728185
GM10830	-2.234074
GM10835	-3.8089879
GM10836	-3.1657178
GM10844	-3.513092
GM10857	-3.9761252
GM10860	-1.549654
GM10864	-2.3842542
GM10865	-1.8578892
GM10866	-2.9078197
GM10867	-4.6469707
GM10871	-1.71645
GM10926	-4.174084
GM10933	-1.931006
GM1096	-3.2101135
GM11110	-1.2294397
GM11123	-0.4206195
GM11149	-2.4741778
GM11223	-2.4928257
GM1123	-2.0994816
GM11266	-1.5013676

GM11346	-3.1721365
GM11381	-2.2262108
GM11468	-3.5120325
GM11517	-2.0480418
GM11529	-2.2657647
GM11541	-4.9903345
GM11543	-3.7158802
GM11545	-2.1151094
GM11595	-1.2476659
GM11627	-4.1018567
GM11634	-2.2549133
GM11691	-5.1252084
GM11710	-1.5654583
GM11735	-5.2635727
GM11744	-2.9498074
GM11747	-2.8574696
GM11938	-3.389773
GM11961	-1.3518817
GM11981	-4.1290846
GM11992	-3.7878442
GM12000	-2.1502643
GM12059	-3.997111
GM12128	-3.7007623
GM12185	-3.4792957
GM12258	-3.3013859
GM12298	-1.8645389
GM12349	-1.9736893
GM12359	-1.2487161
GM12371	-2.73981
GM12408	-3.4386268
GM12409	-3.785082
GM12472	-4.3437014
GM12542	-3.8057308
GM12568	-2.799428
GM12580	-3.5292597
GM12592	-1.8257062
GM12633	-3.4605024
GM12666	-1.0544553
GM12695	-10.161966
GM12703	-1.9619102
GM12789	-3.2660363
GM12790	-3.7422166
GM12794	-3.0333612
GM12863	-3.4286656
GM12866	-1.1828289

GM12888	-3.7368748
GM12992	-3.1273034
GM13011	-3.435105
GM13032	-4.8800545
GM13051	-3.0517066
GM13083	-3.0765765
GM13088	-2.6592774
GM13111	-2.9512868
GM13112	-3.0916657
GM13119	-3.501527
GM13124	-3.595435
GM13125	-2.229701
GM13128	-2.914737
GM13152	-2.8359222
GM13154	-1.5370703
GM13205	-4.53588
GM13212	-1.1393774
GM13219	-2.4958847
GM1322	-4.648609
GM13247	-1.3821707
GM13275	-1.6107945
GM13304	-3.0423167
GM13372	-4.1227984
GM13403	-4.447606
GM13429	-2.1040635
GM13446	-3.885608
GM13580	-3.1569858
GM13710	-2.384635
GM13769	-2.9092271
GM13807	-5.3620996
GM13861	-3.5031395
GM14025	-1.7960389
GM14085	-2.6214569
GM14092	-3.6911237
GM14124	-1.0881897
GM14129	-3.4815059
GM14151	-0.5539386
GM14164	-2.882161
GM14207	-2.9305189
GM14243	-5.709184
GM14317	-3.2519593
GM14340	-1.9298857
GM14461	-3.4696112
GM14483	-3.6533184
GM14484	-2.273113

GM14486	-3.5959628
GM14488	-3.07095
GM14496	-2.587119
GM14499	-2.9885046
GM14634	-2.5178683
GM14725	-2.3120217
GM14743	-0.8937054
GM14762	-1.0645962
GM14851	-7.5103045
GM14920	-3.9316125
GM14964	-2.3420372
GM15023	-4.5889025
GM15104	-4.286994
GM15217	-1.4971986
GM1527	-2.8960478
GM15292	-3.263051
GM15299	-5.006748
GM15319	-1.3237128
GM15401	-1.9966917
GM15412	-3.6712325
GM15413	-3.7633102
GM15417	-1.4289901
GM15441	-2.1940372
GM15478	-4.273676
GM15498	-7.7448635
GM15514	-5.285698
GM156	-1.8664005
GM15612	-3.4649925
GM15628	-2.6662765
GM1564	-1.2734475
GM15762	-3.3564563
GM1604B	-2.2152026
GM16287	-0.3815651
GM1631	-3.1134398
GM16428	-4.003502
GM16441	-1.5751565
GM16462	-5.527996
GM16486	-2.2327132
GM16488	-3.983533
GM16489	-2.2765832
GM16491	-2.064983
GM16498	-3.9135714
GM16505	-1.1341269
GM16508	-2.367619
GM16527	-2.9353433

GM16532	-4.271863
GM1661	-1.8029423
GM16686	-2.533391
GM1673	-1.6482186
GM16863	-3.359451
GM16982	-2.303564
GM17019	-2.2015262
GM1715	-0.5865507
GM17244	-1.5148637
GM17767	-3.0225434
GM17769	-2.5734901
GM17783	-3.905599
GM19410	-3.4761147
GM1965	-3.3674862
GM19668	-0.4008267
GM1968	-2.527574
GM1976	-0.7071934
GM1987	-4.531559
GM1993	-3.8168864
GM2002	-1.2137413
GM2027	-3.2845907
GM2042	-2.6266568
GM20554	-0.6191187
GM20556	-2.4553344
GM20558	-5.8208065
GM20604	-2.8205278
GM20611	-1.9751549
GM20740	-3.417653
GM20747	-3.6908698
GM20754	-1.9622147
GM20767	-4.3880224
GM20806	-4.0655518
GM20837	-4.628445
GM20865	-1.9158437
GM20877	-3.1665678
GM20878	-0.6776826
GM2090	-3.8684616
GM20939	-0.4273729
GM21276	-1.5741479
GM21292	-1.9084139
GM21464	-5.3918877
GM2155	-2.12426
GM21637	-3.0142815
GM21951	-3.0027394
GM2233	-1.7989528

GM2240	-5.486637
GM2483	-2.1293097
GM2491	-4.4911203
GM2511	-2.1073956
GM2582	-0.3101711
GM266	-1.605382
GM2670	-4.722543
GM2716	-1.7470901
GM2800	-4.803954
GM2824	-0.8541322
GM2927	-3.6674986
GM2990	-4.250985
GM2A	-0.8842492
GM3002	-4.9862175
GM3045	-2.2617755
GM3052	-1.9241676
GM3065	-2.3976903
GM3081	-2.5014548
GM3086	-2.81256
GM3134	-0.6858087
GM3146	-1.8566563
GM318	-2.2071438
GM3227	-3.4196417
GM3230	-4.8409204
GM3238	-3.565649
GM3264	-2.490316
GM3300	-1.0262804
GM3317	-2.68371
GM3325	-1.1597776
GM3336	-5.6462045
GM3364	-2.638255
GM3428	-2.96005
GM3509	-2.5571496
GM3510	-3.0918221
GM355	-4.516876
GM3561	-2.1530218
GM3596	-4.116055
GM3598	-4.04162
GM362	-3.3690631
GM3625	-2.1214461
GM364	-2.8535042
GM3646	-3.634571
GM3669	-3.2641563
GM3704	-2.650382
GM3718	-2.0792418

GM3740	-2.8519082
GM3763	-3.98158
GM3775	-2.4646015
GM3807	-1.5867422
GM382	-4.210374
GM3822	-4.9038324
GM3831	-3.3653877
GM3858	-1.8830001
GM3871	-2.0786648
GM3881	-2.0254817
GM3916	-1.6126866
GM3942	-0.9506135
GM3952	-2.2901769
GM3961	-3.634747
GM3985	-4.209523
GM4013	-1.6654303
GM4022	-2.2838397
GM4081	-1.8070402
GM4098	-1.130209
GM4107	-1.3029237
GM4120	-0.3754289
GM4131	-4.105954
GM4132	-2.602158
GM4151	-3.2309153
GM4200	-1.6853743
GM4201	-4.5747166
GM4203	-5.9122686
GM4211	-4.6616645
GM4221	-1.711487
GM4224	-3.7872186
GM4241	-1.5764952
GM4278	-3.7180526
GM4279	-2.4379785
GM4340	-0.7014518
GM4354	-2.1717632
GM436	-1.9209604
GM4371	-3.0186987
GM438	-4.0706663
GM44	-1.9151459
GM4419	-4.53364
GM4430	-0.3352282
GM4432	-3.328448
GM4454	-3.3312228
GM4461	-1.831038
GM4489	-6.132696

GM4532	-1.6055841
GM4544	-1.1813519
GM4567	-3.8448267
GM4593	-1.1048527
GM4598	-5.4648995
GM4610	-2.5472934
GM4633	-2.713167
GM4657	-4.29566
GM4665	-3.3679767
GM4745	-5.50861
GM4755	-5.689489
GM4758	-0.9992275
GM4759	-1.7862687
GM4763	-2.6008267
GM4776	-3.6513448
GM4791	-2.8639216
GM4792	-3.9964185
GM4794	-1.6550071
GM4850	-3.0906796
GM4868	-6.1285477
GM4872	-5.316007
GM4881	-0.5738812
GM4906	-0.6818409
GM4922	-3.2357316
GM4956	-2.7468665
GM4975	-4.5157146
GM4980	-2.5735695
GM4994	-4.6588926
GM5065	-2.1530876
GM5083	-3.0206795
GM5084	-3.8404691
GM5089	-0.6904252
GM5092	-0.5232873
GM5094	-2.3847435
GM5095	-2.1566725
GM5099	-2.4825633
GM5105	-1.5597117
GM5108	-3.847272
GM5113	-1.7105064
GM5118	-2.4486992
GM5122	-3.6772432
GM5124	-5.4262476
GM5127	-4.857584
GM5129	-2.9543736
GM5132	-0.8973568

GM5134	-6.015809
GM5141	-1.3796308
GM5144	-4.500226
GM5148	-3.141596
GM5155	-3.0082045
GM5174	-2.7154753
GM5177	-3.0273035
GM5215	-1.2801173
GM525	-3.090662
GM5258	-2.817793
GM527	-0.6898036
GM5294	-3.2681344
GM5296	-1.5319378
GM53	-4.706484
GM5331	-3.0976658
GM5334	-3.2129095
GM536	-2.7763128
GM5415	-3.2263463
GM5416	-0.7880132
GM5434	-2.5937865
GM5441	-1.493742
GM5447	-2.9611034
GM5458	-1.6871194
GM5468	-4.146059
GM5476	-3.939689
GM5477	-3.3145356
GM5478	-1.1049101
GM5485	-3.3114593
GM5486	-0.5869522
GM5493	-3.8537529
GM553	-4.1790833
GM5532	-5.3331275
GM5535	-3.7982533
GM5547	-3.1415656
GM5553	-1.4248312
GM5563	-5.429826
GM5565	-2.912587
GM5570	-2.3689973
GM5589	-2.4374847
GM5590	-4.2869997
GM5591	-3.483943
GM5593	-0.6529822
GM5606	-5.5557575
GM5607	-2.4203618
GM5608	-3.108725

GM5622	-2.8452783
GM5627	-4.399283
GM5635	-2.692063
GM5636	-2.733579
GM5640	-3.3575876
GM5665	-1.7251321
GM568	-4.635144
GM572	-4.045461
GM5751	-1.495352
GM5796	-2.025329
GM5797	-1.3160324
GM5800	-4.2473435
GM5820	-2.6605844
GM5833	-0.3828714
GM5849	-1.1497469
GM5860	-4.920721
GM5862	-2.605936
GM5868	-4.118329
GM5893	-2.219068
GM590	-4.4233713
GM5901	-2.0592568
GM5916	-2.958661
GM5925	-2.5915413
GM5936	-3.1628938
GM5938	-2.8464274
GM595	-3.700124
GM5953	-3.6116576
GM5958	-2.8817215
GM597	-0.7892585
GM6042	-3.3584676
GM609	-3.8940516
GM6093	-1.907903
GM6116	-2.017065
GM6117	-5.727023
GM614	-3.198042
GM6145	-3.0374422
GM6219	-3.8476
GM6225	-2.279861
GM6260	-2.3801186
GM6268	-2.381391
GM6277	-2.434768
GM6287	-3.3049312
GM6289	-3.2282565
GM6307	-4.0510874
GM6309	-2.276451

GM6329	-2.297769
GM6360	-2.5082436
GM6370	-2.3348145
GM6416	-2.5060182
GM6460	-1.9527202
GM6484	-1.6484549
GM6537	-2.9542215
GM6556	-3.3091347
GM6566	-4.995244
GM6567	-1.9461637
GM6590	-3.173317
GM6614	-6.09486
GM6639	-3.796514
GM6654	-1.0840125
GM6696	-1.1586988
GM6713	-4.2555356
GM6729	-1.6285446
GM6756	-0.2788546
GM6760	-4.6931095
GM6787	-3.9173489
GM6818	-5.4409733
GM684	-2.6857157
GM6846	-3.194965
GM6880	-1.1995182
GM6936	-2.0233767
GM694	-4.167659
GM6943	-4.1327043
GM6958	-2.3614974
GM7056	-2.0500724
GM7068	-1.249085
GM7104	-4.2315416
GM7133	-2.7960317
GM7134	-3.4245963
GM715	-2.7737713
GM7167	-8.299361
GM7168	-1.832066
GM7244	-1.5017853
GM7257	-4.1730585
GM7271	-2.0348866
GM7276	-2.787005
GM7285	-1.863095
GM732	-4.5746155
GM7420	-1.7965899
GM7444	-3.2616193
GM7457	-2.5354047

GM7467	-3.2624063
GM7480	-2.523787
GM7489	-3.5467477
GM7534	-5.076448
GM7538	-2.6144018
GM7544	-2.0591636
GM7562	-2.449469
GM7596	-4.2246017
GM7616	-3.135092
GM7628	-3.636126
GM7644	-2.7170439
GM766	-3.281992
GM7710	-1.5486023
GM7714	-4.277441
GM7827	-2.0015302
GM7849	-4.929921
GM7894	-4.960598
GM7904	-3.4755664
GM7931	-0.9834881
GM7932	-3.3523972
GM7972	-3.9597037
GM7978	-4.8494387
GM7980	-4.514676
GM8005	-1.2861657
GM8013	-2.528907
GM805	-2.5891302
GM806	-2.6235545
GM8075	-1.7614932
GM8094	-4.8965707
GM8140	-2.3363888
GM8149	-4.8178496
GM8165	-3.335743
GM8212	-4.370299
GM8220	-2.0812852
GM8256	-3.5044692
GM826	-2.001555
GM833	-0.9551172
GM8369	-2.2597802
GM8439	-3.5798032
GM8479	-1.7380974
GM8493	-1.0592103
GM8505	-1.7702098
GM8579	-2.432207
GM8587	-0.6831899
GM8633	-0.7502527

GM867	-4.5198803
GM8672	-4.3774214
GM8674	-4.915433
GM8702	-2.762043
GM8817	-5.213969
GM884	-3.7571065
GM904	-4.2298703
GM9047	-3.3030884
GM9054	-1.5032122
GM9099	-1.912549
GM9112	-2.8932943
GM9258	-1.3051283
GM9325	-2.7595243
GM9376	-5.332135
GM9465	-3.039721
GM9513	-3.1514332
GM9530	-1.0375817
GM9548	-3.7382975
GM9558	-2.3362377
GM9573	-3.957778
GM960	-2.7569788
GM973	-2.6001492
GM9733	-3.1671772
GM9734	-1.4377921
GM9744	-1.4467092
GM9758	-3.3380704
GM9767	-4.379287
GM9776	-2.3520257
GM9777	-3.8565142
GM9779	-3.3701358
GM9789	-3.7261784
GM9804	-1.1163912
GM9831	-2.2922702
GM9860	-3.462207
GM9866	-2.8719835
GM9869	-3.1173582
GM9871	-4.203532
GM9873	-3.550217
GM9876	-2.2079585
GM9878	-2.621574
GM9902	-3.857739
GM9910	-1.1153135
GM9914	-3.0044637
GM9917	-2.6731498
GM9918	-3.0286882

GM9919	-2.3183825
GM9928	-4.481199
GM9937	-4.890285
GM9948	-1.7863531
GM9951	-4.032154
GM9958	-2.5813878
GM9959	-3.8883812
GM996	-2.8785641
GM9960	-2.4672246
GM9962	-5.719945
GM9963	-1.7313387
GM9973	-3.1932244
GM9979	-2.3766475
GM9984	-4.6280274
GML	-2.6361954
GMNC	-3.388848
GMNN	-1.8308091
GMPR	-3.3228698
GNA14	-4.583281
GNA15	-2.1552286
GNAI1	-2.084995
GNAO1	-3.1384392
GNAT3	-1.350457
GNAZ	-3.5448718
GNB3	-2.5330825
GNB4	-1.4920967
GNG11	-2.1666396
GNG13	-5.381052
GNG2	-0.9715035
GNG3	-2.6467586
GNG7	-0.8764422
GNGT1	-2.966694
GNGT2	-0.3130901
GNRHR	-3.446246
GON4L	-2.7396338
GP1BA	-4.060013
GP1BB	-3.704181
GP2	-3.4657974
GP5	-4.561072
GP9	-4.4123173
GPAT2	-0.995312
GPBAR1	-4.178072
GPC1	-2.2471216
GPC2	-3.2875834
GPC3	-2.8033957

GPC5	-2.1907725
GPC6	-2.6897671
GPER1	-8.313471
GPHA2	-1.0783119
GPIHBP1	-1.602802
GPLD1	-1.9203682
GPM6A	-6.062023
GPM6B	-0.9602029
GPR101	-3.4812818
GPR111	-4.4325504
GPR112	-1.1427112
GPR114	-1.8142078
GPR115	-5.9183917
GPR116	-1.5640991
GPR123	-3.8559053
GPR124	-2.3174715
GPR126	-2.027704
GPR132	-0.4439333
GPR133	-2.635024
GPR135	-5.1266127
GPR137B	-1.1189563
GPR137B-PS	-1.1804922
GPR137C	-3.9928396
GPR139	-2.1920247
GPR142	-3.3171482
GPR143	-3.480105
GPR15	-3.3621721
GPR151	-1.5284
GPR152	-2.5520015
GPR153	-2.094912
GPR155	-1.2095778
GPR160	-1.1494668
GPR161	-3.2025042
GPR162	-2.3413393
GPR174	-1.2695467
GPR179	-4.137962
GPR180	-2.7773337
GPR183	-3.48455
GPR21	-3.6522722
GPR25	-1.659692
GPR27	-4.6506004
GPR33	-1.777482
GPR34	-2.2909775
GPR35	-2.6430528
GPR37L1	-3.9251323

GPR4	-2.347374
GPR45	-3.0616412
GPR56	-0.6279619
GPR61	-4.8693123
GPR63	-2.6520648
GPR68	-3.299074
GPR88	-4.5405326
GPR89	-0.3522432
GPR97	-0.7230451
GPR98	-2.0004697
GPRASP1	-0.7854407
GPRASP2	-2.2501132
GPRC5A	-1.3699157
GPRC5D	-1.5260406
GPRC6A	-3.2741623
GPRIN2	-1.1278448
GPRIN3	-1.6360428
GPSM1	-1.5482497
GPSM3	-0.6163819
GPX2	-4.8561482
GPX3	-2.792599
GPX6	-4.066255
GPX7	-1.6451037
GPX8	-3.0529494
GRAMD1B	-0.5733125
GRAMD2	-3.9453647
GRAP	-1.2823741
GRAP2	-2.5817504
GRB10	-1.6439688
GRCC10	-0.4390566
GREB1	-2.0408092
GRHL2	-5.6071396
GRIA1	-3.607539
GRIA3	-3.388242
GRID2	-2.7025821
GRID2IP	-1.5071383
GRIFIN	-3.3796384
GRIK2	-2.6480649
GRIK4	-3.740601
GRIN1	-1.0009003
GRIN2A	-3.0978386
GRIN2B	-2.6132207
GRIN2C	-3.130915
GRIN2D	-3.9518013
GRIN3B	-2.1441305

GRIP1	-2.624764
GRIP2	-4.0425825
GRK1	-2.8324559
GRK5	-1.2298374
GRM1	-2.9060202
GRM4	-3.6316257
GRM5	-1.7914331
GRM8	-1.312283
GRPR	-4.6398735
GRRP1	-3.0351405
GRXCR2	-2.785703
GSAP	-0.4571707
GSDMA3	-3.399554
GSDMC4	-5.0121417
GSDMCL1	-1.0994418
GSDMCL2	-5.929964
GSG1	-4.4505415
GSG1L	-4.3923454
GSN	-5.0208607
GSTA4	-2.139278
GSTM1	-0.3939712
GSTM7	-3.070189
GSTO2	-1.9373143
GSX2	-3.1730242
GTF2A1L	-2.8628953
GTPBP3	-1.7460914
GUCY1A2	-3.83785
GUCY1A3	-3.9472916
GUCY1B2	-2.0337627
GUCY1B3	-3.2536077
GUCY2E	-3.3257394
GUCY2F	-3.5981655
GUF1	-1.3147385
GULP1	-3.9510915
GYG	-0.5898774
GYKL1	-4.6250787
GZMA	-0.9967954
GZMC	-0.8345211
GZME	-2.9092112
GZMK	-0.4442842
GZMM	-4.3132176
H1FOO	-5.376087
H1FX	-3.8337502
H2-AA	-0.6046169
H2-AB1	-0.9148462

H2AFB2	-4.011138
H2AFY3	-1.2638559
H2BFM	-2.2907796
H2-DMA	-7.1505904
H2-DMB1	-2.1257384
H2-DMB2	-1.9394491
H2-EA-PS	-1.2262926
H2-EB1	-1.5659902
H2-EB2	-2.0330358
H2-M1	-2.947433
H2-M10.2	-2.5690992
H2-M10.4	-2.8015583
H2-M9	-1.637775
H2-OA	-1.0264516
H2-OB	-2.4911797
H2-T18	-2.9834757
H2-T24	-0.7686567
H2-T3	-2.4826126
H60C	-2.68692
HAND1	-1.7929802
HAPLN3	-3.0805893
HAS2	-3.0236568
HAS2OS	-2.6676097
HAS3	-4.567337
HAVCR1	-0.3866439
HAVCR2	-2.3531065
HBB-BH2	-3.2613437
HBQ1A	-4.319727
HBQ1B	-1.1177354
HCFC1R1	-0.3367236
HCN3	-4.2937374
HCN4	-2.3927839
HCRT	-4.453656
HCRTR1	-1.3347309
HCRTR2	-3.2886555
HCST	-0.7618372
HDAC6	-0.4958713
HDAC7	-0.997206
HDGFRP3	-1.7137799
HDHD1A	-4.3632803
HDHD3	-0.5438235
HEATR9	-5.364872
HEBP2	-2.0647087
HECW2	-2.5037851
HEMK1	-1.349668

HEMT1	-0.830298
HEPACAM2	-3.9682982
HEPH	-2.62569
HEPHL1	-4.187635
HES1	-2.0277073
HES2	-2.299825
HES3	-1.5404475
HES5	-2.476302
HES7	-0.9242475
HEY1	-6.4255486
HEYL	-4.842219
HHATL	-4.5937634
HHIP	-0.4991288
HHIPL1	-3.088714
HHLA1	-3.985444
HIF3A	-2.1564672
HIGD1B	-4.756172
HILS1	-3.685193
HIP1	-1.688082
HIST1H1A	-0.4670072
HIST1H1B	-1.7831979
HIST1H1D	-2.5097568
HIST1H1E	-0.4382703
HIST1H1T	-6.5884285
HIST1H2AA	-0.5314262
HIST1H2AB	-1.1593177
HIST1H2AG	-0.9833043
HIST1H2AN	-0.6608899
HIST1H2AO	-1.0018928
HIST1H2BB	-2.2169116
HIST1H2BE	-0.8534737
HIST1H2BP	-3.2083385
HIST1H3F	-0.7379053
HIST1H3G	-3.078639
HIST1H3I	-2.8528988
HIST2H2BB	-0.7574611
HIST2H2BE	-0.9619694
HIST2H3B	-0.7947357
HIST3H2BB-P	-2.1692173
HIVEP3	-4.406503
HKDC1	-1.2242594
HLX	-1.1087496
HMBOX1	-1.2313924
HMCN1	-4.3715806
HMGA2-PS1	-2.4856524

HMGB4	-5.3689322
HMGB4OS	-2.5610993
HMGCLL1	-2.5400772
HMGN3	-2.3472655
HMHA1	-1.1115057
HMX1	-1.2877259
HMX2	-5.513782
HNF4G	-2.0585628
HNMT	-1.6015816
HOMER3	-0.6539094
HOPX	-2.8228476
HORMAD2	-1.6865296
HOXA1	-1.3311148
HOXA11OS	-1.9951012
HOXA13	-4.096681
HOXA2	-2.1330543
HOXA3	-1.8140795
HOXA4	-0.8582336
HOXA5	-3.5127008
HOXA6	-4.8823347
HOXA7	-2.2837029
HOXB2	-1.3403919
HOXB4	-1.2697828
HOXB5	-3.0427306
HOXB5OS	-4.4193735
HOXB6	-3.8642452
HOXB7	-1.7252564
HOXC10	-6.542216
HOXC11	-2.6677885
HOXC12	-3.816959
HOXC4	-3.4492745
HOXC5	-4.921961
HOXC6	-2.5885444
HOXC9	-2.7521973
HOXD10	-4.260543
HOXD11	-3.0194755
HOXD3	-2.8414893
HOXD3OS1	-3.9296687
HOXD4	-2.5396166
HOXD8	-2.1800454
HPCAL4	-3.7226095
HPDL	-3.5484877
HPGD	-2.1664722
HPS1	-1.1458445
HPSE2	-2.9798765

HRASLS5	-3.9713926
HRC	-3.0250027
HRCT1	-2.3103974
HRH1	-1.7559757
HRH3	-4.0501556
HRH4	-1.7360735
HRK	-2.0847702
HRNR	-3.4610584
HS3ST1	-2.645169
HS3ST3A1	-2.5472112
HS3ST6	-1.9427075
HS6ST2	-3.6260328
HSBP1L1	-2.0001004
HSD17B1	-1.7669361
HSD17B14	-0.4837623
HSD3B6	-3.1064925
HSF4	-0.8822789
HSFY2	-4.1048536
HSH2D	-1.5027418
HSPA12B	-3.180651
HSPA1L	-2.2340455
HSPB1	-1.8392384
HSPB2	-3.765553
HSPB3	-2.607895
HSPB6	-0.9284604
HSPB7	-5.119361
HSPB9	-2.4466789
HSPG2	-1.7200682
HTR1A	-3.2771907
HTR1F	-3.8532763
HTR2A	-4.7265477
HTR2B	-2.4781227
HTR2C	-1.7852902
HTR3B	-2.6585796
HTR4	-2.8219342
HTR6	-2.6138234
HTRA1	-0.5720995
HTRA3	-2.779904
HVCN1	-1.0540371
HYAL6	-3.9230175
HYDIN	-2.6701226
HYLS1	-1.3286488
IBSP	-3.823812
ICA1	-0.282263
ICAM2	-1.9542243

ICAM5	-4.020321
ID1	-1.0879958
ID4	-2.357935
IDI2	-5.1520243
IDS	-2.264801
IER5	-0.9980698
IER5L	-1.4070451
IFI44L	-2.760892
IFITM10	-2.8658001
IFLTD1	-2.8298922
IFNA11	-4.850772
IFNA13	-3.3418355
IFNA14	-2.053518
IFNA16	-3.1804972
IFNA2	-1.5419848
IFNA4	-2.5017169
IFNAB	-1.5561323
IFNK	-1.8835324
IFNL3	-4.358394
IFT74	-1.4405129
IFT81	-0.9679773
IFT88OS	-2.3341362
IGBP1B	-3.9336739
IGDCC3	-2.240612
IGF1OS	-1.1749575
IGF1R	-2.213868
IGF2BP1	-1.318541
IGF2OS	-0.8839679
IGFBP5	-3.0818183
IGFBP6	-5.7468176
IGFL3	-3.658191
IGHV14-2	-2.020379
IGH-V3660	-4.198821
IGH-VJ558	-1.8021286
IGJ	-1.8798306
IGKV4-53	-2.1633627
IGKV4-72	-2.6681926
IGKV6-20	-2.9062924
IGLL1	-1.6682739
IGLON5	-3.0253186
IGLV1	-1.6973183
IGSF1	-1.8908675
IGSF10	-3.6098025
IGSF3	-1.6917725
IHH	-4.6452456

IIGP1B	-2.013615
IKZF2	-1.5422373
IKZF3	-1.0517216
IL12RB2	-1.8489907
IL13	-3.1172824
IL15	-1.5894089
IL16	-0.7346735
IL17B	-4.1241636
IL17C	-1.0470572
IL17D	-2.6824498
IL17F	-2.1244562
IL17RD	-3.5591102
IL17RE	-6.2496033
IL18R1	-3.1141658
IL1BOS	-3.1893613
IL1F5	-2.489891
IL1RL1	-1.3488216
IL1RL2	-1.2410781
IL20	-1.2427075
IL22	-4.077377
IL22RA2	-3.1353164
IL24	-2.5066442
IL25	-6.5983095
IL31	-3.5756052
IL31RA	-2.462565
IL34	-0.7537398
IL4	-2.6888516
IL5	-3.266145
IL5RA	-4.6668553
IL7R	-0.5416775
IL9R	-2.2617722
ILDR1	-3.3284435
INA	-1.9124283
INADL	-0.9204266
INMT	-6.589997
INO80E	-0.5300851
INPP5E	-1.3234992
INPP5J	-2.758684
INPP5K	-0.5397418
INS1	-2.0748682
INS2	-2.305696
INSL5	-1.804456
INSM1	-4.5380564
INSM2	-2.4241507
INSRR	-1.0033386

INTS1	-1.3161905
INTS2	-1.6870251
INTS6	-1.7272449
IP6K3	-2.8035214
IPCEF1	-3.4425569
IQCA	-3.3456707
IQCD	-0.9505971
IQCF1	-3.9366345
IQCF3	-5.3450584
IQCF4	-4.6190624
IQCF5	-2.2766001
IQGAP1	-0.753144
IQGAP3	-3.8520222
IQSEC2	-0.5816901
IQUB	-3.500036
IRF4	-1.7695391
IRGC1	-2.6375394
IRS3	-3.3102145
IRS4	-0.5306115
IRX1	-8.214912
IRX2	-5.101921
IRX3	-4.1872663
IRX3OS	-2.686684
IRX4	-4.74227
IRX5	-3.2556856
ISL1	-3.1460469
ISLR	-3.329973
ISLR2	-3.1240826
ISM1	-5.6660056
ISM2	-5.3023844
ISOC2B	-2.1415343
ISPD	-1.9796957
ITGA11	-3.6248724
ITGA2B	-2.6374872
ITGA3	-3.9975383
ITGA4	-0.5322883
ITGA6	-1.2064121
ITGA8	-8.241262
ITGAD	-4.3971663
ITGAX	-3.4337373
ITGB1BP2	-1.9058647
ITGB2L	-2.6846042
ITGB4	-4.454197
ITGB6	-4.7158966
ITGB7	-1.7952635

ITGB8	-3.4270377
ITGBL1	-2.7272542
ITLN1	-0.7723246
ITM2A	-3.983726
ITPKA	-2.323123
ITPR3	-1.1796105
ITPRIPL1	-3.4725626
ITPRIPL2	-1.5783422
IVL	-5.3558598
IZUMO1	-5.6103
IZUMO2	-5.087632
IZUMO4	-1.1951127
JADE3	-3.8035088
JAG1	-1.8097289
JAG2	-3.8045034
JAKMIP1	-3.928061
JAKMIP2	-4.0041018
JAKMIP3	-4.748972
JAM3	-0.4149952
JAZF1	-3.036702
JPH3	-2.5675907
JPH4	-4.9177666
JUNOS	-4.2563243
JUP	-1.5827518
KALRN	-0.8394525
KANK2	-0.9678366
KANK3	-2.5072658
KANK4	-3.5595565
KANSL1L	-4.243514
KAP	-5.3230543
KATNAL2	-4.4901342
KAZALD1	-3.0510428
KAZN	-2.1951678
KBTBD11	-2.430563
KBTBD13	-2.518593
KCNA10	-2.9899092
KCNA2	-1.8402988
KCNA3	-2.3293962
KCNA5	-2.0576532
KCNA6	-2.5184062
KCNAB1	-2.756017
KCNAB2	-1.5678632
KCNB1	-1.7552047
KCNB2	-3.2253308
KCNC1	-2.9794216

KCNC3	-3.337859
KCNC4	-2.8536992
KCND2	-3.7843378
KCND3	-2.9360642
KCND3OS	-2.4797862
KCNE1L	-0.6394656
KCNE2	-6.0056696
KCNF1	-2.9693851
KCNG1	-3.0865972
KCNG3	-2.4974835
KCNH1	-2.9032078
KCNH7	-2.0347245
KCNH8	-4.1799226
KCNIP2	-5.745323
KCNIP3	-2.0802915
KCNIP4	-2.6707268
KCNJ10	-0.3537624
KCNJ11	-2.1023562
KCNJ12	-2.80246
KCNJ15	-3.8444397
KCNJ3	-2.595623
KCNJ5	-3.660915
KCNJ6	-1.1260643
KCNJ9	-5.4571652
KCNK1	-3.0994327
KCNK12	-3.6049397
KCNK15	-4.9492693
KCNK16	-4.823161
KCNK18	-3.6415246
KCNK3	-4.309755
KCNK4	-1.1538405
KCNK7	-3.6229088
KCNK9	-1.1448591
KCNMA1	-2.891894
KCNMB2	-3.2575586
KCNMB4	-2.8224573
KCNMB4OS2	-3.5748985
KCNN1	-4.0029664
KCNN3	-3.7416015
KCNN4	-4.4372582
KCNQ1	-4.51048
KCNQ2	-2.102223
KCNQ3	-2.4504354
KCNS1	-2.7226515
KCNS3	-5.1417875

KCNU1	-2.147422
KCTD10	-1.1616938
KCTD12B	-2.417822
KCTD16	-3.302088
KCTD7	-6.982626
KDELC2	-1.9071095
KDM2A	-0.8959248
KDM3B	-0.3082423
KDM7A	-0.9722321
KDR	-2.0763152
KEG1	-3.911138
KHDC1C	-5.62344
KHDRBS2	-2.0408018
KIF11	-3.8351672
KIF13B	-3.284433
KIF14	-1.9224672
KIF15	-1.8083735
KIF17	-2.5744853
KIF18A	-1.5224848
KIF1A	-1.4228414
KIF20A	-1.3856585
KIF23	-0.9940283
KIF24	-2.2054353
KIF26B	-1.0911121
KIF2A	-1.7656877
KIF2C	-2.911697
KIF3C	-0.4509428
KIF4-PS	-2.7273831
KIF5A	-2.850134
KIF6	-5.397433
KIF7	-3.574685
KIF9	-1.2567258
KIFC1	-2.0468287
KIFC2	-1.3302331
KIRREL	-0.5141456
KIRREL2	-6.094331
KIRREL3	-0.6133733
KISS1	-3.0064628
KISS1R	-2.938514
KIT	-1.8121712
KITL	-4.7792835
KLC1	-0.7033579
KLC3	-1.8769999
KLF16	-0.9403729
KLF17	-4.087653

KLF5	-3.8502662
KLF8	-2.7940233
KLHDC1	-1.5837278
KLHDC7B	-2.2430859
KLHDC8A	-1.8009315
KLHDC8B	-1.2328565
KLHDC9	-3.1735845
KLHL1	-9.050742
KLHL13	-0.8643839
KLHL14	-0.4407806
KLHL17	-0.4676926
KLHL23	-2.9127243
KLHL29	-2.7358024
KLHL3	-3.6563723
KLHL30	-5.0482044
KLHL31	-4.399263
KLHL32	-3.030139
KLHL34	-5.0457006
KLHL38	-2.027202
KLHL40	-5.1236057
KLHL5	-1.2952738
KLHL8	-0.5006962
KLK1	-1.0677998
KLK10	-4.7395644
KLK11	-4.0958104
KLK12	-2.6337614
KLK13	-5.8363957
KLK15	-2.2022924
KLK1B1	-4.028584
KLK1B11	-2.5315268
KLK1B21	-1.1836617
KLK1B22	-2.5410807
KLK1B26	-1.3008506
KLK1B3	-1.277147
KLK1B4	-1.8376925
KLK1B5	-0.7608988
KLK1B8	-2.9932609
KLK1B9	-1.7653067
KLK4	-2.108654
KLK5	-3.0019603
KLK6	-2.9318962
KLK8	-2.6040177
KLK9	-1.3101897
KLRA10	-3.4232838
KLRA12	-2.4994214

KLRA15	-1.7172675
KLRA16	-0.8764236
KLRA17	-3.725551
KLRA22	-1.5195539
KLRA4	-2.061107
KLRA7	-0.7346227
KLRA8	-1.7003952
KLRB1	-5.0804253
KLRB1A	-2.5727825
KLRB1B	-3.285288
KLRB1C	-2.4907675
KLRB1-PS1	-4.627091
KLRC1	-1.5958316
KLRC3	-2.0304046
KLRD1	-1.5753462
KLRE1	-1.6803117
KLRG1	-3.85193
KLRG2	-3.3041434
KLRI1	-1.2307792
KLRI2	-2.594686
KLRK1	-0.539825
KMT2D	-0.925833
KNDC1	-4.012495
KNTC1	-1.5093052
KPRP	-4.312611
KREMEN2	-4.8299017
KRT10	-2.2476308
KRT13	-1.835263
KRT15	-5.2426014
KRT19	-4.0564804
KRT2	-4.6834383
KRT20	-4.8402376
KRT23	-5.072344
KRT24	-5.269916
KRT26	-5.356721
KRT27	-7.145459
KRT28	-3.283238
KRT33A	-4.2457156
KRT35	-3.3769908
KRT36	-3.0880547
KRT39	-3.3752775
KRT4	-5.0200624
KRT40	-2.4212112
KRT42	-3.4814978
KRT5	-3.4113894

KRT6A	-3.1589112
KRT6B	-3.7806587
KRT7	-5.3775673
KRT72	-4.4958086
KRT74	-4.6090155
KRT75	-1.21247
KRT77	-1.7439306
KRT78	-3.4040284
KRT79	-3.5790882
KRT80	-1.1851668
KRT81	-4.479799
KRT82	-2.106734
KRT86	-3.2185073
KRT9	-2.9038215
KRTAP10-4	-3.241869
KRTAP12-1	-2.364334
KRTAP15	-3.9224079
KRTAP16-1	-4.047616
KRTAP16-3	-3.0073988
KRTAP17-1	-4.16166
KRTAP19-5	-2.4619544
KRTAP21-1	-2.5451164
KRTAP24-1	-3.7389243
KRTAP26-1	-2.546883
KRTAP31-2	-4.979601
KRTAP4-1	-5.093605
KRTAP4-16	-1.1672161
KRTAP4-7	-2.5796218
KRTAP5-4	-1.9498601
KRTAP5-5	-1.9406497
KRTAP8-1	-4.0810456
KRTCAP3	-2.6451051
KRTDAP	-3.6473541
KSR1	-1.2846828
KSR2	-2.1296985
KY	-5.4168735
L1CAM	-2.659653
L3MBTL2	-2.5146794
LACTBL1	-3.5918589
LAD1	-2.6498237
LAIR1	-1.4454632
LALBA	-1.1035471
LAMA2	-3.465304
LAMA3	-2.8495066
LAMA4	-1.0895727

LAMA5	-0.6501129
LAMB1	-1.8115523
LAMB2	-3.8455422
LAMB3	-2.4351742
LAMC1	-0.7833159
LAMC2	-4.8349314
LAMC3	-2.823524
LAMP3	-1.5534174
LAPTM5	-0.2770588
LARP6	-3.8850558
LAT	-3.6835406
LAT2	-1.9183493
LATS2	-0.6956918
LAX1	-4.738757
LAYN	-1.8749666
LBH	-2.8789907
LBX1	-0.5213528
LBX2	-1.243581
LCA5	-3.8764155
LCA5L	-2.272893
LCE1B	-1.1663954
LCE1C	-2.489718
LCE1G	-3.5324411
LCE1H	-2.3770442
LCE1I	-3.414012
LCE1J	-2.2525032
LCE1L	-2.8587093
LCE1M	-3.442721
LCE3C	-2.8434181
LCE3D	-2.1485822
LCK	-0.8597372
LCMT1	-0.4515472
LCN10	-5.103554
LCN11	-3.271888
LCN3	-4.3458557
LCN5	-3.004811
LCN6	-0.8335271
LCT	-2.5450764
LCTL	-5.4626026
LDB2	-3.348372
LDB3	-2.8721259
LDHB	-3.1034768
LDHC	-4.1558013
LDLRAD1	-3.1582334
LDLRAD4	-3.9671924

LDOC1	-3.942946
LDOC1L	-0.8818862
LEF1	-2.7133884
LEFTY1	-1.1793146
LEKR1	-1.032953
LELP1	-1.5081542
LENG1	-0.7523353
LEO1	-1.3497055
LEPREL1	-4.536104
LEPREL2	-2.0679133
LEPREL4	-1.3153622
LETM2	-3.0500183
LFNG	-1.3172941
LGALS1	-1.2967694
LGALS12	-2.017664
LGI2	-2.1380777
LGI3	-7.3219986
LGI4	-1.0218732
LGR6	-1.7452502
LHB	-1.8385193
LHFP	-1.993629
LHFPL2	-0.5061526
LHX1	-3.9375927
LHX4	-3.4533415
LHX9	-1.856807
LIF	-4.6112194
LILRA5	-0.9579209
LIM2	-4.090988
LIMCH1	-7.850774
LIMD2	-0.6235931
LIMK2	-1.8675921
LIN28B	-2.4459312
LIN7B	-2.0237143
LINGO3	-3.2857697
LIPE	-1.9800329
LIPF	-4.7581415
LIPG	-2.0910778
LIPH	-4.2552834
LIPO4	-3.9295497
LIX1	-4.8003263
LKAAEAR1	-3.9427686
LMBR1	-1.16886
LMCD1	-1.724783
LMNB1	-1.0117607
LMO1	-3.1233196

LMO7	-2.6697695
LMOD1	-1.7030354
LMOD2	-2.1359842
LMOD3	-0.4403493
LMTK3	-0.9091461
LMX1B	-3.6939235
LOC1000417	-0.8822024
LOC1005025	-3.0039625
LOC1005030	-5.420265
LOC1008621	-2.9164352
LOC1010556	-4.260281
LOC1010557	-2.717997
LOC1010558	-1.0917535
LOC1010558	-1.8468437
LOC1010559	-4.4333086
LOC1010559	-4.1991863
LOC1010559	-3.2550192
LOC1026318	-3.5706482
LOC1026321	-2.713333
LOC1026322	-2.1407332
LOC1026324	-4.0537844
LOC1026328	-1.3861495
LOC1026330	-2.3326468
LOC1026331	-3.7536902
LOC1026333	-2.9368331
LOC1026334	-2.8327096
LOC1026336	-0.3085673
LOC1026337	-4.7976747
LOC1026338	-3.5376034
LOC1026341	-2.4712121
LOC1026345	-2.437786
LOC1026346	-3.2030597
LOC1026352	-5.149336
LOC1026353	-3.9268084
LOC1026355	-4.235448
LOC1026356	-1.6042166
LOC1026360	-4.7669945
LOC1026361	-2.5542543
LOC1026362	-1.421519
LOC1026367	-1.709872
LOC1026367	-3.1074152
LOC1026369	-3.5808
LOC1026369	-0.7186196
LOC1026370	-2.9961903
LOC1026377	-2.8736246

LOC1026379	-2.6492345
LOC1026380	-2.2866216
LOC1026385	-1.3124914
LOC1026386	-3.2052352
LOC1026387	-4.236853
LOC1026388	-1.3478167
LOC1026388	-1.9227706
LOC1026389	-0.82099
LOC1026389	-2.8343291
LOC1026396	-3.3989317
LOC1026397	-3.0956578
LOC1026398	-0.7710559
LOC1026398	-4.526987
LOC1026405	-1.8038297
LOC1026412	-2.27122
LOC1026412	-3.9355183
LOC1026413	-2.6161354
LOC1026416	-2.3664436
LOC1026416	-2.7524548
LOC1026417	-3.3807104
LOC1026418	-2.01775
LOC1026420	-4.0081854
LOC1026420	-1.728405
LOC1026420	-4.0218873
LOC1026421	-2.3700576
LOC1026422	-3.424786
LOC1026423	-2.294404
LOC1026424	-3.0503912
LOC1026424	-2.8927307
LOC1026425	-3.0620253
LOC1026425	-1.8691819
LOC1026426	-3.735645
LOC1026427	-0.8480735
LOC1026429	-1.3742163
LOC1026429	-1.6756697
LOC1026431	-5.417712
LOC1026431	-3.4229212
LOC106740	-1.379709
LOC171588	-2.5752413
LOR	-2.1542497
LOX	-0.5465212
LOXHD1	-4.1704006
LOXL3	-2.072489
LPAR2	-1.6812351
LPAR3	-2.2735243

LPAR4	-5.41503
LPCAT1	-2.6819642
LPCAT2B	-3.461761
LPHN1	-3.8642168
LPHN2	-2.843385
LPHN3	-2.6204123
LPL	-1.0323675
LRCH3	-4.658965
LRFN1	-1.3663366
LRFN4	-2.2628276
LRFN5	-3.1568437
LRGUK	-2.6944318
LRIT2	-1.5302715
LRP1B	-3.4505334
LRP2	-4.9747267
LRR1	-4.2326765
LRRC1	-3.0756369
LRRC10B	-3.631272
LRRC16A	-1.5695045
LRRC16B	-3.3600457
LRRC18	-1.9368072
LRRC23	-7.678857
LRRC26	-6.6120706
LRRC29	-1.0023384
LRRC30	-1.7329993
LRRC34	-3.271532
LRRC36	-3.082864
LRRC4	-3.4226708
LRRC45	-0.7816403
LRRC46	-2.0697436
LRRC49	-3.301142
LRRC4B	-3.8481426
LRRC4C	-3.1939557
LRRC51	-1.9133334
LRRC56	-0.8995385
LRRC57	-0.4632616
LRRC6	-4.1194196
LRRC63	-1.6260469
LRRC7	-1.2792227
LRRC71	-4.552285
LRRC73	-2.9223342
LRRC74	-5.867107
LRRC75B	-0.8343899
LRRC8B	-1.7553713
LRRC8E	-1.9043076

LRRIQ3	-1.2536345
LRRIQ4	-5.591029
LRRK1	-0.4922335
LRRK2	-1.9186444
LRRN2	-2.833598
LRRN3	-4.355873
LRRN4CL	-2.7589006
LRRTM1	-1.2057192
LRRTM2	-3.6107852
LRTM1	-2.4547
LSMEM2	-1.4129949
LSP1	-1.645139
LTA	-5.1763725
LTBP1	-2.8871298
LTBP2	-5.432625
LTBP3	-3.6868906
LTBP4	-3.2216163
LTC4S	-2.0519316
LTK	-5.372281
LURAP1	-1.6443353
LUZP2	-2.6404788
LXN	-2.3062742
LY6F	-1.4607813
LY6G5B	-2.2455065
LY6G6C	-2.9316158
LY6G6D	-4.661375
LY6G6E	-3.4115987
LY6I	-1.5186837
LY75	-1.5023336
LY9	-0.9017141
LYG2	-4.452819
LYL1	-1.3006165
LYNX1	-3.5652301
LYPD2	-3.4287517
LYPD3	-3.0394814
LYPD6	-2.3539758
LYPD6B	-3.668476
LYPD8	-3.6037521
LYRM9	-2.0759292
LYSMD2	-1.2844174
LYST	-1.9885411
LYZ1	-1.950383
LYZ2	-2.2944791
LYZL1	-3.9962087
LYZL4	-3.2666996

LYZL6	-2.5102262
LZTS1	-2.2419229
LZTS2	-1.011493
M1AP	-3.059508
MAATS1	-3.3472908
MAB21L2	-1.4061024
MAB21L3	-1.5643976
MACC1	-5.2097993
MACF1	-1.3507245
MACROD2OS	-3.3633847
MAD2L1	-1.7166672
MADCAM1	-1.4309137
MAEL	-5.6260233
MAGEA1	-3.6765928
MAGEA10	-4.4789577
MAGEA3	-1.5754328
MAGEA5	-5.869891
MAGEA6	-1.1518874
MAGEA8	-2.092246
MAGEB1	-3.6348376
MAGEB3	-1.9250314
MAGEE1	-1.3695502
MAGEE2	-3.228991
MAGI2	-3.7987862
MAGI3	-1.3686116
MAGIX	-1.6133938
MAL	-3.8001506
MAL2	-1.916673
MALL	-4.296649
MAMDC2	-5.1444244
MAMDC4	-1.8232815
MAML2	-2.5938885
MAML3	-2.1035151
MAMLD1	-3.327628
MAMSTR	-1.6738758
MAN2C1OS	-0.5876803
MANEAL	-4.637903
MAOA	-0.6785317
MAP10	-2.157681
MAP1A	-3.2084076
MAP1LC3A	-0.4138582
MAP2	-4.4993253
MAP2K6	-0.7079897
MAP2K7	-1.1176784
MAP3K10	-3.0419981

MAP3K19	-3.4256306
MAP3K7CL	-2.5985105
MAP3K9	-2.8850524
MAP4K1	-0.6238229
MAP4K2	-0.935869
MAP6	-3.866685
MAP7D2	-2.7646103
MAPK10	-4.2220182
MAPK12	-1.2323401
MAPK13	-0.6962302
MAPK15	-2.1615164
MAPK3	-1.1688054
MAPK8	-8.704477
MAPK8IP1	-2.6412046
MAPK8IP2	-1.9615048
MAPKBP1	-1.9025948
MAPT	-5.1591263
MARVELD1	-2.1569676
MAS1	-2.6196017
MAST1	-3.1189647
MAST4	-0.7169478
MASTL	-1.3589039
MATK	-2.6358244
MATN4	-6.9401855
MB	-5.3046217
MB21D1	-0.5994327
MBD5	-1.7232993
MBIP	-0.5316045
MBLAC2	-0.6855614
MBNL1	-0.6089242
MBNL3	-2.4777837
MBOAT1	-2.3861248
MBOAT2	-3.1759145
MBP	-2.494966
MC5R	-1.5897331
MCC	-2.0046394
MCF2L	-2.484883
MCHR1	-3.7652888
MCIDAS	-0.4232886
MCM2	-0.5809555
MCM8	-1.422184
MCM9	-0.7642436
MCOLN3	-2.7762468
MCPT4	-3.0045786
MCPT8	-3.6533663

MCPT9	-6.3238544
MCPT-PS1	-3.5942292
MDGA2	-2.482857
MDH1B	-3.5831118
MDK	-1.2151833
MDM1	-1.2955263
ME3	-2.32049
MECOM	-1.6384155
MED12L	-0.892616
MED18	-1.3361042
MED9OS	-1.6988355
MEF2C	-2.6513093
MEG3	-1.4244182
MEGF10	-2.0023723
MEGF11	-2.5761702
MEGF6	-3.1056285
MEI1	-4.8220625
MEIG1	-4.8343215
MEIS1	-1.8788412
MEIS3	-1.463882
MEOX1	-5.0233946
MEOX2	-6.4802194
MESP1	-2.2507215
MESP2	-1.7104964
MEST	-2.9372253
METRN	-0.9243064
METTL11B	-5.160274
METTL13	-4.155986
METTL24	-5.190866
METTL7A1	-1.6810949
METTL8	-2.3322437
MEX3A	-4.543232
MEX3B	-2.2857566
MFAP2	-5.6847878
MFAP3L	-3.1675751
MFAP4	-9.124962
MFAP5	-7.7714376
MFGE8	-1.4851997
MFNG	-2.6375346
MFSD6L	-1.8008116
MFSD7A	-0.71176
MFSD9	-1.9643464
MGAT3	-4.0536466
MGAT4C	-3.9241645
MGLL	-0.8470886

MGP	-5.9103727
MGST2	-1.0178144
MGST3	-1.2488344
MIA	-3.2831297
MICAL1	-1.3229783
MICAL2	-1.9987669
MICALCL	-3.7659678
MICU3	-1.756958
MID1IP1	-1.3089178
MID2	-1.984256
MIEF2	-0.611119
MILL2	-3.947464
MIPOL1	-2.1753743
MIRG	-1.467751
MIS18A	-1.6704493
MIS18BP1	-1.8006978
MISP	-5.641671
MIXL1	-2.48403
MKNK1	-1.3142271
MKRN2	-0.8722992
MLANA	-4.8457813
MLC1	-4.0565815
MLF1	-3.3798113
MLIP	-4.521566
MLLT3	-1.7176476
MLPH	-2.2372339
MMP10	-3.305223
MMP11	-4.738107
MMP12	-0.600235
MMP15	-4.682113
MMP17	-1.0648894
MMP2	-3.9207063
MMP21	-2.5388186
MMP23	-3.0386102
MMP24	-1.2646745
MMRN1	-2.6371326
MNAT1	-0.3843639
MNS1	-4.5695763
MOBP	-2.4921484
MOGAT1	-2.5241435
MORC2B	-3.022321
MORN1	-1.1127384
MORN2	-1.1627846
MORN3	-2.795894
MORN5	-3.5854745

MOS	-2.8658886
MOV10L1	-2.6930885
MOXD2	-1.5407896
MPHOSPH9	-2.9240282
MPL	-2.0681682
MPP3	-1.8333216
MPP7	-0.69519
MPPED2	-2.8067904
MPTX1	-0.5987356
MR1	-1.5888228
MRC1	-1.2603872
MRC2	-3.6534305
MRGPRA1	-2.540724
MRGPRA2A	-0.4087582
MRGPRA2B	-0.506103
MRGPRA3	-2.0075793
MRGPRA4	-2.7887144
MRGPRA5	-4.0974827
MRGPRB4	-3.3962424
MRGPRG	-1.3347044
MRGPRH	-2.7083507
MRO	-1.9185557
MROH2A	-2.5929422
MROH5	-0.4150369
MROH7	-4.299556
MROH9	-1.1783206
MRPL28	-5.5635166
MRRF	-0.7681749
MRVI1	-2.1853764
MS4A1	-2.819199
MS4A10	-3.482262
MS4A14	-2.8616421
MS4A15	-1.8107014
MS4A4B	-0.7411611
MS4A7	-0.7522767
MSANTD1	-1.5879526
MSGN1	-3.4674058
MSH4	-1.733861
MSH5	-3.809991
MSL3L2	-1.8991559
MSLN	-3.384521
MSLNL	-3.7391398
MSMB	-3.2130797
MSMP	-4.211418
MSRB3	-0.5158162

MSX1	-3.0258806
MSX2	-1.1515808
MSX3	-3.1936903
MTAP7D3	-2.5068054
MTCH1	-0.9165876
MTCL1	-6.692831
MTERF1B	-1.341295
MTHFD2L	-0.3383131
MTHFSD	-1.2299137
MTL5	-0.8661449
MTNR1B	-4.2654557
MTRR	-1.5137641
MTSS1L	-4.648342
MTURN	-0.9345624
MUC13	-1.4845967
MUC20	-3.2445147
MUC4	-4.7733197
MUC5AC	-2.243279
MUC5B	-6.0325003
MUC6	-3.5174847
MUCL1	-4.8266845
MUM1	-0.4183905
MUP6	-3.1283147
MUP-PS12	-2.2759018
MURC	-3.7403488
MUSTN1	-1.6591017
MUTYH	-0.839525
MVB12B	-2.3027112
MXD3	-3.121687
MXRA7	-3.8950186
MXRA8	-3.9997845
MYB	-3.0650964
MYBPC1	-2.43782
MYBPC3	-1.0219357
MYBPH	-5.4292183
MYBPHL	-3.9412804
MYCBP	-0.600697
MYCBPAP	-2.171922
MYCN	-2.108087
MYCT1	-3.335696
MYH1	-4.094739
MYH10	-1.4375105
MYH11	-4.6817527
MYH13	-4.29805
MYH14	-3.3319528

MYH15	-5.200198
MYH4	-3.6236537
MYH6	-9.297291
MYH7	-6.0963545
MYH7B	-5.0608935
MYH8	-3.7168841
MYL10	-4.2872987
MYL12B	-0.5125902
MYL2	-4.6289062
MYL3	-4.9723845
MYL4	-5.093347
MYL6B	-2.39832
MYL7	-6.598146
MYL9	-4.05803
MYLIP	-0.7277606
MYLK	-2.506159
MYLPF	-2.178628
MYO15	-2.877402
MYO18B	-2.1700504
MYO1A	-3.2862124
MYO1C	-1.9152987
MYO1F	-0.8742998
MYO1G	-0.3588188
MYO1H	-0.9389837
MYO3A	-3.9298482
MYO3B	-1.7912426
MYO5A	-1.461993
MYO5C	-6.689849
MYO9A	-1.3768692
MYOC	-3.961097
MYOCD	-2.1697478
MYOF	-1.6865623
MYOM2	-5.701781
MYOM3	-0.8814251
MYOT	-8.670267
MYOZ1	-3.077298
MYOZ2	-4.5840693
MYOZ3	-2.6907132
MYPN	-4.642521
MYRIP	-3.6666713
MYT1L	-3.443038
MYZAP	-5.1443553
MZB1	-2.7847586
N28178	-2.792044
N4BP1	-1.5825112

NAA11	-2.7958164
NAAA	-1.2740762
NAALADL1	-2.5695355
NAALADL2	-2.0286136
NACAD	-3.970344
NACC1	-0.5049679
NAIF1	-3.1502197
NAIP7	-3.5891516
NALCN	-2.8572197
NANOS2	-2.5442133
NAP1L5	-2.2573736
NAPSA	-2.5371816
NAT14	-3.928741
NAT3	-2.5571203
NAV1	-1.4772234
NAV2	-0.4815412
NAV3	-1.8201208
NBEA	-2.6806188
NBL1	-3.223098
NCALD	-2.283533
NCAN	-2.6583767
NCAPH	-0.7364814
NCCRP1	-2.3034031
NCK2	-2.0708373
NCKAP5	-5.4486923
NCKAP5L	-3.7579384
NCMAP	-4.3503695
NCR1	-2.6237526
NCS1	-1.9426856
NCTC1	-1.6080468
NDC80	-5.724682
NDFIP2	-2.7242856
NDN	-2.4839385
NDNF	-5.3593245
NDP	-1.3675983
NDRG4	-3.8942978
NDST3	-4.0671496
NDST4	-2.074059
NDUFA4L2	-0.8365839
NDUFAF5	-1.0638514
NEB	-2.3287804
NEBL	-3.3845549
NECAB2	-2.1298409
NECAB3	-2.1510787
NEDD9	-1.4659376

NEFH	-3.926942
NEFM	-3.3958569
NEIL3	-2.3465712
NEK10	-2.6650891
NEK11	-2.2825692
NEK2	-1.4619923
NEK5	-2.0266628
NENF	-0.3827379
NEPN	-3.6768844
NES	-2.01436
NESPAS	-3.7274873
NETO1	-2.5440304
NETO2	-2.5122182
NEU2	-0.6809957
NEURL1A	-1.1633332
NEUROD4	-2.4690099
NEUROG1	-1.5410724
NEXN	-3.124419
NF2	-0.5078116
NFAM1	-0.3771498
NFATC2	-2.3367276
NFATC4	-0.9809065
NFE2	-1.6062465
NFE2L3	-1.9657421
NFIA	-0.4024367
NGB	-3.1637228
NGFRAP1	-1.2314355
NHLRC4	-4.150419
NID2	-1.367902
NIM1K	-2.8562348
NIN	-0.554323
NINL	-0.9563582
NIPA1	-1.9165409
NIPAL2	-3.8370986
NIPAL3	-2.1615412
NKAIN2	-2.3797364
NKAIN4	-6.406494
NKD1	-3.6113122
NKG7	-0.6803005
NKPD1	-5.251209
NKX1-1	-1.8272579
NKX1-2	-3.6271532
NKX2-1	-6.738655
NKX2-2	-1.0991719
NKX2-3	-4.761655

NKX2-4	-3.4555757
NKX2-5	-2.9729655
NKX2-9	-2.729356
NKX3-1	-3.1446233
NKX3-2	-5.656945
NKX6-3	-4.45167
NLE1	-2.3584087
NLGN1	-2.3180912
NLGN2	-0.3322985
NLRP10	-4.4238687
NLRP12	-0.862531
NLRP14	-1.4881113
NLRP1B	-1.9959666
NLRP2	-2.4558523
NLRP4A	-3.0524228
NLRP4B	-4.3542957
NLRP4C	-5.7245646
NLRP4D	-2.7350793
NLRP4E	-2.97244
NLRP4F	-5.1914635
NLRP4G	-3.1828527
NLRP5	-1.9758983
NLRP5-PS	-2.254187
NLRP9A	-2.7071729
NLRP9C	-1.9673421
NMBR	-2.8664732
NME5	-4.308916
NME8	-3.123357
NME9	-4.5598154
NMRK2	-2.8029594
NMU	-2.3585753
NMUR1	-4.5625143
NMUR2	-4.8678904
NNAT	-3.2009778
NOL10	-0.5946307
NOL3	-3.7824857
NOP9	-1.3203282
NOS1	-2.778553
NOTCH1	-1.6654165
NOTCH3	-3.7695875
NOTCH4	-1.2629917
NOTO	-3.4813018
NOTUMOS	-2.6452096
NOVA1	-0.9479032
NOVA2	-0.2919521

NOX1	-2.3309445
NOX4	-6.1619797
NPAS1	-3.0700498
NPAS2	-2.7001622
NPAS4	-13.699804
NPAT	-1.6654632
NPB	-1.100929
NPBWR1	-2.9771957
NPC2	-1.0919478
NPCD	-1.1309593
NPFFR1	-2.0735202
NPFFR2	-2.523397
NPHP4	-1.5521619
NPHS1	-2.9871116
NPHS2	-3.9645877
NPM2	-0.788867
NPM3	-1.7709931
NPM3-PS1	-0.8464232
NPNT	-6.2966294
NPPA	-2.8295496
NPPB	-2.476845
NPR2	-0.6724823
NPR3	-4.2156954
NPSR1	-2.4829426
NPVF	-3.3027997
NPW	-7.1438684
NPY1R	-3.5082364
NPY2R	-2.9882371
NPY4R	-3.396518
NR0B2	-3.1594348
NR2F1	-2.3201497
NR2F2	-1.2224548
NR3C2	-4.786118
NR4A2	-4.3390083
NR5A1	-1.0642865
NRADD	-2.7385979
NRAP	-0.8898282
NRARP	-3.0086224
NRCAM	-5.355447
NRD1	-1.242692
NREP	-5.662155
NRG2	-2.7541578
NRGN	-3.0961838
NRIP2	-2.9172595
NRL	-4.990777

NRM	-1.1938822
NRP	-4.81738
NRSN1	-3.0479865
NRTN	-0.4838917
NRXN2	-1.7576151
NSG1	-3.8844268
NSG2	-4.9625797
NSL1	-0.493685
NSUN7	-2.313057
NT5C1A	-2.4821503
NT5DC2	-2.0539355
NTF5	-3.4768534
NTHL1	-3.7684486
NTM	-2.6704879
NTN1	-2.0204666
NTN4	-3.5582252
NTN5	-3.0955994
NTNG1	-4.9911966
NTNG2	-0.6750443
NTRK1	-5.92498
NTRK2	-2.7078545
NTRK3	-1.9271
NTSR1	-3.915521
NUAK1	-3.2303853
NUDT10	-2.821948
NUDT12OS	-2.9524329
NUDT15	-2.2808897
NUDT17	-3.8525925
NUDT18	-0.8962262
NUF2	-1.4167833
NUGGC	-1.4863806
NUMB	-0.5451171
NUMBL	-1.1929042
NUPR1L	-2.7431998
NUSAP1	-1.5837364
NWD1	-1.1555376
NWD2	-2.0293903
NXF2	-1.8640382
NXNL1	-1.2113972
NXNL2	-0.9892864
NXPE2	-1.1296725
NXPE3	-1.6098213
NXPE4	-4.181183
NXPH1	-2.8308764
NXPH3	-3.9854145

NYAP1	-1.3828965
NYAP2	-3.6758852
NYNRIN	-4.7641315
OAS1C	-1.7406096
OAS1D	-2.8526688
OAS1E	-1.0896764
OAS1G	-1.0159893
OAS1H	-3.4164581
OAZ2	-0.5560319
OAZ3	-6.454608
OBOX3	-2.7249286
OBOX5	-4.0356026
OBP1A	-1.4981816
OBP2B	-3.3323922
OBSCN	-3.1826758
OBSL1	-3.1886039
OCIAD2	-2.0997725
OCM	-3.5321362
ODF1	-3.4021568
ODF3	-2.8772564
ODF3B	-1.1620977
ODF3L2	-3.1671414
ODF4	-3.6960573
OFCC1	-3.3972678
OGDHL	-3.3075
OGN	-9.238321
OIP5	-3.7532477
OIT1	-3.6060696
OLAH	-6.073149
OLFM1	-1.8974266
OLFM3	-2.9005601
OLFML1	-3.12277
OLFML2A	-0.7898011
OLFML2B	-1.2703397
OLFML3	-3.1937933
OLFR1	-2.7062008
OLFR10	-2.8481805
OLFR100	-2.2247794
OLFR1000	-3.0175111
OLFR1006	-4.0657883
OLFR1009	-4.2142763
OLFR1010	-2.9056573
OLFR1012	-4.0762563
OLFR1013	-2.8898637
OLFR1014	-2.5638092

OLFR1015	-2.230826
OLFR1018	-2.577928
OLFR1019	-3.8797932
OLFR1024	-2.627102
OLFR1028	-2.7575688
OLFR1030	-4.485518
OLFR1031	-2.4118347
OLFR1032	-3.90897
OLFR1033	-5.6702914
OLFR1039	-3.605436
OLFR1040	-4.927762
OLFR1042	-3.9745135
OLFR1043	-2.4085746
OLFR1045	-2.5351427
OLFR1046	-4.3382373
OLFR104-PS	-4.0112133
OLFR1051	-3.5047815
OLFR1052	-4.5350513
OLFR1054	-4.781423
OLFR1055	-5.4592457
OLFR1056	-3.6884985
OLFR1057	-3.4625077
OLFR105-PS	-4.3205624
OLFR1061	-3.413019
OLFR1062	-2.954939
OLFR1065	-3.6978807
OLFR107	-4.266387
OLFR1082	-2.192444
OLFR1085	-2.6001873
OLFR1086	-2.8429205
OLFR1089	-4.4405785
OLFR1090	-3.2070718
OLFR1097	-1.8454347
OLFR11	-2.454783
OLFR110	-5.566543
OLFR1100	-5.471755
OLFR1101	-3.443006
OLFR1102	-2.8583837
OLFR1107	-5.371327
OLFR111	-4.136386
OLFR1110	-6.9394817
OLFR1111	-5.101495
OLFR1112	-3.6092083
OLFR1115	-1.8796058
OLFR1120	-1.4064655

OLFR1121	-1.7333665
OLFR1122	-1.0232744
OLFR1123	-3.8699
OLFR1124	-4.908984
OLFR1131	-1.9669166
OLFR1135	-3.9078813
OLFR1136	-5.6039543
OLFR1137	-4.330134
OLFR1138	-3.3358498
OLFR114	-3.7898948
OLFR1140	-4.434682
OLFR1141	-3.1677623
OLFR1145	-4.850323
OLFR1152	-4.666602
OLFR1153	-3.094347
OLFR1155	-3.286317
OLFR1156	-3.5294971
OLFR1157	-4.9734764
OLFR1158	-3.673253
OLFR1162	-4.1832895
OLFR1164	-3.2852342
OLFR1168	-1.1557355
OLFR1170	-4.3090267
OLFR1173	-4.8307524
OLFR1178	-3.306279
OLFR1179	-5.8586907
OLFR118	-3.0744956
OLFR1180	-0.7322609
OLFR1181	-3.7600336
OLFR1184	-2.8820486
OLFR1189	-4.2941527
OLFR1193	-5.6670904
OLFR12	-2.1827672
OLFR1200	-4.0880337
OLFR1202	-4.9232025
OLFR1206	-4.537388
OLFR1214	-4.017928
OLFR1215	-3.4362588
OLFR122	-4.153159
OLFR1220	-2.3489327
OLFR1226	-5.0256033
OLFR1231	-3.7246385
OLFR1238	-3.9334455
OLFR124	-3.1439967
OLFR1241	-2.3693564

OLFR1243	-2.4946365
OLFR1245	-4.1239247
OLFR1248	-3.2521985
OLFR1249	-1.0159361
OLFR1250	-1.3734741
OLFR1252	-0.9830203
OLFR1254	-6.5841103
OLFR1256	-2.3671389
OLFR1257	-3.4478579
OLFR1258	-2.8941479
OLFR1259	-3.7952425
OLFR126	-3.3846385
OLFR1261	-4.8312244
OLFR1262	-4.9805527
OLFR1263	-3.1436388
OLFR1264	-2.6305594
OLFR1265	-2.4112837
OLFR1271	-3.38732
OLFR1272	-3.8495288
OLFR1275	-4.9573984
OLFR1276	-1.9138415
OLFR1277	-2.9350173
OLFR1278	-2.9983478
OLFR1279	-2.6877
OLFR1280	-2.3616798
OLFR1286	-3.6784382
OLFR1287	-2.8372145
OLFR1288	-4.11409
OLFR1289	-4.978426
OLFR129	-6.14574
OLFR1297	-3.6808844
OLFR13	-2.656615
OLFR130	-2.7390764
OLFR1300-PS	-3.470248
OLFR1302	-5.1218085
OLFR1303	-3.5324128
OLFR1308	-2.6779058
OLFR131	-3.3963952
OLFR1310	-1.7276037
OLFR1311	-4.609725
OLFR1312	-1.045558
OLFR1313	-4.069194
OLFR1314	-4.7790394
OLFR1316	-1.8254151
OLFR132	-5.8617544

OLFR1320	-3.1565194
OLFR1321	-3.2153938
OLFR1322	-4.2053165
OLFR1323	-3.7047946
OLFR1324	-2.3370554
OLFR1328	-4.1198
OLFR133	-5.049382
OLFR1330	-4.660391
OLFR1333	-8.300614
OLFR1336	-4.794936
OLFR1337	-4.1866064
OLFR1338	-3.031805
OLFR1339	-3.6462533
OLFR1340	-3.9183066
OLFR1341	-3.7176728
OLFR1342	-4.8703275
OLFR1344	-4.492363
OLFR1347	-1.6871514
OLFR1348	-4.939326
OLFR135	-3.709955
OLFR1350	-2.4896455
OLFR1351	-4.96976
OLFR1352	-3.3396451
OLFR1358	-3.2930107
OLFR1361	-2.7782178
OLFR1362	-5.0731983
OLFR1364	-3.9607844
OLFR137	-3.780581
OLFR1370	-2.333606
OLFR1377	-2.9988205
OLFR1378	-4.2318654
OLFR1382	-2.6389897
OLFR1383	-4.10979
OLFR1385	-3.7739267
OLFR1393	-2.053807
OLFR1396	-2.023353
OLFR1410	-0.6181855
OLFR1411	-3.770899
OLFR1414	-3.808276
OLFR1415	-5.2268963
OLFR1417	-1.942409
OLFR1418	-3.7057843
OLFR1419	-2.3430548
OLFR142	-2.513451
OLFR1420	-3.1739516

OLFR1423	-4.4387035
OLFR1425	-3.9877439
OLFR1427	-2.3943589
OLFR1433	-4.661951
OLFR1436	-1.8352404
OLFR1441	-4.804657
OLFR1445	-4.1975965
OLFR1446	-2.0579138
OLFR1448	-4.701627
OLFR145	-2.8671815
OLFR1450	-2.0892162
OLFR1451	-6.7152824
OLFR1454	-5.903279
OLFR1459	-3.8558874
OLFR146	-3.0620031
OLFR1461	-5.1573606
OLFR1462	-2.7785342
OLFR1465	-3.9544032
OLFR147	-4.7931004
OLFR1474	-2.877111
OLFR1490	-6.1882315
OLFR1496	-3.1823049
OLFR1497	-3.3825727
OLFR150	-3.945861
OLFR1500	-6.6003313
OLFR1501	-3.6467216
OLFR1502	-2.6106033
OLFR1505	-0.6677227
OLFR1507	-2.2029853
OLFR1508	-4.7554493
OLFR1509	-1.9350183
OLFR1511	-3.1441123
OLFR1513	-5.1486588
OLFR152	-4.592226
OLFR1532-PS	-3.8392982
OLFR1537	-1.9938388
OLFR155	-2.684548
OLFR156	-3.5702052
OLFR157	-3.7801912
OLFR160	-5.193261
OLFR161	-5.561241
OLFR17	-4.479355
OLFR170	-1.8016198
OLFR176	-2.4781585
OLFR178	-3.6648939

OLFR18	-4.016407
OLFR181	-2.3465176
OLFR183	-3.299775
OLFR186	-4.2517657
OLFR187	-2.9784362
OLFR19	-3.7000904
OLFR190	-3.9268951
OLFR194	-2.1370533
OLFR195	-2.9535415
OLFR196	-4.213356
OLFR198	-1.24091
OLFR20	-3.8531046
OLFR204	-4.6703687
OLFR207	-4.010209
OLFR208	-5.2928505
OLFR209	-2.4728165
OLFR212	-2.6564775
OLFR213	-3.6005206
OLFR218	-3.2321153
OLFR220	-0.6506424
OLFR222	-3.7259557
OLFR223	-3.0147038
OLFR23	-3.700069
OLFR231	-2.8691993
OLFR237-PS1	-3.5987473
OLFR24	-3.7370524
OLFR247	-4.267728
OLFR248	-2.5147157
OLFR250	-5.4697113
OLFR259	-0.7404001
OLFR26	-3.3471403
OLFR270	-4.198057
OLFR272	-5.3615127
OLFR275	-4.9012027
OLFR279	-4.2228723
OLFR281	-6.0365276
OLFR282	-4.9101324
OLFR283	-6.0759716
OLFR288	-2.9061382
OLFR295	-2.9895551
OLFR297	-4.6607823
OLFR3	-1.9097857
OLFR30	-1.7494183
OLFR301	-2.5631363
OLFR303	-2.919994

OLFR304	-2.302512
OLFR305	-2.683084
OLFR307	-5.1415606
OLFR308	-4.8159285
OLFR309	-3.3292155
OLFR310	-4.6285825
OLFR312	-1.1853719
OLFR314	-3.6380706
OLFR317	-2.4797528
OLFR32	-5.3960752
OLFR323	-2.2906916
OLFR328	-3.753148
OLFR330	-2.1017222
OLFR331	-1.7900553
OLFR332	-3.6308808
OLFR338	-3.3810844
OLFR339	-3.2205484
OLFR340	-1.5492251
OLFR344	-3.7496877
OLFR345	-6.466036
OLFR346	-1.8350258
OLFR348	-4.4080844
OLFR351	-4.368497
OLFR352	-4.236847
OLFR354	-4.4931374
OLFR356	-5.09879
OLFR358	-2.7511437
OLFR362	-2.2008178
OLFR365	-3.5208008
OLFR367-PS	-2.5973606
OLFR368	-4.797859
OLFR374	-2.9710264
OLFR38	-5.0341187
OLFR380	-4.158773
OLFR382	-3.9370952
OLFR39	-5.5795584
OLFR390	-4.216611
OLFR391-PS	-3.3380816
OLFR392	-0.6810918
OLFR393	-2.2212062
OLFR398	-4.162875
OLFR401	-2.298485
OLFR402	-4.650916
OLFR406	-4.262723
OLFR417	-2.46902

OLFR418-PS1	-3.4012551
OLFR420	-3.4511347
OLFR421-PS1	-2.731514
OLFR426	-2.230571
OLFR427	-4.325964
OLFR429	-3.4173422
OLFR430	-4.2813783
OLFR432	-5.028296
OLFR433	-7.4909067
OLFR434	-2.3645728
OLFR441	-4.1007185
OLFR444	-2.6217668
OLFR446	-5.6422844
OLFR447	-3.9778814
OLFR453	-2.1342115
OLFR455	-2.9379585
OLFR456	-2.5425508
OLFR458	-5.0791826
OLFR46	-3.5659223
OLFR461	-4.3138947
OLFR462	-2.3732793
OLFR464	-3.0598805
OLFR466	-4.2715735
OLFR467	-3.1644244
OLFR469	-1.3822649
OLFR470	-2.3387575
OLFR477	-1.6135082
OLFR478	-3.1780484
OLFR479	-3.5483704
OLFR48	-2.170681
OLFR480	-4.357289
OLFR481	-5.497017
OLFR487	-4.4627547
OLFR49	-5.383282
OLFR490	-5.1089706
OLFR491	-3.561552
OLFR495	-4.209068
OLFR5	-3.1144605
OLFR50	-3.9408958
OLFR502	-3.1619484
OLFR508	-3.4327538
OLFR512	-3.8995981
OLFR513	-1.3800495
OLFR514	-5.239654
OLFR516	-2.314063

OLFR517	-2.76505
OLFR518	-4.6427975
OLFR52	-2.6636496
OLFR520	-3.7265577
OLFR524	-0.813103
OLFR538	-2.5333996
OLFR543	-4.0424967
OLFR544	-1.1041539
OLFR545	-4.3374233
OLFR547	-4.819867
OLFR550	-2.8339283
OLFR551	-4.9264374
OLFR552	-2.5443227
OLFR554	-6.2152276
OLFR555	-4.121518
OLFR557	-2.2994273
OLFR558	-3.3214931
OLFR559	-3.5892854
OLFR560	-2.4116907
OLFR569	-3.7238433
OLFR570	-3.3532176
OLFR571	-3.9670517
OLFR574	-4.4682107
OLFR576	-1.5084221
OLFR577	-2.8766227
OLFR578	-4.957819
OLFR58	-2.7564306
OLFR582	-6.0251584
OLFR584	-3.677151
OLFR59	-5.8306384
OLFR591	-2.772963
OLFR593	-5.005521
OLFR6	-3.9594345
OLFR60	-2.672064
OLFR601	-2.9330757
OLFR607	-3.419621
OLFR61	-3.5305107
OLFR616	-1.5940204
OLFR617	-4.189784
OLFR619	-4.829225
OLFR62	-3.5395749
OLFR620	-3.3781857
OLFR623	-3.070007
OLFR624	-2.344295
OLFR629	-2.970664

OLFR630	-3.041289
OLFR631	-2.4450219
OLFR632	-7.3736753
OLFR638	-1.9156268
OLFR639	-2.5743272
OLFR640	-3.247326
OLFR642	-4.67867
OLFR644	-2.665273
OLFR645	-5.867797
OLFR646	-3.648549
OLFR648	-3.9543715
OLFR649	-1.5012941
OLFR65	-2.9699888
OLFR651	-3.640846
OLFR652	-4.9411087
OLFR655	-4.674331
OLFR658	-3.066567
OLFR66	-5.3703723
OLFR661	-3.1607304
OLFR665	-2.6244605
OLFR666	-4.1573915
OLFR668	-3.6644094
OLFR67	-2.8392286
OLFR670	-7.0631704
OLFR671	-3.7447853
OLFR676	-4.106727
OLFR678	-5.105495
OLFR681	-2.1884546
OLFR683	-2.3390255
OLFR686	-2.6506033
OLFR688	-2.898287
OLFR691	-4.9610243
OLFR695	-3.364087
OLFR698	-5.153015
OLFR699	-4.845557
OLFR70	-2.9874601
OLFR700	-2.7291121
OLFR701	-4.8364005
OLFR702	-3.4691658
OLFR703	-1.6420498
OLFR707	-4.154587
OLFR71	-5.0783405
OLFR710	-3.135114
OLFR713	-2.921597
OLFR714	-4.913541

OLFR715	-3.6889746
OLFR720	-3.9974318
OLFR722	-4.7419043
OLFR723	-5.264268
OLFR724	-1.6898298
OLFR728	-1.3079019
OLFR729	-4.154849
OLFR738	-2.8673058
OLFR74	-1.1182277
OLFR741	-2.4213688
OLFR743	-4.9322367
OLFR749	-6.0989285
OLFR750	-3.07016
OLFR75-PS1	-1.7611468
OLFR76	-5.1822534
OLFR761	-3.584839
OLFR763	-2.4890528
OLFR764	-2.6707606
OLFR767	-4.174411
OLFR769	-2.4449687
OLFR770	-2.100423
OLFR776	-3.1838548
OLFR780	-3.0318794
OLFR784	-2.3426454
OLFR786	-2.6635141
OLFR787	-0.9065876
OLFR790	-3.2992766
OLFR798	-3.394766
OLFR802	-2.0390058
OLFR803	-2.6772828
OLFR804	-2.4840598
OLFR806	-4.4189262
OLFR815	-3.4655335
OLFR816	-3.9717784
OLFR819	-2.7444625
OLFR820	-3.086059
OLFR821	-2.1737173
OLFR823	-3.4245362
OLFR824	-3.1751494
OLFR827	-2.8049552
OLFR828	-6.067478
OLFR829	-3.986482
OLFR830	-6.5772214
OLFR836	-4.4544516
OLFR837	-4.5501366

OLFR843	-3.5277102
OLFR845	-2.967935
OLFR847	-3.1338768
OLFR849	-4.607146
OLFR850	-5.1185727
OLFR854	-4.784132
OLFR855	-2.6339712
OLFR866	-3.4215755
OLFR867	-2.8179178
OLFR870	-3.3428397
OLFR871	-3.3357425
OLFR873	-3.087327
OLFR874	-3.1195414
OLFR876	-3.3602588
OLFR877	-3.614657
OLFR887	-3.1654572
OLFR891	-3.7170396
OLFR895	-2.7108984
OLFR899	-3.4511821
OLFR9	-3.4110348
OLFR90	-2.8570893
OLFR901	-2.4065533
OLFR904	-4.0154467
OLFR905	-3.1884916
OLFR906	-4.848057
OLFR907	-5.2791986
OLFR910	-4.5682416
OLFR911-PS1	-2.6716094
OLFR913	-4.034998
OLFR914	-2.6990142
OLFR915	-2.572473
OLFR916	-1.6228068
OLFR917	-3.6391492
OLFR918	-4.430967
OLFR920	-2.554912
OLFR921	-4.140309
OLFR922	-6.1132007
OLFR923	-2.5982304
OLFR93	-2.1893132
OLFR930	-1.0781856
OLFR934	-2.667322
OLFR94	-3.4973805
OLFR945	-4.7105446
OLFR951	-3.0024023
OLFR954	-2.9671896

OLFR957	-5.473089
OLFR96	-0.8418617
OLFR961	-4.332322
OLFR963	-3.991998
OLFR969	-2.4788404
OLFR971	-3.7836683
OLFR974	-3.7090592
OLFR975	-5.7586823
OLFR976	-3.0063078
OLFR978	-2.6499164
OLFR979	-6.1132097
OLFR980	-5.671693
OLFR982	-3.9427981
OLFR985	-5.4407
OLFR986	-5.6443105
OLFR994	-2.590437
OLFR995	-3.365391
OLFR998	-4.2873583
OMD	-3.7460454
OMP	-1.5483761
OMT2A	-10.805268
OOEP	-3.7106385
OOG2	-3.6119447
OPN1MW	-2.687832
OPN4	-3.7061658
OPN5	-3.7775462
OPRD1	-4.467351
OPRK1	-4.942906
OPRL1	-3.0298831
ORAI1	-0.3679345
ORLY	-3.052369
OSBP2	-1.611732
OSBPL10	-1.0526402
OSBPL5	-3.0336206
OSBPL6	-3.705402
OSCP1	-1.3678164
OSM	-0.8628254
OSR1	-3.278952
OSTN	-3.8377662
OTOA	-5.2575603
OTOG	-3.3902822
OTOL1	-2.7599933
OTOP1	-1.4355483
OTOR	-2.749732
OTOS	-3.1471207

OTP	-4.2439246
OTUD6A	-4.151596
OTUD7B	-3.9550478
OTX2	-2.5214612
OVGP1	-2.3085034
OVOL1	-3.8230422
OXCT1	-0.8954976
OXCT2A	-1.8091037
OXT	-1.3060596
P2RX1	-1.4475584
P2RX3	-0.7739284
P2RX5	-3.1448033
P2RX6	-4.211773
P2RX7	-0.638196
P2RY1	-0.4625433
P2RY4	-4.7168283
P4HA3	-4.5388813
P4HTM	-3.1130111
PABPC1L2B-I	-2.1582415
PACRG	-1.7029305
PADI2	-3.1504836
PADI3	-3.106358
PADI4	-2.1710334
PADI6	-3.0543466
PAG1	-1.7949853
PAK1	-0.967993
PAK3	-2.4058638
PAK6	-4.999773
PAK7	-1.4630721
PALB2	-1.990514
PALD1	-0.4605811
PALM	-1.3425252
PALMD	-0.5357595
PAM	-1.4302831
PAMR1	-1.6881287
PANCT2	-2.4103863
PANX2	-2.7211916
PAPL	-2.2783802
PAPOLB	-3.0940323
PAPPA	-0.9511573
PAPPA2	-2.1458297
PAPSS1	-1.427767
PAQR4	-2.118998
PAQR6	-3.9677744
PAQR7	-0.2949975

PAQR8	-2.876232
PARD3B	-4.2502465
PARD3BOS1	-4.5002584
PARD6B	-1.697839
PARD6G	-1.991616
PARM1	-1.9457712
PARPBP	-2.8903584
PARVB	-2.281055
PASK	-1.6406679
PATZ1	-2.6253085
PAX2	-4.008635
PAX3	-2.3205414
PAX4	-3.9482226
PAX5	-3.3037364
PAX8	-3.976648
PBK	-2.1795132
PBSN	-2.8062356
PBX4	-5.259904
PCBP3	-0.9270129
PCDH10	-5.3035307
PCDH11X	-3.2144263
PCDH12	-2.6271977
PCDH15	-3.2491846
PCDH17	-4.304936
PCDH18	-4.7437277
PCDH19	-3.7794528
PCDH7	-1.1883512
PCDH9	-2.7936177
PCDHA11	-4.0066304
PCDHA12	-3.3350608
PCDHA9	-2.4554489
PCDHAC1	-4.2775736
PCDHAC2	-4.997778
PCDHB1	-3.0784569
PCDHB10	-3.4241612
PCDHB12	-2.1643574
PCDHB14	-1.6703253
PCDHB15	-3.0303037
PCDHB16	-2.126175
PCDHB17	-1.7016346
PCDHB18	-1.3389406
PCDHB19	-3.3876321
PCDHB2	-2.7320175
PCDHB20	-2.5624
PCDHB21	-1.2844906

PCDHB3	-2.734053
PCDHB6	-2.6402142
PCDHB7	-2.5262263
PCDHB8	-2.8125784
PCDHGA10	-1.7029964
PCDHGA5	-2.4060702
PCDHGA9	-1.1666396
PCDHGB1	-2.0659132
PCDHGC5	-0.8166337
PCED1B	-0.5437009
PCK2	-1.0402033
PCLO	-3.4959943
PCNX	-1.7317841
PCNXL2	-2.7976367
PCOLCE	-2.8512886
PCOLCE2	-4.787314
PCP4L1	-1.4371078
PCSK1	-0.652602
PCSK1N	-2.5842001
PCSK2	-3.1616783
PCYOX1L	-2.1041136
PCYT1B	-2.2841012
PDCD1	-0.7215135
PDCL2	-3.9193544
PDDC1	-0.5113773
PDE10A	-2.6949806
PDE11A	-3.583755
PDE12	-1.3580406
PDE1C	-4.936962
PDE3A	-2.8466845
PDE4A	-1.7407415
PDE4D	-1.9324219
PDE5A	-3.1372678
PDE6C	-3.7847931
PDE6G	-1.5120189
PDE7A	-1.0108523
PDE8B	-5.0303345
PDE9A	-1.3978276
PDGFB	-4.148877
PDGFD	-3.977308
PDGFRA	-2.0253656
PDGFRB	-1.1037819
PDGFRL	-2.2126942
PDHA2	-4.662354
PDIA2	-4.4712753

PDIK1L	-1.2944429
PDLIM2	-3.6144483
PDLIM3	-4.920581
PDPN	-4.921707
PDXP	-1.979975
PDZD7	-3.1871665
PDZD9	-4.4635916
PDZK1IP1	-1.777761
PDZRN4	-4.0866914
PEA15B	-2.734206
PEAK1	-0.7670343
PEAR1	-0.5465105
PECAM1	-1.3196194
PEG12	-2.1514742
PEG13	-1.5101106
PELI3	-1.5509844
PENK	-3.652049
PER3	-2.802474
PERM1	-2.5615022
PET100	-1.0466976
PEX5L	-1.6284089
PF4	-0.9638083
PFKFB4	-0.8082418
PFKM	-1.042073
PFN3	-3.8892152
PFN4	-3.9631836
PFPL	-3.1565773
PGA5	-3.3282864
PGAM2	-4.873536
PGAP1	-0.9854488
PGBD1	-3.719837
PGBD5	-2.6168406
PGC	-1.815301
PGK2	-3.3420494
PGLYRP1	-0.8155322
PGLYRP3	-0.6606338
PGM2L1	-0.7043088
PGM5	-3.2637563
PGPEP1L	-5.3438015
PHACTR1	-4.226072
PHACTR2	-2.085115
PHEX	-2.3741956
PHF20L1	-0.6681259
PHF21B	-1.8233554
PHGDH	-0.5783684

PHGR1	-4.107816
PHLDA3	-0.24524
PHLDB1	-2.3548627
PHLDB2	-1.2904251
PHOSPHO1	-0.3655679
PHOX2A	-2.5486941
PHTF1	-0.2973816
PHTF1OS	-3.7096539
PHYHIP	-5.9018655
PI16	-4.373614
PIANP	-1.0805933
PIBF1	-2.1847234
PIGU	-0.8587058
PIGW	-0.801281
PIGZ	-0.4584851
PIK3C2B	-3.3524055
PIK3CD	-0.5333345
PIK3IP1	-1.8964736
PIK3R3	-1.6309462
PIKFYVE	-2.0389905
PILRB1	-2.3112943
PINC	-3.323091
PINLYP	-1.0576086
PIP	-3.127431
PIP5K1BOS	-3.5529506
PIP5KL1	-3.4557993
PIRT	-1.0231881
PISD-PS2	-2.9656408
PITPNM2	-0.2634351
PITPNM3	-4.2670593
PITX3	-3.3001118
PIWIL2	-1.9327216
PIWIL4	-2.5362878
PKD1L1	-2.4298413
PKD2L1	-6.3652124
PKDREJ	-2.954785
PKHD1	-1.8034241
PKHD1L1	-2.352359
PKIA	-3.1261768
PKN3	-2.945775
PKNOX2	-5.789839
PKP4	-0.724653
PLA2G1B	-4.5824523
PLA2G2A	-2.5498316
PLA2G2C	-1.9413233

PLA2G2D	-2.3142037
PLA2G2E	-2.249686
PLA2G2F	-3.7288294
PLA2G4D	-3.0384834
PLA2G4E	-2.3000553
PLA2G4F	-3.2837994
PLA2R1	-3.8015609
PLAC1	-3.2405987
PLAC9A	-2.1306713
PLAG1	-4.0009766
PLAGL1	-2.0603845
PLAT	-2.1248837
PLAU	-2.818042
PLB1	-4.407584
PLCB2	-2.4047027
PLCB3	-0.4650743
PLCB4	-0.6917069
PLCD1	-0.8784816
PLCD3	-2.078967
PLCG1	-0.5402224
PLCH1	-2.348596
PLCH2	-3.984087
PLD4	-2.0016227
PLD5	-0.8555863
PLD6	-4.274272
PLDI	-5.2980046
PLEKHA1	-0.3534133
PLEKHA2	-1.5322104
PLEKHB1	-1.9668734
PLEKHD1	-5.4789486
PLEKHD1OS	-2.7217894
PLEKHF1	-0.3764553
PLEKHG1	-3.0136127
PLEKHH3	-0.8402321
PLEKHN1	-4.623252
PLEKHO1	-0.366627
PLEKHO2	-0.5389903
PLET1	-2.056988
PLIN1	-2.07865
PLK1	-0.5496261
PLK2	-0.692919
PLK4	-3.904111
PLLP	-3.1153736
PLOD2	-1.6431692
PLP2	-2.4532292

PLS1	-1.909631
PLSCR3	-0.3417494
PLSCR4	-3.6612742
PLTP	-0.6875327
PLVAP	-0.4965417
PLXDC1	-2.9838603
PLXDC2	-4.455303
PLXNA3	-2.6090672
PLXNA4	-2.8318582
PLXNB3	-3.1266844
PMEL	-1.6857674
PMEPA1	-3.0361109
PMIS2	-5.6623726
PMM1	-0.367106
PMP22	-3.1812134
PNCK	-5.1432133
PNLIP	-1.6938322
PNLIPRP1	-1.8494611
PNLIPRP2	-1.9755592
PNMA1	-2.304477
PNMA3	-2.1351633
PNMAL1	-3.5399127
PNMAL2	-2.9655635
PNPLA1	-1.4756565
PNPLA3	-2.6296272
POC1A	-1.9009519
PODN	-4.604445
PODNL1	-3.5674784
PODXL	-4.1510844
PODXL2	-3.651205
POF1B	-3.4016738
POLD1	-1.333709
POLE	-1.3956113
POLI	-0.8122003
POLK	-0.5334151
POLQ	-2.8277116
POLR2A	-0.8482094
POLR3F	-1.3225219
POLR3H	-2.86563
POM121L12	-5.9410453
POM121L2	-2.5101657
POMC	-0.4081616
POPDC2	-1.6881781
POPDC3	-4.389057
POSTN	-2.8543005

POTEG	-4.0344095
POU2AF1	-1.887197
POU2F3	-1.8604205
POU3F1	-2.632001
POU3F2	-5.056535
POU3F4	-1.3058631
POU4F3	-1.9549985
POU5F1	-2.2617373
POU6F2	-0.5213521
PPAPDC1A	-3.3165653
PPBP	-3.3263972
PPEF1	-2.6697752
PPEF2	-3.7937548
PPFIA2	-2.4054513
PPFIA4	-2.4013383
PPIC	-1.8697722
PPIE	-0.8603468
PPIL2	-0.7020242
PPIL6	-2.0857646
PPL	-0.3710773
PPM1J	-0.905638
PPM1M	-0.5731843
PPP1R12B	-2.210868
PPP1R13B	-0.3010056
PPP1R14A	-4.207221
PPP1R14D	-4.3389864
PPP1R16B	-4.802286
PPP1R18	-2.8807003
PPP1R1B	-2.5823956
PPP1R26	-3.5386963
PPP1R27	-0.4108522
PPP1R2-PS9	-1.4690661
PPP1R32	-2.3845105
PPP1R36	-3.5447934
PPP1R3C	-0.6759257
PPP1R3D	-0.8928642
PPP1R3F	-2.2223382
PPP1R8	-0.5153644
PPP1R9A	-0.8676758
PPP1R9B	-0.7317832
PPP2R2B	-2.6264853
PPP2R2C	-3.6856527
PPP2R3A	-2.6397731
PPP3R2	-2.4585092
PPY	-4.2411556

PQLC3	-2.4849372
PRAMEF12OS	-2.4497206
PRAMEL	-4.380892
PRAMEL1	-2.6243901
PRAMEL7	-6.6335564
PRAP1	-4.1184464
PRB1	-4.3035946
PRC1	-1.2892413
PRDM1	-0.5856099
PRDM10	-0.7515791
PRDM11	-2.050859
PRDM12	-1.587733
PRDM15	-3.488546
PRDM16	-3.2609
PRDM5	-2.1524167
PRDM8	-3.7999346
PRDM9	-1.432436
PRDX6	-1.0881145
PRELP	-2.9003277
PREX2	-4.878129
PRF1	-0.8277667
PRG2	-4.37074
PRG3	-2.8113496
PRH1	-0.8841667
PRICKLE1	-1.8695328
PRICKLE2	-5.8090897
PRICKLE4	-4.561343
PRIM2	-1.374228
PRKAB1	-1.7789116
PRKAG2OS1	-3.7444365
PRKAG3	-3.7855468
PRKAR1B	-2.3052728
PRKAR2B	-2.2717633
PRKCA	-1.2920756
PRKCDBP	-2.9422917
PRKCH	-1.8094308
PRKCI	-0.837435
PRKCQ	-1.0938756
PRKD1	-3.7595022
PRKG1	-3.4366632
PRKG2	-5.303439
PRL2B1	-4.3473673
PRL2C1	-3.9979649
PRL2C5	-1.6518757
PRL3A1	-5.6622934

PRL3B1	-2.5428205
PRL3C1	-2.538814
PRL3D1	-2.7659254
PRL4A1	-3.370675
PRL6A1	-2.4447637
PRL7A1	-4.7450066
PRL7C1	-2.4402964
PRL8A6	-1.886421
PRL8A8	-1.5458536
PRLH	-2.7307181
PRM2	-3.9156091
PRM3	-2.293046
PRMT2	-1.9288571
PRMT8	-5.7108345
PRN	-0.9481409
PROCA1	-5.3960457
PROKR1	-2.5701587
PROM1	-1.0124974
PROM2	-2.796719
PROP1	-3.422495
PROSER1	-2.3003206
PRP2	-2.0737176
PRPH	-5.656418
PRPH2	-1.8799059
PRPMP5	-3.3121245
PRPS1L1	-1.9324248
PRPS2	-1.3396652
PRR11	-1.0967808
PRR12	-2.1612074
PRR13	-0.6204488
PRR15	-1.2470615
PRR15L	-5.50858
PRR18	-5.7959156
PRR19	-5.070275
PRR3	-0.6673229
PRR30	-4.517122
PRR5L	-1.2435405
PRR7	-1.1365316
PRR9	-1.8152652
PRRC2B	-0.5932233
PRRG1	-1.5148706
PRRG4	-0.8397126
PRRT1	-1.9122949
PRRT2	-5.853476
PRRT4	-3.3603048

PRRX1	-1.7433698
PRRX2	-1.4149597
PRRXL1	-2.7746775
PRSS12	-5.8127565
PRSS16	-2.8841102
PRSS2	-3.6169083
PRSS22	-3.419889
PRSS23	-1.641684
PRSS23OS	-3.1434216
PRSS27	-2.4168677
PRSS28	-5.5193005
PRSS33	-2.5604725
PRSS34	-4.7509947
PRSS42	-5.359644
PRSS44	-3.424575
PRSS48	-0.3538091
PRSS50	-1.486824
PRSS52	-3.4166017
PRSS54	-6.5101004
PRSS56	-2.5361028
PRSS57	-0.9736636
PRSS58	-4.7327833
PRSS8	-1.24933
PRTN3	-2.6586494
PRUNE	-1.0204132
PRUNE2	-2.2796361
PRX	-6.772603
PSAPL1	-3.4812405
PSCA	-2.26211
PSD2	-2.8559427
PSD4	-1.4364877
PSG18	-3.5139117
PSG19	-3.1898222
PSG21	-4.1986256
PSG25	-3.8734455
PSG27	-1.7672443
PSG28	-3.2962646
PSMA8	-0.7002802
PSMB11	-4.1563034
PSMC3IP	-4.7224264
PSTK	-0.4520729
PSTPIP1	-1.1392808
PTAFR	-1.2091768
PTCD1	-0.7554569
PTCH1	-1.7488027

PTCHD2	-2.8048725
PTCRA	-5.222562
PTF1A	-4.689329
PTGDR2	-5.287161
PTGDS	-0.3311579
PTGER1	-2.3379807
PTGER2	-0.5107117
PTGES3L	-0.5114918
PTGFR	-2.6984155
PTGFRN	-2.8048434
PTGIS	-3.4824302
PTGR1	-2.2830904
PTGS1	-0.9443672
PTH1R	-0.4328601
PTH2	-1.9661126
PTHLH	-3.0186489
PTK2B	-0.7910297
PTN	-5.1992216
PTP4A3	-1.8841007
PTPLAD2	-3.621326
PTPN13	-1.6436341
PTPN18	-1.7298439
PTPN20	-5.033429
PTPN7	-1.6101906
PTPRB	-2.734308
PTPRC	-0.5122116
PTPRCAP	-1.0445287
PTPRE	-0.5131409
PTPRG	-0.3919745
PTPRM	-3.2053742
PTPRN	-4.22484
PTPRN2	-1.4920895
PTPRO	-2.8361502
PTPRQ	-3.8509445
PTPRR	-2.6290045
PTPRS	-3.2301672
PTPRU	-3.832976
PTPRV	-2.431461
PTPRZ1	-3.3217704
PTRF	-3.8509648
PTTG1	-0.4841158
PVALB	-1.9268818
PVRL3	-1.6524374
PVRL4	-1.3201516
PVT1	-0.8356357

PWWP2B	-1.2703378
PXDN	-2.9926052
PXT1	-4.414939
PYGB	-0.8639309
PYGM	-2.0751708
PYROXD1	-1.2477436
QK	-0.9183385
QRFP	-3.770862
QRFPR	-2.8699079
QTRT1	-2.2462342
R3HDM4	-0.375248
R3HDML	-2.6590395
R74862	-1.477035
RAB11FIP1	-1.6617527
RAB11FIP2	-6.4266067
RAB11FIP3	-0.6775982
RAB11FIP4OS	-4.874097
RAB11FIP5	-1.4890597
RAB15	-4.3182034
RAB19	-1.976156
RAB25	-4.3276224
RAB27A	-3.2789927
RAB27B	-4.4312563
RAB38	-0.5240505
RAB3A	-2.466708
RAB3B	-1.7274327
RAB3D	-2.1212294
RAB3IL1	-0.6336696
RAB40B	-2.975441
RAB44	-0.7351081
RAB6B	-4.7410297
RAB9B	-1.3851905
RABGAP1L	-1.504935
RACGAP1	-1.0997841
RAD51AP1	-2.4219525
RAD54L	-2.2912939
RAD9A	-0.8241558
RADIL	-4.0382795
RAET1A	-2.0244584
RAET1B	-1.0995777
RALYL	-1.9149554
RAMP1	-2.631972
RAMP2	-3.1697938
RAMP3	-0.3967619
RANBP17	-2.6554046

RANBP3L	-1.8836162
RAP1GAP2	-3.441936
RAPGEF3	-1.1377842
RARG	-2.5339277
RASA3	-0.9959948
RASAL2	-2.31607
RASEF	-4.283022
RASGEF1A	-7.571888
RASGEF1C	-4.0140495
RASGRF1	-2.4156754
RASGRF2	-3.5903392
RASGRP2	-1.125803
RASGRP3	-3.6955874
RASGRP4	-2.9716399
RASL10A	-4.9516296
RASL11A	-2.589026
RASL11B	-2.4672446
RASL12	-5.670597
RASSF9	-4.787284
RAVER2	-0.454437
RAX	-7.01558
RB1CC1	-3.9927747
RBAKDN	-3.6232352
RBFOX1	-2.82344
RBFOX3	-1.717408
RBL1	-0.5879443
RBM15B	-1.5802836
RBM20	-2.7866907
RBM24	-3.2436402
RBM38	-3.9116747
RBM46OS	-2.7299242
RBMS3	-2.3112786
RBMY	-2.1217408
RBP1	-0.7932775
RBP2	-3.5540164
RBP3	-0.2742288
RBP7	-5.2907953
RBPJ	-0.6768911
RBPJL	-5.3014526
RCAN3	-0.4355481
RCN3	-1.4471724
RCOR2	-3.683941
RCSD1	-2.1140935
RCVRN	-1.8941164
RDH18-PS	-3.2174802

RDH19	-3.271196
RDH8	-1.8087342
RDM1	-1.34356
REC8	-1.2680585
RECK	-2.918188
RECQL	-1.4241803
RECQL4	-1.8020344
REDRUM	-2.4966455
REEP1	-2.313813
REEP2	-3.977968
REEP5	-1.3912857
REG1	-2.9390597
REG2	-3.4695132
REG3A	-1.2312338
REL	-4.422238
REM1	-1.5376267
REM2	-3.0449314
REP15	-1.7655942
RESP18	-1.3549979
RETN	-4.297381
RETNLA	-10.271285
RETNLB	-1.8681254
RETNLG	-1.7562153
REX2	-1.6433754
RFC3	-0.8925819
RFESD	-1.5286574
RFFL	-0.5067895
RFPL4	-2.5581949
RFPL4B	-2.6301332
RFTN2	-4.91949
RFX2	-1.6015201
RFX4	-2.5341647
RFX7	-0.8569405
RGAG1	-5.9332943
RGAG4	-0.6097467
RGCC	-2.7657058
RGL2	-0.5267398
RGMA	-1.6878264
RGMB	-1.4143367
RGR	-3.3806624
RGS10	-1.135474
RGS12	-0.2989523
RGS13	-2.9752932
RGS14	-1.7862594
RGS17	-2.7485552

RGS18	-2.4666252
RGS21	-2.6110828
RGS22	-3.1405456
RGS3	-1.5634587
RGS6	-3.3784876
RGS7	-3.6616592
RGS7BP	-2.1676986
RGS8	-1.8120847
RGS9	-3.8439686
RGS9BP	-1.8076749
RHAG	-2.6879761
RHBDD1	-2.8887064
RHBDL2	-5.9851627
RHBDL3	-3.597326
RHCG	-4.4369364
RHD	-2.29269
RHOF	-1.3594227
RHOH	-2.3071449
RHOV	-2.136506
RHOX10	-4.9929705
RHOX11	-2.353248
RHOX12	-4.172882
RHOX13	-3.3854802
RHOX2C	-3.3244646
RHOX2E	-4.685556
RHOX2H	-3.552929
RHOX3E	-3.4060683
RHOX3F	-3.2722483
RHOX3G	-3.1512704
RHOX3H	-3.334724
RHOX4A	-6.2904634
RHOX4E	-4.2774506
RHOX4G	-4.817008
RHOX5	-0.3665102
RHOX7	-2.8489914
RHOX9	-1.4259651
RHPN1	-1.8752775
RHPN2	-0.9608061
RIBC2	-3.1032364
RIIAD1	-3.7784362
RIMBP3	-3.1473591
RIMS1	-5.2187195
RIMS2	-1.7291651
RIN1	-3.9736352
RIN2	-0.6086991

RIN3	-0.8670495
RINL	-2.5119398
RIPPLY1	-3.4229195
RIPPLY3	-3.3117816
RITA1	-0.7457185
RLBP1	-3.2312133
RLN1	-3.0112824
RLN3	-3.2005074
RLTPR	-0.6265476
RMI1	-0.3997967
RNASE1	-4.0449705
RNASE2A	-3.895213
RNASE2B	-0.7678959
RNASE6	-3.3338313
RNF112	-2.5850914
RNF122	-2.978698
RNF144A	-2.4898295
RNF150	-2.702804
RNF151	-5.964525
RNF165	-1.5027673
RNF180	-2.7753336
RNF186	-1.6228292
RNF207	-4.2930365
RNF208	-3.160699
RNF212	-3.2521687
RNF32	-4.085266
ROBO2	-3.3267896
ROR1	-2.8007617
ROR2	-1.8355517
RORB	-3.3435674
ROS1	-1.9663775
RPE65	-4.600025
RPGR	-1.2654016
RPH3A	-3.5506613
RPH3AL	-1.8057132
RPIA	-0.3816237
RPL10L	-3.0688896
RPP25	-5.574256
RPRD1A	-0.6397572
RPRM	-4.2750187
RPRML	-3.5153089
RPS6KA2	-3.552968
RPS6KA6	-1.5365849
RPTN	-7.028225
RPTOROS	-0.4926846

RPUSD1	-1.9204144
RRAD	-1.1103103
RRAGB	-1.3018351
RRAGD	-1.9811177
RRM2B	-1.7314293
RRP12	-1.3588662
RSF1	-1.0252542
RSPH1	-4.6844068
RSPH10B	-4.1143284
RSPH6A	-5.134808
RSPH9	-2.7552
RSPO1	-4.87467
RSPO4	-3.3644824
RSRC1	-1.6914787
RTBDN	-2.6882005
RTCA	-0.7609103
RTDR1	-3.3191793
RTKN	-0.8131783
RTKN2	-6.5114145
RTN1	-2.1442437
RTN2	-3.0175142
RTP2	-1.1953752
RTTN	-2.0339332
RUFY4	-2.4259415
RUNX1T1	-4.253458
RUNX2	-2.6064024
RUNX2OS1	-3.0573719
RUNX3	-1.0671378
RWDD2A	-4.624442
RXFP2	-1.6887779
RXFP3	-1.8261669
RYR1	-2.5325356
RYR2	-3.4457164
RYR3	-3.1446717
S100A14	-5.75198
S100A16	-0.3144066
S100A2	-3.432939
S100A3	-1.716402
S100A4	-2.1213229
S100A5	-1.035064
S100A6	-2.2071846
S100B	-2.7148771
S100G	-4.490803
S1PR3	-0.8258474
S1PR4	-3.0879364

S1PR5	-2.3261056
SAAL1	-0.5962732
SAG	-1.3003066
SALL2	-2.387023
SAMD11	-3.2773297
SAMD12	-3.574592
SAMD3	-3.1183383
SAMD4	-1.1571052
SAMD5	-2.2590063
SAMT2	-2.3117857
SAPCD1	-2.8053002
SARM1	-2.5806656
SASH3	-0.6796341
SASS6	-1.3869355
SATB2	-1.8870616
SATL1	-1.5945928
SAYSD1	-1.1893623
SBK1	-2.9261808
SBK2	-3.3672776
SBK3	-4.1079526
SBPL	-3.7013726
SBSN	-2.9331465
SCARF2	-2.58758
SCARLETLTR	-5.755546
SCD1	-0.7578623
SCD2	-2.611366
SCD3	-1.2324204
SCEL	-5.536871
SCG3	-2.6198862
SCGB1A1	-11.631142
SCGB1B19	-4.0878925
SCGB1B2	-5.2853646
SCGB1B20	-3.1463606
SCGB1B3	-2.2344582
SCGB1B30	-0.5922685
SCGB1B7	-3.281064
SCGB1C1	-5.4079876
SCGB2B20	-0.9674518
SCGB2B26	-3.5708253
SCGB2B27	-3.501042
SCGB3A1	-10.351069
SCGB3A2	-7.3821807
SCHIP1	-2.542669
SCIN	-2.4306836
SCML2	-6.339472

SCN1A	-2.1221235
SCN2A1	-2.6313999
SCN2B	-1.1877842
SCN3A	-3.2582932
SCN3B	-2.9560387
SCN4A	-1.5691404
SCN4B	-3.592453
SCN7A	-5.829031
SCNN1A	-4.566326
SCNN1B	-6.118347
SCP2D1	-3.1912897
SCRN1	-6.0275664
SCRT2	-1.1952174
SCT	-3.9029698
SCUBE1	-3.498005
SCUBE2	-5.3178034
SCUBE3	-3.6244955
SCX	-4.480941
SDC3	-0.4079664
SDHD	-2.017948
SDK1	-2.1864123
SDK2	-1.4893172
SDPR	-4.119483
SDR9C7	-4.2554893
SEC14L1	-1.2177896
SEC14L3	-7.793702
SEC14L5	-3.0994473
SEC22A	-2.346045
SEC22C	-2.586629
SELM	-1.5179005
SELPLG	-0.974469
SEMA3A	-2.7034657
SEMA3B	-5.1176176
SEMA3C	-7.2651186
SEMA3D	-2.534909
SEMA3E	-4.1948843
SEMA3F	-0.5820358
SEMA3G	-5.5244713
SEMA4D	-1.0148945
SEMA5A	-5.556715
SEMA5B	-2.7966118
SEMA6A	-2.1658628
SEMA6C	-2.0175352
SEMA7A	-2.6044843
SENP6	-1.124434

SERP2	-2.0204747
SERPINA1F	-2.0877926
SERPINA3A	-1.5401862
SERPINA3J	-2.5297964
SERPINA9	-4.1646323
SERPINB10	-3.5322917
SERPINB12	-2.7086482
SERPINB1C	-2.5653439
SERPINB3A	-4.761065
SERPINB3B	-4.914999
SERPINB3D	-4.707
SERPINB6A	-0.4713605
SERPINB6B	-2.0090778
SERPINB6C	-1.2439625
SERPINB6D	-4.9222193
SERPINB6E	-3.2591524
SERPINB9	-0.9848206
SERPINB9F	-2.5414567
SERPINE2	-4.257124
SERPINH1	-1.1720221
SERPINI1	-2.1863387
SERPINI2	-3.7557569
SESTD1	-1.1923993
SETBP1	-1.9942672
SETDB2	-1.0446012
SEZ6	-1.4597538
SEZ6L2	-1.150778
SF3B1	-1.1502559
SFMBT2	-1.2492476
SFRP2	-4.2557497
SFRP5	-2.7276883
SFTA2	-7.210865
SFTPA1	-9.34827
SFTPB	-10.74436
SFTPC	-12.476255
SFTPD	-3.8211005
SFXN3	-1.4093096
SFXN4	-5.507055
SFXN5	-2.8813012
SGCA	-5.6109495
SGCD	-4.801014
SGCE	-0.7923176
SGCG	-4.0503426
SGCZ	-2.31402
SGIP1	-3.6705422

SGOL1	-0.9405963
SGOL2	-1.3149273
SGTB	-3.8506284
SH2D1B1	-3.0743744
SH2D1B2	-5.3242946
SH2D2A	-3.440356
SH2D5	-3.4270306
SH2D6	-2.0229385
SH2D7	-3.9478862
SH3BGR	-3.889637
SH3BP1	-2.839321
SH3BP4	-4.707792
SH3D21	-1.8379278
SH3GL3	-2.4089866
SH3KBP1	-0.7164297
SH3RF1	-2.6439044
SH3RF2	-4.8297763
SH3RF3	-4.2585692
SH3TC1	-0.4560163
SH3TC2	-3.883348
SHANK1	-0.890465
SHANK2	-2.4402156
SHANK3	-0.2716062
SHBG	-5.486718
SHC2	-1.1011677
SHCBP1L	-4.200076
SHD	-4.353591
SHE	-1.567591
SHH	-2.830828
SHISA2	-3.3242931
SHISA4	-3.7093234
SHISA7	-3.431901
SHOX2	-2.9502685
SHROOM3	-5.199319
SHROOM4	-3.9491415
SIAH3	-2.66999
SIDT1	-1.5361583
SIGLEC15	-3.1381614
SIGLEC5	-4.0980806
SIPA1	-0.2412417
SIPA1L2	-1.0593874
SIPA1L3	-1.1502488
SIRPB1A	-2.367754
SIRPB1B	-2.1647284
SIRT6	-0.6994121

SIS	-3.7968776
SIT1	-1.9317217
SIVA1	-0.654495
SIX1	-3.6890311
SIX2	-2.8061304
SIX3	-3.6612163
SIX3OS1	-2.9515274
SIX4	-3.851761
SIX6	-2.0670645
SKA1	-0.7679336
SKA3	-0.4890366
SKAP1	-1.2642145
SKI	-1.5148602
SKIDA1	-3.7460284
SKINT1	-3.7715344
SKINT10	-1.3941143
SKINT11	-2.0554867
SKINT2	-2.6090398
SKINT4	-3.726702
SKINT5	-2.0513597
SKINT6	-2.69405
SKINT8	-3.642172
SKOR1	-2.4349928
SLA2	-1.0939357
SLAIN1	-1.5280836
SLC10A4	-4.1555986
SLC10A5	-1.1199522
SLC12A2	-1.3708179
SLC12A3	-5.0632544
SLC12A6	-0.5892773
SLC13A2	-4.978138
SLC13A4	-1.6749432
SLC14A2	-0.8395937
SLC16A1	-2.0417914
SLC16A11	-0.8771164
SLC16A8	-0.8397639
SLC16A9	-0.983721
SLC17A8	-1.4206855
SLC18B1	-1.4223478
SLC19A3	-2.9238734
SLC1A1	-3.0164304
SLC1A3	-4.3419123
SLC1A5	-1.4800968
SLC1A7	-3.7660089
SLC22A1	-0.908807

SLC22A12	-2.4597328
SLC22A14	-2.7306867
SLC22A17	-1.8759377
SLC22A2	-3.0090997
SLC22A20	-2.6081548
SLC22A22	-3.251623
SLC22A23	-0.5773909
SLC22A30	-2.7065756
SLC22A4	-2.163182
SLC24A1	-2.2484677
SLC24A3	-2.3613951
SLC24A4	-5.084895
SLC25A18	-3.2504222
SLC25A23	-2.4196494
SLC25A24	-0.8826673
SLC25A31	-2.9752274
SLC25A35	-2.767344
SLC25A36	-1.0680926
SLC25A4	-0.9033606
SLC25A43	-3.7748075
SLC25A48	-0.9930857
SLC26A10	-2.7846258
SLC26A4	-4.7327156
SLC26A5	-3.7437468
SLC26A9	-2.5983903
SLC27A1	-1.7448769
SLC27A3	-2.6610382
SLC27A6	-3.2957497
SLC28A1	-0.8151653
SLC28A3	-2.229229
SLC29A3	-0.9288054
SLC29A4	-3.5080566
SLC2A10	-3.3843637
SLC2A13	-3.5163424
SLC2A4	-4.244582
SLC2A7	-3.5455344
SLC30A2	-1.8157682
SLC32A1	-2.338449
SLC34A2	-7.44915
SLC35A5	-2.3652122
SLC35E4	-0.5899389
SLC35F3	-2.6895077
SLC35G2	-1.4942436
SLC35G3	-2.1896458
SLC36A1OS	-2.4014153

SLC36A2	-5.639025
SLC36A3	-2.159306
SLC37A2	-2.1840851
SLC38A10	-1.5893569
SLC38A11	-3.0862217
SLC38A5	-3.4524784
SLC38A9	-0.8118854
SLC39A5	-4.271451
SLC39A8	-1.3265145
SLC44A2	-1.4358766
SLC44A3	-3.4543219
SLC44A4	-2.4556956
SLC45A1	-1.0934436
SLC45A2	-3.4101954
SLC4A1	-0.6444306
SLC4A11	-1.4208913
SLC4A3	-2.7148657
SLC4A5	-3.1081657
SLC4A8	-2.6354904
SLC51A	-4.784477
SLC52A2	-0.7043593
SLC52A3	-3.4428115
SLC5A1	-2.732851
SLC5A10	-2.719633
SLC5A11	-3.4909663
SLC5A12	-4.326432
SLC5A2	-2.3759694
SLC5A3	-0.842623
SLC5A4A	-4.9014654
SLC5A8	-3.412466
SLC5A9	-3.6040154
SLC6A1	-4.23798
SLC6A14	-4.687848
SLC6A16	-3.1473336
SLC6A17	-4.0615015
SLC6A18	-3.2473917
SLC6A19OS	-2.7904766
SLC6A2	-5.166776
SLC6A20A	-2.1077414
SLC6A4	-3.1289964
SLC6A7	-1.4157372
SLC7A10	-6.434286
SLC7A13	-2.7162094
SLC7A14	-2.4343178
SLC7A15	-1.6903605

SLC7A6	-1.9728186
SLC8A1	-1.7310753
SLC8A2	-2.6207347
SLC9A2	-2.859381
SLC9A3R2	-3.6963472
SLC9A5	-2.028627
SLC9A9	-4.747818
SLC9B1	-5.009367
SLC9B2	-2.4660993
SLCO2A1	-1.0777252
SLCO4A1	-1.4538479
SLCO4C1	-4.1278763
SLCO5A1	-3.3611395
SLCO6C1	-3.0759032
SLIT1	-0.436301
SLIT2	-5.405078
SLIT3	-3.1956348
SLITRK3	-5.4706917
SLITRK4	-5.1083436
SLITRK5	-3.9034364
SLITRK6	-4.1140337
SLN	-7.62984
SLURP1	-3.6019454
SLX1B	-0.741941
SLY	-2.1760802
SMAD6	-2.4042156
SMAD7	-1.1885035
SMAD9	-2.7662446
SMARCA1	-1.6991189
SMARCA2	-0.920661
SMARCAL1	-0.4981401
SMARCD1	-1.4581425
SMARCD3	-4.0662766
SMC1B	-0.8651457
SMCO2	-0.8707945
SMCO3	-2.1510782
SMCP	-4.3912945
SMEK3-PS	-1.4298282
SMIM1	-2.011443
SMIM22	-0.6408441
SMIM24	-1.2724111
SMIM5	-4.313048
SMIM6	-1.3217893
SMOK3B	-1.2082582
SMOK4A	-4.3205557

SMPD5	-1.1950791
SMPX	-2.6638522
SMS	-0.2700613
SMTNL1	-3.3901088
SMTNL2	-2.0216835
SMURF1	-0.7034793
SMYD3	-0.7247198
SNAI1	-0.9614201
SNAI3	-3.8979867
SNCA	-1.1569307
SNCAIP	-1.3214061
SNCB	-2.3750563
SNCG	-4.0446815
SNED1	-2.7250822
SNHG11	-4.226285
SNHG18	-1.5673568
SNHG7OS	-1.2306981
SNHG8	-2.2791555
SNN	-2.9719493
SNORA47	-0.903297
SNTN	-2.839973
SNX22	-1.9800444
SNX25	-0.7388108
SNX29	-1.166856
SOAT1	-1.3419392
SOBP	-3.3215115
SOCS5	-1.1051314
SOGA1	-2.3790655
SOGA3	-4.4960284
SOHLH1	-3.3104115
SOHLH2	-0.488534
SORCS1	-3.8545585
SORCS2	-4.1989594
SORL1	-2.7720728
SORT1	-3.0311263
SOX1	-1.1525998
SOX12	-4.169686
SOX14	-2.3727512
SOX15	-1.7069416
SOX17	-4.327808
SOX18	-3.8092122
SOX2	-2.3998513
SOX21	-1.2736607
SOX3	-2.995965
SOX30	-7.2529683

SOX4	-2.5819635
SOX5OS1	-2.250911
SOX6OS	-2.259608
SOX7	-2.7606082
SP3OS	-0.8977842
SP5	-1.1626725
SP6	-1.7455766
SP9	-4.620749
SPA17	-1.8656583
SPACA1	-2.2030065
SPACA4	-2.1708772
SPACA7	-3.328219
SPAG1	-1.5396624
SPAG11B	-2.4559214
SPAG17	-2.4794512
SPAG17OS	-3.5119398
SPAG4	-3.4263105
SPAG5	-2.6279504
SPAG6	-4.5160403
SPAG8	-4.5370255
SPAM1	-2.5577638
SPARC	-0.4432762
SPARCL1	-5.912707
SPATA17	-2.6846783
SPATA18	-2.9421377
SPATA20	-3.5131226
SPATA21	-2.4792552
SPATA24	-1.7277243
SPATA2L	-1.8804269
SPATA31	-2.9245245
SPATA31D1A	-0.3690572
SPATA31D1B	-3.3441603
SPATA31D1D	-3.8680677
SPATA33	-6.280678
SPATA4	-4.8856645
SPATA45	-2.7687585
SPATA6	-0.9454746
SPATC1	-3.7137985
SPATS1	-1.2968926
SPC25	-0.5065935
SPDL1	-2.3864594
SPDYA	-2.5396538
SPDYB	-3.2435858
SPECC1	-0.6803567
SPEER1-PS1	-4.533758

SPEER2	-4.3065786
SPEER4A	-4.38488
SPEER4C	-1.2189326
SPEER4D	-5.629742
SPEER5-PS1	-5.0411983
SPEER8-PS1	-4.112174
SPEER9-PS1	-6.94818
SPEF1	-1.3981564
SPEF2	-0.9235129
SPEM1	-1.7714264
SPERT	-3.7295763
SPIB	-0.8786113
SPIN2D	-2.6114268
SPINK10	-0.5807462
SPINK13	-2.9674654
SPINK2	-3.8057497
SPINK4	-4.9334273
SPINK7	-2.3850229
SPINK8	-1.8937857
SPINT1	-4.3679113
SPINT5	-3.2523398
SPIRE1	-0.8557282
SPN	-2.447036
SPNS2	-1.4931414
SPNS3	-0.3976147
SPO11	-3.0573375
SPOCD1	-2.163884
SPOCK2	-6.725315
SPON1	-2.4327345
SPON2	-4.553386
SPPL2C	-3.2396038
SPRN	-1.8851213
SPRR1B	-3.9031394
SPRR2H	-6.3857145
SPRR2I	-2.51367
SPRR4	-4.0358543
SPRTN	-2.0570068
SPRY1	-3.3184278
SPRY2	-4.0948563
SPRY4	-2.0196338
SPSB1	-1.3414757
SPSB4	-1.9286051
SPTBN1	-1.4375165
SPTBN4	-3.433845
SPTBN5	-3.1313324

SPTSSB	-0.5420165
SRC	-4.3510017
SRCIN1	-2.9711378
SRCRB4D	-1.6201897
SRD5A2	-2.024191
SRGAP1	-1.6682329
SRGAP3	-3.5095313
SRL	-3.9835486
SRMS	-1.9096079
SRPK3	-3.092445
SRPX	-3.748335
SRRM3	-2.4837527
SRRM3OS	-3.9930735
SRRM4OS	-2.5961857
SRSF12	-3.3439972
SRY	-3.7695599
SSC5D	-4.2427454
SSMEM1	-2.0230567
SSPN	-4.0906982
SSPO	-2.6592948
SSTR1	-3.7283037
SSTR4	-0.4694493
SSTR5	-1.3746507
SSTY1	-2.7057617
SSU2	-3.2195518
SSXA1	-2.917395
SSXB1	-4.5006223
SSXB10	-4.7345657
SSXB6	-2.2385955
SSXB7	-3.145343
SSXB8	-2.5703044
SSXB9	-1.8646796
ST14	-2.1110053
ST18	-2.4226274
ST3GAL2	-2.1683373
ST3GAL6	-1.1131358
ST6GAL2	-2.1417356
ST6GALNAC1	-0.4102223
ST6GALNAC2	-3.3687406
ST6GALNAC3	-2.881633
ST8SIA2	-3.6989713
ST8SIA4	-3.2024746
ST8SIA6	-4.3586273
STAC2	-1.4502697
STAC3	-0.8329308

STARD13	-1.5751157
STARD6	-1.6480343
STARD8	-2.1854725
STAT4	-2.6830316
STC2	-4.171197
STEAP3	-3.039448
STIL	-2.1798139
STIM2	-2.0657158
STK32A	-3.0160444
STK32B	-12.766645
STK32C	-4.724153
STK33	-4.083964
STK36	-2.9607873
STMN1	-2.5428116
STMN2	-6.530366
STMN4	-4.320526
STMND1	-4.0416684
STOML3	-3.395558
STON1	-2.0740488
STON2	-1.586901
STOX1	-1.731684
STOX2	-1.5057178
STPG1	-2.444578
STRA8	-3.0412722
STRC	-4.8585076
STRIP2	-1.365156
STRM	-5.978245
STRN	-0.7481496
STX19	-2.8722808
STX1A	-2.3408682
STX6	-0.8668933
STXBP4	-1.3565524
STXBP5L	-2.8555787
STXBP6	-1.0545695
STYK1	-1.6726778
STYXL1	-3.1199965
SUFU	-1.1524756
SULF1	-3.0066624
SULF2	-1.6285105
SULT2A7	-4.0410204
SULT2B1	-2.098916
SULT5A1	-0.4715226
SULT6B1	-2.3326252
SUN1	-1.3678596
SUN3	-2.9507394

SUN5	-1.4909322
SUPT20	-1.0102897
SUPT3	-0.8571687
SUSD1	-3.1932611
SUSD3	-3.4553752
SUSD4	-6.050038
SUV39H1	-1.6108975
SV2C	-0.6092746
SVAL1	-3.8051455
SVEP1	-3.7475886
SVIL	-0.4725096
SVIP	-2.4382782
SVOP	-3.0566115
SVS2	-2.9879239
SVS3B	-2.1136672
SVS4	-2.6842427
SVS5	-4.0961113
SWAP70	-1.0169399
SYCE1L	-3.9562259
SYCN	-5.529434
SYCP3	-0.506475
SYDE1	-1.4925213
SYDE2	-3.5698895
SYN1	-2.6459544
SYN2	-1.3002665
SYN3	-1.2322447
SYNB	-0.9384809
SYNDIG1L	-4.237868
SYNE2	-0.6016958
SYNE3	-1.9934658
SYNGAP1	-3.3237727
SYNGR1	-4.5187783
SYNGR3	-2.6901264
SYNGR4	-2.8887713
SYNM	-3.1550946
SYNPO2	-2.5858538
SYNPR	-9.435177
SYT10	-4.6264324
SYT14	-4.102703
SYT15	-3.5984302
SYT16	-3.298195
SYT17	-3.4870732
SYT3	-2.9258437
SYT5	-3.678722
SYT6	-1.576241

SYT8	-4.2820654
SYTL1	-3.0398142
SYTL2	-3.4657607
SYTL3	-1.2699203
SYTL4	-2.8726315
SYTL5	-2.9986699
TAAR7A	-3.1522198
TAAR7F	-4.628046
TAAR8A	-4.068584
TACO1OS	-3.396057
TACR3	-2.6839867
TACSTD2	-4.1768236
TAF7	-0.3423572
TAF9B	-1.7268112
TAGLN	-1.3606689
TAGLN2	-0.3332069
TAGLN3	-3.3204124
TAL1	-1.0505674
TAMM41	-1.3554637
TANC2	-2.9476695
TAS1R1	-2.9188132
TAS1R2	-3.466458
TAS2R103	-2.7936747
TAS2R104	-3.391212
TAS2R106	-2.5436785
TAS2R110	-3.7697241
TAS2R118	-3.2598279
TAS2R120	-2.5712817
TAS2R121	-2.6171505
TAS2R124	-4.382312
TAS2R125	-2.9353123
TAS2R134	-4.821127
TAS2R135	-1.6370659
TAS2R138	-2.381431
TAS2R139	-2.9816444
TAS2R140	-4.986451
TBATA	-2.0083356
TBC1D10C	-1.1778622
TBC1D12	-0.6993566
TBC1D21	-0.7371888
TBC1D22B	-0.9750235
TBC1D30	-1.7591605
TBC1D31	-1.5533657
TBCE	-2.255798
TBL3	-1.7628524

TBR1	-4.102595
TBX1	-1.2397983
TBX10	-2.5065615
TBX15	-3.47263
TBX2	-3.9379656
TBX20	-3.1414225
TBX21	-1.2347791
TBX3	-2.0598147
TBX3OS1	-3.9534879
TBX4	-7.035715
TBX5	-6.7039056
TBX6	-0.9920313
TBXA2R	-3.570458
TBXAS1	-0.690105
TC2N	-4.306422
TCAM1	-1.3319173
TCAP	-6.52939
TCEA2	-2.189115
TCEA3	-3.2506037
TCEAL3	-1.9660878
TCEAL6	-4.4096384
TCEAL7	-0.7967539
TCEANC	-3.1630702
TCF15	-2.2030318
TCF19	-0.4154716
TCF21	-3.2643273
TCF23	-1.8991511
TCF4	-0.2792046
TCF7	-1.1877241
TCFL5	-3.9381747
TCHH	-2.5786982
TCL1	-3.1004615
TCL1B3	-2.8187761
TCL1B4	-1.8483262
TCL1B5	-3.8969693
TCP10B	-0.9201968
TCP10C	-3.6798525
TCP11	-5.078473
TCP11L1	-0.3437021
TCRA	-2.7354884
TCRB-J	-1.7094309
TCSTV3	-2.2831738
TCTE1	-1.9506028
TCTE2	-6.127515
TCTEX1D4	-4.01898

TCTN2	-0.5843651
TDGF1	-1.270401
TDH	-1.6344142
TDPOZ4	-0.8613462
TDRD1	-3.9660897
TDRD5	-2.3058457
TEAD2	-1.1154878
TECPR1	-0.6595509
TECRL	-3.4048333
TEK	-3.6287453
TEKT2	-4.2351475
TEKT3	-2.3473516
TEKT4	-2.6257942
TEKT5	-3.7466893
TEN1	-0.4789467
TENM2	-4.535871
TESC	-2.1350043
TESCL	-3.0372927
TESPA1	-1.8917991
TET1	-2.4527035
TET3	-2.2303448
TEX101	-5.566887
TEX13	-3.5522082
TEX13A	-3.4403524
TEX15	-3.2858129
TEX19.2	-3.252523
TEX21	-3.945744
TEX22	-2.664241
TEX24	-3.1879241
TEX28	-1.1410487
TEX33	-4.711462
TEX36	-4.7965064
TEX37	-4.5241976
TEX38	-3.471029
TEX43	-3.5143147
TEX9	-0.5842862
TFAP2A	-2.6420016
TFAP2C	-1.6209695
TFAP2D	-2.272397
TFAP4	-0.7559767
TFCP2L1	-1.1829934
TFDP2	-1.2151985
TFEB	-1.0323803
TFF2	-4.2991343
TG	-4.0635457

TGFB1I1	-2.1577513
TGFB2	-4.157117
TGFB3	-2.3253376
TGFBR2	-0.94012
TGIF2LX1	-2.3977551
TGIF2LX2	-3.8704698
TGM4	-3.4471965
TGM6	-0.547292
TGM7	-5.180376
TH	-1.3427143
THADA	-1.6137428
THBD	-2.5943391
THBS3	-4.320559
THEG	-3.276219
THEM5	-1.0286379
THEM6	-1.6413884
THEMIS	-3.7170396
THEMIS3	-2.8544428
THNSL1	-2.3887196
THRA	-1.4139965
THSD4	-3.0478551
THSD7A	-1.4328642
THY1	-1.3589356
TIA1	-9.097546
TIAM1	-1.9779589
TIAM2	-3.2307267
TIE1	-2.7057319
TIMM8A2	-1.5700557
TIMP2	-4.2380104
TIMP3	-1.088505
TIMP4	-2.4325335
TINAG	-5.486599
TK1	-0.5452402
TKTL2	-2.921944
TLCD2	-0.5759285
TLE2	-1.5451112
TLE6	-0.9530118
TLL1	-2.3532932
TLR11	-3.9785774
TLR12	-0.7442615
TLR4	-0.5360222
TLR5	-1.3554497
TLR8	-2.2274761
TLX1	-2.5120635
TLX3	-2.8902955

TM4SF1	-0.4026582
TMC1	-3.286765
TMC2	-4.037284
TMC3	-0.6214327
TMC4	-3.4338787
TMC5	-1.8100653
TMCC2	-3.1717405
TMCC3	-0.7155826
TMCO5B	-3.0807672
TMED11	-3.1859696
TMEFF1	-4.904261
TMEFF2	-2.8461826
TMEM100	-6.1728454
TMEM106A	-1.0073183
TMEM108	-1.6611218
TMEM109	-0.5576866
TMEM116	-3.7588105
TMEM130	-2.4793918
TMEM132A	-2.1410532
TMEM132B	-2.407659
TMEM132C	-4.309862
TMEM139	-3.0115368
TMEM14A	-1.5454082
TMEM150B	-2.5488274
TMEM150C	-3.755563
TMEM151A	-1.3748469
TMEM154	-2.2059712
TMEM156	-2.251002
TMEM159	-1.4058218
TMEM163	-3.5300276
TMEM168	-1.179009
TMEM169	-3.083553
TMEM178	-1.6546583
TMEM179	-4.0630193
TMEM182	-5.5614953
TMEM190	-4.392329
TMEM191C	-2.9547756
TMEM194B	-1.2412109
TMEM196	-2.4919162
TMEM198	-1.0755575
TMEM200A	-3.1534262
TMEM200B	-2.1549673
TMEM201	-0.400522
TMEM204	-2.9863787
TMEM209	-0.6269941

TMEM210	-3.6397696
TMEM212	-6.1219907
TMEM213	-5.107068
TMEM217	-2.166665
TMEM218	-3.1171017
TMEM221	-5.153451
TMEM229A	-3.354199
TMEM231	-2.6547763
TMEM232	-4.167933
TMEM233	-2.2606697
TMEM240	-2.490191
TMEM241	-0.6592839
TMEM245	-0.4545128
TMEM246	-2.73997
TMEM247	-3.0446541
TMEM253	-2.629585
TMEM28	-1.2616454
TMEM29	-0.9507554
TMEM35	-4.481719
TMEM38A	-0.9129152
TMEM40	-2.6192496
TMEM44	-4.832139
TMEM45A	-0.6655533
TMEM45B	-2.547217
TMEM47	-1.4541576
TMEM50B	-1.8273518
TMEM51OS1	-1.4827702
TMEM52	-3.1698146
TMEM54	-1.7560086
TMEM59L	-3.864753
TMEM61	-1.5082695
TMEM63C	-0.8898068
TMEM65	-1.2918396
TMEM71	-1.1393061
TMEM72	-3.0215037
TMEM74	-3.7236152
TMEM79	-2.626296
TMEM81	-1.1732771
TMEM87A	-0.7785122
TMEM88	-0.5304139
TMEM8B	-1.3625549
TMEM91	-1.0715299
TMEM95	-4.5931044
TMOD1	-4.321226
TMOD2	-1.8902701

TMPRSS11D	-0.9762621
TMPRSS11G	-2.1556087
TMPRSS12	-3.6227987
TMPRSS13	-4.5873275
TMPRSS4	-1.1116412
TMPRSS9	-3.0121975
TMSB10	-0.6368911
TMSB15B1	-1.341984
TMTC1	-3.9197397
TNC	-3.031901
TNFAIP8L2	-0.69473
TNFAIP8L3	-1.5197875
TNFRSF13B	-1.2974079
TNFRSF13C	-0.4352171
TNFRSF14	-0.8171871
TNFRSF17	-2.5286095
TNFRSF19	-5.0804396
TNFRSF8	-3.7446663
TNFSF10	-2.369244
TNFSF12TNF!	-1.0638685
TNFSF13B	-1.4675915
TNFSF13OS	-0.8922007
TNFSF14	-0.8164563
TNFSF15	-0.6589294
TNFSF18	-3.0611804
TNFSF8	-2.5218117
TNIK	-2.3248339
TNK1	-3.583297
TNK2OS	-5.440377
TNN	-1.0659726
TNNC1	-2.2849672
TNNC2	-4.0210266
TNNI2	-2.3659263
TNNI3	-6.2982483
TNNT2	-6.7983227
TNNT3	-3.183682
TNR	-3.2343245
TNRC6C	-0.471823
TNS1	-1.2461479
TNS3	-1.0387456
TNS4	-0.747186
TNXB	-2.6304247
TONSL	-1.9782677
TOR4A	-0.7530437
TOX3	-4.443698

TPBPB	-2.2789545
TPCN1	-0.4677274
TPGS2	-2.2944083
TPH2	-2.7275755
TPM1	-0.5544117
TPM2	-3.741347
TPPP	-2.9322548
TPPP2	-2.8184426
TPPP3	-4.688527
TPSG1	-3.6133206
TPTE	-3.2907543
TPX2	-1.3073289
TRABD2B	-4.4742413
TRAF3IP1	-2.7499242
TRAF3IP2	-1.4745486
TRAF3IP3	-1.3636887
TRAF5	-0.746367
TRAIP	-3.6048243
TRAM1L1	-3.491109
TRANK1	-0.751142
TRAPPC9	-0.4495604
TRAV14N-1	-4.869783
TRAV3N-3	-1.842303
TRAV9D-3	-1.9887018
TRAV9N-2	-2.4522145
TRCG1	-1.8301423
TRDN	-2.5149186
TREML1	-2.0215437
TREML2	-2.4029458
TRERF1	-2.8130658
TRGV2	-3.9241002
TRH	-1.7835464
TRIL	-3.0700862
TRIM10	-2.4524822
TRIM15	-4.6711283
TRIM16	-4.6472893
TRIM17	-2.315197
TRIM29	-1.434689
TRIM30C	-0.6717253
TRIM31	-2.3103127
TRIM35	-0.5672972
TRIM36	-3.2776108
TRIM37	-0.5311685
TRIM40	-1.6766787
TRIM42	-4.9904017

TRIM45	-2.6692355
TRIM50	-3.6037784
TRIM54	-4.340719
TRIM55	-5.5671935
TRIM58	-2.569449
TRIM59	-2.0731008
TRIM61	-5.0943336
TRIM62	-1.6761124
TRIM63	-0.8855443
TRIM66	-2.8803105
TRIM69	-3.5367677
TRIM7	-2.384532
TRIM71	-4.497436
TRIM72	-0.4857097
TRIML1	-3.554546
TRIML2	-3.9224443
TRIQK	-1.674638
TRMT12	-0.4686177
TRNP1	-3.2747996
TROAP	-3.801333
TROVE2	-2.2005148
TRP53I11	-0.6304567
TRP53I13	-1.9247305
TRP53TG5	-5.00277
TRP73	-2.567246
TRPC3	-3.0367723
TRPC4	-2.3963933
TRPC5	-3.313726
TRPC6	-4.857053
TRPM1	-1.6903532
TRPM4	-1.064929
TRPM6	-3.36356
TRPM8	-2.6535547
TRPS1	-1.7008939
TRPT1	-0.9520159
TRPV1	-0.9469024
TRPV3	-2.8819091
TRPV4	-1.273982
TRPV6	-3.20908
TRY5	-3.5438466
TSC2	-3.5308678
TSC22D1	-2.438615
TSC22D3	-1.7990358
TSEN34	-0.2682779
TSGA10IP	-0.4256024

TSGA8	-1.3999333
TSHB	-4.761922
TSHZ1	-0.3007324
TSHZ3	-1.8545234
TSKS	-2.7100735
TSLP	-3.2636483
TSPAN1	-7.816577
TSPAN11	-3.7916427
TSPAN13	-4.5163097
TSPAN17	-1.9954023
TSPAN18	-4.542587
TSPAN2	-6.0865946
TSPAN3	-0.8087084
TSPAN32	-5.9005337
TSPAN5	-2.8703363
TSPAN6	-1.351969
TSPAN7	-1.687372
TSPAN8	-6.778787
TSPYL3	-1.3361897
TSPYL4	-1.4961829
TSPYL5	-2.053146
TSR2	-0.5714369
TSSK1	-3.9669566
TSSK3	-2.7185571
TSSK5	-1.9241946
TSSK6	-2.9428658
TSX	-2.4848316
TTBK1	-1.5289471
TTC12	-2.056309
TTC16	-1.8652506
TTC18	-3.902323
TTC21A	-3.49305
TTC22	-3.6328893
TTC23L	-3.2282963
TTC25	-4.365963
TTC26	-2.7348542
TTC28	-2.7789738
TTC29	-4.681655
TTC30A2	-1.2940116
TTC30B	-0.753031
TTC34	-2.1755219
TTC39D	-4.3496866
TTC8	-0.9853687
TTC9	-4.247641
TTL	-0.3376248

TTLL1	-0.8526998
TTLL13	-3.5773873
TTLL2	-1.6179947
TTLL3	-0.8914361
TTLL5	-1.5341976
TTLL6	-2.5610373
TTLL7	-1.548908
TTLL8	-1.0705409
TTLL9	-3.8782256
TTN	-6.2279234
TTYH3	-1.7275648
TUBA1A	-2.3962348
TUBA3A	-2.447836
TUBA3B	-3.2024229
TUBAL3	-1.5092683
TUBB1	-4.1917677
TUBB2B	-2.7850125
TUBB3	-1.08308
TUBG2	-1.4899297
TULP2	-2.4667873
TUSC5	-3.4871662
TVP23A	-1.638469
TWF2	-0.4562042
TWIST2	-1.9762521
TXLNB	-2.5869262
TXLNG	-0.9144454
TXNDC16	-1.8577886
TXNDC2	-1.9439862
TYK2	-0.7879727
TYMS	-1.1866236
TYR	-2.8118348
TYRO3	-1.7825713
TYW3	-2.5290012
U90926	-1.4449759
UBASH3A	-2.9608757
UBE2C	-0.707377
UBE2DNL1	-3.5324414
UBE2U	-1.58427
UBL4B	-3.5717354
UBL7	-0.5586317
UBQLN4	-0.3847728
UBQLNL	-3.315335
UBXN10	-5.364608
UBXN11	-1.1447408
UCHL3	-0.6457787

UCMA	-2.7978494
UCN2	-0.9914675
UCP1	-2.8854272
UCP3	-0.3889921
UFSP1	-1.0846086
UGT8A	-2.795095
UHRF1	-0.7110529
UIMC1	-1.3557544
ULBP1	-2.6435194
ULK4	-4.1815743
UNC119	-0.2624071
UNC13A	-2.8729718
UNC13D	-1.3264163
UNC45B	-1.8516855
UNC45BOS	-2.6190066
UNC5A	-1.7393451
UNC5B	-0.6771619
UNC5C	-3.3521962
UNC5D	-12.737003
UNC79	-2.9110348
UNC80	-2.9477184
UNCX	-2.2584295
UPK1A	-1.4688616
UPK1B	-3.4380846
UPK2	-3.9516106
UPK3A	-4.561471
UPK3BL	-2.4892287
USH1C	-3.5144212
USH1G	-2.1397414
USHBP1	-3.4473503
USP11	-1.6306896
USP13	-2.998117
USP17LE	-2.1331193
USP27X	-3.0317154
USP35	-2.4593804
USP37	-2.2627687
USP44	-3.9634204
USP46	-0.8710907
USP51	-1.8159254
USP54	-1.7479582
USP8	-1.3828866
USP9Y	-3.5795913
UTP14B	-2.7630002
UTRN	-0.98791
UTS2	-2.5710943

UTS2R	-0.5262229
UTY	-2.4164488
UVSSA	-3.6701512
V1RA8	-3.5164292
VAC14	-0.5471766
VAMP3	-0.9426298
VANGL2	-0.5931861
VARS2	-1.3497705
VASH2	-3.2824342
VAV3	-2.0070257
VAX1	-3.8143117
VAX2OS	-1.8910131
VDR	-1.0806425
VEGFA	-0.4799287
VEPH1	-4.911352
VEZF1	-0.590481
VEZT	-1.9917471
VGLL1	-2.5146308
VGLL2	-4.7385993
VGLL3	-3.4013655
VIP	-4.3712053
VIPR2	-4.126794
VLDLR	-0.7086194
VMAC	-0.4736981
VMN1R1	-3.2027745
VMN1R131	-2.403607
VMN1R132	-4.0905914
VMN1R139	-2.1853285
VMN1R148	-3.6579432
VMN1R157	-3.8555784
VMN1R168	-2.4686894
VMN1R17	-2.1019166
VMN1R171	-2.9119177
VMN1R172	-4.1395783
VMN1R185	-3.3058865
VMN1R199	-4.0970182
VMN1R2	-3.10776
VMN1R20	-4.040592
VMN1R202	-3.1817932
VMN1R204	-1.1868749
VMN1R207-F	-2.9330928
VMN1R210	-2.4696271
VMN1R214	-2.5045881
VMN1R216	-2.4547198
VMN1R223	-3.0216622

VMN1R224	-2.9580534
VMN1R225	-2.5213475
VMN1R227	-4.1618495
VMN1R230	-4.3462806
VMN1R235	-3.2656622
VMN1R24	-3.639029
VMN1R28	-3.412644
VMN1R32	-2.6617124
VMN1R33	-3.3675725
VMN1R34	-5.3423214
VMN1R39	-3.228328
VMN1R41	-2.9645388
VMN1R42	-4.783785
VMN1R47	-5.726698
VMN1R56	-1.2968369
VMN1R58	-2.2098718
VMN1R6	-3.2682197
VMN1R62	-1.2486475
VMN1R63	-2.9856515
VMN1R64	-2.55702
VMN1R66	-5.5610447
VMN1R67	-4.81763
VMN1R70	-0.318398
VMN1R72	-2.2809489
VMN1R74	-3.3398201
VMN1R75	-4.810191
VMN1R81	-2.5102274
VMN1R84	-4.4415426
VMN1R86	-4.4074993
VMN2R102	-2.2760882
VMN2R103	-2.575473
VMN2R104	-3.0719671
VMN2R107	-2.921341
VMN2R11	-1.0774329
VMN2R111	-3.1409163
VMN2R114	-3.0295033
VMN2R115	-2.614244
VMN2R118	-5.642839
VMN2R12	-2.701505
VMN2R120	-2.263959
VMN2R121	-3.1582766
VMN2R13	-2.3215332
VMN2R27	-1.4820182
VMN2R3	-2.6550958
VMN2R35	-2.6836643

VMN2R39	-3.946389
VMN2R48	-3.8072205
VMN2R54	-2.9387095
VMN2R55	-3.0162926
VMN2R58	-3.2071602
VMN2R60	-6.6441097
VMN2R62	-5.114252
VMN2R67	-2.8606606
VMN2R7	-3.0496182
VMN2R71	-3.2922869
VMN2R72	-3.40945
VMN2R73	-3.408082
VMN2R77	-5.4496603
VMN2R80	-4.735147
VMN2R82	-3.367467
VMN2R83	-3.1862578
VMN2R85	-3.675939
VMN2R91	-3.3908129
VMN2R98	-2.1524482
VMN2R-PS10	-2.7098935
VMN2R-PS12	-5.187035
VMN2R-PS54	-2.1653178
VMN2R-PS57	-2.7352874
VMO1	-0.4789109
VPREB1	-4.2377915
VPREB2	-6.52313
VPREB3	-4.358542
VPS51	-0.7542527
VPS72	-2.6160483
VSIG10L	-2.9155078
VSIG2	-2.402823
VSIG8	-2.8142445
VSTM2A	-2.7845945
VSTM2L	-5.2370443
VSTM4	-3.2058656
VSTM5	-3.0294106
VSX2	-3.1936827
VWA1	-3.4003093
VWA3A	-3.9312162
VWA5B1	-2.6132996
VWA5B2	-1.3348966
VWC2	-1.2050395
VWDE	-2.5294416
WAP	-2.3173442
WASF3	-1.9052849

WBSCR17	-2.754386
WBSCR25	-3.808164
WBSCR28	-2.3004677
WDFY1	-0.4353735
WDFY4	-1.0742104
WDPCP	-0.3714979
WDR16	-4.879611
WDR20RT	-1.9572811
WDR25	-0.5142906
WDR27	-4.1904726
WDR49	-3.9215133
WDR52	-2.9569802
WDR5B	-1.6613374
WDR62	-2.2595248
WDR63	-1.8957858
WDR64	-3.0109963
WDR65	-5.300933
WDR7	-1.0367951
WDR70	-3.4077299
WDR76	-1.9134011
WDR78	-2.639937
WDR86	-5.2997975
WDR93	-3.2718763
WDR95	-2.358838
WDR96	-3.8317215
WFDC1	-4.2347546
WFDC10	-5.1781693
WFDC11	-3.4152875
WFDC12	-2.466326
WFDC15A	-6.0737505
WFDC15B	-2.096847
WFDC16	-3.6197352
WFDC2	-3.6219156
WFDC3	-1.221601
WFDC6A	-2.4303002
WFDC6B	-2.3472772
WFDC8	-3.3894088
WHRN	-1.9288077
WIBG	-2.6743608
WIF1	-6.347686
WIPF1	-0.9217072
WIPF3	-1.8650067
WISP1	-0.8567154
WISP2	-1.21558
WISP3	-6.534381

WLS	-2.790699
WNK2	-2.2312777
WNK4	-5.549991
WNT1	-1.3467791
WNT10B	-4.454863
WNT11	-2.106145
WNT16	-2.6815758
WNT2	-3.4082952
WNT2B	-2.7390244
WNT4	-2.2974489
WNT5A	-1.0178676
WNT6	-1.510262
WNT7A	-4.217417
WNT8B	-2.8582294
WNT9A	-1.138768
WNT9B	-1.6592138
WSCD1	-3.830307
WSCD2	-3.0673628
WTIP	-0.2950962
WWC1	-0.8743804
WWC2	-0.9681933
WWP1	-0.9340365
WWTR1	-0.8172915
XCR1	-3.014581
XIRP1	-1.9020681
XK	-2.8969772
XKR4	-3.1320682
XKR7	-2.1201558
XKRX	-1.7450242
XLR5B	-1.6291981
XNDC1	-0.8237398
XPNPEP2	-4.033189
XPO7	-0.8025494
XRCC1	-0.7785389
XRCC6BP1	-4.888009
XXYLT1	-0.7352562
XYLT1	-3.0629923
YBEY	-1.6397138
YBX2	-2.7742167
YDJC	-1.2404203
YIPF7	-2.1851552
YLPM1	-1.6937163
YPEL1	-1.9822514
YPEL4	-1.648381
ZAN	-3.8635092

ZAR1	-3.6812088
ZBBX	-4.6490965
ZBTB21	-0.3900459
ZBTB3	-0.7650125
ZBTB32	-1.1797402
ZBTB38	-1.0209899
ZBTB4	-0.6389973
ZBTB44	-0.6773069
ZBTB46	-0.7838028
ZBTB7C	-3.9430833
ZBTB8A	-0.8955774
ZBTBD6	-2.1839254
ZC2HC1A	-1.7441356
ZC2HC1B	-3.5367408
ZC3H6	-1.3775365
ZC3H8	-1.6520402
ZC3HAV1L	-3.1486146
ZC4H2	-3.5276606
ZCCHC12	-3.6390922
ZCCHC13	-3.7129517
ZCCHC16	-3.7336512
ZCCHC18	-4.5410542
ZCCHC5	-3.8105004
ZCWPW2	-3.1882792
ZDHHC1	-2.001879
ZDHHC14	-0.8676951
ZDHHC15	-3.7671108
ZDHHC22	-0.9958885
ZDHHC23	-0.6233151
ZDHHC25	-4.434542
ZDHHC8	-1.8053186
ZEB2	-0.5562103
ZEB2OS	-2.2854283
ZFHX2OS	-2.0818276
ZFHX3	-1.0491874
ZFP108	-1.5628726
ZFP11	-3.1393027
ZFP114	-2.6322417
ZFP119A	-1.6730728
ZFP12	-1.9396985
ZFP142	-1.8586662
ZFP160	-3.5777473
ZFP185	-3.5955832
ZFP235	-1.7307384
ZFP251	-0.6830943

ZFP273	-1.10379
ZFP28	-1.2115599
ZFP287	-2.1980894
ZFP296	-1.7943287
ZFP300	-4.0700617
ZFP329	-0.7610402
ZFP345	-2.6103315
ZFP352	-1.483702
ZFP354B	-1.703098
ZFP354C	-4.537517
ZFP362	-1.1085839
ZFP365	-4.882284
ZFP366	-3.1011167
ZFP36L3	-1.6792889
ZFP37	-3.7268186
ZFP382	-1.7408142
ZFP385A	-0.6542666
ZFP385C	-4.05513
ZFP389	-1.9648137
ZFP398	-0.7391422
ZFP42	-3.7472472
ZFP420	-1.4618149
ZFP428	-2.2073565
ZFP438	-2.3946817
ZFP444	-3.1768448
ZFP454	-3.3659575
ZFP455	-2.0750391
ZFP46	-0.3680713
ZFP462	-1.9403561
ZFP467	-1.2319391
ZFP473	-1.7402129
ZFP474	-5.1285257
ZFP503	-2.2824397
ZFP516	-1.4228618
ZFP521	-3.222133
ZFP526	-2.5462751
ZFP536	-2.1686249
ZFP551	-2.427208
ZFP560	-1.3063014
ZFP563	-3.7105083
ZFP57	-1.2895739
ZFP575	-1.3209949
ZFP580	-1.508934
ZFP583	-1.5129659
ZFP595	-0.5909121

ZFP599	-0.4956908
ZFP60	-1.7317393
ZFP605	-1.2786057
ZFP608	-1.1271198
ZFP609	-1.9911087
ZFP61	-2.29322
ZFP616	-3.433096
ZFP619	-0.7682836
ZFP641	-1.148474
ZFP65	-0.8066876
ZFP652OS	-1.2439268
ZFP667	-1.3791945
ZFP697	-1.8165965
ZFP703	-0.5892341
ZFP704	-0.9639103
ZFP711	-3.8847344
ZFP72	-1.0274785
ZFP735	-2.8717296
ZFP74	-0.9106307
ZFP759	-3.1296372
ZFP773	-0.9247267
ZFP78	-1.4211795
ZFP781	-0.9075649
ZFP783	-1.096457
ZFP786	-4.941637
ZFP808	-6.493881
ZFP82	-0.9895134
ZFP821	-0.5734546
ZFP827	-3.2555466
ZFP84	-0.6384289
ZFP846	-0.4473941
ZFP85	-2.5442913
ZFP850	-2.3122048
ZFP870	-2.6589987
ZFP873	-1.1712279
ZFP874A	-2.0698833
ZFP874B	-0.8933106
ZFP879	-3.9241157
ZFP882	-0.8814774
ZFP92	-1.217481
ZFP933	-1.7406826
ZFP934	-2.4736197
ZFP936	-0.2727432
ZFP939	-4.373227
ZFP94	-3.6768115

ZFP948	-0.3792768
ZFP950	-1.5109725
ZFP951	-2.2202823
ZFP952	-1.1158454
ZFP957	-1.7517321
ZFP958	-1.2407491
ZFR2	-3.631048
ZFY2	-3.4726534
ZFYVE28	-5.226798
ZG16	-2.7983346
ZGPAT	-0.4182117
ZGRF1	-3.8462493
ZIC1	-1.7187409
ZIC2	-2.3659966
ZIC3	-3.260188
ZIC4	-5.2571564
ZIC5	-3.130684
ZKSCAN2	-3.0120766
ZKSCAN4	-3.3320096
ZMAT4	-3.3971593
ZMIZ1OS1	-1.8009796
ZMYND10	-6.3471
ZMYND12	-1.8240132
ZNRF3	-1.2096367
ZNRF4	-5.565144
ZP1	-1.4697917
ZP3	-3.4439802
ZP3R	-3.8843722
ZPBP	-2.3664887
ZPBP2	-2.579691
ZRANB3	-1.2584136
ZRSR2	-1.7735922
ZSCAN10	-3.130966
ZSCAN18	-1.0076993
ZSCAN2	-2.2509542
ZSCAN30	-4.3493795
ZSCAN4B	-0.9857552
ZSCAN5B	-1.7977021
ZWILCH	-1.0778296
AAAS	1.7641504
AACS	1.8990085
AADAC	10.198011
AAED1	1.4622843
AAGAB	2.39801
AAK1	0.77402806

AAMP	1.2547529
AAR2	1.5375035
AARS	3.495485
AARSD1	1.3139474
AASDH	0.3792956
AASDHPPT	1.546778
AASS	5.6108685
AATF	2.1778986
ABAT	3.8640125
ABCA1	4.8437576
ABCA6	4.3265038
ABCA8B	3.615278
ABCB10	1.364672
ABCB11	7.224962
ABCB1B	1.6047218
ABCB4	4.070616
ABCB7	1.3404949
ABCB8	2.1255853
ABCC1	1.2767465
ABCC10	1.6545727
ABCC2	7.929564
ABCC3	4.159813
ABCC4	1.1833179
ABCC6	8.091809
ABCC9	1.2300956
ABCD3	2.03247
ABCD4	0.77493215
ABCE1	2.3756902
ABCF1	2.0834644
ABCF2	1.3196533
ABCF3	2.7914655
ABCG2	1.9630964
ABCG5	7.703603
ABCG8	1.0769417
ABHD1	0.9519136
ABHD10	0.9661555
ABHD11	2.0600913
ABHD13	1.0291793
ABHD14B	1.0001757
ABHD15	3.0421598
ABHD16A	0.3363006
ABHD17A	0.6169274
ABHD17B	1.209274
ABHD17C	1.7721694
ABHD2	1.5192871

ABHD3	1.9793196
ABHD4	1.6006396
ABHD5	1.4631298
ABHD6	1.3915985
ABI1	0.901994
ABI2	0.7630284
ABL1	0.69958425
ABL2	1.2614892
ABRACL	1.5223868
ABTB1	1.1409166
ABTB2	2.2452023
ACAA1A	3.1302335
ACAA1B	3.0791657
ACAA2	2.3690956
ACACA	0.93778014
ACACB	1.0767591
ACAD10	2.1748717
ACAD11	3.8962796
ACAD9	2.229993
ACADL	3.8105357
ACADM	2.5544913
ACADS	2.4094675
ACADSB	1.1192238
ACADVL	3.9285157
ACAP2	0.26970935
ACAT1	2.0846307
ACAT2	5.4466114
ACAT3	5.7748127
ACBD3	1.9903939
ACBD4	1.4162738
ACBD5	0.52333426
ACBD6	2.0983827
ACCS	1.3908291
ACCSL	2.5624847
ACD	1.505274
ACER3	1.4231842
ACIN1	0.91528106
ACKR1	6.9204454
ACLY	0.5935514
ACMSD	1.3799577
ACN9	1.6250331
ACNAT1	3.366413
ACNAT2	3.787193
ACO2	2.07429
ACOT1	0.6750891

ACOT12	3.4889858
ACOT13	1.1595023
ACOT2	1.2229862
ACOT3	2.5321949
ACOT4	4.9418077
ACOT6	1.6916401
ACOT8	1.8415267
ACOT9	0.43072295
ACOX1	4.56022
ACOX2	6.030079
ACP1	2.1951783
ACP2	1.5492461
ACP5	4.431566
ACP6	2.3497179
ACRBP	0.81092143
ACSF2	2.0763967
ACSF3	1.4536946
ACSL1	2.0747068
ACSL3	1.5953343
ACSL4	1.2579367
ACSL5	0.41912627
ACSM1	4.338312
ACSM3	3.697776
ACSM5	1.1543567
ACTB	1.7663622
ACTG1	1.6239283
ACTL6A	1.0761912
ACTN4	0.82789445
ACTR10	2.7998183
ACTR1A	0.9605863
ACTR2	0.7819617
ACTR3	1.3241022
ACTR5	0.74600434
ACTR6	1.1955378
ACTR8	1.8322451
ACVR1	1.4397166
ACVR1B	2.2791274
ACVR2B	0.52676654
ACY3	2.0495622
ADA	1.9854901
ADAL	1.6892736
ADAM10	1.1190073
ADAM17	1.3016305
ADAM23	2.235973
ADAM8	0.90625787

ADAM9	2.3414
ADAMDEC1	0.6666424
ADAMTS1	2.0883558
ADAMTS4	2.791095
ADAMTS7	3.1480718
ADAMTS9	0.92812276
ADAP1	0.4161656
ADAP2	1.6982815
ADAR	1.1739872
ADAT2	2.8145921
ADCK1	1.3407853
ADCK2	1.2367513
ADCK4	4.9560966
ADCK5	0.7552526
ADCY1	3.4454482
ADH1	1.4801762
ADH4	5.8654737
ADH7	2.511626
ADHFE1	3.1170194
ADI1	1.0344212
ADIPOR1	1.3188164
ADIPOR2	2.19646
ADK	4.384063
ADM	2.9368083
ADM2	1.06271
ADNP	1.044348
ADO	1.1624801
ADORA1	3.866649
ADPGK	1.7670195
ADPRH	0.8917086
ADPRHL2	1.3780835
ADPRM	2.6911342
ADRA1B	1.8784363
ADRA2A	4.3086796
ADRB2	2.0161707
ADRBK1	0.5930078
ADRBK2	1.0158937
ADRM1	0.80019355
ADSL	0.7561219
ADSS	1.1651175
ADTRP	2.2691424
AEBP2	1.7799308
AEN	3.0507739
AES	0.6442807
AFF1	0.7551

AFF4	2.931011
AFG3L1	2.7961886
AFG3L2	3.396701
AFM	10.154221
AFMID	0.52967167
AFP	1.4785123
AFTPH	1.9667022
AGA	2.239004
AGAP3	0.50927377
AGFG1	1.5709336
AGFG2	0.99877596
AGGF1	1.2043173
AGK	1.1503103
AGL	1.1378539
AGMAT	3.959705
AGMO	0.9741404
AGO2	1.5956843
AGO3	0.92597747
AGPAT2	3.0882566
AGPAT3	2.5593445
AGPAT5	2.71322
AGPAT6	2.3098314
AGPAT9	3.7668817
AGT	5.9557247
AGTR1A	3.5307677
AGXT	8.442112
AGXT2	7.136546
AHCTF1	0.77573705
AHCY	5.8615665
AHCYL1	1.7923186
AHSA1	1.8439815
AHSG	9.150278
AIDA	0.9262631
AIFM1	3.0862849
AIFM2	2.7848718
AIG1	2.4074342
AIM1	3.1386087
AIMP1	1.9529335
AIMP2	2.5059283
AIP	0.7426541
AK2	2.488777
AK3	1.5787742
AK4	5.6381207
AK6	1.7844317
AKAP1	1.6477325

AKAP10	0.77079153
AKAP11	1.9763014
AKAP12	2.0829175
AKAP13	0.39584565
AKAP8	0.39375448
AKAP8L	1.0915349
AKAP9	1.76141
AKIP1	1.3664362
AKIRIN1	2.1064394
AKIRIN2	1.2189591
AKR1A1	0.8506367
AKR1B7	1.7830136
AKR1B8	0.42182612
AKR1C12	5.916465
AKR1C13	4.2650156
AKR1C18	1.1557326
AKR1C19	3.1408503
AKR1C20	0.55533385
AKR1C6	4.670598
AKR1E1	2.0716505
AKR7A5	1.625911
AKT1	0.37552667
AKT1S1	1.7705328
AKT2	1.7094209
ALAD	0.559531
ALAS1	1.7471993
ALB	8.478748
ALDH16A1	2.028179
ALDH1A3	1.4364126
ALDH1B1	3.581752
ALDH1L1	5.919813
ALDH1L2	2.9281683
ALDH2	2.0160663
ALDH4A1	1.8956859
ALDH5A1	3.7491329
ALDH6A1	3.2057073
ALDH7A1	1.6347215
ALDH8A1	7.6240473
ALDH9A1	2.748226
ALDOA	2.128994
ALDOB	9.570196
ALDOC	1.5298884
ALG1	2.379516
ALG10B	1.5746787
ALG11	2.6987045

ALG12	0.65842533
ALG13	2.2753725
ALG14	2.1432745
ALG2	2.0709674
ALG3	1.883153
ALG5	2.3815668
ALG6	1.1897995
ALG8	3.2656398
ALG9	2.4015834
ALKBH1	2.0114248
ALKBH2	3.3280618
ALKBH3	2.0762742
ALKBH4	0.5180545
ALKBH5	2.420215
ALKBH6	1.9476359
ALKBH7	0.9468863
ALKBH8	1.1045527
ALPK1	4.18443
ALPL	2.7485554
ALS2CL	0.57604194
ALX4	1.739182
ALYREF	1.2738903
AMACR	4.7743444
AMBP	9.354555
AMBRA1	1.7777331
AMD2	0.39277577
AMDHD1	3.5015771
AMDHD2	1.3250382
AMER3	1.0216722
AMFR	2.3498642
AMICA1	0.44801974
AMIGO1	1.4753141
AMIGO2	1.9216304
AMIGO3	1.5895767
AMMECR1	1.0161064
AMMECR1L	2.5759828
AMN	4.082223
AMN1	1.2834084
AMOT	1.4466002
AMPD2	2.0719845
AMPD3	1.1958613
AMY1	7.3139772
AMY2A5	4.8978434
AMZ2	1.0754521
ANAPC1	1.4812825

ANAPC11	0.53557944
ANAPC13	0.6577909
ANAPC15	1.4334252
ANAPC16	1.7024701
ANAPC4	0.8765018
ANAPC5	0.50929236
ANAPC7	1.7686837
ANG	5.880186
ANG2	3.690625
ANG3	2.2014525
ANG4	5.381316
ANGEL2	0.97640395
ANGPT2	1.7369621
ANGPTL3	2.8369524
ANGPTL4	3.2455952
ANGPTL6	0.8800361
ANK	2.4466188
ANKFY1	1.4338939
ANKHD1	1.7884886
ANKIB1	1.9028809
ANKLE2	0.84296465
ANKMY1	1.0241795
ANKRA2	0.9723251
ANKRD10	0.82727337
ANKRD11	1.5026982
ANKRD13B	0.59314036
ANKRD13C	1.7179639
ANKRD16	1.4206212
ANKRD17	2.356706
ANKRD2	2.0989032
ANKRD22	1.077652
ANKRD26	0.7759135
ANKRD27	2.1174352
ANKRD28	2.1691582
ANKRD32	0.49716783
ANKRD37	2.7191122
ANKRD39	1.1153357
ANKRD46	0.78570724
ANKRD49	2.7916057
ANKRD52	2.4527142
ANKRD9	1.1645877
ANKS1	0.57635283
ANKS3	1.0537024
ANKS4B	0.8122089
ANKZF1	2.7219756

ANO10	1.5213258
ANO2	2.6257355
ANP32A	0.3472383
ANP32B	1.0438917
ANPEP	2.3735821
ANXA4	0.28199697
ANXA6	0.4236176
ANXA7	2.1599877
AOAH	2.7830575
AOC2	1.4860361
AOX1	0.8319719
AP1AR	1.6426144
AP1B1	2.064698
AP1G1	1.9360569
AP1M1	1.8481319
AP1S1	1.4118087
AP2A1	1.6873515
AP2A2	0.65497327
AP2M1	2.0479715
AP2S1	1.3650868
AP3B1	1.8562176
AP3D1	2.0403068
AP3M1	2.7299397
AP3S1	2.3650901
AP3S2	0.54414296
AP4B1	2.3264592
AP4M1	1.0177505
AP5B1	2.4234178
AP5M1	1.4747875
AP5S1	3.8754327
AP5Z1	1.3191884
APAF1	1.8777053
APBA3	2.4308026
APBB3	4.876609
APCS	12.513647
APEH	1.8007505
APELA	1.0145812
APEX1	2.7690442
APH1A	0.46074986
APH1B	0.40208912
API5	0.97470117
APIP	2.2601159
APLP2	0.55180097
APMAP	1.3034451
APOA1	10.108978

APOA1BP	0.43929887
APOA2	5.4197235
APOA4	6.2741203
APOA5	7.2972517
APOB	9.317396
APOBEC1	2.341242
APOC1	5.4690523
APOC2	5.5125237
APOC3	7.5229006
APOC4	9.154554
APOE	4.2588253
APOF	9.77665
APOH	10.087458
APOL10B	0.7542155
APOL7A	1.3620648
APOL9A	3.9509122
APOL9B	4.377185
APOLD1	2.7392118
APOM	7.6911736
APON	9.400627
APOO	2.5397265
APOOL	2.423168
APOPT1	2.1962864
APPBP2	1.2662032
APPL1	1.1672719
APPL2	1.2869842
APRT	2.2029274
AQP11	3.3817937
AQP8	4.331229
AQP9	3.6432557
AQR	1.2565565
ARAF	2.5362089
ARAP1	1.0521042
ARCN1	2.336723
AREG	1.6285996
AREL1	2.432459
ARF1	1.4847744
ARF3	0.48222184
ARF4	2.4903147
ARF5	0.4132173
ARF6	1.9711754
ARFGAP1	2.094187
ARFGAP2	2.0984643
ARFGAP3	1.2835224
ARFGEF1	1.5966675

ARFGEF2	1.9813948
ARFIP1	0.9369252
ARFIP2	1.4489281
ARG1	10.152939
ARG2	3.2426794
ARGLU1	1.1570814
ARHGAP1	0.6838949
ARHGAP12	2.165488
ARHGAP17	1.0352108
ARHGAP21	0.4116509
ARHGAP26	2.5345454
ARHGAP35	1.84324
ARHGAP42	0.42233205
ARHGAP5	2.2354991
ARHGDIA	1.0630248
ARHGDIB	0.6011641
ARHGEF10L	2.7513754
ARHGEF11	0.52619076
ARHGEF12	1.1062734
ARHGEF15	3.1658356
ARHGEF26	2.1386826
ARHGEF3	0.7253697
ARHGEF5	1.3789723
ARID1A	0.5341704
ARID1B	0.994117
ARID3A	0.46898246
ARID4A	0.66190624
ARID5A	3.4174516
ARIH1	1.4873857
ARIH2	2.3796341
ARL1	2.6487424
ARL13B	0.5512538
ARL14EP	1.874701
ARL15	1.1152818
ARL16	1.2264788
ARL4A	3.800607
ARL4C	0.63149095
ARL4D	1.4163
ARL5B	1.0271404
ARL6	1.5072029
ARL6IP1	0.41615844
ARL6IP4	0.8719437
ARL6IP5	0.40874982
ARL6IP6	3.4294403
ARL8A	2.5019372

ARL8B	1.583081
ARMC1	1.763912
ARMC10	0.96899295
ARMC5	0.4369805
ARMC8	1.4479959
ARMCX3	2.2294118
ARMCX5	2.0837543
ARNT	1.5204527
ARNTL	1.4195364
ARPC1A	0.74590945
ARPC1B	1.8343146
ARPC2	1.3912728
ARPC3	1.4062426
ARPC4	1.0462573
ARPC5	0.9404123
ARPC5L	0.5972197
ARRB2	1.4251583
ARRDC2	0.8539989
ARRDC4	5.1516247
ARSA	2.6254632
ARSG	3.6315486
AS3MT	3.7769287
ASAH1	0.9012644
ASAP2	0.6738918
ASB13	1.1479394
ASB4	2.5084493
ASB6	1.409764
ASB7	1.5866201
ASB8	1.6156676
ASCC2	1.2618306
ASCC3	1.9969699
ASCL1	0.35844874
ASGR1	7.384157
ASGR2	7.9595184
ASH1L	1.4275653
ASH2L	0.8298166
ASIC3	0.50529015
ASL	6.4272623
ASNA1	1.3474219
ASNS	3.4442732
ASNSD1	1.4525931
ASPA	1.4986994
ASPDH	1.2807474
ASPG	6.673957
ASPH	1.0314789

ASPRV1	2.3569787
ASPSCR1	1.0613096
ASS1	4.755933
ASUN	1.5942466
ASXL1	0.85632443
ASXL2	0.8702519
ATAD1	1.9546688
ATAD2B	0.8635714
ATAD3A	3.165209
ATE1	1.0958936
ATF1	1.8112681
ATF2	1.3243906
ATF3	5.041786
ATF4	3.051211
ATF5	3.5483143
ATF6	2.0384529
ATF6B	1.246144
ATF7IP	1.4644649
ATG10	0.91341615
ATG101	1.295614
ATG12	1.6374943
ATG13	2.3546584
ATG14	2.0060022
ATG16L1	2.119118
ATG16L2	3.7596252
ATG2A	3.298197
ATG2B	1.156127
ATG3	1.6041329
ATG4B	2.1106644
ATG4C	0.9913738
ATG4D	2.3130128
ATG5	1.8071482
ATG7	1.8260777
ATG9A	2.1992137
ATHL1	0.47459388
ATIC	1.0908792
ATL1	1.8015943
ATL2	2.9357688
ATN1	0.95438075
ATOX1	1.6539857
ATP10D	1.0756929
ATP11B	1.2289317
ATP11C	2.4593432
ATP13A1	2.0695112
ATP13A2	0.48125243

ATP13A3	1.0335219
ATP1A1	0.92175937
ATP1B1	0.6911466
ATP1B3	1.4375875
ATP2A2	1.390888
ATP5A1	1.9598367
ATP5B	1.9492972
ATP5C1	2.1086829
ATP5C1-PS	1.7502086
ATP5D	1.9528944
ATP5E	1.8090956
ATP5F1	2.4077046
ATP5G1	2.876181
ATP5G2	1.2120278
ATP5G3	3.0934637
ATP5H	1.3776224
ATP5J	2.0628226
ATP5J2	1.7357728
ATP5K	1.6756675
ATP5L	1.8189971
ATP5O	1.911567
ATP5S	2.9980998
ATP5SL	2.1176288
ATP6	3.7107117
ATP6AP1	1.3655283
ATP6AP2	2.2489665
ATP6V0A1	1.7836292
ATP6V0A2	1.5575082
ATP6V0A4	4.624897
ATP6V0B	1.953346
ATP6V0C	2.0268161
ATP6V0D1	1.315511
ATP6V0E	1.158457
ATP6V1A	1.2692101
ATP6V1B2	1.4291155
ATP6V1C1	2.2962868
ATP6V1D	2.5462248
ATP6V1E1	2.3628786
ATP6V1F	1.642041
ATP6V1G1	1.1172097
ATP6V1H	2.345225
ATP8	2.7905867
ATP8B1	2.557379
ATP9A	1.9691002
ATP9B	1.149445

ATPAF1	1.2301321
ATPAF2	0.8633945
ATR	1.6341422
ATRAID	1.791951
ATRIP	0.8737924
ATRN	0.80184245
ATXN10	1.1731431
ATXN1L	0.42574525
ATXN2	2.388068
ATXN2L	2.1484754
ATXN7	3.5937188
ATXN7L1	0.40603018
ATXN7L3B	2.0126593
AXIN1	1.4074886
AZGP1	9.2260275
AZI2	1.9970174
AZIN1	0.78860974
B2M	2.007596
B3GALNT1	1.3324916
B3GALNT2	0.9080441
B3GALT1	7.891714
B3GALT6	1.4990926
B3GAT3	0.25021315
B3GNT1	0.60077834
B3GNT2	1.6026685
B4GALNT1	1.6245196
B4GALT1	1.5680645
B4GALT3	1.0151389
B4GALT5	0.62862754
B4GALT6	0.41223502
B4GALT7	0.53786397
B9D2	1.7953231
BAAT	6.7504435
BABAM1	1.9571588
BACE1	0.9743836
BACE2	0.43261123
BACH1	2.4616663
BAD	1.0099509
BAG3	1.2468617
BAG4	1.3661041
BAG5	1.5080693
BAG6	1.2410138
BAHCC1	2.3394525
BAHD1	1.4892771
BAIAP2L1	1.1239874

BAK1	1.77403
BANF1	1.4454949
BAP1	2.4991915
BASP1	1.2020817
BATF	2.311742
BATF2	1.8826482
BATF3	4.1581106
BAX	3.043475
BAZ1A	0.794595
BAZ2A	1.3181918
BAZ2B	0.59263825
BBC3	1.065696
BBIP1	0.5611489
BBOX1	3.14128
BBS12	0.30316067
BBS2	0.3469479
BBS4	0.46094012
BBS9	0.677397
BBX	1.0333748
BCAP29	1.491965
BCAP31	1.2166708
BCAR1	1.2971027
BCAR3	0.60691476
BCAS2	1.7936771
BCCIP	3.0132244
BCDIN3D	1.4924538
BCHE	1.5219724
BCKDHA	2.9301426
BCKDHB	5.0024767
BCL10	2.4682276
BCL2A1C	2.2841604
BCL2A1D	2.006523
BCL2L1	2.2031767
BCL2L11	0.76413274
BCL2L13	0.85361886
BCL2L14	0.8658955
BCL2L15	2.2125475
BCL3	3.3917382
BCL6	0.8684094
BCL7A	0.970711
BCL7B	1.3474066
BCL7C	0.45261073
BCLAF1	1.330878
BCMO1	7.2130613
BCO2	2.3277042

BCOR	1.1440914
BCR	0.69991803
BCS1L	1.4178064
BDH2	0.89430594
BDP1	1.2223585
BEAN1	0.6553149
BECN1	1.6473434
BEND3	1.9943635
BET1	2.467408
BET1L	2.3644264
BFAR	2.5206106
BGLAP2	0.82307386
BHLHA15	4.1358023
BHLHE40	3.603235
BHMT	8.005079
BHMT2	9.979908
BICD1	0.64757943
BID	3.776909
BIN1	0.44001746
BIN3	1.180558
BIRC2	4.2831106
BIRC3	2.2500422
BIRC6	1.6226337
BIVM	1.0452013
BLCAP	3.1460092
BLM	1.5318525
BLMH	1.8959849
BLOC1S1	1.4305332
BLOC1S2	1.6849654
BLOC1S3	1.4366112
BLOC1S4	2.1604831
BLOC1S5	1.6691802
BLOC1S6	1.4179609
BLVRB	2.3708465
BLZF1	1.9526293
BMI1	1.0976117
BMP1	0.63533425
BMP2	1.6171458
BMP2K	1.0807154
BMP8A	1.2828865
BMPER	2.629811
BMS1	1.2841511
BNIP1	1.7705369
BNIP3	4.267869
BNIP3L	1.3482759

BOD1	1.3887308
BOK	1.6175106
BOLA2	2.3473814
BOLA3	1.4981067
BOP1	2.4828699
BPHL	0.9677589
BPIFC	2.1433141
BPNT1	1.2556801
BPTF	0.94167733
BRAF	0.9395039
BRAP	2.7153442
BRAT1	1.2332871
BRCC3	1.0949028
BRD1	0.6710777
BRD2	0.88251996
BRD3	0.2894218
BRD4	1.0772707
BRD7	0.7386987
BRD8	1.564354
BRD9	0.833858
BRE	1.8830082
BRF2	1.4170165
BRI3	1.5045073
BRI3BP	1.0800669
BRINP2	1.6133628
BRIP1OS	2.1048188
BRIX1	2.3947556
BRK1	0.59186864
BRMS1	1.8583958
BROX	1.147526
BRPF1	1.6477039
BRPF3	2.513983
BRWD1	0.6672368
BRWD3	1.4484928
BSDC1	2.65292
BSG	1.3085263
BST1	1.0317991
BST2	4.0600796
BTAF1	1.2735679
BTBD10	0.45721412
BTBD16	2.223089
BTBD19	1.2200141
BTBD6	0.7581494
BTBD9	0.7710526
BTC	3.1218362

BTD	3.6573222
BTF3	1.7076027
BTF3L4	1.9765251
BTG1	1.2163002
BTG2	3.334276
BTG3	1.5400331
BTK	0.2761526
BTN1A1	3.724778
BUB3	1.2381618
BUD13	1.083189
BUD31	1.9437635
BYSL	1.4515641
BZW1	2.3962953
BZW2	0.7111752
C1GALT1	1.7914512
C1GALT1C1	1.9006302
C1QA	2.0641377
C1QB	1.9021208
C1QBP	2.8184907
C1QC	1.609268
C1QL3	2.2120235
C1QTNF1	0.8409989
C1RA	4.2563467
C1RB	3.4817126
C1RL	4.9609575
C1S1	2.931946
C1S2	2.8937666
C2	4.0796986
C2CD2	1.8593307
C2CD2L	2.2231576
C3	3.7652035
C4B	4.2947817
C4BP	10.6496315
C8A	4.7938757
C8B	5.4072266
C8G	5.0531435
C9	4.912554
CAAP1	1.6968987
CAB39	0.68304324
CAB39L	1.4200737
CABIN1	0.65797067
CABLES1	2.1014478
CABLES2	1.5595667
CABP5	0.560436
CABYR	2.9464502

CACFD1	2.2055004
CACTIN	1.4133976
CACUL1	2.28466
CACYBP	1.5664742
CAD	1.0541828
CADPS2	0.84618354
CAGE1	0.39277935
CALD1	1.1695826
CALM1	1.0563681
CALM2	0.7973287
CALM3	0.34334064
CALR	3.171757
CALR3	1.6038158
CALU	1.9645483
CAMK1D	0.7785821
CAMK2B	1.9852376
CAMK2D	0.33762145
CAMK2G	0.3442762
CAMK2N1	0.5226593
CAMK2N2	2.8932045
CAMKK1	0.27852535
CAMKMT	0.4796121
CAML	2.113086
CAMSAP1	1.4164736
CAMSAP3	1.3404
CAMTA2	2.0206382
CAND1	1.4743602
CANT1	1.7924447
CANX	1.8752892
CAP1	0.74001384
CAPG	0.3186853
CAPN10	1.041106
CAPN7	1.2701247
CAPNS1	0.80943847
CAPRIN1	1.2733729
CAPZA1	2.9723852
CAPZA2	1.8042214
CAPZB	1.8314598
CAR13	3.1800783
CAR4	0.46820092
CAR5A	2.365781
CAR7	1.0902507
CARD14	2.005852
CARHSP1	2.091398
CARM1	1.6325104

CARNS1	0.752208
CARS	2.7920625
CARS2	1.4514787
CASC3	1.5806739
CASK	0.63549876
CASP1	1.126548
CASP2	0.69609857
CASP4	2.5430982
CASP6	1.3713305
CASP7	2.4249246
CASP8	1.9633458
CASP9	0.7215693
CAST	2.45531
CATSPER2	2.3341806
CBFA2T2	0.84335303
CBFB	0.7776158
CBL	0.86088014
CBLB	1.4766839
CBLC	4.0635185
CBLL1	1.0160549
CBR1	1.2456019
CBR4	2.8788188
CBS	5.870227
CBWD1	1.4921267
CBX1	0.8009708
CBX2	1.5182054
CBX3	0.59820247
CBX4	0.5204046
CC2D1A	0.98372865
CC2D1B	1.6257331
CCAR1	2.0341356
CCAR2	1.1682336
CCBL1	5.181386
CCBL2	5.4173193
CCDC101	1.8346622
CCDC104	1.2060459
CCDC106	1.4130309
CCDC107	0.41152263
CCDC115	0.43186355
CCDC117	1.4730151
CCDC12	0.7985265
CCDC120	1.9995897
CCDC124	0.99569345
CCDC125	0.90410423
CCDC126	0.88461757

CCDC127	0.7181721
CCDC132	1.5476682
CCDC134	1.2701161
CCDC137	0.6104958
CCDC138	3.1221793
CCDC144B	0.71222067
CCDC159	0.56211233
CCDC163	1.740278
CCDC167	0.8614366
CCDC174	0.7451513
CCDC22	2.19301
CCDC23	0.91196895
CCDC25	2.3313158
CCDC28A	0.40246892
CCDC32	0.96504354
CCDC43	1.6956661
CCDC47	1.8824747
CCDC53	1.9839675
CCDC55	1.8879738
CCDC58	3.3516614
CCDC59	1.3661454
CCDC6	1.4543579
CCDC66	1.5175626
CCDC68	0.50571847
CCDC71	1.0562747
CCDC71L	1.5753152
CCDC77	0.6413982
CCDC82	1.1571925
CCDC86	2.478369
CCDC88B	1.0048754
CCDC9	0.8606062
CCDC90B	0.5816972
CCDC93	3.1034565
CCDC97	1.0619271
CCIN	2.515322
CCL11	0.95827556
CCL19	2.7594607
CCL2	6.831405
CCL3	4.173873
CCL4	3.716564
CCL5	0.95115066
CCL7	4.8887434
CCL9	4.6548605
CCM2	1.3699739
CCNC	1.182415

CCNDBP1	0.7845638
CCNE1	2.494014
CCNF	0.4626093
CCNG1	0.7727039
CCNG2	1.8415093
CCNH	0.7939203
CCNI	1.344506
CCNK	2.0100358
CCNL1	2.9537938
CCNL2	2.4648473
CCNT1	1.4767909
CCNT2	1.4678421
CCNYL1	1.2966468
CCP110	1.2943375
CCPG1	1.2001207
CCPG1OS	1.0378573
CCR5	0.6052544
CCR8	1.9520774
CCR9	0.7146504
CCRL2	2.7141736
CCRN4L	2.8269737
CCS	2.023711
CCSER2	0.8016579
CCT2	2.1055357
CCT3	2.3556225
CCT4	1.8330524
CCT5	1.587302
CCT6A	2.0184891
CCT7	1.4157059
CCT8	2.444564
CCZ1	1.6322806
CD14	7.706579
CD164	2.1221888
CD1D1	2.8238242
CD1D2	2.1882107
CD274	3.061506
CD28	2.6459744
CD2AP	1.4340737
CD2BP2	1.2339551
CD300E	2.2798626
CD300LF	1.9699223
CD300LG	1.6320527
CD302	5.308568
CD320	0.87674165
CD33	2.441262

CD38	3.9669635
CD3EAP	1.9514792
CD40	0.5525811
CD47	2.2938173
CD48	0.31588054
CD53	0.4005525
CD55	1.7633402
CD59A	0.6526854
CD5L	4.632515
CD63	1.7921107
CD68	0.72185683
CD81	0.6038201
CD82	1.2178547
CD86	1.0731969
CDADC1	0.58055806
CDC123	2.1406171
CDC14B	2.0705488
CDC16	1.3900635
CDC25A	2.301957
CDC26	1.0523093
CDC34	2.224941
CDC37	0.76668334
CDC37L1	1.7359698
CDC40	1.4777725
CDC42	1.1945732
CDC42EP2	1.2809985
CDC42EP4	0.5569937
CDC42EP5	2.5431144
CDC42SE1	0.54056
CDC42SE2	0.3908608
CDC5L	1.1443555
CDC73	1.1750243
CDCA4	1.264272
CDCP2	0.9280131
CDH2	2.096164
CDH3	3.6125872
CDH6	0.43807006
CDHR2	0.78860116
CDHR5	3.9306643
CDIP1	0.6523807
CDIPT	0.7735293
CDK10	1.3486412
CDK11B	1.0211194
CDK12	1.3376851
CDK13	0.506613

CDK16	0.5912893
CDK17	1.757138
CDK18	2.6982033
CDK2	1.4536533
CDK20	2.3188121
CDK2AP1	1.179158
CDK2AP2	1.3925645
CDK4	1.1520336
CDK5	0.5447898
CDK5R1	0.92715263
CDK5RAP1	2.5883443
CDK5RAP2	1.040417
CDK5RAP3	2.6964643
CDK7	1.787133
CDK8	1.7810433
CDK9	1.5029309
CDKAL1	1.0043714
CDKN1A	2.834955
CDKN1B	0.61811805
CDKN2AIP	1.2265444
CDKN2AIPNL	1.5894692
CDKN2C	0.38085842
CDO1	4.9650764
CDPF1	0.90638757
CDS2	1.1503646
CDT1	1.4713404
CDV3	1.9007099
CDYL	1.5416224
CEACAM2	1.8490655
CEBPA	2.9727962
CEBPB	3.4491303
CEBPD	2.936046
CEBPG	2.9150345
CEBPZ	2.614042
CEBPZOS	0.7315514
CECR5	2.3260891
CELF1	1.9241803
CELSR3	1.8929281
CENPB	1.2791812
CENPJ	0.6866014
CENPL	2.1565866
CENPM	4.011133
CENPO	1.1952896
CENPQ	0.6554136
CENPT	0.690531

CENPV	1.8328269
CENPW	0.86950135
CEP112OS2	2.0001757
CEP120	1.5080383
CEP131	0.45053196
CEP162	1.5618136
CEP164	0.35873437
CEP170	1.2013113
CEP170B	3.4478526
CEP19	0.6816275
CEP192	1.3744719
CEP350	1.3776584
CEP55	3.2330577
CEP57	1.5526869
CEP63	1.483773
CEP83	1.069144
CEP85L	2.7910278
CEP95	1.9509361
CEP97	0.8233483
CEPT1	1.8011534
CERK	1.02898
CERKL	2.5638278
CERS2	1.6599476
CERS6	1.8041444
CES1B	8.894985
CES1C	9.720636
CES1D	2.5284927
CES1E	1.1961539
CES1G	1.4200876
CES2A	9.325131
CES2B	3.820802
CES2C	4.465068
CES2E	5.3235445
CES2G	2.214596
CES3A	6.5715714
CES3B	3.266421
CETN3	1.2222946
CFB	5.9501534
CFDP1	1.6696088
CFH	4.549362
CFHR1	8.524813
CFHR2	4.4097347
CFI	9.88666
CFL1	1.1927459
CFL2	1.7560856

CFLAR	1.9989631
CFP	2.3516314
CGREF1	3.7314303
CGRRF1	1.120363
CH25H	3.2851865
CHAC1	3.9566033
CHAC2	1.6951044
CHAF1B	0.70335937
CHAMP1	1.4849625
CHCHD1	2.095784
CHCHD10	3.0654364
CHCHD2	2.6427004
CHCHD3	2.0641344
CHCHD4	2.4546063
CHCHD5	2.3741505
CHCHD6	1.4274371
CHCHD7	1.5792029
CHD1	1.9595859
CHD1L	0.9203851
CHD2	1.5436203
CHD3	0.4457147
CHD3OS	0.33607435
CHD4	1.4193146
CHD7	2.775295
CHD8	1.4404314
CHD9	1.0425384
CHDH	2.639334
CHERP	1.342056
CHFR	1.2846205
CHIC2	1.9474967
CHID1	2.1009166
CHIL1	0.7352655
CHKA	3.7865822
CHKB	2.5329378
CHM	1.1977298
CHMP1A	1.4474208
CHMP2A	2.3910358
CHMP2B	1.2859342
CHMP3	0.9050381
CHMP4B	1.1917508
CHMP5	1.06826
CHMP6	1.6477201
CHMP7	1.6013348
CHN2	2.0260327
CHORDC1	0.51370215

CHP1	2.2215102
CHPF2	2.6839259
CHRAC1	2.2335174
CHRM3	1.8061302
CHST15	0.8559749
CHSY3	3.3947158
CHTF8	0.62183404
CHTOP	1.4811847
CHUK	3.1329854
CHURC1	2.527261
CIAO1	1.446224
CIAPIN1	2.1532881
CIART	1.0941603
CIB1	2.1294467
CIC	0.97274375
CIDEB	3.197727
CIDEC	1.5593193
CIPC	1.7059443
CIRBP	0.82974696
CIRH1A	2.5350707
CISD1	2.6147802
CISD2	1.6793015
CISD3	2.2010262
CISH	2.593908
CITED2	0.9283879
CIZ1	0.80604815
CKAP5	0.49473357
CKLF	0.43488383
CKMT1	0.5726106
CKS1B	1.3839395
CKS2	0.5460651
CLASP1	0.5790069
CLASRP	1.4366815
CLCA1	1.393307
CLCA2	2.5165293
CLCA4	1.6140265
CLCC1	1.4511235
CLCN2	2.2361748
CLCN3	1.3903282
CLCN4-2	2.349182
CLCN5	0.39781785
CLCN7	2.5114915
CLDN1	0.77826285
CLDN12	3.1811893
CLDN14	7.0430765

CLDN2	5.0428753
CLDN25	2.07877
CLEC16A	1.0597324
CLEC1B	0.5331404
CLEC2E	1.6816893
CLEC4A1	1.6896083
CLEC4D	1.6107938
CLEC4E	3.3974102
CLEC4F	0.48595214
CLEC4G	4.093074
CLIC1	1.0454981
CLIC4	0.8714378
CLINT1	1.9330575
CLIP1	1.514602
CLK1	1.7398002
CLK2	1.2021863
CLK4	2.107478
CLMN	1.9797513
CLN3	0.8629887
CLN5	2.5524933
CLN6	0.38502264
CLN8	3.1357114
CLNS1A	1.6971762
CLOCK	1.2124441
CLP1	1.2325323
CLPB	2.589639
CLPP	2.612252
CLPTM1	1.6732605
CLPTM1L	3.0691254
CLPX	3.4416158
CLTA	1.7638991
CLTB	1.7887018
CLTC	1.4370587
CLU	4.6516685
CLUAP1	0.49753547
CLUH	4.3204517
CLYBL	2.7129822
CMAH	1.5319202
CMAS	1.5743244
CMBL	0.92296386
CMC1	2.3517244
CMC2	0.47525668
CMIP	1.3204048
CML1	3.568413
CML2	1.7876914

CMPK1	2.266063
CMPK2	0.6443393
CMSS1	1.43665
CMTM6	0.6824248
CMTM7	0.26505685
CMTM8	1.7598159
CMTR1	2.0909379
CNBP	1.6231062
CNDP2	2.1155827
CNEP1R1	1.2833922
CNIH1	2.3452652
CNIH4	1.1081555
CNKSR3	2.7332623
CNN3	2.0950062
CNNM2	1.5989487
CNNM3	1.6502111
CNNM4	0.7602031
CNOT1	1.4962275
CNOT10	1.0751011
CNOT2	1.0524218
CNOT4	1.0981979
CNOT6L	2.3287804
CNOT7	1.1321707
CNOT8	0.77618194
CNPPD1	2.3282764
CNPY2	2.3059742
CNPY3	1.7006309
CNR2	0.82663655
CNTF	2.6264584
CNTNAP1	3.7288306
COA3	1.0838139
COA4	0.76832175
COA5	2.2068784
COA6	1.721947
COA7	4.2885866
COASY	2.4374583
COBLL1	1.548456
COG1	0.7062571
COG2	2.1311486
COG3	1.923909
COG4	0.88196635
COG6	0.9776566
COG7	1.4057724
COG8	0.7195976
COIL	1.3494477

COL11A1	1.0342178
COL17A1	1.7436261
COL18A1	4.432412
COL20A1	2.4414744
COL27A1	1.9598906
COL4A1	1.094384
COLEC10	4.15703
COLEC11	3.2445557
COMMD10	1.5941746
COMMD2	1.4654858
COMMD3	1.9441783
COMMD4	1.3661602
COMMD5	1.6975877
COMMD6	1.9273155
COMMD7	1.3986084
COMMD8	0.64222693
COMMD9	1.9338915
COMT	2.2620304
COMTD1	2.5649831
COPA	2.425195
COPB1	2.2640398
COPB2	2.867781
COPE	2.8278124
COPG2	0.39512897
COPRS	2.0298178
COPS2	2.4727151
COPS3	1.6414502
COPS4	1.8732202
COPS5	2.5281608
COPS6	2.1806986
COPS7A	1.8586576
COPS8	1.4530294
COPZ1	2.0089824
COPZ2	0.46271777
COQ10A	2.0192406
COQ10B	2.556863
COQ2	1.7851741
COQ4	1.5263574
COQ5	2.507311
COQ6	2.0043652
COQ7	2.8077662
COQ9	2.3577821
CORO1B	1.3219903
CORO1C	1.5859649
CORO7	1.6368821

COTL1	0.6883838
COX1	1.7108297
COX11	2.930414
COX14	0.8216393
COX15	1.0255525
COX16	1.8310173
COX18	3.11775
COX19	1.602411
COX2	2.1240358
COX20	1.2285812
COX3	2.5423193
COX4I1	2.019441
COX5A	2.629934
COX5B	2.2449615
COX6A1	2.287449
COX6B1	1.6685274
COX6C	2.2811463
COX7A2	2.0987327
COX7A2L	1.395231
COX7B	2.5028002
COX7C	2.684683
COX8A	1.5712302
CP	6.0636826
CPB2	10.885954
CPD	0.46618962
CPEB2	2.7149165
CPEB3	1.7577636
CPEB4	1.4542534
CPLX2	0.46034408
CPN1	7.8551855
CPN2	4.2390575
CPNE1	0.44196677
CPNE2	1.4708159
CPNE8	3.681032
CPOX	2.9700692
CPPED1	2.2893865
CPQ	1.2612169
CPS1	8.693358
CPSF1	1.1931012
CPSF2	1.2879853
CPSF3	1.5910218
CPSF3L	1.6345842
CPSF4	1.707289
CPSF6	1.676008
CPSF7	1.9458334

CPT1A	3.0706303
CPT2	3.2098048
CR1L	1.6542985
CRADD	1.0192239
CRAMP1L	1.1349642
CRBN	1.291703
CRCP	4.0767555
CREB3	2.5302064
CREB3L2	2.106127
CREB3L3	11.944112
CREB5	3.2992709
CREBBP	1.1754944
CREBL2	1.6722062
CREBZF	0.7486074
CREG1	2.2552245
CRELD1	2.4958246
CRELD2	4.546502
CREM	3.298094
CRIPT	1.9158785
CRK	1.5791428
CRKL	1.5078027
CRLF2	1.266361
CRLF3	0.4703815
CRLS1	2.8025396
CRNKL1	1.9294403
CROT	3.0596907
CRP	8.293411
CRTC1	0.99271107
CRTC2	2.2702887
CRTC3	0.56293225
CRY1	2.6371086
CRY2	1.4358785
CRYBB3	7.492955
CRYZ	3.7334049
CRYZL1	1.8488863
CS	0.60891366
CSAD	0.36058164
CSDE1	0.9759629
CSE1L	1.9367058
CSF1	2.1813357
CSF1R	0.5740354
CSF2RA	1.2835801
CSF2RB	1.8874333
CSF2RB2	1.8697164
CSF3	4.684376

CSF3R	2.0599778
CSL	0.7907858
CSNK1A1	1.5658925
CSNK1D	1.6567037
CSNK1E	0.55721307
CSNK1G1	1.1540594
CSNK1G2	1.2110374
CSNK1G3	0.86695933
CSNK2A1	1.6063468
CSNK2A2	1.3863685
CSNK2B	1.6943309
CSPP1	1.3771212
CSPRS	0.68196464
CSRNP1	2.8567874
CSRP2	1.2216079
CSRP2BP	0.97489524
CSRP3	2.2875373
CST7	0.49253488
CSTB	3.3284276
CSTF1	2.2885273
CSTF2T	1.6576254
CSTF3	1.3343866
CTAGE5	2.7892659
CTBS	1.1844583
CTC1	0.62604403
CTCF	0.971966
CTCFLOS	6.370245
CTDNEP1	0.8362925
CTDP1	1.9551008
CTDSP1	1.3592665
CTDSP2	1.5026143
CTH	1.5979183
CTIF	2.9760664
CTLA2A	0.8459041
CTLA2B	1.3748109
CTNNA1	0.36277938
CTNNB1	0.5018203
CTNNBIP1	0.7000134
CTNNBL1	1.3491895
CTNND1	0.7069824
CTNS	2.4651606
CTPS	1.2171195
CTPS2	1.7648771
CTR9	0.578336
CTRC	0.43521428

CTSA	3.6108906
CTSB	2.491697
CTSC	1.9482534
CTSD	1.1964486
CTSH	1.1721146
CTSJ	7.9148207
CTSL	4.292238
CTSS	0.49677777
CTSZ	3.0958097
CTTN	1.8946555
CTU1	0.71434593
CTU2	1.8878
CUL1	2.141806
CUL2	2.003413
CUL3	1.5163729
CUL4A	2.1199105
CUL4B	2.1736162
CUL5	1.8070791
CUL9	0.6642144
CUTA	0.8960459
CUTAL	1.2798612
CUTC	0.97169137
CWC15	1.6370862
CWC22	1.5239546
CWC25	1.5033901
CWC27	1.9164231
CWF19L2	1.3298595
CXADR	1.97313
CXCL1	6.375225
CXCL10	5.5731926
CXCL11	2.0318587
CXCL12	0.9732058
CXCL13	5.2748384
CXCL16	0.7554281
CXCL2	10.168644
CXCL3	6.242814
CXCL5	3.547005
CXCL9	6.1391587
CXXC1	2.0779116
CXXC5	2.0645015
CYB561	1.6602333
CYB561D1	0.26319718
CYB561D2	2.7535808
CYB5A	1.1903651
CYB5B	1.2951729

CYB5D1	0.6619866
CYB5D2	1.1976063
CYB5R1	0.8816154
CYB5R4	1.1511219
CYBA	0.87352586
CYBB	2.1820943
CYC1	2.393275
CYCS	2.8786585
CYFIP1	1.2306454
CYHR1	0.47299027
CYLD	0.4584
CYP17A1	0.60152316
CYP1A2	5.4915733
CYP1B1	1.9333365
CYP20A1	0.9982183
CYP27A1	4.1508493
CYP2A12	6.8975544
CYP2A4	4.670622
CYP2B13	3.1902342
CYP2C29	7.6084204
CYP2C37	7.055399
CYP2C38	4.3285236
CYP2C39	8.757366
CYP2C44	6.2028484
CYP2C50	7.4812565
CYP2C54	6.029004
CYP2C67	5.573121
CYP2C68	4.082613
CYP2C70	7.4702673
CYP2D10	10.581677
CYP2D11	5.2740545
CYP2D12	3.6752255
CYP2D13	2.4246964
CYP2D22	4.063798
CYP2D26	9.728181
CYP2D34	4.7728577
CYP2D37-PS	1.5645158
CYP2D40	3.719281
CYP2D9	4.3227987
CYP2E1	7.420993
CYP2G1	0.95250297
CYP2J6	2.9645016
CYP2J8	2.5480077
CYP2R1	1.3321083
CYP2U1	3.3033874

CYP39A1	0.26971698
CYP3A11	7.7958784
CYP3A13	7.490389
CYP3A16	7.3647976
CYP3A25	8.477789
CYP3A41B	10.766289
CYP3A44	3.3983743
CYP3A57	2.190779
CYP3A59	5.5186872
CYP4A10	4.4000206
CYP4A14	4.7905645
CYP4A31	3.5861971
CYP4A32	1.4705036
CYP4F13	3.1496527
CYP4F14	4.0326595
CYP4F15	3.2169034
CYP4F16	0.60456586
CYP4F17	1.3163288
CYP4V3	2.2449052
CYP51	2.9831538
CYP7A1	2.0230885
CYP7B1	3.077192
CYP8B1	1.8540895
CYPT4	2.2521763
CYTB	1.8087378
CYTH1	1.7489612
CYTH2	1.2709982
CYTH4	0.7610991
DAB2	0.6754265
DACT2	2.836373
DAD1	2.1752203
DAF2	1.7736428
DAGLB	0.8060925
DAK	4.9544334
DANCR	2.577552
DAP	4.449462
DAP3	2.7935536
DAPK3	0.9683459
DARS	0.83457637
DARS2	1.4668612
DAXX	1.720391
DAZAP1	1.8933966
DAZAP2	1.1206138
DBF4	0.6305127
DBI	2.6517155

DBN1	2.7671406
DBNDD2	0.67125916
DBNL	1.2860887
DBR1	0.9835596
DBT	2.3403847
DBX2	2.437096
DCAF10	1.641247
DCAF11	2.2046149
DCAF12	1.3120081
DCAF12L1	1.6462836
DCAF13	2.1562603
DCAF15	1.5936315
DCAF17	1.1629567
DCAF4	1.9269545
DCAF5	1.5808961
DCAF6	1.9003508
DCAF7	0.9798236
DCAF8	1.7004693
DCAKD	1.3412869
DCC	2.24556
DCHS2	0.592536
DCLRE1C	0.5887883
DCN	3.232237
DCP1A	1.0080674
DCP1B	1.6652591
DCTN1	0.6916611
DCTN2	1.4346292
DCTN3	1.4746172
DCTN4	0.70470595
DCTN5	0.80464387
DCTN6	0.6306999
DCTPP1	1.3590114
DCUN1D1	1.2133019
DCUN1D2	0.73710656
DCUN1D4	1.9695146
DCUN1D5	1.8766563
DDA1	1.2583063
DDB1	1.8168652
DDC	3.4253323
DDHD1	1.2716556
DDHD2	0.4682386
DDI2	3.0715535
DDIT3	1.577719
DDIT4	3.1628435
DDO	0.3697827

DDOST	2.0066493
DDRGK1	2.1756465
DDT	3.7805102
DDX1	2.7614954
DDX10	1.991987
DDX17	0.71597123
DDX18	1.4739549
DDX19B	1.9855335
DDX20	1.7549102
DDX21	1.415067
DDX24	1.731179
DDX27	1.3764188
DDX28	5.5980463
DDX31	0.7447207
DDX39	2.3815339
DDX39B	1.6776998
DDX3X	1.8877308
DDX41	1.7422106
DDX42	1.1730688
DDX46	0.9404237
DDX47	1.145994
DDX49	2.4323566
DDX5	1.1712825
DDX50	1.8593748
DDX51	1.2489512
DDX52	2.1427977
DDX54	2.1475217
DDX56	1.3175209
DDX58	1.3350194
DDX59	0.62797904
DDX6	0.9466703
DDX60	1.7992768
DEB1	2.1658232
DECR1	3.3294322
DECR2	2.9652264
DEDD	1.3374393
DEDD2	2.7108858
DEF8	1.053921
DEFB1	1.4854538
DEFB15	0.37920356
DEFB34	1.4906397
DEGS1	1.4997485
DEK	0.7019174
DENND1A	1.6942141
DENND1B	1.2167802

DENND2D	1.1535099
DENND4A	3.091439
DENND5B	2.0424583
DENND6A	0.62978196
DENND6B	1.3724139
DENR	1.9379914
DEPDC7	2.5030699
DERA	1.9637086
DERL1	3.0239923
DERL2	2.7307436
DERL3	3.2053034
DESI1	1.9044464
DESI2	2.565344
DET1	1.2244103
DEXI	1.2210791
DGAT1	1.93662
DGAT2	5.913329
DGCR14	1.0704615
DGCR2	1.314935
DGCR6	0.3955977
DGCR8	1.2485671
DGKD	0.99906135
DGKE	0.32792926
DGKQ	1.834419
DGUOK	0.5783212
DHCR24	2.39275
DHCR7	3.8447378
DHDDS	2.201431
DHDH	1.9780171
DHODH	1.2819953
DHPS	1.5103123
DHRS11	1.4140825
DHRS13	3.7009676
DHRS4	3.1351745
DHRS7	0.35836864
DHRS7B	0.6159489
DHRSX	2.662358
DHX15	2.2723591
DHX16	1.0991514
DHX29	0.8576071
DHX30	1.5500333
DHX32	2.2310998
DHX33	1.1824343
DHX34	0.3637588
DHX35	1.5337651

DHX36	1.9230521
DHX38	1.7334235
DHX40	2.4084108
DHX57	0.9655688
DHX58	2.7309315
DIABLO	2.2997577
DIAP2	1.2553184
DIDO1	0.8555505
DIEXF	1.0461109
DIMT1	2.0127919
DIO1	2.7747169
DIP2C	0.8449676
DIRC2	0.680537
DIS3	2.7738292
DIS3L	1.6017187
DIS3L2	1.4297698
DKC1	1.4847829
DLAT	0.87888074
DLD	3.1049674
DLG1	0.48784184
DLGAP4	1.5987394
DLST	2.5530875
DLX3	1.2993591
DMAP1	1.472089
DMBT1	1.4178665
DMGDH	11.394365
DMPK	0.85920644
DMTF1	2.7574294
DMTN	3.2469823
DMWD	2.2386248
DMXL1	1.6033295
DMXL2	2.1802866
DNA2	1.3458426
DNAAF2	0.93238926
DNAH2	2.9430683
DNAJA1	1.5120747
DNAJA2	2.290993
DNAJA3	3.903744
DNAJB1	0.83804965
DNAJB11	2.7407434
DNAJB14	0.72907853
DNAJB2	2.8621619
DNAJB6	1.5042698
DNAJB9	2.9744575
DNAJC1	1.5259206

DNAJC10	1.4190881
DNAJC11	0.64927506
DNAJC12	3.8652952
DNAJC13	2.345771
DNAJC14	0.95426345
DNAJC15	2.3394015
DNAJC16	2.4240634
DNAJC18	1.9441521
DNAJC19	1.7429817
DNAJC2	1.4397709
DNAJC21	1.8866456
DNAJC22	4.5172405
DNAJC24	1.3541353
DNAJC25	3.12558
DNAJC28	0.8607652
DNAJC3	2.7883418
DNAJC30	1.9629977
DNAJC5	1.7928784
DNAJC7	1.0290091
DNAJC8	0.47430348
DNAL4	0.70087695
DNASE1L3	1.4235632
DNASE2B	1.1625087
DNLZ	2.5292275
DNM1L	1.4125278
DNM2	1.8280146
DNPEP	2.3858802
DNPH1	1.2038989
DNTT	2.0544248
DNTTIP1	1.6581933
DNTTIP2	1.680476
DOCK5	1.5202386
DOHH	1.5838826
DOK2	1.5594609
DOK3	0.96669126
DOLK	1.269057
DOLPP1	1.4851148
DONSON	2.8248866
DOPEY2	1.906266
DOS	1.4416316
DOT1L	2.3372657
DPAGT1	2.2203877
DPCD	0.72265077
DPEP3	1.4379025
DPF2	1.2292187

DPH1	2.5228884
DPH2	2.11847
DPH5	2.4643219
DPH6	1.3482165
DPH7	0.9525237
DPM1	1.1560519
DPM2	1.465442
DPM3	1.0196974
DPP10	0.7429776
DPP3	2.2094247
DPP6	2.4032567
DPP7	2.3695142
DPP8	0.94530416
DPP9	3.6655304
DPY19L1	3.5032165
DPY19L2	0.6898751
DPY30	1.1868675
DPYD	5.2608957
DPYSL5	2.9302366
DR1	1.6433899
DRAM2	1.1358964
DRG1	1.9556329
DRG2	1.93202
DROSHA	0.8138249
DSC2	5.935005
DSCR3	1.6699097
DSE	1.0033634
DSG2	1.9347892
DSN1	0.42273712
DSP	0.94818044
DST	1.503391
DSTN	2.0316174
DTD2	0.7764914
DTNBP1	2.471791
DTWD1	1.4107444
DTX2	1.8465083
DTX3	0.27429795
DTX3L	1.5645335
DTYMK	1.1427662
DUOX2	2.169568
DUOXA1	1.8289442
DUOXA2	4.605796
DUS1L	2.3909485
DUS2	3.5824215
DUS3L	1.3956072

DUSP1	2.0091598
DUSP10	2.4224293
DUSP11	0.8432579
DUSP12	2.0392115
DUSP16	2.7781007
DUSP2	2.9185836
DUSP22	0.2933128
DUSP28	0.83579135
DUSP6	1.1703956
DUSP8	3.10658
DVL1	2.4581106
DVL2	2.0887492
DVL3	1.0193269
DXO	2.1089923
DYM	0.3607185
DYNC1H1	1.7487252
DYNC1I2	0.87790513
DYNC1LI1	1.3066332
DYNC1LI2	0.4392047
DYNLL2	1.4024236
DYNLRB1	0.8001406
DYNLT1A	0.41869497
DYNLT1C	0.5779145
DYNLT1F	0.61043906
DYNLT3	0.8799851
DYRK1A	1.9219391
DYRK2	1.2261467
DYRK3	0.9629538
E2F3	2.1450264
E2F5	1.5612531
E2F6	1.4228518
E2F8	0.75925493
E4F1	1.0068924
EAF1	2.24146
EAF2	4.362053
EAPP	2.6371682
EARS2	0.9114053
EBAG9	2.0789354
EBI3	1.8998406
EBNA1BP2	1.9139946
EBP	2.409064
EBPL	4.008196
ECD	1.6423118
ECE2	3.0511854
ECH1	2.3563917

ECHDC2	3.863868
ECHDC3	1.8909948
ECHS1	2.791948
ECI1	2.9777267
ECI2	2.9492214
ECI3	2.6396048
ECM1	1.4832561
ECSIT	1.8490255
EDC4	1.5824168
EDEM1	2.4611351
EDEM2	2.2686174
EDEM3	2.1823323
EDF1	1.7500169
EDN1	1.3983457
EDRF1	1.4275362
EEA1	1.5979488
EED	1.8509519
EEF1A1	1.9661205
EEF1B2	2.2002904
EEF1D	1.8786647
EEF1E1	2.093665
EEF1G	1.375274
EEF2	2.3846176
EEFSEC	1.3031318
EFCAB14	0.7904303
EFHD2	1.6558545
EFNA1	3.4061358
EFNA5	1.6080453
EFR3A	1.2100089
EFTUD1	1.933634
EFTUD2	1.408354
EGFR	5.638815
EGLN2	1.9769394
EGLN3	4.379937
EGR1	4.7928314
EGR2	1.3181565
EHBP1L1	0.42079473
EHD1	2.3840554
EHD3	0.4233644
EHHADH	4.455394
EHMT1	3.1974237
EHMT2	1.4244316
EI24	2.4130862
EID3	2.1545916
EIF1	1.7210853

EIF1A	3.7878773
EIF1AD	2.609092
EIF1AX	1.8050239
EIF1B	1.2595379
EIF1-PS	1.7024648
EIF2A	2.0520084
EIF2AK1	2.0445755
EIF2AK2	2.4135816
EIF2AK3	2.3863156
EIF2AK4	1.9458358
EIF2B1	3.0600069
EIF2B2	2.1933777
EIF2B3	2.4126728
EIF2B4	2.5518124
EIF2B5	1.770016
EIF2D	2.285834
EIF2S1	2.170431
EIF2S2	2.9245155
EIF2S3X	1.654669
EIF3A	2.228069
EIF3B	1.8287623
EIF3C	1.8796537
EIF3D	2.5671117
EIF3E	1.2327487
EIF3F	1.452543
EIF3G	2.3013837
EIF3H	1.1711648
EIF3I	1.9009788
EIF3J1	2.2738712
EIF3J2	1.4545853
EIF3K	0.92032695
EIF3L	1.6223156
EIF3M	1.8295681
EIF4A1	2.6982806
EIF4A2	1.0024087
EIF4A3	1.2922118
EIF4B	1.7397153
EIF4E	1.6393936
EIF4E2	2.4997141
EIF4E3	0.9703953
EIF4EBP1	3.2074769
EIF4EBP2	1.5621359
EIF4EBP3	5.0108614
EIF4ENIF1	1.7179396
EIF4G1	2.940642

EIF4G2	3.677982
EIF4G3	1.534703
EIF4H	1.3263123
EIF5	3.022689
EIF5A	2.3431065
EIF6	4.082076
ELAC1	0.47459364
ELAC2	1.6518619
ELAVL1	1.704746
ELAVL4	1.8902991
ELF1	0.6340554
ELF2	1.4591835
ELF3	3.4993637
ELK1	2.0119066
ELK4	0.7577183
ELL	3.1884625
ELL2	3.460871
ELMO3	0.6467354
ELMOD2	3.8180606
ELMOD3	0.9523394
ELOF1	1.0391495
ELOVL2	9.4166355
ELOVL3	3.675345
ELOVL6	1.9796131
ELOVL7	2.56822
ELP2	0.9471481
ELP3	1.4513075
ELP4	0.74695206
ELP5	1.6227281
EMC1	0.75063515
EMC2	1.4642684
EMC3	1.3525975
EMC4	1.48718
EMC6	2.2475321
EMC7	1.7509229
EMC8	1.1294372
EMD	0.9386251
EME2	1.8203576
EMG1	2.092376
EMILIN1	1.341397
EMILIN2	2.739362
EML3	0.690099
EML4	1.2825916
EML5	1.5376205
EML6	1.6517324

EMR1	2.6264174
EMR4	1.0019295
ENC1	3.4153326
ENDOG	3.3836992
ENDOV	1.2460914
ENGASE	0.4312737
ENO1	2.306412
ENO1B	1.9417822
ENO2	3.4322517
ENOX2	0.50320125
ENPP3	5.4129343
ENPP4	2.5450828
ENSA	0.6989739
ENTHD2	1.9354985
ENTPD1	0.3768084
ENTPD2	1.2584913
ENTPD4	1.0080578
ENTPD5	1.8503454
ENTPD6	0.80285215
ENTPD8	3.8421428
ENY2	2.534819
EP400	1.3025758
EPB4.1	1.6578176
EPB4.1L3	0.9336927
EPC1	0.39303994
EPC2	1.4273016
EPHA1	3.3256123
EPHA5	3.5640786
EPHB2	1.5794709
EPHB3	3.3060486
EPHX1	0.7332065
EPHX2	5.9157114
EPN1	2.3921373
EPPK1	4.726018
EPRS	3.567166
EPS15	1.3480003
EPS15L1	1.1195157
EPS8L3	2.95193
EPT1	1.3405831
ERAL1	1.9391854
ERAP1	2.8053896
ERBB2IP	1.073467
ERBB3	0.7790406
ERCC2	1.5613163
ERCC3	1.5898659

ERCC4	1.0748675
ERCC5	1.7737145
ERCC6L2	1.164274
ERCC8	0.8782313
ERDR1	2.490557
EREG	4.37996
ERF	1.1841033
ERGIC1	0.9626672
ERGIC2	2.369302
ERGIC3	0.387254
ERH	1.853965
ERI1	1.4087641
ERI2	0.5449219
ERI3	0.7806523
ERICH1	0.3826108
ERICH5	1.3902235
ERLEC1	1.4138834
ERLIN1	2.5878484
ERLIN2	1.5425456
ERMARD	1.0930889
ERMN	2.122073
ERMP1	1.3716471
ERN1	1.2565782
ERO1L	2.7265518
ERO1LB	3.742699
ERP29	2.7419522
ERP44	1.1441548
ERRFI1	5.1740174
ESCO1	1.4209354
ESD	2.315162
ESF1	1.9982474
ESR1	1.0306454
ESR2	1.4923503
ESRP2	2.4619415
ESRRA	2.3877485
ETF1	2.7532718
ETFA	3.1691215
ETFB	2.6581008
ETFDH	3.8698895
ETHE1	1.7350094
ETNK1	0.7780318
ETNK2	6.6645546
ETNPPL	5.9576473
ETS2	2.9088638
ETV3	1.0540404

ETV4	0.640491
ETV6	2.814864
EVA1A	3.81306
EVI5	1.480335
EVI5L	0.8560431
EVL	0.6178944
EWSR1	0.68327165
EXD1	2.8023937
EXD2	1.1962306
EXO5	0.48991466
EXOC1	0.7111676
EXOC2	0.95939183
EXOC3	0.5224793
EXOC3L4	0.83367133
EXOC5	1.3807342
EXOC6	1.2468936
EXOC6B	1.8415802
EXOC7	1.1143882
EXOSC1	1.0742586
EXOSC10	0.32579732
EXOSC2	2.2365894
EXOSC3	2.1381347
EXOSC4	2.6872747
EXOSC5	1.5516851
EXOSC6	1.3667052
EXOSC7	0.6018269
EXOSC8	0.53102994
EXOSC9	3.4310505
EXT1	1.5141618
EXTL1	1.7439876
EXTL2	1.8224671
EXTL3	1.1255543
EYA1	3.5262702
EYA3	2.3570597
EZH1	0.87789655
EZH2	0.99845886
F10	6.367607
F11	8.4922495
F11R	1.9892647
F12	12.398761
F13B	9.064068
F2	10.92795
F5	4.538271
F7	3.4095504
F9	3.6609108

FABP1	5.347333
FABP2	4.419382
FABP4	2.9735582
FABP5	0.30802703
FABP7	4.898073
FADS1	0.88881946
FADS2	1.6980114
FAF1	1.551254
FAF2	3.0687215
FAH	4.496276
FAHD1	1.2186162
FAHD2A	1.2427447
FAIM	1.2665002
FAM102A	0.51628804
FAM107B	0.52494645
FAM114A1	3.0533087
FAM114A2	1.1480515
FAM115A	0.40516496
FAM117B	0.36130548
FAM118A	2.3330452
FAM118B	1.1887882
FAM120A	1.7178161
FAM122A	1.429281
FAM126B	0.43603253
FAM133B	0.35331225
FAM134A	2.8072097
FAM134B	4.4752455
FAM134C	1.1081212
FAM135A	2.7861984
FAM136A	3.1019747
FAM13B	0.5387542
FAM151B	0.6198275
FAM160A2	2.4078276
FAM160B1	1.3836138
FAM160B2	3.072448
FAM162A	3.1370866
FAM167B	3.2607782
FAM168A	0.36849093
FAM168B	1.0742404
FAM169B	4.6247725
FAM172A	1.4768021
FAM173A	1.0500357
FAM173B	1.9870732
FAM174A	0.7311013
FAM178A	3.0216577

FAM179B	0.8416016
FAM185A	2.441533
FAM187B	3.6261528
FAM188A	1.5171177
FAM192A	1.5520895
FAM193A	1.2074606
FAM193B	0.92283654
FAM195A	2.499284
FAM195B	0.7898972
FAM199X	2.681633
FAM19A2	3.962965
FAM203A	1.5655024
FAM204A	1.1422944
FAM206A	0.51781154
FAM207A	1.4924448
FAM208A	1.309377
FAM208B	1.8626969
FAM20A	2.833769
FAM20C	1.7282574
FAM21	2.5513752
FAM210A	1.6591504
FAM210B	1.3882811
FAM213A	3.084758
FAM213B	0.5118387
FAM214A	0.95622754
FAM214B	1.5049846
FAM219A	3.0374782
FAM220A	0.51981044
FAM222A	3.1604683
FAM26F	3.1224787
FAM32A	1.3355005
FAM35A	1.6841483
FAM3A	0.5323646
FAM3C	1.261286
FAM43A	0.45658946
FAM45A	1.394975
FAM46A	2.8239324
FAM50A	2.9332392
FAM53A	1.4509151
FAM53C	1.9043858
FAM58B	1.1437237
FAM63A	1.6431391
FAM63B	1.0508401
FAM71D	2.3207045
FAM71E1	0.9400494

FAM71F2	1.2480843
FAM73A	0.5886376
FAM73B	1.6578367
FAM76A	1.2555082
FAM76B	1.0319788
FAM83F	0.85349154
FAM83G	1.4926741
FAM83H	2.5097
FAM86	1.4264281
FAM92A	2.8214333
FAM96A	2.3068721
FAM96B	2.207248
FAM98A	1.9575632
FAM98B	1.2217135
FAM98C	0.91776586
FANCC	1.8576386
FANCE	1.4715402
FANCF	2.337866
FANCL	1.3634427
FANCM	1.1080596
FAP	1.123755
FARP1	1.1076205
FARP2	2.4276512
FARS2	2.2889879
FARSA	1.4205492
FARSB	2.3253915
FAS	2.8146784
FASTK	1.7990491
FASTKD1	2.600178
FASTKD3	2.788514
FASTKD5	2.4315202
FAT1	1.1747439
FAU	1.9947548
FBL	2.060249
FBP1	9.97539
FBRS	0.9198966
FBRSL1	1.0429466
FBXL12	1.3164041
FBXL12OS	2.378051
FBXL14	0.67246604
FBXL15	2.0926034
FBXL17	1.3834083
FBXL18	1.4815068
FBXL19	0.3119533
FBXL3	1.0734451

FBXL4	1.189194
FBXL5	2.0653226
FBXL6	1.0586007
FBXL8	0.57792234
FBXO11	0.92210317
FBXO18	0.7926152
FBXO21	1.1685951
FBXO22	1.7462947
FBXO3	1.0894091
FBXO30	0.74056363
FBXO31	4.513278
FBXO33	0.9740169
FBXO34	1.2000425
FBXO38	1.6781299
FBXO4	1.9308155
FBXO42	2.4914405
FBXO46	1.3618119
FBXO6	3.7179606
FBXO7	1.8594358
FBXO8	2.1454456
FBXO9	1.4282153
FBXW11	1.8807557
FBXW17	0.75037694
FBXW4	1.2308233
FBXW5	2.072347
FBXW7	0.37753844
FBXW8	0.7138264
FBXW9	1.7033827
FCER1G	1.8337753
FCF1	1.6950428
FCGR1	0.58020186
FCGR2B	2.467879
FCGR3	1.76671
FCGR4	1.3164728
FCGRT	0.7114465
FCHSD1	1.4482114
FCHSD2	2.3444088
FCNA	4.114485
FCRL5	0.9772055
FDFT1	2.5083945
FDPS	3.092683
FDX1	1.4818704
FDX1L	1.9247172
FDXACB1	1.9163649
FDXR	0.8016932

FECH	0.93383193
FEM1A	1.8584425
FEM1B	0.45315862
FEM1C	1.7693679
FEN1	1.1001112
FERMT2	0.9751532
FERMT3	1.229676
FERT2	1.9956591
FETUB	8.128246
FEZ2	1.5303051
FEZF2	0.86075354
FGA	9.173074
FGB	9.663299
FGD4	1.7249825
FGF21	6.048996
FGF23	1.1005595
FGFR1OP	1.6322367
FGFR1OP2	0.6282642
FGFR2	0.5437572
FGFRL1	1.7719676
FGG	10.698914
FGGY	0.6570842
FGL1	10.607961
FGL2	0.7316201
FGR	0.8231394
FH1	4.7726097
FHL3	0.7099397
FIBP	0.9415505
FIGNL2	1.4510999
FIP1L1	1.7195871
FIS1	1.5829175
FITM2	1.8346059
FKBP1A	1.3964064
FKBP2	2.423466
FKBP3	1.1432254
FKBP5	3.2987092
FKBP8	2.5948503
FKBPL	0.4793861
FKRP	1.8713415
FLAD1	3.3471067
FLCN	2.7167408
FLNB	1.6539505
FLOT1	2.2800238
FLOT2	2.1586225
FLYWCH1	0.51705766

FMNL2	0.48836994
FMO4	4.1142626
FMO5	2.6072586
FMR1	1.0755551
FN1	3.496521
FN3KRP	0.45072365
FNBP1	1.2782681
FNBP4	1.315593
FNDC3A	2.3424027
FNDC3B	3.4231164
FNDC4	5.1374073
FNIP1	2.71297
FNIP2	2.2011745
FNTA	0.78771806
FOCAD	2.2400196
FOLH1	1.7909575
FOPNL	1.0461085
FOS	1.4654324
FOSL1	4.559128
FOSL2	0.41616464
FOXA1	0.34364104
FOXA3	6.658639
FOXC2	1.4060923
FOXJ3	1.0584295
FOXK1	0.7548454
FOXK2	1.205353
FOXL2	2.1293223
FOXN2	1.0951865
FOXN3	0.38025475
FOXO1	1.6384065
FOXP4	1.7485197
FOXQ1	2.4284866
FOXRED1	2.40999
FPGS	3.9653041
FPGT	2.003174
FPR1	2.0231125
FPR2	2.650594
FPR3	4.8898745
FRA10AC1	2.723912
FRAT1	2.0004718
FRG1	0.7897389
FRMD4B	1.0466228
FRMD8	0.6374295
FRMD8OS	1.7364154
FRRS1	3.280019

FRS2	1.3791001
FRYL	2.41069
FRZB	3.9842904
FSCN1	1.7838767
FST	5.615451
FTCD	8.865858
FTH1	2.606474
FTL1	1.8939514
FTSJ1	2.1239173
FTSJ3	2.3186066
FUBP3	1.6656334
FUCA1	0.7464211
FUK	1.3262713
FUNDC1	0.8658993
FUNDC2	0.98107743
FUOM	3.0601285
FURIN	2.293762
FXN	0.642534
FXR1	2.185891
FXR2	1.9495056
FXYD1	2.0771472
FYB	1.4813378
FZD4	0.37319493
FZD9	0.86435556
FZR1	1.1863534
G3BP1	2.265917
G3BP2	1.8445618
G6PC	7.3997917
G6PDX	0.8005941
GAA	1.8160326
GAB1	0.4916122
GAB3	2.4071777
GABARAP	2.5221527
GABARAPL1	2.3334434
GABARAPL2	1.999809
GABBR2	0.5601287
GABPA	1.9974186
GABPB1	1.1157072
GABPB2	0.7153194
GABRR1	1.3453147
GADD45B	1.635153
GADD45G	5.0261617
GADD45GIP1	2.4086616
GAK	1.8508642
GALE	2.6963456

GALK1	1.8525264
GALK2	1.3710387
GALNS	1.8792107
GALNT1	0.43646598
GALNT10	0.50089145
GALNT11	1.1652167
GALNT2	1.1511195
GALNT4	0.8696859
GALNT7	1.4091823
GAMT	0.7879112
GAN	0.7753458
GANAB	2.7512815
GANC	0.842026
GAPDH	3.1713672
GAPVD1	1.6210129
GAR1	2.3851998
GAREM	5.509163
GARS	2.3941896
GART	0.78237915
GAS2	2.0302749
GAS2L1	1.3211024
GAS2L3	0.8781557
GAS5	2.276758
GAS6	0.9676988
GAS8	0.8604615
GATA4	3.253361
GATA5	1.664952
GATA6	1.0458128
GATAD1	0.9681585
GATAD2B	0.47055125
GATC	1.113698
GATM	0.871732
GBA	4.0051527
GBA2	0.37561297
GBAS	0.5175953
GBE1	3.0248988
GBP10	1.964009
GBP11	4.2173767
GBP2	2.9137022
GBP2B	2.904524
GBP3	3.2754643
GBP4	0.9987099
GBP5	3.4932168
GBP6	4.0114565
GBP7	2.648053

GBP8	1.2710955
GBP9	2.523854
GC	10.400085
GCAT	3.6676872
GCC1	1.1487117
GCC2	2.0520318
GCDH	5.6809826
GCFC2	1.5029192
GCGR	5.146325
GCH1	5.000861
GCHFR	3.0887344
GCKR	9.840805
GCLC	2.0960224
GCLM	2.0708835
GCM1	1.2294447
GCN1L1	1.4976404
GCNT2	1.3930857
GCSH	1.7809708
GDA	1.0462921
GDAP10	3.4851153
GDAP1L1	0.96990585
GDAP2	2.6873071
GDE1	1.7830737
GDF15	6.4270935
GDF2	3.0460265
GDI1	0.8511579
GDI2	1.5346463
GDPGP1	1.0564387
GEM	0.84222436
GEMIN5	1.4151871
GEMIN6	1.8432384
GEMIN7	0.5805447
GEMIN8	2.0631063
GET4	0.8342221
GFER	3.3600214
GFM1	2.9884522
GFM2	2.1000192
GFOD2	0.9603045
GFPT1	1.7110035
GFRA1	4.4549656
GFRA4	0.5822146
GGA1	1.2291224
GGA2	1.7360284
GGA3	1.2034194
GGACT	1.1365225

GGCT	2.2147443
GGCX	1.9317863
GGH	0.69602656
GGNBP2	1.920718
GGPS1	0.48019004
GGT6	4.7603273
GHDC	1.731714
GHITM	3.0947793
GHR	1.3377254
GID4	0.38418126
GID8	1.5743778
GIGYF1	1.6004527
GIGYF2	1.819598
GIMAP9	1.9128978
GIN1	1.7134981
GINM1	1.9778192
GINS1	1.046993
GINS4	0.5148914
GIPC1	1.2503436
GIT1	0.8678715
GIT2	0.7427347
GJA1	1.3422887
GJA4	1.1672027
GJB1	4.216982
GJB2	8.530104
GJB4	2.129413
GJC3	1.8870319
GKAP1	1.580528
GKN3	0.6931789
GLA	1.4329603
GLB1	0.38072085
GLCE	3.4616945
GLDC	5.863675
GLE1	0.84563184
GLG1	1.4301364
GLIPR2	2.042092
GLMN	1.0999787
GLO1	2.5004895
GLOD4	1.4504302
GLOD5	1.1152077
GLRX	2.959205
GLRX2	2.185688
GLRX3	1.8272059
GLRX5	2.010318
GLS2	4.278488

GLT1D1	3.07106
GLT25D1	2.192146
GLT8D1	1.1507266
GLTPD1	1.8811066
GLTPD2	8.957153
GLTSCR2	1.1699827
GLUD1	1.5468867
GLUL	1.4650624
GLYAT	3.5042036
GLYCTK	4.46241
GLYR1	1.8038123
GM10015	1.9318197
GM10065	1.1956446
GM10094	1.4464543
GM10101	1.0064087
GM10134	0.4264741
GM10142	1.8298752
GM10228	3.0865967
GM10257	0.50515866
GM10272	0.8398342
GM10318	0.3977034
GM10324	0.9881189
GM10387	2.290199
GM10405	3.1046216
GM10420	1.7930298
GM10425	1.3755732
GM10461	1.3805516
GM10631	1.2674067
GM10634	2.0032618
GM10639	0.77342033
GM10640	1.7683427
GM10653	1.9789107
GM10658	5.3624616
GM10680	4.1029253
GM10693	1.5149462
GM10696	2.449503
GM10731	0.80546737
GM10734	3.0689528
GM10765	1.551122
GM10767	1.1910722
GM10768	8.932667
GM10791	1.0014286
GM10796	1.1556745
GM10827	3.45801
GM10839	2.255702

GM10845	1.7061346
GM10851	1.4249644
GM10856	1.5061033
GM10872	4.6514893
GM10910	1.4512558
GM10914	2.279069
GM11033	6.0114737
GM11127	1.695791
GM11213	0.37666106
GM11240	1.5291278
GM11449	1.23792
GM11559	1.0100117
GM11564	1.3952012
GM11783	1.6864743
GM11810	1.09218
GM11974	2.4308803
GM12060	2.7112243
GM12070	3.0434499
GM12216	0.7553103
GM12247	2.206135
GM12250	0.48244452
GM12271	0.46896672
GM12447	1.5817869
GM12503	2.5354025
GM12508	1.8559859
GM12618	1.8542788
GM12620	2.4188645
GM12693	1.4558809
GM12758	2.2942748
GM12816	1.7517252
GM12942	0.94186616
GM13023	0.4353764
GM1305	2.4072182
GM13194	0.90471673
GM13202	2.5043561
GM13215	1.4863694
GM13238	2.0763798
GM13315	3.8618124
GM13570	1.7721415
GM13770	1.5684121
GM13777	1.4642718
GM13826	1.328434
GM13889	0.6469033
GM13931	1.2897925
GM14010	2.3641677

GM14135	0.91986585
GM14288	1.553335
GM14322	1.4795372
GM14325	0.8918946
GM14326	1.1213238
GM14347	1.5836813
GM14391	1.1055682
GM14393	0.72846484
GM14410	1.2703006
GM14420	0.9647777
GM14431	1.9598558
GM14432	1.5265653
GM14440	1.9014413
GM14443	1.4296949
GM14548	1.4676912
GM14858	1.647022
GM15032	1.2275159
GM15056	2.919223
GM15340	1.2582107
GM15348	2.1444285
GM15421	2.4677665
GM15466	0.743541
GM15520	2.6768217
GM15645	2.3110268
GM15760	1.5447452
GM15772	1.7310545
GM15998	3.264672
GM16062	0.6856656
GM16340	2.05248
GM16381	1.0392048
GM16386	0.61167264
GM16515	0.6513765
GM16525	0.95906353
GM16853	1.9216456
GM17748	0.60481095
GM17757	1.5870721
GM17764	1.3504016
GM17821	2.4573796
GM1943	0.70225334
GM19840	1.1866062
GM20056	0.360708
GM2016	3.4822538
GM20746	1.8728349
GM20764	0.73789287
GM2085	1.0552711

GM2099	0.6496484
GM2138	1.2840021
GM21559	0.43176532
GM21944	1.2849357
GM2347	0.81142306
GM2379	2.1670516
GM2382	2.1079166
GM2436	2.11402
GM2518	0.94560266
GM2590	1.2061212
GM2696	0.7668059
GM2701	0.93432045
GM2814	3.02829
GM2848	1.6810634
GM3161	1.3276432
GM3219	0.81706953
GM3239	0.8348186
GM3258	0.89197946
GM3285	1.4066846
GM3289	3.2630908
GM3367	1.2717168
GM3383	0.59740543
GM3404	1.9594805
GM3414	1.5948477
GM3448	2.0112164
GM3455	0.29885387
GM3556	1.9573376
GM3776	1.7480161
GM3805	1.3361838
GM3893	0.89507484
GM4117	4.278537
GM4123	2.3859293
GM4130	1.2193921
GM4342	1.2445061
GM4349	0.64825916
GM4402	2.1327193
GM4470	1.1936114
GM4473	0.94779396
GM4673	1.2923112
GM4786	1.7219317
GM4788	5.0936737
GM4802	1.8652723
GM4832	1.518327
GM4841	3.984388
GM4858	0.9480424

GM4861	2.148243
GM4862	1.4986936
GM4889	1.2113893
GM4910	2.7905653
GM4943	1.6293757
GM4944	0.44433093
GM4951	4.883046
GM4952	5.4983006
GM4963	1.6731522
GM4971	2.477
GM4997	2.2769413
GM5039	1.7399857
GM5067	2.9396577
GM5068	1.0155275
GM5069	3.1469724
GM5079	0.7746346
GM5093	0.42840958
GM5114	1.8793416
GM5115	1.4441149
GM5136	1.5221226
GM5176	1.8184903
GM5396	1.5748079
GM5431	1.5439456
GM5445	1.2996604
GM5453	1.5972707
GM5481	0.8746207
GM5483	1.561239
GM5523	2.9308681
GM5528	0.6157968
GM5544	2.4858382
GM5595	0.5881932
GM561	1.3085048
GM5617	1.5011394
GM5637	3.261284
GM5643	1.0614088
GM5766	2.1833107
GM5767	0.58048916
GM5779	0.95214677
GM5785	2.5390823
GM5801	1.3537128
GM5848	0.8456085
GM5858	0.27958608
GM5878	0.8289139
GM5879	1.389199
GM5908	2.1824806

GM6109	1.5221016
GM6115	1.4962733
GM6155	1.43941
GM6195	0.5092733
GM6211	1.950546
GM6238	2.1119754
GM6251	1.2717874
GM6252	1.974397
GM626	3.4151728
GM6328	2.159418
GM6377	2.683047
GM6402	0.583776
GM6403	3.3596563
GM6404	1.4420812
GM6444	1.1324155
GM6498	2.2847035
GM6524	2.2708957
GM6525	1.4846132
GM6548	1.9992387
GM6570	1.3996456
GM6578	2.0611017
GM6579	1.2817461
GM6583	1.3771482
GM6607	2.0221236
GM6625	0.50225234
GM6658	1.4258604
GM6710	1.707876
GM6921	0.48927808
GM6934	0.81167245
GM6981	3.0324633
GM6987	1.1613088
GM6994	3.4554307
GM7111	2.0888762
GM7120	2.1699598
GM7160	1.2651048
GM7241	1.5993145
GM7265	1.4730752
GM7337	3.0192006
GM7555	1.1659052
GM7634	1.4211366
GM7664	1.2805498
GM7694	1.757307
GM7967	2.4168499
GM8210	0.81627584
GM8437	1.6632657

GM8526	0.55636907
GM8556	1.1069634
GM8580	2.136594
GM8675	0.7330451
GM8709	3.4090278
GM8738	2.837961
GM8801	1.2411788
GM8883	3.6651633
GM8884	1.7453041
GM8909	3.3594162
GM8994	2.4375365
GM8995	0.69957185
GM9049	1.1177075
GM9079	2.3946025
GM9178	2.8120134
GM9234	1.5204055
GM9385	1.9457042
GM9456	2.3518136
GM9457	1.843477
GM9706	3.8641264
GM9725	2.6383488
GM9731	1.1774852
GM9757	0.75123763
GM9802	1.1010652
GM9855	2.0230746
GM9861	1.8071618
GM9883	0.89294755
GM9889	1.3227174
GM9895	2.1775775
GM9920	1.3709323
GM9975	1.094459
GMCL1	1.4823487
GMDS	1.620465
GMEB1	0.9892893
GMEB2	1.6303895
GMFB	0.34906602
GMFG	0.6218965
GMIP	0.49348378
GMPPA	2.3164408
GMPPB	2.7896593
GMPR2	1.3240826
GMPS	1.2158296
GNA11	0.59110856
GNA12	0.79606795
GNA13	2.1331704

GNAI3	2.2496998
GNAS	1.0551617
GNAT1	6.68956
GNB1L	0.888855
GNB2	1.6527641
GNB2L1	1.8833714
GNE	2.849488
GNG12	2.7453077
GNG5	1.6354086
GNL1	0.86484647
GNL2	2.8740222
GNL3	3.3636696
GNL3L	0.59571385
GNMT	4.9071064
GNPAT	1.1737969
GNPDA1	1.771146
GNPNAT1	2.6701462
GNPTAB	0.35464406
GNPTG	1.4206054
GNS	2.1183531
GOLGA1	1.9634507
GOLGA2	1.4117582
GOLGA3	2.5906265
GOLGA4	2.4197214
GOLGA5	2.8461263
GOLGA7	1.4482515
GOLGA7B	2.5719988
GOLGB1	2.8867614
GOLIM4	1.9489548
GOLM1	1.1245229
GOLPH3	2.3138764
GOLPH3L	0.370929
GOLT1B	0.7528405
GOPC	1.649843
GORAB	1.530968
GORASP1	2.1309621
GORASP2	1.401943
GOSR1	1.4200723
GOSR2	1.6343853
GOT1	6.747291
GOT2	2.7400453
GP49A	2.0913026
GPAA1	1.7183118
GPALPP1	0.8692477
GPATCH1	0.81735015

GPATCH11	1.8499286
GPATCH2	1.4506657
GPATCH2L	1.5281484
GPATCH3	1.3412285
GPATCH8	0.8540833
GPBP1	1.7878292
GPBP1L1	0.9357455
GPC4	2.9070504
GPCPD1	4.0038986
GPD1	0.94979167
GPD2	2.2267916
GPHB5	1.0259128
GPHN	2.9933221
GPI1	2.321849
GPKOW	1.2493966
GPN1	0.9308326
GPN2	1.4687498
GPN3	1.0136778
GPR107	1.4189904
GPR108	0.57527375
GPR110	0.50026965
GPR137	1.4585869
GPR146	1.828047
GPR157	1.7564199
GPR171	0.8358259
GPR18	1.1035018
GPR19	1.7041934
GPR22	1.2124906
GPR26	0.8212588
GPR3	1.0362618
GPR31B	1.2426596
GPR6	1.1199973
GPR64	1.605799
GPR65	1.9475372
GPR84	2.8692658
GPR85	2.0548348
GPRC5B	1.7353585
GPRC5C	2.2363408
GPS1	2.1312468
GPS2	0.89783835
GPSM2	0.76158786
GPT	0.9742305
GPT2	6.5675936
GPX1	2.819398
GPX4	0.85455775

GRAMD1A	1.3728697
GRAMD1C	3.7392995
GRAMD3	2.0761201
GRAMD4	1.1815894
GRB14	0.6180713
GRB2	0.569473
GRB7	2.991441
GREB1L	2.3186939
GRHL1	0.47522497
GRHPR	5.1094866
GRINA	3.5709512
GRIPAP1	2.6229599
GRN	2.6480591
GRPEL1	3.7830288
GRPEL2	2.5140884
GRSF1	2.3280795
GRTP1	1.7616704
GRWD1	2.3181489
GSDMA	1.7794147
GSDMC	0.63530755
GSDMD	3.6129253
GSK3A	1.7256048
GSKIP	1.5888543
GSPT1	2.3105962
GSR	2.232166
GSS	3.2275689
GSTA2	0.7274513
GSTA3	0.6006
GSTK1	1.5373366
GSTM3	0.5367148
GSTM5	1.944196
GSTM6	2.3067653
GSTO1	0.36870217
GSTP1	3.0624635
GSTP2	3.2784092
GSTT1	0.5682795
GSTT2	2.614084
GSTT3	2.2097652
GSTZ1	3.3781016
GT(ROSA)26S	1.3195336
GTDC1	1.1622541
GTF2A1	1.2433445
GTF2A2	2.6575835
GTF2B	2.8070786
GTF2E1	2.1161497

GTF2E2	1.3500149
GTF2F1	1.8913038
GTF2F2	1.7914951
GTF2H1	2.562691
GTF2H2	1.3322828
GTF2H3	1.5734122
GTF2H4	1.5610797
GTF2IRD1	2.2500737
GTF2IRD2	0.75038195
GTF3C1	0.6745436
GTF3C2	1.8172953
GTF3C3	1.2321665
GTF3C4	1.6560667
GTF3C5	1.4638355
GTF3C6	1.9756048
GTL3	0.6092708
GTPBP1	0.5322244
GTPBP10	1.1951249
GTPBP2	1.8029425
GTPBP4	1.514117
GTPBP6	1.784147
GTPBP8	2.7581131
GTSE1	2.1697085
GUCA1B	0.6808276
GUCA2A	0.98642945
GUCA2B	3.2806308
GUCD1	3.985037
GUCY2C	1.488311
GUK1	0.86709714
GULO	6.48662
GUSB	1.102021
GVIN1	0.6692655
GXYLT1	0.74372697
GXYLT2	2.9422848
GYK	2.8730013
GYLTL1B	0.37333226
GYPC	0.5143707
GYS1	1.3336365
GYS2	8.119665
GZF1	2.0600164
GZMB	2.1843169
H13	2.6052363
H19	1.0365999
H1F0	1.2556252
H2AFJ	0.37011504

H2AFV	0.7728083
H2AFX	1.1747172
H2AFY	0.8464234
H2AFZ	0.775141
H2-D1	1.68367
H2-K1	2.2013087
H2-K2	2.0826836
H2-KE2	1.5557196
H2-KE6	1.9347694
H2-M2	1.9688518
H2-Q1	3.569917
H2-Q10	4.2580204
H2-Q2	2.17077
H2-Q4	2.4858372
H2-Q5	1.2864254
H2-Q7	2.1313124
H2-Q8	2.0186598
H2-T10	1.724117
H2-T23	2.0500963
H2-T9	1.275676
H3F3B	2.5819309
H6PD	4.620222
HAAO	4.0006313
HABP2	7.271488
HABP4	1.0370972
HACL1	6.177001
HADHA	2.2201922
HADHB	2.4939406
HAGH	2.5457199
HAL	5.5988207
HAMP	9.697153
HAMP2	2.673732
HAND2	1.1034248
HAO1	6.444278
HAO2	1.5663235
HARBI1	2.188656
HARS	2.5276315
HARS2	1.0022285
HAT1	1.804632
HAUS1	0.85972714
HAUS2	1.5004735
HAUS3	1.0626857
HAUS4	0.4604571
HAUS5	0.5765946
HAUS6	0.6179936

HAUS7	2.0194964
HAUS8	0.4480126
HAX1	3.1091392
HBA-A1	0.80455494
HBA-A2	1.75354
HBB-BH1	1.0061092
HBP1	2.2844589
HBS1L	2.7893288
HC	1.7227013
HCAR2	2.7161157
HCCS	1.90978
HCFC1	1.1883223
HCFC2	0.6982827
HCK	0.73631597
HCLS1	1.8962743
HDAC1	0.8725507
HDAC10	0.96432805
HDAC11	0.38737106
HDAC3	1.7109272
HDAC4	1.1891024
HDAC5	0.45646262
HDAC9	0.6153188
HDC	1.1024225
HDDC2	0.3302138
HDGF	1.4378502
HDGFRP2	1.455003
HDHD2	0.7110696
HDLBP	4.0435934
HEATR1	3.061773
HEATR2	1.6632779
HEATR3	2.2120235
HEATR5A	0.9841287
HEATR5B	0.5920279
HEATR6	2.1962645
HEBP1	2.313893
HECA	1.2375352
HECTD1	2.5352814
HECTD2	1.0671899
HECTD3	1.8625505
HECW1	3.7077
HELB	2.5407493
HELQ	1.6646931
HELZ	1.3477566
HELZ2	3.2011912
HEMGN	3.5492084

HERC1	1.2081678
HERC2	0.9729755
HERC4	2.0780942
HERC6	2.9121816
HERPUD1	3.1051667
HERPUD2	2.1779974
HESX1	1.3448997
HEXA	1.0272615
HEXB	1.6743114
HEXDC	0.9346721
HEXIM2	0.38223004
HFE	2.243643
HFE2	3.481314
HGD	3.2202098
HGF	1.431227
HGFAC	2.8764136
HGS	2.4194639
HGSNAT	2.9775908
HHEX	2.2304828
HIAT1	1.345206
HIATL1	1.5047314
HIBADH	2.448694
HIBCH	2.7565606
HIC1	0.52800846
HIC2	1.2686157
HID1	0.7518656
HIF1A	3.6812932
HIF1AN	1.480788
HIGD1A	2.4492486
HIGD2A	1.6067655
HILPDA	2.898554
HINFP	1.777689
HINT1	1.6927593
HINT2	2.5654643
HINT3	2.473735
HIP1R	1.7129734
HIPK1	0.5533445
HIPK2	0.33807445
HIPK3	1.9492176
HIRIP3	0.60106635
HIST1H1C	1.3534777
HIST1H2BA	1.8978908
HIST1H2BC	3.2152474
HIST1H2BM	2.3710144
HIST1H3A	3.157821

HIST1H3D	1.8892186
HIST1H4C	1.0911925
HIST1H4H	0.7032192
HIST1H4J	0.62619996
HIST1H4M	2.6728425
HIST2H2AA2	0.4550135
HIST2H3C2	0.766381
HIVEP1	1.2665937
HIVEP2	2.0230782
HK1	1.6873715
HK2	2.4469445
HK3	2.6721923
HLCS	0.8780067
HLF	0.6823218
HLTF	0.6870005
HMBS	1.3611095
HMCES	1.4661415
HMG20B	1.3336232
HMGA1	2.1493638
HMGB1	1.1062577
HMGB1-RS17	0.79283214
HMGB2	0.4416921
HMGB3	1.7842176
HMGCL	3.6737516
HMGCR	2.184234
HMGCS1	2.8840659
HMGCS2	3.7749026
HMGN1	0.42861247
HMGN2	0.55528045
HMGN5	0.9146693
HMGXB3	1.9399011
HMMR	1.0448191
HMOX1	5.644478
HMOX2	2.4809902
HMX3	0.6252272
HN1L	1.8711548
HNF1A	6.52866
HNF4A	5.859025
HNRNPA0	1.2741888
HNRNPA1	1.1812222
HNRNPA2B1	3.3853223
HNRNPA3	1.511666
HNRNPAB	2.034722
HNRNPD	1.7843864
HNRNPDL	0.96986604

HNRNPH1	0.6096604
HNRNPH2	1.6187041
HNRNPH3	0.8862853
HNRNPK	1.9570467
HNRNPL	0.8423066
HNRNPLL	1.5234468
HNRNPM	1.6570652
HNRNPR	1.2830245
HNRNPU	0.74188733
HNRNPUL1	0.9974382
HOGA1	3.0358212
HOMER2	0.98619103
HOMEZ	1.4720306
HOOK1	1.1430938
HOOK2	2.4956648
HOOK3	1.120189
HP	3.4040737
HP1BP3	0.833442
HPCA	2.1303627
HPD	7.113613
HPGDS	1.0873773
HPN	5.1795645
HPRT	1.7162559
HPS3	0.5811019
HPS4	2.4624789
HPS5	0.47522402
HPS6	0.32680774
HPSE	1.0246675
HPX	11.070635
HRAS	0.86681056
HRG	8.333714
HRSP12	3.0161002
HS2ST1	2.3914816
HS6ST1	2.6842942
HSBP1	1.4372232
HSD11B1	1.4727376
HSD17B10	1.9651849
HSD17B11	1.304755
HSD17B12	3.3849938
HSD17B13	7.844507
HSD17B2	3.438753
HSD17B3	2.5503278
HSD17B4	2.0365846
HSD17B6	2.4134462
HSD17B7	3.111306

HSD3B2	2.189029
HSD3B3	2.0162334
HSD3B7	3.7123253
HSDL1	0.88548493
HSDL2	3.139476
HSF1	1.2051919
HSF2	1.2753136
HSF5	1.9102848
HSP90AA1	1.4105356
HSP90AB1	1.0916808
HSP90B1	3.3142624
HSPA13	3.2906106
HSPA14	1.5121524
HSPA2	1.6000144
HSPA4	2.2572954
HSPA4L	0.9111974
HSPA5	3.784299
HSPA8	2.19788
HSPA9	4.2715216
HSPB11	1.6278265
HSPB8	4.5944576
HSPBP1	1.8243945
HSPD1	3.3986251
HSPE1	2.925245
HSPH1	0.9959905
HTATIP2	2.8162444
HTATSF1	0.68010974
HTR7	1.0395055
HTRA2	0.91226983
HTRA4	1.171119
HTT	2.206896
HUNK	0.3349061
HUS1	0.80982685
HUWE1	1.5111616
HYAL2	1.0418041
HYI	2.5400422
HYKK	1.5340626
HYOU1	2.999829
HYPK	1.6226027
IAH1	1.5094898
IARS	2.487347
IARS2	2.0054648
IBA57	2.1894972
IBTK	4.5030193
ICA1L	1.7492568

ICAM1	1.8903582
ICMT	1.374327
ICT1	1.8399165
ID2	2.6432893
ID3	1.0045273
IDE	1.9871509
IDH1	0.9125352
IDH2	0.49726987
IDH3A	2.1086576
IDH3B	2.083503
IDH3G	1.441417
IDI1	2.347223
IDNK	2.4612806
IDO2	2.240981
IDUA	0.68302226
IER2	1.5854781
IER3	3.555692
IER3IP1	1.3337214
IFFO1	0.91364956
IFFO2	2.5101712
IFI202B	2.863849
IFI204	3.1151063
IFI27	2.4076455
IFI27L2B	2.5072315
IFI35	2.1794574
IFI44	2.2995913
IFI47	3.0770319
IFIH1	2.0148184
IFIT1	2.1584651
IFIT2	2.8731453
IFIT3	1.4300606
IFITM1	1.3079708
IFITM2	3.721876
IFITM3	3.069442
IFITM5	1.0605152
IFITM6	1.3429258
IFITM7	3.7001379
IFNA1	0.6604078
IFNAR1	2.2363026
IFNAR2	2.9784367
IFNG	3.7474668
IFNGR1	0.4965694
IFNGR2	1.5265715
IFRD1	3.1016262
IFRD2	1.8785179

IFT122	0.64787984
IFT20	1.8946459
IFT22	0.44071436
IFT27	0.4046347
IFT46	1.2341082
IFT52	1.7129242
IFT57	1.1321771
IFT80	0.9225452
IFT88	1.2114723
IGBP1	1.4183257
IGDCC4	4.192271
IGF1	3.9057434
IGF2BP2	0.9959264
IGF2BP3	2.3987372
IGF2R	0.4375975
IGFALS	1.2220869
IGFBP1	12.432986
IGFBP2	1.5694821
IGFBP4	2.0461376
IGFBP7	0.47461486
IGHMBP2	0.861572
IGSF11	3.9850047
IGSF23	4.0933065
IGSF5	2.0128596
IGSF6	2.0419066
IGSF8	2.351958
IGSF9	3.5867474
IGTP	3.3637202
IIGP1	4.1008205
IK	1.8527915
IKBKAP	1.2649825
IKBKB	1.9699466
IKBKE	4.3790684
IKBKG	3.024995
IKZF5	0.9821329
IL10	2.288108
IL10RB	0.50256133
IL11	2.8894494
IL12RB1	0.99376583
IL13RA1	2.4228113
IL15RA	3.5840108
IL17A	1.1216958
IL17RA	1.9078076
IL17RB	4.551114
IL17RC	1.6217701

IL18	2.1820152
IL18BP	3.7098987
IL19	2.6219904
IL1B	2.6184552
IL1F6	8.307196
IL1F8	2.3314228
IL1F9	2.133226
IL1R1	5.1426754
IL1R2	4.816951
IL1RAP	3.1045382
IL1RN	4.6321325
IL20RB	1.8642223
IL22RA1	1.7001741
IL23A	4.1164274
IL23R	1.804436
IL27	4.0282507
IL27RA	0.4175725
IL2RA	0.94353247
IL2RB	0.5175369
IL2RG	1.0998876
IL33	0.65226483
IL4I1	0.7843354
IL4RA	1.7801163
IL6	7.301508
IL6RA	0.7107675
IL6ST	1.4659069
ILF2	0.8355763
ILF3	1.6622007
ILK	0.40771985
ILKAP	1.2720954
ILVBL	2.1907885
IMAGE:61964	0.2935617
IMMP1L	1.5814736
IMMP2L	3.8894742
IMMT	2.3958318
IMP3	1.9912603
IMP4	2.6701677
IMPA1	1.663194
IMPACT	1.9543841
IMPAD1	1.6666262
IMPDH2	1.9424932
IMPG1	1.6427591
IMPG2	1.9253795
INCA1	1.9328482
INF2	1.0435851

ING1	0.6466453
ING3	0.43403792
ING5	1.5592515
INHBA	3.570976
INHBB	4.2191086
INHBC	6.2006655
INHBE	3.0423086
INIP	0.37650704
INO80B	0.49511313
INO80C	3.0346844
INO80D	1.0878618
INPP1	2.3507621
INPP4A	1.403358
INPP5B	0.6660793
INPPL1	1.1010745
INSC	7.888791
INSIG1	2.116977
INSIG2	5.1397324
INSL6	0.9656713
INSR	2.2053416
INTS10	0.3575437
INTS12	1.861131
INTS3	0.6818495
INTS4	0.7601304
INTS5	1.911478
INTS7	1.6634626
INTS8	1.3710196
INTS9	0.57636094
INTU	1.1709223
INVS	0.7666824
IP6K1	1.698838
IP6K2	0.67082334
IPMK	1.5740459
IPO11	1.8586919
IPO13	0.3398292
IPO4	1.6096804
IPO5	1.851867
IPO7	1.6274679
IPO8	1.5237839
IPO9	1.0584402
IPPK	1.4293883
IQCC	0.586884
IQCE	0.5561309
IQCG	0.48977685
IQGAP2	2.8810685

IRAK1	0.78391576
IRAK1BP1	1.0085952
IRAK2	1.6917689
IRAK3	5.0336866
IRAK4	0.5209422
IREB2	1.4977889
IRF1	2.2785275
IRF2	1.4437268
IRF2BP1	1.0737627
IRF2BP2	1.8753235
IRF3	1.9987185
IRF6	2.0035846
IRF7	2.2491696
IRF8	2.2422798
IRF9	1.1820786
IRG1	4.3506966
IRGM1	3.201121
IRGM2	2.2130287
IRGQ	1.5663483
IRS1	0.5869281
IRS2	3.9137294
IRX6	1.2463641
ISCA1	1.7757041
ISCA2	1.811065
ISCU	1.9743288
ISG15	4.145357
ISG20	1.7159245
ISG20L2	1.5442796
ISOC1	1.5706813
ISOC2A	1.3145628
IST1	1.943084
ISY1	2.0710814
ISYNA1	0.9810622
ITCH	1.7392132
ITFG1	1.9768202
ITFG2	2.142515
ITFG3	2.1051905
ITGA10	0.2595551
ITGA2	0.8881717
ITGA5	1.6015675
ITGA7	1.9898446
ITGA9	1.867862
ITGAL	1.4654362
ITGAM	1.315675
ITGAV	0.28603363

ITGB1	0.4585688
ITGB1BP1	1.6138008
ITGB2	1.1052191
ITGB3	1.5746953
ITIH1	8.176365
ITIH2	6.850836
ITIH3	11.47596
ITIH4	7.339513
ITIH5	0.9001517
ITM2B	0.43737864
ITPA	2.1854627
ITPK1	0.90950847
ITPKC	3.2122247
ITPR1	1.6767213
ITPR2	0.9494889
ITPRIP	1.0336277
ITSN1	3.030481
ITSN2	1.3785989
IVD	1.6698925
IVNS1ABP	0.58214116
IWS1	0.91265273
IYD	4.527674
JADE1	1.1204641
JADE2	1.054743
JAGN1	2.271405
JAK1	2.015786
JAK2	0.57288766
JAK3	1.8317883
JAM2	1.7744348
JARID2	0.5819986
JDP2	1.2167475
JKAMP	1.4535534
JMJD1C	3.1148245
JMJD6	1.4857075
JMY	1.5784564
JOSD1	2.6004188
JOSD2	1.1320207
JPX	2.0082154
JTB	1.7522418
JUN	1.7830541
JUNB	2.7314746
JUND	1.7742865
K230015D01	0.9610691
KANK1	1.8205769
KANSL2	1.164161

KANSL3	2.2151911
KARS	2.3130958
KAT2A	0.7362201
KAT2B	1.429105
KAT5	0.6847217
KAT7	1.4790914
KAT8	2.4143045
KATNA1	2.1063879
KATNAL1	1.2686975
KATNB1	0.66657186
KATNBL1	1.1400723
KBTBD2	1.2098863
KBTBD4	2.0399644
KBTBD7	1.3342259
KBTBD8	1.3210187
KCMF1	2.0784323
KCNA4	3.3905303
KCND1	0.596509
KCNE4	2.98636
KCNJ8	3.0198295
KCNK13	1.4908783
KCNK5	1.1312865
KCNN2	5.326069
KCNQ1OT1	0.7599695
KCNT2	2.407714
KCP	2.4133868
KCTD1	0.8107369
KCTD13	1.0912645
KCTD14	1.9401846
KCTD15	2.2389872
KCTD17	1.0195324
KCTD18	1.2893322
KCTD19	2.1124516
KCTD20	2.4903347
KCTD21	0.50579214
KCTD3	2.4846547
KCTD5	1.1123774
KCTD6	1.7162864
KCTD9	1.9240978
KDELR1	0.9930937
KDELR2	3.5646517
KDM1A	1.1144898
KDM1B	1.0116193
KDM2B	0.6908343
KDM3A	2.287729

KDM4A	1.8362148
KDM4B	1.678788
KDM4C	0.5574539
KDM5A	1.6545556
KDM5B	1.5447595
KDM5C	1.5527918
KDM6A	1.6253107
KDM6B	1.4226644
KDM8	0.88331485
KDSR	1.0653994
KEAP1	2.3408911
KHDRBS1	1.522362
KHDRBS3	1.949064
KHK	4.5314865
KHNYN	2.1244438
KHSRP	1.6300476
KIF12	1.3425932
KIF19A	1.2032309
KIF1B	1.0938466
KIF1C	0.48630548
KIF21A	3.3074949
KIFAP3	0.29269624
KIN	0.35402846
KIRREL3OS	1.5831208
KIZ	1.4334309
KLB	2.2710643
KLC2	1.0351112
KLC4	1.3520782
KLF10	1.8943789
KLF11	0.76547813
KLF12	0.9501307
KLF13	0.8489258
KLF15	2.946729
KLF2	0.52039504
KLF3	1.9597256
KLF9	1.5459793
KLHDC10	2.415439
KLHDC2	2.5738146
KLHDC3	2.2017996
KLHDC4	1.4354985
KLHL11	0.56920123
KLHL12	1.6311653
KLHL15	3.3030725
KLHL18	1.211756
KLHL2	2.9392955

KLHL21	2.286641
KLHL24	0.7445445
KLHL25	2.2903821
KLHL26	0.75745845
KLHL28	1.0402076
KLHL6	1.2414155
KLHL7	0.9764738
KLHL9	1.175844
KLK1B16	1.8981748
KLKB1	2.3063078
KLRA1	1.4279668
KLRA2	2.1533875
KMO	4.0177107
KMT2B	1.2391322
KMT2C	0.75304294
KNG1	9.90896
KNG2	11.8559065
KNOP1	1.6652849
KPNA1	1.8871243
KPNA2	1.8839362
KPNA3	2.0152462
KPNA4	0.9331777
KPNA6	0.9051893
KPNB1	0.84157443
KPTN	0.946152
KRAS	1.1206901
KRBA1	1.7207224
KRCC1	2.509197
KREMEN1	0.608722
KRI1	1.8783438
KRIT1	1.2608607
KRR1	1.3682611
KRT17	1.6932578
KRT18	4.685648
KRT222	0.3768804
KRT8	3.6674483
KRT85	0.56410813
KRTAP11-1	8.227596
KRTAP19-4	1.3308108
KRTAP20-2	2.605028
KRTAP3-3	1.8844075
KRTAP4-8	2.1015975
KRTAP5-1	0.30231118
KRTAP9-5	1.9412861
KRTCAP2	2.6310298

KTI12	2.3120477
KTN1	1.6195009
KXD1	1.5346429
KYNU	3.3953376
L1TD1	1.6453745
L2HGDH	1.9678679
L3HYPDH	2.229664
L3MBTL3	0.5545211
L7RN6	1.4082077
LACC1	3.58115
LACTB	1.8209774
LACTB2	2.5890915
LAGE3	0.47964263
LAMP1	1.1186388
LAMP2	2.1891296
LAMTOR1	1.6336424
LAMTOR2	2.4092743
LAMTOR3	1.4106681
LAMTOR4	1.2968476
LAMTOR5	1.4476321
LANCL1	1.3564775
LANCL2	1.6035478
LAP3	3.0823472
LAPTM4A	1.5560639
LARP1	2.503506
LARP1B	2.9347565
LARP4	2.5775588
LARP4B	1.47805
LARP7	1.2691977
LARS	2.5751383
LARS2	1.6252029
LAS1L	1.3283246
LASP1	0.98687005
LATS1	1.0239508
LBP	4.678337
LBR	0.40247798
LCAT	7.4585495
LCLAT1	1.9870479
LCMT2	2.0098703
LCN2	6.275546
LCOR	2.4109876
LCORL	1.3232586
LCP1	1.1925318
LCP2	0.8907249
LDB1	1.1435897

LDHA	3.4418366
LDHD	5.510539
LDLR	1.7505739
LDLRAD3	3.6118429
LDLRAP1	0.6864984
LEAP2	4.854226
LECT2	6.051893
LEFTY2	0.342453
LEMD2	1.5082009
LEMD3	0.36574268
LENEP	2.5087001
LENG9	3.0728128
LEPR	4.853097
LEPRE1	0.61886525
LEPROT	1.2841551
LEPROTL1	0.918828
LETM1	2.0144947
LETMD1	1.2526085
LGALS3BP	2.3279054
LGALS4	1.1641309
LGALS8	3.175483
LGALS9	3.2488186
LGALSL	0.38834834
LGMN	2.8928726
LGR4	2.0230298
LGR5	1.101258
LHPP	2.9284904
LHX1OS	2.1276941
LHX2	2.9569411
LHX6	0.57897735
LIAS	2.1128595
LIG3	1.3734739
LIG4	0.79763746
LILRA6	1.7144406
LILRB4	2.3999817
LIMD1	0.8646076
LIME1	0.40199637
LIMK1	0.27668405
LIMS2	0.66403794
LIN28A	1.0244985
LIN37	1.4864895
LIN52	0.6578951
LIN54	0.3893969
LIN7A	2.1100895
LIN7C	0.9563005

LINGO4	3.099388
LINS	1.7914546
LIPA	1.0925119
LIPC	5.877385
LIPO1	0.51009274
LIPT1	2.0666661
LIPT2	0.5241623
LITAF	3.5588424
LIX1L	0.84302473
LLGL2	1.2104523
LLPH	1.4029033
LMAN1	3.085171
LMAN2	2.872452
LMAN2L	1.0578663
LMBR1L	1.4574182
LMBRD1	1.2098172
LMBRD2	1.6831717
LMF1	2.2761905
LMF2	0.7916553
LMNA	0.9725454
LMO4	0.6267173
LMTK2	1.0933421
LNX1	0.91303205
LNX2	1.314431
LOC1000488	3.4520442
LOC1008617	2.5320718
LOC1008617	1.7744052
LOC1008624	0.90556216
LOC1010557	2.1119378
LOC1010558	1.3463466
LOC1010558	1.3311017
LOC1010558	1.2196877
LOC1010559	1.5626581
LOC1010560	0.5629766
LOC1010560	2.1526158
LOC1010562	1.7899377
LOC1026319	1.7567794
LOC1026322	1.8131888
LOC1026324	1.6853716
LOC1026324	3.5606768
LOC1026327	1.5561521
LOC1026330	1.6132667
LOC1026332	2.1090972
LOC1026335	1.7474391
LOC1026336	0.7288263

LOC1026338	1.3144305
LOC1026340	1.9238365
LOC1026341	3.7904947
LOC1026344	0.9156897
LOC1026347	1.2985256
LOC1026350	1.1368892
LOC1026357	1.9953594
LOC1026362	1.3816106
LOC1026366	2.2605789
LOC1026371	2.3318336
LOC1026372	2.6859467
LOC1026376	1.2528536
LOC1026377	2.299216
LOC1026381	3.6260116
LOC1026385	1.9096668
LOC1026385	3.3633168
LOC1026386	1.0017703
LOC1026387	0.80434775
LOC1026388	1.3009479
LOC1026388	3.009637
LOC1026399	0.5971625
LOC1026399	1.453357
LOC1026399	0.91425014
LOC1026402	1.0188
LOC1026410	2.111447
LOC1026410	0.97378135
LOC1026410	0.9157078
LOC1026412	1.3726997
LOC1026413	4.0714025
LOC1026413	1.5869181
LOC1026413	2.105355
LOC1026413	1.1084869
LOC1026413	2.9152632
LOC1026414	1.256882
LOC1026415	1.062248
LOC1026415	0.76570106
LOC1026416	2.547242
LOC1026416	1.9564354
LOC1026416	2.1967561
LOC1026416	1.6905997
LOC1026416	0.40118194
LOC1026419	1.3681624
LOC1026420	0.34029078
LOC1026422	1.3878939
LOC1026423	1.4179418

LOC1026424	0.359174
LOC1026424	1.1358135
LOC1026424	1.1489484
LOC1026425	0.8146641
LOC1026426	0.64696383
LOC1026426	1.3428967
LOC1026427	0.32048607
LOC1026427	0.87872815
LOC1026428	0.82425904
LOC1026428	1.169205
LOC1026430	6.311714
LOC1026430	1.2238333
LOC1026431	2.4729917
LOC1026432	1.0008318
LOC547349	1.9256203
LOC630751	5.4762974
LOC674896	1.5871608
LONP1	3.8339546
LONP2	2.3308742
LONRF1	1.3267405
LONRF3	0.7283976
LOXL2	1.592109
LPAR6	1.5209968
LPCAT2	0.79821944
LPCAT3	1.5673864
LPGAT1	3.103863
LPIN1	4.998122
LPIN2	1.908087
LPIN3	2.2316191
LPP	1.7484171
LPXN	1.8018973
LRBA	1.3039963
LRCH1	0.58695626
LRFN3	2.7006047
LRG1	6.993614
LRIF1	2.0638747
LRIG1	1.0667975
LRIG3	1.7509296
LRIT1	5.428351
LRMP	1.788274
LRP1	2.2703664
LRP10	2.2462933
LRP12	1.1954296
LRP2BP	1.1057904
LRP3	1.1951549

LRP5	1.6032231
LRP6	0.91416335
LRP8OS1	4.4360394
LRPPRC	3.3984244
LRRC14	1.4800577
LRRC14B	2.4447718
LRRC19	2.2253854
LRRC20	1.4899871
LRRC24	1.5709636
LRRC25	1.7581518
LRRC28	2.18698
LRRC3	1.0233009
LRRC31	1.7594492
LRRC38	1.9080071
LRRC3B	1.0236535
LRRC40	2.2620218
LRRC41	1.8993509
LRRC42	2.7887375
LRRC47	2.1013234
LRRC48	0.36469293
LRRC58	1.7354376
LRRC59	3.2015269
LRRC61	0.49256134
LRRC8A	2.8961647
LRRC8C	0.8871937
LRRC8D	2.4716518
LRWD1	1.1833951
LSG1	2.5171802
LSM1	2.175998
LSM10	1.5612013
LSM11	1.9356337
LSM12	1.639816
LSM14A	1.8908169
LSM14B	0.49469447
LSM2	1.5456922
LSM3	0.43139672
LSM4	2.1817157
LSM5	1.4210126
LSM6	1.0174339
LSM7	0.9934056
LSM8	1.4008882
LSR	3.6303728
LSS	2.484629
LTA4H	0.82564473
LTB	1.2601211

LTB4R1	0.95800185
LTB4R2	1.9822407
LTBR	1.9651616
LTN1	1.6048226
LTV1	2.4393418
LUC7L	2.076142
LUC7L2	2.7546484
LUC7L3	1.1652882
LUM	1.0780609
LURAP1L	2.8367155
LY6D	2.1728423
LY6G	1.9008939
LY6K	1.7495964
LY96	2.6905177
LYN	1.6929715
LYPD5	2.7031195
LYPLA1	1.9838521
LYPLA2	1.5870831
LYPLAL1	2.5984333
LYRM4	1.7206714
LYRM5	2.3964965
LYSMD1	1.9496639
LYSMD3	2.5793784
LYSMD4	1.8868506
LYVE1	1.350559
LZIC	2.2886581
LZTFL1	1.7547829
LZTR1	0.4775684
LZTS3	0.7416718
M6PR	1.8504107
MACROD1	0.88740563
MAD1L1	1.8121617
MAD2L1BP	1.1043904
MAD2L2	2.2565691
MADD	3.2345588
MAEA	1.5196621
MAF	2.229853
MAF1	0.7712095
MAFB	2.8737895
MAFF	4.2769823
MAFG	2.5522277
MAFK	2.0555751
MAG	1.1604035
MAGEA7-PS	1.4855781
MAGED1	2.3940942

MAGOH	1.767698
MAGOHB	1.2770488
MAGT1	2.1683557
MAK16	1.246083
MALAT1	0.5579822
MALSU1	2.7014315
MALT1	2.357395
MAML1	1.3619888
MAN1A	3.7791455
MAN1A2	0.58408
MAN1B1	1.3971217
MAN2A1	3.1404874
MAN2B1	0.64237285
MAN2B2	0.67645574
MAN2C1	1.8176429
MANBA	0.888633
MANEA	1.1955335
MANF	3.9534104
MANSC1	0.93607306
MAOB	1.465215
MAP1LC3B	2.4971755
MAP1S	1.0585496
MAP2K1	2.6594136
MAP2K2	2.5691159
MAP2K3	1.6721599
MAP2K4	1.4800174
MAP2K5	1.1151001
MAP3K1	2.0036986
MAP3K11	0.9215472
MAP3K14	1.1595452
MAP3K15	0.78624964
MAP3K4	0.7257011
MAP3K5	1.5363591
MAP3K6	2.128425
MAP3K7	1.9393547
MAP3K8	3.5246217
MAP4K3	0.41883302
MAP4K4	1.141783
MAP4K5	1.1460087
MAP6D1	1.2337258
MAP7	2.4378655
MAP7D1	0.6619866
MAPK1	1.4395683
MAPK14	0.7399318
MAPK1IP1	1.627414

MAPK1IP1L	1.872978
MAPK6	2.2216833
MAPK7	1.5290539
MAPK8IP3	1.4082859
MAPK9	0.9235642
MAPKAPK2	2.1906354
MAPKAPK5	1.8922656
MAPRE1	1.0638878
MAPRE3	2.1039207
MARCKS	0.4565723
MARCKSL1	3.1055992
MARCO	4.727949
MARF1	0.3974061
MARK1	1.2100451
MARK2	0.63731074
MARK4	1.664799
MARS	3.0050337
MARS2	1.7545872
MARVELD2	2.1958053
MASP1	3.3028305
MASP2	5.5667315
MAST2	1.3518827
MAST3	1.3533733
MAT1A	7.5861526
MAT2A	1.7685444
MAT2B	0.6686208
MATR3	1.6356032
MAVS	2.9405873
MAX	0.88038087
MAZ	0.3728416
MB21D2	2.4231608
MBD1	5.1864614
MBD2	1.1510241
MBD3	0.89807916
MBD6	1.9348648
MBL1	4.58012
MBL2	11.083679
MBLAC1	0.75930905
MBOAT7	1.7108223
MBTD1	1.520416
MBTPS1	1.6614988
MC3R	1.6232481
MCAM	3.0676377
MCAT	1.9602978
MCCC1	2.0577881

MCCC1OS	3.5825367
MCCC2	0.8744345
MCFD2	3.1231081
MCL1	0.99170613
MCM10	1.4694757
MCM3	1.1697004
MCM4	0.89845586
MCM7	1.1930525
MCMBP	1.190707
MCOLN1	2.444782
MCPH1	0.8663759
MCRS1	0.5615294
MCTP2	3.8160655
MCTS1	1.4190619
MCTS2	2.1520522
MCUR1	1.9401915
MDC1	0.8435643
MDFIC	1.0413091
MDH1	2.704717
MDH2	2.6019628
MDM2	1.7120702
MDN1	2.0451424
ME1	1.808265
MEA1	1.9757903
MEAF6	0.75833964
MECP2	0.4951794
MECR	0.67220163
MED1	2.0480268
MED10	1.5482438
MED11	0.7622907
MED12	0.6865866
MED13	0.692852
MED15	1.4390867
MED16	2.2806368
MED17	1.8827868
MED20	1.649059
MED21	1.4965937
MED22	0.59243846
MED23	0.87352324
MED24	1.2557809
MED26	1.7815354
MED27	1.1787741
MED28	1.1374934
MED29	1.3016431
MED30	0.68114305

MED31	0.95577884
MED4	1.5443742
MED6	1.2891414
MED7	2.1196616
MED8	2.27234
MED9	2.383444
MEDAG	1.9674556
MEF2A	2.7557895
MEF2D	0.81613994
MEFV	3.0162437
MEGF8	1.0493438
MEGF9	1.0148096
MEIOB	3.713288
MEIS2	0.8760023
MELK	2.0744917
MEMO1	1.4442546
MEN1	0.48331952
MEPCE	1.1809685
MERTK	1.1812179
MESDC2	1.2233255
METAP1	2.2168534
METAP1D	2.7816298
METAP2	1.8354962
METRNL	1.0691874
METTL1	2.562937
METTL10	0.725548
METTL14	1.0589812
METTL15	1.4581432
METTL16	1.4319088
METTL18	1.8332474
METTL2	0.6887965
METTL20	3.1401403
METTL21A	0.9383795
METTL22	2.297618
METTL25	0.79896235
METTL3	1.5467417
METTL4	1.0883377
METTL5	1.5117781
METTL6	1.804322
METTL7B	2.8661969
METTL9	1.6737964
MEX3C	1.1402667
MEX3D	0.94052196
MFAP1A	0.9544871
MFAP1B	0.6499698

MFAP3	1.2916553
MFF	1.1455991
MFI2	1.4831514
MFN1	2.548845
MFN2	1.7009351
MFSD1	1.5348322
MFSD10	1.1189048
MFSD11	1.1425219
MFSD12	0.96241355
MFSD2A	5.5003433
MFSD2B	1.2156599
MFSD3	1.6042345
MFSD5	1.671066
MFSD6	0.3937676
MFSD7B	2.0474145
MFSD8	0.7179675
MGA	1.0181229
MGAM	6.031534
MGARP	5.1508493
MGAT1	1.3537529
MGAT2	1.9609139
MGAT4B	0.52032876
MGAT5B	1.7121217
MGEA5	1.6999128
MGMT	2.5952003
MGRN1	1.7070711
MGST1	3.552942
MIA2	3.9242904
MIA3	2.0620854
MIB1	2.1259477
MIB2	1.2545717
MICALL1	1.5492585
MICALL2	0.92736435
MICU1	1.2588112
MICU2	1.5970142
MID1	3.5726483
MIDN	1.3005059
MIEF1	1.7079632
MIEN1	2.119755
MIER1	0.6244252
MIER2	2.2718952
MIER3	2.0859993
MIF	3.8110144
MIF4GD	1.2520249
MIIP	0.7032254

MILR1	2.0581136
MINA	2.5724695
MINOS1	0.7250469
MINPP1	2.4645665
MIOS	1.2752075
MIOX	2.4874542
MIPEP	0.38312483
MIR17HG	2.3997443
MIR22HG	2.2187774
MIS12	2.1335084
MITD1	1.5139253
MITF	0.26484656
MKKS	0.6162913
MKL1	0.7098634
MKL2	0.8566916
MKLN1	1.6129558
MKNK2	2.245352
MKRN1	0.47454906
MKS1	1.2355001
MKX	2.9182072
MLEC	2.0422595
MLF2	1.354625
MLH1	1.2599056
MLH3	0.9619732
MLKL	3.4912841
MLLT1	1.0324347
MLLT10	0.75554824
MLLT4	1.482085
MLLT6	2.407088
MLX	2.3768418
MLXIP	0.41572523
MLXIPL	4.4121494
MLYCD	3.1757839
MMAA	0.83847785
MMAB	1.3561585
MMACHC	2.901481
MMADHC	3.226422
MMGT1	0.8653376
MMP14	0.99277425
MMP3	0.9164357
MMP7	3.1476314
MMP8	1.5244944
MMS22L	3.7419412
MNDA	1.0017493
MNT	1.3912771

MOAP1	1.7248046
MOB1A	1.0173008
MOB1B	0.8646469
MOB2	0.84444356
MOB3B	0.3961377
MOB3C	0.81169105
MOB4	1.6971953
MOCOS	4.1491766
MOCS1	3.1813667
MOCS2	2.2092254
MOCS3	2.6326168
MOGS	2.6427362
MON1A	2.5330064
MON1B	0.999501
MON2	1.7779431
MORC1	0.44987726
MORC2A	1.2789204
MORC3	2.0715444
MORC4	2.9921186
MORF4L1	1.744559
MORF4L2	2.227968
MOSPD1	1.9624603
MOSPD2	1.441252
MOSPD3	0.45277715
MOV10	2.663112
MPC1	2.3806298
MPC2	2.5778558
MPDU1	1.9343927
MPEG1	1.9238551
MPG	2.0281217
MPHOSPH10	1.2246053
MPHOSPH6	1.0671918
MPHOSPH8	1.7161644
MPI	1.8693984
MPND	2.513623
MPP6	4.3781157
MPPE1	0.7206886
MPST	0.99724174
MPV17	1.9579895
MPV17L2	1.17012
MPZL1	0.77965045
MPZL2	4.8303957
MPZL3	1.3553255
MRAP	4.737463
MRE11A	1.8756301

MREG	4.030116
MRFAP1	1.6196873
MRGBP	1.2658479
MRI1	1.4821436
MRM1	0.9242809
MROH1	1.5469091
MRPL1	1.2220597
MRPL10	2.352063
MRPL11	1.907532
MRPL12	1.8829224
MRPL13	2.041231
MRPL14	1.2379138
MRPL15	3.217553
MRPL16	3.2270257
MRPL17	3.7089374
MRPL18	1.9245732
MRPL19	1.6978111
MRPL2	1.3016732
MRPL20	2.1151812
MRPL21	2.02762
MRPL22	1.6611845
MRPL23	1.664717
MRPL24	2.5386045
MRPL27	2.2708595
MRPL3	1.7488482
MRPL30	2.1942942
MRPL32	1.8061178
MRPL33	0.9533808
MRPL34	1.4078362
MRPL36	2.176223
MRPL37	2.830273
MRPL38	3.9017866
MRPL39	1.9461405
MRPL4	1.6602209
MRPL40	1.9944799
MRPL41	2.6208088
MRPL42	1.4516065
MRPL43	2.2894957
MRPL44	2.0139654
MRPL45	2.7078846
MRPL46	2.5452945
MRPL47	2.4665167
MRPL48	1.2347791
MRPL49	0.6383512
MRPL50	2.6534374

MRPL51	2.6103518
MRPL52	2.7514727
MRPL53	1.9195712
MRPL54	2.164436
MRPL55	1.8177783
MRPL57	2.1134727
MRPL9	2.3662016
MRPS10	2.000602
MRPS12	2.4925568
MRPS14	1.1625106
MRPS15	2.4853103
MRPS16	1.9497535
MRPS17	1.615711
MRPS18A	1.069639
MRPS18B	4.29893
MRPS18C	1.9332922
MRPS2	2.5705411
MRPS21	0.82959914
MRPS22	2.37971
MRPS23	1.5565898
MRPS24	1.880944
MRPS25	0.910661
MRPS26	1.7127202
MRPS27	1.9805007
MRPS28	2.2133472
MRPS30	2.3622925
MRPS31	2.8808482
MRPS33	1.8079784
MRPS34	2.8167293
MRPS35	1.2022765
MRPS36	0.90627074
MRPS5	2.462498
MRPS6	1.5527995
MRPS7	2.308462
MRPS9	2.0307143
MRS2	0.6538353
MRTO4	2.416877
MS4A4A	3.1452057
MS4A4C	3.175009
MS4A6B	1.77407
MS4A6C	2.518153
MS4A6D	3.706238
MS4A8A	3.0303996
MSANTD2	0.4766965
MSANTD4	1.709379

MSH2	1.7993968
MSH6	1.5461004
MSL1	0.9473045
MSL2	0.9200747
MSL3	1.6553466
MSMO1	3.0086882
MSR1	4.268133
MSRA	3.3483903
MSRB1	1.644851
MSRB2	0.92779565
MST1	8.118801
MST1R	2.0335462
MT1	5.5462894
MT2	5.89396
MT3	0.5759964
MTA2	1.5376651
MTA3	1.71049
MTAP	0.537313
MTBP	2.1713722
MTCH2	2.7875865
MTDH	1.7844346
MTERF1A	0.81498265
MTERFD1	1.6945717
MTERFD2	1.9331992
MTF1	0.8417835
MTF2	1.214782
MTFMT	1.8004715
MTFP1	1.9980538
MTFR1	2.8420417
MTFR1L	1.2445195
MTFR2	3.5024927
MTG1	1.5701253
MTG2	2.6348293
MTHFD1	0.63649774
MTHFD1L	2.6537406
MTHFD2	2.370527
MTHFR	1.1433771
MTHFS	2.143769
MTIF2	2.7832897
MTIF3	0.66981554
MTM1	1.020536
MTMR1	1.3943169
MTMR10	1.0060453
MTMR11	3.2222695
MTMR12	1.2022712

MTMR14	1.8104036
MTMR2	0.91957736
MTMR3	0.641453
MTMR4	2.0253174
MTMR6	0.6464226
MTMR7	2.3451402
MTMR9	0.7420106
MTO1	1.9356277
MTOR	2.4041243
MTPAP	2.0877755
MTPN	1.5356948
MTRF1L	1.4607179
MTSS1	4.175313
MTTP	5.8911114
MTUS1	1.4562047
MTUS2	0.4896536
MTX1	2.2647069
MTX2	2.7416317
MUG1	10.176828
MUG2	10.782334
MUL1	3.5600603
MUP1	2.8136013
MUP17	3.6892157
MUP19	2.1937048
MUP2	1.5355687
MUP20	2.3852818
MUP21	3.7331402
MUP3	6.146245
MUP4	3.882414
MUP5	5.274135
MUT	2.414355
MVB12A	3.2702038
MVD	2.0152476
MVK	0.8314445
MVP	3.8907988
MX1	1.0553982
MX2	2.5412443
MXD1	2.4221485
MYBBP1A	1.5773985
MYC	3.5218165
MYCBP2	1.2528927
MYD88	2.5263698
MYEOV2	1.4521778
MYF5	1.7550788
MYF6	0.6591065

MYG1	1.3177173
MYH9	0.6220305
MYL12A	0.35061383
MYL6	0.49586844
MYO18A	0.88771605
MYO19	1.1708944
MYO5B	0.4856894
MYO7B	1.0683289
MYO9B	0.94408584
MYSM1	0.9405153
MZT1	1.2798784
MZT2	1.9498475
N4BP2	2.2701104
N4BP2L1	0.7761252
N4BP2L2	0.3683467
N6AMT1	1.0460927
N6AMT2	1.3647649
NAA10	0.9219053
NAA15	1.9223897
NAA20	1.8547714
NAA25	3.7238772
NAA30	1.8077509
NAA35	1.7994773
NAA38	1.2548249
NAA40	1.623637
NAA50	1.6966445
NAA60	0.74577785
NAB1	2.0907218
NAB2	1.8850324
NABP1	1.4711285
NABP2	1.192579
NACA	1.4870818
NACC2	0.92605805
NADK	2.4815462
NADK2	2.0316355
NADSYN1	3.7658904
NAE1	1.9124787
NAF1	1.412523
NAGA	0.62470174
NAGK	1.6386945
NAGLU	1.6982558
NAGS	2.6459672
NAIP2	1.5442469
NAIP5	2.5311453
NAIP6	1.1918237

NAMPT	4.114393
NANOG	1.2475257
NANP	2.7230477
NANS	2.1949708
NAP1L1	1.7782862
NAPA	2.5774734
NAPB	0.90107894
NAPG	1.2489784
NAPRT1	4.2884426
NARF	1.3620889
NARFL	1.5627992
NARG2	1.1062479
NARS	2.8997371
NARS2	2.099736
NASP	1.3924134
NAT10	1.727701
NAT2	2.1308267
NAT6	0.89323163
NAT8	1.4047465
NBAS	2.7573926
NBN	1.1222179
NBR1	2.532367
NCAPD3	0.6043315
NCAPH2	1.0734475
NCBP1	1.2357028
NCDN	1.2599804
NCEH1	1.2056558
NCF1	1.6230605
NCF2	0.5713656
NCF4	1.3759553
NCK1	2.7557623
NCKAP1	1.6536825
NCKAP1L	0.8945596
NCL	2.8855379
NCLN	2.1095593
NCOA1	0.61060023
NCOA2	2.3814375
NCOA4	2.358431
NCOA5	0.9901836
NCOA6	0.3926351
NCOR2	0.94507
NCSTN	0.9492018
ND1	2.4100106
ND2	2.247397
ND3	2.7424867

ND4	2.3370726
ND4L	2.6467965
ND5	2.8434026
ND6	1.0743315
NDC1	1.4571307
NDEL1	1.7342384
NDFIP1	1.7568152
NDNL2	0.41877198
NDOR1	2.070843
NDRG1	3.5418122
NDRG2	2.088001
NDRG3	0.76605344
NDST2	1.7387407
NDUFA1	1.9823511
NDUFA10	1.8538725
NDUFA11	1.3466775
NDUFA12	1.8211277
NDUFA13	1.6578572
NDUFA2	1.7879918
NDUFA3	1.7733529
NDUFA4	2.52581
NDUFA5	1.331929
NDUFA6	1.5860364
NDUFA7	1.6795018
NDUFA8	1.9288843
NDUFA9	2.9783738
NDUFAB1	2.5321782
NDUFAF1	0.6277511
NDUFAF2	1.2516081
NDUFAF3	0.88866067
NDUFAF4	3.1454656
NDUFAF6	1.8156645
NDUFAF7	1.5876257
NDUFB10	1.0330646
NDUFB11	1.132385
NDUFB2	2.1299188
NDUFB3	1.0698645
NDUFB4	1.6380689
NDUFB5	1.3370774
NDUFB6	2.3987534
NDUFB7	2.2309153
NDUFB8	1.9152234
NDUFB9	2.2293875
NDUFC1	1.9321306
NDUFC2	2.1703317

NDUFS1	2.6069515
NDUFS2	2.0731452
NDUFS3	2.3379176
NDUFS4	2.459336
NDUFS5	1.4651659
NDUFS6	1.7317297
NDUFS7	1.7137411
NDUFS8	2.0680263
NDUFV1	2.568134
NDUFV2	1.9616554
NDUFV3	1.3187697
NEAT1	1.6892068
NECAP1	2.2075284
NECAP2	1.6490352
NEDD1	1.9143565
NEDD4	1.2105377
NEDD4L	3.3187215
NEDD8	0.95954204
NEK1	0.6012986
NEK3	2.000746
NEK4	2.1945155
NEK6	3.0097349
NEK7	1.6604807
NEK8	1.6035469
NEK9	1.2611759
NELFA	1.0996301
NELFB	0.97934127
NELFCD	2.5270393
NELFE	1.117338
NEMF	1.7616627
NET1	1.5763962
NEU1	3.1839721
NEU3	1.3096321
NEURL1B	3.5571978
NEURL2	0.97157264
NEURL3	3.7659624
NEURL4	1.4306481
NF1	1.657984
NFE2L1	1.1377633
NFE2L2	1.6774704
NFIC	0.42509675
NFIL3	3.9184272
NFKB2	2.396919
NFKBIA	2.5546033
NFKBIB	3.7119367

NFKBID	1.1797006
NFKBIE	1.468838
NFKBIL1	1.5991986
NFKBIZ	1.3696458
NFRKB	1.5095384
NFS1	2.4868033
NFU1	1.7718222
NFX1	0.50518394
NFXL1	2.9591162
NFYA	0.8502991
NFYB	1.7286141
NFYC	1.5588696
NGDN	2.5548065
NGEF	1.0089967
NGF	1.0832789
NGFR	0.7785771
NGLY1	1.4758935
NGRN	1.6959603
NHEJ1	1.0916159
NHLRC3	0.9442222
NHP2	2.0687444
NHP2L1	2.0177972
NHSL1	0.6324892
NICN1	0.45882154
NID1	1.2806017
NIF3L1	2.6983826
NIFK	1.9782307
NINJ1	4.071474
NIPA2	0.9919975
NIPAL1	5.876749
NIPBL	1.0580633
NIPSNAP1	4.0660324
NIPSNAP3B	3.4880736
NISCH	0.7893913
NIT1	2.6436684
NKAP	0.5981612
NKD2	1.6874163
NKIRAS1	2.09177
NKIRAS2	0.9110153
NKRF	1.5560663
NKTR	2.0105307
NLGN3	0.6426332
NLK	0.2783029
NLN	2.3884385
NLRC3	0.32440257

NLRC4	1.1069448
NLRC5	1.9927442
NLRP3	0.7039306
NLRP6	0.8137567
NMD3	1.643686
NME1	2.5037725
NME2	2.8622382
NME4	1.3560629
NME6	3.8761384
NME7	1.0581329
NMI	2.396201
NMNAT1	3.192724
NMNAT3	2.7727244
NMRAL1	1.2913749
NMRK1	3.2078001
NMT1	2.0285332
NNMT	6.6302576
NNT	1.9594319
NOA1	1.0313795
NOB1	2.0370827
NOC2L	2.3666055
NOC3L	0.78451324
NOC4L	2.236873
NOD1	0.7752936
NODAL	1.1997769
NOG	0.47256804
NOL11	1.2334192
NOL12	3.5865471
NOL6	1.8419812
NOL7	1.2585146
NOL8	1.1035225
NOLC1	2.0995471
NOM1	2.2270982
NOMO1	2.4255764
NONO	0.95544314
NOP10	1.3366411
NOP14	2.7065265
NOP16	2.7918537
NOP2	2.967532
NOP56	1.3256218
NOP58	2.9934595
NOS1AP	4.086995
NOS2	7.3571596
NOS3	1.4264929
NOSIP	1.0929649

NOTCH2	0.644655
NOXA1	1.2956593
NPC1	2.5741136
NPDC1	0.4340217
NPEPL1	1.2627757
NPEPPS	1.5837505
NPHP1	0.44956708
NPHP3	1.015384
NPL	2.3250206
NPLOC4	2.403096
NPM1	1.9237235
NPPC	2.0419714
NPRL3	0.98902583
NPTN	0.49034238
NPY	3.8314302
NQO1	0.8595731
NQO2	0.69093156
NR1D2	2.3807719
NR1H2	1.3220842
NR1H3	2.6312997
NR1H4	7.7155714
NR1I2	3.8525913
NR1I3	1.0525653
NR2C1	1.5986793
NR2C2	1.2624929
NR2C2AP	1.1583283
NR2F6	1.8694775
NR3C1	0.3893416
NR4A1	1.8552005
NR5A2	2.1430428
NR6A1	1.894943
NRAS	0.93217826
NRBF2	2.4743302
NRBP1	1.2760293
NRBP2	1.8560836
NRDE2	0.97400284
NRG1	0.9984019
NRG4	7.884054
NRIP1	1.4271252
NRN1	0.26886868
NRN1L	1.2064586
NRP2	0.7418301
NRSN2	0.93366456
NSA2	1.1256802
NSD1	0.4496143

NSDHL	2.9696014
NSF	0.8974588
NSFL1C	2.270346
NSMAF	0.54890895
NSMCE1	1.360379
NSMCE2	1.5331771
NSMCE4A	1.146384
NSMF	2.4576662
NSUN2	2.4106548
NSUN3	1.5315764
NSUN4	1.0086808
NT5C	0.73107886
NT5C2	1.7337735
NT5C3	2.25292
NT5C3B	0.6748779
NT5DC1	1.7334158
NT5DC3	2.1403716
NT5E	0.70137715
NT5M	1.8133523
NTF3	2.8862028
NTMT1	1.9548509
NTN3	2.4902637
NTPCR	0.8266418
NUAK2	1.660496
NUB1	0.9991219
NUBP1	2.7587135
NUBP2	2.0518243
NUBPL	2.7561839
NUCB1	1.3234174
NUCB2	1.3286221
NUCKS1	1.9809616
NUDC	1.3892596
NUDCD1	0.78833604
NUDCD2	2.0514696
NUDCD3	0.7120373
NUDT1	1.4894459
NUDT12	4.4943433
NUDT19	3.4787567
NUDT2	1.0350599
NUDT21	1.794019
NUDT22	1.9632909
NUDT3	0.3533013
NUDT4	3.043689
NUDT5	1.8766534
NUDT6	1.9697998

NUDT7	4.5734396
NUDT8	1.6851323
NUDT9	1.680557
NUFIP1	1.9528282
NUFIP2	1.9016101
NUMA1	0.34986663
NUP107	0.7451179
NUP133	0.9440129
NUP153	1.0769794
NUP155	0.41758323
NUP160	1.6552308
NUP205	2.0981362
NUP35	1.1934788
NUP37	1.5986784
NUP43	1.7107866
NUP50	3.267536
NUP54	2.6140425
NUP62	1.6285069
NUP85	1.4315794
NUP88	1.638315
NUP93	1.0232799
NUP98	1.4959795
NUPL2	0.5498562
NUS1	1.4057863
NXF1	1.5751107
NXPH4	1.0394406
NXT1	1.9426625
NXT2	1.6858313
OAF	6.4787645
OARD1	1.1864855
OAS1A	2.9424198
OAS1B	2.574344
OAS1F	2.431905
OAS2	1.4593346
OAS3	1.2869372
OASL1	5.1181097
OASL2	1.9017646
OAT	2.184231
OAZ1	1.2048304
OCEL1	1.607415
OCIAD1	2.345194
OCRL	0.86354804
OCSTAMP	1.7997358
ODC1	2.8571298
ODF2	1.7014239

ODF2L	2.0560238
OFD1	2.276068
OGDH	1.3048036
OGFOD1	3.3558862
OGFOD2	1.2199395
OGFOD3	1.151438
OGFR	2.6511314
OGT	1.0870397
OIT3	4.7445717
OLA1	1.4471653
OLFM4	1.8729725
OLFR1116-PS	2.0418105
OLFR1229	1.3125916
OLFR1247	0.79362917
OLFR1260	3.2067733
OLFR139	2.1850207
OLFR141	1.2028902
OLFR1412	0.33548594
OLFR143	1.8713732
OLFR1443	0.36857915
OLFR1466	1.2088468
OLFR149	0.85935974
OLFR153	0.8666682
OLFR224	0.69068
OLFR424	4.5803537
OLFR549	0.81224775
OLFR609	1.7546604
OLFR740	2.4079554
OLFR796	0.30761528
OLFR97	1.6361754
OLIG1	7.0806026
OLR1	1.274867
OMA1	0.7417877
ONECUT1	2.4519265
ONECUT2	7.3923616
OPA1	2.1510274
OPA3	1.7622216
OPLAH	3.6556551
OPTN	2.726762
ORAI3	0.5556462
ORAOV1	0.44844007
ORC2	0.8129995
ORC3	2.0647519
ORC5	1.6551931
ORC6	0.59492135

ORM1	10.751582
ORM2	11.931323
ORM3	4.24842
ORMDL1	1.8949201
ORMDL2	1.9737618
ORMDL3	2.5442345
OS9	2.5069711
OSBP	1.4497049
OSBPL11	1.2528603
OSBPL1A	3.4719627
OSBPL2	0.89231133
OSBPL9	1.2885058
OSCAR	4.381407
OSER1	2.12526
OSGEP	0.5063813
OSGEPL1	2.8395536
OSGIN1	2.6891563
OSMR	2.715881
OST4	1.4722478
OSTC	2.44261
OSTF1	1.0946443
OSTM1	0.8276737
OTC	6.737586
OTOF	0.9525411
OTUD3	3.0332687
OTUD4	2.0465953
OTUD5	2.1230223
OTUD6B	1.9543502
OTULIN	1.8027413
OVCA2	0.70794106
OVOL2	1.4573715
OXA1L	1.4159896
OXLD1	0.7555945
OXNAD1	3.3301857
OXR1	0.73272157
OXSM	2.7908962
OXSR1	1.5909674
P2RX4	2.3019502
P2RY13	2.5997422
P2RY14	0.5526197
P2RY2	4.5931835
P2RY6	1.1358902
P4HA2	1.2609274
P4HB	3.9626305
PA2G4	1.9683406

PABPC1	1.881058
PABPC4	2.2677324
PABPN1	1.6825912
PACRGL	0.5275848
PACSIN1	0.48438478
PACSIN3	3.0503552
PADI1	0.76180744
PAF1	1.6004822
PAFAH1B1	1.5475886
PAFAH1B2	1.9097605
PAFAH2	2.7388933
PAGR1A	1.0536592
PAH	9.021723
PAICS	2.226073
PAIP1	1.2516048
PAIP2	0.9439838
PAIP2B	1.535703
PAK1IP1	2.0071213
PAK2	1.1297987
PAK4	3.392911
PALLD	0.6542342
PAM16	2.2687948
PAN2	1.8484185
PAN3	0.793854
PANK1	3.0313678
PANK2	1.0902698
PANK3	1.260118
PANK4	1.5449622
PAOX	1.2521203
PAPD4	2.3586628
PAPD5	1.281255
PAPD7	1.2110465
PAPOLA	1.665303
PAPSS2	1.6438696
PAQR9	4.1458015
PARD3	0.54205155
PARD6A	1.0185604
PARG	1.324743
PARK7	1.9238856
PARL	1.7639616
PARN	1.1583445
PARP1	0.98652077
PARP10	2.9386985
PARP11	1.3443158
PARP12	2.684183

PARP14	2.5132825
PARP16	4.1945515
PARP2	1.5005543
PARP3	1.208061
PARP4	1.4097903
PARP6	0.858402
PARP8	0.74992204
PARP9	3.1885397
PARS2	0.56213355
PARVA	0.56074834
PATL1	1.9380586
PAWR	2.9800942
PAXBP1	1.3550808
PBDC1	2.910569
PBLD1	1.8551221
PBLD2	3.6190116
PBRM1	1.0543008
PBX2	0.5853479
PCBD1	3.9730318
PCBD2	1.6828024
PCBP1	2.0585368
PCBP2	1.5739887
PCBP4	1.6563475
PCCA	1.6482027
PCCB	1.6906028
PCDH1	0.77837205
PCED1A	0.99643874
PCF11	1.084563
PCGF1	0.9875629
PCGF3	1.1285403
PCGF5	2.0357153
PCGF6	3.5516798
PCID2	2.392295
PCK1	6.8706536
PCM1	0.7789099
PCMT1	1.7464416
PCMTD1	1.8643687
PCMTD2	0.85995746
PCNA	0.6308806
PCNXL3	1.605169
PCSK5	1.545028
PCSK6	3.870578
PCSK9	4.7213154
PCX	5.1158247
PCYOX1	2.6419551

PCYT1A	1.2622302
PCYT2	3.9139907
PDAP1	2.2973335
PDCD10	2.446656
PDCD2	2.2835925
PDCD2L	1.6054184
PDCD4	0.80448174
PDCD5	2.149676
PDCD6	2.3001935
PDCD6IP	2.5283835
PDCD7	1.261728
PDCL	1.0833457
PDCL3	1.1989143
PDE1B	0.46753716
PDE2A	1.4008558
PDE3B	2.2194622
PDE4B	3.0515134
PDE4C	1.4969532
PDE4DIP	2.2734754
PDE6D	0.794986
PDE6H	2.3316782
PDE8A	0.28973413
PDF	1.3167422
PDGFC	2.6192052
PDHA1	0.7887051
PDHB	1.6534908
PDHX	2.1881711
PDIA3	3.0561764
PDIA4	4.162307
PDIA5	4.470992
PDIA6	2.8604867
PDILT	3.8192177
PDK1	2.7813208
PDK4	4.4373665
PDLIM1	1.222614
PDLIM7	1.7175648
PDP2	1.3150039
PDPK1	1.5819504
PDPR	1.6510742
PDRG1	0.56181645
PDS5A	1.2697866
PDS5B	0.5850527
PDSS1	1.8779657
PDSS2	1.3467164
PDXDC1	0.4334061

PDXK	1.3222396
PDZD11	1.4299419
PDZD8	1.7058449
PDZK1	5.9404764
PDZRN3	1.1095712
PEBP1	1.9855301
PECR	3.5442526
PEF1	1.26563
PEG3	1.2497962
PELI1	0.9049127
PELI2	0.5883379
PELO	2.3969243
PELP1	1.6862185
PEMT	4.4690094
PEPD	3.1711996
PER1	2.3636954
PERP	2.0515645
PES1	1.8298967
PET112	2.5540292
PEX1	0.59674144
PEX10	0.75190926
PEX11A	3.833194
PEX11B	2.21198
PEX11G	3.5415142
PEX13	3.1836483
PEX14	2.7298293
PEX16	3.8588278
PEX19	2.020871
PEX2	2.3819153
PEX26	1.7433712
PEX3	2.267133
PEX5	2.9486454
PEX6	1.86714
PEX7	2.6479776
PFDN1	1.3438184
PFDN2	1.6739929
PFDN4	1.5645466
PFKFB1	4.2010555
PFKFB2	2.0583837
PFKFB3	4.7890472
PFKL	3.4031985
PFN1	1.3386228
PGAM1	3.5510633
PGAM5	1.8295233
PGAP2	0.5050638

PGAP3	1.437494
PGD	1.1767905
PGGT1B	1.5222075
PGK1	3.9687111
PGLS	1.3771355
PGLYRP2	5.7325354
PGM1	1.5591757
PGM2	3.8857358
PGM3	2.639323
PGP	0.957175
PGPEP1	2.442983
PGRMC1	2.50926
PGRMC2	0.2594533
PGS1	1.1092188
PHACTR4	1.7755125
PHB	1.7775872
PHB2	2.3174999
PHC1	0.3657415
PHC2	0.3886311
PHF1	0.5206344
PHF10	2.213928
PHF11A	2.7548187
PHF11B	0.6886318
PHF11C	1.4197023
PHF11D	1.8085468
PHF12	1.2258835
PHF13	1.8186486
PHF14	0.6679456
PHF2	1.1560087
PHF20	0.96231914
PHF21A	0.7888038
PHF23	1.9641025
PHF3	1.5606716
PHF5A	1.9914396
PHF7	0.9765651
PHF8	1.0553405
PHIP	1.3937991
PHKA2	0.47290778
PHKG1	0.2606573
PHKG2	1.7907774
PHLDA1	1.6987927
PHLPP1	0.5406923
PHLPP2	0.35005713
PHOSPHO2	1.2742288
PHPT1	1.6757095

PHRF1	1.0643675
PHTF2	0.8163283
PHYH	5.537959
PHYHD1	1.8388045
PHYKPL	1.5636036
PI15	1.3536894
PI4K2A	2.968105
PI4K2B	0.32503676
PI4KA	2.0997274
PI4KB	0.93283916
PIAS1	0.5166614
PIAS2	2.438718
PIAS4	1.3636434
PICALM	1.9534128
PICK1	1.2089112
PID1	3.501335
PIDD1	0.41691542
PIF1	0.7540798
PIGA	2.4261382
PIGF	1.5358927
PIGG	1.4131212
PIGK	1.8078406
PIGL	1.7892103
PIGN	0.9589665
PIGO	1.4052804
PIGP	1.4431765
PIGQ	0.809778
PIGR	2.009463
PIGS	1.403508
PIGT	1.2118375
PIGV	0.51109695
PIGX	1.8327453
PIGYL	1.3684142
PIH1D1	1.727143
PIK3AP1	1.1979702
PIK3C2A	2.1221247
PIK3C2G	1.2288325
PIK3CA	0.46694255
PIK3CB	1.1492751
PIK3R1	0.5093167
PIK3R2	1.1095321
PIK3R4	1.430979
PIK3R5	1.5761268
PILRA	1.4079792
PILRB2	1.1173773

PIM1	2.586098
PIM2	0.73392415
PIM3	3.1566513
PIN1	1.713218
PIN1RT1	1.5411041
PIN4	1.5566013
PINX1	1.7091911
PIP4K2C	1.3395283
PIP5K1A	7.522791
PIPOX	6.094598
PIR	1.1079638
PIRA11	1.2350781
PIRA2	2.5451639
PIRA6	1.7246511
PIRA7	1.738179
PIRB	2.0215352
PISD	1.2734697
PISD-PS1	1.1830971
PITHD1	0.9270823
PITPNA	1.2087667
PITPNB	1.4665473
PITPNC1	0.7245729
PITPNM1	0.5940659
PITPNM2OS1	1.2686098
PITRM1	2.6940844
PJA1	1.7370765
PJA2	0.92537
PKDCC	3.7382295
PKLR	2.4850745
PKM	2.0325072
PKN1	1.1251843
PKN2	1.9781201
PKNOX1	2.4055226
PKP1	1.8382609
PKP2	2.3348958
PKP3	1.1880424
PLA1A	6.3308983
PLA2G12A	3.1482728
PLA2G12B	5.9600487
PLA2G4A	0.8750818
PLA2G4B	1.7901156
PLA2G6	2.0378654
PLA2G7	2.3196738
PLAA	2.5786054
PLAC8	1.2046134

PLAGL2	0.6225481
PLAUR	1.3766739
PLBD1	2.8011
PLBD2	2.049772
PLCD4	1.5099151
PLCE1	0.5499563
PLCL1	0.6296067
PLCL2	1.2538645
PLCXD1	0.79528093
PLCXD2	6.076438
PLD1	1.726877
PLEC	1.8580372
PLEK	1.1361353
PLEKHA3	2.336929
PLEKHA4	1.5366123
PLEKHA5	1.3252361
PLEKHA6	0.8998711
PLEKHA8	2.0718396
PLEKHF2	0.74678206
PLEKHG2	0.25638938
PLEKHG3	3.5436785
PLEKHG4	4.9198055
PLEKHH1	0.6834781
PLEKHJ1	1.8436933
PLEKHM1	1.8915598
PLEKHM2	1.5130951
PLEKHS1	2.7932723
PLG	7.6522865
PLIN2	4.6030707
PLIN3	2.3672173
PLIN4	3.7955854
PLIN5	6.522341
PLK3	6.301262
PLOD3	1.7491148
PLRG1	2.288779
PLSCR1	3.9455392
PLXNA1	0.93429303
PLXNA2	1.0558026
PLXNB1	1.8725169
PLXNB2	1.604142
PM20D1	1.5183196
PMAIP1	0.6214988
PMF1	1.1993606
PML	2.0327504
PMM2	2.077223

PMPCA	2.4401782
PMPCB	3.02401
PMS1	1.4431236
PMVK	1.5479476
PNISR	0.69398236
PNKD	0.46399283
PNKP	1.6162708
PNMA2	1.1595333
PNN	0.8245323
PNO1	3.056155
PNP	3.9839323
PNP2	0.5746398
PNPLA2	2.432185
PNPLA6	1.0874331
PNPLA7	3.4673536
PNPO	3.5234458
PNPT1	2.5622542
PNRC1	3.8150284
PNRC2	1.2302387
POC1B	0.42278337
POC5	1.513005
POFUT1	0.7295835
POFUT2	0.333807
POGK	0.9634192
POGLUT1	1.6096876
POGZ	1.7265916
POLA2	0.9918723
POLB	1.9440134
POLD2	0.6042123
POLD3	0.9430487
POLD4	1.0869629
POLDIP2	1.7352026
POLDIP3	1.2577636
POLE3	0.8583977
POLE4	3.401868
POLG	1.7962263
POLH	0.3481803
POLL	0.91688514
POLM	0.88034844
POLR1A	2.5246136
POLR1B	1.1209333
POLR1C	2.149605
POLR1D	1.1070569
POLR1E	2.2104166
POLR2B	1.7053254

POLR2C	1.4214952
POLR2E	0.5859287
POLR2F	1.5216639
POLR2G	0.91713357
POLR2H	1.7966096
POLR2I	0.9085214
POLR2J	1.4854605
POLR2K	1.3728502
POLR2L	2.2259858
POLR2M	1.2208822
POLR3A	1.729563
POLR3B	1.4091523
POLR3C	1.8230846
POLR3D	3.1320298
POLR3E	1.2241645
POLR3G	0.27144122
POLR3GL	0.77629685
POLR3K	0.4995072
POLRMT	2.3502886
POM121	1.4967191
POMGNT1	1.4741156
POMGNT2	0.60088086
POMK	0.8137908
POMP	1.6065252
POMT1	0.6639726
POMT2	1.6113484
PON1	3.747481
PON2	0.73412895
PON3	1.2146094
POP1	0.7590058
POP4	1.53665
POP5	1.8087051
POP7	1.5931699
POR	3.284744
POT1A	3.096863
POU2F2	0.5081842
POU5F2	0.534044
POU6F1	0.9338629
PPA1	4.607114
PPA2	1.1527407
PPAN	2.5259583
PPAP2A	0.39546323
PPAP2B	1.3741362
PPAP2C	3.6151998
PPAPDC1B	3.4203203

PPAPDC2	0.74792695
PPARA	3.8113139
PPARD	0.8829863
PPARG	1.2625077
PPARGC1A	4.914756
PPAT	1.698837
PPCS	1.5607722
PPDPF	0.26591086
PPFIA1	1.2317398
PPFIA3	1.6476197
PPFIBP1	1.2242692
PPFIBP2	1.1558893
PPIA	1.8299265
PPIB	2.717601
PPID	0.95958686
PPIF	3.5185144
PPIFOS	1.5601342
PPIG	1.2247293
PPIL1	0.4278798
PPIL3	0.97152305
PPIL4	1.2823107
PPIP5K1	0.8803439
PPIP5K2	2.719528
PPM1A	0.5904834
PPM1B	2.014663
PPM1F	0.60616803
PPM1G	1.2304537
PPM1H	1.2994473
PPM1K	2.0104158
PPM1L	2.6029594
PPME1	2.3451807
PPNR	1.549412
PPP1CA	0.9332497
PPP1CB	0.9127495
PPP1CC	1.2954338
PPP1R11	2.011823
PPP1R12A	0.6439645
PPP1R12C	1.005564
PPP1R13L	2.0668304
PPP1R14B	1.6989739
PPP1R15A	2.1562707
PPP1R15B	0.4890535
PPP1R16A	1.6406305
PPP1R21	1.6387699
PPP1R35	0.78130555

PPP1R37	1.0697248
PPP1R3B	2.8555357
PPP1R3G	9.031089
PPP1R7	1.9361613
PPP2CA	1.9027636
PPP2CB	1.0935705
PPP2R1A	1.3852398
PPP2R1B	0.60673356
PPP2R2A	1.5098298
PPP2R2D	2.8114069
PPP2R3C	2.0675762
PPP2R4	0.75296474
PPP2R5A	0.4089396
PPP2R5B	2.0692642
PPP2R5D	0.8324306
PPP3CC	0.6255996
PPP3R1	0.82769036
PPP4C	1.2148144
PPP4R1	1.7557318
PPP4R2	1.1886289
PPP4R4	1.0786502
PPP5C	1.191756
PPP6C	1.5801198
PPP6R1	0.68478656
PPP6R3	1.3943594
PPRC1	2.2763364
PPT1	1.0719364
PPT2	1.5778635
PPTC7	1.5240958
PQBP1	0.60917616
PQLC1	1.3405569
PQLC2	5.0406504
PRAMEF8	1.5161917
PRCC	0.6054399
PRDM2	0.8624928
PRDM4	1.7390139
PRDX1	2.7438796
PRDX2	0.97061944
PRDX3	2.9451244
PRDX4	2.3419235
PRDX5	3.5195863
PREB	3.1238377
PRELID1	1.3173325
PRELID2	5.0426283
PREPL	1.4658058

PRG4	7.104583
PRICKLE3	0.74920344
PRIM1	1.0932786
PRIMPOL	0.66586757
PRKAA1	1.036577
PRKAA2	2.2955096
PRKAB2	0.8194208
PRKACB	0.8865001
PRKAG1	1.3975909
PRKAG2	0.9413302
PRKAR1A	2.418461
PRKAR2A	0.8401141
PRKCB	1.8187664
PRKCSH	3.1988218
PRKD3	0.7040713
PRKDC	0.8393569
PRKRIP1	1.3620055
PRKRIR	0.5234699
PRLR	3.6236238
PRMT1	1.4519479
PRMT10	1.7733562
PRMT3	2.0789807
PRMT5	1.4099095
PRMT7	1.673342
PRNP	0.9483168
PROC	8.430636
PROCR	5.4188013
PRODH	3.205177
PRODH2	5.5446587
PROK2	4.560918
PRORSD1	2.4655912
PROSC	3.05838
PROSER2	1.2364166
PROX1	4.9716415
PROZ	6.116661
PRPF18	1.0038273
PRPF19	2.226928
PRPF3	0.5628803
PRPF31	1.8362544
PRPF38A	1.8089693
PRPF38B	1.4029062
PRPF39	1.3780646
PRPF4	1.1975677
PRPF40A	2.8688185
PRPF40B	0.3980186

PRPF4B	1.4107928
PRPF6	2.3354466
PRPF8	1.4500463
PRPS1	1.927243
PRPS1L3	1.3897645
PRPSAP1	2.1291125
PRPSAP2	0.9778893
PRR14	1.0604174
PRR14L	1.4976509
PRR16	2.4403331
PRR24	0.7982924
PRRC1	0.5624025
PRRC2A	1.0216191
PRRC2C	1.0272291
PRRG2	1.0289204
PRSS29	0.4670329
PRSS37	1.5411704
PRSS51	2.515652
PSAP	0.6529629
PSAT1	1.9521296
PSD	0.43827796
PSD3	1.2721565
PSEN1	2.613765
PSEN2	2.723194
PSENEN	1.661402
PSG16	0.7954619
PSG23	4.1755323
PSG-PS1	2.049845
PSIP1	0.66631436
PSMA1	2.3598735
PSMA2	1.8246934
PSMA3	1.9681704
PSMA4	2.5531738
PSMA5	2.493365
PSMA6	2.3955052
PSMA7	2.702419
PSMB1	1.6063979
PSMB10	2.7331088
PSMB2	1.5918543
PSMB3	1.8156049
PSMB4	1.7072346
PSMB5	2.2803075
PSMB6	2.009318
PSMB7	2.3617456
PSMB8	1.600395

PSMB9	2.2254703
PSMC1	2.2352288
PSMC2	2.6010034
PSMC3	1.9042923
PSMC4	2.6416142
PSMC5	1.7622168
PSMC6	2.3521645
PSMD1	2.3744128
PSMD10	2.3901713
PSMD11	2.5178082
PSMD12	3.0432336
PSMD13	2.0882323
PSMD14	2.1390703
PSMD2	2.5317767
PSMD3	2.6707065
PSMD4	2.5235574
PSMD5	1.5384433
PSMD6	2.3541062
PSMD7	2.1885955
PSMD8	3.10621
PSMD9	2.8612554
PSME1	1.9957645
PSME2B	2.051069
PSME3	3.5088704
PSME4	2.0488274
PSMF1	1.9789069
PSMG1	2.0415533
PSMG2	0.4870937
PSMG3	1.7633274
PSMG4	2.3197815
PSPC1	2.9129627
PSPH	0.9960487
PSTPIP2	1.4007766
PTAR1	1.0098834
PTBP1	2.1304715
PTBP3	1.2190711
PTCD2	1.6966641
PTCD3	2.1929572
PTCH2	0.95438886
PTDSS1	0.6681144
PTEN	1.3819973
PTER	4.6712017
PTGES	2.0395505
PTGES2	3.401333
PTGES3	1.828512

PTGR2	0.8248193
PTGS2	1.7139003
PTH2R	1.8393326
PTMA	0.6118953
PTP4A1	4.686947
PTP4A2	0.6018536
PTPLA	2.028054
PTPLAD1	1.9472568
PTPLB	3.4200246
PTPMT1	2.485534
PTPN11	1.483566
PTPN12	1.3500817
PTPN2	3.131045
PTPN21	1.2872312
PTPN22	1.5000999
PTPN23	3.028694
PTPN3	2.9551356
PTPN5	0.8682864
PTPN6	1.1114142
PTPRA	0.771507
PTPRF	0.5449264
PTPRJ	0.8756354
PTRH1	1.9548671
PTRH2	0.69217324
PTRHD1	1.5816963
PTS	0.8277893
PTTG1IP	1.6291721
PTX3	1.8712857
PUF60	1.3949983
PUM1	1.131715
PUM2	1.0941813
PURB	0.57526183
PUS1	2.6845667
PUS10	2.0443928
PUS3	1.4562447
PUS7	1.5720341
PUS7L	0.6648452
PUSL1	1.3595941
PVR	3.5604532
PVRL1	2.266711
PVRL2	1.5727742
PWP1	2.5293553
PWP2	2.306999
PXDC1	1.6146057
PXK	2.1700997

PXMP2	3.0302618
PXMP4	2.672866
PXN	0.4828465
PYCARD	0.4968822
PYCRL	1.041569
PYDC3	1.3454185
PYGL	1.2082922
PYGO2	1.6501024
PYHIN1	1.3129101
PYROXD2	1.8637674
PYURF	0.90133643
PZP	7.5044346
QARS	2.2405107
QDPR	1.9218433
QPCT	2.4410121
QPCTL	2.77722
QPRT	5.4057293
QRICH1	1.4220002
QRSL1	1.9945292
QSER1	0.7641573
QSOX1	4.765438
QTRTD1	2.4523582
R3HCC1	1.4506514
R3HCC1L	1.2189815
R3HDM1	1.704005
R3HDM2	1.3761308
RAB1	2.8238857
RAB10	1.3667014
RAB11A	1.990011
RAB11B	0.87519765
RAB11FIP4	0.33147144
RAB12	0.39371657
RAB13	0.8455162
RAB14	1.0101969
RAB17	0.46514773
RAB18	2.0244634
RAB1B	0.7204282
RAB20	1.7706454
RAB21	1.2232432
RAB22A	2.4195173
RAB23	0.3226006
RAB24	2.294884
RAB26OS	3.2370038
RAB28	0.7721484
RAB2A	1.4987581

RAB2B	0.691201
RAB30	3.6369317
RAB31	0.9241369
RAB32	2.6956232
RAB33B	1.1118886
RAB35	1.7226393
RAB36	0.8042989
RAB3C	1.1158984
RAB3GAP1	2.046744
RAB3GAP2	2.060345
RAB3IP	2.0243375
RAB40C	1.6556919
RAB43	2.2036917
RAB4A	1.8695166
RAB4B	1.7033517
RAB5A	2.1403172
RAB5B	0.65530705
RAB5C	0.5750773
RAB6A	1.7413356
RAB7	3.529906
RAB7L1	0.67803645
RAB8A	1.5910599
RAB9	2.3911397
RABAC1	0.90360093
RABEP1	1.3490818
RABEP2	0.81304574
RABEPK	0.97407126
RABGEF1	1.2795198
RABGGTA	1.5579095
RABGGTB	2.40188
RABIF	1.0141196
RABL2	0.6136904
RABL3	2.7322567
RAC1	1.4253738
RAC2	1.3408573
RAD1	0.7695582
RAD17	2.5590289
RAD18	2.3237355
RAD21	0.91627955
RAD23B	2.5544674
RAD51B	1.5853989
RAD51C	3.1553295
RAD51D	0.49197197
RAD52	1.8163688
RAD54L2	1.8310437

RAE1	1.9624527
RAF1	1.4819534
RAI1	0.654299
RALA	1.149101
RALB	1.2436302
RALGAPA1	0.6790805
RALGAPA2	1.7251947
RALGAPB	0.5383575
RALGDS	2.7592394
RALGPS1	2.9343917
RALGPS2	2.0468047
RALY	0.6735909
RAN	2.4012926
RANBP1	1.9975336
RANBP10	1.5811851
RANBP2	1.974982
RANBP3	2.6294482
RANBP9	1.6687362
RANGAP1	2.8662202
RAP1B	0.7693126
RAP1GAP	0.4388864
RAP1GDS1	0.92709184
RAP2A	0.38059402
RAPGEF2	2.285519
RAPGEF4	3.472611
RAPGEF6	0.89774585
RAPH1	4.3026342
RAPSN	0.7813189
RARRES1	3.2198594
RARRES2	2.283332
RARS	2.7950845
RARS2	2.0391548
RASA1	0.71719384
RASA2	0.66416955
RASA4	1.6421449
RASAL1	4.669737
RASD1	4.654708
RASD2	1.2901237
RASGEF1B	3.8836105
RASIP1	0.39412284
RASL2-9	2.493758
RASSF1	1.5326211
RASSF4	1.3902495
RASSF5	0.73909783
RASSF6	2.294602

RB1	0.46425748
RBAK	1.105835
RBBP4	0.966882
RBBP5	1.8421652
RBBP6	1.6521094
RBBP7	1.4638832
RBBP8	1.132314
RBBP9	0.4981234
RBCK1	1.9652832
RBFA	1.1780179
RBFOX2	1.7861941
RBKS	3.6787412
RBM10	1.678704
RBM12	1.3781831
RBM12B1	2.298841
RBM14	1.4303696
RBM15	3.3732512
RBM17	2.3158991
RBM18	1.3759019
RBM19	2.6867626
RBM22	1.3936062
RBM25	1.3612173
RBM26	1.7142231
RBM27	1.5015888
RBM28	1.3526189
RBM3	1.9300044
RBM33	3.1203868
RBM34	1.57781
RBM39	1.5383928
RBM3OS	2.4141047
RBM4	2.0141876
RBM42	1.9040916
RBM43	0.7466996
RBM45	1.2918344
RBM47	1.5165999
RBM48	1.3393126
RBM4B	2.6769326
RBM5	0.87475324
RBM6	1.0349925
RBM7	1.1731217
RBM8A	1.65889
RBMS1	1.1523197
RBMX	1.3072264
RBMXL1	1.3288519
RBP4	6.488577

RBPMS	1.0471327
RBPMS2	3.4889023
RBX1	1.3042257
RC3H1	0.77436566
RC3H2	1.1963108
RCAN1	3.113497
RCAN2	1.9588883
RCBTB1	2.027078
RCBTB2	1.1933653
RCC2	1.9235704
RCCD1	0.7887912
RCE1	0.95727897
RCHY1	2.1950295
RCL1	6.009879
RCN1	0.41977715
RCOR1	1.0641544
RCOR3	0.68600535
RDH1	2.2550294
RDH10	1.5151751
RDH11	2.6415904
RDH12	0.5997617
RDH13	2.0406709
RDH14	2.7128656
RDH5	4.5128326
RDH7	4.526211
RDH9	2.7902296
RDX	1.0065596
REC114	0.7272408
RECQL5	0.38789225
REEP3	2.522476
REEP4	0.5774591
REEP6	2.6892688
REG3B	10.502556
REG3G	1.6734273
RELA	2.9477847
RELB	1.4823511
RELL1	2.0741994
REN1	1.0357554
REN2	5.1134815
RENBP	0.42809796
REPIN1	2.8600261
REPS1	2.1141899
REPS2	1.1605084
RER1	2.0355318
REST	1.2875826

RETSAT	3.6918662
REV3L	0.90564656
REXO1	1.8215015
REXO2	1.8904445
REXO4	1.728677
RFC1	1.048096
RFC2	0.9794848
RFC5	0.8993013
RFK	0.9872768
RFT1	2.2952678
RFTN1	0.5175612
RFWD2	1.5294015
RFWD3	1.496392
RFX1	0.65547824
RFX5	1.1449742
RFXANK	0.8401687
RFXAP	0.5189786
RGL1	0.60117316
RGL3	3.6458657
RGN	6.7069407
RGS1	2.7510073
RGS16	6.753647
RGS20	1.2121861
RGS4	0.44319558
RGS5	4.0777664
RHBDD2	1.877734
RHBDD3	1.9830086
RHBDF1	1.0187595
RHBDF2	2.626396
RHBDL1	0.47936726
RHBG	2.3086975
RHEB	1.3739245
RHEBL1	1.0398998
RHNO1	0.89901924
RHOA	0.5634415
RHOB	0.85016704
RHOBTB1	1.6992977
RHOBTB2	0.403167
RHOBTB3	1.1643541
RHOC	2.7403939
RHOD	3.2909858
RHOG	0.35139441
RHOQ	1.734669
RHOT1	1.2138493
RHOT2	1.3155258

RHOU	4.5693045
RHOX8	1.9959018
RIBC1	2.008474
RIC8	1.6733234
RIC8B	0.6782658
RICTOR	1.8903568
RIF1	1.1205864
RILP	1.8749435
RILPL2	1.6488588
RINT1	2.0257585
RIOK1	0.9713557
RIOK2	2.6166875
RIOK3	1.83763
RIPK1	2.6817415
RIPK2	3.9448712
RIPK3	2.0395086
RIPK4	0.693877
RIT1	1.3138702
RLF	2.149999
RLIM	1.6567647
RMDN1	3.6733387
RMDN2	0.35167623
RMDN3	3.0224006
RMND1	2.3859107
RMND5A	0.8924515
RMND5B	1.0467997
RMRP	0.545511
RN18S	1.7223043
RN28S1	1.9589539
RN4.5S	2.4602757
RN45S	1.9349606
RNASE10	0.9978354
RNASE13	2.6955366
RNASE4	3.7178028
RNASEH1	1.3484895
RNASEH2A	1.5484023
RNASEH2B	1.8868349
RNASEK	1.314182
RNASET2A	1.3980033
RND1	6.654503
RND3	2.3525217
RNF10	1.5894482
RNF103	2.304242
RNF11	1.8166487
RNF111	1.6565406

RNF113A1	0.81844115
RNF113A2	2.368056
RNF114	1.8111432
RNF115	1.4271591
RNF121	1.4059069
RNF123	2.59535
RNF125	5.244853
RNF126	0.29663348
RNF128	5.142874
RNF13	2.060363
RNF130	2.0800889
RNF135	1.4341352
RNF138	1.5348585
RNF138RT1	3.5747187
RNF139	1.5123551
RNF14	2.4340842
RNF146	1.0053847
RNF149	3.9909418
RNF152	1.4634507
RNF167	0.6803739
RNF168	0.9319961
RNF169	1.9528043
RNF170	0.99897265
RNF181	0.6203196
RNF185	2.4069426
RNF187	0.7437732
RNF19A	0.9766834
RNF19B	2.167806
RNF2	1.4500611
RNF213	3.082947
RNF214	0.9026108
RNF215	0.5540812
RNF216	1.5482051
RNF217	2.8236892
RNF220	1.1183803
RNF224	0.46284962
RNF24	1.378334
RNF25	0.6618378
RNF26	1.2274592
RNF31	1.4889987
RNF34	0.8525965
RNF4	2.2152994
RNF40	3.1497443
RNF41	0.8652072
RNF43	0.42505145

RNF44	1.2544677
RNF5	0.75418687
RNF6	1.4554074
RNF7	1.3937795
RNF8	1.0510566
RNFT1	1.9664619
RNGTT	0.7394674
RNH1	1.8476737
RNLS	0.5466826
RNMT	1.6766679
RNMTL1	0.6992619
RNPC3	1.5800211
RNPEP	1.1039755
RNPEPL1	0.7693436
RNPS1	1.6210535
RNU12	0.94806504
RNU1B6	1.3751953
RNU2-10	1.3157852
RNU3B1	2.6677797
ROCK1	0.43993497
ROM1	0.6369698
ROMO1	1.9083116
ROPN1L	2.387439
RORA	1.9050133
RORC	1.7299197
RP9	2.300001
RPA1	0.5203688
RPA2	1.0036864
RPAIN	2.3023975
RPAP1	1.470257
RPAP2	2.137521
RPAP3	4.711795
RPE	1.3650429
RPF1	1.8871748
RPF2	2.756356
RPGRIP1	1.4711993
RPL10	1.8148565
RPL10A	2.2398138
RPL11	1.6336162
RPL12	1.8017728
RPL13	1.4312007
RPL13A	1.5129292
RPL14	0.9005172
RPL15	1.6629698
RPL17	1.547971

RPL18	1.7334483
RPL18A	1.2940538
RPL19	1.7108319
RPL21	1.2656877
RPL22	1.4696724
RPL22L1	2.622699
RPL23	1.3922422
RPL23A	1.0933573
RPL24	1.3552201
RPL26	1.3503673
RPL27	1.4520681
RPL27A	1.5461714
RPL28	1.4437006
RPL29	1.5623324
RPL3	1.675545
RPL30	1.1137993
RPL31	1.2279556
RPL31-PS12	1.1651661
RPL32	0.6523187
RPL34	1.5049684
RPL34-PS1	0.91723895
RPL35	1.4430418
RPL35A	1.2971017
RPL36	1.6406338
RPL36A	1.6566584
RPL36AL	2.3121736
RPL37A	1.573554
RPL38	1.8168449
RPL39	1.4521492
RPL39L	1.5682368
RPL4	1.881005
RPL41	2.5365152
RPL5	1.0801904
RPL5-PS2	1.4935043
RPL6	1.4798105
RPL7	1.7923429
RPL7A	1.9133928
RPL7L1	2.4715016
RPL8	1.4138305
RPL9	1.2895021
RPLP0	1.7852023
RPLP1	1.1699188
RPLP2	1.8923082
RPN1	3.351967
RPN2	1.9023845

RPP14	1.6122587
RPP21	1.3712752
RPP25L	1.4076092
RPP30	1.5056932
RPP38	2.1276739
RPP40	3.5655458
RPRD1B	0.70791554
RPRD2	0.92761374
RPS10	1.2719283
RPS11	1.4690268
RPS11-PS1	1.4334285
RPS12	1.7012725
RPS12L2	1.6185794
RPS12-PS16	1.536216
RPS13	1.7040832
RPS14	1.5376263
RPS15	0.7275398
RPS15A	1.6501243
RPS16	1.5092804
RPS17	1.5823572
RPS18	1.7455518
RPS19	0.9768932
RPS19BP1	2.1930916
RPS2	2.175472
RPS20	1.6561928
RPS23	1.637419
RPS24	1.1668942
RPS25	1.4241521
RPS26	1.4218042
RPS27	1.5406742
RPS27A	1.5959189
RPS27L	3.3917563
RPS28	1.6278234
RPS29	1.1763668
RPS3	1.5794532
RPS3A1	1.4191716
RPS4L	2.0020487
RPS4X	1.3300893
RPS5	1.6536856
RPS6	1.2863352
RPS6KA3	0.97905993
RPS6KA4	1.2864478
RPS6KB1	2.0456073
RPS6KB2	1.1851163
RPS6KC1	0.9241991

RPS6KL1	1.0194943
RPS7	2.1231492
RPS8	1.5524876
RPS9	1.27278
RPSA	1.8927338
RPTOR	1.6439393
RPUSD3	2.7363641
RPUSD4	0.39378333
RQCD1	2.3973038
RRAGA	3.0124876
RRAGC	2.276883
RRAS	0.9827521
RRAS2	1.5440142
RRBP1	3.1044352
RREB1	1.4997313
RRM1	0.3474853
RRN3	1.7230985
RRNAD1	0.50673103
RRP1	1.4697154
RRP15	2.3979275
RRP1B	0.5235119
RRP7A	1.6042078
RRP8	1.8534474
RRP9	2.13393
RRS1	2.5354397
RS1	0.4387548
RS5-8S1	1.3843277
RSAD1	0.7506585
RSAD2	3.7793596
RSL1	1.9426091
RSL1D1	2.2706468
RSL24D1	1.2978442
RSPH3A	1.1212757
RSPH3B	2.175114
RSPH4A	1.0595024
RSPO3	2.623648
RSPRY1	1.980057
RSRC2	1.8996418
RSRP1	1.4851859
RSU1	0.32803988
RTCB	1.5629699
RTEL1	1.3282211
RTF1	1.1828144
RTFDC1	1.7425864
RTN3	1.2891705

RTN4	0.98480916
RTN4IP1	0.827626
RTN4RL2	1.5554256
RTP3	4.78409
RTP4	2.0718172
RUFY1	1.375994
RUFY2	0.6242316
RUFY3	1.7093842
RUNDC1	1.5167634
RUNDC3B	1.7512865
RUNX1	0.6101053
RUSC1	0.8858073
RUSC2	1.7406127
RUVBL1	1.6613228
RWDD2B	1.2224433
RWDD4A	0.7688768
RXRA	2.3270087
RXRB	2.6801684
RXRG	0.8702009
RYBP	0.9010427
RYK	0.8042743
S100A10	0.7108786
S100A11	0.8462446
S100A8	1.6714017
S100A9	1.8159254
S100PBP	0.7443912
S1PR2	1.2160103
SAA1	8.592663
SAA2	10.639465
SAA3	7.9789724
SAA4	5.556882
SAC3D1	1.1289642
SACM1L	1.7654445
SACS	1.4334276
SAE1	0.7662575
SAFB	1.5239794
SAFB2	1.753032
SALL1	6.3707523
SAMD1	1.0183914
SAMD10	1.4928992
SAMD4B	2.248309
SAMD8	2.2533638
SAMD9L	1.805412
SAMHD1	0.3036263
SAMM50	2.5231774

SAMSN1	1.598048
SAP130	1.6380918
SAP18	0.5096719
SAP30	1.302948
SAP30BP	1.937793
SAP30L	1.0845053
SAR1A	1.8299124
SAR1B	2.26817
SARDH	3.4387047
SARNP	1.4461544
SARS	2.1547468
SARS2	0.5070455
SART3	1.5790966
SASH1	1.9030464
SAT1	1.4610007
SAT2	0.73134685
SATB1	0.25551176
SAV1	1.1551974
SBDS	1.7927353
SBF1	2.0591018
SBF2	0.85744834
SBNO1	1.468435
SBNO2	2.1563456
SC5D	3.7884667
SCAF1	0.87223697
SCAF4	1.2243879
SCAF8	1.3152153
SCAMP1	2.0311615
SCAMP2	0.89609265
SCAMP3	1.9082644
SCAMP4	2.2479231
SCAMP5	3.4902434
SCAND1	1.785002
SCAP	2.6914694
SCAPER	0.95085335
SCARA5	0.89992356
SCARB1	2.5341952
SCARB2	1.5383785
SCFD1	2.1751478
SCFD2	2.1241376
SCG5	1.600347
SCIMP	1.1563385
SCLY	3.4598272
SCN1B	1.0303524
SCN5A	0.5606313

SCN8A	2.3405836
SCNM1	1.1604936
SCO2	3.4256642
SCOC	0.6410761
SCP2	3.6061208
SCPEP1	2.2716935
SCRIB	1.7044156
SCRN2	2.3533804
SCRN3	2.0481303
SCYL1	2.1261704
SCYL2	2.894787
SCYL3	1.8987396
SDAD1	2.190386
SDC1	2.5777724
SDC2	1.8372056
SDC4	4.793871
SDCBP	1.0514553
SDCCAG3	1.4087102
SDCCAG8	0.8157823
SDE2	1.7666414
SDF2	1.7232492
SDF2L1	3.9936721
SDF4	1.6388142
SDHA	2.52544
SDHAF1	1.9932258
SDHAF2	1.7317293
SDHB	2.198928
SDHC	2.2290537
SDR39U1	1.5264771
SDR42E1	5.015441
SDS	6.087145
SDSL	7.2748795
SEBOX	2.5010142
SEC1	2.750982
SEC11A	2.017423
SEC11C	1.4572732
SEC13	2.3417323
SEC14L2	3.1464703
SEC14L4	1.9487016
SEC16A	3.0600421
SEC16B	4.395461
SEC22B	2.0358937
SEC23A	2.984462
SEC23B	2.2238667
SEC23IP	2.0416343

SEC24A	3.2739766
SEC24B	1.5101984
SEC24C	1.8461754
SEC24D	3.4788754
SEC31A	2.3697202
SEC31B	0.8572352
SEC61A1	2.9561646
SEC61B	2.2582881
SEC61G	2.3421462
SEC62	1.7260487
SEC63	2.5153644
SECISBP2	1.323703
SECISBP2L	0.6424606
SEH1L	2.6288383
SEL1L	1.4680789
SEL1L3	1.2841549
SELE	1.4767208
SELENBP1	1.1684844
SELK	2.6437705
SELL	0.78937554
SELO	2.8025262
SELP	4.7967415
SELT	1.8010538
SEMA4A	2.3207972
SEMA4B	1.0677824
SEMA4C	3.3779821
SEMA4G	3.697138
SEMA6B	2.8097298
SEMA6D	3.1023657
SENP2	1.3302267
SENP3	1.5684793
SENP5	1.7125888
SENP7	0.4535694
SEPHS2	4.440526
SEPN1	0.33833385
SEPP1	2.9790626
SEPSECS	1.9132509
SEPW1	0.5122292
SERBP1	2.2113569
SERF1	1.0765598
SERF2	1.3548481
SERGEF	2.2519386
SERHL	1.6074317
SERINC1	2.586374
SERINC3	1.5168622

SERINC5	1.8874338
SERP1	3.3321874
SERPINA10	10.216758
SERPINA11	6.8673925
SERPINA1A	8.537084
SERPINA1C	8.161804
SERPINA1D	9.447071
SERPINA1E	8.505521
SERPINA3C	6.4998426
SERPINA3F	4.39835
SERPINA3G	3.3070114
SERPINA3H	3.491893
SERPINA3I	5.3376207
SERPINA3K	12.490768
SERPINA3M	9.015566
SERPINA3N	9.388249
SERPINA4-PS	1.7184267
SERPINA6	5.7121725
SERPINA7	6.7677774
SERPINB2	2.5224411
SERPINB7	0.6330843
SERPINB8	3.3357236
SERPINB9B	1.8249631
SERPINC1	8.95936
SERPIND1	7.4273167
SERPINE1	7.1763334
SERPINF1	4.8805523
SERPINF2	8.38274
SERPING1	4.0239553
SERTAD1	1.1512668
SERTAD2	0.85892415
SERTAD3	0.8076234
SESN2	0.8352711
SET	1.7016532
SETD1A	1.7206028
SETD1B	1.1146567
SETD2	1.3880441
SETD4	0.9232385
SETD5	1.0295074
SETD6	2.381039
SETD7	0.36456704
SETD8	0.47354436
SETDB1	1.4369619
SETMAR	1.0490458
SETX	2.1422226

SEZ6L	1.7101479
SF1	1.6818593
SF3A1	1.7112257
SF3A3	1.6483276
SF3B2	1.7549894
SF3B3	1.7255313
SF3B4	1.3241761
SF3B5	1.4752333
SF3B6	1.6393678
SFI1	1.6531341
SFMBT1	2.1521595
SFPQ	1.663728
SFR1	2.1189811
SFSWAP	1.3865621
SFT2D1	1.4604003
SFT2D2	0.9597485
SFT2D3	1.5593865
SFXN1	3.411639
SFXN2	0.617306
SGK1	0.7308347
SGK2	3.133221
SGK3	0.9914911
SGMS1	2.5544136
SGPL1	2.1971757
SGPP1	1.4150145
SGSH	3.342448
SGSM1	1.5473835
SGSM3	1.6530468
SGTA	1.7228987
SH2B1	3.6318924
SH2B2	3.0663602
SH2D4B	1.2990923
SH3BGRL	0.45531774
SH3BGRL2	1.2190831
SH3BP2	1.9038346
SH3BP5	0.30017734
SH3BP5L	0.2933004
SH3D19	0.311754
SH3GL1	1.3268864
SH3GLB1	1.8547995
SH3GLB2	0.39450908
SH3PXD2B	1.4450848
SH3YL1	2.8990724
SHARPIN	1.7410991
SHB	1.4275591

SHC1	2.0966942
SHC4	0.53207946
SHF	1.5526373
SHFM1	1.4479654
SHISA5	0.9032018
SHKBP1	1.0605724
SHMT1	2.58587
SHMT2	2.458195
SHOC2	1.8239987
SHPK	2.7774985
SHQ1	2.4293694
SHROOM1	0.30330396
SHROOM2	1.4395688
SIAE	1.3055952
SIAH1A	0.7753875
SIAH1B	0.41657996
SIAH2	0.80493283
SIDT2	2.7326968
SIGIRR	1.4262502
SIGLECE	1.3538759
SIGLECH	1.9327471
SIGMAR1	2.1967347
SIK1	1.7102387
SIK2	1.5824964
SIK3	1.8464582
SIKE1	0.7787874
SIL1	2.5697
SIMC1	0.46529293
SIN3A	1.8848369
SIN3B	0.83692336
SIPA1L1	0.7914989
SIRPA	1.0712631
SIRT1	1.2972262
SIRT2	1.2407863
SIRT3	3.3226564
SIRT4	0.32824397
SIRT5	1.3504312
SIRT7	1.0462768
SKAP2	1.0015032
SKIL	1.1434381
SKINT7	3.1883748
SKIV2L	1.6591694
SKIV2L2	1.1687138
SKP1A	0.9630983
SLA	1.6205881

SLAIN2	1.4616053
SLAMF1	4.515275
SLAMF7	1.9867837
SLAMF9	1.2511308
SLBP	0.68222547
SLC10A1	2.2013512
SLC10A3	2.3099558
SLC10A6	4.07749
SLC11A1	3.6465442
SLC11A2	1.8921854
SLC12A1	0.5998523
SLC12A4	2.0292509
SLC12A5	0.7459967
SLC12A7	1.4772274
SLC12A9	0.73044634
SLC13A3	5.236945
SLC13A5	6.118149
SLC15A3	2.4523275
SLC15A4	1.7129819
SLC16A10	3.6785495
SLC16A14	0.53294325
SLC16A2	0.8851893
SLC16A3	2.8952672
SLC16A5	5.0379457
SLC16A6	4.1011534
SLC17A1	10.86853
SLC17A2	7.1264515
SLC17A3	2.0131934
SLC17A6	1.1791043
SLC17A9	3.6460607
SLC19A1	1.5443075
SLC19A2	4.8070383
SLC1A2	3.48921
SLC1A4	3.4876316
SLC20A1	3.5765069
SLC20A2	2.2374218
SLC22A18	2.6212685
SLC22A21	1.816072
SLC22A26	8.489222
SLC22A27	4.084005
SLC22A29	2.8621128
SLC22A5	3.0249817
SLC22A6	1.409019
SLC22A7	1.7957747
SLC23A1	3.1389372

SLC24A5	1.8790085
SLC25A1	2.9749262
SLC25A11	1.3492453
SLC25A12	2.5595987
SLC25A13	4.5517893
SLC25A14	1.0788319
SLC25A15	4.106596
SLC25A16	2.2046187
SLC25A17	0.91065097
SLC25A19	0.921407
SLC25A20	2.1643069
SLC25A21	1.8325086
SLC25A22	5.573862
SLC25A25	4.835082
SLC25A26	0.94141364
SLC25A28	2.3619487
SLC25A3	2.497411
SLC25A30	2.0882318
SLC25A32	2.6632898
SLC25A33	4.176299
SLC25A34	1.6483982
SLC25A38	1.1707213
SLC25A39	3.4754436
SLC25A40	1.022234
SLC25A42	3.6422813
SLC25A44	1.9802263
SLC25A45	0.49567747
SLC25A46	1.5093901
SLC25A47	8.856436
SLC25A5	2.4817407
SLC25A51	1.6886184
SLC26A1	5.2035294
SLC26A11	1.7333496
SLC26A2	0.6776316
SLC26A6	0.3567841
SLC27A2	7.8536577
SLC27A5	9.353384
SLC29A1	1.283539
SLC29A2	1.726541
SLC2A1	3.5512712
SLC2A2	5.8043823
SLC2A4RG-PS	0.7446563
SLC2A5	3.7014506
SLC2A6	2.934305
SLC2A8	0.98343396

SLC2A9	1.4722686
SLC30A1	1.3511293
SLC30A10	6.713048
SLC30A4	1.6670253
SLC30A5	2.4301336
SLC30A6	2.2043242
SLC30A7	1.6820295
SLC30A9	1.9161842
SLC31A1	1.530266
SLC31A2	2.000408
SLC33A1	2.829254
SLC35A1	1.2875321
SLC35A2	1.3963888
SLC35A3	2.3101637
SLC35A4	0.79612184
SLC35B1	3.3192112
SLC35B2	1.5940797
SLC35B3	2.03556
SLC35B4	0.76935554
SLC35C1	1.8353007
SLC35C2	1.9935215
SLC35D1	2.8071487
SLC35D2	3.3119423
SLC35E1	1.9439342
SLC35E2	0.6617348
SLC35E3	0.7994175
SLC35F5	3.3622677
SLC35F6	1.6350396
SLC35G1	2.7059596
SLC37A1	1.9671695
SLC37A3	2.6232402
SLC37A4	1.9675958
SLC38A1	0.43509555
SLC38A2	4.200495
SLC38A3	8.11275
SLC38A4	6.063301
SLC38A6	0.65825653
SLC38A7	0.3999908
SLC39A1	2.838487
SLC39A11	1.6011536
SLC39A13	0.638489
SLC39A14	6.6810617
SLC39A3	0.26777673
SLC39A4	2.9137518
SLC39A6	0.7296684

SLC39A7	2.3520958
SLC39A9	0.5596585
SLC3A1	6.7823343
SLC3A2	2.430466
SLC40A1	1.730252
SLC41A1	1.6074431
SLC41A2	6.348486
SLC41A3	1.9703581
SLC43A1	4.3942175
SLC43A2	0.4861653
SLC45A4	0.89765525
SLC46A1	1.7040775
SLC48A1	0.7060821
SLC4A1AP	0.4939673
SLC4A2	1.3850572
SLC4A4	6.2886333
SLC4A9	2.3616946
SLC51B	3.990708
SLC5A6	2.2491605
SLC6A12	7.428343
SLC6A13	5.6419983
SLC6A19	1.6703517
SLC6A6	0.69628644
SLC6A8	1.6208065
SLC6A9	1.4429004
SLC7A1	1.8662035
SLC7A11	0.41645312
SLC7A2	4.134715
SLC7A3	1.5772281
SLC7A5	1.0119014
SLC7A6OS	2.6127918
SLC7A7	1.7875383
SLC7A8	1.1828375
SLC8B1	1.9444602
SLC9A1	0.44851422
SLC9A3R1	3.2742693
SLC9A6	1.6837089
SLC9A8	2.3774903
SLCO1A1	2.4296699
SLCO1A4	2.739391
SLCO1B2	9.475862
SLCO2B1	1.3715391
SLFN2	1.6327655
SLFN3	0.834038
SLFN4	2.3606436

SLFN5	2.1196878
SLFN8	0.5559685
SLK	1.2152579
SLMAP	1.2435477
SLMO2	2.0715635
SLPI	5.052969
SLTM	2.180467
SLX4	1.6421874
SMAD1	1.400079
SMAD2	0.9051964
SMAD4	1.3614881
SMAD5	1.125973
SMAGP	1.2293141
SMAP1	1.2933357
SMAP2	1.3227351
SMARCA4	1.1636894
SMARCA5	1.548157
SMARCA5-PS	1.312432
SMARCAD1	1.5820396
SMARCB1	0.69554114
SMARCC1	0.5735376
SMARCC2	0.7876494
SMARCE1	0.28429055
SMC1A	1.0898106
SMC3	1.1494496
SMC4	0.43676257
SMC5	2.4124534
SMC6	0.84603906
SMCHD1	0.8916266
SMCO4	0.76565623
SMCR8	2.7258666
SMDT1	1.5707705
SMEK1	1.1359279
SMEK2	1.4400122
SMG1	2.4922745
SMG5	2.3488529
SMG6	1.0651162
SMG7	1.5189707
SMG9	1.0759461
SMIM11	0.7471769
SMIM12	1.2844994
SMIM13	1.8959372
SMIM14	1.6968262
SMIM19	1.7194393
SMIM20	2.5665066

SMIM3	0.54729486
SMIM7	1.831054
SMIM8	1.825525
SMLR1	7.37656
SMN1	1.2312644
SMNDC1	2.2379272
SMOC1	7.3053904
SMOC2	0.34099317
SMOX	2.2144945
SMPD1	2.2061422
SMPD3	1.8987956
SMPD4	1.1326377
SMPDL3A	0.7193868
SMPDL3B	0.7567377
SMU1	1.544627
SMURF2	0.2835791
SMYD1	0.90912294
SMYD2	1.2671449
SNAP23	1.3465021
SNAP25	0.38605714
SNAP29	1.7033751
SNAP47	1.2754796
SNAPC1	2.1199815
SNAPC2	1.3791959
SNAPC4	1.7610471
SNAPIN	0.49011588
SND1	1.8178518
SNF8	1.6210787
SNHG1	2.742852
SNHG12	3.230748
SNHG3	2.41669
SNHG5	0.96442604
SNHG6	4.341239
SNHG7	2.1651742
SNIP1	1.1116712
SNORA74A	1.4022174
SNRNP200	1.3831456
SNRNP27	1.436939
SNRNP48	2.3032243
SNRNP70	1.6241043
SNRPA	1.7027233
SNRPA1	1.7637489
SNRPB	0.71700406
SNRPB2	0.9293554
SNRPC	1.7571871

SNRPD1	1.3900645
SNRPD2	1.4278629
SNRPD3	1.3521597
SNRPE	1.0065477
SNRPG	0.91719556
SNRPN	0.9827161
SNTB2	2.7500536
SNTG1	0.3452494
SNTG2	2.2229838
SNUPN	1.2687938
SNW1	1.5282924
SNX1	1.3882864
SNX10	4.0256386
SNX11	0.9910338
SNX12	1.310889
SNX13	2.0396202
SNX14	1.3269413
SNX15	2.3220131
SNX16	2.2388752
SNX17	0.88286996
SNX18	2.1712983
SNX19	1.9090788
SNX2	1.0365136
SNX24	1.4403789
SNX27	0.6786916
SNX3	1.6724088
SNX30	0.85166454
SNX33	1.5743926
SNX4	1.084296
SNX5	1.4160469
SNX6	0.7412889
SNX7	2.3948448
SNX8	2.4053476
SNX9	1.2763474
SOAT2	6.162285
SOCS1	4.221631
SOCS2	0.9782679
SOCS3	4.5939703
SOCS4	1.3485217
SOCS7	1.4702575
SOD1	1.9786191
SOD2	5.5737934
SON	1.6007345
SORBS1	1.2909806
SORBS2	2.3776646

SORBS3	0.71492124
SORCS3	1.8882155
SORD	1.0204837
SOS2	0.80905795
SOWAHB	1.9583719
SOWAHC	2.7417986
SOWAHD	1.6009552
SOX9	5.796933
SP1	1.4174078
SP110	3.1357486
SP140	1.2638848
SPAG7	3.334159
SPAG9	1.1511586
SPAST	2.3020709
SPATA1	0.45544124
SPATA13	0.9084084
SPATA2	0.62375236
SPATA22	4.650294
SPATA5	2.3543599
SPATA5L1	0.9488051
SPATA7	0.3089404
SPATC1L	0.739347
SPCS1	1.9469063
SPCS2	3.8038118
SPCS3	2.5605633
SPDEF	0.89039993
SPEN	1.1418002
SPG11	1.3021066
SPG20	1.6536691
SPG21	1.7865522
SPG7	1.5410106
SPHK1	1.2945259
SPHK2	0.8719809
SPI1	0.2917695
SPIC	3.725939
SPIDR	2.3335412
SPIN1	1.9603298
SPINK3	4.629876
SPNS1	1.9953048
SPOP	0.40539908
SPP1	1.431256
SPP2	8.440487
SPPL2A	3.5885866
SPPL2B	1.3178685
SPR	1.2277715

SPRED1	1.3490546
SPRED2	1.3822823
SPRR1A	0.31284785
SPRR2A2	0.9005058
SPRR2D	2.0716653
SPRYD4	3.966575
SPRYD7	2.3154142
SPSB2	2.0004523
SPSB3	1.5406649
SPTAN1	0.40512633
SPTBN2	2.6637526
SPTLC1	1.0372188
SPTLC2	0.91585755
SPTSSA	0.7214143
SPTY2D1	2.1039479
SQLE	4.2430677
SQRDL	2.9852874
SQSTM1	2.781216
SRA1	3.126055
SRD5A1	0.63054776
SRD5A3	0.6077335
SREBF2	1.4728484
SREK1	3.6952798
SREK1IP1	1.163152
SRF	1.0070636
SRFBP1	2.6238663
SRGN	1.4704931
SRI	0.9935491
SRM	2.7131178
SRP14	0.95607257
SRP19	2.3830316
SRP54A	1.416487
SRP54C	3.0583751
SRP68	2.4122422
SRP72	2.653602
SRP9	2.3482454
SRPK1	2.7862308
SRPR	3.6057904
SRPRB	0.7631259
SRR	2.2261302
SRRD	1.6609678
SRRM1	0.81443954
SRRM2	0.34275603
SRRM4	5.142954
SRSF1	1.1713579

SRSF10	0.98466444
SRSF11	0.9175761
SRSF2	0.49978328
SRSF3	1.0008032
SRSF4	1.0647814
SRSF5	2.520491
SRSF6	2.0099752
SRSF7	1.7716148
SRSF9	1.2084224
SRXN1	4.833973
SS18	1.0195706
SS18L1	1.5586958
SSB	1.5850155
SSBP1	2.0433643
SSBP4	0.47661614
SSH1	1.9026842
SSH3	1.9394891
SSNA1	0.7266333
SSR1	2.0306804
SSR2	2.2215612
SSR3	2.6213639
SSR4	2.891608
SSRP1	1.0620048
SSSCA1	1.9803789
SSU72	2.4295042
SSX2IP	1.9367058
ST13	2.6621516
ST3GAL1	2.9770257
ST3GAL3	2.8644164
ST3GAL4	2.6319478
ST3GAL5	4.477087
ST5	1.1578557
ST6GAL1	3.2325227
ST6GALNAC4	1.321914
ST6GALNAC6	1.1469638
ST7	2.4494426
ST7L	1.8331373
ST8SIA3	1.5423112
STAB1	2.119325
STAB2	5.8527384
STAG1	1.7869413
STAG2	0.71262383
STAM	0.8353207
STAM2	1.2816365
STAMBP	1.0479753

STAMBPL1	0.71078706
STAP2	4.6295633
STARD10	2.493159
STARD3	0.9582441
STARD4	1.4455826
STARD5	2.6126096
STARD7	2.0776913
STAT2	1.9275601
STAT3	3.1285164
STAT5A	1.3024032
STAT5B	0.9447367
STAT6	1.3157108
STAU1	1.713018
STAU2	0.7706268
STBD1	2.3734343
STEAP1	5.0412426
STEAP2	3.8173225
STEAP4	8.876439
STFA1	0.5820453
STFA2L1	0.6865928
STFA3	0.8386259
STIM1	0.58117414
STIP1	1.9087327
STK11	1.4644239
STK11IP	1.6324778
STK16	1.4751618
STK17B	0.97616935
STK19	2.2932775
STK24	0.68623424
STK25	1.6981142
STK3	0.96688175
STK35	1.5749063
STK38	1.3149426
STK38L	1.5410821
STK40	2.3858984
STOM	1.2692273
STOML2	1.8348434
STRA13	0.54031444
STRADA	1.4409602
STRADB	0.7162924
STRAP	1.5576842
STRBP	2.5217311
STRIP1	1.2904313
STRN3	0.94203496
STRN4	1.6488554

STS	3.2543747
STT3A	2.5981004
STT3B	1.5797746
STUB1	1.811372
STX12	1.4582775
STX16	1.5735993
STX17	0.76307917
STX18	2.418197
STX2	0.6636715
STX4A	1.757716
STX5A	2.0042832
STX7	0.5771172
STX8	1.4494565
STXBP2	1.4252698
STXBP3A	2.5130904
STXBP3B	2.2801936
SUB1	1.0435436
SUCLA2	2.4359767
SUCLG1	2.3212378
SUCLG2	4.0168333
SUCNR1	1.7333832
SUCO	3.5207765
SUDS3	0.85557103
SUGP1	2.1102273
SUGT1	1.7642243
SULT1A1	4.3864393
SULT1B1	5.894328
SULT1C2	1.981482
SULT1D1	3.250322
SULT1E1	11.381954
SULT2A1	5.8045425
SULT2A2	5.9207125
SULT2A4	4.769102
SULT2A6	4.7991056
SULT3A1	3.8738682
SULT4A1	0.38064122
SUMF1	1.4593742
SUMF2	0.8076041
SUMO1	0.70468163
SUMO2	0.6100271
SUMO3	1.0163205
SUN2	1.8229821
SUOX	2.4297674
SUPT16	2.9330058
SUPT5	1.528897

SUPT6	1.1686313
SUPT7L	3.1661673
SUPV3L1	3.1026747
SURF1	0.752229
SURF2	1.8554957
SURF4	3.2424667
SURF6	1.7429283
SUSD2	1.5214684
SUV39H2	3.4304402
SUV420H1	1.2376559
SUV420H2	0.87480664
SUZ12	2.0349467
SVA	1.2640874
SWI5	2.6657436
SWT1	1.08236
SYAP1	1.367882
SYBU	1.9935951
SYCE2	1.467447
SYCP1	2.4037123
SYF2	1.2103136
SYK	1.3185232
SYMPK	2.1616714
SYNC	1.0058281
SYNCRIP	1.8754609
SYNGR2	0.98666215
SYNJ1	1.0998604
SYNJ2	1.2348793
SYNJ2BP	1.8576972
SYNPO	0.96985483
SYNPO2L	3.4614851
SYPL	0.9700725
SYS1	2.0647242
SYT1	3.0129232
SYT12	1.7364595
SYVN1	3.8489826
SZRD1	1.9422691
SZT2	0.6582644
TAB1	0.37772393
TAB2	1.8461864
TAB3	1.9684865
TAC1	2.4822946
TACC1	0.53731704
TACC2	1.4576352
TACC3	0.49944806
TACO1	2.4981422

TACR2	0.7583735
TADA1	1.5701301
TADA2A	1.3764293
TADA2B	1.1575675
TADA3	0.29414344
TAF1	0.45852065
TAF10	1.852555
TAF11	1.3265088
TAF12	1.4668171
TAF13	0.5689361
TAF15	0.7897074
TAF1B	1.4764462
TAF1C	1.1640301
TAF1D	2.0137804
TAF2	1.3428681
TAF3	1.1264312
TAF4A	1.3523209
TAF5	2.438471
TAF5L	1.790786
TAF6	1.1904209
TAF6L	1.4767268
TAF7L	1.3969357
TAF8	0.70408225
TAF9	3.0803602
TALDO1	1.042763
TANC1	0.7575884
TANGO2	1.9980028
TANK	1.7973905
TAOK3	0.34276533
TAP1	2.567372
TAP2	2.8332198
TAPBP	2.2525814
TAPBPL	2.6406224
TAPT1	2.1760132
TARBP2	1.9853394
TARDBP	1.4418514
TARS	3.3148406
TARS2	0.3476379
TARSL2	1.1002359
TAS1R3	1.1057773
TAS2R109	0.8648956
TASP1	1.2638354
TAT	9.93847
TATDN1	1.91224
TATDN2	0.6140785

TATDN3	0.9323404
TAX1BP1	2.2119462
TAX1BP3	0.72936845
TAZ	1.2960508
TBC1D1	0.54859805
TBC1D10A	0.34724355
TBC1D10B	1.4247873
TBC1D13	1.1247818
TBC1D14	1.0922768
TBC1D15	3.40461
TBC1D16	1.2733138
TBC1D17	2.2629378
TBC1D2	0.3991754
TBC1D20	1.452564
TBC1D22A	1.5578678
TBC1D23	1.3718102
TBC1D24	5.5905514
TBC1D25	0.88950515
TBC1D2B	0.54899955
TBC1D5	1.1939485
TBC1D7	1.0298941
TBC1D8	2.5273886
TBC1D8B	2.315234
TBC1D9	1.2710941
TBC1D9B	1.0419476
TBCA	1.1324594
TBCB	1.633456
TBCC	1.7022302
TBCCD1	2.2852106
TBCD	1.4529727
TBCEL	1.1784327
TBK1	3.2366788
TBL1X	0.52764726
TBL1XR1	1.2657287
TBL2	0.68519235
TBP	0.5442481
TBPL1	1.6294072
TBRG1	2.9653165
TBRG4	2.1178467
TCAIM	2.2583168
TCEA1	2.1033213
TCEAL5	4.350527
TCEAL8	1.630624
TCEB1	2.2214506
TCEB2	1.8566134

TCEB3	1.4373977
TCERG1	1.4395947
TCF12	0.7622931
TCF20	0.8236015
TCF24	0.9315953
TCF25	2.1590092
TCHP	0.72064924
TCIRG1	2.6619132
TCN2	1.3256018
TCOF1	0.5910022
TCP1	2.131345
TCP11L2	1.8422506
TCTA	0.49385977
TCTEX1D2	1.2957804
TDG	2.2107785
TDO2	7.3877754
TDP2	1.8063776
TDPOZ1	2.3099375
TDRD7	1.7684
TDRP	1.0107305
TEAD1	1.5187976
TEAD3	1.6886306
TEC	1.2323437
TECPR2	0.80724573
TECR	1.543411
TECTB	2.5933042
TEF	0.46120095
TEFM	1.4439728
TELO2	0.44683528
TEP1	1.0151422
TERF1	1.6202028
TES	0.4868214
TESK1	1.1865184
TET2	0.86915636
TEX10	1.4536235
TEX12	1.4452214
TEX2	3.5844915
TEX261	1.0197799
TEX264	2.023782
TEX30	2.4303
TFAM	2.1288035
TFB1M	3.231694
TFB2M	2.3997467
TFDP1	1.7055838
TFE3	3.0967033

TFEC	1.6484628
TFF3	3.7842467
TFG	2.5490177
TFIP11	1.3603251
TFPI	0.8218782
TFPI2	2.1250284
TFPT	1.4165876
TFR2	7.805114
TFRC	0.89610934
TGDS	1.4568052
TGFA	2.209845
TGFBR1	0.67451715
TGFBRAP1	1.131717
TGIF1	3.7826178
TGM1	2.9151413
TGM2	3.7613468
TGM3	1.0950682
TGOLN1	2.2210553
TGOLN2	0.48448634
TGS1	1.2568591
TGTP1	3.4843514
TGTP2	2.607189
THA1	3.444709
THAP2	1.3067663
THAP4	1.5746748
THAP6	1.0641017
THAP7	1.5773704
THBS1	1.1552093
THEM4	3.2592862
THEM7	1.7336125
THEMIS2	0.7842176
THG1L	1.3887599
THNSL2	0.8323815
THOC1	1.7515006
THOC2	0.98981524
THOC3	1.9064248
THOC5	2.130388
THOC6	1.2174609
THOC7	1.6514885
THOP1	1.4298568
THPO	3.6741102
THRAP3	1.107794
THRB	1.3093312
THRSP	3.6543515
THSD1	0.7141936

THTPA	1.1130202
THUMPD1	2.017896
THUMPD3	2.0234807
THYN1	2.8821318
TIAL1	1.683336
TICAM1	1.1511948
TIFA	7.7142076
TIGD2	2.679579
TIGD4	1.8410685
TIMD2	2.5060189
TIMD4	1.63939
TIMM10	3.457999
TIMM10B	2.679114
TIMM13	1.9580262
TIMM17A	1.8802574
TIMM17B	3.5171897
TIMM21	2.047848
TIMM22	0.70990634
TIMM23	3.0307024
TIMM44	2.2174714
TIMM50	1.6465476
TIMM8A1	4.032034
TIMM8B	2.052789
TIMM9	2.492287
TIMMDC1	2.1518605
TIMP1	4.9962063
TINAGL1	2.5709646
TINF2	2.3133037
TIPARP	3.5548508
TIPIN	1.3417928
TIPRL	2.1166542
TIRAP	2.5466087
TJAP1	1.6216786
TJP3	1.8645093
TK2	1.4360607
TKT	0.7923229
TLCD1	3.5670226
TLDC1	1.0185714
TLDC2	0.98589444
TLE1	1.600836
TLK1	1.3673193
TLK2	2.057031
TLR1	1.7871199
TLR13	0.67610765
TLR2	3.1554701

TLR3	1.4207106
TLR6	2.1066854
TLR7	0.83662295
TLR9	2.0769737
TM2D1	1.5189292
TM2D2	0.64969087
TM2D3	1.7988298
TM4SF4	7.4966326
TM4SF5	1.5939443
TM6SF1	0.31087327
TM6SF2	3.6330454
TM7SF2	4.9272795
TM7SF3	2.8932536
TM9SF1	2.0776646
TM9SF2	2.4846122
TM9SF3	1.5713522
TM9SF4	3.7697165
TMA16	2.623661
TMA7	1.2836621
TMBIM1	0.6133616
TMBIM4	3.0467565
TMBIM6	1.4357407
TMC7	2.4156053
TMCC1	0.3996389
TMCO1	1.3586271
TMCO4	2.0281527
TMCO5	1.6514366
TMCO6	3.1056087
TMED1	1.0240409
TMED10	1.7766812
TMED2	2.5006435
TMED3	1.6433694
TMED4	2.5829923
TMED5	3.0168145
TMED7	2.2747035
TMED8	0.67266965
TMED9	1.838125
TMEM102	1.1110542
TMEM106B	2.0446327
TMEM106C	1.5615766
TMEM11	2.049815
TMEM110	0.43873787
TMEM115	1.506707
TMEM120A	4.3275185
TMEM123	0.8902457

TMEM125	1.9191973
TMEM126A	2.6652138
TMEM126B	2.2384708
TMEM127	1.2133243
TMEM128	1.8286016
TMEM129	2.1366427
TMEM131	1.1676862
TMEM132E	7.8940964
TMEM134	1.0943167
TMEM135	1.4340289
TMEM138	0.7308688
TMEM140	2.6020162
TMEM141	1.1842716
TMEM144	1.3922033
TMEM147	1.5016987
TMEM14C	1.0466583
TMEM150A	2.709974
TMEM151B	0.37642193
TMEM158	2.6544516
TMEM160	0.9073961
TMEM161A	1.2766597
TMEM161B	0.9113853
TMEM165	0.3197019
TMEM167	2.731682
TMEM167B	0.9221661
TMEM170	2.0193424
TMEM170B	1.2729504
TMEM171	1.2584932
TMEM176A	2.0603802
TMEM176B	2.1886237
TMEM177	0.41604137
TMEM179B	1.7562625
TMEM18	1.0767095
TMEM181A	1.0287092
TMEM181B-I	0.9034467
TMEM183A	2.484738
TMEM184B	0.9920995
TMEM184C	1.9934947
TMEM185B	2.266092
TMEM186	2.1317973
TMEM189	0.97516227
TMEM19	2.571655
TMEM192	2.3436491
TMEM194	0.4219134
TMEM199	2.3814278

TMEM2	2.3741748
TMEM202	1.015645
TMEM203	2.7354372
TMEM205	2.261258
TMEM206	0.7227919
TMEM208	2.0846937
TMEM214	2.9366586
TMEM215	0.72368693
TMEM219	1.037164
TMEM220	0.7252004
TMEM222	1.8800972
TMEM230	1.5017278
TMEM234	0.8627217
TMEM238	0.9768393
TMEM239	3.403509
TMEM242	0.8228371
TMEM243	0.7885301
TMEM248	2.6343896
TMEM25	1.0536559
TMEM251	3.0688274
TMEM252	2.2794309
TMEM254A	1.7213962
TMEM254C	0.6817148
TMEM255B	0.7169647
TMEM256	2.2063854
TMEM258	1.8947136
TMEM259	1.9483297
TMEM261	1.6910717
TMEM263	1.9138143
TMEM30A	1.2773011
TMEM30B	1.2072184
TMEM33	1.6780298
TMEM37	0.6878698
TMEM38B	1.9655364
TMEM39A	0.44048047
TMEM39B	0.32350183
TMEM41A	1.7231224
TMEM41B	0.66513705
TMEM42	0.4993894
TMEM5	1.4380126
TMEM50A	1.861903
TMEM51	2.440327
TMEM53	4.4434557
TMEM55B	2.0222747
TMEM56	4.2034636

TMEM57	1.3196833
TMEM59	0.7018764
TMEM60	2.0724223
TMEM62	2.1104538
TMEM63B	0.47438407
TMEM64	1.6868632
TMEM66	1.9576623
TMEM67	1.9729369
TMEM68	1.3060181
TMEM70	2.8072565
TMEM8	2.465944
TMEM80	0.57974577
TMEM82	3.399821
TMEM86A	1.7270219
TMEM86B	1.9971197
TMEM87B	3.2610962
TMEM88B	2.0138693
TMEM9	0.4641564
TMEM97	4.0544252
TMEM98	0.38684773
TMEM9B	0.9981048
TMF1	1.2250769
TMIE	1.2256546
TMLHE	1.4101324
TMPRSS11B	1.3865936
TMPRSS3	3.9323547
TMPRSS6	3.453274
TMPRSS7	1.1442513
TMSB15A	1.066293
TMSB4X	0.84238124
TMTC2	1.3257496
TMTC3	0.9208939
TMUB1	1.4596474
TMUB2	1.676203
TMX1	2.3463652
TMX2	1.3646858
TMX4	1.8362386
TNF	4.244586
TNFAIP1	1.1355035
TNFAIP2	3.1792967
TNFAIP3	5.247488
TNFAIP8L1	2.8470395
TNFRSF10B	2.108526
TNFRSF11B	2.934666
TNFRSF12A	5.4741993

TNFRSF1A	2.2135608
TNFRSF1B	1.2645538
TNFRSF21	1.196537
TNFRSF22	2.7385025
TNFRSF23	2.7764728
TNFRSF4	1.4649465
TNFRSF9	3.648309
TNFSF11	1.1197088
TNFSF9	2.4653876
TNIP1	3.1249626
TNIP2	2.5131862
TNKS	0.82971954
TNKS2	1.1992843
TNNT1	0.3625605
TNPO1	2.231067
TNPO2	2.4255865
TNPO3	2.4849021
TNRC18	0.6902249
TNRC6A	0.83282685
TNRC6B	1.2760394
TOB1	2.8473256
TOB2	2.2151597
TOE1	1.4661028
TOLLIP	1.6692741
TOM1	1.9945333
TOM1L1	2.0160625
TOM1L2	1.6178625
TOMM20	2.5193717
TOMM22	1.1628969
TOMM34	0.8930638
TOMM40	1.4923012
TOMM40L	1.0193865
TOMM5	1.2163255
TOMM6	1.9770973
TOMM7	1.3984416
TOMM70A	1.7541525
TOP1	1.2931716
TOP1MT	3.1273735
TOP3A	1.8802595
TOP3B	0.6990135
TOPORS	2.5437973
TOPORSOS	0.6979811
TOR1A	2.7326896
TOR1AIP1	1.6108062
TOR1AIP2	2.8065984

TOR1B	1.3572466
TOR2A	2.7271469
TOR3A	0.6628754
TOX	5.190653
TOX4	1.8112166
TPD52	1.2258337
TPD52L1	4.090708
TPD52L2	1.8774321
TPGS1	1.4874327
TPI1	4.0703726
TPK1	0.8210437
TPM3	0.5509765
TPMT	3.9009101
TPP1	1.7043617
TPP2	1.311455
TPR	0.81210494
TPRA1	1.1406062
TPRGL	0.61435914
TPRKB	2.511935
TPRN	0.94741416
TPST2	1.0791242
TPT1	1.7629023
TRA2A	0.95558333
TRABD	3.1420224
TRADD	1.9492948
TRAF1	0.44112086
TRAF2	2.5141637
TRAF3	1.0559762
TRAF4	3.422548
TRAF6	1.5576911
TRAFD1	2.0235426
TRAK2	1.0160632
TRAM1	2.349913
TRAM2	1.562433
TRAP1	3.0544465
TRAPPC1	1.4833233
TRAPPC10	1.2950199
TRAPPC11	2.7602012
TRAPPC12	1.9789603
TRAPPC13	1.8548143
TRAPPC2	0.8284285
TRAPPC2L	1.2865837
TRAPPC3	2.1393201
TRAPPC3L	1.4826169
TRAPPC4	1.248205

TRAPPC5	2.2604654
TRAPPC6A	1.271724
TRAPPC6B	1.8991215
TRAPPC8	0.8891251
TRAT1	2.876739
TRDMT1	0.8996651
TREH	6.198758
TREM1	0.8703182
TREM3	0.9644885
TREX1	2.6933005
TRF	4.563835
TRIAP1	0.9063089
TRIB1	3.6766222
TRIB3	4.731695
TRIM11	2.5563886
TRIM12A	2.0865366
TRIM12C	1.7212641
TRIM13	0.65975547
TRIM14	2.3336203
TRIM2	0.9997456
TRIM21	1.2790763
TRIM23	1.6904674
TRIM24	3.4252832
TRIM25	2.6994507
TRIM26	1.2555263
TRIM27	2.6159613
TRIM28	1.3450692
TRIM3	0.5442908
TRIM30A	2.02583
TRIM30D	2.4949658
TRIM32	1.279196
TRIM33	0.41078258
TRIM34A	0.79655194
TRIM34B	0.9151962
TRIM39	1.3950863
TRIM41	2.0836747
TRIM43C	0.76584744
TRIM44	0.45645022
TRIM47	2.50717
TRIM5	0.9906802
TRIM56	1.549525
TRIM6	2.3693404
TRIM68	1.0459709
TRIM8	0.8653977
TRIO	0.83149076

TRIOBP	0.50217414
TRIP10	0.6835096
TRIP11	1.5170882
TRIP13	0.87333274
TRIP4	1.4638903
TRIP6	1.9998267
TRIT1	1.1760557
TRMT1	1.5015147
TRMT10C	1.720571
TRMT11	2.0169375
TRMT112	1.4404557
TRMT13	0.8173816
TRMT1L	1.4716985
TRMT2A	1.5551212
TRMT2B	1.4469283
TRMT5	0.3115132
TRMT6	0.64380836
TRMT61A	3.8379614
TRMT61B	1.5936921
TRMU	1.2519677
TRNAU1AP	2.2304704
TRNT1	2.017986
TRP53BP1	1.1984744
TRP53INP1	4.769203
TRP53INP2	1.1593792
TRP53RK	1.5272894
TRP63	1.0652988
TRPC4AP	1.8108904
TRPM2	1.5095618
TRPM7	1.1561048
TRRAP	0.918643
TRUB2	2.1646187
TSC1	2.2811458
TSC22D4	0.59630656
TSEN2	0.71002793
TSEN54	1.96682
TSFM	1.079263
TSG101	1.4513433
TSHZ2	0.6194627
TSKU	1.3386612
TSN	1.2216742
TSNAX	1.1909878
TSPAN12	2.7034976
TSPAN31	3.9051526
TSPAN33	2.3858287

TSPAN4	2.9654715
TSPAN9	0.3863542
TSPO	1.0253127
TSPO2	0.76885724
TSPYL1	0.7632086
TSPYL2	2.5916774
TSR1	1.9409287
TSR3	2.0944874
TSSC4	1.8313653
TST	1.818423
TSTA3	2.3978627
TSTD1	3.8918254
TSTD2	1.782448
TSTD3	2.0616024
TTBK2	1.0732534
TTC1	2.222813
TTC13	1.8367331
TTC14	0.7121408
TTC17	1.3361075
TTC19	1.8761928
TTC21B	0.5389092
TTC23	1.7244537
TTC27	1.5358198
TTC3	0.8145611
TTC30A1	0.9243834
TTC32	2.77653
TTC33	1.468476
TTC36	4.4481506
TTC37	1.0758929
TTC38	1.9627125
TTC39C	1.9764183
TTC4	1.5899603
TTC7	0.81414676
TTC7B	0.3110125
TTC9C	2.0634463
TTF2	1.6283834
TTI1	1.6871197
TTI2	0.8086407
TTLL12	0.95724654
TTLL4	2.4984324
TTPA	6.776161
TTPAL	2.2960684
TTR	7.582732
TTYH2	2.4599178
TUBA1B	0.4064467

TUBA1C	2.9036038
TUBA4A	2.030533
TUBA8	1.7631094
TUBB2A	2.7173913
TUBB2A-PS2	2.3955395
TUBB4A	0.82625604
TUBB4B	1.542556
TUBB5	1.174609
TUBB6	2.6449463
TUBD1	0.77225566
TUBE1	0.38651228
TUBG1	1.5402901
TUBGCP2	1.5490384
TUBGCP3	1.286762
TUBGCP4	0.96627116
TUBGCP5	0.7750859
TUBGCP6	0.930464
TUFM	2.6951592
TUFT1	0.46551156
TUG1	1.4744551
TULP4	0.28523993
TUSC1	1.3410413
TUSC2	1.8743689
TVP23B	1.6653378
TWF1	2.1966717
TXLNA	0.98151803
TXN1	2.2896998
TXN2	2.0613878
TXNDC11	2.388312
TXNDC12	1.9513109
TXNDC15	1.7205036
TXNDC17	1.9403074
TXNDC5	1.7020772
TXNDC9	1.8962886
TXNL1	1.649863
TXNL4A	1.6665795
TXNL4B	0.73262095
TXNRD1	3.4853232
TXNRD2	2.6432254
TYMP	2.786442
TYMS-PS	0.7226186
TYSND1	1.7050526
TYW1	1.0665421
TYW5	1.4818676
U2AF1	1.7632735

U2SURP	0.96837234
UAP1	2.0157511
UAP1L1	1.0390799
UBA1	1.454375
UBA1Y	0.5360632
UBA2	1.2959502
UBA3	0.7923527
UBA5	3.014396
UBA52	1.5109997
UBA6	1.5787663
UBA7	2.0798447
UBAC1	1.7919137
UBALD1	2.1523998
UBALD2	1.4921896
UBAP1	2.5243862
UBAP2	0.9549382
UBAP2L	0.5003221
UBB	1.958622
UBC	2.1371117
UBD	4.747978
UBE2A	1.2130916
UBE2B	2.0071309
UBE2D2A	1.4738643
UBE2D3	2.5245965
UBE2E1	1.3842189
UBE2E2	2.7039964
UBE2E3	1.251363
UBE2F	2.420265
UBE2G1	1.6215904
UBE2G2	1.5666325
UBE2H	0.54317117
UBE2I	0.6590121
UBE2J1	2.1067994
UBE2J2	2.1487858
UBE2K	1.7431648
UBE2L3	1.6782725
UBE2L6	2.2582872
UBE2M	2.0100954
UBE2N	0.9089358
UBE2O	1.0415823
UBE2Q1	1.2368805
UBE2Q2	1.6397846
UBE2QL1	2.0192327
UBE2R2	1.4547637
UBE2V1	0.64661

UBE2V2	3.8580015
UBE2W	1.1482232
UBE2Z	1.8710134
UBE3A	1.7761681
UBE3B	0.95391965
UBE3C	1.4319558
UBE4A	1.3532603
UBE4B	2.0620925
UBFD1	2.3967545
UBIAD1	1.821945
UBL3	0.85255694
UBL4	1.8396618
UBL5	1.0053265
UBLCP1	1.5741732
UBN1	1.610698
UBN2	0.6811631
UBP1	1.525208
UBQLN1	2.3253462
UBQLN2	1.5725749
UBR1	0.8565743
UBR3	0.8169153
UBR4	2.0962389
UBR5	1.7230928
UBR7	1.4136531
UBTD1	1.5407374
UBTF	3.3609674
UBXN1	1.9930379
UBXN2A	1.7569873
UBXN2B	1.083986
UBXN6	2.2698452
UBXN7	0.9868264
UBXN8	2.7102668
UCHL4	2.4500473
UCHL5	1.4830096
UCK1	2.1502593
UCK2	2.1957405
UCKL1	1.8513772
UFC1	2.4227054
UFD1L	2.9524715
UFL1	2.3932703
UFM1	2.2194698
UFSP2	2.8408
UGCG	2.1401794
UGDH	2.628864
UGGT1	3.080345

UGP2	2.1096685
UGT1A10	3.3656185
UGT1A6B	3.483803
UGT2A3	4.925971
UGT2B1	2.0214162
UGT2B34	6.352518
UGT2B35	7.316736
UGT2B36	8.459707
UGT2B37	4.9288826
UGT2B38	5.476617
UGT2B5	5.180588
UGT3A1	0.5424764
UGT3A2	4.0694695
UHMK1	0.35205913
UHRF1BP1	2.2434595
UHRF1BP1L	1.9637778
UHRF2	1.9890425
ULK1	2.4103692
ULK2	1.194135
ULK3	2.0902297
UMODL1	1.7320031
UMPS	2.7013333
UNC119B	3.5452654
UNC45A	1.8644407
UNC50	1.6090453
UNC93A	3.8130262
UNC93B1	1.7959096
UNG	3.4591033
UNK	3.1086786
UOX	9.03797
UPB1	7.8422203
UPF1	0.4251597
UPF3A	0.96037364
UPF3B	1.4174216
UPP1	2.8174808
UPP2	2.9547243
UQCC2	1.2280171
UQCR10	1.7886155
UQCR11	1.7736375
UQCRB	2.349797
UQCRC1	2.2213018
UQCRC2	2.798156
UQCRFS1	2.8521497
UQCRH	1.8475845
UQCRQ	3.3479536

URAD	2.203345
URAH	6.3469877
URB1	1.7132137
URB2	2.022227
URGCP	1.7267272
URI1	1.474539
URM1	0.5668223
UROC1	3.927625
UROD	2.03345
UROS	2.0294325
USE1	1.3140752
USF1	0.51361823
USF2	1.9591086
USMG5	2.4807699
USO1	2.7014496
USP1	1.7162864
USP10	2.3571904
USP12	1.2177079
USP14	3.0898607
USP15	1.9420292
USP16	2.4514744
USP17LA	1.597209
USP17LD	1.3122027
USP18	3.6482294
USP19	2.1860845
USP20	0.4762187
USP22	1.0338359
USP24	1.5229468
USP25	1.1909468
USP28	0.9534738
USP3	1.9092987
USP30	2.6188576
USP32	1.7643726
USP33	1.6015303
USP36	1.386425
USP38	1.9474657
USP4	1.6871002
USP40	1.6654398
USP42	1.5404994
USP45	1.4671009
USP47	0.98369575
USP48	1.0209124
USP5	1.2980826
USP50	1.8235261
USP53	2.8780062

USP7	1.4345019
USP9X	1.7772467
USPL1	1.7600205
UTP11L	1.8343513
UTP14A	1.3993475
UTP15	1.6457264
UTP23	2.6645334
UTP3	1.829148
UTP6	1.7272718
UVRAG	1.5183551
UXT	2.2966797
VAMP2	0.29885602
VAMP4	0.829921
VAMP7	2.200926
VAMP8	0.5000527
VANGL1	1.3047063
VAPA	1.687619
VAPB	1.0603683
VARS	2.4935234
VASH1	1.2347867
VASP	1.2247612
VAT1	0.5924084
VAV1	1.4587705
VAX2	1.6331813
VBP1	1.1348073
VCAM1	1.2052796
VCAN	0.9712167
VCP	2.1617224
VCPIP1	2.20003
VCPKMT	1.7261817
VDAC1	1.0774558
VDAC2	1.2548645
VDAC3	1.9247406
VEGFB	1.6280625
VEGFC	1.9035718
VGF	0.47711587
VHL	1.3269532
VILL	1.9799845
VIMP	2.5700948
VIPAS39	1.2311423
VIPR1	2.752628
VKORC1	2.6068923
VKORC1L1	1.0267117
VMA21	1.788801
VMN1R10	1.8296537

VMN1R173	1.8144424
VMN1R197	1.3596363
VMN1R219	2.2399235
VMN1R52	1.8450823
VMN1R53	2.1829295
VMN1R65	4.6099052
VMN2R1	1.8799467
VMN2R113	2.1377225
VMP1	3.3119586
VNN1	2.351352
VNN3	8.400918
VOPP1	0.39177108
VPRBP	1.4888198
VPS11	1.4950869
VPS13A	0.39129114
VPS13B	1.4563258
VPS13D	1.4080222
VPS16	1.9373357
VPS18	2.52049
VPS25	1.8509576
VPS26A	1.8865416
VPS26B	1.6602046
VPS28	1.6117384
VPS29	1.7025201
VPS33A	1.2072532
VPS33B	1.4260738
VPS35	1.9453404
VPS36	0.6757562
VPS37A	1.1358113
VPS37B	2.299064
VPS37C	1.2703853
VPS39	1.1236188
VPS41	1.1416614
VPS45	0.41145325
VPS4B	1.8601716
VPS52	1.6490004
VPS54	1.0014837
VPS9D1	0.63501096
VRK2	0.733273
VRK3	2.126476
VSIG4	4.1426697
VTA1	1.8815811
VTI1A	0.6636181
VTI1B	1.7395308
VTN	9.691994

VWA3B	1.4214971
VWA5A	1.1776941
VWA8	2.9834054
VWA9	1.1628501
VWCE	7.0554514
WAC	1.1515257
WAPAL	2.4258378
WARS	1.2772787
WARS2	0.6731839
WASF2	0.9502213
WASH	1.2973635
WASL	1.47661
WBP11	2.1840522
WBP1L	3.8667061
WBP2	1.6087005
WBP5	1.3563731
WBSCR16	3.055156
WBSCR22	1.0234978
WBSCR27	0.6326487
WDFY2	0.43931246
WDFY3	1.1797333
WDR1	2.3461392
WDR11	1.2580211
WDR12	2.172766
WDR20	0.70405316
WDR24	1.9141686
WDR26	2.0781982
WDR3	1.6725633
WDR31	2.7139885
WDR33	1.4910419
WDR34	2.4261792
WDR35	0.61149263
WDR36	2.0289853
WDR37	0.6888666
WDR4	2.9497073
WDR41	1.0231059
WDR43	1.8887146
WDR45	1.0795186
WDR45B	1.6107557
WDR46	2.0559356
WDR48	1.7558925
WDR5	2.0479639
WDR53	1.5203393
WDR55	2.832484
WDR59	2.1174474

WDR60	0.457155
WDR61	1.8888128
WDR73	2.1923969
WDR74	2.120856
WDR75	1.3506567
WDR77	1.3598192
WDR81	2.1420777
WDR82	1.075938
WDR83	1.5806835
WDR83OS	1.20384
WDR89	3.5338147
WDR91	0.75997424
WDR92	3.8108413
WDTC1	0.73486924
WDYHV1	1.3061371
WFDC17	4.026886
WFDC18	2.6249478
WFDC5	1.4410582
WFS1	2.3059876
WHAMM	2.7184522
WHSC1L1	1.2208788
WIPF2	1.9174564
WIPI1	0.5576589
WIPI2	2.444886
WIZ	1.9994771
WNT10A	3.9796934
WRAP53	0.3653581
WRAP73	1.5414908
WRNIP1	2.874458
WSB1	2.4579947
WTAP	1.845689
WWOX	0.8829229
WWP2	2.1224082
XAB2	0.61475587
XAF1	2.74668
XBP1	3.7264204
XCL1	0.2909379
XDH	3.3872588
XIAP	1.3179333
XIST	2.7666967
XKR8	2.298951
XKR9	2.8100166
XLR3A	1.1602399
XLR3B	1.5550056
XPA	2.8773644

XPC	0.70402694
XPNPEP1	1.9780667
XPO1	0.92230487
XPO4	1.9158392
XPO5	1.2536447
XPO6	1.9232912
XPOT	1.6998379
XPR1	1.0867612
XRCC4	1.5040405
XRCC5	1.8828385
XRCC6	1.3277957
XRN1	0.8992343
XRN2	2.0218642
XYLB	3.9069831
XYLT2	1.4169362
YAE1D1	1.2169683
YAF2	1.0270417
YAP1	0.7962773
YARS	1.3978579
YARS2	2.8101246
YBX1	0.8293669
YBX3	1.2213681
YEATS4	1.1070697
YES1	1.6000705
YIF1A	3.194813
YIF1B	1.1932371
YIPF1	1.3480852
YIPF2	1.7318723
YIPF3	1.1760709
YIPF4	1.5874708
YIPF5	2.6226127
YIPF6	0.9573798
YKT6	1.012974
YME1L1	2.1956134
YOD1	2.697273
YPEL2	2.0507312
YPEL5	1.9291399
YRDC	2.467294
YTHDC2	2.1687722
YTHDF1	1.252465
YTHDF2	1.4920204
YTHDF3	1.2849772
YWHAB	0.7647989
YWHAE	1.776139
YWHAG	1.6296256

YWHAH	1.0395105
YWHAQ	0.8762562
YWHAZ	0.7005193
YY1	1.1880963
ZADH2	0.7000334
ZBED5	1.3092237
ZBED6	1.744904
ZBP1	3.4072306
ZBTB10	1.7087986
ZBTB11	0.8225641
ZBTB12	0.5421717
ZBTB16	2.7988498
ZBTB18	1.056617
ZBTB2	0.9480455
ZBTB20	1.7697284
ZBTB22	1.1335442
ZBTB24	0.5622597
ZBTB25	1.4245732
ZBTB26	1.240202
ZBTB33	1.1880696
ZBTB34	0.45571303
ZBTB39	1.7561078
ZBTB40	0.9917338
ZBTB43	2.1760762
ZBTB48	1.3320346
ZBTB5	1.1727936
ZBTB6	0.8088329
ZBTB7A	0.71556973
ZBTB7B	1.3234365
ZBTB8B	1.0886917
ZBTB8OS	2.236912
ZBTB9	0.39233708
ZC3H11A	1.679198
ZC3H12A	1.8815198
ZC3H12C	1.1898198
ZC3H12D	0.37791038
ZC3H13	2.333999
ZC3H14	0.42702365
ZC3H15	2.1218545
ZC3H18	1.5520365
ZC3H3	1.1573722
ZC3H7A	2.3147862
ZC3H7B	0.38950706
ZC3HAV1	1.8307283
ZC3HC1	1.1284969

ZCCHC10	0.80238056
ZCCHC11	2.6349795
ZCCHC14	0.86116934
ZCCHC17	1.0977776
ZCCHC2	1.4844716
ZCCHC24	2.0577266
ZCCHC3	0.79974556
ZCCHC6	1.6856544
ZCCHC7	1.6466653
ZCCHC8	1.7765391
ZCCHC9	3.257332
ZCRB1	1.5586846
ZDHHC12	2.5297587
ZDHHC13	2.267679
ZDHHC16	1.7049053
ZDHHC17	1.1412086
ZDHHC18	0.44532943
ZDHHC2	1.5861261
ZDHHC20	0.84559107
ZDHHC21	0.70820355
ZDHHC24	1.6393743
ZDHHC4	0.64544487
ZDHHC5	2.0933254
ZDHHC6	2.4202578
ZDHHC7	1.030514
ZDHHC9	1.1700413
ZER1	0.83594584
ZFAND1	1.6811621
ZFAND2A	1.6200845
ZFAND2B	2.1287477
ZFAND3	1.0718648
ZFAND4	2.842851
ZFAND5	0.9706619
ZFAND6	2.0893576
ZFAT	0.630816
ZFC3H1	2.3735263
ZFHX4	2.6428874
ZFML	0.8903706
ZFP1	1.0251067
ZFP105	0.59010863
ZFP106	1.5792634
ZFP110	1.8196533
ZFP113	1.6373193
ZFP119B	1.7870791
ZFP120	0.89897966

ZFP128	0.63663197
ZFP131	1.6568224
ZFP133-PS	2.9656498
ZFP14	1.2368484
ZFP146	0.8893435
ZFP148	0.59755635
ZFP180	1.8924615
ZFP182	0.7223351
ZFP189	2.3930986
ZFP191	1.3461616
ZFP202	2.1967614
ZFP207	1.5690691
ZFP212	1.0005052
ZFP213	1.4997346
ZFP217	0.79768586
ZFP229	0.8648486
ZFP236	2.7445862
ZFP26	0.8692591
ZFP260	0.9589584
ZFP263	1.5668976
ZFP264	2.2190185
ZFP275	1.9204361
ZFP276	1.1495717
ZFP277	1.1787231
ZFP280B	1.3496537
ZFP280C	1.6943214
ZFP280D	1.0862038
ZFP281	2.691682
ZFP282	1.2055228
ZFP292	2.2735107
ZFP3	1.3106198
ZFP30	0.7605407
ZFP316	0.9414942
ZFP317	0.85592294
ZFP318	1.2404883
ZFP326	1.4325917
ZFP330	3.1163032
ZFP335	2.0569248
ZFP341	0.7399235
ZFP35	1.2809451
ZFP354A	0.422251
ZFP358	1.1413
ZFP36	4.127426
ZFP367	0.8544431
ZFP36L1	2.2696269

ZFP36L2	0.84445596
ZFP384	1.5448194
ZFP386	1.1470907
ZFP397	1.0697544
ZFP40	0.34203124
ZFP407	1.1252325
ZFP408	1.6928027
ZFP410	1.4571841
ZFP414	0.6049268
ZFP426	1.0612051
ZFP429	2.6898339
ZFP433	1.6638656
ZFP442	0.9356811
ZFP445	1.3772624
ZFP446	1.7443209
ZFP451	0.40542102
ZFP457	0.99345946
ZFP458	1.2896562
ZFP488	1.9897063
ZFP507	1.7322087
ZFP51	1.1365783
ZFP511	1.1875122
ZFP512	1.0312023
ZFP513	2.537526
ZFP518B	0.74949574
ZFP523	0.6365135
ZFP524	1.4330423
ZFP53	0.7063105
ZFP553	1.4208457
ZFP558	0.9833343
ZFP566	0.962178
ZFP568	2.5296304
ZFP574	0.8008692
ZFP58	0.4799552
ZFP59	0.8695569
ZFP592	1.3260553
ZFP593	1.7305672
ZFP597	0.67298317
ZFP598	3.2118642
ZFP607	0.51983786
ZFP612	3.9329784
ZFP618	0.9538908
ZFP62	1.0265718
ZFP622	2.0539367
ZFP623	2.014295

ZFP626	0.7233224
ZFP628	1.7235451
ZFP629	0.6290381
ZFP637	0.5395086
ZFP639	1.6425812
ZFP64	1.4024265
ZFP653	2.405132
ZFP654	1.078099
ZFP655	2.987397
ZFP658	2.323723
ZFP661	1.9894569
ZFP663	0.3822286
ZFP668	1.5770733
ZFP672	0.91964316
ZFP68	1.0154507
ZFP687	1.1721804
ZFP688	0.97329736
ZFP689	1.4699135
ZFP691	2.4848406
ZFP692	1.2194707
ZFP706	0.99437785
ZFP707	0.4521973
ZFP709	1.3813736
ZFP715	1.6769991
ZFP746	1.650059
ZFP747	0.9589803
ZFP758	0.5645063
ZFP764	0.5431421
ZFP768	0.7282622
ZFP770	1.3695736
ZFP771	1.5707953
ZFP777	1.7637436
ZFP787	2.094084
ZFP790	1.4068682
ZFP791	3.5458963
ZFP800	2.5402305
ZFP809	1.5121658
ZFP81	0.54153085
ZFP811	1.2940509
ZFP825	1.0784335
ZFP830	1.1754
ZFP865	0.7999575
ZFP867	1.4808271
ZFP868	1.5936434
ZFP869	1.5139277

ZFP871	1.8457491
ZFP872	2.2090495
ZFP9	1.5334637
ZFP90	0.8851707
ZFP91	0.8479042
ZFP93	1.0393233
ZFP931	1.6515062
ZFP935	1.0063691
ZFP938	1.5109539
ZFP943	1.3532627
ZFP944	0.606725
ZFP945	0.8056185
ZFP946	1.5746443
ZFP947	1.4802921
ZFP949	0.9641907
ZFP953	0.9557116
ZFP954	1.861131
ZFP955A	0.50233436
ZFP955B	1.2768767
ZFP956	0.4558761
ZFP959	1.7855422
ZFP960	0.94607186
ZFP961	1.1651165
ZFP963	1.0808659
ZFPL1	0.62326884
ZFPM1	2.3938677
ZFYVE1	1.2841156
ZFYVE16	1.369271
ZFYVE19	1.7329085
ZFYVE20	1.1497991
ZFYVE26	2.997327
ZFYVE27	1.3164618
ZGLP1	0.3593061
ZHX1	1.0518959
ZHX2	0.80755854
ZHX3	1.5666854
ZKSCAN1	1.770015
ZKSCAN17	2.1630442
ZKSCAN3	1.5315187
ZKSCAN5	1.225951
ZKSCAN6	2.3033779
ZKSCAN7	1.2518785
ZMAT1	0.87430024
ZMAT2	1.9279039
ZMAT3	0.64627457

ZMAT5	0.8395529
ZMIZ1	0.4163816
ZMIZ2	0.3874452
ZMPSTE24	1.9877212
ZMYM2	0.8141873
ZMYM3	1.0333021
ZMYM4	0.36830497
ZMYM5	0.9618027
ZMYM6	1.0547769
ZMYND15	1.122772
ZMYND19	1.643606
ZMYND8	0.3938992
ZNFX1	1.9758556
ZNHIT1	2.4699857
ZNHIT2	2.0289075
ZNHIT3	0.7472191
ZNHIT6	1.2219512
ZNRD1	1.124006
ZNRD1AS	2.247523
ZNRF1	3.0695922
ZNRF2	1.8060188
ZPR1	3.3598835
ZRANB1	1.9015925
ZRANB2	1.8171413
ZRSR1	0.9823892
ZSCAN12	1.9303687
ZSCAN21	1.6447008
ZSCAN22	2.2971237
ZSCAN26	1.8168552
ZSCAN29	1.4605961
ZSWIM3	0.60669017
ZSWIM5	0.5378027
ZSWIM7	2.9487803
ZSWIM8	1.6805346
ZUFSP	1.7766192
ZW10	1.9985969
ZWINT	3.3023264
ZXDB	0.25377607
ZXDC	1.1263449
ZYG11B	1.4737761
ZYX	2.4390628
ZZEF1	0.9962504
ZZZ3	1.5741413
